# Common causes of EID sample rejection in Zimbabwe and how to mitigate them

**DOI:** 10.1371/journal.pone.0210136

**Published:** 2019-08-08

**Authors:** Charles Chiku, Maria Zolfo, Mbazi Senkoro, Mzwandile Mabhala, Hannock Tweya, Patience Musasa, Fungai D. Shukusho, Exervia Mazarura, Angela Mushavi, Douglas Mangwanya

**Affiliations:** 1 National Microbiology Reference Laboratory, Harare, Zimbabwe; 2 Institute of Tropical Medicine, Antwerp, Belgium; 3 National Institute for Medical Research, Muhimbili, Tanzania; 4 University of Chester, Chester, United Kingdom; 5 The Lighthouse Trust, Lilongwe, Malawi; 6 Prevention of Mother to Child Transmission of HIV, Harare, Zimbabwe; 7 Directorate of Laboratory Services, Harare, Zimbabwe; University of Ghana College of Health Sciences, GHANA

## Abstract

Early infant diagnosis (EID) of HIV provides an opportunity for early HIV detection and access to appropriate Antiretroviral treatment (ART). Dried Blood Spot (DBS) samples are used for EID of exposed infants, born to HIV-positive mothers. However, DBS rejection rates in Zimbabwe have been exceeding the target of less than 2% per month set by the National Microbiology Reference Laboratory (NMRL), in Harare. The aim of this study was to determine the DBS sample rejection rate, the reasons for rejection and the possible associations between rejection and level of health facility where the samples were collected. This is an analytical cross-sectional study using routine DBS sample data from the NMRL in Harare, Zimbabwe, between January and December 2017.A total of 34 950 DBS samples were received at the NMRL. Of these, 1291(4%) were rejected. Reasons for rejection were insufficient specimen volume (72%), missing request form (11%), missing sample (6%), cross-contamination (6%), mismatch of information (4%) and clotted sample (1%). Samples collected from clinics/rural health facilities were five times more likely to be rejected compared to those from a central hospital. Rejection rates were above the set target of <2%. The reasons for rejection were ‘pre-analytical’ errors including labelling errors, missing or inconsistent data, and insufficient blood collected. Samples collected at primary healthcare facilities had higher rejection rates.

## Introduction

Prevention of mother-to-child transmission (PMTCT) of HIV is one of the most important challenges in the global elimination of paediatric HIV infection [1, 2]. WHO recommends EID to be performed as part of the PMTCT cascade on HIV-exposed infants within four to six weeks of age [1].

In February 2013, Zimbabwe’s Ministry of Health and Child Care (MoHCC) adopted Option B+, the implementation of lifelong ART for all pregnant and breastfeeding HIV-positive mothers, regardless of CD4 count and clinical stage [3]. This policy change represented a paradigm shift in the implementation of PMTCT and ART programmes. However, only half (51%) of the HIV-exposed infants has access to EID-testing at age six to eight weeks or at earliest possible opportunity [1]. If the EID-test is negative at 6–8 weeks and the risk of HIV-exposure through breast feeding continues, the test must be repeated at weaning. Thereafter, a rapid test carried out after 18 months of age will provide the definitive diagnosis [4]. Early initiation of ART within the first 12 weeks of life, has shown a 75% reduction in AIDS related illness and mortality [4, 5].

**Fig 1:**
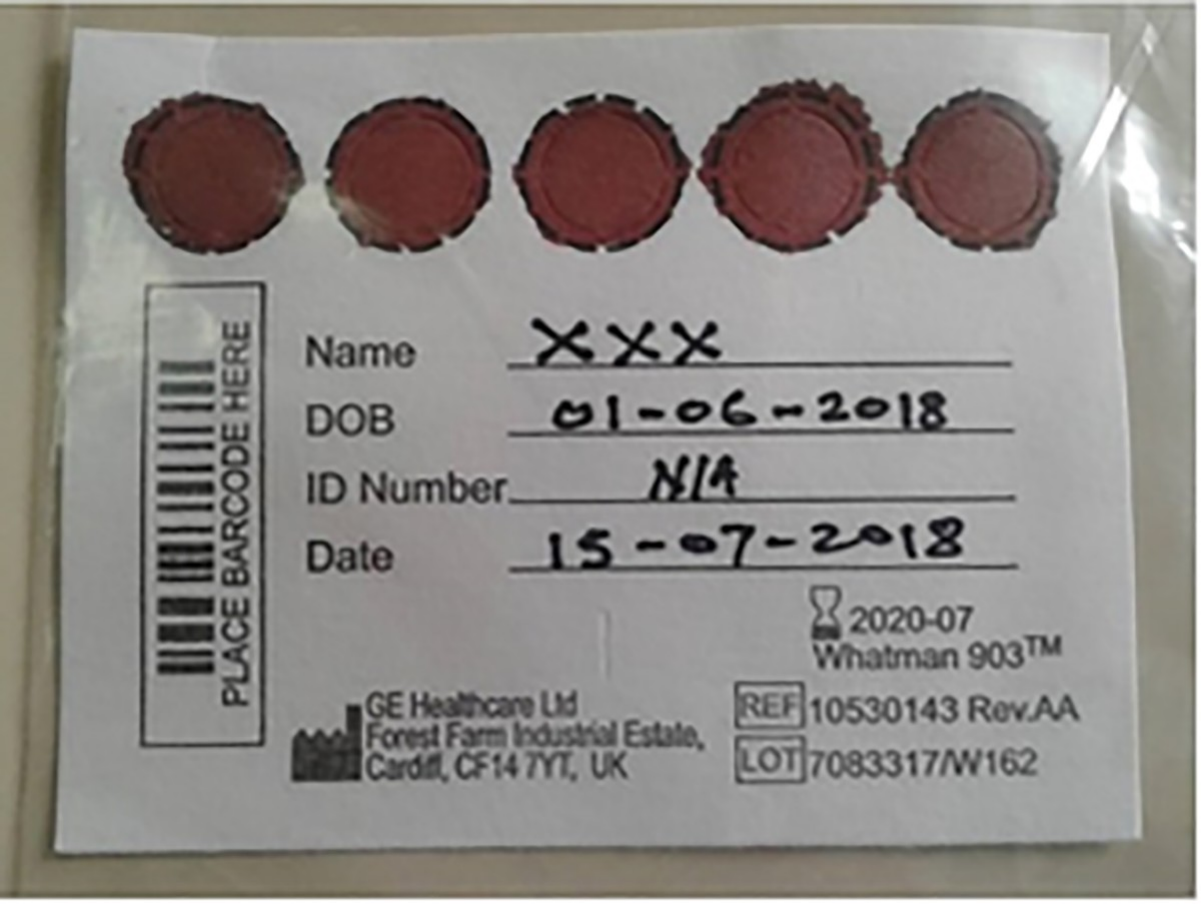
Five circles blood spot card.

Dried Blood Spot (DBS) samples are preferred to EDTA anticoagulated whole blood samples for EID-testing because they permit infant HIV-testing even in areas with limited resources for collection, storage and transportation of blood samples. DBS samples are collected by pricking the heel of the infant using a blood lancet, dripping the blood onto the five circles of a DBS card (see Fig 1), and leave to dry for two to four hours on a dry and dust-free surface before packaging and sending it by courier to the National Microbiology Reference Laboratory (NMRL) in Harare.

NMRL is one of the few laboratories in Zimbabwe that tests EID DBS samples using Roche AmpliPrep/Cobas TaqMan 96 analyzer with technological capability of analysing one full spot protocol for testing. Box 1 outlines NMRL DBS rejection criteria.

**Box 1. DBS rejection criteria at NMRL.**

1. Incomplete identification on the request and/or DBS card;
2. DBS card without request form/Request form without DBS card;
3. Specimens with evidence of contamination, leakage or spillage in transit;
4. DBS sample with traces of blood clots or clumps;
5. DBS sample with less than 3 full circles (insufficient).

The laboratory rejects all samples that meet the criteria listed in Box 1.

Fig 1 below shows a DBS sample accepted for testing. All samples that did not meet the volume criteria were rejected.

There is evidence that insufficient volume of specimen is one of the major reasons for the high DBS sample rejection rate. It is unclear why this is the case, given that the evidence also suggests that the technology for testing DBS samples can analyse and produce conclusive results from a minimum of two insufficient spot samples [6]. DBS sample rejection may lead to missed diagnostic opportunities (MDO) in EID eligible infants. Insufficient sample volume is not the only contributing factor to DBS sample rejection. Others include poor sample collection techniques, poor documentation of both samples and request forms, and inconclusive results on assay in the laboratory.

These issues are not unique to the Zimbabwean PMTCT and ART programme. Comparable programmes in other African countries reported similar problems and it is estimated that sample collection errors account for 60–70% of all rejections [7]. A few examples: the results of a study carried out in South Africa showed that 3.7% of samples were rejected due to ‘pre-analytical’ errors including labelling errors, sample damage, missing or inconsistent data, and insufficient volume [8]. Similarly, in a study conducted in Nigeria, the main reasons for rejection were poor collection technique (26.3%), improper labelling (16.4%) and insufficient blood collection (14.8%) [9]. The same study also showed that DBS collected at primary and secondary health care level were two to three times more likely to be rejected than those collected in tertiary healthcare facilities [10].

In Zimbabwe, feedback from NMRL to submitting healthcare facilities can take up to fourteen days from sample collection [11]. It takes a further few weeks before the healthcare facility informs the mother-baby pair that the sample was rejected and that a new DBS sample is required [10]. Inevitably, this results in delayed diagnosis and treatment of HIV-infected infants, MDO and subsequently loss to follow-up.

The lack of laboratory surveillance in Zimbabwe to monitor and track EID-related processes has been a cause for concern. In addition, there are very few studies that investigate the reasons for DBS rejection [6]. The absence of data on DBS rejection rates means that information about appropriate and corrective actions to improve the EID-programme and reduce loss to follow-up is limited.

The aim of this study was to determine the proportion of DBS samples sent to the NMRL and rejected between January and December 2017 and the reasons for this rejection.

## Methods

### Study design

A cross- sectional study that used routine data from EID-laboratory information management system (LIMS). DBS samples collected from all facilities in 5provinces of Zimbabwe and sent to NMRL were logged into EID-LIMS. Records of rejected samples in EID-LIMS were validated against source hard copy documents. Descriptive analysis was performed to describe the variables in relation to number and proportion of rejected DBS, reasons for rejection and level of the health facilities.

### Study setting

#### General setting

Zimbabwe is a country in Southern Africa, with a population of 17 million people in 2017 [11]. The country is divided into 8 provinces and 2 cities that are also provinces (Bulawayo and Harare).

It has a four-tiered health care system, which includes (i) the primary health care facilities (predominantly rural health centres and polyclinics), (ii) district health centres (including mission hospitals), (iii) provincial hospitals and (iv) tertiary referral or central hospitals. EID-samples from health facilities located in 5 provinces (Harare, Mashonaland West, Mashonaland East, Mashonaland Central and Midlands Provinces) are tested at NMRL. The results are subsequently returned to the facility for initiation of treatment and care of the infants.

#### Specific setting

The NMRL is based in Harare. It was established in 2007 and is the first accredited laboratory to perform HIV DNA PCR. Until 2013, NMRL was the only laboratory that processed DBS samples for EID nationally. In 2013, the Zimbabwean government decentralised laboratory services. This means that the NMRL now only processes EID- samples from 5 provinces while the laboratories from Mpilo and Mutare process EID-samples from nearby health facilities. This study analysed the DBS sample rejection rate of samples from these 5 provinces sent to the NMRL.

Box 1 shows the criteria for rejection.

DBS samples that met the above criteria were rejected and the laboratory conveyed feedback, including reason for rejection, to the health facility. The health facility in turn, contacted the care provider requesting another sample.

#### Study population and period

DBS samples collected from HIV-exposed infants from all facilities in the five provinces and sent to NMRL between January and December 2017, were included in the study.

#### Data collection and validation

Data on rejected samples from the EID-LIMS were validated against source documents of the rejected DBS samples that were kept as hard copies. The data extraction was done cumulatively and disaggregated by health facility on a monthly basis. Variables collected included: date of sample receipt; number of the laboratory request form; total number of DBS samples received; total number of rejected samples; number of samples rejected by health facility level; health facility name, level, district, and province of rejected DBS samples; reason for rejection (S1 Fig).

#### Data analysis and statistics

Descriptive analysis was conducted to describe the variables in relation to number and proportion of rejected DBS samples, reasons for rejection and level of the referring health facility. The Chi square test was performed using STATA version 13 (Stata Corp, Texas USA) and presented as odd ratios (OR) with 95% confidence intervals (CI). Differences at 5% level were regarded as significant

#### Ethical considerations

Ethical approval: Permission to conduct the study was obtained from the Director of NMRL, the Union Ethics Review Committee (reference number EAG/07/18) and the Medical Research Council of Zimbabwe (Ethical approval reference number MRCZ/E/194).

## Results

Between January and December 2017, 34 950 DBS samples were received at the NMRL, 1291 (4%) samples were rejected. Table 1 shows the proportion of DBS samples rejected by month. The proportion of rejected samples ranged from 3% to 6% with the highest rejection rate observed in September.

**Table 1:** Number and proportion of rejected DBS samples at the NMRL, between January and December 2017.

	Dried Blood Spot samples		
	Received	Rejected (%)	
Total	34 950	1291	(4)
Months			
January	4138	105	(3)
February	2959	98	(3)
March	4123	128	(3)
April	3253	114	(4)
May	3533	132	(4)
June	3150	107	(3)
July	2668	106	(4)
August	2593	105	(4)
September	2191	125	(6)
October	2377	125	(5)
November	2189	88	(4)
December	1776	58	(3)

Table 2 shows the proportion of DBS samples rejected including the level of health facility. DBS samples collected from clinics or rural health centres, district/faith-based hospitals and provincial hospital were more likely to be rejected compared to those from a central hospital.

**Table 2:** The relationship between DBS samples rejected by the NMRL and the level of health facility from which they were collected between January and December 2017.

	Dried Blood Spot samples					
Level of health facility	Received (%)		Rejected (%)		Odds Ratio (95% CI)	
Central hospital	1619	(99)	13	(1)	1	
Clinic/Rural health centre	31 460	(97)	1089	(3)	4.58	(2.65–7.64)
District/faith-based hospital	5721	(97)	147	(3)	3.37	(1.91–5.95)
Provincial hospital	346	(98)	8	(2)	2.92	(1.20–7.11)

Thirty-four DBS samples for which no reason for rejection was stated on the request form were excluded from this analysis. The major reason for rejection of DBS samples was insufficient specimen volume. If there was insufficient blood on all 5 spots of the DBS card, the volume was considered inadequate and the sample rejected (Table 3). Other reasons for rejections were missing request form, missing sample, cross contamination, clotted sample, or mismatched information.

**Table 3:** Proportion of DBS samples rejected at the NMRL between January and December 2017, rejection reason and type of health facility.

	N	(%)	Clinic/rural health centre		District hospital		Provincial hospital		Central hospital	
Rejected DBS samples	1257		1089		147		8		13	
Insufficient	909	(72)	801	(73)	94	(64)	3	(38)	11	(84)
Missing request form	133	(11)	100	(9)	28	(19)	5	(63)	0	(0)
Missing sample	77	(6)	65	(6)	11	(7)	0	(0)	1	(8)
Clotted sample	11	(1)	10	(1)	0	(0)	0	(0)	1	(8)
Mismatched info	52	(4)	42	(4)	10	(7)	0	(0)	0	(0)
Cross contamination	75	(6)	71	(7)	4	(3)	0	(0)	0	(0)

Table 4 shows the number and proportion of rejected DBS samples by province. Rejection rate ranged from 2% to 7% among the 5 provinces. Mashonaland West (7%) and Midlands Province (4%) showed the highest rejection rates.

**Table 4:** Proportion of rejected DBS samples at NMRL and Provinces, January—December 2017.

	Dried Blood Spot samples		
	Received	Rejected (%)	
Total	34 950	1291	(4)
Province			
Harare	9267	165	(2)
Mash Central	6548	187	(3)
Mash East	8525	337	(3)
Mash West	5197	367	(7)
Midlands	5413	235	(4)

^Mash^ Mashonaland

## Discussion

This is the first study in Zimbabwe’s EID-programme that assesses the magnitude of and reasons for the rejection of DBS samples sent by five provinces to NMRL for analysis. The national maximum rejection target for DBS samples is <2% per month. This study found that the NMRL’s rejection rate is above the national target. These findings are similar to those of a study conducted in KwaZulu Natal province in South Africa where the rejection rate was 4% [6]. An even higher aggregated EID-programme DBS rejection rate of 7.4% was reported in a similar study conducted in Mashonaland West province, Zimbabwe, in 2017 [10]. However, the latter study only investigated one province while our study looked at five provinces.

Analysis showed that samples collected at primary healthcare facilities (rural clinics) where most patients receive care were five times more likely to be rejected. Findings are similar to a Nigerian study where the bulk of rejected samples came from facilities that serve most patients [9]. One possible explanation for this higher rejection rate in primary healthcare facilities is the high demand and the lack of financial resources for staff recruitment and equipment in these facilities. This may explain the inadequate quality assurance mechanisms when collecting samples.

The other possible explanation could be that rural facilities are unable to offer adequate “in-service” staff training. They can only afford to send a small number of staff for training in large urban centres. Upon return they provide cascade training to the rest of the team. The research by Smit *et al* reported that “in-service” cascading of skill is not effective as a training model because the quality appears to be poorer. Smit *et al* claim that staff orientation and mentorship on DBS collection, storage and transportation is essential to standardise skills and improve DBS sample collection [12]. Their conclusions are consistent with findings from Nkengasong’s study which showed that sensitising health care workers on sample collection, handling and completion of laboratory request forms reduced errors from 19.05% to 6.76% [13].

The current study found that a large proportion of the rejections was due to insufficient blood volume. These findings were consistent with the results of the study in South Africa, where 48% of all rejection was due to inadequate sample collection [6]. A lower proportion, i.e. 14.8% of rejection due to insufficient blood collection, was observed in a Nigerian study [7].

A previous study has shown that for analysis purposes the combination of two insufficient DBS samples still yields satisfactory results. Govender *et al* showed that the combined use of two insufficient spots avoided 10 504 samples being rejected due to insufficient blood volume. However, there was no denominator available in this study to allow us to determine the proportion of prevented rejections [6].

However, Govender *et al* conducted a validation study on the modified testing method to determine if the use of two insufficient DBS protocol can yield the same results as a validated method of one full spot protocol [6]. This study showed that the two insufficient spot protocol yielded results that are comparable to the validated one full spot protocol. These findings may lead NMRL to revise its rejection criteria in relation to emerging evidence and consider accepting samples with two full spots.

Furthermore, there has been evidence that implementation of a quality management system improves identification of flaws of the system while corrective actions eliminate the root causes of the problems, thereby reducing the sample rejection rate [14, 15].

This study has found that Mashonaland West Province had the highest proportion of rejected DBS samples, followed by Midlands Province. Further investigation is required to establish the possible causes.

These rejections may result in MDO of HIV-infected infants, loss to follow-up and delay of early ART-initiation of HIV-positive infants. DBS rejections also contribute to delays in accessing laboratory results for EID-testing, have serious implications on the PMTCT programme and lives of infants that may need life-long ART [8,10].

### Limitations

The EID-programme does not have a unique patient identifier, we were thus unable to track whether another DBS sample was collected and the extent of the delay in this second collection.

We were not able to observe the staffing levels and workload of the health facilities that sent samples to NMRL. Additionally, we could not verify the qualifications, competencies and training of staff for DBS sample collection. These factors may however have an impact on quality assurance mechanisms for sample collection

There were no available data to track the interval between sample collection and informing the mother that the sample had been rejected. Such data could show whether sample rejection adversely influences early ART-initiation for infants.

### Recommendations

- NMRL to monitor the rejection rates and notify the health facility management in real time to enable corrective and preventive actions and avoid delays due to rejected DBS.
- Revision of the user manual to make it clear that the blood droplet must spread along the DBS card to reach the marked sections of the circle.
- Develop mentorship programmes on DBS sample collection, storage and transportation especially in areas where there are high rejection rates.
- As samples probably pass through district laboratories or facilities with laboratories first, these could evaluate the quality of the samples before sending them to NMRL. This would allow identification of deficiencies closer to the collection site, and corrective actions could be taken at district level before sending the sample to the NMRL.
- The quality management system of laboratory processes needs to be strengthened to identify deficiencies in DBS sample management. It enables corrective actions to reduce sample rejection rates that impact on patient care.
- NMRL needs to conduct a laboratory validation of “two insufficient spot protocol” against a” one full spot protocol” so that the rejection criteria can be reviewed based on the scientific evidence of the validation.

## Conclusions

This study has shown that DBS rejection rates are above the national target. The main reason for rejection was insufficient volume samples. Clinic/rural health centres have a higher rejection rate than central hospitals. Overall, there is need to monitor rejection rates in real time, so that corrective and preventive actions may be taken to reduce or eliminate causes of DBS sample rejection.

## Supporting information

S1 Fig Data collected on received and rejected dried blood spot specimens at NMRL. (PDF)
