# Supplementary material for: Common causes of EID sample rejection in Zimbabwe and how to mitigate them
Source: PLoS One. 2019 Aug 8;14(8):e0210136. doi: 10.1371/journal.pone.0210136 (PMC6687112; doi:10.1371/journal.pone.0210136)
Supplement: S1 Fig — (PDF) [file pone.0210136.s001.pdf]

iddbs	facility	level	dbsrej	receipt0	dbsrejrsn	_Ilevel_2	_Ilevel_3	_Ilevel_4
2013278282	20	rural/clinic	No	03/01/2017		0	0	0
2015335082	20	rural/clinic	No	03/01/2017		0	0	0
2015392519	20	rural/clinic	No	03/01/2017		0	0	0
2013260396	20	rural/clinic	No	03/01/2017		0	0	0
2013260393	20	rural/clinic	No	03/01/2017		0	0	0
2013260395	377	rural/clinic	No	03/01/2017		0	0	0
2012284616	377	rural/clinic	No	03/01/2017		0	0	0
2011142544	377	rural/clinic	No	03/01/2017		0	0	0
2014344462	377	rural/clinic	No	03/01/2017		0	0	0
2015293909	377	rural/clinic	No	03/01/2017		0	0	0
2015335567	377	rural/clinic	No	03/01/2017		0	0	0
2015408086	377	rural/clinic	No	03/01/2017		0	0	0
2012331318	377	rural/clinic	No	03/01/2017		0	0	0
2012291532	377	rural/clinic	No	03/01/2017		0	0	0
2012317278	377	rural/clinic	No	03/01/2017		0	0	0
2012291531	377	rural/clinic	No	03/01/2017		0	0	0
2012246279	377	rural/clinic	No	03/01/2017		0	0	0
2014365142	377	rural/clinic	No	03/01/2017		0	0	0
2015335638	377	rural/clinic	No	03/01/2017		0	0	0
2015358101	377	rural/clinic	No	03/01/2017		0	0	0
2015335639	104	rural/clinic	No	03/01/2017		0	0	0
2015361093	104	rural/clinic	No	03/01/2017		0	0	0
2015335640	104	rural/clinic	No	03/01/2017		0	0	0
2015368257	104	rural/clinic	No	03/01/2017		0	0	0
2015368256	104	rural/clinic	No	03/01/2017		0	0	0
2015335641	104	rural/clinic	No	03/01/2017		0	0	0
2011204469	104	rural/clinic	No	03/01/2017		0	0	0
2015418364	104	rural/clinic	No	03/01/2017		0	0	0
2015335642	104	rural/clinic	No	03/01/2017		0	0	0
2015352351	104	rural/clinic	No	03/01/2017		0	0	0
2012245710	104	rural/clinic	No	03/01/2017		0	0	0
2015418365	104	rural/clinic	No	03/01/2017		0	0	0
2015301431	104	rural/clinic	No	03/01/2017		0	0	0
2012245711	104	rural/clinic	No	03/01/2017		0	0	0
2015301432	104	rural/clinic	No	03/01/2017		0	0	0
2012245712	104	rural/clinic	No	03/01/2017		0	0	0
2012245713	104	rural/clinic	No	03/01/2017		0	0	0
2015357619	104	rural/clinic	No	03/01/2017		0	0	0
2012295382	104	rural/clinic	No	03/01/2017		0	0	0
2015357620	104	rural/clinic	No	03/01/2017		0	0	0
2012252686	104	rural/clinic	No	03/01/2017		0	0	0
2012295383	104	rural/clinic	No	03/01/2017		0	0	0
2015340820	104	rural/clinic	No	03/01/2017		0	0	0
2012295384	104	rural/clinic	No	03/01/2017		0	0	0
2015300656	104	rural/clinic	No	03/01/2017		0	0	0
2012295385	104	rural/clinic	No	03/01/2017		0	0	0
2012295386	710	rural/clinic	No	03/01/2017		0	0	0
2012267306	710	rural/clinic	No	03/01/2017		0	0	0
2012295387	710	rural/clinic	No	03/01/2017		0	0	0
2014358660	710	rural/clinic	No	03/01/2017		0	0	0
2012358271	710	rural/clinic	No	03/01/2017		0	0	0
2014358663	710	rural/clinic	No	03/01/2017		0	0	0
2012358272	710	rural/clinic	No	03/01/2017		0	0	0
2012358273	710	rural/clinic	No	03/01/2017		0	0	0
2012358274	710	rural/clinic	No	03/01/2017		0	0	0
2010099620	710	rural/clinic	No	03/01/2017		0	0	0
2014347723	710	rural/clinic	No	03/01/2017		0	0	0
2015385370	710	rural/clinic	No	03/01/2017		0	0	0
2014334364	710	rural/clinic	No	03/01/2017		0	0	0
2014311528	710	rural/clinic	No	03/01/2017		0	0	0
2015349058	710	rural/clinic	No	03/01/2017		0	0	0
2012388369	710	rural/clinic	No	03/01/2017		0	0	0
2014339243	710	rural/clinic	No	03/01/2017		0	0	0
2014287964	710	rural/clinic	No	03/01/2017		0	0	0
2012284617	710	rural/clinic	No	03/01/2017		0	0	0
2014339245	710	rural/clinic	No	03/01/2017		0	0	0
2014339244	710	rural/clinic	No	03/01/2017		0	0	0
2015300653	710	rural/clinic	No	03/01/2017		0	0	0
2014355894	710	rural/clinic	No	03/01/2017		0	0	0
2014288419	710	rural/clinic	No	03/01/2017		0	0	0
2014288417	710	rural/clinic	No	03/01/2017		0	0	0
2015300654	710	rural/clinic	No	03/01/2017		0	0	0
2015315970	710	rural/clinic	No	03/01/2017		0	0	0
2014334672	710	rural/clinic	No	03/01/2017		0	0	0
2012281045	710	rural/clinic	No	03/01/2017		0	0	0
2015315971	710	rural/clinic	No	03/01/2017		0	0	0
2014291609	710	rural/clinic	No	03/01/2017		0	0	0
2014291610	710	rural/clinic	No	03/01/2017		0	0	0
2012344490	710	rural/clinic	No	03/01/2017		0	0	0
2015385921	710	rural/clinic	No	03/01/2017		0	0	0
2015385919	710	rural/clinic	No	03/01/2017		0	0	0

2014356149	710	rural/clinic	No	03/01/2017	0	0	0
201066570	710	rural/clinic	No	03/01/2017	0	0	0
2014355895	710	rural/clinic	No	03/01/2017	0	0	0
2014311529	710	rural/clinic	No	03/01/2017	0	0	0
2014339242	710	rural/clinic	No	03/01/2017	0	0	0
2015385371	710	rural/clinic	No	03/01/2017	0	0	0
2015385373	710	rural/clinic	No	03/01/2017	0	0	0
2015385372	710	rural/clinic	No	03/01/2017	0	0	0
2015339217	710	rural/clinic	No	03/01/2017	0	0	0
2015339218	710	rural/clinic	No	03/01/2017	0	0	0
2015339220	710	rural/clinic	No	03/01/2017	0	0	0
2015339221	710	rural/clinic	No	03/01/2017	0	0	0
2014346273	710	rural/clinic	No	03/01/2017	0	0	0
2014344326	710	rural/clinic	No	03/01/2017	0	0	0
2013284891	710	rural/clinic	No	03/01/2017	0	0	0
2012365981	710	rural/clinic	No	03/01/2017	0	0	0
2015360569	710	rural/clinic	No	03/01/2017	0	0	0
2015360568	710	rural/clinic	No	03/01/2017	0	0	0
2015390936	710	rural/clinic	No	03/01/2017	0	0	0
2015360566	710	rural/clinic	No	03/01/2017	0	0	0
2015339219	710	rural/clinic	No	03/01/2017	0	0	0
2011164386	710	rural/clinic	No	03/01/2017	0	0	0
2014368748	710	rural/clinic	No	03/01/2017	0	0	0
2014368747	710	rural/clinic	No	03/01/2017	0	0	0
2015305212	710	rural/clinic	No	03/01/2017	0	0	0
2015305213	710	rural/clinic	No	03/01/2017	0	0	0
2015305214	710	rural/clinic	No	03/01/2017	0	0	0
2015305215	710	rural/clinic	No	03/01/2017	0	0	0
2015305216	710	rural/clinic	No	03/01/2017	0	0	0
2015305217	710	rural/clinic	No	03/01/2017	0	0	0
2015300655	710	rural/clinic	No	03/01/2017	0	0	0
2014383521	710	rural/clinic	No	03/01/2017	0	0	0
2014347724	710	rural/clinic	No	03/01/2017	0	0	0
2012365138	710	rural/clinic	No	03/01/2017	0	0	0
2014334342	710	rural/clinic	No	03/01/2017	0	0	0
2015360706	710	rural/clinic	No	03/01/2017	0	0	0
2014333795	710	rural/clinic	No	03/01/2017	0	0	0
2014319626	710	rural/clinic	No	03/01/2017	0	0	0
2011142490	710	rural/clinic	No	03/01/2017	0	0	0
2015360705	710	rural/clinic	No	03/01/2017	0	0	0
2015377526	710	rural/clinic	No	03/01/2017	0	0	0
2015414635	710	rural/clinic	No	03/01/2017	0	0	0
2015377527	710	rural/clinic	No	03/01/2017	0	0	0
2015377528	710	rural/clinic	No	03/01/2017	0	0	0
2015335403	710	rural/clinic	No	03/01/2017	0	0	0
2015335404	710	rural/clinic	No	03/01/2017	0	0	0
2015414636	710	rural/clinic	No	03/01/2017	0	0	0
2015414637	710	rural/clinic	No	03/01/2017	0	0	0
2015414639	710	rural/clinic	No	03/01/2017	0	0	0
2015335402	710	rural/clinic	No	03/01/2017	0	0	0
2015349913	710	rural/clinic	No	03/01/2017	0	0	0
2015289272	710	rural/clinic	No	03/01/2017	0	0	0
201529518	710	rural/clinic	No	03/01/2017	0	0	0
2014309376	710	rural/clinic	No	03/01/2017	0	0	0
2015294863	710	rural/clinic	No	03/01/2017	0	0	0
2013256358	710	rural/clinic	No	03/01/2017	0	0	0
2015289320	710	rural/clinic	No	03/01/2017	0	0	0
2015289561	710	rural/clinic	No	03/01/2017	0	0	0
2015289274	710	rural/clinic	No	03/01/2017	0	0	0
2012261378	710	rural/clinic	No	03/01/2017	0	0	0
2015355715	710	rural/clinic	No	03/01/2017	0	0	0
2015289319	710	rural/clinic	No	03/01/2017	0	0	0
2015355716	710	rural/clinic	No	03/01/2017	0	0	0
2015377532	710	rural/clinic	No	03/01/2017	0	0	0
2015385595	489	rural/clinic	No	03/01/2017	0	0	0
2015377533	489	rural/clinic	No	03/01/2017	0	0	0
2015385594	489	rural/clinic	No	03/01/2017	0	0	0
2015385588	489	rural/clinic	No	03/01/2017	0	0	0
2015385596	489	rural/clinic	No	03/01/2017	0	0	0
2015377534	489	rural/clinic	No	03/01/2017	0	0	0
2015385589	489	rural/clinic	No	03/01/2017	0	0	0
2013256359	489	rural/clinic	No	03/01/2017	0	0	0
2015377531	489	rural/clinic	No	03/01/2017	0	0	0
2015408657	489	rural/clinic	No	03/01/2017	0	0	0
2015377530	489	rural/clinic	No	03/01/2017	0	0	0
2015408658	489	rural/clinic	No	03/01/2017	0	0	0
2012321324	489	rural/clinic	No	03/01/2017	0	0	0
2015335637	489	rural/clinic	No	03/01/2017	0	0	0
2012261443	489	rural/clinic	No	03/01/2017	0	0	0
2013256253	489	rural/clinic	No	03/01/2017	0	0	0
2015335566	489	rural/clinic	No	03/01/2017	0	0	0
2013256354	489	rural/clinic	No	03/01/2017	0	0	0

2011213198	489	rural/clinic	No	03/01/2017	0	0	0
2015377529	489	rural/clinic	No	03/01/2017	0	0	0
2013256355	489	rural/clinic	No	03/01/2017	0	0	0
2015360704	489	rural/clinic	No	03/01/2017	0	0	0
2014360315	489	rural/clinic	No	03/01/2017	0	0	0
2014360316	489	rural/clinic	No	03/01/2017	0	0	0
2014360317	489	rural/clinic	No	03/01/2017	0	0	0
2015293823	489	rural/clinic	No	03/01/2017	0	0	0
2015294034	489	rural/clinic	No	03/01/2017	0	0	0
2014344600	489	rural/clinic	No	03/01/2017	0	0	0
2014327651	489	rural/clinic	No	03/01/2017	0	0	0
2015293625	489	rural/clinic	No	03/01/2017	0	0	0
2015413575	489	rural/clinic	No	03/01/2017	0	0	0
2015344629	489	rural/clinic	No	03/01/2017	0	0	0
2015344627	489	rural/clinic	No	03/01/2017	0	0	0
2015344625	489	rural/clinic	No	03/01/2017	0	0	0
2015344626	489	rural/clinic	No	03/01/2017	0	0	0
2015344628	489	rural/clinic	No	03/01/2017	0	0	0
2015344630	73	rural/clinic	No	03/01/2017	0	0	0
2015319415	73	rural/clinic	No	03/01/2017	0	0	0
2015319416	73	rural/clinic	No	03/01/2017	0	0	0
2015319412	73	rural/clinic	No	03/01/2017	0	0	0
2015319413	73	rural/clinic	No	03/01/2017	0	0	0
2015412795	73	rural/clinic	No	03/01/2017	0	0	0
2015413796	73	rural/clinic	No	03/01/2017	0	0	0
2015413797	73	rural/clinic	No	03/01/2017	0	0	0
2015413799	73	rural/clinic	No	03/01/2017	0	0	0
2015413800	73	rural/clinic	No	03/01/2017	0	0	0
2015413794	73	rural/clinic	No	03/01/2017	0	0	0
2015344056	73	rural/clinic	No	03/01/2017	0	0	0
2015344057	73	rural/clinic	No	03/01/2017	0	0	0
2015293624	73	rural/clinic	No	03/01/2017	0	0	0
2015314775	73	rural/clinic	No	03/01/2017	0	0	0
2015314776	73	rural/clinic	No	03/01/2017	0	0	0
2014360312	73	rural/clinic	No	03/01/2017	0	0	0
2012322685	73	rural/clinic	No	03/01/2017	0	0	0
2015293626	73	rural/clinic	No	03/01/2017	0	0	0
2014360313	73	rural/clinic	No	03/01/2017	0	0	0
2014360314	73	rural/clinic	No	03/01/2017	0	0	0
2015314779	73	rural/clinic	No	03/01/2017	0	0	0
2015314780	711	rural/clinic	No	03/01/2017	0	0	0
2015303504	711	rural/clinic	No	03/01/2017	0	0	0
2011195901	711	rural/clinic	No	03/01/2017	0	0	0
2015413887	711	rural/clinic	No	03/01/2017	0	0	0
2015413888	711	rural/clinic	No	03/01/2017	0	0	0
2011125792	711	rural/clinic	No	03/01/2017	0	0	0
2015397459	711	rural/clinic	No	03/01/2017	0	0	0
2015397460	711	rural/clinic	No	03/01/2017	0	0	0
2015397461	711	rural/clinic	No	03/01/2017	0	0	0
2015397462	711	rural/clinic	No	03/01/2017	0	0	0
2011191044	711	rural/clinic	No	03/01/2017	0	0	0
2011191045	711	rural/clinic	No	03/01/2017	0	0	0
2015303168	711	rural/clinic	No	03/01/2017	0	0	0
2015303169	711	rural/clinic	No	03/01/2017	0	0	0
2015303170	711	rural/clinic	No	03/01/2017	0	0	0
2012379643	711	rural/clinic	No	03/01/2017	0	0	0
2013271294	711	rural/clinic	No	03/01/2017	0	0	0
2014318823	711	rural/clinic	No	03/01/2017	0	0	0
2014343023	711	rural/clinic	No	03/01/2017	0	0	0
2014377399	711	rural/clinic	No	03/01/2017	0	0	0
2014383964	711	rural/clinic	No	03/01/2017	0	0	0
2015418704	711	rural/clinic	No	03/01/2017	0	0	0
2015418706	711	rural/clinic	No	03/01/2017	0	0	0
2015324217	711	rural/clinic	No	03/01/2017	0	0	0
2015324216	711	rural/clinic	No	03/01/2017	0	0	0
2015413576	711	rural/clinic	No	03/01/2017	0	0	0
2014372210	711	rural/clinic	No	03/01/2017	0	0	0
2014372211	711	rural/clinic	No	03/01/2017	0	0	0
2015344058	711	rural/clinic	No	03/01/2017	0	0	0
2015344059	711	rural/clinic	No	03/01/2017	0	0	0
2015344060	711	rural/clinic	No	03/01/2017	0	0	0
2015344061	711	rural/clinic	No	03/01/2017	0	0	0
2015413529	711	rural/clinic	No	03/01/2017	0	0	0
2015413527	711	rural/clinic	No	03/01/2017	0	0	0
2015294615	711	rural/clinic	No	03/01/2017	0	0	0
2015344631	711	rural/clinic	No	03/01/2017	0	0	0
2015294038	711	rural/clinic	No	03/01/2017	0	0	0
2015294035	711	rural/clinic	No	03/01/2017	0	0	0
2015294037	711	rural/clinic	No	03/01/2017	0	0	0
2015294036	711	rural/clinic	No	03/01/2017	0	0	0
2015294040	711	rural/clinic	No	03/01/2017	0	0	0
2015294039	711	rural/clinic	No	03/01/2017	0	0	0

2014327653	711	rural/clinic	No	03/01/2017	0	0	0
2014327654	711	rural/clinic	No	03/01/2017	0	0	0
2014327655	711	rural/clinic	No	03/01/2017	0	0	0
2014327652	711	rural/clinic	No	03/01/2017	0	0	0
2014313656	711	rural/clinic	No	03/01/2017	0	0	0
2014290713	711	rural/clinic	No	03/01/2017	0	0	0
2014290712	711	rural/clinic	No	03/01/2017	0	0	0
2014290709	711	rural/clinic	No	03/01/2017	0	0	0
2014290711	711	rural/clinic	No	03/01/2017	0	0	0
2014290710	711	rural/clinic	No	03/01/2017	0	0	0
2014358169	711	rural/clinic	No	03/01/2017	0	0	0
2013255251	711	rural/clinic	No	03/01/2017	0	0	0
2014307200	711	rural/clinic	No	03/01/2017	0	0	0
2015384018	711	rural/clinic	No	03/01/2017	0	0	0
2015384019	711	rural/clinic	No	03/01/2017	0	0	0
2014346934	711	rural/clinic	No	03/01/2017	0	0	0
2015377686	711	rural/clinic	No	03/01/2017	0	0	0
2015377687	711	rural/clinic	No	03/01/2017	0	0	0
2015377688	711	rural/clinic	No	03/01/2017	0	0	0
2015377689	711	rural/clinic	No	03/01/2017	0	0	0
2015377690	711	rural/clinic	No	03/01/2017	0	0	0
2012379646	711	rural/clinic	No	03/01/2017	0	0	0
2012363266	711	rural/clinic	No	03/01/2017	0	0	0
2015377691	711	rural/clinic	No	03/01/2017	0	0	0
2012363259	711	rural/clinic	No	03/01/2017	0	0	0
2015373074	711	rural/clinic	No	03/01/2017	0	0	0
2015377692	711	rural/clinic	No	03/01/2017	0	0	0
2014316752	711	rural/clinic	No	03/01/2017	0	0	0
2015373073	711	rural/clinic	No	03/01/2017	0	0	0
2015377693	711	rural/clinic	No	03/01/2017	0	0	0
2015373068	711	rural/clinic	No	03/01/2017	0	0	0
2012379650	711	rural/clinic	No	03/01/2017	0	0	0
2015319418	711	rural/clinic	No	03/01/2017	0	0	0
2014375590	711	rural/clinic	No	03/01/2017	0	0	0
2015350216	711	rural/clinic	No	03/01/2017	0	0	0
2015369911	711	rural/clinic	No	03/01/2017	0	0	0
2015369910	711	rural/clinic	No	03/01/2017	0	0	0
2014368219	711	rural/clinic	No	03/01/2017	0	0	0
2015368859	711	rural/clinic	No	03/01/2017	0	0	0
2015368861	711	rural/clinic	No	03/01/2017	0	0	0
2014315585	711	rural/clinic	No	03/01/2017	0	0	0
2015368862	711	rural/clinic	No	03/01/2017	0	0	0
2015319417	711	rural/clinic	No	03/01/2017	0	0	0
2015369906	711	rural/clinic	No	03/01/2017	0	0	0
2014356950	711	rural/clinic	No	03/01/2017	0	0	0
2011224274	711	rural/clinic	No	03/01/2017	0	0	0
2015369907	711	rural/clinic	No	03/01/2017	0	0	0
2014382413	711	rural/clinic	No	03/01/2017	0	0	0
2011224277	711	rural/clinic	No	03/01/2017	0	0	0
2015377937	711	rural/clinic	No	03/01/2017	0	0	0
2014327101	711	rural/clinic	No	03/01/2017	0	0	0
2014382417	711	rural/clinic	No	03/01/2017	0	0	0
2014382416	711	rural/clinic	No	03/01/2017	0	0	0
2012365466	711	rural/clinic	No	03/01/2017	0	0	0
2012365467	711	rural/clinic	No	03/01/2017	0	0	0
2014382411	711	rural/clinic	No	03/01/2017	0	0	0
2015414916	711	rural/clinic	No	03/01/2017	0	0	0
2011225139	711	rural/clinic	No	03/01/2017	0	0	0
2015377941	136	rural/clinic	No	03/01/2017	0	0	0
2015377940	136	rural/clinic	No	03/01/2017	0	0	0
2015414914	136	rural/clinic	No	03/01/2017	0	0	0
2011224275	136	rural/clinic	No	03/01/2017	0	0	0
2015369763	136	rural/clinic	No	03/01/2017	0	0	0
2015369761	136	rural/clinic	No	03/01/2017	0	0	0
2015377938	136	rural/clinic	No	03/01/2017	0	0	0
2015369762	136	rural/clinic	No	03/01/2017	0	0	0
2012305691	136	rural/clinic	No	03/01/2017	0	0	0
2012263734	136	rural/clinic	No	03/01/2017	0	0	0
2015414915	231	rural/clinic	No	03/01/2017	0	0	0
2012263735	231	rural/clinic	No	03/01/2017	0	0	0
2014344744	231	rural/clinic	No	03/01/2017	0	0	0
2015293910	231	rural/clinic	No	03/01/2017	0	0	0
2012263736	231	rural/clinic	No	03/01/2017	0	0	0
2015293912	231	rural/clinic	No	03/01/2017	0	0	0
2015334039	231	rural/clinic	No	03/01/2017	0	0	0
2015334038	231	rural/clinic	No	03/01/2017	0	0	0
2015334037	231	rural/clinic	No	03/01/2017	0	0	0
2015334036	231	rural/clinic	No	03/01/2017	0	0	0
2012263737	231	rural/clinic	No	03/01/2017	0	0	0
2015334033	231	rural/clinic	No	03/01/2017	0	0	0
2015359384	231	rural/clinic	No	03/01/2017	0	0	0
2015334027	231	rural/clinic	No	03/01/2017	0	0	0

2015334026	231	rural/clinic	No	03/01/2017	0	0	0
2015334025	231	rural/clinic	No	03/01/2017	0	0	0
2015334024	565	rural/clinic	No	03/01/2017	0	0	0
2015334023	565	rural/clinic	No	03/01/2017	0	0	0
2014344743	565	rural/clinic	No	03/01/2017	0	0	0
2014344742	565	rural/clinic	No	03/01/2017	0	0	0
2015294291	565	rural/clinic	No	03/01/2017	0	0	0
2015294292	565	rural/clinic	No	03/01/2017	0	0	0
2015294290	565	rural/clinic	No	03/01/2017	0	0	0
2015294289	565	rural/clinic	No	03/01/2017	0	0	0
2014344697	565	rural/clinic	No	03/01/2017	0	0	0
2014344696	565	rural/clinic	No	03/01/2017	0	0	0
2015311750	24	rural/clinic	No	03/01/2017	0	0	0
2015293911	24	rural/clinic	No	03/01/2017	0	0	0
2012350509	24	rural/clinic	No	03/01/2017	0	0	0
2015294288	24	rural/clinic	No	03/01/2017	0	0	0
201079324	24	rural/clinic	No	03/01/2017	0	0	0
2015294286	24	rural/clinic	No	03/01/2017	0	0	0
2012350512	24	rural/clinic	No	03/01/2017	0	0	0
2014376700	24	rural/clinic	No	03/01/2017	0	0	0
2015324117	24	rural/clinic	No	03/01/2017	0	0	0
2015324116	24	rural/clinic	No	03/01/2017	0	0	0
2015324115	24	rural/clinic	No	03/01/2017	0	0	0
2014291937	24	rural/clinic	No	03/01/2017	0	0	0
2015408166	24	rural/clinic	No	03/01/2017	0	0	0
2015318229	24	rural/clinic	No	03/01/2017	0	0	0
2015299761	24	rural/clinic	No	03/01/2017	0	0	0
2015294284	24	rural/clinic	No	03/01/2017	0	0	0
2015318230	24	rural/clinic	No	03/01/2017	0	0	0
2015335173	24	rural/clinic	No	03/01/2017	0	0	0
2015418625	24	rural/clinic	No	03/01/2017	0	0	0
2015335174	24	rural/clinic	No	03/01/2017	0	0	0
2015335172	24	rural/clinic	No	03/01/2017	0	0	0
2014358661	24	rural/clinic	No	03/01/2017	0	0	0
2015335175	24	rural/clinic	No	03/01/2017	0	0	0
2014358166	24	rural/clinic	No	03/01/2017	0	0	0
2014358666	24	rural/clinic	No	03/01/2017	0	0	0
2014358167	24	rural/clinic	No	03/01/2017	0	0	0
2014329220	24	rural/clinic	No	03/01/2017	0	0	0
2013266234	24	rural/clinic	No	03/01/2017	0	0	0
2014358667	24	rural/clinic	No	03/01/2017	0	0	0
2012242568	24	rural/clinic	No	03/01/2017	0	0	0
2012242571	24	rural/clinic	No	03/01/2017	0	0	0
2012242569	24	rural/clinic	No	03/01/2017	0	0	0
2012242570	24	rural/clinic	No	03/01/2017	0	0	0
2015357247	24	rural/clinic	No	03/01/2017	0	0	0
2015357248	24	rural/clinic	No	03/01/2017	0	0	0
2015357249	24	rural/clinic	No	03/01/2017	0	0	0
2015357246	24	rural/clinic	No	03/01/2017	0	0	0
2015357245	24	rural/clinic	No	03/01/2017	0	0	0
2014320288	24	rural/clinic	No	03/01/2017	0	0	0
2014320290	24	rural/clinic	No	03/01/2017	0	0	0
2015302470	24	rural/clinic	No	03/01/2017	0	0	0
2014358664	24	rural/clinic	No	03/01/2017	0	0	0
2014358665	24	rural/clinic	No	03/01/2017	0	0	0
2014358662	24	rural/clinic	No	03/01/2017	0	0	0
2015418627	24	rural/clinic	No	03/01/2017	0	0	0
2015418628	24	rural/clinic	No	03/01/2017	0	0	0
2015362401	24	rural/clinic	No	03/01/2017	0	0	0
2013256353	24	rural/clinic	No	03/01/2017	0	0	0
2015362402	24	rural/clinic	No	03/01/2017	0	0	0
2015362403	24	rural/clinic	No	03/01/2017	0	0	0
2015305747	24	rural/clinic	No	03/01/2017	0	0	0
2011213199	24	rural/clinic	No	03/01/2017	0	0	0
2013256352	24	rural/clinic	No	03/01/2017	0	0	0
2015305748	24	rural/clinic	No	03/01/2017	0	0	0
2015305749	24	rural/clinic	No	03/01/2017	0	0	0
2011213200	24	rural/clinic	No	03/01/2017	0	0	0
2013256357	24	rural/clinic	No	03/01/2017	0	0	0
2013256356	24	rural/clinic	No	03/01/2017	0	0	0
2013256351	24	rural/clinic	No	03/01/2017	0	0	0
2014317797	24	rural/clinic	No	03/01/2017	0	0	0
2014385224	24	rural/clinic	No	03/01/2017	0	0	0
2014385227	24	rural/clinic	No	03/01/2017	0	0	0
2015365327	24	rural/clinic	No	03/01/2017	0	0	0
2015365330	24	rural/clinic	No	03/01/2017	0	0	0
2015365335	24	rural/clinic	No	03/01/2017	0	0	0
2015408727	24	rural/clinic	No	03/01/2017	0	0	0
2015408731	24	rural/clinic	No	03/01/2017	0	0	0
2015408730	24	rural/clinic	No	03/01/2017	0	0	0
2015408729	24	rural/clinic	No	03/01/2017	0	0	0
2015408728	24	rural/clinic	No	03/01/2017	0	0	0

2013278281	24	rural/clinic	No	03/01/2017	0	0	0
2015392410	24	rural/clinic	No	03/01/2017	0	0	0
2015392509	24	rural/clinic	No	03/01/2017	0	0	0
2015392510	24	rural/clinic	No	03/01/2017	0	0	0
2015392511	24	rural/clinic	No	03/01/2017	0	0	0
2015392512	24	rural/clinic	No	03/01/2017	0	0	0
2015392513	24	rural/clinic	No	03/01/2017	0	0	0
2015343040	24	rural/clinic	No	03/01/2017	0	0	0
2015343042	24	rural/clinic	No	03/01/2017	0	0	0
2015392514	567	rural/clinic	No	03/01/2017	0	0	0
2015373078	567	rural/clinic	No	03/01/2017	0	0	0
2015373079	567	rural/clinic	No	03/01/2017	0	0	0
2015392515	567	rural/clinic	No	03/01/2017	0	0	0
2015373080	567	rural/clinic	No	03/01/2017	0	0	0
2015392517	567	rural/clinic	No	03/01/2017	0	0	0
2015373081	567	rural/clinic	No	03/01/2017	0	0	0
2015373082	567	rural/clinic	No	03/01/2017	0	0	0
2015392518	567	rural/clinic	No	03/01/2017	0	0	0
2015374377	567	rural/clinic	No	03/01/2017	0	0	0
2015392520	567	rural/clinic	No	03/01/2017	0	0	0
2015374378	567	rural/clinic	No	03/01/2017	0	0	0
2015392521	567	rural/clinic	No	03/01/2017	0	0	0
2014296860	567	rural/clinic	No	03/01/2017	0	0	0
2014296861	567	rural/clinic	No	03/01/2017	0	0	0
2014296862	567	rural/clinic	No	03/01/2017	0	0	0
2014296863	567	rural/clinic	No	03/01/2017	0	0	0
2015374379	567	rural/clinic	No	03/01/2017	0	0	0
2015374380	567	rural/clinic	No	03/01/2017	0	0	0
2015374381	567	rural/clinic	No	03/01/2017	0	0	0
2015374384	567	rural/clinic	No	03/01/2017	0	0	0
2015374386	567	rural/clinic	No	03/01/2017	0	0	0
2015374387	567	rural/clinic	No	03/01/2017	0	0	0
2015374389	567	rural/clinic	No	03/01/2017	0	0	0
2015374390	567	rural/clinic	No	06/01/2017	0	0	0
2015374395	567	rural/clinic	No	06/01/2017	0	0	0
2015374393	567	rural/clinic	No	06/01/2017	0	0	0
2015401509	567	rural/clinic	No	06/01/2017	0	0	0
2015401511	567	rural/clinic	No	06/01/2017	0	0	0
2015369462	567	rural/clinic	No	06/01/2017	0	0	0
2015369464	567	rural/clinic	No	06/01/2017	0	0	0
2015401515	567	rural/clinic	No	06/01/2017	0	0	0
2015401516	567	rural/clinic	No	06/01/2017	0	0	0
2015365184	567	rural/clinic	No	06/01/2017	0	0	0
2015322711	567	rural/clinic	No	06/01/2017	0	0	0
2015322712	567	rural/clinic	No	06/01/2017	0	0	0
2015322710	567	rural/clinic	No	06/01/2017	0	0	0
2015343038	567	rural/clinic	No	03/01/2017	0	0	0
2015343031	567	rural/clinic	No	03/01/2017	0	0	0
2012363263	567	rural/clinic	No	03/01/2017	0	0	0
2012363264	85	rural/clinic	No	03/01/2017	0	0	0
2012363256	85	rural/clinic	No	03/01/2017	0	0	0
2012363265	85	rural/clinic	No	03/01/2017	0	0	0
2012363260	85	rural/clinic	No	03/01/2017	0	0	0
2015365331	85	rural/clinic	No	03/01/2017	0	0	0
2015365317	85	rural/clinic	No	03/01/2017	0	0	0
2015365336	85	rural/clinic	No	03/01/2017	0	0	0
2015365318	85	rural/clinic	No	03/01/2017	0	0	0
2015322713	85	rural/clinic	No	03/01/2017	0	0	0
2015365329	85	rural/clinic	No	03/01/2017	0	0	0
2015365321	85	rural/clinic	No	03/01/2017	0	0	0
2015365320	85	rural/clinic	No	03/01/2017	0	0	0
2015343493	85	rural/clinic	No	03/01/2017	0	0	0
2015343494	85	rural/clinic	No	03/01/2017	0	0	0
2013260397	85	rural/clinic	No	03/01/2017	0	0	0
2013260394	85	rural/clinic	No	03/01/2017	0	0	0
2013260392	85	rural/clinic	No	03/01/2017	0	0	0
2015343044	85	rural/clinic	No	03/01/2017	0	0	0
2015365319	85	rural/clinic	No	03/01/2017	0	0	0
2015306816	85	rural/clinic	No	03/01/2017	0	0	0
2015306813	85	rural/clinic	No	03/01/2017	0	0	0
2015306815	85	rural/clinic	No	03/01/2017	0	0	0
2015306814	85	rural/clinic	No	03/01/2017	0	0	0
2015303172	85	rural/clinic	No	03/01/2017	0	0	0
2014365702	85	rural/clinic	No	03/01/2017	0	0	0
2014365703	85	rural/clinic	No	03/01/2017	0	0	0
2015343046	85	rural/clinic	No	03/01/2017	0	0	0
2012295385	149	rural/clinic	No	03/01/2017	0	0	0
2015385370	149	rural/clinic	No	03/01/2017	0	0	0
2015385370	149	rural/clinic	No	03/01/2017	0	0	0
2015322554	149	rural/clinic	No	03/01/2017	0	0	0
2014355894	149	rural/clinic	No	03/01/2017	0	0	0
2014355894	149	rural/clinic	No	03/01/2017	0	0	0

2014288417	149	rural/clinic	No	03/01/2017	0	0	0
2014288417	149	rural/clinic	No	03/01/2017	0	0	0
2015365179	149	rural/clinic	No	03/01/2017	0	0	0
2015365183	149	rural/clinic	No	03/01/2017	0	0	0
2015365182	149	rural/clinic	No	03/01/2017	0	0	0
2015365175	149	rural/clinic	No	03/01/2017	0	0	0
2012379648	149	rural/clinic	No	03/01/2017	0	0	0
2015365171	149	rural/clinic	No	03/01/2017	0	0	0
2015365172	149	rural/clinic	No	03/01/2017	0	0	0
2014296864	149	rural/clinic	No	03/01/2017	0	0	0
2014296865	149	rural/clinic	No	03/01/2017	0	0	0
2014296866	149	rural/clinic	No	03/01/2017	0	0	0
2014296867	149	rural/clinic	No	03/01/2017	0	0	0
2015396247	149	rural/clinic	No	03/01/2017	0	0	0
2012363262	149	rural/clinic	No	03/01/2017	0	0	0
2012363261	149	rural/clinic	No	03/01/2017	0	0	0
2015392405	149	rural/clinic	No	03/01/2017	0	0	0
2012307505	149	rural/clinic	No	03/01/2017	0	0	0
2015305785	149	rural/clinic	No	03/01/2017	0	0	0
2015305784	149	rural/clinic	No	03/01/2017	0	0	0
2015322077	149	rural/clinic	No	03/01/2017	0	0	0
2015322078	149	rural/clinic	No	03/01/2017	0	0	0
2015334028	149	rural/clinic	No	03/01/2017	0	0	0
2015334029	149	rural/clinic	No	03/01/2017	0	0	0
2015334030	149	rural/clinic	No	03/01/2017	0	0	0
2015334032	149	rural/clinic	No	03/01/2017	0	0	0
2015334031	149	rural/clinic	No	03/01/2017	0	0	0
2015334034	149	rural/clinic	No	03/01/2017	0	0	0
2012328990	149	rural/clinic	No	03/01/2017	0	0	0
2012328991	149	rural/clinic	No	03/01/2017	0	0	0
2013254380	149	rural/clinic	No	03/01/2017	0	0	0
2015394610	149	rural/clinic	No	03/01/2017	0	0	0
2015394611	149	rural/clinic	No	03/01/2017	0	0	0
2015394612	149	rural/clinic	No	03/01/2017	0	0	0
2015369467	788	rural/clinic	No	06/01/2017	0	0	0
2012390126	788	rural/clinic	No	06/01/2017	0	0	0
2015302471	788	rural/clinic	No	03/01/2017	0	0	0
2012390128	788	rural/clinic	No	06/01/2017	0	0	0
2012390129	788	rural/clinic	No	06/01/2017	0	0	0
2012390130	788	rural/clinic	No	06/01/2017	0	0	0
2012390131	788	rural/clinic	No	06/01/2017	0	0	0
2015405086	788	rural/clinic	No	06/01/2017	0	0	0
2015302472	788	rural/clinic	No	03/01/2017	0	0	0
2014363859	788	rural/clinic	No	03/01/2017	0	0	0
2014363860	788	rural/clinic	No	03/01/2017	0	0	0
2015362310	568	district/faith-based	No	03/01/2017	1	0	0
2015362311	568	district/faith-based	No	03/01/2017	1	0	0
2015362312	568	district/faith-based	No	03/01/2017	1	0	0
2015362313	568	district/faith-based	No	03/01/2017	1	0	0
2015362314	568	district/faith-based	No	03/01/2017	1	0	0
2015362315	568	district/faith-based	No	03/01/2017	1	0	0
2012353584	568	district/faith-based	No	03/01/2017	1	0	0
2012353585	568	district/faith-based	No	03/01/2017	1	0	0
2012353583	568	district/faith-based	No	03/01/2017	1	0	0
2014319041	568	district/faith-based	No	03/01/2017	1	0	0
2011193470	568	district/faith-based	No	03/01/2017	1	0	0
2011193471	568	district/faith-based	No	03/01/2017	1	0	0
2011193472	568	district/faith-based	No	03/01/2017	1	0	0
2015361779	568	district/faith-based	No	03/01/2017	1	0	0
2015361780	568	district/faith-based	No	03/01/2017	1	0	0
2013270343	568	district/faith-based	No	03/01/2017	1	0	0
2015368120	568	district/faith-based	No	03/01/2017	1	0	0
2015368121	568	district/faith-based	No	03/01/2017	1	0	0
2015368122	568	district/faith-based	No	03/01/2017	1	0	0
2015368123	568	district/faith-based	No	03/01/2017	1	0	0
2015368124	568	district/faith-based	No	03/01/2017	1	0	0
2015368125	568	district/faith-based	No	03/01/2017	1	0	0
2015368126	568	district/faith-based	No	03/01/2017	1	0	0
2013274235	568	district/faith-based	No	03/01/2017	1	0	0
2013274236	568	district/faith-based	No	03/01/2017	1	0	0
2012340196	568	district/faith-based	No	03/01/2017	1	0	0
2015414457	568	district/faith-based	No	03/01/2017	1	0	0
2015372583	568	district/faith-based	No	03/01/2017	1	0	0
2015372586	568	district/faith-based	No	03/01/2017	1	0	0
2015372587	568	district/faith-based	No	03/01/2017	1	0	0
2015372588	568	district/faith-based	No	03/01/2017	1	0	0
2015372589	568	district/faith-based	No	03/01/2017	1	0	0
2015372590	568	district/faith-based	No	03/01/2017	1	0	0
2015372591	568	district/faith-based	No	03/01/2017	1	0	0
2015372595	568	district/faith-based	No	03/01/2017	1	0	0
2015372596	568	district/faith-based	No	03/01/2017	1	0	0
2015372597	568	district/faith-based	No	03/01/2017	1	0	0

2015372598	568	district/faith-based	No	03/01/2017	1	0	0
2015372599	568	district/faith-based	No	03/01/2017	1	0	0
2015372600	568	district/faith-based	No	03/01/2017	1	0	0
2014326615	568	district/faith-based	No	03/01/2017	1	0	0
2015413617	568	district/faith-based	No	03/01/2017	1	0	0
2015413618	568	district/faith-based	No	03/01/2017	1	0	0
2015413619	568	district/faith-based	No	03/01/2017	1	0	0
2015413620	568	district/faith-based	No	03/01/2017	1	0	0
2015344826	568	district/faith-based	No	03/01/2017	1	0	0
2015344828	568	district/faith-based	No	03/01/2017	1	0	0
2015418629	568	district/faith-based	No	03/01/2017	1	0	0
2015349914	568	district/faith-based	No	03/01/2017	1	0	0
2015349915	568	district/faith-based	No	03/01/2017	1	0	0
2011224432	568	district/faith-based	No	03/01/2017	1	0	0
2011224433	568	district/faith-based	No	03/01/2017	1	0	0
2014368013	568	district/faith-based	No	03/01/2017	1	0	0
2012304195	568	district/faith-based	No	03/01/2017	1	0	0
2011225347	568	district/faith-based	No	03/01/2017	1	0	0
2015344829	568	district/faith-based	No	03/01/2017	1	0	0
2015344827	568	district/faith-based	No	03/01/2017	1	0	0
2014383027	568	district/faith-based	No	03/01/2017	1	0	0
2015301076	568	district/faith-based	No	03/01/2017	1	0	0
2015301077	568	district/faith-based	No	03/01/2017	1	0	0
2015301078	568	district/faith-based	No	03/01/2017	1	0	0
2014323098	568	district/faith-based	No	03/01/2017	1	0	0
2014323099	568	district/faith-based	No	03/01/2017	1	0	0
2014323100	568	district/faith-based	No	03/01/2017	1	0	0
2015383568	568	district/faith-based	No	03/01/2017	1	0	0
2015383567	568	district/faith-based	No	03/01/2017	1	0	0
2014382908	568	district/faith-based	No	03/01/2017	1	0	0
2015365322	568	district/faith-based	No	03/01/2017	1	0	0
2015365323	568	district/faith-based	No	03/01/2017	1	0	0
2015365324	568	district/faith-based	No	03/01/2017	1	0	0
2015365325	568	district/faith-based	No	05/01/2017	1	0	0
2015365326	568	district/faith-based	No	05/01/2017	1	0	0
2015365328	568	district/faith-based	No	05/01/2017	1	0	0
2015365332	568	district/faith-based	No	05/01/2017	1	0	0
2015365333	568	district/faith-based	No	05/01/2017	1	0	0
2015365334	568	district/faith-based	No	05/01/2017	1	0	0
2015365337	568	district/faith-based	No	05/01/2017	1	0	0
2013267189	568	district/faith-based	No	05/01/2017	1	0	0
2015352983	568	district/faith-based	No	05/01/2017	1	0	0
2015335093	568	district/faith-based	No	05/01/2017	1	0	0
2015335095	568	district/faith-based	No	05/01/2017	1	0	0
2015335096	568	district/faith-based	No	05/01/2017	1	0	0
2015384519	568	district/faith-based	No	05/01/2017	1	0	0
2015384520	568	district/faith-based	No	05/01/2017	1	0	0
2015384521	568	district/faith-based	No	05/01/2017	1	0	0
2015369468	568	district/faith-based	No	05/01/2017	1	0	0
2015319414	568	district/faith-based	No	03/01/2017	1	0	0
2015319414	568	district/faith-based	No	03/01/2017	1	0	0
2015319416	568	district/faith-based	No	03/01/2017	1	0	0
2015319416	568	district/faith-based	No	03/01/2017	1	0	0
2015384019	568	district/faith-based	No	03/01/2017	1	0	0
2015384019	568	district/faith-based	No	03/01/2017	1	0	0
2015368859	568	district/faith-based	No	03/01/2017	1	0	0
2015368859	568	district/faith-based	No	03/01/2017	1	0	0
2014317797	568	district/faith-based	No	03/01/2017	1	0	0
2014317797	568	district/faith-based	No	03/01/2017	1	0	0
2015414915	568	district/faith-based	No	03/01/2017	1	0	0
2015414915	568	district/faith-based	No	03/01/2017	1	0	0
2015413794	568	district/faith-based	No	03/01/2017	1	0	0
2015413794	568	district/faith-based	No	03/01/2017	1	0	0
2015314776	568	district/faith-based	No	03/01/2017	1	0	0
2015314776	568	district/faith-based	No	03/01/2017	1	0	0
2015334042	568	district/faith-based	No	06/01/2017	1	0	0
2015334044	568	district/faith-based	No	06/01/2017	1	0	0
2015334045	568	district/faith-based	No	06/01/2017	1	0	0
2015348879	568	district/faith-based	No	06/01/2017	1	0	0
2015348880	568	district/faith-based	No	06/01/2017	1	0	0
2014338227	568	district/faith-based	No	06/01/2017	1	0	0
2014338229	568	district/faith-based	No	06/01/2017	1	0	0
2013267187	568	district/faith-based	No	06/01/2017	1	0	0
2013267188	568	district/faith-based	No	06/01/2017	1	0	0
2013267190	568	district/faith-based	No	06/01/2017	1	0	0
2015295322	568	district/faith-based	No	06/01/2017	1	0	0
2015408732	568	district/faith-based	No	06/01/2017	1	0	0
2015365190	568	district/faith-based	No	10/01/2017	1	0	0
2015408737	568	district/faith-based	No	06/01/2017	1	0	0
2015343050	568	district/faith-based	No	10/01/2017	1	0	0
2015369469	568	district/faith-based	No	05/01/2017	1	0	0
2015408734	105	rural/clinic	No	10/01/2017	0	0	0

2015413058	105	rural/clinic	No	05/01/2017	0	0	0
2015408736	105	rural/clinic	No	10/01/2017	0	0	0
2015413059	105	rural/clinic	No	05/01/2017	0	0	0
2015413060	105	rural/clinic	No	05/01/2017	0	0	0
2015413061	105	rural/clinic	No	05/01/2017	0	0	0
2015365191	105	rural/clinic	No	10/01/2017	0	0	0
2015413062	105	rural/clinic	No	05/01/2017	0	0	0
2015365181	105	rural/clinic	No	10/01/2017	0	0	0
2015365174	105	rural/clinic	No	10/01/2017	0	0	0
2015365189	105	rural/clinic	No	10/01/2017	0	0	0
2015413063	105	rural/clinic	No	05/01/2017	0	0	0
2015373324	105	rural/clinic	No	05/01/2017	0	0	0
2015365052	105	rural/clinic	No	10/01/2017	0	0	0
2015373325	105	rural/clinic	No	05/01/2017	0	0	0
2015365192	105	rural/clinic	No	10/01/2017	0	0	0
2014370423	105	rural/clinic	No	05/01/2017	0	0	0
2014345832	105	rural/clinic	No	05/01/2017	0	0	0
2014370422	105	rural/clinic	No	05/01/2017	0	0	0
2014345831	105	rural/clinic	No	05/01/2017	0	0	0
2015343047	105	rural/clinic	No	10/01/2017	0	0	0
2015373097	105	rural/clinic	No	10/01/2017	0	0	0
2015373095	105	rural/clinic	No	10/01/2017	0	0	0
2015373091	105	rural/clinic	No	10/01/2017	0	0	0
2015373090	105	rural/clinic	No	10/01/2017	0	0	0
2015373089	105	rural/clinic	No	10/01/2017	0	0	0
2015373087	105	rural/clinic	No	10/01/2017	0	0	0
2014369136	105	rural/clinic	No	10/01/2017	0	0	0
2015314784	105	rural/clinic	No	10/01/2017	0	0	0
2015314785	105	rural/clinic	No	10/01/2017	0	0	0
2015314788	105	rural/clinic	No	10/01/2017	0	0	0
2015314787	105	rural/clinic	No	10/01/2017	0	0	0
2015314786	105	rural/clinic	No	10/01/2017	0	0	0
2015303173	105	rural/clinic	No	10/01/2017	0	0	0
2014326731	105	rural/clinic	No	10/01/2017	0	0	0
2015372592	105	rural/clinic	No	10/01/2017	0	0	0
2015372594	105	rural/clinic	No	10/01/2017	0	0	0
2015369158	105	rural/clinic	No	10/01/2017	0	0	0
2015372593	105	rural/clinic	No	10/01/2017	0	0	0
2015369160	412	rural/clinic	No	10/01/2017	0	0	0
2015369159	412	rural/clinic	No	10/01/2017	0	0	0
2015368405	412	rural/clinic	No	10/01/2017	0	0	0
2015368401	412	rural/clinic	No	10/01/2017	0	0	0
2013253927	412	rural/clinic	No	10/01/2017	0	0	0
2013254100	412	rural/clinic	No	10/01/2017	0	0	0
2015373094	412	rural/clinic	No	10/01/2017	0	0	0
2015373092	412	rural/clinic	No	10/01/2017	0	0	0
2015364503	412	rural/clinic	No	10/01/2017	0	0	0
2015322080	412	rural/clinic	No	10/01/2017	0	0	0
2015369364	412	rural/clinic	No	05/01/2017	0	0	0
2015369365	412	rural/clinic	No	05/01/2017	0	0	0
2015379006	412	rural/clinic	No	05/01/2017	0	0	0
2015379007	522	rural/clinic	No	05/01/2017	0	0	0
2015379008	522	rural/clinic	No	05/01/2017	0	0	0
2015379009	522	rural/clinic	No	05/01/2017	0	0	0
2015379010	522	rural/clinic	No	05/01/2017	0	0	0
2015379011	522	rural/clinic	No	05/01/2017	0	0	0
2015379012	522	rural/clinic	No	05/01/2017	0	0	0
2015379013	522	rural/clinic	No	05/01/2017	0	0	0
2015379014	522	rural/clinic	No	05/01/2017	0	0	0
2015379015	522	rural/clinic	No	05/01/2017	0	0	0
2015379016	522	rural/clinic	No	05/01/2017	0	0	0
2015379017	522	rural/clinic	No	05/01/2017	0	0	0
2015382454	522	rural/clinic	No	05/01/2017	0	0	0
2015382455	522	rural/clinic	No	05/01/2017	0	0	0
2015382456	522	rural/clinic	No	05/01/2017	0	0	0
2015382457	522	rural/clinic	No	05/01/2017	0	0	0
2015382458	522	rural/clinic	No	05/01/2017	0	0	0
2015382459	522	rural/clinic	No	05/01/2017	0	0	0
2015382460	522	rural/clinic	No	05/01/2017	0	0	0
2015382461	522	rural/clinic	No	05/01/2017	0	0	0
2015384021	522	rural/clinic	No	05/01/2017	0	0	0
2014346935	522	rural/clinic	No	05/01/2017	0	0	0
2015346354	522	rural/clinic	No	05/01/2017	0	0	0
2015346355	522	rural/clinic	No	05/01/2017	0	0	0
2012377873	522	rural/clinic	No	05/01/2017	0	0	0
2014333545	522	rural/clinic	No	05/01/2017	0	0	0
2014339392	522	rural/clinic	No	05/01/2017	0	0	0
2014339393	522	rural/clinic	No	05/01/2017	0	0	0
2014300529	522	rural/clinic	No	05/01/2017	0	0	0
2015339453	522	rural/clinic	No	05/01/2017	0	0	0
2013264338	522	rural/clinic	No	05/01/2017	0	0	0
2014297780	522	rural/clinic	No	05/01/2017	0	0	0

2015294140	522	rural/clinic	No	05/01/2017	0	0	0
2015331657	522	rural/clinic	No	05/01/2017	0	0	0
2015331658	522	rural/clinic	No	05/01/2017	0	0	0
2015331659	522	rural/clinic	No	05/01/2017	0	0	0
2012379339	522	rural/clinic	No	05/01/2017	0	0	0
2015331706	522	rural/clinic	No	05/01/2017	0	0	0
2014296399	522	rural/clinic	No	05/01/2017	0	0	0
2014300528	522	rural/clinic	No	05/01/2017	0	0	0
2014296400	522	rural/clinic	No	05/01/2017	0	0	0
2014340262	522	rural/clinic	No	05/01/2017	0	0	0
2015331601	522	rural/clinic	No	05/01/2017	0	0	0
2014300420	522	rural/clinic	No	05/01/2017	0	0	0
2014296383	522	rural/clinic	No	05/01/2017	0	0	0
2014296372	522	rural/clinic	No	05/01/2017	0	0	0
2013261745	522	rural/clinic	No	05/01/2017	0	0	0
2014296393	522	rural/clinic	No	05/01/2017	0	0	0
2013264339	522	rural/clinic	No	05/01/2017	0	0	0
2015331705	522	rural/clinic	No	05/01/2017	0	0	0
2015331709	522	rural/clinic	No	05/01/2017	0	0	0
2015331708	522	rural/clinic	No	05/01/2017	0	0	0
2012369962	522	rural/clinic	No	05/01/2017	0	0	0
2015319552	522	rural/clinic	No	05/01/2017	0	0	0
2015319553	522	rural/clinic	No	05/01/2017	0	0	0
2015319554	522	rural/clinic	No	05/01/2017	0	0	0
2015319555	522	rural/clinic	No	05/01/2017	0	0	0
2015319556	522	rural/clinic	No	05/01/2017	0	0	0
2015319557	378	rural/clinic	No	05/01/2017	0	0	0
2015319558	378	rural/clinic	No	05/01/2017	0	0	0
2015319559	378	rural/clinic	No	05/01/2017	0	0	0
2015319560	378	rural/clinic	No	05/01/2017	0	0	0
2014338231	378	rural/clinic	No	05/01/2017	0	0	0
2015288349	378	rural/clinic	No	05/01/2017	0	0	0
2012291828	378	rural/clinic	No	05/01/2017	0	0	0
2015418707	378	rural/clinic	No	05/01/2017	0	0	0
2015418705	378	rural/clinic	No	05/01/2017	0	0	0
2012291827	378	rural/clinic	No	05/01/2017	0	0	0
2015408165	378	rural/clinic	No	05/01/2017	0	0	0
2015352407	378	rural/clinic	No	05/01/2017	0	0	0
2014372264	378	rural/clinic	No	05/01/2017	0	0	0
2014372263	378	rural/clinic	No	05/01/2017	0	0	0
2014372261	378	rural/clinic	No	05/01/2017	0	0	0
2014372262	378	rural/clinic	No	05/01/2017	0	0	0
2014306987	378	rural/clinic	No	05/01/2017	0	0	0
2013255739	378	rural/clinic	No	05/01/2017	0	0	0
2014297896	378	rural/clinic	No	09/01/2017	0	0	0
2014297897	378	rural/clinic	No	09/01/2017	0	0	0
2011240267	378	rural/clinic	No	09/01/2017	0	0	0
2011240268	378	rural/clinic	No	09/01/2017	0	0	0
2011240269	378	rural/clinic	No	09/01/2017	0	0	0
2011240270	378	rural/clinic	No	09/01/2017	0	0	0
2014370214	378	rural/clinic	No	11/01/2017	0	0	0
2015359391	378	rural/clinic	No	11/01/2017	0	0	0
2015418939	378	rural/clinic	No	11/01/2017	0	0	0
2014344699	378	rural/clinic	No	11/01/2017	0	0	0
2011212590	378	rural/clinic	No	11/01/2017	0	0	0
2015344310	378	rural/clinic	No	11/01/2017	0	0	0
2015413355	378	rural/clinic	No	11/01/2017	0	0	0
2015359389	378	rural/clinic	No	11/01/2017	0	0	0
2014295873	378	rural/clinic	No	11/01/2017	0	0	0
2011240275	378	rural/clinic	No	09/01/2017	0	0	0
2011240276	378	rural/clinic	No	09/01/2017	0	0	0
2011240278	378	rural/clinic	No	09/01/2017	0	0	0
2011240279	378	rural/clinic	No	09/01/2017	0	0	0
2011240280	378	rural/clinic	No	09/01/2017	0	0	0
2011240281	378	rural/clinic	No	09/01/2017	0	0	0
2011240282	378	rural/clinic	No	09/01/2017	0	0	0
2011240283	378	rural/clinic	No	09/01/2017	0	0	0
2011240284	378	rural/clinic	No	09/01/2017	0	0	0
2011240285	378	rural/clinic	No	09/01/2017	0	0	0
2011240289	378	rural/clinic	No	09/01/2017	0	0	0
2015303171	378	rural/clinic	No	10/01/2017	0	0	0
2015351116	378	rural/clinic	No	10/01/2017	0	0	0
2015351117	378	rural/clinic	No	10/01/2017	0	0	0
2015351118	378	rural/clinic	No	10/01/2017	0	0	0
2015351119	378	rural/clinic	No	10/01/2017	0	0	0
2015351120	378	rural/clinic	No	10/01/2017	0	0	0
2013254140	378	rural/clinic	No	10/01/2017	0	0	0
2013254139	378	rural/clinic	No	10/01/2017	0	0	0
2015286577	378	rural/clinic	No	11/01/2017	0	0	0
2015286578	378	rural/clinic	No	11/01/2017	0	0	0
2015286579	378	rural/clinic	No	11/01/2017	0	0	0
2015286580	378	rural/clinic	No	11/01/2017	0	0	0

2015378716	378	rural/clinic	No	11/01/2017	0	0	0
2014341796	378	rural/clinic	No	11/01/2017	0	0	0
2014341797	378	rural/clinic	No	11/01/2017	0	0	0
2015377694	378	rural/clinic	No	11/01/2017	0	0	0
2015377695	378	rural/clinic	No	11/01/2017	0	0	0
2015377696	378	rural/clinic	No	11/01/2017	0	0	0
2015377697	378	rural/clinic	No	11/01/2017	0	0	0
2015377698	378	rural/clinic	No	11/01/2017	0	0	0
2015335643	378	rural/clinic	No	11/01/2017	0	0	0
2015335644	378	rural/clinic	No	11/01/2017	0	0	0
2014375592	378	rural/clinic	No	11/01/2017	0	0	0
2014375594	378	rural/clinic	No	11/01/2017	0	0	0
2015368801	378	rural/clinic	No	11/01/2017	0	0	0
2015368802	378	rural/clinic	No	11/01/2017	0	0	0
2015390255	378	rural/clinic	No	11/01/2017	0	0	0
2014375591	378	rural/clinic	No	11/01/2017	0	0	0
2014375593	378	rural/clinic	No	11/01/2017	0	0	0
2015368803	378	rural/clinic	No	11/01/2017	0	0	0
2013266105	378	rural/clinic	No	11/01/2017	0	0	0
2013266106	378	rural/clinic	No	11/01/2017	0	0	0
2012363258	378	rural/clinic	No	11/01/2017	0	0	0
2014302187	378	rural/clinic	No	11/01/2017	0	0	0
2014329132	378	rural/clinic	No	11/01/2017	0	0	0
2014369029	378	rural/clinic	No	11/01/2017	0	0	0
2014358668	378	rural/clinic	No	11/01/2017	0	0	0
2014329133	378	rural/clinic	No	11/01/2017	0	0	0
2012361531	378	rural/clinic	No	11/01/2017	0	0	0
2012363268	378	rural/clinic	No	11/01/2017	0	0	0
2014369000	378	rural/clinic	No	11/01/2017	0	0	0
2014368997	378	rural/clinic	No	11/01/2017	0	0	0
2014329131	378	rural/clinic	No	11/01/2017	0	0	0
2014368996	378	rural/clinic	No	11/01/2017	0	0	0
2012353592	378	rural/clinic	No	11/01/2017	0	0	0
2012353590	378	rural/clinic	No	11/01/2017	0	0	0
2012353591	378	rural/clinic	No	11/01/2017	0	0	0
2012353593	378	rural/clinic	No	11/01/2017	0	0	0
2012364930	378	rural/clinic	No	11/01/2017	0	0	0
2012364931	378	rural/clinic	No	11/01/2017	0	0	0
2015405704	378	rural/clinic	No	11/01/2017	0	0	0
2012314005	378	rural/clinic	No	11/01/2017	0	0	0
2012314006	378	rural/clinic	No	11/01/2017	0	0	0
2015369366	378	rural/clinic	No	11/01/2017	0	0	0
2015369367	378	rural/clinic	No	11/01/2017	0	0	0
2015342303	378	rural/clinic	No	11/01/2017	0	0	0
2015390937	378	rural/clinic	No	11/01/2017	0	0	0
2012245714	378	rural/clinic	No	11/01/2017	0	0	0
2012245715	378	rural/clinic	No	11/01/2017	0	0	0
2012245716	378	rural/clinic	No	11/01/2017	0	0	0
2014358171	378	rural/clinic	No	11/01/2017	0	0	0
2014358170	378	rural/clinic	No	11/01/2017	0	0	0
2014358172	378	rural/clinic	No	11/01/2017	0	0	0
2014311428	378	rural/clinic	No	11/01/2017	0	0	0
2014311429	378	rural/clinic	No	11/01/2017	0	0	0
2014311430	378	rural/clinic	No	11/01/2017	0	0	0
2014311431	378	rural/clinic	No	11/01/2017	0	0	0
2014311432	378	rural/clinic	No	11/01/2017	0	0	0
2014356843	378	rural/clinic	No	11/01/2017	0	0	0
2015331756	378	rural/clinic	No	11/01/2017	0	0	0
2014329223	378	rural/clinic	No	11/01/2017	0	0	0
2014358669	378	rural/clinic	No	11/01/2017	0	0	0
2014368995	378	rural/clinic	No	11/01/2017	0	0	0
2014368998	378	rural/clinic	No	11/01/2017	0	0	0
2012363255	378	rural/clinic	No	11/01/2017	0	0	0
2012363267	378	rural/clinic	No	11/01/2017	0	0	0
2013273639	378	rural/clinic	No	13/01/2017	0	0	0
2013260392	378	rural/clinic	No	03/01/2017	0	0	0
2013260392	378	rural/clinic	No	03/01/2017	0	0	0
2014309376	378	rural/clinic	No	03/01/2017	0	0	0
2014309376	378	rural/clinic	No	03/01/2017	0	0	0
2015377534	378	rural/clinic	No	03/01/2017	0	0	0
2015377534	378	rural/clinic	No	03/01/2017	0	0	0
2015360569	378	rural/clinic	No	03/01/2017	0	0	0
2015360569	378	rural/clinic	No	03/01/2017	0	0	0
2015335402	378	rural/clinic	No	03/01/2017	0	0	0
2015335402	378	rural/clinic	No	03/01/2017	0	0	0
2014368993	378	rural/clinic	No	11/01/2017	0	0	0
2014368999	378	rural/clinic	No	11/01/2017	0	0	0
2014369184	378	rural/clinic	No	11/01/2017	0	0	0
2011115897	378	rural/clinic	No	11/01/2017	0	0	0
2011115898	378	rural/clinic	No	11/01/2017	0	0	0
2014358670	378	rural/clinic	No	11/01/2017	0	0	0
2012379637	378	rural/clinic	No	11/01/2017	0	0	0

2012379635	378	rural/clinic	No	11/01/2017	0	0	0
2014368994	378	rural/clinic	No	11/01/2017	0	0	0
2015339223	378	rural/clinic	No	11/01/2017	0	0	0
2015339222	378	rural/clinic	No	11/01/2017	0	0	0
2012353588	378	rural/clinic	No	11/01/2017	0	0	0
2012353586	378	rural/clinic	No	11/01/2017	0	0	0
2012353587	378	rural/clinic	No	11/01/2017	0	0	0
2012353589	378	rural/clinic	No	11/01/2017	0	0	0
2014329224	378	rural/clinic	No	11/01/2017	0	0	0
2014329221	378	rural/clinic	No	11/01/2017	0	0	0
2014329225	378	rural/clinic	No	11/01/2017	0	0	0
2014329226	378	rural/clinic	No	11/01/2017	0	0	0
2012361046	378	rural/clinic	No	11/01/2017	0	0	0
2012291827	378	rural/clinic	No	05/01/2017	0	0	0
2012291827	378	rural/clinic	No	05/01/2017	0	0	0
2014323100	378	rural/clinic	No	03/01/2017	0	0	0
2014323100	378	rural/clinic	No	03/01/2017	0	0	0
2012304195	378	rural/clinic	No	03/01/2017	0	0	0
2012304195	378	rural/clinic	No	03/01/2017	0	0	0
2011225347	378	rural/clinic	No	03/01/2017	0	0	0
2011225347	378	rural/clinic	No	03/01/2017	0	0	0
2015291897	378	rural/clinic	No	11/01/2017	0	0	0
2015291899	378	rural/clinic	No	11/01/2017	0	0	0
2015372583	378	rural/clinic	No	03/01/2017	0	0	0
2015372583	378	rural/clinic	No	03/01/2017	0	0	0
2011141544	378	rural/clinic	No	11/01/2017	0	0	0
2014289463	378	rural/clinic	No	11/01/2017	0	0	0
2014289462	378	rural/clinic	No	11/01/2017	0	0	0
2015291900	378	rural/clinic	No	11/01/2017	0	0	0
2014289461	378	rural/clinic	No	11/01/2017	0	0	0
2014289459	378	rural/clinic	No	11/01/2017	0	0	0
2011141545	378	rural/clinic	No	11/01/2017	0	0	0
2014289464	378	rural/clinic	No	11/01/2017	0	0	0
2014289465	378	rural/clinic	No	11/01/2017	0	0	0
2011141542	378	rural/clinic	No	11/01/2017	0	0	0
2014308885	378	rural/clinic	No	11/01/2017	0	0	0
2011152391	378	rural/clinic	No	11/01/2017	0	0	0
2011152392	378	rural/clinic	No	11/01/2017	0	0	0
2014326729	378	rural/clinic	No	11/01/2017	0	0	0
2014326730	378	rural/clinic	No	11/01/2017	0	0	0
2015405906	378	rural/clinic	No	11/01/2017	0	0	0
2012369012	378	rural/clinic	No	11/01/2017	0	0	0
2014365370	378	rural/clinic	No	11/01/2017	0	0	0
2011152225	378	rural/clinic	No	11/01/2017	0	0	0
2012330875	378	rural/clinic	No	11/01/2017	0	0	0
2014326728	378	rural/clinic	No	11/01/2017	0	0	0
2014365369	378	rural/clinic	No	11/01/2017	0	0	0
2015405407	378	rural/clinic	No	11/01/2017	0	0	0
2012330874	378	rural/clinic	No	11/01/2017	0	0	0
2014295874	378	rural/clinic	No	11/01/2017	0	0	0
2015405961	378	rural/clinic	No	11/01/2017	0	0	0
2015405960	378	rural/clinic	No	11/01/2017	0	0	0
2014326727	378	rural/clinic	No	11/01/2017	0	0	0
2014326726	378	rural/clinic	No	11/01/2017	0	0	0
2012369014	378	rural/clinic	No	11/01/2017	0	0	0
2012369013	378	rural/clinic	No	11/01/2017	0	0	0
2012369016	378	rural/clinic	No	11/01/2017	0	0	0
2015405905	378	rural/clinic	No	11/01/2017	0	0	0
2013261288	378	rural/clinic	No	11/01/2017	0	0	0
2015289275	378	rural/clinic	No	11/01/2017	0	0	0
2013256364	378	rural/clinic	No	11/01/2017	0	0	0
2015377538	378	rural/clinic	No	11/01/2017	0	0	0
2015377537	378	rural/clinic	No	11/01/2017	0	0	0
2014309378	378	rural/clinic	No	11/01/2017	0	0	0
2014339394	378	rural/clinic	No	05/01/2017	0	0	0
201438611	378	rural/clinic	No	05/01/2017	0	0	0
2014386114	378	rural/clinic	No	05/01/2017	0	0	0
2014386115	378	rural/clinic	No	05/01/2017	0	0	0
2014386116	378	rural/clinic	No	05/01/2017	0	0	0
2014386117	378	rural/clinic	No	05/01/2017	0	0	0
2011155219	378	rural/clinic	No	05/01/2017	0	0	0
2013256369	378	rural/clinic	No	11/01/2017	0	0	0
2013256368	378	rural/clinic	No	11/01/2017	0	0	0
2015295187	378	rural/clinic	No	11/01/2017	0	0	0
2013256367	378	rural/clinic	No	11/01/2017	0	0	0
2015335568	378	rural/clinic	No	11/01/2017	0	0	0
2014363583	378	rural/clinic	No	11/01/2017	0	0	0
2015355717	378	rural/clinic	No	11/01/2017	0	0	0
2015355718	378	rural/clinic	No	11/01/2017	0	0	0
2015355766	378	rural/clinic	No	11/01/2017	0	0	0
2012317310	378	rural/clinic	No	11/01/2017	0	0	0
2012321326	378	rural/clinic	No	11/01/2017	0	0	0

2012321327	378	rural/clinic	No	11/01/2017	0	0	0
2014359726	378	rural/clinic	No	11/01/2017	0	0	0
2015385598	378	rural/clinic	No	11/01/2017	0	0	0
2015289562	378	rural/clinic	No	11/01/2017	0	0	0
2015295185	378	rural/clinic	No	11/01/2017	0	0	0
2015295188	378	rural/clinic	No	11/01/2017	0	0	0
2015335408	378	rural/clinic	No	11/01/2017	0	0	0
2015335570	378	rural/clinic	No	11/01/2017	0	0	0
2014343864	378	rural/clinic	No	11/01/2017	0	0	0
2013256361	378	rural/clinic	No	11/01/2017	0	0	0
2013256362	378	rural/clinic	No	11/01/2017	0	0	0
2014309377	378	rural/clinic	No	11/01/2017	0	0	0
2015335407	378	rural/clinic	No	11/01/2017	0	0	0
2015362404	378	rural/clinic	No	11/01/2017	0	0	0
2013256365	378	rural/clinic	No	11/01/2017	0	0	0
2015355767	378	rural/clinic	No	11/01/2017	0	0	0
2015355768	378	rural/clinic	No	11/01/2017	0	0	0
2015335569	378	rural/clinic	No	11/01/2017	0	0	0
2015377535	378	rural/clinic	No	11/01/2017	0	0	0
2015377536	378	rural/clinic	No	11/01/2017	0	0	0
2015295186	378	rural/clinic	No	11/01/2017	0	0	0
2012305690	378	rural/clinic	No	11/01/2017	0	0	0
2012263733	378	rural/clinic	No	11/01/2017	0	0	0
2015331169	378	rural/clinic	No	11/01/2017	0	0	0
2012284226	378	rural/clinic	No	11/01/2017	0	0	0
2014347725	378	rural/clinic	No	11/01/2017	0	0	0
2014287965	378	rural/clinic	No	11/01/2017	0	0	0
2015368768	378	rural/clinic	No	11/01/2017	0	0	0
2015368769	378	rural/clinic	No	11/01/2017	0	0	0
2015413329	378	rural/clinic	No	11/01/2017	0	0	0
2014337216	378	rural/clinic	No	11/01/2017	0	0	0
2013264382	378	rural/clinic	No	11/01/2017	0	0	0
2015303105	378	rural/clinic	No	11/01/2017	0	0	0
2015303107	378	rural/clinic	No	11/01/2017	0	0	0
2014336893	378	rural/clinic	No	11/01/2017	0	0	0
2014355148	378	rural/clinic	No	11/01/2017	0	0	0
2014355149	378	rural/clinic	No	11/01/2017	0	0	0
2014355150	378	rural/clinic	No	11/01/2017	0	0	0
2012293696	378	rural/clinic	No	11/01/2017	0	0	0
2012290393	378	rural/clinic	No	11/01/2017	0	0	0
2014336939	378	rural/clinic	No	11/01/2017	0	0	0
2015413326	378	rural/clinic	No	11/01/2017	0	0	0
2011114841	378	rural/clinic	No	11/01/2017	0	0	0
2015413328	378	rural/clinic	No	11/01/2017	0	0	0
2015413327	378	rural/clinic	No	11/01/2017	0	0	0
2014365072	378	rural/clinic	No	11/01/2017	0	0	0
2014365074	378	rural/clinic	No	11/01/2017	0	0	0
2014365075	378	rural/clinic	No	11/01/2017	0	0	0
2014336822	378	rural/clinic	No	11/01/2017	0	0	0
2012314565	378	rural/clinic	No	11/01/2017	0	0	0
2011153306	378	rural/clinic	No	11/01/2017	0	0	0
2015303108	378	rural/clinic	No	11/01/2017	0	0	0
2015303109	413	rural/clinic	No	11/01/2017	0	0	0
2014336817	413	rural/clinic	No	11/01/2017	0	0	0
2012387236	413	rural/clinic	No	11/01/2017	0	0	0
2012387237	413	rural/clinic	No	11/01/2017	0	0	0
2012387235	413	rural/clinic	No	11/01/2017	0	0	0
2012295388	413	rural/clinic	No	11/01/2017	0	0	0
2012295389	413	rural/clinic	No	11/01/2017	0	0	0
2012295390	413	rural/clinic	No	11/01/2017	0	0	0
2012295391	413	rural/clinic	No	11/01/2017	0	0	0
2012295392	413	rural/clinic	No	11/01/2017	0	0	0
2015346357	413	rural/clinic	No	11/01/2017	0	0	0
2015463358	413	rural/clinic	No	11/01/2017	0	0	0
2013255253	413	rural/clinic	No	11/01/2017	0	0	0
2013255252	413	rural/clinic	No	11/01/2017	0	0	0
2015340821	413	rural/clinic	No	11/01/2017	0	0	0
2012306335	413	rural/clinic	No	11/01/2017	0	0	0
2012306336	413	rural/clinic	No	11/01/2017	0	0	0
2015357623	413	rural/clinic	No	11/01/2017	0	0	0
2015357621	413	rural/clinic	No	11/01/2017	0	0	0
2015357622	413	rural/clinic	No	11/01/2017	0	0	0
2014290233	413	rural/clinic	No	11/01/2017	0	0	0
2011235680	413	rural/clinic	No	11/01/2017	0	0	0
2011200271	413	rural/clinic	No	11/01/2017	0	0	0
2011200272	413	rural/clinic	No	11/01/2017	0	0	0
2015305221	106	rural/clinic	No	11/01/2017	0	0	0
20153052201	106	rural/clinic	No	11/01/2017	0	0	0
2015305219	106	rural/clinic	No	11/01/2017	0	0	0
2015305218	106	rural/clinic	No	11/01/2017	0	0	0
2013263571	106	rural/clinic	No	11/01/2017	0	0	0
2013263577	106	rural/clinic	No	11/01/2017	0	0	0

201326352	106	rural/clinic	No	11/01/2017	0	0	0
2013263575	106	rural/clinic	No	11/01/2017	0	0	0
2013263578	106	rural/clinic	No	11/01/2017	0	0	0
2013263574	106	rural/clinic	No	11/01/2017	0	0	0
2014338232	106	rural/clinic	No	11/01/2017	0	0	0
2013263573	106	rural/clinic	No	11/01/2017	0	0	0
2013263576	106	rural/clinic	No	11/01/2017	0	0	0
2014338233	106	rural/clinic	No	11/01/2017	0	0	0
2012291484	106	rural/clinic	No	11/01/2017	0	0	0
2012246174	106	rural/clinic	No	11/01/2017	0	0	0
2014346447	106	rural/clinic	No	11/01/2017	0	0	0
2014368749	106	rural/clinic	No	11/01/2017	0	0	0
2014368750	106	rural/clinic	No	11/01/2017	0	0	0
2015418940	106	rural/clinic	No	11/01/2017	0	0	0
2015418941	106	rural/clinic	No	11/01/2017	0	0	0
2015414640	106	rural/clinic	No	11/01/2017	0	0	0
2011226089	106	rural/clinic	No	11/01/2017	0	0	0
2015412066	106	rural/clinic	No	11/01/2017	0	0	0
2015375836	106	rural/clinic	No	11/01/2017	0	0	0
2015412065	106	rural/clinic	No	11/01/2017	0	0	0
2015412067	106	rural/clinic	No	11/01/2017	0	0	0
2011226090	106	rural/clinic	No	11/01/2017	0	0	0
2015359390	106	rural/clinic	No	11/01/2017	0	0	0
2015331869	106	rural/clinic	No	11/01/2017	0	0	0
2014287893	106	rural/clinic	No	11/01/2017	0	0	0
2015290020	106	rural/clinic	No	11/01/2017	0	0	0
2014287894	106	rural/clinic	No	11/01/2017	0	0	0
2012253914	106	rural/clinic	No	11/01/2017	0	0	0
2015375831	106	rural/clinic	No	11/01/2017	0	0	0
2015375835	106	rural/clinic	No	11/01/2017	0	0	0
2015359388	106	rural/clinic	No	11/01/2017	0	0	0
2015359386	106	rural/clinic	No	11/01/2017	0	0	0
2015359383	106	rural/clinic	No	11/01/2017	0	0	0
2015359385	106	rural/clinic	No	11/01/2017	0	0	0
2015359382	106	rural/clinic	No	11/01/2017	0	0	0
2014310150	106	rural/clinic	No	11/01/2017	0	0	0
2015344632	106	rural/clinic	No	11/01/2017	0	0	0
2015344633	106	rural/clinic	No	11/01/2017	0	0	0
2015315489	106	rural/clinic	No	11/01/2017	0	0	0
2014360318	106	rural/clinic	No	11/01/2017	0	0	0
2015315491	106	rural/clinic	No	11/01/2017	0	0	0
2014360319	106	rural/clinic	No	11/01/2017	0	0	0
2015413531	106	rural/clinic	No	11/01/2017	0	0	0
2015315488	106	rural/clinic	No	11/01/2017	0	0	0
2015413532	106	rural/clinic	No	11/01/2017	0	0	0
2015315486	106	rural/clinic	No	11/01/2017	0	0	0
2015413533	106	rural/clinic	No	11/01/2017	0	0	0
2015413534	106	rural/clinic	No	11/01/2017	0	0	0
2015315490	106	rural/clinic	No	11/01/2017	0	0	0
2015413535	106	rural/clinic	No	11/01/2017	0	0	0
2015413536	106	rural/clinic	No	11/01/2017	0	0	0
2015385378	106	rural/clinic	No	11/01/2017	0	0	0
2015413537	106	rural/clinic	No	11/01/2017	0	0	0
2015385375	25	rural/clinic	No	11/01/2017	0	0	0
201513530	25	rural/clinic	No	11/01/2017	0	0	0
2015294041	25	rural/clinic	No	11/01/2017	0	0	0
2012345091	25	rural/clinic	No	11/01/2017	0	0	0
2014360320	25	rural/clinic	No	11/01/2017	0	0	0
2014327656	25	rural/clinic	No	11/01/2017	0	0	0
2015293628	25	rural/clinic	No	11/01/2017	0	0	0
2015293629	25	rural/clinic	No	11/01/2017	0	0	0
2015303505	25	rural/clinic	No	11/01/2017	0	0	0
2015319419	25	rural/clinic	No	11/01/2017	0	0	0
2015385421	25	rural/clinic	No	11/01/2017	0	0	0
2012243449	25	rural/clinic	No	11/01/2017	0	0	0
2011196001	25	rural/clinic	No	11/01/2017	0	0	0
2015315487	25	rural/clinic	No	11/01/2017	0	0	0
2011196002	25	rural/clinic	No	11/01/2017	0	0	0
2015294294	25	rural/clinic	No	11/01/2017	0	0	0
2015385420	25	rural/clinic	No	11/01/2017	0	0	0
2015294295	25	rural/clinic	No	11/01/2017	0	0	0
2015385419	25	rural/clinic	No	11/01/2017	0	0	0
2015294293	25	rural/clinic	No	11/01/2017	0	0	0
2015294296	25	rural/clinic	No	11/01/2017	0	0	0
2015385418	25	rural/clinic	No	11/01/2017	0	0	0
2014309735	25	rural/clinic	No	11/01/2017	0	0	0
2015385417	25	rural/clinic	No	11/01/2017	0	0	0
2015315972	25	rural/clinic	No	11/01/2017	0	0	0
2014339246	25	rural/clinic	No	11/01/2017	0	0	0
2015385423	25	rural/clinic	No	11/01/2017	0	0	0
2015385424	25	rural/clinic	No	11/01/2017	0	0	0
2015303816	25	rural/clinic	No	11/01/2017	0	0	0

2015385425	25	rural/clinic	No	11/01/2017	0	0	0
2014309734	25	rural/clinic	No	11/01/2017	0	0	0
2015385426	25	rural/clinic	No	11/01/2017	0	0	0
2015303465	25	rural/clinic	No	11/01/2017	0	0	0
2015385422	25	rural/clinic	No	11/01/2017	0	0	0
2015337605	25	rural/clinic	No	11/01/2017	0	0	0
2015337607	25	rural/clinic	No	11/01/2017	0	0	0
2015303462	25	rural/clinic	No	11/01/2017	0	0	0
2015337606	25	rural/clinic	No	11/01/2017	0	0	0
2015303467	25	rural/clinic	No	11/01/2017	0	0	0
2011156595	25	rural/clinic	No	11/01/2017	0	0	0
2015303468	25	rural/clinic	No	11/01/2017	0	0	0
2011156596	25	rural/clinic	No	11/01/2017	0	0	0
2012388370	25	rural/clinic	No	11/01/2017	0	0	0
2015338703	25	rural/clinic	No	11/01/2017	0	0	0
2015385377	25	rural/clinic	No	11/01/2017	0	0	0
2015324421	25	rural/clinic	No	11/01/2017	0	0	0
2015324420	25	rural/clinic	No	11/01/2017	0	0	0
2015324422	25	rural/clinic	No	11/01/2017	0	0	0
2015324418	25	rural/clinic	No	11/01/2017	0	0	0
2015385923	25	rural/clinic	No	11/01/2017	0	0	0
2015338702	25	rural/clinic	No	11/01/2017	0	0	0
2015335570	86	rural/clinic	No	11/01/2017	0	0	0
2015335570	86	rural/clinic	No	11/01/2017	0	0	0
2015295186	86	rural/clinic	No	11/01/2017	0	0	0
2015295186	86	rural/clinic	No	11/01/2017	0	0	0
2015291900	86	rural/clinic	No	11/01/2017	0	0	0
2015291900	86	rural/clinic	No	11/01/2017	0	0	0
2014289464/D	86	rural/clinic	No	11/01/2017	0	0	0
2014289464/D	86	rural/clinic	No	11/01/2017	0	0	0
2015293914	86	rural/clinic	No	11/01/2017	0	0	0
2015385376	86	rural/clinic	No	11/01/2017	0	0	0
2015303463	86	rural/clinic	No	11/01/2017	0	0	0
2012321327	86	rural/clinic	No	11/01/2017	0	0	0
2012321327	86	rural/clinic	No	11/01/2017	0	0	0
2015293913	86	rural/clinic	No	11/01/2017	0	0	0
2014339247	86	rural/clinic	No	11/01/2017	0	0	0
2016167837	86	rural/clinic	No	11/01/2017	0	0	0
2014344698	86	rural/clinic	No	11/01/2017	0	0	0
2015324417	86	rural/clinic	No	11/01/2017	0	0	0
2014288566	86	rural/clinic	No	11/01/2017	0	0	0
2014288587	86	rural/clinic	No	11/01/2017	0	0	0
2012312844	86	rural/clinic	No	11/01/2017	0	0	0
2015302477	86	rural/clinic	No	11/01/2017	0	0	0
2013249484	86	rural/clinic	No	11/01/2017	0	0	0
2013249277	86	rural/clinic	No	11/01/2017	0	0	0
2015302480	86	rural/clinic	No	11/01/2017	0	0	0
2015342272	86	rural/clinic	No	11/01/2017	0	0	0
2014288569	86	rural/clinic	No	11/01/2017	0	0	0
2014288568	86	rural/clinic	No	11/01/2017	0	0	0
2012259136	86	rural/clinic	No	11/01/2017	0	0	0
2012259137	86	rural/clinic	No	11/01/2017	0	0	0
2012259138	86	rural/clinic	No	11/01/2017	0	0	0
2015334050	86	rural/clinic	No	11/01/2017	0	0	0
2015334040	86	rural/clinic	No	11/01/2017	0	0	0
2015334048	86	rural/clinic	No	11/01/2017	0	0	0
2015334041	86	rural/clinic	No	11/01/2017	0	0	0
2015334043	86	rural/clinic	No	11/01/2017	0	0	0
2012265278	86	rural/clinic	No	11/01/2017	0	0	0
2011157091	86	rural/clinic	No	11/01/2017	0	0	0
2014363997	86	rural/clinic	No	05/01/2017	0	0	0
2015361672	86	rural/clinic	No	11/01/2017	0	0	0
2014368746	86	rural/clinic	No	05/01/2017	0	0	0
2014365143	86	rural/clinic	No	05/01/2017	0	0	0
2015369909	86	rural/clinic	No	05/01/2017	0	0	0
2015361673	86	rural/clinic	No	11/01/2017	0	0	0
2014291808	86	rural/clinic	No	05/01/2017	0	0	0
2015361675	86	rural/clinic	No	11/01/2017	0	0	0
2015361674	86	rural/clinic	No	11/01/2017	0	0	0
2013271296	86	rural/clinic	No	11/01/2017	0	0	0
2011233793	86	rural/clinic	No	11/01/2017	0	0	0
2011233792	86	rural/clinic	No	11/01/2017	0	0	0
2014330640	86	rural/clinic	No	11/01/2017	0	0	0
2014330639	86	rural/clinic	No	11/01/2017	0	0	0
2013255738	86	rural/clinic	No	05/01/2017	0	0	0
2015377417	86	rural/clinic	No	05/01/2017	0	0	0
2015377416	86	rural/clinic	No	05/01/2017	0	0	0
2012306134	569	rural/clinic	No	11/01/2017	0	0	0
2015302483	569	rural/clinic	No	11/01/2017	0	0	0
2015346218	569	rural/clinic	No	11/01/2017	0	0	0
2013271295	569	rural/clinic	No	11/01/2017	0	0	0
2015397769	569	rural/clinic	No	11/01/2017	0	0	0

2015373084	569	rural/clinic	No	12/01/2017	0	0	0
2015397768	569	rural/clinic	No	11/01/2017	0	0	0
2015340462	569	rural/clinic	No	05/01/2017	0	0	0
2015397767	569	rural/clinic	No	11/01/2017	0	0	0
2015373093	569	rural/clinic	No	12/01/2017	0	0	0
2014382410	569	rural/clinic	No	11/01/2017	0	0	0
2015413053	569	rural/clinic	No	05/01/2017	0	0	0
2011225140	52	provincial	No	12/01/2017	0	1	0
2015413057	52	provincial	No	05/01/2017	0	1	0
2014382415	52	provincial	No	11/01/2017	0	1	0
2015413056	52	provincial	No	05/01/2017	0	1	0
2015397770	52	provincial	No	11/01/2017	0	1	0
2012283586	52	provincial	No	12/01/2017	0	1	0
2015302479	52	provincial	No	11/01/2017	0	1	0
2015373085	52	provincial	No	12/01/2017	0	1	0
2015361702	52	provincial	No	11/01/2017	0	1	0
2011225141	52	provincial	No	12/01/2017	0	1	0
2014318826	52	provincial	No	11/01/2017	0	1	0
2014318824	52	provincial	No	11/01/2017	0	1	0
2015413055	52	provincial	No	05/01/2017	0	1	0
2012283584	52	provincial	No	12/01/2017	0	1	0
2014318825	52	provincial	No	11/01/2017	0	1	0
2015373051	52	provincial	No	12/01/2017	0	1	0
2014302841	52	provincial	No	12/01/2017	0	1	0
2014318827	52	provincial	No	11/01/2017	0	1	0
2015405085	52	provincial	No	12/01/2017	0	1	0
2015413054	52	provincial	No	05/01/2017	0	1	0
2014318828	52	provincial	No	11/01/2017	0	1	0
2015373625	52	provincial	No	11/01/2017	0	1	0
2015302473	52	provincial	No	11/01/2017	0	1	0
2015373627	52	provincial	No	11/01/2017	0	1	0
2015373150	52	provincial	No	12/01/2017	0	1	0
2012283585	52	provincial	No	12/01/2017	0	1	0
2014327232	52	provincial	No	11/01/2017	0	1	0
2015373628	52	provincial	No	11/01/2017	0	1	0
2014327234	52	provincial	No	11/01/2017	0	1	0
2015413052	52	provincial	No	05/01/2017	0	1	0
2015373629	52	provincial	No	11/01/2017	0	1	0
2015302476	52	provincial	No	11/01/2017	0	1	0
2015413051	52	provincial	No	05/01/2017	0	1	0
2015373149	52	provincial	No	12/01/2017	0	1	0
2015302474	52	provincial	No	11/01/2017	0	1	0
2015373630	52	provincial	No	11/01/2017	0	1	0
2012384773	52	provincial	No	05/01/2017	0	1	0
2015357519	52	provincial	No	11/01/2017	0	1	0
2014327233	52	provincial	No	11/01/2017	0	1	0
2015373631	52	provincial	No	11/01/2017	0	1	0
2014338226	52	provincial	No	05/01/2017	0	1	0
2015357356	52	provincial	No	11/01/2017	0	1	0
2015373086	52	provincial	No	12/01/2017	0	1	0
2015373632	52	provincial	No	11/01/2017	0	1	0
2014338228	52	provincial	No	05/01/2017	0	1	0
2015325170	52	provincial	No	11/01/2017	0	1	0
2015373633	52	provincial	No	11/01/2017	0	1	0
2014302840	52	provincial	No	12/01/2017	0	1	0
2015325169	52	provincial	No	11/01/2017	0	1	0
2014338230	52	provincial	No	05/01/2017	0	1	0
2015357352	52	provincial	No	11/01/2017	0	1	0
2015373634	52	provincial	No	11/01/2017	0	1	0
2013252931	52	provincial	No	12/01/2017	0	1	0
2015357179	52	provincial	No	11/01/2017	0	1	0
2015373635	52	provincial	No	11/01/2017	0	1	0
2015357354	52	provincial	No	11/01/2017	0	1	0
2015373076	52	provincial	No	12/01/2017	0	1	0
2015373636	52	provincial	No	11/01/2017	0	1	0
2015357353	52	provincial	No	11/01/2017	0	1	0
2015378938	197	rural/clinic	No	05/01/2017	0	0	0
2014354756	197	rural/clinic	No	12/01/2017	0	0	0
2015373077	197	rural/clinic	No	12/01/2017	0	0	0
2015378937	197	rural/clinic	No	05/01/2017	0	0	0
2011225644	197	rural/clinic	No	12/01/2017	0	0	0
2015373088	197	rural/clinic	No	12/01/2017	0	0	0
2015373072	197	rural/clinic	No	12/01/2017	0	0	0
2014354755	197	rural/clinic	No	12/01/2017	0	0	0
2015373071	197	rural/clinic	No	12/01/2017	0	0	0
2014301917	197	rural/clinic	No	12/01/2017	0	0	0
2011199096	197	rural/clinic	No	12/01/2017	0	0	0
2015373070	197	rural/clinic	No	12/01/2017	0	0	0
2015413973	197	rural/clinic	No	11/01/2017	0	0	0
2015319563	197	rural/clinic	No	12/01/2017	0	0	0
2015413970	197	rural/clinic	No	11/01/2017	0	0	0
2015373069	197	rural/clinic	No	12/01/2017	0	0	0

2011199238	197	rural/clinic	No	12/01/2017	0	0	0
2015413972	197	rural/clinic	No	11/01/2017	0	0	0
2015338701	197	rural/clinic	No	11/01/2017	0	0	0
2015319564	197	rural/clinic	No	12/01/2017	0	0	0
2015373067	197	rural/clinic	No	12/01/2017	0	0	0
2015385374	197	rural/clinic	No	11/01/2017	0	0	0
2015325977	197	rural/clinic	No	12/01/2017	0	0	0
2014356150	197	rural/clinic	No	11/01/2017	0	0	0
2015319561	197	rural/clinic	No	12/01/2017	0	0	0
2015373066	197	rural/clinic	No	12/01/2017	0	0	0
2012323772	197	rural/clinic	No	11/01/2017	0	0	0
2012323773	197	rural/clinic	No	11/01/2017	0	0	0
2014355837	197	rural/clinic	No	11/01/2017	0	0	0
2015373065	197	rural/clinic	No	12/01/2017	0	0	0
2014355838	197	rural/clinic	No	11/01/2017	0	0	0
2015325976	197	rural/clinic	No	12/01/2017	0	0	0
2015319562	197	rural/clinic	No	12/01/2017	0	0	0
2015373064	197	rural/clinic	No	12/01/2017	0	0	0
2014355839	197	rural/clinic	No	11/01/2017	0	0	0
2014355840	197	rural/clinic	No	11/01/2017	0	0	0
2015383596	197	rural/clinic	No	12/01/2017	0	0	0
2015373063	197	rural/clinic	No	12/01/2017	0	0	0
2014355836	540	rural/clinic	No	11/01/2017	0	0	0
2015315479	540	rural/clinic	No	11/01/2017	0	0	0
2015324419	540	rural/clinic	No	11/01/2017	0	0	0
2015373060	540	rural/clinic	No	12/01/2017	0	0	0
2015315480	540	rural/clinic	No	11/01/2017	0	0	0
2014317739	540	rural/clinic	No	12/01/2017	0	0	0
2015377851	540	rural/clinic	No	12/01/2017	0	0	0
2015373061	540	rural/clinic	No	12/01/2017	0	0	0
2015315481	540	rural/clinic	No	11/01/2017	0	0	0
2015315482	540	rural/clinic	No	11/01/2017	0	0	0
2015315483	540	rural/clinic	No	11/01/2017	0	0	0
2015315484	540	rural/clinic	No	11/01/2017	0	0	0
2015360083	540	rural/clinic	No	12/01/2017	0	0	0
2015340463	540	rural/clinic	No	12/01/2017	0	0	0
2015377852	540	rural/clinic	No	12/01/2017	0	0	0
2015315485	540	rural/clinic	No	11/01/2017	0	0	0
2015340464	540	rural/clinic	No	12/01/2017	0	0	0
2014311532	540	rural/clinic	No	11/01/2017	0	0	0
2014328429	540	rural/clinic	No	12/01/2017	0	0	0
2012387238	754	rural/clinic	No	12/01/2017	0	0	0
2015385505	754	rural/clinic	No	12/01/2017	0	0	0
2014328430	754	rural/clinic	No	12/01/2017	0	0	0
2015385506	754	rural/clinic	No	12/01/2017	0	0	0
2014328428	754	rural/clinic	No	12/01/2017	0	0	0
2015385507	754	rural/clinic	No	12/01/2017	0	0	0
2015293257	754	rural/clinic	No	05/01/2017	0	0	0
2014377405	754	rural/clinic	No	12/01/2017	0	0	0
2015373326	754	rural/clinic	No	12/01/2017	0	0	0
2015293258	754	rural/clinic	No	05/01/2017	0	0	0
2014363998	754	rural/clinic	No	05/01/2017	0	0	0
2014363996	754	rural/clinic	No	05/01/2017	0	0	0
2015373327	754	rural/clinic	No	12/01/2017	0	0	0
2015377948	273	rural/clinic	No	12/01/2017	0	0	0
2011143189	273	rural/clinic	No	05/01/2017	0	0	0
2014326616	273	rural/clinic	No	12/01/2017	0	0	0
2013273638	273	rural/clinic	No	05/01/2017	0	0	0
2012269009	273	rural/clinic	No	12/01/2017	0	0	0
2015377944	273	rural/clinic	No	12/01/2017	0	0	0
2012387239	273	rural/clinic	No	12/01/2017	0	0	0
2015294141	273	rural/clinic	No	11/01/2017	0	0	0
2015378935	273	rural/clinic	No	05/01/2017	0	0	0
2014327081	273	rural/clinic	No	12/01/2017	0	0	0
2015378936	273	rural/clinic	No	05/01/2017	0	0	0
2015289822	273	rural/clinic	No	12/01/2017	0	0	0
2015358108	273	rural/clinic	No	05/01/2017	0	0	0
2014386120	273	rural/clinic	No	12/01/2017	0	0	0
2015358102	273	rural/clinic	No	05/01/2017	0	0	0
2014326617	273	rural/clinic	No	12/01/2017	0	0	0
2012387240	273	rural/clinic	No	12/01/2017	0	0	0
2015358107	273	rural/clinic	No	05/01/2017	0	0	0
2015377947	273	rural/clinic	No	12/01/2017	0	0	0
2014300531	273	rural/clinic	No	12/01/2017	0	0	0
2011235741	273	rural/clinic	No	12/01/2017	0	0	0
2014296312	273	rural/clinic	No	12/01/2017	0	0	0
2015412989	273	rural/clinic	No	12/01/2017	0	0	0
2015384352	273	rural/clinic	No	12/01/2017	0	0	0
2015358103	273	rural/clinic	No	05/01/2017	0	0	0
2013264344	273	rural/clinic	No	12/01/2017	0	0	0
2011235740	273	rural/clinic	No	12/01/2017	0	0	0
2015286254	273	rural/clinic	No	12/01/2017	0	0	0

2014377404	273	rural/clinic	No	12/01/2017	0	0	0
2015384353	273	rural/clinic	No	12/01/2017	0	0	0
2015357624	273	rural/clinic	No	12/01/2017	0	0	0
2015377853	273	rural/clinic	No	12/01/2017	0	0	0
2015357625	273	rural/clinic	No	12/01/2017	0	0	0
2015384354	273	rural/clinic	No	12/01/2017	0	0	0
2012268993	273	rural/clinic	No	05/01/2017	0	0	0
2015402266	273	rural/clinic	No	05/01/2017	0	0	0
2015301080	273	rural/clinic	No	12/01/2017	0	0	0
2012268992	273	rural/clinic	No	05/01/2017	0	0	0
2014386111	273	rural/clinic	No	05/01/2017	0	0	0
2015301434	273	rural/clinic	No	12/01/2017	0	0	0
2011110040	273	rural/clinic	No	12/01/2017	0	0	0
2014386112	273	rural/clinic	No	05/01/2017	0	0	0
2015301079	273	rural/clinic	No	12/01/2017	0	0	0
2011235568	273	rural/clinic	No	12/01/2017	0	0	0
2015402261	273	rural/clinic	No	05/01/2017	0	0	0
2015402262	273	rural/clinic	No	05/01/2017	0	0	0
2012269007	273	rural/clinic	No	12/01/2017	0	0	0
2013264670	273	rural/clinic	No	12/01/2017	0	0	0
2011235569	273	rural/clinic	No	12/01/2017	0	0	0
2015402263	393	rural/clinic	No	05/01/2017	0	0	0
2012306337	393	rural/clinic	No	12/01/2017	0	0	0
2012269008	393	rural/clinic	No	12/01/2017	0	0	0
2015402264	393	rural/clinic	No	05/01/2017	0	0	0
2014346888	393	rural/clinic	No	12/01/2017	0	0	0
2015402265	393	rural/clinic	No	05/01/2017	0	0	0
2011200273	393	rural/clinic	No	12/01/2017	0	0	0
2014301670	393	rural/clinic	No	12/01/2017	0	0	0
2011155218	393	rural/clinic	No	05/01/2017	0	0	0
2011235847	393	rural/clinic	No	12/01/2017	0	0	0
2014301884	393	rural/clinic	No	05/01/2017	0	0	0
2011235419	393	rural/clinic	No	12/01/2017	0	0	0
2014346889	393	rural/clinic	No	12/01/2017	0	0	0
2014301885	393	rural/clinic	No	05/01/2017	0	0	0
2014329134	393	rural/clinic	No	12/01/2017	0	0	0
2015405751	393	rural/clinic	No	05/01/2017	0	0	0
2014378262	393	rural/clinic	No	12/01/2017	0	0	0
2014329130	393	rural/clinic	No	12/01/2017	0	0	0
2015405084	393	rural/clinic	No	05/01/2017	0	0	0
2015405079	393	rural/clinic	No	05/01/2017	0	0	0
2014372265	875	rural/clinic	No	12/01/2017	0	0	0
2013247130	875	rural/clinic	No	12/01/2017	0	0	0
2014385682	875	rural/clinic	No	05/01/2017	0	0	0
2014301883	875	rural/clinic	No	05/01/2017	0	0	0
2015397911/D	875	rural/clinic	No	12/01/2017	0	0	0
2015413067	875	rural/clinic	No	12/01/2017	0	0	0
2014301886	29	rural/clinic	No	05/01/2017	0	0	0
2011144108	29	rural/clinic	No	05/01/2017	0	0	0
2015383570	29	rural/clinic	No	12/01/2017	0	0	0
2013323653	29	rural/clinic	No	12/01/2017	0	0	0
2015413066	29	rural/clinic	No	12/01/2017	0	0	0
2014385612	29	rural/clinic	No	05/01/2017	0	0	0
2015383571	29	rural/clinic	No	12/01/2017	0	0	0
2011144107	29	rural/clinic	No	05/01/2017	0	0	0
2015413065	29	rural/clinic	No	12/01/2017	0	0	0
2015373360	29	rural/clinic	No	12/01/2017	0	0	0
2015383573	29	rural/clinic	No	12/01/2017	0	0	0
2015383572	29	rural/clinic	No	12/01/2017	0	0	0
2015413064	29	rural/clinic	No	12/01/2017	0	0	0
2012341035	29	rural/clinic	No	05/01/2017	0	0	0
2015349917	29	rural/clinic	No	12/01/2017	0	0	0
2015379306	29	rural/clinic	No	12/01/2017	0	0	0
2015373359	29	rural/clinic	No	12/01/2017	0	0	0
2014350955	29	rural/clinic	No	05/01/2017	0	0	0
201549918	29	rural/clinic	No	12/01/2017	0	0	0
2014335204	29	rural/clinic	No	05/01/2017	0	0	0
201549921	29	rural/clinic	No	12/01/2017	0	0	0
2015373358	29	rural/clinic	No	12/01/2017	0	0	0
2015349920	29	rural/clinic	No	12/01/2017	0	0	0
2012251526	29	rural/clinic	No	12/01/2017	0	0	0
2015373357	29	rural/clinic	No	12/01/2017	0	0	0
2012251523	29	rural/clinic	No	18/01/2017	0	0	0
2015335357	29	rural/clinic	No	12/01/2017	0	0	0
2014344283	29	rural/clinic	No	05/01/2017	0	0	0
2015343045	29	rural/clinic	No	12/01/2017	0	0	0
2012251527	29	rural/clinic	No	12/01/2017	0	0	0
2015335358	29	rural/clinic	No	12/01/2017	0	0	0
2015360572	29	rural/clinic	No	05/01/2017	0	0	0
2015335359	29	rural/clinic	No	12/01/2017	0	0	0
2014360878	29	rural/clinic	No	05/01/2017	0	0	0
2011216817	29	rural/clinic	No	12/01/2017	0	0	0

2015360571	29	rural/clinic	No	05/01/2017	0	0	0
2015335360	29	rural/clinic	No	12/01/2017	0	0	0
2012251525	29	rural/clinic	No	12/01/2017	0	0	0
2015335361	29	rural/clinic	No	12/01/2017	0	0	0
2015343043	29	rural/clinic	No	12/01/2017	0	0	0
2015335363	232	rural/clinic	No	12/01/2017	0	0	0
2015335364	232	rural/clinic	No	12/01/2017	0	0	0
2014365144	232	rural/clinic	No	12/01/2017	0	0	0
2014365145	232	rural/clinic	No	12/01/2017	0	0	0
2012251524	232	rural/clinic	No	12/01/2017	0	0	0
2014365146	232	rural/clinic	No	12/01/2017	0	0	0
2014365147	232	rural/clinic	No	12/01/2017	0	0	0
2015358110	232	rural/clinic	No	12/01/2017	0	0	0
2015358111	232	rural/clinic	No	12/01/2017	0	0	0
2015358106	232	rural/clinic	No	12/01/2017	0	0	0
2015362321	232	rural/clinic	No	12/01/2017	0	0	0
2015358109	232	rural/clinic	No	12/01/2017	0	0	0
2015362320	232	rural/clinic	No	12/01/2017	0	0	0
2015402267	232	rural/clinic	No	12/01/2017	0	0	0
2015402268	232	rural/clinic	No	12/01/2017	0	0	0
2015362322	232	rural/clinic	No	12/01/2017	0	0	0
2015373356	232	rural/clinic	No	12/01/2017	0	0	0
2015402269	232	rural/clinic	No	12/01/2017	0	0	0
2015402270	232	rural/clinic	No	12/01/2017	0	0	0
2015373355	232	rural/clinic	No	12/01/2017	0	0	0
2015402271	232	rural/clinic	No	12/01/2017	0	0	0
2011232657	232	rural/clinic	No	12/01/2017	0	0	0
2014287171	87	rural/clinic	No	12/01/2017	0	0	0
2015386342	87	rural/clinic	No	12/01/2017	0	0	0
2015386345	87	rural/clinic	No	12/01/2017	0	0	0
2015317336	87	rural/clinic	No	12/01/2017	0	0	0
2015386346	87	rural/clinic	No	12/01/2017	0	0	0
2015362317	87	rural/clinic	No	12/01/2017	0	0	0
2014358319	87	rural/clinic	No	12/01/2017	0	0	0
2015386347	87	rural/clinic	No	12/01/2017	0	0	0
2014314538	87	rural/clinic	No	12/01/2017	0	0	0
2015386349	87	rural/clinic	No	12/01/2017	0	0	0
2015362316	87	rural/clinic	No	12/01/2017	0	0	0
2014378712	87	rural/clinic	No	12/01/2017	0	0	0
2015386350	87	rural/clinic	No	12/01/2017	0	0	0
2015384022	87	rural/clinic	No	12/01/2017	0	0	0
2015358252	87	rural/clinic	No	12/01/2017	0	0	0
2014314536	87	rural/clinic	No	12/01/2017	0	0	0
2015358253	87	rural/clinic	No	12/01/2017	0	0	0
2014378711	87	rural/clinic	No	12/01/2017	0	0	0
2015358254	87	rural/clinic	No	12/01/2017	0	0	0
2015384023	87	rural/clinic	No	12/01/2017	0	0	0
2013325501	87	rural/clinic	No	05/01/2017	0	0	0
2014314537	87	rural/clinic	No	12/01/2017	0	0	0
2014335207	19	rural/clinic	No	05/01/2017	0	0	0
2015358255	19	rural/clinic	No	12/01/2017	0	0	0
2014360879	19	rural/clinic	No	05/01/2017	0	0	0
2014358312	19	rural/clinic	No	12/01/2017	0	0	0
2014335211	19	rural/clinic	No	05/01/2017	0	0	0
2015386324	19	rural/clinic	No	12/01/2017	0	0	0
2015384024	19	rural/clinic	No	12/01/2017	0	0	0
2014335205	19	rural/clinic	No	05/01/2017	0	0	0
2014335208	19	rural/clinic	No	05/01/2017	0	0	0
2014386118	19	rural/clinic	No	12/01/2017	0	0	0
2014314535	19	rural/clinic	No	12/01/2017	0	0	0
2014335206	19	rural/clinic	No	05/01/2017	0	0	0
2014335209	19	rural/clinic	No	05/01/2017	0	0	0
2015335097	19	rural/clinic	No	12/01/2017	0	0	0
2014358316	19	rural/clinic	No	12/01/2017	0	0	0
2012390127	19	rural/clinic	No	05/01/2017	0	0	0
2014386119	19	rural/clinic	No	12/01/2017	0	0	0
2014358315	19	rural/clinic	No	12/01/2017	0	0	0
2014386121	19	rural/clinic	No	12/01/2017	0	0	0
2013272998	19	rural/clinic	No	12/01/2017	0	0	0
2015335099	19	rural/clinic	No	12/01/2017	0	0	0
2014386122	19	rural/clinic	No	12/01/2017	0	0	0
2014351217	19	rural/clinic	No	12/01/2017	0	0	0
2014386123	19	rural/clinic	No	12/01/2017	0	0	0
2015335100	19	rural/clinic	No	12/01/2017	0	0	0
2014287172	19	rural/clinic	No	12/01/2017	0	0	0
2015397463	19	rural/clinic	No	09/01/2017	0	0	0
2014386124	19	rural/clinic	No	12/01/2017	0	0	0
2013272999	19	rural/clinic	No	12/01/2017	0	0	0
2015377001	19	rural/clinic	No	12/01/2017	0	0	0
2014386125	19	rural/clinic	No	12/01/2017	0	0	0
2014358318	19	rural/clinic	No	12/01/2017	0	0	0
2014386126	19	rural/clinic	No	12/01/2017	0	0	0

2014307743	19	rural/clinic	No	12/01/2017	0	0	0
2014386127	19	rural/clinic	No	12/01/2017	0	0	0
2015377002	19	rural/clinic	No	12/01/2017	0	0	0
2015397464	19	rural/clinic	No	09/01/2017	0	0	0
2014307744	19	rural/clinic	No	12/01/2017	0	0	0
2015386323	19	rural/clinic	No	12/01/2017	0	0	0
2015397465	19	rural/clinic	No	09/01/2017	0	0	0
2011167949	19	rural/clinic	No	12/01/2017	0	0	0
2011199071	19	rural/clinic	No	12/01/2017	0	0	0
2015397466	19	rural/clinic	No	09/01/2017	0	0	0
2014327080	19	rural/clinic	No	12/01/2017	0	0	0
2011167950	19	rural/clinic	No	12/01/2017	0	0	0
2015386322	19	rural/clinic	No	12/01/2017	0	0	0
2015335362	19	rural/clinic	No	12/01/2017	0	0	0
2011216822	19	rural/clinic	No	12/01/2017	0	0	0
2015386321	19	rural/clinic	No	12/01/2017	0	0	0
2015386320	19	rural/clinic	No	12/01/2017	0	0	0
2011216821	19	rural/clinic	No	12/01/2017	0	0	0
2015397467	19	rural/clinic	No	09/01/2017	0	0	0
2011225142	19	rural/clinic	No	12/01/2017	0	0	0
2015349919	19	rural/clinic	No	12/01/2017	0	0	0
2015397468	19	rural/clinic	No	09/01/2017	0	0	0
2015386326	19	rural/clinic	No	12/01/2017	0	0	0
2015397469	19	rural/clinic	No	09/01/2017	0	0	0
2015373354	19	rural/clinic	No	12/01/2017	0	0	0
2015349916	19	rural/clinic	No	12/01/2017	0	0	0
2015397470	19	rural/clinic	No	09/01/2017	0	0	0
2015386327	19	rural/clinic	No	12/01/2017	0	0	0
2015373353	19	rural/clinic	No	12/01/2017	0	0	0
2015397471	19	rural/clinic	No	09/01/2017	0	0	0
2015386329	19	rural/clinic	No	12/01/2017	0	0	0
2015397472	19	rural/clinic	No	09/01/2017	0	0	0
2015324253	19	rural/clinic	No	12/01/2017	0	0	0
2015397473	19	rural/clinic	No	09/01/2017	0	0	0
2015324251	19	rural/clinic	No	12/01/2017	0	0	0
2015386328	19	rural/clinic	No	12/01/2017	0	0	0
2015352412	19	rural/clinic	No	12/01/2017	0	0	0
2015332004	19	rural/clinic	No	12/01/2017	0	0	0
2015368771	19	rural/clinic	No	13/01/2017	0	0	0
2015386344	19	rural/clinic	No	12/01/2017	0	0	0
2015352410	19	rural/clinic	No	12/01/2017	0	0	0
2015386348	19	rural/clinic	No	12/01/2017	0	0	0
2015397474	19	rural/clinic	No	09/01/2017	0	0	0
2015352408	19	rural/clinic	No	12/01/2017	0	0	0
2015386341	19	rural/clinic	No	12/01/2017	0	0	0
2015386340	19	rural/clinic	No	12/01/2017	0	0	0
2014297892	19	rural/clinic	No	09/01/2017	0	0	0
2015352409	19	rural/clinic	No	12/01/2017	0	0	0
2014297893	19	rural/clinic	No	09/01/2017	0	0	0
2015386339	19	rural/clinic	No	12/01/2017	0	0	0
2015352411	19	rural/clinic	No	12/01/2017	0	0	0
2015368773	19	rural/clinic	No	17/01/2017	0	0	0
2014297894	19	rural/clinic	No	09/01/2017	0	0	0
2015386338	19	rural/clinic	No	12/01/2017	0	0	0
2014297895	19	rural/clinic	No	09/01/2017	0	0	0
2015324219	19	rural/clinic	No	12/01/2017	0	0	0
2015386337	19	rural/clinic	No	12/01/2017	0	0	0
2015352413	19	rural/clinic	No	12/01/2017	0	0	0
2015386325	19	rural/clinic	No	12/01/2017	0	0	0
2015368774	19	rural/clinic	No	17/01/2017	0	0	0
2015386331	19	rural/clinic	No	12/01/2017	0	0	0
2015352414	19	rural/clinic	No	12/01/2017	0	0	0
2015386332	19	rural/clinic	No	12/01/2017	0	0	0
2015332005	19	rural/clinic	No	12/01/2017	0	0	0
2015368770	19	rural/clinic	No	17/01/2017	0	0	0
2015386333	19	rural/clinic	No	12/01/2017	0	0	0
2012291829	19	rural/clinic	No	12/01/2017	0	0	0
2015386335	19	rural/clinic	No	12/01/2017	0	0	0
2015408088	19	rural/clinic	No	12/01/2017	0	0	0
2015386334	19	rural/clinic	No	12/01/2017	0	0	0
2014298578	19	rural/clinic	No	13/01/2017	0	0	0
2011142216	19	rural/clinic	No	05/01/2017	0	0	0
2012291831	19	rural/clinic	No	12/01/2017	0	0	0
2015386336	19	rural/clinic	No	12/01/2017	0	0	0
2011144001	19	rural/clinic	No	05/01/2017	0	0	0
2014298579	19	rural/clinic	No	17/01/2017	0	0	0
2015358112	19	rural/clinic	No	12/01/2017	0	0	0
2015408087	19	rural/clinic	No	12/01/2017	0	0	0
2014300418	19	rural/clinic	No	05/01/2017	0	0	0
2015368130	19	rural/clinic	No	12/01/2017	0	0	0
2014335213	19	rural/clinic	No	12/01/2017	0	0	0
2014298577	19	rural/clinic	No	17/01/2017	0	0	0

2015368129	19	rural/clinic	No	12/01/2017	0	0	0
2014300419	19	rural/clinic	No	05/01/2017	0	0	0
2014335212	19	rural/clinic	No	12/01/2017	0	0	0
2015368127	19	rural/clinic	No	12/01/2017	0	0	0
2014337037	19	rural/clinic	No	05/01/2017	0	0	0
2014298576	19	rural/clinic	No	17/01/2017	0	0	0
2015368128	19	rural/clinic	No	12/01/2017	0	0	0
2015360575	19	rural/clinic	No	12/01/2017	0	0	0
2013264340	19	rural/clinic	No	05/01/2017	0	0	0
2014337035	19	rural/clinic	No	05/01/2017	0	0	0
2012340198	19	rural/clinic	No	12/01/2017	0	0	0
2014344284	19	rural/clinic	No	12/01/2017	0	0	0
2015384474	19	rural/clinic	No	17/01/2017	0	0	0
2015335094	19	rural/clinic	No	05/01/2017	0	0	0
2015368262	19	rural/clinic	No	12/01/2017	0	0	0
201532751	19	rural/clinic	No	12/01/2017	0	0	0
2014383028	19	rural/clinic	No	05/01/2017	0	0	0
2015360573	19	rural/clinic	No	12/01/2017	0	0	0
2012242573	19	rural/clinic	No	17/01/2017	0	0	0
2014383029	19	rural/clinic	No	05/01/2017	0	0	0
2015412988	19	rural/clinic	No	05/01/2017	0	0	0
2015401519	19	rural/clinic	No	12/01/2017	0	0	0
2015368261	19	rural/clinic	No	12/01/2017	0	0	0
2015372905	19	rural/clinic	No	17/01/2017	0	0	0
2015401517	19	rural/clinic	No	12/01/2017	0	0	0
2015368260	19	rural/clinic	No	12/01/2017	0	0	0
2014382907	19	rural/clinic	No	05/01/2017	0	0	0
2015401514	233	rural/clinic	No	12/01/2017	0	0	0
2015372907	233	rural/clinic	No	17/01/2017	0	0	0
2015368259	233	rural/clinic	No	12/01/2017	0	0	0
2011224796	233	rural/clinic	No	05/01/2017	0	0	0
2015401518	233	rural/clinic	No	12/01/2017	0	0	0
2015368258	233	rural/clinic	No	12/01/2017	0	0	0
2015401510	233	rural/clinic	No	12/01/2017	0	0	0
2013270344	233	rural/clinic	No	12/01/2017	0	0	0
2015372908	233	rural/clinic	No	17/01/2017	0	0	0
2014319425	233	rural/clinic	No	05/01/2017	0	0	0
2015401513	233	rural/clinic	No	12/01/2017	0	0	0
2013270345	233	rural/clinic	No	12/01/2017	0	0	0
2014333796	233	rural/clinic	No	05/01/2017	0	0	0
2014289475	233	rural/clinic	No	17/01/2017	0	0	0
2015401512	233	rural/clinic	No	12/01/2017	0	0	0
2014319426	233	rural/clinic	No	05/01/2017	0	0	0
2011198683	233	rural/clinic	No	12/01/2017	0	0	0
2015401508	233	rural/clinic	No	12/01/2017	0	0	0
2014291809	233	rural/clinic	No	05/01/2017	0	0	0
2012262052	233	rural/clinic	No	12/01/2017	0	0	0
2014291810	233	rural/clinic	No	05/01/2017	0	0	0
2015401507	233	rural/clinic	No	12/01/2017	0	0	0
2012262054	233	rural/clinic	No	12/01/2017	0	0	0
2014291811	233	rural/clinic	No	05/01/2017	0	0	0
2015401505	233	rural/clinic	No	12/01/2017	0	0	0
2015289823	233	rural/clinic	No	12/01/2017	0	0	0
2015401504	233	rural/clinic	No	12/01/2017	0	0	0
2015401506	233	rural/clinic	No	12/01/2017	0	0	0
2014289473/D	233	rural/clinic	No	17/01/2017	0	0	0
2012253955	233	rural/clinic	No	12/01/2017	0	0	0
2015335176	233	rural/clinic	No	12/01/2017	0	0	0
2015335177	233	rural/clinic	No	12/01/2017	0	0	0
2014289466/D	233	rural/clinic	No	17/01/2017	0	0	0
2015335178	233	rural/clinic	No	12/01/2017	0	0	0
2014289471/D	233	rural/clinic	No	17/01/2017	0	0	0
2015368772	233	rural/clinic	No	17/01/2017	0	0	0
2015335179	233	rural/clinic	No	12/01/2017	0	0	0
2014300530	233	rural/clinic	No	12/01/2017	0	0	0
2011113935	233	rural/clinic	No	12/01/2017	0	0	0
2014289472/D	233	rural/clinic	No	17/01/2017	0	0	0
2014365634	233	rural/clinic	No	05/01/2017	0	0	0
2015294173	233	rural/clinic	No	12/01/2017	0	0	0
2011113934	233	rural/clinic	No	12/01/2017	0	0	0
2015372906	233	rural/clinic	No	17/01/2017	0	0	0
2014296311	233	rural/clinic	No	12/01/2017	0	0	0
2015331872	233	rural/clinic	No	12/01/2017	0	0	0
2015414782	233	rural/clinic	No	12/01/2017	0	0	0
2015372904	233	rural/clinic	No	17/01/2017	0	0	0
2015294142	233	rural/clinic	No	12/01/2017	0	0	0
2011113937	233	rural/clinic	No	12/01/2017	0	0	0
2015372903	233	rural/clinic	No	17/01/2017	0	0	0
2014321451	233	rural/clinic	No	12/01/2017	0	0	0
2014317734	233	rural/clinic	No	12/01/2017	0	0	0
2011113933	233	rural/clinic	No	12/01/2017	0	0	0
2014289474	233	rural/clinic	No	17/01/2017	0	0	0

2014317735	233	rural/clinic	No	12/01/2017	0	0	0
2015375838	233	rural/clinic	No	12/01/2017	0	0	0
2014385480	233	rural/clinic	No	12/01/2017	0	0	0
2015331871	233	rural/clinic	No	12/01/2017	0	0	0
2014289467	233	rural/clinic	No	17/01/2017	0	0	0
2013264343	233	rural/clinic	No	12/01/2017	0	0	0
2015339454	233	rural/clinic	No	12/01/2017	0	0	0
2011113936	233	rural/clinic	No	12/01/2017	0	0	0
2011141546	233	rural/clinic	No	17/01/2017	0	0	0
2014385482	233	rural/clinic	No	12/01/2017	0	0	0
2015331873	233	rural/clinic	No	12/01/2017	0	0	0
2013264383	233	rural/clinic	No	17/01/2017	0	0	0
2014385479	233	rural/clinic	No	12/01/2017	0	0	0
2015375837	233	rural/clinic	No	12/01/2017	0	0	0
2015303052	233	rural/clinic	No	17/01/2017	0	0	0
2015294174	233	rural/clinic	No	12/01/2017	0	0	0
2015289824	414	rural/clinic	No	12/01/2017	0	0	0
2015303051	414	rural/clinic	No	17/01/2017	0	0	0
2012253957	414	rural/clinic	No	12/01/2017	0	0	0
2015294175	414	rural/clinic	No	12/01/2017	0	0	0
2014317736	414	rural/clinic	No	12/01/2017	0	0	0
2015413323	414	rural/clinic	No	17/01/2017	0	0	0
2014385478	414	rural/clinic	No	12/01/2017	0	0	0
2012253954	414	rural/clinic	No	12/01/2017	0	0	0
2012285448	414	rural/clinic	No	12/01/2017	0	0	0
2013261925	414	rural/clinic	No	17/01/2017	0	0	0
2013264342	414	rural/clinic	No	12/01/2017	0	0	0
2011113938	414	rural/clinic	No	12/01/2017	0	0	0
2015413322	414	rural/clinic	No	17/01/2017	0	0	0
2013264341	414	rural/clinic	No	12/01/2017	0	0	0
2015412069	414	rural/clinic	No	12/01/2017	0	0	0
2014317737	414	rural/clinic	No	12/01/2017	0	0	0
2015413320	414	rural/clinic	No	17/01/2017	0	0	0
2014317738	414	rural/clinic	No	12/01/2017	0	0	0
2014296308	414	rural/clinic	No	12/01/2017	0	0	0
2013283240	414	rural/clinic	No	12/01/2017	0	0	0
2015412068	414	rural/clinic	No	12/01/2017	0	0	0
2015360081	414	rural/clinic	No	12/01/2017	0	0	0
2015379305	414	rural/clinic	No	12/01/2017	0	0	0
2015413321	414	rural/clinic	No	17/01/2017	0	0	0
2015414555	414	rural/clinic	No	12/01/2017	0	0	0
2015343036	414	rural/clinic	No	12/01/2017	0	0	0
2014318396	414	rural/clinic	No	12/01/2017	0	0	0
2014318395	414	rural/clinic	No	12/01/2017	0	0	0
2015413330	414	rural/clinic	No	17/01/2017	0	0	0
2014318393	414	rural/clinic	No	12/01/2017	0	0	0
2015343035	414	rural/clinic	No	12/01/2017	0	0	0
2014318394	414	rural/clinic	No	12/01/2017	0	0	0
2015414554	414	rural/clinic	No	12/01/2017	0	0	0
2015343039	414	rural/clinic	No	12/01/2017	0	0	0
2014365696	414	rural/clinic	No	17/01/2017	0	0	0
2014340263	414	rural/clinic	No	12/01/2017	0	0	0
2015343037	414	rural/clinic	No	12/01/2017	0	0	0
2014318399	414	rural/clinic	No	12/01/2017	0	0	0
2015339455	414	rural/clinic	No	12/01/2017	0	0	0
2015413331	414	rural/clinic	No	17/01/2017	0	0	0
2015414783	414	rural/clinic	No	12/01/2017	0	0	0
2014297781	414	rural/clinic	No	12/01/2017	0	0	0
2014365695	414	rural/clinic	No	17/01/2017	0	0	0
2014330344	414	rural/clinic	No	12/01/2017	0	0	0
2012378364	414	rural/clinic	No	12/01/2017	0	0	0
2014318398	414	rural/clinic	No	12/01/2017	0	0	0
2014318397	414	rural/clinic	No	12/01/2017	0	0	0
2015382851	414	rural/clinic	No	12/01/2017	0	0	0
2015413332	414	rural/clinic	No	17/01/2017	0	0	0
2013271297	414	rural/clinic	No	12/01/2017	0	0	0
2015413333	414	rural/clinic	No	17/01/2017	0	0	0
2014318829	414	rural/clinic	No	12/01/2017	0	0	0
2014326725	414	rural/clinic	No	17/01/2017	0	0	0
2014382418	414	rural/clinic	No	12/01/2017	0	0	0
2015382852	414	rural/clinic	No	12/01/2017	0	0	0
2014350678	414	rural/clinic	No	17/01/2017	0	0	0
2015382853	414	rural/clinic	No	12/01/2017	0	0	0
2012369493	414	rural/clinic	No	17/01/2017	0	0	0
2015382855	414	rural/clinic	No	12/01/2017	0	0	0
2015291439	414	rural/clinic	No	12/01/2017	0	0	0
2015382854	414	rural/clinic	No	12/01/2017	0	0	0
2015291436	755	rural/clinic	No	12/01/2017	0	0	0
2013261289	755	rural/clinic	No	17/01/2017	0	0	0
2015401520	755	rural/clinic	No	12/01/2017	0	0	0
2014362747	755	rural/clinic	No	17/01/2017	0	0	0
2015291438	755	rural/clinic	No	12/01/2017	0	0	0

2014350298	755	rural/clinic	No	17/01/2017	0	0	0
2015286253	755	rural/clinic	No	12/01/2017	0	0	0
2015291437	755	rural/clinic	No	12/01/2017	0	0	0
2014362748	755	rural/clinic	No	17/01/2017	0	0	0
2014319493	755	rural/clinic	No	12/01/2017	0	0	0
2014350300	755	rural/clinic	No	17/01/2017	0	0	0
2014362749	755	rural/clinic	No	17/01/2017	0	0	0
2015291440	755	rural/clinic	No	12/01/2017	0	0	0
2011143192	755	rural/clinic	No	12/01/2017	0	0	0
2015286256	755	rural/clinic	No	12/01/2017	0	0	0
2014350299	755	rural/clinic	No	17/01/2017	0	0	0
2015361703	755	rural/clinic	No	12/01/2017	0	0	0
2015286257	755	rural/clinic	No	12/01/2017	0	0	0
2015362405	755	rural/clinic	No	17/01/2017	0	0	0
2015412501	755	rural/clinic	No	12/01/2017	0	0	0
2014308886	755	rural/clinic	No	17/01/2017	0	0	0
2015286255	755	rural/clinic	No	12/01/2017	0	0	0
2011212249	755	rural/clinic	No	17/01/2017	0	0	0
2014385481	755	rural/clinic	No	12/01/2017	0	0	0
2015378940	755	rural/clinic	No	12/01/2017	0	0	0
2011212250	755	rural/clinic	No	17/01/2017	0	0	0
2014297898	755	rural/clinic	No	17/01/2017	0	0	0
2015378941	755	rural/clinic	No	12/01/2017	0	0	0
2014297899	755	rural/clinic	No	17/01/2017	0	0	0
2015373362	755	rural/clinic	No	12/01/2017	0	0	0
2015373367	755	rural/clinic	No	12/01/2017	0	0	0
2015373365	755	rural/clinic	No	12/01/2017	0	0	0
2014297900	755	rural/clinic	No	17/01/2017	0	0	0
2013256374	755	rural/clinic	No	17/01/2017	0	0	0
2015373366	755	rural/clinic	No	12/01/2017	0	0	0
2015373361	755	rural/clinic	No	12/01/2017	0	0	0
2015294864	755	rural/clinic	No	17/01/2017	0	0	0
2015373363	755	rural/clinic	No	12/01/2017	0	0	0
2015322501	755	rural/clinic	No	17/01/2017	0	0	0
2015373368	755	rural/clinic	No	12/01/2017	0	0	0
2015326502	755	rural/clinic	No	17/01/2017	0	0	0
2015373369	755	rural/clinic	No	12/01/2017	0	0	0
2015294865	755	rural/clinic	No	17/01/2017	0	0	0
2015373364	755	rural/clinic	No	12/01/2017	0	0	0
2015294866	755	rural/clinic	No	17/01/2017	0	0	0
2012290671	755	rural/clinic	No	12/01/2017	0	0	0
2015295189	755	rural/clinic	No	17/01/2017	0	0	0
2012290672	755	rural/clinic	No	12/01/2017	0	0	0
2014333546	755	rural/clinic	No	12/01/2017	0	0	0
2014333547	755	rural/clinic	No	12/01/2017	0	0	0
2014333548	755	rural/clinic	No	12/01/2017	0	0	0
2015335409	755	rural/clinic	No	17/01/2017	0	0	0
2015326503	755	rural/clinic	No	17/01/2017	0	0	0
2015288350	755	rural/clinic	No	12/01/2017	0	0	0
2015418942	755	rural/clinic	No	17/01/2017	0	0	0
2015355769	755	rural/clinic	No	17/01/2017	0	0	0
2014339395	755	rural/clinic	No	17/01/2017	0	0	0
2015377539	755	rural/clinic	No	17/01/2017	0	0	0
2015418943	755	rural/clinic	No	17/01/2017	0	0	0
2011232658	755	rural/clinic	No	12/01/2017	0	0	0
2015377540	755	rural/clinic	No	17/01/2017	0	0	0
2015418103	755	rural/clinic	No	17/01/2017	0	0	0
2011232660	755	rural/clinic	No	12/01/2017	0	0	0
2014328226	755	rural/clinic	No	17/01/2017	0	0	0
2015377541	755	rural/clinic	No	17/01/2017	0	0	0
2015418101	755	rural/clinic	No	17/01/2017	0	0	0
2014319627	755	rural/clinic	No	17/01/2017	0	0	0
2015418102	755	rural/clinic	No	17/01/2017	0	0	0
2015360708	755	rural/clinic	No	17/01/2017	0	0	0
2011232656	755	rural/clinic	No	12/01/2017	0	0	0
2011232659	755	rural/clinic	No	12/01/2017	0	0	0
2015368403	755	rural/clinic	No	17/01/2017	0	0	0
2015360707	755	rural/clinic	No	17/01/2017	0	0	0
2015355369	755	rural/clinic	No	17/01/2017	0	0	0
2015368404	755	rural/clinic	No	17/01/2017	0	0	0
2012295643	755	rural/clinic	No	12/01/2017	0	0	0
2014319628	755	rural/clinic	No	17/01/2017	0	0	0
2014383523	755	rural/clinic	No	12/01/2017	0	0	0
2012251522	755	rural/clinic	No	19/01/2017	0	0	0
2014302506	755	rural/clinic	No	17/01/2017	0	0	0
2015360709	755	rural/clinic	No	17/01/2017	0	0	0
2014327032	755	rural/clinic	No	19/01/2017	0	0	0
2015368407	755	rural/clinic	No	17/01/2017	0	0	0
2011145389	755	rural/clinic	No	19/01/2017	0	0	0
2011221431	755	rural/clinic	No	17/01/2017	0	0	0
2015313382	755	rural/clinic	No	17/01/2017	0	0	0
2015368410	755	rural/clinic	No	19/01/2017	0	0	0

2012259755	755	rural/clinic	No	19/01/2017	0	0	0
2011133905	755	rural/clinic	No	17/01/2017	0	0	0
2015418366	755	rural/clinic	No	17/01/2017	0	0	0
2015313383	755	rural/clinic	No	17/01/2017	0	0	0
2015368406	755	rural/clinic	No	13/01/2017	0	0	0
2011133904	755	rural/clinic	No	17/01/2017	0	0	0
2015418367	755	rural/clinic	No	17/01/2017	0	0	0
2015313384	755	rural/clinic	No	17/01/2017	0	0	0
2011221432	755	rural/clinic	No	17/01/2017	0	0	0
2015382462	755	rural/clinic	No	17/01/2017	0	0	0
2015313385	755	rural/clinic	No	17/01/2017	0	0	0
2015355365	755	rural/clinic	No	17/01/2017	0	0	0
2015382463	755	rural/clinic	No	17/01/2017	0	0	0
2015313386	755	rural/clinic	No	17/01/2017	0	0	0
2015313387	755	rural/clinic	No	17/01/2017	0	0	0
2015355364	755	rural/clinic	No	17/01/2017	0	0	0
2015368413	755	rural/clinic	No	13/01/2017	0	0	0
2015368411	755	rural/clinic	No	13/01/2017	0	0	0
2015368408	755	rural/clinic	No	17/01/2017	0	0	0
2015313388	755	rural/clinic	No	17/01/2017	0	0	0
2015382464	755	rural/clinic	No	17/01/2017	0	0	0
2015344830	755	rural/clinic	No	17/01/2017	0	0	0
2015414919	755	rural/clinic	No	17/01/2017	0	0	0
2015382465	755	rural/clinic	No	17/01/2017	0	0	0
2015414920	755	rural/clinic	No	17/01/2017	0	0	0
2015382466	755	rural/clinic	No	17/01/2017	0	0	0
2015414917	755	rural/clinic	No	17/01/2017	0	0	0
2015414918	755	rural/clinic	No	17/01/2017	0	0	0
2015382467	755	rural/clinic	No	17/01/2017	0	0	0
2015382468	755	rural/clinic	No	17/01/2017	0	0	0
2015413621	755	rural/clinic	No	17/01/2017	0	0	0
2015373328	755	rural/clinic	No	17/01/2017	0	0	0
2015413623	755	rural/clinic	No	17/01/2017	0	0	0
2015413626	755	rural/clinic	No	17/01/2017	0	0	0
2014311433	755	rural/clinic	No	17/01/2017	0	0	0
2015413624	755	rural/clinic	No	17/01/2017	0	0	0
2015355371	755	rural/clinic	No	17/01/2017	0	0	0
2014311434	755	rural/clinic	No	17/01/2017	0	0	0
2015335645	755	rural/clinic	No	17/01/2017	0	0	0
2015401526	755	rural/clinic	No	17/01/2017	0	0	0
2015377699	755	rural/clinic	No	17/01/2017	0	0	0
2012340197	755	rural/clinic	No	17/01/2017	0	0	0
2015401523	755	rural/clinic	No	17/01/2017	0	0	0
2015377700	755	rural/clinic	No	17/01/2017	0	0	0
2015286855	755	rural/clinic	No	17/01/2017	0	0	0
2015401525	755	rural/clinic	No	17/01/2017	0	0	0
2015286854	755	rural/clinic	No	17/01/2017	0	0	0
2015377601	755	rural/clinic	No	17/01/2017	0	0	0
2015401524	755	rural/clinic	No	17/01/2017	0	0	0
2014363844	755	rural/clinic	No	17/01/2017	0	0	0
2015368414	755	rural/clinic	No	13/01/2017	0	0	0
2014363865	755	rural/clinic	No	17/01/2017	0	0	0
2015377602	755	rural/clinic	No	17/01/2017	0	0	0
2015365338	755	rural/clinic	No	17/01/2017	0	0	0
2014363863	755	rural/clinic	No	17/01/2017	0	0	0
2015368416	755	rural/clinic	No	13/01/2017	0	0	0
2014358174	755	rural/clinic	No	17/01/2017	0	0	0
2014371492	755	rural/clinic	No	17/01/2017	0	0	0
2014363864	755	rural/clinic	No	17/01/2017	0	0	0
2015368415	755	rural/clinic	No	13/01/2017	0	0	0
2014358173	755	rural/clinic	No	17/01/2017	0	0	0
2012244643	755	rural/clinic	No	17/01/2017	0	0	0
2015368417	755	rural/clinic	No	13/01/2017	0	0	0
2012276141	755	rural/clinic	No	17/01/2017	0	0	0
2012346711	755	rural/clinic	No	17/01/2017	0	0	0
2014351196	755	rural/clinic	No	17/01/2017	0	0	0
2015368412	755	rural/clinic	No	13/01/2017	0	0	0
2012244276	755	rural/clinic	No	17/01/2017	0	0	0
2015405709	755	rural/clinic	No	13/01/2017	0	0	0
2014351195	755	rural/clinic	No	17/01/2017	0	0	0
2015362326	755	rural/clinic	No	17/01/2017	0	0	0
2015405711	755	rural/clinic	No	13/01/2017	0	0	0
2012246172	755	rural/clinic	No	17/01/2017	0	0	0
2014363866	755	rural/clinic	No	17/01/2017	0	0	0
2015405710	755	rural/clinic	No	13/01/2017	0	0	0
2015362325/D	755	rural/clinic	No	17/01/2017	0	0	0
2012276142	755	rural/clinic	No	17/01/2017	0	0	0
2012244641	755	rural/clinic	No	17/01/2017	0	0	0
2012275979	755	rural/clinic	No	17/01/2017	0	0	0
2014333998	755	rural/clinic	No	13/01/2017	0	0	0
2012275980	755	rural/clinic	No	17/01/2017	0	0	0
2015362324	755	rural/clinic	No	17/01/2017	0	0	0

2014329228	755	rural/clinic	No	17/01/2017	0	0	0
2015362323	755	rural/clinic	No	17/01/2017	0	0	0
2013284893	755	rural/clinic	No	17/01/2017	0	0	0
2012244275	755	rural/clinic	No	17/01/2017	0	0	0
2012246173	755	rural/clinic	No	17/01/2017	0	0	0
2014371491	755	rural/clinic	No	17/01/2017	0	0	0
2014297956	755	rural/clinic	No	17/01/2017	0	0	0
2015405708	755	rural/clinic	No	13/01/2017	0	0	0
2015405712	755	rural/clinic	No	13/01/2017	0	0	0
2012246169	755	rural/clinic	No	17/01/2017	0	0	0
2015369712	755	rural/clinic	No	17/01/2017	0	0	0
2015405705	755	rural/clinic	No	13/01/2017	0	0	0
2012244642	755	rural/clinic	No	17/01/2017	0	0	0
2015339806	755	rural/clinic	No	13/01/2017	0	0	0
2015369713	755	rural/clinic	No	17/01/2017	0	0	0
2015339807	755	rural/clinic	No	13/01/2017	0	0	0
2014346274	755	rural/clinic	No	17/01/2017	0	0	0
2013284894	755	rural/clinic	No	17/01/2017	0	0	0
2015369715	755	rural/clinic	No	17/01/2017	0	0	0
2013284892	755	rural/clinic	No	17/01/2017	0	0	0
2015369716	755	rural/clinic	No	17/01/2017	0	0	0
2015339809	755	rural/clinic	No	13/01/2017	0	0	0
2014346275	755	rural/clinic	No	17/01/2017	0	0	0
2015369714	755	rural/clinic	No	17/01/2017	0	0	0
2012246177	755	rural/clinic	No	17/01/2017	0	0	0
2015377818	755	rural/clinic	No	17/01/2017	0	0	0
2015339808	755	rural/clinic	No	13/01/2017	0	0	0
2015377817	755	rural/clinic	No	17/01/2017	0	0	0
2013270492	755	rural/clinic	No	13/01/2017	0	0	0
2015334049	755	rural/clinic	No	13/01/2017	0	0	0
2015414304	755	rural/clinic	No	17/01/2017	0	0	0
2015293915	755	rural/clinic	No	13/01/2017	0	0	0
2015414720	755	rural/clinic	No	17/01/2017	0	0	0
2015334047	755	rural/clinic	No	13/01/2017	0	0	0
2014326729	755	rural/clinic	No	11/01/2017	0	0	0
2014326729	755	rural/clinic	No	11/01/2017	0	0	0
2015405961	755	rural/clinic	No	11/01/2017	0	0	0
2015405961	755	rural/clinic	No	11/01/2017	0	0	0
2014326726	755	rural/clinic	No	11/01/2017	0	0	0
2014326726	755	rural/clinic	No	11/01/2017	0	0	0
2015414721	755	rural/clinic	No	17/01/2017	0	0	0
2015369912	755	rural/clinic	No	17/01/2017	0	0	0
2012246178/D	755	rural/clinic	No	17/01/2017	0	0	0
2015414719	755	rural/clinic	No	17/01/2017	0	0	0
2012291485	755	rural/clinic	No	17/01/2017	0	0	0
2015414718	755	rural/clinic	No	17/01/2017	0	0	0
2015418176	755	rural/clinic	No	17/01/2017	0	0	0
2012295389	755	rural/clinic	No	11/01/2017	0	0	0
2012295389	755	rural/clinic	No	11/01/2017	0	0	0
2012295392	755	rural/clinic	No	11/01/2017	0	0	0
2012295392	755	rural/clinic	No	11/01/2017	0	0	0
2012312845	755	rural/clinic	No	17/01/2017	0	0	0
2014367619	755	rural/clinic	No	17/01/2017	0	0	0
2015340821	755	rural/clinic	No	11/01/2017	0	0	0
2015340821	755	rural/clinic	No	11/01/2017	0	0	0
2011235680	755	rural/clinic	No	11/01/2017	0	0	0
2011235680	755	rural/clinic	No	11/01/2017	0	0	0
2012270100	755	rural/clinic	No	17/01/2017	0	0	0
2011156595	755	rural/clinic	No	11/01/2017	0	0	0
2011156595	755	rural/clinic	No	11/01/2017	0	0	0
2011156596	755	rural/clinic	No	11/01/2017	0	0	0
2011156596	755	rural/clinic	No	11/01/2017	0	0	0
2012312844	755	rural/clinic	No	11/01/2017	0	0	0
2012312844	755	rural/clinic	No	11/01/2017	0	0	0
2014288568	755	rural/clinic	No	11/01/2017	0	0	0
2014288568	755	rural/clinic	No	11/01/2017	0	0	0
2014362745	755	rural/clinic	No	17/01/2017	0	0	0
2012253127	755	rural/clinic	No	17/01/2017	0	0	0
2015359386	755	rural/clinic	No	11/01/2017	0	0	0
2015359386	755	rural/clinic	No	11/01/2017	0	0	0
2014362746	755	rural/clinic	No	17/01/2017	0	0	0
2012253126	755	rural/clinic	No	17/01/2017	0	0	0
2015293916	755	rural/clinic	No	13/01/2017	0	0	0
2015359822	755	rural/clinic	No	17/01/2017	0	0	0
2015397475	755	rural/clinic	No	13/01/2017	0	0	0
2015303469	755	rural/clinic	No	13/01/2017	0	0	0
2015359825	755	rural/clinic	No	17/01/2017	0	0	0
2015359824	755	rural/clinic	No	17/01/2017	0	0	0
2015397476	755	rural/clinic	No	13/01/2017	0	0	0
2015294444	755	rural/clinic	No	13/01/2017	0	0	0
2015397477	755	rural/clinic	No	13/01/2017	0	0	0
2015334051	755	rural/clinic	No	13/01/2017	0	0	0

2014311531	755	rural/clinic	No	13/01/2017	0	0	0
2015397478	755	rural/clinic	No	13/01/2017	0	0	0
2015369470	755	rural/clinic	No	13/01/2017	0	0	0
2015401521	755	rural/clinic	No	13/01/2017	0	0	0
2015373626	755	rural/clinic	No	13/01/2017	0	0	0
2015401522	755	rural/clinic	No	13/01/2017	0	0	0
2014311530	755	rural/clinic	No	13/01/2017	0	0	0
2013253025	755	rural/clinic	No	19/01/2017	0	0	0
2014309584	755	rural/clinic	No	13/01/2017	0	0	0
2014309585	755	rural/clinic	No	13/01/2017	0	0	0
2011143193	755	rural/clinic	No	19/01/2017	0	0	0
2012281048	755	rural/clinic	No	13/01/2017	0	0	0
2015382359	755	rural/clinic	No	13/01/2017	0	0	0
2015382358	755	rural/clinic	No	13/01/2017	0	0	0
2015382355	755	rural/clinic	No	13/01/2017	0	0	0
2015351121	755	rural/clinic	No	13/01/2017	0	0	0
2015286583	755	rural/clinic	No	19/01/2017	0	0	0
2014334163	755	rural/clinic	No	19/01/2017	0	0	0
2014334164	755	rural/clinic	No	19/01/2017	0	0	0
2015286581	755	rural/clinic	No	19/01/2017	0	0	0
2014313547	755	rural/clinic	No	19/01/2017	0	0	0
2014289470/D	755	rural/clinic	No	17/01/2017	0	0	0
2014344529	755	rural/clinic	No	19/01/2017	0	0	0
2012339731	755	rural/clinic	No	19/01/2017	0	0	0
2014371819	755	rural/clinic	No	19/01/2017	0	0	0
2015286582	755	rural/clinic	No	19/01/2017	0	0	0
2015297062	755	rural/clinic	No	13/01/2017	0	0	0
2014371818	755	rural/clinic	No	19/01/2017	0	0	0
2015355902	755	rural/clinic	No	19/01/2017	0	0	0
2015377950	755	rural/clinic	No	13/01/2017	0	0	0
2012261180	755	rural/clinic	No	19/01/2017	0	0	0
2015413068	755	rural/clinic	No	19/01/2017	0	0	0
2015297351	755	rural/clinic	No	13/01/2017	0	0	0
2015413069	755	rural/clinic	No	19/01/2017	0	0	0
2015369474	755	rural/clinic	No	19/01/2017	0	0	0
2015413070	755	rural/clinic	No	19/01/2017	0	0	0
2015413071	755	rural/clinic	No	19/01/2017	0	0	0
2015369471	755	rural/clinic	No	19/01/2017	0	0	0
2015413072	755	rural/clinic	No	19/01/2017	0	0	0
2015413073	755	rural/clinic	No	19/01/2017	0	0	0
2015369472	755	rural/clinic	No	19/01/2017	0	0	0
2015413074	755	rural/clinic	No	19/01/2017	0	0	0
2015297061	755	rural/clinic	No	13/01/2017	0	0	0
2015369473	755	rural/clinic	No	19/01/2017	0	0	0
2015413075	755	rural/clinic	No	19/01/2017	0	0	0
2015377942	755	rural/clinic	No	13/01/2017	0	0	0
2015373099	755	rural/clinic	No	19/01/2017	0	0	0
2015413076	755	rural/clinic	No	19/01/2017	0	0	0
2015377943	755	rural/clinic	No	13/01/2017	0	0	0
2015373096	755	rural/clinic	No	19/01/2017	0	0	0
2014335215	755	rural/clinic	No	19/01/2017	0	0	0
2015360577	755	rural/clinic	No	19/01/2017	0	0	0
2014309581	755	rural/clinic	No	19/01/2017	0	0	0
2015377946	755	rural/clinic	No	13/01/2017	0	0	0
2014335214	755	rural/clinic	No	19/01/2017	0	0	0
2015391857	755	rural/clinic	No	13/01/2017	0	0	0
2015360576	755	rural/clinic	No	19/01/2017	0	0	0
2012358216	755	rural/clinic	No	19/01/2017	0	0	0
2015391858	755	rural/clinic	No	13/01/2017	0	0	0
2015360574	755	rural/clinic	No	19/01/2017	0	0	0
2015397107	755	rural/clinic	No	13/01/2017	0	0	0
2015355372	755	rural/clinic	No	19/01/2017	0	0	0
2014344285	755	rural/clinic	No	19/01/2017	0	0	0
2015397106	755	rural/clinic	No	13/01/2017	0	0	0
2011133906	755	rural/clinic	No	19/01/2017	0	0	0
2013258604	755	rural/clinic	No	19/01/2017	0	0	0
2015397104	755	rural/clinic	No	13/01/2017	0	0	0
2015390941	755	rural/clinic	No	19/01/2017	0	0	0
2015355366	755	rural/clinic	No	19/01/2017	0	0	0
2015368409	755	rural/clinic	No	19/01/2017	0	0	0
2015397105	755	rural/clinic	No	13/01/2017	0	0	0
2014363987	755	rural/clinic	No	19/01/2017	0	0	0
2015355367	755	rural/clinic	No	19/01/2017	0	0	0
2015286258	755	rural/clinic	No	19/01/2017	0	0	0
2015355368	755	rural/clinic	No	19/01/2017	0	0	0
2014289708	755	rural/clinic	No	19/01/2017	0	0	0
2015286261	755	rural/clinic	No	19/01/2017	0	0	0
2015373373	755	rural/clinic	No	19/01/2017	0	0	0
2015286260	755	rural/clinic	No	19/01/2017	0	0	0
2015373372	755	rural/clinic	No	19/01/2017	0	0	0
2015286259	755	rural/clinic	No	19/01/2017	0	0	0
2015373371	755	rural/clinic	No	19/01/2017	0	0	0

2014324231	755	rural/clinic	No	19/01/2017	0	0	0
2015373370	755	rural/clinic	No	19/01/2017	0	0	0
2013267193	755	rural/clinic	No	19/01/2017	0	0	0
2015373330	755	rural/clinic	No	19/01/2017	0	0	0
2013267192	755	rural/clinic	No	19/01/2017	0	0	0
2015373331	755	rural/clinic	No	19/01/2017	0	0	0
2015373332	755	rural/clinic	No	19/01/2017	0	0	0
2013267191	755	rural/clinic	No	19/01/2017	0	0	0
2015373329	755	rural/clinic	No	19/01/2017	0	0	0
2015377003	755	rural/clinic	No	19/01/2017	0	0	0
2015401529	755	rural/clinic	No	19/01/2017	0	0	0
2015377004	755	rural/clinic	No	19/01/2017	0	0	0
2015359826	755	rural/clinic	No	13/01/2017	0	0	0
2015401528	755	rural/clinic	No	19/01/2017	0	0	0
2015359823	755	rural/clinic	No	13/01/2017	0	0	0
2015377005	755	rural/clinic	No	19/01/2017	0	0	0
2015359821	755	rural/clinic	No	13/01/2017	0	0	0
2012284022	755	rural/clinic	No	13/01/2017	0	0	0
2015401527	755	rural/clinic	No	19/01/2017	0	0	0
2015377006	755	rural/clinic	No	19/01/2017	0	0	0
2012253237	755	rural/clinic	No	13/01/2017	0	0	0
2011226471	755	rural/clinic	No	19/01/2017	0	0	0
2012384774	755	rural/clinic	No	19/01/2017	0	0	0
2015342273	755	rural/clinic	No	13/01/2017	0	0	0
2012265633	755	rural/clinic	No	19/01/2017	0	0	0
2012384775	755	rural/clinic	No	19/01/2017	0	0	0
2015342274	755	rural/clinic	No	13/01/2017	0	0	0
2012253199	755	rural/clinic	No	13/01/2017	0	0	0
2014339736	755	rural/clinic	No	19/01/2017	0	0	0
2014301452	755	rural/clinic	No	13/01/2017	0	0	0
2014333549	755	rural/clinic	No	19/01/2017	0	0	0
2015335365	755	rural/clinic	No	19/01/2017	0	0	0
2014301453	755	rural/clinic	No	13/01/2017	0	0	0
2015361781	755	rural/clinic	No	19/01/2017	0	0	0
20143335550	755	rural/clinic	No	19/01/2017	0	0	0
2015297060	755	rural/clinic	No	13/01/2017	0	0	0
2014357505	755	rural/clinic	No	19/01/2017	0	0	0
2015305231	755	rural/clinic	No	19/01/2017	0	0	0
2014357507	755	rural/clinic	No	19/01/2017	0	0	0
2015305230	755	rural/clinic	No	19/01/2017	0	0	0
2014357506	755	rural/clinic	No	19/01/2017	0	0	0
2015305232	755	rural/clinic	No	19/01/2017	0	0	0
2014357508	755	rural/clinic	No	19/01/2017	0	0	0
2014357509	755	rural/clinic	No	19/01/2017	0	0	0
2015305233	755	rural/clinic	No	19/01/2017	0	0	0
2015358115	755	rural/clinic	No	19/01/2017	0	0	0
2015297059	755	rural/clinic	No	13/01/2017	0	0	0
2015358114	755	rural/clinic	No	19/01/2017	0	0	0
2015305234	755	rural/clinic	No	19/01/2017	0	0	0
2015358113	755	rural/clinic	No	19/01/2017	0	0	0
2015297056/d	755	rural/clinic	No	13/01/2017	0	0	0
2014381897	755	rural/clinic	No	19/01/2017	0	0	0
2014301451	755	rural/clinic	No	13/01/2017	0	0	0
2015358257	755	rural/clinic	No	19/01/2017	0	0	0
2015297253	755	rural/clinic	No	13/01/2017	0	0	0
2015358256	755	rural/clinic	No	19/01/2017	0	0	0
2015305229	755	rural/clinic	No	19/01/2017	0	0	0
2015358258	755	rural/clinic	No	19/01/2017	0	0	0
2015297252	755	rural/clinic	No	13/01/2017	0	0	0
2015358259	755	rural/clinic	No	19/01/2017	0	0	0
2015305224	755	rural/clinic	No	19/01/2017	0	0	0
2015297251	755	rural/clinic	No	13/01/2017	0	0	0
2015358116	755	rural/clinic	No	19/01/2017	0	0	0
2014326077	755	rural/clinic	No	13/01/2017	0	0	0
2014326075	755	rural/clinic	No	13/01/2017	0	0	0
2015305225	755	rural/clinic	No	19/01/2017	0	0	0
2011155220	755	rural/clinic	No	19/01/2017	0	0	0
2014326074	755	rural/clinic	No	13/01/2017	0	0	0
2014326076	755	rural/clinic	No	13/01/2017	0	0	0
2015368131	755	rural/clinic	No	19/01/2017	0	0	0
2015412763	755	rural/clinic	No	13/01/2017	0	0	0
2015305226	755	rural/clinic	No	19/01/2017	0	0	0
2015368132	755	rural/clinic	No	19/01/2017	0	0	0
2012376645	755	rural/clinic	No	13/01/2017	0	0	0
2011198684	755	rural/clinic	No	19/01/2017	0	0	0
2015305227	755	rural/clinic	No	19/01/2017	0	0	0
2012376644	755	rural/clinic	No	13/01/2017	0	0	0
2014372436	755	rural/clinic	No	19/01/2017	0	0	0
2015305228	755	rural/clinic	No	19/01/2017	0	0	0
2014335991	755	rural/clinic	No	13/01/2017	0	0	0
2012331319	755	rural/clinic	No	19/01/2017	0	0	0
2012376774	755	rural/clinic	No	13/01/2017	0	0	0

2015385510	755	rural/clinic	No	19/01/2017	0	0	0
2014369839	755	rural/clinic	No	13/01/2017	0	0	0
2012331317	755	rural/clinic	No	19/01/2017	0	0	0
2014383024	755	rural/clinic	No	13/01/2017	0	0	0
2012291534	755	rural/clinic	No	19/01/2017	0	0	0
2015385509	755	rural/clinic	No	19/01/2017	0	0	0
2014383025	755	rural/clinic	No	13/01/2017	0	0	0
2012291535	755	rural/clinic	No	19/01/2017	0	0	0
2011163953	755	rural/clinic	No	19/01/2017	0	0	0
2011115899	755	rural/clinic	No	13/01/2017	0	0	0
2015385508	755	rural/clinic	No	19/01/2017	0	0	0
2012363272	755	rural/clinic	No	13/01/2017	0	0	0
2014383524	755	rural/clinic	No	19/01/2017	0	0	0
2015346359	755	rural/clinic	No	19/01/2017	0	0	0
2012363273	755	rural/clinic	No	13/01/2017	0	0	0
2014383522	755	rural/clinic	No	19/01/2017	0	0	0
2013255443	755	rural/clinic	No	19/01/2017	0	0	0
2015346360	755	rural/clinic	No	19/01/2017	0	0	0
2012291533	755	rural/clinic	No	19/01/2017	0	0	0
2015305235	755	rural/clinic	No	19/01/2017	0	0	0
2012363275	755	rural/clinic	No	13/01/2017	0	0	0
2013266107	755	rural/clinic	No	17/01/2017	0	0	0
2015346361	755	rural/clinic	No	19/01/2017	0	0	0
2012361048	755	rural/clinic	No	19/01/2017	0	0	0
2012363269	755	rural/clinic	No	17/01/2017	0	0	0
2012363271	755	rural/clinic	No	17/01/2017	0	0	0
2015346362	755	rural/clinic	No	19/01/2017	0	0	0
2012387241	755	rural/clinic	No	19/01/2017	0	0	0
2012290673	755	rural/clinic	No	19/01/2017	0	0	0
2015346363	755	rural/clinic	No	19/01/2017	0	0	0
2014349911	755	rural/clinic	No	17/01/2017	0	0	0
2015373641	755	rural/clinic	No	19/01/2017	0	0	0
2015373650	755	rural/clinic	No	19/01/2017	0	0	0
2015357358	755	rural/clinic	No	17/01/2017	0	0	0
2015346364	755	rural/clinic	No	19/01/2017	0	0	0
2012368532	755	rural/clinic	No	17/01/2017	0	0	0
2015373649	755	rural/clinic	No	19/01/2017	0	0	0
2014349912	755	rural/clinic	No	17/01/2017	0	0	0
2015357357	755	rural/clinic	No	17/01/2017	0	0	0
2015302484	755	rural/clinic	No	17/01/2017	0	0	0
2015373648	755	rural/clinic	No	19/01/2017	0	0	0
2015302485	755	rural/clinic	No	17/01/2017	0	0	0
2015373647	755	rural/clinic	No	19/01/2017	0	0	0
2015302486	755	rural/clinic	No	17/01/2017	0	0	0
2015373646	755	rural/clinic	No	19/01/2017	0	0	0
2012368531	755	rural/clinic	No	17/01/2017	0	0	0
2015373645	755	rural/clinic	No	19/01/2017	0	0	0
2015413975	755	rural/clinic	No	17/01/2017	0	0	0
2015373644	755	rural/clinic	No	19/01/2017	0	0	0
2015301435	755	rural/clinic	No	19/01/2017	0	0	0
2012259753	755	rural/clinic	No	17/01/2017	0	0	0
2015373643	755	rural/clinic	No	19/01/2017	0	0	0
2012259754	755	rural/clinic	No	17/01/2017	0	0	0
2015289162	755	rural/clinic	No	17/01/2017	0	0	0
2015373642	755	rural/clinic	No	19/01/2017	0	0	0
2015301436	755	rural/clinic	No	19/01/2017	0	0	0
2014338235	755	rural/clinic	No	17/01/2017	0	0	0
2015332006	755	rural/clinic	No	19/01/2017	0	0	0
2015365339	755	rural/clinic	No	17/01/2017	0	0	0
2015408091	755	rural/clinic	No	19/01/2017	0	0	0
2015340822	755	rural/clinic	No	19/01/2017	0	0	0
2015408093	755	rural/clinic	No	19/01/2017	0	0	0
2015365340	755	rural/clinic	No	17/01/2017	0	0	0
2015408090	755	rural/clinic	No	19/01/2017	0	0	0
2015340823	755	rural/clinic	No	19/01/2017	0	0	0
2015365348	755	rural/clinic	No	17/01/2017	0	0	0
2015408089	755	rural/clinic	No	19/01/2017	0	0	0
2015365345	755	rural/clinic	No	17/01/2017	0	0	0
2014290234	755	rural/clinic	No	19/01/2017	0	0	0
2013264051	755	rural/clinic	No	17/01/2017	0	0	0
2015408092	755	rural/clinic	No	19/01/2017	0	0	0
2014341800	755	rural/clinic	No	17/01/2017	0	0	0
2014341799	755	rural/clinic	No	17/01/2017	0	0	0
2015301437	755	rural/clinic	No	19/01/2017	0	0	0
2015334053	755	rural/clinic	No	19/01/2017	0	0	0
2015414367	755	rural/clinic	No	17/01/2017	0	0	0
2015414368	755	rural/clinic	No	17/01/2017	0	0	0
2015334054	755	rural/clinic	No	19/01/2017	0	0	0
2012242574	755	rural/clinic	No	17/01/2017	0	0	0
2015301438	755	rural/clinic	No	19/01/2017	0	0	0
2012242572	755	rural/clinic	No	17/01/2017	0	0	0
201052000	755	rural/clinic	No	17/01/2017	0	0	0

2014371817	755	rural/clinic	No	19/01/2017	0	0	0
2014347814	755	rural/clinic	No	17/01/2017	0	0	0
2011222296	755	rural/clinic	No	19/01/2017	0	0	0
2015351122	755	rural/clinic	No	17/01/2017	0	0	0
2011200182	755	rural/clinic	No	19/01/2017	0	0	0
2015351123	755	rural/clinic	No	17/01/2017	0	0	0
2015334401	755	rural/clinic	No	19/01/2017	0	0	0
2012365139	755	rural/clinic	No	17/01/2017	0	0	0
2012265280	755	rural/clinic	No	19/01/2017	0	0	0
2011192486	755	rural/clinic	No	19/01/2017	0	0	0
2015357626	755	rural/clinic	No	19/01/2017	0	0	0
2015291442	755	rural/clinic	No	19/01/2017	0	0	0
2014357150	755	rural/clinic	No	19/01/2017	0	0	0
2015349522	755	rural/clinic	No	19/01/2017	0	0	0
2011224027	755	rural/clinic	No	19/01/2017	0	0	0
2015291441	755	rural/clinic	No	19/01/2017	0	0	0
2013271298	755	rural/clinic	No	19/01/2017	0	0	0
2014318830	755	rural/clinic	No	19/01/2017	0	0	0
2014318831	755	rural/clinic	No	19/01/2017	0	0	0
2014318832	755	rural/clinic	No	19/01/2017	0	0	0
2015361676	755	rural/clinic	No	19/01/2017	0	0	0
2015412502	903	rural/clinic	No	19/01/2017	0	0	0
2014315519	903	rural/clinic	No	19/01/2017	0	0	0
2014315518	903	rural/clinic	No	19/01/2017	0	0	0
2014315520	903	rural/clinic	No	19/01/2017	0	0	0
2015291443	903	rural/clinic	No	19/01/2017	0	0	0
2015361677	903	rural/clinic	No	19/01/2017	0	0	0
2015361679	903	rural/clinic	No	19/01/2017	0	0	0
2014305212	903	rural/clinic	No	19/01/2017	0	0	0
2015325036	903	rural/clinic	No	19/01/2017	0	0	0
2012251529	903	rural/clinic	No	19/01/2017	0	0	0
2012251528	903	rural/clinic	No	19/01/2017	0	0	0
2014368015	903	rural/clinic	No	19/01/2017	0	0	0
2015349716	903	rural/clinic	No	19/01/2017	0	0	0
2014303337	903	rural/clinic	No	19/01/2017	0	0	0
2014303336	903	rural/clinic	No	19/01/2017	0	0	0
2015349715	903	rural/clinic	No	19/01/2017	0	0	0
2015349717	170	rural/clinic	No	19/01/2017	0	0	0
2015325035	170	rural/clinic	No	19/01/2017	0	0	0
2015349923	170	rural/clinic	No	19/01/2017	0	0	0
2015349925	170	rural/clinic	No	19/01/2017	0	0	0
2015349924	170	rural/clinic	No	19/01/2017	0	0	0
2015349922	170	rural/clinic	No	19/01/2017	0	0	0
2011225364	170	rural/clinic	No	19/01/2017	0	0	0
2011179929	170	rural/clinic	No	19/01/2017	0	0	0
2014368014	170	rural/clinic	No	19/01/2017	0	0	0
2012304196	170	rural/clinic	No	19/01/2017	0	0	0
2011199072	170	rural/clinic	No	19/01/2017	0	0	0
2011216823	170	rural/clinic	No	19/01/2017	0	0	0
2014332076	170	rural/clinic	No	19/01/2017	0	0	0
2011223934	170	rural/clinic	No	19/01/2017	0	0	0
2014357149	170	rural/clinic	No	19/01/2017	0	0	0
2014327033	170	rural/clinic	No	19/01/2017	0	0	0
2014288967	170	rural/clinic	No	19/01/2017	0	0	0
2015295641	170	rural/clinic	No	19/01/2017	0	0	0
2014288968	170	rural/clinic	No	19/01/2017	0	0	0
2015295640	170	rural/clinic	No	19/01/2017	0	0	0
2015397025	170	rural/clinic	No	19/01/2017	0	0	0
2014358724	902	rural/clinic	No	17/01/2017	0	0	0
2014358726	902	rural/clinic	No	17/01/2017	0	0	0
2014358725	902	rural/clinic	No	17/01/2017	0	0	0
2014358727	902	rural/clinic	No	17/01/2017	0	0	0
2014358721	902	rural/clinic	No	17/01/2017	0	0	0
2014358723	902	rural/clinic	No	17/01/2017	0	0	0
2014358722	902	rural/clinic	No	17/01/2017	0	0	0
2014346937	902	rural/clinic	No	17/01/2017	0	0	0
2014346936	902	rural/clinic	No	17/01/2017	0	0	0
2011144006	902	rural/clinic	No	17/01/2017	0	0	0
2015331604	902	rural/clinic	No	17/01/2017	0	0	0
2015331610	523	rural/clinic	No	17/01/2017	0	0	0
2013264346	523	rural/clinic	No	17/01/2017	0	0	0
2013264345	523	rural/clinic	No	17/01/2017	0	0	0
2015294144	523	rural/clinic	No	17/01/2017	0	0	0
2015294143	523	rural/clinic	No	17/01/2017	0	0	0
2014385483	523	rural/clinic	No	17/01/2017	0	0	0
2014385484	523	rural/clinic	No	17/01/2017	0	0	0
2014385485	523	rural/clinic	No	17/01/2017	0	0	0
2012300986	150	rural/clinic	No	19/01/2017	0	0	0
2011142904	150	rural/clinic	No	19/01/2017	0	0	0
2015293256	150	rural/clinic	No	19/01/2017	0	0	0
2014319492	150	rural/clinic	No	19/01/2017	0	0	0
2014317338	150	rural/clinic	No	19/01/2017	0	0	0

2014306080	150	rural/clinic	No	19/01/2017	0	0	0
2014306078	150	rural/clinic	No	19/01/2017	0	0	0
2014306079	150	rural/clinic	No	19/01/2017	0	0	0
2011232666	150	rural/clinic	No	19/01/2017	0	0	0
2011232668	150	rural/clinic	No	19/01/2017	0	0	0
2011232664	150	rural/clinic	No	19/01/2017	0	0	0
2012369800	150	rural/clinic	No	19/01/2017	0	0	0
2015384352	150	rural/clinic	No	12/01/2017	0	0	0
2015384352	150	rural/clinic	No	12/01/2017	0	0	0
2013247130	150	rural/clinic	No	12/01/2017	0	0	0
2013247130	150	rural/clinic	No	12/01/2017	0	0	0
2011232667	150	rural/clinic	No	19/01/2017	0	0	0
2011232661	150	rural/clinic	No	19/01/2017	0	0	0
2015357352	150	rural/clinic	No	11/01/2017	0	0	0
2015357352	150	rural/clinic	No	11/01/2017	0	0	0
2015357354	150	rural/clinic	No	11/01/2017	0	0	0
2015357354	150	rural/clinic	No	11/01/2017	0	0	0
2011232662	150	rural/clinic	No	19/01/2017	0	0	0
2014307312	150	rural/clinic	No	19/01/2017	0	0	0
2014307310	88	rural/clinic	No	19/01/2017	0	0	0
2014307314	88	rural/clinic	No	19/01/2017	0	0	0
2014317337	88	rural/clinic	No	19/01/2017	0	0	0
2012369799	88	rural/clinic	No	19/01/2017	0	0	0
2014307316	88	rural/clinic	No	19/01/2017	0	0	0
2014307313	88	rural/clinic	No	19/01/2017	0	0	0
2014307311	88	rural/clinic	No	19/01/2017	0	0	0
2014307317	88	rural/clinic	No	19/01/2017	0	0	0
2014307315	88	rural/clinic	No	19/01/2017	0	0	0
2014307318	88	rural/clinic	No	19/01/2017	0	0	0
2015335181	88	rural/clinic	No	19/01/2017	0	0	0
2015335180	88	rural/clinic	No	19/01/2017	0	0	0
2015360084	88	rural/clinic	No	19/01/2017	0	0	0
2012389490	88	rural/clinic	No	19/01/2017	0	0	0
2014378468	88	rural/clinic	No	19/01/2017	0	0	0
2014355836	198	rural/clinic	No	11/01/2017	0	0	0
2014355836	198	rural/clinic	No	11/01/2017	0	0	0
2014358274	198	rural/clinic	No	19/01/2017	0	0	0
2014378467	198	rural/clinic	No	19/01/2017	0	0	0
2014317604	198	rural/clinic	No	19/01/2017	0	0	0
2014317611	198	rural/clinic	No	19/01/2017	0	0	0
2014371606	198	rural/clinic	No	19/01/2017	0	0	0
2014317607	198	rural/clinic	No	19/01/2017	0	0	0
2014317605	198	rural/clinic	No	19/01/2017	0	0	0
2013283239	198	rural/clinic	No	19/01/2017	0	0	0
2014317612	9	rural/clinic	No	19/01/2017	0	0	0
2014317610	9	rural/clinic	No	19/01/2017	0	0	0
2013283238	9	rural/clinic	No	19/01/2017	0	0	0
2014317424	9	rural/clinic	No	19/01/2017	0	0	0
2014317608	9	rural/clinic	No	19/01/2017	0	0	0
2014317609	9	rural/clinic	No	19/01/2017	0	0	0
2014317603	9	rural/clinic	No	19/01/2017	0	0	0
2015346365	9	rural/clinic	No	19/01/2017	0	0	0
2013255738	9	rural/clinic	No	05/01/2017	0	0	0
2013255738	9	rural/clinic	No	05/01/2017	0	0	0
2015377417	9	rural/clinic	No	05/01/2017	0	0	0
2015377417	9	rural/clinic	No	05/01/2017	0	0	0
2015377416	9	rural/clinic	No	05/01/2017	0	0	0
2015377416	9	rural/clinic	No	05/01/2017	0	0	0
2015413054	9	rural/clinic	No	05/01/2017	0	0	0
2015413054	9	rural/clinic	No	05/01/2017	0	0	0
2015286251	9	rural/clinic	No	05/01/2017	0	0	0
2015286251	9	rural/clinic	No	05/01/2017	0	0	0
2014364000	9	rural/clinic	No	05/01/2017	0	0	0
2014364000	9	rural/clinic	No	05/01/2017	0	0	0
2015351125	9	rural/clinic	No	23/01/2017	0	0	0
2015397480	9	rural/clinic	No	23/01/2017	0	0	0
2015397479	9	rural/clinic	No	23/01/2017	0	0	0
19703WRONG	9	rural/clinic	No	23/01/2017	0	0	0
2015373202	9	rural/clinic	No	23/01/2017	0	0	0
2015351124	9	rural/clinic	No	23/01/2017	0	0	0
2015392424	9	rural/clinic	No	23/01/2017	0	0	0
2014311538	9	rural/clinic	No	23/01/2017	0	0	0
2012291536	9	rural/clinic	No	23/01/2017	0	0	0
2011148053	9	rural/clinic	No	23/01/2017	0	0	0
2011148054	9	rural/clinic	No	23/01/2017	0	0	0
2015374397	9	rural/clinic	No	23/01/2017	0	0	0
2015374398	9	rural/clinic	No	23/01/2017	0	0	0
2015350484	9	rural/clinic	No	23/01/2017	0	0	0
2015350485	89	rural/clinic	No	23/01/2017	0	0	0
2015350486	89	rural/clinic	No	23/01/2017	0	0	0
201550487	89	rural/clinic	No	23/01/2017	0	0	0
2015350489	89	rural/clinic	No	23/01/2017	0	0	0

2015374301	89	rural/clinic	No	23/01/2017	0	0	0
2015392428	89	rural/clinic	No	23/01/2017	0	0	0
2014296872	89	rural/clinic	No	23/01/2017	0	0	0
2015392427	89	rural/clinic	No	23/01/2017	0	0	0
2015362407	89	rural/clinic	No	23/01/2017	0	0	0
2015328506	89	rural/clinic	No	23/01/2017	0	0	0
2015322559	89	rural/clinic	No	23/01/2017	0	0	0
2015322558	89	rural/clinic	No	23/01/2017	0	0	0
2015319424	89	rural/clinic	No	23/01/2017	0	0	0
2015319422	89	rural/clinic	No	23/01/2017	0	0	0
2015401530	89	rural/clinic	No	23/01/2017	0	0	0
2015401532	89	rural/clinic	No	23/01/2017	0	0	0
2015300659	89	rural/clinic	No	23/01/2017	0	0	0
2015300661	89	rural/clinic	No	23/01/2017	0	0	0
2013256366	89	rural/clinic	No	23/01/2017	0	0	0
2015362408	89	rural/clinic	No	23/01/2017	0	0	0
2015300660	89	rural/clinic	No	23/01/2017	0	0	0
2015362409	89	rural/clinic	No	23/01/2017	0	0	0
2015362406	89	rural/clinic	No	23/01/2017	0	0	0
2015362410	904	rural/clinic	No	23/01/2017	0	0	0
2015314781	904	rural/clinic	No	23/01/2017	0	0	0
2014363750	904	rural/clinic	No	23/01/2017	0	0	0
2013248181	904	rural/clinic	No	23/01/2017	0	0	0
2015300658	904	rural/clinic	No	23/01/2017	0	0	0
2013256372	904	rural/clinic	No	23/01/2017	0	0	0
2013256371	905	district/faith-based	No	23/01/2017	1	0	0
2013256373	905	district/faith-based	No	23/01/2017	1	0	0
2012350616	905	district/faith-based	No	23/01/2017	1	0	0
2012291538	905	district/faith-based	No	23/01/2017	1	0	0
2012291540	905	district/faith-based	No	23/01/2017	1	0	0
2012291539	905	district/faith-based	No	23/01/2017	1	0	0
2012291537	905	district/faith-based	No	23/01/2017	1	0	0
2015392422	905	district/faith-based	No	23/01/2017	1	0	0
2015392421	756	rural/clinic	No	23/01/2017	0	0	0
2015328508	756	rural/clinic	No	23/01/2017	0	0	0
2015328507	756	rural/clinic	No	23/01/2017	0	0	0
2012350615	756	rural/clinic	No	23/01/2017	0	0	0
2013278286	756	rural/clinic	No	23/01/2017	0	0	0
2015372907	756	rural/clinic	No	17/01/2017	0	0	0
2015372907	756	rural/clinic	No	17/01/2017	0	0	0
2015397476	756	rural/clinic	No	13/01/2017	0	0	0
2015397476	756	rural/clinic	No	13/01/2017	0	0	0
2015392528	756	rural/clinic	No	23/01/2017	0	0	0
2015392526	756	rural/clinic	No	23/01/2017	0	0	0
2013278287	756	rural/clinic	No	23/01/2017	0	0	0
2012251525	74	rural/clinic	No	12/01/2017	0	0	0
2012251525	74	rural/clinic	No	12/01/2017	0	0	0
2015335363	74	rural/clinic	No	12/01/2017	0	0	0
2015335363	74	rural/clinic	No	12/01/2017	0	0	0
2012251524	74	rural/clinic	No	12/01/2017	0	0	0
2012251524	74	rural/clinic	No	12/01/2017	0	0	0
2014386112	74	rural/clinic	No	05/01/2017	0	0	0
2014386112	74	rural/clinic	No	05/01/2017	0	0	0
2015405084	74	rural/clinic	No	05/01/2017	0	0	0
2015405084	74	rural/clinic	No	05/01/2017	0	0	0
2012341035	74	rural/clinic	No	05/01/2017	0	0	0
2012341035	74	rural/clinic	No	05/01/2017	0	0	0
2015385420	74	rural/clinic	No	11/01/2017	0	0	0
2015385420	74	rural/clinic	No	11/01/2017	0	0	0
2015301080	74	rural/clinic	No	12/01/2017	0	0	0
2015301080	74	rural/clinic	No	12/01/2017	0	0	0
2015383571	74	rural/clinic	No	12/01/2017	0	0	0
2015383571	74	rural/clinic	No	12/01/2017	0	0	0
2015392525	74	rural/clinic	No	23/01/2017	0	0	0
2014338238	151	rural/clinic	No	23/01/2017	0	0	0
2015405708	151	rural/clinic	No	13/01/2017	0	0	0
2015405708	151	rural/clinic	No	13/01/2017	0	0	0
2013267195	151	rural/clinic	No	23/01/2017	0	0	0
2014338234	151	rural/clinic	No	23/01/2017	0	0	0
2014338237	151	rural/clinic	No	23/01/2017	0	0	0
2014338236	151	rural/clinic	No	23/01/2017	0	0	0
2014311536	151	rural/clinic	No	23/01/2017	0	0	0
2014363747	151	rural/clinic	No	23/01/2017	0	0	0
2014336893	151	rural/clinic	No	11/01/2017	0	0	0
2014336893	151	rural/clinic	No	11/01/2017	0	0	0
2015390940	151	rural/clinic	No	23/01/2017	0	0	0
2015390939	151	rural/clinic	No	23/01/2017	0	0	0
2015390938	151	rural/clinic	No	23/01/2017	0	0	0
2015322714	151	rural/clinic	No	23/01/2017	0	0	0
2015373204	151	rural/clinic	No	23/01/2017	0	0	0
2015373205	151	rural/clinic	No	23/01/2017	0	0	0
2015373206	151	rural/clinic	No	23/01/2017	0	0	0

2015373201	151	rural/clinic	No	23/01/2017	0	0	0
2015373100	151	rural/clinic	No	23/01/2017	0	0	0
2015373083	151	rural/clinic	No	23/01/2017	0	0	0
2015322561	151	rural/clinic	No	23/01/2017	0	0	0
2015322562	151	rural/clinic	No	23/01/2017	0	0	0
2015322565	151	rural/clinic	No	23/01/2017	0	0	0
2014311537	151	rural/clinic	No	23/01/2017	0	0	0
2015322566	151	rural/clinic	No	23/01/2017	0	0	0
2015344831	151	rural/clinic	No	23/01/2017	0	0	0
2015413675	151	rural/clinic	No	23/01/2017	0	0	0
2015392419	151	rural/clinic	No	23/01/2017	0	0	0
2015413676	151	rural/clinic	No	23/01/2017	0	0	0
2015413679	151	rural/clinic	No	23/01/2017	0	0	0
2015413680	151	rural/clinic	No	23/01/2017	0	0	0
2015306820	151	rural/clinic	No	23/01/2017	0	0	0
2015306821	541	rural/clinic	No	23/01/2017	0	0	0
2015413673	541	rural/clinic	No	23/01/2017	0	0	0
2015413677	541	rural/clinic	No	23/01/2017	0	0	0
2012280588	541	rural/clinic	No	23/01/2017	0	0	0
2012272324	541	rural/clinic	No	23/01/2017	0	0	0
2012280585	541	rural/clinic	No	23/01/2017	0	0	0
2012280590	541	rural/clinic	No	23/01/2017	0	0	0
2014353619	541	rural/clinic	No	23/01/2017	0	0	0
2015362661	541	rural/clinic	No	24/01/2017	0	0	0
2015362660	541	rural/clinic	No	24/01/2017	0	0	0
2015362659	541	rural/clinic	No	24/01/2017	0	0	0
2015362658	541	rural/clinic	No	24/01/2017	0	0	0
2015394616	541	rural/clinic	No	23/01/2017	0	0	0
2015362657	541	rural/clinic	No	24/01/2017	0	0	0
2015394617	541	rural/clinic	No	23/01/2017	0	0	0
2015362656	541	rural/clinic	No	24/01/2017	0	0	0
2012280589	541	rural/clinic	No	23/01/2017	0	0	0
2015303174	906	rural/clinic	No	23/01/2017	0	0	0
2015359827	906	rural/clinic	No	24/01/2017	0	0	0
2015295330	906	rural/clinic	No	23/01/2017	0	0	0
2012253238	906	rural/clinic	No	24/01/2017	0	0	0
2015295328	906	rural/clinic	No	23/01/2017	0	0	0
2012312846	906	rural/clinic	No	24/01/2017	0	0	0
2014326735	906	rural/clinic	No	23/01/2017	0	0	0
2014326732	906	rural/clinic	No	23/01/2017	0	0	0
2015377603	906	rural/clinic	No	24/01/2017	0	0	0
2014326733	906	rural/clinic	No	23/01/2017	0	0	0
2014326734	906	rural/clinic	No	23/01/2017	0	0	0
2015385342	906	rural/clinic	No	24/01/2017	0	0	0
2015385344	906	rural/clinic	No	24/01/2017	0	0	0
2015385345	906	rural/clinic	No	24/01/2017	0	0	0
2015385343	906	rural/clinic	No	24/01/2017	0	0	0
2015385334	906	rural/clinic	No	24/01/2017	0	0	0
2015295190	906	rural/clinic	No	24/01/2017	0	0	0
2014363584	906	rural/clinic	No	24/01/2017	0	0	0
2014363586	906	rural/clinic	No	24/01/2017	0	0	0
2014363585	906	rural/clinic	No	24/01/2017	0	0	0
2015355720	906	rural/clinic	No	24/01/2017	0	0	0
2015355719	906	rural/clinic	No	24/01/2017	0	0	0
2013256375	906	rural/clinic	No	24/01/2017	0	0	0
2015377542	906	rural/clinic	No	24/01/2017	0	0	0
2013256376	906	rural/clinic	No	24/01/2017	0	0	0
2013256377	906	rural/clinic	No	24/01/2017	0	0	0
2012317279	906	rural/clinic	No	24/01/2017	0	0	0
2015335646	906	rural/clinic	No	24/01/2017	0	0	0
2015335647	906	rural/clinic	No	24/01/2017	0	0	0
2015335648	906	rural/clinic	No	24/01/2017	0	0	0
2015335649	906	rural/clinic	No	24/01/2017	0	0	0
2012295952	906	rural/clinic	No	24/01/2017	0	0	0
2012295953	906	rural/clinic	No	24/01/2017	0	0	0
2012295396	906	rural/clinic	No	24/01/2017	0	0	0
2012295397	906	rural/clinic	No	24/01/2017	0	0	0
2012295398	906	rural/clinic	No	24/01/2017	0	0	0
2012295399	906	rural/clinic	No	24/01/2017	0	0	0
2012295400	906	rural/clinic	No	24/01/2017	0	0	0
2015382860	906	rural/clinic	No	24/01/2017	0	0	0
2015382859	906	rural/clinic	No	24/01/2017	0	0	0
2015382858	906	rural/clinic	No	24/01/2017	0	0	0
2015382857	906	rural/clinic	No	24/01/2017	0	0	0
2015382856	906	rural/clinic	No	24/01/2017	0	0	0
2012246181	906	rural/clinic	No	24/01/2017	0	0	0
2012246180	906	rural/clinic	No	24/01/2017	0	0	0
2012246175	906	rural/clinic	No	24/01/2017	0	0	0
2012246179	906	rural/clinic	No	24/01/2017	0	0	0
2012246176	906	rural/clinic	No	24/01/2017	0	0	0
2014346277	906	rural/clinic	No	24/01/2017	0	0	0
2012291445	906	rural/clinic	No	24/01/2017	0	0	0

2014375595	906	rural/clinic	No	24/01/2017	0	0	0
2014367620	906	rural/clinic	No	24/01/2017	0	0	0
2012365468	906	rural/clinic	No	24/01/2017	0	0	0
2015377819	906	rural/clinic	No	24/01/2017	0	0	0
2015377820	906	rural/clinic	No	24/01/2017	0	0	0
2015414306	906	rural/clinic	No	24/01/2017	0	0	0
2015414305	906	rural/clinic	No	24/01/2017	0	0	0
2015414307	906	rural/clinic	No	24/01/2017	0	0	0
2015390256	906	rural/clinic	No	24/01/2017	0	0	0
2015414308	906	rural/clinic	No	24/01/2017	0	0	0
2014297957	906	rural/clinic	No	24/01/2017	0	0	0
2015418106	906	rural/clinic	No	24/01/2017	0	0	0
2015418105	906	rural/clinic	No	24/01/2017	0	0	0
2015418104	906	rural/clinic	No	24/01/2017	0	0	0
2015418107	906	rural/clinic	No	24/01/2017	0	0	0
2015418109	906	rural/clinic	No	24/01/2017	0	0	0
2014368745	906	rural/clinic	No	24/01/2017	0	0	0
2015418108	906	rural/clinic	No	24/01/2017	0	0	0
2015418944	906	rural/clinic	No	24/01/2017	0	0	0
2015418945	906	rural/clinic	No	24/01/2017	0	0	0
2015418946	906	rural/clinic	No	24/01/2017	0	0	0
2015288740	906	rural/clinic	No	24/01/2017	0	0	0
2015288739	906	rural/clinic	No	24/01/2017	0	0	0
2015301085	476	rural/clinic	No	24/01/2017	0	0	0
2015301084	476	rural/clinic	No	24/01/2017	0	0	0
2015301083	476	rural/clinic	No	24/01/2017	0	0	0
2015412253	476	rural/clinic	No	24/01/2017	0	0	0
2015297353	476	rural/clinic	No	24/01/2017	0	0	0
2015297355	476	rural/clinic	No	24/01/2017	0	0	0
2015377854	907	rural/clinic	No	24/01/2017	0	0	0
2014328292	907	rural/clinic	No	24/01/2017	0	0	0
2015412995	907	rural/clinic	No	24/01/2017	0	0	0
2015412994	907	rural/clinic	No	24/01/2017	0	0	0
2015412992	907	rural/clinic	No	24/01/2017	0	0	0
2015412993	907	rural/clinic	No	24/01/2017	0	0	0
2015412991	907	rural/clinic	No	24/01/2017	0	0	0
2015412990	907	rural/clinic	No	24/01/2017	0	0	0
2015383575	137	rural/clinic	No	24/01/2017	0	0	0
2014368836	137	rural/clinic	No	24/01/2017	0	0	0
2015383576	137	rural/clinic	No	24/01/2017	0	0	0
2015412108	137	rural/clinic	No	24/01/2017	0	0	0
2015383574	137	rural/clinic	No	24/01/2017	0	0	0
2014328393	379	rural/clinic	No	24/01/2017	0	0	0
2015412252	379	rural/clinic	No	24/01/2017	0	0	0
2014383032	379	rural/clinic	No	24/01/2017	0	0	0
2015412110	379	rural/clinic	No	24/01/2017	0	0	0
2015412107	379	rural/clinic	No	24/01/2017	0	0	0
2015412109	379	rural/clinic	No	24/01/2017	0	0	0
2015301081	379	rural/clinic	No	24/01/2017	0	0	0
2012363277	379	rural/clinic	No	24/01/2017	0	0	0
2015301082	379	rural/clinic	No	24/01/2017	0	0	0
2012363284	379	rural/clinic	No	24/01/2017	0	0	0
2012363279	379	rural/clinic	No	24/01/2017	0	0	0
2014358674	379	rural/clinic	No	24/01/2017	0	0	0
2014358676	379	rural/clinic	No	24/01/2017	0	0	0
2014358675	379	rural/clinic	No	24/01/2017	0	0	0
2015401533	379	rural/clinic	No	24/01/2017	0	0	0
2015401531	379	rural/clinic	No	24/01/2017	0	0	0
2014311436	379	rural/clinic	No	24/01/2017	0	0	0
2014311435	379	rural/clinic	No	24/01/2017	0	0	0
2012361049	379	rural/clinic	No	24/01/2017	0	0	0
2015373209	379	rural/clinic	No	24/01/2017	0	0	0
2015373208	379	rural/clinic	No	24/01/2017	0	0	0
2015373207	379	rural/clinic	No	24/01/2017	0	0	0
2015373203	379	rural/clinic	No	24/01/2017	0	0	0
2015413628	379	rural/clinic	No	24/01/2017	0	0	0
2015413627	379	rural/clinic	No	24/01/2017	0	0	0
2015359393	379	rural/clinic	No	24/01/2017	0	0	0
2014342784	379	rural/clinic	No	24/01/2017	0	0	0
2015359394	379	rural/clinic	No	24/01/2017	0	0	0
2015359376	379	rural/clinic	No	24/01/2017	0	0	0
2012262053	379	rural/clinic	No	24/01/2017	0	0	0
2012253916	379	rural/clinic	No	24/01/2017	0	0	0
2015359395	379	rural/clinic	No	24/01/2017	0	0	0
2015359392	379	rural/clinic	No	24/01/2017	0	0	0
2012253915	379	rural/clinic	No	24/01/2017	0	0	0
2014363749	379	rural/clinic	No	24/01/2017	0	0	0
2014363748	379	rural/clinic	No	24/01/2017	0	0	0
2012245719	379	rural/clinic	No	24/01/2017	0	0	0
2012245720	379	rural/clinic	No	24/01/2017	0	0	0
2012245717	379	rural/clinic	No	24/01/2017	0	0	0
2012245718	379	rural/clinic	No	24/01/2017	0	0	0

2014310203	379	rural/clinic	No	24/01/2017	0	0	0
2015331874	350	rural/clinic	No	24/01/2017	0	0	0
2015289825	350	rural/clinic	No	24/01/2017	0	0	0
2014287895	350	rural/clinic	No	24/01/2017	0	0	0
2012284737	350	rural/clinic	No	24/01/2017	0	0	0
2015359202	350	rural/clinic	No	24/01/2017	0	0	0
2012284618	350	rural/clinic	No	24/01/2017	0	0	0
2011128276	350	rural/clinic	No	24/01/2017	0	0	0
2015300657	350	rural/clinic	No	24/01/2017	0	0	0
2014334673	350	rural/clinic	No	24/01/2017	0	0	0
2012275981	350	rural/clinic	No	24/01/2017	0	0	0
2015302489	350	rural/clinic	No	24/01/2017	0	0	0
2015302487	350	rural/clinic	No	24/01/2017	0	0	0
2015357359	350	rural/clinic	No	24/01/2017	0	0	0
2014349913	350	rural/clinic	No	24/01/2017	0	0	0
2015357360	350	rural/clinic	No	24/01/2017	0	0	0
2012306135	350	rural/clinic	No	24/01/2017	0	0	0
2015302488	350	rural/clinic	No	24/01/2017	0	0	0
2015357521	350	rural/clinic	No	24/01/2017	0	0	0
2015339226	350	rural/clinic	No	24/01/2017	0	0	0
2015339228	75	rural/clinic	No	24/01/2017	0	0	0
2015339232	75	rural/clinic	No	24/01/2017	0	0	0
2015339229	75	rural/clinic	No	24/01/2017	0	0	0
2015339230	75	rural/clinic	No	24/01/2017	0	0	0
2015339231	75	rural/clinic	No	24/01/2017	0	0	0
2015339233	75	rural/clinic	No	24/01/2017	0	0	0
2015339224	75	rural/clinic	No	24/01/2017	0	0	0
2015339225	75	rural/clinic	No	24/01/2017	0	0	0
2015413622	75	rural/clinic	No	24/01/2017	0	0	0
2015413625	75	rural/clinic	No	24/01/2017	0	0	0
2015414921	75	rural/clinic	No	24/01/2017	0	0	0
2014298232	75	rural/clinic	No	24/01/2017	0	0	0
2014298233	75	rural/clinic	No	24/01/2017	0	0	0
2013266235	75	rural/clinic	No	24/01/2017	0	0	0
2013266236	75	rural/clinic	No	24/01/2017	0	0	0
2013266237/D	75	rural/clinic	No	24/01/2017	0	0	0
2015362665	75	rural/clinic	No	24/01/2017	0	0	0
2015362664	75	rural/clinic	No	24/01/2017	0	0	0
2014302190	75	rural/clinic	No	24/01/2017	0	0	0
2014302188	75	rural/clinic	No	24/01/2017	0	0	0
2012325680	75	rural/clinic	No	24/01/2017	0	0	0
2014302191	75	rural/clinic	No	24/01/2017	0	0	0
2014302189	75	rural/clinic	No	24/01/2017	0	0	0
2015320851	75	rural/clinic	No	24/01/2017	0	0	0
2015320852	75	rural/clinic	No	24/01/2017	0	0	0
2014369030	75	rural/clinic	No	24/01/2017	0	0	0
2014329135	75	rural/clinic	No	24/01/2017	0	0	0
2015320854	75	rural/clinic	No	24/01/2017	0	0	0
2015320853	75	rural/clinic	No	24/01/2017	0	0	0
2015320855	75	rural/clinic	No	24/01/2017	0	0	0
2013266108	75	rural/clinic	No	24/01/2017	0	0	0
2015373067	75	rural/clinic	No	12/01/2017	0	0	0
2015373067	75	rural/clinic	No	12/01/2017	0	0	0
2015340463	75	rural/clinic	No	12/01/2017	0	0	0
2015340463	75	rural/clinic	No	12/01/2017	0	0	0
2015373327	75	rural/clinic	No	12/01/2017	0	0	0
2015373327	75	rural/clinic	No	12/01/2017	0	0	0
2014326616	75	rural/clinic	No	12/01/2017	0	0	0
2014326616	75	rural/clinic	No	12/01/2017	0	0	0
2015376201	75	rural/clinic	No	24/01/2017	0	0	0
2015344832	75	rural/clinic	No	24/01/2017	0	0	0
2014302840	75	rural/clinic	No	12/01/2017	0	0	0
2014302840	75	rural/clinic	No	12/01/2017	0	0	0
2011225644	908	rural/clinic	No	12/01/2017	0	0	0
2011225644	908	rural/clinic	No	12/01/2017	0	0	0
2012353594	908	rural/clinic	No	24/01/2017	0	0	0
2015397471	908	rural/clinic	No	09/01/2017	0	0	0
2015397471	908	rural/clinic	No	09/01/2017	0	0	0
2012353599	908	rural/clinic	No	24/01/2017	0	0	0
2012353598	908	rural/clinic	No	24/01/2017	0	0	0
2012353600	908	rural/clinic	No	24/01/2017	0	0	0
2012353595	908	rural/clinic	No	24/01/2017	0	0	0
20111282	908	rural/clinic	No	24/01/2017	0	0	0
2015369369	908	rural/clinic	No	26/01/2017	0	0	0
2015369368	908	rural/clinic	No	26/01/2017	0	0	0
2015369370	908	rural/clinic	No	26/01/2017	0	0	0
2015373213	908	rural/clinic	No	26/01/2017	0	0	0
2015373212	908	rural/clinic	No	26/01/2017	0	0	0
2015373214	908	rural/clinic	No	26/01/2017	0	0	0
2015373215	908	rural/clinic	No	26/01/2017	0	0	0
2015373218	909	rural/clinic	No	26/01/2017	0	0	0
2015373210	909	rural/clinic	No	26/01/2017	0	0	0

2015340466	909	rural/clinic	No	26/01/2017	0	0	0
2011184289	909	rural/clinic	No	26/01/2017	0	0	0
2015340467	909	rural/clinic	No	26/01/2017	0	0	0
2015340468	909	rural/clinic	No	26/01/2017	0	0	0
2015340469	909	rural/clinic	No	26/01/2017	0	0	0
2015340471	909	rural/clinic	No	26/01/2017	0	0	0
2015340465	909	rural/clinic	No	26/01/2017	0	0	0
2012386050	910	rural/clinic	No	26/01/2017	0	0	0
2015384026	910	rural/clinic	No	26/01/2017	0	0	0
2015363701	910	rural/clinic	No	26/01/2017	0	0	0
2014365152	910	rural/clinic	No	26/01/2017	0	0	0
2015363702	910	rural/clinic	No	26/01/2017	0	0	0
2012361050	910	rural/clinic	No	26/01/2017	0	0	0
2012290388	910	rural/clinic	No	26/01/2017	0	0	0
2015303110	910	rural/clinic	No	26/01/2017	0	0	0
2015303111	910	rural/clinic	No	26/01/2017	0	0	0
2015303054	910	rural/clinic	No	26/01/2017	0	0	0
2015405610	910	rural/clinic	No	26/01/2017	0	0	0
2014336894	910	rural/clinic	No	26/01/2017	0	0	0
2014326462	910	rural/clinic	No	26/01/2017	0	0	0
2014326463	910	rural/clinic	No	26/01/2017	0	0	0
2014357510	910	rural/clinic	No	26/01/2017	0	0	0
2011157138	910	rural/clinic	No	26/01/2017	0	0	0
2011157139	910	rural/clinic	No	26/01/2017	0	0	0
2015384355	910	rural/clinic	No	26/01/2017	0	0	0
2015392527	910	rural/clinic	No	23/01/2017	0	0	0
2015377419	910	rural/clinic	No	26/01/2017	0	0	0
2015385335	910	rural/clinic	No	24/01/2017	0	0	0
2015418947	910	rural/clinic	No	24/01/2017	0	0	0
2015377420	910	rural/clinic	No	26/01/2017	0	0	0
2015405908	910	rural/clinic	No	26/01/2017	0	0	0
2012289595	910	rural/clinic	No	26/01/2017	0	0	0
2014346890	910	rural/clinic	No	26/01/2017	0	0	0
2015348113	910	rural/clinic	No	26/01/2017	0	0	0
2015377421	910	rural/clinic	No	26/01/2017	0	0	0
2015303576	910	rural/clinic	No	26/01/2017	0	0	0
2015384025	910	rural/clinic	No	26/01/2017	0	0	0
2015377359	910	rural/clinic	No	26/01/2017	0	0	0
2015332701	910	rural/clinic	No	26/01/2017	0	0	0
2015360085	910	rural/clinic	No	26/01/2017	0	0	0
2015377009	910	rural/clinic	No	25/01/2017	0	0	0
2011293826	910	rural/clinic	No	26/01/2017	0	0	0
2012353596	910	rural/clinic	No	24/01/2017	0	0	0
2014338241	910	rural/clinic	No	26/01/2017	0	0	0
2012353597	910	rural/clinic	No	24/01/2017	0	0	0
2012304315	910	rural/clinic	No	24/01/2017	0	0	0
2014338240	910	rural/clinic	No	26/01/2017	0	0	0
2012304318	910	rural/clinic	No	24/01/2017	0	0	0
2012304316	910	rural/clinic	No	24/01/2017	0	0	0
2014338239	910	rural/clinic	No	26/01/2017	0	0	0
2015414642	910	rural/clinic	No	24/01/2017	0	0	0
2014338242	910	rural/clinic	No	26/01/2017	0	0	0
2015362327	910	rural/clinic	No	24/01/2017	0	0	0
2014329231	910	rural/clinic	No	24/01/2017	0	0	0
2012384777	910	rural/clinic	No	26/01/2017	0	0	0
2015362328	910	rural/clinic	No	24/01/2017	0	0	0
2014358175	415	rural/clinic	No	24/01/2017	0	0	0
2012384776	415	rural/clinic	No	26/01/2017	0	0	0
2015414784	415	rural/clinic	No	24/01/2017	0	0	0
2014339399	415	rural/clinic	No	24/01/2017	0	0	0
2013264350	415	rural/clinic	No	26/01/2017	0	0	0
2014339398	415	rural/clinic	No	24/01/2017	0	0	0
2013262201	415	rural/clinic	No	26/01/2017	0	0	0
2013267194	415	rural/clinic	No	24/01/2017	0	0	0
2015362662	415	rural/clinic	No	24/01/2017	0	0	0
2013264347	415	rural/clinic	No	26/01/2017	0	0	0
2015362663	415	rural/clinic	No	24/01/2017	0	0	0
2014383033	415	rural/clinic	No	24/01/2017	0	0	0
2013264348	415	rural/clinic	No	26/01/2017	0	0	0
2014383030	415	rural/clinic	No	24/01/2017	0	0	0
2012304314	415	rural/clinic	No	24/01/2017	0	0	0
2013264349	415	rural/clinic	No	26/01/2017	0	0	0
2012304313	415	rural/clinic	No	24/01/2017	0	0	0
2015331710	415	rural/clinic	No	26/01/2017	0	0	0
2013262203	415	rural/clinic	No	26/01/2017	0	0	0
2015315496	415	rural/clinic	No	24/01/2017	0	0	0
2013262204	415	rural/clinic	No	26/01/2017	0	0	0
2015315497	415	rural/clinic	No	24/01/2017	0	0	0
2015315470	415	rural/clinic	No	24/01/2017	0	0	0
2015315499	415	rural/clinic	No	24/01/2017	0	0	0
2015315500	415	rural/clinic	No	24/01/2017	0	0	0
2015315494	415	rural/clinic	No	24/01/2017	0	0	0

2015385922	415	rural/clinic	No	24/01/2017	0	0	0
2014340261/D	415	rural/clinic	No	26/01/2017	0	0	0
2015385379	415	rural/clinic	No	24/01/2017	0	0	0
2013252785	415	rural/clinic	No	26/01/2017	0	0	0
2015315498	415	rural/clinic	No	24/01/2017	0	0	0
2015385339	415	rural/clinic	No	24/01/2017	0	0	0
2013262202	415	rural/clinic	No	26/01/2017	0	0	0
2015385336	415	rural/clinic	No	24/01/2017	0	0	0
2015315974	415	rural/clinic	No	24/01/2017	0	0	0
2015413540	415	rural/clinic	No	26/01/2017	0	0	0
2015385340	415	rural/clinic	No	24/01/2017	0	0	0
2015413538	415	rural/clinic	No	26/01/2017	0	0	0
2015324029	415	rural/clinic	No	24/01/2017	0	0	0
201066572	415	rural/clinic	No	24/01/2017	0	0	0
2015413541	415	rural/clinic	No	26/01/2017	0	0	0
2015385380	415	rural/clinic	No	24/01/2017	0	0	0
2015413539	415	rural/clinic	No	26/01/2017	0	0	0
2014355841	415	rural/clinic	No	24/01/2017	0	0	0
2015293826	415	rural/clinic	No	26/01/2017	0	0	0
2014355844	415	rural/clinic	No	24/01/2017	0	0	0
2015293825	415	rural/clinic	No	26/01/2017	0	0	0
2014291538	415	rural/clinic	No	24/01/2017	0	0	0
2015315471	415	rural/clinic	No	24/01/2017	0	0	0
2015293824	415	rural/clinic	No	26/01/2017	0	0	0
2015324423	415	rural/clinic	No	24/01/2017	0	0	0
2014291539	415	rural/clinic	No	24/01/2017	0	0	0
2015344641	415	rural/clinic	No	26/01/2017	0	0	0
2015385338	415	rural/clinic	No	24/01/2017	0	0	0
2012333747	415	rural/clinic	No	26/01/2017	0	0	0
2012358276	415	rural/clinic	No	24/01/2017	0	0	0
2015337608	415	rural/clinic	No	24/01/2017	0	0	0
2012333748	415	rural/clinic	No	26/01/2017	0	0	0
2015385337	415	rural/clinic	No	24/01/2017	0	0	0
2014372219	415	rural/clinic	No	26/01/2017	0	0	0
2015324030	415	rural/clinic	No	24/01/2017	0	0	0
2015385381	415	rural/clinic	No	24/01/2017	0	0	0
2014291536	415	rural/clinic	No	24/01/2017	0	0	0
2014372220	415	rural/clinic	No	26/01/2017	0	0	0
2015384769	415	rural/clinic	No	24/01/2017	0	0	0
2014372221	415	rural/clinic	No	26/01/2017	0	0	0
2014372212	415	rural/clinic	No	26/01/2017	0	0	0
2014309582	415	rural/clinic	No	24/01/2017	0	0	0
2014372213	415	rural/clinic	No	26/01/2017	0	0	0
2014291542	415	rural/clinic	No	24/01/2017	0	0	0
2015384771	415	rural/clinic	No	24/01/2017	0	0	0
2014372214	415	rural/clinic	No	26/01/2017	0	0	0
2014309583	415	rural/clinic	No	24/01/2017	0	0	0
2015385926	415	rural/clinic	No	24/01/2017	0	0	0
2014372216	415	rural/clinic	No	26/01/2017	0	0	0
2015384772	415	rural/clinic	No	24/01/2017	0	0	0
2014372217	415	rural/clinic	No	26/01/2017	0	0	0
2015337007	415	rural/clinic	No	24/01/2017	0	0	0
2014372215	415	rural/clinic	No	26/01/2017	0	0	0
2015337006	415	rural/clinic	No	24/01/2017	0	0	0
2014355896	415	rural/clinic	No	24/01/2017	0	0	0
2015413664	415	rural/clinic	No	26/01/2017	0	0	0
2015337005	415	rural/clinic	No	24/01/2017	0	0	0
2015413665	415	rural/clinic	No	26/01/2017	0	0	0
2012358275	415	rural/clinic	No	24/01/2017	0	0	0
2015413671	415	rural/clinic	No	26/01/2017	0	0	0
2012388371	415	rural/clinic	No	24/01/2017	0	0	0
2014311643	415	rural/clinic	No	24/01/2017	0	0	0
2015413672	415	rural/clinic	No	26/01/2017	0	0	0
2015413666	415	rural/clinic	No	26/01/2017	0	0	0
2015413667	415	rural/clinic	No	26/01/2017	0	0	0
2014311644	415	rural/clinic	No	24/01/2017	0	0	0
2014311645	415	rural/clinic	No	24/01/2017	0	0	0
2015413668	415	rural/clinic	No	26/01/2017	0	0	0
2014311641	415	rural/clinic	No	24/01/2017	0	0	0
2014291533	415	rural/clinic	No	24/01/2017	0	0	0
2015413669	415	rural/clinic	No	26/01/2017	0	0	0
2015315493	415	rural/clinic	No	24/01/2017	0	0	0
2015413670	415	rural/clinic	No	26/01/2017	0	0	0
2015315492	415	rural/clinic	No	24/01/2017	0	0	0
2015384770	415	rural/clinic	No	24/01/2017	0	0	0
2015294043	415	rural/clinic	No	26/01/2017	0	0	0
2015315973	651	rural/clinic	No	24/01/2017	0	0	0
2015337001	651	rural/clinic	No	24/01/2017	0	0	0
2015294042	651	rural/clinic	No	26/01/2017	0	0	0
2014291541	651	rural/clinic	No	24/01/2017	0	0	0
2012322038	651	rural/clinic	No	26/01/2017	0	0	0
2015344062	651	rural/clinic	No	26/01/2017	0	0	0

2015344063	651	rural/clinic	No	26/01/2017	0	0	0
2015385341	651	rural/clinic	No	24/01/2017	0	0	0
2015413577	651	rural/clinic	No	26/01/2017	0	0	0
2015385428	651	rural/clinic	No	24/01/2017	0	0	0
2015385427	651	rural/clinic	No	24/01/2017	0	0	0
2015319425	651	rural/clinic	No	26/01/2017	0	0	0
2015337004	651	rural/clinic	No	24/01/2017	0	0	0
2015337003	651	rural/clinic	No	24/01/2017	0	0	0
2012320384	651	rural/clinic	No	26/01/2017	0	0	0
2015337002	651	rural/clinic	No	24/01/2017	0	0	0
2012290671	651	rural/clinic	No	12/01/2017	0	0	0
2012290671	651	rural/clinic	No	12/01/2017	0	0	0
2011232660	651	rural/clinic	No	12/01/2017	0	0	0
2011232660	651	rural/clinic	No	12/01/2017	0	0	0
2015385925	651	rural/clinic	No	24/01/2017	0	0	0
2014355843	651	rural/clinic	No	24/01/2017	0	0	0
2015335409	651	rural/clinic	No	17/01/2017	0	0	0
2015335409	651	rural/clinic	No	17/01/2017	0	0	0
2015377539	651	rural/clinic	No	17/01/2017	0	0	0
2015377539	651	rural/clinic	No	17/01/2017	0	0	0
2015360709	651	rural/clinic	No	17/01/2017	0	0	0
2015360709	651	rural/clinic	No	17/01/2017	0	0	0
2014358316	651	rural/clinic	No	12/01/2017	0	0	0
2014358316	542	rural/clinic	No	12/01/2017	0	0	0
2014287172	542	rural/clinic	No	12/01/2017	0	0	0
2014287172	542	rural/clinic	No	12/01/2017	0	0	0
2014358318	542	rural/clinic	No	12/01/2017	0	0	0
2014358318	542	rural/clinic	No	12/01/2017	0	0	0
2015352413	542	rural/clinic	No	12/01/2017	0	0	0
2015352413	542	rural/clinic	No	12/01/2017	0	0	0
2015342273	542	rural/clinic	No	13/01/2017	0	0	0
2015342273	542	rural/clinic	No	13/01/2017	0	0	0
2015413889	542	rural/clinic	No	26/01/2017	0	0	0
2015413890	542	rural/clinic	No	26/01/2017	0	0	0
2015344721	542	rural/clinic	No	26/01/2017	0	0	0
2015344722	542	rural/clinic	No	26/01/2017	0	0	0
2015315495	542	rural/clinic	No	24/01/2017	0	0	0
2015337609	542	rural/clinic	No	24/01/2017	0	0	0
2015303053	542	rural/clinic	No	26/01/2017	0	0	0
2015385429	542	rural/clinic	No	24/01/2017	0	0	0
2015340470	542	rural/clinic	No	26/01/2017	0	0	0
2015337008	542	rural/clinic	No	24/01/2017	0	0	0
2015385924	542	rural/clinic	No	24/01/2017	0	0	0
2014302389	542	rural/clinic	No	24/01/2017	0	0	0
2011156597	542	rural/clinic	No	24/01/2017	0	0	0
2015351126	542	rural/clinic	No	25/01/2017	0	0	0
2015351127	542	rural/clinic	No	25/01/2017	0	0	0
2015351128	542	rural/clinic	No	25/01/2017	0	0	0
2015384522	609	rural/clinic	No	26/01/2017	0	0	0
2011227008	609	rural/clinic	No	26/01/2017	0	0	0
2011227006	609	rural/clinic	No	26/01/2017	0	0	0
2012289596	609	rural/clinic	No	26/01/2017	0	0	0
2011294468	609	rural/clinic	No	26/01/2017	0	0	0
2015344718	609	rural/clinic	No	26/01/2017	0	0	0
2015344719	609	rural/clinic	No	26/01/2017	0	0	0
201544720	609	rural/clinic	No	26/01/2017	0	0	0
2015293630	652	rural/clinic	No	26/01/2017	0	0	0
2015314789	652	rural/clinic	No	26/01/2017	0	0	0
2015314790	652	rural/clinic	No	26/01/2017	0	0	0
2015314791	652	rural/clinic	No	26/01/2017	0	0	0
2015314792	652	rural/clinic	No	26/01/2017	0	0	0
2014360321	652	rural/clinic	No	26/01/2017	0	0	0
2014360322	652	rural/clinic	No	26/01/2017	0	0	0
2014360323	652	rural/clinic	No	26/01/2017	0	0	0
2014360325	652	rural/clinic	No	26/01/2017	0	0	0
2011232671	652	rural/clinic	No	26/01/2017	0	0	0
2014360328	652	rural/clinic	No	26/01/2017	0	0	0
2011232672	652	rural/clinic	No	26/01/2017	0	0	0
2011232669	652	rural/clinic	No	26/01/2017	0	0	0
2014373093	652	rural/clinic	No	26/01/2017	0	0	0
2014328758	652	rural/clinic	No	26/01/2017	0	0	0
2014360327	652	rural/clinic	No	26/01/2017	0	0	0
2014351218	652	rural/clinic	No	26/01/2017	0	0	0
2014360329	652	rural/clinic	No	26/01/2017	0	0	0
2013266996	652	rural/clinic	No	26/01/2017	0	0	0
2014360330	652	rural/clinic	No	26/01/2017	0	0	0
2013266995	652	rural/clinic	No	26/01/2017	0	0	0
2015358470	652	rural/clinic	No	26/01/2017	0	0	0
2014360332	652	rural/clinic	No	26/01/2017	0	0	0
2014360331	652	rural/clinic	No	26/01/2017	0	0	0
2015358469	652	rural/clinic	No	26/01/2017	0	0	0
2015358468	652	rural/clinic	No	26/01/2017	0	0	0

2015358467	652	rural/clinic	No	26/01/2017	0	0	0
2015368134	652	rural/clinic	No	26/01/2017	0	0	0
2015358466	652	rural/clinic	No	26/01/2017	0	0	0
2015368135	652	rural/clinic	No	26/01/2017	0	0	0
2015358473	652	rural/clinic	No	26/01/2017	0	0	0
2015368263	652	rural/clinic	No	26/01/2017	0	0	0
2015358474	652	rural/clinic	No	26/01/2017	0	0	0
2015368137	652	rural/clinic	No	26/01/2017	0	0	0
2015358472	652	rural/clinic	No	26/01/2017	0	0	0
2015368136	652	rural/clinic	No	26/01/2017	0	0	0
2015377008	652	rural/clinic	No	26/01/2017	0	0	0
2015368133	652	rural/clinic	No	26/01/2017	0	0	0
2015377007	652	rural/clinic	No	26/01/2017	0	0	0
2015303570	652	rural/clinic	No	26/01/2017	0	0	0
2015303571	652	rural/clinic	No	26/01/2017	0	0	0
2015303572	652	rural/clinic	No	26/01/2017	0	0	0
2015303573	652	rural/clinic	No	26/01/2017	0	0	0
2015303574	652	rural/clinic	No	26/01/2017	0	0	0
2014347003	652	rural/clinic	No	26/01/2017	0	0	0
2015303575	652	rural/clinic	No	26/01/2017	0	0	0
2014347002	652	rural/clinic	No	26/01/2017	0	0	0
2015303577	652	rural/clinic	No	26/01/2017	0	0	0
2011168517	652	rural/clinic	No	26/01/2017	0	0	0
2015303578	652	rural/clinic	No	26/01/2017	0	0	0
2011227007	652	rural/clinic	No	26/01/2017	0	0	0
2015303579	652	rural/clinic	No	26/01/2017	0	0	0
2012390993	652	rural/clinic	No	26/01/2017	0	0	0
2015303581	652	rural/clinic	No	26/01/2017	0	0	0
2015303582	652	rural/clinic	No	26/01/2017	0	0	0
2015303583	652	rural/clinic	No	26/01/2017	0	0	0
2015384523	652	rural/clinic	No	26/01/2017	0	0	0
2011227275	652	rural/clinic	No	26/01/2017	0	0	0
2015303584	652	rural/clinic	No	26/01/2017	0	0	0
2011168516	652	rural/clinic	No	26/01/2017	0	0	0
2015352986	652	rural/clinic	No	26/01/2017	0	0	0
2015303585	652	rural/clinic	No	26/01/2017	0	0	0
2012390992	652	rural/clinic	No	26/01/2017	0	0	0
2015352985	652	rural/clinic	No	28/01/2017	0	0	0
2015303586	652	rural/clinic	No	26/01/2017	0	0	0
2015351893	652	rural/clinic	No	26/01/2017	0	0	0
2015346367	652	rural/clinic	No	26/01/2017	0	0	0
2015303587	652	rural/clinic	No	26/01/2017	0	0	0
2015346366	652	rural/clinic	No	26/01/2017	0	0	0
2015351892	652	rural/clinic	No	26/01/2017	0	0	0
2014385683	652	rural/clinic	No	26/01/2017	0	0	0
2015303588	652	rural/clinic	No	26/01/2017	0	0	0
2015351891	652	rural/clinic	No	26/01/2017	0	0	0
2014297342	652	rural/clinic	No	26/01/2017	0	0	0
2015303589	652	rural/clinic	No	26/01/2017	0	0	0
2012390134	652	rural/clinic	No	26/01/2017	0	0	0
2015351889	652	rural/clinic	No	26/01/2017	0	0	0
2015303368	652	rural/clinic	No	26/01/2017	0	0	0
2015351888	652	rural/clinic	No	26/01/2017	0	0	0
2015303590	652	rural/clinic	No	26/01/2017	0	0	0
2014301918	652	rural/clinic	No	26/01/2017	0	0	0
2012390133	652	rural/clinic	No	26/01/2017	0	0	0
2015303591	652	rural/clinic	No	26/01/2017	0	0	0
2015351887	652	rural/clinic	No	26/01/2017	0	0	0
2015289826	652	rural/clinic	No	26/01/2017	0	0	0
2014309698	652	rural/clinic	No	26/01/2017	0	0	0
2015351886	652	rural/clinic	No	26/01/2017	0	0	0
2012253958	652	rural/clinic	No	26/01/2017	0	0	0
2015351885	652	rural/clinic	No	26/01/2017	0	0	0
2012253959	652	rural/clinic	No	26/01/2017	0	0	0
2014309699	652	rural/clinic	No	26/01/2017	0	0	0
2015375842	652	rural/clinic	No	26/01/2017	0	0	0
2014309700	652	rural/clinic	No	26/01/2017	0	0	0
2015351860	652	rural/clinic	No	26/01/2017	0	0	0
2015375843	652	rural/clinic	No	26/01/2017	0	0	0
2015351857	652	rural/clinic	No	26/01/2017	0	0	0
2015375845	652	rural/clinic	No	26/01/2017	0	0	0
2015351861	652	rural/clinic	No	26/01/2017	0	0	0
2015351856	652	rural/clinic	No	26/01/2017	0	0	0
2015352417	652	rural/clinic	No	26/01/2017	0	0	0
2015375847	652	rural/clinic	No	26/01/2017	0	0	0
2015408094	652	rural/clinic	No	26/01/2017	0	0	0
2015351855	652	rural/clinic	No	26/01/2017	0	0	0
2014288165	652	rural/clinic	No	26/01/2017	0	0	0
2015352415	652	rural/clinic	No	26/01/2017	0	0	0
2015375206	652	rural/clinic	No	26/01/2017	0	0	0
2015352416	652	rural/clinic	No	26/01/2017	0	0	0
2015408167	652	rural/clinic	No	26/01/2017	0	0	0

2011113939	652	rural/clinic	No	26/01/2017	0	0	0
2011113940	652	rural/clinic	No	26/01/2017	0	0	0
2014291938	652	rural/clinic	No	26/01/2017	0	0	0
2012313801	652	rural/clinic	No	26/01/2017	0	0	0
2015365196	652	rural/clinic	No	26/01/2017	0	0	0
2014291936	652	rural/clinic	No	26/01/2017	0	0	0
2015359398	652	rural/clinic	No	26/01/2017	0	0	0
2015365197	652	rural/clinic	No	26/01/2017	0	0	0
2015324114	652	rural/clinic	No	26/01/2017	0	0	0
2014310204	652	rural/clinic	No	26/01/2017	0	0	0
2015351869	652	rural/clinic	No	26/01/2017	0	0	0
2015365056	652	rural/clinic	No	26/01/2017	0	0	0
2012253956	652	rural/clinic	No	26/01/2017	0	0	0
2015351859	652	rural/clinic	No	26/01/2017	0	0	0
2015375844	652	rural/clinic	No	26/01/2017	0	0	0
2015365057	652	rural/clinic	No	26/01/2017	0	0	0
2015324121	652	rural/clinic	No	26/01/2017	0	0	0
2015351858	652	rural/clinic	No	26/01/2017	0	0	0
2015368138	652	rural/clinic	No	26/01/2017	0	0	0
2015365058	652	rural/clinic	No	26/01/2017	0	0	0
2015375951	652	rural/clinic	No	26/01/2017	0	0	0
2015335092	652	rural/clinic	No	26/01/2017	0	0	0
2015365060	652	rural/clinic	No	26/01/2017	0	0	0
2015335091	652	rural/clinic	No	26/01/2017	0	0	0
2015335090	652	rural/clinic	No	26/01/2017	0	0	0
2012262055	652	rural/clinic	No	26/01/2017	0	0	0
2015335089	652	rural/clinic	No	26/01/2017	0	0	0
2015365059	652	rural/clinic	No	26/01/2017	0	0	0
2015365064	652	rural/clinic	No	26/01/2017	0	0	0
2015335088	652	rural/clinic	No	26/01/2017	0	0	0
2015358117	652	rural/clinic	No	26/01/2017	0	0	0
2015358118	652	rural/clinic	No	26/01/2017	0	0	0
2015358119	652	rural/clinic	No	26/01/2017	0	0	0
2015375846	652	rural/clinic	No	26/01/2017	0	0	0
2015358120	652	rural/clinic	No	26/01/2017	0	0	0
2015402278	652	rural/clinic	No	26/01/2017	0	0	0
2014372328	652	rural/clinic	No	26/01/2017	0	0	0
2014287896	652	rural/clinic	No	26/01/2017	0	0	0
2015402273	652	rural/clinic	No	26/01/2017	0	0	0
2015365065	652	rural/clinic	No	26/01/2017	0	0	0
2015402277	652	rural/clinic	No	26/01/2017	0	0	0
2014372721	652	rural/clinic	No	26/01/2017	0	0	0
2015365200	652	rural/clinic	No	26/01/2017	0	0	0
2015402274	652	rural/clinic	No	26/01/2017	0	0	0
2012350095	652	rural/clinic	No	26/01/2017	0	0	0
2014372720	652	rural/clinic	No	26/01/2017	0	0	0
2015402276	652	rural/clinic	No	26/01/2017	0	0	0
2015365063	652	rural/clinic	No	26/01/2017	0	0	0
2012350094	652	rural/clinic	No	26/01/2017	0	0	0
2015402279	652	rural/clinic	No	26/01/2017	0	0	0
2012350096	652	rural/clinic	No	26/01/2017	0	0	0
2012366141	652	rural/clinic	No	26/01/2017	0	0	0
2015402280	652	rural/clinic	No	26/01/2017	0	0	0
2012253917	652	rural/clinic	No	26/01/2017	0	0	0
2015402281	442	rural/clinic	No	26/01/2017	0	0	0
2014335296	442	rural/clinic	No	26/01/2017	0	0	0
2015402282	442	rural/clinic	No	26/01/2017	0	0	0
2014298298	442	rural/clinic	No	26/01/2017	0	0	0
2014335295	442	rural/clinic	No	26/01/2017	0	0	0
2015358260	442	rural/clinic	No	26/01/2017	0	0	0
2014298296	442	rural/clinic	No	26/01/2017	0	0	0
2015358261	442	rural/clinic	No	26/01/2017	0	0	0
2012285635	442	rural/clinic	No	26/01/2017	0	0	0
2014335294	442	rural/clinic	No	26/01/2017	0	0	0
2015358262	442	rural/clinic	No	26/01/2017	0	0	0
2014298297	442	rural/clinic	No	26/01/2017	0	0	0
2012285857	442	rural/clinic	No	26/01/2017	0	0	0
2015324120	442	rural/clinic	No	26/01/2017	0	0	0
2015358263	442	rural/clinic	No	26/01/2017	0	0	0
2015375101	442	rural/clinic	No	26/01/2017	0	0	0
2012285856	442	rural/clinic	No	26/01/2017	0	0	0
2015358264	442	rural/clinic	No	26/01/2017	0	0	0
2011221333	442	rural/clinic	No	26/01/2017	0	0	0
2015334062	442	rural/clinic	No	26/01/2017	0	0	0
2015358265	442	rural/clinic	No	26/01/2017	0	0	0
2015375840	442	rural/clinic	No	26/01/2017	0	0	0
2011221334	442	rural/clinic	No	26/01/2017	0	0	0
2015358266	442	rural/clinic	No	26/01/2017	0	0	0
2015334063	442	rural/clinic	No	26/01/2017	0	0	0
2015358268	442	rural/clinic	No	26/01/2017	0	0	0
2015331875	442	rural/clinic	No	26/01/2017	0	0	0
2014371158	442	rural/clinic	No	26/01/2017	0	0	0

2015334071	442	rural/clinic	No	26/01/2017	0	0	0
2015358267	442	rural/clinic	No	26/01/2017	0	0	0
2011133907	442	rural/clinic	No	26/01/2017	0	0	0
2015334068	442	rural/clinic	No	26/01/2017	0	0	0
2015375841	442	rural/clinic	No	26/01/2017	0	0	0
2014386128	442	rural/clinic	No	26/01/2017	0	0	0
2014386129	442	rural/clinic	No	26/01/2017	0	0	0
2015355375	442	rural/clinic	No	26/01/2017	0	0	0
2015334067	442	rural/clinic	No	26/01/2017	0	0	0
2014386130	442	rural/clinic	No	26/01/2017	0	0	0
2015359399	442	rural/clinic	No	26/01/2017	0	0	0
2014386131	442	rural/clinic	No	26/01/2017	0	0	0
2015334072	442	rural/clinic	No	26/01/2017	0	0	0
2015355373	442	rural/clinic	No	26/01/2017	0	0	0
2015359397	442	rural/clinic	No	26/01/2017	0	0	0
2014386132	442	rural/clinic	No	26/01/2017	0	0	0
2015382861	442	rural/clinic	No	26/01/2017	0	0	0
2015306822	442	rural/clinic	No	26/01/2017	0	0	0
2015375953	442	rural/clinic	No	26/01/2017	0	0	0
2015361782	442	rural/clinic	No	26/01/2017	0	0	0
2015359400	442	rural/clinic	No	26/01/2017	0	0	0
2015306826	442	rural/clinic	No	26/01/2017	0	0	0
2015382862	152	rural/clinic	No	26/01/2017	0	0	0
2014365371	152	rural/clinic	No	26/01/2017	0	0	0
2015365199	152	rural/clinic	No	26/01/2017	0	0	0
2014350628	152	rural/clinic	No	26/01/2017	0	0	0
2015382864	152	rural/clinic	No	26/01/2017	0	0	0
2015331876	152	rural/clinic	No	26/01/2017	0	0	0
2014365373	152	rural/clinic	No	26/01/2017	0	0	0
2015382863	152	rural/clinic	No	26/01/2017	0	0	0
2011113941	152	rural/clinic	No	26/01/2017	0	0	0
2015405907	152	rural/clinic	No	26/01/2017	0	0	0
2011224799	152	rural/clinic	No	26/01/2017	0	0	0
2015363001	152	rural/clinic	No	26/01/2017	0	0	0
2011224798	152	rural/clinic	No	26/01/2017	0	0	0
2015375839	152	rural/clinic	No	26/01/2017	0	0	0
2011224800	152	rural/clinic	No	26/01/2017	0	0	0
2015322018	152	rural/clinic	No	26/01/2017	0	0	0
2012330878	152	rural/clinic	No	26/01/2017	0	0	0
2015412071	152	rural/clinic	No	26/01/2017	0	0	0
2014302508	443	district/faith-based	No	26/01/2017	1	0	0
2011223935	443	district/faith-based	No	26/01/2017	1	0	0
2012369494	443	district/faith-based	No	26/01/2017	1	0	0
2011152128	443	district/faith-based	No	26/01/2017	1	0	0
2015412072	443	district/faith-based	No	26/01/2017	1	0	0
2011225144	443	district/faith-based	No	26/01/2017	1	0	0
2014302509	443	district/faith-based	No	26/01/2017	1	0	0
2014338310	443	district/faith-based	No	26/01/2017	1	0	0
2015412070	443	district/faith-based	No	26/01/2017	1	0	0
2012251530	443	district/faith-based	No	26/01/2017	1	0	0
2014302507	443	district/faith-based	No	26/01/2017	1	0	0
2015412073	443	district/faith-based	No	26/01/2017	1	0	0
2015322021	443	district/faith-based	No	26/01/2017	1	0	0
2015368420	443	district/faith-based	No	26/01/2017	1	0	0
2012284398	443	district/faith-based	No	26/01/2017	1	0	0
2015349926	443	district/faith-based	No	26/01/2017	1	0	0
2015368421	443	district/faith-based	No	26/01/2017	1	0	0
2015344640	443	district/faith-based	No	26/01/2017	1	0	0
2012284399	443	district/faith-based	No	26/01/2017	1	0	0
2015340002	443	district/faith-based	No	26/01/2017	1	0	0
2015344634	443	district/faith-based	No	26/01/2017	1	0	0
2015414458	443	district/faith-based	No	26/01/2017	1	0	0
2015344635	443	district/faith-based	No	26/01/2017	1	0	0
2014344286	443	district/faith-based	No	26/01/2017	1	0	0
2015325981	443	district/faith-based	No	26/01/2017	1	0	0
2012284397	443	district/faith-based	No	26/01/2017	1	0	0
2015390943	443	district/faith-based	No	26/01/2017	1	0	0
2012284400	443	district/faith-based	No	26/01/2017	1	0	0
2015325978	443	district/faith-based	No	26/01/2017	1	0	0
2015344638	443	district/faith-based	No	26/01/2017	1	0	0
2015390942	443	district/faith-based	No	26/01/2017	1	0	0
2015360579	443	district/faith-based	No	26/01/2017	1	0	0
2015344639	443	district/faith-based	No	26/01/2017	1	0	0
2015325980	443	district/faith-based	No	26/01/2017	1	0	0
2015360578	443	district/faith-based	No	26/01/2017	1	0	0
2012332914	443	district/faith-based	No	26/01/2017	1	0	0
2015325979	443	district/faith-based	No	26/01/2017	1	0	0
2015360581	443	district/faith-based	No	26/01/2017	1	0	0
2015344624	443	district/faith-based	No	26/01/2017	1	0	0
2012294462	443	district/faith-based	No	26/01/2017	1	0	0
2014381899	443	district/faith-based	No	26/01/2017	1	0	0
2015340001	443	district/faith-based	No	26/01/2017	1	0	0

2014381898	443	district/faith-based	No	26/01/2017	1	0	0
2015344642	443	district/faith-based	No	26/01/2017	1	0	0
2014344288	443	district/faith-based	No	26/01/2017	1	0	0
2014344287	443	district/faith-based	No	26/01/2017	1	0	0
2015305236	443	district/faith-based	No	26/01/2017	1	0	0
2015344637	443	district/faith-based	No	26/01/2017	1	0	0
2015360580	443	district/faith-based	No	26/01/2017	1	0	0
2012304197	443	district/faith-based	No	26/01/2017	1	0	0
2014316755	443	district/faith-based	No	26/01/2017	1	0	0
2015344636	443	district/faith-based	No	26/01/2017	1	0	0
2015305238	443	district/faith-based	No	26/01/2017	1	0	0
2014316754	443	district/faith-based	No	26/01/2017	1	0	0
2015339234	443	district/faith-based	No	26/01/2017	1	0	0
2014316753	443	district/faith-based	No	26/01/2017	1	0	0
2014339397	443	district/faith-based	No	26/01/2017	1	0	0
2015339235	443	district/faith-based	No	26/01/2017	1	0	0
2015305237	443	district/faith-based	No	26/01/2017	1	0	0
2015313393	443	district/faith-based	No	26/01/2017	1	0	0
2011232673	443	district/faith-based	No	26/01/2017	1	0	0
2015313391	443	district/faith-based	No	26/01/2017	1	0	0
2015313389	443	district/faith-based	No	26/01/2017	1	0	0
2015313390	443	district/faith-based	No	26/01/2017	1	0	0
2014313657	443	district/faith-based	No	26/01/2017	1	0	0
2015313392	443	district/faith-based	No	26/01/2017	1	0	0
2015408662	443	district/faith-based	No	26/01/2017	1	0	0
2015408661	443	district/faith-based	No	26/01/2017	1	0	0
2015373333	443	district/faith-based	No	26/01/2017	1	0	0
2015373334	443	district/faith-based	No	26/01/2017	1	0	0
2015373335	443	district/faith-based	No	26/01/2017	1	0	0
2014329234	443	district/faith-based	No	26/01/2017	1	0	0
2015401534	443	district/faith-based	No	26/01/2017	1	0	0
2014329233	443	district/faith-based	No	26/01/2017	1	0	0
2014329232	443	district/faith-based	No	26/01/2017	1	0	0
2013283243	443	district/faith-based	No	26/01/2017	1	0	0
2013283242	443	district/faith-based	No	26/01/2017	1	0	0
2013283241	443	district/faith-based	No	26/01/2017	1	0	0
2015352984	443	district/faith-based	No	26/01/2017	1	0	0
2015352987	443	district/faith-based	No	26/01/2017	1	0	0
2014313658	443	district/faith-based	No	26/01/2017	1	0	0
2012289594	443	district/faith-based	No	26/01/2017	1	0	0
2015378942	443	district/faith-based	No	26/01/2017	1	0	0
2015378944	443	district/faith-based	No	26/01/2017	1	0	0
2015378943	443	district/faith-based	No	26/01/2017	1	0	0
2015286264	443	district/faith-based	No	26/01/2017	1	0	0
2015286263	443	district/faith-based	No	26/01/2017	1	0	0
2015286266	443	district/faith-based	No	26/01/2017	1	0	0
2015286265	443	district/faith-based	No	26/01/2017	1	0	0
2014372437	443	district/faith-based	No	26/01/2017	1	0	0
2014372266	443	district/faith-based	No	26/01/2017	1	0	0
2012265912	443	district/faith-based	No	26/01/2017	1	0	0
2015369476	443	district/faith-based	No	26/01/2017	1	0	0
2015369477	443	district/faith-based	No	26/01/2017	1	0	0
2015369479	443	district/faith-based	No	26/01/2017	1	0	0
2015369478	443	district/faith-based	No	26/01/2017	1	0	0
2015373376	443	district/faith-based	No	26/01/2017	1	0	0
2015373375	443	district/faith-based	No	26/01/2017	1	0	0
2015373374	443	district/faith-based	No	26/01/2017	1	0	0
2015373377	443	district/faith-based	No	26/01/2017	1	0	0
2015358475	443	district/faith-based	No	26/01/2017	1	0	0
2014318399	443	district/faith-based	No	12/01/2017	1	0	0
2014318399	443	district/faith-based	No	12/01/2017	1	0	0
2014318398	443	district/faith-based	No	12/01/2017	1	0	0
2014318398	443	district/faith-based	No	12/01/2017	1	0	0
2015382851	443	district/faith-based	No	12/01/2017	1	0	0
2015382851	443	district/faith-based	No	12/01/2017	1	0	0
2011143192	443	district/faith-based	No	12/01/2017	1	0	0
2011143192	443	district/faith-based	No	12/01/2017	1	0	0
2015291436	443	district/faith-based	No	12/01/2017	1	0	0
2015291436	443	district/faith-based	No	12/01/2017	1	0	0
2015313386	443	district/faith-based	No	17/01/2017	1	0	0
2015313386	443	district/faith-based	No	17/01/2017	1	0	0
2015313387	443	district/faith-based	No	17/01/2017	1	0	0
2015313387	443	district/faith-based	No	17/01/2017	1	0	0
2015414918	443	district/faith-based	No	17/01/2017	1	0	0
2015414918	443	district/faith-based	No	17/01/2017	1	0	0
2015413621	443	district/faith-based	No	17/01/2017	1	0	0
2015413621	443	district/faith-based	No	17/01/2017	1	0	0
2015335645	443	district/faith-based	No	17/01/2017	1	0	0
2015335645	443	district/faith-based	No	17/01/2017	1	0	0
2012340197	443	district/faith-based	No	17/01/2017	1	0	0
2012340197	443	district/faith-based	No	17/01/2017	1	0	0
2014363865	443	district/faith-based	No	17/01/2017	1	0	0

2014363865	443	district/faith-based	No	17/01/2017	1	0	0
2012276141	443	district/faith-based	No	17/01/2017	1	0	0
2012276141	443	district/faith-based	No	17/01/2017	1	0	0
2014322424	443	district/faith-based	No	26/01/2017	1	0	0
2014322422	443	district/faith-based	No	26/01/2017	1	0	0
2012310138	443	district/faith-based	No	26/01/2017	1	0	0
2012310139	443	district/faith-based	No	26/01/2017	1	0	0
2012310140	443	district/faith-based	No	26/01/2017	1	0	0
2014322423	443	district/faith-based	No	26/01/2017	1	0	0
2012310137	443	district/faith-based	No	26/01/2017	1	0	0
2014296873	443	district/faith-based	No	26/01/2017	1	0	0
2014296874	443	district/faith-based	No	26/01/2017	1	0	0
2014296875	443	district/faith-based	No	26/01/2017	1	0	0
2015306825	443	district/faith-based	No	26/01/2017	1	0	0
2015358476	443	district/faith-based	No	26/01/2017	1	0	0
2015286513	443	district/faith-based	No	26/01/2017	1	0	0
2015286515	443	district/faith-based	No	26/01/2017	1	0	0
2015286516	443	district/faith-based	No	26/01/2017	1	0	0
2015286517	443	district/faith-based	No	26/01/2017	1	0	0
2012259755/D	443	district/faith-based	No	26/01/2017	1	0	0
2012370304	443	district/faith-based	No	26/01/2017	1	0	0
2011117485	443	district/faith-based	No	26/01/2017	1	0	0
2013247132	443	district/faith-based	No	26/01/2017	1	0	0
2011117487	443	district/faith-based	No	26/01/2017	1	0	0
2014287016	443	district/faith-based	No	26/01/2017	1	0	0
2014378263	443	district/faith-based	No	26/01/2017	1	0	0
2014378264	443	district/faith-based	No	26/01/2017	1	0	0
2012268779	443	district/faith-based	No	26/01/2017	1	0	0
2015358480	443	district/faith-based	No	26/01/2017	1	0	0
2015358479	443	district/faith-based	No	26/01/2017	1	0	0
2015358478	443	district/faith-based	No	26/01/2017	1	0	0
2015358477	443	district/faith-based	No	26/01/2017	1	0	0
2015358481	443	district/faith-based	No	26/01/2017	1	0	0
2015326504	443	district/faith-based	No	26/01/2017	1	0	0
2015326505	443	district/faith-based	No	26/01/2017	1	0	0
2015326506	443	district/faith-based	No	26/01/2017	1	0	0
2015358455	443	district/faith-based	No	26/01/2017	1	0	0
2015358454	443	district/faith-based	No	26/01/2017	1	0	0
2015358471	443	district/faith-based	No	26/01/2017	1	0	0
2013266997	443	district/faith-based	No	26/01/2017	1	0	0
2013266999	443	district/faith-based	No	26/01/2017	1	0	0
2013267000	443	district/faith-based	No	26/01/2017	1	0	0
2015358451	443	district/faith-based	No	26/01/2017	1	0	0
2015358452	443	district/faith-based	No	26/01/2017	1	0	0
2015358453	443	district/faith-based	No	26/01/2017	1	0	0
2015358463	443	district/faith-based	No	26/01/2017	1	0	0
2015358462	443	district/faith-based	No	26/01/2017	1	0	0
2015358461	757	rural/clinic	No	26/01/2017	0	0	0
2015358460	757	rural/clinic	No	26/01/2017	0	0	0
2015358459	757	rural/clinic	No	26/01/2017	0	0	0
2015358458	757	rural/clinic	No	26/01/2017	0	0	0
2015358457	757	rural/clinic	No	26/01/2017	0	0	0
2015358465	757	rural/clinic	No	26/01/2017	0	0	0
2015358464	757	rural/clinic	No	26/01/2017	0	0	0
2015412068	757	rural/clinic	No	12/01/2017	0	0	0
2015412068	757	rural/clinic	No	12/01/2017	0	0	0
2015343036	757	rural/clinic	No	12/01/2017	0	0	0
2015343036	757	rural/clinic	No	12/01/2017	0	0	0
2015331872	757	rural/clinic	No	12/01/2017	0	0	0
2015331872	757	rural/clinic	No	12/01/2017	0	0	0
2011113937	757	rural/clinic	No	12/01/2017	0	0	0
2011113937	757	rural/clinic	No	12/01/2017	0	0	0
2015386332	757	rural/clinic	No	12/01/2017	0	0	0
2015386332	757	rural/clinic	No	12/01/2017	0	0	0
2015303506	757	rural/clinic	No	31/01/2017	0	0	0
2015413976	757	rural/clinic	No	31/01/2017	0	0	0
2015384475	757	rural/clinic	No	31/01/2017	0	0	0
2012365140	757	rural/clinic	No	31/01/2017	0	0	0
2012350617	757	rural/clinic	No	31/01/2017	0	0	0
2012350618	757	rural/clinic	No	31/01/2017	0	0	0
2015331170	757	rural/clinic	No	31/01/2017	0	0	0
2012305692	757	rural/clinic	No	31/01/2017	0	0	0
2012284619	757	rural/clinic	No	31/01/2017	0	0	0
2013271690	757	rural/clinic	No	31/01/2017	0	0	0
2014287968	757	rural/clinic	No	31/01/2017	0	0	0
2014287967	757	rural/clinic	No	31/01/2017	0	0	0
2012305966	757	rural/clinic	No	31/01/2017	0	0	0
2014287966	757	rural/clinic	No	31/01/2017	0	0	0
2014348064	757	rural/clinic	No	31/01/2017	0	0	0
2011204470	757	rural/clinic	No	31/01/2017	0	0	0
2011204471	757	rural/clinic	No	31/01/2017	0	0	0
2012245721	182	rural/clinic	No	31/01/2017	0	0	0

2012245722	182	rural/clinic	No	31/01/2017	0	0	0
2015289276	182	rural/clinic	No	31/01/2017	0	0	0
2012321328	182	rural/clinic	No	31/01/2017	0	0	0
2012321329	182	rural/clinic	No	31/01/2017	0	0	0
2015335410	182	rural/clinic	No	31/01/2017	0	0	0
2015335413	182	rural/clinic	No	31/01/2017	0	0	0
2015335414	182	rural/clinic	No	31/01/2017	0	0	0
2015335416	182	rural/clinic	No	31/01/2017	0	0	0
2015377544	182	rural/clinic	No	31/01/2017	0	0	0
2014329230	182	rural/clinic	No	31/01/2017	0	0	0
2015377545	182	rural/clinic	No	31/01/2017	0	0	0
2015384801	182	rural/clinic	No	31/01/2017	0	0	0
2011221922	182	rural/clinic	No	31/01/2017	0	0	0
2013256370	182	rural/clinic	No	31/01/2017	0	0	0
2013256378	182	rural/clinic	No	31/01/2017	0	0	0
2013256379	182	rural/clinic	No	31/01/2017	0	0	0
2015289563	182	rural/clinic	No	31/01/2017	0	0	0
2015335411	182	rural/clinic	No	31/01/2017	0	0	0
2015335412	182	rural/clinic	No	31/01/2017	0	0	0
2012290674	182	rural/clinic	No	31/01/2017	0	0	0
2015418630	182	rural/clinic	No	31/01/2017	0	0	0
2015418632	182	rural/clinic	No	31/01/2017	0	0	0
2015418635	182	rural/clinic	No	31/01/2017	0	0	0
2013255254	182	rural/clinic	No	31/01/2017	0	0	0
2014383525	182	rural/clinic	No	31/01/2017	0	0	0
2014358176	182	rural/clinic	No	31/01/2017	0	0	0
2012276144	182	rural/clinic	No	31/01/2017	0	0	0
2012289782	182	rural/clinic	No	31/01/2017	0	0	0
2012276143	182	rural/clinic	No	31/01/2017	0	0	0
2012275982	182	rural/clinic	No	31/01/2017	0	0	0
2015418114	182	rural/clinic	No	31/01/2017	0	0	0
2015418113	182	rural/clinic	No	31/01/2017	0	0	0
2015418110	182	rural/clinic	No	31/01/2017	0	0	0
2015418949	182	rural/clinic	No	31/01/2017	0	0	0
2015418111	182	rural/clinic	No	31/01/2017	0	0	0
2015418112	182	rural/clinic	No	31/01/2017	0	0	0
2015401539	182	rural/clinic	No	31/01/2017	0	0	0
2015401537	182	rural/clinic	No	31/01/2017	0	0	0
2015401540	182	rural/clinic	No	31/01/2017	0	0	0
2015418948	182	rural/clinic	No	31/01/2017	0	0	0
2013266238	182	rural/clinic	No	31/01/2017	0	0	0
2014375598	182	rural/clinic	No	31/01/2017	0	0	0
2015377821	182	rural/clinic	No	31/01/2017	0	0	0
2015414310	182	rural/clinic	No	31/01/2017	0	0	0
2014375596	182	rural/clinic	No	31/01/2017	0	0	0
2014375597	182	rural/clinic	No	31/01/2017	0	0	0
2015375832	182	rural/clinic	No	11/01/2017	0	0	0
2015375833	182	rural/clinic	No	31/01/2017	0	0	0
2012243132	182	rural/clinic	No	12/01/2017	0	0	0
2012293131	182	rural/clinic	No	12/01/2017	0	0	0
2011011592	182	rural/clinic	No	12/01/2017	0	0	0
201700000	182	rural/clinic	No	12/01/2017	0	0	0
2014375599	182	rural/clinic	No	31/01/2017	0	0	0
2015369717	76	rural/clinic	No	31/01/2017	0	0	0
2015369718	76	rural/clinic	No	31/01/2017	0	0	0
2014375600	76	rural/clinic	No	31/01/2017	0	0	0
2013270493	76	rural/clinic	No	31/01/2017	0	0	0
2015340824	76	rural/clinic	No	31/01/2017	0	0	0
2015340825	76	rural/clinic	No	31/01/2017	0	0	0
2012306338	76	rural/clinic	No	31/01/2017	0	0	0
2011236178	76	rural/clinic	No	31/01/2017	0	0	0
2011235742	76	rural/clinic	No	31/01/2017	0	0	0
2011236076	76	rural/clinic	No	31/01/2017	0	0	0
2015301439	76	rural/clinic	No	31/01/2017	0	0	0
2015340827	76	rural/clinic	No	31/01/2017	0	0	0
2015340826	76	rural/clinic	No	31/01/2017	0	0	0
2015301440	76	rural/clinic	No	31/01/2017	0	0	0
20170000	76	rural/clinic	No	12/01/2017	0	0	0
2015414641	76	rural/clinic	No	31/01/2017	0	0	0
2015334502	76	rural/clinic	No	31/01/2017	0	0	0
2015357627	76	rural/clinic	No	31/01/2017	0	0	0
2011212645	76	rural/clinic	No	31/01/2017	0	0	0
2011192834	76	rural/clinic	No	31/01/2017	0	0	0
2015334501	76	rural/clinic	No	31/01/2017	0	0	0
2014382911	76	rural/clinic	No	31/01/2017	0	0	0
2015293918	76	rural/clinic	No	31/01/2017	0	0	0
2013264052	76	rural/clinic	No	31/01/2017	0	0	0
2015383577	76	rural/clinic	No	31/01/2017	0	0	0
2014382910	76	rural/clinic	No	31/01/2017	0	0	0
2015377855	76	rural/clinic	No	31/01/2017	0	0	0
2014356844	76	rural/clinic	No	31/01/2017	0	0	0
2014334674	76	rural/clinic	No	31/01/2017	0	0	0

2015377856	76	rural/clinic	No	31/01/2017	0	0	0
2014345830	76	rural/clinic	No	31/01/2017	0	0	0
2015335415	76	rural/clinic	No	31/01/2017	0	0	0
2014348063	76	rural/clinic	No	31/01/2017	0	0	0
2014328350	76	rural/clinic	No	31/01/2017	0	0	0
2011192833	76	rural/clinic	No	31/01/2017	0	0	0
2015414371	76	rural/clinic	No	31/01/2017	0	0	0
2013284898	76	rural/clinic	No	31/01/2017	0	0	0
2015414370	76	rural/clinic	No	31/01/2017	0	0	0
2013284896	76	rural/clinic	No	31/01/2017	0	0	0
2015413074	76	rural/clinic	No	19/01/2017	0	0	0
2015413074	76	rural/clinic	No	19/01/2017	0	0	0
2014335214	76	rural/clinic	No	19/01/2017	0	0	0
2014335214	138	rural/clinic	No	19/01/2017	0	0	0
2013284895	138	rural/clinic	No	31/01/2017	0	0	0
2012253127	138	rural/clinic	No	17/01/2017	0	0	0
2012253127	138	rural/clinic	No	17/01/2017	0	0	0
2015359824	138	rural/clinic	No	17/01/2017	0	0	0
2015359824	138	rural/clinic	No	17/01/2017	0	0	0
2014371492	138	rural/clinic	No	17/01/2017	0	0	0
2014371492	138	rural/clinic	No	17/01/2017	0	0	0
2012246169	138	rural/clinic	No	17/01/2017	0	0	0
2012246169	138	rural/clinic	No	17/01/2017	0	0	0
2012244646	138	rural/clinic	No	31/01/2017	0	0	0
2015359833	138	rural/clinic	No	31/01/2017	0	0	0
2012284023	138	rural/clinic	No	31/01/2017	0	0	0
2015331758	138	rural/clinic	No	31/01/2017	0	0	0
2015331757	138	rural/clinic	No	31/01/2017	0	0	0
2014361222	138	rural/clinic	No	31/01/2017	0	0	0
201542007	138	rural/clinic	No	31/01/2017	0	0	0
2012244644	351	rural/clinic	No	31/01/2017	0	0	0
2012368532	351	rural/clinic	No	17/01/2017	0	0	0
2012368532	351	rural/clinic	No	17/01/2017	0	0	0
2015413975	351	rural/clinic	No	17/01/2017	0	0	0
2015413975	351	rural/clinic	No	17/01/2017	0	0	0
2012246183	351	rural/clinic	No	31/01/2017	0	0	0
2015342006	351	rural/clinic	No	31/01/2017	0	0	0
2012246182	351	rural/clinic	No	31/01/2017	0	0	0
2015294297	351	rural/clinic	No	31/01/2017	0	0	0
2011170793	351	rural/clinic	No	31/01/2017	0	0	0
2015334402	351	rural/clinic	No	31/01/2017	0	0	0
2011212646	351	rural/clinic	No	31/01/2017	0	0	0
2012322906	351	rural/clinic	No	31/01/2017	0	0	0
2012322902	351	rural/clinic	No	31/01/2017	0	0	0
2015316066	351	rural/clinic	No	31/01/2017	0	0	0
2015316068	351	rural/clinic	No	31/01/2017	0	0	0
2012322915	351	rural/clinic	No	31/01/2017	0	0	0
2012322909	351	rural/clinic	No	31/01/2017	0	0	0
2015358456/D	351	rural/clinic	No	26/01/2017	0	0	0
2011222297	351	rural/clinic	No	31/01/2017	0	0	0
2012322912	351	rural/clinic	No	31/01/2017	0	0	0
2014344700	351	rural/clinic	No	31/01/2017	0	0	0
2015317607	758	rural/clinic	No	31/01/2017	0	0	0
2015293921	758	rural/clinic	No	31/01/2017	0	0	0
2015293920	758	rural/clinic	No	31/01/2017	0	0	0
2011212183	758	rural/clinic	No	31/01/2017	0	0	0
2015293917	758	rural/clinic	No	31/01/2017	0	0	0
2015293922	758	rural/clinic	No	31/01/2017	0	0	0
2015293923	758	rural/clinic	No	31/01/2017	0	0	0
2015334055	758	rural/clinic	No	31/01/2017	0	0	0
2015334064	758	rural/clinic	No	31/01/2017	0	0	0
2015334057	758	rural/clinic	No	31/01/2017	0	0	0
2015334056	758	rural/clinic	No	31/01/2017	0	0	0
2015334061	758	rural/clinic	No	31/01/2017	0	0	0
2015310112	758	rural/clinic	No	31/01/2017	0	0	0
2014309214	758	rural/clinic	No	31/01/2017	0	0	0
2011112301	758	rural/clinic	No	31/01/2017	0	0	0
2015313651	758	rural/clinic	No	31/01/2017	0	0	0
2014321999	758	rural/clinic	No	31/01/2017	0	0	0
2011216149	758	rural/clinic	No	31/01/2017	0	0	0
2015310175	758	rural/clinic	No	31/01/2017	0	0	0
2014309213	758	rural/clinic	No	31/01/2017	0	0	0
2014348566	758	rural/clinic	No	31/01/2017	0	0	0
2014348567	758	rural/clinic	No	31/01/2017	0	0	0
2014348568	758	rural/clinic	No	31/01/2017	0	0	0
2014348569	758	rural/clinic	No	31/01/2017	0	0	0
2014348570	758	rural/clinic	No	31/01/2017	0	0	0
2014358672	758	rural/clinic	No	31/01/2017	0	0	0
2012335657	758	rural/clinic	No	31/01/2017	0	0	0
2012335655	758	rural/clinic	No	31/01/2017	0	0	0
2014358671	259	rural/clinic	No	31/01/2017	0	0	0
2012363285	259	rural/clinic	No	31/01/2017	0	0	0

2012363286	259	rural/clinic	No	31/01/2017	0	0	0
2012352338	259	rural/clinic	No	31/01/2017	0	0	0
2012352341	259	rural/clinic	No	31/01/2017	0	0	0
2012307510	259	rural/clinic	No	31/01/2017	0	0	0
2014329028	259	rural/clinic	No	31/01/2017	0	0	0
2014329027	259	rural/clinic	No	31/01/2017	0	0	0
2012307509	259	rural/clinic	No	31/01/2017	0	0	0
2015320856	259	rural/clinic	No	31/01/2017	0	0	0
2015320857	259	rural/clinic	No	31/01/2017	0	0	0
2015320858	259	rural/clinic	No	31/01/2017	0	0	0
2014358673	259	rural/clinic	No	31/01/2017	0	0	0
2013253934	259	rural/clinic	No	27/01/2017	0	0	0
2014302193	259	rural/clinic	No	31/01/2017	0	0	0
2015368864	259	rural/clinic	No	27/01/2017	0	0	0
2014302194	259	rural/clinic	No	31/01/2017	0	0	0
2014302195	259	rural/clinic	No	31/01/2017	0	0	0
2015331607	259	rural/clinic	No	27/01/2017	0	0	0
2014302192	259	rural/clinic	No	31/01/2017	0	0	0
2015331615	911	rural/clinic	No	27/01/2017	0	0	0
2014369025	911	rural/clinic	No	31/01/2017	0	0	0
2014357502	911	rural/clinic	No	19/01/2017	0	0	0
2014369027	911	rural/clinic	No	31/01/2017	0	0	0
2015331617	911	rural/clinic	No	27/01/2017	0	0	0
2015331612	911	rural/clinic	No	27/01/2017	0	0	0
2012307508	911	rural/clinic	No	31/01/2017	0	0	0
2014369028	911	rural/clinic	No	31/01/2017	0	0	0
2015331606	911	rural/clinic	No	27/01/2017	0	0	0
2012361047	911	rural/clinic	No	11/01/2017	0	0	0
2015360711	911	rural/clinic	No	31/01/2017	0	0	0
2015331609	911	rural/clinic	No	27/01/2017	0	0	0
2014357501	911	rural/clinic	No	11/01/2017	0	0	0
2014357504	911	rural/clinic	No	11/01/2017	0	0	0
2015295338	911	rural/clinic	No	31/01/2017	0	0	0
2015360710	911	rural/clinic	No	31/01/2017	0	0	0
2015331608	911	rural/clinic	No	27/01/2017	0	0	0
2014324100	911	rural/clinic	No	11/01/2017	0	0	0
2015325924	911	rural/clinic	No	31/01/2017	0	0	0
2014357503	911	rural/clinic	No	11/01/2017	0	0	0
2015408659	911	rural/clinic	No	11/01/2017	0	0	0
2015295334	911	rural/clinic	No	02/02/2017	0	0	0
2015408660	911	rural/clinic	No	11/01/2017	0	0	0
2011141543	911	rural/clinic	No	11/01/2017	0	0	0
2015357364	911	rural/clinic	No	31/01/2017	0	0	0
2015357361	911	rural/clinic	No	31/01/2017	0	0	0
2015331619	911	rural/clinic	No	27/01/2017	0	0	0
2015295336	911	rural/clinic	No	31/01/2017	0	0	0
2014320289	911	rural/clinic	No	31/01/2017	0	0	0
2015331613	911	rural/clinic	No	27/01/2017	0	0	0
2014327236	911	rural/clinic	No	31/01/2017	0	0	0
2015357250	911	rural/clinic	No	31/01/2017	0	0	0
2015357363	183	rural/clinic	No	31/01/2017	0	0	0
2015331611	183	rural/clinic	No	27/01/2017	0	0	0
2012328391	183	rural/clinic	No	31/01/2017	0	0	0
2014319209	183	rural/clinic	No	31/01/2017	0	0	0
2015331605	183	rural/clinic	No	27/01/2017	0	0	0
2014296389	183	rural/clinic	No	27/01/2017	0	0	0
2015295340	183	rural/clinic	No	31/01/2017	0	0	0
2015374304	183	rural/clinic	No	27/01/2017	0	0	0
2015334058	183	rural/clinic	No	31/01/2017	0	0	0
2015374305	183	rural/clinic	No	27/01/2017	0	0	0
2015302492	183	rural/clinic	No	31/01/2017	0	0	0
2015334059	183	rural/clinic	No	31/01/2017	0	0	0
2015302493	183	rural/clinic	No	31/01/2017	0	0	0
2015334060	183	rural/clinic	No	31/01/2017	0	0	0
2012328398	183	rural/clinic	No	31/01/2017	0	0	0
2015374306	183	rural/clinic	No	27/01/2017	0	0	0
2015334065	183	rural/clinic	No	31/01/2017	0	0	0
2015302491	183	rural/clinic	No	31/01/2017	0	0	0
2015374307	183	rural/clinic	No	27/01/2017	0	0	0
2015334066	183	rural/clinic	No	31/01/2017	0	0	0
2015362334	139	rural/clinic	No	31/01/2017	0	0	0
2015357362	139	rural/clinic	No	31/01/2017	0	0	0
2015374308	139	rural/clinic	No	27/01/2017	0	0	0
2015374309	139	rural/clinic	No	27/01/2017	0	0	0
2015362333	139	rural/clinic	No	31/01/2017	0	0	0
2012328399	139	rural/clinic	No	31/01/2017	0	0	0
2015362331	139	rural/clinic	No	31/01/2017	0	0	0
2015325923	139	rural/clinic	No	31/01/2017	0	0	0
2015362330	139	rural/clinic	No	31/01/2017	0	0	0
2014327235	139	rural/clinic	No	31/01/2017	0	0	0
2015374311	139	rural/clinic	No	27/01/2017	0	0	0
2015357180	139	rural/clinic	No	31/01/2017	0	0	0

2014320291	139	rural/clinic	No	31/01/2017	0	0	0
2014385614	139	rural/clinic	No	19/01/2017	0	0	0
2015374314	912	rural/clinic	No	27/01/2017	0	0	0
2015302490	912	rural/clinic	No	31/01/2017	0	0	0
2015374315	912	rural/clinic	No	27/01/2017	0	0	0
2014333549	912	rural/clinic	No	19/01/2017	0	0	0
2014333549	912	rural/clinic	No	19/01/2017	0	0	0
20143335550	912	rural/clinic	No	19/01/2017	0	0	0
20143335550	912	rural/clinic	No	19/01/2017	0	0	0
2015305231	912	rural/clinic	No	19/01/2017	0	0	0
2015305231	912	rural/clinic	No	19/01/2017	0	0	0
2015305234	912	rural/clinic	No	19/01/2017	0	0	0
2015305234	912	rural/clinic	No	19/01/2017	0	0	0
2014381897	912	rural/clinic	No	19/01/2017	0	0	0
2014381897	912	rural/clinic	No	19/01/2017	0	0	0
2015405087	912	rural/clinic	No	27/01/2017	0	0	0
2015305225	912	rural/clinic	No	19/01/2017	0	0	0
2015305225	912	rural/clinic	No	19/01/2017	0	0	0
2015369719	912	rural/clinic	No	31/01/2017	0	0	0
2015358114	912	rural/clinic	No	19/01/2017	0	0	0
2015358114	912	rural/clinic	No	19/01/2017	0	0	0
2011203825	912	rural/clinic	No	31/01/2017	0	0	0
2012390132	912	rural/clinic	No	27/01/2017	0	0	0
2012290673	912	rural/clinic	No	19/01/2017	0	0	0
2012290673	912	rural/clinic	No	19/01/2017	0	0	0
2015373645	912	rural/clinic	No	19/01/2017	0	0	0
2015373645	912	rural/clinic	No	19/01/2017	0	0	0
2015369767	912	rural/clinic	No	31/01/2017	0	0	0
2015405088	912	rural/clinic	No	27/01/2017	0	0	0
2015369765	912	rural/clinic	No	31/01/2017	0	0	0
2012390137	912	rural/clinic	No	27/01/2017	0	0	0
2015414922	912	rural/clinic	No	31/01/2017	0	0	0
2012390135	912	rural/clinic	No	27/01/2017	0	0	0
2011203823	912	rural/clinic	No	31/01/2017	0	0	0
2015414923	912	rural/clinic	No	31/01/2017	0	0	0
2015362329	912	rural/clinic	No	31/01/2017	0	0	0
2015414924	912	rural/clinic	No	31/01/2017	0	0	0
2015286858	912	rural/clinic	No	27/01/2017	0	0	0
2015369766	912	rural/clinic	No	31/01/2017	0	0	0
2015368418	912	rural/clinic	No	27/01/2017	0	0	0
2015362332	912	rural/clinic	No	31/01/2017	0	0	0
2012388372	912	rural/clinic	No	31/01/2017	0	0	0
2014357720	912	rural/clinic	No	31/01/2017	0	0	0
2012388373	912	rural/clinic	No	31/01/2017	0	0	0
2014357716	912	rural/clinic	No	31/01/2017	0	0	0
2015368427	912	rural/clinic	No	27/01/2017	0	0	0
2012388374	912	rural/clinic	No	31/01/2017	0	0	0
2015286859	912	rural/clinic	No	27/01/2017	0	0	0
2011203822	912	rural/clinic	No	31/01/2017	0	0	0
2014357718	912	rural/clinic	No	31/01/2017	0	0	0
2015368419	912	rural/clinic	No	27/01/2017	0	0	0
2015385929	912	rural/clinic	No	31/01/2017	0	0	0
2012364933	912	rural/clinic	No	27/01/2017	0	0	0
2015385928	912	rural/clinic	No	31/01/2017	0	0	0
2014357719	912	rural/clinic	No	31/01/2017	0	0	0
2015385927	912	rural/clinic	No	31/01/2017	0	0	0
2015405706	912	rural/clinic	No	27/01/2017	0	0	0
2014345841	912	rural/clinic	No	31/01/2017	0	0	0
2014311646	912	rural/clinic	No	31/01/2017	0	0	0
2012364935	912	rural/clinic	No	27/01/2017	0	0	0
2014345837	912	rural/clinic	No	31/01/2017	0	0	0
2014311534	912	rural/clinic	No	31/01/2017	0	0	0
2014345838	912	rural/clinic	No	31/01/2017	0	0	0
2014291939	912	rural/clinic	No	31/01/2017	0	0	0
2014345839	912	rural/clinic	No	31/01/2017	0	0	0
2012364934	912	rural/clinic	No	27/01/2017	0	0	0
2014291612	912	rural/clinic	No	31/01/2017	0	0	0
2014345840	912	rural/clinic	No	31/01/2017	0	0	0
2014291613	912	rural/clinic	No	31/01/2017	0	0	0
2014345828	912	rural/clinic	No	31/01/2017	0	0	0
2014311533	912	rural/clinic	No	31/01/2017	0	0	0
2014291941	912	rural/clinic	No	31/01/2017	0	0	0
2014345827	912	rural/clinic	No	31/01/2017	0	0	0
2014345829	912	rural/clinic	No	31/01/2017	0	0	0
2012364932	912	rural/clinic	No	27/01/2017	0	0	0
2014311535	912	rural/clinic	No	31/01/2017	0	0	0
2014345833	912	rural/clinic	No	31/01/2017	0	0	0
2015337013	912	rural/clinic	No	31/01/2017	0	0	0
2012379642	912	rural/clinic	No	31/01/2017	0	0	0
2014345834	912	rural/clinic	No	31/01/2017	0	0	0
2015337015	912	rural/clinic	No	31/01/2017	0	0	0
2014345835	912	rural/clinic	No	31/01/2017	0	0	0

2014321417	912	rural/clinic	No	27/01/2017	0	0	0
2015337016	912	rural/clinic	No	31/01/2017	0	0	0
2012349260	912	rural/clinic	No	31/01/2017	0	0	0
2014345836	912	rural/clinic	No	31/01/2017	0	0	0
2014378715	912	rural/clinic	No	31/01/2017	0	0	0
2015337009	912	rural/clinic	No	31/01/2017	0	0	0
2012349261	912	rural/clinic	No	31/01/2017	0	0	0
2015361783	912	rural/clinic	No	31/01/2017	0	0	0
2015361784	912	rural/clinic	No	31/01/2017	0	0	0
2015319570	912	rural/clinic	No	31/01/2017	0	0	0
2015319565	912	rural/clinic	No	31/01/2017	0	0	0
2015319567	912	rural/clinic	No	31/01/2017	0	0	0
2015319566	912	rural/clinic	No	31/01/2017	0	0	0
2015365346	912	rural/clinic	No	31/01/2017	0	0	0
2015365342	912	rural/clinic	No	31/01/2017	0	0	0
2015365349	912	rural/clinic	No	31/01/2017	0	0	0
2015365350	912	rural/clinic	No	31/01/2017	0	0	0
2015319568	912	rural/clinic	No	31/01/2017	0	0	0
2015319569	912	rural/clinic	No	31/01/2017	0	0	0
2015337012	912	rural/clinic	No	31/01/2017	0	0	0
2015337010	912	rural/clinic	No	31/01/2017	0	0	0
2015337011	912	rural/clinic	No	31/01/2017	0	0	0
2015337014	912	rural/clinic	No	31/01/2017	0	0	0
2015385347	912	rural/clinic	No	31/01/2017	0	0	0
2014314701	912	rural/clinic	No	31/01/2017	0	0	0
2015385346	912	rural/clinic	No	31/01/2017	0	0	0
2015385349	912	rural/clinic	No	31/01/2017	0	0	0
2012349262	912	rural/clinic	No	02/02/2017	0	0	0
2015385348	912	rural/clinic	No	31/01/2017	0	0	0
2015326159	912	rural/clinic	No	31/01/2017	0	0	0
2015326158	912	rural/clinic	No	31/01/2017	0	0	0
2012349269	912	rural/clinic	No	31/01/2017	0	0	0
2014321416	912	rural/clinic	No	31/01/2017	0	0	0
2011232663	912	rural/clinic	No	31/01/2017	0	0	0
2014378716	912	rural/clinic	No	31/01/2017	0	0	0
2014321414	912	rural/clinic	No	31/01/2017	0	0	0
2014321415	912	rural/clinic	No	31/01/2017	0	0	0
2014321514	912	rural/clinic	No	31/01/2017	0	0	0
2015346368	912	rural/clinic	No	31/01/2017	0	0	0
2015346369	912	rural/clinic	No	31/01/2017	0	0	0
2015346370	912	rural/clinic	No	31/01/2017	0	0	0
2011232670	912	rural/clinic	No	31/01/2017	0	0	0
2015365341	416	rural/clinic	No	31/01/2017	0	0	0
2015365347	416	rural/clinic	No	31/01/2017	0	0	0
201437813	416	rural/clinic	No	31/01/2017	0	0	0
2015346371	416	rural/clinic	No	31/01/2017	0	0	0
2015365343	416	rural/clinic	No	31/01/2017	0	0	0
2014378714	416	rural/clinic	No	31/01/2017	0	0	0
2015343499	416	rural/clinic	No	31/01/2017	0	0	0
2015343500	416	rural/clinic	No	31/01/2017	0	0	0
2015346220	416	rural/clinic	No	31/01/2017	0	0	0
2015373211	416	rural/clinic	No	31/01/2017	0	0	0
2012377412	416	rural/clinic	No	31/01/2017	0	0	0
2015343498	416	rural/clinic	No	31/01/2017	0	0	0
2012377410	416	rural/clinic	No	31/01/2017	0	0	0
2014315522	416	rural/clinic	No	31/01/2017	0	0	0
2015373216	416	rural/clinic	No	31/01/2017	0	0	0
2015361704	416	rural/clinic	No	31/01/2017	0	0	0
2015365344	416	rural/clinic	No	31/01/2017	0	0	0
2014315521	416	rural/clinic	No	31/01/2017	0	0	0
2015418179	416	rural/clinic	No	31/01/2017	0	0	0
2015373217	789	rural/clinic	No	31/01/2017	0	0	0
2014330345	789	rural/clinic	No	31/01/2017	0	0	0
2015397771	789	rural/clinic	No	31/01/2017	0	0	0
2014321412	789	rural/clinic	No	31/01/2017	0	0	0
2015373220	789	rural/clinic	No	31/01/2017	0	0	0
2014318834	789	rural/clinic	No	31/01/2017	0	0	0
2014318833	789	rural/clinic	No	31/01/2017	0	0	0
2014369139	789	rural/clinic	No	31/01/2017	0	0	0
2013271299	789	rural/clinic	No	31/01/2017	0	0	0
2015418631	789	rural/clinic	No	31/01/2017	0	0	0
2015373221	789	rural/clinic	No	31/01/2017	0	0	0
2015418633	789	rural/clinic	No	31/01/2017	0	0	0
2015418178	789	rural/clinic	No	31/01/2017	0	0	0
2014291940	913	rural/clinic	No	31/01/2017	0	0	0
2015361681	913	rural/clinic	No	31/01/2017	0	0	0
2015418634	913	rural/clinic	No	31/01/2017	0	0	0
2015361680	913	rural/clinic	No	31/01/2017	0	0	0
2011203824	913	rural/clinic	No	31/01/2017	0	0	0
2015291444	913	rural/clinic	No	31/01/2017	0	0	0
2012328396	913	rural/clinic	No	31/01/2017	0	0	0
2015346219	913	rural/clinic	No	31/01/2017	0	0	0

2011203820	913	rural/clinic	No	31/01/2017	0	0	0
2015310909	913	rural/clinic	No	31/01/2017	0	0	0
2011233795	913	rural/clinic	No	31/01/2017	0	0	0
2014369137	913	rural/clinic	No	31/01/2017	0	0	0
2011233796	913	rural/clinic	No	31/01/2017	0	0	0
2011233797	913	rural/clinic	No	31/01/2017	0	0	0
2014321413	913	rural/clinic	No	31/01/2017	0	0	0
2011203821	913	rural/clinic	No	31/01/2017	0	0	0
2012349264	913	rural/clinic	No	31/01/2017	0	0	0
2011233798	913	rural/clinic	No	31/01/2017	0	0	0
2015391860	913	rural/clinic	No	31/01/2017	0	0	0
2015317387	913	rural/clinic	No	31/01/2017	0	0	0
2012328397	913	rural/clinic	No	31/01/2017	0	0	0
2011233799	913	rural/clinic	No	31/01/2017	0	0	0
2011233800	913	rural/clinic	No	31/01/2017	0	0	0
2015317386	913	rural/clinic	No	31/01/2017	0	0	0
2015397110	913	rural/clinic	No	31/01/2017	0	0	0
2015313608	913	rural/clinic	No	31/01/2017	0	0	0
2013262770	913	rural/clinic	No	31/01/2017	0	0	0
2015318232	913	rural/clinic	No	31/01/2017	0	0	0
2015313610	913	rural/clinic	No	31/01/2017	0	0	0
2013247021	352	rural/clinic	No	31/01/2017	0	0	0
2014329236	352	rural/clinic	No	31/01/2017	0	0	0
2015317594	352	rural/clinic	No	31/01/2017	0	0	0
2014329237	352	rural/clinic	No	31/01/2017	0	0	0
2011117488	352	rural/clinic	No	31/01/2017	0	0	0
2015317595	352	rural/clinic	No	31/01/2017	0	0	0
2015299765	352	rural/clinic	No	31/01/2017	0	0	0
2014329229	352	rural/clinic	No	31/01/2017	0	0	0
2015295335	352	rural/clinic	No	31/01/2017	0	0	0
2015317596	352	rural/clinic	No	31/01/2017	0	0	0
2014329239	352	rural/clinic	No	31/01/2017	0	0	0
2015317597	352	rural/clinic	No	31/01/2017	0	0	0
2015295339	352	rural/clinic	No	31/01/2017	0	0	0
2015317598	352	rural/clinic	No	31/01/2017	0	0	0
2014329238	352	rural/clinic	No	31/01/2017	0	0	0
2014353161	352	rural/clinic	No	31/01/2017	0	0	0
2014329235	352	rural/clinic	No	31/01/2017	0	0	0
2013262769	352	rural/clinic	No	31/01/2017	0	0	0
2015377604	352	rural/clinic	No	31/01/2017	0	0	0
2015377605	352	rural/clinic	No	31/01/2017	0	0	0
2014353162	352	rural/clinic	No	31/01/2017	0	0	0
2015377606	352	rural/clinic	No	31/01/2017	0	0	0
2015377607	352	rural/clinic	No	31/01/2017	0	0	0
2013262771	352	rural/clinic	No	31/01/2017	0	0	0
2011196003	352	rural/clinic	No	31/01/2017	0	0	0
2013273000	352	rural/clinic	No	31/01/2017	0	0	0
2014286901	352	rural/clinic	No	31/01/2017	0	0	0
2013247019	352	rural/clinic	No	31/01/2017	0	0	0
2015401543	352	rural/clinic	No	02/02/2017	0	0	0
2013247022	352	rural/clinic	No	31/01/2017	0	0	0
2015401541	352	rural/clinic	No	02/02/2017	0	0	0
2015401538	352	rural/clinic	No	02/02/2017	0	0	0
2011141548	352	rural/clinic	No	31/01/2017	0	0	0
2015401536	352	rural/clinic	No	02/02/2017	0	0	0
2011141549	352	rural/clinic	No	31/01/2017	0	0	0
2014379531	352	rural/clinic	No	02/02/2017	0	0	0
2014289469	352	rural/clinic	No	31/01/2017	0	0	0
2014385231	352	rural/clinic	No	02/02/2017	0	0	0
2014289478	352	rural/clinic	No	31/01/2017	0	0	0
2015382362	352	rural/clinic	No	02/02/2017	0	0	0
2015325873	352	rural/clinic	No	02/02/2017	0	0	0
2015369908	352	rural/clinic	No	03/01/2017	0	0	0
2015382360	352	rural/clinic	No	02/02/2017	0	0	0
2015382361	352	rural/clinic	No	02/02/2017	0	0	0
2015334702	352	rural/clinic	No	02/02/2017	0	0	0
2014289479	352	rural/clinic	No	31/01/2017	0	0	0
2014385234	352	rural/clinic	No	02/02/2017	0	0	0
2014289480	352	rural/clinic	No	31/01/2017	0	0	0
2015334701	352	rural/clinic	No	02/02/2017	0	0	0
2014289468	352	rural/clinic	No	31/01/2017	0	0	0
2015369480	352	rural/clinic	No	02/02/2017	0	0	0
2014289481	352	rural/clinic	No	31/01/2017	0	0	0
2014341052	352	rural/clinic	No	02/02/2017	0	0	0
2015351129	352	rural/clinic	No	31/01/2017	0	0	0
2015369481	352	rural/clinic	No	02/02/2017	0	0	0
2015351130	352	rural/clinic	No	01/02/2017	0	0	0
2015401542	352	rural/clinic	No	02/02/2017	0	0	0
2015314771	352	rural/clinic	No	01/02/2017	0	0	0
2012363288	352	rural/clinic	No	02/02/2017	0	0	0
2012363282	352	rural/clinic	No	02/02/2017	0	0	0
2015302482	352	rural/clinic	No	11/01/2017	0	0	0

2015335098	352	rural/clinic	No	11/01/2017	0	0	0
2012363291	352	rural/clinic	No	02/02/2017	0	0	0
2013247131	352	rural/clinic	No	11/01/2017	0	0	0
2012363274	352	rural/clinic	No	02/02/2017	0	0	0
2012363281	352	rural/clinic	No	02/02/2017	0	0	0
2015357351	352	rural/clinic	No	11/01/2017	0	0	0
2012363280	352	rural/clinic	No	02/02/2017	0	0	0
2012363287	352	rural/clinic	No	02/02/2017	0	0	0
2012363289	352	rural/clinic	No	02/02/2017	0	0	0
2015401535	352	rural/clinic	No	02/02/2017	0	0	0
2014359722	352	rural/clinic	No	11/01/2017	0	0	0
2015290480	352	rural/clinic	No	11/01/2017	0	0	0
2015368903	352	rural/clinic	No	11/01/2017	0	0	0
2014311523	352	rural/clinic	No	11/01/2017	0	0	0
2014290230	352	rural/clinic	No	11/01/2017	0	0	0
2015413574	352	rural/clinic	No	03/01/2017	0	0	0
2014344289	352	rural/clinic	No	02/02/2017	0	0	0
2015377683	352	rural/clinic	No	03/01/2017	0	0	0
2012353578	352	rural/clinic	No	03/01/2017	0	0	0
2015335185	352	rural/clinic	No	02/02/2017	0	0	0
2015339206	352	rural/clinic	No	03/01/2017	0	0	0
2015335184	352	rural/clinic	No	02/02/2017	0	0	0
2011144994	352	rural/clinic	No	02/02/2017	0	0	0
2012290677	352	rural/clinic	No	02/02/2017	0	0	0
2015310908	352	rural/clinic	No	31/01/2017	0	0	0
2012290676	352	rural/clinic	No	02/02/2017	0	0	0
2012290675	352	rural/clinic	No	02/02/2017	0	0	0
2012290679	352	rural/clinic	No	02/02/2017	0	0	0
2015377011	352	rural/clinic	No	02/02/2017	0	0	0
2015335186	352	rural/clinic	No	02/02/2017	0	0	0
2012246280	352	rural/clinic	No	02/02/2017	0	0	0
2014372438	352	rural/clinic	No	02/02/2017	0	0	0
2014290715	490	rural/clinic	No	02/02/2017	0	0	0
2012331320	490	rural/clinic	No	02/02/2017	0	0	0
2014290718	490	rural/clinic	No	02/02/2017	0	0	0
2015408096	490	rural/clinic	No	02/02/2017	0	0	0
2015302855	490	rural/clinic	No	02/02/2017	0	0	0
2015352420	490	rural/clinic	No	02/02/2017	0	0	0
2015302853	490	rural/clinic	No	02/02/2017	0	0	0
2015352419	490	rural/clinic	No	02/02/2017	0	0	0
2012304198	490	rural/clinic	No	02/02/2017	0	0	0
2015352421	490	rural/clinic	No	02/02/2017	0	0	0
2012259756	490	rural/clinic	No	02/02/2017	0	0	0
2015352422	490	rural/clinic	No	02/02/2017	0	0	0
2015368426	490	rural/clinic	No	02/02/2017	0	0	0
2014372723	490	rural/clinic	No	02/02/2017	0	0	0
2015368422	490	rural/clinic	No	02/02/2017	0	0	0
2015368424	490	rural/clinic	No	02/02/2017	0	0	0
2014372722	490	rural/clinic	No	02/02/2017	0	0	0
2015368425	490	rural/clinic	No	02/02/2017	0	0	0
2015324254	490	rural/clinic	No	02/02/2017	0	0	0
2015368428	490	rural/clinic	No	02/02/2017	0	0	0
2015324255	490	rural/clinic	No	02/02/2017	0	0	0
2015368423	490	rural/clinic	No	02/02/2017	0	0	0
2015332008	490	rural/clinic	No	02/02/2017	0	0	0
2014383965	490	rural/clinic	No	02/02/2017	0	0	0
2015336877	490	rural/clinic	No	02/02/2017	0	0	0
2015408095	490	rural/clinic	No	02/02/2017	0	0	0
2015305241	490	rural/clinic	No	02/02/2017	0	0	0
2014335297	490	rural/clinic	No	02/02/2017	0	0	0
2015305240	490	rural/clinic	No	02/02/2017	0	0	0
2015352418	490	rural/clinic	No	02/02/2017	0	0	0
2015305052	490	rural/clinic	No	02/02/2017	0	0	0
2015305239	490	rural/clinic	No	02/02/2017	0	0	0
2012291834	490	rural/clinic	No	02/02/2017	0	0	0
2012291833	490	rural/clinic	No	02/02/2017	0	0	0
2014381900	490	rural/clinic	No	02/02/2017	0	0	0
2015373338	490	rural/clinic	No	02/02/2017	0	0	0
2015332007	490	rural/clinic	No	02/02/2017	0	0	0
2015373336	490	rural/clinic	No	02/02/2017	0	0	0
2014335519	490	rural/clinic	No	02/02/2017	0	0	0
2015373337	490	rural/clinic	No	02/02/2017	0	0	0
2014335521	490	rural/clinic	No	02/02/2017	0	0	0
2015286519	490	rural/clinic	No	02/02/2017	0	0	0
2012387243	490	rural/clinic	No	02/02/2017	0	0	0
2014335520	490	rural/clinic	No	02/02/2017	0	0	0
2012387242	490	rural/clinic	No	02/02/2017	0	0	0
2015358278	394	rural/clinic	No	02/02/2017	0	0	0
2015412765	394	rural/clinic	No	02/02/2017	0	0	0
2011155222	394	rural/clinic	No	02/02/2017	0	0	0
2015412764	394	rural/clinic	No	02/02/2017	0	0	0
2015358280	394	rural/clinic	No	02/02/2017	0	0	0

2015412254	394	rural/clinic	No	02/02/2017	0	0	0
2015414367	394	rural/clinic	No	17/01/2017	0	0	0
2015414367	394	rural/clinic	No	17/01/2017	0	0	0
2015414368	394	rural/clinic	No	17/01/2017	0	0	0
2015414368	394	rural/clinic	No	17/01/2017	0	0	0
201052000	394	rural/clinic	No	17/01/2017	0	0	0
201052000	394	rural/clinic	No	17/01/2017	0	0	0
2015351122	394	rural/clinic	No	17/01/2017	0	0	0
2015351122	394	rural/clinic	No	17/01/2017	0	0	0
2015351123	394	rural/clinic	No	17/01/2017	0	0	0
2015351123	394	rural/clinic	No	17/01/2017	0	0	0
2012365139	394	rural/clinic	No	17/01/2017	0	0	0
2012365139	394	rural/clinic	No	17/01/2017	0	0	0
2014326078	394	rural/clinic	No	02/02/2017	0	0	0
2015358279	394	rural/clinic	No	02/02/2017	0	0	0
2012305583	394	rural/clinic	No	02/02/2017	0	0	0
2015358275	394	rural/clinic	No	02/02/2017	0	0	0
2015297063	394	rural/clinic	No	02/02/2017	0	0	0
2011155221	394	rural/clinic	No	02/02/2017	0	0	0
2012376589	394	rural/clinic	No	02/02/2017	0	0	0
2012376590	394	rural/clinic	No	02/02/2017	0	0	0
2014312267	394	rural/clinic	No	02/02/2017	0	0	0
2012376591	394	rural/clinic	No	02/02/2017	0	0	0
2015358269	394	rural/clinic	No	02/02/2017	0	0	0
2014301326	394	rural/clinic	No	02/02/2017	0	0	0
2014317425	394	rural/clinic	No	02/02/2017	0	0	0
2015358270	394	rural/clinic	No	02/02/2017	0	0	0
2014315519	394	rural/clinic	No	19/01/2017	0	0	0
2014315519	394	rural/clinic	No	19/01/2017	0	0	0
2015349924	394	rural/clinic	No	19/01/2017	0	0	0
2015358121	260	rural/clinic	No	02/02/2017	0	0	0
2015372017	260	rural/clinic	No	02/02/2017	0	0	0
2015372016	260	rural/clinic	No	02/02/2017	0	0	0
2015358123	260	rural/clinic	No	02/02/2017	0	0	0
2014305938	260	rural/clinic	No	02/02/2017	0	0	0
2015358126	260	rural/clinic	No	02/02/2017	0	0	0
2014317339	260	rural/clinic	No	02/02/2017	0	0	0
2015358125	260	rural/clinic	No	02/02/2017	0	0	0
2014317340	260	rural/clinic	No	02/02/2017	0	0	0
2015358122	260	rural/clinic	No	02/02/2017	0	0	0
2012352823	234	rural/clinic	No	02/02/2017	0	0	0
2015358124	234	rural/clinic	No	02/02/2017	0	0	0
2012352820	234	rural/clinic	No	02/02/2017	0	0	0
2012352821	234	rural/clinic	No	02/02/2017	0	0	0
2011232679	234	rural/clinic	No	02/02/2017	0	0	0
2013264178	234	rural/clinic	No	02/02/2017	0	0	0
2013264177	234	rural/clinic	No	02/02/2017	0	0	0
2011232674	234	rural/clinic	No	02/02/2017	0	0	0
2013264176	234	rural/clinic	No	02/02/2017	0	0	0
2011232675	234	rural/clinic	No	02/02/2017	0	0	0
2014317741	234	rural/clinic	No	02/02/2017	0	0	0
2011232678	234	rural/clinic	No	02/02/2017	0	0	0
2014317426	234	rural/clinic	No	02/02/2017	0	0	0
2014358320	234	rural/clinic	No	02/02/2017	0	0	0
2014358321	234	rural/clinic	No	02/02/2017	0	0	0
2014378469	234	rural/clinic	No	02/02/2017	0	0	0
2014358322	234	rural/clinic	No	02/02/2017	0	0	0
2014378470	234	rural/clinic	No	02/02/2017	0	0	0
2012352822	234	rural/clinic	No	02/02/2017	0	0	0
2015384524	234	rural/clinic	No	02/02/2017	0	0	0
2015360088	234	rural/clinic	No	02/02/2017	0	0	0
2015377156	234	rural/clinic	No	02/02/2017	0	0	0
2015377010	234	rural/clinic	No	02/02/2017	0	0	0
2015360086	234	rural/clinic	No	02/02/2017	0	0	0
2011229212	234	rural/clinic	No	02/02/2017	0	0	0
2015301353	234	rural/clinic	No	02/02/2017	0	0	0
2014317513	234	rural/clinic	No	02/02/2017	0	0	0
2015301351	234	rural/clinic	No	02/02/2017	0	0	0
2014317514	234	rural/clinic	No	02/02/2017	0	0	0
2012251533	234	rural/clinic	No	02/02/2017	0	0	0
2015360089	234	rural/clinic	No	02/02/2017	0	0	0
2012251532	234	rural/clinic	No	02/02/2017	0	0	0
2015301352	234	rural/clinic	No	02/02/2017	0	0	0
2015340476	234	rural/clinic	No	02/02/2017	0	0	0
2015349928	234	rural/clinic	No	02/02/2017	0	0	0
2015340475	234	rural/clinic	No	02/02/2017	0	0	0
2015349927	234	rural/clinic	No	02/02/2017	0	0	0
2015340474	234	rural/clinic	No	02/02/2017	0	0	0
2012363283	234	rural/clinic	No	02/02/2017	0	0	0
2015340473	234	rural/clinic	No	02/02/2017	0	0	0
2015340472	234	rural/clinic	No	02/02/2017	0	0	0
2014290717	234	rural/clinic	No	02/02/2017	0	0	0

2014290716	234	rural/clinic	No	02/02/2017	0	0	0
2014290714	234	rural/clinic	No	02/02/2017	0	0	0
2015373228	234	rural/clinic	No	02/02/2017	0	0	0
2015373225	234	rural/clinic	No	02/02/2017	0	0	0
2015373219	234	rural/clinic	No	02/02/2017	0	0	0
2015373222	234	rural/clinic	No	02/02/2017	0	0	0
2015373227	234	rural/clinic	No	02/02/2017	0	0	0
2015373223	234	rural/clinic	No	02/02/2017	0	0	0
2015373224	234	rural/clinic	No	02/02/2017	0	0	0
2015373226	234	rural/clinic	No	02/02/2017	0	0	0
2014335218	234	rural/clinic	No	02/02/2017	0	0	0
2014361044	234	rural/clinic	No	02/02/2017	0	0	0
2015360583	234	rural/clinic	No	02/02/2017	0	0	0
2015360582	234	rural/clinic	No	02/02/2017	0	0	0
2014335216	234	rural/clinic	No	02/02/2017	0	0	0
2014335221	234	rural/clinic	No	02/02/2017	0	0	0
2014335217	234	rural/clinic	No	02/02/2017	0	0	0
2015413080	234	rural/clinic	No	02/02/2017	0	0	0
2015413079	234	rural/clinic	No	02/02/2017	0	0	0
2015413078	234	rural/clinic	No	02/02/2017	0	0	0
2015413077	234	rural/clinic	No	02/02/2017	0	0	0
2015413084	234	rural/clinic	No	02/02/2017	0	0	0
2015413083	234	rural/clinic	No	02/02/2017	0	0	0
2015413081	234	rural/clinic	No	02/02/2017	0	0	0
2015413082	234	rural/clinic	No	02/02/2017	0	0	0
2012341037	234	rural/clinic	No	02/02/2017	0	0	0
2012341038	234	rural/clinic	No	02/02/2017	0	0	0
2012341036	687	rural/clinic	No	02/02/2017	0	0	0
2014347980	687	district/faith-based	No	02/02/2017	1	0	0
2014365148	687	district/faith-based	No	02/02/2017	1	0	0
2014385684	687	district/faith-based	No	02/02/2017	1	0	0
2014365149	687	district/faith-based	No	02/02/2017	1	0	0
2013272758	687	district/faith-based	No	02/02/2017	1	0	0
2015321757	687	district/faith-based	No	02/02/2017	1	0	0
2011117486	687	district/faith-based	No	02/02/2017	1	0	0
2013272757	687	district/faith-based	No	02/02/2017	1	0	0
2015286271	687	district/faith-based	No	02/02/2017	1	0	0
2012384778	687	district/faith-based	No	02/02/2017	1	0	0
2014319519	687	district/faith-based	No	02/02/2017	1	0	0
2015413630	687	district/faith-based	No	02/02/2017	1	0	0
2015413629	687	district/faith-based	No	02/02/2017	1	0	0
2015286270	687	district/faith-based	No	02/02/2017	1	0	0
2015335183	687	district/faith-based	No	02/02/2017	1	0	0
2015335182	687	district/faith-based	No	02/02/2017	1	0	0
2015351131	687	district/faith-based	No	03/02/2017	1	0	0
2015286269	687	district/faith-based	No	02/02/2017	1	0	0
2015286267	687	district/faith-based	No	02/02/2017	1	0	0
2015351132	687	district/faith-based	No	03/02/2017	1	0	0
2015351133	687	district/faith-based	No	03/02/2017	1	0	0
2015286268	687	district/faith-based	No	02/02/2017	1	0	0
2015378946	687	district/faith-based	No	02/02/2017	1	0	0
2015378945	687	district/faith-based	No	02/02/2017	1	0	0
2014319719	294	rural/clinic	No	02/02/2017	0	0	0
2014319718	294	rural/clinic	No	02/02/2017	0	0	0
2015373381	294	rural/clinic	No	02/02/2017	0	0	0
2015373380	294	rural/clinic	No	02/02/2017	0	0	0
2015373379	294	rural/clinic	No	02/02/2017	0	0	0
2015373378	294	rural/clinic	No	02/02/2017	0	0	0
2014317427	294	rural/clinic	No	02/02/2017	0	0	0
2014317613	294	rural/clinic	No	02/02/2017	0	0	0
2014317614	294	rural/clinic	No	02/02/2017	0	0	0
2015369483	294	rural/clinic	No	02/02/2017	0	0	0
2015369484	294	rural/clinic	No	02/02/2017	0	0	0
2015369482	914	rural/clinic	No	02/02/2017	0	0	0
2015369486	914	rural/clinic	No	02/02/2017	0	0	0
2015369485	610	rural/clinic	No	02/02/2017	0	0	0
2011144997	610	rural/clinic	No	02/02/2017	0	0	0
2011144996	610	rural/clinic	No	02/02/2017	0	0	0
2012369964	610	rural/clinic	No	02/02/2017	0	0	0
2011144995	610	rural/clinic	No	02/02/2017	0	0	0
2011144998	610	rural/clinic	No	02/02/2017	0	0	0
2015339456	610	rural/clinic	No	02/02/2017	0	0	0
2015339457	610	rural/clinic	No	02/02/2017	0	0	0
2015352993	610	rural/clinic	No	02/02/2017	0	0	0
2015352992	610	rural/clinic	No	02/02/2017	0	0	0
2015352991	610	rural/clinic	No	02/02/2017	0	0	0
2015352990	610	rural/clinic	No	02/02/2017	0	0	0
2014287173	610	rural/clinic	No	02/02/2017	0	0	0
2015352989	610	rural/clinic	No	02/02/2017	0	0	0
2015352988	610	rural/clinic	No	02/02/2017	0	0	0
2015289163	610	rural/clinic	No	02/02/2017	0	0	0
2015286585	610	rural/clinic	No	02/02/2017	0	0	0

2015378718	610	rural/clinic	No	02/02/2017	0	0	0
2015378717	610	rural/clinic	No	02/02/2017	0	0	0
2015378719	610	rural/clinic	No	02/02/2017	0	0	0
2014313548	610	rural/clinic	No	02/02/2017	0	0	0
2014313549	610	rural/clinic	No	02/02/2017	0	0	0
2015351125	610	rural/clinic	No	23/01/2017	0	0	0
2015351125	610	rural/clinic	No	23/01/2017	0	0	0
2015351124	610	rural/clinic	No	23/01/2017	0	0	0
2015351124	610	rural/clinic	No	23/01/2017	0	0	0
2014344530	610	rural/clinic	No	02/02/2017	0	0	0
2015286584	610	rural/clinic	No	02/02/2017	0	0	0
2015378720	610	rural/clinic	No	02/02/2017	0	0	0
2013273393	610	rural/clinic	No	02/02/2017	0	0	0
2015359832	610	rural/clinic	No	24/01/2017	0	0	0
2015359832	610	rural/clinic	No	24/01/2017	0	0	0
2015385342	610	rural/clinic	No	24/01/2017	0	0	0
2015385342	610	rural/clinic	No	24/01/2017	0	0	0
2015368139	610	rural/clinic	No	02/02/2017	0	0	0
2015349922	610	rural/clinic	No	19/01/2017	0	0	0
2015349922	610	rural/clinic	No	19/01/2017	0	0	0
2015368140	171	rural/clinic	No	02/02/2017	0	0	0
2015335647	171	rural/clinic	No	24/01/2017	0	0	0
2015335647	171	rural/clinic	No	24/01/2017	0	0	0
2015335649	171	rural/clinic	No	24/01/2017	0	0	0
2015335649	171	rural/clinic	No	24/01/2017	0	0	0
2015382857	171	rural/clinic	No	24/01/2017	0	0	0
2015382857	171	rural/clinic	No	24/01/2017	0	0	0
2015382856	171	rural/clinic	No	24/01/2017	0	0	0
2015382856	171	rural/clinic	No	24/01/2017	0	0	0
2012246181	171	rural/clinic	No	24/01/2017	0	0	0
2012246181	171	rural/clinic	No	24/01/2017	0	0	0
2015418944	171	rural/clinic	No	24/01/2017	0	0	0
2015418944	171	rural/clinic	No	24/01/2017	0	0	0
2015369371	491	rural/clinic	No	02/02/2017	0	0	0
2015369374	491	rural/clinic	No	02/02/2017	0	0	0
2012363277	491	rural/clinic	No	24/01/2017	0	0	0
2012363277	491	rural/clinic	No	24/01/2017	0	0	0
2015369372	491	rural/clinic	No	02/02/2017	0	0	0
2015369373	491	rural/clinic	No	02/02/2017	0	0	0
2014363586	491	rural/clinic	No	24/01/2017	0	0	0
2014363586	491	rural/clinic	No	24/01/2017	0	0	0
2014303051	491	rural/clinic	No	02/02/2017	0	0	0
2014303052	491	rural/clinic	No	02/02/2017	0	0	0
2013267198	491	rural/clinic	No	02/02/2017	0	0	0
2013267197	491	rural/clinic	No	02/02/2017	0	0	0
2013267196	491	rural/clinic	No	02/02/2017	0	0	0
2014338245	491	rural/clinic	No	02/02/2017	0	0	0
2014338244	491	rural/clinic	No	02/02/2017	0	0	0
2015382479	491	rural/clinic	No	02/02/2017	0	0	0
2015382478	491	rural/clinic	No	02/02/2017	0	0	0
2015382477	491	rural/clinic	No	02/02/2017	0	0	0
2015382476	491	rural/clinic	No	02/02/2017	0	0	0
2015382475	491	rural/clinic	No	02/02/2017	0	0	0
2015382474	491	rural/clinic	No	02/02/2017	0	0	0
2015382473	491	rural/clinic	No	02/02/2017	0	0	0
2014302196	172	rural/clinic	No	07/02/2017	0	0	0
2014302197	172	rural/clinic	No	07/02/2017	0	0	0
2015362336	172	rural/clinic	No	07/02/2017	0	0	0
2012363292	172	rural/clinic	No	07/02/2017	0	0	0
2015362335	172	rural/clinic	No	07/02/2017	0	0	0
2014369032	172	rural/clinic	No	07/02/2017	0	0	0
2014369031	172	rural/clinic	No	07/02/2017	0	0	0
2014369187	172	rural/clinic	No	07/02/2017	0	0	0
2014358177	172	rural/clinic	No	07/02/2017	0	0	0
2014358678	172	rural/clinic	No	07/02/2017	0	0	0
2012361532	172	rural/clinic	No	07/02/2017	0	0	0
2015388502	172	rural/clinic	No	07/02/2017	0	0	0
2012363951	172	rural/clinic	No	07/02/2017	0	0	0
2015320861	172	rural/clinic	No	07/02/2017	0	0	0
2012295954	172	rural/clinic	No	07/02/2017	0	0	0
2014366065	172	rural/clinic	No	07/02/2017	0	0	0
2012325679	172	rural/clinic	No	07/02/2017	0	0	0
2012295955	172	rural/clinic	No	07/02/2017	0	0	0
2014307406	172	rural/clinic	No	07/02/2017	0	0	0
2012295956	172	rural/clinic	No	07/02/2017	0	0	0
2012361942	172	rural/clinic	No	07/02/2017	0	0	0
2012295957	172	rural/clinic	No	07/02/2017	0	0	0
2012295958	172	rural/clinic	No	07/02/2017	0	0	0
2014302200	172	rural/clinic	No	07/02/2017	0	0	0
2015335650	915	district/faith-based	No	07/02/2017	1	0	0
2013285120	915	district/faith-based	No	07/02/2017	1	0	0
2014339400	915	district/faith-based	No	07/02/2017	1	0	0

2013285121	915	district/faith-based	No	07/02/2017	1	0	0
2015388501	915	district/faith-based	No	07/02/2017	1	0	0
2014307407	915	district/faith-based	No	07/02/2017	1	0	0
2014375651	915	district/faith-based	No	07/02/2017	1	0	0
2013263584	915	district/faith-based	No	07/02/2017	1	0	0
2014334167	915	district/faith-based	No	07/02/2017	1	0	0
2014334165	915	district/faith-based	No	07/02/2017	1	0	0
2013263583	915	district/faith-based	No	07/02/2017	1	0	0
2015286586	915	district/faith-based	No	07/02/2017	1	0	0
2014334166	915	district/faith-based	No	07/02/2017	1	0	0
2015385432	915	district/faith-based	No	07/02/2017	1	0	0
2013263582	915	district/faith-based	No	07/02/2017	1	0	0
2015385431	915	district/faith-based	No	07/02/2017	1	0	0
2015385433	915	district/faith-based	No	07/02/2017	1	0	0
2013263581	915	district/faith-based	No	07/02/2017	1	0	0
2015385430	915	district/faith-based	No	07/02/2017	1	0	0
2015315477	915	district/faith-based	No	07/02/2017	1	0	0
2013263585	915	district/faith-based	No	07/02/2017	1	0	0
2015315476	915	district/faith-based	No	07/02/2017	1	0	0
2013263580	915	district/faith-based	No	07/02/2017	1	0	0
2015315473	915	district/faith-based	No	07/02/2017	1	0	0
2015315475	915	district/faith-based	No	07/02/2017	1	0	0
2015315472	915	district/faith-based	No	07/02/2017	1	0	0
2015324424	915	district/faith-based	No	07/02/2017	1	0	0
2015305053	915	district/faith-based	No	07/02/2017	1	0	0
2015315976	295	rural/clinic	No	07/02/2017	0	0	0
2015324461	295	rural/clinic	No	07/02/2017	0	0	0
2012306136	295	rural/clinic	No	07/02/2017	0	0	0
2015325171	295	rural/clinic	No	07/02/2017	0	0	0
2015302495	295	rural/clinic	No	07/02/2017	0	0	0
2015385383	295	rural/clinic	No	07/02/2017	0	0	0
2015302496	295	rural/clinic	No	07/02/2017	0	0	0
2015385382	295	rural/clinic	No	07/02/2017	0	0	0
2012306137	295	rural/clinic	No	07/02/2017	0	0	0
201066573	295	rural/clinic	No	07/02/2017	0	0	0
2015357365	295	rural/clinic	No	07/02/2017	0	0	0
2014339249	295	rural/clinic	No	07/02/2017	0	0	0
2015357367	295	rural/clinic	No	07/02/2017	0	0	0
2012358277	295	rural/clinic	No	07/02/2017	0	0	0
2015357366	295	rural/clinic	No	07/02/2017	0	0	0
2012345093	295	rural/clinic	No	07/02/2017	0	0	0
2012345094	295	rural/clinic	No	07/02/2017	0	0	0
2015302494	295	rural/clinic	No	07/02/2017	0	0	0
2012345092	688	rural/clinic	No	07/02/2017	0	0	0
2015325172	688	rural/clinic	No	07/02/2017	0	0	0
2012345095	688	rural/clinic	No	07/02/2017	0	0	0
2015368775	688	rural/clinic	No	07/02/2017	0	0	0
2015337351	688	rural/clinic	No	07/02/2017	0	0	0
2015368776	688	rural/clinic	No	07/02/2017	0	0	0
2012295151	688	rural/clinic	No	07/02/2017	0	0	0
201066574	235	rural/clinic	No	07/02/2017	0	0	0
2015331171	235	rural/clinic	No	07/02/2017	0	0	0
2014339248	235	rural/clinic	No	07/02/2017	0	0	0
2015300662	235	rural/clinic	No	07/02/2017	0	0	0
2014339250	235	rural/clinic	No	07/02/2017	0	0	0
2015385930	235	rural/clinic	No	07/02/2017	0	0	0
2015300664	235	rural/clinic	No	07/02/2017	0	0	0
2015315978	235	rural/clinic	No	07/02/2017	0	0	0
2015300663	235	rural/clinic	No	07/02/2017	0	0	0
2015315977	235	rural/clinic	No	07/02/2017	0	0	0
2015418950	235	rural/clinic	No	07/02/2017	0	0	0
2011134952	235	rural/clinic	No	07/02/2017	0	0	0
2014346449	235	rural/clinic	No	07/02/2017	0	0	0
2011134953	235	rural/clinic	No	07/02/2017	0	0	0
2014346450	235	rural/clinic	No	07/02/2017	0	0	0
2011134951	235	rural/clinic	No	07/02/2017	0	0	0
2015362751	235	rural/clinic	No	07/02/2017	0	0	0
2014342501	235	rural/clinic	No	07/02/2017	0	0	0
2015418116	235	rural/clinic	No	07/02/2017	0	0	0
2015294869	235	rural/clinic	No	07/02/2017	0	0	0
2015418115	235	rural/clinic	No	07/02/2017	0	0	0
2015377546	235	rural/clinic	No	07/02/2017	0	0	0
2015377543	235	rural/clinic	No	07/02/2017	0	0	0
2014369185	235	rural/clinic	No	07/02/2017	0	0	0
2015355770	235	rural/clinic	No	07/02/2017	0	0	0
2013266066	235	rural/clinic	No	07/02/2017	0	0	0
2015355771	235	rural/clinic	No	07/02/2017	0	0	0
2015295194	235	rural/clinic	No	07/02/2017	0	0	0
2014369186	235	rural/clinic	No	07/02/2017	0	0	0
2014346448	235	rural/clinic	No	07/02/2017	0	0	0
2015295191/d	235	rural/clinic	No	07/02/2017	0	0	0
2012317311	235	rural/clinic	No	07/02/2017	0	0	0

2015382869	235	rural/clinic	No	07/02/2017	0	0	0
2015355721	235	rural/clinic	No	07/02/2017	0	0	0
2015382868	235	rural/clinic	No	07/02/2017	0	0	0
2015382867	235	rural/clinic	No	07/02/2017	0	0	0
2015385599	235	rural/clinic	No	07/02/2017	0	0	0
2015382866	235	rural/clinic	No	07/02/2017	0	0	0
2015377547	235	rural/clinic	No	07/02/2017	0	0	0
2015382865	235	rural/clinic	No	07/02/2017	0	0	0
2015339237	235	rural/clinic	No	07/02/2017	0	0	0
2012261444	235	rural/clinic	No	07/02/2017	0	0	0
2015339238	235	rural/clinic	No	07/02/2017	0	0	0
2015335417	235	rural/clinic	No	07/02/2017	0	0	0
2015339239	235	rural/clinic	No	07/02/2017	0	0	0
2013256388	235	rural/clinic	No	07/02/2017	0	0	0
2015339240	235	rural/clinic	No	07/02/2017	0	0	0
2013256387	235	rural/clinic	No	07/02/2017	0	0	0
2015339241	235	rural/clinic	No	07/02/2017	0	0	0
2015339242	235	rural/clinic	No	07/02/2017	0	0	0
2015339243	235	rural/clinic	No	07/02/2017	0	0	0
2015339244	235	rural/clinic	No	07/02/2017	0	0	0
2015339245	235	rural/clinic	No	07/02/2017	0	0	0
2014345842	235	rural/clinic	No	07/02/2017	0	0	0
2014345843	235	rural/clinic	No	07/02/2017	0	0	0
2012387245	235	rural/clinic	No	07/02/2017	0	0	0
2014326619	235	rural/clinic	No	07/02/2017	0	0	0
2014326618	235	rural/clinic	No	07/02/2017	0	0	0
2014326622	235	rural/clinic	No	07/02/2017	0	0	0
2014326620	235	rural/clinic	No	07/02/2017	0	0	0
2014326621	235	rural/clinic	No	07/02/2017	0	0	0
2013254141	235	rural/clinic	No	07/02/2017	0	0	0
2013254142	235	rural/clinic	No	07/02/2017	0	0	0
2015335571	235	rural/clinic	No	07/02/2017	0	0	0
2015351134	235	rural/clinic	No	07/02/2017	0	0	0
2014309379	235	rural/clinic	No	07/02/2017	0	0	0
2015295193	235	rural/clinic	No	07/02/2017	0	0	0
2015397484	235	rural/clinic	No	07/02/2017	0	0	0
2015295192	235	rural/clinic	No	07/02/2017	0	0	0
2015295195	235	rural/clinic	No	07/02/2017	0	0	0
2015397483	235	rural/clinic	No	07/02/2017	0	0	0
2015397482	235	rural/clinic	No	07/02/2017	0	0	0
2015289277	235	rural/clinic	No	07/02/2017	0	0	0
2012295137	235	rural/clinic	No	07/02/2017	0	0	0
2015397481	235	rural/clinic	No	07/02/2017	0	0	0
2015355723	235	rural/clinic	No	07/02/2017	0	0	0
2015294867	235	rural/clinic	No	07/02/2017	0	0	0
2015397490	235	rural/clinic	No	07/02/2017	0	0	0
2015294868	235	rural/clinic	No	07/02/2017	0	0	0
2015294870	235	rural/clinic	No	07/02/2017	0	0	0
2015397489	235	rural/clinic	No	07/02/2017	0	0	0
2015355722	235	rural/clinic	No	07/02/2017	0	0	0
2015340828	235	rural/clinic	No	07/02/2017	0	0	0
2015397488	235	rural/clinic	No	07/02/2017	0	0	0
2011235848	235	rural/clinic	No	07/02/2017	0	0	0
2015397487	235	rural/clinic	No	07/02/2017	0	0	0
2015397486	235	rural/clinic	No	07/02/2017	0	0	0
2012306339	235	rural/clinic	No	07/02/2017	0	0	0
2015397485	235	rural/clinic	No	07/02/2017	0	0	0
2015340831	235	rural/clinic	No	07/02/2017	0	0	0
2015340832	235	rural/clinic	No	07/02/2017	0	0	0
2015397493	235	rural/clinic	No	07/02/2017	0	0	0
2015291446	235	rural/clinic	No	07/02/2017	0	0	0
2014382422	235	rural/clinic	No	07/02/2017	0	0	0
2015397492	235	rural/clinic	No	07/02/2017	0	0	0
2014382423	235	rural/clinic	No	07/02/2017	0	0	0
2015397491	235	rural/clinic	No	07/02/2017	0	0	0
2012363290	235	rural/clinic	No	07/02/2017	0	0	0
2014382419	235	rural/clinic	No	07/02/2017	0	0	0
2015320860	235	rural/clinic	No	07/02/2017	0	0	0
2012377874	235	rural/clinic	No	07/02/2017	0	0	0
2015320859	235	rural/clinic	No	07/02/2017	0	0	0
2015361682	235	rural/clinic	No	07/02/2017	0	0	0
2013266067	235	rural/clinic	No	07/02/2017	0	0	0
2013285122	235	rural/clinic	No	07/02/2017	0	0	0
2014370184	235	rural/clinic	No	07/02/2017	0	0	0
2014370186	235	rural/clinic	No	07/02/2017	0	0	0
2014370187	235	rural/clinic	No	07/02/2017	0	0	0
2012325678	235	rural/clinic	No	07/02/2017	0	0	0
2012363293	235	rural/clinic	No	07/02/2017	0	0	0
2014350555	235	rural/clinic	No	07/02/2017	0	0	0
2014370188	235	rural/clinic	No	07/02/2017	0	0	0
2014370189	235	rural/clinic	No	07/02/2017	0	0	0
2013271300	235	rural/clinic	No	07/02/2017	0	0	0

2011192932	235	rural/clinic	No	07/02/2017	0	0	0
2011192933	235	rural/clinic	No	07/02/2017	0	0	0
2015365067	235	rural/clinic	No	07/02/2017	0	0	0
2015365068	235	rural/clinic	No	07/02/2017	0	0	0
2015414926	235	rural/clinic	No	07/02/2017	0	0	0
2015365071	235	rural/clinic	No	07/02/2017	0	0	0
2015365066	235	rural/clinic	No	07/02/2017	0	0	0
2015314357	235	rural/clinic	No	07/02/2017	0	0	0
2015414925	235	rural/clinic	No	07/02/2017	0	0	0
2015314356	235	rural/clinic	No	07/02/2017	0	0	0
2012245723	235	rural/clinic	No	07/02/2017	0	0	0
2015408744	235	rural/clinic	No	07/02/2017	0	0	0
2015322352	235	rural/clinic	No	07/02/2017	0	0	0
2015408750	235	rural/clinic	No	07/02/2017	0	0	0
2015361678	235	rural/clinic	No	19/01/2017	0	0	0
2015408747	235	rural/clinic	No	07/02/2017	0	0	0
2015408746	235	rural/clinic	No	07/02/2017	0	0	0
2015314304	235	rural/clinic	No	07/02/2017	0	0	0
2015408742	235	rural/clinic	No	07/02/2017	0	0	0
2015295448	235	rural/clinic	No	07/02/2017	0	0	0
2014329247	235	rural/clinic	No	07/02/2017	0	0	0
2014317743	235	rural/clinic	No	07/02/2017	0	0	0
19792 WRONG	235	rural/clinic	No	07/02/2017	0	0	0
2014317746	235	rural/clinic	No	07/02/2017	0	0	0
2014317742	235	rural/clinic	No	07/02/2017	0	0	0
2014317745	235	rural/clinic	No	07/02/2017	0	0	0
2014317747	235	rural/clinic	No	07/02/2017	0	0	0
2015295447	235	rural/clinic	No	07/02/2017	0	0	0
2014329248	235	rural/clinic	No	07/02/2017	0	0	0
2011225146	235	rural/clinic	No	07/02/2017	0	0	0
2012365142	235	rural/clinic	No	07/02/2017	0	0	0
2011225145	353	rural/clinic	No	07/02/2017	0	0	0
2011225147	353	rural/clinic	No	07/02/2017	0	0	0
2012365141	353	rural/clinic	No	07/02/2017	0	0	0
2011225148	353	rural/clinic	No	07/02/2017	0	0	0
2014371677	353	rural/clinic	No	07/02/2017	0	0	0
2011224278	353	rural/clinic	No	07/02/2017	0	0	0
2015418177	353	rural/clinic	No	07/02/2017	0	0	0
2014327108	353	rural/clinic	No	07/02/2017	0	0	0
2014375595	353	rural/clinic	No	24/01/2017	0	0	0
2014375595	353	rural/clinic	No	24/01/2017	0	0	0
2015414307	353	rural/clinic	No	24/01/2017	0	0	0
2015414307	353	rural/clinic	No	24/01/2017	0	0	0
2015418180	353	rural/clinic	No	07/02/2017	0	0	0
2014382421	353	rural/clinic	No	07/02/2017	0	0	0
2015413667	353	rural/clinic	No	26/01/2017	0	0	0
2015413667	353	rural/clinic	No	26/01/2017	0	0	0
2011224276	353	rural/clinic	No	07/02/2017	0	0	0
2015384772	353	rural/clinic	No	24/01/2017	0	0	0
2015384772	353	rural/clinic	No	24/01/2017	0	0	0
2011225149	353	rural/clinic	No	07/02/2017	0	0	0
2015351138	353	rural/clinic	No	07/02/2017	0	0	0
2014358680	353	rural/clinic	No	07/02/2017	0	0	0
2014358681	353	rural/clinic	No	07/02/2017	0	0	0
2015351137	353	rural/clinic	No	07/02/2017	0	0	0
2014358679	353	rural/clinic	No	07/02/2017	0	0	0
2015377421	353	rural/clinic	No	26/01/2017	0	0	0
2015377421	353	rural/clinic	No	26/01/2017	0	0	0
2015293826	353	rural/clinic	No	26/01/2017	0	0	0
2015293826	353	rural/clinic	No	26/01/2017	0	0	0
2015351136	353	rural/clinic	No	07/02/2017	0	0	0
2015339224	353	rural/clinic	No	24/01/2017	0	0	0
2015339224	107	rural/clinic	No	24/01/2017	0	0	0
2015413625	107	rural/clinic	No	24/01/2017	0	0	0
2015413625	107	rural/clinic	No	24/01/2017	0	0	0
2015297352	107	rural/clinic	No	07/02/2017	0	0	0
2014302189	107	rural/clinic	No	24/01/2017	0	0	0
2014302189	107	rural/clinic	No	24/01/2017	0	0	0
2015351135	107	rural/clinic	No	07/02/2017	0	0	0
2015297064	107	rural/clinic	No	07/02/2017	0	0	0
2014335140	107	rural/clinic	No	07/02/2017	0	0	0
2015294446	107	rural/clinic	No	07/02/2017	0	0	0
2015297065	107	rural/clinic	No	07/02/2017	0	0	0
2015297066	107	rural/clinic	No	07/02/2017	0	0	0
2015297067	107	rural/clinic	No	07/02/2017	0	0	0
2015297354	107	rural/clinic	No	07/02/2017	0	0	0
2014288393	107	rural/clinic	No	07/02/2017	0	0	0
2015314307	417	rural/clinic	No	07/02/2017	0	0	0
2015334504	417	rural/clinic	No	07/02/2017	0	0	0
2015314305	417	rural/clinic	No	07/02/2017	0	0	0
2015293926	417	rural/clinic	No	07/02/2017	0	0	0
2015314308	417	rural/clinic	No	07/02/2017	0	0	0

2015314314	417	rural/clinic	No	07/02/2017	0	0	0
2015293927	417	rural/clinic	No	07/02/2017	0	0	0
2015314310	417	rural/clinic	No	07/02/2017	0	0	0
2015314309	417	rural/clinic	No	07/02/2017	0	0	0
2015314303	417	rural/clinic	No	07/02/2017	0	0	0
2014288391	417	rural/clinic	No	07/02/2017	0	0	0
2015314302	417	rural/clinic	No	07/02/2017	0	0	0
2014288392	417	rural/clinic	No	07/02/2017	0	0	0
2015314301	417	rural/clinic	No	07/02/2017	0	0	0
2015303820	417	rural/clinic	No	07/02/2017	0	0	0
2015314306	417	rural/clinic	No	07/02/2017	0	0	0
2015303819	417	rural/clinic	No	07/02/2017	0	0	0
2015303818	417	rural/clinic	No	07/02/2017	0	0	0
2011212591	417	rural/clinic	No	07/02/2017	0	0	0
2015293924	417	rural/clinic	No	07/02/2017	0	0	0
2012261181	417	rural/clinic	No	07/02/2017	0	0	0
2015303817	417	rural/clinic	No	07/02/2017	0	0	0
2015344311	417	rural/clinic	No	07/02/2017	0	0	0
2015344312	417	rural/clinic	No	07/02/2017	0	0	0
2015334035	417	rural/clinic	No	07/02/2017	0	0	0
2015334074	417	rural/clinic	No	07/02/2017	0	0	0
2015334073	417	rural/clinic	No	07/02/2017	0	0	0
2015334069	417	rural/clinic	No	07/02/2017	0	0	0
2014314705	417	rural/clinic	No	07/02/2017	0	0	0
2014314706	417	rural/clinic	No	07/02/2017	0	0	0
2014314702	417	rural/clinic	No	07/02/2017	0	0	0
2011195780	417	rural/clinic	No	07/02/2017	0	0	0
2015319428	417	rural/clinic	No	07/02/2017	0	0	0
2012322686	417	rural/clinic	No	07/02/2017	0	0	0
2014360333	417	rural/clinic	No	07/02/2017	0	0	0
2014360334	417	rural/clinic	No	07/02/2017	0	0	0
2015322719	417	rural/clinic	No	07/02/2017	0	0	0
2014327657	417	rural/clinic	No	07/02/2017	0	0	0
2015322718	417	rural/clinic	No	07/02/2017	0	0	0
2014327658	417	rural/clinic	No	07/02/2017	0	0	0
2015322571	417	rural/clinic	No	07/02/2017	0	0	0
2015322572	417	rural/clinic	No	07/02/2017	0	0	0
2014327659	417	rural/clinic	No	07/02/2017	0	0	0
2015322088	417	rural/clinic	No	07/02/2017	0	0	0
2014327660	417	rural/clinic	No	07/02/2017	0	0	0
2014327661	417	rural/clinic	No	07/02/2017	0	0	0
2014327662	417	rural/clinic	No	07/02/2017	0	0	0
2014327663	417	rural/clinic	No	07/02/2017	0	0	0
2015293634	417	rural/clinic	No	07/02/2017	0	0	0
2015322087	417	rural/clinic	No	07/02/2017	0	0	0
2012320385	417	rural/clinic	No	07/02/2017	0	0	0
2015348885	417	rural/clinic	No	07/02/2017	0	0	0
2015344064	417	rural/clinic	No	07/02/2017	0	0	0
2015328510	417	rural/clinic	No	07/02/2017	0	0	0
2015344065	417	rural/clinic	No	07/02/2017	0	0	0
2015348883	417	rural/clinic	No	07/02/2017	0	0	0
2015344066	417	rural/clinic	No	07/02/2017	0	0	0
2015413682	417	rural/clinic	No	07/02/2017	0	0	0
2015416510	417	rural/clinic	No	07/02/2017	0	0	0
2015413683	417	rural/clinic	No	07/02/2017	0	0	0
2014372223	417	rural/clinic	No	07/02/2017	0	0	0
2015344724	417	rural/clinic	No	07/02/2017	0	0	0
2015319427	417	rural/clinic	No	07/02/2017	0	0	0
2015294616	417	rural/clinic	No	07/02/2017	0	0	0
2015294617	417	rural/clinic	No	07/02/2017	0	0	0
2015294618	417	rural/clinic	No	07/02/2017	0	0	0
2015294619	417	rural/clinic	No	07/02/2017	0	0	0
2015413578	417	rural/clinic	No	07/02/2017	0	0	0
2015416511	417	rural/clinic	No	07/02/2017	0	0	0
2015405092	417	rural/clinic	No	07/02/2017	0	0	0
2012390141	417	rural/clinic	No	07/02/2017	0	0	0
2015405089	417	rural/clinic	No	07/02/2017	0	0	0
2015405090	417	rural/clinic	No	07/02/2017	0	0	0
2012390139	417	rural/clinic	No	07/02/2017	0	0	0
2012390138	417	rural/clinic	No	07/02/2017	0	0	0
2015405091	417	rural/clinic	No	07/02/2017	0	0	0
2015334083	417	rural/clinic	No	07/02/2017	0	0	0
2015303474	417	rural/clinic	No	07/02/2017	0	0	0
2015334087	417	rural/clinic	No	07/02/2017	0	0	0
2015334084	417	rural/clinic	No	07/02/2017	0	0	0
2015334085	417	rural/clinic	No	07/02/2017	0	0	0
2015334077	417	rural/clinic	No	07/02/2017	0	0	0
2015334078	417	rural/clinic	No	07/02/2017	0	0	0
2015334075	417	rural/clinic	No	07/02/2017	0	0	0
2015334076	417	rural/clinic	No	07/02/2017	0	0	0
2015359392	417	rural/clinic	No	24/01/2017	0	0	0
2015359392	417	rural/clinic	No	24/01/2017	0	0	0

2015331874	417	rural/clinic	No	24/01/2017	0	0	0
2015331874	417	rural/clinic	No	24/01/2017	0	0	0
2015359202	417	rural/clinic	No	24/01/2017	0	0	0
2015359202	417	rural/clinic	No	24/01/2017	0	0	0
2015357359	417	rural/clinic	No	24/01/2017	0	0	0
2015357359	417	rural/clinic	No	24/01/2017	0	0	0
2012353600	417	rural/clinic	No	24/01/2017	0	0	0
2012353600	417	rural/clinic	No	24/01/2017	0	0	0
2012353596	417	rural/clinic	No	24/01/2017	0	0	0
2012353596	417	rural/clinic	No	24/01/2017	0	0	0
2012353597	417	rural/clinic	No	24/01/2017	0	0	0
2012353597	417	rural/clinic	No	24/01/2017	0	0	0
2015293827	417	rural/clinic	No	07/02/2017	0	0	0
2015344643	417	rural/clinic	No	07/02/2017	0	0	0
2015344644	417	rural/clinic	No	07/02/2017	0	0	0
2015355368	417	rural/clinic	No	19/01/2017	0	0	0
2015355368	417	rural/clinic	No	19/01/2017	0	0	0
2015344645	417	rural/clinic	No	07/02/2017	0	0	0
2015344646	417	rural/clinic	No	07/02/2017	0	0	0
2011195845	417	rural/clinic	No	07/02/2017	0	0	0
2015293633	417	rural/clinic	No	07/02/2017	0	0	0
2015293635	417	rural/clinic	No	07/02/2017	0	0	0
2017000000	417	rural/clinic	No	08/02/2017	0	0	0
2015373214	417	rural/clinic	No	26/01/2017	0	0	0
2015373214	417	rural/clinic	No	26/01/2017	0	0	0
2015373215	417	rural/clinic	No	26/01/2017	0	0	0
2015373215	417	rural/clinic	No	26/01/2017	0	0	0
2015384026	417	rural/clinic	No	26/01/2017	0	0	0
2015384026	417	rural/clinic	No	26/01/2017	0	0	0
2012253958	417	rural/clinic	No	26/01/2017	0	0	0
2012253958	417	rural/clinic	No	26/01/2017	0	0	0
2015375843	417	rural/clinic	No	26/01/2017	0	0	0
2015375843	417	rural/clinic	No	26/01/2017	0	0	0
2011113940	417	rural/clinic	No	26/01/2017	0	0	0
2011113940	417	rural/clinic	No	26/01/2017	0	0	0
2014287896	417	rural/clinic	No	26/01/2017	0	0	0
2014287896	417	rural/clinic	No	26/01/2017	0	0	0
2012350510	417	rural/clinic	No	03/01/2017	0	0	0
2011216823/D	417	rural/clinic	No	19/01/2017	0	0	0
2014328757/D	417	rural/clinic	No	19/01/2017	0	0	0
2015414309	417	rural/clinic	No	24/01/2017	0	0	0
2015334052	417	rural/clinic	No	13/01/2017	0	0	0
2015355370	417	rural/clinic	No	17/01/2017	0	0	0
2014317740	417	rural/clinic	No	19/01/2017	0	0	0
2015418175	417	rural/clinic	No	17/01/2017	0	0	0
2011233794	417	rural/clinic	No	19/01/2017	0	0	0
2013267199	417	rural/clinic	No	09/02/2017	0	0	0
2013267200	417	rural/clinic	No	09/02/2017	0	0	0
2015375850	296	rural/clinic	No	09/02/2017	0	0	0
2012259758	296	rural/clinic	No	09/02/2017	0	0	0
2012259759	296	rural/clinic	No	09/02/2017	0	0	0
2012262247	296	rural/clinic	No	09/02/2017	0	0	0
2015289831	296	rural/clinic	No	09/02/2017	0	0	0
2015289164	296	rural/clinic	No	09/02/2017	0	0	0
2014342786	296	rural/clinic	No	09/02/2017	0	0	0
2014350957	296	rural/clinic	No	09/02/2017	0	0	0
2014347981	296	rural/clinic	No	09/02/2017	0	0	0
2012390140	296	rural/clinic	No	09/02/2017	0	0	0
2012262056	296	rural/clinic	No	09/02/2017	0	0	0
2012390136	296	rural/clinic	No	09/02/2017	0	0	0
2015375849	296	rural/clinic	No	09/02/2017	0	0	0
2015375848	296	rural/clinic	No	09/02/2017	0	0	0
2012259757	759	rural/clinic	No	09/02/2017	0	0	0
2012265634	759	rural/clinic	No	09/02/2017	0	0	0
2015377257	759	rural/clinic	No	09/02/2017	0	0	0
2014326740	759	rural/clinic	No	09/02/2017	0	0	0
2015384028	759	rural/clinic	No	09/02/2017	0	0	0
2015405962	759	rural/clinic	No	09/02/2017	0	0	0
2015384027	759	rural/clinic	No	09/02/2017	0	0	0
2014346941	759	rural/clinic	No	09/02/2017	0	0	0
2014350429	759	rural/clinic	No	09/02/2017	0	0	0
2015363002	759	rural/clinic	No	09/02/2017	0	0	0
2014346940	759	rural/clinic	No	09/02/2017	0	0	0
2014346939	759	rural/clinic	No	09/02/2017	0	0	0
2014326736	759	rural/clinic	No	09/02/2017	0	0	0
2014346938	759	rural/clinic	No	09/02/2017	0	0	0
2013244109	759	rural/clinic	No	09/02/2017	0	0	0
2014326739	759	rural/clinic	No	09/02/2017	0	0	0
2015355903	759	rural/clinic	No	09/02/2017	0	0	0
2015355901	759	rural/clinic	No	09/02/2017	0	0	0
2015369375	759	rural/clinic	No	09/02/2017	0	0	0
2015301442	759	rural/clinic	No	09/02/2017	0	0	0

2015369376	759	rural/clinic	No	09/02/2017	0	0	0
2015369377	759	rural/clinic	No	09/02/2017	0	0	0
2015405613	759	rural/clinic	No	09/02/2017	0	0	0
2011192487	759	rural/clinic	No	09/02/2017	0	0	0
2015405612	759	rural/clinic	No	09/02/2017	0	0	0
2012306340	759	rural/clinic	No	09/02/2017	0	0	0
2011235849	759	rural/clinic	No	09/02/2017	0	0	0
2012384780	759	rural/clinic	No	09/02/2017	0	0	0
2012384779	759	rural/clinic	No	09/02/2017	0	0	0
2015405611	759	rural/clinic	No	09/02/2017	0	0	0
2015286522	524	rural/clinic	No	09/02/2017	0	0	0
2014365636	524	rural/clinic	No	09/02/2017	0	0	0
2015413324	524	rural/clinic	No	09/02/2017	0	0	0
2015335366	524	rural/clinic	No	09/02/2017	0	0	0
2015413325	524	rural/clinic	No	09/02/2017	0	0	0
2015335367	524	rural/clinic	No	09/02/2017	0	0	0
2015377012	524	rural/clinic	No	09/02/2017	0	0	0
2014365697	524	rural/clinic	No	09/02/2017	0	0	0
2015377013	524	rural/clinic	No	09/02/2017	0	0	0
2015405660	524	rural/clinic	No	09/02/2017	0	0	0
2015377014	524	rural/clinic	No	09/02/2017	0	0	0
2015413211	524	rural/clinic	No	09/02/2017	0	0	0
2015377015	524	rural/clinic	No	09/02/2017	0	0	0
2013264384	524	rural/clinic	No	09/02/2017	0	0	0
2013264385	524	rural/clinic	No	09/02/2017	0	0	0
2012290387	524	rural/clinic	No	09/02/2017	0	0	0
2014326737	524	rural/clinic	No	09/02/2017	0	0	0
2014326738	524	rural/clinic	No	09/02/2017	0	0	0
2011143195	524	rural/clinic	No	09/02/2017	0	0	0
2015377016	524	rural/clinic	No	09/02/2017	0	0	0
2011143194	524	rural/clinic	No	09/02/2017	0	0	0
2015363003	524	rural/clinic	No	09/02/2017	0	0	0
2014350679	524	rural/clinic	No	09/02/2017	0	0	0
2015377017	524	rural/clinic	No	09/02/2017	0	0	0
2011142906	524	rural/clinic	No	09/02/2017	0	0	0
2013264055	524	rural/clinic	No	09/02/2017	0	0	0
2015377018	524	rural/clinic	No	09/02/2017	0	0	0
2015379501	524	rural/clinic	No	09/02/2017	0	0	0
2015286272	524	rural/clinic	No	09/02/2017	0	0	0
2012390994	524	rural/clinic	No	09/02/2017	0	0	0
2015286273	524	rural/clinic	No	09/02/2017	0	0	0
2015384525	524	rural/clinic	No	09/02/2017	0	0	0
2015384526	524	rural/clinic	No	10/02/2017	0	0	0
2014385486	524	rural/clinic	No	09/02/2017	0	0	0
2011142905	524	rural/clinic	No	09/02/2017	0	0	0
2011123779	524	rural/clinic	No	09/02/2017	0	0	0
2012289597	524	rural/clinic	No	09/02/2017	0	0	0
2011183065	524	rural/clinic	No	09/02/2017	0	0	0
2014302510	524	rural/clinic	No	09/02/2017	0	0	0
2011188704	524	rural/clinic	No	09/02/2017	0	0	0
2014298034	524	rural/clinic	No	09/02/2017	0	0	0
2014302511	524	rural/clinic	No	09/02/2017	0	0	0
2014385487	524	rural/clinic	No	09/02/2017	0	0	0
2015351140	524	rural/clinic	No	09/02/2017	0	0	0
2014385488	524	rural/clinic	No	09/02/2017	0	0	0
2011203829	524	rural/clinic	No	09/02/2017	0	0	0
2014337039	524	rural/clinic	No	09/02/2017	0	0	0
2011203828	524	rural/clinic	No	09/02/2017	0	0	0
2012344075	524	rural/clinic	No	09/02/2017	0	0	0
2014337040	524	rural/clinic	No	09/02/2017	0	0	0
2015360090	524	rural/clinic	No	09/02/2017	0	0	0
2015355376	524	rural/clinic	No	09/02/2017	0	0	0
2015382871	524	rural/clinic	No	09/02/2017	0	0	0
2014340267	524	rural/clinic	No	09/02/2017	0	0	0
2015382870	524	rural/clinic	No	09/02/2017	0	0	0
2014378471	524	rural/clinic	No	09/02/2017	0	0	0
2014378472	524	rural/clinic	No	09/02/2017	0	0	0
2015382872	524	rural/clinic	No	09/02/2017	0	0	0
2014340266	524	rural/clinic	No	09/02/2017	0	0	0
2011124083	524	rural/clinic	No	09/02/2017	0	0	0
2015360091	524	rural/clinic	No	09/02/2017	0	0	0
2014340265	524	rural/clinic	No	09/02/2017	0	0	0
2014317744	524	rural/clinic	No	09/02/2017	0	0	0
2011203827	524	rural/clinic	No	09/02/2017	0	0	0
2014346891	524	rural/clinic	No	09/02/2017	0	0	0
2013262208	524	rural/clinic	No	09/02/2017	0	0	0
2014346892	524	rural/clinic	No	09/02/2017	0	0	0
2011141102	524	rural/clinic	No	09/02/2017	0	0	0
2013264671	524	rural/clinic	No	09/02/2017	0	0	0
2011141101	524	rural/clinic	No	09/02/2017	0	0	0
2013262207	524	rural/clinic	No	09/02/2017	0	0	0
2013262206	524	rural/clinic	No	09/02/2017	0	0	0

2013262205	524	rural/clinic	No	09/02/2017	0	0	0
2015331661	524	rural/clinic	No	09/02/2017	0	0	0
2014317428	444	rural/clinic	No	09/02/2017	0	0	0
2014317616	444	rural/clinic	No	09/02/2017	0	0	0
2015336878	444	rural/clinic	No	09/02/2017	0	0	0
2015344833	444	rural/clinic	No	09/02/2017	0	0	0
2015344834	444	rural/clinic	No	09/02/2017	0	0	0
2014357514	444	rural/clinic	No	09/02/2017	0	0	0
2014357516	444	rural/clinic	No	09/02/2017	0	0	0
2013252786	444	rural/clinic	No	09/02/2017	0	0	0
2014357512	444	rural/clinic	No	09/02/2017	0	0	0
2015294146	444	rural/clinic	No	09/02/2017	0	0	0
2014381899	444	rural/clinic	No	26/01/2017	0	0	0
2014381899	444	rural/clinic	No	26/01/2017	0	0	0
2014381898	444	rural/clinic	No	26/01/2017	0	0	0
2014381898	444	rural/clinic	No	26/01/2017	0	0	0
2015305236	444	rural/clinic	No	26/01/2017	0	0	0
2015305236	444	rural/clinic	No	26/01/2017	0	0	0
2012369963	444	rural/clinic	No	09/02/2017	0	0	0
2015352423	444	rural/clinic	No	09/02/2017	0	0	0
2015375953	444	rural/clinic	No	26/01/2017	0	0	0
2015375953	444	rural/clinic	No	26/01/2017	0	0	0
2015412072	444	rural/clinic	No	26/01/2017	0	0	0
2015412072	444	rural/clinic	No	26/01/2017	0	0	0
2015346367	444	rural/clinic	No	26/01/2017	0	0	0
2015346367	444	rural/clinic	No	26/01/2017	0	0	0
2012390134	444	rural/clinic	No	26/01/2017	0	0	0
2012390134	444	rural/clinic	No	26/01/2017	0	0	0
2015286858	444	rural/clinic	No	27/01/2017	0	0	0
2015286858	444	rural/clinic	No	27/01/2017	0	0	0
2012364934	444	rural/clinic	No	27/01/2017	0	0	0
2012364934	444	rural/clinic	No	27/01/2017	0	0	0
2015382861	444	rural/clinic	No	26/01/2017	0	0	0
2015382861	444	rural/clinic	No	26/01/2017	0	0	0
2015382864	444	rural/clinic	No	26/01/2017	0	0	0
2015382864	444	rural/clinic	No	26/01/2017	0	0	0
2015358473	444	rural/clinic	No	26/01/2017	0	0	0
2015358473	444	rural/clinic	No	26/01/2017	0	0	0
2014357513	444	rural/clinic	No	09/02/2017	0	0	0
2014321417	444	rural/clinic	No	27/01/2017	0	0	0
2014321417	444	rural/clinic	No	27/01/2017	0	0	0
2015365342	444	rural/clinic	No	31/01/2017	0	0	0
2015365342	444	rural/clinic	No	31/01/2017	0	0	0
2015365344	444	rural/clinic	No	31/01/2017	0	0	0
2015365344	444	rural/clinic	No	31/01/2017	0	0	0
2015418634	444	rural/clinic	No	31/01/2017	0	0	0
2015418634	444	rural/clinic	No	31/01/2017	0	0	0
2012328396	444	rural/clinic	No	31/01/2017	0	0	0
2012328396	444	rural/clinic	No	31/01/2017	0	0	0
2014357515	444	rural/clinic	No	09/02/2017	0	0	0
2014357511	444	rural/clinic	No	09/02/2017	0	0	0
2015352986	444	rural/clinic	No	26/01/2017	0	0	0
2015352986	444	rural/clinic	No	26/01/2017	0	0	0
2015352987	444	rural/clinic	No	26/01/2017	0	0	0
2015352987	444	rural/clinic	No	26/01/2017	0	0	0
2015313398	444	rural/clinic	No	09/02/2017	0	0	0
2015363001	444	rural/clinic	No	26/01/2017	0	0	0
2015363001	444	rural/clinic	No	26/01/2017	0	0	0
2015313397	444	rural/clinic	No	09/02/2017	0	0	0
2015313396	444	rural/clinic	No	09/02/2017	0	0	0
2015313395	444	rural/clinic	No	09/02/2017	0	0	0
2015352425	444	rural/clinic	No	09/02/2017	0	0	0
2015313394	444	rural/clinic	No	09/02/2017	0	0	0
2014303492	444	rural/clinic	No	09/02/2017	0	0	0
2015352424	444	rural/clinic	No	09/02/2017	0	0	0
2014335522	444	rural/clinic	No	09/02/2017	0	0	0
2014335523	444	rural/clinic	No	09/02/2017	0	0	0
2015375955	444	rural/clinic	No	09/02/2017	0	0	0
2012291835	444	rural/clinic	No	09/02/2017	0	0	0
2015408097	444	rural/clinic	No	09/02/2017	0	0	0
2015375954	492	rural/clinic	No	09/02/2017	0	0	0
2015338452	492	rural/clinic	No	09/02/2017	0	0	0
2015375956	492	rural/clinic	No	09/02/2017	0	0	0
2015346372	492	rural/clinic	No	09/02/2017	0	0	0
2015375957	492	rural/clinic	No	09/02/2017	0	0	0
2015346373	492	rural/clinic	No	09/02/2017	0	0	0
2015361785	492	rural/clinic	No	09/02/2017	0	0	0
2015368141	492	rural/clinic	No	09/02/2017	0	0	0
2015368142	492	rural/clinic	No	09/02/2017	0	0	0
2012340199	492	rural/clinic	No	09/02/2017	0	0	0
2015289827	492	rural/clinic	No	09/02/2017	0	0	0
2015331877	492	rural/clinic	No	09/02/2017	0	0	0

2012340200	492	rural/clinic	No	09/02/2017	0	0	0
2014298299	492	rural/clinic	No	09/02/2017	0	0	0
2011181147	492	rural/clinic	No	09/02/2017	0	0	0
2015368203	492	rural/clinic	No	09/02/2017	0	0	0
2014288166	492	rural/clinic	No	09/02/2017	0	0	0
2015368202	492	rural/clinic	No	09/02/2017	0	0	0
2015289828	492	rural/clinic	No	09/02/2017	0	0	0
2015368201	492	rural/clinic	No	09/02/2017	0	0	0
2015412074	492	rural/clinic	No	09/02/2017	0	0	0
2015412075	492	rural/clinic	No	09/02/2017	0	0	0
2014298300	492	rural/clinic	No	09/02/2017	0	0	0
2014288001	492	rural/clinic	No	09/02/2017	0	0	0
2015375958	492	rural/clinic	No	09/02/2017	0	0	0
2015351139	492	rural/clinic	No	09/02/2017	0	0	0
2013273394	492	rural/clinic	No	09/02/2017	0	0	0
2013264053	492	rural/clinic	No	09/02/2017	0	0	0
2013273395	492	rural/clinic	No	09/02/2017	0	0	0
2012291541	492	rural/clinic	No	09/02/2017	0	0	0
2015289165	492	rural/clinic	No	09/02/2017	0	0	0
2015377256	492	rural/clinic	No	09/02/2017	0	0	0
2011204472	492	rural/clinic	No	09/02/2017	0	0	0
2015414372	492	rural/clinic	No	09/02/2017	0	0	0
2013264054	492	rural/clinic	No	09/02/2017	0	0	0
2015291447	492	rural/clinic	No	09/02/2017	0	0	0
2014365635	492	rural/clinic	No	09/02/2017	0	0	0
2015382873	492	rural/clinic	No	09/02/2017	0	0	0
2015346222	492	rural/clinic	No	09/02/2017	0	0	0
2014350428	492	rural/clinic	No	09/02/2017	0	0	0
2015346223	492	rural/clinic	No	09/02/2017	0	0	0
2015346221	492	rural/clinic	No	09/02/2017	0	0	0
2015288578	492	rural/clinic	No	09/02/2017	0	0	0
2014305214	492	rural/clinic	No	09/02/2017	0	0	0
2014305213	492	rural/clinic	No	09/02/2017	0	0	0
2015333251	492	rural/clinic	No	09/02/2017	0	0	0
2014377400	492	rural/clinic	No	09/02/2017	0	0	0
2015333951	492	rural/clinic	No	09/02/2017	0	0	0
2014318839	492	rural/clinic	No	09/02/2017	0	0	0
2014318842	493	provincial	No	09/02/2017	0	1	0
2014318841	493	provincial	No	09/02/2017	0	1	0
2014318840	493	provincial	No	09/02/2017	0	1	0
2014318838	493	provincial	No	09/02/2017	0	1	0
2014318836	493	provincial	No	09/02/2017	0	1	0
2014318835	493	provincial	No	09/02/2017	0	1	0
2014318837	493	provincial	No	09/02/2017	0	1	0
2015397772	493	provincial	No	09/02/2017	0	1	0
2014330346	493	provincial	No	09/02/2017	0	1	0
2015372909	493	provincial	No	09/02/2017	0	1	0
2011141550	493	provincial	No	09/02/2017	0	1	0
2011141547	493	provincial	No	09/02/2017	0	1	0
2014289483	493	provincial	No	09/02/2017	0	1	0
2014289484	493	provincial	No	09/02/2017	0	1	0
2014289486	493	provincial	No	09/02/2017	0	1	0
2014289485	493	provincial	No	09/02/2017	0	1	0
2014289477	493	provincial	No	09/02/2017	0	1	0
2015372018	493	provincial	No	09/02/2017	0	1	0
2015358134	493	provincial	No	09/02/2017	0	1	0
2015358133	493	provincial	No	09/02/2017	0	1	0
2012246281	493	provincial	No	09/02/2017	0	1	0
2013258725	493	provincial	No	09/02/2017	0	1	0
2014335220	493	provincial	No	09/02/2017	0	1	0
2014335219	493	provincial	No	09/02/2017	0	1	0
2014361045	493	provincial	No	09/02/2017	0	1	0
2015401544	493	provincial	No	09/02/2017	0	1	0
2015401546	493	provincial	No	09/02/2017	0	1	0
2015340477	493	provincial	No	09/02/2017	0	1	0
2015340478	493	provincial	No	09/02/2017	0	1	0
2015340479	493	provincial	No	09/02/2017	0	1	0
2015305242	493	provincial	No	09/02/2017	0	1	0
2015305243	493	provincial	No	09/02/2017	0	1	0
2015373234	493	provincial	No	09/02/2017	0	1	0
2015373233	493	provincial	No	09/02/2017	0	1	0
2015358129	493	provincial	No	09/02/2017	0	1	0
2015373232	493	provincial	No	09/02/2017	0	1	0
2015358132	493	provincial	No	09/02/2017	0	1	0
2015373231	493	provincial	No	09/02/2017	0	1	0
2015358130	493	provincial	No	09/02/2017	0	1	0
2015358131	493	provincial	No	09/02/2017	0	1	0
2015373230	493	provincial	No	09/02/2017	0	1	0
2014386139	493	provincial	No	09/02/2017	0	1	0
2015373339	493	provincial	No	09/02/2017	0	1	0
2014386138	493	provincial	No	09/02/2017	0	1	0
2015373340	493	provincial	No	09/02/2017	0	1	0

2015373386	493	provincial	No	09/02/2017	0	1	0
2015373385	493	provincial	No	09/02/2017	0	1	0
2014386137	493	provincial	No	09/02/2017	0	1	0
2015373384	493	provincial	No	09/02/2017	0	1	0
2014386136	493	provincial	No	09/02/2017	0	1	0
2015373383	493	provincial	No	09/02/2017	0	1	0
2014386135	493	provincial	No	09/02/2017	0	1	0
2015373382	493	provincial	No	09/02/2017	0	1	0
2014386134	493	provincial	No	09/02/2017	0	1	0
2015369491	493	provincial	No	09/02/2017	0	1	0
2014386133	493	provincial	No	09/02/2017	0	1	0
2014386140	493	provincial	No	09/02/2017	0	1	0
2015369490	493	provincial	No	09/02/2017	0	1	0
2014386141	493	provincial	No	09/02/2017	0	1	0
2015369489	493	provincial	No	09/02/2017	0	1	0
2014386142	493	provincial	No	09/02/2017	0	1	0
2014386143	493	provincial	No	09/02/2017	0	1	0
2014386144	493	provincial	No	09/02/2017	0	1	0
2015369488	493	provincial	No	09/02/2017	0	1	0
2015402290	493	provincial	No	09/02/2017	0	1	0
2015369487	493	provincial	No	09/02/2017	0	1	0
2015402289	493	provincial	No	09/02/2017	0	1	0
2015402288	493	provincial	No	09/02/2017	0	1	0
2015402287	493	provincial	No	09/02/2017	0	1	0
2015402286	493	provincial	No	09/02/2017	0	1	0
2015402285	493	provincial	No	09/02/2017	0	1	0
2015402284	493	provincial	No	09/02/2017	0	1	0
2014303053	493	provincial	No	09/02/2017	0	1	0
2015358284	493	provincial	No	09/02/2017	0	1	0
2015358285	493	provincial	No	09/02/2017	0	1	0
2015358286	493	provincial	No	09/02/2017	0	1	0
2015358283	493	provincial	No	09/02/2017	0	1	0
2015358282	493	provincial	No	09/02/2017	0	1	0
2015358281	493	provincial	No	09/02/2017	0	1	0
2014303054	493	provincial	No	09/02/2017	0	1	0
2015358128	493	provincial	No	09/02/2017	0	1	0
2014303055	493	provincial	No	09/02/2017	0	1	0
2015358127	493	provincial	No	09/02/2017	0	1	0
2015357628	493	provincial	No	09/02/2017	0	1	0
2011235743	493	provincial	No	09/02/2017	0	1	0
2012306446	493	provincial	No	09/02/2017	0	1	0
2012339732	493	provincial	No	09/02/2017	0	1	0
2015301441	493	provincial	No	09/02/2017	0	1	0
2011200075	493	provincial	No	09/02/2017	0	1	0
2014289482	493	provincial	No	09/02/2017	0	1	0
2015357629	493	provincial	No	09/02/2017	0	1	0
2011236077	493	provincial	No	09/02/2017	0	1	0
2011236078	493	provincial	No	09/02/2017	0	1	0
2014316762	493	provincial	No	10/02/2017	0	1	0
2014316761	493	provincial	No	10/02/2017	0	1	0
2014316759	493	provincial	No	10/02/2017	0	1	0
2014316760	493	provincial	No	10/02/2017	0	1	0
2015319430	493	provincial	No	10/02/2017	0	1	0
2015319429	493	provincial	No	10/02/2017	0	1	0
2011196006	493	provincial	No	10/02/2017	0	1	0
2011196005	493	provincial	No	10/02/2017	0	1	0
2011196007	493	provincial	No	10/02/2017	0	1	0
2015373229	493	provincial	No	10/02/2017	0	1	0
2015382363	493	provincial	No	10/02/2017	0	1	0
2015382364	493	provincial	No	10/02/2017	0	1	0
2015322717	493	provincial	No	10/02/2017	0	1	0
2011196004	493	provincial	No	10/02/2017	0	1	0
2011196008	493	provincial	No	10/02/2017	0	1	0
2014287409	493	provincial	No	10/02/2017	0	1	0
2012349532	493	provincial	No	10/02/2017	0	1	0
2015248886	493	provincial	No	10/02/2017	0	1	0
2015328511	493	provincial	No	10/02/2017	0	1	0
2015348887	493	provincial	No	10/02/2017	0	1	0
2014316758	493	provincial	No	10/02/2017	0	1	0
2012349533	493	provincial	No	10/02/2017	0	1	0
2015397494	493	provincial	No	13/02/2017	0	1	0
2015397495	493	provincial	No	13/02/2017	0	1	0
2015397496	493	provincial	No	13/02/2017	0	1	0
2015397497	493	provincial	No	13/02/2017	0	1	0
2015365058	493	provincial	No	26/01/2017	0	1	0
2015365058	493	provincial	No	26/01/2017	0	1	0
2015397498	493	provincial	No	13/02/2017	0	1	0
2011233795	493	provincial	No	31/01/2017	0	1	0
2011233795	493	provincial	No	31/01/2017	0	1	0
2011233797	493	provincial	No	31/01/2017	0	1	0
2011233797	493	provincial	No	31/01/2017	0	1	0
2015397499	493	provincial	No	13/02/2017	0	1	0

2015338451	493	provincial	No	31/01/2017	0	1	0
2015338451	493	provincial	No	31/01/2017	0	1	0
2014329236	493	provincial	No	31/01/2017	0	1	0
2014329236	493	provincial	No	31/01/2017	0	1	0
2014329237	493	provincial	No	31/01/2017	0	1	0
2014329237	493	provincial	No	31/01/2017	0	1	0
2015397500	493	provincial	No	13/02/2017	0	1	0
2017011	493	provincial	No	26/01/2017	0	1	0
2015402283	493	provincial	No	26/01/2017	0	1	0
20170111	493	provincial	No	24/01/2017	0	1	0
201701111	493	provincial	No	24/01/2017	0	1	0
2017011111	493	provincial	No	24/01/2017	0	1	0
20170111111	493	provincial	No	24/01/2017	0	1	0
2.02E+11	493	provincial	No	24/01/2017	0	1	0
2012242575	493	provincial	No	14/02/2017	0	1	0
2012284738	493	provincial	No	14/02/2017	0	1	0
2015413092	493	provincial	No	14/02/2017	0	1	0
2015413091	493	provincial	No	14/02/2017	0	1	0
2015413090	493	provincial	No	14/02/2017	0	1	0
2015413089	493	provincial	No	14/02/2017	0	1	0
2015413085	493	provincial	No	14/02/2017	0	1	0
2015413086	493	provincial	No	14/02/2017	0	1	0
2015413087	493	provincial	No	14/02/2017	0	1	0
2015413088	493	provincial	No	14/02/2017	0	1	0
2015319579	493	provincial	No	14/02/2017	0	1	0
2015319574	493	provincial	No	14/02/2017	0	1	0
2015319573	493	provincial	No	14/02/2017	0	1	0
2012307509	493	provincial	No	31/01/2017	0	1	0
2012307509	493	provincial	No	31/01/2017	0	1	0
2012328391	493	provincial	No	31/01/2017	0	1	0
2012328391	493	provincial	No	31/01/2017	0	1	0
2015295340	493	provincial	No	31/01/2017	0	1	0
2015295340	493	provincial	No	31/01/2017	0	1	0
2012328398	493	provincial	No	31/01/2017	0	1	0
2012328398	493	provincial	No	31/01/2017	0	1	0
2012328397	493	provincial	No	31/01/2017	0	1	0
2012328397	493	provincial	No	31/01/2017	0	1	0
2015295333	493	provincial	No	31/01/2017	0	1	0
2015295333	493	provincial	No	31/01/2017	0	1	0
2015295335	493	provincial	No	31/01/2017	0	1	0
2015295335	493	provincial	No	31/01/2017	0	1	0
2015319572	493	provincial	No	14/02/2017	0	1	0
2015362331	493	provincial	No	31/01/2017	0	1	0
2015362331	493	provincial	No	31/01/2017	0	1	0
2015362332	493	provincial	No	31/01/2017	0	1	0
2015362332	493	provincial	No	31/01/2017	0	1	0
2015369476	493	provincial	No	26/01/2017	0	1	0
2015369476	493	provincial	No	26/01/2017	0	1	0
2015358475	493	provincial	No	26/01/2017	0	1	0
2015358475	493	provincial	No	26/01/2017	0	1	0
2015319571	493	provincial	No	14/02/2017	0	1	0
2015319577	493	provincial	No	14/02/2017	0	1	0
2015319575	493	provincial	No	14/02/2017	0	1	0
2015319576	493	provincial	No	14/02/2017	0	1	0
2015413093	493	provincial	No	14/02/2017	0	1	0
2014302198	493	provincial	No	14/02/2017	0	1	0
2014302199	493	provincial	No	14/02/2017	0	1	0
2012361943	493	provincial	No	14/02/2017	0	1	0
2012352342	77	rural/clinic	No	14/02/2017	0	0	0
2012267310	77	rural/clinic	No	14/02/2017	0	0	0
2012267311	77	rural/clinic	No	14/02/2017	0	0	0
2012267312	77	rural/clinic	No	14/02/2017	0	0	0
2013266065	77	rural/clinic	No	14/02/2017	0	0	0
2015377609	77	rural/clinic	No	14/02/2017	0	0	0
2015377610	77	rural/clinic	No	14/02/2017	0	0	0
2015377611	77	rural/clinic	No	14/02/2017	0	0	0
2015377612	77	rural/clinic	No	14/02/2017	0	0	0
2015377613	77	rural/clinic	No	14/02/2017	0	0	0
2015377614	77	rural/clinic	No	14/02/2017	0	0	0
2013271691	77	rural/clinic	No	14/02/2017	0	0	0
2014287969	77	rural/clinic	No	14/02/2017	0	0	0
2011128278	77	rural/clinic	No	14/02/2017	0	0	0
2012305967	77	rural/clinic	No	14/02/2017	0	0	0
2015300665	77	rural/clinic	No	14/02/2017	0	0	0
2014334343	77	rural/clinic	No	14/02/2017	0	0	0
2015300668	77	rural/clinic	No	14/02/2017	0	0	0
2013271692	77	rural/clinic	No	14/02/2017	0	0	0
2012263739	77	rural/clinic	No	14/02/2017	0	0	0
2015300667	77	rural/clinic	No	14/02/2017	0	0	0
2012263738	77	rural/clinic	No	14/02/2017	0	0	0
2015300666	77	rural/clinic	No	14/02/2017	0	0	0
2015414644	77	rural/clinic	No	14/02/2017	0	0	0

2014358179	77	rural/clinic	No	14/02/2017	0	0	0
2014358178	77	rural/clinic	No	14/02/2017	0	0	0
2015414785	77	rural/clinic	No	14/02/2017	0	0	0
2013249486	77	rural/clinic	No	14/02/2017	0	0	0
2012312847	77	rural/clinic	No	14/02/2017	0	0	0
2013249485	77	rural/clinic	No	14/02/2017	0	0	0
2015359751	77	rural/clinic	No	14/02/2017	0	0	0
2015342008	77	rural/clinic	No	14/02/2017	0	0	0
2014356845	77	rural/clinic	No	14/02/2017	0	0	0
2012244278	77	rural/clinic	No	14/02/2017	0	0	0
2012244277	77	rural/clinic	No	14/02/2017	0	0	0
2015418639	77	rural/clinic	No	14/02/2017	0	0	0
2015418636	77	rural/clinic	No	14/02/2017	0	0	0
2014291614	77	rural/clinic	No	14/02/2017	0	0	0
2015337352	77	rural/clinic	No	14/02/2017	0	0	0
2015338706	236	rural/clinic	No	14/02/2017	0	0	0
2015338704	236	rural/clinic	No	14/02/2017	0	0	0
2015338705	236	rural/clinic	No	14/02/2017	0	0	0
2014291579	236	rural/clinic	No	14/02/2017	0	0	0
2014291580	236	rural/clinic	No	14/02/2017	0	0	0
2015337354	236	rural/clinic	No	14/02/2017	0	0	0
2015337353	236	rural/clinic	No	14/02/2017	0	0	0
2014355897	236	rural/clinic	No	14/02/2017	0	0	0
2015337022	236	rural/clinic	No	14/02/2017	0	0	0
2015337023	236	rural/clinic	No	14/02/2017	0	0	0
2015337024	236	rural/clinic	No	14/02/2017	0	0	0
2015418119	236	rural/clinic	No	14/02/2017	0	0	0
2014342504	236	rural/clinic	No	14/02/2017	0	0	0
2015418117	236	rural/clinic	No	14/02/2017	0	0	0
2012242576	236	rural/clinic	No	14/02/2017	0	0	0
2015362344	236	rural/clinic	No	14/02/2017	0	0	0
2015362343	236	rural/clinic	No	14/02/2017	0	0	0
2015362342	236	rural/clinic	No	14/02/2017	0	0	0
2015362341	236	rural/clinic	No	14/02/2017	0	0	0
2015362340	236	rural/clinic	No	14/02/2017	0	0	0
2015362339	236	rural/clinic	No	14/02/2017	0	0	0
2015337025	236	rural/clinic	No	14/02/2017	0	0	0
2015362338	236	rural/clinic	No	14/02/2017	0	0	0
2015337026	236	rural/clinic	No	14/02/2017	0	0	0
2015362337	236	rural/clinic	No	14/02/2017	0	0	0
2012365144	236	rural/clinic	No	14/02/2017	0	0	0
2015385935	236	rural/clinic	No	14/02/2017	0	0	0
2012365143	236	rural/clinic	No	14/02/2017	0	0	0
2012259760	236	rural/clinic	No	14/02/2017	0	0	0
2011156599	236	rural/clinic	No	14/02/2017	0	0	0
2011156600	236	rural/clinic	No	14/02/2017	0	0	0
2012323775	236	rural/clinic	No	14/02/2017	0	0	0
2015315975	236	rural/clinic	No	14/02/2017	0	0	0
2014291581	236	rural/clinic	No	14/02/2017	0	0	0
2015385384	236	rural/clinic	No	14/02/2017	0	0	0
2015337018	236	rural/clinic	No	14/02/2017	0	0	0
2015337019	236	rural/clinic	No	14/02/2017	0	0	0
2015337020	236	rural/clinic	No	14/02/2017	0	0	0
2015337611	236	rural/clinic	No	14/02/2017	0	0	0
2015337610	236	rural/clinic	No	14/02/2017	0	0	0
2015337021	236	rural/clinic	No	14/02/2017	0	0	0
2015324463	236	rural/clinic	No	14/02/2017	0	0	0
2015324462	236	rural/clinic	No	14/02/2017	0	0	0
2015385931	236	rural/clinic	No	14/02/2017	0	0	0
2015340824	236	rural/clinic	No	31/01/2017	0	0	0
2015340824	236	rural/clinic	No	31/01/2017	0	0	0
2015385349	236	rural/clinic	No	31/01/2017	0	0	0
2015385349	236	rural/clinic	No	31/01/2017	0	0	0
2015317598	236	rural/clinic	No	31/01/2017	0	0	0
2015317598	236	rural/clinic	No	31/01/2017	0	0	0
2015385932	236	rural/clinic	No	14/02/2017	0	0	0
2012322906	236	rural/clinic	No	31/01/2017	0	0	0
2012322906	236	rural/clinic	No	31/01/2017	0	0	0
2015385934	236	rural/clinic	No	14/02/2017	0	0	0
2015385933	236	rural/clinic	No	14/02/2017	0	0	0
2012323774	236	rural/clinic	No	14/02/2017	0	0	0
2015337017	236	rural/clinic	No	14/02/2017	0	0	0
2015383582	236	rural/clinic	No	14/02/2017	0	0	0
2015383581	236	rural/clinic	No	14/02/2017	0	0	0
2015383583	236	rural/clinic	No	14/02/2017	0	0	0
2015383580	236	rural/clinic	No	14/02/2017	0	0	0
2014382912	236	rural/clinic	No	14/02/2017	0	0	0
2015297357	236	rural/clinic	No	14/02/2017	0	0	0
2015301090	236	rural/clinic	No	14/02/2017	0	0	0
2015412996	236	rural/clinic	No	14/02/2017	0	0	0
2014377406	236	rural/clinic	No	14/02/2017	0	0	0
2014370424	236	rural/clinic	No	14/02/2017	0	0	0

2015383579	236	rural/clinic	No	14/02/2017	0	0	0
2014328394	236	rural/clinic	No	14/02/2017	0	0	0
2015351141	236	rural/clinic	No	14/02/2017	0	0	0
2015301087	236	rural/clinic	No	14/02/2017	0	0	0
2011240296	236	rural/clinic	No	14/02/2017	0	0	0
2011240298	236	rural/clinic	No	14/02/2017	0	0	0
2011240299	236	rural/clinic	No	14/02/2017	0	0	0
2015301086	236	rural/clinic	No	14/02/2017	0	0	0
2015377857	55	rural/clinic	No	14/02/2017	0	0	0
2015326270	55	rural/clinic	No	14/02/2017	0	0	0
2015377858	55	rural/clinic	No	14/02/2017	0	0	0
2015326272	55	rural/clinic	No	14/02/2017	0	0	0
2015301089	55	rural/clinic	No	14/02/2017	0	0	0
2015326274	55	rural/clinic	No	14/02/2017	0	0	0
2015297356	55	rural/clinic	No	14/02/2017	0	0	0
2015326276	55	rural/clinic	No	14/02/2017	0	0	0
2014308887	55	rural/clinic	No	14/02/2017	0	0	0
2015326277	55	rural/clinic	No	14/02/2017	0	0	0
2013261290	55	rural/clinic	No	14/02/2017	0	0	0
2015326279	55	rural/clinic	No	14/02/2017	0	0	0
2015413356	55	rural/clinic	No	14/02/2017	0	0	0
2014365375	55	rural/clinic	No	14/02/2017	0	0	0
2015326280	55	rural/clinic	No	14/02/2017	0	0	0
2015326283	55	rural/clinic	No	14/02/2017	0	0	0
2015326284	55	rural/clinic	No	14/02/2017	0	0	0
2015326286	55	rural/clinic	No	14/02/2017	0	0	0
2015326287	55	rural/clinic	No	14/02/2017	0	0	0
2015326288	55	rural/clinic	No	14/02/2017	0	0	0
2015326289	55	rural/clinic	No	14/02/2017	0	0	0
2015326290	55	rural/clinic	No	14/02/2017	0	0	0
2015326293	55	rural/clinic	No	14/02/2017	0	0	0
2015326294	55	rural/clinic	No	14/02/2017	0	0	0
2015326507	55	rural/clinic	No	14/02/2017	0	0	0
2015326508	55	rural/clinic	No	14/02/2017	0	0	0
2014375653	55	rural/clinic	No	14/02/2017	0	0	0
2014375652	55	rural/clinic	No	14/02/2017	0	0	0
2014375654	55	rural/clinic	No	14/02/2017	0	0	0
2014291812	55	rural/clinic	No	14/02/2017	0	0	0
2014375656	55	rural/clinic	No	14/02/2017	0	0	0
2014375655	55	rural/clinic	No	14/02/2017	0	0	0
2012330879	55	rural/clinic	No	14/02/2017	0	0	0
2014363224	55	rural/clinic	No	14/02/2017	0	0	0
2015405963	55	rural/clinic	No	14/02/2017	0	0	0
2014350361	55	rural/clinic	No	14/02/2017	0	0	0
2015405408	55	rural/clinic	No	14/02/2017	0	0	0
2015414313	55	rural/clinic	No	14/02/2017	0	0	0
2015414314	55	rural/clinic	No	14/02/2017	0	0	0
2015414312	55	rural/clinic	No	14/02/2017	0	0	0
2015414315	55	rural/clinic	No	14/02/2017	0	0	0
2015414311	55	rural/clinic	No	14/02/2017	0	0	0
2015413978	55	rural/clinic	No	14/02/2017	0	0	0
2015413977	55	rural/clinic	No	14/02/2017	0	0	0
2015376101	55	rural/clinic	No	14/02/2017	0	0	0
2014329243	55	rural/clinic	No	14/02/2017	0	0	0
2014329241	55	rural/clinic	No	14/02/2017	0	0	0
2014329249	55	rural/clinic	No	14/02/2017	0	0	0
2014329242	55	rural/clinic	No	14/02/2017	0	0	0
2014329246	55	rural/clinic	No	14/02/2017	0	0	0
2014329244	55	rural/clinic	No	14/02/2017	0	0	0
2014347815	55	rural/clinic	No	14/02/2017	0	0	0
2015405409	55	rural/clinic	No	14/02/2017	0	0	0
2015369723	55	rural/clinic	No	14/02/2017	0	0	0
2015369722	55	rural/clinic	No	14/02/2017	0	0	0
2015369725	55	rural/clinic	No	14/02/2017	0	0	0
2014298235	55	rural/clinic	No	14/02/2017	0	0	0
2014298234	55	rural/clinic	No	14/02/2017	0	0	0
2015369721	55	rural/clinic	No	14/02/2017	0	0	0
2014298236	55	rural/clinic	No	14/02/2017	0	0	0
2015369720	55	rural/clinic	No	14/02/2017	0	0	0
2015414930	55	rural/clinic	No	14/02/2017	0	0	0
2015414929	55	rural/clinic	No	14/02/2017	0	0	0
2015414927	55	rural/clinic	No	14/02/2017	0	0	0
2015414928	55	rural/clinic	No	14/02/2017	0	0	0
2015351142	55	rural/clinic	No	14/02/2017	0	0	0
2015418118	55	rural/clinic	No	14/02/2017	0	0	0
2014342503	55	rural/clinic	No	14/02/2017	0	0	0
2012349254	55	rural/clinic	No	31/01/2017	0	0	0
2014314451	55	rural/clinic	No	15/02/2017	0	0	0
2014314452	55	rural/clinic	No	15/02/2017	0	0	0
2013256391	55	rural/clinic	No	15/02/2017	0	0	0
2013256390	55	rural/clinic	No	15/02/2017	0	0	0
2013256392	55	rural/clinic	No	15/02/2017	0	0	0

2015385600	55	rural/clinic	No	15/02/2017	0	0	0
2015358471	55	rural/clinic	No	26/01/2017	0	0	0
2015358471	55	rural/clinic	No	26/01/2017	0	0	0
2013266999	55	rural/clinic	No	26/01/2017	0	0	0
2013266999	55	rural/clinic	No	26/01/2017	0	0	0
2015358463	55	rural/clinic	No	26/01/2017	0	0	0
2015358463	55	rural/clinic	No	26/01/2017	0	0	0
2013256380	55	rural/clinic	No	15/02/2017	0	0	0
2014358176	55	rural/clinic	No	31/01/2017	0	0	0
2014358176	55	rural/clinic	No	31/01/2017	0	0	0
2012276143	55	rural/clinic	No	31/01/2017	0	0	0
2012276143	55	rural/clinic	No	31/01/2017	0	0	0
2013256381	55	rural/clinic	No	15/02/2017	0	0	0
2015358478	55	rural/clinic	No	26/01/2017	0	0	0
2015358478	55	rural/clinic	No	26/01/2017	0	0	0
2014345837	55	rural/clinic	No	31/01/2017	0	0	0
2014345837	55	rural/clinic	No	31/01/2017	0	0	0
2013256382	55	rural/clinic	No	15/02/2017	0	0	0
2014314454	55	rural/clinic	No	15/02/2017	0	0	0
2013256394	55	rural/clinic	No	15/02/2017	0	0	0
2014314455	55	rural/clinic	No	15/02/2017	0	0	0
2014314453	55	rural/clinic	No	15/02/2017	0	0	0
2013256386	55	rural/clinic	No	15/02/2017	0	0	0
2013256385	55	rural/clinic	No	15/02/2017	0	0	0
2013256384	55	rural/clinic	No	15/02/2017	0	0	0
2013256383	55	rural/clinic	No	15/02/2017	0	0	0
2015392430	55	rural/clinic	No	15/02/2017	0	0	0
2015392431	55	rural/clinic	No	15/02/2017	0	0	0
2015392432	55	rural/clinic	No	15/02/2017	0	0	0
2015373247	55	rural/clinic	No	15/02/2017	0	0	0
2012284024	55	rural/clinic	No	15/02/2017	0	0	0
2015378947	55	rural/clinic	No	16/02/2017	0	0	0
2013255302	55	rural/clinic	No	16/02/2017	0	0	0
2015352352	55	rural/clinic	No	15/02/2017	0	0	0
2015390945	55	rural/clinic	No	15/02/2017	0	0	0
2015390944	55	rural/clinic	No	15/02/2017	0	0	0
2015373238	55	rural/clinic	No	15/02/2017	0	0	0
2015390947	55	rural/clinic	No	15/02/2017	0	0	0
2015373237	55	rural/clinic	No	15/02/2017	0	0	0
2012291544	55	rural/clinic	No	15/02/2017	0	0	0
2015373236	55	rural/clinic	No	15/02/2017	0	0	0
2015344068	55	rural/clinic	No	16/02/2017	0	0	0
2015373246	55	rural/clinic	No	16/02/2017	0	0	0
2015334601	55	rural/clinic	No	16/02/2017	0	0	0
2012291543	55	rural/clinic	No	15/02/2017	0	0	0
2012291542	55	rural/clinic	No	15/02/2017	0	0	0
2015377423	55	rural/clinic	No	16/02/2017	0	0	0
2015331760	55	rural/clinic	No	14/02/2017	0	0	0
2012340787	55	rural/clinic	No	15/02/2017	0	0	0
2015385512	55	rural/clinic	No	16/02/2017	0	0	0
2012280594	55	rural/clinic	No	15/02/2017	0	0	0
2015352353	55	rural/clinic	No	15/02/2017	0	0	0
2015331759	55	rural/clinic	No	14/02/2017	0	0	0
2014346942	55	rural/clinic	No	16/02/2017	0	0	0
2013249278	55	rural/clinic	No	14/02/2017	0	0	0
2012350312	55	rural/clinic	No	15/02/2017	0	0	0
2014385615	55	rural/clinic	No	16/02/2017	0	0	0
2013249487	276	rural/clinic	No	14/02/2017	0	0	0
2015311304	276	rural/clinic	No	15/02/2017	0	0	0
2015359835	276	rural/clinic	No	14/02/2017	0	0	0
2014338248	276	rural/clinic	No	16/02/2017	0	0	0
2012350313	276	rural/clinic	No	15/02/2017	0	0	0
2015359834	276	rural/clinic	No	14/02/2017	0	0	0
2012291545	276	rural/clinic	No	16/02/2017	0	0	0
2015335418	276	rural/clinic	No	14/02/2017	0	0	0
2011213998	276	rural/clinic	No	15/02/2017	0	0	0
2014372268	276	rural/clinic	No	16/02/2017	0	0	0
201079326	276	rural/clinic	No	15/02/2017	0	0	0
2014343866	276	rural/clinic	No	14/02/2017	0	0	0
2015311305	276	rural/clinic	No	15/02/2017	0	0	0
2013255445	276	rural/clinic	No	16/02/2017	0	0	0
2013255444	276	rural/clinic	No	16/02/2017	0	0	0
2015311303	276	rural/clinic	No	15/02/2017	0	0	0
2014372267	276	rural/clinic	No	16/02/2017	0	0	0
2012350311	276	rural/clinic	No	15/02/2017	0	0	0
2012350318	276	rural/clinic	No	15/02/2017	0	0	0
2012350315	276	rural/clinic	No	15/02/2017	0	0	0
2013263588	276	rural/clinic	No	16/02/2017	0	0	0
2012317312	276	rural/clinic	No	14/02/2017	0	0	0
2013263587	276	rural/clinic	No	16/02/2017	0	0	0
2011213997	276	rural/clinic	No	15/02/2017	0	0	0
2015335572	276	rural/clinic	No	14/02/2017	0	0	0

2013263589	276	rural/clinic	No	16/02/2017	0	0	0
2015356570	276	rural/clinic	No	15/02/2017	0	0	0
2015394619	276	rural/clinic	No	15/02/2017	0	0	0
2015369381	276	rural/clinic	No	16/02/2017	0	0	0
2015356565	276	rural/clinic	No	15/02/2017	0	0	0
2015369380	276	rural/clinic	No	16/02/2017	0	0	0
2012300833	276	rural/clinic	No	15/02/2017	0	0	0
2015369378	276	rural/clinic	No	16/02/2017	0	0	0
2015392434	276	rural/clinic	No	15/02/2017	0	0	0
2015369379	276	rural/clinic	No	16/02/2017	0	0	0
2015392435	276	rural/clinic	No	15/02/2017	0	0	0
2013270346	276	rural/clinic	No	16/02/2017	0	0	0
2013254663	276	rural/clinic	No	15/02/2017	0	0	0
2015368264	276	rural/clinic	No	16/02/2017	0	0	0
2012328993	276	rural/clinic	No	15/02/2017	0	0	0
2015368143	276	rural/clinic	No	16/02/2017	0	0	0
2015392440	276	rural/clinic	No	15/02/2017	0	0	0
2013254662	276	rural/clinic	No	15/02/2017	0	0	0
2013274237	276	rural/clinic	No	16/02/2017	0	0	0
2012328400	276	rural/clinic	No	15/02/2017	0	0	0
2011198685	276	rural/clinic	No	16/02/2017	0	0	0
2014353621	276	rural/clinic	No	15/02/2017	0	0	0
2013251311	276	rural/clinic	No	15/02/2017	0	0	0
2015289166	276	rural/clinic	No	16/02/2017	0	0	0
2015319589	276	rural/clinic	No	16/02/2017	0	0	0
2014353623	276	rural/clinic	No	15/02/2017	0	0	0
2015286276	276	rural/clinic	No	16/02/2017	0	0	0
2015286274	276	rural/clinic	No	16/02/2017	0	0	0
2015286277	276	rural/clinic	No	16/02/2017	0	0	0
2015286275	276	rural/clinic	No	16/02/2017	0	0	0
2015340482	276	rural/clinic	No	16/02/2017	0	0	0
2015332702	276	rural/clinic	No	16/02/2017	0	0	0
2015340480	276	rural/clinic	No	16/02/2017	0	0	0
2015340481	276	rural/clinic	No	16/02/2017	0	0	0
2015360585	276	rural/clinic	No	16/02/2017	0	0	0
2015313423	276	rural/clinic	No	16/02/2017	0	0	0
2015313400	276	rural/clinic	No	16/02/2017	0	0	0
2014353624	276	rural/clinic	No	15/02/2017	0	0	0
2015360584	276	rural/clinic	No	16/02/2017	0	0	0
2015313399	276	rural/clinic	No	16/02/2017	0	0	0
2013251312	276	rural/clinic	No	15/02/2017	0	0	0
2014360880	276	rural/clinic	No	16/02/2017	0	0	0
2015313422	276	rural/clinic	No	16/02/2017	0	0	0
2010099621	276	rural/clinic	No	16/02/2017	0	0	0
2013251313	276	rural/clinic	No	15/02/2017	0	0	0
2014335223	276	rural/clinic	No	16/02/2017	0	0	0
2014353622	276	rural/clinic	No	15/02/2017	0	0	0
2015359201	276	rural/clinic	No	16/02/2017	0	0	0
2014335222	276	rural/clinic	No	16/02/2017	0	0	0
2012350620	276	rural/clinic	No	16/02/2017	0	0	0
2015397115	276	rural/clinic	No	15/02/2017	0	0	0
2013255303	276	rural/clinic	No	16/02/2017	0	0	0
2012294463	276	rural/clinic	No	16/02/2017	0	0	0
2015397116	276	rural/clinic	No	15/02/2017	0	0	0
2013263586	276	rural/clinic	No	16/02/2017	0	0	0
2015391861	276	rural/clinic	No	15/02/2017	0	0	0
2015394620	395	rural/clinic	No	15/02/2017	0	0	0
2015362752	395	rural/clinic	No	16/02/2017	0	0	0
2015391862	395	rural/clinic	No	15/02/2017	0	0	0
2015362753	395	rural/clinic	No	16/02/2017	0	0	0
2015346375	395	rural/clinic	No	16/02/2017	0	0	0
2012350619	395	rural/clinic	No	16/02/2017	0	0	0
2015346374	395	rural/clinic	No	16/02/2017	0	0	0
2011145391	395	rural/clinic	No	16/02/2017	0	0	0
2015362754	395	rural/clinic	No	16/02/2017	0	0	0
2011145390	395	rural/clinic	No	16/02/2017	0	0	0
2015361786	395	rural/clinic	No	16/02/2017	0	0	0
2015295341	395	rural/clinic	No	15/02/2017	0	0	0
2015305055	184	rural/clinic	No	16/02/2017	0	0	0
2012387248	184	rural/clinic	No	16/02/2017	0	0	0
2015316063	184	rural/clinic	No	15/02/2017	0	0	0
2013262209	184	rural/clinic	No	16/02/2017	0	0	0
2011141103	184	rural/clinic	No	16/02/2017	0	0	0
2012387246	184	rural/clinic	No	16/02/2017	0	0	0
2015377550	184	rural/clinic	No	14/02/2017	0	0	0
2015316064	184	rural/clinic	No	15/02/2017	0	0	0
2013262211	184	rural/clinic	No	16/02/2017	0	0	0
2015377424	184	rural/clinic	No	16/02/2017	0	0	0
2015316065	184	rural/clinic	No	15/02/2017	0	0	0
2012268995	184	rural/clinic	No	16/02/2017	0	0	0
2015335419	184	rural/clinic	No	14/02/2017	0	0	0
2013262210	90	rural/clinic	No	16/02/2017	0	0	0

2015316074	90	rural/clinic	No	15/02/2017	0	0	0
2015414557	90	rural/clinic	No	16/02/2017	0	0	0
2015335420	90	rural/clinic	No	14/02/2017	0	0	0
2015414556	90	rural/clinic	No	16/02/2017	0	0	0
2015316073	90	rural/clinic	No	15/02/2017	0	0	0
2015377548	90	rural/clinic	No	14/02/2017	0	0	0
2014297782	90	rural/clinic	No	16/02/2017	0	0	0
2015384802	90	rural/clinic	No	14/02/2017	0	0	0
2012387247	90	rural/clinic	No	16/02/2017	0	0	0
2014340268	90	rural/clinic	No	16/02/2017	0	0	0
2014345846	90	rural/clinic	No	16/02/2017	0	0	0
2014385489	90	rural/clinic	No	16/02/2017	0	0	0
2014359727	90	rural/clinic	No	14/02/2017	0	0	0
2014333797	90	rural/clinic	No	14/02/2017	0	0	0
2014363868	90	rural/clinic	No	14/02/2017	0	0	0
2014363867	90	rural/clinic	No	14/02/2017	0	0	0
2015316071	570	rural/clinic	No	15/02/2017	0	0	0
2015331713	570	rural/clinic	No	16/02/2017	0	0	0
2014345845	570	rural/clinic	No	16/02/2017	0	0	0
2014345844	570	rural/clinic	No	16/02/2017	0	0	0
2015331711	570	rural/clinic	No	16/02/2017	0	0	0
2015316070	570	rural/clinic	No	15/02/2017	0	0	0
2015331712	570	rural/clinic	No	16/02/2017	0	0	0
2015306075	570	rural/clinic	No	15/02/2017	0	0	0
2015328452	570	rural/clinic	No	16/02/2017	0	0	0
2014337042	570	rural/clinic	No	16/02/2017	0	0	0
2011143946	570	rural/clinic	No	16/02/2017	0	0	0
2011143945	570	rural/clinic	No	16/02/2017	0	0	0
2015293636	570	rural/clinic	No	16/02/2017	0	0	0
2015293638	570	rural/clinic	No	16/02/2017	0	0	0
2015319432	570	rural/clinic	No	16/02/2017	0	0	0
2015401550	570	rural/clinic	No	16/02/2017	0	0	0
2015319431	570	rural/clinic	No	16/02/2017	0	0	0
2015294044	570	rural/clinic	No	16/02/2017	0	0	0
2015328451	570	rural/clinic	No	16/02/2017	0	0	0
2015413543	570	rural/clinic	No	16/02/2017	0	0	0
2015401549	570	rural/clinic	No	16/02/2017	0	0	0
2015401548	570	rural/clinic	No	16/02/2017	0	0	0
2015413542	570	rural/clinic	No	16/02/2017	0	0	0
2015401547	570	rural/clinic	No	16/02/2017	0	0	0
2015413544	570	rural/clinic	No	16/02/2017	0	0	0
2015369495	570	rural/clinic	No	16/02/2017	0	0	0
2015413685	570	rural/clinic	No	16/02/2017	0	0	0
2015344647	570	rural/clinic	No	16/02/2017	0	0	0
2015344648	570	rural/clinic	No	16/02/2017	0	0	0
2014385492	570	rural/clinic	No	16/02/2017	0	0	0
2015369496	570	rural/clinic	No	16/02/2017	0	0	0
2015369494	570	rural/clinic	No	16/02/2017	0	0	0
2013254143	570	rural/clinic	No	16/02/2017	0	0	0
2015369497	570	rural/clinic	No	16/02/2017	0	0	0
2015373244	570	rural/clinic	No	16/02/2017	0	0	0
2015373243	570	rural/clinic	No	16/02/2017	0	0	0
2015373242	570	rural/clinic	No	16/02/2017	0	0	0
2015358140	570	rural/clinic	No	16/02/2017	0	0	0
2015373235	570	rural/clinic	No	16/02/2017	0	0	0
2015358139	570	rural/clinic	No	16/02/2017	0	0	0
2015373241	630	district/faith-based	No	16/02/2017	1	0	0
2015305792	630	district/faith-based	No	15/02/2017	1	0	0
2012283589	630	district/faith-based	No	16/02/2017	1	0	0
2015358136	630	district/faith-based	No	16/02/2017	1	0	0
2012283588	630	district/faith-based	No	16/02/2017	1	0	0
2015358294	630	district/faith-based	No	16/02/2017	1	0	0
2012283587	630	district/faith-based	No	16/02/2017	1	0	0
2015358287	630	district/faith-based	No	16/02/2017	1	0	0
2015358293	630	district/faith-based	No	16/02/2017	1	0	0
2014311539	630	district/faith-based	No	15/02/2017	1	0	0
2015358288	630	district/faith-based	No	16/02/2017	1	0	0
2015321802	630	district/faith-based	No	16/02/2017	1	0	0
2015358292	630	district/faith-based	No	16/02/2017	1	0	0
2014354758	630	district/faith-based	No	16/02/2017	1	0	0
2015358289	630	district/faith-based	No	16/02/2017	1	0	0
2013252932	630	district/faith-based	No	16/02/2017	1	0	0
2015358290	630	district/faith-based	No	16/02/2017	1	0	0
2015337301	630	district/faith-based	No	15/02/2017	1	0	0
2015358291	630	district/faith-based	No	16/02/2017	1	0	0
2014385686	630	district/faith-based	No	16/02/2017	1	0	0
2014385685	630	district/faith-based	No	16/02/2017	1	0	0
2015358135	630	district/faith-based	No	16/02/2017	1	0	0
2012341039	630	district/faith-based	No	16/02/2017	1	0	0
2013252933	630	district/faith-based	No	16/02/2017	1	0	0
2014311540	630	district/faith-based	No	15/02/2017	1	0	0
2012268994	630	district/faith-based	No	16/02/2017	1	0	0

2012245724	630	district/faith-based	No	16/02/2017	1	0	0
2015385511	630	district/faith-based	No	16/02/2017	1	0	0
2012245725	630	district/faith-based	No	16/02/2017	1	0	0
2014316756	630	district/faith-based	No	16/02/2017	1	0	0
2014357517	630	district/faith-based	No	16/02/2017	1	0	0
2014316757	630	district/faith-based	No	16/02/2017	1	0	0
2015319592	630	district/faith-based	No	16/02/2017	1	0	0
2914311541	630	district/faith-based	No	15/02/2017	1	0	0
2014357721	543	rural/clinic	No	16/02/2017	0	0	0
2015319590	543	rural/clinic	No	16/02/2017	0	0	0
2015319591	543	rural/clinic	No	16/02/2017	0	0	0
2014357722	543	rural/clinic	No	16/02/2017	0	0	0
2015319584	543	rural/clinic	No	16/02/2017	0	0	0
2014357723	543	rural/clinic	No	16/02/2017	0	0	0
2015319585	543	rural/clinic	No	16/02/2017	0	0	0
2015373342	56	rural/clinic	No	16/02/2017	0	0	0
2015368434	56	rural/clinic	No	14/02/2017	0	0	0
2012290680	56	rural/clinic	No	14/02/2017	0	0	0
2012290681	56	rural/clinic	No	14/02/2017	0	0	0
2015314315	56	rural/clinic	No	15/02/2017	0	0	0
2015344835	56	rural/clinic	No	14/02/2017	0	0	0
2014311437	56	rural/clinic	No	14/02/2017	0	0	0
2015314323	56	rural/clinic	No	15/02/2017	0	0	0
2014311439	56	rural/clinic	No	14/02/2017	0	0	0
2014311440	56	rural/clinic	No	14/02/2017	0	0	0
2015314316	56	rural/clinic	No	15/02/2017	0	0	0
2015368551	56	rural/clinic	No	14/02/2017	0	0	0
2015368552	56	rural/clinic	No	14/02/2017	0	0	0
2015368553	56	rural/clinic	No	14/02/2017	0	0	0
2015317611	56	rural/clinic	No	15/02/2017	0	0	0
2015368554	56	rural/clinic	No	14/02/2017	0	0	0
2015368555	56	rural/clinic	No	14/02/2017	0	0	0
2013256256	56	rural/clinic	No	14/02/2017	0	0	0
2015317610	56	rural/clinic	No	15/02/2017	0	0	0
2012299658	790	rural/clinic	No	15/02/2017	0	0	0
2013256257	790	rural/clinic	No	14/02/2017	0	0	0
2012346712	790	rural/clinic	No	14/02/2017	0	0	0
2014358201	790	rural/clinic	No	14/02/2017	0	0	0
2014327109	790	rural/clinic	No	15/02/2017	0	0	0
2015316067	790	rural/clinic	No	15/02/2017	0	0	0
2014353620	790	rural/clinic	No	15/02/2017	0	0	0
2012263742	790	rural/clinic	No	14/02/2017	0	0	0
2012263740	790	rural/clinic	No	15/02/2017	0	0	0
2012350099	790	rural/clinic	No	15/02/2017	0	0	0
2012263743	790	rural/clinic	No	15/02/2017	0	0	0
2012263741	790	rural/clinic	No	15/02/2017	0	0	0
2012350090	790	rural/clinic	No	15/02/2017	0	0	0
2015373388	790	rural/clinic	No	16/02/2017	0	0	0
2015373387	790	rural/clinic	No	16/02/2017	0	0	0
2012350098	790	rural/clinic	No	15/02/2017	0	0	0
2014360328	790	rural/clinic	No	26/01/2017	0	0	0
2015357180	445	rural/clinic	No	31/01/2017	0	0	0
2014289480	445	rural/clinic	No	31/01/2017	0	0	0
2014289480	445	rural/clinic	No	31/01/2017	0	0	0
2013254384/D	445	rural/clinic	No	15/02/2017	0	0	0
2015392532	445	rural/clinic	No	15/02/2017	0	0	0
2015356566	445	rural/clinic	No	15/02/2017	0	0	0
2014368221	445	rural/clinic	No	20/02/2017	0	0	0
2014368220	445	rural/clinic	No	20/02/2017	0	0	0
2014383035	445	rural/clinic	No	20/02/2017	0	0	0
2014383036	445	rural/clinic	No	20/02/2017	0	0	0
2015397858	445	rural/clinic	No	20/02/2017	0	0	0
2014383037	445	rural/clinic	No	20/02/2017	0	0	0
2015383586	445	rural/clinic	No	20/02/2017	0	0	0
2014328395	445	rural/clinic	No	20/02/2017	0	0	0
2015344649	445	rural/clinic	No	16/02/2017	0	0	0
2014368222	445	rural/clinic	No	20/02/2017	0	0	0
2015334602	445	rural/clinic	No	16/02/2017	0	0	0
2015408095	445	rural/clinic	No	02/02/2017	0	0	0
2015408095	445	rural/clinic	No	02/02/2017	0	0	0
2015314319	445	rural/clinic	No	15/02/2017	0	0	0
2015344650	445	rural/clinic	No	16/02/2017	0	0	0
2012363274	445	rural/clinic	No	02/02/2017	0	0	0
2012363274	140	rural/clinic	No	02/02/2017	0	0	0
2012272328	140	rural/clinic	No	15/02/2017	0	0	0
2014372222	140	rural/clinic	No	16/02/2017	0	0	0
2015358126	140	rural/clinic	No	02/02/2017	0	0	0
2015358126	140	rural/clinic	No	02/02/2017	0	0	0
2015358122	140	rural/clinic	No	02/02/2017	0	0	0
2015358122	140	rural/clinic	No	02/02/2017	0	0	0
2012272329	140	rural/clinic	No	15/02/2017	0	0	0
2015368426	140	rural/clinic	No	02/02/2017	0	0	0

2015368426	140	rural/clinic	No	02/02/2017	0	0	0
2012272330	140	rural/clinic	No	15/02/2017	0	0	0
2012340786	140	rural/clinic	No	16/02/2017	0	0	0
2012280593	140	rural/clinic	No	15/02/2017	0	0	0
2015366423	140	rural/clinic	No	15/02/2017	0	0	0
2015366424	140	rural/clinic	No	15/02/2017	0	0	0
2014372224	185	rural/clinic	No	16/02/2017	0	0	0
2014372225	185	rural/clinic	No	16/02/2017	0	0	0
2014368224	185	rural/clinic	No	20/02/2017	0	0	0
2015344725	185	rural/clinic	No	16/02/2017	0	0	0
2014368223	185	rural/clinic	No	20/02/2017	0	0	0
2015344726	185	rural/clinic	No	16/02/2017	0	0	0
2015350231	185	rural/clinic	No	20/02/2017	0	0	0
2014360335	185	rural/clinic	No	16/02/2017	0	0	0
2015350233	185	rural/clinic	No	20/02/2017	0	0	0
2014360336	185	rural/clinic	No	16/02/2017	0	0	0
2014360337	185	rural/clinic	No	16/02/2017	0	0	0
2014338247	185	rural/clinic	No	20/02/2017	0	0	0
2014338249	297	rural/clinic	No	20/02/2017	0	0	0
2014338250	297	rural/clinic	No	20/02/2017	0	0	0
2012336601	297	rural/clinic	No	20/02/2017	0	0	0
2012336602	297	rural/clinic	No	20/02/2017	0	0	0
2012259852	297	rural/clinic	No	20/02/2017	0	0	0
2012336605	297	rural/clinic	No	20/02/2017	0	0	0
2012259853	297	rural/clinic	No	20/02/2017	0	0	0
2012336603	297	rural/clinic	No	20/02/2017	0	0	0
2012259854	297	rural/clinic	No	20/02/2017	0	0	0
2015286512	321	rural/clinic	No	20/02/2017	0	0	0
2015405717	321	rural/clinic	No	20/02/2017	0	0	0
2012364941	321	rural/clinic	No	20/02/2017	0	0	0
2014324346	321	rural/clinic	No	15/02/2017	0	0	0
2012364942	321	rural/clinic	No	20/02/2017	0	0	0
2014324349	321	rural/clinic	No	15/02/2017	0	0	0
2014324348	321	rural/clinic	No	15/02/2017	0	0	0
2012364943	321	rural/clinic	No	20/02/2017	0	0	0
2014296876	321	rural/clinic	No	15/02/2017	0	0	0
2015293828	321	rural/clinic	No	16/02/2017	0	0	0
2014324350	321	rural/clinic	No	15/02/2017	0	0	0
2012364944	321	rural/clinic	No	20/02/2017	0	0	0
2015344067	321	rural/clinic	No	16/02/2017	0	0	0
2011186252	321	rural/clinic	No	15/02/2017	0	0	0
2015413579	321	rural/clinic	No	16/02/2017	0	0	0
2015306833	321	rural/clinic	No	15/02/2017	0	0	0
2015368445	321	rural/clinic	No	20/02/2017	0	0	0
2015413580	321	rural/clinic	No	16/02/2017	0	0	0
2012300834	321	rural/clinic	No	15/02/2017	0	0	0
2015293637	321	rural/clinic	No	16/02/2017	0	0	0
2012300832	321	rural/clinic	No	15/02/2017	0	0	0
2015368447	321	rural/clinic	No	20/02/2017	0	0	0
2015299430	321	rural/clinic	No	16/02/2017	0	0	0
2015351146	321	rural/clinic	No	20/02/2017	0	0	0
2014326746	321	rural/clinic	No	20/02/2017	0	0	0
2015351144	321	rural/clinic	No	20/02/2017	0	0	0
2015351143	321	rural/clinic	No	20/02/2017	0	0	0
2014324347	321	rural/clinic	No	15/02/2017	0	0	0
2015374319	321	rural/clinic	No	20/02/2017	0	0	0
2015351145	321	rural/clinic	No	20/02/2017	0	0	0
2012300831	321	rural/clinic	No	15/02/2017	0	0	0
2015351147	321	rural/clinic	No	20/02/2017	0	0	0
2015299767	321	rural/clinic	No	15/02/2017	0	0	0
2015397501	321	rural/clinic	No	20/02/2017	0	0	0
2015374321	321	rural/clinic	No	20/02/2017	0	0	0
2015356568	321	rural/clinic	No	15/02/2017	0	0	0
2015356567	321	rural/clinic	No	15/02/2017	0	0	0
2015397502	321	rural/clinic	No	20/02/2017	0	0	0
2015286518	321	rural/clinic	No	15/02/2017	0	0	0
2015374322	321	rural/clinic	No	20/02/2017	0	0	0
2015286511	321	rural/clinic	No	15/02/2017	0	0	0
2015397503	321	rural/clinic	No	20/02/2017	0	0	0
2015368431	654	rural/clinic	No	15/02/2017	0	0	0
2015397504	654	rural/clinic	No	20/02/2017	0	0	0
2015374324	654	rural/clinic	No	20/02/2017	0	0	0
2012364936	654	rural/clinic	No	15/02/2017	0	0	0
2015397505	654	rural/clinic	No	20/02/2017	0	0	0
2015368435	654	rural/clinic	No	15/02/2017	0	0	0
2015306834	654	rural/clinic	No	15/02/2017	0	0	0
2015397506	654	rural/clinic	No	20/02/2017	0	0	0
2011186251	654	rural/clinic	No	15/02/2017	0	0	0
2015374325	654	rural/clinic	No	20/02/2017	0	0	0
2015397507	654	rural/clinic	No	20/02/2017	0	0	0
2015369493	654	rural/clinic	No	15/02/2017	0	0	0
2015397508	654	rural/clinic	No	20/02/2017	0	0	0

2015369498	654	rural/clinic	No	15/02/2017	0	0	0
2015397509	654	rural/clinic	No	20/02/2017	0	0	0
2015401545	153	rural/clinic	No	15/02/2017	0	0	0
2015374326	153	rural/clinic	No	20/02/2017	0	0	0
2015369492	153	rural/clinic	No	15/02/2017	0	0	0
2015374327	153	rural/clinic	No	20/02/2017	0	0	0
2014301672	153	rural/clinic	No	20/02/2017	0	0	0
2015374328	396	rural/clinic	No	20/02/2017	0	0	0
2015397857	396	rural/clinic	No	20/02/2017	0	0	0
2015397859	396	rural/clinic	No	20/02/2017	0	0	0
2015374331	396	rural/clinic	No	20/02/2017	0	0	0
2015397860	396	rural/clinic	No	20/02/2017	0	0	0
2015397856	396	rural/clinic	No	20/02/2017	0	0	0
2015374329	396	rural/clinic	No	20/02/2017	0	0	0
2015397855	396	rural/clinic	No	20/02/2017	0	0	0
2015288741	916	rural/clinic	No	20/02/2017	0	0	0
2015350493	916	rural/clinic	No	20/02/2017	0	0	0
2014383034	916	rural/clinic	No	20/02/2017	0	0	0
2015297361	916	rural/clinic	No	20/02/2017	0	0	0
2015374337	916	rural/clinic	No	20/02/2017	0	0	0
2015297362	916	rural/clinic	No	20/02/2017	0	0	0
2014301456	322	rural/clinic	No	20/02/2017	0	0	0
2015374336	322	rural/clinic	No	20/02/2017	0	0	0
2014301454	322	rural/clinic	No	20/02/2017	0	0	0
2015374334	322	rural/clinic	No	20/02/2017	0	0	0
2015383584	322	rural/clinic	No	20/02/2017	0	0	0
2015350492	322	rural/clinic	No	20/02/2017	0	0	0
2015383585	322	rural/clinic	No	20/02/2017	0	0	0
2015374335	322	rural/clinic	No	20/02/2017	0	0	0
2014301671	322	rural/clinic	No	20/02/2017	0	0	0
2012272331	322	rural/clinic	No	20/02/2017	0	0	0
2014301455	323	rural/clinic	No	20/02/2017	0	0	0
2012280591	323	rural/clinic	No	20/02/2017	0	0	0
2012340785	323	rural/clinic	No	20/02/2017	0	0	0
2015303175	323	rural/clinic	No	20/02/2017	0	0	0
2013244110	323	rural/clinic	No	20/02/2017	0	0	0
2015303177	323	rural/clinic	No	20/02/2017	0	0	0
2014326745	323	rural/clinic	No	20/02/2017	0	0	0
2015368444	323	rural/clinic	No	20/02/2017	0	0	0
2012269010	323	rural/clinic	No	20/02/2017	0	0	0
2012269011	323	rural/clinic	No	20/02/2017	0	0	0
2015297301	323	rural/clinic	No	20/02/2017	0	0	0
2015412999	323	rural/clinic	No	20/02/2017	0	0	0
2015413000	323	rural/clinic	No	20/02/2017	0	0	0
2015301091	323	rural/clinic	No	20/02/2017	0	0	0
2015301092	323	rural/clinic	No	20/02/2017	0	0	0
2015377859	323	rural/clinic	No	20/02/2017	0	0	0
2015377860	323	rural/clinic	No	20/02/2017	0	0	0
2014301457	323	rural/clinic	No	20/02/2017	0	0	0
2015303176	323	rural/clinic	No	20/02/2017	0	0	0
2015376103	154	rural/clinic	No	21/02/2017	0	0	0
2015384477	154	rural/clinic	No	21/02/2017	0	0	0
2015384478	154	rural/clinic	No	21/02/2017	0	0	0
123	154	rural/clinic	No	21/02/2017	0	0	0
223	154	rural/clinic	No	21/02/2017	0	0	0
323	154	rural/clinic	No	21/02/2017	0	0	0
423	154	rural/clinic	No	21/02/2017	0	0	0
523	154	rural/clinic	No	21/02/2017	0	0	0
623	154	rural/clinic	No	21/02/2017	0	0	0
723	154	rural/clinic	No	21/02/2017	0	0	0
823	154	rural/clinic	No	21/02/2017	0	0	0
923	154	rural/clinic	No	21/02/2017	0	0	0
1023	154	rural/clinic	No	21/02/2017	0	0	0
1123	154	rural/clinic	No	21/02/2017	0	0	0
1223	154	rural/clinic	No	21/02/2017	0	0	0
1323	154	rural/clinic	No	21/02/2017	0	0	0
1423	154	rural/clinic	No	21/02/2017	0	0	0
1523	154	rural/clinic	No	21/02/2017	0	0	0
1623	154	rural/clinic	No	21/02/2017	0	0	0
1723	154	rural/clinic	No	21/02/2017	0	0	0
1823	154	rural/clinic	No	21/02/2017	0	0	0
1923	154	rural/clinic	No	21/02/2017	0	0	0
1923	154	rural/clinic	No	21/02/2017	0	0	0
2014365637	154	rural/clinic	No	21/02/2017	0	0	0
2015333801	154	rural/clinic	No	21/02/2017	0	0	0
2011212647	154	rural/clinic	No	21/02/2017	0	0	0
2012295393	154	rural/clinic	No	07/02/2017	0	0	0
2012295394	154	rural/clinic	No	07/02/2017	0	0	0
2012295395	154	rural/clinic	No	07/02/2017	0	0	0
2012387244	154	rural/clinic	No	02/02/2017	0	0	0
2015413079	154	rural/clinic	No	02/02/2017	0	0	0
2015413079	154	rural/clinic	No	02/02/2017	0	0	0

2015378946	154	rural/clinic	No	02/02/2017	0	0	0
2015378946	154	rural/clinic	No	02/02/2017	0	0	0
2015376102	154	rural/clinic	No	21/02/2017	0	0	0
2015418122	154	rural/clinic	No	21/02/2017	0	0	0
2015373222	154	rural/clinic	No	02/02/2017	0	0	0
2015373222	154	rural/clinic	No	02/02/2017	0	0	0
2015335189	154	rural/clinic	No	21/02/2017	0	0	0
2015378717	154	rural/clinic	No	02/02/2017	0	0	0
2015378717	154	rural/clinic	No	02/02/2017	0	0	0
2013267196	154	rural/clinic	No	02/02/2017	0	0	0
2013267196	154	rural/clinic	No	02/02/2017	0	0	0
2015335187	154	rural/clinic	No	21/02/2017	0	0	0
2015351132	154	rural/clinic	No	03/02/2017	0	0	0
2015351132	154	rural/clinic	No	03/02/2017	0	0	0
2012295955	154	rural/clinic	No	07/02/2017	0	0	0
2012295955	154	rural/clinic	No	07/02/2017	0	0	0
2012295956	154	rural/clinic	No	07/02/2017	0	0	0
2012295956	154	rural/clinic	No	07/02/2017	0	0	0
2012295957	154	rural/clinic	No	07/02/2017	0	0	0
2012295957	154	rural/clinic	No	07/02/2017	0	0	0
2013263585	154	rural/clinic	No	07/02/2017	0	0	0
2013263585	154	rural/clinic	No	07/02/2017	0	0	0
2015337351	154	rural/clinic	No	07/02/2017	0	0	0
2015337351	154	rural/clinic	No	07/02/2017	0	0	0
2015315977	154	rural/clinic	No	07/02/2017	0	0	0
2015315977	154	rural/clinic	No	07/02/2017	0	0	0
2012275984	154	rural/clinic	No	21/02/2017	0	0	0
2014319429	154	rural/clinic	No	21/02/2017	0	0	0
2011123686	154	rural/clinic	No	21/02/2017	0	0	0
2014303287	154	rural/clinic	No	21/02/2017	0	0	0
2015302102	154	rural/clinic	No	21/02/2017	0	0	0
2012363295	154	rural/clinic	No	21/02/2017	0	0	0
2015363703	154	rural/clinic	No	21/02/2017	0	0	0
2011223936	154	rural/clinic	No	21/02/2017	0	0	0
2014369182	154	rural/clinic	No	21/02/2017	0	0	0
2011223937	154	rural/clinic	No	21/02/2017	0	0	0
2015339502	154	rural/clinic	No	21/02/2017	0	0	0
2012290394	154	rural/clinic	No	21/02/2017	0	0	0
2015339508	154	rural/clinic	No	21/02/2017	0	0	0
2011224028	154	rural/clinic	No	21/02/2017	0	0	0
2015339509	154	rural/clinic	No	21/02/2017	0	0	0
2015339506	154	rural/clinic	No	21/02/2017	0	0	0
201433895	154	rural/clinic	No	21/02/2017	0	0	0
2015339505	154	rural/clinic	No	21/02/2017	0	0	0
2011225150	154	rural/clinic	No	21/02/2017	0	0	0
2015339504	154	rural/clinic	No	21/02/2017	0	0	0
2015339507	154	rural/clinic	No	21/02/2017	0	0	0
2015363705	154	rural/clinic	No	21/02/2017	0	0	0
2014368016	154	rural/clinic	No	21/02/2017	0	0	0
2015339501	154	rural/clinic	No	21/02/2017	0	0	0
2015339503	154	rural/clinic	No	21/02/2017	0	0	0
2015414932	154	rural/clinic	No	21/02/2017	0	0	0
2013253932	154	rural/clinic	No	21/02/2017	0	0	0
2015339804	154	rural/clinic	No	21/02/2017	0	0	0
2015414931	154	rural/clinic	No	21/02/2017	0	0	0
2014336940	397	rural/clinic	No	21/02/2017	0	0	0
2013253933	397	rural/clinic	No	21/02/2017	0	0	0
2014298237	397	rural/clinic	No	21/02/2017	0	0	0
2014302842	397	rural/clinic	No	21/02/2017	0	0	0
2013253929	397	rural/clinic	No	21/02/2017	0	0	0
2015414934	397	rural/clinic	No	21/02/2017	0	0	0
2015414933	397	rural/clinic	No	21/02/2017	0	0	0
2015340004	397	rural/clinic	No	21/02/2017	0	0	0
2015363704	397	rural/clinic	No	21/02/2017	0	0	0
2014332077	397	rural/clinic	No	21/02/2017	0	0	0
2015339805	397	rural/clinic	No	21/02/2017	0	0	0
2015414647	397	rural/clinic	No	21/02/2017	0	0	0
2015414646	397	rural/clinic	No	21/02/2017	0	0	0
2011199073	397	rural/clinic	No	21/02/2017	0	0	0
2015414645	397	rural/clinic	No	21/02/2017	0	0	0
2014291583	397	rural/clinic	No	21/02/2017	0	0	0
2015303112	397	rural/clinic	No	21/02/2017	0	0	0
2012365146	397	rural/clinic	No	21/02/2017	0	0	0
2015357653	397	rural/clinic	No	21/02/2017	0	0	0
2012365145	397	rural/clinic	No	21/02/2017	0	0	0
2014291584	397	rural/clinic	No	21/02/2017	0	0	0
2015413334	397	rural/clinic	No	21/02/2017	0	0	0
2015301443	397	rural/clinic	No	21/02/2017	0	0	0
2012252687	141	rural/clinic	No	21/02/2017	0	0	0
2015357630	141	district/faith-based	No	21/02/2017	1	0	0
2015413335	141	district/faith-based	No	21/02/2017	1	0	0
2015325452	141	district/faith-based	No	21/02/2017	1	0	0

2012306341	141	district/faith-based	No	21/02/2017	1	0	0
2014291582	141	district/faith-based	No	21/02/2017	1	0	0
2015340833	141	district/faith-based	No	21/02/2017	1	0	0
2015413336	141	district/faith-based	No	21/02/2017	1	0	0
2012365646	141	district/faith-based	No	21/02/2017	1	0	0
2015340834	141	district/faith-based	No	21/02/2017	1	0	0
2015335188	141	district/faith-based	No	21/02/2017	1	0	0
2015286523	141	district/faith-based	No	21/02/2017	1	0	0
2015324033	141	district/faith-based	No	21/02/2017	1	0	0
2015362345	141	district/faith-based	No	21/02/2017	1	0	0
2015413337	141	district/faith-based	No	21/02/2017	1	0	0
2012365647	141	district/faith-based	No	21/02/2017	1	0	0
2015286524	141	district/faith-based	No	21/02/2017	1	0	0
2015385936	141	district/faith-based	No	21/02/2017	1	0	0
2015362348	141	district/faith-based	No	21/02/2017	1	0	0
2015286525	141	district/faith-based	No	21/02/2017	1	0	0
2015385937	141	district/faith-based	No	21/02/2017	1	0	0
2014334168	141	district/faith-based	No	21/02/2017	1	0	0
2015413100	141	district/faith-based	No	21/02/2017	1	0	0
2011114842	141	district/faith-based	No	21/02/2017	1	0	0
2015362346	141	district/faith-based	No	21/02/2017	1	0	0
2015385938	141	district/faith-based	No	21/02/2017	1	0	0
2015362347	141	district/faith-based	No	21/02/2017	1	0	0
2011144801	141	district/faith-based	No	21/02/2017	1	0	0
2015384775	141	district/faith-based	No	21/02/2017	1	0	0
2014351198	141	district/faith-based	No	21/02/2017	1	0	0
2012251535	141	district/faith-based	No	21/02/2017	1	0	0
2012339733	141	district/faith-based	No	21/02/2017	1	0	0
2016326461	141	district/faith-based	No	21/02/2017	1	0	0
2015349933	141	district/faith-based	No	21/02/2017	1	0	0
2014344531	141	district/faith-based	No	21/02/2017	1	0	0
2014351197	141	district/faith-based	No	21/02/2017	1	0	0
2014319428	141	district/faith-based	No	21/02/2017	1	0	0
2014327034	141	district/faith-based	No	21/02/2017	1	0	0
2014290320	141	district/faith-based	No	21/02/2017	1	0	0
2015349721	141	district/faith-based	No	21/02/2017	1	0	0
2015384774	141	district/faith-based	No	21/02/2017	1	0	0
2015286863	141	district/faith-based	No	21/02/2017	1	0	0
2015349722	141	district/faith-based	No	21/02/2017	1	0	0
2012388375	141	district/faith-based	No	21/02/2017	1	0	0
2012289892	141	district/faith-based	No	21/02/2017	1	0	0
20153021036	141	district/faith-based	No	21/02/2017	1	0	0
2014378265	141	district/faith-based	No	21/02/2017	1	0	0
2014291615	141	district/faith-based	No	21/02/2017	1	0	0
2015325983	16	referral	No	21/02/2017	0	0	1
2013261927	16	referral	No	21/02/2017	0	0	1
2014355845	16	referral	No	21/02/2017	0	0	1
2014290321	16	referral	No	21/02/2017	0	0	1
2012268780	16	referral	No	21/02/2017	0	0	1
2015325982	16	referral	No	21/02/2017	0	0	1
2015325984	16	referral	No	21/02/2017	0	0	1
2015286862	16	referral	No	21/02/2017	0	0	1
2014311647	16	referral	No	21/02/2017	0	0	1
2015349934	16	referral	No	21/02/2017	0	0	1
2015286861	16	referral	No	21/02/2017	0	0	1
2015349935	16	referral	No	21/02/2017	0	0	1
2013247133	16	referral	No	21/02/2017	0	0	1
2015324464	16	referral	No	21/02/2017	0	0	1
2015286860	16	referral	No	21/02/2017	0	0	1
2013261926	16	referral	No	21/02/2017	0	0	1
2012251534	16	referral	No	21/02/2017	0	0	1
2015337028	16	referral	No	21/02/2017	0	0	1
2012276145	16	referral	No	21/02/2017	0	0	1
2014378266	16	referral	No	21/02/2017	0	0	1
2015382480	16	referral	No	21/02/2017	0	0	1
2014311648	16	referral	No	21/02/2017	0	0	1
2015382481	16	referral	No	21/02/2017	0	0	1
2014327237	16	referral	No	21/02/2017	0	0	1
2015355508	16	referral	No	21/02/2017	0	0	1
2014342507	16	referral	No	21/02/2017	0	0	1
2015382482	16	referral	No	21/02/2017	0	0	1
2014336823	16	referral	No	21/02/2017	0	0	1
2015349930	16	referral	No	21/02/2017	0	0	1
2013271833	16	referral	No	21/02/2017	0	0	1
2015382483	16	referral	No	21/02/2017	0	0	1
2015337355	16	referral	No	21/02/2017	0	0	1
2011229213	16	referral	No	21/02/2017	0	0	1
2015382484	16	referral	No	21/02/2017	0	0	1
2015357369	16	referral	No	21/02/2017	0	0	1
2015382494	16	referral	No	21/02/2017	0	0	1
2011199239	16	referral	No	21/02/2017	0	0	1
2015315979	16	referral	No	21/02/2017	0	0	1

2015357368	16	referral	No	21/02/2017	0	0	1
2015382493	16	referral	No	21/02/2017	0	0	1
2011199240	16	referral	No	21/02/2017	0	0	1
2015382492	16	referral	No	21/02/2017	0	0	1
2015315981	16	referral	No	21/02/2017	0	0	1
2015357370	16	referral	No	21/02/2017	0	0	1
2014336824	16	referral	No	21/02/2017	0	0	1
2015418121	16	referral	No	21/02/2017	0	0	1
2015340003	16	referral	No	21/02/2017	0	0	1
2015382491	16	referral	No	21/02/2017	0	0	1
2014319211	16	referral	No	21/02/2017	0	0	1
2015315980	16	referral	No	21/02/2017	0	0	1
2014302978	16	referral	No	21/02/2017	0	0	1
2015382490	16	referral	No	21/02/2017	0	0	1
2015340051	16	referral	No	21/02/2017	0	0	1
2015349929	16	referral	No	21/02/2017	0	0	1
2015382489	16	referral	No	21/02/2017	0	0	1
2015303059	16	referral	No	21/02/2017	0	0	1
2015337027	16	referral	No	21/02/2017	0	0	1
2015382488	16	referral	No	21/02/2017	0	0	1
2015368556	16	referral	No	21/02/2017	0	0	1
2015340052	16	referral	No	21/02/2017	0	0	1
2015358493	16	referral	No	21/02/2017	0	0	1
2014327238	16	referral	No	21/02/2017	0	0	1
2015382486	16	referral	No	21/02/2017	0	0	1
2015368557	16	referral	No	21/02/2017	0	0	1
2015303055	16	referral	No	21/02/2017	0	0	1
2015358492	16	referral	No	21/02/2017	0	0	1
2015325925	16	referral	No	21/02/2017	0	0	1
2015368558	16	referral	No	21/02/2017	0	0	1
2015382485	16	referral	No	21/02/2017	0	0	1
2012314007	16	referral	No	21/02/2017	0	0	1
2013256258	16	referral	No	21/02/2017	0	0	1
2015358491	16	referral	No	21/02/2017	0	0	1
2012259851	16	referral	No	21/02/2017	0	0	1
2014342506	16	referral	No	21/02/2017	0	0	1
2015303058	16	referral	No	21/02/2017	0	0	1
2015358490	16	referral	No	21/02/2017	0	0	1
2015372910	16	referral	No	21/02/2017	0	0	1
2013256259	16	referral	No	21/02/2017	0	0	1
2014371494	16	referral	No	21/02/2017	0	0	1
2014342505	16	referral	No	21/02/2017	0	0	1
2014314707	16	referral	No	21/02/2017	0	0	1
2014371495	16	referral	No	21/02/2017	0	0	1
2015358489	16	referral	No	21/02/2017	0	0	1
2015289167	16	referral	No	21/02/2017	0	0	1
2015418186	16	referral	No	21/02/2017	0	0	1
2015358488	16	referral	No	21/02/2017	0	0	1
2014314714	16	referral	No	21/02/2017	0	0	1
2015418120	16	referral	No	21/02/2017	0	0	1
2012314567	16	referral	No	21/02/2017	0	0	1
2015418184	16	referral	No	21/02/2017	0	0	1
2015328453	16	referral	No	21/02/2017	0	0	1
2014314709	16	referral	No	21/02/2017	0	0	1
2015358487	16	referral	No	21/02/2017	0	0	1
2012346713	16	referral	No	21/02/2017	0	0	1
2014314708	16	referral	No	21/02/2017	0	0	1
2014342508	16	referral	No	21/02/2017	0	0	1
2015413981	16	referral	No	21/02/2017	0	0	1
2015358499	16	referral	No	21/02/2017	0	0	1
2012314566	16	referral	No	21/02/2017	0	0	1
2015418183	16	referral	No	21/02/2017	0	0	1
2014314710	16	referral	No	21/02/2017	0	0	1
2015358498	16	referral	No	21/02/2017	0	0	1
2011134956	16	referral	No	21/02/2017	0	0	1
2015418182	16	referral	No	21/02/2017	0	0	1
2014314711	16	referral	No	21/02/2017	0	0	1
2015418185	16	referral	No	21/02/2017	0	0	1
2015358497	16	referral	No	21/02/2017	0	0	1
2011134955	16	referral	No	21/02/2017	0	0	1
2014314713	16	referral	No	21/02/2017	0	0	1
2011134957	16	referral	No	21/02/2017	0	0	1
2015358495	16	referral	No	21/02/2017	0	0	1
2014314712	16	referral	No	21/02/2017	0	0	1
2012244279	16	referral	No	21/02/2017	0	0	1
2015303057	16	referral	No	21/02/2017	0	0	1
2015358494	16	referral	No	21/02/2017	0	0	1
2011134954	16	referral	No	21/02/2017	0	0	1
2012244280	16	referral	No	21/02/2017	0	0	1
2015413101	16	referral	No	21/02/2017	0	0	1
2015358484	16	referral	No	21/02/2017	0	0	1
2015413099	16	referral	No	21/02/2017	0	0	1

2014371493	16	referral	No	21/02/2017	0	0	1
2014358181	16	referral	No	21/02/2017	0	0	1
2015413098	16	referral	No	21/02/2017	0	0	1
2015358486	16	referral	No	21/02/2017	0	0	1
2015413097	16	referral	No	21/02/2017	0	0	1
2015303056	16	referral	No	21/02/2017	0	0	1
2014358180	16	referral	No	21/02/2017	0	0	1
2015358482	16	referral	No	21/02/2017	0	0	1
2014344362	16	referral	No	21/02/2017	0	0	1
2015413096	16	referral	No	21/02/2017	0	0	1
2015418181	16	referral	No	21/02/2017	0	0	1
2015413095	16	referral	No	21/02/2017	0	0	1
2015358483	16	referral	No	21/02/2017	0	0	1
2015357371	16	referral	No	21/02/2017	0	0	1
2015358485	16	referral	No	21/02/2017	0	0	1
2015413094	16	referral	No	21/02/2017	0	0	1
2014375661	16	referral	No	21/02/2017	0	0	1
2014319212	16	referral	No	21/02/2017	0	0	1
2015325926	16	referral	No	21/02/2017	0	0	1
2015325174	16	referral	No	21/02/2017	0	0	1
2015340054	16	referral	No	21/02/2017	0	0	1
2015375548	16	referral	No	21/02/2017	0	0	1
2015412767	16	referral	No	21/02/2017	0	0	1
2014350091	16	referral	No	21/02/2017	0	0	1
2015377615	16	referral	No	21/02/2017	0	0	1
2015412766	16	referral	No	21/02/2017	0	0	1
2012350097	16	referral	No	21/02/2017	0	0	1
2015325173	16	referral	No	21/02/2017	0	0	1
2015357522	16	referral	No	21/02/2017	0	0	1
2012376646	16	referral	No	21/02/2017	0	0	1
2014375660	16	referral	No	21/02/2017	0	0	1
2014327110	16	referral	No	21/02/2017	0	0	1
2015377616	16	referral	No	21/02/2017	0	0	1
2015302500	16	referral	No	21/02/2017	0	0	1
2014368837	16	referral	No	21/02/2017	0	0	1
2015302499	16	referral	No	21/02/2017	0	0	1
2015377617	16	referral	No	21/02/2017	0	0	1
2014368838	16	referral	No	21/02/2017	0	0	1
2015302498	16	referral	No	21/02/2017	0	0	1
2015377618	16	referral	No	21/02/2017	0	0	1
2015412768	16	referral	No	21/02/2017	0	0	1
2014297958	16	referral	No	21/02/2017	0	0	1
2015302497	16	referral	No	21/02/2017	0	0	1
2012304317	16	referral	No	21/02/2017	0	0	1
2015297068	16	referral	No	21/02/2017	0	0	1
2012269012	16	referral	No	21/02/2017	0	0	1
2015377619	16	referral	No	21/02/2017	0	0	1
2015297069	16	referral	No	21/02/2017	0	0	1
2012304320	16	referral	No	21/02/2017	0	0	1
2015362756	16	referral	No	21/02/2017	0	0	1
2014297959	16	referral	No	21/02/2017	0	0	1
2012304319	16	referral	No	21/02/2017	0	0	1
2015362755	16	referral	No	21/02/2017	0	0	1
2015414316	16	referral	No	21/02/2017	0	0	1
2015414317	16	referral	No	21/02/2017	0	0	1
2014313550	16	referral	No	21/02/2017	0	0	1
2014344401	16	referral	No	21/02/2017	0	0	1
2014310205	16	referral	No	21/02/2017	0	0	1
2015414319	16	referral	No	21/02/2017	0	0	1
2015286590	16	referral	No	21/02/2017	0	0	1
2015375960	16	referral	No	21/02/2017	0	0	1
2015303470	16	referral	No	21/02/2017	0	0	1
2015375959	16	referral	No	21/02/2017	0	0	1
2015375961	16	referral	No	21/02/2017	0	0	1
2015286591	16	referral	No	21/02/2017	0	0	1
2014350090	16	referral	No	21/02/2017	0	0	1
2015334093	16	referral	No	21/02/2017	0	0	1
2015286592	16	referral	No	21/02/2017	0	0	1
2014319127	16	referral	No	21/02/2017	0	0	1
2012259140	16	referral	No	21/02/2017	0	0	1
2015414318	16	referral	No	21/02/2017	0	0	1
2015286589	16	referral	No	21/02/2017	0	0	1
2013256395	16	referral	No	21/02/2017	0	0	1
2015357182	16	referral	No	21/02/2017	0	0	1
2012259139	16	referral	No	21/02/2017	0	0	1
2015286588	16	referral	No	21/02/2017	0	0	1
2015335422	16	referral	No	21/02/2017	0	0	1
2015357181	16	referral	No	21/02/2017	0	0	1
2015334079	16	referral	No	21/02/2017	0	0	1
2015286587	16	referral	No	21/02/2017	0	0	1
2015334094	16	referral	No	21/02/2017	0	0	1
2015368868	16	referral	No	21/02/2017	0	0	1

2015334090	16	referral	No	21/02/2017	0	0	1
2015378723	16	referral	No	21/02/2017	0	0	1
2015378724	16	referral	No	21/02/2017	0	0	1
2015334089	16	referral	No	21/02/2017	0	0	1
2014363587	16	referral	No	21/02/2017	0	0	1
2015334088	16	referral	No	21/02/2017	0	0	1
2015378722	16	referral	No	21/02/2017	0	0	1
2015334086	16	referral	No	21/02/2017	0	0	1
2015294871	16	referral	No	21/02/2017	0	0	1
2015378721	16	referral	No	21/02/2017	0	0	1
2015334082	16	referral	No	21/02/2017	0	0	1
2012261445	16	referral	No	21/02/2017	0	0	1
2012261446	16	referral	No	21/02/2017	0	0	1
2015334080	16	referral	No	21/02/2017	0	0	1
2015334081	16	referral	No	21/02/2017	0	0	1
2015355725	16	referral	No	21/02/2017	0	0	1
2014375659	16	referral	No	21/02/2017	0	0	1
2015294300	16	referral	No	21/02/2017	0	0	1
2011223601	16	referral	No	21/02/2017	0	0	1
2012317313	16	referral	No	21/02/2017	0	0	1
2014371820	16	referral	No	21/02/2017	0	0	1
2015335573	16	referral	No	21/02/2017	0	0	1
2014375657	16	referral	No	21/02/2017	0	0	1
201085802	16	referral	No	21/02/2017	0	0	1
201085803	16	referral	No	21/02/2017	0	0	1
2013256389	16	referral	No	21/02/2017	0	0	1
2014288571	16	referral	No	21/02/2017	0	0	1
2015337951	16	referral	No	21/02/2017	0	0	1
2014288570	16	referral	No	21/02/2017	0	0	1
2015369913	16	referral	No	21/02/2017	0	0	1
2014344464	16	referral	No	21/02/2017	0	0	1
2011222298	16	referral	No	21/02/2017	0	0	1
2015377549	16	referral	No	21/02/2017	0	0	1
2015369914	16	referral	No	21/02/2017	0	0	1
2014344463	16	referral	No	21/02/2017	0	0	1
2015335421	16	referral	No	21/02/2017	0	0	1
2015294299	16	referral	No	21/02/2017	0	0	1
2014314540	16	referral	No	21/02/2017	0	0	1
2014311363	16	referral	No	21/02/2017	0	0	1
2014375658	16	referral	No	21/02/2017	0	0	1
2014373094	16	referral	No	21/02/2017	0	0	1
2014337217	16	referral	No	21/02/2017	0	0	1
2014378718	16	referral	No	21/02/2017	0	0	1
2015390257	16	referral	No	21/02/2017	0	0	1
2014373095	16	referral	No	21/02/2017	0	0	1
2014351219	16	referral	No	21/02/2017	0	0	1
2013272678	16	referral	No	21/02/2017	0	0	1
2013264056	16	referral	No	21/02/2017	0	0	1
2013264057	16	referral	No	21/02/2017	0	0	1
2015414787	16	referral	No	21/02/2017	0	0	1
2015414786	16	referral	No	21/02/2017	0	0	1
2015414788	16	referral	No	21/02/2017	0	0	1
2015384476	16	referral	No	21/02/2017	0	0	1
2015377315	16	referral	No	21/02/2017	0	0	1
2015377316	16	referral	No	21/02/2017	0	0	1
2015377159	16	referral	No	21/02/2017	0	0	1
2015377158	16	referral	No	21/02/2017	0	0	1
2015377160	16	referral	No	21/02/2017	0	0	1
2013272680	16	referral	No	21/02/2017	0	0	1
2014351220	16	referral	No	21/02/2017	0	0	1
2014339738	16	referral	No	21/02/2017	0	0	1
2015324032	16	referral	No	21/02/2017	0	0	1
2013272679	16	referral	No	21/02/2017	0	0	1
2015384527	16	referral	No	21/02/2017	0	0	1
2014306081	16	referral	No	21/02/2017	0	0	1
2012353425	16	referral	No	21/02/2017	0	0	1
2015377022	16	referral	No	21/02/2017	0	0	1
2012353424	16	referral	No	21/02/2017	0	0	1
2015377021	16	referral	No	21/02/2017	0	0	1
2015377019	16	referral	No	21/02/2017	0	0	1
2015377020	16	referral	No	21/02/2017	0	0	1
2014317341	16	referral	No	21/02/2017	0	0	1
2011226474	16	referral	No	21/02/2017	0	0	1
2014378719	16	referral	No	21/02/2017	0	0	1
2014378720	16	referral	No	21/02/2017	0	0	1
2011226472	16	referral	No	21/02/2017	0	0	1
2014314539	16	referral	No	21/02/2017	0	0	1
2011226473	16	referral	No	21/02/2017	0	0	1
2014347004	16	referral	No	21/02/2017	0	0	1
201434005	16	referral	No	21/02/2017	0	0	1
2014347006	16	referral	No	21/02/2017	0	0	1
2015335368	16	referral	No	21/02/2017	0	0	1

2015335369	16	referral	No	21/02/2017	0	0	1
2012390996	16	referral	No	21/02/2017	0	0	1
2012390995	16	referral	No	21/02/2017	0	0	1
2014339739	16	referral	No	21/02/2017	0	0	1
2017123684	16	referral	No	21/02/2017	0	0	1
2011168515	16	referral	No	21/02/2017	0	0	1
2015376202	16	referral	No	21/02/2017	0	0	1
2012352345	16	referral	No	21/02/2017	0	0	1
2012352346	16	referral	No	21/02/2017	0	0	1
2012352344	16	referral	No	21/02/2017	0	0	1
2014358728	16	referral	No	21/02/2017	0	0	1
2014358729	16	referral	No	21/02/2017	0	0	1
2012361945	16	referral	No	21/02/2017	0	0	1
2012352343	16	referral	No	21/02/2017	0	0	1
2014366064	16	referral	No	21/02/2017	0	0	1
2015320863	16	referral	No	21/02/2017	0	0	1
2015320862	16	referral	No	21/02/2017	0	0	1
2012361946	16	referral	No	21/02/2017	0	0	1
2011204758	16	referral	No	21/02/2017	0	0	1
2014358682	16	referral	No	21/02/2017	0	0	1
2012267313	16	referral	No	21/02/2017	0	0	1
2012329102	16	referral	No	21/02/2017	0	0	1
2011115900	16	referral	No	21/02/2017	0	0	1
2014369033	16	referral	No	21/02/2017	0	0	1
2012363294	16	referral	No	21/02/2017	0	0	1
2015344065	16	referral	No	07/02/2017	0	0	1
2015344065	16	referral	No	07/02/2017	0	0	1
2015319427	16	referral	No	07/02/2017	0	0	1
2015319427	16	referral	No	07/02/2017	0	0	1
2015293635	16	referral	No	07/02/2017	0	0	1
2015293635	16	referral	No	07/02/2017	0	0	1
2015322571	16	referral	No	07/02/2017	0	0	1
2015322571	16	referral	No	07/02/2017	0	0	1
2015348885	298	rural/clinic	No	07/02/2017	0	0	0
2015348885	298	rural/clinic	No	07/02/2017	0	0	0
2015351137	298	rural/clinic	No	07/02/2017	0	0	0
2015351137	298	rural/clinic	No	07/02/2017	0	0	0
2011225145	298	rural/clinic	No	07/02/2017	0	0	0
2011225145	298	rural/clinic	No	07/02/2017	0	0	0
2014309379	298	rural/clinic	No	07/02/2017	0	0	0
2014309379	298	rural/clinic	No	07/02/2017	0	0	0
2015418115	298	rural/clinic	No	07/02/2017	0	0	0
2015418115	298	rural/clinic	No	07/02/2017	0	0	0
2014326619	446	rural/clinic	No	07/02/2017	0	0	0
2015397485	446	rural/clinic	No	07/02/2017	0	0	0
2015397485	446	rural/clinic	No	07/02/2017	0	0	0
2014369031	446	rural/clinic	No	07/02/2017	0	0	0
2014369031	446	rural/clinic	No	07/02/2017	0	0	0
2015315477	446	rural/clinic	No	07/02/2017	0	0	0
2015315477	446	rural/clinic	No	07/02/2017	0	0	0
2015315478	446	rural/clinic	No	07/02/2017	0	0	0
2015315478	446	rural/clinic	No	07/02/2017	0	0	0
2015315474	446	rural/clinic	No	07/02/2017	0	0	0
2015315474	446	rural/clinic	No	07/02/2017	0	0	0
2015305053	446	rural/clinic	No	07/02/2017	0	0	0
2015305053	446	rural/clinic	No	07/02/2017	0	0	0
2015302495	446	rural/clinic	No	07/02/2017	0	0	0
2015302495	446	rural/clinic	No	07/02/2017	0	0	0
2015357367	446	rural/clinic	No	07/02/2017	0	0	0
2015357367	446	rural/clinic	No	07/02/2017	0	0	0
2012352824	446	rural/clinic	No	21/02/2017	0	0	0
2012352825	446	rural/clinic	No	21/02/2017	0	0	0
2013272682	446	rural/clinic	No	21/02/2017	0	0	0
2013272683	446	rural/clinic	No	21/02/2017	0	0	0
2013272681	446	rural/clinic	No	21/02/2017	0	0	0
2012369802	446	rural/clinic	No	21/02/2017	0	0	0
2014311441	446	rural/clinic	No	21/02/2017	0	0	0
2012313096	446	rural/clinic	No	21/02/2017	0	0	0
2012313097	446	rural/clinic	No	21/02/2017	0	0	0
2015333953	446	rural/clinic	No	21/02/2017	0	0	0
2011193474	446	rural/clinic	No	21/02/2017	0	0	0
2011193477	446	rural/clinic	No	21/02/2017	0	0	0
2015346087	446	rural/clinic	No	21/02/2017	0	0	0
2015346086	446	rural/clinic	No	21/02/2017	0	0	0
2015385514	446	rural/clinic	No	21/02/2017	0	0	0
2015385513	446	rural/clinic	No	21/02/2017	0	0	0
2014358325	446	rural/clinic	No	21/02/2017	0	0	0
2014373096	446	rural/clinic	No	21/02/2017	0	0	0
2011232682	446	rural/clinic	No	21/02/2017	0	0	0
2014306084	446	rural/clinic	No	21/02/2017	0	0	0
2014306083	446	rural/clinic	No	21/02/2017	0	0	0
2014306085	446	rural/clinic	No	21/02/2017	0	0	0

2014358323	446	rural/clinic	No	21/02/2017	0	0	0
2012295959	446	rural/clinic	No	21/02/2017	0	0	0
2012295960	446	rural/clinic	No	21/02/2017	0	0	0
2012295961	446	rural/clinic	No	21/02/2017	0	0	0
2014303059	446	rural/clinic	No	21/02/2017	0	0	0
2014303060	446	rural/clinic	No	21/02/2017	0	0	0
2014303061	446	rural/clinic	No	21/02/2017	0	0	0
2014303062	446	rural/clinic	No	21/02/2017	0	0	0
2015382875	446	rural/clinic	No	21/02/2017	0	0	0
2015382876	446	rural/clinic	No	21/02/2017	0	0	0
2015382874	446	rural/clinic	No	21/02/2017	0	0	0
2015382877	446	rural/clinic	No	21/02/2017	0	0	0
2015382878	446	rural/clinic	No	21/02/2017	0	0	0
2012253961	446	rural/clinic	No	21/02/2017	0	0	0
2014356952	446	rural/clinic	No	21/02/2017	0	0	0
2012253960	446	rural/clinic	No	21/02/2017	0	0	0
2014356951/D	446	rural/clinic	No	21/02/2017	0	0	0
2012284985	446	rural/clinic	No	21/02/2017	0	0	0
2012284983	446	rural/clinic	No	21/02/2017	0	0	0
2012284984	446	rural/clinic	No	21/02/2017	0	0	0
2015355378	446	rural/clinic	No	23/02/2017	0	0	0
2015355377	446	rural/clinic	No	23/02/2017	0	0	0
2014328228	418	rural/clinic	No	23/02/2017	0	0	0
2015358296	418	rural/clinic	No	23/02/2017	0	0	0
2015289834	418	rural/clinic	No	23/02/2017	0	0	0
2015358299	418	rural/clinic	No	23/02/2017	0	0	0
2015358298	418	rural/clinic	No	23/02/2017	0	0	0
2015375308	418	rural/clinic	No	23/02/2017	0	0	0
2014342787	418	rural/clinic	No	23/02/2017	0	0	0
2015358297	418	rural/clinic	No	23/02/2017	0	0	0
2015289833	418	rural/clinic	No	23/02/2017	0	0	0
2015402295	418	rural/clinic	No	23/02/2017	0	0	0
2015375963	418	rural/clinic	No	23/02/2017	0	0	0
2015402294	418	rural/clinic	No	23/02/2017	0	0	0
2015375962	418	rural/clinic	No	23/02/2017	0	0	0
2015377024	418	rural/clinic	No	23/02/2017	0	0	0
2015402296	418	rural/clinic	No	23/02/2017	0	0	0
2015375401	418	rural/clinic	No	23/02/2017	0	0	0
2015377258	418	rural/clinic	No	23/02/2017	0	0	0
2015331881	418	rural/clinic	No	23/02/2017	0	0	0
2015402291	418	rural/clinic	No	23/02/2017	0	0	0
2015377259	418	rural/clinic	No	23/02/2017	0	0	0
2014346943	418	rural/clinic	No	23/02/2017	0	0	0
2015402292	418	rural/clinic	No	23/02/2017	0	0	0
2015373343	418	rural/clinic	No	23/02/2017	0	0	0
2014317748	418	rural/clinic	No	23/02/2017	0	0	0
2014317515	418	rural/clinic	No	23/02/2017	0	0	0
2015402293	418	rural/clinic	No	23/02/2017	0	0	0
2015331880	418	rural/clinic	No	23/02/2017	0	0	0
2015360092	418	rural/clinic	No	23/02/2017	0	0	0
2015377025	418	rural/clinic	No	23/02/2017	0	0	0
2015386145	418	rural/clinic	No	23/02/2017	0	0	0
2012390997	418	rural/clinic	No	23/02/2017	0	0	0
2014317517	418	rural/clinic	No	23/02/2017	0	0	0
2015379502	418	rural/clinic	No	23/02/2017	0	0	0
2014317516	418	rural/clinic	No	23/02/2017	0	0	0
2015377317	418	rural/clinic	No	23/02/2017	0	0	0
2015360095	418	rural/clinic	No	23/02/2017	0	0	0
2015335370	418	rural/clinic	No	23/02/2017	0	0	0
2015360094	418	rural/clinic	No	23/02/2017	0	0	0
2011226475	418	rural/clinic	No	23/02/2017	0	0	0
2013274238	418	rural/clinic	No	23/02/2017	0	0	0
2015360096	418	rural/clinic	No	23/02/2017	0	0	0
2014386146	418	rural/clinic	No	23/02/2017	0	0	0
2015368265	418	rural/clinic	No	23/02/2017	0	0	0
2015360097	418	rural/clinic	No	23/02/2017	0	0	0
2015368147	418	rural/clinic	No	23/02/2017	0	0	0
2014386147	418	rural/clinic	No	23/02/2017	0	0	0
2015408109	418	rural/clinic	No	23/02/2017	0	0	0
2015375402	418	rural/clinic	No	23/02/2017	0	0	0
2015368146	418	rural/clinic	No	23/02/2017	0	0	0
2015408108	418	rural/clinic	No	23/02/2017	0	0	0
2015368145	418	rural/clinic	No	23/02/2017	0	0	0
2015375405	418	rural/clinic	No	23/02/2017	0	0	0
2015368144	418	rural/clinic	No	23/02/2017	0	0	0
2011155223	418	rural/clinic	No	23/02/2017	0	0	0
2015408110	418	rural/clinic	No	23/02/2017	0	0	0
2015375404	418	rural/clinic	No	23/02/2017	0	0	0
2015368148	418	rural/clinic	No	23/02/2017	0	0	0
2012389492	418	rural/clinic	No	23/02/2017	0	0	0
2015368149	418	rural/clinic	No	23/02/2017	0	0	0
2015373152	418	rural/clinic	No	23/02/2017	0	0	0

2015372019	418	rural/clinic	No	23/02/2017	0	0	0
2015326295	418	rural/clinic	No	23/02/2017	0	0	0
2015326296	418	rural/clinic	No	23/02/2017	0	0	0
2015373153	418	rural/clinic	No	23/02/2017	0	0	0
2015326297	418	rural/clinic	No	23/02/2017	0	0	0
2015373154	418	rural/clinic	No	23/02/2017	0	0	0
2015326298	418	rural/clinic	No	23/02/2017	0	0	0
2015373155	418	rural/clinic	No	23/02/2017	0	0	0
2015375403	418	rural/clinic	No	23/02/2017	0	0	0
2015358145	418	rural/clinic	No	23/02/2017	0	0	0
2015326299	418	rural/clinic	No	23/02/2017	0	0	0
2015373156	418	rural/clinic	No	23/02/2017	0	0	0
2015326451	418	rural/clinic	No	23/02/2017	0	0	0
2015373157	418	rural/clinic	No	23/02/2017	0	0	0
2015326452	418	rural/clinic	No	23/02/2017	0	0	0
2014356955	418	rural/clinic	No	23/02/2017	0	0	0
2015373158	418	rural/clinic	No	23/02/2017	0	0	0
2015326453	418	rural/clinic	No	23/02/2017	0	0	0
2015402298	418	rural/clinic	No	23/02/2017	0	0	0
2015368204	418	rural/clinic	No	23/02/2017	0	0	0
2015326454	418	rural/clinic	No	23/02/2017	0	0	0
2015368450	418	rural/clinic	No	23/02/2017	0	0	0
2015326456	418	rural/clinic	No	23/02/2017	0	0	0
2014312268	418	rural/clinic	No	23/02/2017	0	0	0
2014356953	418	rural/clinic	No	23/02/2017	0	0	0
2015368448	418	rural/clinic	No	23/02/2017	0	0	0
2015326457	418	rural/clinic	No	23/02/2017	0	0	0
2011113943	418	rural/clinic	No	23/02/2017	0	0	0
2015368443	418	rural/clinic	No	23/02/2017	0	0	0
2015326458	418	rural/clinic	No	23/02/2017	0	0	0
2015368441	418	rural/clinic	No	23/02/2017	0	0	0
2011113942	57	rural/clinic	No	23/02/2017	0	0	0
2015402299	57	rural/clinic	No	23/02/2017	0	0	0
2015326459	57	rural/clinic	No	23/02/2017	0	0	0
2015368440	57	rural/clinic	No	23/02/2017	0	0	0
2015326460	57	rural/clinic	No	23/02/2017	0	0	0
2014356954	57	rural/clinic	No	23/02/2017	0	0	0
2015368439	57	rural/clinic	No	23/02/2017	0	0	0
2015326461	57	rural/clinic	No	23/02/2017	0	0	0
2015368438	57	rural/clinic	No	23/02/2017	0	0	0
2014335224	57	rural/clinic	No	23/02/2017	0	0	0
2015326462	57	rural/clinic	No	23/02/2017	0	0	0
2015331879	57	rural/clinic	No	23/02/2017	0	0	0
2015368437	57	rural/clinic	No	23/02/2017	0	0	0
2015326464	57	rural/clinic	No	23/02/2017	0	0	0
2015368442	57	rural/clinic	No	23/02/2017	0	0	0
2015331878	57	rural/clinic	No	23/02/2017	0	0	0
2015326509	57	rural/clinic	No	23/02/2017	0	0	0
2013325504	57	rural/clinic	No	23/02/2017	0	0	0
2015418642	57	rural/clinic	No	23/02/2017	0	0	0
2015326511	57	rural/clinic	No	23/02/2017	0	0	0
2012265916	57	rural/clinic	No	23/02/2017	0	0	0
2015331902	57	rural/clinic	No	23/02/2017	0	0	0
2014356956	57	rural/clinic	No	23/02/2017	0	0	0
2014371391	57	rural/clinic	No	23/02/2017	0	0	0
2014303495	57	rural/clinic	No	23/02/2017	0	0	0
2014371392	57	rural/clinic	No	23/02/2017	0	0	0
2015325039	57	rural/clinic	No	23/02/2017	0	0	0
2015360586	57	rural/clinic	No	23/02/2017	0	0	0
2014368018	57	rural/clinic	No	23/02/2017	0	0	0
2014368017	57	rural/clinic	No	23/02/2017	0	0	0
2014303289	57	rural/clinic	No	23/02/2017	0	0	0
2015360587	57	rural/clinic	No	23/02/2017	0	0	0
2011179930	57	rural/clinic	No	23/02/2017	0	0	0
2014362806	57	rural/clinic	No	23/02/2017	0	0	0
2011225645	57	rural/clinic	No	23/02/2017	0	0	0
2014332078	57	rural/clinic	No	23/02/2017	0	0	0
2014362805	57	rural/clinic	No	23/02/2017	0	0	0
2015331901	57	rural/clinic	No	23/02/2017	0	0	0
2015349451	57	rural/clinic	No	23/02/2017	0	0	0
2015319596	57	rural/clinic	No	23/02/2017	0	0	0
2014365150	57	rural/clinic	No	23/02/2017	0	0	0
2015349938	57	rural/clinic	No	23/02/2017	0	0	0
2015319597	57	rural/clinic	No	23/02/2017	0	0	0
2015349939	57	rural/clinic	No	23/02/2017	0	0	0
2014362804	57	rural/clinic	No	23/02/2017	0	0	0
2015351148	57	rural/clinic	No	23/02/2017	0	0	0
2014365136	57	rural/clinic	No	23/02/2017	0	0	0
2015319594	57	rural/clinic	No	23/02/2017	0	0	0
2015351149	57	rural/clinic	No	23/02/2017	0	0	0
2015319595	571	rural/clinic	No	23/02/2017	0	0	0
2012384782	571	rural/clinic	No	23/02/2017	0	0	0

2014337038	571	rural/clinic	No	23/02/2017	0	0	0
2014337041	571	rural/clinic	No	23/02/2017	0	0	0
2012312299	571	rural/clinic	No	23/02/2017	0	0	0
2013262212	571	rural/clinic	No	23/02/2017	0	0	0
2011144009	571	rural/clinic	No	23/02/2017	0	0	0
2015324225	571	rural/clinic	No	23/02/2017	0	0	0
2011144012	571	rural/clinic	No	23/02/2017	0	0	0
2015358142	571	rural/clinic	No	23/02/2017	0	0	0
2014335298	571	rural/clinic	No	23/02/2017	0	0	0
2012369965	571	rural/clinic	No	23/02/2017	0	0	0
2015294150	571	rural/clinic	No	23/02/2017	0	0	0
2012265915	571	rural/clinic	No	23/02/2017	0	0	0
2012291838	571	rural/clinic	No	23/02/2017	0	0	0
2012325682	571	rural/clinic	No	23/02/2017	0	0	0
2015358141	571	rural/clinic	No	23/02/2017	0	0	0
2015294149	571	rural/clinic	No	23/02/2017	0	0	0
2015324227	571	rural/clinic	No	23/02/2017	0	0	0
2013266109	571	rural/clinic	No	23/02/2017	0	0	0
2015358143	571	rural/clinic	No	23/02/2017	0	0	0
2015294148	571	rural/clinic	No	23/02/2017	0	0	0
2014300532	571	rural/clinic	No	23/02/2017	0	0	0
2012325683	571	rural/clinic	No	23/02/2017	0	0	0
2015358144	571	rural/clinic	No	23/02/2017	0	0	0
2012291839	571	rural/clinic	No	23/02/2017	0	0	0
2011144109	571	rural/clinic	No	23/02/2017	0	0	0
2012325681	199	rural/clinic	No	23/02/2017	0	0	0
2014386148	199	rural/clinic	No	23/02/2017	0	0	0
2014329138	199	rural/clinic	No	23/02/2017	0	0	0
2012283591	199	rural/clinic	No	23/02/2017	0	0	0
2014386149	199	rural/clinic	No	23/02/2017	0	0	0
2014385687	199	rural/clinic	No	23/02/2017	0	0	0
2012325684	199	rural/clinic	No	23/02/2017	0	0	0
2015352430	199	rural/clinic	No	23/02/2017	0	0	0
2014386150	199	rural/clinic	No	23/02/2017	0	0	0
2013252934	199	rural/clinic	No	23/02/2017	0	0	0
2014369034	199	rural/clinic	No	23/02/2017	0	0	0
2015402501	199	rural/clinic	No	23/02/2017	0	0	0
2012387249	199	rural/clinic	No	23/02/2017	0	0	0
2015402502	199	rural/clinic	No	23/02/2017	0	0	0
2014372329	199	rural/clinic	No	23/02/2017	0	0	0
2015373390	199	rural/clinic	No	23/02/2017	0	0	0
2013253026	199	rural/clinic	No	23/02/2017	0	0	0
2013264672	199	rural/clinic	No	23/02/2017	0	0	0
2015418708	199	rural/clinic	No	23/02/2017	0	0	0
2015369488	199	rural/clinic	No	09/02/2017	0	0	0
2015369488	199	rural/clinic	No	09/02/2017	0	0	0
2015358285	199	rural/clinic	No	09/02/2017	0	0	0
2015358285	199	rural/clinic	No	09/02/2017	0	0	0
2014303053	199	rural/clinic	No	09/02/2017	0	0	0
2014303053	199	rural/clinic	No	09/02/2017	0	0	0
2015301441	199	rural/clinic	No	09/02/2017	0	0	0
2015382870	199	rural/clinic	No	09/02/2017	0	0	0
2015382870	199	rural/clinic	No	09/02/2017	0	0	0
2015331661	199	rural/clinic	No	09/02/2017	0	0	0
2015331661	199	rural/clinic	No	09/02/2017	0	0	0
2015338452	199	rural/clinic	No	09/02/2017	0	0	0
2015338452	199	rural/clinic	No	09/02/2017	0	0	0
2015368202	199	rural/clinic	No	09/02/2017	0	0	0
2015368202	199	rural/clinic	No	09/02/2017	0	0	0
2011181147	199	rural/clinic	No	09/02/2017	0	0	0
2015384027	199	rural/clinic	No	09/02/2017	0	0	0
2015369376	199	rural/clinic	No	09/02/2017	0	0	0
2015369376	199	rural/clinic	No	09/02/2017	0	0	0
2015373391	199	rural/clinic	No	23/02/2017	0	0	0
2014357511	199	rural/clinic	No	09/02/2017	0	0	0
2014357511	200	district/faith-based	No	09/02/2017	1	0	0
2015373392	200	district/faith-based	No	23/02/2017	1	0	0
2015351140	200	district/faith-based	No	09/02/2017	1	0	0
2015351140	200	district/faith-based	No	09/02/2017	1	0	0
2015324226	200	district/faith-based	No	23/02/2017	1	0	0
2015373393	200	district/faith-based	No	23/02/2017	1	0	0
2015373394	200	district/faith-based	No	23/02/2017	1	0	0
2015373652	200	district/faith-based	No	23/02/2017	1	0	0
2015373653	200	district/faith-based	No	23/02/2017	1	0	0
2015340484	200	district/faith-based	No	23/02/2017	1	0	0
2015373654	200	district/faith-based	No	23/02/2017	1	0	0
2015340485	200	district/faith-based	No	23/02/2017	1	0	0
2015402503	200	district/faith-based	No	23/02/2017	1	0	0
2015340483	200	district/faith-based	No	23/02/2017	1	0	0
2015402504	200	district/faith-based	No	23/02/2017	1	0	0
2015358295	200	district/faith-based	No	23/02/2017	1	0	0
2014302512	200	district/faith-based	No	23/02/2017	1	0	0

2014302513	200	district/faith-based	No	23/02/2017	1	0	0
2015340486	200	district/faith-based	No	24/02/2017	1	0	0
2015386351	200	district/faith-based	No	23/02/2017	1	0	0
2013283501	200	district/faith-based	No	23/02/2017	1	0	0
2015358300	200	district/faith-based	No	23/02/2017	1	0	0
2014329137	200	district/faith-based	No	23/02/2017	1	0	0
2015286279	200	district/faith-based	No	23/02/2017	1	0	0
2015373651	200	district/faith-based	No	23/02/2017	1	0	0
2012331322	200	district/faith-based	No	23/02/2017	1	0	0
2015286280	200	district/faith-based	No	23/02/2017	1	0	0
2012331321	200	district/faith-based	No	23/02/2017	1	0	0
2014319494	200	district/faith-based	No	23/02/2017	1	0	0
2014317619	200	district/faith-based	No	23/02/2017	1	0	0
2014317618	200	district/faith-based	No	23/02/2017	1	0	0
2014317617	200	district/faith-based	No	23/02/2017	1	0	0
2015378948	200	district/faith-based	No	23/02/2017	1	0	0
2014317429	200	district/faith-based	No	23/02/2017	1	0	0
2013283245	200	district/faith-based	No	23/02/2017	1	0	0
2015286262	200	district/faith-based	No	23/02/2017	1	0	0
2013283244	200	district/faith-based	No	23/02/2017	1	0	0
2015413103	200	district/faith-based	No	23/02/2017	1	0	0
2013267888	200	district/faith-based	No	23/02/2017	1	0	0
2015413104	200	district/faith-based	No	24/02/2017	1	0	0
2013267889	200	district/faith-based	No	23/02/2017	1	0	0
2013267887	200	district/faith-based	No	23/02/2017	1	0	0
2015413105	200	district/faith-based	No	23/02/2017	1	0	0
2015384029	200	district/faith-based	No	23/02/2017	1	0	0
2015413102	200	district/faith-based	No	23/02/2017	1	0	0
2015360093	200	district/faith-based	No	23/02/2017	1	0	0
2015373655	200	district/faith-based	No	23/02/2017	1	0	0
2015377023	200	district/faith-based	No	23/02/2017	1	0	0
2015373657	200	district/faith-based	No	23/02/2017	1	0	0
2015373658	200	district/faith-based	No	23/02/2017	1	0	0
2015352994	200	district/faith-based	No	23/02/2017	1	0	0
2015352995	200	district/faith-based	No	23/02/2017	1	0	0
2015352996	200	district/faith-based	No	23/02/2017	1	0	0
2015352997	200	district/faith-based	No	23/02/2017	1	0	0
2012359901	200	district/faith-based	No	23/02/2017	1	0	0
2015352998	200	district/faith-based	No	23/02/2017	1	0	0
2012359902	200	district/faith-based	No	23/02/2017	1	0	0
2015352999	200	district/faith-based	No	23/02/2017	1	0	0
2015353000	200	district/faith-based	No	23/02/2017	1	0	0
2015305059	200	district/faith-based	No	23/02/2017	1	0	0
2015305247	200	district/faith-based	No	23/02/2017	1	0	0
2013258726	200	district/faith-based	No	23/02/2017	1	0	0
2015319593	200	district/faith-based	No	23/02/2017	1	0	0
2014373207	200	district/faith-based	No	23/02/2017	1	0	0
2014365141	200	district/faith-based	No	23/02/2017	1	0	0
2014365139	200	district/faith-based	No	23/02/2017	1	0	0
2014365140	200	district/faith-based	No	23/02/2017	1	0	0
2011199074	200	district/faith-based	No	23/02/2017	1	0	0
2015373656	200	district/faith-based	No	23/02/2017	1	0	0
2014291852	200	district/faith-based	No	23/02/2017	1	0	0
2015351150	200	district/faith-based	No	23/02/2017	1	0	0
2014291851	200	district/faith-based	No	23/02/2017	1	0	0
20170000001	200	district/faith-based	No	23/02/2017	1	0	0
20170000002	200	district/faith-based	No	23/02/2017	1	0	0
20170000003	200	district/faith-based	No	23/02/2017	1	0	0
2015351954	200	district/faith-based	No	24/02/2017	1	0	0
2015351953	200	district/faith-based	No	24/02/2017	1	0	0
2015351952	200	district/faith-based	No	24/02/2017	1	0	0
2015351951	200	district/faith-based	No	24/02/2017	1	0	0
2015351894	200	district/faith-based	No	24/02/2017	1	0	0
2015351900	200	district/faith-based	No	24/02/2017	1	0	0
2015351899	200	district/faith-based	No	24/02/2017	1	0	0
2015351898	200	district/faith-based	No	24/02/2017	1	0	0
2015351897	200	district/faith-based	No	24/02/2017	1	0	0
2015351896	200	district/faith-based	No	24/02/2017	1	0	0
2015351895	200	district/faith-based	No	24/02/2017	1	0	0
2015322028	200	district/faith-based	No	24/02/2017	1	0	0
2012349263	200	district/faith-based	No	24/02/2017	1	0	0
2015317614	200	district/faith-based	No	24/02/2017	1	0	0
2012364940	200	district/faith-based	No	24/02/2017	1	0	0
2013253936	200	district/faith-based	No	24/02/2017	1	0	0
2015368867	200	district/faith-based	No	24/02/2017	1	0	0
2013253937	200	district/faith-based	No	24/02/2017	1	0	0
2015368865	200	district/faith-based	No	24/02/2017	1	0	0
2015368866	200	district/faith-based	No	24/02/2017	1	0	0
2015416512	200	district/faith-based	No	24/02/2017	1	0	0
2015416513	200	district/faith-based	No	24/02/2017	1	0	0
2015416514	200	district/faith-based	No	24/02/2017	1	0	0
2015328515	200	district/faith-based	No	24/02/2017	1	0	0

2015418637	200	district/faith-based	No	24/02/2017	1	0	0
2015418638	200	district/faith-based	No	24/02/2017	1	0	0
2015418640	200	district/faith-based	No	24/02/2017	1	0	0
2015418641	200	district/faith-based	No	24/02/2017	1	0	0
2013251312	200	district/faith-based	No	15/02/2017	1	0	0
2013251312	200	district/faith-based	No	15/02/2017	1	0	0
2014329245	200	district/faith-based	No	24/02/2017	1	0	0
2014329250	200	district/faith-based	No	24/02/2017	1	0	0
2015322724	200	district/faith-based	No	24/02/2017	1	0	0
2015322726	200	district/faith-based	No	24/02/2017	1	0	0
2011156599	200	district/faith-based	No	14/02/2017	1	0	0
2011156599	200	district/faith-based	No	14/02/2017	1	0	0
2015385384	200	district/faith-based	No	14/02/2017	1	0	0
2015385384	200	district/faith-based	No	14/02/2017	1	0	0
2015385932	200	district/faith-based	No	14/02/2017	1	0	0
2015385932	200	district/faith-based	No	14/02/2017	1	0	0
2015322568	200	district/faith-based	No	24/02/2017	1	0	0
2015322722	200	district/faith-based	No	24/02/2017	1	0	0
2012352342	200	district/faith-based	No	14/02/2017	1	0	0
2012352342	200	district/faith-based	No	14/02/2017	1	0	0
2012267310	200	district/faith-based	No	14/02/2017	1	0	0
2012267310	200	district/faith-based	No	14/02/2017	1	0	0
2012267311	200	district/faith-based	No	14/02/2017	1	0	0
2012305967	200	district/faith-based	No	14/02/2017	1	0	0
2012305967	200	district/faith-based	No	14/02/2017	1	0	0
2015397500	200	district/faith-based	No	13/02/2017	1	0	0
2015397500	200	district/faith-based	No	13/02/2017	1	0	0
2015335419	200	district/faith-based	No	14/02/2017	1	0	0
2015335419	200	district/faith-based	No	14/02/2017	1	0	0
2015322723	200	district/faith-based	No	24/02/2017	1	0	0
2015414927	200	district/faith-based	No	14/02/2017	1	0	0
2015414927	200	district/faith-based	No	14/02/2017	1	0	0
2015322024	200	district/faith-based	No	24/02/2017	1	0	0
2014375656	200	district/faith-based	No	14/02/2017	1	0	0
2014375656	200	district/faith-based	No	14/02/2017	1	0	0
2015322026	200	district/faith-based	No	24/02/2017	1	0	0
2015322725	200	district/faith-based	No	24/02/2017	1	0	0
2015322027	200	district/faith-based	No	24/02/2017	1	0	0
2013253938	200	district/faith-based	No	24/02/2017	1	0	0
2015354459	200	district/faith-based	No	24/02/2017	1	0	0
2015354460	200	district/faith-based	No	24/02/2017	1	0	0
2015354461	200	district/faith-based	No	24/02/2017	1	0	0
2015354462	200	district/faith-based	No	24/02/2017	1	0	0
2015354463	200	district/faith-based	No	24/02/2017	1	0	0
2015354464	200	district/faith-based	No	24/02/2017	1	0	0
2015354465	200	district/faith-based	No	24/02/2017	1	0	0
2015354466	200	district/faith-based	No	24/02/2017	1	0	0
2015354467	200	district/faith-based	No	24/02/2017	1	0	0
2015305057	58	rural/clinic	No	16/02/2017	0	0	0
2015305056	58	rural/clinic	No	16/02/2017	0	0	0
2015305056	58	rural/clinic	No	16/02/2017	0	0	0
2015373341	58	rural/clinic	No	16/02/2017	0	0	0
2015368433	58	rural/clinic	No	15/02/2017	0	0	0
2015368433	58	rural/clinic	No	15/02/2017	0	0	0
2012242576	58	rural/clinic	No	14/02/2017	0	0	0
2012242576	58	rural/clinic	No	14/02/2017	0	0	0
2015351142	58	rural/clinic	No	14/02/2017	0	0	0
2015351142	58	rural/clinic	No	14/02/2017	0	0	0
2014314539	58	rural/clinic	No	21/02/2017	0	0	0
2014314539	58	rural/clinic	No	21/02/2017	0	0	0
2015335421	58	rural/clinic	No	21/02/2017	0	0	0
2015335421	58	rural/clinic	No	21/02/2017	0	0	0
2012353425	58	rural/clinic	No	21/02/2017	0	0	0
2012353425	58	rural/clinic	No	21/02/2017	0	0	0
2012268780	58	rural/clinic	No	21/02/2017	0	0	0
2012268780	58	rural/clinic	No	21/02/2017	0	0	0
2014342505	58	rural/clinic	No	21/02/2017	0	0	0
2014342505	58	rural/clinic	No	21/02/2017	0	0	0
2013256395	58	rural/clinic	No	21/02/2017	0	0	0
2013256395	58	rural/clinic	No	21/02/2017	0	0	0
2015378721	58	rural/clinic	No	21/02/2017	0	0	0
2015378721	58	rural/clinic	No	21/02/2017	0	0	0
2015358486	58	rural/clinic	No	21/02/2017	0	0	0
2015358486	58	rural/clinic	No	21/02/2017	0	0	0
2015358482	58	rural/clinic	No	21/02/2017	0	0	0
2015358482	58	rural/clinic	No	21/02/2017	0	0	0
2015374329	58	rural/clinic	No	20/02/2017	0	0	0
2015374329	58	rural/clinic	No	20/02/2017	0	0	0
2012336602	58	rural/clinic	No	20/02/2017	0	0	0
2012336602	58	rural/clinic	No	20/02/2017	0	0	0
2015351144	58	rural/clinic	No	20/02/2017	0	0	0
2015351144	58	rural/clinic	No	20/02/2017	0	0	0

2015351143	58	rural/clinic	No	20/02/2017	0	0	0
2015351143	58	rural/clinic	No	20/02/2017	0	0	0
2015351145	58	rural/clinic	No	20/02/2017	0	0	0
2015351145	58	rural/clinic	No	20/02/2017	0	0	0
2015293638	58	rural/clinic	No	16/02/2017	0	0	0
2015293638	58	rural/clinic	No	16/02/2017	0	0	0
2011145391	58	rural/clinic	No	16/02/2017	0	0	0
2011145391	58	rural/clinic	No	16/02/2017	0	0	0
2011240234	58	rural/clinic	No	16/02/2017	0	0	0
2011240234	58	rural/clinic	No	16/02/2017	0	0	0
2012283587	58	rural/clinic	No	16/02/2017	0	0	0
2012283587	58	rural/clinic	No	16/02/2017	0	0	0
2015363004	58	rural/clinic	No	28/02/2017	0	0	0
2015392537	58	rural/clinic	No	28/02/2017	0	0	0
2014338248	58	rural/clinic	No	16/02/2017	0	0	0
2014338248	58	rural/clinic	No	16/02/2017	0	0	0
2013255445	58	rural/clinic	No	16/02/2017	0	0	0
2013255445	58	rural/clinic	No	16/02/2017	0	0	0
2014350296	58	rural/clinic	No	28/02/2017	0	0	0
2014350363	58	rural/clinic	No	28/02/2017	0	0	0
2015383584	58	rural/clinic	No	20/02/2017	0	0	0
2015383584	58	rural/clinic	No	20/02/2017	0	0	0
2014301455	58	rural/clinic	No	20/02/2017	0	0	0
2014301455	58	rural/clinic	No	20/02/2017	0	0	0
2012269011	58	rural/clinic	No	20/02/2017	0	0	0
2012269011	58	rural/clinic	No	20/02/2017	0	0	0
2011152129	58	rural/clinic	No	23/02/2017	0	0	0
2015392542	58	rural/clinic	No	28/02/2017	0	0	0
2014314715	322	rural/clinic	No	01/03/2017	0	0	0
2015392545	322	rural/clinic	No	28/02/2017	0	0	0
2014350362	322	rural/clinic	No	28/02/2017	0	0	0
2014314717	322	rural/clinic	No	01/03/2017	0	0	0
2014326749	322	rural/clinic	No	28/02/2017	0	0	0
2014314716	322	rural/clinic	No	01/03/2017	0	0	0
2014326748	322	rural/clinic	No	28/02/2017	0	0	0
2015328458	322	rural/clinic	No	28/02/2017	0	0	0
2015331905	322	rural/clinic	No	28/02/2017	0	0	0
2014326747	322	rural/clinic	No	28/02/2017	0	0	0
2014337218	322	rural/clinic	No	01/03/2017	0	0	0
2015331904	322	rural/clinic	No	28/02/2017	0	0	0
2014326744	322	rural/clinic	No	28/02/2017	0	0	0
2015392543	689	rural/clinic	No	28/02/2017	0	0	0
2014360342	689	rural/clinic	No	28/02/2017	0	0	0
2015392547	689	rural/clinic	No	28/02/2017	0	0	0
2014336896	689	rural/clinic	No	01/03/2017	0	0	0
2014327664	689	rural/clinic	No	28/02/2017	0	0	0
2014326742	689	rural/clinic	No	28/02/2017	0	0	0
2015392538	689	rural/clinic	No	28/02/2017	0	0	0
2015413891	689	rural/clinic	No	28/02/2017	0	0	0
2014331608	689	rural/clinic	No	28/02/2017	0	0	0
2014336897	689	rural/clinic	No	28/02/2017	0	0	0
2015293645	611	rural/clinic	No	28/02/2017	0	0	0
2015301445	611	rural/clinic	No	28/02/2017	0	0	0
2015293829	611	rural/clinic	No	28/02/2017	0	0	0
2015334081	611	rural/clinic	No	21/02/2017	0	0	0
2015334081	611	rural/clinic	No	21/02/2017	0	0	0
2014344463	611	rural/clinic	No	21/02/2017	0	0	0
2014344463	611	rural/clinic	No	21/02/2017	0	0	0
2015293830	611	rural/clinic	No	28/02/2017	0	0	0
2012306447	611	rural/clinic	No	28/02/2017	0	0	0
2012330880	611	rural/clinic	No	28/02/2017	0	0	0
2012290396	611	rural/clinic	No	28/02/2017	0	0	0
2015294046	611	rural/clinic	No	28/02/2017	0	0	0
2015413101	611	rural/clinic	No	21/02/2017	0	0	0
2015413101	611	rural/clinic	No	21/02/2017	0	0	0
2015357631	611	rural/clinic	No	28/02/2017	0	0	0
2011199240	108	rural/clinic	No	21/02/2017	0	0	0
2011199240	108	rural/clinic	No	21/02/2017	0	0	0
2012306342	108	rural/clinic	No	28/02/2017	0	0	0
2015337029	108	rural/clinic	No	28/02/2017	0	0	0
2012290397	108	rural/clinic	No	28/02/2017	0	0	0
2011134955	108	rural/clinic	No	21/02/2017	0	0	0
2011134955	108	rural/clinic	No	21/02/2017	0	0	0
2015297368	108	rural/clinic	No	28/02/2017	0	0	0
2015362674	108	rural/clinic	No	28/02/2017	0	0	0
2015362675	108	rural/clinic	No	28/02/2017	0	0	0
2015331881	108	rural/clinic	No	23/02/2017	0	0	0
2015331881	108	rural/clinic	No	23/02/2017	0	0	0
2015344851	108	rural/clinic	No	28/02/2017	0	0	0
2015297364	108	rural/clinic	No	28/02/2017	0	0	0
2015362673	108	rural/clinic	No	28/02/2017	0	0	0
2014328431	108	rural/clinic	No	28/02/2017	0	0	0

2015314795	108	rural/clinic	No	28/02/2017	0	0	0
2012340794	108	rural/clinic	No	28/02/2017	0	0	0
2014319043	108	rural/clinic	No	28/02/2017	0	0	0
2015314796	108	rural/clinic	No	28/02/2017	0	0	0
2015377861	108	rural/clinic	No	28/02/2017	0	0	0
2012272332	108	rural/clinic	No	28/02/2017	0	0	0
2015314797	108	rural/clinic	No	28/02/2017	0	0	0
2015382367	108	rural/clinic	No	28/02/2017	0	0	0
2012272334	108	rural/clinic	No	28/02/2017	0	0	0
2014330642	108	rural/clinic	No	28/02/2017	0	0	0
2015383587	108	rural/clinic	No	28/02/2017	0	0	0
2015314798	108	rural/clinic	No	28/02/2017	0	0	0
2012272333	108	rural/clinic	No	28/02/2017	0	0	0
2015383591	108	rural/clinic	No	28/02/2017	0	0	0
2015286864	108	rural/clinic	No	28/02/2017	0	0	0
2015337030	108	rural/clinic	No	28/02/2017	0	0	0
2014330641	108	rural/clinic	No	28/02/2017	0	0	0
2015397510	108	rural/clinic	No	28/02/2017	0	0	0
2015383588	108	rural/clinic	No	28/02/2017	0	0	0
2015397511	108	rural/clinic	No	28/02/2017	0	0	0
2015337034	108	rural/clinic	No	28/02/2017	0	0	0
2015382368	108	rural/clinic	No	28/02/2017	0	0	0
2015397512	108	rural/clinic	No	28/02/2017	0	0	0
2015337031	108	rural/clinic	No	28/02/2017	0	0	0
2015333252	108	rural/clinic	No	28/02/2017	0	0	0
2015397513	108	rural/clinic	No	28/02/2017	0	0	0
2012247406	108	rural/clinic	No	28/02/2017	0	0	0
2015337033	108	rural/clinic	No	28/02/2017	0	0	0
2015328456	108	rural/clinic	No	28/02/2017	0	0	0
2011134958	108	rural/clinic	No	28/02/2017	0	0	0
2014318846	108	rural/clinic	No	28/02/2017	0	0	0
2011134959	108	rural/clinic	No	28/02/2017	0	0	0
2014318843	108	rural/clinic	No	28/02/2017	0	0	0
2015413690	108	rural/clinic	No	28/02/2017	0	0	0
2015337032	108	rural/clinic	No	28/02/2017	0	0	0
2015413687	108	rural/clinic	No	28/02/2017	0	0	0
2015299773	108	rural/clinic	No	28/02/2017	0	0	0
2015299772	108	rural/clinic	No	28/02/2017	0	0	0
2014353628	108	rural/clinic	No	28/02/2017	0	0	0
2015295345	108	rural/clinic	No	28/02/2017	0	0	0
2015295344	108	rural/clinic	No	28/02/2017	0	0	0
2015316956	108	rural/clinic	No	28/02/2017	0	0	0
2014353626	108	rural/clinic	No	28/02/2017	0	0	0
2015299770	108	rural/clinic	No	28/02/2017	0	0	0
2015392548	108	rural/clinic	No	28/02/2017	0	0	0
2015392546	108	rural/clinic	No	28/02/2017	0	0	0
2014291543	108	rural/clinic	No	28/02/2017	0	0	0
2015315982	108	rural/clinic	No	28/02/2017	0	0	0
2015383589	108	rural/clinic	No	28/02/2017	0	0	0
2015288742	108	rural/clinic	No	28/02/2017	0	0	0
2014318844	108	rural/clinic	No	28/02/2017	0	0	0
2015297367	108	rural/clinic	No	28/02/2017	0	0	0
2015337612	108	rural/clinic	No	28/02/2017	0	0	0
2015297366	108	rural/clinic	No	28/02/2017	0	0	0
2014289495	108	rural/clinic	No	28/02/2017	0	0	0
2015355510	108	rural/clinic	No	28/02/2017	0	0	0
2015344069	108	rural/clinic	No	28/02/2017	0	0	0
2014289489	108	rural/clinic	No	28/02/2017	0	0	0
2014318845	108	rural/clinic	No	28/02/2017	0	0	0
2015413980	108	rural/clinic	No	28/02/2017	0	0	0
2012344493	108	rural/clinic	No	28/02/2017	0	0	0
2015301589	108	rural/clinic	No	28/02/2017	0	0	0
2015413634	108	rural/clinic	No	28/02/2017	0	0	0
2015324425	108	rural/clinic	No	28/02/2017	0	0	0
2014289487	108	rural/clinic	No	28/02/2017	0	0	0
2015413635	108	rural/clinic	No	28/02/2017	0	0	0
2014315523	108	rural/clinic	No	28/02/2017	0	0	0
2014289488	108	rural/clinic	No	28/02/2017	0	0	0
2015413637	108	rural/clinic	No	28/02/2017	0	0	0
2015392544	791	rural/clinic	No	28/02/2017	0	0	0
2015324427	447	rural/clinic	No	28/02/2017	0	0	0
2015413638	447	rural/clinic	No	28/02/2017	0	0	0
2015361705	447	rural/clinic	No	28/02/2017	0	0	0
2015385437	447	rural/clinic	No	28/02/2017	0	0	0
2015413636	447	rural/clinic	No	28/02/2017	0	0	0
2015385436	447	rural/clinic	No	28/02/2017	0	0	0
2012284571	447	rural/clinic	No	28/02/2017	0	0	0
2014289492	447	rural/clinic	No	28/02/2017	0	0	0
2014370215	447	rural/clinic	No	28/02/2017	0	0	0
2010099622	447	rural/clinic	No	28/02/2017	0	0	0
2013254802	447	rural/clinic	No	28/02/2017	0	0	0
2015300671	447	rural/clinic	No	28/02/2017	0	0	0

2015324465	447	rural/clinic	No	28/02/2017	0	0	0
2015300669	447	rural/clinic	No	28/02/2017	0	0	0
2012377875	447	rural/clinic	No	28/02/2017	0	0	0
2015300670	447	rural/clinic	No	28/02/2017	0	0	0
2014289494/D	447	rural/clinic	No	28/02/2017	0	0	0
2011193476	447	rural/clinic	No	28/02/2017	0	0	0
2012305693	447	rural/clinic	No	28/02/2017	0	0	0
2015328454	447	rural/clinic	No	28/02/2017	0	0	0
2013266239	447	rural/clinic	No	28/02/2017	0	0	0
2015289321	447	rural/clinic	No	28/02/2017	0	0	0
2013266241	447	rural/clinic	No	28/02/2017	0	0	0
2015338707	447	rural/clinic	No	28/02/2017	0	0	0
2015328455	447	rural/clinic	No	28/02/2017	0	0	0
2015299768	447	rural/clinic	No	28/02/2017	0	0	0
2015340055	447	rural/clinic	No	28/02/2017	0	0	0
2014291616	447	rural/clinic	No	28/02/2017	0	0	0
2015355509	447	rural/clinic	No	28/02/2017	0	0	0
2015335574	447	rural/clinic	No	28/02/2017	0	0	0
2015299769	447	rural/clinic	No	28/02/2017	0	0	0
2014291617	447	rural/clinic	No	28/02/2017	0	0	0
2013256393	447	rural/clinic	No	28/02/2017	0	0	0
2015331764	447	rural/clinic	No	28/02/2017	0	0	0
2015324426	447	rural/clinic	No	28/02/2017	0	0	0
2015342304	447	rural/clinic	No	28/02/2017	0	0	0
2014322427	447	rural/clinic	No	28/02/2017	0	0	0
2015359836	447	rural/clinic	No	28/02/2017	0	0	0
2013256396	447	rural/clinic	No	28/02/2017	0	0	0
2013271834	447	rural/clinic	No	28/02/2017	0	0	0
2015384773	447	rural/clinic	No	28/02/2017	0	0	0
2014350092	447	rural/clinic	No	28/02/2017	0	0	0
2015289322	447	rural/clinic	No	28/02/2017	0	0	0
2012263920	447	rural/clinic	No	28/02/2017	0	0	0
2015357373	447	rural/clinic	No	28/02/2017	0	0	0
2015359837	447	rural/clinic	No	28/02/2017	0	0	0
2012276146	447	rural/clinic	No	28/02/2017	0	0	0
2015340056	447	rural/clinic	No	28/02/2017	0	0	0
2015331765	447	rural/clinic	No	28/02/2017	0	0	0
2015340053	447	rural/clinic	No	28/02/2017	0	0	0
2015331761	447	rural/clinic	No	28/02/2017	0	0	0
2015357372	447	rural/clinic	No	28/02/2017	0	0	0
2012253200	447	rural/clinic	No	28/02/2017	0	0	0
2015359752	447	rural/clinic	No	28/02/2017	0	0	0
2014327112	447	rural/clinic	No	28/02/2017	0	0	0
2015331762	447	rural/clinic	No	28/02/2017	0	0	0
2015325927	447	rural/clinic	No	28/02/2017	0	0	0
2015376104	447	rural/clinic	No	28/02/2017	0	0	0
2015331763	447	rural/clinic	No	28/02/2017	0	0	0
2013271836	447	rural/clinic	No	28/02/2017	0	0	0
2015377624	447	rural/clinic	No	28/02/2017	0	0	0
2015377623	447	rural/clinic	No	28/02/2017	0	0	0
2015418126	447	rural/clinic	No	28/02/2017	0	0	0
2012295965	447	rural/clinic	No	28/02/2017	0	0	0
2015377622	447	rural/clinic	No	28/02/2017	0	0	0
2015377621	447	rural/clinic	No	28/02/2017	0	0	0
2012295964	447	rural/clinic	No	28/02/2017	0	0	0
2015377620	447	rural/clinic	No	28/02/2017	0	0	0
2012295963	447	rural/clinic	No	28/02/2017	0	0	0
2015352355	447	rural/clinic	No	28/02/2017	0	0	0
2015352356	447	rural/clinic	No	28/02/2017	0	0	0
2015414936	447	rural/clinic	No	28/02/2017	0	0	0
2012295962	447	rural/clinic	No	28/02/2017	0	0	0
2015418125	447	rural/clinic	No	28/02/2017	0	0	0
2015413546	447	rural/clinic	No	28/02/2017	0	0	0
2015414935	447	rural/clinic	No	28/02/2017	0	0	0
2015368778	447	rural/clinic	No	28/02/2017	0	0	0
2015418123	447	rural/clinic	No	28/02/2017	0	0	0
2014303067	447	rural/clinic	No	28/02/2017	0	0	0
2014303066	447	rural/clinic	No	28/02/2017	0	0	0
2014303065	447	rural/clinic	No	28/02/2017	0	0	0
2015368781	447	rural/clinic	No	28/02/2017	0	0	0
2015418124	447	rural/clinic	No	28/02/2017	0	0	0
2014303064	447	rural/clinic	No	28/02/2017	0	0	0
2015368779	447	rural/clinic	No	28/02/2017	0	0	0
2014375662	447	rural/clinic	No	28/02/2017	0	0	0
2012246184	447	rural/clinic	No	28/02/2017	0	0	0
2015355773	447	rural/clinic	No	28/02/2017	0	0	0
2014342509	447	rural/clinic	No	28/02/2017	0	0	0
2015355772	447	rural/clinic	No	28/02/2017	0	0	0
2014344334	447	rural/clinic	No	28/02/2017	0	0	0
2015368780	447	rural/clinic	No	28/02/2017	0	0	0
2015368777	447	rural/clinic	No	28/02/2017	0	0	0
2014370426	447	rural/clinic	No	28/02/2017	0	0	0

2014344333	447	rural/clinic	No	28/02/2017	0	0	0
2014370425	447	rural/clinic	No	28/02/2017	0	0	0
2014344335	447	rural/clinic	No	28/02/2017	0	0	0
2014344336	447	rural/clinic	No	28/02/2017	0	0	0
2012290683	447	rural/clinic	No	28/02/2017	0	0	0
2012290682	447	rural/clinic	No	28/02/2017	0	0	0
2015382881	836	rural/clinic	No	28/02/2017	0	0	0
2015382882	836	rural/clinic	No	28/02/2017	0	0	0
2014331606	836	rural/clinic	No	28/02/2017	0	0	0
2015382879	836	rural/clinic	No	28/02/2017	0	0	0
2014303063	836	rural/clinic	No	28/02/2017	0	0	0
2015388503	836	rural/clinic	No	28/02/2017	0	0	0
2014331607	836	rural/clinic	No	28/02/2017	0	0	0
2014375663	836	rural/clinic	No	28/02/2017	0	0	0
2015369916	836	rural/clinic	No	28/02/2017	0	0	0
2015369915	836	rural/clinic	No	28/02/2017	0	0	0
2014289491	836	rural/clinic	No	28/02/2017	0	0	0
2014289490	836	rural/clinic	No	28/02/2017	0	0	0
2014289493	836	rural/clinic	No	28/02/2017	0	0	0
2012363953	836	rural/clinic	No	28/02/2017	0	0	0
2015331903	836	rural/clinic	No	28/02/2017	0	0	0
2012363954	836	rural/clinic	No	28/02/2017	0	0	0
2012363955	836	rural/clinic	No	28/02/2017	0	0	0
2015369499	836	rural/clinic	No	28/02/2017	0	0	0
2014355898	836	rural/clinic	No	28/02/2017	0	0	0
2012363278	836	rural/clinic	No	28/02/2017	0	0	0
2014358683	836	rural/clinic	No	28/02/2017	0	0	0
2014355899	836	rural/clinic	No	28/02/2017	0	0	0
2012267314	836	rural/clinic	No	28/02/2017	0	0	0
2015337356	836	rural/clinic	No	28/02/2017	0	0	0
2015392448	836	rural/clinic	No	28/02/2017	0	0	0
2011204760	836	rural/clinic	No	28/02/2017	0	0	0
2011204767	836	rural/clinic	No	28/02/2017	0	0	0
2013278289	836	rural/clinic	No	28/02/2017	0	0	0
2015324034	836	rural/clinic	No	28/02/2017	0	0	0
2014344337	836	rural/clinic	No	28/02/2017	0	0	0
2015286526	836	rural/clinic	No	28/02/2017	0	0	0
2012345096	836	rural/clinic	No	28/02/2017	0	0	0
2014357303	836	rural/clinic	No	28/02/2017	0	0	0
2012344492	836	rural/clinic	No	28/02/2017	0	0	0
2015297071	836	rural/clinic	No	28/02/2017	0	0	0
2015339518	836	rural/clinic	No	28/02/2017	0	0	0
2015339510	836	rural/clinic	No	28/02/2017	0	0	0
2013256260	836	rural/clinic	No	28/02/2017	0	0	0
2015297359	836	rural/clinic	No	28/02/2017	0	0	0
2015368560	836	rural/clinic	No	28/02/2017	0	0	0
2015339511	836	rural/clinic	No	28/02/2017	0	0	0
2015339512	836	rural/clinic	No	28/02/2017	0	0	0
2015297358	836	rural/clinic	No	28/02/2017	0	0	0
2015339513	836	rural/clinic	No	28/02/2017	0	0	0
2015339514	836	rural/clinic	No	28/02/2017	0	0	0
2015339515	836	rural/clinic	No	28/02/2017	0	0	0
2015339516	836	rural/clinic	No	28/02/2017	0	0	0
2015297076	836	rural/clinic	No	28/02/2017	0	0	0
2015339517	836	rural/clinic	No	28/02/2017	0	0	0
2015293646	859	rural/clinic	No	28/02/2017	0	0	0
2014360339	859	rural/clinic	No	28/02/2017	0	0	0
2015297074	859	rural/clinic	No	28/02/2017	0	0	0
2014360340	859	rural/clinic	No	28/02/2017	0	0	0
2015297360	859	rural/clinic	No	28/02/2017	0	0	0
2014360343	859	rural/clinic	No	28/02/2017	0	0	0
2014357302	859	rural/clinic	No	28/02/2017	0	0	0
2015297363	859	rural/clinic	No	28/02/2017	0	0	0
2014360344	859	rural/clinic	No	28/02/2017	0	0	0
2014360345	859	rural/clinic	No	28/02/2017	0	0	0
2015293640	859	rural/clinic	No	28/02/2017	0	0	0
2015297073	859	rural/clinic	No	28/02/2017	0	0	0
2015293639	859	rural/clinic	No	28/02/2017	0	0	0
2015413545	859	rural/clinic	No	28/02/2017	0	0	0
2015297070	859	rural/clinic	No	28/02/2017	0	0	0
2011195846	859	rural/clinic	No	28/02/2017	0	0	0
2015294045	859	rural/clinic	No	28/02/2017	0	0	0
2015293641	859	rural/clinic	No	28/02/2017	0	0	0
2015318234	859	rural/clinic	No	28/02/2017	0	0	0
2015293642	859	rural/clinic	No	28/02/2017	0	0	0
2015368559	859	rural/clinic	No	28/02/2017	0	0	0
2015293643	859	rural/clinic	No	28/02/2017	0	0	0
2012252688	859	rural/clinic	No	28/02/2017	0	0	0
2012272758	859	rural/clinic	No	28/02/2017	0	0	0
2015293644	859	rural/clinic	No	28/02/2017	0	0	0
2015413582	109	district/faith-based	No	28/02/2017	1	0	0
2015413583	109	district/faith-based	No	28/02/2017	1	0	0

2015413581	109	district/faith-based	No	28/02/2017	1	0	0
2015392441	109	district/faith-based	No	28/02/2017	1	0	0
2011192488	109	district/faith-based	No	28/02/2017	1	0	0
2014377622	109	district/faith-based	No	28/02/2017	1	0	0
2015301444	109	district/faith-based	No	28/02/2017	1	0	0
2014372226	109	district/faith-based	No	28/02/2017	1	0	0
2011235744	109	district/faith-based	No	28/02/2017	1	0	0
2014372227	109	district/faith-based	No	28/02/2017	1	0	0
2013254703	109	district/faith-based	No	28/02/2017	1	0	0
2015344723	109	district/faith-based	No	28/02/2017	1	0	0
2013254701	109	district/faith-based	No	28/02/2017	1	0	0
2015413684	109	district/faith-based	No	28/02/2017	1	0	0
2015413693	109	district/faith-based	No	28/02/2017	1	0	0
2013254702	109	district/faith-based	No	28/02/2017	1	0	0
2015413694	109	district/faith-based	No	28/02/2017	1	0	0
2012280600	109	district/faith-based	No	28/02/2017	1	0	0
2015413692	109	district/faith-based	No	28/02/2017	1	0	0
2011211583	109	district/faith-based	No	28/02/2017	1	0	0
2015334603	109	district/faith-based	No	28/02/2017	1	0	0
2011148061	109	district/faith-based	No	28/02/2017	1	0	0
2015405911	109	district/faith-based	No	28/02/2017	1	0	0
2014357301	109	district/faith-based	No	28/02/2017	1	0	0
2015405910	109	district/faith-based	No	28/02/2017	1	0	0
2011152130	109	district/faith-based	No	28/02/2017	1	0	0
2015363005	109	district/faith-based	No	28/02/2017	1	0	0
2015392438	109	district/faith-based	No	28/02/2017	1	0	0
2014353629	109	district/faith-based	No	28/02/2017	1	0	0
2012272757	109	district/faith-based	No	28/02/2017	1	0	0
2012272335	109	district/faith-based	No	28/02/2017	1	0	0
2012340792	109	district/faith-based	No	28/02/2017	1	0	0
2012340791	109	district/faith-based	No	28/02/2017	1	0	0
2012340790	109	district/faith-based	No	28/02/2017	1	0	0
2012340793	109	district/faith-based	No	28/02/2017	1	0	0
2013278291	109	district/faith-based	No	28/02/2017	1	0	0
2015323801	109	district/faith-based	No	28/02/2017	1	0	0
2013260400	109	district/faith-based	No	28/02/2017	1	0	0
2013278290	109	district/faith-based	No	28/02/2017	1	0	0
2013260398	109	district/faith-based	No	28/02/2017	1	0	0
2015392539	109	district/faith-based	No	28/02/2017	1	0	0
2013278288	109	district/faith-based	No	28/02/2017	1	0	0
2015388504	109	district/faith-based	No	28/02/2017	1	0	0
2015339507	109	district/faith-based	No	21/02/2017	1	0	0
2015339507	109	district/faith-based	No	21/02/2017	1	0	0
2015414931	109	district/faith-based	No	21/02/2017	1	0	0
2015414931	109	district/faith-based	No	21/02/2017	1	0	0
2015388505	109	district/faith-based	No	28/02/2017	1	0	0
2012310631	109	district/faith-based	No	28/02/2017	1	0	0
2015294149	109	district/faith-based	No	23/02/2017	1	0	0
2015294149	109	district/faith-based	No	23/02/2017	1	0	0
2014385687	109	district/faith-based	No	23/02/2017	1	0	0
2014385687	109	district/faith-based	No	23/02/2017	1	0	0
2015377021	109	district/faith-based	No	21/02/2017	1	0	0
2015377021	109	district/faith-based	No	21/02/2017	1	0	0
2014375660	109	district/faith-based	No	21/02/2017	1	0	0
2014375660	109	district/faith-based	No	21/02/2017	1	0	0
2015414787	109	district/faith-based	No	21/02/2017	1	0	0
2013266109	109	district/faith-based	No	23/02/2017	1	0	0
2013266109	109	district/faith-based	No	23/02/2017	1	0	0
2014335298	109	district/faith-based	No	23/02/2017	1	0	0
2014335298	109	district/faith-based	No	23/02/2017	1	0	0
2015352430	109	district/faith-based	No	23/02/2017	1	0	0
2015352430	109	district/faith-based	No	23/02/2017	1	0	0
2015340484	109	district/faith-based	No	23/02/2017	1	0	0
2015340484	109	district/faith-based	No	23/02/2017	1	0	0
2014291852	109	district/faith-based	No	23/02/2017	1	0	0
2014291852	109	district/faith-based	No	23/02/2017	1	0	0
2015351150	109	district/faith-based	No	23/02/2017	1	0	0
2015351150	109	district/faith-based	No	23/02/2017	1	0	0
2012259761	109	district/faith-based	No	03/03/2017	1	0	0
2012290688	109	district/faith-based	No	03/03/2017	1	0	0
2015293452	109	district/faith-based	No	03/03/2017	1	0	0
2012290686	109	district/faith-based	No	03/03/2017	1	0	0
2014358326	109	district/faith-based	No	03/03/2017	1	0	0
2012290685	109	district/faith-based	No	03/03/2017	1	0	0
2012290684	109	district/faith-based	No	03/03/2017	1	0	0
2015293454	109	district/faith-based	No	03/03/2017	1	0	0
2015335193	109	district/faith-based	No	03/03/2017	1	0	0
2015293453	109	district/faith-based	No	03/03/2017	1	0	0
2015335191	109	district/faith-based	No	03/03/2017	1	0	0
2015335190	109	district/faith-based	No	03/03/2017	1	0	0
2015335192	109	district/faith-based	No	03/03/2017	1	0	0
2014385498	109	district/faith-based	No	03/03/2017	1	0	0

2015293455	109	district/faith-based	No	03/03/2017	1	0	0
2014385496	109	district/faith-based	No	03/03/2017	1	0	0
2015290355	109	district/faith-based	No	03/03/2017	1	0	0
2014385497	109	district/faith-based	No	03/03/2017	1	0	0
2014296314	109	district/faith-based	No	03/03/2017	1	0	0
2011144011	109	district/faith-based	No	03/03/2017	1	0	0
2015290356	109	district/faith-based	No	03/03/2017	1	0	0
2014385495	109	district/faith-based	No	03/03/2017	1	0	0
2015290354	109	district/faith-based	No	03/03/2017	1	0	0
2014385493	109	district/faith-based	No	03/03/2017	1	0	0
2015339458	109	district/faith-based	No	03/03/2017	1	0	0
2015290649	109	district/faith-based	No	03/03/2017	1	0	0
2014347983	109	district/faith-based	No	03/03/2017	1	0	0
2014385494	109	district/faith-based	No	03/03/2017	1	0	0
2015339459	109	district/faith-based	No	03/03/2017	1	0	0
2014347982	109	district/faith-based	No	03/03/2017	1	0	0
2013262215	109	district/faith-based	No	03/03/2017	1	0	0
2015369652	109	district/faith-based	No	03/03/2017	1	0	0
2015290648	109	district/faith-based	No	03/03/2017	1	0	0
2013262214	109	district/faith-based	No	03/03/2017	1	0	0
2015369653	109	district/faith-based	No	03/03/2017	1	0	0
2015369651	109	district/faith-based	No	03/03/2017	1	0	0
2015290650	109	district/faith-based	No	03/03/2017	1	0	0
2013262213	109	district/faith-based	No	03/03/2017	1	0	0
2015294176	109	district/faith-based	No	03/03/2017	1	0	0
2015369654	109	district/faith-based	No	03/03/2017	1	0	0
2015290352	109	district/faith-based	No	03/03/2017	1	0	0
2014352473	109	district/faith-based	No	03/03/2017	1	0	0
2015333910	109	district/faith-based	No	03/03/2017	1	0	0
2015290351	109	district/faith-based	No	03/03/2017	1	0	0
2014352472	109	district/faith-based	No	03/03/2017	1	0	0
2014352474	109	district/faith-based	No	03/03/2017	1	0	0
2015290353	109	district/faith-based	No	03/03/2017	1	0	0
2015288579	109	district/faith-based	No	03/03/2017	1	0	0
2014335845	109	district/faith-based	No	03/03/2017	1	0	0
2015333908	109	district/faith-based	No	03/03/2017	1	0	0
2015297072	109	district/faith-based	No	03/03/2017	1	0	0
2015325660	109	district/faith-based	No	03/03/2017	1	0	0
2015333909	109	district/faith-based	No	03/03/2017	1	0	0
2014335842	109	district/faith-based	No	03/03/2017	1	0	0
2015333912	109	district/faith-based	No	03/03/2017	1	0	0
2011127595	109	district/faith-based	No	03/03/2017	1	0	0
2014335843	109	district/faith-based	No	03/03/2017	1	0	0
2015333952	109	district/faith-based	No	03/03/2017	1	0	0
2015333954	109	district/faith-based	No	03/03/2017	1	0	0
2014377251	109	district/faith-based	No	03/03/2017	1	0	0
2011127596	109	district/faith-based	No	03/03/2017	1	0	0
2014319497	109	district/faith-based	No	03/03/2017	1	0	0
2014319495	109	district/faith-based	No	03/03/2017	1	0	0
2012376647	109	district/faith-based	No	03/03/2017	1	0	0
2012376592	109	district/faith-based	No	03/03/2017	1	0	0
2011142271	109	district/faith-based	No	03/03/2017	1	0	0
2015350659	109	district/faith-based	No	03/03/2017	1	0	0
2014301458	109	district/faith-based	No	03/03/2017	1	0	0
2015349942	109	district/faith-based	No	03/03/2017	1	0	0
2015350657	109	district/faith-based	No	03/03/2017	1	0	0
2014301459	109	district/faith-based	No	03/03/2017	1	0	0
2015349943	109	district/faith-based	No	03/03/2017	1	0	0
2014369843	109	district/faith-based	No	03/03/2017	1	0	0
2015350658	109	district/faith-based	No	03/03/2017	1	0	0
2014303286	109	district/faith-based	No	03/03/2017	1	0	0
2014369840	109	district/faith-based	No	03/03/2017	1	0	0
2011224031	109	district/faith-based	No	03/03/2017	1	0	0
2014369841	109	district/faith-based	No	03/03/2017	1	0	0
2015349937	109	district/faith-based	No	03/03/2017	1	0	0
2014369842	109	district/faith-based	No	03/03/2017	1	0	0
2013264179	109	district/faith-based	No	03/03/2017	1	0	0
2011234893	109	district/faith-based	No	03/03/2017	1	0	0
2011234892	109	district/faith-based	No	03/03/2017	1	0	0
2015328037	109	district/faith-based	No	03/03/2017	1	0	0
2015355726	109	district/faith-based	No	03/03/2017	1	0	0
2011234891	109	district/faith-based	No	03/03/2017	1	0	0
2015297077	109	district/faith-based	No	03/03/2017	1	0	0
2015301355	109	district/faith-based	No	03/03/2017	1	0	0
2014317620	109	district/faith-based	No	03/03/2017	1	0	0
2015297075	109	district/faith-based	No	03/03/2017	1	0	0
2015349525	109	district/faith-based	No	03/03/2017	1	0	0
2014378476	109	district/faith-based	No	03/03/2017	1	0	0
2015301354	109	district/faith-based	No	03/03/2017	1	0	0
2015412769	109	district/faith-based	No	03/03/2017	1	0	0
2015349931	109	district/faith-based	No	03/03/2017	1	0	0
2015349941	109	district/faith-based	No	03/03/2017	1	0	0

2015402300	109	district/faith-based	No	03/03/2017	1	0	0
2015402351	109	district/faith-based	No	03/03/2017	1	0	0
2015349940	109	district/faith-based	No	03/03/2017	1	0	0
2014378475	109	district/faith-based	No	03/03/2017	1	0	0
2015349932	109	district/faith-based	No	03/03/2017	1	0	0
2015402352	109	district/faith-based	No	03/03/2017	1	0	0
2014378474	109	district/faith-based	No	03/03/2017	1	0	0
2015402353	109	district/faith-based	No	03/03/2017	1	0	0
2015402354	109	district/faith-based	No	03/03/2017	1	0	0
2015402505	109	district/faith-based	No	03/03/2017	1	0	0
2015402506	109	district/faith-based	No	03/03/2017	1	0	0
2015325038	109	district/faith-based	No	03/03/2017	1	0	0
2014378473	109	district/faith-based	No	03/03/2017	1	0	0
2015402507	109	district/faith-based	No	03/03/2017	1	0	0
2015373659	109	district/faith-based	No	03/03/2017	1	0	0
2015402508	109	district/faith-based	No	03/03/2017	1	0	0
201536100	109	district/faith-based	No	03/03/2017	1	0	0
2015373660	109	district/faith-based	No	03/03/2017	1	0	0
2015402509	109	district/faith-based	No	03/03/2017	1	0	0
2015373661	109	district/faith-based	No	03/03/2017	1	0	0
2015360099	109	district/faith-based	No	03/03/2017	1	0	0
2015402510	109	district/faith-based	No	03/03/2017	1	0	0
2015415901	109	district/faith-based	No	03/03/2017	1	0	0
2015402511	109	district/faith-based	No	03/03/2017	1	0	0
2015415252	109	district/faith-based	No	03/03/2017	1	0	0
2015402512	109	district/faith-based	No	03/03/2017	1	0	0
2015358150	109	district/faith-based	No	03/03/2017	1	0	0
2015415251	109	district/faith-based	No	03/03/2017	1	0	0
2015358147	109	district/faith-based	No	03/03/2017	1	0	0
2015358149	109	district/faith-based	No	03/03/2017	1	0	0
2015373662	109	district/faith-based	No	03/03/2017	1	0	0
2015373389	109	district/faith-based	No	03/03/2017	1	0	0
2015358146	109	district/faith-based	No	03/03/2017	1	0	0
2015373663	109	district/faith-based	No	03/03/2017	1	0	0
2015373664	109	district/faith-based	No	03/03/2017	1	0	0
2015373395	109	district/faith-based	No	03/03/2017	1	0	0
2015358148	109	district/faith-based	No	03/03/2017	1	0	0
2015373665	109	district/faith-based	No	03/03/2017	1	0	0
2015373666	109	district/faith-based	No	03/03/2017	1	0	0
2011117490	109	district/faith-based	No	03/03/2017	1	0	0
2015386361	109	district/faith-based	No	03/03/2017	1	0	0
2015386360	109	district/faith-based	No	03/03/2017	1	0	0
2015373396	109	district/faith-based	No	03/03/2017	1	0	0
2015386359	109	district/faith-based	No	03/03/2017	1	0	0
2015373398	109	district/faith-based	No	03/03/2017	1	0	0
2015386358	109	district/faith-based	No	03/03/2017	1	0	0
2012290678	109	district/faith-based	No	14/02/2017	1	0	0
2015386357	109	district/faith-based	No	03/03/2017	1	0	0
2015373399	109	district/faith-based	No	03/03/2017	1	0	0
2011117489	109	district/faith-based	No	03/03/2017	1	0	0
2015314799	109	district/faith-based	No	28/02/2017	1	0	0
2015386354	109	district/faith-based	No	03/03/2017	1	0	0
2011117491	109	district/faith-based	No	03/03/2017	1	0	0
2011232680	109	district/faith-based	No	03/03/2017	1	0	0
2015373400	109	district/faith-based	No	03/03/2017	1	0	0
2015334605	109	district/faith-based	No	28/02/2017	1	0	0
2011232681	109	district/faith-based	No	03/03/2017	1	0	0
2015328457	109	district/faith-based	No	03/03/2017	1	0	0
2014358327	109	district/faith-based	No	03/03/2017	1	0	0
2012352826	109	district/faith-based	No	03/03/2017	1	0	0
2013272685	109	district/faith-based	No	03/03/2017	1	0	0
2014317342	109	district/faith-based	No	03/03/2017	1	0	0
2014307745	109	district/faith-based	No	03/03/2017	1	0	0
2014358324	109	district/faith-based	No	03/03/2017	1	0	0
2015368266	109	district/faith-based	No	03/03/2017	1	0	0
2015326551	109	district/faith-based	No	03/03/2017	1	0	0
2015368150	109	district/faith-based	No	03/03/2017	1	0	0
2014291853	109	district/faith-based	No	03/03/2017	1	0	0
2012315320	109	district/faith-based	No	03/03/2017	1	0	0
2014379442	109	district/faith-based	No	03/03/2017	1	0	0
2015319126	109	district/faith-based	No	03/03/2017	1	0	0
2015319123	109	district/faith-based	No	03/03/2017	1	0	0
2015319124	109	district/faith-based	No	03/03/2017	1	0	0
2015398792	109	district/faith-based	No	03/03/2017	1	0	0
2015319125	109	district/faith-based	No	03/03/2017	1	0	0
2015319232	109	district/faith-based	No	03/03/2017	1	0	0
2015353485	109	district/faith-based	No	03/03/2017	1	0	0
2015319231	109	district/faith-based	No	03/03/2017	1	0	0
2015319229	109	district/faith-based	No	03/03/2017	1	0	0
2015319230	109	district/faith-based	No	03/03/2017	1	0	0
2015319121	109	district/faith-based	No	03/03/2017	1	0	0
2015319120	109	district/faith-based	No	03/03/2017	1	0	0

2015319119	109	district/faith-based	No	03/03/2017	1	0	0
2015373344	109	district/faith-based	No	03/03/2017	1	0	0
2014324686	109	district/faith-based	No	03/03/2017	1	0	0
2015373345	109	district/faith-based	No	03/03/2017	1	0	0
2015373346	109	district/faith-based	No	03/03/2017	1	0	0
2014324687	109	district/faith-based	No	03/03/2017	1	0	0
2015373347	109	district/faith-based	No	03/03/2017	1	0	0
2014329303	109	district/faith-based	No	03/03/2017	1	0	0
2015373348	109	district/faith-based	No	03/03/2017	1	0	0
2015338287	109	district/faith-based	No	03/03/2017	1	0	0
2014324688	109	district/faith-based	No	03/03/2017	1	0	0
2015386355	109	district/faith-based	No	03/03/2017	1	0	0
2015386356	109	district/faith-based	No	03/03/2017	1	0	0
2015373349	109	district/faith-based	No	03/03/2017	1	0	0
2015386352	109	district/faith-based	No	03/03/2017	1	0	0
2014314202	109	district/faith-based	No	03/03/2017	1	0	0
2015369382	109	district/faith-based	No	03/03/2017	1	0	0
2012387250	109	district/faith-based	No	03/03/2017	1	0	0
2015369383	109	district/faith-based	No	03/03/2017	1	0	0
2014314201	109	district/faith-based	No	03/03/2017	1	0	0
2015320494	109	district/faith-based	No	03/03/2017	1	0	0
2015369384	109	district/faith-based	No	03/03/2017	1	0	0
2011129333	109	district/faith-based	No	03/03/2017	1	0	0
2015413107	109	district/faith-based	No	03/03/2017	1	0	0
2015398790	109	district/faith-based	No	03/03/2017	1	0	0
2015323052	109	district/faith-based	No	03/03/2017	1	0	0
2014290235	109	district/faith-based	No	03/03/2017	1	0	0
2015323051	109	district/faith-based	No	03/03/2017	1	0	0
2014290236	109	district/faith-based	No	03/03/2017	1	0	0
2015357632	109	district/faith-based	No	03/03/2017	1	0	0
2014352471	109	district/faith-based	No	03/03/2017	1	0	0
2014352470	109	district/faith-based	No	03/03/2017	1	0	0
2015319118	109	district/faith-based	No	03/03/2017	1	0	0
2014385688	109	district/faith-based	No	03/03/2017	1	0	0
2015301446	109	district/faith-based	No	03/03/2017	1	0	0
2015413106	109	district/faith-based	No	03/03/2017	1	0	0
2015301447	109	district/faith-based	No	03/03/2017	1	0	0
2015357633	109	district/faith-based	No	03/03/2017	1	0	0
2014289500	109	district/faith-based	No	03/03/2017	1	0	0
2014301891	109	district/faith-based	No	03/03/2017	1	0	0
2014289499	109	district/faith-based	No	03/03/2017	1	0	0
2014382430	109	district/faith-based	No	03/03/2017	1	0	0
2014289497	109	district/faith-based	No	03/03/2017	1	0	0
2014289498	109	district/faith-based	No	03/03/2017	1	0	0
2014289496	109	district/faith-based	No	03/03/2017	1	0	0
2012384784	109	district/faith-based	No	03/03/2017	1	0	0
2011489252	109	district/faith-based	No	03/03/2017	1	0	0
2011133601	109	district/faith-based	No	03/03/2017	1	0	0
2011133602	109	district/faith-based	No	03/03/2017	1	0	0
2015305248	109	district/faith-based	No	03/03/2017	1	0	0
2015305249	109	district/faith-based	No	03/03/2017	1	0	0
2015305250	109	district/faith-based	No	03/03/2017	1	0	0
2015361790	109	district/faith-based	No	03/03/2017	1	0	0
2014335226	109	district/faith-based	No	03/03/2017	1	0	0
2014335225	109	district/faith-based	No	03/03/2017	1	0	0
2015360590	109	district/faith-based	No	03/03/2017	1	0	0
2015360589	690	rural/clinic	No	03/03/2017	0	0	0
2015360591	690	rural/clinic	No	03/03/2017	0	0	0
2012330880	690	rural/clinic	No	28/02/2017	0	0	0
2012330880	690	rural/clinic	No	28/02/2017	0	0	0
2015351894	324	rural/clinic	No	24/02/2017	0	0	0
2015351894	324	rural/clinic	No	24/02/2017	0	0	0
2014330642	324	rural/clinic	No	28/02/2017	0	0	0
2015293830	324	rural/clinic	No	28/02/2017	0	0	0
2015293830	324	rural/clinic	No	28/02/2017	0	0	0
2015301589	324	rural/clinic	No	28/02/2017	0	0	0
2015301589	324	rural/clinic	No	28/02/2017	0	0	0
2015378948	324	rural/clinic	No	23/02/2017	0	0	0
2015378948	324	rural/clinic	No	23/02/2017	0	0	0
2015360097	324	rural/clinic	No	23/02/2017	0	0	0
2015360097	324	rural/clinic	No	23/02/2017	0	0	0
2015326452	324	rural/clinic	No	23/02/2017	0	0	0
2015326452	324	rural/clinic	No	23/02/2017	0	0	0
2015326456	324	rural/clinic	No	23/02/2017	0	0	0
2015326456	324	rural/clinic	No	23/02/2017	0	0	0
2015326509	238	rural/clinic	No	23/02/2017	0	0	0
2015326509	238	rural/clinic	No	23/02/2017	0	0	0
2015414786	238	rural/clinic	No	21/02/2017	0	0	0
2014330347	238	rural/clinic	No	03/03/2017	0	0	0
2012313098	238	rural/clinic	No	03/03/2017	0	0	0
2012313099	238	rural/clinic	No	03/03/2017	0	0	0
2015346088	238	rural/clinic	No	03/03/2017	0	0	0

2015333253	238	rural/clinic	No	03/03/2017	0	0	0
2015346224	238	rural/clinic	No	03/03/2017	0	0	0
2015358144	238	rural/clinic	No	23/02/2017	0	0	0
2015358144	238	rural/clinic	No	23/02/2017	0	0	0
2015333911	238	rural/clinic	No	03/03/2017	0	0	0
2012377876	238	rural/clinic	No	03/03/2017	0	0	0
2015405909	238	rural/clinic	No	28/02/2017	0	0	0
2014356846	238	rural/clinic	No	28/02/2017	0	0	0
2015383590	238	rural/clinic	No	28/02/2017	0	0	0
2014320526	238	rural/clinic	No	28/02/2017	0	0	0
2012242577	238	rural/clinic	No	28/02/2017	0	0	0
2015385435	238	rural/clinic	No	28/02/2017	0	0	0
2015385434	238	rural/clinic	No	28/02/2017	0	0	0
2015385439	238	rural/clinic	No	28/02/2017	0	0	0
2015385438	238	rural/clinic	No	28/02/2017	0	0	0
2013266240	238	rural/clinic	No	28/02/2017	0	0	0
2015377028	238	rural/clinic	No	03/03/2017	0	0	0
2015377029	238	rural/clinic	No	03/03/2017	0	0	0
2015340492	238	rural/clinic	No	03/03/2017	0	0	0
2015355379	238	rural/clinic	No	03/03/2017	0	0	0
2015352431	238	rural/clinic	No	03/03/2017	0	0	0
2015340487	238	rural/clinic	No	03/03/2017	0	0	0
2015352432	238	rural/clinic	No	03/03/2017	0	0	0
2015340488	238	rural/clinic	No	03/03/2017	0	0	0
2015368449	917	rural/clinic	No	03/03/2017	0	0	0
2015340489	917	rural/clinic	No	03/03/2017	0	0	0
2015360592	917	rural/clinic	No	03/03/2017	0	0	0
2015360593	917	rural/clinic	No	03/03/2017	0	0	0
2015340490	78	rural/clinic	No	03/03/2017	0	0	0
2015335371	78	rural/clinic	No	03/03/2017	0	0	0
2015335372	78	rural/clinic	No	03/03/2017	0	0	0
2015384528	78	rural/clinic	No	03/03/2017	0	0	0
2012390998	78	rural/clinic	No	03/03/2017	0	0	0
2015384529	78	rural/clinic	No	03/03/2017	0	0	0
2011168519	78	rural/clinic	No	03/03/2017	0	0	0
2015379503	78	rural/clinic	No	03/03/2017	0	0	0
2015377026	78	rural/clinic	No	03/03/2017	0	0	0
2015377027	78	rural/clinic	No	03/03/2017	0	0	0
2015334603	78	rural/clinic	No	28/02/2017	0	0	0
2015334603	78	rural/clinic	No	28/02/2017	0	0	0
2015331764	78	rural/clinic	No	28/02/2017	0	0	0
2015331764	78	rural/clinic	No	28/02/2017	0	0	0
2012252688	78	rural/clinic	No	28/02/2017	0	0	0
2012252688	78	rural/clinic	No	28/02/2017	0	0	0
2012290683	78	rural/clinic	No	28/02/2017	0	0	0
2012290683	78	rural/clinic	No	28/02/2017	0	0	0
2014319496	78	rural/clinic	No	02/03/2017	0	0	0
2014300421	110	rural/clinic	No	02/03/2017	0	0	0
2012290687	110	rural/clinic	No	02/03/2017	0	0	0
2011129331	110	rural/clinic	No	02/03/2017	0	0	0
2011129330	110	rural/clinic	No	02/03/2017	0	0	0
2011129332	110	rural/clinic	No	02/03/2017	0	0	0
2012285869/D	110	rural/clinic	No	03/03/2017	0	0	0
2012285863	110	rural/clinic	No	03/03/2017	0	0	0
2012285868	110	rural/clinic	No	03/03/2017	0	0	0
2012285866	110	rural/clinic	No	03/03/2017	0	0	0
2012285870	110	rural/clinic	No	03/03/2017	0	0	0
2012285865	110	rural/clinic	No	03/03/2017	0	0	0
2015322091	110	rural/clinic	No	03/03/2017	0	0	0
2015322092	110	rural/clinic	No	03/03/2017	0	0	0
2015322094	110	rural/clinic	No	03/03/2017	0	0	0
2012367295	110	rural/clinic	No	03/03/2017	0	0	0
2014385240	110	rural/clinic	No	03/03/2017	0	0	0
2014385241	110	rural/clinic	No	03/03/2017	0	0	0
2014385242	110	rural/clinic	No	03/03/2017	0	0	0
2014294801	110	rural/clinic	No	03/03/2017	0	0	0
2012285637	110	rural/clinic	No	03/03/2017	0	0	0
2012285639	110	rural/clinic	No	03/03/2017	0	0	0
2012285636	110	rural/clinic	No	03/03/2017	0	0	0
2014344335	110	rural/clinic	No	28/02/2017	0	0	0
2014344335	110	rural/clinic	No	28/02/2017	0	0	0
2014355898	110	rural/clinic	No	28/02/2017	0	0	0
2014355898	110	rural/clinic	No	28/02/2017	0	0	0
2015363005	110	rural/clinic	No	28/02/2017	0	0	0
2015363005	110	rural/clinic	No	28/02/2017	0	0	0
2012285638	110	rural/clinic	No	03/03/2017	0	0	0
2012285864	110	rural/clinic	No	03/03/2017	0	0	0
2012285867	110	rural/clinic	No	03/03/2017	0	0	0
2015319125	110	rural/clinic	No	03/03/2017	0	0	0
2015319125	110	rural/clinic	No	03/03/2017	0	0	0
2015319119	110	rural/clinic	No	03/03/2017	0	0	0
2015319119	110	rural/clinic	No	03/03/2017	0	0	0

2012291548	110	rural/clinic	No	03/03/2017	0	0	0
2012291549	110	rural/clinic	No	03/03/2017	0	0	0
2013248185	712	rural/clinic	No	03/03/2017	0	0	0
2013248184	712	rural/clinic	No	03/03/2017	0	0	0
2014296880	712	rural/clinic	No	03/03/2017	0	0	0
2014296879	712	rural/clinic	No	03/03/2017	0	0	0
2014296882	712	rural/clinic	No	03/03/2017	0	0	0
2014296883	712	rural/clinic	No	03/03/2017	0	0	0
2015352354	712	rural/clinic	No	03/03/2017	0	0	0
2012291547	712	rural/clinic	No	03/03/2017	0	0	0
2012291546	712	rural/clinic	No	03/03/2017	0	0	0
2012298035	712	rural/clinic	No	03/03/2017	0	0	0
2012298036	712	rural/clinic	No	03/03/2017	0	0	0
2015344837	712	rural/clinic	No	03/03/2017	0	0	0
2015344838	712	rural/clinic	No	03/03/2017	0	0	0
2015344836	712	rural/clinic	No	03/03/2017	0	0	0
2015344840	712	rural/clinic	No	03/03/2017	0	0	0
2015344839	712	rural/clinic	No	03/03/2017	0	0	0
2015303508	712	rural/clinic	No	03/03/2017	0	0	0
2015303507	712	rural/clinic	No	03/03/2017	0	0	0
2015314794	712	rural/clinic	No	03/03/2017	0	0	0
2015300676	712	rural/clinic	No	03/03/2017	0	0	0
2015300678	712	rural/clinic	No	03/03/2017	0	0	0
2015300675	712	rural/clinic	No	03/03/2017	0	0	0
2015300674	712	rural/clinic	No	03/03/2017	0	0	0
2012305694	712	rural/clinic	No	03/03/2017	0	0	0
2015300677	712	rural/clinic	No	03/03/2017	0	0	0
2012305695	712	rural/clinic	No	03/03/2017	0	0	0
2015300673	712	rural/clinic	No	03/03/2017	0	0	0
2012298039	712	rural/clinic	No	03/03/2017	0	0	0
2012298038	712	rural/clinic	No	03/03/2017	0	0	0
2012298037	712	rural/clinic	No	03/03/2017	0	0	0
2011171445	712	rural/clinic	No	03/03/2017	0	0	0
2011171446	712	rural/clinic	No	03/03/2017	0	0	0
2011171443	712	rural/clinic	No	03/03/2017	0	0	0
2014316764	712	rural/clinic	No	03/03/2017	0	0	0
2014316763	325	rural/clinic	No	03/03/2017	0	0	0
2014360339	325	rural/clinic	No	28/02/2017	0	0	0
2014360339	325	rural/clinic	No	28/02/2017	0	0	0
2015415252	325	rural/clinic	No	03/03/2017	0	0	0
2015415252	325	rural/clinic	No	03/03/2017	0	0	0
2014289499	325	rural/clinic	No	03/03/2017	0	0	0
2014289499	325	rural/clinic	No	03/03/2017	0	0	0
2015373168	325	rural/clinic	No	03/03/2017	0	0	0
2015358146	325	rural/clinic	No	03/03/2017	0	0	0
2015358146	325	rural/clinic	No	03/03/2017	0	0	0
2015386358	325	rural/clinic	No	03/03/2017	0	0	0
2015386358	325	rural/clinic	No	03/03/2017	0	0	0
2015349525	325	rural/clinic	No	03/03/2017	0	0	0
2015349525	325	rural/clinic	No	03/03/2017	0	0	0
2015325038	325	rural/clinic	No	03/03/2017	0	0	0
2015325038	325	rural/clinic	No	03/03/2017	0	0	0
2015373165	325	rural/clinic	No	03/03/2017	0	0	0
2015373164	325	rural/clinic	No	03/03/2017	0	0	0
2015373163	325	rural/clinic	No	03/03/2017	0	0	0
2015373167	202	rural/clinic	No	03/03/2017	0	0	0
2015373161	202	rural/clinic	No	03/03/2017	0	0	0
2015373160	202	rural/clinic	No	03/03/2017	0	0	0
2015373159	202	rural/clinic	No	03/03/2017	0	0	0
2015373151	202	rural/clinic	No	03/03/2017	0	0	0
2015373250	202	rural/clinic	No	03/03/2017	0	0	0
2015373248	202	rural/clinic	No	03/03/2017	0	0	0
2015373169	202	rural/clinic	No	03/03/2017	0	0	0
2012336607	202	rural/clinic	No	03/03/2017	0	0	0
2012259859	202	rural/clinic	No	03/03/2017	0	0	0
2012259860	573	rural/clinic	No	03/03/2017	0	0	0
2012259856	573	rural/clinic	No	03/03/2017	0	0	0
2012259857	573	rural/clinic	No	03/03/2017	0	0	0
2012259858	573	rural/clinic	No	03/03/2017	0	0	0
2012259862	573	rural/clinic	No	03/03/2017	0	0	0
2012336606	573	rural/clinic	No	03/03/2017	0	0	0
2015348884	573	rural/clinic	No	03/03/2017	0	0	0
2015328519	573	rural/clinic	No	03/03/2017	0	0	0
2015328522	573	rural/clinic	No	03/03/2017	0	0	0
2015328520	573	rural/clinic	No	03/03/2017	0	0	0
2015348888	573	rural/clinic	No	03/03/2017	0	0	0
2012367296	573	rural/clinic	No	03/03/2017	0	0	0
2015402297	573	rural/clinic	No	23/02/2017	0	0	0
2015290353	573	rural/clinic	No	03/03/2017	0	0	0
2015290353	573	rural/clinic	No	03/03/2017	0	0	0
2015325660	573	rural/clinic	No	03/03/2017	0	0	0
2015325660	573	rural/clinic	No	03/03/2017	0	0	0

2015369651	573	rural/clinic	No	03/03/2017	0	0	0
2015369651	573	rural/clinic	No	03/03/2017	0	0	0
2015360590	573	rural/clinic	No	03/03/2017	0	0	0
2015360590	573	rural/clinic	No	03/03/2017	0	0	0
2015360591	573	rural/clinic	No	03/03/2017	0	0	0
2015360591	573	rural/clinic	No	03/03/2017	0	0	0
2012352826	573	rural/clinic	No	03/03/2017	0	0	0
2012352826	573	rural/clinic	No	03/03/2017	0	0	0
2015326551	573	rural/clinic	No	03/03/2017	0	0	0
2015326551	573	rural/clinic	No	03/03/2017	0	0	0
2015306118	573	rural/clinic	No	07/03/2017	0	0	0
2015306120	573	rural/clinic	No	07/03/2017	0	0	0
2015324428	573	rural/clinic	No	07/03/2017	0	0	0
2015376108	573	rural/clinic	No	07/03/2017	0	0	0
2012374272	573	rural/clinic	No	07/03/2017	0	0	0
2012374274	573	rural/clinic	No	07/03/2017	0	0	0
2015376107	573	rural/clinic	No	07/03/2017	0	0	0
2012295152	573	rural/clinic	No	07/03/2017	0	0	0
2014303056	573	rural/clinic	No	14/02/2017	0	0	0
2012374273	573	rural/clinic	No	07/03/2017	0	0	0
2015344842	573	rural/clinic	No	07/03/2017	0	0	0
2015385939	573	rural/clinic	No	07/03/2017	0	0	0
2014303058	573	rural/clinic	No	14/02/2017	0	0	0
2015344841	573	rural/clinic	No	07/03/2017	0	0	0
2015349101	573	rural/clinic	No	07/03/2017	0	0	0
2015384777	573	rural/clinic	No	07/03/2017	0	0	0
2015376109	573	rural/clinic	No	07/03/2017	0	0	0
2015385385	573	rural/clinic	No	07/03/2017	0	0	0
2015310601	573	rural/clinic	No	07/03/2017	0	0	0
2014327113	573	rural/clinic	No	07/03/2017	0	0	0
2015337040	573	rural/clinic	No	07/03/2017	0	0	0
2014350093	573	rural/clinic	No	07/03/2017	0	0	0
2014297783	573	rural/clinic	No	07/03/2017	0	0	0
2013249413	573	rural/clinic	No	07/03/2017	0	0	0
2012313804	573	rural/clinic	No	07/03/2017	0	0	0
2014291589	573	rural/clinic	No	07/03/2017	0	0	0
2015359753	573	rural/clinic	No	07/03/2017	0	0	0
2015326514	573	rural/clinic	No	07/03/2017	0	0	0
2014320292	573	rural/clinic	No	07/03/2017	0	0	0
2014291591	573	rural/clinic	No	07/03/2017	0	0	0
2015326513	573	rural/clinic	No	07/03/2017	0	0	0
2012312848	573	rural/clinic	No	07/03/2017	0	0	0
2015326515	573	rural/clinic	No	07/03/2017	0	0	0
2014291588	573	rural/clinic	No	07/03/2017	0	0	0
2015412080	573	rural/clinic	No	07/03/2017	0	0	0
2015357375	573	rural/clinic	No	07/03/2017	0	0	0
2015375966	573	rural/clinic	No	07/03/2017	0	0	0
2015342277	573	rural/clinic	No	07/03/2017	0	0	0
2014291587	573	rural/clinic	No	07/03/2017	0	0	0
2015375407	573	rural/clinic	No	07/03/2017	0	0	0
2015369385	573	rural/clinic	No	07/03/2017	0	0	0
2014291590	573	rural/clinic	No	07/03/2017	0	0	0
2012253239	573	rural/clinic	No	07/03/2017	0	0	0
2015375970	573	rural/clinic	No	07/03/2017	0	0	0
2013261228	573	rural/clinic	No	07/03/2017	0	0	0
2015375967	573	rural/clinic	No	07/03/2017	0	0	0
2014327239	573	rural/clinic	No	07/03/2017	0	0	0
2015300681	573	rural/clinic	No	07/03/2017	0	0	0
2014311649	573	rural/clinic	No	07/03/2017	0	0	0
2015306119	573	rural/clinic	No	07/03/2017	0	0	0
2014356848	573	rural/clinic	No	07/03/2017	0	0	0
2015375968	573	rural/clinic	No	07/03/2017	0	0	0
2014370428	573	rural/clinic	No	07/03/2017	0	0	0
2015375102	573	rural/clinic	No	07/03/2017	0	0	0
2015300679	573	rural/clinic	No	07/03/2017	0	0	0
2015340061	573	rural/clinic	No	07/03/2017	0	0	0
2013261227	573	rural/clinic	No	07/03/2017	0	0	0
2014314719	573	rural/clinic	No	07/03/2017	0	0	0
2014356847	573	rural/clinic	No	07/03/2017	0	0	0
2011113945	573	rural/clinic	No	07/03/2017	0	0	0
2013261230	573	rural/clinic	No	07/03/2017	0	0	0
2012284739	573	rural/clinic	No	07/03/2017	0	0	0
2012253920	573	rural/clinic	No	07/03/2017	0	0	0
2015340060	573	rural/clinic	No	07/03/2017	0	0	0
2014314718	573	rural/clinic	No	07/03/2017	0	0	0
2015375969	573	rural/clinic	No	07/03/2017	0	0	0
2015331766	573	rural/clinic	No	07/03/2017	0	0	0
2014334344	573	rural/clinic	No	07/03/2017	0	0	0
2015413640	573	rural/clinic	No	07/03/2017	0	0	0
2015375406	573	rural/clinic	No	07/03/2017	0	0	0
2015300672	573	rural/clinic	No	07/03/2017	0	0	0
2015413639	573	rural/clinic	No	07/03/2017	0	0	0

2015340059	573	rural/clinic	No	07/03/2017	0	0	0
2015342276	573	rural/clinic	No	07/03/2017	0	0	0
2013271694	573	rural/clinic	No	07/03/2017	0	0	0
2014288676	573	rural/clinic	No	07/03/2017	0	0	0
2014303057	573	rural/clinic	No	14/02/2017	0	0	0
2015342275	573	rural/clinic	No	07/03/2017	0	0	0
2013271693	573	rural/clinic	No	07/03/2017	0	0	0
2013371835	573	rural/clinic	No	07/03/2017	0	0	0
2015334706	573	rural/clinic	No	07/03/2017	0	0	0
2011232685	573	rural/clinic	No	07/03/2017	0	0	0
2012253921	573	rural/clinic	No	07/03/2017	0	0	0
2014290721	111	rural/clinic	No	07/03/2017	0	0	0
2015300680	111	rural/clinic	No	07/03/2017	0	0	0
2015340058	111	rural/clinic	No	07/03/2017	0	0	0
2011113944	111	rural/clinic	No	07/03/2017	0	0	0
2015306115	111	rural/clinic	No	07/03/2017	0	0	0
2015360098	111	rural/clinic	No	23/02/2017	0	0	0
2015334707	111	rural/clinic	No	07/03/2017	0	0	0
2012253919/D	111	rural/clinic	No	07/03/2017	0	0	0
2015340057	111	rural/clinic	No	07/03/2017	0	0	0
2.02E+13	111	rural/clinic	No	23/02/2017	0	0	0
2014290720	111	rural/clinic	No	07/03/2017	0	0	0
2015334709	111	rural/clinic	No	07/03/2017	0	0	0
2011113946	111	rural/clinic	No	07/03/2017	0	0	0
2015357376	111	rural/clinic	No	07/03/2017	0	0	0
2015299779	111	rural/clinic	No	07/03/2017	0	0	0
2.02E+15	111	rural/clinic	No	07/03/2017	0	0	0
2011113947	111	rural/clinic	No	07/03/2017	0	0	0
2014347727	111	rural/clinic	No	07/03/2017	0	0	0
2014290722	111	rural/clinic	No	07/03/2017	0	0	0
2015357374	111	rural/clinic	No	07/03/2017	0	0	0
2015375965	111	rural/clinic	No	07/03/2017	0	0	0
2012305696	111	rural/clinic	No	07/03/2017	0	0	0
2015357378	111	rural/clinic	No	07/03/2017	0	0	0
2015334711	111	rural/clinic	No	07/03/2017	0	0	0
2014290719	111	rural/clinic	No	07/03/2017	0	0	0
2015334712	111	rural/clinic	No	07/03/2017	0	0	0
2015318236	111	rural/clinic	No	07/03/2017	0	0	0
2012368533	111	rural/clinic	No	07/03/2017	0	0	0
2012253962	111	rural/clinic	No	07/03/2017	0	0	0
2015334708	79	rural/clinic	No	07/03/2017	0	0	0
2015299777	79	rural/clinic	No	07/03/2017	0	0	0
2012284740	79	rural/clinic	No	07/03/2017	0	0	0
2015334705	79	rural/clinic	No	07/03/2017	0	0	0
2013264059	79	rural/clinic	No	07/03/2017	0	0	0
2015299778	79	rural/clinic	No	07/03/2017	0	0	0
2014356959	79	rural/clinic	No	07/03/2017	0	0	0
2014334365	79	rural/clinic	No	07/03/2017	0	0	0
2015334713	79	rural/clinic	No	07/03/2017	0	0	0
2014363588	79	rural/clinic	No	07/03/2017	0	0	0
2015318235	79	rural/clinic	No	07/03/2017	0	0	0
2014356957	79	rural/clinic	No	07/03/2017	0	0	0
2015289168	79	rural/clinic	No	07/03/2017	0	0	0
2015289564	79	rural/clinic	No	07/03/2017	0	0	0
2015414937	79	rural/clinic	No	07/03/2017	0	0	0
2015299780	79	rural/clinic	No	07/03/2017	0	0	0
2015382885	79	rural/clinic	No	07/03/2017	0	0	0
2015306117	79	rural/clinic	No	07/03/2017	0	0	0
2014356958	79	rural/clinic	No	07/03/2017	0	0	0
2015414938	79	rural/clinic	No	07/03/2017	0	0	0
2015382883	79	rural/clinic	No	07/03/2017	0	0	0
2015306116	79	rural/clinic	No	07/03/2017	0	0	0
2012295967	79	rural/clinic	No	07/03/2017	0	0	0
2015316960	79	rural/clinic	No	07/03/2017	0	0	0
2015412077	79	rural/clinic	No	07/03/2017	0	0	0
2011232683	79	rural/clinic	No	07/03/2017	0	0	0
2015334511	79	rural/clinic	No	07/03/2017	0	0	0
2012259861	79	rural/clinic	No	07/03/2017	0	0	0
2015362349	79	rural/clinic	No	07/03/2017	0	0	0
2015316957	79	rural/clinic	No	07/03/2017	0	0	0
2012253918	79	rural/clinic	No	07/03/2017	0	0	0
2011232684	79	rural/clinic	No	07/03/2017	0	0	0
2014303069	79	rural/clinic	No	07/03/2017	0	0	0
2014326626	79	rural/clinic	No	07/03/2017	0	0	0
2014326624	79	rural/clinic	No	07/03/2017	0	0	0
2012313803	79	rural/clinic	No	07/03/2017	0	0	0
2015360712	79	rural/clinic	No	07/03/2017	0	0	0
2015412078	79	rural/clinic	No	07/03/2017	0	0	0
2014326623	79	rural/clinic	No	07/03/2017	0	0	0
2015334505	79	rural/clinic	No	07/03/2017	0	0	0
2011142491	79	rural/clinic	No	07/03/2017	0	0	0
2014326625	79	rural/clinic	No	07/03/2017	0	0	0

2011232686	79	rural/clinic	No	07/03/2017	0	0	0
2011134965	79	rural/clinic	No	07/03/2017	0	0	0
2015334510	79	rural/clinic	No	07/03/2017	0	0	0
2015412076	79	rural/clinic	No	07/03/2017	0	0	0
2011134964	79	rural/clinic	No	07/03/2017	0	0	0
2012313802	79	rural/clinic	No	07/03/2017	0	0	0
2014288394	79	rural/clinic	No	07/03/2017	0	0	0
2014314456	79	rural/clinic	No	07/03/2017	0	0	0
2014328759	79	rural/clinic	No	07/03/2017	0	0	0
2011134962	79	rural/clinic	No	07/03/2017	0	0	0
2015335576	79	rural/clinic	No	07/03/2017	0	0	0
2013264058	79	rural/clinic	No	07/03/2017	0	0	0
2012313805	79	rural/clinic	No	07/03/2017	0	0	0
2011134963	79	rural/clinic	No	07/03/2017	0	0	0
2015297078	79	rural/clinic	No	07/03/2017	0	0	0
2015379021	79	rural/clinic	No	07/03/2017	0	0	0
2011134961	79	rural/clinic	No	07/03/2017	0	0	0
2015334405	79	rural/clinic	No	07/03/2017	0	0	0
2015297079	79	rural/clinic	No	07/03/2017	0	0	0
2015375964	79	rural/clinic	No	07/03/2017	0	0	0
2015334404	79	rural/clinic	No	07/03/2017	0	0	0
2015337952	79	rural/clinic	No	07/03/2017	0	0	0
2011134960	79	rural/clinic	No	07/03/2017	0	0	0
2015379022	79	rural/clinic	No	07/03/2017	0	0	0
2015334406	79	rural/clinic	No	07/03/2017	0	0	0
2015297365	79	rural/clinic	No	07/03/2017	0	0	0
2011134966	79	rural/clinic	No	07/03/2017	0	0	0
2015337953	79	rural/clinic	No	07/03/2017	0	0	0
2015377625	79	rural/clinic	No	07/03/2017	0	0	0
2015379018	79	rural/clinic	No	07/03/2017	0	0	0
2012390144	79	rural/clinic	No	07/03/2017	0	0	0
2011134970	79	rural/clinic	No	07/03/2017	0	0	0
2015344313	79	rural/clinic	No	07/03/2017	0	0	0
2015337956	79	rural/clinic	No	07/03/2017	0	0	0
2012350100	79	rural/clinic	No	07/03/2017	0	0	0
2011134969	79	rural/clinic	No	07/03/2017	0	0	0
2014326750	79	rural/clinic	No	07/03/2017	0	0	0
2015289280	631	rural/clinic	No	07/03/2017	0	0	0
2015379019	631	rural/clinic	No	07/03/2017	0	0	0
2015305060	631	rural/clinic	No	07/03/2017	0	0	0
2011142545	631	rural/clinic	No	07/03/2017	0	0	0
2011204781	631	rural/clinic	No	07/03/2017	0	0	0
2013244111	631	rural/clinic	No	07/03/2017	0	0	0
2014343870	631	rural/clinic	No	07/03/2017	0	0	0
2015305061	631	rural/clinic	No	07/03/2017	0	0	0
2015379020	631	rural/clinic	No	07/03/2017	0	0	0
2012390142	631	rural/clinic	No	07/03/2017	0	0	0
2012245726	299	rural/clinic	No	07/03/2017	0	0	0
2014319427	299	rural/clinic	No	07/03/2017	0	0	0
2012390143	299	rural/clinic	No	07/03/2017	0	0	0
2012245727	299	rural/clinic	No	07/03/2017	0	0	0
2015360713	299	rural/clinic	No	07/03/2017	0	0	0
2015368869	299	rural/clinic	No	07/03/2017	0	0	0
2012245728	299	rural/clinic	No	07/03/2017	0	0	0
2015418370	299	rural/clinic	No	07/03/2017	0	0	0
2015357377	299	rural/clinic	No	07/03/2017	0	0	0
2015339812	299	rural/clinic	No	07/03/2017	0	0	0
2015412079	299	rural/clinic	No	07/03/2017	0	0	0
2015369161	299	rural/clinic	No	07/03/2017	0	0	0
2015342009	299	rural/clinic	No	07/03/2017	0	0	0
2015339814	299	rural/clinic	No	07/03/2017	0	0	0
2015293933	299	rural/clinic	No	07/03/2017	0	0	0
2015293456	299	rural/clinic	No	07/03/2017	0	0	0
2015293457	299	rural/clinic	No	07/03/2017	0	0	0
2015368871	299	rural/clinic	No	07/03/2017	0	0	0
2015293458	299	rural/clinic	No	07/03/2017	0	0	0
2015293459	299	rural/clinic	No	07/03/2017	0	0	0
2015369655	299	rural/clinic	No	07/03/2017	0	0	0
2015293460	299	rural/clinic	No	07/03/2017	0	0	0
2015339811	299	rural/clinic	No	07/03/2017	0	0	0
2015369656	299	rural/clinic	No	07/03/2017	0	0	0
2015377626	299	rural/clinic	No	07/03/2017	0	0	0
2015339522	299	rural/clinic	No	07/03/2017	0	0	0
2015339523	299	rural/clinic	No	07/03/2017	0	0	0
2015418643	713	rural/clinic	No	07/03/2017	0	0	0
2012295966	713	rural/clinic	No	07/03/2017	0	0	0
2015339519	713	rural/clinic	No	07/03/2017	0	0	0
2015414648	713	rural/clinic	No	07/03/2017	0	0	0
2015339520	713	rural/clinic	No	07/03/2017	0	0	0
2015384479	713	rural/clinic	No	07/03/2017	0	0	0
2015339521	713	rural/clinic	No	07/03/2017	0	0	0
2015382498	713	rural/clinic	No	07/03/2017	0	0	0

2015339810	713	rural/clinic	No	07/03/2017	0	0	0
2012322916	713	rural/clinic	No	07/03/2017	0	0	0
2015382495	713	rural/clinic	No	07/03/2017	0	0	0
2012322924	713	rural/clinic	No	07/03/2017	0	0	0
2015339815	713	rural/clinic	No	07/03/2017	0	0	0
2015390301	713	rural/clinic	No	07/03/2017	0	0	0
2015382496	713	rural/clinic	No	07/03/2017	0	0	0
2015334508	713	rural/clinic	No	07/03/2017	0	0	0
2012322920	713	rural/clinic	No	07/03/2017	0	0	0
2015382497	713	rural/clinic	No	07/03/2017	0	0	0
2015382499	713	rural/clinic	No	07/03/2017	0	0	0
2015339816	713	rural/clinic	No	07/03/2017	0	0	0
2015382500	713	rural/clinic	No	07/03/2017	0	0	0
2015368872	713	rural/clinic	No	07/03/2017	0	0	0
2015368874	713	rural/clinic	No	07/03/2017	0	0	0
2015368870	713	rural/clinic	No	07/03/2017	0	0	0
2015368873	713	rural/clinic	No	07/03/2017	0	0	0
2015369726	713	rural/clinic	No	07/03/2017	0	0	0
2013253940	713	rural/clinic	No	07/03/2017	0	0	0
2012322922	713	rural/clinic	No	07/03/2017	0	0	0
2012322930	713	rural/clinic	No	07/03/2017	0	0	0
2011204768	713	rural/clinic	No	07/03/2017	0	0	0
2012322929	713	rural/clinic	No	07/03/2017	0	0	0
2015369727	713	rural/clinic	No	07/03/2017	0	0	0
2013253941	713	rural/clinic	No	07/03/2017	0	0	0
2012322933	713	rural/clinic	No	07/03/2017	0	0	0
2011204769	713	rural/clinic	No	07/03/2017	0	0	0
2015368875	713	rural/clinic	No	07/03/2017	0	0	0
2012322934	713	rural/clinic	No	07/03/2017	0	0	0
2015295197	713	rural/clinic	No	07/03/2017	0	0	0
2015339813	713	rural/clinic	No	07/03/2017	0	0	0
2012322928	713	rural/clinic	No	07/03/2017	0	0	0
2012295139	713	rural/clinic	No	07/03/2017	0	0	0
2015369728	713	rural/clinic	No	07/03/2017	0	0	0
2011204771	713	rural/clinic	No	07/03/2017	0	0	0
2012322913	713	rural/clinic	No	07/03/2017	0	0	0
2012321330	713	rural/clinic	No	07/03/2017	0	0	0
2012322919	713	rural/clinic	No	07/03/2017	0	0	0
2013270495	713	rural/clinic	No	07/03/2017	0	0	0
2012322927	612	rural/clinic	No	07/03/2017	0	0	0
2014359728	612	rural/clinic	No	07/03/2017	0	0	0
2014375664	612	rural/clinic	No	07/03/2017	0	0	0
2011204772	612	rural/clinic	No	07/03/2017	0	0	0
2015369162	612	rural/clinic	No	07/03/2017	0	0	0
2012322925	612	rural/clinic	No	07/03/2017	0	0	0
2013265066	612	rural/clinic	No	07/03/2017	0	0	0
2015334506	612	rural/clinic	No	07/03/2017	0	0	0
2015295196	612	rural/clinic	No	07/03/2017	0	0	0
2011204773	612	rural/clinic	No	07/03/2017	0	0	0
2013256399	612	rural/clinic	No	07/03/2017	0	0	0
2015334507	612	rural/clinic	No	07/03/2017	0	0	0
2014358677	612	rural/clinic	No	07/03/2017	0	0	0
2015355728	612	rural/clinic	No	07/03/2017	0	0	0
2015334407	612	rural/clinic	No	07/03/2017	0	0	0
2012276148	612	rural/clinic	No	07/03/2017	0	0	0
2015355727	612	rural/clinic	No	07/03/2017	0	0	0
2015414649	612	rural/clinic	No	07/03/2017	0	0	0
2014311364	612	rural/clinic	No	07/03/2017	0	0	0
2011204778	612	rural/clinic	No	07/03/2017	0	0	0
2015335423	612	rural/clinic	No	07/03/2017	0	0	0
2015334097	612	rural/clinic	No	07/03/2017	0	0	0
2015289279	612	rural/clinic	No	07/03/2017	0	0	0
2015286857	612	rural/clinic	No	07/03/2017	0	0	0
2012361533	612	rural/clinic	No	07/03/2017	0	0	0
2015334096	612	rural/clinic	No	07/03/2017	0	0	0
2015335424	448	rural/clinic	No	07/03/2017	0	0	0
2011222300	448	rural/clinic	No	07/03/2017	0	0	0
2012261182	448	rural/clinic	No	07/03/2017	0	0	0
2012293002	448	rural/clinic	No	07/03/2017	0	0	0
2015335425	448	rural/clinic	No	07/03/2017	0	0	0
2015334201	448	rural/clinic	No	07/03/2017	0	0	0
2014363869	448	rural/clinic	No	07/03/2017	0	0	0
2015337954	448	rural/clinic	No	07/03/2017	0	0	0
2015335575	448	rural/clinic	No	07/03/2017	0	0	0
2012261379	448	rural/clinic	No	07/03/2017	0	0	0
2011204776	448	rural/clinic	No	07/03/2017	0	0	0
2015337955	448	rural/clinic	No	07/03/2017	0	0	0
2014311365	448	rural/clinic	No	07/03/2017	0	0	0
2015286856	448	rural/clinic	No	07/03/2017	0	0	0
2012330881	448	rural/clinic	No	07/03/2017	0	0	0
2014319629	448	rural/clinic	No	07/03/2017	0	0	0
2014311366	448	rural/clinic	No	07/03/2017	0	0	0

2014308888	448	rural/clinic	No	07/03/2017	0	0	0
2011222299	448	rural/clinic	No	07/03/2017	0	0	0
2012276147	448	rural/clinic	No	07/03/2017	0	0	0
2014308889	448	rural/clinic	No	07/03/2017	0	0	0
2014307408	448	rural/clinic	No	07/03/2017	0	0	0
2014365704	448	rural/clinic	No	07/03/2017	0	0	0
2014307409	448	rural/clinic	No	07/03/2017	0	0	0
2015363006	448	rural/clinic	No	07/03/2017	0	0	0
2014350364	448	rural/clinic	No	07/03/2017	0	0	0
2015377028	448	rural/clinic	No	03/03/2017	0	0	0
2015377028	448	rural/clinic	No	03/03/2017	0	0	0
2015384529	448	rural/clinic	No	03/03/2017	0	0	0
2015384529	448	rural/clinic	No	03/03/2017	0	0	0
2015377027	448	rural/clinic	No	03/03/2017	0	0	0
2015377027	448	rural/clinic	No	03/03/2017	0	0	0
2015373248	448	rural/clinic	No	03/03/2017	0	0	0
2015373248	448	rural/clinic	No	03/03/2017	0	0	0
2012322931	448	rural/clinic	No	07/03/2017	0	0	0
2014365377	448	rural/clinic	No	07/03/2017	0	0	0
2011223602	448	rural/clinic	No	07/03/2017	0	0	0
2012322917	448	rural/clinic	No	07/03/2017	0	0	0
20133266068	448	rural/clinic	No	07/03/2017	0	0	0
2014365376	448	rural/clinic	No	07/03/2017	0	0	0
2015334509	448	rural/clinic	No	07/03/2017	0	0	0
2015320864	448	rural/clinic	No	07/03/2017	0	0	0
2014365378	449	rural/clinic	No	07/03/2017	0	0	0
2015340487	449	rural/clinic	No	03/03/2017	0	0	0
2015320865	449	rural/clinic	No	07/03/2017	0	0	0
2015363051	449	rural/clinic	No	07/03/2017	0	0	0
2015320866	449	rural/clinic	No	07/03/2017	0	0	0
2015318716	449	rural/clinic	No	07/03/2017	0	0	0
2012361944	449	rural/clinic	No	07/03/2017	0	0	0
2014289709	449	rural/clinic	No	07/03/2017	0	0	0
2015313615	449	rural/clinic	No	07/03/2017	0	0	0
2014307410	449	rural/clinic	No	07/03/2017	0	0	0
2015372915	449	rural/clinic	No	07/03/2017	0	0	0
2015372914	449	rural/clinic	No	07/03/2017	0	0	0
2015313424	449	rural/clinic	No	07/03/2017	0	0	0
2014348231	449	rural/clinic	No	07/03/2017	0	0	0
2015372913	449	rural/clinic	No	07/03/2017	0	0	0
2015313425	449	rural/clinic	No	07/03/2017	0	0	0
2012323655	449	rural/clinic	No	17/07/2017	0	0	0
2015372912	449	rural/clinic	No	07/03/2017	0	0	0
2015313426	449	rural/clinic	No	07/03/2017	0	0	0
2015372911	449	rural/clinic	No	07/03/2017	0	0	0
2015313427	449	rural/clinic	No	07/03/2017	0	0	0
2012268781	449	rural/clinic	No	07/03/2017	0	0	0
2012359151	449	rural/clinic	No	07/03/2017	0	0	0
2012323654	449	rural/clinic	No	07/03/2017	0	0	0
2015313431	449	rural/clinic	No	07/03/2017	0	0	0
2015355905	449	rural/clinic	No	07/03/2017	0	0	0
2015313428	449	rural/clinic	No	07/03/2017	0	0	0
2015355904	449	rural/clinic	No	07/03/2017	0	0	0
2014350451	449	rural/clinic	No	07/03/2017	0	0	0
2014358182	449	rural/clinic	No	07/03/2017	0	0	0
2015313432	449	rural/clinic	No	07/03/2017	0	0	0
2014358183	449	rural/clinic	No	07/03/2017	0	0	0
2012346714	398	rural/clinic	No	07/03/2017	0	0	0
2015313430	398	rural/clinic	No	07/03/2017	0	0	0
2014358184	398	rural/clinic	No	07/03/2017	0	0	0
2012346715	398	rural/clinic	No	07/03/2017	0	0	0
2014345847	398	rural/clinic	No	07/03/2017	0	0	0
2014345848	398	rural/clinic	No	07/03/2017	0	0	0
2014371679	398	rural/clinic	No	07/03/2017	0	0	0
2012374275	398	rural/clinic	No	07/03/2017	0	0	0
2014345849	398	rural/clinic	No	07/03/2017	0	0	0
2014371678	398	rural/clinic	No	07/03/2017	0	0	0
2015351651	398	rural/clinic	No	07/03/2017	0	0	0
2015337039	277	rural/clinic	No	07/03/2017	0	0	0
2012291449	277	rural/clinic	No	07/03/2017	0	0	0
2015418188	277	rural/clinic	No	07/03/2017	0	0	0
2015418187	277	rural/clinic	No	07/03/2017	0	0	0
2012244281	277	rural/clinic	No	07/03/2017	0	0	0
2012246185	277	rural/clinic	No	07/03/2017	0	0	0
2014371497	277	rural/clinic	No	07/03/2017	0	0	0
2012246186	277	rural/clinic	No	07/03/2017	0	0	0
2015286593	277	rural/clinic	No	07/03/2017	0	0	0
2015286594	277	rural/clinic	No	07/03/2017	0	0	0
2014334169	277	rural/clinic	No	07/03/2017	0	0	0
2014338502	277	rural/clinic	No	07/03/2017	0	0	0
2014338501	277	rural/clinic	No	07/03/2017	0	0	0
2012314008	277	rural/clinic	No	07/03/2017	0	0	0

2013255255	277	rural/clinic	No	07/03/2017	0	0	0
2015376106	277	rural/clinic	No	07/03/2017	0	0	0
2015337036	277	rural/clinic	No	07/03/2017	0	0	0
2015384776	277	rural/clinic	No	07/03/2017	0	0	0
201066578	277	rural/clinic	No	07/03/2017	0	0	0
2015337358	277	rural/clinic	No	07/03/2017	0	0	0
2014371159	277	rural/clinic	No	07/03/2017	0	0	0
2015315984	277	rural/clinic	No	07/03/2017	0	0	0
201066575	277	rural/clinic	No	07/03/2017	0	0	0
2015315987	277	rural/clinic	No	07/03/2017	0	0	0
2015385940	277	rural/clinic	No	07/03/2017	0	0	0
2015315986	277	rural/clinic	No	07/03/2017	0	0	0
201066571	277	rural/clinic	No	07/03/2017	0	0	0
2015315985	277	rural/clinic	No	07/03/2017	0	0	0
201066577	277	rural/clinic	No	07/03/2017	0	0	0
2015314800	277	rural/clinic	No	28/02/2017	0	0	0
2015337038	277	rural/clinic	No	07/03/2017	0	0	0
2015337357	277	rural/clinic	No	07/03/2017	0	0	0
2012388376	277	rural/clinic	No	07/03/2017	0	0	0
2012388377	277	rural/clinic	No	07/03/2017	0	0	0
2011222798	277	rural/clinic	No	07/03/2017	0	0	0
2013276153	277	rural/clinic	No	09/03/2017	0	0	0
2015299057	277	rural/clinic	No	09/03/2017	0	0	0
2014291854	277	rural/clinic	No	09/03/2017	0	0	0
2014370216	277	rural/clinic	No	09/03/2017	0	0	0
2015397912	277	rural/clinic	No	09/03/2017	0	0	0
23	277	rural/clinic	No	09/03/2017	0	0	0
23	277	rural/clinic	No	09/03/2017	0	0	0
23	277	rural/clinic	No	09/03/2017	0	0	0
23	277	rural/clinic	No	09/03/2017	0	0	0
23	277	rural/clinic	No	09/03/2017	0	0	0
23	277	rural/clinic	No	09/03/2017	0	0	0
23	277	rural/clinic	No	09/03/2017	0	0	0
23	277	rural/clinic	No	09/03/2017	0	0	0
23	277	rural/clinic	No	09/03/2017	0	0	0
23	277	rural/clinic	No	09/03/2017	0	0	0
23	277	rural/clinic	No	09/03/2017	0	0	0
23	277	rural/clinic	No	09/03/2017	0	0	0
23	277	rural/clinic	No	09/03/2017	0	0	0
23	277	rural/clinic	No	09/03/2017	0	0	0
23	277	rural/clinic	No	09/03/2017	0	0	0
23	277	rural/clinic	No	09/03/2017	0	0	0
23	277	rural/clinic	No	09/03/2017	0	0	0
23	277	rural/clinic	No	09/03/2017	0	0	0
23	277	rural/clinic	No	09/03/2017	0	0	0
23	277	rural/clinic	No	09/03/2017	0	0	0
2011204473	277	rural/clinic	No	09/03/2017	0	0	0
2015328463	277	rural/clinic	No	10/03/2017	0	0	0
2012262488	277	rural/clinic	No	09/03/2017	0	0	0
2011200076	277	rural/clinic	No	09/03/2017	0	0	0
2015325454	277	rural/clinic	No	09/03/2017	0	0	0
2015377864	277	rural/clinic	No	09/03/2017	0	0	0
2015340835	277	rural/clinic	No	09/03/2017	0	0	0
2015344853	277	rural/clinic	No	09/03/2017	0	0	0
2015331908	277	rural/clinic	No	10/03/2017	0	0	0
2011144110	277	rural/clinic	No	09/03/2017	0	0	0
2014314652	277	rural/clinic	No	09/03/2017	0	0	0
2015302105	277	rural/clinic	No	09/03/2017	0	0	0
2014372269	277	rural/clinic	No	10/03/2017	0	0	0
2015377863	277	rural/clinic	No	09/03/2017	0	0	0
2014305215	277	rural/clinic	No	09/03/2017	0	0	0
2015332302	277	rural/clinic	No	10/03/2017	0	0	0
2015297303	277	rural/clinic	No	09/03/2017	0	0	0
2015349947	277	rural/clinic	No	09/03/2017	0	0	0
2015357634	277	rural/clinic	No	10/03/2017	0	0	0
2015331910	277	rural/clinic	No	09/03/2017	0	0	0
2015349945	277	rural/clinic	No	09/03/2017	0	0	0
2015297304	277	rural/clinic	No	09/03/2017	0	0	0
2012283592	277	rural/clinic	No	09/03/2017	0	0	0
2011235681	277	rural/clinic	No	10/03/2017	0	0	0
2015325453	277	rural/clinic	No	09/03/2017	0	0	0
2014327665	277	rural/clinic	No	10/03/2017	0	0	0
2014347984	277	rural/clinic	No	09/03/2017	0	0	0
2015346089	277	rural/clinic	No	09/03/2017	0	0	0
2015405093	277	rural/clinic	No	09/03/2017	0	0	0
2014360347	277	rural/clinic	No	10/03/2017	0	0	0
2012283593	277	rural/clinic	No	09/03/2017	0	0	0
2011125794	277	rural/clinic	No	09/03/2017	0	0	0
2014382431	277	rural/clinic	No	09/03/2017	0	0	0
2014360348	277	rural/clinic	No	09/03/2017	0	0	0
2011125795	277	rural/clinic	No	09/03/2017	0	0	0
2015413691	277	rural/clinic	No	09/03/2017	0	0	0

2011193478	277	rural/clinic	No	09/03/2017	0	0	0
2014360349	277	rural/clinic	No	09/03/2017	0	0	0
2015413695	277	rural/clinic	No	09/03/2017	0	0	0
2014297343	277	rural/clinic	No	09/03/2017	0	0	0
2015293831	277	rural/clinic	No	09/03/2017	0	0	0
2015413584	277	rural/clinic	No	09/03/2017	0	0	0
2015344727	277	rural/clinic	No	09/03/2017	0	0	0
2015413892	277	rural/clinic	No	09/03/2017	0	0	0
2015287306	277	rural/clinic	No	09/03/2017	0	0	0
2012291550	277	rural/clinic	No	09/03/2017	0	0	0
2015344852	277	rural/clinic	No	09/03/2017	0	0	0
2015287307	277	rural/clinic	No	09/03/2017	0	0	0
2015344856	277	rural/clinic	No	09/03/2017	0	0	0
2014372229	239	rural/clinic	No	09/03/2017	0	0	0
2014330643	239	rural/clinic	No	09/03/2017	0	0	0
2014372230	239	rural/clinic	No	09/03/2017	0	0	0
2014368842	239	rural/clinic	No	09/03/2017	0	0	0
2012359903	239	rural/clinic	No	09/03/2017	0	0	0
2015344858	239	rural/clinic	No	09/03/2017	0	0	0
2015293648	239	rural/clinic	No	09/03/2017	0	0	0
2014330645	239	rural/clinic	No	09/03/2017	0	0	0
2014368843	239	rural/clinic	No	09/03/2017	0	0	0
2014360350	239	rural/clinic	No	09/03/2017	0	0	0
2012359904	239	rural/clinic	No	09/03/2017	0	0	0
2015334901	239	rural/clinic	No	09/03/2017	0	0	0
2015344859	239	rural/clinic	No	09/03/2017	0	0	0
2015412255	239	rural/clinic	No	09/03/2017	0	0	0
2012359905	239	rural/clinic	No	09/03/2017	0	0	0
2015346090	239	rural/clinic	No	09/03/2017	0	0	0
2015334902	239	rural/clinic	No	09/03/2017	0	0	0
2014377407	239	rural/clinic	No	09/03/2017	0	0	0
2015344860	239	rural/clinic	No	09/03/2017	0	0	0
2015414789	239	rural/clinic	No	09/03/2017	0	0	0
2015414790	239	rural/clinic	No	09/03/2017	0	0	0
2012359906	239	rural/clinic	No	09/03/2017	0	0	0
2015413893	239	rural/clinic	No	09/03/2017	0	0	0
2015344861	239	rural/clinic	No	09/03/2017	0	0	0
2015414791	239	rural/clinic	No	09/03/2017	0	0	0
2011133604	239	rural/clinic	No	09/03/2017	0	0	0
2015297369	239	rural/clinic	No	09/03/2017	0	0	0
2015351402	239	rural/clinic	No	09/03/2017	0	0	0
2015297302	239	rural/clinic	No	09/03/2017	0	0	0
2015344072	239	rural/clinic	No	09/03/2017	0	0	0
2015335194	239	rural/clinic	No	09/03/2017	0	0	0
2015351403	239	rural/clinic	No	09/03/2017	0	0	0
2015335195	239	rural/clinic	No	09/03/2017	0	0	0
2014335992	239	rural/clinic	No	09/03/2017	0	0	0
2015351404	239	rural/clinic	No	09/03/2017	0	0	0
2015335196	239	rural/clinic	No	09/03/2017	0	0	0
2015383592	239	rural/clinic	No	09/03/2017	0	0	0
2015335197	239	rural/clinic	No	09/03/2017	0	0	0
2015351405	239	rural/clinic	No	09/03/2017	0	0	0
2015344073	239	rural/clinic	No	09/03/2017	0	0	0
2015305062	239	rural/clinic	No	09/03/2017	0	0	0
2015397913	239	rural/clinic	No	09/03/2017	0	0	0
2011192835	239	rural/clinic	No	09/03/2017	0	0	0
2014314651	239	rural/clinic	No	09/03/2017	0	0	0
2015397914	239	rural/clinic	No	09/03/2017	0	0	0
2015383593	239	rural/clinic	No	09/03/2017	0	0	0
2015351401	239	rural/clinic	No	09/03/2017	0	0	0
2011226476	239	rural/clinic	No	09/03/2017	0	0	0
2015319433	239	rural/clinic	No	09/03/2017	0	0	0
2015397915	239	rural/clinic	No	09/03/2017	0	0	0
2015319581	239	rural/clinic	No	09/03/2017	0	0	0
2015351661	239	rural/clinic	No	09/03/2017	0	0	0
2012376776	239	rural/clinic	No	09/03/2017	0	0	0
2012390999	239	rural/clinic	No	09/03/2017	0	0	0
2015319582	239	rural/clinic	No	09/03/2017	0	0	0
2014301673	239	rural/clinic	No	09/03/2017	0	0	0
2014360881	239	rural/clinic	No	09/03/2017	0	0	0
2014346893	239	rural/clinic	No	09/03/2017	0	0	0
2015412111	239	rural/clinic	No	09/03/2017	0	0	0
2014347008	239	rural/clinic	No	09/03/2017	0	0	0
2015402358	239	rural/clinic	No	09/03/2017	0	0	0
2012322687	239	rural/clinic	No	09/03/2017	0	0	0
2014360882	239	rural/clinic	No	09/03/2017	0	0	0
2015377034	239	rural/clinic	No	09/03/2017	0	0	0
2015402359	239	rural/clinic	No	09/03/2017	0	0	0
2015319583	239	rural/clinic	No	09/03/2017	0	0	0
2014360883	239	rural/clinic	No	09/03/2017	0	0	0
2015338290	239	rural/clinic	No	09/03/2017	0	0	0
2014335227	239	rural/clinic	No	09/03/2017	0	0	0

2015338289	239	rural/clinic	No	09/03/2017	0	0	0
2015351653	239	rural/clinic	No	09/03/2017	0	0	0
2014301674	239	rural/clinic	No	09/03/2017	0	0	0
2015326555	239	rural/clinic	No	09/03/2017	0	0	0
2014360884	239	rural/clinic	No	09/03/2017	0	0	0
2015338288	239	rural/clinic	No	09/03/2017	0	0	0
2015326554	278	rural/clinic	No	09/03/2017	0	0	0
2011155224	278	rural/clinic	No	09/03/2017	0	0	0
2015351654	278	rural/clinic	No	09/03/2017	0	0	0
2015326553	278	rural/clinic	No	09/03/2017	0	0	0
2012376775	278	rural/clinic	No	09/03/2017	0	0	0
2015372020	278	rural/clinic	No	09/03/2017	0	0	0
2015326556	278	rural/clinic	No	09/03/2017	0	0	0
2015351655	278	rural/clinic	No	09/03/2017	0	0	0
2015402513	278	rural/clinic	No	09/03/2017	0	0	0
2013274239	278	rural/clinic	No	09/03/2017	0	0	0
2015377862	278	rural/clinic	No	09/03/2017	0	0	0
2015402514	278	rural/clinic	No	09/03/2017	0	0	0
2014361047	278	rural/clinic	No	09/03/2017	0	0	0
2015351656/D	278	rural/clinic	No	09/03/2017	0	0	0
2013274241	278	rural/clinic	No	09/03/2017	0	0	0
2015351657	278	rural/clinic	No	09/03/2017	0	0	0
2015301093	278	rural/clinic	No	09/03/2017	0	0	0
2015402515	278	rural/clinic	No	09/03/2017	0	0	0
2014327082	278	rural/clinic	No	09/03/2017	0	0	0
2015351658	278	rural/clinic	No	09/03/2017	0	0	0
2015301094	278	rural/clinic	No	09/03/2017	0	0	0
2015351659	278	rural/clinic	No	09/03/2017	0	0	0
2015349949	278	rural/clinic	No	09/03/2017	0	0	0
2015325040	278	rural/clinic	No	09/03/2017	0	0	0
2014361046	278	rural/clinic	No	09/03/2017	0	0	0
2015351660	278	rural/clinic	No	09/03/2017	0	0	0
2015301096	278	rural/clinic	No	09/03/2017	0	0	0
2014356962	278	rural/clinic	No	09/03/2017	0	0	0
2014356965	278	rural/clinic	No	09/03/2017	0	0	0
2015301097	278	rural/clinic	No	09/03/2017	0	0	0
2014310206	278	rural/clinic	No	09/03/2017	0	0	0
2014327084	278	rural/clinic	No	09/03/2017	0	0	0
2015349944	278	rural/clinic	No	09/03/2017	0	0	0
2013255305	278	rural/clinic	No	09/03/2017	0	0	0
2014368839	278	rural/clinic	No	09/03/2017	0	0	0
2014303292	278	rural/clinic	No	09/03/2017	0	0	0
2014327083	278	rural/clinic	No	09/03/2017	0	0	0
2015360501	278	rural/clinic	No	09/03/2017	0	0	0
2014356960	278	rural/clinic	No	09/03/2017	0	0	0
2014303290	278	rural/clinic	No	09/03/2017	0	0	0
2014356961	278	rural/clinic	No	09/03/2017	0	0	0
2014288129	278	rural/clinic	No	09/03/2017	0	0	0
2015331882	278	rural/clinic	No	09/03/2017	0	0	0
2014356964	278	rural/clinic	No	09/03/2017	0	0	0
2015331884	278	rural/clinic	No	09/03/2017	0	0	0
WRONGWRONG	278	rural/clinic	No	09/03/2017	0	0	0
WRONGWRONG	278	rural/clinic	No	09/03/2017	0	0	0
WRONGWRONG	278	rural/clinic	No	09/03/2017	0	0	0
WRONGWRONG	278	rural/clinic	No	09/03/2017	0	0	0
WRONGWRONG	278	rural/clinic	No	09/03/2017	0	0	0
WRONGWRONG	278	rural/clinic	No	09/03/2017	0	0	0
2015331883	278	rural/clinic	No	09/03/2017	0	0	0
2012253922	278	rural/clinic	No	09/03/2017	0	0	0
2011224435	278	rural/clinic	No	09/03/2017	0	0	0
2015289836	278	rural/clinic	No	09/03/2017	0	0	0
2014356963	278	rural/clinic	No	09/03/2017	0	0	0
2014303293	278	rural/clinic	No	09/03/2017	0	0	0
2012313806	278	rural/clinic	No	09/03/2017	0	0	0
2015352440	278	rural/clinic	No	09/03/2017	0	0	0
2015336702	278	rural/clinic	No	09/03/2017	0	0	0
2015301251	278	rural/clinic	No	09/03/2017	0	0	0
2014296315	278	rural/clinic	No	09/03/2017	0	0	0
2011142715	278	rural/clinic	No	09/03/2017	0	0	0
2011145392	278	rural/clinic	No	09/03/2017	0	0	0
2015302104	278	rural/clinic	No	09/03/2017	0	0	0
2014368840	278	rural/clinic	No	09/03/2017	0	0	0
2013262218	278	rural/clinic	No	09/03/2017	0	0	0
2015336701	278	rural/clinic	No	09/03/2017	0	0	0
2014350042	278	rural/clinic	No	09/03/2017	0	0	0
2011145394	278	rural/clinic	No	09/03/2017	0	0	0
2014385499	278	rural/clinic	No	09/03/2017	0	0	0
2014368841	278	rural/clinic	No	09/03/2017	0	0	0
2015286281	278	rural/clinic	No	09/03/2017	0	0	0
2015377033	278	rural/clinic	No	09/03/2017	0	0	0
2011145393	278	rural/clinic	No	09/03/2017	0	0	0
2014302514	278	rural/clinic	No	09/03/2017	0	0	0

2015286282	278	rural/clinic	No	09/03/2017	0	0	0
2015377032	278	rural/clinic	No	09/03/2017	0	0	0
2015339001	278	rural/clinic	No	09/03/2017	0	0	0
2014319498	278	rural/clinic	No	09/03/2017	0	0	0
2014385500	278	rural/clinic	No	09/03/2017	0	0	0
2014302515	278	rural/clinic	No	09/03/2017	0	0	0
2014296313	278	rural/clinic	No	09/03/2017	0	0	0
2015377031	278	rural/clinic	No	09/03/2017	0	0	0
2014319722	278	rural/clinic	No	09/03/2017	0	0	0
2013262217	278	rural/clinic	No	09/03/2017	0	0	0
2015339002	278	rural/clinic	No	09/03/2017	0	0	0
2015339003	278	rural/clinic	No	09/03/2017	0	0	0
2014289656	278	rural/clinic	No	09/03/2017	0	0	0
2015319578	278	rural/clinic	No	09/03/2017	0	0	0
2012369966	278	rural/clinic	No	09/03/2017	0	0	0
2012289599	278	rural/clinic	No	09/03/2017	0	0	0
2015286284	278	rural/clinic	No	09/03/2017	0	0	0
2015377318	278	rural/clinic	No	09/03/2017	0	0	0
2015286283	278	rural/clinic	No	09/03/2017	0	0	0
2015319580	278	rural/clinic	No	09/03/2017	0	0	0
2015377030	278	rural/clinic	No	09/03/2017	0	0	0
2012305584	278	rural/clinic	No	09/03/2017	0	0	0
2015335373	278	rural/clinic	No	09/03/2017	0	0	0
2015402355	278	rural/clinic	No	09/03/2017	0	0	0
2015402516	278	rural/clinic	No	09/03/2017	0	0	0
2015377162	278	rural/clinic	No	09/03/2017	0	0	0
2015377161	278	rural/clinic	No	09/03/2017	0	0	0
2015402356	278	rural/clinic	No	09/03/2017	0	0	0
2015377163	278	rural/clinic	No	09/03/2017	0	0	0
2015288602	278	rural/clinic	No	09/03/2017	0	0	0
2015340493	278	rural/clinic	No	09/03/2017	0	0	0
2015377164	278	rural/clinic	No	09/03/2017	0	0	0
2015288601	278	rural/clinic	No	09/03/2017	0	0	0
2015402517	278	rural/clinic	No	09/03/2017	0	0	0
2015340494	278	rural/clinic	No	09/03/2017	0	0	0
2012289598	278	rural/clinic	No	09/03/2017	0	0	0
2014291855	278	rural/clinic	No	09/03/2017	0	0	0
2015301095	278	rural/clinic	No	09/03/2017	0	0	0
2015340495	278	rural/clinic	No	09/03/2017	0	0	0
2015402518	278	rural/clinic	No	09/03/2017	0	0	0
2014291856	278	rural/clinic	No	09/03/2017	0	0	0
2014347007	278	rural/clinic	No	09/03/2017	0	0	0
2015340496	278	rural/clinic	No	09/03/2017	0	0	0
2015340497	278	rural/clinic	No	09/03/2017	0	0	0
2014350043	278	rural/clinic	No	09/03/2017	0	0	0
2015402519	278	rural/clinic	No	09/03/2017	0	0	0
2015340498	278	rural/clinic	No	09/03/2017	0	0	0
2014291857	278	rural/clinic	No	09/03/2017	0	0	0
2015340499	278	rural/clinic	No	09/03/2017	0	0	0
2015385515	278	rural/clinic	No	09/03/2017	0	0	0
2015331906	278	rural/clinic	No	09/03/2017	0	0	0
2014291858	278	rural/clinic	No	09/03/2017	0	0	0
2015340500	278	rural/clinic	No	09/03/2017	0	0	0
2014362807	278	rural/clinic	No	09/03/2017	0	0	0
2015331907	278	rural/clinic	No	09/03/2017	0	0	0
2014362808	278	rural/clinic	No	09/03/2017	0	0	0
2015368205	278	rural/clinic	No	09/03/2017	0	0	0
2015414373	278	rural/clinic	No	09/03/2017	0	0	0
2014350041	278	rural/clinic	No	09/03/2017	0	0	0
2011198686	278	rural/clinic	No	09/03/2017	0	0	0
2011198689	278	rural/clinic	No	09/03/2017	0	0	0
2014345850	278	rural/clinic	No	09/03/2017	0	0	0
2015325987	278	rural/clinic	No	09/03/2017	0	0	0
2015369657	278	rural/clinic	No	09/03/2017	0	0	0
2015369658	278	rural/clinic	No	09/03/2017	0	0	0
2015288501	278	rural/clinic	No	09/03/2017	0	0	0
2014368019	278	rural/clinic	No	09/03/2017	0	0	0
2014346304	278	rural/clinic	No	09/03/2017	0	0	0
2015369659	278	rural/clinic	No	09/03/2017	0	0	0
2014346303	278	rural/clinic	No	09/03/2017	0	0	0
2015369660	278	rural/clinic	No	09/03/2017	0	0	0
2012251536	278	rural/clinic	No	09/03/2017	0	0	0
2015369661	278	rural/clinic	No	09/03/2017	0	0	0
2015325041	278	rural/clinic	No	09/03/2017	0	0	0
2015369662	278	rural/clinic	No	09/03/2017	0	0	0
2011198688	278	rural/clinic	No	09/03/2017	0	0	0
2015375972	278	rural/clinic	No	09/03/2017	0	0	0
2015289835	278	rural/clinic	No	09/03/2017	0	0	0
2015375057	278	rural/clinic	No	09/03/2017	0	0	0
2012291841	278	rural/clinic	No	09/03/2017	0	0	0
2015352437	278	rural/clinic	No	09/03/2017	0	0	0
2015352438	278	rural/clinic	No	09/03/2017	0	0	0

2015324229	278	rural/clinic	No	09/03/2017	0	0	0
2015324230	278	rural/clinic	No	09/03/2017	0	0	0
2014346306	278	rural/clinic	No	09/03/2017	0	0	0
2014346305	278	rural/clinic	No	09/03/2017	0	0	0
2015352433	278	rural/clinic	No	09/03/2017	0	0	0
2014346301	278	rural/clinic	No	09/03/2017	0	0	0
2012291842	278	rural/clinic	No	09/03/2017	0	0	0
2014346302	278	rural/clinic	No	09/03/2017	0	0	0
2015332906	278	rural/clinic	No	09/03/2017	0	0	0
2014383967	278	rural/clinic	No	09/03/2017	0	0	0
2015352434	278	rural/clinic	No	09/03/2017	0	0	0
2015324228	278	rural/clinic	No	09/03/2017	0	0	0
2015352435	278	rural/clinic	No	09/03/2017	0	0	0
2015412081	278	rural/clinic	No	09/03/2017	0	0	0
2015352439	278	rural/clinic	No	09/03/2017	0	0	0
2015352436	278	rural/clinic	No	09/03/2017	0	0	0
2015331909	278	rural/clinic	No	10/03/2017	0	0	0
2015412076	278	rural/clinic	No	07/03/2017	0	0	0
2015412076	278	rural/clinic	No	07/03/2017	0	0	0
2015355728	278	rural/clinic	No	07/03/2017	0	0	0
2015355728	278	rural/clinic	No	07/03/2017	0	0	0
2015337954	278	rural/clinic	No	07/03/2017	0	0	0
2015337954	278	rural/clinic	No	07/03/2017	0	0	0
2015300681	278	rural/clinic	No	07/03/2017	0	0	0
2015300681	420	rural/clinic	No	07/03/2017	0	0	0
2011113946	420	rural/clinic	No	07/03/2017	0	0	0
2011113946	420	rural/clinic	No	07/03/2017	0	0	0
2011204773	420	rural/clinic	No	07/03/2017	0	0	0
2011204773	420	rural/clinic	No	07/03/2017	0	0	0
2014307409	420	rural/clinic	No	07/03/2017	0	0	0
2014307409	420	rural/clinic	No	07/03/2017	0	0	0
2015351652	420	rural/clinic	No	07/03/2017	0	0	0
2015351652	420	rural/clinic	No	07/03/2017	0	0	0
2015334705	420	rural/clinic	No	07/03/2017	0	0	0
2015339816	420	rural/clinic	No	07/03/2017	0	0	0
2015339816	420	rural/clinic	No	07/03/2017	0	0	0
2015297365	420	rural/clinic	No	07/03/2017	0	0	0
2012350100	420	rural/clinic	No	07/03/2017	0	0	0
2012350100	420	rural/clinic	No	07/03/2017	0	0	0
2012259861	326	rural/clinic	No	07/03/2017	0	0	0
2012259861	326	rural/clinic	No	07/03/2017	0	0	0
2011168518	326	rural/clinic	No	10/03/2017	0	0	0
2015379021	326	rural/clinic	No	07/03/2017	0	0	0
2015379021	326	rural/clinic	No	07/03/2017	0	0	0
2012295966	326	rural/clinic	No	07/03/2017	0	0	0
2012295966	326	rural/clinic	No	07/03/2017	0	0	0
2015369727	326	rural/clinic	No	07/03/2017	0	0	0
2015369727	326	rural/clinic	No	07/03/2017	0	0	0
2012276148	326	rural/clinic	No	07/03/2017	0	0	0
2012276148	326	rural/clinic	No	07/03/2017	0	0	0
20133266068	326	rural/clinic	No	07/03/2017	0	0	0
20133266068	326	rural/clinic	No	07/03/2017	0	0	0
2015414649	326	rural/clinic	No	07/03/2017	0	0	0
2015414649	326	rural/clinic	No	07/03/2017	0	0	0
2015372911	326	rural/clinic	No	07/03/2017	0	0	0
2015372911	326	rural/clinic	No	07/03/2017	0	0	0
2015397518	326	rural/clinic	No	13/03/2017	0	0	0
2015397519	326	rural/clinic	No	13/03/2017	0	0	0
2014327114	326	rural/clinic	No	07/03/2017	0	0	0
2015397514	326	rural/clinic	No	13/03/2017	0	0	0
2015397516	326	rural/clinic	No	13/03/2017	0	0	0
2015397517	326	rural/clinic	No	13/03/2017	0	0	0
2015397520	326	rural/clinic	No	13/03/2017	0	0	0
2015340491	326	rural/clinic	No	03/03/2017	0	0	0
2015373166	326	rural/clinic	No	03/03/2017	0	0	0
2014291856	326	rural/clinic	No	09/03/2017	0	0	0
2014291856	326	rural/clinic	No	09/03/2017	0	0	0
2015340496	326	rural/clinic	No	09/03/2017	0	0	0
2015340496	326	rural/clinic	No	09/03/2017	0	0	0
2015331906	326	rural/clinic	No	09/03/2017	0	0	0
2015331906	326	rural/clinic	No	09/03/2017	0	0	0
2014345850	326	rural/clinic	No	09/03/2017	0	0	0
2014345850	326	rural/clinic	No	09/03/2017	0	0	0
2015337358	525	rural/clinic	No	07/03/2017	0	0	0
2015337358	525	rural/clinic	No	07/03/2017	0	0	0
2015397915	525	rural/clinic	No	09/03/2017	0	0	0
2015397915	525	rural/clinic	No	09/03/2017	0	0	0
2015375972	525	rural/clinic	No	09/03/2017	0	0	0
2015375972	525	rural/clinic	No	09/03/2017	0	0	0
2015372020	525	rural/clinic	No	09/03/2017	0	0	0
2015372020	525	rural/clinic	No	09/03/2017	0	0	0
2015331882	525	rural/clinic	No	09/03/2017	0	0	0

2015331882	525	rural/clinic	No	09/03/2017	0	0	0
2014360348	525	rural/clinic	No	09/03/2017	0	0	0
2014360348	525	rural/clinic	No	09/03/2017	0	0	0
2015319433	525	rural/clinic	No	09/03/2017	0	0	0
2015319433	525	rural/clinic	No	09/03/2017	0	0	0
2012359903	525	rural/clinic	No	09/03/2017	0	0	0
2012359903	525	rural/clinic	No	09/03/2017	0	0	0
2015351402	525	rural/clinic	No	09/03/2017	0	0	0
2015351402	525	rural/clinic	No	09/03/2017	0	0	0
2014346893	525	rural/clinic	No	09/03/2017	0	0	0
2014346893	525	rural/clinic	No	09/03/2017	0	0	0
2014330645	525	rural/clinic	No	09/03/2017	0	0	0
2015414790	525	rural/clinic	No	09/03/2017	0	0	0
2015414790	525	rural/clinic	No	09/03/2017	0	0	0
2015357634	525	rural/clinic	No	10/03/2017	0	0	0
2015357634	525	rural/clinic	No	10/03/2017	0	0	0
2014385500	525	rural/clinic	No	09/03/2017	0	0	0
2014385500	525	rural/clinic	No	09/03/2017	0	0	0
2015339003	525	rural/clinic	No	09/03/2017	0	0	0
2015339003	525	rural/clinic	No	09/03/2017	0	0	0
2015402519	525	rural/clinic	No	09/03/2017	0	0	0
2015402519	525	rural/clinic	No	09/03/2017	0	0	0
20170000004	525	rural/clinic	No	14/03/2017	0	0	0
20170000005	525	rural/clinic	No	14/03/2017	0	0	0
20170000006	525	rural/clinic	No	14/03/2017	0	0	0
20170000007	525	rural/clinic	No	14/03/2017	0	0	0
2015310602	525	rural/clinic	No	14/03/2017	0	0	0
2014344403	525	rural/clinic	No	14/03/2017	0	0	0
2015344314	525	rural/clinic	No	14/03/2017	0	0	0
2015334409	525	rural/clinic	No	14/03/2017	0	0	0
2015355731	525	rural/clinic	No	14/03/2017	0	0	0
2012317280	525	rural/clinic	No	14/03/2017	0	0	0
2WRONG	525	rural/clinic	No	14/03/2017	0	0	0
2015377822	525	rural/clinic	No	14/03/2017	0	0	0
2015377823	525	rural/clinic	No	14/03/2017	0	0	0
2015377825	525	rural/clinic	No	14/03/2017	0	0	0
2014369040	525	rural/clinic	No	14/03/2017	0	0	0
2012352347	525	rural/clinic	No	14/03/2017	0	0	0
2013266069	525	rural/clinic	No	14/03/2017	0	0	0
2011113948	525	rural/clinic	No	14/03/2017	0	0	0
2013266070	525	rural/clinic	No	14/03/2017	0	0	0
2015412082	525	rural/clinic	No	14/03/2017	0	0	0
2014357304	525	rural/clinic	No	14/03/2017	0	0	0
2015412083	525	rural/clinic	No	14/03/2017	0	0	0
2015412085	525	rural/clinic	No	14/03/2017	0	0	0
2012245729	525	rural/clinic	No	14/03/2017	0	0	0
2015314342	525	rural/clinic	No	15/03/2017	0	0	0
2012313807	525	rural/clinic	No	14/03/2017	0	0	0
2015314344	525	rural/clinic	No	15/03/2017	0	0	0
2012245730	525	rural/clinic	No	14/03/2017	0	0	0
2015314327	525	rural/clinic	No	15/03/2017	0	0	0
2012245731	525	rural/clinic	No	14/03/2017	0	0	0
2015418369	525	rural/clinic	No	14/03/2017	0	0	0
2015314333	525	rural/clinic	No	15/03/2017	0	0	0
2015352357	525	rural/clinic	No	14/03/2017	0	0	0
2015314335	525	rural/clinic	No	15/03/2017	0	0	0
2015352358	525	rural/clinic	No	14/03/2017	0	0	0
2015352359	525	rural/clinic	No	14/03/2017	0	0	0
2013283502	525	rural/clinic	No	14/03/2017	0	0	0
2015314336	525	rural/clinic	No	15/03/2017	0	0	0
2014288130	525	rural/clinic	No	14/03/2017	0	0	0
2015352360	525	rural/clinic	No	14/03/2017	0	0	0
2015314339	525	rural/clinic	No	15/03/2017	0	0	0
2015390946	525	rural/clinic	No	14/03/2017	0	0	0
2012313808	525	rural/clinic	No	14/03/2017	0	0	0
2015390948	525	rural/clinic	No	14/03/2017	0	0	0
2015314348	525	rural/clinic	No	15/03/2017	0	0	0
2015390949	525	rural/clinic	No	14/03/2017	0	0	0
2015377627	525	rural/clinic	No	14/03/2017	0	0	0
2015314349	525	rural/clinic	No	15/03/2017	0	0	0
2015377628	525	rural/clinic	No	14/03/2017	0	0	0
2015314329	525	rural/clinic	No	15/03/2017	0	0	0
2015314324	525	rural/clinic	No	15/03/2017	0	0	0
2015377629	525	rural/clinic	No	14/03/2017	0	0	0
2014334345	525	rural/clinic	No	14/03/2017	0	0	0
2015314331	525	rural/clinic	No	15/03/2017	0	0	0
2015377630	525	rural/clinic	No	14/03/2017	0	0	0
2014287970	525	rural/clinic	No	14/03/2017	0	0	0
2014351199	525	rural/clinic	No	14/03/2017	0	0	0
2015314343	525	rural/clinic	No	15/03/2017	0	0	0
2014287971	525	rural/clinic	No	14/03/2017	0	0	0
2012284572	525	rural/clinic	No	14/03/2017	0	0	0

2014363870	525	rural/clinic	No	14/03/2017	0	0	0
2012305968	525	rural/clinic	No	14/03/2017	0	0	0
2015354474	525	rural/clinic	No	15/03/2017	0	0	0
2014358185	525	rural/clinic	No	14/03/2017	0	0	0
2015362801	525	rural/clinic	No	14/03/2017	0	0	0
2015376112	525	rural/clinic	No	14/03/2017	0	0	0
2015354476	525	rural/clinic	No	15/03/2017	0	0	0
2015362802	526	rural/clinic	No	14/03/2017	0	0	0
2015413641	526	rural/clinic	No	14/03/2017	0	0	0
2015369386	526	rural/clinic	No	14/03/2017	0	0	0
2015362803	526	rural/clinic	No	14/03/2017	0	0	0
2015369387	526	rural/clinic	No	14/03/2017	0	0	0
2015369388	526	rural/clinic	No	14/03/2017	0	0	0
2012259867	526	rural/clinic	No	14/03/2017	0	0	0
2015405912	112	rural/clinic	No	14/03/2017	0	0	0
2015354478	112	rural/clinic	No	15/03/2017	0	0	0
2012259866	112	rural/clinic	No	14/03/2017	0	0	0
2015354475	112	rural/clinic	No	15/03/2017	0	0	0
2013261292	112	rural/clinic	No	14/03/2017	0	0	0
2015354481	112	rural/clinic	No	15/03/2017	0	0	0
2013261291	112	rural/clinic	No	14/03/2017	0	0	0
2012350621	112	rural/clinic	No	15/03/2017	0	0	0
2014363192	112	rural/clinic	No	14/03/2017	0	0	0
2015363052	714	rural/clinic	No	14/03/2017	0	0	0
2012263744	714	rural/clinic	No	15/03/2017	0	0	0
2014383526	714	rural/clinic	No	14/03/2017	0	0	0
2013255256	714	rural/clinic	No	14/03/2017	0	0	0
2012350623	714	rural/clinic	No	15/03/2017	0	0	0
2012350622	714	rural/clinic	No	15/03/2017	0	0	0
2012263745	714	rural/clinic	No	15/03/2017	0	0	0
2015360716	714	rural/clinic	No	14/03/2017	0	0	0
2012263746	714	rural/clinic	No	15/03/2017	0	0	0
2013273640	714	rural/clinic	No	14/03/2017	0	0	0
2012350624	714	rural/clinic	No	15/03/2017	0	0	0
2015331638	714	rural/clinic	No	15/03/2017	0	0	0
2011142799	714	rural/clinic	No	14/03/2017	0	0	0
2011142798	714	rural/clinic	No	14/03/2017	0	0	0
2015293362	714	rural/clinic	No	14/03/2017	0	0	0
2015376110	714	rural/clinic	No	14/03/2017	0	0	0
2015376111	714	rural/clinic	No	14/03/2017	0	0	0
2015324430	714	rural/clinic	No	14/03/2017	0	0	0
2015338455	714	rural/clinic	No	14/03/2017	0	0	0
2015324431	714	rural/clinic	No	14/03/2017	0	0	0
2015338456	714	rural/clinic	No	14/03/2017	0	0	0
2015315988	714	rural/clinic	No	14/03/2017	0	0	0
2015338453	714	rural/clinic	No	14/03/2017	0	0	0
2012365469	714	rural/clinic	No	14/03/2017	0	0	0
2015338454	714	rural/clinic	No	14/03/2017	0	0	0
2015337045	714	rural/clinic	No	14/03/2017	0	0	0
2012359152	715	rural/clinic	No	14/03/2017	0	0	0
2015337614	715	rural/clinic	No	14/03/2017	0	0	0
2012359153	715	rural/clinic	No	14/03/2017	0	0	0
2015385942	715	rural/clinic	No	14/03/2017	0	0	0
2015293363	715	rural/clinic	No	14/03/2017	0	0	0
2014311542	715	rural/clinic	No	14/03/2017	0	0	0
2015360714	715	rural/clinic	No	14/03/2017	0	0	0
2015360715	715	rural/clinic	No	14/03/2017	0	0	0
2011223201	715	rural/clinic	No	14/03/2017	0	0	0
2015315989	715	rural/clinic	No	14/03/2017	0	0	0
2015368562	715	rural/clinic	No	14/03/2017	0	0	0
2015337616	715	rural/clinic	No	14/03/2017	0	0	0
2015368563	715	rural/clinic	No	14/03/2017	0	0	0
2015325928	715	rural/clinic	No	14/03/2017	0	0	0
2015384779	715	rural/clinic	No	14/03/2017	0	0	0
2015368564	450	rural/clinic	No	14/03/2017	0	0	0
2015313433	450	rural/clinic	No	14/03/2017	0	0	0
2015384780	450	rural/clinic	No	14/03/2017	0	0	0
2012358278	450	rural/clinic	No	14/03/2017	0	0	0
2015365865	450	rural/clinic	No	14/03/2017	0	0	0
2015337359	450	rural/clinic	No	14/03/2017	0	0	0
2014288398	450	rural/clinic	No	14/03/2017	0	0	0
2014311543	450	rural/clinic	No	14/03/2017	0	0	0
2012322935	450	rural/clinic	No	14/03/2017	0	0	0
2012358279	450	rural/clinic	No	14/03/2017	0	0	0
2014344404	450	rural/clinic	No	14/03/2017	0	0	0
2015324429	450	rural/clinic	No	14/03/2017	0	0	0
2014353630	450	rural/clinic	No	15/03/2017	0	0	0
2015313434	450	rural/clinic	No	14/03/2017	0	0	0
2015337613	450	rural/clinic	No	14/03/2017	0	0	0
2014344402	450	rural/clinic	No	14/03/2017	0	0	0
2015313435	450	rural/clinic	No	14/03/2017	0	0	0
2015394628	450	rural/clinic	No	15/03/2017	0	0	0

2015313436	450	rural/clinic	No	14/03/2017	0	0	0
2015344318	450	rural/clinic	No	14/03/2017	0	0	0
2015313437	450	rural/clinic	No	14/03/2017	0	0	0
2015394629	450	rural/clinic	No	15/03/2017	0	0	0
2011223603	450	rural/clinic	No	14/03/2017	0	0	0
2015337041	450	rural/clinic	No	14/03/2017	0	0	0
2015313438	450	rural/clinic	No	14/03/2017	0	0	0
2015337615	450	rural/clinic	No	14/03/2017	0	0	0
2012388378	450	rural/clinic	No	14/03/2017	0	0	0
2015394630	450	rural/clinic	No	15/03/2017	0	0	0
2014303071	450	rural/clinic	No	14/03/2017	0	0	0
2015337043	450	rural/clinic	No	14/03/2017	0	0	0
2015322578	450	rural/clinic	No	15/03/2017	0	0	0
2015288502	450	rural/clinic	No	14/03/2017	0	0	0
2015337044	450	rural/clinic	No	14/03/2017	0	0	0
2015322579	450	rural/clinic	No	15/03/2017	0	0	0
2015362350	450	rural/clinic	No	14/03/2017	0	0	0
2015337046	450	rural/clinic	No	14/03/2017	0	0	0
2011212648	450	rural/clinic	No	14/03/2017	0	0	0
2015324466	450	rural/clinic	No	14/03/2017	0	0	0
2015324467	450	rural/clinic	No	14/03/2017	0	0	0
2015322586	450	rural/clinic	No	15/03/2017	0	0	0
2015324035	450	rural/clinic	No	14/03/2017	0	0	0
2014291593	450	rural/clinic	No	14/03/2017	0	0	0
2015322583	450	rural/clinic	No	15/03/2017	0	0	0
2014291592	450	rural/clinic	No	14/03/2017	0	0	0
2015344317	450	rural/clinic	No	14/03/2017	0	0	0
2015364351	450	rural/clinic	No	14/03/2017	0	0	0
2015337042	792	rural/clinic	No	14/03/2017	0	0	0
2015364352	792	rural/clinic	No	14/03/2017	0	0	0
2015344316	792	rural/clinic	No	14/03/2017	0	0	0
2015337047	792	rural/clinic	No	14/03/2017	0	0	0
2012322936	792	rural/clinic	No	14/03/2017	0	0	0
2013249414	792	rural/clinic	No	14/03/2017	0	0	0
201066579	792	rural/clinic	No	14/03/2017	0	0	0
2015384778	792	rural/clinic	No	14/03/2017	0	0	0
2012265279	792	rural/clinic	No	14/03/2017	0	0	0
2013249279	792	rural/clinic	No	14/03/2017	0	0	0
2012388379	792	rural/clinic	No	14/03/2017	0	0	0
2012304322	792	rural/clinic	No	14/03/2017	0	0	0
2014291859	792	rural/clinic	No	15/03/2017	0	0	0
2012304323	792	rural/clinic	No	14/03/2017	0	0	0
2014288397	792	rural/clinic	No	14/03/2017	0	0	0
2014288396	792	rural/clinic	No	14/03/2017	0	0	0
2012304321	792	rural/clinic	No	14/03/2017	0	0	0
2011212959	792	rural/clinic	No	14/03/2017	0	0	0
2014288573	792	rural/clinic	No	14/03/2017	0	0	0
2014288574	792	rural/clinic	No	14/03/2017	0	0	0
2015355729	792	rural/clinic	No	14/03/2017	0	0	0
2015355730	792	rural/clinic	No	14/03/2017	0	0	0
2014359729	792	rural/clinic	No	14/03/2017	0	0	0
2015335427	792	rural/clinic	No	14/03/2017	0	0	0
2015384803	792	rural/clinic	No	14/03/2017	0	0	0
2012321331	792	rural/clinic	No	14/03/2017	0	0	0
2015335426	792	rural/clinic	No	14/03/2017	0	0	0
2014375667	792	rural/clinic	No	14/03/2017	0	0	0
2013270494	792	rural/clinic	No	14/03/2017	0	0	0
2015369919	155	rural/clinic	No	14/03/2017	0	0	0
2015293934	155	rural/clinic	No	14/03/2017	0	0	0
2014297960	155	rural/clinic	No	14/03/2017	0	0	0
2015377824	155	rural/clinic	No	14/03/2017	0	0	0
2015394627	155	rural/clinic	No	15/03/2017	0	0	0
2012365470	155	rural/clinic	No	14/03/2017	0	0	0
2015369917	155	rural/clinic	No	14/03/2017	0	0	0
2015334408	155	rural/clinic	No	14/03/2017	0	0	0
2015369918	155	rural/clinic	No	14/03/2017	0	0	0
2011223604	155	rural/clinic	No	14/03/2017	0	0	0
2015394626	155	rural/clinic	No	15/03/2017	0	0	0
2014375665	155	rural/clinic	No	14/03/2017	0	0	0
2015334202	155	rural/clinic	No	14/03/2017	0	0	0
2012328999	155	rural/clinic	No	15/03/2017	0	0	0
2014375666	155	rural/clinic	No	14/03/2017	0	0	0
2015334203	155	rural/clinic	No	14/03/2017	0	0	0
2015390258	155	rural/clinic	No	14/03/2017	0	0	0
2012340798	155	rural/clinic	No	15/03/2017	0	0	0
2014291813	155	rural/clinic	No	14/03/2017	0	0	0
2015293935	155	rural/clinic	No	14/03/2017	0	0	0
2015368782	155	rural/clinic	No	14/03/2017	0	0	0
2014288677	155	rural/clinic	No	14/03/2017	0	0	0
2012340797	155	rural/clinic	No	15/03/2017	0	0	0
2015414939	155	rural/clinic	No	14/03/2017	0	0	0
2015418127	155	rural/clinic	No	14/03/2017	0	0	0

2012340799	155	rural/clinic	No	15/03/2017	0	0	0
2015418128	155	rural/clinic	No	14/03/2017	0	0	0
2015414724	155	rural/clinic	No	14/03/2017	0	0	0
2015414725	155	rural/clinic	No	14/03/2017	0	0	0
2014358202	155	rural/clinic	No	14/03/2017	0	0	0
2014318234	155	rural/clinic	No	15/03/2017	0	0	0
2015414726	155	rural/clinic	No	14/03/2017	0	0	0
2015418129	155	rural/clinic	No	14/03/2017	0	0	0
2015414940	155	rural/clinic	No	14/03/2017	0	0	0
2015413340	155	rural/clinic	No	14/03/2017	0	0	0
2015414650	155	rural/clinic	No	14/03/2017	0	0	0
2015413341	155	rural/clinic	No	14/03/2017	0	0	0
2011134968	155	rural/clinic	No	14/03/2017	0	0	0
2015322588	155	rural/clinic	No	15/03/2017	0	0	0
2015405614	155	rural/clinic	No	14/03/2017	0	0	0
2011134971	155	rural/clinic	No	14/03/2017	0	0	0
2015322581	155	rural/clinic	No	15/03/2017	0	0	0
2013264387	155	rural/clinic	No	14/03/2017	0	0	0
2015320867	155	rural/clinic	No	14/03/2017	0	0	0
2015405615	155	rural/clinic	No	14/03/2017	0	0	0
2014362871	155	rural/clinic	No	14/03/2017	0	0	0
2013264386	155	rural/clinic	No	14/03/2017	0	0	0
2013261928	155	rural/clinic	No	14/03/2017	0	0	0
2015413339	155	rural/clinic	No	14/03/2017	0	0	0
2012314569	155	rural/clinic	No	14/03/2017	0	0	0
2015322584	155	rural/clinic	No	15/03/2017	0	0	0
2012314568	155	rural/clinic	No	14/03/2017	0	0	0
2015363707	155	rural/clinic	No	14/03/2017	0	0	0
2015322727	155	rural/clinic	No	15/03/2017	0	0	0
2014337220	155	rural/clinic	No	14/03/2017	0	0	0
2015322582	155	rural/clinic	No	15/03/2017	0	0	0
2015303113	155	rural/clinic	No	14/03/2017	0	0	0
2015322730	155	rural/clinic	No	15/03/2017	0	0	0
2015363706	155	rural/clinic	No	14/03/2017	0	0	0
2015405661	155	rural/clinic	No	14/03/2017	0	0	0
2014365154	155	rural/clinic	No	14/03/2017	0	0	0
2014365155	155	rural/clinic	No	14/03/2017	0	0	0
2014365157	155	rural/clinic	No	14/03/2017	0	0	0
2014365153	155	rural/clinic	No	14/03/2017	0	0	0
2014336898	155	rural/clinic	No	14/03/2017	0	0	0
2014309380	155	rural/clinic	No	14/03/2017	0	0	0
2015388508	155	rural/clinic	No	14/03/2017	0	0	0
2015357379	155	rural/clinic	No	14/03/2017	0	0	0
2015358155	574	rural/clinic	No	16/03/2017	0	0	0
2012350319	574	rural/clinic	No	15/03/2017	0	0	0
2012369803	574	rural/clinic	No	16/03/2017	0	0	0
2015331635	574	rural/clinic	No	16/03/2017	0	0	0
2015328460	574	rural/clinic	No	15/03/2017	0	0	0
2014306087	574	rural/clinic	No	16/03/2017	0	0	0
2015339006	574	rural/clinic	No	16/03/2017	0	0	0
2018339005	574	rural/clinic	No	16/03/2017	0	0	0
2014306090	574	rural/clinic	No	16/03/2017	0	0	0
2015328461	574	rural/clinic	No	15/03/2017	0	0	0
2015339004	574	rural/clinic	No	16/03/2017	0	0	0
2015351960	574	rural/clinic	No	16/03/2017	0	0	0
2015339007	574	rural/clinic	No	16/03/2017	0	0	0
2015351962	574	rural/clinic	No	16/03/2017	0	0	0
2013247705	574	rural/clinic	No	16/03/2017	0	0	0
2015351963	574	rural/clinic	No	16/03/2017	0	0	0
2013247706	574	rural/clinic	No	16/03/2017	0	0	0
2012341040	574	rural/clinic	No	16/03/2017	0	0	0
2014301919	574	rural/clinic	No	16/03/2017	0	0	0
2014301920	574	rural/clinic	No	16/03/2017	0	0	0
2014347985	574	rural/clinic	No	16/03/2017	0	0	0
2012341041	574	rural/clinic	No	16/03/2017	0	0	0
2013253027	574	rural/clinic	No	16/03/2017	0	0	0
2014297344	574	rural/clinic	No	16/03/2017	0	0	0
2015369663	574	rural/clinic	No	16/03/2017	0	0	0
2015369667	574	rural/clinic	No	16/03/2017	0	0	0
2015369668	574	rural/clinic	No	16/03/2017	0	0	0
2015369669	574	rural/clinic	No	16/03/2017	0	0	0
2015369670	574	rural/clinic	No	16/03/2017	0	0	0
2015369674	574	rural/clinic	No	16/03/2017	0	0	0
2015369675	574	rural/clinic	No	16/03/2017	0	0	0
2015326557	574	rural/clinic	No	16/03/2017	0	0	0
2015368267	574	rural/clinic	No	16/03/2017	0	0	0
2015373667	574	rural/clinic	No	16/03/2017	0	0	0
2015373668	574	rural/clinic	No	16/03/2017	0	0	0
2015373669	574	rural/clinic	No	16/03/2017	0	0	0
2015373670	574	rural/clinic	No	16/03/2017	0	0	0
2015373671	574	rural/clinic	No	16/03/2017	0	0	0
2015373672	574	rural/clinic	No	16/03/2017	0	0	0

2015373673	574	rural/clinic	No	16/03/2017	0	0	0
2015373674	574	rural/clinic	No	16/03/2017	0	0	0
2015373675	574	rural/clinic	No	16/03/2017	0	0	0
2015373676	574	rural/clinic	No	16/03/2017	0	0	0
2015373677	574	rural/clinic	No	16/03/2017	0	0	0
2015373678	574	rural/clinic	No	16/03/2017	0	0	0
2015373679	574	rural/clinic	No	16/03/2017	0	0	0
2015373680	574	rural/clinic	No	16/03/2017	0	0	0
2015373681	574	rural/clinic	No	16/03/2017	0	0	0
2013247704	574	rural/clinic	No	16/03/2017	0	0	0
2013262216	574	rural/clinic	No	16/03/2017	0	0	0
2013262219	574	rural/clinic	No	16/03/2017	0	0	0
2015339301	574	rural/clinic	No	16/03/2017	0	0	0
2011144999	574	rural/clinic	No	16/03/2017	0	0	0
2011145000	574	rural/clinic	No	16/03/2017	0	0	0
2013247703	574	rural/clinic	No	16/03/2017	0	0	0
2015331633	574	rural/clinic	No	16/03/2017	0	0	0
2015331634	574	rural/clinic	No	16/03/2017	0	0	0
2015331631	574	rural/clinic	No	16/03/2017	0	0	0
2015331632	574	rural/clinic	No	16/03/2017	0	0	0
2015331715	574	rural/clinic	No	16/03/2017	0	0	0
2015331716	574	rural/clinic	No	16/03/2017	0	0	0
2015331714	574	rural/clinic	No	16/03/2017	0	0	0
2015373682	574	rural/clinic	No	16/03/2017	0	0	0
2014296316	574	rural/clinic	No	16/03/2017	0	0	0
2014296317	574	rural/clinic	No	16/03/2017	0	0	0
2015373683	574	rural/clinic	No	16/03/2017	0	0	0
2014298037	574	rural/clinic	No	16/03/2017	0	0	0
2015402520	574	rural/clinic	No	16/03/2017	0	0	0
2014298035	574	rural/clinic	No	16/03/2017	0	0	0
2014298036	574	rural/clinic	No	16/03/2017	0	0	0
2015331662	574	rural/clinic	No	16/03/2017	0	0	0
2015331663	574	rural/clinic	No	16/03/2017	0	0	0
2013252787	574	rural/clinic	No	16/03/2017	0	0	0
2011144013	574	rural/clinic	No	16/03/2017	0	0	0
2015402521	574	rural/clinic	No	16/03/2017	0	0	0
2011144014	574	rural/clinic	No	16/03/2017	0	0	0
2015402522	574	rural/clinic	No	16/03/2017	0	0	0
2011144015	574	rural/clinic	No	16/03/2017	0	0	0
2015331628	574	rural/clinic	No	16/03/2017	0	0	0
2015331630	574	rural/clinic	No	16/03/2017	0	0	0
2015402523	574	rural/clinic	No	16/03/2017	0	0	0
2015331626	574	rural/clinic	No	16/03/2017	0	0	0
2015331625	574	rural/clinic	No	16/03/2017	0	0	0
2015402524	574	rural/clinic	No	16/03/2017	0	0	0
2015331629	574	rural/clinic	No	16/03/2017	0	0	0
2015402525	574	rural/clinic	No	16/03/2017	0	0	0
2015331636	574	rural/clinic	No	16/03/2017	0	0	0
2011197676	574	rural/clinic	No	16/03/2017	0	0	0
2015286285	574	rural/clinic	No	16/03/2017	0	0	0
2011133605	574	rural/clinic	No	16/03/2017	0	0	0
2014296319	574	rural/clinic	No	16/03/2017	0	0	0
2015305063	574	rural/clinic	No	16/03/2017	0	0	0
2014312269	574	rural/clinic	No	16/03/2017	0	0	0
2015305064	574	rural/clinic	No	16/03/2017	0	0	0
2015358157	574	rural/clinic	No	16/03/2017	0	0	0
2011133607	574	rural/clinic	No	16/03/2017	0	0	0
2015358156	574	rural/clinic	No	16/03/2017	0	0	0
2011133608	574	rural/clinic	No	16/03/2017	0	0	0
2015358154	574	rural/clinic	No	16/03/2017	0	0	0
2011133606	574	rural/clinic	No	16/03/2017	0	0	0
2011133609	574	rural/clinic	No	16/03/2017	0	0	0
2015358153	574	rural/clinic	No	16/03/2017	0	0	0
2015358152	574	rural/clinic	No	16/03/2017	0	0	0
2014317622	574	rural/clinic	No	16/03/2017	0	0	0
2015297080	574	rural/clinic	No	16/03/2017	0	0	0
2014306088	574	rural/clinic	No	16/03/2017	0	0	0
2015303606	574	rural/clinic	No	16/03/2017	0	0	0
2014321518	574	rural/clinic	No	16/03/2017	0	0	0
2015303605	574	rural/clinic	No	16/03/2017	0	0	0
2014321517	574	rural/clinic	No	16/03/2017	0	0	0
2015303604	574	rural/clinic	No	16/03/2017	0	0	0
2014321516	574	rural/clinic	No	16/03/2017	0	0	0
2015303603	574	rural/clinic	No	16/03/2017	0	0	0
2014328754	574	rural/clinic	No	16/03/2017	0	0	0
2014328755	574	rural/clinic	No	16/03/2017	0	0	0
2015287309	574	rural/clinic	No	16/03/2017	0	0	0
2015303601	574	rural/clinic	No	16/03/2017	0	0	0
2014346944	574	rural/clinic	No	16/03/2017	0	0	0
2015335199	574	rural/clinic	No	16/03/2017	0	0	0
2014296320	574	rural/clinic	No	16/03/2017	0	0	0
2015335200	574	rural/clinic	No	16/03/2017	0	0	0

201198727	574	rural/clinic	No	16/03/2017	0	0	0
2015335198	574	rural/clinic	No	16/03/2017	0	0	0
2015331627	574	rural/clinic	No	16/03/2017	0	0	0
2015382889	574	rural/clinic	No	16/03/2017	0	0	0
2015344355	574	rural/clinic	No	16/03/2017	0	0	0
2015303599	574	rural/clinic	No	16/03/2017	0	0	0
2015382888	574	rural/clinic	No	16/03/2017	0	0	0
2015303598	574	rural/clinic	No	16/03/2017	0	0	0
2015303597	574	rural/clinic	No	16/03/2017	0	0	0
2015382886	574	rural/clinic	No	16/03/2017	0	0	0
2015303596	574	rural/clinic	No	16/03/2017	0	0	0
2015303594	574	rural/clinic	No	16/03/2017	0	0	0
2015303593	574	rural/clinic	No	16/03/2017	0	0	0
2015344357	10	rural/clinic	No	16/03/2017	0	0	0
2015344075	10	rural/clinic	No	16/03/2017	0	0	0
2015297081	10	rural/clinic	No	16/03/2017	0	0	0
2011157093	10	rural/clinic	No	16/03/2017	0	0	0
2015412772	10	rural/clinic	No	16/03/2017	0	0	0
2011157092	10	rural/clinic	No	16/03/2017	0	0	0
2012259143	10	rural/clinic	No	16/03/2017	0	0	0
2015412770	10	rural/clinic	No	16/03/2017	0	0	0
2012259142	10	rural/clinic	No	16/03/2017	0	0	0
2015412771	10	rural/clinic	No	16/03/2017	0	0	0
2012259144	10	rural/clinic	No	16/03/2017	0	0	0
2015344356	10	rural/clinic	No	16/03/2017	0	0	0
2014326081	10	rural/clinic	No	16/03/2017	0	0	0
2015344351	10	rural/clinic	No	16/03/2017	0	0	0
2014326080	10	rural/clinic	No	16/03/2017	0	0	0
2014303496	10	rural/clinic	No	16/03/2017	0	0	0
2014303497	10	rural/clinic	No	16/03/2017	0	0	0
2014288168	10	rural/clinic	No	16/03/2017	0	0	0
2014303498	10	rural/clinic	No	16/03/2017	0	0	0
2015339525	10	rural/clinic	No	16/03/2017	0	0	0
2015344352	10	rural/clinic	No	16/03/2017	0	0	0
2015344353	10	rural/clinic	No	16/03/2017	0	0	0
2015290023	10	rural/clinic	No	16/03/2017	0	0	0
2015344354	10	rural/clinic	No	16/03/2017	0	0	0
2015404951	10	rural/clinic	No	16/03/2017	0	0	0
2015375103	10	rural/clinic	No	16/03/2017	0	0	0
2015290100	10	rural/clinic	No	16/03/2017	0	0	0
2015310652	10	rural/clinic	No	16/03/2017	0	0	0
2015386370	10	rural/clinic	No	16/03/2017	0	0	0
2014309381	10	rural/clinic	No	16/03/2017	0	0	0
2015310651	10	rural/clinic	No	16/03/2017	0	0	0
2015286278	10	rural/clinic	No	16/03/2017	0	0	0
2014298580	10	rural/clinic	No	16/03/2017	0	0	0
2015290099	10	rural/clinic	No	16/03/2017	0	0	0
2011143197	10	rural/clinic	No	16/03/2017	0	0	0
2011143196	10	rural/clinic	No	16/03/2017	0	0	0
2011181148	10	rural/clinic	No	16/03/2017	0	0	0
2015310653	10	rural/clinic	No	16/03/2017	0	0	0
2014356966	10	rural/clinic	No	16/03/2017	0	0	0
2015355906	10	rural/clinic	No	16/03/2017	0	0	0
2015290098	10	rural/clinic	No	16/03/2017	0	0	0
2015408666	10	rural/clinic	No	16/03/2017	0	0	0
2015358511	10	rural/clinic	No	16/03/2017	0	0	0
2015358510	10	rural/clinic	No	16/03/2017	0	0	0
2015408667	10	rural/clinic	No	16/03/2017	0	0	0
2015358509	10	rural/clinic	No	16/03/2017	0	0	0
2015344358	10	rural/clinic	No	16/03/2017	0	0	0
2014306988	10	rural/clinic	No	16/03/2017	0	0	0
2015303607	10	rural/clinic	No	16/03/2017	0	0	0
2015358508	10	rural/clinic	No	16/03/2017	0	0	0
2015288504	10	rural/clinic	No	16/03/2017	0	0	0
2013258727	10	rural/clinic	No	16/03/2017	0	0	0
2015358507	10	rural/clinic	No	16/03/2017	0	0	0
2014372439	10	rural/clinic	No	16/03/2017	0	0	0
2015358504	10	rural/clinic	No	16/03/2017	0	0	0
2014319721	10	rural/clinic	No	16/03/2017	0	0	0
2015408168	10	rural/clinic	No	16/03/2017	0	0	0
2015358506	10	rural/clinic	No	15/03/2017	0	0	0
2015332013	10	rural/clinic	No	16/03/2017	0	0	0
2015358505	10	rural/clinic	No	16/03/2017	0	0	0
2015324231	10	rural/clinic	No	16/03/2017	0	0	0
2014335524	10	rural/clinic	No	16/03/2017	0	0	0
2015358500	10	rural/clinic	No	16/03/2017	0	0	0
2014346310	10	rural/clinic	No	16/03/2017	0	0	0
2014319720	10	rural/clinic	No	16/03/2017	0	0	0
2014346308	10	rural/clinic	No	16/03/2017	0	0	0
2015358503	10	rural/clinic	No	16/03/2017	0	0	0
2015414374	10	rural/clinic	No	16/03/2017	0	0	0
2014346307	10	rural/clinic	No	16/03/2017	0	0	0

2015358502	10	rural/clinic	No	16/03/2017	0	0	0
2012291840	10	rural/clinic	No	16/03/2017	0	0	0
2012290693	10	rural/clinic	No	16/03/2017	0	0	0
2015351955	10	rural/clinic	No	16/03/2017	0	0	0
2015358501	10	rural/clinic	No	16/03/2017	0	0	0
2015319435	10	rural/clinic	No	16/03/2017	0	0	0
2015351956	10	rural/clinic	No	16/03/2017	0	0	0
2015351957	10	rural/clinic	No	16/03/2017	0	0	0
2012290692	10	rural/clinic	No	16/03/2017	0	0	0
2015319434	10	rural/clinic	No	16/03/2017	0	0	0
2015351958	10	rural/clinic	No	16/03/2017	0	0	0
2012290694	10	rural/clinic	No	16/03/2017	0	0	0
2015351959	10	rural/clinic	No	16/03/2017	0	0	0
2011232688	10	rural/clinic	No	16/03/2017	0	0	0
2012290691	10	rural/clinic	No	16/03/2017	0	0	0
2014307746	10	rural/clinic	No	16/03/2017	0	0	0
2011196009	10	rural/clinic	No	16/03/2017	0	0	0
2012290695	10	rural/clinic	No	16/03/2017	0	0	0
2011232687	10	rural/clinic	No	16/03/2017	0	0	0
2012290689	10	rural/clinic	No	16/03/2017	0	0	0
2014346894	10	rural/clinic	No	16/03/2017	0	0	0
2015334611	10	rural/clinic	No	16/03/2017	0	0	0
2014347010	10	rural/clinic	No	16/03/2017	0	0	0
2012290690	10	rural/clinic	No	16/03/2017	0	0	0
2014347009	10	rural/clinic	No	16/03/2017	0	0	0
2015334610	10	rural/clinic	No	16/03/2017	0	0	0
2012265917	10	rural/clinic	No	16/03/2017	0	0	0
2014347011	10	rural/clinic	No	16/03/2017	0	0	0
2014327666	10	rural/clinic	No	16/03/2017	0	0	0
2012370161	10	rural/clinic	No	16/03/2017	0	0	0
2014356967	10	rural/clinic	No	16/03/2017	0	0	0
2014288131	10	rural/clinic	No	16/03/2017	0	0	0
2015293832	10	rural/clinic	No	16/03/2017	0	0	0
2014288167	10	rural/clinic	No	16/03/2017	0	0	0
2012323656	10	rural/clinic	No	16/03/2017	0	0	0
2015334903	10	rural/clinic	No	16/03/2017	0	0	0
2015334904	10	rural/clinic	No	16/03/2017	0	0	0
2011225365	10	rural/clinic	No	16/03/2017	0	0	0
2014350044	10	rural/clinic	No	16/03/2017	0	0	0
2015334905	10	rural/clinic	No	16/03/2017	0	0	0
2015334906	10	rural/clinic	No	16/03/2017	0	0	0
2015349526	10	rural/clinic	No	16/03/2017	0	0	0
2015334907	10	rural/clinic	No	16/03/2017	0	0	0
2015349527	10	rural/clinic	No	16/03/2017	0	0	0
2015339248	10	rural/clinic	No	16/03/2017	0	0	0
2015339249	10	rural/clinic	No	16/03/2017	0	0	0
2015339250	10	rural/clinic	No	16/03/2017	0	0	0
2015334908/D	10	rural/clinic	No	16/03/2017	0	0	0
2015339246	10	rural/clinic	No	16/03/2017	0	0	0
2015339247	10	rural/clinic	No	16/03/2017	0	0	0
2015384357	10	rural/clinic	No	16/03/2017	0	0	0
2012359910	10	rural/clinic	No	16/03/2017	0	0	0
2015384356	10	rural/clinic	No	16/03/2017	0	0	0
2015382887	10	rural/clinic	No	16/03/2017	0	0	0
2012359912	10	rural/clinic	No	16/03/2017	0	0	0
2014327036	10	rural/clinic	No	16/03/2017	0	0	0
2012359909	381	rural/clinic	No	16/03/2017	0	0	0
2014327035	381	rural/clinic	No	16/03/2017	0	0	0
2015385516	381	rural/clinic	No	16/03/2017	0	0	0
2015301358	381	rural/clinic	No	16/03/2017	0	0	0
2012359908	381	rural/clinic	No	16/03/2017	0	0	0
2015301454	381	rural/clinic	No	16/03/2017	0	0	0
2014378477	381	rural/clinic	No	16/03/2017	0	0	0
2011199075	381	rural/clinic	No	16/03/2017	0	0	0
2012359907	381	rural/clinic	No	16/03/2017	0	0	0
2012265635	381	rural/clinic	No	16/03/2017	0	0	0
2012389494	381	rural/clinic	No	16/03/2017	0	0	0
2014346946	381	rural/clinic	No	16/03/2017	0	0	0
2015344863	381	rural/clinic	No	16/03/2017	0	0	0
2013267891	381	rural/clinic	No	16/03/2017	0	0	0
2015344074	381	rural/clinic	No	16/03/2017	0	0	0
2015415253	381	rural/clinic	No	16/03/2017	0	0	0
2014346945	381	rural/clinic	No	16/03/2017	0	0	0
2013267890	381	rural/clinic	No	16/03/2017	0	0	0
2015384030	381	rural/clinic	No	16/03/2017	0	0	0
2015386368	381	rural/clinic	No	16/03/2017	0	0	0
2014290237	381	rural/clinic	No	16/03/2017	0	0	0
2015386366	381	rural/clinic	No	16/03/2017	0	0	0
2015386372	381	rural/clinic	No	16/03/2017	0	0	0
2015386371	381	rural/clinic	No	16/03/2017	0	0	0
2011236079	381	rural/clinic	No	16/03/2017	0	0	0
2015386369	381	rural/clinic	No	16/03/2017	0	0	0

2015386364	381	rural/clinic	No	16/03/2017	0	0	0
2015386365	381	rural/clinic	No	16/03/2017	0	0	0
2015386362	381	rural/clinic	No	16/03/2017	0	0	0
2015386353	381	rural/clinic	No	16/03/2017	0	0	0
2015386363	381	rural/clinic	No	16/03/2017	0	0	0
2014335230	381	rural/clinic	No	16/03/2017	0	0	0
2014335229	381	rural/clinic	No	16/03/2017	0	0	0
2014335228	381	rural/clinic	No	16/03/2017	0	0	0
2012365982	381	rural/clinic	No	16/03/2017	0	0	0
2014344290	381	rural/clinic	No	16/03/2017	0	0	0
2012365983	381	rural/clinic	No	16/03/2017	0	0	0
2015384031	381	rural/clinic	No	16/03/2017	0	0	0
2015303608	381	rural/clinic	No	16/03/2017	0	0	0
2015357635	381	rural/clinic	No	16/03/2017	0	0	0
2012306448	381	rural/clinic	No	16/03/2017	0	0	0
2015357636	381	rural/clinic	No	16/03/2017	0	0	0
2015357637	381	rural/clinic	No	16/03/2017	0	0	0
2012359154	381	rural/clinic	No	16/03/2017	0	0	0
2012359155	381	rural/clinic	No	16/03/2017	0	0	0
2015339534	381	rural/clinic	No	16/03/2017	0	0	0
2015339533	381	rural/clinic	No	16/03/2017	0	0	0
2015339530	381	rural/clinic	No	16/03/2017	0	0	0
2015339531	381	rural/clinic	No	16/03/2017	0	0	0
2015339532	381	rural/clinic	No	16/03/2017	0	0	0
2015339529	381	rural/clinic	No	16/03/2017	0	0	0
2015339527	381	rural/clinic	No	16/03/2017	0	0	0
2015339528	381	rural/clinic	No	16/03/2017	0	0	0
2015339526	381	rural/clinic	No	16/03/2017	0	0	0
2015376112	381	rural/clinic	No	14/03/2017	0	0	0
2015376112	381	rural/clinic	No	14/03/2017	0	0	0
2015397519	381	rural/clinic	No	13/03/2017	0	0	0
2015397519	381	rural/clinic	No	13/03/2017	0	0	0
2011113948	381	rural/clinic	No	14/03/2017	0	0	0
2011113948	381	rural/clinic	No	14/03/2017	0	0	0
2015397517	381	rural/clinic	No	13/03/2017	0	0	0
2015397517	381	rural/clinic	No	13/03/2017	0	0	0
2015340491	381	rural/clinic	No	03/03/2017	0	0	0
2015340491	381	rural/clinic	No	03/03/2017	0	0	0
2015314349	381	rural/clinic	No	15/03/2017	0	0	0
2015314349	381	rural/clinic	No	15/03/2017	0	0	0
2012259866	381	rural/clinic	No	14/03/2017	0	0	0
2012259866	381	rural/clinic	No	14/03/2017	0	0	0
2015293362	381	rural/clinic	No	14/03/2017	0	0	0
2015293362	381	rural/clinic	No	14/03/2017	0	0	0
2014288397	381	rural/clinic	No	14/03/2017	0	0	0
2014288397	381	rural/clinic	No	14/03/2017	0	0	0
2015334408	381	rural/clinic	No	14/03/2017	0	0	0
2015334408	381	rural/clinic	No	14/03/2017	0	0	0
2011134968	381	rural/clinic	No	14/03/2017	0	0	0
2011134968	381	rural/clinic	No	14/03/2017	0	0	0
2015357382	381	rural/clinic	No	14/03/2017	0	0	0
2015357382	381	rural/clinic	No	14/03/2017	0	0	0
2015363706	381	rural/clinic	No	14/03/2017	0	0	0
2015363706	381	rural/clinic	No	14/03/2017	0	0	0
2015357381	381	rural/clinic	No	14/03/2017	0	0	0
2015357381	381	rural/clinic	No	14/03/2017	0	0	0
2015338453	381	rural/clinic	No	14/03/2017	0	0	0
2015338453	381	rural/clinic	No	14/03/2017	0	0	0
2015337046	381	rural/clinic	No	14/03/2017	0	0	0
2015337046	381	rural/clinic	No	14/03/2017	0	0	0
2015334904	381	rural/clinic	No	16/03/2017	0	0	0
2014378477	381	rural/clinic	No	16/03/2017	0	0	0
2014378477	381	rural/clinic	No	16/03/2017	0	0	0
2014298037	381	rural/clinic	No	16/03/2017	0	0	0
2014298037	381	rural/clinic	No	16/03/2017	0	0	0
2015358507	381	rural/clinic	No	16/03/2017	0	0	0
2015358507	381	rural/clinic	No	16/03/2017	0	0	0
2015351958	381	rural/clinic	No	16/03/2017	0	0	0
2015351958	381	rural/clinic	No	16/03/2017	0	0	0
2012259144	381	rural/clinic	No	16/03/2017	0	0	0
2012259144	381	rural/clinic	No	16/03/2017	0	0	0
2015315983	381	rural/clinic	No	07/03/2017	0	0	0
2012246190	381	rural/clinic	No	21/03/2017	0	0	0
2012244648	381	rural/clinic	No	21/03/2017	0	0	0
2012325685	381	rural/clinic	No	21/03/2017	0	0	0
2012244649	381	rural/clinic	No	21/03/2017	0	0	0
2012244647	381	rural/clinic	No	21/03/2017	0	0	0
2012244650	381	rural/clinic	No	21/03/2017	0	0	0
2012325686	381	rural/clinic	No	21/03/2017	0	0	0
2012244282	381	rural/clinic	No	21/03/2017	0	0	0
2011204786	381	rural/clinic	No	21/03/2017	0	0	0
2014357807	381	rural/clinic	No	21/03/2017	0	0	0

2012363296	381	rural/clinic	No	21/03/2017	0	0	0
2014357308	381	rural/clinic	No	21/03/2017	0	0	0
2011204782	381	rural/clinic	No	21/03/2017	0	0	0
2011204783	381	rural/clinic	No	21/03/2017	0	0	0
2014357307	381	rural/clinic	No	21/03/2017	0	0	0
2011204784	381	rural/clinic	No	21/03/2017	0	0	0
2014357305	381	rural/clinic	No	21/03/2017	0	0	0
2012246187	381	rural/clinic	No	21/03/2017	0	0	0
2012246188	381	rural/clinic	No	21/03/2017	0	0	0
2015331911	381	rural/clinic	No	21/03/2017	0	0	0
2015331913	381	rural/clinic	No	21/03/2017	0	0	0
2015331912	381	rural/clinic	No	21/03/2017	0	0	0
2015331915	381	rural/clinic	No	21/03/2017	0	0	0
2012358280	381	rural/clinic	No	21/03/2017	0	0	0
2015385941	381	rural/clinic	No	21/03/2017	0	0	0
2015385946	381	rural/clinic	No	21/03/2017	0	0	0
2011212960	381	rural/clinic	No	21/03/2017	0	0	0
2015385947	381	rural/clinic	No	21/03/2017	0	0	0
2014314457	381	rural/clinic	No	21/03/2017	0	0	0
2011204785	381	rural/clinic	No	21/03/2017	0	0	0
2014291595	381	rural/clinic	No	21/03/2017	0	0	0
2014303073	381	rural/clinic	No	21/03/2017	0	0	0
2014303074	381	rural/clinic	No	21/03/2017	0	0	0
2014303075	381	rural/clinic	No	21/03/2017	0	0	0
2015335577	381	rural/clinic	No	21/03/2017	0	0	0
2014303076	381	rural/clinic	No	21/03/2017	0	0	0
2014291596	381	rural/clinic	No	21/03/2017	0	0	0
2015335580	381	rural/clinic	No	21/03/2017	0	0	0
2015382891	381	rural/clinic	No	21/03/2017	0	0	0
2015335430	381	rural/clinic	No	21/03/2017	0	0	0
2014291594	381	rural/clinic	No	21/03/2017	0	0	0
2015382895	381	rural/clinic	No	21/03/2017	0	0	0
2015289565	381	rural/clinic	No	21/03/2017	0	0	0
2015382894	381	rural/clinic	No	21/03/2017	0	0	0
2014355900	381	rural/clinic	No	21/03/2017	0	0	0
2015382893	381	rural/clinic	No	21/03/2017	0	0	0
2015335429	381	rural/clinic	No	21/03/2017	0	0	0
2015315994	381	rural/clinic	No	21/03/2017	0	0	0
2015382892	381	rural/clinic	No	21/03/2017	0	0	0
2015362458	381	rural/clinic	No	21/03/2017	0	0	0
2015413979	381	rural/clinic	No	21/03/2017	0	0	0
2015315993	381	rural/clinic	No	21/03/2017	0	0	0
2015413982	381	rural/clinic	No	21/03/2017	0	0	0
2015335582	381	rural/clinic	No	21/03/2017	0	0	0
2015315992	381	rural/clinic	No	21/03/2017	0	0	0
2015289323	381	rural/clinic	No	21/03/2017	0	0	0
2015413983	381	rural/clinic	No	21/03/2017	0	0	0
2015315991	381	rural/clinic	No	21/03/2017	0	0	0
2015335428	381	rural/clinic	No	21/03/2017	0	0	0
2015413984	381	rural/clinic	No	21/03/2017	0	0	0
2015315990	381	rural/clinic	No	21/03/2017	0	0	0
2015413986	381	rural/clinic	No	21/03/2017	0	0	0
2015289325	381	rural/clinic	No	21/03/2017	0	0	0
2015351666	381	rural/clinic	No	21/03/2017	0	0	0
2015385943	382	rural/clinic	No	21/03/2017	0	0	0
2015351665	382	rural/clinic	No	21/03/2017	0	0	0
2015289324	382	rural/clinic	No	21/03/2017	0	0	0
2015351664	382	rural/clinic	No	21/03/2017	0	0	0
2015385944	382	rural/clinic	No	21/03/2017	0	0	0
2015362456	382	rural/clinic	No	21/03/2017	0	0	0
2015351663	382	rural/clinic	No	21/03/2017	0	0	0
2015362460	382	rural/clinic	No	21/03/2017	0	0	0
2015385945	382	rural/clinic	No	21/03/2017	0	0	0
2015351662	382	rural/clinic	No	21/03/2017	0	0	0
2015355774	382	rural/clinic	No	21/03/2017	0	0	0
2012388380	382	rural/clinic	No	21/03/2017	0	0	0
2015413985	382	rural/clinic	No	21/03/2017	0	0	0
2015335431	382	rural/clinic	No	21/03/2017	0	0	0
2015337360	382	rural/clinic	No	21/03/2017	0	0	0
2012336608	382	rural/clinic	No	21/03/2017	0	0	0
2015337361	382	rural/clinic	No	21/03/2017	0	0	0
2015335578	382	rural/clinic	No	21/03/2017	0	0	0
2012310740	382	rural/clinic	No	21/03/2017	0	0	0
2015340954	382	rural/clinic	No	21/03/2017	0	0	0
2014355846	382	rural/clinic	No	21/03/2017	0	0	0
2015340956	382	rural/clinic	No	21/03/2017	0	0	0
2014355847	382	rural/clinic	No	21/03/2017	0	0	0
2015340958	382	rural/clinic	No	21/03/2017	0	0	0
2015340955	382	rural/clinic	No	21/03/2017	0	0	0
2014314722	382	rural/clinic	No	21/03/2017	0	0	0
2015340957	382	rural/clinic	No	21/03/2017	0	0	0
2015340953	382	rural/clinic	No	21/03/2017	0	0	0

2015351608	382	rural/clinic	No	21/03/2017	0	0	0
2015355651	382	rural/clinic	No	21/03/2017	0	0	0
2015340951	382	rural/clinic	No	21/03/2017	0	0	0
2015340952	382	rural/clinic	No	21/03/2017	0	0	0
2015351607	382	rural/clinic	No	21/03/2017	0	0	0
2015413548	382	rural/clinic	No	21/03/2017	0	0	0
2014311650	382	rural/clinic	No	21/03/2017	0	0	0
2015364360	382	rural/clinic	No	21/03/2017	0	0	0
2012374277	382	rural/clinic	No	21/03/2017	0	0	0
2015364359	382	rural/clinic	No	21/03/2017	0	0	0
2012374278	382	rural/clinic	No	21/03/2017	0	0	0
2015351606	382	rural/clinic	No	21/03/2017	0	0	0
2014288420	382	rural/clinic	No	21/03/2017	0	0	0
2012374276	382	rural/clinic	No	21/03/2017	0	0	0
2015364358	382	rural/clinic	No	21/03/2017	0	0	0
2015364357	382	rural/clinic	No	21/03/2017	0	0	0
2015351605	382	rural/clinic	No	21/03/2017	0	0	0
2015324468	382	rural/clinic	No	21/03/2017	0	0	0
2015364356	382	rural/clinic	No	21/03/2017	0	0	0
2015364355	382	rural/clinic	No	21/03/2017	0	0	0
2015303063	382	rural/clinic	No	21/03/2017	0	0	0
2015364354	382	rural/clinic	No	21/03/2017	0	0	0
2015351604	382	rural/clinic	No	21/03/2017	0	0	0
2015351603	382	rural/clinic	No	21/03/2017	0	0	0
2015303062	382	rural/clinic	No	21/03/2017	0	0	0
2015364353	382	rural/clinic	No	21/03/2017	0	0	0
2014314206	382	rural/clinic	No	21/03/2017	0	0	0
2015303060	382	rural/clinic	No	21/03/2017	0	0	0
2014314207	382	rural/clinic	No	21/03/2017	0	0	0
2015287352	382	rural/clinic	No	21/03/2017	0	0	0
2015303061	382	rural/clinic	No	21/03/2017	0	0	0
2015287353	382	rural/clinic	No	21/03/2017	0	0	0
2015303064	382	rural/clinic	No	21/03/2017	0	0	0
2014311446	382	rural/clinic	No	21/03/2017	0	0	0
2015413344	382	rural/clinic	No	21/03/2017	0	0	0
2015413342	382	rural/clinic	No	21/03/2017	0	0	0
2014311447	382	rural/clinic	No	21/03/2017	0	0	0
2015405666	382	rural/clinic	No	21/03/2017	0	0	0
2014311442	382	rural/clinic	No	21/03/2017	0	0	0
2015405665	382	rural/clinic	No	21/03/2017	0	0	0
2014311443	382	rural/clinic	No	21/03/2017	0	0	0
2015405663	382	rural/clinic	No	21/03/2017	0	0	0
2015405664	382	rural/clinic	No	21/03/2017	0	0	0
2014311444	382	rural/clinic	No	21/03/2017	0	0	0
2015351602	382	rural/clinic	No	21/03/2017	0	0	0
2015405662	382	rural/clinic	No	21/03/2017	0	0	0
2014311445	382	rural/clinic	No	21/03/2017	0	0	0
2015351601	382	rural/clinic	No	21/03/2017	0	0	0
2014311448	382	rural/clinic	No	21/03/2017	0	0	0
2015373183	382	rural/clinic	No	21/03/2017	0	0	0
2015287351	382	rural/clinic	No	21/03/2017	0	0	0
2015373181	382	rural/clinic	No	21/03/2017	0	0	0
2015373178	382	rural/clinic	No	21/03/2017	0	0	0
2015368566	382	rural/clinic	No	21/03/2017	0	0	0
2015373186	382	rural/clinic	No	21/03/2017	0	0	0
2015373175	382	rural/clinic	No	21/03/2017	0	0	0
2015373172	382	rural/clinic	No	21/03/2017	0	0	0
2015373173	382	rural/clinic	No	21/03/2017	0	0	0
2015373174	382	rural/clinic	No	21/03/2017	0	0	0
2013256263	382	rural/clinic	No	21/03/2017	0	0	0
2013256262	382	rural/clinic	No	21/03/2017	0	0	0
2015373188	382	rural/clinic	No	21/03/2017	0	0	0
2013256261	382	rural/clinic	No	21/03/2017	0	0	0
2015377631	382	rural/clinic	No	21/03/2017	0	0	0
2015377632	382	rural/clinic	No	21/03/2017	0	0	0
2015377633	382	rural/clinic	No	21/03/2017	0	0	0
2015373182	382	rural/clinic	No	21/03/2017	0	0	0
2015373189	382	rural/clinic	No	21/03/2017	0	0	0
2015377634	382	rural/clinic	No	21/03/2017	0	0	0
2015373191	382	rural/clinic	No	21/03/2017	0	0	0
2015377635	382	rural/clinic	No	21/03/2017	0	0	0
2012377877	382	rural/clinic	No	21/03/2017	0	0	0
2014375669	382	rural/clinic	No	21/03/2017	0	0	0
2014315525	382	rural/clinic	No	21/03/2017	0	0	0
2014315524	382	rural/clinic	No	21/03/2017	0	0	0
2014375670	382	rural/clinic	No	21/03/2017	0	0	0
2014305216	382	rural/clinic	No	21/03/2017	0	0	0
2015368807	382	rural/clinic	No	21/03/2017	0	0	0
2015346225	382	rural/clinic	No	21/03/2017	0	0	0
2011193479	382	rural/clinic	No	21/03/2017	0	0	0
2015368808	382	rural/clinic	No	21/03/2017	0	0	0
2011193480	382	rural/clinic	No	21/03/2017	0	0	0

2014330644	382	rural/clinic	No	21/03/2017	0	0	0
2015288580	382	rural/clinic	No	21/03/2017	0	0	0
2015368805/D	382	rural/clinic	No	21/03/2017	0	0	0
2015414942	382	rural/clinic	No	21/03/2017	0	0	0
2012246191	382	rural/clinic	No	21/03/2017	0	0	0
2014375671	382	rural/clinic	No	21/03/2017	0	0	0
2012246189	382	rural/clinic	No	21/03/2017	0	0	0
2014375668	382	rural/clinic	No	21/03/2017	0	0	0
2015368806	382	rural/clinic	No	21/03/2017	0	0	0
2014375672	382	rural/clinic	No	21/03/2017	0	0	0
2015377157	382	rural/clinic	No	21/02/2017	0	0	0
2015368804	382	rural/clinic	No	21/03/2017	0	0	0
2015397521	382	rural/clinic	No	21/03/2017	0	0	0
2014297961	382	rural/clinic	No	21/03/2017	0	0	0
2015397522	382	rural/clinic	No	21/03/2017	0	0	0
2015397523	382	rural/clinic	No	21/03/2017	0	0	0
2015397524	382	rural/clinic	No	21/03/2017	0	0	0
2015414727	382	rural/clinic	No	21/03/2017	0	0	0
2015397525	382	rural/clinic	No	21/03/2017	0	0	0
2015368878	382	rural/clinic	No	21/03/2017	0	0	0
2015397526	382	rural/clinic	No	21/03/2017	0	0	0
2015368877	382	rural/clinic	No	21/03/2017	0	0	0
2015397527	382	rural/clinic	No	21/03/2017	0	0	0
2015397528	382	rural/clinic	No	21/03/2017	0	0	0
2015368879	382	rural/clinic	No	21/03/2017	0	0	0
2015397529	382	rural/clinic	No	21/03/2017	0	0	0
2014291862	382	rural/clinic	No	21/03/2017	0	0	0
2014291860	382	rural/clinic	No	21/03/2017	0	0	0
2012266990	382	rural/clinic	No	21/03/2017	0	0	0
2014261861	382	rural/clinic	No	21/03/2017	0	0	0
2012349537	382	rural/clinic	No	15/03/2017	0	0	0
2012349537	382	rural/clinic	No	15/03/2017	0	0	0
2012266989	382	rural/clinic	No	21/03/2017	0	0	0
2014358203	382	rural/clinic	No	21/03/2017	0	0	0
2012266987	382	rural/clinic	No	21/03/2017	0	0	0
2015418130	382	rural/clinic	No	21/03/2017	0	0	0
2012266988	382	rural/clinic	No	21/03/2017	0	0	0
2015418131	382	rural/clinic	No	21/03/2017	0	0	0
2015339007	382	rural/clinic	No	16/03/2017	0	0	0
2015339007	382	rural/clinic	No	16/03/2017	0	0	0
2013247705	382	rural/clinic	No	16/03/2017	0	0	0
2013247705	382	rural/clinic	No	16/03/2017	0	0	0
2015351410	382	rural/clinic	No	21/03/2017	0	0	0
2014314720	382	rural/clinic	No	21/03/2017	0	0	0
2015351408	382	rural/clinic	No	21/03/2017	0	0	0
2015413343	382	rural/clinic	No	21/03/2017	0	0	0
2012341040	382	rural/clinic	No	16/03/2017	0	0	0
2012341040	382	rural/clinic	No	16/03/2017	0	0	0
2015369675	382	rural/clinic	No	16/03/2017	0	0	0
2015369675	382	rural/clinic	No	16/03/2017	0	0	0
2015351407	382	rural/clinic	No	21/03/2017	0	0	0
2015363753	382	rural/clinic	No	21/03/2017	0	0	0
2014314723	382	rural/clinic	No	21/03/2017	0	0	0
2015334608	382	rural/clinic	No	15/03/2017	0	0	0
2015334608	382	rural/clinic	No	15/03/2017	0	0	0
2015351406	382	rural/clinic	No	21/03/2017	0	0	0
2015363755	382	rural/clinic	No	21/03/2017	0	0	0
2014306088	382	rural/clinic	No	16/03/2017	0	0	0
2014306088	382	rural/clinic	No	16/03/2017	0	0	0
2014327036	382	rural/clinic	No	16/03/2017	0	0	0
2015351411	382	rural/clinic	No	21/03/2017	0	0	0
2015363756	382	rural/clinic	No	21/03/2017	0	0	0
2015351409	382	rural/clinic	No	21/03/2017	0	0	0
2015322579	382	rural/clinic	No	15/03/2017	0	0	0
2015322579	382	rural/clinic	No	15/03/2017	0	0	0
2015322583	382	rural/clinic	No	15/03/2017	0	0	0
2015322583	382	rural/clinic	No	15/03/2017	0	0	0
2015363754	382	rural/clinic	No	21/03/2017	0	0	0
2013255257	382	rural/clinic	No	21/03/2017	0	0	0
2015357635	382	rural/clinic	No	16/03/2017	0	0	0
2015357635	382	rural/clinic	No	16/03/2017	0	0	0
2012359154	382	rural/clinic	No	16/03/2017	0	0	0
2012359154	382	rural/clinic	No	16/03/2017	0	0	0
2015339530	382	rural/clinic	No	16/03/2017	0	0	0
2015333802	382	rural/clinic	No	21/03/2017	0	0	0
2012306138	382	rural/clinic	No	21/03/2017	0	0	0
2015286529	382	rural/clinic	No	21/03/2017	0	0	0
2012323656	382	rural/clinic	No	16/03/2017	0	0	0
2012323656	382	rural/clinic	No	16/03/2017	0	0	0
2015357391	382	rural/clinic	No	21/03/2017	0	0	0
2015359840	382	rural/clinic	No	21/03/2017	0	0	0
2015357390	382	rural/clinic	No	21/03/2017	0	0	0

2015357388	382	rural/clinic	No	21/03/2017	0	0	0
2015359838	382	rural/clinic	No	21/03/2017	0	0	0
2014314721	382	rural/clinic	No	21/03/2017	0	0	0
2015357389	382	rural/clinic	No	21/03/2017	0	0	0
2014314724	382	rural/clinic	No	21/03/2017	0	0	0
2014327242	382	rural/clinic	No	21/03/2017	0	0	0
2014314725	382	rural/clinic	No	21/03/2017	0	0	0
2014320294	382	rural/clinic	No	21/03/2017	0	0	0
2015376116	382	rural/clinic	No	21/03/2017	0	0	0
2015376117	382	rural/clinic	No	21/03/2017	0	0	0
2015357184	382	rural/clinic	No	21/03/2017	0	0	0
2011142492	382	rural/clinic	No	21/03/2017	0	0	0
2015357384	382	rural/clinic	No	21/03/2017	0	0	0
2014363874	382	rural/clinic	No	21/03/2017	0	0	0
2015357383	382	rural/clinic	No	21/03/2017	0	0	0
2014363873	382	rural/clinic	No	21/03/2017	0	0	0
2015357385	382	rural/clinic	No	21/03/2017	0	0	0
2015340067	382	rural/clinic	No	21/03/2017	0	0	0
2012253129	382	rural/clinic	No	21/03/2017	0	0	0
2015357185	382	rural/clinic	No	21/03/2017	0	0	0
2014363871	382	rural/clinic	No	21/03/2017	0	0	0
2015357386	382	rural/clinic	No	21/03/2017	0	0	0
2013285124	382	rural/clinic	No	21/03/2017	0	0	0
2012312849	382	rural/clinic	No	21/03/2017	0	0	0
2013285123	382	rural/clinic	No	21/03/2017	0	0	0
2015414793	382	rural/clinic	No	21/03/2017	0	0	0
2012312850	382	rural/clinic	No	21/03/2017	0	0	0
2014369188	382	rural/clinic	No	21/03/2017	0	0	0
2015414792	382	rural/clinic	No	21/03/2017	0	0	0
2013249488	382	rural/clinic	No	21/03/2017	0	0	0
2014369189	382	rural/clinic	No	21/03/2017	0	0	0
2015414797	382	rural/clinic	No	21/03/2017	0	0	0
2012253128	382	rural/clinic	No	21/03/2017	0	0	0
2012393956	382	rural/clinic	No	21/03/2017	0	0	0
2015414795	382	rural/clinic	No	21/03/2017	0	0	0
2015342280	382	rural/clinic	No	21/03/2017	0	0	0
2013266110	382	rural/clinic	No	21/03/2017	0	0	0
2015414796	382	rural/clinic	No	21/03/2017	0	0	0
2015342279	382	rural/clinic	No	21/03/2017	0	0	0
2015376203	382	rural/clinic	No	21/03/2017	0	0	0
2014311449	382	rural/clinic	No	21/03/2017	0	0	0
2015342278	382	rural/clinic	No	21/03/2017	0	0	0
2012325688	382	rural/clinic	No	21/03/2017	0	0	0
2013273641	382	rural/clinic	No	21/03/2017	0	0	0
2015359841	382	rural/clinic	No	21/03/2017	0	0	0
2011204790	382	rural/clinic	No	21/03/2017	0	0	0
2014379443	382	rural/clinic	No	21/03/2017	0	0	0
2015413117	382	rural/clinic	No	21/03/2017	0	0	0
2012267315	382	rural/clinic	No	21/03/2017	0	0	0
2015414941	382	rural/clinic	No	21/03/2017	0	0	0
2015413116	382	rural/clinic	No	21/03/2017	0	0	0
2013266113	382	rural/clinic	No	21/03/2017	0	0	0
2015414794	382	rural/clinic	No	21/03/2017	0	0	0
2015320868	382	rural/clinic	No	21/03/2017	0	0	0
2015413115	382	rural/clinic	No	21/03/2017	0	0	0
2013266112	382	rural/clinic	No	21/03/2017	0	0	0
0	382	rural/clinic	No	21/03/2017	0	0	0
2015413113	382	rural/clinic	No	21/03/2017	0	0	0
2015413114	382	rural/clinic	No	21/03/2017	0	0	0
2014358684	382	rural/clinic	No	21/03/2017	0	0	0
2015413112	382	rural/clinic	No	21/03/2017	0	0	0
2015414728	382	rural/clinic	No	21/03/2017	0	0	0
2015413111	382	rural/clinic	No	21/03/2017	0	0	0
2015335581	382	rural/clinic	No	21/03/2017	0	0	0
2015413110	382	rural/clinic	No	21/03/2017	0	0	0
2015413109	382	rural/clinic	No	21/03/2017	0	0	0
2012295968	382	rural/clinic	No	21/03/2017	0	0	0
2015413108	382	rural/clinic	No	21/03/2017	0	0	0
2012295974	382	rural/clinic	No	21/03/2017	0	0	0
2014347729	382	rural/clinic	No	21/03/2017	0	0	0
2015359839	382	rural/clinic	No	21/03/2017	0	0	0
2014347726	382	rural/clinic	No	21/03/2017	0	0	0
2014347731	382	rural/clinic	No	21/03/2017	0	0	0
2015349059	382	rural/clinic	No	21/03/2017	0	0	0
2012284741	382	rural/clinic	No	21/03/2017	0	0	0
2013266111	382	rural/clinic	No	21/03/2017	0	0	0
2014287972	382	rural/clinic	No	21/03/2017	0	0	0
2015320869	382	rural/clinic	No	21/03/2017	0	0	0
2014366066	382	rural/clinic	No	21/03/2017	0	0	0
2014347730	382	rural/clinic	No	21/03/2017	0	0	0
2013266071	382	rural/clinic	No	21/03/2017	0	0	0
2012335659	382	rural/clinic	No	21/03/2017	0	0	0

2012335662	382	rural/clinic	No	21/03/2017	0	0	0
2012293008	382	rural/clinic	No	21/03/2017	0	0	0
2015344320	382	rural/clinic	No	21/03/2017	0	0	0
2014369041	382	rural/clinic	No	21/03/2017	0	0	0
2012295973	382	rural/clinic	No	21/03/2017	0	0	0
2014369042	382	rural/clinic	No	21/03/2017	0	0	0
2012325687	382	rural/clinic	No	21/03/2017	0	0	0
2012295972	382	rural/clinic	No	21/03/2017	0	0	0
2012295971	382	rural/clinic	No	21/03/2017	0	0	0
2012295970	382	rural/clinic	No	21/03/2017	0	0	0
2012295969	382	rural/clinic	No	21/03/2017	0	0	0
2015335579	382	rural/clinic	No	21/03/2017	0	0	0
2015384804	382	rural/clinic	No	21/03/2017	0	0	0
2012261447	382	rural/clinic	No	21/03/2017	0	0	0
2014343871	382	rural/clinic	No	21/03/2017	0	0	0
2015295199	382	rural/clinic	No	21/03/2017	0	0	0
2015337303	382	rural/clinic	No	21/03/2017	0	0	0
2015337304	382	rural/clinic	No	21/03/2017	0	0	0
2011204759	382	rural/clinic	No	21/03/2017	0	0	0
2013254704	382	rural/clinic	No	21/03/2017	0	0	0
2015322361	382	rural/clinic	No	21/03/2017	0	0	0
2015337302	382	rural/clinic	No	21/03/2017	0	0	0
2011204762	382	rural/clinic	No	21/03/2017	0	0	0
2014348802	382	rural/clinic	No	21/03/2017	0	0	0
2015362452	382	rural/clinic	No	21/03/2017	0	0	0
2014348801	382	rural/clinic	No	21/03/2017	0	0	0
2015362451	382	rural/clinic	No	21/03/2017	0	0	0
2015314372	382	rural/clinic	No	21/03/2017	0	0	0
2015362412	382	rural/clinic	No	21/03/2017	0	0	0
2013256397	382	rural/clinic	No	21/03/2017	0	0	0
2015314360	382	rural/clinic	No	21/03/2017	0	0	0
2015382415	382	rural/clinic	No	21/03/2017	0	0	0
2015365075	382	rural/clinic	No	21/03/2017	0	0	0
2013256398	382	rural/clinic	No	21/03/2017	0	0	0
2015365078	382	rural/clinic	No	21/03/2017	0	0	0
2013256400	382	rural/clinic	No	21/03/2017	0	0	0
2015314369	382	rural/clinic	No	21/03/2017	0	0	0
2015362454	382	rural/clinic	No	21/03/2017	0	0	0
2015362416	382	rural/clinic	No	21/03/2017	0	0	0
2015314361	382	rural/clinic	No	21/03/2017	0	0	0
2015365076	382	rural/clinic	No	21/03/2017	0	0	0
2015362417	382	rural/clinic	No	21/03/2017	0	0	0
2015365081	382	rural/clinic	No	21/03/2017	0	0	0
2015362415	382	rural/clinic	No	21/03/2017	0	0	0
2015362453	382	rural/clinic	No	21/03/2017	0	0	0
2015392862	382	rural/clinic	No	21/03/2017	0	0	0
2015362455	382	rural/clinic	No	21/03/2017	0	0	0
2015305778	382	rural/clinic	No	21/03/2017	0	0	0
2014369140	382	rural/clinic	No	21/03/2017	0	0	0
2015305776	382	rural/clinic	No	21/03/2017	0	0	0
2015365090	382	rural/clinic	No	21/03/2017	0	0	0
2011232677	382	rural/clinic	No	21/03/2017	0	0	0
2012307519	382	rural/clinic	No	21/03/2017	0	0	0
2012310142	382	rural/clinic	No	21/03/2017	0	0	0
2012307518	382	rural/clinic	No	21/03/2017	0	0	0
2012310141	382	rural/clinic	No	21/03/2017	0	0	0
2012307517	382	rural/clinic	No	21/03/2017	0	0	0
2015305775	382	rural/clinic	No	21/03/2017	0	0	0
2015305777	382	rural/clinic	No	21/03/2017	0	0	0
2012310143	382	rural/clinic	No	21/03/2017	0	0	0
2014322428	382	rural/clinic	No	21/03/2017	0	0	0
2015295349	382	rural/clinic	No	21/03/2017	0	0	0
2012243572	382	rural/clinic	No	21/03/2017	0	0	0
2014322426	382	rural/clinic	No	21/03/2017	0	0	0
2015295346	382	rural/clinic	No	21/03/2017	0	0	0
2015314370	382	rural/clinic	No	21/03/2017	0	0	0
2013254385	382	rural/clinic	No	21/03/2017	0	0	0
2015331641	382	rural/clinic	No	21/03/2017	0	0	0
2015295350	382	rural/clinic	No	21/03/2017	0	0	0
2015331642	716	rural/clinic	No	21/03/2017	0	0	0
2015331643	716	rural/clinic	No	21/03/2017	0	0	0
2015295348	716	rural/clinic	No	21/03/2017	0	0	0
2015331644	716	rural/clinic	No	21/03/2017	0	0	0
2015331645	716	rural/clinic	No	21/03/2017	0	0	0
2015314362	716	rural/clinic	No	21/03/2017	0	0	0
2015295347	716	rural/clinic	No	21/03/2017	0	0	0
2015365088	716	rural/clinic	No	21/03/2017	0	0	0
2015331647	918	rural/clinic	No	21/03/2017	0	0	0
2015391504	918	rural/clinic	No	21/03/2017	0	0	0
2015296052	918	rural/clinic	No	21/03/2017	0	0	0
2015365085	918	rural/clinic	No	21/03/2017	0	0	0
2015323805	59	rural/clinic	No	21/03/2017	0	0	0

2015391502	59	rural/clinic	No	21/03/2017	0	0	0
2012272766	59	rural/clinic	No	21/03/2017	0	0	0
2015322362	59	rural/clinic	No	21/03/2017	0	0	0
2015323807	59	rural/clinic	No	21/03/2017	0	0	0
2012272767	59	rural/clinic	No	21/03/2017	0	0	0
2015322363	59	rural/clinic	No	21/03/2017	0	0	0
2015296051	59	rural/clinic	No	21/03/2017	0	0	0
2015296060	59	rural/clinic	No	21/03/2017	0	0	0
2015322364	59	rural/clinic	No	21/03/2017	0	0	0
2015323806	59	rural/clinic	No	21/03/2017	0	0	0
2015296053	59	rural/clinic	No	21/03/2017	0	0	0
2012285872	59	rural/clinic	No	21/03/2017	0	0	0
2011204787	59	rural/clinic	No	21/03/2017	0	0	0
2011204791	59	rural/clinic	No	21/03/2017	0	0	0
2015374410	59	rural/clinic	No	21/03/2017	0	0	0
2015296059	59	rural/clinic	No	21/03/2017	0	0	0
2015374411	59	rural/clinic	No	21/03/2017	0	0	0
2015392860	59	rural/clinic	No	21/03/2017	0	0	0
2011204788	59	rural/clinic	No	21/03/2017	0	0	0
2015392861	59	rural/clinic	No	21/03/2017	0	0	0
2011204777	59	rural/clinic	No	21/03/2017	0	0	0
2015297377	59	rural/clinic	No	21/03/2017	0	0	0
2015392863	59	rural/clinic	No	21/03/2017	0	0	0
2015297084	59	rural/clinic	No	21/03/2017	0	0	0
2012363298	59	rural/clinic	No	21/03/2017	0	0	0
2015297201	59	rural/clinic	No	21/03/2017	0	0	0
2015392866	59	rural/clinic	No	21/03/2017	0	0	0
2015297371	59	rural/clinic	No	21/03/2017	0	0	0
2012363299	59	rural/clinic	No	21/03/2017	0	0	0
2015392865	59	rural/clinic	No	21/03/2017	0	0	0
2015297372	59	rural/clinic	No	21/03/2017	0	0	0
2015392856	59	rural/clinic	No	21/03/2017	0	0	0
2015297374	59	rural/clinic	No	21/03/2017	0	0	0
2012363300	59	rural/clinic	No	21/03/2017	0	0	0
2015297376	59	rural/clinic	No	21/03/2017	0	0	0
2015392857	59	rural/clinic	No	21/03/2017	0	0	0
2015297082	59	rural/clinic	No	21/03/2017	0	0	0
2012363297	59	rural/clinic	No	21/03/2017	0	0	0
2015392853	59	rural/clinic	No	21/03/2017	0	0	0
2015297379	59	rural/clinic	No	21/03/2017	0	0	0
2015297375	59	rural/clinic	No	21/03/2017	0	0	0
2015392854	59	rural/clinic	No	21/03/2017	0	0	0
2015392855	59	rural/clinic	No	21/03/2017	0	0	0
2015297373	59	rural/clinic	No	21/03/2017	0	0	0
2015392858	59	rural/clinic	No	21/03/2017	0	0	0
2015297083	59	rural/clinic	No	21/03/2017	0	0	0
2015392859	59	rural/clinic	No	21/03/2017	0	0	0
2011204763	59	rural/clinic	No	21/03/2017	0	0	0
2011204765	59	rural/clinic	No	21/03/2017	0	0	0
2011204775	59	rural/clinic	No	21/03/2017	0	0	0
2011204774	59	rural/clinic	No	21/03/2017	0	0	0
2011204770	59	rural/clinic	No	21/03/2017	0	0	0
2011204766	59	rural/clinic	No	21/03/2017	0	0	0
2011204764	59	rural/clinic	No	21/03/2017	0	0	0
2011204761	59	rural/clinic	No	21/03/2017	0	0	0
2011204780	59	rural/clinic	No	21/03/2017	0	0	0
2015365084	59	rural/clinic	No	21/03/2017	0	0	0
2015374409	59	rural/clinic	No	21/03/2017	0	0	0
2015374408	59	rural/clinic	No	21/03/2017	0	0	0
2015374403	59	rural/clinic	No	21/03/2017	0	0	0
2015374402	59	rural/clinic	No	21/03/2017	0	0	0
2015374350	59	rural/clinic	No	21/03/2017	0	0	0
2015374348	59	rural/clinic	No	21/03/2017	0	0	0
2015374346	59	rural/clinic	No	21/03/2017	0	0	0
2015374345	59	rural/clinic	No	21/03/2017	0	0	0
2015374344	59	rural/clinic	No	21/03/2017	0	0	0
2015374343	59	rural/clinic	No	21/03/2017	0	0	0
2015374342	59	rural/clinic	No	21/03/2017	0	0	0
2015349523	59	rural/clinic	No	09/03/2017	0	0	0
2014383039	59	rural/clinic	No	23/03/2017	0	0	0
2015377867	59	rural/clinic	No	23/03/2017	0	0	0
2015326517	59	rural/clinic	No	23/03/2017	0	0	0
2015326518	59	rural/clinic	No	23/03/2017	0	0	0
2012294464	59	rural/clinic	No	23/03/2017	0	0	0
2015326519	59	rural/clinic	No	23/03/2017	0	0	0
2013283248	59	rural/clinic	No	23/03/2017	0	0	0
2015326516	59	rural/clinic	No	23/03/2017	0	0	0
2015286630	59	rural/clinic	No	23/03/2017	0	0	0
2013258605	59	rural/clinic	No	23/03/2017	0	0	0
2015355386	59	rural/clinic	No	23/03/2017	0	0	0
2015390950	59	rural/clinic	No	23/03/2017	0	0	0
2015355382	59	rural/clinic	No	23/03/2017	0	0	0

2014335141	59	rural/clinic	No	23/03/2017	0	0	0
2013255740	59	rural/clinic	No	23/03/2017	0	0	0
2015301450	59	rural/clinic	No	23/03/2017	0	0	0
2011157141	59	rural/clinic	No	23/03/2017	0	0	0
2011157140	59	rural/clinic	No	23/03/2017	0	0	0
2015358160	59	rural/clinic	No	23/03/2017	0	0	0
2011157142	59	rural/clinic	No	23/03/2017	0	0	0
2015358161	59	rural/clinic	No	23/03/2017	0	0	0
2015358162	59	rural/clinic	No	23/03/2017	0	0	0
2015385518	59	rural/clinic	No	23/03/2017	0	0	0
2015358158	59	rural/clinic	No	23/03/2017	0	0	0
2015358159	59	rural/clinic	No	23/03/2017	0	0	0
2012251541	575	district/faith-based	No	23/03/2017	1	0	0
2015338751	575	district/faith-based	No	23/03/2017	1	0	0
2012251537	575	district/faith-based	No	23/03/2017	1	0	0
2015338752	575	district/faith-based	No	23/03/2017	1	0	0
2012251540	575	district/faith-based	No	23/03/2017	1	0	0
2015355907	575	district/faith-based	No	23/03/2017	1	0	0
2012251539	575	district/faith-based	No	23/03/2017	1	0	0
2015297305	575	district/faith-based	No	23/03/2017	1	0	0
2014319499	575	district/faith-based	No	23/03/2017	1	0	0
2012251538	575	district/faith-based	No	23/03/2017	1	0	0
2015297309	575	district/faith-based	No	23/03/2017	1	0	0
2011143619	575	district/faith-based	No	23/03/2017	1	0	0
2015301459	575	district/faith-based	No	23/03/2017	1	0	0
2015301458	575	district/faith-based	No	23/03/2017	1	0	0
2015297306	575	district/faith-based	No	23/03/2017	1	0	0
2014319723	575	district/faith-based	No	23/03/2017	1	0	0
2014289514	575	district/faith-based	No	23/03/2017	1	0	0
2015369677	575	district/faith-based	No	23/03/2017	1	0	0
2015301456	575	district/faith-based	No	23/03/2017	1	0	0
2014382287	575	district/faith-based	No	23/03/2017	1	0	0
2015369679	575	district/faith-based	No	22/03/2017	1	0	0
2015301457	575	district/faith-based	No	23/03/2017	1	0	0
2015414658	575	district/faith-based	No	23/03/2017	1	0	0
2015413120	575	district/faith-based	No	23/03/2017	1	0	0
2014382288	575	district/faith-based	No	23/03/2017	1	0	0
2015324256	575	district/faith-based	No	23/03/2017	1	0	0
2015413121	575	district/faith-based	No	23/03/2017	1	0	0
2014382289	575	district/faith-based	No	23/03/2017	1	0	0
2015324232	575	district/faith-based	No	23/03/2017	1	0	0
2015413118	575	district/faith-based	No	23/03/2017	1	0	0
2015324233	575	district/faith-based	No	23/03/2017	1	0	0
2014383038	575	district/faith-based	No	23/03/2017	1	0	0
2015413119	575	district/faith-based	No	23/03/2017	1	0	0
2014372330	575	district/faith-based	No	23/03/2017	1	0	0
2015383595	575	district/faith-based	No	23/03/2017	1	0	0
2015351417	575	district/faith-based	No	23/03/2017	1	0	0
2015351416	575	district/faith-based	No	23/03/2017	1	0	0
2015297453	575	district/faith-based	No	23/03/2017	1	0	0
2015351415	575	district/faith-based	No	23/03/2017	1	0	0
2014372724	575	district/faith-based	No	23/03/2017	1	0	0
2015297451	575	district/faith-based	No	23/03/2017	1	0	0
2015351414	575	district/faith-based	No	23/03/2017	1	0	0
2015352443	575	district/faith-based	No	23/03/2017	1	0	0
2015351413	575	district/faith-based	No	23/03/2017	1	0	0
2014382290	575	district/faith-based	No	23/03/2017	1	0	0
2015351412	575	district/faith-based	No	23/03/2017	1	0	0
2015352442	575	district/faith-based	No	23/03/2017	1	0	0
2014368844	575	district/faith-based	No	23/03/2017	1	0	0
2014326087	575	district/faith-based	No	23/03/2017	1	0	0
2015352444	575	district/faith-based	No	23/03/2017	1	0	0
2014368845	575	district/faith-based	No	23/03/2017	1	0	0
2015297085	575	district/faith-based	No	23/03/2017	1	0	0
2013283249	575	district/faith-based	No	23/03/2017	1	0	0
2014326084	575	district/faith-based	No	23/03/2017	1	0	0
2013283250	575	district/faith-based	No	23/03/2017	1	0	0
2014326085	575	district/faith-based	No	23/03/2017	1	0	0
2015332953	575	district/faith-based	No	23/03/2017	1	0	0
2014326086	575	district/faith-based	No	23/03/2017	1	0	0
2015332952	575	district/faith-based	No	23/03/2017	1	0	0
2014328396	575	district/faith-based	No	23/03/2017	1	0	0
2015289170	575	district/faith-based	No	23/03/2017	1	0	0
2015332951	575	district/faith-based	No	23/03/2017	1	0	0
2015397861	575	district/faith-based	No	23/03/2017	1	0	0
2013266115	575	district/faith-based	No	23/03/2017	1	0	0
2015313441	575	district/faith-based	No	23/03/2017	1	0	0
2015383594	575	district/faith-based	No	23/03/2017	1	0	0
2015313442	575	district/faith-based	No	23/03/2017	1	0	0
2015377865	575	district/faith-based	No	23/03/2017	1	0	0
2015313440	575	district/faith-based	No	23/03/2017	1	0	0
2015297370	575	district/faith-based	No	23/03/2017	1	0	0

2015377871	575	district/faith-based	No	23/03/2017	1	0	0
2014369043	575	district/faith-based	No	23/03/2017	1	0	0
2015313439	575	district/faith-based	No	23/03/2017	1	0	0
2014369044	575	district/faith-based	No	23/03/2017	1	0	0
2015377870	575	district/faith-based	No	23/03/2017	1	0	0
2013274243	575	district/faith-based	No	23/03/2017	1	0	0
2014371562	575	district/faith-based	No	23/03/2017	1	0	0
2015326558	575	district/faith-based	No	23/03/2017	1	0	0
2014371554	575	district/faith-based	No	23/03/2017	1	0	0
2015377866	575	district/faith-based	No	23/03/2017	1	0	0
2015377868	575	district/faith-based	No	23/03/2017	1	0	0
2012265918	575	district/faith-based	No	23/03/2017	1	0	0
2015368269	575	district/faith-based	No	23/03/2017	1	0	0
2015377869	575	district/faith-based	No	23/03/2017	1	0	0
2015368268	575	district/faith-based	No	23/03/2017	1	0	0
2013274242	575	district/faith-based	No	23/03/2017	1	0	0
2011199076	575	district/faith-based	No	23/03/2017	1	0	0
2015362501	575	district/faith-based	No	23/03/2017	1	0	0
2015301452	575	district/faith-based	No	23/03/2017	1	0	0
2015373199	575	district/faith-based	No	23/03/2017	1	0	0
2015373198	575	district/faith-based	No	23/03/2017	1	0	0
2014327085	575	district/faith-based	No	23/03/2017	1	0	0
2011221335	575	district/faith-based	No	23/03/2017	1	0	0
2015355383	575	district/faith-based	No	23/03/2017	1	0	0
2015373197	575	district/faith-based	No	23/03/2017	1	0	0
2014302517	575	district/faith-based	No	23/03/2017	1	0	0
2014302516	575	district/faith-based	No	23/03/2017	1	0	0
2011133909	575	district/faith-based	No	23/03/2017	1	0	0
2015349455	575	district/faith-based	No	23/03/2017	1	0	0
2011133908	575	district/faith-based	No	23/03/2017	1	0	0
2015373195	575	district/faith-based	No	23/03/2017	1	0	0
2012344494	575	district/faith-based	No	23/03/2017	1	0	0
2011199077	575	district/faith-based	No	23/03/2017	1	0	0
2015373196	575	district/faith-based	No	23/03/2017	1	0	0
2015301253	575	district/faith-based	No	23/03/2017	1	0	0
2015355384	575	district/faith-based	No	23/03/2017	1	0	0
2015373194	575	district/faith-based	No	23/03/2017	1	0	0
2011133613	575	district/faith-based	No	23/03/2017	1	0	0
2015373193	575	district/faith-based	No	23/03/2017	1	0	0
2011199243	575	district/faith-based	No	23/03/2017	1	0	0
2011133610	575	district/faith-based	No	23/03/2017	1	0	0
2011199244	575	district/faith-based	No	23/03/2017	1	0	0
2011133611	575	district/faith-based	No	23/03/2017	1	0	0
2015383600	575	district/faith-based	No	23/03/2017	1	0	0
2011133612	575	district/faith-based	No	23/03/2017	1	0	0
2015301099	575	district/faith-based	No	23/03/2017	1	0	0
2014297345	575	district/faith-based	No	23/03/2017	1	0	0
2015349454	575	district/faith-based	No	23/03/2017	1	0	0
2015301098	575	district/faith-based	No	23/03/2017	1	0	0
2011229216	575	district/faith-based	No	23/03/2017	1	0	0
2015326302	575	district/faith-based	No	23/03/2017	1	0	0
2015383596/D	575	district/faith-based	No	23/03/2017	1	0	0
2015383598	575	district/faith-based	No	23/03/2017	1	0	0
2015383599	575	district/faith-based	No	23/03/2017	1	0	0
2015326303	575	district/faith-based	No	23/03/2017	1	0	0
2015326304	575	district/faith-based	No	23/03/2017	1	0	0
2015301100	575	district/faith-based	No	23/03/2017	1	0	0
2015326305	575	district/faith-based	No	23/03/2017	1	0	0
2015326306	575	district/faith-based	No	23/03/2017	1	0	0
2015326307	575	district/faith-based	No	23/03/2017	1	0	0
2015326308	575	district/faith-based	No	23/03/2017	1	0	0
2015297454	575	district/faith-based	No	23/03/2017	1	0	0
2015326309	575	district/faith-based	No	23/03/2017	1	0	0
2015326311	575	district/faith-based	No	23/03/2017	1	0	0
2015297452	575	district/faith-based	No	23/03/2017	1	0	0
2015326312	575	district/faith-based	No	23/03/2017	1	0	0
2014382285	575	district/faith-based	No	23/03/2017	1	0	0
2015326313	575	district/faith-based	No	23/03/2017	1	0	0
2013262220	575	district/faith-based	No	23/03/2017	1	0	0
2014382286	575	district/faith-based	No	23/03/2017	1	0	0
2015326314	575	district/faith-based	No	23/03/2017	1	0	0
2014300534	575	district/faith-based	No	23/03/2017	1	0	0
2015326465	60	rural/clinic	No	23/03/2017	0	0	0
2015397531	60	rural/clinic	No	24/03/2017	0	0	0
2015331665	60	rural/clinic	No	23/03/2017	0	0	0
2015326466	60	rural/clinic	No	23/03/2017	0	0	0
2015326467	60	rural/clinic	No	23/03/2017	0	0	0
2015326468	60	rural/clinic	No	23/03/2017	0	0	0
2015326469	60	rural/clinic	No	23/03/2017	0	0	0
2015326470	60	rural/clinic	No	23/03/2017	0	0	0
2014337044	60	rural/clinic	No	23/03/2017	0	0	0
2015326472	60	rural/clinic	No	23/03/2017	0	0	0

2014300422	60	rural/clinic	No	23/03/2017	0	0	0
2015326473	60	rural/clinic	No	23/03/2017	0	0	0
2015331717	60	rural/clinic	No	23/03/2017	0	0	0
2015326474	60	rural/clinic	No	23/03/2017	0	0	0
2015326475	60	rural/clinic	No	23/03/2017	0	0	0
2015326476	60	rural/clinic	No	23/03/2017	0	0	0
2015326477	60	rural/clinic	No	23/03/2017	0	0	0
2015402366	60	rural/clinic	No	23/03/2017	0	0	0
2015402363	60	rural/clinic	No	23/03/2017	0	0	0
2015402364	60	rural/clinic	No	23/03/2017	0	0	0
2015402365	60	rural/clinic	No	23/03/2017	0	0	0
2015402362	60	rural/clinic	No	23/03/2017	0	0	0
2015402361	60	rural/clinic	No	23/03/2017	0	0	0
2015402360	60	rural/clinic	No	23/03/2017	0	0	0
2015402357	60	rural/clinic	No	23/03/2017	0	0	0
2015402526	60	rural/clinic	No	23/03/2017	0	0	0
2015402527	60	rural/clinic	No	23/03/2017	0	0	0
2015402528	60	rural/clinic	No	23/03/2017	0	0	0
2015402529	60	rural/clinic	No	23/03/2017	0	0	0
2015402530	60	rural/clinic	No	23/03/2017	0	0	0
2015339011	60	rural/clinic	No	23/03/2017	0	0	0
2014340269	60	rural/clinic	No	23/03/2017	0	0	0
2014340270	60	rural/clinic	No	23/03/2017	0	0	0
2015326521	60	rural/clinic	No	23/03/2017	0	0	0
2015326522	60	rural/clinic	No	23/03/2017	0	0	0
2015326301	60	rural/clinic	No	23/03/2017	0	0	0
2015339008	60	rural/clinic	No	23/03/2017	0	0	0
2015339009	60	rural/clinic	No	23/03/2017	0	0	0
2014340271	60	rural/clinic	No	23/03/2017	0	0	0
2015339010	60	rural/clinic	No	23/03/2017	0	0	0
2014300423	60	rural/clinic	No	23/03/2017	0	0	0
2012314010	60	rural/clinic	No	23/03/2017	0	0	0
2012314009	60	rural/clinic	No	23/03/2017	0	0	0
2015286596	60	rural/clinic	No	23/03/2017	0	0	0
2015286597	60	rural/clinic	No	23/03/2017	0	0	0
2015378726	60	rural/clinic	No	23/03/2017	0	0	0
2015378725	113	rural/clinic	No	23/03/2017	0	0	0
2015286629	113	rural/clinic	No	23/03/2017	0	0	0
2015286595	113	rural/clinic	No	23/03/2017	0	0	0
2015286628	113	rural/clinic	No	23/03/2017	0	0	0
2015286627	113	rural/clinic	No	23/03/2017	0	0	0
2014357727	113	rural/clinic	No	23/03/2017	0	0	0
2014357728	113	rural/clinic	No	23/03/2017	0	0	0
2014357726	113	rural/clinic	No	23/03/2017	0	0	0
2015339539	113	rural/clinic	No	23/03/2017	0	0	0
2015339541	113	rural/clinic	No	23/03/2017	0	0	0
2015339540	477	rural/clinic	No	23/03/2017	0	0	0
2015339537	477	rural/clinic	No	23/03/2017	0	0	0
2015339535	477	rural/clinic	No	23/03/2017	0	0	0
2015339536	477	rural/clinic	No	23/03/2017	0	0	0
2014320527	477	rural/clinic	No	23/03/2017	0	0	0
2015301448	477	rural/clinic	No	23/03/2017	0	0	0
2015301449	477	rural/clinic	No	23/03/2017	0	0	0
2015340836	477	rural/clinic	No	23/03/2017	0	0	0
2015340837	477	rural/clinic	No	23/03/2017	0	0	0
2015340838	477	rural/clinic	No	23/03/2017	0	0	0
2015340839	477	rural/clinic	No	23/03/2017	0	0	0
2015340840	477	rural/clinic	No	23/03/2017	0	0	0
2014337043	477	rural/clinic	No	23/03/2017	0	0	0
2015383597	477	rural/clinic	No	23/03/2017	0	0	0
2011117492	477	rural/clinic	No	23/03/2017	0	0	0
2012294465	477	rural/clinic	No	23/03/2017	0	0	0
2013283247	760	rural/clinic	No	23/03/2017	0	0	0
2015355385	760	rural/clinic	No	23/03/2017	0	0	0
2015355380	760	rural/clinic	No	23/03/2017	0	0	0
2015413114	760	rural/clinic	No	21/03/2017	0	0	0
2015413114	760	rural/clinic	No	21/03/2017	0	0	0
2015413343	760	rural/clinic	No	21/03/2017	0	0	0
2012335659	760	rural/clinic	No	21/03/2017	0	0	0
2015382893	760	rural/clinic	No	21/03/2017	0	0	0
2015382893	760	rural/clinic	No	21/03/2017	0	0	0
2015351608	760	rural/clinic	No	21/03/2017	0	0	0
2014375668	760	rural/clinic	No	21/03/2017	0	0	0
2014375668	760	rural/clinic	No	21/03/2017	0	0	0
2015287351	498	rural/clinic	No	21/03/2017	0	0	0
2015287351	498	rural/clinic	No	21/03/2017	0	0	0
2015320868	498	rural/clinic	No	21/03/2017	0	0	0
2012363296	498	rural/clinic	No	21/03/2017	0	0	0
2012363296	498	rural/clinic	No	21/03/2017	0	0	0
2014303075	498	rural/clinic	No	21/03/2017	0	0	0
2014303075	498	rural/clinic	No	21/03/2017	0	0	0
2015335580	498	rural/clinic	No	21/03/2017	0	0	0

2015335580	498	rural/clinic	No	21/03/2017	0	0	0
2015289324	498	rural/clinic	No	21/03/2017	0	0	0
2015289324	498	rural/clinic	No	21/03/2017	0	0	0
2015340954	498	rural/clinic	No	21/03/2017	0	0	0
2015340954	498	rural/clinic	No	21/03/2017	0	0	0
2015340958	498	rural/clinic	No	21/03/2017	0	0	0
2015340958	498	rural/clinic	No	21/03/2017	0	0	0
2015331913	498	rural/clinic	No	21/03/2017	0	0	0
2015331913	498	rural/clinic	No	21/03/2017	0	0	0
2015385941	498	rural/clinic	No	21/03/2017	0	0	0
2015385947	203	district/faith-based	No	21/03/2017	1	0	0
2014291594	203	district/faith-based	No	21/03/2017	1	0	0
2014291594	203	district/faith-based	No	21/03/2017	1	0	0
2012259869	203	district/faith-based	No	23/03/2017	1	0	0
2014291860	203	district/faith-based	No	21/03/2017	1	0	0
2014291860	203	district/faith-based	No	21/03/2017	1	0	0
2015406451	203	district/faith-based	No	24/03/2017	1	0	0
2015406452	203	district/faith-based	No	24/03/2017	1	0	0
2015373200	203	district/faith-based	No	24/03/2017	1	0	0
2015373190	203	district/faith-based	No	24/03/2017	1	0	0
2015373170	204	rural/clinic	No	24/03/2017	0	0	0
2015373171	204	rural/clinic	No	24/03/2017	0	0	0
2015373176	204	rural/clinic	No	24/03/2017	0	0	0
2015373177	632	rural/clinic	No	24/03/2017	0	0	0
2015373184	632	rural/clinic	No	24/03/2017	0	0	0
2015373185	632	rural/clinic	No	24/03/2017	0	0	0
2015373187	632	rural/clinic	No	24/03/2017	0	0	0
2015373192	632	rural/clinic	No	24/03/2017	0	0	0
2015373179	632	rural/clinic	No	24/03/2017	0	0	0
2015373180	632	rural/clinic	No	24/03/2017	0	0	0
2012310146	632	rural/clinic	No	24/03/2017	0	0	0
2011204789	261	rural/clinic	No	24/03/2017	0	0	0
2012310144	261	rural/clinic	No	24/03/2017	0	0	0
2011144005	261	rural/clinic	No	24/03/2017	0	0	0
2011144016	261	rural/clinic	No	24/03/2017	0	0	0
2011144017	261	rural/clinic	No	24/03/2017	0	0	0
2011144020	261	rural/clinic	No	24/03/2017	0	0	0
2011144021	261	rural/clinic	No	24/03/2017	0	0	0
2011144023	261	rural/clinic	No	24/03/2017	0	0	0
2015405094	261	rural/clinic	No	24/03/2017	0	0	0
2015405095	261	rural/clinic	No	24/03/2017	0	0	0
2012390145	261	rural/clinic	No	24/03/2017	0	0	0
2012390146	261	rural/clinic	No	24/03/2017	0	0	0
2012390147	261	rural/clinic	No	24/03/2017	0	0	0
2015331720	261	rural/clinic	No	24/03/2017	0	0	0
2015331721	261	rural/clinic	No	24/03/2017	0	0	0
2015331722	261	rural/clinic	No	24/03/2017	0	0	0
2015331723	261	rural/clinic	No	24/03/2017	0	0	0
2015403252	261	rural/clinic	No	24/03/2017	0	0	0
2015403253	261	rural/clinic	No	24/03/2017	0	0	0
2015303179	261	rural/clinic	No	24/03/2017	0	0	0
2015303178	261	rural/clinic	No	24/03/2017	0	0	0
2015331718	261	rural/clinic	No	24/03/2017	0	0	0
2015331719	261	rural/clinic	No	24/03/2017	0	0	0
2015362452	261	rural/clinic	No	21/03/2017	0	0	0
2015362452	261	rural/clinic	No	21/03/2017	0	0	0
2015314372	261	rural/clinic	No	21/03/2017	0	0	0
2015326469	261	rural/clinic	No	23/03/2017	0	0	0
2015326469	261	rural/clinic	No	23/03/2017	0	0	0
2015301458	261	rural/clinic	No	23/03/2017	0	0	0
2015301458	261	rural/clinic	No	23/03/2017	0	0	0
2014326086	261	rural/clinic	No	23/03/2017	0	0	0
2015358158	261	rural/clinic	No	23/03/2017	0	0	0
2012251537	527	rural/clinic	No	23/03/2017	0	0	0
2012251537	527	rural/clinic	No	23/03/2017	0	0	0
2014355847	527	rural/clinic	No	21/03/2017	0	0	0
2014355847	527	rural/clinic	No	21/03/2017	0	0	0
2015397539	527	rural/clinic	No	28/03/2017	0	0	0
2015397538	527	rural/clinic	No	28/03/2017	0	0	0
2015397537	527	rural/clinic	No	28/03/2017	0	0	0
2015397536	527	rural/clinic	No	28/03/2017	0	0	0
2015397535	527	rural/clinic	No	28/03/2017	0	0	0
2015397534	527	rural/clinic	No	28/03/2017	0	0	0
2015297533	527	rural/clinic	No	28/03/2017	0	0	0
2015397532	527	rural/clinic	No	28/03/2017	0	0	0
2015397530	527	rural/clinic	No	28/03/2017	0	0	0
2015303066	527	rural/clinic	No	28/03/2017	0	0	0
2015303067	527	rural/clinic	No	28/03/2017	0	0	0
2015303065	527	rural/clinic	No	28/03/2017	0	0	0
2015303068	527	rural/clinic	No	28/03/2017	0	0	0
2014362872	527	rural/clinic	No	28/03/2017	0	0	0
2015405617	527	rural/clinic	No	28/03/2017	0	0	0

2015333804	527	rural/clinic	No	28/03/2017	0	0	0
2015405616	655	rural/clinic	No	28/03/2017	0	0	0
2015413648	655	rural/clinic	No	28/03/2017	0	0	0
2015413642	655	rural/clinic	No	28/03/2017	0	0	0
2015413647	655	rural/clinic	No	28/03/2017	0	0	0
2015413644	655	rural/clinic	No	28/03/2017	0	0	0
2015413646	655	rural/clinic	No	28/03/2017	0	0	0
2015413643	655	rural/clinic	No	28/03/2017	0	0	0
2015413645	655	rural/clinic	No	28/03/2017	0	0	0
2015335435	655	rural/clinic	No	28/03/2017	0	0	0
2015335436	655	rural/clinic	No	28/03/2017	0	0	0
2015355733	655	rural/clinic	No	28/03/2017	0	0	0
1	655	rural/clinic	No	28/03/2017	0	0	0
2	655	rural/clinic	No	28/03/2017	0	0	0
3	655	rural/clinic	No	28/03/2017	0	0	0
4	655	rural/clinic	No	28/03/2017	0	0	0
5	655	rural/clinic	No	28/03/2017	0	0	0
6	65	rural/clinic	No	28/03/2017	0	0	0
7	65	rural/clinic	No	28/03/2017	0	0	0
8	65	rural/clinic	No	28/03/2017	0	0	0
9	65	rural/clinic	No	28/03/2017	0	0	0
10	65	rural/clinic	No	28/03/2017	0	0	0
11	65	rural/clinic	No	28/03/2017	0	0	0
12	65	rural/clinic	No	28/03/2017	0	0	0
13	65	rural/clinic	No	28/03/2017	0	0	0
14	65	rural/clinic	No	28/03/2017	0	0	0
15	65	rural/clinic	No	28/03/2017	0	0	0
16	65	rural/clinic	No	28/03/2017	0	0	0
17	65	rural/clinic	No	28/03/2017	0	0	0
18	65	rural/clinic	No	28/03/2017	0	0	0
19	65	rural/clinic	No	28/03/2017	0	0	0
20	65	rural/clinic	No	28/03/2017	0	0	0
21	65	rural/clinic	No	28/03/2017	0	0	0
22	65	rural/clinic	No	28/03/2017	0	0	0
23	65	rural/clinic	No	28/03/2017	0	0	0
24	65	rural/clinic	No	28/03/2017	0	0	0
25	65	rural/clinic	No	28/03/2017	0	0	0
26	65	rural/clinic	No	28/03/2017	0	0	0
27	65	rural/clinic	No	28/03/2017	0	0	0
28	65	rural/clinic	No	28/03/2017	0	0	0
29	65	rural/clinic	No	28/03/2017	0	0	0
30	65	rural/clinic	No	28/03/2017	0	0	0
31	33	rural/clinic	No	28/03/2017	0	0	0
32	33	rural/clinic	No	28/03/2017	0	0	0
33	33	rural/clinic	No	28/03/2017	0	0	0
34	33	rural/clinic	No	28/03/2017	0	0	0
35	33	rural/clinic	No	28/03/2017	0	0	0
36	33	rural/clinic	No	28/03/2017	0	0	0
37	33	rural/clinic	No	28/03/2017	0	0	0
38	33	rural/clinic	No	28/03/2017	0	0	0
39	33	rural/clinic	No	28/03/2017	0	0	0
40	33	rural/clinic	No	28/03/2017	0	0	0
41	33	rural/clinic	No	28/03/2017	0	0	0
42	33	rural/clinic	No	28/03/2017	0	0	0
43	33	rural/clinic	No	28/03/2017	0	0	0
44	33	rural/clinic	No	28/03/2017	0	0	0
45	33	rural/clinic	No	28/03/2017	0	0	0
46	33	rural/clinic	No	28/03/2017	0	0	0
47	33	rural/clinic	No	28/03/2017	0	0	0
48	33	rural/clinic	No	28/03/2017	0	0	0
49	33	rural/clinic	No	28/03/2017	0	0	0
50	33	rural/clinic	No	28/03/2017	0	0	0
51	33	rural/clinic	No	28/03/2017	0	0	0
52	33	rural/clinic	No	28/03/2017	0	0	0
53	33	rural/clinic	No	28/03/2017	0	0	0
54	33	rural/clinic	No	28/03/2017	0	0	0
55	33	rural/clinic	No	28/03/2017	0	0	0
56	33	rural/clinic	No	28/03/2017	0	0	0
57	33	rural/clinic	No	28/03/2017	0	0	0
58	33	rural/clinic	No	28/03/2017	0	0	0
59	33	rural/clinic	No	28/03/2017	0	0	0
60	33	rural/clinic	No	28/03/2017	0	0	0
61	33	rural/clinic	No	28/03/2017	0	0	0
62	33	rural/clinic	No	28/03/2017	0	0	0
63	33	rural/clinic	No	28/03/2017	0	0	0
64	33	rural/clinic	No	28/03/2017	0	0	0
65	33	rural/clinic	No	28/03/2017	0	0	0
66	33	rural/clinic	No	28/03/2017	0	0	0
67	33	rural/clinic	No	28/03/2017	0	0	0
68	33	rural/clinic	No	28/03/2017	0	0	0
69	33	rural/clinic	No	28/03/2017	0	0	0
70	33	rural/clinic	No	28/03/2017	0	0	0

71	33	rural/clinic	No	28/03/2017	0	0	0
72	33	rural/clinic	No	28/03/2017	0	0	0
73	33	rural/clinic	No	28/03/2017	0	0	0
74	33	rural/clinic	No	28/03/2017	0	0	0
75	33	rural/clinic	No	28/03/2017	0	0	0
76	33	rural/clinic	No	28/03/2017	0	0	0
77	33	rural/clinic	No	28/03/2017	0	0	0
78	33	rural/clinic	No	28/03/2017	0	0	0
79	33	rural/clinic	No	28/03/2017	0	0	0
80	33	rural/clinic	No	28/03/2017	0	0	0
81	33	rural/clinic	No	28/03/2017	0	0	0
82	33	rural/clinic	No	28/03/2017	0	0	0
83	33	rural/clinic	No	28/03/2017	0	0	0
84	33	rural/clinic	No	28/03/2017	0	0	0
85	33	rural/clinic	No	28/03/2017	0	0	0
86	33	rural/clinic	No	28/03/2017	0	0	0
87	33	rural/clinic	No	28/03/2017	0	0	0
88	33	rural/clinic	No	28/03/2017	0	0	0
89	33	rural/clinic	No	28/03/2017	0	0	0
90	33	rural/clinic	No	28/03/2017	0	0	0
91	33	rural/clinic	No	28/03/2017	0	0	0
92	33	rural/clinic	No	28/03/2017	0	0	0
93	33	rural/clinic	No	28/03/2017	0	0	0
94	33	rural/clinic	No	28/03/2017	0	0	0
95	33	rural/clinic	No	28/03/2017	0	0	0
96	33	rural/clinic	No	28/03/2017	0	0	0
97	33	rural/clinic	No	28/03/2017	0	0	0
98	33	rural/clinic	No	28/03/2017	0	0	0
99	33	rural/clinic	No	28/03/2017	0	0	0
100	33	rural/clinic	No	28/03/2017	0	0	0
101	33	rural/clinic	No	28/03/2017	0	0	0
102	33	rural/clinic	No	28/03/2017	0	0	0
103	33	rural/clinic	No	28/03/2017	0	0	0
104	33	rural/clinic	No	28/03/2017	0	0	0
105	33	rural/clinic	No	28/03/2017	0	0	0
1105	33	rural/clinic	No	28/03/2017	0	0	0
2105	33	rural/clinic	No	28/03/2017	0	0	0
3105	33	rural/clinic	No	28/03/2017	0	0	0
4105	33	rural/clinic	No	28/03/2017	0	0	0
5105	33	rural/clinic	No	28/03/2017	0	0	0
6105	33	rural/clinic	No	28/03/2017	0	0	0
7105	33	rural/clinic	No	28/03/2017	0	0	0
8105	33	rural/clinic	No	28/03/2017	0	0	0
9105	33	rural/clinic	No	28/03/2017	0	0	0
1001	33	rural/clinic	No	28/03/2017	0	0	0
1002	33	rural/clinic	No	28/03/2017	0	0	0
1003	45	rural/clinic	No	28/03/2017	0	0	0
89	45	rural/clinic	No	28/03/2017	0	0	0
90	45	rural/clinic	No	28/03/2017	0	0	0
91	45	rural/clinic	No	28/03/2017	0	0	0
92	45	rural/clinic	No	28/03/2017	0	0	0
93	45	rural/clinic	No	28/03/2017	0	0	0
94	45	rural/clinic	No	28/03/2017	0	0	0
95	45	rural/clinic	No	28/03/2017	0	0	0
96	45	rural/clinic	No	28/03/2017	0	0	0
97	45	rural/clinic	No	28/03/2017	0	0	0
98	45	rural/clinic	No	28/03/2017	0	0	0
99	45	rural/clinic	No	28/03/2017	0	0	0
100	45	rural/clinic	No	28/03/2017	0	0	0
101	45	rural/clinic	No	28/03/2017	0	0	0
102	45	rural/clinic	No	28/03/2017	0	0	0
103	45	rural/clinic	No	28/03/2017	0	0	0
104	45	rural/clinic	No	28/03/2017	0	0	0
105	45	rural/clinic	No	28/03/2017	0	0	0
1105	45	rural/clinic	No	28/03/2017	0	0	0
2105	45	rural/clinic	No	28/03/2017	0	0	0
3105	45	rural/clinic	No	28/03/2017	0	0	0
4105	45	rural/clinic	No	28/03/2017	0	0	0
5105	45	rural/clinic	No	28/03/2017	0	0	0
6105	45	rural/clinic	No	28/03/2017	0	0	0
7105	45	rural/clinic	No	28/03/2017	0	0	0
8105	45	rural/clinic	No	28/03/2017	0	0	0
9105	45	rural/clinic	No	28/03/2017	0	0	0
1001	45	rural/clinic	No	28/03/2017	0	0	0
1002	45	rural/clinic	No	28/03/2017	0	0	0
77	45	rural/clinic	No	28/03/2017	0	0	0
78	45	rural/clinic	No	28/03/2017	0	0	0
79	45	rural/clinic	No	28/03/2017	0	0	0
80	45	rural/clinic	No	28/03/2017	0	0	0
81	45	rural/clinic	No	28/03/2017	0	0	0
82	45	rural/clinic	No	28/03/2017	0	0	0
83	45	rural/clinic	No	28/03/2017	0	0	0

84	45	rural/clinic	No	28/03/2017	0	0	0
85	45	rural/clinic	No	28/03/2017	0	0	0
86	45	rural/clinic	No	28/03/2017	0	0	0
87	45	rural/clinic	No	28/03/2017	0	0	0
88	45	rural/clinic	No	28/03/2017	0	0	0
89	45	rural/clinic	No	28/03/2017	0	0	0
90	45	rural/clinic	No	28/03/2017	0	0	0
91	45	rural/clinic	No	28/03/2017	0	0	0
92	45	rural/clinic	No	28/03/2017	0	0	0
93	45	rural/clinic	No	28/03/2017	0	0	0
94	45	rural/clinic	No	28/03/2017	0	0	0
95	45	rural/clinic	No	28/03/2017	0	0	0
96	45	rural/clinic	No	28/03/2017	0	0	0
97	45	rural/clinic	No	28/03/2017	0	0	0
98	45	rural/clinic	No	28/03/2017	0	0	0
99	45	rural/clinic	No	28/03/2017	0	0	0
100	45	rural/clinic	No	28/03/2017	0	0	0
2015383594	45	rural/clinic	No	28/03/2017	0	0	0
2015313442	45	rural/clinic	No	28/03/2017	0	0	0
2015377865	45	rural/clinic	No	28/03/2017	0	0	0
2015313440	45	rural/clinic	No	28/03/2017	0	0	0
2015297370	45	rural/clinic	No	28/03/2017	0	0	0
2015377871	45	rural/clinic	No	28/03/2017	0	0	0
2014369043	45	rural/clinic	No	28/03/2017	0	0	0
2015313439	45	rural/clinic	No	28/03/2017	0	0	0
2014369044	45	rural/clinic	No	28/03/2017	0	0	0
2015377870	45	rural/clinic	No	28/03/2017	0	0	0
2013274243	45	rural/clinic	No	28/03/2017	0	0	0
2014371562	45	rural/clinic	No	28/03/2017	0	0	0
2015326558	45	rural/clinic	No	28/03/2017	0	0	0
2014371554	45	rural/clinic	No	28/03/2017	0	0	0
2015377866	45	rural/clinic	No	28/03/2017	0	0	0
2015377868	45	rural/clinic	No	28/03/2017	0	0	0
2012265918	45	rural/clinic	No	28/03/2017	0	0	0
2015368269	45	rural/clinic	No	28/03/2017	0	0	0
2015377869	45	rural/clinic	No	28/03/2017	0	0	0
2015368268	45	rural/clinic	No	28/03/2017	0	0	0
2013274242	45	rural/clinic	No	28/03/2017	0	0	0
2011199076	45	rural/clinic	No	28/03/2017	0	0	0
2015362501	45	rural/clinic	No	28/03/2017	0	0	0
2015301452	45	rural/clinic	No	28/03/2017	0	0	0
2015373199	45	rural/clinic	No	28/03/2017	0	0	0
2015373198	45	rural/clinic	No	28/03/2017	0	0	0
2014327085	45	rural/clinic	No	28/03/2017	0	0	0
2011221335	45	rural/clinic	No	28/03/2017	0	0	0
2015355383	45	rural/clinic	No	28/03/2017	0	0	0
2015373197	45	rural/clinic	No	28/03/2017	0	0	0
2014302517	45	rural/clinic	No	28/03/2017	0	0	0
2014302516	45	rural/clinic	No	28/03/2017	0	0	0
2011133909	45	rural/clinic	No	28/03/2017	0	0	0
	45	rural/clinic	No	28/03/2017	0	0	0
	45	rural/clinic	No	28/03/2017	0	0	0
	45	rural/clinic	No	28/03/2017	0	0	0
	45	rural/clinic	No	28/03/2017	0	0	0
	45	rural/clinic	No	28/03/2017	0	0	0
	45	rural/clinic	No	28/03/2017	0	0	0
	45	rural/clinic	No	28/03/2017	0	0	0
	45	rural/clinic	No	28/03/2017	0	0	0
	45	rural/clinic	No	28/03/2017	0	0	0
	45	rural/clinic	No	28/03/2017	0	0	0
	45	rural/clinic	No	28/03/2017	0	0	0
	45	rural/clinic	No	28/03/2017	0	0	0
	45	rural/clinic	No	28/03/2017	0	0	0
2012364938	45	rural/clinic	No	28/03/2017	0	0	0
2016270496	45	rural/clinic	No	28/03/2017	0	0	0
2015377870	45	rural/clinic	No	23/03/2017	0	0	0
2015377870	45	rural/clinic	No	23/03/2017	0	0	0
2015335432	45	rural/clinic	No	28/03/2017	0	0	0
2015335433	45	rural/clinic	No	28/03/2017	0	0	0
2015335434	45	rural/clinic	No	28/03/2017	0	0	0
2015337959	45	rural/clinic	No	28/03/2017	0	0	0
2015338457	45	rural/clinic	No	28/03/2017	0	0	0
2012352350	45	rural/clinic	No	28/03/2017	0	0	0
2012352348	45	rural/clinic	No	28/03/2017	0	0	0
2012352349	45	rural/clinic	No	28/03/2017	0	0	0
2015337960	45	rural/clinic	No	28/03/2017	0	0	0
2014359730	45	rural/clinic	No	28/03/2017	0	0	0
2015362457	45	rural/clinic	No	28/03/2017	0	0	0
2012335663	45	rural/clinic	No	28/03/2017	0	0	0
2015362461	45	rural/clinic	No	28/03/2017	0	0	0
2015362463	45	rural/clinic	No	28/03/2017	0	0	0
2015362464	45	rural/clinic	No	28/03/2017	0	0	0

2015377641	45	rural/clinic	No	28/03/2017	0	0	0
2015362465	45	rural/clinic	No	28/03/2017	0	0	0
2015377640	45	rural/clinic	No	28/03/2017	0	0	0
2015289566	45	rural/clinic	No	28/03/2017	0	0	0
2015289567	45	rural/clinic	No	28/03/2017	0	0	0
2012316542	45	rural/clinic	No	28/03/2017	0	0	0
2013270497	45	rural/clinic	No	28/03/2017	0	0	0
2015351615	45	rural/clinic	No	28/03/2017	0	0	0
2015289169	45	rural/clinic	No	28/03/2017	0	0	0
2015289326	45	rural/clinic	No	28/03/2017	0	0	0
2014348065	45	rural/clinic	No	28/03/2017	0	0	0
2015335583	45	rural/clinic	No	28/03/2017	0	0	0
2013270498	45	rural/clinic	No	28/03/2017	0	0	0
2015355775	45	rural/clinic	No	28/03/2017	0	0	0
20170000008	45	rural/clinic	No	28/03/2017	0	0	0
2015289284	45	rural/clinic	No	28/03/2017	0	0	0
2012291488	45	rural/clinic	No	28/03/2017	0	0	0
2015337958	45	rural/clinic	No	28/03/2017	0	0	0
2015377636	45	rural/clinic	No	28/03/2017	0	0	0
2012335664	45	rural/clinic	No	28/03/2017	0	0	0
2015333914	45	rural/clinic	No	28/03/2017	0	0	0
2015357392	45	rural/clinic	No	28/03/2017	0	0	0
2015331886	45	rural/clinic	No	28/03/2017	0	0	0
2015379045	45	rural/clinic	No	28/03/2017	0	0	0
2015379044	45	rural/clinic	No	28/03/2017	0	0	0
2015379047	45	rural/clinic	No	28/03/2017	0	0	0
2015337957	45	rural/clinic	No	28/03/2017	0	0	0
2015379046	45	rural/clinic	No	28/03/2017	0	0	0
2015289281	45	rural/clinic	No	28/03/2017	0	0	0
2015379037	45	rural/clinic	No	28/03/2017	0	0	0
2015289283	45	rural/clinic	No	28/03/2017	0	0	0
2015377638	45	rural/clinic	No	28/03/2017	0	0	0
2012261380	45	rural/clinic	No	28/03/2017	0	0	0
2015377637	45	rural/clinic	No	28/03/2017	0	0	0
2015379036	45	rural/clinic	No	28/03/2017	0	0	0
2015379033	45	rural/clinic	No	28/03/2017	0	0	0
2015289282	45	rural/clinic	No	28/03/2017	0	0	0
2015359842	45	rural/clinic	No	28/03/2017	0	0	0
2015379034	45	rural/clinic	No	28/03/2017	0	0	0
2015355732	45	rural/clinic	No	28/03/2017	0	0	0
2015340068	45	rural/clinic	No	28/03/2017	0	0	0
2015379032	45	rural/clinic	No	28/03/2017	0	0	0
2014314458	45	rural/clinic	No	28/03/2017	0	0	0
2015357526	45	rural/clinic	No	28/03/2017	0	0	0
2011113949	45	rural/clinic	No	28/03/2017	0	0	0
2015379030	45	rural/clinic	No	28/03/2017	0	0	0
2015331887	45	rural/clinic	No	28/03/2017	0	0	0
2015379031	45	rural/clinic	No	28/03/2017	0	0	0
2015379027	45	rural/clinic	No	28/03/2017	0	0	0
2015331885	45	rural/clinic	No	28/03/2017	0	0	0
2015357397	45	rural/clinic	No	28/03/2017	0	0	0
2015379029	45	rural/clinic	No	28/03/2017	0	0	0
2015375976	45	rural/clinic	No	28/03/2017	0	0	0
2015357524	45	rural/clinic	No	28/03/2017	0	0	0
2015379028	45	rural/clinic	No	28/03/2017	0	0	0
2015357523	45	rural/clinic	No	28/03/2017	0	0	0
2015379026	45	rural/clinic	No	28/03/2017	0	0	0
2015375977	45	rural/clinic	No	28/03/2017	0	0	0
2015379025	45	rural/clinic	No	28/03/2017	0	0	0
2015357396	45	rural/clinic	No	28/03/2017	0	0	0
2015375974	45	rural/clinic	No	28/03/2017	0	0	0
2014330647	45	rural/clinic	No	28/03/2017	0	0	0
2015375973	45	rural/clinic	No	28/03/2017	0	0	0
2015379024	45	rural/clinic	No	28/03/2017	0	0	0
2015379023	45	rural/clinic	No	28/03/2017	0	0	0
2015375975	45	rural/clinic	No	28/03/2017	0	0	0
2015379039	45	rural/clinic	No	28/03/2017	0	0	0
2015331889	45	rural/clinic	No	28/03/2017	0	0	0
2015331888	45	rural/clinic	No	28/03/2017	0	0	0
2015379035	45	rural/clinic	No	28/03/2017	0	0	0
2012253965	45	rural/clinic	No	28/03/2017	0	0	0
2013256265	45	rural/clinic	No	28/03/2017	0	0	0
2015379040	45	rural/clinic	No	28/03/2017	0	0	0
2012253963	45	rural/clinic	No	28/03/2017	0	0	0
2014356970	45	rural/clinic	No	28/03/2017	0	0	0
2013256264	45	rural/clinic	No	28/03/2017	0	0	0
2012266992	45	rural/clinic	No	28/03/2017	0	0	0
2014356969	45	rural/clinic	No	28/03/2017	0	0	0
2013256266	45	rural/clinic	No	28/03/2017	0	0	0
2012266991	45	rural/clinic	No	28/03/2017	0	0	0
2014288004	45	rural/clinic	No	28/03/2017	0	0	0
2015368567	45	rural/clinic	No	28/03/2017	0	0	0

2015418135	45	rural/clinic	No	28/03/2017	0	0	0
2014288003	45	rural/clinic	No	28/03/2017	0	0	0
2014288005	45	rural/clinic	No	28/03/2017	0	0	0
2015418133	45	rural/clinic	No	28/03/2017	0	0	0
2015368568	45	rural/clinic	No	28/03/2017	0	0	0
2014356968	45	rural/clinic	No	28/03/2017	0	0	0
2014342510	45	rural/clinic	No	28/03/2017	0	0	0
2011181149	45	rural/clinic	No	28/03/2017	0	0	0
2015418132	45	rural/clinic	No	28/03/2017	0	0	0
2012253964	45	rural/clinic	No	28/03/2017	0	0	0
2014342511	45	rural/clinic	No	28/03/2017	0	0	0
2012368534	45	rural/clinic	No	28/03/2017	0	0	0
2015310655	45	rural/clinic	No	28/03/2017	0	0	0
2014320293	45	rural/clinic	No	28/03/2017	0	0	0
2015310656	45	rural/clinic	No	28/03/2017	0	0	0
2015325178	45	rural/clinic	No	28/03/2017	0	0	0
2015351616	45	rural/clinic	No	28/03/2017	0	0	0
2015351614	45	rural/clinic	No	28/03/2017	0	0	0
2015351613	45	rural/clinic	No	28/03/2017	0	0	0
2015310654	45	rural/clinic	No	28/03/2017	0	0	0
2015351611	45	rural/clinic	No	28/03/2017	0	0	0
2015357395	45	rural/clinic	No	28/03/2017	0	0	0
2015351609	45	rural/clinic	No	28/03/2017	0	0	0
2012284986	45	rural/clinic	No	28/03/2017	0	0	0
2015325179	45	rural/clinic	No	28/03/2017	0	0	0
2015351610	45	rural/clinic	No	28/03/2017	0	0	0
2014320295	45	rural/clinic	No	28/03/2017	0	0	0
2014330646	45	rural/clinic	No	28/03/2017	0	0	0
2011223202	45	rural/clinic	No	28/03/2017	0	0	0
2015325177	45	rural/clinic	No	28/03/2017	0	0	0
2015412505	45	rural/clinic	No	28/03/2017	0	0	0
2014291546	45	rural/clinic	No	28/03/2017	0	0	0
2013271838	45	rural/clinic	No	28/03/2017	0	0	0
2015412503	45	rural/clinic	No	28/03/2017	0	0	0
2014299901	45	rural/clinic	No	28/03/2017	0	0	0
2015346226	45	rural/clinic	No	28/03/2017	0	0	0
2015357525	45	rural/clinic	No	28/03/2017	0	0	0
2015346227	45	rural/clinic	No	28/03/2017	0	0	0
2011144804	45	rural/clinic	No	28/03/2017	0	0	0
2014304801	45	rural/clinic	No	28/03/2017	0	0	0
2011144803	45	rural/clinic	No	28/03/2017	0	0	0
2014299902	45	rural/clinic	No	28/03/2017	0	0	0
2015333913	45	rural/clinic	No	28/03/2017	0	0	0
2014289511	45	rural/clinic	No	28/03/2017	0	0	0
2013264060	45	rural/clinic	No	28/03/2017	0	0	0
2014299903	45	rural/clinic	No	28/03/2017	0	0	0
2013264061	45	rural/clinic	No	28/03/2017	0	0	0
2012242578	45	rural/clinic	No	28/03/2017	0	0	0
2015369920	45	rural/clinic	No	28/03/2017	0	0	0
2015414943	45	rural/clinic	No	28/03/2017	0	0	0
2014299904	45	rural/clinic	No	28/03/2017	0	0	0
2015369921	45	rural/clinic	No	28/03/2017	0	0	0
2014299905	45	rural/clinic	No	28/03/2017	0	0	0
2014360953	45	rural/clinic	No	28/03/2017	0	0	0
2014299906	45	rural/clinic	No	28/03/2017	0	0	0
2014375675	45	rural/clinic	No	28/03/2017	0	0	0
2015418195	45	rural/clinic	No	28/03/2017	0	0	0
2015337048	45	rural/clinic	No	28/03/2017	0	0	0
2014375673	45	rural/clinic	No	28/03/2017	0	0	0
2015418192	45	rural/clinic	No	28/03/2017	0	0	0
2014299909	45	rural/clinic	No	28/03/2017	0	0	0
2015418190	45	rural/clinic	No	28/03/2017	0	0	0
2014291815	45	rural/clinic	No	28/03/2017	0	0	0
2014299908	45	rural/clinic	No	28/03/2017	0	0	0
2015418191	45	rural/clinic	No	28/03/2017	0	0	0
2014291814	45	rural/clinic	No	28/03/2017	0	0	0
2014299907	45	rural/clinic	No	28/03/2017	0	0	0
2015418189	45	rural/clinic	No	28/03/2017	0	0	0
2014397962	45	rural/clinic	No	28/03/2017	0	0	0
2011156598	45	rural/clinic	No	28/03/2017	0	0	0
2014344339	45	rural/clinic	No	28/03/2017	0	0	0
2015385440	45	rural/clinic	No	28/03/2017	0	0	0
2014297963	45	rural/clinic	No	28/03/2017	0	0	0
2015315995	45	rural/clinic	No	28/03/2017	0	0	0
2014344340	45	rural/clinic	No	28/03/2017	0	0	0
2014314543	45	rural/clinic	No	28/03/2017	0	0	0
2015315996	45	rural/clinic	No	28/03/2017	0	0	0
2014344349	45	rural/clinic	No	28/03/2017	0	0	0
2015415051	45	rural/clinic	No	28/03/2017	0	0	0
2015315997	45	rural/clinic	No	28/03/2017	0	0	0
2014344338	45	rural/clinic	No	28/03/2017	0	0	0
2014358328	45	rural/clinic	No	28/03/2017	0	0	0

2015385949	45	rural/clinic	No	28/03/2017	0	0	0
2014360954	45	rural/clinic	No	28/03/2017	0	0	0
2014378723	45	rural/clinic	No	28/03/2017	0	0	0
2015385948	45	rural/clinic	No	28/03/2017	0	0	0
2014305941	45	rural/clinic	No	28/03/2017	0	0	0
2015355511	45	rural/clinic	No	28/03/2017	0	0	0
2015362805	45	rural/clinic	No	28/03/2017	0	0	0
2014314542	45	rural/clinic	No	28/03/2017	0	0	0
2015337362	45	rural/clinic	No	28/03/2017	0	0	0
2015364367	45	rural/clinic	No	28/03/2017	0	0	0
2014305940	45	rural/clinic	No	28/03/2017	0	0	0
2015337363	45	rural/clinic	No	28/03/2017	0	0	0
2014307320	45	rural/clinic	No	28/03/2017	0	0	0
2015364366	45	rural/clinic	No	28/03/2017	0	0	0
2015338708	45	rural/clinic	No	28/03/2017	0	0	0
2014307322	45	rural/clinic	No	28/03/2017	0	0	0
2014307323	45	rural/clinic	No	28/03/2017	0	0	0
2015364365	45	rural/clinic	No	28/03/2017	0	0	0
2015338710	45	rural/clinic	No	28/03/2017	0	0	0
2014307321	45	rural/clinic	No	28/03/2017	0	0	0
2012323776	45	rural/clinic	No	28/03/2017	0	0	0
2014307319	45	rural/clinic	No	28/03/2017	0	0	0
2015364364	45	rural/clinic	No	28/03/2017	0	0	0
2014355848	45	rural/clinic	No	28/03/2017	0	0	0
2015364362	45	rural/clinic	No	28/03/2017	0	0	0
2014358186	45	rural/clinic	No	28/03/2017	0	0	0
2015337364	45	rural/clinic	No	28/03/2017	0	0	0
2015364361	45	rural/clinic	No	28/03/2017	0	0	0
2015338709	45	rural/clinic	No	28/03/2017	0	0	0
2015363007	45	rural/clinic	No	28/03/2017	0	0	0
	45	rural/clinic	No	28/03/2017	0	0	0
	45	rural/clinic	No	28/03/2017	0	0	0
	45	rural/clinic	No	28/03/2017	0	0	0
	45	rural/clinic	No	28/03/2017	0	0	0
	45	rural/clinic	No	28/03/2017	0	0	0
	45	rural/clinic	No	28/03/2017	0	0	0
	45	rural/clinic	No	28/03/2017	0	0	0
	45	rural/clinic	No	28/03/2017	0	0	0
	45	rural/clinic	No	28/03/2017	0	0	0
	45	rural/clinic	No	28/03/2017	0	0	0
	45	rural/clinic	No	28/03/2017	0	0	0
	45	rural/clinic	No	28/03/2017	0	0	0
	45	rural/clinic	No	28/03/2017	0	0	0
	45	rural/clinic	No	28/03/2017	0	0	0
	45	rural/clinic	No	28/03/2017	0	0	0
	45	rural/clinic	No	28/03/2017	0	0	0
	45	rural/clinic	No	28/03/2017	0	0	0
	45	rural/clinic	No	28/03/2017	0	0	0
	45	rural/clinic	No	28/03/2017	0	0	0
	45	rural/clinic	No	28/03/2017	0	0	0
	45	rural/clinic	No	28/03/2017	0	0	0
	45	rural/clinic	No	28/03/2017	0	0	0
	45	rural/clinic	No	28/03/2017	0	0	0
	45	rural/clinic	No	28/03/2017	0	0	0
	45	rural/clinic	No	28/03/2017	0	0	0
	45	rural/clinic	No	28/03/2017	0	0	0
	45	rural/clinic	No	28/03/2017	0	0	0
	45	rural/clinic	No	28/03/2017	0	0	0
	45	rural/clinic	No	28/03/2017	0	0	0
	45	rural/clinic	No	28/03/2017	0	0	0
	45	rural/clinic	No	28/03/2017	0	0	0
	45	rural/clinic	No	28/03/2017	0	0	0
	45	rural/clinic	No	28/03/2017	0	0	0
	45	rural/clinic	No	28/03/2017	0	0	0
	45	rural/clinic	No	28/03/2017	0	0	0
	45	rural/clinic	No	28/03/2017	0	0	0
	45	rural/clinic	No	28/03/2017	0	0	0
	45	rural/clinic	No	28/03/2017	0	0	0
	45	rural/clinic	No	28/03/2017	0	0	0
	45	rural/clinic	No	28/03/2017	0	0	0
	45	rural/clinic	No	28/03/2017	0	0	0
	45	rural/clinic	No	28/03/2017	0	0	0
	45	rural/clinic	No	28/03/2017	0	0	0
	45	rural/clinic	No	28/03/2017	0	0	0
	45	rural/clinic	No	28/03/2017	0	0	0
	45	rural/clinic	No	28/03/2017	0	0	0
	45	rural/clinic	No	28/03/2017	0	0	0
	45	rural/clinic	No	28/03/2017	0	0	0
	45	rural/clinic	No	28/03/2017	0	0	0
	45	rural/clinic	No	28/03/2017	0	0	0
	45	rural/clinic	No	28/03/2017	0	0	0
	45	rural/clinic	No	28/03/2017	0	0	0

	45	rural/clinic	No	28/03/2017	0	0	0
	45	rural/clinic	No	28/03/2017	0	0	0
	45	rural/clinic	No	28/03/2017	0	0	0
	45	rural/clinic	No	28/03/2017	0	0	0
	45	rural/clinic	No	28/03/2017	0	0	0
	45	rural/clinic	No	28/03/2017	0	0	0
	45	rural/clinic	No	28/03/2017	0	0	0
	45	rural/clinic	No	28/03/2017	0	0	0
	45	rural/clinic	No	28/03/2017	0	0	0
	45	rural/clinic	No	28/03/2017	0	0	0
	45	rural/clinic	No	28/03/2017	0	0	0
	45	rural/clinic	No	28/03/2017	0	0	0
	45	rural/clinic	No	28/03/2017	0	0	0
	45	rural/clinic	No	28/03/2017	0	0	0
	45	rural/clinic	No	28/03/2017	0	0	0
	45	rural/clinic	No	28/03/2017	0	0	0
	45	rural/clinic	No	28/03/2017	0	0	0
	45	rural/clinic	No	28/03/2017	0	0	0
	45	rural/clinic	No	28/03/2017	0	0	0
	45	rural/clinic	No	28/03/2017	0	0	0
	45	rural/clinic	No	28/03/2017	0	0	0
	45	rural/clinic	No	28/03/2017	0	0	0
	45	rural/clinic	No	28/03/2017	0	0	0
	45	rural/clinic	No	28/03/2017	0	0	0
	45	rural/clinic	No	28/03/2017	0	0	0
	45	rural/clinic	No	28/03/2017	0	0	0
	45	rural/clinic	No	28/03/2017	0	0	0
	45	rural/clinic	No	28/03/2017	0	0	0
	45	rural/clinic	No	28/03/2017	0	0	0
	45	rural/clinic	No	28/03/2017	0	0	0
	45	rural/clinic	No	28/03/2017	0	0	0
	45	rural/clinic	No	28/03/2017	0	0	0
	45	rural/clinic	No	28/03/2017	0	0	0
	45	rural/clinic	No	28/03/2017	0	0	0
	45	rural/clinic	No	28/03/2017	0	0	0
	45	rural/clinic	No	28/03/2017	0	0	0
	45	rural/clinic	No	28/03/2017	0	0	0
	45	rural/clinic	No	28/03/2017	0	0	0
	45	rural/clinic	No	28/03/2017	0	0	0
	45	rural/clinic	No	28/03/2017	0	0	0
	45	rural/clinic	No	28/03/2017	0	0	0
	45	rural/clinic	No	28/03/2017	0	0	0
	45	rural/clinic	No	28/03/2017	0	0	0
	45	rural/clinic	No	28/03/2017	0	0	0
	45	rural/clinic	No	28/03/2017	0	0	0
	45	rural/clinic	No	28/03/2017	0	0	0
	45	rural/clinic	No	28/03/2017	0	0	0
	45	rural/clinic	No	28/03/2017	0	0	0
	45	rural/clinic	No	28/03/2017	0	0	0
	45	rural/clinic	No	28/03/2017	0	0	0
	45	rural/clinic	No	28/03/2017	0	0	0
	45	rural/clinic	No	28/03/2017	0	0	0
	45	rural/clinic	No	28/03/2017	0	0	0
	45	rural/clinic	No	28/03/2017	0	0	0
	45	rural/clinic	No	28/03/2017	0	0	0
	45	rural/clinic	No	28/03/2017	0	0	0
	45	rural/clinic	No	28/03/2017	0	0	0
	45	rural/clinic	No	28/03/2017	0	0	0
	45	rural/clinic	No	28/03/2017	0	0	0
2012345098	45	rural/clinic	No	28/03/2017	0	0	0
2015363008	45	rural/clinic	No	28/03/2017	0	0	0
2015363055	45	rural/clinic	No	28/03/2017	0	0	0
2015385441	45	rural/clinic	No	28/03/2017	0	0	0
2012345097	45	rural/clinic	No	28/03/2017	0	0	0
2015363056	46	rural/clinic	No	28/03/2017	0	0	0
2015337050	46	rural/clinic	No	28/03/2017	0	0	0
2015363054	919	rural/clinic	No	28/03/2017	0	0	0
2015337049	919	rural/clinic	No	28/03/2017	0	0	0
2014362809	919	rural/clinic	No	28/03/2017	0	0	0
2015405913	919	rural/clinic	No	28/03/2017	0	0	0
2014347816	919	rural/clinic	No	28/03/2017	0	0	0
2015323053	919	rural/clinic	No	28/03/2017	0	0	0
2015360717	919	rural/clinic	No	28/03/2017	0	0	0
2014350365	919	rural/clinic	No	28/03/2017	0	0	0
2013244113	919	rural/clinic	No	28/03/2017	0	0	0
2013244112	919	rural/clinic	No	28/03/2017	0	0	0
2014365380	919	rural/clinic	No	28/03/2017	0	0	0
2013261293	919	rural/clinic	No	28/03/2017	0	0	0
2014365379	919	rural/clinic	No	28/03/2017	0	0	0
2015360719	919	rural/clinic	No	28/03/2017	0	0	0
2014350630	919	rural/clinic	No	28/03/2017	0	0	0
2014350629	499	rural/clinic	No	28/03/2017	0	0	0

2012369019	499	rural/clinic	No	28/03/2017	0	0	0
2015360718	499	rural/clinic	No	28/03/2017	0	0	0
2012369017	499	rural/clinic	No	28/03/2017	0	0	0
2011141600	499	rural/clinic	No	28/03/2017	0	0	0
2012369020	499	rural/clinic	No	28/03/2017	0	0	0
2014364201	499	rural/clinic	No	28/03/2017	0	0	0
2012369018	205	rural/clinic	No	28/03/2017	0	0	0
2011141599	205	rural/clinic	No	28/03/2017	0	0	0
2012336609	205	rural/clinic	No	28/03/2017	0	0	0
2015405714	205	rural/clinic	No	28/03/2017	0	0	0
0	205	rural/clinic	No	28/03/2017	0	0	0
2014289509	205	rural/clinic	No	28/03/2017	0	0	0
2012364937	205	rural/clinic	No	28/03/2017	0	0	0
2014348235	205	rural/clinic	No	28/03/2017	0	0	0
2014289508	656	district/faith-based	No	28/03/2017	1	0	0
2012364945	656	district/faith-based	No	28/03/2017	1	0	0
2014289510	656	district/faith-based	No	28/03/2017	1	0	0
2015286865	656	district/faith-based	No	28/03/2017	1	0	0
2015403151	656	district/faith-based	No	28/03/2017	1	0	0
2014289504	656	district/faith-based	No	28/03/2017	1	0	0
2012289619	656	district/faith-based	No	28/03/2017	1	0	0
2015405713	656	district/faith-based	No	28/03/2017	1	0	0
2014289501	656	district/faith-based	No	28/03/2017	1	0	0
2014348234	656	district/faith-based	No	28/03/2017	1	0	0
2014289505	656	district/faith-based	No	28/03/2017	1	0	0
2014363872	656	district/faith-based	No	28/03/2017	1	0	0
2014289503	656	district/faith-based	No	28/03/2017	1	0	0
2012289893	656	district/faith-based	No	28/03/2017	1	0	0
2012289684	656	district/faith-based	No	28/03/2017	1	0	0
2014348233	656	district/faith-based	No	28/03/2017	1	0	0
2015289171	656	district/faith-based	No	28/03/2017	1	0	0
2015342305	656	district/faith-based	No	28/03/2017	1	0	0
2012359915	656	district/faith-based	No	28/03/2017	1	0	0
2012359914	656	district/faith-based	No	28/03/2017	1	0	0
2012359913	656	district/faith-based	No	28/03/2017	1	0	0
2014289502	656	district/faith-based	No	28/03/2017	1	0	0
2015397551	656	district/faith-based	No	28/03/2017	1	0	0
2014289507	656	district/faith-based	No	28/03/2017	1	0	0
2014289506	656	district/faith-based	No	28/03/2017	1	0	0
2015397552	656	district/faith-based	No	28/03/2017	1	0	0
2015293462	656	district/faith-based	No	28/03/2017	1	0	0
2015351612	656	district/faith-based	No	28/03/2017	1	0	0
2015293463	656	district/faith-based	No	28/03/2017	1	0	0
2015293464	656	district/faith-based	No	28/03/2017	1	0	0
2015293461	656	district/faith-based	No	28/03/2017	1	0	0
2011144802	656	district/faith-based	No	28/03/2017	1	0	0
2014289513	656	district/faith-based	No	28/03/2017	1	0	0
2014289512	656	district/faith-based	No	28/03/2017	1	0	0
2015405715	656	district/faith-based	No	28/03/2017	1	0	0
2015405718	656	district/faith-based	No	28/03/2017	1	0	0
2015403152	656	district/faith-based	No	28/03/2017	1	0	0
2015379041	656	district/faith-based	No	28/03/2017	1	0	0
2015405707	656	district/faith-based	No	28/03/2017	1	0	0
2015405716	656	district/faith-based	No	28/03/2017	1	0	0
2015351667	656	district/faith-based	No	28/03/2017	1	0	0
2015379042	656	district/faith-based	No	28/03/2017	1	0	0
2014308890	656	district/faith-based	No	28/03/2017	1	0	0
2014308891	656	district/faith-based	No	28/03/2017	1	0	0
2012335658	656	district/faith-based	No	28/03/2017	1	0	0
2012335661	656	district/faith-based	No	28/03/2017	1	0	0
2012335660	656	district/faith-based	No	28/03/2017	1	0	0
2015377639	656	district/faith-based	No	28/03/2017	1	0	0
2015379043	656	district/faith-based	No	28/03/2017	1	0	0
2014350680	656	district/faith-based	No	28/03/2017	1	0	0
2015373198	656	district/faith-based	No	23/03/2017	1	0	0
2015373198	656	district/faith-based	No	23/03/2017	1	0	0
2011133909	656	district/faith-based	No	23/03/2017	1	0	0
2014289514	656	district/faith-based	No	23/03/2017	1	0	0
2014289514	656	district/faith-based	No	23/03/2017	1	0	0
2014382286	656	district/faith-based	No	23/03/2017	1	0	0
2014382286	656	district/faith-based	No	23/03/2017	1	0	0
2015373176	656	district/faith-based	No	24/03/2017	1	0	0
2015373176	656	district/faith-based	No	24/03/2017	1	0	0
2011144016	656	district/faith-based	No	24/03/2017	1	0	0
2011144016	656	district/faith-based	No	24/03/2017	1	0	0
2015373407	656	district/faith-based	No	11/05/2017	1	0	0
2015383598	656	district/faith-based	No	23/03/2017	1	0	0
2015383598	656	district/faith-based	No	23/03/2017	1	0	0
2015326476	656	district/faith-based	No	23/03/2017	1	0	0
2015326476	656	district/faith-based	No	23/03/2017	1	0	0
2015326521	656	district/faith-based	No	23/03/2017	1	0	0
2015326521	656	district/faith-based	No	23/03/2017	1	0	0

2015339535	656	district/faith-based	No	23/03/2017	1	0	0
2015331664	656	district/faith-based	No	16/03/2017	1	0	0
2014321519	656	district/faith-based	No	16/03/2017	1	0	0
2015288503	656	district/faith-based	No	16/03/2017	1	0	0
2015360508	656	district/faith-based	No	30/03/2017	1	0	0
2015360509	656	district/faith-based	No	30/03/2017	1	0	0
2014372331	656	district/faith-based	No	30/03/2017	1	0	0
2015385519	656	district/faith-based	No	30/03/2017	1	0	0
2015360504	656	district/faith-based	No	30/03/2017	1	0	0
2014346712	656	district/faith-based	No	30/03/2017	1	0	0
2013255342	656	district/faith-based	No	30/03/2017	1	0	0
2015385521	656	district/faith-based	No	30/03/2017	1	0	0
2015413124	656	district/faith-based	No	30/03/2017	1	0	0
2015413123	656	district/faith-based	No	30/03/2017	1	0	0
2015413122	656	district/faith-based	No	30/03/2017	1	0	0
2015351419	656	district/faith-based	No	30/03/2017	1	0	0
2015373685	656	district/faith-based	No	30/03/2017	1	0	0
2015351418	656	district/faith-based	No	30/03/2017	1	0	0
2012304325	656	district/faith-based	No	30/03/2017	1	0	0
2014328760	656	district/faith-based	No	30/03/2017	1	0	0
2012304326	656	district/faith-based	No	30/03/2017	1	0	0
2014291864	656	district/faith-based	No	30/03/2017	1	0	0
2011133617	656	district/faith-based	No	30/03/2017	1	0	0
2015373686	656	district/faith-based	No	30/03/2017	1	0	0
2014291867	656	district/faith-based	No	30/03/2017	1	0	0
2015373687	656	district/faith-based	No	30/03/2017	1	0	0
2012304324	656	district/faith-based	No	30/03/2017	1	0	0
2014291863	656	district/faith-based	No	30/03/2017	1	0	0
2015397916	656	district/faith-based	No	30/03/2017	1	0	0
2015397917	656	district/faith-based	No	30/03/2017	1	0	0
2014291865	656	district/faith-based	No	30/03/2017	1	0	0
2015373688	656	district/faith-based	No	30/03/2017	1	0	0
2012304327	656	district/faith-based	No	30/03/2017	1	0	0
2015301460	656	district/faith-based	No	30/03/2017	1	0	0
2015301455	656	district/faith-based	No	30/03/2017	1	0	0
2014291866	656	district/faith-based	No	30/03/2017	1	0	0
2015301461	656	district/faith-based	No	30/03/2017	1	0	0
2015349529	656	district/faith-based	No	30/03/2017	1	0	0
2015377213	656	district/faith-based	No	30/03/2017	1	0	0
2011225408	656	district/faith-based	No	30/03/2017	1	0	0
2015373689	656	district/faith-based	No	30/03/2017	1	0	0
2015377212	656	district/faith-based	No	30/03/2017	1	0	0
2015349453	656	district/faith-based	No	30/03/2017	1	0	0
2011225409	656	district/faith-based	No	30/03/2017	1	0	0
2015384034	656	district/faith-based	No	30/03/2017	1	0	0
2011225410	656	district/faith-based	No	30/03/2017	1	0	0
2014298582	656	district/faith-based	No	30/03/2017	1	0	0
2015384035	656	district/faith-based	No	30/03/2017	1	0	0
2015373690	656	district/faith-based	No	30/03/2017	1	0	0
2013262223	656	district/faith-based	No	30/03/2017	1	0	0
2015373691	656	district/faith-based	No	30/03/2017	1	0	0
2015384033	656	district/faith-based	No	30/03/2017	1	0	0
2015349456	656	district/faith-based	No	30/03/2017	1	0	0
2015373692	656	district/faith-based	No	30/03/2017	1	0	0
2015384032	656	district/faith-based	No	30/03/2017	1	0	0
2015301462	656	district/faith-based	No	30/03/2017	1	0	0
2015355390	656	district/faith-based	No	30/03/2017	1	0	0
2015305065	656	district/faith-based	No	30/03/2017	1	0	0
2015373693	656	district/faith-based	No	30/03/2017	1	0	0
2012312300	656	district/faith-based	No	30/03/2017	1	0	0
2015373694	656	district/faith-based	No	30/03/2017	1	0	0
2011133618	656	district/faith-based	No	30/03/2017	1	0	0
2015325989	656	district/faith-based	No	30/03/2017	1	0	0
2014382914	656	district/faith-based	No	30/03/2017	1	0	0
2015373695	656	district/faith-based	No	30/03/2017	1	0	0
2011133616	656	district/faith-based	No	30/03/2017	1	0	0
2015325990	656	district/faith-based	No	30/03/2017	1	0	0
2014303295	656	district/faith-based	No	30/03/2017	1	0	0
2012305697	656	district/faith-based	No	30/03/2017	1	0	0
2015301451	656	district/faith-based	No	30/03/2017	1	0	0
2013273396	656	district/faith-based	No	30/03/2017	1	0	0
2015373696	656	district/faith-based	No	30/03/2017	1	0	0
2011133614	656	district/faith-based	No	30/03/2017	1	0	0
2015373697	656	district/faith-based	No	30/03/2017	1	0	0
2014301460	656	district/faith-based	No	30/03/2017	1	0	0
2015301453	656	district/faith-based	No	30/03/2017	1	0	0
2012246282	656	district/faith-based	No	30/03/2017	1	0	0
2015349528	656	district/faith-based	No	30/03/2017	1	0	0
2011229215	656	district/faith-based	No	30/03/2017	1	0	0
2015373698	656	district/faith-based	No	30/03/2017	1	0	0
2015325988	656	district/faith-based	No	30/03/2017	1	0	0
2012291845	656	district/faith-based	No	30/03/2017	1	0	0

2014327038	656	district/faith-based	No	30/03/2017	1	0	0
2015373699	656	district/faith-based	No	30/03/2017	1	0	0
2011199242	656	district/faith-based	No	30/03/2017	1	0	0
2015373700	656	district/faith-based	No	30/03/2017	1	0	0
2011225366	656	district/faith-based	No	30/03/2017	1	0	0
2015400001	656	district/faith-based	No	30/03/2017	1	0	0
2015400002	656	district/faith-based	No	30/03/2017	1	0	0
2015415256	656	district/faith-based	No	30/03/2017	1	0	0
2014368020	656	district/faith-based	No	30/03/2017	1	0	0
2015369389	656	district/faith-based	No	30/03/2017	1	0	0
2011203831	656	district/faith-based	No	30/03/2017	1	0	0
2014302843	656	district/faith-based	No	30/03/2017	1	0	0
2015415255	656	district/faith-based	No	30/03/2017	1	0	0
2012253924	656	district/faith-based	No	30/03/2017	1	0	0
2012275985	656	district/faith-based	No	30/03/2017	1	0	0
2015334725	656	district/faith-based	No	30/03/2017	1	0	0
2015415254	656	district/faith-based	No	30/03/2017	1	0	0
2015383701	656	district/faith-based	No	30/03/2017	1	0	0
2014298038	656	district/faith-based	No	30/03/2017	1	0	0
2015375409	656	district/faith-based	No	30/03/2017	1	0	0
2014337045	656	district/faith-based	No	30/03/2017	1	0	0
2015375408	656	district/faith-based	No	30/03/2017	1	0	0
2015297088	656	district/faith-based	No	30/03/2017	1	0	0
2013247712	656	district/faith-based	No	30/03/2017	1	0	0
2015331890	656	district/faith-based	No	30/03/2017	1	0	0
2015297086	656	district/faith-based	No	30/03/2017	1	0	0
2015334724	656	district/faith-based	No	30/03/2017	1	0	0
2013247711	656	district/faith-based	No	30/03/2017	1	0	0
2012253966	656	district/faith-based	No	30/03/2017	1	0	0
2015368271	656	district/faith-based	No	30/03/2017	1	0	0
2015377874	656	district/faith-based	No	30/03/2017	1	0	0
2015331891	656	district/faith-based	No	30/03/2017	1	0	0
2013262224	656	district/faith-based	No	30/03/2017	1	0	0
2015326563	656	district/faith-based	No	30/03/2017	1	0	0
2014382913	656	district/faith-based	No	30/03/2017	1	0	0
2011193270	633	district/faith-based	No	30/03/2017	1	0	0
2015326562	633	district/faith-based	No	30/03/2017	1	0	0
2015326561	633	district/faith-based	No	30/03/2017	1	0	0
2015326560	633	district/faith-based	No	30/03/2017	1	0	0
2015402541	633	district/faith-based	No	30/03/2017	1	0	0
2015326559	633	district/faith-based	No	30/03/2017	1	0	0
2013274245	633	district/faith-based	No	30/03/2017	1	0	0
2012294467	633	district/faith-based	No	30/03/2017	1	0	0
2013274244	633	district/faith-based	No	30/03/2017	1	0	0
2012294466	633	district/faith-based	No	30/03/2017	1	0	0
2013262222	633	district/faith-based	No	30/03/2017	1	0	0
2013270347	633	district/faith-based	No	30/03/2017	1	0	0
2013262221	633	district/faith-based	No	30/03/2017	1	0	0
2013262225	633	district/faith-based	No	30/03/2017	1	0	0
2015368273	633	district/faith-based	No	30/03/2017	1	0	0
2015331666	633	district/faith-based	No	30/03/2017	1	0	0
2013247710	633	district/faith-based	No	30/03/2017	1	0	0
2015368272	633	district/faith-based	No	30/03/2017	1	0	0
2013247709	633	district/faith-based	No	30/03/2017	1	0	0
2015368270	633	district/faith-based	No	30/03/2017	1	0	0
2013247708	633	district/faith-based	No	30/03/2017	1	0	0
2015297456	633	district/faith-based	No	30/03/2017	1	0	0
2013247707	633	district/faith-based	No	30/03/2017	1	0	0
2015358170	633	district/faith-based	No	30/03/2017	1	0	0
2015303369	633	district/faith-based	No	30/03/2017	1	0	0
2012341042	633	district/faith-based	No	30/03/2017	1	0	0
2015321758	633	district/faith-based	No	30/03/2017	1	0	0
2015375209	633	district/faith-based	No	30/03/2017	1	0	0
2015297455	633	district/faith-based	No	30/03/2017	1	0	0
2014312270	633	district/faith-based	No	30/03/2017	1	0	0
2012253925	633	district/faith-based	No	30/03/2017	1	0	0
2015412112	633	district/faith-based	No	30/03/2017	1	0	0
2015358164	633	district/faith-based	No	30/03/2017	1	0	0
2014288006	633	district/faith-based	No	30/03/2017	1	0	0
2012269013	633	district/faith-based	No	30/03/2017	1	0	0
2015358163	633	district/faith-based	No	30/03/2017	1	0	0
2015397862	633	district/faith-based	No	30/03/2017	1	0	0
2014356972	633	district/faith-based	No	30/03/2017	1	0	0
2015397864	633	district/faith-based	No	30/03/2017	1	0	0
2015397863	633	district/faith-based	No	30/03/2017	1	0	0
2015376121	633	district/faith-based	No	30/03/2017	1	0	0
2015383703	633	district/faith-based	No	30/03/2017	1	0	0
2015376122	633	district/faith-based	No	30/03/2017	1	0	0
2015331893	633	district/faith-based	No	30/03/2017	1	0	0
2015297457	633	district/faith-based	No	30/03/2017	1	0	0
2015376123	633	district/faith-based	No	30/03/2017	1	0	0
2015383705	633	district/faith-based	No	30/03/2017	1	0	0

2014356971	633	district/faith-based	No	30/03/2017	1	0	0
2015340959	633	district/faith-based	No	30/03/2017	1	0	0
2015331892	633	district/faith-based	No	30/03/2017	1	0	0
2015377873	633	district/faith-based	No	30/03/2017	1	0	0
2015340960	633	district/faith-based	No	30/03/2017	1	0	0
2015377872	633	district/faith-based	No	30/03/2017	1	0	0
2014287897	633	district/faith-based	No	30/03/2017	1	0	0
2015340961	633	district/faith-based	No	30/03/2017	1	0	0
2012253923	633	district/faith-based	No	30/03/2017	1	0	0
2015355381	633	district/faith-based	No	30/03/2017	1	0	0
2014298581	633	district/faith-based	No	30/03/2017	1	0	0
2012344077	633	district/faith-based	No	30/03/2017	1	0	0
2015375980	633	district/faith-based	No	30/03/2017	1	0	0
2015355387	633	district/faith-based	No	30/03/2017	1	0	0
2015358167	633	district/faith-based	No	30/03/2017	1	0	0
2015355389	633	district/faith-based	No	30/03/2017	1	0	0
2015358168	633	district/faith-based	No	30/03/2017	1	0	0
2015355388	633	district/faith-based	No	30/03/2017	1	0	0
2015358166	633	district/faith-based	No	30/03/2017	1	0	0
2015358169	633	district/faith-based	No	30/03/2017	1	0	0
2015375978	633	district/faith-based	No	30/03/2017	1	0	0
2015358165	633	district/faith-based	No	30/03/2017	1	0	0
2011190739	633	district/faith-based	No	30/03/2017	1	0	0
2014302518	633	district/faith-based	No	30/03/2017	1	0	0
2015375979	633	district/faith-based	No	30/03/2017	1	0	0
2014382291	633	district/faith-based	No	30/03/2017	1	0	0
2015338458	633	district/faith-based	No	30/03/2017	1	0	0
2015335375	633	district/faith-based	No	30/03/2017	1	0	0
2015362503	633	district/faith-based	No	30/03/2017	1	0	0
2014382293	633	district/faith-based	No	30/03/2017	1	0	0
2015402367	633	district/faith-based	No	30/03/2017	1	0	0
2015377045	633	district/faith-based	No	30/03/2017	1	0	0
2014382292	633	district/faith-based	No	30/03/2017	1	0	0
2015495651	633	district/faith-based	No	30/03/2017	1	0	0
2014301462	633	district/faith-based	No	30/03/2017	1	0	0
2015402368	633	district/faith-based	No	30/03/2017	1	0	0
2015377040	633	district/faith-based	No	30/03/2017	1	0	0
2014301461	633	district/faith-based	No	30/03/2017	1	0	0
2015362502	633	district/faith-based	No	30/03/2017	1	0	0
2015377044	633	district/faith-based	No	30/03/2017	1	0	0
2015383704	633	district/faith-based	No	30/03/2017	1	0	0
2015402369	633	district/faith-based	No	30/03/2017	1	0	0
2012359918	633	district/faith-based	No	30/03/2017	1	0	0
2015377166	633	district/faith-based	No	30/03/2017	1	0	0
2015383702	633	district/faith-based	No	30/03/2017	1	0	0
2015297380	633	district/faith-based	No	30/03/2017	1	0	0
2012359919	633	district/faith-based	No	30/03/2017	1	0	0
2015377043	633	district/faith-based	No	30/03/2017	1	0	0
2012359917	633	district/faith-based	No	30/03/2017	1	0	0
2015297378	633	district/faith-based	No	30/03/2017	1	0	0
2011155227	633	district/faith-based	No	30/03/2017	1	0	0
2012359916	633	district/faith-based	No	30/03/2017	1	0	0
2011232694	633	district/faith-based	No	30/03/2017	1	0	0
2011155226	633	district/faith-based	No	30/03/2017	1	0	0
2012359920	633	district/faith-based	No	30/03/2017	1	0	0
2011232693	633	district/faith-based	No	30/03/2017	1	0	0
2011235378	633	district/faith-based	No	30/03/2017	1	0	0
2011232692	633	district/faith-based	No	30/03/2017	1	0	0
2011232691	633	district/faith-based	No	30/03/2017	1	0	0
2011232690	633	district/faith-based	No	30/03/2017	1	0	0
2015377165	633	district/faith-based	No	30/03/2017	1	0	0
2011232689	544	rural/clinic	No	30/03/2017	0	0	0
2011155225	544	rural/clinic	No	30/03/2017	0	0	0
2014307324	544	rural/clinic	No	30/03/2017	0	0	0
2015402531	544	rural/clinic	No	30/03/2017	0	0	0
2015340841	544	rural/clinic	No	30/03/2017	0	0	0
2013272688	544	rural/clinic	No	30/03/2017	0	0	0
2015340842	544	rural/clinic	No	30/03/2017	0	0	0
2015402532	544	rural/clinic	No	30/03/2017	0	0	0
2014321522	544	rural/clinic	No	30/03/2017	0	0	0
2012252690	544	rural/clinic	No	30/03/2017	0	0	0
2013272686	544	rural/clinic	No	30/03/2017	0	0	0
2015402533	544	rural/clinic	No	30/03/2017	0	0	0
2012252689	544	rural/clinic	No	30/03/2017	0	0	0
2013272687	544	rural/clinic	No	30/03/2017	0	0	0
2015340843	544	rural/clinic	No	30/03/2017	0	0	0
2015377168	719	rural/clinic	No	30/03/2017	0	0	0
2015402534	719	rural/clinic	No	30/03/2017	0	0	0
2014317430	719	rural/clinic	No	30/03/2017	0	0	0
2015340844	719	rural/clinic	No	30/03/2017	0	0	0
2015377041	719	rural/clinic	No	30/03/2017	0	0	0
2015402535	719	rural/clinic	No	30/03/2017	0	0	0

2014317431	719	rural/clinic	No	30/03/2017	0	0	0
201043356	719	rural/clinic	No	30/03/2017	0	0	0
2012391000	719	rural/clinic	No	30/03/2017	0	0	0
2014377623	719	rural/clinic	No	30/03/2017	0	0	0
2015402537	719	rural/clinic	No	30/03/2017	0	0	0
2015331173	719	rural/clinic	No	30/03/2017	0	0	0
2015402538	719	rural/clinic	No	30/03/2017	0	0	0
2015340201	719	rural/clinic	No	30/03/2017	0	0	0
2011192151	719	rural/clinic	No	30/03/2017	0	0	0
2015402539	719	rural/clinic	No	30/03/2017	0	0	0
2014347732	719	rural/clinic	No	30/03/2017	0	0	0
2015358518	719	rural/clinic	No	30/03/2017	0	0	0
2015402540	719	rural/clinic	No	30/03/2017	0	0	0
2015385520	719	rural/clinic	No	30/03/2017	0	0	0
2015358517	719	rural/clinic	No	30/03/2017	0	0	0
2015402542	719	rural/clinic	No	30/03/2017	0	0	0
2015358516	719	rural/clinic	No	30/03/2017	0	0	0
2015402543	719	rural/clinic	No	30/03/2017	0	0	0
2015331174	719	rural/clinic	No	30/03/2017	0	0	0
2015300682	719	rural/clinic	No	30/03/2017	0	0	0
2015386382	719	rural/clinic	No	30/03/2017	0	0	0
2015358519	719	rural/clinic	No	30/03/2017	0	0	0
2014287974	719	rural/clinic	No	30/03/2017	0	0	0
2015386379	719	rural/clinic	No	30/03/2017	0	0	0
2015335376	719	rural/clinic	No	30/03/2017	0	0	0
2015358515	719	rural/clinic	No	30/03/2017	0	0	0
2012350625	719	rural/clinic	No	30/03/2017	0	0	0
2015331175	719	rural/clinic	No	30/03/2017	0	0	0
2015377035	262	rural/clinic	No	30/03/2017	0	0	0
2015386380	262	rural/clinic	No	30/03/2017	0	0	0
2015331177	262	rural/clinic	No	30/03/2017	0	0	0
2015358513	262	rural/clinic	No	30/03/2017	0	0	0
2015331176	262	rural/clinic	No	30/03/2017	0	0	0
2015377167	262	rural/clinic	No	30/03/2017	0	0	0
2015358512	262	rural/clinic	No	30/03/2017	0	0	0
2015377038	262	rural/clinic	No	30/03/2017	0	0	0
2015386381	262	rural/clinic	No	30/03/2017	0	0	0
2014287973	142	rural/clinic	No	30/03/2017	0	0	0
2015286287	142	rural/clinic	No	30/03/2017	0	0	0
2012290696	142	rural/clinic	No	30/03/2017	0	0	0
2015386384	142	rural/clinic	No	30/03/2017	0	0	0
2012290697	142	rural/clinic	No	30/03/2017	0	0	0
2015377039	142	rural/clinic	No	30/03/2017	0	0	0
2015286286	142	rural/clinic	No	30/03/2017	0	0	0
2012290698	142	rural/clinic	No	30/03/2017	0	0	0
2013273642	142	rural/clinic	No	30/03/2017	0	0	0
2015386377	186	rural/clinic	No	30/03/2017	0	0	0
2015286288	186	rural/clinic	No	30/03/2017	0	0	0
2015386385	186	rural/clinic	No	30/03/2017	0	0	0
2015386387	186	rural/clinic	No	30/03/2017	0	0	0
2015286290	186	rural/clinic	No	30/03/2017	0	0	0
2015386386	186	rural/clinic	No	30/03/2017	0	0	0
2014319500	186	rural/clinic	No	30/03/2017	0	0	0
2015386373	186	rural/clinic	No	30/03/2017	0	0	0
2015286289	186	rural/clinic	No	30/03/2017	0	0	0
2015386374	186	rural/clinic	No	30/03/2017	0	0	0
2015386378	186	rural/clinic	No	30/03/2017	0	0	0
2015386376	186	rural/clinic	No	30/03/2017	0	0	0
2015378950	186	rural/clinic	No	30/03/2017	0	0	0
2015286291	186	rural/clinic	No	30/03/2017	0	0	0
2015386367	186	rural/clinic	No	30/03/2017	0	0	0
2015362804	186	rural/clinic	No	30/03/2017	0	0	0
2015386375	186	rural/clinic	No	30/03/2017	0	0	0
2014372270	186	rural/clinic	No	30/03/2017	0	0	0
2014335235	186	rural/clinic	No	30/03/2017	0	0	0
2014335233	186	rural/clinic	No	30/03/2017	0	0	0
2015369684	186	rural/clinic	No	30/03/2017	0	0	0
2015360507	186	rural/clinic	No	30/03/2017	0	0	0
2014335234	186	rural/clinic	No	30/03/2017	0	0	0
2014335232	186	rural/clinic	No	30/03/2017	0	0	0
2015360506	186	rural/clinic	No	30/03/2017	0	0	0
2015360505	186	rural/clinic	No	30/03/2017	0	0	0
2015369682	186	rural/clinic	No	30/03/2017	0	0	0
2015297089	186	rural/clinic	No	30/03/2017	0	0	0
2015352449	186	rural/clinic	No	30/03/2017	0	0	0
2015352450	186	rural/clinic	No	30/03/2017	0	0	0
2015377037	186	rural/clinic	No	30/03/2017	0	0	0
2015377042	186	rural/clinic	No	30/03/2017	0	0	0
2015352445	186	rural/clinic	No	30/03/2017	0	0	0
2015309191	186	rural/clinic	No	30/03/2017	0	0	0
2015352447	186	rural/clinic	No	30/03/2017	0	0	0
2014372725	186	rural/clinic	No	30/03/2017	0	0	0

2014322627	186	rural/clinic	No	30/03/2017	0	0	0
2014383970	186	rural/clinic	No	30/03/2017	0	0	0
2011133615	186	rural/clinic	No	30/03/2017	0	0	0
2015332015	186	rural/clinic	No	30/03/2017	0	0	0
2015351617	186	rural/clinic	No	30/03/2017	0	0	0
2015332014	186	rural/clinic	No	30/03/2017	0	0	0
2012265919	186	rural/clinic	No	30/03/2017	0	0	0
2015332016	186	rural/clinic	No	30/03/2017	0	0	0
2014346714	186	rural/clinic	No	30/03/2017	0	0	0
2014346710	186	rural/clinic	No	30/03/2017	0	0	0
2014335525	186	rural/clinic	No	30/03/2017	0	0	0
2014346713	186	rural/clinic	No	30/03/2017	0	0	0
2014346709	186	rural/clinic	No	30/03/2017	0	0	0
2015352448	186	rural/clinic	No	30/03/2017	0	0	0
2014346711	634	rural/clinic	No	30/03/2017	0	0	0
2015332907	634	rural/clinic	No	30/03/2017	0	0	0
2014346704	634	rural/clinic	No	30/03/2017	0	0	0
2014346705	634	rural/clinic	No	30/03/2017	0	0	0
2015352446	634	rural/clinic	No	30/03/2017	0	0	0
2014346706	634	rural/clinic	No	30/03/2017	0	0	0
2014346707	634	rural/clinic	No	30/03/2017	0	0	0
2014383968	634	rural/clinic	No	30/03/2017	0	0	0
2015406252	634	rural/clinic	No	30/03/2017	0	0	0
2015408169	634	rural/clinic	No	30/03/2017	0	0	0
2015406254	634	rural/clinic	No	30/03/2017	0	0	0
2015408170	634	rural/clinic	No	30/03/2017	0	0	0
2015406256	634	rural/clinic	No	30/03/2017	0	0	0
2014346313	634	rural/clinic	No	30/03/2017	0	0	0
2015406253	634	rural/clinic	No	30/03/2017	0	0	0
2015406251	634	rural/clinic	No	30/03/2017	0	0	0
2012384787	634	rural/clinic	No	30/03/2017	0	0	0
2014383969	634	rural/clinic	No	30/03/2017	0	0	0
2012384786	634	rural/clinic	No	30/03/2017	0	0	0
2014346314	634	rural/clinic	No	30/03/2017	0	0	0
2012384785	300	rural/clinic	No	30/03/2017	0	0	0
2012370845	300	rural/clinic	No	30/03/2017	0	0	0
	300	rural/clinic	No	28/03/2017	0	0	0
	300	rural/clinic	No	28/03/2017	0	0	0
	300	rural/clinic	No	28/03/2017	0	0	0
	300	rural/clinic	No	28/03/2017	0	0	0
	300	rural/clinic	No	28/03/2017	0	0	0
	300	rural/clinic	No	28/03/2017	0	0	0
	300	rural/clinic	No	28/03/2017	0	0	0
	300	rural/clinic	No	28/03/2017	0	0	0
	300	rural/clinic	No	28/03/2017	0	0	0
	300	rural/clinic	No	28/03/2017	0	0	0
	300	rural/clinic	No	28/03/2017	0	0	0
	300	rural/clinic	No	28/03/2017	0	0	0
	300	rural/clinic	No	28/03/2017	0	0	0
	300	rural/clinic	No	28/03/2017	0	0	0
	300	rural/clinic	No	28/03/2017	0	0	0
	300	rural/clinic	No	28/03/2017	0	0	0
	300	rural/clinic	No	28/03/2017	0	0	0
	300	rural/clinic	No	28/03/2017	0	0	0
	327	rural/clinic	No	28/03/2017	0	0	0
	327	rural/clinic	No	28/03/2017	0	0	0
	327	rural/clinic	No	28/03/2017	0	0	0
	327	rural/clinic	No	28/03/2017	0	0	0
	327	rural/clinic	No	28/03/2017	0	0	0
	327	rural/clinic	No	28/03/2017	0	0	0
	327	rural/clinic	No	28/03/2017	0	0	0
	327	rural/clinic	No	28/03/2017	0	0	0
	327	rural/clinic	No	28/03/2017	0	0	0
	327	rural/clinic	No	28/03/2017	0	0	0
	327	rural/clinic	No	28/03/2017	0	0	0
	327	rural/clinic	No	28/03/2017	0	0	0
	327	rural/clinic	No	28/03/2017	0	0	0
	327	rural/clinic	No	28/03/2017	0	0	0
	327	rural/clinic	No	28/03/2017	0	0	0
	91	rural/clinic	No	28/03/2017	0	0	0
	91	rural/clinic	No	28/03/2017	0	0	0
	91	rural/clinic	No	28/03/2017	0	0	0
	91	rural/clinic	No	28/03/2017	0	0	0
	91	rural/clinic	No	28/03/2017	0	0	0
	91	rural/clinic	No	28/03/2017	0	0	0
	91	rural/clinic	No	28/03/2017	0	0	0
	91	rural/clinic	No	28/03/2017	0	0	0
	263	rural/clinic	No	28/03/2017	0	0	0
	263	rural/clinic	No	28/03/2017	0	0	0
	263	rural/clinic	No	28/03/2017	0	0	0
	263	rural/clinic	No	28/03/2017	0	0	0
	263	rural/clinic	No	28/03/2017	0	0	0

263	rural/clinic	No	28/03/2017	0	0	0
263	rural/clinic	No	28/03/2017	0	0	0
263	rural/clinic	No	28/03/2017	0	0	0
263	rural/clinic	No	28/03/2017	0	0	0
263	rural/clinic	No	28/03/2017	0	0	0
263	rural/clinic	No	28/03/2017	0	0	0
263	rural/clinic	No	28/03/2017	0	0	0
263	rural/clinic	No	28/03/2017	0	0	0
263	rural/clinic	No	28/03/2017	0	0	0
263	rural/clinic	No	28/03/2017	0	0	0
263	rural/clinic	No	28/03/2017	0	0	0
263	rural/clinic	No	28/03/2017	0	0	0
263	rural/clinic	No	28/03/2017	0	0	0
263	rural/clinic	No	28/03/2017	0	0	0
263	rural/clinic	No	28/03/2017	0	0	0
263	rural/clinic	No	28/03/2017	0	0	0
263	rural/clinic	No	28/03/2017	0	0	0
263	rural/clinic	No	28/03/2017	0	0	0
263	rural/clinic	No	28/03/2017	0	0	0
263	rural/clinic	No	28/03/2017	0	0	0
263	rural/clinic	No	28/03/2017	0	0	0
263	rural/clinic	No	28/03/2017	0	0	0
263	rural/clinic	No	28/03/2017	0	0	0
263	rural/clinic	No	28/03/2017	0	0	0
263	rural/clinic	No	28/03/2017	0	0	0
263	rural/clinic	No	28/03/2017	0	0	0
263	rural/clinic	No	28/03/2017	0	0	0
263	rural/clinic	No	28/03/2017	0	0	0
263	rural/clinic	No	28/03/2017	0	0	0
263	rural/clinic	No	28/03/2017	0	0	0
263	rural/clinic	No	28/03/2017	0	0	0
263	rural/clinic	No	28/03/2017	0	0	0
263	rural/clinic	No	28/03/2017	0	0	0
263	rural/clinic	No	28/03/2017	0	0	0
263	rural/clinic	No	28/03/2017	0	0	0
263	rural/clinic	No	28/03/2017	0	0	0
263	rural/clinic	No	28/03/2017	0	0	0
263	rural/clinic	No	28/03/2017	0	0	0
263	rural/clinic	No	28/03/2017	0	0	0
263	rural/clinic	No	28/03/2017	0	0	0
264	rural/clinic	No	28/03/2017	0	0	0
264	rural/clinic	No	28/03/2017	0	0	0
264	rural/clinic	No	28/03/2017	0	0	0
264	rural/clinic	No	28/03/2017	0	0	0
264	rural/clinic	No	28/03/2017	0	0	0
264	rural/clinic	No	28/03/2017	0	0	0
264	rural/clinic	No	28/03/2017	0	0	0
264	rural/clinic	No	28/03/2017	0	0	0
264	rural/clinic	No	28/03/2017	0	0	0
264	rural/clinic	No	28/03/2017	0	0	0
264	rural/clinic	No	28/03/2017	0	0	0
264	rural/clinic	No	28/03/2017	0	0	0
264	rural/clinic	No	28/03/2017	0	0	0
264	rural/clinic	No	28/03/2017	0	0	0
264	rural/clinic	No	28/03/2017	0	0	0
301	rural/clinic	No	28/03/2017	0	0	0
301	rural/clinic	No	28/03/2017	0	0	0
301	rural/clinic	No	28/03/2017	0	0	0
301	rural/clinic	No	28/03/2017	0	0	0
301	rural/clinic	No	28/03/2017	0	0	0
301	rural/clinic	No	28/03/2017	0	0	0
301	rural/clinic	No	28/03/2017	0	0	0
301	rural/clinic	No	28/03/2017	0	0	0
301	rural/clinic	No	28/03/2017	0	0	0
301	rural/clinic	No	28/03/2017	0	0	0
301	rural/clinic	No	28/03/2017	0	0	0
301	rural/clinic	No	28/03/2017	0	0	0
301	rural/clinic	No	28/03/2017	0	0	0
301	rural/clinic	No	28/03/2017	0	0	0
301	rural/clinic	No	28/03/2017	0	0	0
301	rural/clinic	No	28/03/2017	0	0	0
301	rural/clinic	No	28/03/2017	0	0	0
301	rural/clinic	No	28/03/2017	0	0	0
301	rural/clinic	No	28/03/2017	0	0	0
301	rural/clinic	No	28/03/2017	0	0	0
301	rural/clinic	No	28/03/2017	0	0	0
301	rural/clinic	No	28/03/2017	0	0	0
500	rural/clinic	No	28/03/2017	0	0	0
500	rural/clinic	No	28/03/2017	0	0	0
500	rural/clinic	No	28/03/2017	0	0	0
500	rural/clinic	No	28/03/2017	0	0	0
500	rural/clinic	No	28/03/2017	0	0	0

500	rural/clinic	No	28/03/2017	0	0	0
500	rural/clinic	No	28/03/2017	0	0	0
500	rural/clinic	No	28/03/2017	0	0	0
500	rural/clinic	No	28/03/2017	0	0	0
500	rural/clinic	No	28/03/2017	0	0	0
500	rural/clinic	No	28/03/2017	0	0	0
500	rural/clinic	No	28/03/2017	0	0	0
500	rural/clinic	No	28/03/2017	0	0	0
500	rural/clinic	No	28/03/2017	0	0	0
500	rural/clinic	No	28/03/2017	0	0	0
500	rural/clinic	No	28/03/2017	0	0	0
500	rural/clinic	No	28/03/2017	0	0	0
500	rural/clinic	No	28/03/2017	0	0	0
500	rural/clinic	No	28/03/2017	0	0	0
500	rural/clinic	No	28/03/2017	0	0	0
500	rural/clinic	No	28/03/2017	0	0	0
500	rural/clinic	No	28/03/2017	0	0	0
500	rural/clinic	No	28/03/2017	0	0	0
11	rural/clinic	No	28/03/2017	0	0	0
11	rural/clinic	No	28/03/2017	0	0	0
11	rural/clinic	No	28/03/2017	0	0	0
11	rural/clinic	No	28/03/2017	0	0	0
11	rural/clinic	No	28/03/2017	0	0	0
11	rural/clinic	No	28/03/2017	0	0	0
11	rural/clinic	No	28/03/2017	0	0	0
11	rural/clinic	No	28/03/2017	0	0	0
11	rural/clinic	No	28/03/2017	0	0	0
11	rural/clinic	No	28/03/2017	0	0	0
11	rural/clinic	No	28/03/2017	0	0	0
11	rural/clinic	No	28/03/2017	0	0	0
11	rural/clinic	No	28/03/2017	0	0	0
11	rural/clinic	No	28/03/2017	0	0	0
11	rural/clinic	No	28/03/2017	0	0	0
11	rural/clinic	No	28/03/2017	0	0	0
11	rural/clinic	No	28/03/2017	0	0	0
11	rural/clinic	No	28/03/2017	0	0	0
11	rural/clinic	No	28/03/2017	0	0	0
11	rural/clinic	No	28/03/2017	0	0	0
11	rural/clinic	No	28/03/2017	0	0	0
11	rural/clinic	No	28/03/2017	0	0	0
11	rural/clinic	No	28/03/2017	0	0	0
11	rural/clinic	No	28/03/2017	0	0	0
11	rural/clinic	No	28/03/2017	0	0	0
11	rural/clinic	No	28/03/2017	0	0	0
11	rural/clinic	No	28/03/2017	0	0	0
11	rural/clinic	No	28/03/2017	0	0	0
11	rural/clinic	No	28/03/2017	0	0	0
11	rural/clinic	No	28/03/2017	0	0	0
11	rural/clinic	No	28/03/2017	0	0	0
11	rural/clinic	No	28/03/2017	0	0	0
11	rural/clinic	No	28/03/2017	0	0	0
11	rural/clinic	No	28/03/2017	0	0	0
11	rural/clinic	No	28/03/2017	0	0	0
11	rural/clinic	No	28/03/2017	0	0	0
11	rural/clinic	No	28/03/2017	0	0	0
11	rural/clinic	No	28/03/2017	0	0	0
11	rural/clinic	No	28/03/2017	0	0	0
11	rural/clinic	No	28/03/2017	0	0	0
11	rural/clinic	No	28/03/2017	0	0	0
11	rural/clinic	No	28/03/2017	0	0	0
11	rural/clinic	No	28/03/2017	0	0	0
11	rural/clinic	No	28/03/2017	0	0	0
812	rural/clinic	No	28/03/2017	0	0	0
812	rural/clinic	No	28/03/2017	0	0	0
812	rural/clinic	No	28/03/2017	0	0	0
812	rural/clinic	No	28/03/2017	0	0	0
812	rural/clinic	No	28/03/2017	0	0	0
812	rural/clinic	No	28/03/2017	0	0	0
812	rural/clinic	No	28/03/2017	0	0	0
812	rural/clinic	No	28/03/2017	0	0	0
812	rural/clinic	No	28/03/2017	0	0	0
812	rural/clinic	No	28/03/2017	0	0	0
812	rural/clinic	No	28/03/2017	0	0	0
812	rural/clinic	No	28/03/2017	0	0	0
812	rural/clinic	No	28/03/2017	0	0	0
812	rural/clinic	No	28/03/2017	0	0	0
812	rural/clinic	No	28/03/2017	0	0	0
812	rural/clinic	No	28/03/2017	0	0	0
812	rural/clinic	No	28/03/2017	0	0	0
812	rural/clinic	No	28/03/2017	0	0	0
812	rural/clinic	No	28/03/2017	0	0	0
812	rural/clinic	No	28/03/2017	0	0	0

812	rural/clinic	No	28/03/2017	0	0	0
812	rural/clinic	No	28/03/2017	0	0	0
812	rural/clinic	No	28/03/2017	0	0	0
812	rural/clinic	No	28/03/2017	0	0	0
812	rural/clinic	No	28/03/2017	0	0	0
812	rural/clinic	No	28/03/2017	0	0	0
812	rural/clinic	No	28/03/2017	0	0	0
812	rural/clinic	No	28/03/2017	0	0	0
812	rural/clinic	No	28/03/2017	0	0	0
812	rural/clinic	No	28/03/2017	0	0	0
812	rural/clinic	No	28/03/2017	0	0	0
812	rural/clinic	No	28/03/2017	0	0	0
812	rural/clinic	No	28/03/2017	0	0	0
812	rural/clinic	No	28/03/2017	0	0	0
812	rural/clinic	No	28/03/2017	0	0	0
812	rural/clinic	No	28/03/2017	0	0	0
812	rural/clinic	No	28/03/2017	0	0	0
501	rural/clinic	No	28/03/2017	0	0	0
501	rural/clinic	No	28/03/2017	0	0	0
501	rural/clinic	No	28/03/2017	0	0	0
501	rural/clinic	No	28/03/2017	0	0	0
501	rural/clinic	No	28/03/2017	0	0	0
501	rural/clinic	No	28/03/2017	0	0	0
501	rural/clinic	No	28/03/2017	0	0	0
501	rural/clinic	No	28/03/2017	0	0	0
501	rural/clinic	No	28/03/2017	0	0	0
501	rural/clinic	No	28/03/2017	0	0	0
501	rural/clinic	No	28/03/2017	0	0	0
501	rural/clinic	No	28/03/2017	0	0	0
501	rural/clinic	No	28/03/2017	0	0	0
501	rural/clinic	No	28/03/2017	0	0	0
501	rural/clinic	No	28/03/2017	0	0	0
501	rural/clinic	No	28/03/2017	0	0	0
635	rural/clinic	No	28/03/2017	0	0	0
635	rural/clinic	No	28/03/2017	0	0	0
635	rural/clinic	No	28/03/2017	0	0	0
635	rural/clinic	No	28/03/2017	0	0	0
635	rural/clinic	No	28/03/2017	0	0	0
635	rural/clinic	No	28/03/2017	0	0	0
635	rural/clinic	No	28/03/2017	0	0	0
635	rural/clinic	No	28/03/2017	0	0	0
635	rural/clinic	No	28/03/2017	0	0	0
635	rural/clinic	No	28/03/2017	0	0	0
635	rural/clinic	No	28/03/2017	0	0	0
635	rural/clinic	No	28/03/2017	0	0	0
691	rural/clinic	No	28/03/2017	0	0	0
691	rural/clinic	No	28/03/2017	0	0	0
691	rural/clinic	No	28/03/2017	0	0	0
691	rural/clinic	No	28/03/2017	0	0	0
691	rural/clinic	No	28/03/2017	0	0	0
691	rural/clinic	No	28/03/2017	0	0	0
691	rural/clinic	No	28/03/2017	0	0	0
691	rural/clinic	No	28/03/2017	0	0	0
691	rural/clinic	No	28/03/2017	0	0	0
691	rural/clinic	No	28/03/2017	0	0	0
691	rural/clinic	No	28/03/2017	0	0	0
691	rural/clinic	No	28/03/2017	0	0	0
691	rural/clinic	No	28/03/2017	0	0	0
691	rural/clinic	No	28/03/2017	0	0	0
691	rural/clinic	No	28/03/2017	0	0	0
691	rural/clinic	No	28/03/2017	0	0	0
691	rural/clinic	No	28/03/2017	0	0	0
691	rural/clinic	No	28/03/2017	0	0	0
793	rural/clinic	No	28/03/2017	0	0	0
793	rural/clinic	No	28/03/2017	0	0	0
793	rural/clinic	No	28/03/2017	0	0	0
793	rural/clinic	No	28/03/2017	0	0	0
793	rural/clinic	No	28/03/2017	0	0	0
793	rural/clinic	No	28/03/2017	0	0	0
793	rural/clinic	No	28/03/2017	0	0	0
793	rural/clinic	No	28/03/2017	0	0	0
793	rural/clinic	No	28/03/2017	0	0	0
793	rural/clinic	No	28/03/2017	0	0	0
793	rural/clinic	No	28/03/2017	0	0	0
793	rural/clinic	No	28/03/2017	0	0	0
793	rural/clinic	No	28/03/2017	0	0	0
793	rural/clinic	No	28/03/2017	0	0	0
793	rural/clinic	No	28/03/2017	0	0	0
793	rural/clinic	No	28/03/2017	0	0	0
793	rural/clinic	No	28/03/2017	0	0	0
793	rural/clinic	No	28/03/2017	0	0	0
614	rural/clinic	No	28/03/2017	0	0	0

	614	rural/clinic	No	28/03/2017	0	0	0
	614	rural/clinic	No	28/03/2017	0	0	0
	614	rural/clinic	No	28/03/2017	0	0	0
	92	rural/clinic	No	28/03/2017	0	0	0
	92	rural/clinic	No	28/03/2017	0	0	0
	92	rural/clinic	No	28/03/2017	0	0	0
	92	rural/clinic	No	28/03/2017	0	0	0
	92	rural/clinic	No	28/03/2017	0	0	0
	92	rural/clinic	No	28/03/2017	0	0	0
	92	rural/clinic	No	28/03/2017	0	0	0
	92	rural/clinic	No	28/03/2017	0	0	0
	92	rural/clinic	No	28/03/2017	0	0	0
	92	rural/clinic	No	28/03/2017	0	0	0
	92	rural/clinic	No	28/03/2017	0	0	0
	92	rural/clinic	No	28/03/2017	0	0	0
	92	rural/clinic	No	28/03/2017	0	0	0
	92	rural/clinic	No	28/03/2017	0	0	0
	92	rural/clinic	No	28/03/2017	0	0	0
	92	rural/clinic	No	28/03/2017	0	0	0
	92	rural/clinic	No	28/03/2017	0	0	0
	92	rural/clinic	No	28/03/2017	0	0	0
	92	rural/clinic	No	28/03/2017	0	0	0
	92	rural/clinic	No	28/03/2017	0	0	0
	92	rural/clinic	No	28/03/2017	0	0	0
	92	rural/clinic	No	28/03/2017	0	0	0
	92	rural/clinic	No	28/03/2017	0	0	0
	92	rural/clinic	No	28/03/2017	0	0	0
	92	rural/clinic	No	28/03/2017	0	0	0
	92	rural/clinic	No	28/03/2017	0	0	0
	92	rural/clinic	No	28/03/2017	0	0	0
	92	rural/clinic	No	28/03/2017	0	0	0
	92	rural/clinic	No	28/03/2017	0	0	0
	92	rural/clinic	No	28/03/2017	0	0	0
	92	rural/clinic	No	28/03/2017	0	0	0
	92	rural/clinic	No	28/03/2017	0	0	0
	92	rural/clinic	No	28/03/2017	0	0	0
0	92	rural/clinic	No	28/03/2017	0	0	0
2015379044	92	rural/clinic	No	28/03/2017	0	0	0
2015379044	92	rural/clinic	No	28/03/2017	0	0	0
2015379033	92	rural/clinic	No	28/03/2017	0	0	0
2015379033	92	rural/clinic	No	28/03/2017	0	0	0
2012364937	92	rural/clinic	No	28/03/2017	0	0	0
2012364937	92	rural/clinic	No	28/03/2017	0	0	0
2015379045	92	rural/clinic	No	28/03/2017	0	0	0
2015379045	92	rural/clinic	No	28/03/2017	0	0	0
2015338710	92	rural/clinic	No	28/03/2017	0	0	0
2015338710	92	rural/clinic	No	28/03/2017	0	0	0
2014365380	92	rural/clinic	No	28/03/2017	0	0	0
2014365380	92	rural/clinic	No	28/03/2017	0	0	0
2014305940	92	rural/clinic	No	28/03/2017	0	0	0
2014305940	92	rural/clinic	No	28/03/2017	0	0	0
2014307323	92	rural/clinic	No	28/03/2017	0	0	0
2015397535	92	rural/clinic	No	28/03/2017	0	0	0
2015397535	92	rural/clinic	No	28/03/2017	0	0	0
2015413644	92	rural/clinic	No	28/03/2017	0	0	0
2015413644	92	rural/clinic	No	28/03/2017	0	0	0
2014288005	92	rural/clinic	No	28/03/2017	0	0	0
2014288005	92	rural/clinic	No	28/03/2017	0	0	0
2014346311	92	rural/clinic	No	30/03/2017	0	0	0
2015294047	92	rural/clinic	No	30/03/2017	0	0	0
2015294049	851	district/faith-based	No	30/03/2017	1	0	0
2015293833	851	district/faith-based	No	30/03/2017	1	0	0
2015334918	851	district/faith-based	No	30/03/2017	1	0	0
2015334919	851	district/faith-based	No	30/03/2017	1	0	0
2011135652	851	district/faith-based	No	30/03/2017	1	0	0
2015293649	851	district/faith-based	No	30/03/2017	1	0	0
2015344729	851	district/faith-based	No	30/03/2017	1	0	0
2015344728	851	district/faith-based	No	30/03/2017	1	0	0
2015334612	851	district/faith-based	No	30/03/2017	1	0	0
2015334613	851	district/faith-based	No	30/03/2017	1	0	0
2014372231	851	district/faith-based	No	30/03/2017	1	0	0
2014372232	851	district/faith-based	No	30/03/2017	1	0	0
2014372233	851	district/faith-based	No	30/03/2017	1	0	0
2015413987	851	district/faith-based	No	30/03/2017	1	0	0
2015375971	851	district/faith-based	No	09/03/2017	1	0	0
2011196010	851	district/faith-based	No	30/03/2017	1	0	0
2011196011	851	district/faith-based	No	30/03/2017	1	0	0
2011196012	851	district/faith-based	No	30/03/2017	1	0	0
2015413586	851	district/faith-based	No	30/03/2017	1	0	0
2015413588	851	district/faith-based	No	30/03/2017	1	0	0
2015413587	851	district/faith-based	No	30/03/2017	1	0	0
2015334917	851	district/faith-based	No	30/03/2017	1	0	0

2015334913	851	district/faith-based	No	30/03/2017	1	0	0
2015334914	851	district/faith-based	No	30/03/2017	1	0	0
2015334915	851	district/faith-based	No	30/03/2017	1	0	0
2015334916	851	district/faith-based	No	30/03/2017	1	0	0
2015334910	851	district/faith-based	No	30/03/2017	1	0	0
2015334909	851	district/faith-based	No	30/03/2017	1	0	0
2015334912	851	district/faith-based	No	30/03/2017	1	0	0
2015334911	851	district/faith-based	No	30/03/2017	1	0	0
2015344864	851	district/faith-based	No	30/03/2017	1	0	0
2015344866	851	district/faith-based	No	30/03/2017	1	0	0
2015344867	851	district/faith-based	No	30/03/2017	1	0	0
2015413585	851	district/faith-based	No	30/03/2017	1	0	0
2015334703	851	district/faith-based	No	30/03/2017	1	0	0
2015334704	851	district/faith-based	No	30/03/2017	1	0	0
2015334714	851	district/faith-based	No	30/03/2017	1	0	0
2015293834	851	district/faith-based	No	30/03/2017	1	0	0
2015413990	851	district/faith-based	No	30/03/2017	1	0	0
2015413989	851	district/faith-based	No	30/03/2017	1	0	0
2015413590	851	district/faith-based	No	30/03/2017	1	0	0
2015413589	851	district/faith-based	No	30/03/2017	1	0	0
2015413988	851	district/faith-based	No	30/03/2017	1	0	0
2015413991	851	district/faith-based	No	30/03/2017	1	0	0
2015344865	851	district/faith-based	No	30/03/2017	1	0	0
2015413894	851	district/faith-based	No	30/03/2017	1	0	0
2015362504	851	district/faith-based	No	30/03/2017	1	0	0
2015369390	851	district/faith-based	No	30/03/2017	1	0	0
2012344076	851	district/faith-based	No	30/03/2017	1	0	0
2014291870	851	district/faith-based	No	31/03/2017	1	0	0
2014291869	851	district/faith-based	No	31/03/2017	1	0	0
2014291868	851	district/faith-based	No	31/03/2017	1	0	0
2015377040	658	rural/clinic	No	30/03/2017	0	0	0
2015385521	658	rural/clinic	No	30/03/2017	0	0	0
2015385521	658	rural/clinic	No	30/03/2017	0	0	0
2015413123	658	rural/clinic	No	30/03/2017	0	0	0
2015413123	658	rural/clinic	No	30/03/2017	0	0	0
2015390303	658	rural/clinic	No	04/04/2017	0	0	0
2012284742	658	rural/clinic	No	04/04/2017	0	0	0
2014369045	658	rural/clinic	No	04/04/2017	0	0	0
2014334676	658	rural/clinic	No	04/04/2017	0	0	0
2012295975	658	rural/clinic	No	04/04/2017	0	0	0
2015349060	658	rural/clinic	No	04/04/2017	0	0	0
2012295976	658	rural/clinic	No	04/04/2017	0	0	0
2015400018	658	rural/clinic	No	04/04/2017	0	0	0
2014334346	658	rural/clinic	No	04/04/2017	0	0	0
2012359156	658	rural/clinic	No	04/04/2017	0	0	0
2012321332	658	rural/clinic	No	04/04/2017	0	0	0
2015325930	658	rural/clinic	No	04/04/2017	0	0	0
2014326630	658	rural/clinic	No	04/04/2017	0	0	0
2015302401	658	rural/clinic	No	04/04/2017	0	0	0
2015357400	658	rural/clinic	No	04/04/2017	0	0	0
2012388382	658	rural/clinic	No	04/04/2017	0	0	0
2014327116	658	rural/clinic	No	04/04/2017	0	0	0
2015357189	658	rural/clinic	No	04/04/2017	0	0	0
2015368783	658	rural/clinic	No	04/04/2017	0	0	0
2015357188	658	rural/clinic	No	04/04/2017	0	0	0
2014301463	658	rural/clinic	No	04/04/2017	0	0	0
2015325929	658	rural/clinic	No	04/04/2017	0	0	0
2013271839	658	rural/clinic	No	04/04/2017	0	0	0
2015414329	658	rural/clinic	No	04/04/2017	0	0	0
2015340069	658	rural/clinic	No	04/04/2017	0	0	0
2014342513	658	rural/clinic	No	04/04/2017	0	0	0
2015302402	658	rural/clinic	No	04/04/2017	0	0	0
2015357399	658	rural/clinic	No	04/04/2017	0	0	0
2014326628	658	rural/clinic	No	04/04/2017	0	0	0
2015340071	658	rural/clinic	No	04/04/2017	0	0	0
2015340070	658	rural/clinic	No	04/04/2017	0	0	0
2014327117	658	rural/clinic	No	04/04/2017	0	0	0
2014344363	658	rural/clinic	No	04/04/2017	0	0	0
2015357187	658	rural/clinic	No	04/04/2017	0	0	0
20170000009	658	rural/clinic	No	05/04/2017	0	0	0
2014360957	658	rural/clinic	No	04/04/2017	0	0	0
2015357186	895	rural/clinic	No	04/04/2017	0	0	0
2014360956	895	rural/clinic	No	04/04/2017	0	0	0
2015372916	895	rural/clinic	No	04/04/2017	0	0	0
2013325943	895	rural/clinic	No	04/04/2017	0	0	0
2017000010	895	rural/clinic	No	05/04/2017	0	0	0
2012244283	895	rural/clinic	No	04/04/2017	0	0	0
2015293466	895	rural/clinic	No	04/04/2017	0	0	0
2012246192	895	rural/clinic	No	04/04/2017	0	0	0
2013253944	895	rural/clinic	No	04/04/2017	0	0	0
2011144805	895	rural/clinic	No	04/04/2017	0	0	0
2012246193	895	rural/clinic	No	04/04/2017	0	0	0

2017000011	895	rural/clinic	No	05/04/2017	0	0	0
2015293465	895	rural/clinic	No	04/04/2017	0	0	0
2015299789	761	rural/clinic	No	04/04/2017	0	0	0
2014371498	761	rural/clinic	No	04/04/2017	0	0	0
2017000012	761	rural/clinic	No	05/04/2017	0	0	0
2012295977	761	rural/clinic	No	04/04/2017	0	0	0
2014316767	761	rural/clinic	No	04/04/2017	0	0	0
2015318250	761	rural/clinic	No	04/04/2017	0	0	0
2015364373	761	rural/clinic	No	04/04/2017	0	0	0
2014316766	761	rural/clinic	No	04/04/2017	0	0	0
2014346279	761	rural/clinic	No	04/04/2017	0	0	0
2015364371	761	rural/clinic	No	04/04/2017	0	0	0
2014316765	761	rural/clinic	No	04/04/2017	0	0	0
2015364370	761	rural/clinic	No	04/04/2017	0	0	0
2014314464	761	rural/clinic	No	04/04/2017	0	0	0
2011128281	576	rural/clinic	No	04/04/2017	0	0	0
2015364368	576	rural/clinic	No	04/04/2017	0	0	0
2015377642	576	rural/clinic	No	04/04/2017	0	0	0
2011128279	576	rural/clinic	No	04/04/2017	0	0	0
2015403155	576	rural/clinic	No	04/04/2017	0	0	0
2015289568	576	rural/clinic	No	04/04/2017	0	0	0
2015377643	576	rural/clinic	No	04/04/2017	0	0	0
2015403154	576	rural/clinic	No	04/04/2017	0	0	0
2015377644	576	rural/clinic	No	04/04/2017	0	0	0
2015403161	692	district/faith-based	No	04/04/2017	1	0	0
2015297384	692	district/faith-based	No	04/04/2017	1	0	0
2014319630	692	district/faith-based	No	04/04/2017	1	0	0
2014306001	692	district/faith-based	No	04/04/2017	1	0	0
2015403160	692	district/faith-based	No	04/04/2017	1	0	0
2014306002	692	district/faith-based	No	04/04/2017	1	0	0
2014319631	692	district/faith-based	No	04/04/2017	1	0	0
2014301327	692	district/faith-based	No	04/04/2017	1	0	0
2015360720	692	district/faith-based	No	04/04/2017	1	0	0
2015403159	692	district/faith-based	No	04/04/2017	1	0	0
2012388381	692	district/faith-based	No	04/04/2017	1	0	0
2014328433	692	district/faith-based	No	04/04/2017	1	0	0
2014348067	692	district/faith-based	No	04/04/2017	1	0	0
2014328432	692	district/faith-based	No	04/04/2017	1	0	0
2011223203	692	district/faith-based	No	04/04/2017	1	0	0
2015403158	692	district/faith-based	No	04/04/2017	1	0	0
2011204792	692	district/faith-based	No	04/04/2017	1	0	0
2015383708	692	district/faith-based	No	04/04/2017	1	0	0
2011204796	692	district/faith-based	No	04/04/2017	1	0	0
2015414325	692	district/faith-based	No	04/04/2017	1	0	0
2015338711	692	district/faith-based	No	04/04/2017	1	0	0
2015403157	692	rural/clinic	No	04/04/2017	0	0	0
2015414324	615	rural/clinic	No	04/04/2017	0	0	0
2012363957	615	rural/clinic	No	04/04/2017	0	0	0
2014296891	615	rural/clinic	No	04/04/2017	0	0	0
2015414327	615	rural/clinic	No	04/04/2017	0	0	0
2012305698	615	rural/clinic	No	04/04/2017	0	0	0
2015385951	615	rural/clinic	No	04/04/2017	0	0	0
2015414323	615	rural/clinic	No	04/04/2017	0	0	0
2011204793	615	rural/clinic	No	04/04/2017	0	0	0
2015300685	615	rural/clinic	No	04/04/2017	0	0	0
2015414326	615	rural/clinic	No	04/04/2017	0	0	0
2012358282	615	rural/clinic	No	04/04/2017	0	0	0
2011204794	794	rural/clinic	No	04/04/2017	0	0	0
2015414322	794	rural/clinic	No	04/04/2017	0	0	0
2014296893	794	rural/clinic	No	04/04/2017	0	0	0
2011223204	920	referral	No	04/04/2017	0	0	1
2012362552	920	referral	No	04/04/2017	0	0	1
2015300684	920	referral	No	04/04/2017	0	0	1
2015414330	920	referral	No	04/04/2017	0	0	1
2014296895	920	referral	No	04/04/2017	0	0	1
2015338712	920	referral	No	04/04/2017	0	0	1
2015414328	920	referral	No	04/04/2017	0	0	1
2012363952	920	referral	No	04/04/2017	0	0	1
2014296897	920	referral	No	04/04/2017	0	0	1
2015315998	920	referral	No	04/04/2017	0	0	1
2015414321	920	referral	No	04/04/2017	0	0	1
2012362551	920	referral	No	04/04/2017	0	0	1
2015414331	920	referral	No	04/04/2017	0	0	1
2014358685	920	referral	No	04/04/2017	0	0	1
2015414320	920	referral	No	04/04/2017	0	0	1
2012293003	920	referral	No	04/04/2017	0	0	1
2014375676	920	referral	No	04/04/2017	0	0	1
2015320870	920	referral	No	04/04/2017	0	0	1
2014375678	920	referral	No	04/04/2017	0	0	1
2011204797	920	referral	No	04/04/2017	0	0	1
2014375677	920	referral	No	04/04/2017	0	0	1
2011204798	920	referral	No	04/04/2017	0	0	1

2015377826	920	referral	No	04/04/2017	0	0	1
2014296896	920	referral	No	04/04/2017	0	0	1
2011204799	920	referral	No	04/04/2017	0	0	1
2015390260	920	referral	No	04/04/2017	0	0	1
2014367621	920	referral	No	04/04/2017	0	0	1
2015376205	920	referral	No	04/04/2017	0	0	1
2012349279	920	referral	No	04/04/2017	0	0	1
2013255258	920	referral	No	04/04/2017	0	0	1
2013266114	920	referral	No	04/04/2017	0	0	1
2012349278	920	referral	No	04/04/2017	0	0	1
2012245734	920	referral	No	04/04/2017	0	0	1
2015320871	920	referral	No	04/04/2017	0	0	1
2012390148	920	referral	No	04/04/2017	0	0	1
2012245733	920	referral	No	04/04/2017	0	0	1
2015320872	920	referral	No	04/04/2017	0	0	1
2015300686	920	referral	No	04/04/2017	0	0	1
2015385388	920	referral	No	04/04/2017	0	0	1
2015385389	920	referral	No	04/04/2017	0	0	1
2015359755	920	referral	No	04/04/2017	0	0	1
2015300683	920	referral	No	04/04/2017	0	0	1
2012245732	920	referral	No	04/04/2017	0	0	1
2012390149	920	referral	No	04/04/2017	0	0	1
2015359754	920	referral	No	04/04/2017	0	0	1
2015335807	920	referral	No	04/04/2017	0	0	1
2015363301	920	referral	No	04/04/2017	0	0	1
2015335808	920	referral	No	04/04/2017	0	0	1
2015331767	920	referral	No	04/04/2017	0	0	1
2015376119	920	referral	No	04/04/2017	0	0	1
2015331768	920	referral	No	04/04/2017	0	0	1
2015376120	920	referral	No	04/04/2017	0	0	1
2015342281	920	referral	No	04/04/2017	0	0	1
2012349277	920	referral	No	04/04/2017	0	0	1
2015415906	920	referral	No	04/04/2017	0	0	1
2015385387	920	referral	No	04/04/2017	0	0	1
2015342282	920	referral	No	04/04/2017	0	0	1
2015328525	920	referral	No	04/04/2017	0	0	1
2015339542	920	referral	No	04/04/2017	0	0	1
2013249280	920	referral	No	04/04/2017	0	0	1
2013249489	920	referral	No	04/04/2017	0	0	1
2014314729	920	referral	No	04/04/2017	0	0	1
2015416517	920	referral	No	04/04/2017	0	0	1
2012253240	920	referral	No	04/04/2017	0	0	1
2015415902	920	referral	No	04/04/2017	0	0	1
2014314730	920	referral	No	04/04/2017	0	0	1
2015416516	920	referral	No	04/04/2017	0	0	1
2012289784	920	referral	No	04/04/2017	0	0	1
2015348891	920	referral	No	04/04/2017	0	0	1
2015415907	920	referral	No	04/04/2017	0	0	1
2015328526	920	referral	No	04/04/2017	0	0	1
2014290272	920	referral	No	04/04/2017	0	0	1
2015352361	920	referral	No	04/04/2017	0	0	1
2015352362	920	referral	No	04/04/2017	0	0	1
2015348894	920	referral	No	04/04/2017	0	0	1
2015418371	920	referral	No	04/04/2017	0	0	1
2015286867	920	referral	No	04/04/2017	0	0	1
2015313446	920	referral	No	04/04/2017	0	0	1
2015313443	920	referral	No	04/04/2017	0	0	1
2012289783	920	referral	No	04/04/2017	0	0	1
2015313444	920	referral	No	04/04/2017	0	0	1
2014351200	920	referral	No	04/04/2017	0	0	1
2014314462	920	referral	No	04/04/2017	0	0	1
2015313448	920	referral	No	04/04/2017	0	0	1
2012289785	920	referral	No	04/04/2017	0	0	1
2015313447	920	referral	No	04/04/2017	0	0	1
2014311547	920	referral	No	04/04/2017	0	0	1
2015400003	920	referral	No	04/04/2017	0	0	1
2014342514	920	referral	No	04/04/2017	0	0	1
2015400004	920	referral	No	04/04/2017	0	0	1
2015400005	920	referral	No	04/04/2017	0	0	1
2015400006	920	referral	No	04/04/2017	0	0	1
2015418134	920	referral	No	04/04/2017	0	0	1
2015400007	920	referral	No	04/04/2017	0	0	1
2015418136	920	referral	No	04/04/2017	0	0	1
2015335585	920	referral	No	04/04/2017	0	0	1
2015400008	920	referral	No	04/04/2017	0	0	1
2015335584	920	referral	No	04/04/2017	0	0	1
2015400009	920	referral	No	04/04/2017	0	0	1
2014363589	920	referral	No	04/04/2017	0	0	1
2015400010	920	referral	No	04/04/2017	0	0	1
2015355652	920	referral	No	04/04/2017	0	0	1
2015384805	920	referral	No	04/04/2017	0	0	1
2015400011	920	referral	No	04/04/2017	0	0	1

2015400012	920	referral	No	04/04/2017	0	0	1
2014343872	920	referral	No	04/04/2017	0	0	1
2015400013	920	referral	No	04/04/2017	0	0	1
2014343873	920	referral	No	04/04/2017	0	0	1
2015400014	920	referral	No	04/04/2017	0	0	1
2015400015	920	referral	No	04/04/2017	0	0	1
2015400016	920	referral	No	04/04/2017	0	0	1
2014343874	920	referral	No	04/04/2017	0	0	1
2015400017	920	referral	No	04/04/2017	0	0	1
2015337964	920	referral	No	04/04/2017	0	0	1
2015400021	920	referral	No	04/04/2017	0	0	1
2012316544	920	referral	No	04/04/2017	0	0	1
2014348066	920	referral	No	04/04/2017	0	0	1
2012261381	920	referral	No	04/04/2017	0	0	1
2015297456	920	referral	No	30/03/2017	0	0	1
2015397864	920	referral	No	30/03/2017	0	0	1
2015397864	920	referral	No	30/03/2017	0	0	1
2011134972	920	referral	No	04/04/2017	0	0	1
2012374279	920	referral	No	04/04/2017	0	0	1
2012261448	920	referral	No	04/04/2017	0	0	1
2011134973	920	referral	No	04/04/2017	0	0	1
2015358170	920	referral	No	30/03/2017	0	0	1
2015335951	920	referral	No	04/04/2017	0	0	1
2015358170	920	referral	No	30/03/2017	0	0	1
2015402367	920	referral	No	30/03/2017	0	0	1
2015402367	920	referral	No	30/03/2017	0	0	1
2015386387	920	referral	No	30/03/2017	0	0	1
2015386387	920	referral	No	30/03/2017	0	0	1
2015386367	920	referral	No	30/03/2017	0	0	1
2015386367	920	referral	No	30/03/2017	0	0	1
2015335952	920	referral	No	04/04/2017	0	0	1
2015373699	920	referral	No	30/03/2017	0	0	1
2015373699	920	referral	No	30/03/2017	0	0	1
2014356971	920	referral	No	30/03/2017	0	0	1
2014356971	920	referral	No	30/03/2017	0	0	1
2015337961	920	referral	No	04/04/2017	0	0	1
2015340201	920	referral	No	30/03/2017	0	0	1
2015340201	920	referral	No	30/03/2017	0	0	1
2012374282	920	referral	No	04/04/2017	0	0	1
2012374281	920	referral	No	04/04/2017	0	0	1
2012374280	920	referral	No	04/04/2017	0	0	1
2012374283	920	referral	No	04/04/2017	0	0	1
2012388383	920	referral	No	04/04/2017	0	0	1
2015415903	920	referral	No	04/04/2017	0	0	1
2015415904	920	referral	No	04/04/2017	0	0	1
2015337962	920	referral	No	04/04/2017	0	0	1
201066580	920	referral	No	04/04/2017	0	0	1
2012365149	920	referral	No	04/04/2017	0	0	1
2012365147	920	referral	No	04/04/2017	0	0	1
2012365148	920	referral	No	04/04/2017	0	0	1
2011134975	920	referral	No	04/04/2017	0	0	1
2015403256	920	referral	No	04/04/2017	0	0	1
2011134974	920	referral	No	04/04/2017	0	0	1
2015403255	920	referral	No	04/04/2017	0	0	1
2015403254	920	referral	No	04/04/2017	0	0	1
2011134976	920	referral	No	04/04/2017	0	0	1
2011195781	920	referral	No	04/04/2017	0	0	1
2015403257	920	referral	No	04/04/2017	0	0	1
2011134977	920	referral	No	04/04/2017	0	0	1
2015294620	920	referral	No	04/04/2017	0	0	1
2015362468	920	referral	No	04/04/2017	0	0	1
2015403258	920	referral	No	04/04/2017	0	0	1
2011134978	920	referral	No	04/04/2017	0	0	1
2015362466	920	referral	No	04/04/2017	0	0	1
2015413549	920	referral	No	04/04/2017	0	0	1
2015362462	920	referral	No	04/04/2017	0	0	1
2015344076	920	referral	No	04/04/2017	0	0	1
2011134980	920	referral	No	04/04/2017	0	0	1
2011134979	920	referral	No	04/04/2017	0	0	1
2015344077	920	referral	No	04/04/2017	0	0	1
2014314463	920	referral	No	04/04/2017	0	0	1
2014314726	920	referral	No	04/04/2017	0	0	1
2015368570	920	referral	No	04/04/2017	0	0	1
2014314727	920	referral	No	04/04/2017	0	0	1
2014314459	920	referral	No	04/04/2017	0	0	1
2011144007	920	referral	No	04/04/2017	0	0	1
2014314732	920	referral	No	04/04/2017	0	0	1
2015368569	920	referral	No	04/04/2017	0	0	1
2014314728	920	referral	No	04/04/2017	0	0	1
2015382371	920	referral	No	04/04/2017	0	0	1
2015362459	920	referral	No	04/04/2017	0	0	1
2014314731	920	referral	No	04/04/2017	0	0	1

2013256267	920	referral	No	04/04/2017	0	0	1
2014314461	920	referral	No	04/04/2017	0	0	1
2015382370	920	referral	No	04/04/2017	0	0	1
2014314733	920	referral	No	04/04/2017	0	0	1
2014367402	920	referral	No	04/04/2017	0	0	1
2014314734	920	referral	No	04/04/2017	0	0	1
2015374853	920	referral	No	04/04/2017	0	0	1
2015297458	920	referral	No	04/04/2017	0	0	1
2015368784	920	referral	No	04/04/2017	0	0	1
2015297459	920	referral	No	04/04/2017	0	0	1
2011000001	920	referral	No	07/03/2017	0	0	1
2014314460	920	referral	No	04/04/2017	0	0	1
2014367403	920	referral	No	04/04/2017	0	0	1
2014367401	920	referral	No	04/04/2017	0	0	1
2015374856	920	referral	No	04/04/2017	0	0	1
2015414946	920	referral	No	04/04/2017	0	0	1
2015362418	920	referral	No	04/04/2017	0	0	1
2015374851	920	referral	No	04/04/2017	0	0	1
2011140934	920	referral	No	04/04/2017	0	0	1
2011140933	920	referral	No	04/04/2017	0	0	1
2015374852	920	referral	No	04/04/2017	0	0	1
2011140930	920	referral	No	04/04/2017	0	0	1
2015369686	920	referral	No	04/04/2017	0	0	1
2011140926	920	referral	No	04/04/2017	0	0	1
2015369687	920	referral	No	04/04/2017	0	0	1
2011140927	920	referral	No	04/04/2017	0	0	1
2015339818	920	referral	No	04/04/2017	0	0	1
2011140928	920	referral	No	04/04/2017	0	0	1
2015369685	920	referral	No	04/04/2017	0	0	1
2011140929	920	referral	No	04/04/2017	0	0	1
2011140931	920	referral	No	04/04/2017	0	0	1
2015369665	920	referral	No	04/04/2017	0	0	1
2015414944	920	referral	No	04/04/2017	0	0	1
2011140932	920	referral	No	04/04/2017	0	0	1
2015339819	920	referral	No	04/04/2017	0	0	1
2015286869	920	referral	No	04/04/2017	0	0	1
2015286868	920	referral	No	04/04/2017	0	0	1
2012316543	920	referral	No	04/04/2017	0	0	1
2011135347	920	referral	No	04/04/2017	0	0	1
2011135341	920	referral	No	04/04/2017	0	0	1
2014363225	920	referral	No	04/04/2017	0	0	1
2015405964	920	referral	No	04/04/2017	0	0	1
2011135345	920	referral	No	04/04/2017	0	0	1
2012259872	920	referral	No	04/04/2017	0	0	1
2015334722	920	referral	No	04/04/2017	0	0	1
2015334723	920	referral	No	04/04/2017	0	0	1
2014296900	920	referral	No	04/04/2017	0	0	1
2015339817	920	referral	No	04/04/2017	0	0	1
2015413345	920	referral	No	04/04/2017	0	0	1
2015303115	920	referral	No	04/04/2017	0	0	1
2015303114	920	referral	No	04/04/2017	0	0	1
2012244284	920	referral	No	04/04/2017	0	0	1
2015303116	920	referral	No	04/04/2017	0	0	1
2015418196	920	referral	No	04/04/2017	0	0	1
2015413346	920	referral	No	04/04/2017	0	0	1
2015418198	920	referral	No	04/04/2017	0	0	1
2013261929	920	referral	No	04/04/2017	0	0	1
2015305066	920	referral	No	04/04/2017	0	0	1
2015305067	920	referral	No	04/04/2017	0	0	1
2015305068	920	referral	No	04/04/2017	0	0	1
2015418199	920	referral	No	04/04/2017	0	0	1
2011128280	920	referral	No	04/04/2017	0	0	1
2014309975	920	referral	No	04/04/2017	0	0	1
2014347733	920	referral	No	04/04/2017	0	0	1
2014334675	920	referral	No	04/04/2017	0	0	1
2015300690	920	referral	No	04/04/2017	0	0	1
2012259870/D	920	referral	No	04/04/2017	0	0	1
2015344844	920	referral	No	04/04/2017	0	0	1
2015390302	920	referral	No	04/04/2017	0	0	1
2015390304	920	referral	No	04/04/2017	0	0	1
2014358187	920	referral	No	04/04/2017	0	0	1
2015300688	920	referral	No	04/04/2017	0	0	1
2015300689	920	referral	No	04/04/2017	0	0	1
2015300687	920	referral	No	04/04/2017	0	0	1
2014296892	920	referral	No	04/04/2017	0	0	1
2015418197	920	referral	No	04/04/2017	0	0	1
2014357309	920	referral	No	04/04/2017	0	0	1
2015414945	920	referral	No	04/04/2017	0	0	1
2015368402	920	referral	No	06/04/2017	0	0	1
2015369683	920	referral	No	06/04/2017	0	0	1
2015369680	920	referral	No	06/04/2017	0	0	1
2015369681	920	referral	No	06/04/2017	0	0	1

2015369678	920	referral	No	06/04/2017	0	0	1
2015369673	920	referral	No	06/04/2017	0	0	1
2015369676	920	referral	No	06/04/2017	0	0	1
2015369672	920	referral	No	06/04/2017	0	0	1
2015369671	920	referral	No	06/04/2017	0	0	1
2015369666	920	referral	No	06/04/2017	0	0	1
2015369664	920	referral	No	06/04/2017	0	0	1
2012259864	920	referral	No	06/04/2017	0	0	1
2012259863	920	referral	No	06/04/2017	0	0	1
2012259871	920	referral	No	06/04/2017	0	0	1
2015328467	920	referral	No	06/04/2017	0	0	1
2015328465	920	referral	No	06/04/2017	0	0	1
2015328466	920	referral	No	06/04/2017	0	0	1
2015418193	920	referral	No	06/04/2017	0	0	1
2014371569	920	referral	No	06/04/2017	0	0	1
2014371568	920	referral	No	06/04/2017	0	0	1
2014371570	920	referral	No	06/04/2017	0	0	1
2011148062	920	referral	No	06/04/2017	0	0	1
2011148064	920	referral	No	06/04/2017	0	0	1
2015296058	920	referral	No	06/04/2017	0	0	1
2015296056	920	referral	No	06/04/2017	0	0	1
2015296055	920	referral	No	06/04/2017	0	0	1
2012272343	920	referral	No	06/04/2017	0	0	1
2012272344	920	referral	No	06/04/2017	0	0	1
2013254706	920	referral	No	06/04/2017	0	0	1
2012272342	920	referral	No	06/04/2017	0	0	1
2012272345	920	referral	No	06/04/2017	0	0	1
2015397125	920	referral	No	06/04/2017	0	0	1
2015334914	920	referral	No	30/03/2017	0	0	1
2015334914	920	referral	No	30/03/2017	0	0	1
2014346311	920	referral	No	30/03/2017	0	0	1
2015358513	920	referral	No	30/03/2017	0	0	1
2015358513	920	referral	No	30/03/2017	0	0	1
2014317431	920	referral	No	30/03/2017	0	0	1
2014317431	920	referral	No	30/03/2017	0	0	1
2015391870	920	referral	No	06/04/2017	0	0	1
2015391869	920	referral	No	06/04/2017	0	0	1
2015397122	920	referral	No	06/04/2017	0	0	1
2015397121	920	referral	No	06/04/2017	0	0	1
2015397123	920	referral	No	06/04/2017	0	0	1
2015397124	920	referral	No	06/04/2017	0	0	1
2015397120	920	referral	No	06/04/2017	0	0	1
2015391873	920	referral	No	06/04/2017	0	0	1
2015391868	920	referral	No	06/04/2017	0	0	1
2015397119	920	referral	No	06/04/2017	0	0	1
2015391874	920	referral	No	06/04/2017	0	0	1
2015391875	920	referral	No	06/04/2017	0	0	1
2015391876	920	referral	No	06/04/2017	0	0	1
2015296054	920	referral	No	06/04/2017	0	0	1
2015296061	920	referral	No	06/04/2017	0	0	1
2015344847	920	referral	No	06/04/2017	0	0	1
2015344846	920	referral	No	06/04/2017	0	0	1
2015344848	920	referral	No	06/04/2017	0	0	1
2015344843	920	referral	No	06/04/2017	0	0	1
2015344855	920	referral	No	06/04/2017	0	0	1
2015344872	920	referral	No	06/04/2017	0	0	1
2015344862	920	referral	No	06/04/2017	0	0	1
2015344854	920	referral	No	06/04/2017	0	0	1
2015405723	920	referral	No	06/04/2017	0	0	1
2015286636	920	referral	No	06/04/2017	0	0	1
2015286634	920	referral	No	06/04/2017	0	0	1
2015405721	920	referral	No	06/04/2017	0	0	1
2015405719	920	referral	No	06/04/2017	0	0	1
2015286632	920	referral	No	06/04/2017	0	0	1
2015405722	920	referral	No	06/04/2017	0	0	1
2015286633	920	referral	No	06/04/2017	0	0	1
2015286635	920	referral	No	06/04/2017	0	0	1
2015286528	920	referral	No	06/04/2017	0	0	1
2015286521	920	referral	No	06/04/2017	0	0	1
2015405720	920	referral	No	06/04/2017	0	0	1
2012322943	920	referral	No	06/04/2017	0	0	1
2013253942	920	referral	No	06/04/2017	0	0	1
2015339821	920	referral	No	06/04/2017	0	0	1
2014358686	920	referral	No	06/04/2017	0	0	1
2014358687	920	referral	No	06/04/2017	0	0	1
2014371555	920	referral	No	06/04/2017	0	0	1
2015418194	920	referral	No	06/04/2017	0	0	1
2015394637	920	referral	No	08/04/2017	0	0	1
2013251503	920	referral	No	06/04/2017	0	0	1
2015394635	920	referral	No	06/04/2017	0	0	1
2015394634	920	referral	No	06/04/2017	0	0	1
2015394633	920	referral	No	06/04/2017	0	0	1

2012329000	920	referral	No	06/04/2017	0	0	1
2015394636	920	referral	No	06/04/2017	0	0	1
2015328464	920	referral	No	06/04/2017	0	0	1
2015331918	920	referral	No	06/04/2017	0	0	1
2015331914	920	referral	No	06/04/2017	0	0	1
2014327111	920	referral	No	06/04/2017	0	0	1
2014382427	920	referral	No	06/04/2017	0	0	1
2014382429	920	referral	No	06/04/2017	0	0	1
2014382432	920	referral	No	06/04/2017	0	0	1
2014327115	920	referral	No	06/04/2017	0	0	1
2015301260	920	referral	No	06/04/2017	0	0	1
2015301259	920	referral	No	06/04/2017	0	0	1
2011224279	920	referral	No	06/04/2017	0	0	1
2011224280	920	referral	No	06/04/2017	0	0	1
2015297087	920	referral	No	06/04/2017	0	0	1
2012322942	920	referral	No	06/04/2017	0	0	1
2012322945	920	referral	No	06/04/2017	0	0	1
2015405724	920	referral	No	06/04/2017	0	0	1
2015297383	920	referral	No	06/04/2017	0	0	1
2015369692	920	referral	No	06/04/2017	0	0	1
19003	920	referral	No	06/04/2017	0	0	1
19003	920	referral	No	06/04/2017	0	0	1
2014303499	920	referral	No	06/04/2017	0	0	1
2015287386	920	referral	No	06/04/2017	0	0	1
2015340751	920	referral	No	06/04/2017	0	0	1
2015297381	920	referral	No	06/04/2017	0	0	1
2015408171	920	referral	No	06/04/2017	0	0	1
2014287424	920	referral	No	06/04/2017	0	0	1
2015379311	920	referral	No	06/04/2017	0	0	1
2014357524	920	referral	No	06/04/2017	0	0	1
2014357519	920	referral	No	06/04/2017	0	0	1
2012261185	920	referral	No	06/04/2017	0	0	1
2014357523	920	referral	No	06/04/2017	0	0	1
2014357518	920	referral	No	06/04/2017	0	0	1
2014287423	920	referral	No	06/04/2017	0	0	1
2014356975	920	referral	No	06/04/2017	0	0	1
2015374256	920	referral	No	06/04/2017	0	0	1
2014356974	920	referral	No	06/04/2017	0	0	1
2015374257	920	referral	No	06/04/2017	0	0	1
2015375984	920	referral	No	06/04/2017	0	0	1
2015375104	920	referral	No	06/04/2017	0	0	1
2012298696	920	referral	No	06/04/2017	0	0	1
2012262248	920	referral	No	06/04/2017	0	0	1
2014298583	920	referral	No	06/04/2017	0	0	1
2015310603	920	referral	No	06/04/2017	0	0	1
2014324701	920	referral	No	06/04/2017	0	0	1
2015310605	920	referral	No	06/04/2017	0	0	1
2015375981	920	referral	No	06/04/2017	0	0	1
2015310604	920	referral	No	06/04/2017	0	0	1
2011113950	920	referral	No	06/04/2017	0	0	1
2012262249	920	referral	No	06/04/2017	0	0	1
2015334410	920	referral	No	06/04/2017	0	0	1
2015375982	920	referral	No	06/04/2017	0	0	1
2015385522	920	referral	No	06/04/2017	0	0	1
2012262250	920	referral	No	06/04/2017	0	0	1
2015375983	920	referral	No	06/04/2017	0	0	1
2015364602	920	referral	No	06/04/2017	0	0	1
2011181150	920	referral	No	06/04/2017	0	0	1
2014346899	920	referral	No	06/04/2017	0	0	1
2014349202	920	referral	No	06/04/2017	0	0	1
2014356973	920	referral	No	06/04/2017	0	0	1
2013250981	920	referral	No	06/04/2017	0	0	1
2014298584	920	referral	No	06/04/2017	0	0	1
2015366921	920	referral	No	06/04/2017	0	0	1
2014349213	920	referral	No	06/04/2017	0	0	1
2015392787	920	referral	No	06/04/2017	0	0	1
2014324702	920	referral	No	06/04/2017	0	0	1
2014349212	920	referral	No	06/04/2017	0	0	1
2015323962	920	referral	No	06/04/2017	0	0	1
2014347986	920	referral	No	06/04/2017	0	0	1
2015377261	920	referral	No	06/04/2017	0	0	1
2014349207	920	referral	No	06/04/2017	0	0	1
2012341043	920	referral	No	06/04/2017	0	0	1
2014346947	920	referral	No	06/04/2017	0	0	1
2014349205	920	referral	No	06/04/2017	0	0	1
2014385616	920	referral	No	06/04/2017	0	0	1
2011226479	920	referral	No	06/04/2017	0	0	1
2015321803	920	referral	No	06/04/2017	0	0	1
2015321759	920	referral	No	06/04/2017	0	0	1
2015323961	920	referral	No	06/04/2017	0	0	1
2015340845	920	referral	No	06/04/2017	0	0	1
2014349203	920	referral	No	06/04/2017	0	0	1

2011200275	920	referral	No	06/04/2017	0	0	1
2014349204	920	referral	No	06/04/2017	0	0	1
2014344467	920	referral	No	06/04/2017	0	0	1
2014349209	920	referral	No	06/04/2017	0	0	1
2015405725	920	referral	No	06/04/2017	0	0	1
2015366922	920	referral	No	06/04/2017	0	0	1
2015286631	920	referral	No	06/04/2017	0	0	1
2015323964	920	referral	No	06/04/2017	0	0	1
2015333254	920	referral	No	06/04/2017	0	0	1
2015369163	920	referral	No	06/04/2017	0	0	1
2014309586	920	referral	No	06/04/2017	0	0	1
2015323968	920	referral	No	06/04/2017	0	0	1
2013250978	920	referral	No	06/04/2017	0	0	1
2014311544	920	referral	No	06/04/2017	0	0	1
2015288603	920	referral	No	06/04/2017	0	0	1
2011140925	920	referral	No	06/04/2017	0	0	1
2015334514	920	referral	No	06/04/2017	0	0	1
2015392876	920	referral	No	06/04/2017	0	0	1
2015333916	920	referral	No	06/04/2017	0	0	1
2014311545	920	referral	No	06/04/2017	0	0	1
2015334521	920	referral	No	06/04/2017	0	0	1
2017000013	920	referral	No	06/04/2017	0	0	1
2014309587	920	referral	No	06/04/2017	0	0	1
2015392877	920	referral	No	06/04/2017	0	0	1
2014290238	920	referral	No	06/04/2017	0	0	1
2015326564	920	referral	No	06/04/2017	0	0	1
2014302519	920	referral	No	06/04/2017	0	0	1
2011223605	920	referral	No	06/04/2017	0	0	1
2015326565	920	referral	No	06/04/2017	0	0	1
2015357638	920	referral	No	06/04/2017	0	0	1
2011223608	920	referral	No	06/04/2017	0	0	1
2015392871	920	referral	No	06/04/2017	0	0	1
2015333915	920	referral	No	06/04/2017	0	0	1
2015313308	920	referral	No	06/04/2017	0	0	1
2015357640	920	referral	No	06/04/2017	0	0	1
2015397801	920	referral	No	06/04/2017	0	0	1
2011223609	920	referral	No	06/04/2017	0	0	1
2015392709	920	referral	No	06/04/2017	0	0	1
2015357639	920	referral	No	06/04/2017	0	0	1
2015313309	920	referral	No	06/04/2017	0	0	1
2014302520	920	referral	No	06/04/2017	0	0	1
2011140924	920	referral	No	06/04/2017	0	0	1
2015397802	920	referral	No	06/04/2017	0	0	1
2015351420	920	referral	No	06/04/2017	0	0	1
2011140923	920	referral	No	06/04/2017	0	0	1
2015392706	920	referral	No	06/04/2017	0	0	1
2012350333	920	referral	No	06/04/2017	0	0	1
2015351421	920	referral	No	06/04/2017	0	0	1
2014330649	920	referral	No	06/04/2017	0	0	1
2011140922	920	referral	No	06/04/2017	0	0	1
2012350335	920	referral	No	06/04/2017	0	0	1
2015351422	920	referral	No	06/04/2017	0	0	1
2011140921	920	referral	No	06/04/2017	0	0	1
2015346230	920	referral	No	06/04/2017	0	0	1
2012350329	920	referral	No	06/04/2017	0	0	1
2011140920	920	referral	No	06/04/2017	0	0	1
2015392875	920	referral	No	06/04/2017	0	0	1
2014382433	920	referral	No	06/04/2017	0	0	1
2011140919	920	referral	No	06/04/2017	0	0	1
2015311602	920	referral	No	06/04/2017	0	0	1
2015392873	920	referral	No	06/04/2017	0	0	1
2011140918	920	referral	No	06/04/2017	0	0	1
2015311603	920	referral	No	06/04/2017	0	0	1
201546091	920	referral	No	06/04/2017	0	0	1
2011140917	920	referral	No	06/04/2017	0	0	1
2014305217	920	referral	No	06/04/2017	0	0	1
2015311313	920	referral	No	06/04/2017	0	0	1
2011140916	920	referral	No	06/04/2017	0	0	1
2015392874	920	referral	No	06/04/2017	0	0	1
2011140915	920	referral	No	06/04/2017	0	0	1
2012350332	920	referral	No	06/04/2017	0	0	1
2015333955	920	referral	No	06/04/2017	0	0	1
2015351423	920	referral	No	06/04/2017	0	0	1
2015334411	920	referral	No	06/04/2017	0	0	1
2015351424	920	referral	No	06/04/2017	0	0	1
2012350330	920	referral	No	06/04/2017	0	0	1
2014304802	920	referral	No	06/04/2017	0	0	1
2015369691	920	referral	No	06/04/2017	0	0	1
2015303475	920	referral	No	06/04/2017	0	0	1
2012350326	920	referral	No	06/04/2017	0	0	1
2015369688	920	referral	No	06/04/2017	0	0	1
2015358176	920	referral	No	06/04/2017	0	0	1

2015311312	920	referral	No	06/04/2017	0	0	1
2015397553	920	referral	No	06/04/2017	0	0	1
2015303476	920	referral	No	06/04/2017	0	0	1
2012350331	920	referral	No	06/04/2017	0	0	1
2012259144/D	920	referral	No	06/04/2017	0	0	1
2015397554	920	referral	No	06/04/2017	0	0	1
201535173	920	referral	No	06/04/2017	0	0	1
2015313310	920	referral	No	06/04/2017	0	0	1
2012350328	920	referral	No	06/04/2017	0	0	1
2012322944	920	referral	No	06/04/2017	0	0	1
2015351964	920	referral	No	06/04/2017	0	0	1
2015313311	920	referral	No	06/04/2017	0	0	1
2012350334	920	referral	No	06/04/2017	0	0	1
2015358172	920	referral	No	06/04/2017	0	0	1
2012265920	920	referral	No	06/04/2017	0	0	1
2015313312	920	referral	No	06/04/2017	0	0	1
2012259145	920	referral	No	06/04/2017	0	0	1
2012350327	920	referral	No	06/04/2017	0	0	1
2014371825	920	referral	No	06/04/2017	0	0	1
2015286292	920	referral	No	06/04/2017	0	0	1
2015313313	920	referral	No	06/04/2017	0	0	1
2012259147	920	referral	No	06/04/2017	0	0	1
2014385245	920	referral	No	06/04/2017	0	0	1
201538390	920	referral	No	06/04/2017	0	0	1
2015286702	920	referral	No	06/04/2017	0	0	1
2015344319	920	referral	No	06/04/2017	0	0	1
2015313314	920	referral	No	06/04/2017	0	0	1
2015322033	920	referral	No	06/04/2017	0	0	1
2014319520	920	referral	No	06/04/2017	0	0	1
2011223607	920	referral	No	06/04/2017	0	0	1
2015286537	920	referral	No	06/04/2017	0	0	1
2012259146	920	referral	No	06/04/2017	0	0	1
2012265104	920	referral	No	06/04/2017	0	0	1
2012289600	920	referral	No	06/04/2017	0	0	1
2015379049	920	referral	No	06/04/2017	0	0	1
2011223606	920	referral	No	06/04/2017	0	0	1
2015286530	920	referral	No	06/04/2017	0	0	1
2014338551	920	referral	No	06/04/2017	0	0	1
2012265105	920	referral	No	06/04/2017	0	0	1
2015379050	920	referral	No	06/04/2017	0	0	1
2015334512	920	referral	No	06/04/2017	0	0	1
2011212184	920	referral	No	06/04/2017	0	0	1
2015286701	920	referral	No	06/04/2017	0	0	1
2015286532	920	referral	No	06/04/2017	0	0	1
2015322372	920	referral	No	06/04/2017	0	0	1
2015379048	920	referral	No	06/04/2017	0	0	1
2015344315	920	referral	No	06/04/2017	0	0	1
2015408668	920	referral	No	06/04/2017	0	0	1
2011142908	920	referral	No	06/04/2017	0	0	1
2015303602	920	referral	No	06/04/2017	0	0	1
2014385246	920	referral	No	06/04/2017	0	0	1
2014371823	920	referral	No	06/04/2017	0	0	1
2015408669	920	referral	No	06/04/2017	0	0	1
2015286533	920	referral	No	06/04/2017	0	0	1
2015324234	920	referral	No	06/04/2017	0	0	1
2015303609	920	referral	No	06/04/2017	0	0	1
2014371822	920	referral	No	06/04/2017	0	0	1
2015408670	920	referral	No	06/04/2017	0	0	1
2015352701	920	referral	No	06/04/2017	0	0	1
2015334518	920	referral	No	06/04/2017	0	0	1
2015408671	920	referral	No	06/04/2017	0	0	1
2015303610	920	referral	No	06/04/2017	0	0	1
2014379533	920	referral	No	06/04/2017	0	0	1
2015324236	920	referral	No	06/04/2017	0	0	1
2015286536	920	referral	No	06/04/2017	0	0	1
2015293940	920	referral	No	06/04/2017	0	0	1
2015351624	920	referral	No	06/04/2017	0	0	1
2015324235	920	referral	No	06/04/2017	0	0	1
2015351623	920	referral	No	06/04/2017	0	0	1
2014318237	920	referral	No	06/04/2017	0	0	1
2015303611	920	referral	No	06/04/2017	0	0	1
2015351622	920	referral	No	06/04/2017	0	0	1
2012291846	920	referral	No	06/04/2017	0	0	1
2015322595	920	referral	No	06/04/2017	0	0	1
2015293938	920	referral	No	06/04/2017	0	0	1
2015351620	920	referral	No	06/04/2017	0	0	1
2015332908	920	referral	No	06/04/2017	0	0	1
2015286535	920	referral	No	06/04/2017	0	0	1
2015322597	920	referral	No	06/04/2017	0	0	1
2015351619	920	referral	No	06/04/2017	0	0	1
2015293939	920	referral	No	06/04/2017	0	0	1
2015324237	920	referral	No	06/04/2017	0	0	1

2015303612	920	referral	No	06/04/2017	0	0	1
2015351618	920	referral	No	06/04/2017	0	0	1
2015334519	920	referral	No	06/04/2017	0	0	1
2015322378	920	referral	No	06/04/2017	0	0	1
2015294051	920	referral	No	06/04/2017	0	0	1
2015286538	920	referral	No	06/04/2017	0	0	1
2015322368	920	referral	No	06/04/2017	0	0	1
2015294052	920	referral	No	06/04/2017	0	0	1
2015334513	920	referral	No	06/04/2017	0	0	1
2015362807	920	referral	No	06/04/2017	0	0	1
2015303613	920	referral	No	06/04/2017	0	0	1
2015334517	920	referral	No	06/04/2017	0	0	1
2012322939	920	referral	No	06/04/2017	0	0	1
2014291872	920	referral	No	06/04/2017	0	0	1
2014385243	920	referral	No	06/04/2017	0	0	1
2012290699	920	referral	No	06/04/2017	0	0	1
2012322938	920	referral	No	06/04/2017	0	0	1
2014291871	920	referral	No	06/04/2017	0	0	1
2015391509	920	referral	No	06/04/2017	0	0	1
2012290700	920	referral	No	06/04/2017	0	0	1
2015334522	920	referral	No	06/04/2017	0	0	1
2015303614	920	referral	No	06/04/2017	0	0	1
2015293937	920	referral	No	10/04/2017	0	0	1
2015313301	920	referral	No	06/04/2017	0	0	1
2015391507	920	referral	No	06/04/2017	0	0	1
2012322941	920	referral	No	06/04/2017	0	0	1
2014362810	920	referral	No	06/04/2017	0	0	1
2015313302	920	referral	No	06/04/2017	0	0	1
2012261183	920	referral	No	06/04/2017	0	0	1
2015391510	920	referral	No	06/04/2017	0	0	1
2011225404	920	referral	No	06/04/2017	0	0	1
2012322940	920	referral	No	06/04/2017	0	0	1
2015293936	920	referral	No	06/04/2017	0	0	1
2015406459	920	referral	No	06/04/2017	0	0	1
2015406455	920	referral	No	06/04/2017	0	0	1
2015293707	920	referral	No	06/04/2017	0	0	1
2015406458	920	referral	No	06/04/2017	0	0	1
2015406457	920	referral	No	06/04/2017	0	0	1
2011223610	920	referral	No	06/04/2017	0	0	1
2015406454	920	referral	No	06/04/2017	0	0	1
2015303615	920	referral	No	06/04/2017	0	0	1
2015406462	920	referral	No	06/04/2017	0	0	1
2015334515	920	referral	No	06/04/2017	0	0	1
2015406461	920	referral	No	06/04/2017	0	0	1
2015406460	920	referral	No	06/04/2017	0	0	1
2013264180	920	referral	No	06/04/2017	0	0	1
2015377426	920	referral	No	06/04/2017	0	0	1
2015334516	920	referral	No	06/04/2017	0	0	1
2015414375	920	referral	No	06/04/2017	0	0	1
2015414376	920	referral	No	06/04/2017	0	0	1
2015331924	920	referral	No	06/04/2017	0	0	1
2015331923	920	referral	No	06/04/2017	0	0	1
2012261184	920	referral	No	06/04/2017	0	0	1
2015331922	920	referral	No	06/04/2017	0	0	1
2014288678	920	referral	No	06/04/2017	0	0	1
2015331921	920	referral	No	06/04/2017	0	0	1
2015331920	920	referral	No	06/04/2017	0	0	1
2015377260	920	referral	No	06/04/2017	0	0	1
2015331919	920	referral	No	06/04/2017	0	0	1
2015331917	920	referral	No	06/04/2017	0	0	1
2017265637	920	referral	No	06/04/2017	0	0	1
2015331916	920	referral	No	06/04/2017	0	0	1
2015313303	920	referral	No	06/04/2017	0	0	1
2014351223	920	referral	No	06/04/2017	0	0	1
2017265636	920	referral	No	06/04/2017	0	0	1
2015383708	920	referral	No	04/04/2017	0	0	1
2015383708	920	referral	No	04/04/2017	0	0	1
2015414325	920	referral	No	04/04/2017	0	0	1
2015414325	920	referral	No	04/04/2017	0	0	1
2015313304	920	referral	No	06/04/2017	0	0	1
2014351222	920	referral	No	06/04/2017	0	0	1
2015313305	920	referral	No	06/04/2017	0	0	1
2015313306	920	referral	No	06/04/2017	0	0	1
2015364601	920	referral	No	06/04/2017	0	0	1
2015313307	920	referral	No	06/04/2017	0	0	1
2012359921	920	referral	No	06/04/2017	0	0	1
2015364603	920	referral	No	06/04/2017	0	0	1
2015355908	920	referral	No	06/04/2017	0	0	1
2015325992	920	referral	No	06/04/2017	0	0	1
2015286867	920	referral	No	04/04/2017	0	0	1
2015400010	920	referral	No	04/04/2017	0	0	1
2015400010	920	referral	No	04/04/2017	0	0	1

2012359922	920	referral	No	06/04/2017	0	0	1
2015303616	920	referral	No	06/04/2017	0	0	1
2014327040	920	referral	No	06/04/2017	0	0	1
2014314545	920	referral	No	06/04/2017	0	0	1
2012359923	920	referral	No	06/04/2017	0	0	1
2015342306	920	referral	No	04/04/2017	0	0	1
2015342306	920	referral	No	04/04/2017	0	0	1
2011212592	920	referral	No	10/04/2017	0	0	1
2011225407	920	referral	No	06/04/2017	0	0	1
2015379312	920	referral	No	06/04/2017	0	0	1
2014314547	920	referral	No	06/04/2017	0	0	1
2015325991	920	referral	No	06/04/2017	0	0	1
2014291868	920	referral	No	31/03/2017	0	0	1
2014291868	920	referral	No	31/03/2017	0	0	1
2012259872	920	referral	No	04/04/2017	0	0	1
2012259872	920	referral	No	04/04/2017	0	0	1
2015413346	920	referral	No	04/04/2017	0	0	1
2015305066	920	referral	No	04/04/2017	0	0	1
2015305066	920	referral	No	04/04/2017	0	0	1
2012259870/D	920	referral	No	04/04/2017	0	0	1
2012259870/D	920	referral	No	04/04/2017	0	0	1
2014358187	920	referral	No	04/04/2017	0	0	1
2014358187	920	referral	No	04/04/2017	0	0	1
2011223611	920	referral	No	06/04/2017	0	0	1
2014314546	920	referral	No	06/04/2017	0	0	1
2015379307	920	referral	No	06/04/2017	0	0	1
2015325042	920	referral	No	06/04/2017	0	0	1
2015362806	920	referral	No	06/04/2017	0	0	1
2014328761	920	referral	No	06/04/2017	0	0	1
2013271839	920	referral	No	04/04/2017	0	0	1
2013271839	920	referral	No	04/04/2017	0	0	1
2015340008	920	referral	No	06/04/2017	0	0	1
2015340965	920	referral	No	06/04/2017	0	0	1
2014358329	920	referral	No	06/04/2017	0	0	1
2015340005	920	referral	No	06/04/2017	0	0	1
2015340962	920	referral	No	06/04/2017	0	0	1
2014371821	920	referral	No	06/04/2017	0	0	1
2015340006	920	referral	No	06/04/2017	0	0	1
2015340963	920	referral	No	06/04/2017	0	0	1
2014327086	920	referral	No	06/04/2017	0	0	1
2012284742	920	referral	No	04/04/2017	0	0	1
2012284742	920	referral	No	04/04/2017	0	0	1
2015349060	920	referral	No	04/04/2017	0	0	1
2014327116	920	referral	No	04/04/2017	0	0	1
2015338753	920	referral	No	06/04/2017	0	0	1
2014344466	920	referral	No	06/04/2017	0	0	1
2017167838	920	referral	No	06/04/2017	0	0	1
2014370430	920	referral	No	06/04/2017	0	0	1
2012244283	920	referral	No	04/04/2017	0	0	1
2012244283	920	referral	No	04/04/2017	0	0	1
2011199078	920	referral	No	06/04/2017	0	0	1
2014316768	920	referral	No	04/04/2017	0	0	1
2014316768	920	referral	No	04/04/2017	0	0	1
2014370429	920	referral	No	06/04/2017	0	0	1
2015303618	920	referral	No	06/04/2017	0	0	1
2014309382	920	referral	No	06/04/2017	0	0	1
2015368784	920	referral	No	04/04/2017	0	0	1
2012251544	920	referral	No	06/04/2017	0	0	1
2014367403	920	referral	No	04/04/2017	0	0	1
2015339818	920	referral	No	04/04/2017	0	0	1
2015418199	920	referral	No	04/04/2017	0	0	1
2017167839	920	referral	No	06/04/2017	0	0	1
2015414945	920	referral	No	04/04/2017	0	0	1
2012261186	920	referral	No	06/04/2017	0	0	1
2015397541	920	referral	No	06/04/2017	0	0	1
2012251543	920	referral	No	06/04/2017	0	0	1
2011140908	920	referral	No	06/04/2017	0	0	1
2015334524	920	referral	No	06/04/2017	0	0	1
2015397540	920	referral	No	06/04/2017	0	0	1
2011140909	920	referral	No	06/04/2017	0	0	1
2015397542	920	referral	No	06/04/2017	0	0	1
2011140910	920	referral	No	06/04/2017	0	0	1
2015334523	920	referral	No	06/04/2017	0	0	1
2012251542	920	referral	No	06/04/2017	0	0	1
2011140911	920	referral	No	06/04/2017	0	0	1
2015397543	920	referral	No	06/04/2017	0	0	1
2011140912	920	referral	No	06/04/2017	0	0	1
2012318935	920	referral	No	06/04/2017	0	0	1
2015397544	920	referral	No	06/04/2017	0	0	1
2015301359	920	referral	No	06/04/2017	0	0	1
2011140913	920	referral	No	06/04/2017	0	0	1
2015301360	920	referral	No	06/04/2017	0	0	1

2015384537	920	referral	No	06/04/2017	0	0	1
2011140914	920	referral	No	06/04/2017	0	0	1
2011224438	920	referral	No	06/04/2017	0	0	1
2015335378	920	referral	No	06/04/2017	0	0	1
2011199079	920	referral	No	06/04/2017	0	0	1
2011140935	920	referral	No	06/04/2017	0	0	1
2014371824	920	referral	No	06/04/2017	0	0	1
2011140936	920	referral	No	06/04/2017	0	0	1
2015289172	920	referral	No	06/04/2017	0	0	1
2014378726	920	referral	No	06/04/2017	0	0	1
2015351673	920	referral	No	06/04/2017	0	0	1
2011140937	920	referral	No	06/04/2017	0	0	1
2015384533	920	referral	No	06/04/2017	0	0	1
2011140938	920	referral	No	06/04/2017	0	0	1
2014378725	920	referral	No	06/04/2017	0	0	1
2015351670	920	referral	No	06/04/2017	0	0	1
2015351671	920	referral	No	06/04/2017	0	0	1
2015384535	920	referral	No	06/04/2017	0	0	1
2014378727	920	referral	No	06/04/2017	0	0	1
2011140939	920	referral	No	06/04/2017	0	0	1
2015351672	920	referral	No	06/04/2017	0	0	1
2011140940	920	referral	No	06/04/2017	0	0	1
2014291873	920	referral	No	06/04/2017	0	0	1
2014321418	920	referral	No	06/04/2017	0	0	1
2015351668	920	referral	No	06/04/2017	0	0	1
2011226478	920	referral	No	06/04/2017	0	0	1
2011140941	920	referral	No	06/04/2017	0	0	1
2014358331	920	referral	No	06/04/2017	0	0	1
2015351669	920	referral	No	06/04/2017	0	0	1
2015326524	920	referral	No	07/04/2017	0	0	1
2011140942	920	referral	No	06/04/2017	0	0	1
2015335377	920	referral	No	06/04/2017	0	0	1
2014358330	920	referral	No	06/04/2017	0	0	1
2015334520	920	referral	No	06/04/2017	0	0	1
2014305942	920	referral	No	06/04/2017	0	0	1
2015384536	920	referral	No	06/04/2017	0	0	1
2011144806	920	referral	No	07/04/2017	0	0	1
2011140902	920	referral	No	06/04/2017	0	0	1
2015351674	920	referral	No	06/04/2017	0	0	1
2013272690	920	referral	No	06/04/2017	0	0	1
2011140903	920	referral	No	06/04/2017	0	0	1
2015351676	920	referral	No	06/04/2017	0	0	1
2015377047	920	referral	No	06/04/2017	0	0	1
2013272689	920	referral	No	06/04/2017	0	0	1
2015351675	920	referral	No	06/04/2017	0	0	1
2011140904	920	referral	No	06/04/2017	0	0	1
2011144807	920	referral	No	07/04/2017	0	0	1
2011140905	920	referral	No	06/04/2017	0	0	1
2014314544	920	referral	No	06/04/2017	0	0	1
2012344078	920	referral	No	06/04/2017	0	0	1
2015384531	920	referral	No	06/04/2017	0	0	1
2011140906	920	referral	No	06/04/2017	0	0	1
2014289415	920	referral	No	07/04/2017	0	0	1
2014305943	920	referral	No	06/04/2017	0	0	1
2011140907	920	referral	No	06/04/2017	0	0	1
2014347012	920	referral	No	06/04/2017	0	0	1
2012344496	920	referral	No	06/04/2017	0	0	1
2014286902	920	referral	No	06/04/2017	0	0	1
2015326530	920	referral	No	07/04/2017	0	0	1
2015323966	920	referral	No	06/04/2017	0	0	1
2015355391	920	referral	No	06/04/2017	0	0	1
2015414377	920	referral	No	06/04/2017	0	0	1
2015323967	920	referral	No	06/04/2017	0	0	1
2015379504	920	referral	No	06/04/2017	0	0	1
2015326490	920	referral	No	07/04/2017	0	0	1
2015414378	920	referral	No	06/04/2017	0	0	1
2015392878	920	referral	No	06/04/2017	0	0	1
2014346898	920	referral	No	06/04/2017	0	0	1
2015326484	920	referral	No	07/04/2017	0	0	1
2015392872	920	referral	No	06/04/2017	0	0	1
2011142907	920	referral	No	06/04/2017	0	0	1
2011226477	920	referral	No	06/04/2017	0	0	1
2015323965	920	referral	No	06/04/2017	0	0	1
2015330475	920	referral	No	06/04/2017	0	0	1
2015392707	920	referral	No	06/04/2017	0	0	1
2015377048	920	referral	No	06/04/2017	0	0	1
2015326485	920	referral	No	07/04/2017	0	0	1
2015384534	920	referral	No	06/04/2017	0	0	1
2013258606	920	referral	No	06/04/2017	0	0	1
2015326489	920	referral	No	07/04/2017	0	0	1
2014346897	920	referral	No	06/04/2017	0	0	1
2015332703	920	referral	No	06/04/2017	0	0	1

2015377046	920	referral	No	06/04/2017	0	0	1
2014302900	920	referral	No	06/04/2017	0	0	1
2015326487	920	referral	No	07/04/2017	0	0	1
2015384532	920	referral	No	06/04/2017	0	0	1
2015360502	920	referral	No	06/04/2017	0	0	1
2015326488	920	referral	No	07/04/2017	0	0	1
2014346895	920	referral	No	06/04/2017	0	0	1
2015360503	920	referral	No	06/04/2017	0	0	1
2015377049	920	referral	No	06/04/2017	0	0	1
2015360510	920	referral	No	06/04/2017	0	0	1
2015326486	920	referral	No	07/04/2017	0	0	1
2015360511	920	referral	No	06/04/2017	0	0	1
2015286600	920	referral	No	06/04/2017	0	0	1
2015332451	920	referral	No	06/04/2017	0	0	1
2015326483	920	referral	No	07/04/2017	0	0	1
2015286599	920	referral	No	06/04/2017	0	0	1
2015379309	920	referral	No	06/04/2017	0	0	1
2015286598	920	referral	No	06/04/2017	0	0	1
2015379310	920	referral	No	06/04/2017	0	0	1
2015326482	920	referral	No	07/04/2017	0	0	1
2014334171	920	referral	No	06/04/2017	0	0	1
2015379308	920	referral	No	06/04/2017	0	0	1
2014334172	920	referral	No	06/04/2017	0	0	1
2015326481	920	referral	No	07/04/2017	0	0	1
2014334170	920	referral	No	06/04/2017	0	0	1
2015287801	920	referral	No	04/04/2017	0	0	1
2012323658	920	referral	No	06/04/2017	0	0	1
2015378727	920	referral	No	06/04/2017	0	0	1
2012323657	920	referral	No	06/04/2017	0	0	1
2012323659	920	referral	No	06/04/2017	0	0	1
2015378728	920	referral	No	06/04/2017	0	0	1
2012323660	920	referral	No	06/04/2017	0	0	1
2012314011	920	referral	No	06/04/2017	0	0	1
2014357521	920	referral	No	06/04/2017	0	0	1
2012314013	30	rural/clinic	No	06/04/2017	0	0	0
2014357520	30	rural/clinic	No	06/04/2017	0	0	0
2015402544	30	rural/clinic	No	06/04/2017	0	0	0
2015402545	30	rural/clinic	No	06/04/2017	0	0	0
2015402546	30	rural/clinic	No	06/04/2017	0	0	0
2015402547	30	rural/clinic	No	06/04/2017	0	0	0
2015402548	30	rural/clinic	No	06/04/2017	0	0	0
2015402549	30	rural/clinic	No	06/04/2017	0	0	0
2015402550	30	rural/clinic	No	06/04/2017	0	0	0
2015402601	30	rural/clinic	No	06/04/2017	0	0	0
2015386391	30	rural/clinic	No	06/04/2017	0	0	0
2015386397	30	rural/clinic	No	06/04/2017	0	0	0
2015386394	30	rural/clinic	No	06/04/2017	0	0	0
2015386396	30	rural/clinic	No	06/04/2017	0	0	0
2015358171	30	rural/clinic	No	06/04/2017	0	0	0
2015386392	30	rural/clinic	No	06/04/2017	0	0	0
2015386393	30	rural/clinic	No	06/04/2017	0	0	0
2015386398	30	rural/clinic	No	06/04/2017	0	0	0
2015386395	30	rural/clinic	No	06/04/2017	0	0	0
2015386388	30	rural/clinic	No	06/04/2017	0	0	0
2015326328	30	rural/clinic	No	07/04/2017	0	0	0
2015386389	30	rural/clinic	No	06/04/2017	0	0	0
2015326326	30	rural/clinic	No	07/04/2017	0	0	0
2015402373	30	rural/clinic	No	06/04/2017	0	0	0
2015326478	30	rural/clinic	No	07/04/2017	0	0	0
2015402374	30	rural/clinic	No	06/04/2017	0	0	0
2015326329	30	rural/clinic	No	07/04/2017	0	0	0
2015402370	30	rural/clinic	No	06/04/2017	0	0	0
2015402371	30	rural/clinic	No	06/04/2017	0	0	0
2015402376	30	rural/clinic	No	10/04/2017	0	0	0
2015326330	30	rural/clinic	No	07/04/2017	0	0	0
2015402375	30	rural/clinic	No	06/04/2017	0	0	0
2015402372	30	rural/clinic	No	06/04/2017	0	0	0
2015402377	30	rural/clinic	No	06/04/2017	0	0	0
2015358174	30	rural/clinic	No	06/04/2017	0	0	0
2015358175	30	rural/clinic	No	06/04/2017	0	0	0
2015326479	30	rural/clinic	No	07/04/2017	0	0	0
2015326480	30	rural/clinic	No	07/04/2017	0	0	0
2015326531	30	rural/clinic	No	07/04/2017	0	0	0
2015326532	30	rural/clinic	No	07/04/2017	0	0	0
2015326525	30	rural/clinic	No	07/04/2017	0	0	0
2015326523	30	rural/clinic	No	07/04/2017	0	0	0
2015326520	30	rural/clinic	No	07/04/2017	0	0	0
2015326529	30	rural/clinic	No	07/04/2017	0	0	0
2015326528	30	rural/clinic	No	07/04/2017	0	0	0
2015326527	30	rural/clinic	No	07/04/2017	0	0	0
2015326526	30	rural/clinic	No	07/04/2017	0	0	0
2015326315	30	rural/clinic	No	07/04/2017	0	0	0

2015326316	30	rural/clinic	No	07/04/2017	0	0	0
2015326317	30	rural/clinic	No	07/04/2017	0	0	0
2015326318	30	rural/clinic	No	07/04/2017	0	0	0
2015326319	30	rural/clinic	No	07/04/2017	0	0	0
2015326320	30	rural/clinic	No	07/04/2017	0	0	0
2015326325	30	rural/clinic	No	07/04/2017	0	0	0
2015326324	30	rural/clinic	No	07/04/2017	0	0	0
2015326321	30	rural/clinic	No	07/04/2017	0	0	0
2015326322	30	rural/clinic	No	07/04/2017	0	0	0
2015326323	30	rural/clinic	No	07/04/2017	0	0	0
2015338711	30	rural/clinic	No	04/04/2017	0	0	0
2015338711	30	rural/clinic	No	04/04/2017	0	0	0
2015338712	30	rural/clinic	No	04/04/2017	0	0	0
2015338712	30	rural/clinic	No	04/04/2017	0	0	0
2015320871	30	rural/clinic	No	04/04/2017	0	0	0
2015320871	30	rural/clinic	No	04/04/2017	0	0	0
2015359755	30	rural/clinic	No	04/04/2017	0	0	0
2015359755	30	rural/clinic	No	04/04/2017	0	0	0
2015342282	30	rural/clinic	No	04/04/2017	0	0	0
2015342282	30	rural/clinic	No	04/04/2017	0	0	0
2012289784	30	rural/clinic	No	04/04/2017	0	0	0
2012289784	30	rural/clinic	No	04/04/2017	0	0	0
2015391875	30	rural/clinic	No	06/04/2017	0	0	0
2015374433	30	rural/clinic	No	07/04/2017	0	0	0
2015374414	30	rural/clinic	No	07/04/2017	0	0	0
2015374418	30	rural/clinic	No	07/04/2017	0	0	0
2015374419	30	rural/clinic	No	07/04/2017	0	0	0
2015374422	30	rural/clinic	No	07/04/2017	0	0	0
2015376113	30	rural/clinic	No	07/04/2017	0	0	0
2015376114	30	rural/clinic	No	07/04/2017	0	0	0
2015376115	30	rural/clinic	No	07/04/2017	0	0	0
2015376118	30	rural/clinic	No	07/04/2017	0	0	0
2015376105	30	rural/clinic	No	07/04/2017	0	0	0
2011140944	30	rural/clinic	No	07/04/2017	0	0	0
2011140945	30	rural/clinic	No	07/04/2017	0	0	0
2011140947	30	rural/clinic	No	07/04/2017	0	0	0
2011140948	30	rural/clinic	No	07/04/2017	0	0	0
2011140949	30	rural/clinic	No	07/04/2017	0	0	0
2011140950	30	rural/clinic	No	07/04/2017	0	0	0
2011141053	30	rural/clinic	No	07/04/2017	0	0	0
2011141054	30	rural/clinic	No	07/04/2017	0	0	0
2015303499	30	rural/clinic	No	07/04/2017	0	0	0
2015303477	30	rural/clinic	No	07/04/2017	0	0	0
2014349202	30	rural/clinic	No	06/04/2017	0	0	0
2015348891	30	rural/clinic	No	04/04/2017	0	0	0
2015348891	30	rural/clinic	No	04/04/2017	0	0	0
2011140909	30	rural/clinic	No	06/04/2017	0	0	0
2011140936	30	rural/clinic	No	06/04/2017	0	0	0
2011140936	30	rural/clinic	No	06/04/2017	0	0	0
2015313310	30	rural/clinic	No	06/04/2017	0	0	0
2015313310	30	rural/clinic	No	06/04/2017	0	0	0
2015397554	30	rural/clinic	No	06/04/2017	0	0	0
2015397554	30	rural/clinic	No	06/04/2017	0	0	0
2014372238	30	rural/clinic	No	11/04/2017	0	0	0
2013249415	30	rural/clinic	No	11/04/2017	0	0	0
2013249416	30	rural/clinic	No	11/04/2017	0	0	0
2011195849	30	rural/clinic	No	11/04/2017	0	0	0
2014314736	30	rural/clinic	No	11/04/2017	0	0	0
2014314737	30	rural/clinic	No	11/04/2017	0	0	0
2014314738	30	rural/clinic	No	11/04/2017	0	0	0
2015342308	30	rural/clinic	No	11/04/2017	0	0	0
2015377648	30	rural/clinic	No	11/04/2017	0	0	0
2015377649	30	rural/clinic	No	11/04/2017	0	0	0
2015377650	30	rural/clinic	No	11/04/2017	0	0	0
2015368951	30	rural/clinic	No	11/04/2017	0	0	0
2015368952	30	rural/clinic	No	11/04/2017	0	0	0
2015368953	20	rural/clinic	No	11/04/2017	0	0	0
2015368954	20	rural/clinic	No	11/04/2017	0	0	0
2014358188	20	rural/clinic	No	11/04/2017	0	0	0
2012304328	20	rural/clinic	No	11/04/2017	0	0	0
2012304329	20	rural/clinic	No	11/04/2017	0	0	0
2012304330	20	rural/clinic	No	11/04/2017	0	0	0
2014301676	20	rural/clinic	No	11/04/2017	0	0	0
2014328397	20	rural/clinic	No	11/04/2017	0	0	0
2015297389	20	rural/clinic	No	11/04/2017	0	0	0
2015412451	20	rural/clinic	No	11/04/2017	0	0	0
2015294050	20	rural/clinic	No	11/04/2017	0	0	0
2015297387	20	rural/clinic	No	11/04/2017	0	0	0
2015297314	20	rural/clinic	No	11/04/2017	0	0	0
2014327672	20	rural/clinic	No	11/04/2017	0	0	0
2015297315	20	rural/clinic	No	11/04/2017	0	0	0
2014327671	20	rural/clinic	No	11/04/2017	0	0	0

2015297316	20	rural/clinic	No	11/04/2017	0	0	0
2014327669	20	rural/clinic	No	11/04/2017	0	0	0
2015297317	20	rural/clinic	No	11/04/2017	0	0	0
2014327670	20	rural/clinic	No	11/04/2017	0	0	0
2014382297	20	rural/clinic	No	11/04/2017	0	0	0
2014372234	20	rural/clinic	No	11/04/2017	0	0	0
2014335995	20	rural/clinic	No	11/04/2017	0	0	0
2014372235	20	rural/clinic	No	11/04/2017	0	0	0
2015397920	20	rural/clinic	No	11/04/2017	0	0	0
2014372236	20	rural/clinic	No	11/04/2017	0	0	0
2014382294	20	rural/clinic	No	11/04/2017	0	0	0
2014372237	20	rural/clinic	No	11/04/2017	0	0	0
2014382295	20	rural/clinic	No	11/04/2017	0	0	0
2015334728	20	rural/clinic	No	11/04/2017	0	0	0
2015377880	20	rural/clinic	No	11/04/2017	0	0	0
2011234895	20	rural/clinic	No	11/04/2017	0	0	0
2015377878	20	rural/clinic	No	11/04/2017	0	0	0
2015377879	20	rural/clinic	No	11/04/2017	0	0	0
2015383709	20	rural/clinic	No	11/04/2017	0	0	0
2015297319	20	rural/clinic	No	11/04/2017	0	0	0
2015297320	20	rural/clinic	No	11/04/2017	0	0	0
2015297318	20	rural/clinic	No	11/04/2017	0	0	0
2015412256	20	rural/clinic	No	11/04/2017	0	0	0
2015344871	20	rural/clinic	No	11/04/2017	0	0	0
2011192838	20	rural/clinic	No	11/04/2017	0	0	0
2015286638	20	rural/clinic	No	11/04/2017	0	0	0
2015397921	20	rural/clinic	No	11/04/2017	0	0	0
2015378729	20	rural/clinic	No	11/04/2017	0	0	0
2015297385	20	rural/clinic	No	11/04/2017	0	0	0
2015378730	20	rural/clinic	No	11/04/2017	0	0	0
2011192836	20	rural/clinic	No	11/04/2017	0	0	0
2014368846	20	rural/clinic	No	11/04/2017	0	0	0
2014335994	20	rural/clinic	No	11/04/2017	0	0	0
2015397919	20	rural/clinic	No	11/04/2017	0	0	0
2015397918	20	rural/clinic	No	11/04/2017	0	0	0
2012293697	20	rural/clinic	No	11/04/2017	0	0	0
2014335993	20	rural/clinic	No	11/04/2017	0	0	0
2012242582	20	rural/clinic	No	11/04/2017	0	0	0
2012259873	20	rural/clinic	No	11/04/2017	0	0	0
2015302403	20	rural/clinic	No	11/04/2017	0	0	0
2015303118	20	rural/clinic	No	11/04/2017	0	0	0
2015302404	20	rural/clinic	No	11/04/2017	0	0	0
2012321334	20	rural/clinic	No	11/04/2017	0	0	0
2015340076	20	rural/clinic	No	11/04/2017	0	0	0
2014331380	20	rural/clinic	No	11/04/2017	0	0	0
2015295555	20	rural/clinic	No	11/04/2017	0	0	0
2015340077	20	rural/clinic	No	11/04/2017	0	0	0
2014355849	20	rural/clinic	No	11/04/2017	0	0	0
2014327119	20	rural/clinic	No	11/04/2017	0	0	0
2014288422	20	rural/clinic	No	11/04/2017	0	0	0
2015325181	20	rural/clinic	No	11/04/2017	0	0	0
2012358283	20	rural/clinic	No	11/04/2017	0	0	0
2012358287	20	rural/clinic	No	11/04/2017	0	0	0
2015325180	20	rural/clinic	No	11/04/2017	0	0	0
2012358285	20	rural/clinic	No	11/04/2017	0	0	0
2014350094	20	rural/clinic	No	11/04/2017	0	0	0
2014291620	20	rural/clinic	No	11/04/2017	0	0	0
2014349916	20	rural/clinic	No	11/04/2017	0	0	0
2015355512	20	rural/clinic	No	11/04/2017	0	0	0
2015368573	20	rural/clinic	No	11/04/2017	0	0	0
2015368574	20	rural/clinic	No	11/04/2017	0	0	0
2015368571	20	rural/clinic	No	11/04/2017	0	0	0
2015368572	20	rural/clinic	No	11/04/2017	0	0	0
2015385953	20	rural/clinic	No	11/04/2017	0	0	0
2012246194	20	rural/clinic	No	11/04/2017	0	0	0
2015418139	20	rural/clinic	No	11/04/2017	0	0	0
2012246195	20	rural/clinic	No	11/04/2017	0	0	0
2012246196	20	rural/clinic	No	11/04/2017	0	0	0
2012246197/D	20	rural/clinic	No	11/04/2017	0	0	0
2015418140	20	rural/clinic	No	11/04/2017	0	0	0
2012246198	20	rural/clinic	No	11/04/2017	0	0	0
2014342516	20	rural/clinic	No	11/04/2017	0	0	0
2014358207	20	rural/clinic	No	11/04/2017	0	0	0
2014358204	20	rural/clinic	No	11/04/2017	0	0	0
2014358206	20	rural/clinic	No	11/04/2017	0	0	0
2014342515	20	rural/clinic	No	11/04/2017	0	0	0
2014342512	20	rural/clinic	No	11/04/2017	0	0	0
2015339544	20	rural/clinic	No	11/04/2017	0	0	0
2015339545	20	rural/clinic	No	11/04/2017	0	0	0
2015339546	20	rural/clinic	No	11/04/2017	0	0	0
2015339547	20	rural/clinic	No	11/04/2017	0	0	0
2015334920	20	rural/clinic	No	11/04/2017	0	0	0

2015334921	20	rural/clinic	No	11/04/2017	0	0	0
2012246199	20	rural/clinic	No	11/04/2017	0	0	0
2015413591	20	rural/clinic	No	11/04/2017	0	0	0
2015413592	20	rural/clinic	No	11/04/2017	0	0	0
2015413593	20	rural/clinic	No	11/04/2017	0	0	0
2015344078	20	rural/clinic	No	11/04/2017	0	0	0
2015344079	20	rural/clinic	No	11/04/2017	0	0	0
2011125796	20	rural/clinic	No	11/04/2017	0	0	0
2012246200	20	rural/clinic	No	11/04/2017	0	0	0
2015344730	20	rural/clinic	No	11/04/2017	0	0	0
2015344731	20	rural/clinic	No	11/04/2017	0	0	0
2014360958	20	rural/clinic	No	11/04/2017	0	0	0
2015340006	20	rural/clinic	No	06/04/2017	0	0	0
2015340006	20	rural/clinic	No	06/04/2017	0	0	0
2015413992	20	rural/clinic	No	11/04/2017	0	0	0
2012251543	20	rural/clinic	No	06/04/2017	0	0	0
2015287801	20	rural/clinic	No	04/04/2017	0	0	0
2015287801	20	rural/clinic	No	04/04/2017	0	0	0
2012291489	20	rural/clinic	No	11/04/2017	0	0	0
2014291871	20	rural/clinic	No	06/04/2017	0	0	0
2014291871	20	rural/clinic	No	06/04/2017	0	0	0
2015313303	20	rural/clinic	No	06/04/2017	0	0	0
2015313303	20	rural/clinic	No	06/04/2017	0	0	0
2014357310	20	rural/clinic	No	11/04/2017	0	0	0
2012350333	20	rural/clinic	No	06/04/2017	0	0	0
2015413993	20	rural/clinic	No	11/04/2017	0	0	0
2014357817	20	rural/clinic	No	11/04/2017	0	0	0
2015413994	20	rural/clinic	No	11/04/2017	0	0	0
2012344078	20	rural/clinic	No	06/04/2017	0	0	0
2012344496	20	rural/clinic	No	06/04/2017	0	0	0
2012344496	20	rural/clinic	No	06/04/2017	0	0	0
2015360502	20	rural/clinic	No	06/04/2017	0	0	0
2015360502	20	rural/clinic	No	06/04/2017	0	0	0
2015379308	20	rural/clinic	No	06/04/2017	0	0	0
2015379308	20	rural/clinic	No	06/04/2017	0	0	0
2015413995	20	rural/clinic	No	11/04/2017	0	0	0
2015406454	20	rural/clinic	No	06/04/2017	0	0	0
2015406454	20	rural/clinic	No	06/04/2017	0	0	0
2015406460	20	rural/clinic	No	06/04/2017	0	0	0
2015406460	20	rural/clinic	No	06/04/2017	0	0	0
2012245735	20	rural/clinic	No	11/04/2017	0	0	0
2015413996	20	rural/clinic	No	11/04/2017	0	0	0
2015369730	20	rural/clinic	No	11/04/2017	0	0	0
2015303611	20	rural/clinic	No	06/04/2017	0	0	0
2015303611	20	rural/clinic	No	06/04/2017	0	0	0
2015414948	20	rural/clinic	No	11/04/2017	0	0	0
2015286536	20	rural/clinic	No	06/04/2017	0	0	0
2015414949	20	rural/clinic	No	11/04/2017	0	0	0
2015377046	20	rural/clinic	No	06/04/2017	0	0	0
2014334171	20	rural/clinic	No	06/04/2017	0	0	0
2014334171	20	rural/clinic	No	06/04/2017	0	0	0
2015414947	20	rural/clinic	No	11/04/2017	0	0	0
2015369729	20	rural/clinic	No	11/04/2017	0	0	0
2015369731	20	rural/clinic	No	11/04/2017	0	0	0
2014298238/D	20	rural/clinic	No	11/04/2017	0	0	0
2015287354	20	rural/clinic	No	11/04/2017	0	0	0
2015287355	20	rural/clinic	No	11/04/2017	0	0	0
2015287356	20	rural/clinic	No	11/04/2017	0	0	0
2015287357	20	rural/clinic	No	11/04/2017	0	0	0
2015287358	20	rural/clinic	No	11/04/2017	0	0	0
2015287359	20	rural/clinic	No	11/04/2017	0	0	0
2015359843	20	rural/clinic	No	11/04/2017	0	0	0
2015359844	20	rural/clinic	No	11/04/2017	0	0	0
2015359845	20	rural/clinic	No	11/04/2017	0	0	0
2015359846	20	rural/clinic	No	11/04/2017	0	0	0
2012284025	20	rural/clinic	No	11/04/2017	0	0	0
2014356850	20	rural/clinic	No	11/04/2017	0	0	0
2015361601	20	rural/clinic	No	11/04/2017	0	0	0
2015397545	20	rural/clinic	No	11/04/2017	0	0	0
2015397515	20	rural/clinic	No	11/04/2017	0	0	0
2014327673	20	rural/clinic	No	11/04/2017	0	0	0
2015334726	20	rural/clinic	No	11/04/2017	0	0	0
2015413997	20	rural/clinic	No	11/04/2017	0	0	0
2015413896	20	rural/clinic	No	11/04/2017	0	0	0
2012260082	20	rural/clinic	No	11/04/2017	0	0	0
2012260083	20	rural/clinic	No	11/04/2017	0	0	0
2015319436	20	rural/clinic	No	11/04/2017	0	0	0
2012358284	20	rural/clinic	No	11/04/2017	0	0	0
2011195902	20	rural/clinic	No	11/04/2017	0	0	0
2015344868	20	rural/clinic	No	11/04/2017	0	0	0
2015344869	20	rural/clinic	No	11/04/2017	0	0	0
2015294872	20	rural/clinic	No	11/04/2017	0	0	0

2015344870	20	rural/clinic	No	11/04/2017	0	0	0
2015334727	20	rural/clinic	No	11/04/2017	0	0	0
2012358286	20	rural/clinic	No	11/04/2017	0	0	0
2011135653	20	rural/clinic	No	11/04/2017	0	0	0
2015293650	20	rural/clinic	No	11/04/2017	0	0	0
2015362419	20	rural/clinic	No	11/04/2017	0	0	0
2015324569	20	rural/clinic	No	11/04/2017	0	0	0
2011195847	20	rural/clinic	No	11/04/2017	0	0	0
2011195848	20	rural/clinic	No	11/04/2017	0	0	0
2015289569	20	rural/clinic	No	11/04/2017	0	0	0
2014327667	20	rural/clinic	No	11/04/2017	0	0	0
2014327668	20	rural/clinic	No	11/04/2017	0	0	0
2015334614	20	rural/clinic	No	11/04/2017	0	0	0
2015334615	20	rural/clinic	No	11/04/2017	0	0	0
2012295979	20	rural/clinic	No	11/04/2017	0	0	0
2015360723	20	rural/clinic	No	11/04/2017	0	0	0
2014375679	20	rural/clinic	No	11/04/2017	0	0	0
2014375680	20	rural/clinic	No	11/04/2017	0	0	0
2014375681	20	rural/clinic	No	11/04/2017	0	0	0
2014375682	20	rural/clinic	No	11/04/2017	0	0	0
2014297964	451	rural/clinic	No	11/04/2017	0	0	0
2015368809	451	rural/clinic	No	11/04/2017	0	0	0
2015368810	451	rural/clinic	No	11/04/2017	0	0	0
2015414332	451	rural/clinic	No	11/04/2017	0	0	0
2015369922	451	rural/clinic	No	11/04/2017	0	0	0
2015303069	451	rural/clinic	No	11/04/2017	0	0	0
2015303070	451	rural/clinic	No	11/04/2017	0	0	0
2015363708	451	rural/clinic	No	11/04/2017	0	0	0
2015363709	451	rural/clinic	No	11/04/2017	0	0	0
2015326533	451	rural/clinic	No	13/04/2017	0	0	0
2012293698	451	rural/clinic	No	11/04/2017	0	0	0
2012293699	451	rural/clinic	No	11/04/2017	0	0	0
2014336899	451	rural/clinic	No	11/04/2017	0	0	0
2014314208	451	rural/clinic	No	11/04/2017	0	0	0
2014314209	451	rural/clinic	No	11/04/2017	0	0	0
2015364374	451	rural/clinic	No	11/04/2017	0	0	0
2014371567	451	rural/clinic	No	11/04/2017	0	0	0
2014371565	451	rural/clinic	No	11/04/2017	0	0	0
2014371566	451	rural/clinic	No	11/04/2017	0	0	0
2014371564	451	rural/clinic	No	11/04/2017	0	0	0
2014296323	451	rural/clinic	No	11/04/2017	0	0	0
2014296322	451	rural/clinic	No	11/04/2017	0	0	0
2014296321	451	rural/clinic	No	11/04/2017	0	0	0
2013262227	114	rural/clinic	No	11/04/2017	0	0	0
2013262228	114	rural/clinic	No	11/04/2017	0	0	0
2013262226	114	rural/clinic	No	11/04/2017	0	0	0
2014358688	114	rural/clinic	No	11/04/2017	0	0	0
2014307502	114	rural/clinic	No	11/04/2017	0	0	0
2012295978	114	rural/clinic	No	11/04/2017	0	0	0
2012295980	114	rural/clinic	No	11/04/2017	0	0	0
2014327243	114	rural/clinic	No	11/04/2017	0	0	0
2015340072	114	rural/clinic	No	11/04/2017	0	0	0
2013271840	114	rural/clinic	No	11/04/2017	0	0	0
2014349915	114	rural/clinic	No	11/04/2017	0	0	0
2014320296	114	rural/clinic	No	11/04/2017	0	0	0
2015340074	114	rural/clinic	No	11/04/2017	0	0	0
2015340073	114	rural/clinic	No	11/04/2017	0	0	0
2012368535	114	rural/clinic	No	11/04/2017	0	0	0
2015340075	114	rural/clinic	No	11/04/2017	0	0	0
2014327118	114	rural/clinic	No	11/04/2017	0	0	0
2014349914	114	rural/clinic	No	11/04/2017	0	0	0
2014357816	114	rural/clinic	No	11/04/2017	0	0	0
2014357815	114	rural/clinic	No	11/04/2017	0	0	0
2012346718	114	rural/clinic	No	11/04/2017	0	0	0
2012346717	114	rural/clinic	No	11/04/2017	0	0	0
2015294873	114	rural/clinic	No	11/04/2017	0	0	0
2015294874	114	rural/clinic	No	11/04/2017	0	0	0
2012321333	114	rural/clinic	No	11/04/2017	0	0	0
2015335440	114	rural/clinic	No	11/04/2017	0	0	0
2015335441	114	rural/clinic	No	11/04/2017	0	0	0
2015335953	114	rural/clinic	No	11/04/2017	0	0	0
2015335954	114	rural/clinic	No	11/04/2017	0	0	0
2015335955	114	rural/clinic	No	11/04/2017	0	0	0
2015335956	114	rural/clinic	No	11/04/2017	0	0	0
2015355735	114	rural/clinic	No	11/04/2017	0	0	0
2014363590	114	rural/clinic	No	11/04/2017	0	0	0
2015337966	114	rural/clinic	No	11/04/2017	0	0	0
2015337965	114	rural/clinic	No	11/04/2017	0	0	0
2015384806	114	rural/clinic	No	11/04/2017	0	0	0
2015355734	114	rural/clinic	No	11/04/2017	0	0	0
2015413550	114	rural/clinic	No	11/04/2017	0	0	0
2015368651	114	rural/clinic	No	11/04/2017	0	0	0

2015368652	114	rural/clinic	No	11/04/2017	0	0	0
2015368653	114	rural/clinic	No	11/04/2017	0	0	0
2012358281	114	rural/clinic	No	11/04/2017	0	0	0
2012388386	114	rural/clinic	No	11/04/2017	0	0	0
2015385952	114	rural/clinic	No	11/04/2017	0	0	0
2015337307	114	rural/clinic	No	11/04/2017	0	0	0
2015324433	114	rural/clinic	No	11/04/2017	0	0	0
2015324434	114	rural/clinic	No	11/04/2017	0	0	0
2015315901	114	rural/clinic	No	11/04/2017	0	0	0
2015324471	114	rural/clinic	No	11/04/2017	0	0	0
2014299912	114	rural/clinic	No	11/04/2017	0	0	0
2015324469	114	rural/clinic	No	11/04/2017	0	0	0
2015324470	114	rural/clinic	No	11/04/2017	0	0	0
201066581	114	rural/clinic	No	11/04/2017	0	0	0
2012388384	114	rural/clinic	No	11/04/2017	0	0	0
2014299913	114	rural/clinic	No	11/04/2017	0	0	0
2012388385	114	rural/clinic	No	11/04/2017	0	0	0
2014299910	114	rural/clinic	No	11/04/2017	0	0	0
2014299911	114	rural/clinic	No	11/04/2017	0	0	0
2014288421	114	rural/clinic	No	11/04/2017	0	0	0
2013254803	114	rural/clinic	No	11/04/2017	0	0	0
2015324432	114	rural/clinic	No	11/04/2017	0	0	0
2014311548	114	rural/clinic	No	11/04/2017	0	0	0
2014299916	114	rural/clinic	No	11/04/2017	0	0	0
2014299915	114	rural/clinic	No	11/04/2017	0	0	0
2014299914	114	rural/clinic	No	11/04/2017	0	0	0
2015337365	114	rural/clinic	No	11/04/2017	0	0	0
2015337366	114	rural/clinic	No	11/04/2017	0	0	0
2012345099	114	rural/clinic	No	11/04/2017	0	0	0
2015337617	114	rural/clinic	No	11/04/2017	0	0	0
2015337305	114	rural/clinic	No	11/04/2017	0	0	0
2015316000	114	rural/clinic	No	11/04/2017	0	0	0
2015384781	114	rural/clinic	No	11/04/2017	0	0	0
2015351965	114	rural/clinic	No	13/04/2017	0	0	0
2015351966	114	rural/clinic	No	13/04/2017	0	0	0
2015351967	114	rural/clinic	No	13/04/2017	0	0	0
2015351968	114	rural/clinic	No	19/04/2017	0	0	0
2015351969	114	rural/clinic	No	13/04/2017	0	0	0
2015351970	114	rural/clinic	No	13/04/2017	0	0	0
2015351971	114	rural/clinic	No	13/04/2017	0	0	0
2015351972	114	rural/clinic	No	13/04/2017	0	0	0
2015351973	114	rural/clinic	No	13/04/2017	0	0	0
2015351974	114	rural/clinic	No	13/04/2017	0	0	0
2015351680	114	rural/clinic	No	13/04/2017	0	0	0
2015326491	114	rural/clinic	No	13/04/2017	0	0	0
2015400019	114	rural/clinic	No	13/04/2017	0	0	0
2015326492	114	rural/clinic	No	13/04/2017	0	0	0
2015400022	114	rural/clinic	No	13/04/2017	0	0	0
2015326493	243	rural/clinic	No	13/04/2017	0	0	0
2015400023	243	rural/clinic	No	13/04/2017	0	0	0
2015326494	243	rural/clinic	No	13/04/2017	0	0	0
2015400024	243	rural/clinic	No	13/04/2017	0	0	0
2015326495	243	rural/clinic	No	13/04/2017	0	0	0
2014400025	243	rural/clinic	No	13/04/2017	0	0	0
2015326496	243	rural/clinic	No	13/04/2017	0	0	0
2015400026	243	rural/clinic	No	13/04/2017	0	0	0
2015326497	243	rural/clinic	No	13/04/2017	0	0	0
2015400027	243	rural/clinic	No	13/04/2017	0	0	0
2015326498	243	rural/clinic	No	13/04/2017	0	0	0
2015400028	243	rural/clinic	No	13/04/2017	0	0	0
2015326534	243	rural/clinic	No	13/04/2017	0	0	0
2015400029	243	rural/clinic	No	13/04/2017	0	0	0
2015326535	243	rural/clinic	No	13/04/2017	0	0	0
2015349946	243	rural/clinic	No	13/04/2017	0	0	0
2015364506	243	rural/clinic	No	13/04/2017	0	0	0
2011229214	243	rural/clinic	No	13/04/2017	0	0	0
2015400031	243	rural/clinic	No	13/04/2017	0	0	0
2014287174	243	rural/clinic	No	13/04/2017	0	0	0
2015400030	243	rural/clinic	No	13/04/2017	0	0	0
2015301464	243	rural/clinic	No	13/04/2017	0	0	0
2014287175	243	rural/clinic	No	13/04/2017	0	0	0
2012268524/D	243	rural/clinic	No	13/04/2017	0	0	0
2015325986	243	rural/clinic	No	13/04/2017	0	0	0
2012268525	243	rural/clinic	No	13/04/2017	0	0	0
2015349948	243	rural/clinic	No	13/04/2017	0	0	0
2013284285	243	rural/clinic	No	13/04/2017	0	0	0
2015301465	243	rural/clinic	No	13/04/2017	0	0	0
2015301466	243	rural/clinic	No	13/04/2017	0	0	0
2013284286	243	rural/clinic	No	13/04/2017	0	0	0
2015325996	243	rural/clinic	No	13/04/2017	0	0	0
2013272759	243	rural/clinic	No	13/04/2017	0	0	0
2015325997	243	rural/clinic	No	13/04/2017	0	0	0

2015415261	243	rural/clinic	No	13/04/2017	0	0	0
2015351625	243	rural/clinic	No	13/04/2017	0	0	0
2015355393	243	rural/clinic	No	13/04/2017	0	0	0
2015351626	243	rural/clinic	No	13/04/2017	0	0	0
2015355392	243	rural/clinic	No	13/04/2017	0	0	0
2015351627	243	rural/clinic	No	13/04/2017	0	0	0
2015355394	243	rural/clinic	No	13/04/2017	0	0	0
2015351628	243	rural/clinic	No	13/04/2017	0	0	0
2015286294	243	rural/clinic	No	13/04/2017	0	0	0
2015351629	243	rural/clinic	No	13/04/2017	0	0	0
2014289657	243	rural/clinic	No	13/04/2017	0	0	0
2015351630	243	rural/clinic	No	13/04/2017	0	0	0
2015351631	243	rural/clinic	No	13/04/2017	0	0	0
2015286704	243	rural/clinic	No	13/04/2017	0	0	0
2015351632	243	rural/clinic	No	13/04/2017	0	0	0
2015286293	243	rural/clinic	No	13/04/2017	0	0	0
2015351633	243	rural/clinic	No	13/04/2017	0	0	0
2014338553	243	rural/clinic	No	13/04/2017	0	0	0
2015351634	243	rural/clinic	No	13/04/2017	0	0	0
2015378856	243	rural/clinic	No	13/04/2017	0	0	0
2015351635	243	rural/clinic	No	19/04/2017	0	0	0
2015286703	243	rural/clinic	No	13/04/2017	0	0	0
2012359924	243	rural/clinic	No	13/04/2017	0	0	0
2014319522	243	rural/clinic	No	13/04/2017	0	0	0
2012359925	243	rural/clinic	No	13/04/2017	0	0	0
2015385524	243	rural/clinic	No	13/04/2017	0	0	0
2015338754	243	rural/clinic	No	13/04/2017	0	0	0
2012336611	899	rural/clinic	No	13/04/2017	0	0	0
2012336612	899	rural/clinic	No	13/04/2017	0	0	0
2015338755	899	rural/clinic	No	13/04/2017	0	0	0
2012336613	899	rural/clinic	No	13/04/2017	0	0	0
2015351425	899	rural/clinic	No	13/04/2017	0	0	0
2014369046	899	rural/clinic	No	13/04/2017	0	0	0
2015351426	899	rural/clinic	No	13/04/2017	0	0	0
2015351427	899	rural/clinic	No	13/04/2017	0	0	0
2015313315	899	rural/clinic	No	13/04/2017	0	0	0
2015369699	899	rural/clinic	No	13/04/2017	0	0	0
2015313316	899	rural/clinic	No	13/04/2017	0	0	0
2015313317	899	rural/clinic	No	13/04/2017	0	0	0
2015369694	899	rural/clinic	No	13/04/2017	0	0	0
2015313318	899	rural/clinic	No	13/04/2017	0	0	0
2015313319	899	rural/clinic	No	13/04/2017	0	0	0
2011203843	899	rural/clinic	No	13/04/2017	0	0	0
2014346312	899	rural/clinic	No	13/04/2017	0	0	0
2015415259	899	rural/clinic	No	13/04/2017	0	0	0
2014335526	899	rural/clinic	No	13/04/2017	0	0	0
2015415260	899	rural/clinic	No	13/04/2017	0	0	0
2014372440	899	rural/clinic	No	13/04/2017	0	0	0
2012389495	899	rural/clinic	No	13/04/2017	0	0	0
2014372441	899	rural/clinic	No	13/04/2017	0	0	0
2014358275	899	rural/clinic	No	13/04/2017	0	0	0
2014303077	899	rural/clinic	No	13/04/2017	0	0	0
2012389496	899	rural/clinic	No	13/04/2017	0	0	0
2014303078	899	rural/clinic	No	13/04/2017	0	0	0
2015415909	899	rural/clinic	No	13/04/2017	0	0	0
2014303079	899	rural/clinic	No	13/04/2017	0	0	0
2011203834	899	rural/clinic	No	13/04/2017	0	0	0
2011203833	899	rural/clinic	No	13/04/2017	0	0	0
2014370431	899	rural/clinic	No	13/04/2017	0	0	0
2014378479	899	rural/clinic	No	13/04/2017	0	0	0
2015413125	899	rural/clinic	No	13/04/2017	0	0	0
2011203832	899	rural/clinic	No	13/04/2017	0	0	0
2015413126	899	rural/clinic	No	13/04/2017	0	0	0
2011203841	899	rural/clinic	No	13/04/2017	0	0	0
2015413127	899	rural/clinic	No	13/04/2017	0	0	0
2011203842	899	rural/clinic	No	13/04/2017	0	0	0
2015413128	899	rural/clinic	No	13/04/2017	0	0	0
2015408111	899	rural/clinic	No	13/04/2017	0	0	0
2015415257	899	rural/clinic	No	13/04/2017	0	0	0
2015415908	899	rural/clinic	No	13/04/2017	0	0	0
2015413129	899	rural/clinic	No	13/04/2017	0	0	0
2015415258	899	rural/clinic	No	13/04/2017	0	0	0
2015413130	899	rural/clinic	No	13/04/2017	0	0	0
2014317343	899	rural/clinic	No	13/04/2017	0	0	0
2015413131	899	rural/clinic	No	13/04/2017	0	0	0
2014314548	899	rural/clinic	No	13/04/2017	0	0	0
2014314211	899	rural/clinic	No	13/04/2017	0	0	0
2012369805	899	rural/clinic	No	13/04/2017	0	0	0
2015300691	899	rural/clinic	No	13/04/2017	0	0	0
2014328762	899	rural/clinic	No	13/04/2017	0	0	0
2011232696	899	rural/clinic	No	13/04/2017	0	0	0
2014334366	452	rural/clinic	No	13/04/2017	0	0	0

2012263747	452	rural/clinic	No	13/04/2017	0	0	0
2015289173	452	rural/clinic	No	13/04/2017	0	0	0
2011232695	452	rural/clinic	No	13/04/2017	0	0	0
2014303081	452	rural/clinic	No	13/04/2017	0	0	0
2011232697	452	rural/clinic	No	13/04/2017	0	0	0
2014303082	452	rural/clinic	No	13/04/2017	0	0	0
2015331925	452	rural/clinic	No	13/04/2017	0	0	0
2015331926	452	rural/clinic	No	13/04/2017	0	0	0
2015331927	452	rural/clinic	No	13/04/2017	0	0	0
2015369394	452	rural/clinic	No	13/04/2017	0	0	0
2015369396	452	rural/clinic	No	13/04/2017	0	0	0
2015355909	452	rural/clinic	No	13/04/2017	0	0	0
2015369397	452	rural/clinic	No	13/04/2017	0	0	0
2015355910	452	rural/clinic	No	13/04/2017	0	0	0
2014317624	452	rural/clinic	No	13/04/2017	0	0	0
2015340966	452	rural/clinic	No	13/04/2017	0	0	0
201053902	452	rural/clinic	No	13/04/2017	0	0	0
2015340967	452	rural/clinic	No	13/04/2017	0	0	0
201053901	452	rural/clinic	No	13/04/2017	0	0	0
2014357525	452	rural/clinic	No	13/04/2017	0	0	0
2014317623	452	rural/clinic	No	13/04/2017	0	0	0
2014357526	452	rural/clinic	No	13/04/2017	0	0	0
2014357527	452	rural/clinic	No	13/04/2017	0	0	0
2014317432	452	rural/clinic	No	13/04/2017	0	0	0
2014357528	452	rural/clinic	No	13/04/2017	0	0	0
2015377431	452	rural/clinic	No	13/04/2017	0	0	0
2013263590	452	rural/clinic	No	13/04/2017	0	0	0
2015377427	452	rural/clinic	No	13/04/2017	0	0	0
2013263591	452	rural/clinic	No	13/04/2017	0	0	0
2015377428	452	rural/clinic	No	13/04/2017	0	0	0
2013263592	452	rural/clinic	No	13/04/2017	0	0	0
2015377430	452	rural/clinic	No	13/04/2017	0	0	0
2015373360	34	rural/clinic	No	13/04/2017	0	0	0
2015373358	34	rural/clinic	No	13/04/2017	0	0	0
2015373357	34	rural/clinic	No	13/04/2017	0	0	0
2015373356	34	rural/clinic	No	13/04/2017	0	0	0
2015373355	34	rural/clinic	No	13/04/2017	0	0	0
2015373354	34	rural/clinic	No	13/04/2017	0	0	0
2015373353	34	rural/clinic	No	13/04/2017	0	0	0
2015373362	34	rural/clinic	No	13/04/2017	0	0	0
2015373367	34	rural/clinic	No	13/04/2017	0	0	0
2015373365	34	rural/clinic	No	13/04/2017	0	0	0
2015373366	34	rural/clinic	No	13/04/2017	0	0	0
2015373361	34	rural/clinic	No	13/04/2017	0	0	0
2015373363	34	rural/clinic	No	13/04/2017	0	0	0
2015373368	34	rural/clinic	No	13/04/2017	0	0	0
2015373369	34	rural/clinic	No	13/04/2017	0	0	0
2015373364	34	rural/clinic	No	13/04/2017	0	0	0
2015373373	34	rural/clinic	No	13/04/2017	0	0	0
2015373372	34	rural/clinic	No	13/04/2017	0	0	0
2015373371	34	rural/clinic	No	13/04/2017	0	0	0
2015373370	34	rural/clinic	No	13/04/2017	0	0	0
2015373376	34	rural/clinic	No	13/04/2017	0	0	0
2015373375	34	rural/clinic	No	13/04/2017	0	0	0
2015373374	34	rural/clinic	No	13/04/2017	0	0	0
2015373377	34	rural/clinic	No	13/04/2017	0	0	0
2015373381	34	rural/clinic	No	13/04/2017	0	0	0
2015373380	34	rural/clinic	No	13/04/2017	0	0	0
2015373379	34	rural/clinic	No	13/04/2017	0	0	0
2015373378	34	rural/clinic	No	13/04/2017	0	0	0
2015373386	34	rural/clinic	No	13/04/2017	0	0	0
2015373385	34	rural/clinic	No	13/04/2017	0	0	0
2015373384	34	rural/clinic	No	13/04/2017	0	0	0
2015373383	34	rural/clinic	No	13/04/2017	0	0	0
2015373382	34	rural/clinic	No	13/04/2017	0	0	0
2015373388	34	rural/clinic	No	13/04/2017	0	0	0
2015373387	34	rural/clinic	No	13/04/2017	0	0	0
2015373390	34	rural/clinic	No	13/04/2017	0	0	0
2015373391	34	rural/clinic	No	13/04/2017	0	0	0
2015373392	34	rural/clinic	No	13/04/2017	0	0	0
2015373393	34	rural/clinic	No	13/04/2017	0	0	0
2015373394	34	rural/clinic	No	13/04/2017	0	0	0
2015373389	34	rural/clinic	No	13/04/2017	0	0	0
2015373395	34	rural/clinic	No	13/04/2017	0	0	0
2015373396	34	rural/clinic	No	13/04/2017	0	0	0
2015373398	34	rural/clinic	No	13/04/2017	0	0	0
2015373399	34	rural/clinic	No	13/04/2017	0	0	0
2015373400	34	rural/clinic	No	13/04/2017	0	0	0
2015351608	34	rural/clinic	No	13/04/2017	0	0	0
2015351607	34	rural/clinic	No	13/04/2017	0	0	0
2015351606	34	rural/clinic	No	13/04/2017	0	0	0
2015351605	34	rural/clinic	No	13/04/2017	0	0	0

2015351604	34	rural/clinic	No	13/04/2017	0	0	0
2015351603	34	rural/clinic	No	13/04/2017	0	0	0
2015351602	34	rural/clinic	No	13/04/2017	0	0	0
2015351601	34	rural/clinic	No	13/04/2017	0	0	0
2015351608	34	rural/clinic	No	13/04/2017	0	0	0
2015351615	34	rural/clinic	No	13/04/2017	0	0	0
2015351616	34	rural/clinic	No	13/04/2017	0	0	0
2015351614	34	rural/clinic	No	13/04/2017	0	0	0
2015351613	34	rural/clinic	No	13/04/2017	0	0	0
2015351611	34	rural/clinic	No	13/04/2017	0	0	0
2015351609	34	rural/clinic	No	13/04/2017	0	0	0
2015351610	34	rural/clinic	No	13/04/2017	0	0	0
2015351612	34	rural/clinic	No	13/04/2017	0	0	0
2015373407	34	rural/clinic	No	13/04/2017	0	0	0
2015351617	34	rural/clinic	No	13/04/2017	0	0	0
2015351624	34	rural/clinic	No	13/04/2017	0	0	0
2015351623	34	rural/clinic	No	13/04/2017	0	0	0
2015351622	34	rural/clinic	No	13/04/2017	0	0	0
2015351620	34	rural/clinic	No	13/04/2017	0	0	0
2015351619	34	rural/clinic	No	13/04/2017	0	0	0
2015351618	34	rural/clinic	No	13/04/2017	0	0	0
2015351625	34	rural/clinic	No	13/04/2017	0	0	0
2015351626	34	rural/clinic	No	13/04/2017	0	0	0
2015351627	34	rural/clinic	No	13/04/2017	0	0	0
2015351628	34	rural/clinic	No	13/04/2017	0	0	0
2015351629	34	rural/clinic	No	13/04/2017	0	0	0
2015351630	34	rural/clinic	No	13/04/2017	0	0	0
2015351631	34	rural/clinic	No	13/04/2017	0	0	0
2015351632	34	rural/clinic	No	13/04/2017	0	0	0
2015351633	34	rural/clinic	No	13/04/2017	0	0	0
2015351634	34	rural/clinic	No	13/04/2017	0	0	0
2015351635	34	rural/clinic	No	13/04/2017	0	0	0
2015351643	34	rural/clinic	No	13/04/2017	0	0	0
2015351644	34	rural/clinic	No	13/04/2017	0	0	0
2015351645	34	rural/clinic	No	13/04/2017	0	0	0
2015351646	34	rural/clinic	No	13/04/2017	0	0	0
2015351647	34	rural/clinic	No	13/04/2017	0	0	0
2015351648	34	rural/clinic	No	13/04/2017	0	0	0
2015351649	34	rural/clinic	No	13/04/2017	0	0	0
2015351636	34	rural/clinic	No	13/04/2017	0	0	0
2015351637	34	rural/clinic	No	13/04/2017	0	0	0
2015351638	34	rural/clinic	No	13/04/2017	0	0	0
2015351639	34	rural/clinic	No	13/04/2017	0	0	0
2015351640	34	rural/clinic	No	13/04/2017	0	0	0
2015351641	34	rural/clinic	No	13/04/2017	0	0	0
2015351642	34	rural/clinic	No	13/04/2017	0	0	0
2015351648	34	rural/clinic	No	13/04/2017	0	0	0
2015351648	34	rural/clinic	No	13/04/2017	0	0	0
2015373402	34	rural/clinic	No	13/04/2017	0	0	0
2015373403	34	rural/clinic	No	13/04/2017	0	0	0
2015373404	34	rural/clinic	No	13/04/2017	0	0	0
2015373405	34	rural/clinic	No	13/04/2017	0	0	0
2015373406	34	rural/clinic	No	13/04/2017	0	0	0
2015373408	34	rural/clinic	No	13/04/2017	0	0	0
2015373410	34	rural/clinic	No	13/04/2017	0	0	0
2015373409	34	rural/clinic	No	13/04/2017	0	0	0
2015373411	34	rural/clinic	No	13/04/2017	0	0	0
2015351650	34	rural/clinic	No	13/04/2017	0	0	0
2015373401	34	rural/clinic	No	13/04/2017	0	0	0
2015373412	34	rural/clinic	No	13/04/2017	0	0	0
2015373413	34	rural/clinic	No	13/04/2017	0	0	0
2015373414	34	rural/clinic	No	13/04/2017	0	0	0
2015373415	34	rural/clinic	No	13/04/2017	0	0	0
2015373416	34	rural/clinic	No	13/04/2017	0	0	0
2015373417	34	rural/clinic	No	13/04/2017	0	0	0
2015373418	34	rural/clinic	No	13/04/2017	0	0	0
2015373419	34	rural/clinic	No	13/04/2017	0	0	0
2015373420	34	rural/clinic	No	13/04/2017	0	0	0
2015373421	34	rural/clinic	No	13/04/2017	0	0	0
2015373422	34	rural/clinic	No	13/04/2017	0	0	0
2015373423	34	rural/clinic	No	13/04/2017	0	0	0
2015373424	34	rural/clinic	No	13/04/2017	0	0	0
2015373425	34	rural/clinic	No	13/04/2017	0	0	0
2015373426	34	rural/clinic	No	13/04/2017	0	0	0
2015373427	34	rural/clinic	No	13/04/2017	0	0	0
2015373428	34	rural/clinic	No	13/04/2017	0	0	0
2015373429	34	rural/clinic	No	13/04/2017	0	0	0
2015373430	34	rural/clinic	No	13/04/2017	0	0	0
2015373431	34	rural/clinic	No	13/04/2017	0	0	0
2015373432	34	rural/clinic	No	13/04/2017	0	0	0
2015373433	34	rural/clinic	No	13/04/2017	0	0	0
2015373434	34	rural/clinic	No	13/04/2017	0	0	0

2015373435	34	rural/clinic	No	13/04/2017	0	0	0
2015373436	34	rural/clinic	No	13/04/2017	0	0	0
2015373437	34	rural/clinic	No	13/04/2017	0	0	0
2015373438	34	rural/clinic	No	13/04/2017	0	0	0
2015373439	34	rural/clinic	No	13/04/2017	0	0	0
2015373440	34	rural/clinic	No	13/04/2017	0	0	0
2015373441	34	rural/clinic	No	13/04/2017	0	0	0
2015373442	34	rural/clinic	No	13/04/2017	0	0	0
2015373443	34	rural/clinic	No	13/04/2017	0	0	0
2015373444	34	rural/clinic	No	13/04/2017	0	0	0
2015373440	34	rural/clinic	No	13/04/2017	0	0	0
2015373440	34	rural/clinic	No	13/04/2017	0	0	0
2015373449	34	rural/clinic	No	13/04/2017	0	0	0
2015373448	34	rural/clinic	No	13/04/2017	0	0	0
2015373447	34	rural/clinic	No	13/04/2017	0	0	0
2015373446	34	rural/clinic	No	13/04/2017	0	0	0
2015373445	34	rural/clinic	No	13/04/2017	0	0	0
2015406002	34	rural/clinic	No	13/04/2017	0	0	0
2015406001	34	rural/clinic	No	28/03/2017	0	0	0
2015373450	34	rural/clinic	No	13/04/2017	0	0	0
2015406003	34	rural/clinic	No	07/04/2017	0	0	0
2015406004	34	rural/clinic	No	07/04/2017	0	0	0
2015406007	34	rural/clinic	No	13/04/2017	0	0	0
2015406006	34	rural/clinic	No	06/04/2017	0	0	0
2015406005	34	rural/clinic	No	06/04/2017	0	0	0
2015406011	34	rural/clinic	No	06/04/2017	0	0	0
2015406009	34	rural/clinic	No	06/04/2017	0	0	0
2015406010	34	rural/clinic	No	06/04/2017	0	0	0
2015406008	34	rural/clinic	No	06/04/2017	0	0	0
2015406013	34	rural/clinic	No	06/04/2017	0	0	0
2015406018	34	rural/clinic	No	06/04/2017	0	0	0
2015406017	34	rural/clinic	No	13/04/2017	0	0	0
2015406016	34	rural/clinic	No	13/04/2017	0	0	0
2015406015	34	rural/clinic	No	13/04/2017	0	0	0
2015406014	34	rural/clinic	No	06/04/2017	0	0	0
2015406038	34	rural/clinic	No	13/04/2017	0	0	0
2015406031	34	rural/clinic	No	13/04/2017	0	0	0
2015406036	34	rural/clinic	No	13/04/2017	0	0	0
2015406035	34	rural/clinic	No	13/04/2017	0	0	0
2015406032	34	rural/clinic	No	13/04/2017	0	0	0
2015406037	34	rural/clinic	No	13/04/2017	0	0	0
2015406033	34	rural/clinic	No	13/04/2017	0	0	0
2015406034	34	rural/clinic	No	13/04/2017	0	0	0
2015406047	34	rural/clinic	No	13/04/2017	0	0	0
2015406046	34	rural/clinic	No	13/04/2017	0	0	0
2015406045	34	rural/clinic	No	13/04/2017	0	0	0
2015406044	34	rural/clinic	No	13/04/2017	0	0	0
2015406043	34	rural/clinic	No	13/04/2017	0	0	0
2015406042	34	rural/clinic	No	13/04/2017	0	0	0
2015406041	34	rural/clinic	No	13/04/2017	0	0	0
2015406040	34	rural/clinic	No	13/04/2017	0	0	0
2015406039	34	rural/clinic	No	13/04/2017	0	0	0
2015406019	34	rural/clinic	No	13/04/2017	0	0	0
2015406021	34	rural/clinic	No	13/04/2017	0	0	0
2015406022	34	rural/clinic	No	13/04/2017	0	0	0
2015406023	34	rural/clinic	No	13/04/2017	0	0	0
2015406024	34	rural/clinic	No	13/04/2017	0	0	0
2015406025	34	rural/clinic	No	13/04/2017	0	0	0
2015406026	34	rural/clinic	No	13/04/2017	0	0	0
2015406027	34	rural/clinic	No	13/04/2017	0	0	0
2015406028	34	rural/clinic	No	13/04/2017	0	0	0
2015406029	34	rural/clinic	No	13/04/2017	0	0	0
2015406030	34	rural/clinic	No	13/04/2017	0	0	0
2015406047	34	rural/clinic	No	11/04/2017	0	0	0
2015406047	34	rural/clinic	No	11/04/2017	0	0	0
2015406050	34	rural/clinic	No	11/04/2017	0	0	0
2015406049	34	rural/clinic	No	11/04/2017	0	0	0
2015406048	34	rural/clinic	No	11/04/2017	0	0	0
2015406056	34	rural/clinic	No	11/04/2017	0	0	0
2015406055	34	rural/clinic	No	11/04/2017	0	0	0
2015406054	34	rural/clinic	No	11/04/2017	0	0	0
2015406053	34	rural/clinic	No	11/04/2017	0	0	0
2015406052	34	rural/clinic	No	11/04/2017	0	0	0
2015406051	34	rural/clinic	No	11/04/2017	0	0	0
2015406059	34	rural/clinic	No	11/04/2017	0	0	0
2015406062	34	rural/clinic	No	11/04/2017	0	0	0
2015406063	34	rural/clinic	No	11/04/2017	0	0	0
2015406064	34	rural/clinic	No	11/04/2017	0	0	0
2015406060	34	rural/clinic	No	11/04/2017	0	0	0
2015406057	34	rural/clinic	No	11/04/2017	0	0	0
2015406058	34	rural/clinic	No	11/04/2017	0	0	0
2015406061	34	rural/clinic	No	11/04/2017	0	0	0

2015406068	34	rural/clinic	No	11/04/2017	0	0	0
2015406067	34	rural/clinic	No	11/04/2017	0	0	0
2015406065	34	rural/clinic	No	11/04/2017	0	0	0
2015406066	34	rural/clinic	No	11/04/2017	0	0	0
2015406080	34	rural/clinic	No	11/04/2017	0	0	0
2015406081	34	rural/clinic	No	11/04/2017	0	0	0
2015406082	34	rural/clinic	No	11/04/2017	0	0	0
2015406083	34	rural/clinic	No	11/04/2017	0	0	0
2015406084	34	rural/clinic	No	11/04/2017	0	0	0
2015406073	34	rural/clinic	No	11/04/2017	0	0	0
2015406070	34	rural/clinic	No	11/04/2017	0	0	0
2015406071	34	rural/clinic	No	11/04/2017	0	0	0
2015406072	34	rural/clinic	No	11/04/2017	0	0	0
2015406074	34	rural/clinic	No	11/04/2017	0	0	0
2015406076	34	rural/clinic	No	11/04/2017	0	0	0
2015406077	34	rural/clinic	No	11/04/2017	0	0	0
2015406078	34	rural/clinic	No	11/04/2017	0	0	0
2015406079	34	rural/clinic	No	11/04/2017	0	0	0
2015406083	34	rural/clinic	No	11/04/2017	0	0	0
2015406084	34	rural/clinic	No	11/04/2017	0	0	0
2015406087	34	rural/clinic	No	11/04/2017	0	0	0
2015406086	34	rural/clinic	No	11/04/2017	0	0	0
2015406085	34	rural/clinic	No	11/04/2017	0	0	0
2015406085	34	rural/clinic	No	11/04/2017	0	0	0
2015406085	34	rural/clinic	No	11/04/2017	0	0	0
2015406088	34	rural/clinic	No	11/04/2017	0	0	0
2015406089	34	rural/clinic	No	11/04/2017	0	0	0
2015406090	34	rural/clinic	No	11/04/2017	0	0	0
2015406091	34	rural/clinic	No	11/04/2017	0	0	0
2015406092	34	rural/clinic	No	11/04/2017	0	0	0
2015406093	34	rural/clinic	No	11/04/2017	0	0	0
2015406094	34	rural/clinic	No	11/04/2017	0	0	0
2015406095	34	rural/clinic	No	11/04/2017	0	0	0
2015406096	34	rural/clinic	No	11/04/2017	0	0	0
2015406097	34	rural/clinic	No	11/04/2017	0	0	0
2015406098	34	rural/clinic	No	11/04/2017	0	0	0
2015406099	34	rural/clinic	No	11/04/2017	0	0	0
2015406098	34	rural/clinic	No	11/04/2017	0	0	0
2015406098	34	rural/clinic	No	11/04/2017	0	0	0
2015338957	34	rural/clinic	No	11/04/2017	0	0	0
2015338956	34	rural/clinic	No	11/04/2017	0	0	0
2015338955	34	rural/clinic	No	11/04/2017	0	0	0
2015338954	34	rural/clinic	No	11/04/2017	0	0	0
2015338953	34	rural/clinic	No	11/04/2017	0	0	0
2015338952	34	rural/clinic	No	11/04/2017	0	0	0
2015406100	34	rural/clinic	No	11/04/2017	0	0	0
2015338964	34	rural/clinic	No	11/04/2017	0	0	0
2015338963	34	rural/clinic	No	11/04/2017	0	0	0
2015338962	34	rural/clinic	No	11/04/2017	0	0	0
2015338961	34	rural/clinic	No	11/04/2017	0	0	0
2015338960	34	rural/clinic	No	11/04/2017	0	0	0
2015338959	34	rural/clinic	No	11/04/2017	0	0	0
2015338958	34	rural/clinic	No	11/04/2017	0	0	0
2015338968	34	rural/clinic	No	11/04/2017	0	0	0
2015338967	34	rural/clinic	No	11/04/2017	0	0	0
2015338966	34	rural/clinic	No	11/04/2017	0	0	0
2015338965	34	rural/clinic	No	11/04/2017	0	0	0
2015338969	34	rural/clinic	No	11/04/2017	0	0	0
2015338970	34	rural/clinic	No	11/04/2017	0	0	0
2015338971	34	rural/clinic	No	11/04/2017	0	0	0
2015338972	34	rural/clinic	No	11/04/2017	0	0	0
2015338973	34	rural/clinic	No	11/04/2017	0	0	0
2015338974	34	rural/clinic	No	11/04/2017	0	0	0
2015338975	34	rural/clinic	No	11/04/2017	0	0	0
2015338976	34	rural/clinic	No	11/04/2017	0	0	0
2015338977	34	rural/clinic	No	11/04/2017	0	0	0
2015338978	34	rural/clinic	No	11/04/2017	0	0	0
2015338979	34	rural/clinic	No	11/04/2017	0	0	0
2015338980	34	rural/clinic	No	11/04/2017	0	0	0
2015338981	34	rural/clinic	No	11/04/2017	0	0	0
2015338982	34	rural/clinic	No	11/04/2017	0	0	0
2015338983	34	rural/clinic	No	11/04/2017	0	0	0
2015338984	34	rural/clinic	No	11/04/2017	0	0	0
2015338983	34	rural/clinic	No	11/04/2017	0	0	0
2015338983	34	rural/clinic	No	11/04/2017	0	0	0
2015338989	34	rural/clinic	No	11/04/2017	0	0	0
2015338988	34	rural/clinic	No	11/04/2017	0	0	0
2015338987	34	rural/clinic	No	11/04/2017	0	0	0
2015338986	34	rural/clinic	No	11/04/2017	0	0	0
2015338985	34	rural/clinic	No	11/04/2017	0	0	0
2015313172	34	rural/clinic	No	11/04/2017	0	0	0
2014370423	31	rural/clinic	No	11/04/2017	0	0	0

2014370422	31	rural/clinic	No	11/04/2017	0	0	0
2014370424	31	rural/clinic	No	11/04/2017	0	0	0
2014370426	31	rural/clinic	No	11/04/2017	0	0	0
2014370425	31	rural/clinic	No	11/04/2017	0	0	0
2014370428	31	rural/clinic	No	11/04/2017	0	0	0
2014370430	31	rural/clinic	No	11/04/2017	0	0	0
2014370429	31	rural/clinic	No	11/04/2017	0	0	0
2014370431	31	rural/clinic	No	11/04/2017	0	0	0
2014370434	31	rural/clinic	No	11/04/2017	0	0	0
2014370432	31	rural/clinic	No	11/04/2017	0	0	0
2014370433	31	rural/clinic	No	11/04/2017	0	0	0
2014370435	31	rural/clinic	No	11/04/2017	0	0	0
2014370436	31	rural/clinic	No	06/04/2017	0	0	0
2014370437	31	rural/clinic	No	06/04/2017	0	0	0
2014370438	31	rural/clinic	No	11/04/2017	0	0	0
2014370439	31	rural/clinic	No	11/04/2017	0	0	0
2014370440	31	rural/clinic	No	11/04/2017	0	0	0
2014370441	31	rural/clinic	No	11/04/2017	0	0	0
2015406069	31	rural/clinic	No	11/04/2017	0	0	0
2014370442	31	rural/clinic	No	11/04/2017	0	0	0
2014370446	31	rural/clinic	No	11/04/2017	0	0	0
2014370444	31	rural/clinic	No	11/04/2017	0	0	0
2014370447	31	rural/clinic	No	11/04/2017	0	0	0
2014370445	31	rural/clinic	No	11/04/2017	0	0	0
2015376201	115	rural/clinic	No	11/04/2017	0	0	0
2015376202	115	rural/clinic	No	19/04/2017	0	0	0
2012329102	115	rural/clinic	No	19/04/2017	0	0	0
2015376203	115	rural/clinic	No	19/04/2017	0	0	0
2015376205	115	rural/clinic	No	19/04/2017	0	0	0
2015376208	115	rural/clinic	No	19/04/2017	0	0	0
2015376207	115	rural/clinic	No	19/04/2017	0	0	0
2015376206	115	rural/clinic	No	19/04/2017	0	0	0
2015376211	115	rural/clinic	No	13/04/2017	0	0	0
2015376209	115	rural/clinic	No	13/04/2017	0	0	0
2015376210	115	rural/clinic	No	13/04/2017	0	0	0
2015376206	115	rural/clinic	No	13/04/2017	0	0	0
2015376213	115	rural/clinic	No	13/04/2017	0	0	0
2015376212	115	rural/clinic	No	13/04/2017	0	0	0
2015376214	115	rural/clinic	No	13/04/2017	0	0	0
2015376216	115	rural/clinic	No	13/04/2017	0	0	0
2015376218	115	rural/clinic	No	13/04/2017	0	0	0
2015376217	115	rural/clinic	No	13/04/2017	0	0	0
2015376216	115	rural/clinic	No	13/04/2017	0	0	0
2015376215	115	rural/clinic	No	13/04/2017	0	0	0
2015376219	115	rural/clinic	No	13/04/2017	0	0	0
2015376220	115	rural/clinic	No	13/04/2017	0	0	0
2015376222	115	rural/clinic	No	13/04/2017	0	0	0
2015376224	115	rural/clinic	No	13/04/2017	0	0	0
2015376223	115	rural/clinic	No	13/04/2017	0	0	0
2015376221	115	rural/clinic	No	13/04/2017	0	0	0
2015376223	115	rural/clinic	No	13/04/2017	0	0	0
2015376223	115	rural/clinic	No	13/04/2017	0	0	0
2012284022	616	rural/clinic	No	13/04/2017	0	0	0
2012284023	616	district/faith-based	No	13/04/2017	1	0	0
2012284024	616	district/faith-based	No	13/04/2017	1	0	0
2012284025	616	district/faith-based	No	13/04/2017	1	0	0
2012284027	616	district/faith-based	No	13/04/2017	1	0	0
2012284026	616	district/faith-based	No	13/04/2017	1	0	0
2012284028	616	district/faith-based	No	13/04/2017	1	0	0
2012284029	616	district/faith-based	No	13/04/2017	1	0	0
2012284030	616	district/faith-based	No	13/04/2017	1	0	0
20170218951	616	district/faith-based	No	13/04/2017	1	0	0
20170218953	616	district/faith-based	No	13/04/2017	1	0	0
20170218952	616	district/faith-based	No	13/04/2017	1	0	0
20170218954	616	district/faith-based	No	13/04/2017	1	0	0
2015403101	921	rural/clinic	No	13/04/2017	0	0	0
2015403102	921	rural/clinic	No	13/04/2017	0	0	0
2015403104	921	rural/clinic	No	13/04/2017	0	0	0
2015403105	921	rural/clinic	No	13/04/2017	0	0	0
2015403103	921	rural/clinic	No	13/04/2017	0	0	0
2015403106	921	rural/clinic	No	13/04/2017	0	0	0
2015403106	921	rural/clinic	No	13/04/2017	0	0	0
2015403106	921	rural/clinic	No	13/04/2017	0	0	0
2014288566	502	rural/clinic	No	13/04/2017	0	0	0
2014288587	502	rural/clinic	No	13/04/2017	0	0	0
2014288569	502	rural/clinic	No	13/04/2017	0	0	0
2014288568	502	rural/clinic	No	13/04/2017	0	0	0
2014288568	502	rural/clinic	No	13/04/2017	0	0	0
2014288568	502	rural/clinic	No	13/04/2017	0	0	0
2014288571	502	rural/clinic	No	13/04/2017	0	0	0
2014288570	502	rural/clinic	No	13/04/2017	0	0	0
2014288573	502	rural/clinic	No	13/04/2017	0	0	0

2014288574	502	rural/clinic	No	13/04/2017	0	0	0
2014288575	502	rural/clinic	No	13/04/2017	0	0	0
2014288576	502	rural/clinic	No	13/04/2017	0	0	0
2014288577	502	rural/clinic	No	13/04/2017	0	0	0
2014288578	502	rural/clinic	No	13/04/2017	0	0	0
2014288579	502	rural/clinic	No	13/04/2017	0	0	0
2014288580	502	rural/clinic	No	13/04/2017	0	0	0
2014288581	502	rural/clinic	No	13/04/2017	0	0	0
2014288582	502	rural/clinic	No	13/04/2017	0	0	0
2014288588	502	rural/clinic	No	13/04/2017	0	0	0
2014288586	502	rural/clinic	No	13/04/2017	0	0	0
2014288585	502	rural/clinic	No	13/04/2017	0	0	0
2014288584	502	rural/clinic	No	13/04/2017	0	0	0
2014288583	502	rural/clinic	No	13/04/2017	0	0	0
2014288589	502	rural/clinic	No	13/04/2017	0	0	0
2014288587/D	502	rural/clinic	No	13/04/2017	0	0	0
2014288590	502	rural/clinic	No	13/04/2017	0	0	0
2014288591	502	rural/clinic	No	13/04/2017	0	0	0
2015377050	502	rural/clinic	No	13/04/2017	0	0	0
2015335101	502	rural/clinic	No	13/04/2017	0	0	0
2013254391	502	rural/clinic	No	13/04/2017	0	0	0
2015335102	502	rural/clinic	No	13/04/2017	0	0	0
2015335103	502	rural/clinic	No	13/04/2017	0	0	0
2015328469	502	rural/clinic	No	13/04/2017	0	0	0
2015335104	502	rural/clinic	No	13/04/2017	0	0	0
2015328468	423	rural/clinic	No	13/04/2017	0	0	0
2015335105	423	rural/clinic	No	13/04/2017	0	0	0
2015354488	423	rural/clinic	No	13/04/2017	0	0	0
2011123695	423	rural/clinic	No	13/04/2017	0	0	0
2015377320	423	rural/clinic	No	13/04/2017	0	0	0
2015377321	423	rural/clinic	No	13/04/2017	0	0	0
2014337046	423	rural/clinic	No	13/04/2017	0	0	0
2013262231	423	rural/clinic	No	13/04/2017	0	0	0
2015334527	423	rural/clinic	No	13/04/2017	0	0	0
2014300424	423	rural/clinic	No	13/04/2017	0	0	0
2015331724	423	rural/clinic	No	13/04/2017	0	0	0
2015331667	423	rural/clinic	No	13/04/2017	0	0	0
2014297784	423	rural/clinic	No	13/04/2017	0	0	0
2012304328	423	rural/clinic	No	11/04/2017	0	0	0
2012304328	423	rural/clinic	No	11/04/2017	0	0	0
2014300536	423	rural/clinic	No	13/04/2017	0	0	0
2015287356	423	rural/clinic	No	11/04/2017	0	0	0
2015287356	423	rural/clinic	No	11/04/2017	0	0	0
2011140947	423	rural/clinic	No	07/04/2017	0	0	0
2011140947	423	rural/clinic	No	07/04/2017	0	0	0
2014297785	38	rural/clinic	No	13/04/2017	0	0	0
2014297786	38	rural/clinic	No	13/04/2017	0	0	0
2015354487	38	rural/clinic	No	13/04/2017	0	0	0
2015339461	38	rural/clinic	No	13/04/2017	0	0	0
2015354490	38	rural/clinic	No	13/04/2017	0	0	0
2015414558	38	rural/clinic	No	13/04/2017	0	0	0
2015354482	38	rural/clinic	No	13/04/2017	0	0	0
2014300537	38	rural/clinic	No	13/04/2017	0	0	0
2011145395	38	rural/clinic	No	13/04/2017	0	0	0
2012379342	38	rural/clinic	No	13/04/2017	0	0	0
2012379344	38	rural/clinic	No	13/04/2017	0	0	0
2012379343	38	rural/clinic	No	13/04/2017	0	0	0
2012379345	38	rural/clinic	No	13/04/2017	0	0	0
2015314066	38	rural/clinic	No	13/04/2017	0	0	0
2011144026	38	rural/clinic	No	13/04/2017	0	0	0
2011144018	38	rural/clinic	No	13/04/2017	0	0	0
2015314340	38	rural/clinic	No	13/04/2017	0	0	0
2011144024	38	rural/clinic	No	13/04/2017	0	0	0
2011144027	38	rural/clinic	No	13/04/2017	0	0	0
2014314051	38	rural/clinic	No	13/04/2017	0	0	0
2011144025	38	rural/clinic	No	13/04/2017	0	0	0
2015314052	38	rural/clinic	No	13/04/2017	0	0	0
2011144019	38	rural/clinic	No	13/04/2017	0	0	0
2015314054	38	rural/clinic	No	13/04/2017	0	0	0
2011144022	38	rural/clinic	No	13/04/2017	0	0	0
2015314053	38	rural/clinic	No	13/04/2017	0	0	0
2015314056	38	rural/clinic	No	13/04/2017	0	0	0
2011198692	38	rural/clinic	No	13/04/2017	0	0	0
2011198691	38	rural/clinic	No	13/04/2017	0	0	0
2015314057	38	rural/clinic	No	13/04/2017	0	0	0
2011198690	38	rural/clinic	No	13/04/2017	0	0	0
2015314058	38	rural/clinic	No	13/04/2017	0	0	0
2013274246	38	rural/clinic	No	13/04/2017	0	0	0
2013274247	38	rural/clinic	No	13/04/2017	0	0	0
2015326568	38	rural/clinic	No	13/04/2017	0	0	0
2015368275	38	rural/clinic	No	13/04/2017	0	0	0
2015314059	38	rural/clinic	No	13/04/2017	0	0	0

2015368276	38	rural/clinic	No	13/04/2017	0	0	0
2015314060	38	rural/clinic	No	13/04/2017	0	0	0
2015368274	38	rural/clinic	No	13/04/2017	0	0	0
2015368278	38	rural/clinic	No	13/04/2017	0	0	0
2015314061	38	rural/clinic	No	13/04/2017	0	0	0
2015368277	38	rural/clinic	No	13/04/2017	0	0	0
2015314062	38	rural/clinic	No	13/04/2017	0	0	0
2015368279	38	rural/clinic	No	13/04/2017	0	0	0
2015314063	38	rural/clinic	No	13/04/2017	0	0	0
2015326567	38	rural/clinic	No	13/04/2017	0	0	0
2015314055	38	rural/clinic	No	13/04/2017	0	0	0
2015326566	38	rural/clinic	No	13/04/2017	0	0	0
2015314065	38	rural/clinic	No	13/04/2017	0	0	0
2015369690	38	rural/clinic	No	13/04/2017	0	0	0
2015369695	38	rural/clinic	No	13/04/2017	0	0	0
2014291875	38	rural/clinic	No	14/04/2017	0	0	0
2015294352	38	rural/clinic	No	13/04/2017	0	0	0
2014335237	38	rural/clinic	No	13/04/2017	0	0	0
2015369689	38	rural/clinic	No	13/04/2017	0	0	0
2014335236	38	rural/clinic	No	13/04/2017	0	0	0
2015369697	38	rural/clinic	No	13/04/2017	0	0	0
2015358520	38	rural/clinic	No	13/04/2017	0	0	0
2015369696	38	rural/clinic	No	13/04/2017	0	0	0
2015358521	38	rural/clinic	No	13/04/2017	0	0	0
2014358693	38	rural/clinic	No	13/04/2017	0	0	0
2015358524	38	rural/clinic	No	13/04/2017	0	0	0
2014358691	38	rural/clinic	No	13/04/2017	0	0	0
2015358523	38	rural/clinic	No	13/04/2017	0	0	0
2014321521	38	rural/clinic	No	13/04/2017	0	0	0
2015358522	38	rural/clinic	No	13/04/2017	0	0	0
2012336616	38	rural/clinic	No	13/04/2017	0	0	0
2014321520	38	rural/clinic	No	13/04/2017	0	0	0
2012336615	38	rural/clinic	No	13/04/2017	0	0	0
2011232699	38	rural/clinic	No	13/04/2017	0	0	0
2015361401	38	rural/clinic	No	13/04/2017	0	0	0
2011232698	38	rural/clinic	No	13/04/2017	0	0	0
2015361403	38	rural/clinic	No	13/04/2017	0	0	0
2011232676	38	rural/clinic	No	13/04/2017	0	0	0
2015361405	38	rural/clinic	No	13/04/2017	0	0	0
2011232700	38	rural/clinic	No	13/04/2017	0	0	0
2015361407	38	rural/clinic	No	13/04/2017	0	0	0
2011203836	38	rural/clinic	No	13/04/2017	0	0	0
2015361402	38	rural/clinic	No	13/04/2017	0	0	0
2011203837	38	rural/clinic	No	13/04/2017	0	0	0
2011203838	38	rural/clinic	No	13/04/2017	0	0	0
2015361406	38	rural/clinic	No	13/04/2017	0	0	0
2015372021	38	rural/clinic	No	13/04/2017	0	0	0
2015358178	38	rural/clinic	No	13/04/2017	0	0	0
2015358179	38	rural/clinic	No	13/04/2017	0	0	0
2015358180	38	rural/clinic	No	13/04/2017	0	0	0
2012268997	38	rural/clinic	No	13/04/2017	0	0	0
2015386399	38	rural/clinic	No	13/04/2017	0	0	0
2015358177	38	rural/clinic	No	13/04/2017	0	0	0
2015361404	38	rural/clinic	No	13/04/2017	0	0	0
2014365705	38	rural/clinic	No	13/04/2017	0	0	0
2015303181	38	rural/clinic	No	13/04/2017	0	0	0
2015332960	38	rural/clinic	No	13/04/2017	0	0	0
2015332955	38	rural/clinic	No	13/04/2017	0	0	0
2015332962	38	rural/clinic	No	13/04/2017	0	0	0
2015332961	38	rural/clinic	No	13/04/2017	0	0	0
2015332958	38	rural/clinic	No	13/04/2017	0	0	0
2015332957	38	rural/clinic	No	13/04/2017	0	0	0
2015332959	38	rural/clinic	No	13/04/2017	0	0	0
2015324122	38	rural/clinic	No	13/04/2017	0	0	0
2011203840	38	rural/clinic	No	13/04/2017	0	0	0
2011144004	38	rural/clinic	No	13/04/2017	0	0	0
2011144008	38	rural/clinic	No	13/04/2017	0	0	0
2011144010	38	rural/clinic	No	13/04/2017	0	0	0
2012336610	38	rural/clinic	No	13/04/2017	0	0	0
2012259874	38	rural/clinic	No	13/04/2017	0	0	0
2012336614	38	rural/clinic	No	13/04/2017	0	0	0
2015356576	38	rural/clinic	No	13/04/2017	0	0	0
2015356574	38	rural/clinic	No	13/04/2017	0	0	0
2015299785	38	rural/clinic	No	13/04/2017	0	0	0
2015318238	38	rural/clinic	No	13/04/2017	0	0	0
2015316965	38	rural/clinic	No	13/04/2017	0	0	0
2015318237	38	rural/clinic	No	13/04/2017	0	0	0
2015403265	38	rural/clinic	No	13/04/2017	0	0	0
2015403266	38	rural/clinic	No	13/04/2017	0	0	0
2015405096	38	rural/clinic	No	13/04/2017	0	0	0
2012390150	38	rural/clinic	No	13/04/2017	0	0	0
2015382378	38	rural/clinic	No	19/04/2017	0	0	0

2015382376	38	rural/clinic	No	19/04/2017	0	0	0
2015382375	38	rural/clinic	No	19/04/2017	0	0	0
2015382373	38	rural/clinic	No	19/04/2017	0	0	0
2015382374	38	rural/clinic	No	19/04/2017	0	0	0
2015406463	38	rural/clinic	No	19/04/2017	0	0	0
2015406464	38	rural/clinic	No	19/04/2017	0	0	0
2015406465	38	rural/clinic	No	19/04/2017	0	0	0
2015406466	38	rural/clinic	No	19/04/2017	0	0	0
2015406473	38	rural/clinic	No	19/04/2017	0	0	0
2015406474	38	rural/clinic	No	19/04/2017	0	0	0
2015406475	38	rural/clinic	No	19/04/2017	0	0	0
2015406478	38	rural/clinic	No	19/04/2017	0	0	0
2015406479	38	rural/clinic	No	19/04/2017	0	0	0
2015299793	38	rural/clinic	No	19/04/2017	0	0	0
2012300839	38	rural/clinic	No	19/04/2017	0	0	0
2015299792	38	rural/clinic	No	19/04/2017	0	0	0
2015318242	38	rural/clinic	No	19/04/2017	0	0	0
2015319437	38	rural/clinic	No	19/04/2017	0	0	0
2015319439	38	rural/clinic	No	19/04/2017	0	0	0
2015319438	38	rural/clinic	No	19/04/2017	0	0	0
2015319441	38	rural/clinic	No	19/04/2017	0	0	0
2015319440	38	rural/clinic	No	19/04/2017	0	0	0
2013262783	38	rural/clinic	No	19/04/2017	0	0	0
2013262786	38	rural/clinic	No	19/04/2017	0	0	0
2012349281	38	rural/clinic	No	19/04/2017	0	0	0
2013262784	38	rural/clinic	No	19/04/2017	0	0	0
2014291877	38	rural/clinic	No	21/04/2017	0	0	0
2014291876	38	rural/clinic	No	21/04/2017	0	0	0
2014291878	38	rural/clinic	No	21/04/2017	0	0	0
2015334624	38	rural/clinic	No	19/04/2017	0	0	0
2015334621	38	rural/clinic	No	19/04/2017	0	0	0
2015334627	38	rural/clinic	No	19/04/2017	0	0	0
2015334625	38	rural/clinic	No	19/04/2017	0	0	0
2015334626	38	rural/clinic	No	19/04/2017	0	0	0
2011141064	38	rural/clinic	No	19/04/2017	0	0	0
2011141063	38	rural/clinic	No	19/04/2017	0	0	0
2015397918	38	rural/clinic	No	11/04/2017	0	0	0
2015397918	38	rural/clinic	No	11/04/2017	0	0	0
2014328397	38	rural/clinic	No	11/04/2017	0	0	0
2014328397	38	rural/clinic	No	11/04/2017	0	0	0
2014382294	38	rural/clinic	No	11/04/2017	0	0	0
2014382294	38	rural/clinic	No	11/04/2017	0	0	0
2015297319	38	rural/clinic	No	11/04/2017	0	0	0
2015297319	38	rural/clinic	No	11/04/2017	0	0	0
2011141061	38	rural/clinic	No	19/04/2017	0	0	0
2011141062	38	rural/clinic	No	19/04/2017	0	0	0
2017265636	38	rural/clinic	No	06/04/2017	0	0	0
2017265636	38	rural/clinic	No	06/04/2017	0	0	0
2015293945	38	rural/clinic	No	19/04/2017	0	0	0
2011141065	38	rural/clinic	No	19/04/2017	0	0	0
2014375683	38	rural/clinic	No	11/04/2017	0	0	0
2014375683	38	rural/clinic	No	11/04/2017	0	0	0
2014365707	38	rural/clinic	No	19/04/2017	0	0	0
2012346717	38	rural/clinic	No	11/04/2017	0	0	0
2015363058	38	rural/clinic	No	19/04/2017	0	0	0
2015303183	38	rural/clinic	No	19/04/2017	0	0	0
2015315901	38	rural/clinic	No	11/04/2017	0	0	0
2015303182	38	rural/clinic	No	19/04/2017	0	0	0
2014327119	38	rural/clinic	No	11/04/2017	0	0	0
2014327119	38	rural/clinic	No	11/04/2017	0	0	0
2014357817	38	rural/clinic	No	11/04/2017	0	0	0
2014357817	38	rural/clinic	No	11/04/2017	0	0	0
2014365706	38	rural/clinic	No	19/04/2017	0	0	0
2014291620	38	rural/clinic	No	11/04/2017	0	0	0
2014291620	38	rural/clinic	No	11/04/2017	0	0	0
2015418140	38	rural/clinic	No	11/04/2017	0	0	0
2015418140	38	rural/clinic	No	11/04/2017	0	0	0
2015334920	38	rural/clinic	No	11/04/2017	0	0	0
2015334921	38	rural/clinic	No	11/04/2017	0	0	0
2015413591	38	rural/clinic	No	11/04/2017	0	0	0
2015413592	38	rural/clinic	No	11/04/2017	0	0	0
2014253946	38	rural/clinic	No	19/04/2017	0	0	0
2014368846	38	rural/clinic	No	11/04/2017	0	0	0
2014368846	38	rural/clinic	No	11/04/2017	0	0	0
2012321334	38	rural/clinic	No	11/04/2017	0	0	0
2012321334	38	rural/clinic	No	11/04/2017	0	0	0
2013253947	38	rural/clinic	No	19/04/2017	0	0	0
2015369700	38	rural/clinic	No	19/04/2017	0	0	0
2015339824	38	rural/clinic	No	19/04/2017	0	0	0
2013270499	38	rural/clinic	No	19/04/2017	0	0	0
2015339826	38	rural/clinic	No	19/04/2017	0	0	0
2015368882	38	rural/clinic	No	19/04/2017	0	0	0

2015339823	38	rural/clinic	No	19/04/2017	0	0	0
2013270500	38	rural/clinic	No	19/04/2017	0	0	0
2013253945	38	rural/clinic	No	19/04/2017	0	0	0
2015339825	38	rural/clinic	No	19/04/2017	0	0	0
2015368883	38	rural/clinic	No	19/04/2017	0	0	0
2014307546	38	rural/clinic	No	16/05/2017	0	0	0
2015355735	38	rural/clinic	No	11/04/2017	0	0	0
2015355735	38	rural/clinic	No	11/04/2017	0	0	0
2015326535	38	rural/clinic	No	13/04/2017	0	0	0
2015326535	38	rural/clinic	No	13/04/2017	0	0	0
2015293941	38	rural/clinic	No	13/04/2017	0	0	0
2015293941	38	rural/clinic	No	13/04/2017	0	0	0
2014311546	38	rural/clinic	No	13/04/2017	0	0	0
2014372234	38	rural/clinic	No	11/04/2017	0	0	0
2014372234	38	rural/clinic	No	11/04/2017	0	0	0
2015334615	38	rural/clinic	No	11/04/2017	0	0	0
2015334615	38	rural/clinic	No	11/04/2017	0	0	0
2015385524	38	rural/clinic	No	13/04/2017	0	0	0
2015385524	38	rural/clinic	No	13/04/2017	0	0	0
2012336613	38	rural/clinic	No	13/04/2017	0	0	0
2012336613	38	rural/clinic	No	13/04/2017	0	0	0
2014369046	38	rural/clinic	No	13/04/2017	0	0	0
2015400019	38	rural/clinic	No	13/04/2017	0	0	0
2015400019	38	rural/clinic	No	13/04/2017	0	0	0
2014357525	38	rural/clinic	No	13/04/2017	0	0	0
2014357525	38	rural/clinic	No	13/04/2017	0	0	0
2015305069	38	rural/clinic	No	13/04/2017	0	0	0
2015305069	38	rural/clinic	No	13/04/2017	0	0	0
2015305070	38	rural/clinic	No	13/04/2017	0	0	0
2015305070	38	rural/clinic	No	13/04/2017	0	0	0
2015305071	38	rural/clinic	No	13/04/2017	0	0	0
2015305071	38	rural/clinic	No	13/04/2017	0	0	0
2014314211	38	rural/clinic	No	13/04/2017	0	0	0
2012263747	38	rural/clinic	No	13/04/2017	0	0	0
2012263747	38	rural/clinic	No	13/04/2017	0	0	0
2012350626	38	rural/clinic	No	13/04/2017	0	0	0
2012350626	38	rural/clinic	No	13/04/2017	0	0	0
2015406477	38	rural/clinic	No	21/04/2017	0	0	0
2015334750	38	rural/clinic	No	21/04/2017	0	0	0
2015334738	38	rural/clinic	No	21/04/2017	0	0	0
2014358699	38	rural/clinic	No	21/04/2017	0	0	0
2014358695	38	rural/clinic	No	21/04/2017	0	0	0
2014358696	38	rural/clinic	No	21/04/2017	0	0	0
2015334739	38	rural/clinic	No	21/04/2017	0	0	0
2015334740	38	rural/clinic	No	21/04/2017	0	0	0
2015334741	38	rural/clinic	No	21/04/2017	0	0	0
2015334742	38	rural/clinic	No	21/04/2017	0	0	0
2015334743	38	rural/clinic	No	21/04/2017	0	0	0
2015334744	38	rural/clinic	No	21/04/2017	0	0	0
2015334745	38	rural/clinic	No	21/04/2017	0	0	0
2015334746	38	rural/clinic	No	21/04/2017	0	0	0
2015334747	38	rural/clinic	No	21/04/2017	0	0	0
2015334748	38	rural/clinic	No	21/04/2017	0	0	0
2015334749	38	rural/clinic	No	21/04/2017	0	0	0
2014349220	38	rural/clinic	No	21/04/2017	0	0	0
2014349221	38	rural/clinic	No	21/04/2017	0	0	0
2014349222	38	rural/clinic	No	21/04/2017	0	0	0
2014349223	38	rural/clinic	No	21/04/2017	0	0	0
2014349216	38	rural/clinic	No	21/04/2017	0	0	0
2013248190	38	rural/clinic	No	21/04/2017	0	0	0
2015374264	38	rural/clinic	No	21/04/2017	0	0	0
2015374262	38	rural/clinic	No	21/04/2017	0	0	0
2015374266	38	rural/clinic	No	21/04/2017	0	0	0
2012298698	38	rural/clinic	No	21/04/2017	0	0	0
2012298697	38	rural/clinic	No	21/04/2017	0	0	0
2014287425	38	rural/clinic	No	21/04/2017	0	0	0
2015322038	38	rural/clinic	No	21/04/2017	0	0	0
2015322740	38	rural/clinic	No	21/04/2017	0	0	0
2015322737	38	rural/clinic	No	21/04/2017	0	0	0
2015322738	38	rural/clinic	No	21/04/2017	0	0	0
2015322739	38	rural/clinic	No	21/04/2017	0	0	0
2012285878	38	rural/clinic	No	21/04/2017	0	0	0
2012285879	38	rural/clinic	No	21/04/2017	0	0	0
2012285645	38	rural/clinic	No	21/04/2017	0	0	0
2015339013	38	rural/clinic	No	13/04/2017	0	0	0
2015339013	38	rural/clinic	No	13/04/2017	0	0	0
2015372021	38	rural/clinic	No	13/04/2017	0	0	0
2015372021	38	rural/clinic	No	13/04/2017	0	0	0
2015386399	38	rural/clinic	No	13/04/2017	0	0	0
2015386399	38	rural/clinic	No	13/04/2017	0	0	0
2015335101	38	rural/clinic	No	13/04/2017	0	0	0
2015335101	38	rural/clinic	No	13/04/2017	0	0	0

2015406468	38	rural/clinic	No	13/04/2017	0	0	0
2015368279	38	rural/clinic	No	13/04/2017	0	0	0
2015368279	38	rural/clinic	No	13/04/2017	0	0	0
2014291875	38	rural/clinic	No	14/04/2017	0	0	0
2014291875	38	rural/clinic	No	14/04/2017	0	0	0
2015369700	38	rural/clinic	No	19/04/2017	0	0	0
2015339824	38	rural/clinic	No	19/04/2017	0	0	0
2015339824	38	rural/clinic	No	19/04/2017	0	0	0
2014291876	38	rural/clinic	No	21/04/2017	0	0	0
2014291876	38	rural/clinic	No	21/04/2017	0	0	0
2014314215	38	rural/clinic	No	11/05/2017	0	0	0
2011225405	38	rural/clinic	No	25/04/2017	0	0	0
2011225406	38	rural/clinic	No	25/04/2017	0	0	0
2015400038	38	rural/clinic	No	26/04/2017	0	0	0
0	38	rural/clinic	No	25/04/2017	0	0	0
201432039	38	rural/clinic	No	25/04/2017	0	0	0
2015400037	38	rural/clinic	No	25/04/2017	0	0	0
2015400036	38	rural/clinic	No	25/04/2017	0	0	0
2015400035	38	rural/clinic	No	25/04/2017	0	0	0
2012369025	38	rural/clinic	No	25/04/2017	0	0	0
2014346720	38	rural/clinic	No	25/04/2017	0	0	0
2015400034	38	rural/clinic	No	25/04/2017	0	0	0
2014327041	38	rural/clinic	No	25/04/2017	0	0	0
2012369024	38	rural/clinic	No	25/04/2017	0	0	0
2015301501	38	rural/clinic	No	25/04/2017	0	0	0
2015400033	38	rural/clinic	No	25/04/2017	0	0	0
2014346719	38	rural/clinic	No	25/04/2017	0	0	0
2015400032	38	rural/clinic	No	25/04/2017	0	0	0
2014303296	38	rural/clinic	No	25/04/2017	0	0	0
2014290724	38	rural/clinic	No	25/04/2017	0	0	0
2015390261	38	rural/clinic	No	25/04/2017	0	0	0
2015301463	38	rural/clinic	No	25/04/2017	0	0	0
2014375686	38	rural/clinic	No	25/04/2017	0	0	0
2013261294	38	rural/clinic	No	25/04/2017	0	0	0
2014290725	38	rural/clinic	No	25/04/2017	0	0	0
2012304332	38	rural/clinic	No	25/04/2017	0	0	0
2011224436	38	rural/clinic	No	25/04/2017	0	0	0
2014365366	38	rural/clinic	No	25/04/2017	0	0	0
2012304331	38	rural/clinic	No	25/04/2017	0	0	0
2014290723	38	rural/clinic	No	25/04/2017	0	0	0
2011224437	38	rural/clinic	No	25/04/2017	0	0	0
2015402602	38	rural/clinic	No	25/04/2017	0	0	0
2015363009	38	rural/clinic	No	25/04/2017	0	0	0
2015402603	38	rural/clinic	No	25/04/2017	0	0	0
2014338311	38	rural/clinic	No	25/04/2017	0	0	0
2015324437	38	rural/clinic	No	25/04/2017	0	0	0
2015402604	38	rural/clinic	No	25/04/2017	0	0	0
2015301256	38	rural/clinic	No	25/04/2017	0	0	0
2015402605	38	rural/clinic	No	25/04/2017	0	0	0
2015363011	38	rural/clinic	No	25/04/2017	0	0	0
2015337618	38	rural/clinic	No	25/04/2017	0	0	0
2015402606	38	rural/clinic	No	25/04/2017	0	0	0
2015301255	38	rural/clinic	No	25/04/2017	0	0	0
2011152131	38	rural/clinic	No	25/04/2017	0	0	0
2015402607	38	rural/clinic	No	25/04/2017	0	0	0
2015363012	38	rural/clinic	No	25/04/2017	0	0	0
2015301257	38	rural/clinic	No	25/04/2017	0	0	0
2015402608	38	rural/clinic	No	25/04/2017	0	0	0
2015402609	38	rural/clinic	No	25/04/2017	0	0	0
2014363226	38	rural/clinic	No	25/04/2017	0	0	0
2015302857	38	rural/clinic	No	25/04/2017	0	0	0
2015402610	38	rural/clinic	No	25/04/2017	0	0	0
2015315999	38	rural/clinic	No	25/04/2017	0	0	0
2015402611	38	rural/clinic	No	25/04/2017	0	0	0
2015349452	38	rural/clinic	No	25/04/2017	0	0	0
2014371161	38	rural/clinic	No	25/04/2017	0	0	0
2015413132	38	rural/clinic	No	25/04/2017	0	0	0
2015324472	38	rural/clinic	No	25/04/2017	0	0	0
2014350046	38	rural/clinic	No	25/04/2017	0	0	0
2015362814	38	rural/clinic	No	11/05/2017	0	0	0
2015413133	38	rural/clinic	No	25/04/2017	0	0	0
2011229217	38	rural/clinic	No	25/04/2017	0	0	0
2014291618	38	rural/clinic	No	25/04/2017	0	0	0
2013261295	38	rural/clinic	No	25/04/2017	0	0	0
2013266117	38	rural/clinic	No	25/04/2017	0	0	0
2011199083	38	rural/clinic	No	25/04/2017	0	0	0
2015337502	38	rural/clinic	No	25/04/2017	0	0	0
2013261296	38	rural/clinic	No	25/04/2017	0	0	0
2015376124	38	rural/clinic	No	26/04/2017	0	0	0
2015413134	38	rural/clinic	No	25/04/2017	0	0	0
2015405410	38	rural/clinic	No	25/04/2017	0	0	0
2015376125	38	rural/clinic	No	25/04/2017	0	0	0

2014314212	38	rural/clinic	No	25/04/2017	0	0	0
2015337503	38	rural/clinic	No	25/04/2017	0	0	0
2014302845	38	rural/clinic	No	25/04/2017	0	0	0
2014307523	38	rural/clinic	No	25/04/2017	0	0	0
2014314213	38	rural/clinic	No	25/04/2017	0	0	0
2015363057	38	rural/clinic	No	25/04/2017	0	0	0
2015324436	38	rural/clinic	No	25/04/2017	0	0	0
2015340007	38	rural/clinic	No	25/04/2017	0	0	0
2015358181	38	rural/clinic	No	25/04/2017	0	0	0
2014306003	38	rural/clinic	No	25/04/2017	0	0	0
2014365382	38	rural/clinic	No	25/04/2017	0	0	0
2015358182	38	rural/clinic	No	25/04/2017	0	0	0
2015363010	38	rural/clinic	No	25/04/2017	0	0	0
2015358183	38	rural/clinic	No	25/04/2017	0	0	0
2014291621	38	rural/clinic	No	25/04/2017	0	0	0
2014307522	38	rural/clinic	No	25/04/2017	0	0	0
2011199081	38	rural/clinic	No	25/04/2017	0	0	0
2014363193	38	rural/clinic	No	25/04/2017	0	0	0
2014307521	38	rural/clinic	No	25/04/2017	0	0	0
2015358184	38	rural/clinic	No	25/04/2017	0	0	0
2014302938	38	rural/clinic	No	25/04/2017	0	0	0
2012369021	38	rural/clinic	No	25/04/2017	0	0	0
2015368955	38	rural/clinic	No	25/04/2017	0	0	0
2014307520	38	rural/clinic	No	25/04/2017	0	0	0
2015338652	38	rural/clinic	No	25/04/2017	0	0	0
2015368956	38	rural/clinic	No	25/04/2017	0	0	0
2012363961	38	rural/clinic	No	25/04/2017	0	0	0
2014365381	38	rural/clinic	No	25/04/2017	0	0	0
2014358697	38	rural/clinic	No	25/04/2017	0	0	0
2015368957	38	rural/clinic	No	25/04/2017	0	0	0
2015349457	38	rural/clinic	No	25/04/2017	0	0	0
2015338651	38	rural/clinic	No	25/04/2017	0	0	0
2012363960	38	rural/clinic	No	25/04/2017	0	0	0
2012369022	38	rural/clinic	No	25/04/2017	0	0	0
2015368958	38	rural/clinic	No	25/04/2017	0	0	0
2015338653	38	rural/clinic	No	25/04/2017	0	0	0
2015368959	38	rural/clinic	No	25/04/2017	0	0	0
2012369023	38	rural/clinic	No	25/04/2017	0	0	0
2012363959	38	rural/clinic	No	25/04/2017	0	0	0
2015412089	38	rural/clinic	No	25/04/2017	0	0	0
2012363958	38	rural/clinic	No	25/04/2017	0	0	0
2013261930	38	rural/clinic	No	25/04/2017	0	0	0
2011222800	38	rural/clinic	No	25/04/2017	0	0	0
2014358698	38	rural/clinic	No	25/04/2017	0	0	0
2013261932	38	rural/clinic	No	25/04/2017	0	0	0
2011225412	38	rural/clinic	No	25/04/2017	0	0	0
2013283503	38	rural/clinic	No	25/04/2017	0	0	0
2015338655	38	rural/clinic	No	25/04/2017	0	0	0
2014358694	38	rural/clinic	No	25/04/2017	0	0	0
2013261931	38	rural/clinic	No	25/04/2017	0	0	0
2014333800	38	rural/clinic	No	25/04/2017	0	0	0
2015338654	38	rural/clinic	No	25/04/2017	0	0	0
2011225411	38	rural/clinic	No	25/04/2017	0	0	0
2015360721	38	rural/clinic	No	25/04/2017	0	0	0
2015412086	38	rural/clinic	No	25/04/2017	0	0	0
2015367202	38	rural/clinic	No	25/04/2017	0	0	0
2015360725	38	rural/clinic	No	25/04/2017	0	0	0
2014298586	38	rural/clinic	No	25/04/2017	0	0	0
2011222799	38	rural/clinic	No	25/04/2017	0	0	0
2015367203	38	rural/clinic	No	25/04/2017	0	0	0
2015360724	38	rural/clinic	No	25/04/2017	0	0	0
2014302847	38	rural/clinic	No	25/04/2017	0	0	0
2014298585	38	rural/clinic	No	25/04/2017	0	0	0
2015362808	38	rural/clinic	No	25/04/2017	0	0	0
2014324703	38	rural/clinic	No	25/04/2017	0	0	0
2011229218	38	rural/clinic	No	25/04/2017	0	0	0
2013264389	38	rural/clinic	No	25/04/2017	0	0	0
2012359931	38	rural/clinic	No	25/04/2017	0	0	0
2014324704	38	rural/clinic	No	25/04/2017	0	0	0
2012359930	38	rural/clinic	No	25/04/2017	0	0	0
2014290273	38	rural/clinic	No	25/04/2017	0	0	0
2011229219	38	rural/clinic	No	25/04/2017	0	0	0
2015349001	38	rural/clinic	No	25/04/2017	0	0	0
2014299917	38	rural/clinic	No	25/04/2017	0	0	0
2012359929	38	rural/clinic	No	25/04/2017	0	0	0
201432087	38	rural/clinic	No	25/04/2017	0	0	0
2012266993	38	rural/clinic	No	25/04/2017	0	0	0
2015413349	38	rural/clinic	No	25/04/2017	0	0	0
2014299918	38	rural/clinic	No	25/04/2017	0	0	0
2015413348	38	rural/clinic	No	25/04/2017	0	0	0
2012266995	38	rural/clinic	No	25/04/2017	0	0	0
2015301361	38	rural/clinic	No	25/04/2017	0	0	0

2015385391	38	rural/clinic	No	25/04/2017	0	0	0
2015413347	38	rural/clinic	No	25/04/2017	0	0	0
2011216824	38	rural/clinic	No	25/04/2017	0	0	0
201066582	38	rural/clinic	No	25/04/2017	0	0	0
2012290398	38	rural/clinic	No	25/04/2017	0	0	0
2015355513	38	rural/clinic	No	25/04/2017	0	0	0
2015362474	38	rural/clinic	No	25/04/2017	0	0	0
2015303071	38	rural/clinic	No	25/04/2017	0	0	0
2015414950	38	rural/clinic	No	25/04/2017	0	0	0
2012266994	922	rural/clinic	No	25/04/2017	0	0	0
2015303072	922	rural/clinic	No	25/04/2017	0	0	0
2015368813	922	rural/clinic	No	25/04/2017	0	0	0
2015362475	922	rural/clinic	No	25/04/2017	0	0	0
2015385390	922	rural/clinic	No	25/04/2017	0	0	0
2014374008	922	rural/clinic	No	25/04/2017	0	0	0
2015303073	922	rural/clinic	No	25/04/2017	0	0	0
2015368811	116	district/faith-based	No	25/04/2017	1	0	0
2011191290	116	district/faith-based	No	25/04/2017	1	0	0
2015413350	116	district/faith-based	No	25/04/2017	1	0	0
2015315902	116	district/faith-based	No	25/04/2017	1	0	0
2014374009	116	district/faith-based	No	25/04/2017	1	0	0
2015368812	116	district/faith-based	No	25/04/2017	1	0	0
2015367201	116	district/faith-based	No	25/04/2017	1	0	0
2012359928	116	district/faith-based	No	25/04/2017	1	0	0
2011223205	116	district/faith-based	No	25/04/2017	1	0	0
2015414333	116	district/faith-based	No	25/04/2017	1	0	0
2015367204	116	district/faith-based	No	25/04/2017	1	0	0
2012359927	116	district/faith-based	No	25/04/2017	1	0	0
2012384792	116	district/faith-based	No	25/04/2017	1	0	0
2015338714	116	district/faith-based	No	25/04/2017	1	0	0
2015384481	116	district/faith-based	No	25/04/2017	1	0	0
2015363758	116	district/faith-based	No	25/04/2017	1	0	0
2015414302	116	district/faith-based	No	25/04/2017	1	0	0
2015384039	116	district/faith-based	No	25/04/2017	1	0	0
2015338713	116	district/faith-based	No	25/04/2017	1	0	0
2012314015	116	district/faith-based	No	25/04/2017	1	0	0
2014305219	116	district/faith-based	No	25/04/2017	1	0	0
2015384037	116	district/faith-based	No	25/04/2017	1	0	0
2012306343	116	district/faith-based	No	25/04/2017	1	0	0
2014305220	116	district/faith-based	No	25/04/2017	1	0	0
2015414334	116	district/faith-based	No	25/04/2017	1	0	0
2014346948	116	district/faith-based	No	25/04/2017	1	0	0
2014382439	116	district/faith-based	No	25/04/2017	1	0	0
2012314016	116	district/faith-based	No	25/04/2017	1	0	0
2015414335	116	district/faith-based	No	25/04/2017	1	0	0
2015384158	116	district/faith-based	No	25/04/2017	1	0	0
2014382437	116	district/faith-based	No	25/04/2017	1	0	0
2014375684	116	district/faith-based	No	25/04/2017	1	0	0
2015337367	116	district/faith-based	No	25/04/2017	1	0	0
2012314017	116	district/faith-based	No	25/04/2017	1	0	0
2015349002	116	district/faith-based	No	25/04/2017	1	0	0
2015384163	116	district/faith-based	No	25/04/2017	1	0	0
2014382436	116	district/faith-based	No	25/04/2017	1	0	0
2015331895	116	district/faith-based	No	25/04/2017	1	0	0
2012365471	116	district/faith-based	No	25/04/2017	1	0	0
2015384036	116	district/faith-based	No	25/04/2017	1	0	0
2014382438	116	district/faith-based	No	25/04/2017	1	0	0
2015384164	116	district/faith-based	No	25/04/2017	1	0	0
2013283504	116	district/faith-based	No	25/04/2017	1	0	0
2015324435	116	district/faith-based	No	25/04/2017	1	0	0
2012365472	116	district/faith-based	No	25/04/2017	1	0	0
2011193473	116	district/faith-based	No	25/04/2017	1	0	0
2015290026	116	district/faith-based	No	25/04/2017	1	0	0
2015384162	116	district/faith-based	No	25/04/2017	1	0	0
2015290025	116	district/faith-based	No	25/04/2017	1	0	0
2015333956	116	district/faith-based	No	25/04/2017	1	0	0
2014291816	116	district/faith-based	No	25/04/2017	1	0	0
2015384038	116	district/faith-based	No	25/04/2017	1	0	0
2012266996	116	district/faith-based	No	25/04/2017	1	0	0
2015375411	116	district/faith-based	No	25/04/2017	1	0	0
2011192934	116	district/faith-based	No	25/04/2017	1	0	0
2015377827	116	district/faith-based	No	25/04/2017	1	0	0
2015289845	116	district/faith-based	No	25/04/2017	1	0	0
2015333255	116	district/faith-based	No	25/04/2017	1	0	0
2012266997	116	district/faith-based	No	25/04/2017	1	0	0
2012384788	116	district/faith-based	No	25/04/2017	1	0	0
2015377828	116	district/faith-based	No	25/04/2017	1	0	0
2015375410	116	district/faith-based	No	25/04/2017	1	0	0
2012314018	116	district/faith-based	No	25/04/2017	1	0	0
2015412504	116	district/faith-based	No	25/04/2017	1	0	0
2015407056	116	district/faith-based	No	25/04/2017	1	0	0
2012384789	116	district/faith-based	No	25/04/2017	1	0	0

2014297966	116	district/faith-based	No	25/04/2017	1	0	0
2015375986	116	district/faith-based	No	25/04/2017	1	0	0
2012384790	116	district/faith-based	No	25/04/2017	1	0	0
2014330349	116	district/faith-based	No	25/04/2017	1	0	0
2015364386	116	district/faith-based	No	25/04/2017	1	0	0
2015289844	116	district/faith-based	No	25/04/2017	1	0	0
2012384791	116	district/faith-based	No	25/04/2017	1	0	0
2015286541	116	district/faith-based	No	25/04/2017	1	0	0
2014356978	116	district/faith-based	No	25/04/2017	1	0	0
2014305218	116	district/faith-based	No	25/04/2017	1	0	0
2015364385	116	district/faith-based	No	25/04/2017	1	0	0
2014356981	116	district/faith-based	No	25/04/2017	1	0	0
2011236080	116	district/faith-based	No	25/04/2017	1	0	0
2015369923	116	district/faith-based	No	25/04/2017	1	0	0
2014346721	116	district/faith-based	No	25/04/2017	1	0	0
2014356980	116	district/faith-based	No	25/04/2017	1	0	0
2015333957	116	district/faith-based	No	25/04/2017	1	0	0
2015364384	116	district/faith-based	No	25/04/2017	1	0	0
2014346715	116	district/faith-based	No	25/04/2017	1	0	0
2015369732	116	district/faith-based	No	25/04/2017	1	0	0
2014356979	116	district/faith-based	No	25/04/2017	1	0	0
2015407057	116	district/faith-based	No	25/04/2017	1	0	0
2015340204	116	district/faith-based	No	25/04/2017	1	0	0
2014319049	116	district/faith-based	No	25/04/2017	1	0	0
2014346716	116	district/faith-based	No	25/04/2017	1	0	0
2014356977	116	district/faith-based	No	25/04/2017	1	0	0
2015269925	116	district/faith-based	No	25/04/2017	1	0	0
2014346717	116	district/faith-based	No	25/04/2017	1	0	0
2015364379	116	district/faith-based	No	25/04/2017	1	0	0
2015340206	116	district/faith-based	No	25/04/2017	1	0	0
2014315701	116	district/faith-based	No	25/04/2017	1	0	0
2014342788	116	district/faith-based	No	25/04/2017	1	0	0
2014375685	116	district/faith-based	No	25/04/2017	1	0	0
2014346718	116	district/faith-based	No	25/04/2017	1	0	0
2015375985	116	district/faith-based	No	25/04/2017	1	0	0
2015364383	116	district/faith-based	No	25/04/2017	1	0	0
2015364378	116	district/faith-based	No	25/04/2017	1	0	0
2015340752	116	district/faith-based	No	25/04/2017	1	0	0
2015369924	116	district/faith-based	No	25/04/2017	1	0	0
2014319050	116	district/faith-based	No	25/04/2017	1	0	0
2017REJ	116	district/faith-based	No	06/04/2017	1	0	0
2014356976	116	district/faith-based	No	25/04/2017	1	0	0
2014297965	116	district/faith-based	No	25/04/2017	1	0	0
2015364377	116	district/faith-based	No	25/04/2017	1	0	0
2015364382	116	district/faith-based	No	25/04/2017	1	0	0
2015357644	116	district/faith-based	No	25/04/2017	1	0	0
2015346232	116	district/faith-based	No	25/04/2017	1	0	0
2015310606	116	district/faith-based	No	25/04/2017	1	0	0
2014344745	116	district/faith-based	No	06/04/2017	1	0	0
2014326633	116	district/faith-based	No	25/04/2017	1	0	0
2015340846	116	district/faith-based	No	25/04/2017	1	0	0
2015346231	116	district/faith-based	No	25/04/2017	1	0	0
2015339752	116	district/faith-based	No	25/04/2017	1	0	0
2015364376	116	district/faith-based	No	25/04/2017	1	0	0
2015364381	116	district/faith-based	No	25/04/2017	1	0	0
2014344746	116	district/faith-based	No	06/04/2017	1	0	0
2015339753	116	district/faith-based	No	25/04/2017	1	0	0
2014358689	116	district/faith-based	No	25/04/2017	1	0	0
2015340847	116	district/faith-based	No	25/04/2017	1	0	0
2015364375	116	district/faith-based	No	25/04/2017	1	0	0
2014344748	116	district/faith-based	No	06/04/2017	1	0	0
2013255341	116	district/faith-based	No	25/04/2017	1	0	0
201564380	116	district/faith-based	No	25/04/2017	1	0	0
2014307516	116	district/faith-based	No	25/04/2017	1	0	0
2015340848	116	district/faith-based	No	25/04/2017	1	0	0
2014326634	116	district/faith-based	No	25/04/2017	1	0	0
2014344747	116	district/faith-based	No	06/04/2017	1	0	0
2015319444	116	district/faith-based	No	25/04/2017	1	0	0
2015332454	116	district/faith-based	No	25/04/2017	1	0	0
2015340849	116	district/faith-based	No	25/04/2017	1	0	0
2015332456	116	district/faith-based	No	25/04/2017	1	0	0
2014360960	116	district/faith-based	No	25/04/2017	1	0	0
20170REJ	116	district/faith-based	No	06/04/2017	1	0	0
2014326631	116	district/faith-based	No	25/04/2017	1	0	0
2014307515	116	district/faith-based	No	25/04/2017	1	0	0
2015319445	116	district/faith-based	No	25/04/2017	1	0	0
2015340850	116	district/faith-based	No	25/04/2017	1	0	0
2014335239	116	district/faith-based	No	25/04/2017	1	0	0
201701REJ	116	district/faith-based	No	06/04/2017	1	0	0
2011200183	116	district/faith-based	No	25/04/2017	1	0	0
2012335665	116	district/faith-based	No	25/04/2017	1	0	0
2014326632	116	district/faith-based	No	25/04/2017	1	0	0

2014358001	116	district/faith-based	No	25/04/2017	1	0	0
201702REJ	116	district/faith-based	No	06/04/2017	1	0	0
2015402379	116	district/faith-based	No	25/04/2017	1	0	0
2014335238	116	district/faith-based	No	25/04/2017	1	0	0
2015357645	116	district/faith-based	No	25/04/2017	1	0	0
2014369194	116	district/faith-based	No	25/04/2017	1	0	0
2015338461	116	district/faith-based	No	25/04/2017	1	0	0
2015368786	116	district/faith-based	No	25/04/2017	1	0	0
2011212593	116	district/faith-based	No	06/04/2017	1	0	0
2015402383	116	district/faith-based	No	25/04/2017	1	0	0
2015357646	116	district/faith-based	No	25/04/2017	1	0	0
2012325689	116	district/faith-based	No	25/04/2017	1	0	0
2014346282	116	district/faith-based	No	25/04/2017	1	0	0
2015338460	116	district/faith-based	No	25/04/2017	1	0	0
2015287360	116	district/faith-based	No	11/04/2017	1	0	0
2015332455	116	district/faith-based	No	25/04/2017	1	0	0
2014327674	116	district/faith-based	No	25/04/2017	1	0	0
2012325690	116	district/faith-based	No	25/04/2017	1	0	0
2014313659	116	district/faith-based	No	25/04/2017	1	0	0
2014346280	116	district/faith-based	No	25/04/2017	1	0	0
201703REJ	116	district/faith-based	No	04/04/2017	1	0	0
2012361948	116	district/faith-based	No	25/04/2017	1	0	0
2015368577	116	district/faith-based	No	25/04/2017	1	0	0
2015357398	116	district/faith-based	No	04/04/2017	1	0	0
2015303619	116	district/faith-based	No	25/04/2017	1	0	0
2015402378	116	district/faith-based	No	25/04/2017	1	0	0
2014346281	116	district/faith-based	No	25/04/2017	1	0	0
2014362815	116	district/faith-based	No	25/04/2017	1	0	0
2015368578	116	district/faith-based	No	25/04/2017	1	0	0
2014303339	116	district/faith-based	No	06/04/2017	1	0	0
2014366067	116	district/faith-based	No	25/04/2017	1	0	0
2015203622	116	district/faith-based	No	25/04/2017	1	0	0
2014362814	116	district/faith-based	No	25/04/2017	1	0	0
2015368579	116	district/faith-based	No	25/04/2017	1	0	0
2014357312	116	district/faith-based	No	25/04/2017	1	0	0
2012267316	116	district/faith-based	No	25/04/2017	1	0	0
2012365150	116	district/faith-based	No	25/04/2017	1	0	0
2014362812	116	district/faith-based	No	25/04/2017	1	0	0
2015303623	116	district/faith-based	No	25/04/2017	1	0	0
2015368580	116	district/faith-based	No	25/04/2017	1	0	0
2015402381	116	district/faith-based	No	25/04/2017	1	0	0
2014362811	116	district/faith-based	No	25/04/2017	1	0	0
2012267317	116	district/faith-based	No	25/04/2017	1	0	0
2014357313	80	rural/clinic	No	25/04/2017	0	0	0
2014334173	80	rural/clinic	No	25/04/2017	0	0	0
2014307412	80	rural/clinic	No	25/04/2017	0	0	0
2015402380	80	rural/clinic	No	25/04/2017	0	0	0
2014357311	80	rural/clinic	No	25/04/2017	0	0	0
2014362813	80	rural/clinic	No	25/04/2017	0	0	0
2014338503	80	rural/clinic	No	25/04/2017	0	0	0
2013266072	80	rural/clinic	No	25/04/2017	0	0	0
2012291490	80	rural/clinic	No	25/04/2017	0	0	0
2015402382	80	rural/clinic	No	25/04/2017	0	0	0
2014307411	80	rural/clinic	No	25/04/2017	0	0	0
2015303624	80	rural/clinic	No	25/04/2017	0	0	0
2015294058	80	rural/clinic	No	25/04/2017	0	0	0
2014334174	80	rural/clinic	No	25/04/2017	0	0	0
2014307518	80	rural/clinic	No	25/04/2017	0	0	0
2015372803	80	rural/clinic	No	25/04/2017	0	0	0
2015294057	80	rural/clinic	No	25/04/2017	0	0	0
2014302846	80	rural/clinic	No	25/04/2017	0	0	0
2015409003	80	rural/clinic	No	25/04/2017	0	0	0
2014307517	80	rural/clinic	No	25/04/2017	0	0	0
2015372801	80	rural/clinic	No	25/04/2017	0	0	0
2015294056	80	rural/clinic	No	25/04/2017	0	0	0
2014358690	80	rural/clinic	No	25/04/2017	0	0	0
2015372802	80	rural/clinic	No	25/04/2017	0	0	0
2015409008	80	rural/clinic	No	25/04/2017	0	0	0
2015294055	80	rural/clinic	No	25/04/2017	0	0	0
2015301262	80	rural/clinic	No	25/04/2017	0	0	0
2014307507	80	rural/clinic	No	25/04/2017	0	0	0
2015294054	80	rural/clinic	No	25/04/2017	0	0	0
2014309383	80	rural/clinic	No	25/04/2017	0	0	0
2015414301	80	rural/clinic	No	26/04/2017	0	0	0
2014350045	80	rural/clinic	No	25/04/2017	0	0	0
2011294800	80	rural/clinic	No	25/04/2017	0	0	0
2015409002	80	rural/clinic	No	25/04/2017	0	0	0
2015294053	80	rural/clinic	No	25/04/2017	0	0	0
2014342518	80	rural/clinic	No	25/04/2017	0	0	0
2011134982	354	rural/clinic	No	25/04/2017	0	0	0
2015368787	354	rural/clinic	No	25/04/2017	0	0	0
2014342517	354	rural/clinic	No	25/04/2017	0	0	0

2011225370	354	rural/clinic	No	25/04/2017	0	0	0
2015303625	354	rural/clinic	No	25/04/2017	0	0	0
2011134984	354	rural/clinic	No	25/04/2017	0	0	0
2014342519	354	rural/clinic	No	25/04/2017	0	0	0
2015409007	354	rural/clinic	No	25/04/2017	0	0	0
2015418144	354	rural/clinic	No	25/04/2017	0	0	0
2011200276	354	rural/clinic	No	25/04/2017	0	0	0
2011134983	354	rural/clinic	No	25/04/2017	0	0	0
2015418143	354	rural/clinic	No	25/04/2017	0	0	0
2014358002	354	rural/clinic	No	25/04/2017	0	0	0
2011134985	354	rural/clinic	No	25/04/2017	0	0	0
2015302412	354	rural/clinic	No	25/04/2017	0	0	0
2012369495	354	rural/clinic	No	25/04/2017	0	0	0
2011134986	354	rural/clinic	No	25/04/2017	0	0	0
2011225647	354	rural/clinic	No	25/04/2017	0	0	0
2015413358	354	rural/clinic	No	26/04/2017	0	0	0
2012369026	354	rural/clinic	No	25/04/2017	0	0	0
2014358003	354	rural/clinic	No	25/04/2017	0	0	0
2015302413	354	rural/clinic	No	25/04/2017	0	0	0
2015357642	354	rural/clinic	No	25/04/2017	0	0	0
2011134981	354	rural/clinic	No	25/04/2017	0	0	0
2011191291	354	rural/clinic	No	26/04/2017	0	0	0
2015357641	354	rural/clinic	No	25/04/2017	0	0	0
2014350097	354	rural/clinic	No	25/04/2017	0	0	0
2011199080	354	rural/clinic	No	25/04/2017	0	0	0
2015409004	354	rural/clinic	No	25/04/2017	0	0	0
2012276251	354	rural/clinic	No	25/04/2017	0	0	0
2012294468	354	rural/clinic	No	26/04/2017	0	0	0
2012368541	354	rural/clinic	No	25/04/2017	0	0	0
2011236179	354	rural/clinic	No	25/04/2017	0	0	0
2012289894	354	rural/clinic	No	25/04/2017	0	0	0
2011225369	354	rural/clinic	No	25/04/2017	0	0	0
2012317314	354	rural/clinic	No	26/04/2017	0	0	0
2015409001	354	rural/clinic	No	25/04/2017	0	0	0
2012306140	355	rural/clinic	No	25/04/2017	0	0	0
2011236180	355	rural/clinic	No	25/04/2017	0	0	0
2014363877	355	rural/clinic	No	25/04/2017	0	0	0
2012317317	355	rural/clinic	No	26/04/2017	0	0	0
2014302982	355	rural/clinic	No	25/04/2017	0	0	0
2015302411	355	rural/clinic	No	25/04/2017	0	0	0
2015340202	355	rural/clinic	No	25/04/2017	0	0	0
2014371500	355	rural/clinic	No	25/04/2017	0	0	0
2015301502	355	rural/clinic	No	25/04/2017	0	0	0
2014363876	355	rural/clinic	No	25/04/2017	0	0	0
2014350096	355	rural/clinic	No	25/04/2017	0	0	0
2015303621	923	rural/clinic	No	26/04/2017	0	0	0
2014302981	923	rural/clinic	No	25/04/2017	0	0	0
2015340203	923	rural/clinic	No	25/04/2017	0	0	0
2015418200	923	rural/clinic	No	25/04/2017	0	0	0
2012368540	923	rural/clinic	No	25/04/2017	0	0	0
2014363875	923	rural/clinic	No	25/04/2017	0	0	0
2015368785	923	rural/clinic	No	26/04/2017	0	0	0
2014363751	659	rural/clinic	No	25/04/2017	0	0	0
2014306004	659	rural/clinic	No	25/04/2017	0	0	0
2011235745	659	rural/clinic	No	25/04/2017	0	0	0
2014332079	659	rural/clinic	No	25/04/2017	0	0	0
2014319129	659	rural/clinic	No	25/04/2017	0	0	0
2014374010	659	rural/clinic	No	26/04/2017	0	0	0
2015405914	659	rural/clinic	No	25/04/2017	0	0	0
2014302844	659	rural/clinic	No	25/04/2017	0	0	0
2014333799	659	rural/clinic	No	26/04/2017	0	0	0
2014306006	659	rural/clinic	No	25/04/2017	0	0	0
2015303620	659	rural/clinic	No	26/04/2017	0	0	0
2015405915	453	rural/clinic	No	25/04/2017	0	0	0
2015340083	453	rural/clinic	No	25/04/2017	0	0	0
2014306005	453	rural/clinic	No	25/04/2017	0	0	0
2014332080	453	rural/clinic	No	25/04/2017	0	0	0
2015357643	453	rural/clinic	No	25/04/2017	0	0	0
2012368538	453	rural/clinic	No	25/04/2017	0	0	0
2014306008	453	rural/clinic	No	25/04/2017	0	0	0
2011225646	453	rural/clinic	No	25/04/2017	0	0	0
2012306449	453	rural/clinic	No	25/04/2017	0	0	0
2015325932	453	rural/clinic	No	25/04/2017	0	0	0
2015325995	453	rural/clinic	No	25/04/2017	0	0	0
2014306007	453	rural/clinic	No	25/04/2017	0	0	0
2015302414	453	rural/clinic	No	25/04/2017	0	0	0
2015357801	453	rural/clinic	No	25/04/2017	0	0	0
2015357527	453	rural/clinic	No	25/04/2017	0	0	0
2015325994	453	rural/clinic	No	25/04/2017	0	0	0
2012306450	453	rural/clinic	No	25/04/2017	0	0	0
2014350095	453	rural/clinic	No	25/04/2017	0	0	0
2015400039	453	rural/clinic	No	25/04/2017	0	0	0

2015325993	453	rural/clinic	No	25/04/2017	0	0	0
2012306139	453	rural/clinic	No	25/04/2017	0	0	0
2015397603	453	rural/clinic	No	25/04/2017	0	0	0
2015302408	453	rural/clinic	No	25/04/2017	0	0	0
2015397602	453	rural/clinic	No	25/04/2017	0	0	0
2011179931	453	rural/clinic	No	25/04/2017	0	0	0
2015357528	453	rural/clinic	No	25/04/2017	0	0	0
2015400040	453	rural/clinic	No	25/04/2017	0	0	0
2015397601	453	rural/clinic	No	25/04/2017	0	0	0
2011225368	453	rural/clinic	No	25/04/2017	0	0	0
2015302409	453	rural/clinic	No	25/04/2017	0	0	0
2015357192	453	rural/clinic	No	25/04/2017	0	0	0
2015400041	453	rural/clinic	No	25/04/2017	0	0	0
2014327088	453	rural/clinic	No	25/04/2017	0	0	0
2015397550	453	rural/clinic	No	25/04/2017	0	0	0
2015357191	453	rural/clinic	No	25/04/2017	0	0	0
2015397549	453	rural/clinic	No	25/04/2017	0	0	0
2015357190	453	rural/clinic	No	25/04/2017	0	0	0
2015397548	453	rural/clinic	No	25/04/2017	0	0	0
2013271841	453	rural/clinic	No	25/04/2017	0	0	0
2015397547	453	rural/clinic	No	25/04/2017	0	0	0
2015302405	453	rural/clinic	No	25/04/2017	0	0	0
2015397546	453	rural/clinic	No	25/04/2017	0	0	0
2015326536	453	rural/clinic	No	25/04/2017	0	0	0
2014327244	453	rural/clinic	No	25/04/2017	0	0	0
2015326537	453	rural/clinic	No	25/04/2017	0	0	0
2015326538	453	rural/clinic	No	25/04/2017	0	0	0
2015326539	453	rural/clinic	No	25/04/2017	0	0	0
2015302410	453	rural/clinic	No	25/04/2017	0	0	0
2015326499	453	rural/clinic	No	25/04/2017	0	0	0
2015340078	453	rural/clinic	No	25/04/2017	0	0	0
2015326500	453	rural/clinic	No	25/04/2017	0	0	0
2015326351	453	rural/clinic	No	25/04/2017	0	0	0
2015340079	453	rural/clinic	No	25/04/2017	0	0	0
2015326352	453	rural/clinic	No	25/04/2017	0	0	0
2015326353	453	rural/clinic	No	25/04/2017	0	0	0
2015326354	453	rural/clinic	No	25/04/2017	0	0	0
2012368536	453	rural/clinic	No	25/04/2017	0	0	0
2015326355	453	rural/clinic	No	25/04/2017	0	0	0
2012368539	453	rural/clinic	No	25/04/2017	0	0	0
2015326356	453	rural/clinic	No	25/04/2017	0	0	0
2015326357	453	rural/clinic	No	25/04/2017	0	0	0
2012259875	453	rural/clinic	No	25/04/2017	0	0	0
2015302406	453	rural/clinic	No	25/04/2017	0	0	0
2015335586	453	rural/clinic	No	25/04/2017	0	0	0
2015340080	453	rural/clinic	No	25/04/2017	0	0	0
2014343875	453	rural/clinic	No	25/04/2017	0	0	0
2015340082	453	rural/clinic	No	25/04/2017	0	0	0
2015384807	453	rural/clinic	No	25/04/2017	0	0	0
2015340081	453	rural/clinic	No	25/04/2017	0	0	0
2015302407	453	rural/clinic	No	25/04/2017	0	0	0
2015337967	453	rural/clinic	No	25/04/2017	0	0	0
2012261382	453	rural/clinic	No	25/04/2017	0	0	0
2014372769	453	rural/clinic	No	25/04/2017	0	0	0
2014314467	453	rural/clinic	No	25/04/2017	0	0	0
2015335439	453	rural/clinic	No	25/04/2017	0	0	0
2015335437	453	rural/clinic	No	25/04/2017	0	0	0
2015335957	453	rural/clinic	No	25/04/2017	0	0	0
2015355776	453	rural/clinic	No	25/04/2017	0	0	0
2015355737	453	rural/clinic	No	25/04/2017	0	0	0
2014314465	453	rural/clinic	No	25/04/2017	0	0	0
2014314466	453	rural/clinic	No	25/04/2017	0	0	0
2015335442	453	rural/clinic	No	25/04/2017	0	0	0
2015337968	453	rural/clinic	No	25/04/2017	0	0	0
2014340324	453	rural/clinic	No	25/04/2017	0	0	0
2015325931	453	rural/clinic	No	25/04/2017	0	0	0
2015382912	453	rural/clinic	No	25/04/2017	0	0	0
2015382906	453	rural/clinic	No	25/04/2017	0	0	0
2015355738	453	rural/clinic	No	25/04/2017	0	0	0
2015382902	453	rural/clinic	No	25/04/2017	0	0	0
2014340323	453	rural/clinic	No	25/04/2017	0	0	0
2015335587	453	rural/clinic	No	25/04/2017	0	0	0
2015382903	453	rural/clinic	No	25/04/2017	0	0	0
2015382904	453	rural/clinic	No	25/04/2017	0	0	0
2015355736	453	rural/clinic	No	25/04/2017	0	0	0
2015382905	453	rural/clinic	No	25/04/2017	0	0	0
2015382910	453	rural/clinic	No	25/04/2017	0	0	0
2015382909	453	rural/clinic	No	25/04/2017	0	0	0
2015382908	453	rural/clinic	No	25/04/2017	0	0	0
2015382900	453	rural/clinic	No	25/04/2017	0	0	0
2015382899	453	rural/clinic	No	25/04/2017	0	0	0
2015382898	453	rural/clinic	No	25/04/2017	0	0	0

2015382896	453	rural/clinic	No	25/04/2017	0	0	0
2015382897	453	rural/clinic	No	25/04/2017	0	0	0
2015382901	453	rural/clinic	No	25/04/2017	0	0	0
2015349003	453	rural/clinic	No	25/04/2017	0	0	0
2015349004	453	rural/clinic	No	25/04/2017	0	0	0
2011193272	453	rural/clinic	No	25/04/2017	0	0	0
2014303338	453	rural/clinic	No	25/04/2017	0	0	0
2015360726	453	rural/clinic	No	25/04/2017	0	0	0
2015339548	453	rural/clinic	No	25/04/2017	0	0	0
2015339549	453	rural/clinic	No	25/04/2017	0	0	0
2014307327	453	rural/clinic	No	27/04/2017	0	0	0
2015351430	453	rural/clinic	No	27/04/2017	0	0	0
2014373099	453	rural/clinic	No	27/04/2017	0	0	0
2015415053	453	rural/clinic	No	27/04/2017	0	0	0
2014307325	453	rural/clinic	No	27/04/2017	0	0	0
2015364604	453	rural/clinic	No	27/04/2017	0	0	0
2015331929	453	rural/clinic	No	27/04/2017	0	0	0
2015331935	453	rural/clinic	No	27/04/2017	0	0	0
2015331936	453	rural/clinic	No	27/04/2017	0	0	0
2015331934	453	rural/clinic	No	27/04/2017	0	0	0
2014306094	453	rural/clinic	No	27/04/2017	0	0	0
2015412452	453	rural/clinic	No	27/04/2017	0	0	0
2014306091	453	rural/clinic	No	27/04/2017	0	0	0
2015412453	453	rural/clinic	No	27/04/2017	0	0	0
2014306096	453	rural/clinic	No	27/04/2017	0	0	0
2015351431	453	rural/clinic	No	27/04/2017	0	0	0
2014306097	453	rural/clinic	No	27/04/2017	0	0	0
2015351432	453	rural/clinic	No	27/04/2017	0	0	0
2014306092	453	rural/clinic	No	27/04/2017	0	0	0
2015351433	453	rural/clinic	No	27/04/2017	0	0	0
2015288746	453	rural/clinic	No	27/04/2017	0	0	0
2014306098	453	rural/clinic	No	27/04/2017	0	0	0
2015404953	453	rural/clinic	No	27/04/2017	0	0	0
2014306093	453	rural/clinic	No	27/04/2017	0	0	0
2015404952	453	rural/clinic	No	27/04/2017	0	0	0
2014306095	453	rural/clinic	No	27/04/2017	0	0	0
2015288745	453	rural/clinic	No	27/04/2017	0	0	0
2015414380	453	rural/clinic	No	27/04/2017	0	0	0
2015288744	453	rural/clinic	No	27/04/2017	0	0	0
2012359157	453	rural/clinic	No	27/04/2017	0	0	0
2014350453	453	rural/clinic	No	27/04/2017	0	0	0
2011117496	453	rural/clinic	No	27/04/2017	0	0	0
2015355914	453	rural/clinic	No	27/04/2017	0	0	0
2015288743	453	rural/clinic	No	27/04/2017	0	0	0
2015355913	81	rural/clinic	No	27/04/2017	0	0	0
2014350452	924	rural/clinic	No	27/04/2017	0	0	0
2015377885	924	rural/clinic	No	27/04/2017	0	0	0
2015355912	924	rural/clinic	No	27/04/2017	0	0	0
2014378267	924	rural/clinic	No	27/04/2017	0	0	0
2015355911	924	rural/clinic	No	27/04/2017	0	0	0
2015369398	924	rural/clinic	No	27/04/2017	0	0	0
2015369399	924	rural/clinic	No	27/04/2017	0	0	0
2015377884	924	rural/clinic	No	27/04/2017	0	0	0
2015288505	924	rural/clinic	No	27/04/2017	0	0	0
2014314214	924	rural/clinic	No	27/04/2017	0	0	0
2015377883	924	rural/clinic	No	27/04/2017	0	0	0
2015340969	924	rural/clinic	No	27/04/2017	0	0	0
2015368283	924	rural/clinic	No	27/04/2017	0	0	0
2014370434	924	rural/clinic	No	27/04/2017	0	0	0
2015368282	924	rural/clinic	No	27/04/2017	0	0	0
2015340968	924	rural/clinic	No	27/04/2017	0	0	0
2014370432	924	rural/clinic	No	27/04/2017	0	0	0
2015340971	924	rural/clinic	No	27/04/2017	0	0	0
2012365921	924	rural/clinic	No	27/04/2017	0	0	0
2015328053	924	rural/clinic	No	27/04/2017	0	0	0
2015340972	924	rural/clinic	No	27/04/2017	0	0	0
2015368284	924	rural/clinic	No	27/04/2017	0	0	0
2015328056	924	rural/clinic	No	27/04/2017	0	0	0
2015328054	924	rural/clinic	No	27/04/2017	0	0	0
2015368281	924	rural/clinic	No	27/04/2017	0	0	0
2015340970	924	rural/clinic	No	27/04/2017	0	0	0
2012359158	924	rural/clinic	No	27/04/2017	0	0	0
2015368206	924	rural/clinic	No	27/04/2017	0	0	0
2015328055	924	rural/clinic	No	27/04/2017	0	0	0
2015301624	924	rural/clinic	No	27/04/2017	0	0	0
2015326573	924	rural/clinic	No	27/04/2017	0	0	0
2014291879	924	rural/clinic	No	27/04/2017	0	0	0
2015326571	924	rural/clinic	No	27/04/2017	0	0	0
2011215294	924	rural/clinic	No	27/04/2017	0	0	0
2015326365	924	rural/clinic	No	27/04/2017	0	0	0
2015326572	924	rural/clinic	No	27/04/2017	0	0	0
2011215293	924	rural/clinic	No	27/04/2017	0	0	0

2015326570	924	rural/clinic	No	27/04/2017	0	0	0
2015305073	924	rural/clinic	No	27/04/2017	0	0	0
2015326569	924	rural/clinic	No	27/04/2017	0	0	0
2013248189	924	rural/clinic	No	27/04/2017	0	0	0
2015305072	924	rural/clinic	No	27/04/2017	0	0	0
2015368280	924	rural/clinic	No	27/04/2017	0	0	0
2013273643	924	rural/clinic	No	27/04/2017	0	0	0
2011133623	924	rural/clinic	No	27/04/2017	0	0	0
2015360851	924	rural/clinic	No	27/04/2017	0	0	0
2011133624	924	rural/clinic	No	27/04/2017	0	0	0
2011142909	924	rural/clinic	No	27/04/2017	0	0	0
2015286705	924	rural/clinic	No	27/04/2017	0	0	0
2011188714	924	rural/clinic	No	27/04/2017	0	0	0
2011133625	924	rural/clinic	No	27/04/2017	0	0	0
2011133626	924	rural/clinic	No	27/04/2017	0	0	0
2015286296	924	rural/clinic	No	27/04/2017	0	0	0
2011213933	924	rural/clinic	No	27/04/2017	0	0	0
2011133629	924	rural/clinic	No	27/04/2017	0	0	0
2015286295	924	rural/clinic	No	27/04/2017	0	0	0
2015305074	924	rural/clinic	No	27/04/2017	0	0	0
2015286707	924	rural/clinic	No	27/04/2017	0	0	0
2015305075	924	rural/clinic	No	27/04/2017	0	0	0
2015286706	924	rural/clinic	No	27/04/2017	0	0	0
2015408673	924	rural/clinic	No	27/04/2017	0	0	0
2015357655	924	rural/clinic	No	27/04/2017	0	0	0
2015313330	924	rural/clinic	No	27/04/2017	0	0	0
2015340207	924	rural/clinic	No	27/04/2017	0	0	0
2015408674	924	rural/clinic	No	27/04/2017	0	0	0
2015340205	924	rural/clinic	No	27/04/2017	0	0	0
2015406488	924	rural/clinic	No	27/04/2017	0	0	0
2015357647	924	rural/clinic	No	27/04/2017	0	0	0
2015313331	924	rural/clinic	No	27/04/2017	0	0	0
2015313334	924	rural/clinic	No	27/04/2017	0	0	0
2015406487	924	rural/clinic	No	27/04/2017	0	0	0
2011200077	924	rural/clinic	No	27/04/2017	0	0	0
2015313323	924	rural/clinic	No	27/04/2017	0	0	0
2015340208	924	rural/clinic	No	27/04/2017	0	0	0
2015406476	924	rural/clinic	No	27/04/2017	0	0	0
2015313320	924	rural/clinic	No	27/04/2017	0	0	0
2015340209	924	rural/clinic	No	27/04/2017	0	0	0
2015406490	924	rural/clinic	No	27/04/2017	0	0	0
2015313322	924	rural/clinic	No	27/04/2017	0	0	0
20133255323	924	rural/clinic	No	27/04/2017	0	0	0
2015313325	924	rural/clinic	No	27/04/2017	0	0	0
2015406481	924	rural/clinic	No	27/04/2017	0	0	0
2013255331	924	rural/clinic	No	27/04/2017	0	0	0
2013255317	924	rural/clinic	No	27/04/2017	0	0	0
2015406480	924	rural/clinic	No	27/04/2017	0	0	0
2014371393	924	rural/clinic	No	27/04/2017	0	0	0
2015332458	924	rural/clinic	No	27/04/2017	0	0	0
2014291880	924	rural/clinic	No	27/04/2017	0	0	0
2014360885	924	rural/clinic	No	27/04/2017	0	0	0
2014312406	924	rural/clinic	No	27/04/2017	0	0	0
2015303513	924	rural/clinic	No	27/04/2017	0	0	0
2014312407	924	rural/clinic	No	27/04/2017	0	0	0
2015413998	924	rural/clinic	No	27/04/2017	0	0	0
2015332457	924	rural/clinic	No	27/04/2017	0	0	0
2015413897	924	rural/clinic	No	27/04/2017	0	0	0
2012359938	924	rural/clinic	No	27/04/2017	0	0	0
2014335245	924	rural/clinic	No	27/04/2017	0	0	0
2014372245	924	rural/clinic	No	27/04/2017	0	0	0
2012359937	924	rural/clinic	No	27/04/2017	0	0	0
2014335246	924	rural/clinic	No	27/04/2017	0	0	0
2015344732	924	rural/clinic	No	27/04/2017	0	0	0
2012359936	924	rural/clinic	No	27/04/2017	0	0	0
2014335552	924	rural/clinic	No	27/04/2017	0	0	0
2014335243	924	rural/clinic	No	27/04/2017	0	0	0
2012359934	924	rural/clinic	No	27/04/2017	0	0	0
2014335553	265	rural/clinic	No	27/04/2017	0	0	0
2014335242	265	rural/clinic	No	27/04/2017	0	0	0
2012359935	265	rural/clinic	No	27/04/2017	0	0	0
2015334735	265	rural/clinic	No	27/04/2017	0	0	0
2015334737	265	rural/clinic	No	27/04/2017	0	0	0
2014335240	265	rural/clinic	No	27/04/2017	0	0	0
2012359932	265	rural/clinic	No	27/04/2017	0	0	0
2014335241	265	rural/clinic	No	27/04/2017	0	0	0
2015334733	265	rural/clinic	No	27/04/2017	0	0	0
2012359933	265	rural/clinic	No	27/04/2017	0	0	0
2015334732	265	rural/clinic	No	27/04/2017	0	0	0
2015324238	265	rural/clinic	No	27/04/2017	0	0	0
2013263595	265	rural/clinic	No	27/04/2017	0	0	0
2015334736	265	rural/clinic	No	27/04/2017	0	0	0

2014346316	265	rural/clinic	No	27/04/2017	0	0	0
2013263597	265	rural/clinic	No	27/04/2017	0	0	0
2014335554	265	rural/clinic	No	27/04/2017	0	0	0
2015352706	265	rural/clinic	No	27/04/2017	0	0	0
2014335555	265	rural/clinic	No	27/04/2017	0	0	0
2014335557	265	rural/clinic	No	27/04/2017	0	0	0
2015352707	265	rural/clinic	No	27/04/2017	0	0	0
2014335551	265	rural/clinic	No	27/04/2017	0	0	0
2014335556	265	rural/clinic	No	27/04/2017	0	0	0
2011135659	265	rural/clinic	No	27/04/2017	0	0	0
2014314549	265	rural/clinic	No	27/04/2017	0	0	0
2011135660	265	rural/clinic	No	27/04/2017	0	0	0
2014314550	265	rural/clinic	No	27/04/2017	0	0	0
2014327502	265	rural/clinic	No	27/04/2017	0	0	0
2015344876	265	rural/clinic	No	27/04/2017	0	0	0
2014287176	265	rural/clinic	No	27/04/2017	0	0	0
2014327503	265	rural/clinic	No	27/04/2017	0	0	0
2015344877	265	rural/clinic	No	27/04/2017	0	0	0
2014327504	265	rural/clinic	No	27/04/2017	0	0	0
2014327505	265	rural/clinic	No	27/04/2017	0	0	0
2015344878	279	rural/clinic	No	27/04/2017	0	0	0
2014327506	279	rural/clinic	No	27/04/2017	0	0	0
2014294151	279	rural/clinic	No	27/04/2017	0	0	0
2015344879	279	rural/clinic	No	27/04/2017	0	0	0
2014327501	279	rural/clinic	No	27/04/2017	0	0	0
2014294152	279	rural/clinic	No	27/04/2017	0	0	0
2015415052	279	rural/clinic	No	27/04/2017	0	0	0
2015413649	279	rural/clinic	No	27/04/2017	0	0	0
2014317344	279	rural/clinic	No	27/04/2017	0	0	0
2015413650	279	rural/clinic	No	27/04/2017	0	0	0
2012291849	279	rural/clinic	No	27/04/2017	0	0	0
2015364509	279	rural/clinic	No	27/04/2017	0	0	0
2012291848	279	rural/clinic	No	27/04/2017	0	0	0
2015369168	279	rural/clinic	No	27/04/2017	0	0	0
2015415054	279	rural/clinic	No	27/04/2017	0	0	0
2012291850	279	rural/clinic	No	27/04/2017	0	0	0
2014307326	279	rural/clinic	No	27/04/2017	0	0	0
2014346315	279	rural/clinic	No	27/04/2017	0	0	0
2015369171	279	rural/clinic	No	27/04/2017	0	0	0
2015369170	279	rural/clinic	No	27/04/2017	0	0	0
2015408173	279	rural/clinic	No	27/04/2017	0	0	0
2015360852	279	rural/clinic	No	27/04/2017	0	0	0
2015369169	279	rural/clinic	No	27/04/2017	0	0	0
2014372332	279	rural/clinic	No	27/04/2017	0	0	0
2015326574	279	rural/clinic	No	27/04/2017	0	0	0
2014372333	279	rural/clinic	No	27/04/2017	0	0	0
2015369172	279	rural/clinic	No	27/04/2017	0	0	0
2015413895	279	rural/clinic	No	27/04/2017	0	0	0
2015332910	279	rural/clinic	No	27/04/2017	0	0	0
2014329070	279	rural/clinic	No	27/04/2017	0	0	0
2015332911	279	rural/clinic	No	27/04/2017	0	0	0
2015369167	279	rural/clinic	No	27/04/2017	0	0	0
2015349061	279	rural/clinic	No	27/04/2017	0	0	0
2013258452	279	rural/clinic	No	27/04/2017	0	0	0
2015324239	279	rural/clinic	No	27/04/2017	0	0	0
2012284484	279	rural/clinic	No	27/04/2017	0	0	0
2015368575	279	rural/clinic	No	27/04/2017	0	0	0
2015384482	279	rural/clinic	No	27/04/2017	0	0	0
2015352708	279	rural/clinic	No	27/04/2017	0	0	0
2015384483	279	rural/clinic	No	27/04/2017	0	0	0
2015368576	279	rural/clinic	No	27/04/2017	0	0	0
2015372918	279	rural/clinic	No	27/04/2017	0	0	0
2015402384	279	rural/clinic	No	27/04/2017	0	0	0
2015418372	279	rural/clinic	No	27/04/2017	0	0	0
2015402385	279	rural/clinic	No	27/04/2017	0	0	0
2012345736	279	rural/clinic	No	27/04/2017	0	0	0
2015402386	279	rural/clinic	No	27/04/2017	0	0	0
2015352363	279	rural/clinic	No	27/04/2017	0	0	0
2015402387	279	rural/clinic	No	27/04/2017	0	0	0
2015352364	279	rural/clinic	No	27/04/2017	0	0	0
2015402388	279	rural/clinic	No	27/04/2017	0	0	0
2015352365	279	rural/clinic	No	27/04/2017	0	0	0
2015402389	279	rural/clinic	No	27/04/2017	0	0	0
2014369844	279	rural/clinic	No	27/04/2017	0	0	0
2011155228	279	rural/clinic	No	27/04/2017	0	0	0
2015358186	279	rural/clinic	No	27/04/2017	0	0	0
2015358187	279	rural/clinic	No	27/04/2017	0	0	0
2015358185	279	rural/clinic	No	27/04/2017	0	0	0
2014329071	279	rural/clinic	No	27/04/2017	0	0	0
2015377882	279	rural/clinic	No	27/04/2017	0	0	0
2015383713	279	rural/clinic	No	27/04/2017	0	0	0
2015352709	279	rural/clinic	No	27/04/2017	0	0	0

2014328436	279	rural/clinic	No	27/04/2017	0	0	0
2015332020	279	rural/clinic	No	27/04/2017	0	0	0
2015297394	279	rural/clinic	No	27/04/2017	0	0	0
2015297328	279	rural/clinic	No	27/04/2017	0	0	0
2015297327	279	rural/clinic	No	27/04/2017	0	0	0
2015297324	279	rural/clinic	No	27/04/2017	0	0	0
2015324258	279	rural/clinic	No	27/04/2017	0	0	0
2015297329	279	rural/clinic	No	27/04/2017	0	0	0
2014378555	279	rural/clinic	No	27/04/2017	0	0	0
2015297325	279	rural/clinic	No	27/04/2017	0	0	0
2014378556	279	rural/clinic	No	27/04/2017	0	0	0
2015297326	279	rural/clinic	No	27/04/2017	0	0	0
2014378554	762	rural/clinic	No	27/04/2017	0	0	0
2015383714	762	rural/clinic	No	27/04/2017	0	0	0
2015383717	762	rural/clinic	No	27/04/2017	0	0	0
2014335300	762	rural/clinic	No	27/04/2017	0	0	0
2015383716	762	rural/clinic	No	27/04/2017	0	0	0
2014378552	762	rural/clinic	No	27/04/2017	0	0	0
2014335299	762	rural/clinic	No	27/04/2017	0	0	0
2015383715	762	rural/clinic	No	27/04/2017	0	0	0
2014334348	762	rural/clinic	No	27/04/2017	0	0	0
2015297399	762	rural/clinic	No	27/04/2017	0	0	0
2014334677	762	rural/clinic	No	27/04/2017	0	0	0
2015297400	762	rural/clinic	No	27/04/2017	0	0	0
2014331410	763	rural/clinic	No	27/04/2017	0	0	0
2015297398	763	rural/clinic	No	27/04/2017	0	0	0
2011128282	763	rural/clinic	No	27/04/2017	0	0	0
2015297397	763	rural/clinic	No	27/04/2017	0	0	0
2015297462	763	rural/clinic	No	27/04/2017	0	0	0
2010099623	763	rural/clinic	No	27/04/2017	0	0	0
2015300693	763	rural/clinic	No	27/04/2017	0	0	0
2015331178	763	rural/clinic	No	27/04/2017	0	0	0
2010099624	763	rural/clinic	No	27/04/2017	0	0	0
2012350629	763	rural/clinic	No	27/04/2017	0	0	0
2015297463	763	rural/clinic	No	27/04/2017	0	0	0
2014287976	763	rural/clinic	No	27/04/2017	0	0	0
2015297464	763	rural/clinic	No	27/04/2017	0	0	0
2014347735	763	rural/clinic	No	27/04/2017	0	0	0
2014347736	763	rural/clinic	No	27/04/2017	0	0	0
2015297465	763	rural/clinic	No	27/04/2017	0	0	0
2014347734	763	rural/clinic	No	27/04/2017	0	0	0
2015297466	763	rural/clinic	No	27/04/2017	0	0	0
2014287975	763	rural/clinic	No	27/04/2017	0	0	0
2012284573	763	rural/clinic	No	27/04/2017	0	0	0
2015297467	763	rural/clinic	No	27/04/2017	0	0	0
2012284743	763	rural/clinic	No	27/04/2017	0	0	0
2014328400	763	rural/clinic	No	27/04/2017	0	0	0
2014334347	763	rural/clinic	No	27/04/2017	0	0	0
2015313328	763	rural/clinic	No	27/04/2017	0	0	0
2015313321	763	rural/clinic	No	27/04/2017	0	0	0
2015313332	577	rural/clinic	No	27/04/2017	0	0	0
2015313326	577	rural/clinic	No	27/04/2017	0	0	0
2015313324	577	rural/clinic	No	27/04/2017	0	0	0
2015313329	117	rural/clinic	No	27/04/2017	0	0	0
2015313333	117	rural/clinic	No	27/04/2017	0	0	0
2015313327	117	rural/clinic	No	27/04/2017	0	0	0
2015362509	117	rural/clinic	No	27/04/2017	0	0	0
2015362510	602	rural/clinic	No	27/04/2017	0	0	0
2015297258	602	rural/clinic	No	27/04/2017	0	0	0
2015297256	602	rural/clinic	No	27/04/2017	0	0	0
2015377432	602	rural/clinic	No	27/04/2017	0	0	0
2013264220	602	rural/clinic	No	27/04/2017	0	0	0
2015297255	602	rural/clinic	No	27/04/2017	0	0	0
2013264221	660	rural/clinic	No	27/04/2017	0	0	0
2014314653	660	rural/clinic	No	27/04/2017	0	0	0
2011124086	660	rural/clinic	No	27/04/2017	0	0	0
2015297257	660	rural/clinic	No	27/04/2017	0	0	0
2015351428	660	rural/clinic	No	27/04/2017	0	0	0
2015297099	660	rural/clinic	No	27/04/2017	0	0	0
2015351429	660	rural/clinic	No	27/04/2017	0	0	0
2015294178	660	rural/clinic	No	27/04/2017	0	0	0
2013247714	660	rural/clinic	No	27/04/2017	0	0	0
2015403272	660	rural/clinic	No	27/04/2017	0	0	0
2015403273	660	rural/clinic	No	27/04/2017	0	0	0
2014298039	660	rural/clinic	No	27/04/2017	0	0	0
2015403270	660	rural/clinic	No	27/04/2017	0	0	0
2015331637	660	rural/clinic	No	27/04/2017	0	0	0
2015403269	660	rural/clinic	No	27/04/2017	0	0	0
2015331639	660	rural/clinic	No	27/04/2017	0	0	0
2015403251	660	rural/clinic	No	27/04/2017	0	0	0
2015331650	660	rural/clinic	No	27/04/2017	0	0	0
2015403259	660	rural/clinic	No	27/04/2017	0	0	0

2015294177	660	rural/clinic	No	27/04/2017	0	0	0
2012369967	660	rural/clinic	No	27/04/2017	0	0	0
2015331648	578	rural/clinic	No	27/04/2017	0	0	0
2015339016	578	rural/clinic	No	27/04/2017	0	0	0
2015339462	578	rural/clinic	No	27/04/2017	0	0	0
2013247716	578	rural/clinic	No	27/04/2017	0	0	0
2014300426	578	rural/clinic	No	27/04/2017	0	0	0
2015403261	578	rural/clinic	No	27/04/2017	0	0	0
2015403262	764	rural/clinic	No	27/04/2017	0	0	0
2015403263	764	rural/clinic	No	27/04/2017	0	0	0
2015403271	764	rural/clinic	No	27/04/2017	0	0	0
2015331649	764	rural/clinic	No	27/04/2017	0	0	0
2013247715	764	rural/clinic	No	27/04/2017	0	0	0
2015372803	764	rural/clinic	No	25/04/2017	0	0	0
2015372803	764	rural/clinic	No	25/04/2017	0	0	0
2014302981	764	rural/clinic	No	25/04/2017	0	0	0
2014302981	764	rural/clinic	No	25/04/2017	0	0	0
2015325993	764	rural/clinic	No	25/04/2017	0	0	0
2015325993	764	rural/clinic	No	25/04/2017	0	0	0
2015331895	764	rural/clinic	No	25/04/2017	0	0	0
2015331895	764	rural/clinic	No	25/04/2017	0	0	0
2012266993	764	rural/clinic	No	25/04/2017	0	0	0
2012266995	764	rural/clinic	No	25/04/2017	0	0	0
2012266994	764	rural/clinic	No	25/04/2017	0	0	0
2015368813	764	rural/clinic	No	25/04/2017	0	0	0
2015368811	764	rural/clinic	No	25/04/2017	0	0	0
2014305220	764	rural/clinic	No	25/04/2017	0	0	0
2014305220	764	rural/clinic	No	25/04/2017	0	0	0
2015409007	764	rural/clinic	No	25/04/2017	0	0	0
2015409007	764	rural/clinic	No	25/04/2017	0	0	0
2015402611	764	rural/clinic	No	25/04/2017	0	0	0
2015402611	764	rural/clinic	No	25/04/2017	0	0	0
2015413134	764	rural/clinic	No	25/04/2017	0	0	0
2015413134	764	rural/clinic	No	25/04/2017	0	0	0
2011152131	764	rural/clinic	No	25/04/2017	0	0	0
2011152131	764	rural/clinic	No	25/04/2017	0	0	0
2015405410	764	rural/clinic	No	25/04/2017	0	0	0
2015363057	764	rural/clinic	No	25/04/2017	0	0	0
2015363057	764	rural/clinic	No	25/04/2017	0	0	0
2013261931	764	rural/clinic	No	25/04/2017	0	0	0
2013261931	764	rural/clinic	No	25/04/2017	0	0	0
2012304332	764	rural/clinic	No	25/04/2017	0	0	0
2012304332	764	rural/clinic	No	25/04/2017	0	0	0
2015402604	764	rural/clinic	No	25/04/2017	0	0	0
2015402604	764	rural/clinic	No	25/04/2017	0	0	0
2015402605	764	rural/clinic	No	25/04/2017	0	0	0
2015402605	764	rural/clinic	No	25/04/2017	0	0	0
2011225405	764	rural/clinic	No	25/04/2017	0	0	0
2012363961	328	rural/clinic	No	25/04/2017	0	0	0
2012363961	328	rural/clinic	No	25/04/2017	0	0	0
2015377322	328	rural/clinic	No	02/05/2017	0	0	0
2015293467	328	rural/clinic	No	02/05/2017	0	0	0
2014289417	328	rural/clinic	No	02/05/2017	0	0	0
2015335106	328	rural/clinic	No	02/05/2017	0	0	0
2015377170	328	rural/clinic	No	02/05/2017	0	0	0
2015335107	328	rural/clinic	No	02/05/2017	0	0	0
2014368021	328	rural/clinic	No	02/05/2017	0	0	0
2015375210	328	rural/clinic	No	02/05/2017	0	0	0
2015384541	328	rural/clinic	No	02/05/2017	0	0	0
2014339741	328	rural/clinic	No	02/05/2017	0	0	0
2014339742	328	rural/clinic	No	02/05/2017	0	0	0
2011199082	328	rural/clinic	No	02/05/2017	0	0	0
2014339743	328	rural/clinic	No	02/05/2017	0	0	0
2014339740	328	rural/clinic	No	02/05/2017	0	0	0
2015293468	328	rural/clinic	No	02/05/2017	0	0	0
2015301469	328	rural/clinic	No	02/05/2017	0	0	0
2015301470	328	rural/clinic	No	02/05/2017	0	0	0
2015293470	328	rural/clinic	No	02/05/2017	0	0	0
2015349531	328	rural/clinic	No	02/05/2017	0	0	0
2014327042	328	rural/clinic	No	02/05/2017	0	0	0
2011226480	328	rural/clinic	No	02/05/2017	0	0	0
2015293471	328	rural/clinic	No	02/05/2017	0	0	0
2012251545	720	rural/clinic	No	02/05/2017	0	0	0
2015397610	720	rural/clinic	No	02/05/2017	0	0	0
2015384540	720	rural/clinic	No	02/05/2017	0	0	0
2015397609	720	rural/clinic	No	02/05/2017	0	0	0
2015377169	720	rural/clinic	No	02/05/2017	0	0	0
2015384538	720	rural/clinic	No	02/05/2017	0	0	0
2014358209	118	rural/clinic	No	02/05/2017	0	0	0
2015413901	118	rural/clinic	No	02/05/2017	0	0	0
2014309588	118	rural/clinic	No	02/05/2017	0	0	0
2015335108	118	rural/clinic	No	02/05/2017	0	0	0

2011135658	118	rural/clinic	No	02/05/2017	0	0	0
2015326358	118	rural/clinic	No	02/05/2017	0	0	0
2015331772	118	rural/clinic	No	02/05/2017	0	0	0
2011135654	118	rural/clinic	No	02/05/2017	0	0	0
2015326359	118	rural/clinic	No	02/05/2017	0	0	0
2015334922	118	rural/clinic	No	02/05/2017	0	0	0
2015326360	118	rural/clinic	No	02/05/2017	0	0	0
2014287898	118	rural/clinic	No	02/05/2017	0	0	0
2015334923	118	rural/clinic	No	02/05/2017	0	0	0
2015326361	118	rural/clinic	No	02/05/2017	0	0	0
2014288007	118	rural/clinic	No	02/05/2017	0	0	0
2015326362	118	rural/clinic	No	02/05/2017	0	0	0
2012253967	118	rural/clinic	No	02/05/2017	0	0	0
2015334924	118	rural/clinic	No	02/05/2017	0	0	0
2015326363	118	rural/clinic	No	02/05/2017	0	0	0
2015326364	118	rural/clinic	No	02/05/2017	0	0	0
2015326541	118	rural/clinic	No	02/05/2017	0	0	0
2015344845	118	rural/clinic	No	02/05/2017	0	0	0
2012269014	118	rural/clinic	No	02/05/2017	0	0	0
2015293835	118	rural/clinic	No	02/05/2017	0	0	0
2015297392	118	rural/clinic	No	02/05/2017	0	0	0
2015326540	118	rural/clinic	No	02/05/2017	0	0	0
2015297321	118	rural/clinic	No	02/05/2017	0	0	0
2015335112	118	rural/clinic	No	02/05/2017	0	0	0
2015303180	118	rural/clinic	No	02/05/2017	0	0	0
2015297323	118	rural/clinic	No	02/05/2017	0	0	0
2015334620	118	rural/clinic	No	02/05/2017	0	0	0
2015303184	118	rural/clinic	No	02/05/2017	0	0	0
2015297322	118	rural/clinic	No	02/05/2017	0	0	0
2015335113	118	rural/clinic	No	02/05/2017	0	0	0
2015397606	118	rural/clinic	No	02/05/2017	0	0	0
2011195850	118	rural/clinic	No	02/05/2017	0	0	0
2015335111	118	rural/clinic	No	02/05/2017	0	0	0
2014328398	661	rural/clinic	No	02/05/2017	0	0	0
2015384539	661	rural/clinic	No	02/05/2017	0	0	0
2015397607	661	rural/clinic	No	02/05/2017	0	0	0
2012320386	661	rural/clinic	No	02/05/2017	0	0	0
2015349534	661	rural/clinic	No	02/05/2017	0	0	0
2015397605	661	rural/clinic	No	02/05/2017	0	0	0
2012320388	661	rural/clinic	No	02/05/2017	0	0	0
2015349533	661	rural/clinic	No	02/05/2017	0	0	0
2015359847	661	rural/clinic	No	02/05/2017	0	0	0
2015397604	661	rural/clinic	No	02/05/2017	0	0	0
2012320387	661	rural/clinic	No	02/05/2017	0	0	0
2015369400	661	rural/clinic	No	02/05/2017	0	0	0
2015290024	661	rural/clinic	No	02/05/2017	0	0	0
2015417051	661	rural/clinic	No	02/05/2017	0	0	0
2011193271	661	rural/clinic	No	02/05/2017	0	0	0
2015417052	661	rural/clinic	No	02/05/2017	0	0	0
2015303511	661	rural/clinic	No	02/05/2017	0	0	0
2015377876	661	rural/clinic	No	02/05/2017	0	0	0
2015417053	661	rural/clinic	No	02/05/2017	0	0	0
2015310658	661	rural/clinic	No	02/05/2017	0	0	0
2015377875	661	rural/clinic	No	02/05/2017	0	0	0
2015310296	661	rural/clinic	No	02/05/2017	0	0	0
2012262489	661	rural/clinic	No	02/05/2017	0	0	0
2015310295	661	rural/clinic	No	02/05/2017	0	0	0
2015297382	661	rural/clinic	No	02/05/2017	0	0	0
2015303512	661	rural/clinic	No	02/05/2017	0	0	0
2015297388	661	rural/clinic	No	02/05/2017	0	0	0
2011135655	244	rural/clinic	No	02/05/2017	0	0	0
2015400052	244	rural/clinic	No	02/05/2017	0	0	0
2012262058/D	244	rural/clinic	No	02/05/2017	0	0	0
2015400051	244	rural/clinic	No	02/05/2017	0	0	0
201482915	244	rural/clinic	No	02/05/2017	0	0	0
2011135656	244	rural/clinic	No	02/05/2017	0	0	0
2015412088	244	rural/clinic	No	02/05/2017	0	0	0
2015400050	244	rural/clinic	No	02/05/2017	0	0	0
2011135657	244	rural/clinic	No	02/05/2017	0	0	0
2015331894	244	rural/clinic	No	02/05/2017	0	0	0
2015397868	244	rural/clinic	No	02/05/2017	0	0	0
2015400049	244	rural/clinic	No	02/05/2017	0	0	0
2015293469	244	rural/clinic	No	02/05/2017	0	0	0
2015400048	244	rural/clinic	No	02/05/2017	0	0	0
2014289421	244	rural/clinic	No	02/05/2017	0	0	0
2015397869	244	rural/clinic	No	02/05/2017	0	0	0
2015400047	244	rural/clinic	No	02/05/2017	0	0	0
2015336322	244	rural/clinic	No	02/05/2017	0	0	0
2015400046	244	rural/clinic	No	02/05/2017	0	0	0
2014289418	244	rural/clinic	No	02/05/2017	0	0	0
2015400045	244	rural/clinic	No	02/05/2017	0	0	0
2015383710	795	rural/clinic	No	02/05/2017	0	0	0

2015334731	795	rural/clinic	No	02/05/2017	0	0	0
2015400044	795	rural/clinic	No	02/05/2017	0	0	0
2014372240	546	rural/clinic	No	02/05/2017	0	0	0
2014289422	546	rural/clinic	No	02/05/2017	0	0	0
2015397866	546	rural/clinic	No	02/05/2017	0	0	0
2014372239	546	rural/clinic	No	02/05/2017	0	0	0
2014289424	546	rural/clinic	No	02/05/2017	0	0	0
2015418141	546	rural/clinic	No	02/05/2017	0	0	0
2014372241	546	rural/clinic	No	02/05/2017	0	0	0
2014289423	546	rural/clinic	No	02/05/2017	0	0	0
2015334729	546	rural/clinic	No	02/05/2017	0	0	0
2014358208	546	rural/clinic	No	02/05/2017	0	0	0
2015297391	546	rural/clinic	No	02/05/2017	0	0	0
2015372401	546	rural/clinic	No	02/05/2017	0	0	0
2015418142	546	rural/clinic	No	02/05/2017	0	0	0
2014289519	546	rural/clinic	No	02/05/2017	0	0	0
2015383711	546	rural/clinic	No	02/05/2017	0	0	0
2014309589	546	rural/clinic	No	02/05/2017	0	0	0
2015372404	546	rural/clinic	No	02/05/2017	0	0	0
2015310657	546	rural/clinic	No	02/05/2017	0	0	0
2014328399	546	rural/clinic	No	02/05/2017	0	0	0
2014289420	546	rural/clinic	No	02/05/2017	0	0	0
2015365086	546	rural/clinic	No	02/05/2017	0	0	0
2014378729	383	rural/clinic	No	02/05/2017	0	0	0
2015372402	383	rural/clinic	No	02/05/2017	0	0	0
2014289416	383	rural/clinic	No	02/05/2017	0	0	0
2015397867	383	rural/clinic	No	02/05/2017	0	0	0
2014378731	383	rural/clinic	No	02/05/2017	0	0	0
2015372917	383	rural/clinic	No	02/05/2017	0	0	0
2015331770	383	rural/clinic	No	02/05/2017	0	0	0
2012369807	383	rural/clinic	No	02/05/2017	0	0	0
2011144808	383	rural/clinic	No	02/05/2017	0	0	0
2015331771	383	rural/clinic	No	02/05/2017	0	0	0
2015335110	383	rural/clinic	No	02/05/2017	0	0	0
2015331769	383	rural/clinic	No	02/05/2017	0	0	0
2015331928	383	rural/clinic	No	02/05/2017	0	0	0
2012369808	383	rural/clinic	No	02/05/2017	0	0	0
2011229167	383	rural/clinic	No	02/05/2017	0	0	0
2015331931	383	rural/clinic	No	02/05/2017	0	0	0
2015363062	383	rural/clinic	No	02/05/2017	0	0	0
2015409006	383	rural/clinic	No	04/05/2017	0	0	0
2013260275	383	rural/clinic	No	02/05/2017	0	0	0
2015363061	383	rural/clinic	No	02/05/2017	0	0	0
2015331932	383	rural/clinic	No	02/05/2017	0	0	0
2015405001	383	rural/clinic	No	02/05/2017	0	0	0
2015409005	383	rural/clinic	No	02/05/2017	0	0	0
2015331930	383	rural/clinic	No	02/05/2017	0	0	0
2014307519	383	rural/clinic	No	02/05/2017	0	0	0
2015331933	383	rural/clinic	No	02/05/2017	0	0	0
2015392885	383	rural/clinic	No	02/05/2017	0	0	0
2015405002	383	rural/clinic	No	02/05/2017	0	0	0
2014307524	383	rural/clinic	No	02/05/2017	0	0	0
2015405003	383	rural/clinic	No	02/05/2017	0	0	0
2015328472	383	rural/clinic	No	02/05/2017	0	0	0
2015405099	383	rural/clinic	No	02/05/2017	0	0	0
2015328470	383	rural/clinic	No	02/05/2017	0	0	0
2014307528	383	rural/clinic	No	02/05/2017	0	0	0
2014307526	383	rural/clinic	No	02/05/2017	0	0	0
2014307533	383	rural/clinic	No	02/05/2017	0	0	0
2015332956	383	rural/clinic	No	02/05/2017	0	0	0
2015405100	383	rural/clinic	No	02/05/2017	0	0	0
2014307534	383	rural/clinic	No	02/05/2017	0	0	0
2014287429	383	rural/clinic	No	02/05/2017	0	0	0
2014369097	383	rural/clinic	No	02/05/2017	0	0	0
2013254667	383	rural/clinic	No	02/05/2017	0	0	0
2014287430	383	rural/clinic	No	02/05/2017	0	0	0
2014287428	383	rural/clinic	No	02/05/2017	0	0	0
2014369096	383	rural/clinic	No	02/05/2017	0	0	0
2013254668	383	rural/clinic	No	02/05/2017	0	0	0
2012259878	383	rural/clinic	No	02/05/2017	0	0	0
2011203835	383	rural/clinic	No	02/05/2017	0	0	0
2015296057	383	rural/clinic	No	02/05/2017	0	0	0
2012259880	383	rural/clinic	No	02/05/2017	0	0	0
2012259876	383	rural/clinic	No	02/05/2017	0	0	0
2013278294	383	rural/clinic	No	02/05/2017	0	0	0
2015372407	383	rural/clinic	No	02/05/2017	0	0	0
2012336620	383	rural/clinic	No	02/05/2017	0	0	0
2015392881	383	rural/clinic	No	02/05/2017	0	0	0
2012336618	383	rural/clinic	No	02/05/2017	0	0	0
2015372406	383	rural/clinic	No	02/05/2017	0	0	0
2015372405	383	rural/clinic	No	02/05/2017	0	0	0
2013327295	383	rural/clinic	No	02/05/2017	0	0	0

2012336617	383	rural/clinic	No	02/05/2017	0	0	0
2015372403	383	rural/clinic	No	02/05/2017	0	0	0
2015314380	383	rural/clinic	No	02/05/2017	0	0	0
2015314078	383	rural/clinic	No	02/05/2017	0	0	0
2015314386	383	rural/clinic	No	02/05/2017	0	0	0
2015314070	383	rural/clinic	No	02/05/2017	0	0	0
2015354953	383	rural/clinic	No	02/05/2017	0	0	0
2015314383	383	rural/clinic	No	02/05/2017	0	0	0
2012300840	383	rural/clinic	No	02/05/2017	0	0	0
2012307521	383	rural/clinic	No	02/05/2017	0	0	0
2015314371	383	rural/clinic	No	02/05/2017	0	0	0
2015314079	383	rural/clinic	No	03/05/2017	0	0	0
2015314381	383	rural/clinic	No	02/05/2017	0	0	0
2015392880	383	rural/clinic	No	02/05/2017	0	0	0
2015314080	383	rural/clinic	No	03/05/2017	0	0	0
2015314385	383	rural/clinic	No	02/05/2017	0	0	0
2015318243	383	rural/clinic	No	02/05/2017	0	0	0
2015314069	383	rural/clinic	No	02/05/2017	0	0	0
2015392879	383	rural/clinic	No	02/05/2017	0	0	0
2015314384	383	rural/clinic	No	02/05/2017	0	0	0
2015354951	383	rural/clinic	No	02/05/2017	0	0	0
2012300838	383	rural/clinic	No	02/05/2017	0	0	0
2012307522	383	rural/clinic	No	02/05/2017	0	0	0
2015394640	383	rural/clinic	No	02/05/2017	0	0	0
2015314081	383	rural/clinic	No	02/05/2017	0	0	0
2014353637	383	rural/clinic	No	02/05/2017	0	0	0
2015415905	383	rural/clinic	No	02/05/2017	0	0	0
2015406489	383	rural/clinic	No	02/05/2017	0	0	0
2014353636	383	rural/clinic	No	02/05/2017	0	0	0
2015301252	383	rural/clinic	No	02/05/2017	0	0	0
2015418853	383	rural/clinic	No	02/05/2017	0	0	0
2015323808	383	rural/clinic	No	02/05/2017	0	0	0
2014382424	694	rural/clinic	No	02/05/2017	0	0	0
2015418854	694	rural/clinic	No	02/05/2017	0	0	0
2015392882	694	rural/clinic	No	02/05/2017	0	0	0
2014327120	694	rural/clinic	No	02/05/2017	0	0	0
2015418855	694	rural/clinic	No	02/05/2017	0	0	0
2015391515	694	rural/clinic	No	02/05/2017	0	0	0
2015418856	95	rural/clinic	No	02/05/2017	0	0	0
2015391516	95	rural/clinic	No	02/05/2017	0	0	0
2014382420	95	rural/clinic	No	02/05/2017	0	0	0
2015374441	95	rural/clinic	No	02/05/2017	0	0	0
2015406484	95	rural/clinic	No	02/05/2017	0	0	0
2015391514	95	rural/clinic	No	02/05/2017	0	0	0
2015406485	95	rural/clinic	No	02/05/2017	0	0	0
2015374440	95	rural/clinic	No	02/05/2017	0	0	0
2015406483	95	rural/clinic	No	02/05/2017	0	0	0
2015391517	95	rural/clinic	No	02/05/2017	0	0	0
2015374439	95	rural/clinic	No	02/05/2017	0	0	0
2014369141	95	rural/clinic	No	02/05/2017	0	0	0
2015406482	95	rural/clinic	No	02/05/2017	0	0	0
2015374438	95	rural/clinic	No	02/05/2017	0	0	0
2015406486	95	rural/clinic	No	02/05/2017	0	0	0
2015364507	95	rural/clinic	No	02/05/2017	0	0	0
2015379560	95	rural/clinic	No	02/05/2017	0	0	0
2014321421	95	rural/clinic	No	02/05/2017	0	0	0
2015379559	95	rural/clinic	No	02/05/2017	0	0	0
2014321420	95	rural/clinic	No	02/05/2017	0	0	0
2015379558	95	rural/clinic	No	02/05/2017	0	0	0
2014321422	95	rural/clinic	No	02/05/2017	0	0	0
2015379557	95	rural/clinic	No	02/05/2017	0	0	0
2015364508	95	rural/clinic	No	02/05/2017	0	0	0
2015379555	95	rural/clinic	No	02/05/2017	0	0	0
2015364504	95	rural/clinic	No	02/05/2017	0	0	0
2015379556	95	rural/clinic	No	02/05/2017	0	0	0
2014378730	95	rural/clinic	No	02/05/2017	0	0	0
2015379554	95	rural/clinic	No	02/05/2017	0	0	0
2014321423	95	rural/clinic	No	02/05/2017	0	0	0
2015379553	95	rural/clinic	No	02/05/2017	0	0	0
2014369143	95	rural/clinic	No	02/05/2017	0	0	0
2015348893	95	rural/clinic	No	02/05/2017	0	0	0
2014321424	95	rural/clinic	No	02/05/2017	0	0	0
2014307531	95	rural/clinic	No	02/05/2017	0	0	0
2015328529	95	rural/clinic	No	02/05/2017	0	0	0
2015348899	95	rural/clinic	No	02/05/2017	0	0	0
2015328530	95	rural/clinic	No	02/05/2017	0	0	0
2014366652	329	rural/clinic	No	02/05/2017	0	0	0
2014382435	329	rural/clinic	No	02/05/2017	0	0	0
2015301261	329	rural/clinic	No	02/05/2017	0	0	0
2014382440	329	rural/clinic	No	02/05/2017	0	0	0
2014382441	329	rural/clinic	No	02/05/2017	0	0	0
2015297390	329	rural/clinic	No	02/05/2017	0	0	0

2015297100	329	rural/clinic	No	02/05/2017	0	0	0
2015297254	329	rural/clinic	No	02/05/2017	0	0	0
2015297093	329	rural/clinic	No	02/05/2017	0	0	0
2015297395	329	rural/clinic	No	02/05/2017	0	0	0
2015297396	329	rural/clinic	No	02/05/2017	0	0	0
2015297393	329	rural/clinic	No	02/05/2017	0	0	0
2015369165	329	rural/clinic	No	02/05/2017	0	0	0
2015369166	329	rural/clinic	No	02/05/2017	0	0	0
2015369164	329	rural/clinic	No	02/05/2017	0	0	0
2015323813	329	rural/clinic	No	02/05/2017	0	0	0
2012335665	329	rural/clinic	No	25/04/2017	0	0	0
2011134986	329	rural/clinic	No	25/04/2017	0	0	0
2011134986	329	rural/clinic	No	25/04/2017	0	0	0
2015203622	329	rural/clinic	No	25/04/2017	0	0	0
2011191290	329	rural/clinic	No	25/04/2017	0	0	0
2011191290	329	rural/clinic	No	25/04/2017	0	0	0
2015294054	329	rural/clinic	No	25/04/2017	0	0	0
2015294054	547	district/faith-based	No	25/04/2017	1	0	0
2015357190	547	district/faith-based	No	25/04/2017	1	0	0
2015357190	547	district/faith-based	No	25/04/2017	1	0	0
2015340078	547	district/faith-based	No	25/04/2017	1	0	0
2015340078	547	district/faith-based	No	25/04/2017	1	0	0
2015377432	547	district/faith-based	No	27/04/2017	1	0	0
2015377432	547	district/faith-based	No	27/04/2017	1	0	0
2015351433	547	district/faith-based	No	27/04/2017	1	0	0
2015351433	547	district/faith-based	No	27/04/2017	1	0	0
2014291880	547	district/faith-based	No	27/04/2017	1	0	0
2014291880	547	district/faith-based	No	27/04/2017	1	0	0
2014357732	547	district/faith-based	No	03/05/2017	1	0	0
2014367622	547	district/faith-based	No	03/05/2017	1	0	0
2013255259	547	district/faith-based	No	03/05/2017	1	0	0
2015377829	547	district/faith-based	No	03/05/2017	1	0	0
2015368960	547	district/faith-based	No	03/05/2017	1	0	0
2014307536	547	district/faith-based	No	03/05/2017	1	0	0
2015368961	547	district/faith-based	No	03/05/2017	1	0	0
2012295981	547	district/faith-based	No	03/05/2017	1	0	0
2012295982	547	district/faith-based	No	03/05/2017	1	0	0
2015376208	547	district/faith-based	No	03/05/2017	1	0	0
2012295983	547	district/faith-based	No	03/05/2017	1	0	0
2013266073	547	district/faith-based	No	03/05/2017	1	0	0
2014307535	547	district/faith-based	No	03/05/2017	1	0	0
2013266074	547	district/faith-based	No	03/05/2017	1	0	0
2014366069	547	district/faith-based	No	03/05/2017	1	0	0
2014374015	547	district/faith-based	No	03/05/2017	1	0	0
2014374014	547	district/faith-based	No	03/05/2017	1	0	0
2012335666	547	district/faith-based	No	03/05/2017	1	0	0
2014374013	547	district/faith-based	No	03/05/2017	1	0	0
2014307413	547	district/faith-based	No	03/05/2017	1	0	0
2014374011	547	district/faith-based	No	03/05/2017	1	0	0
2014307414	547	district/faith-based	No	03/05/2017	1	0	0
2014374012	547	district/faith-based	No	03/05/2017	1	0	0
2014298239	547	district/faith-based	No	03/05/2017	1	0	0
2014306010	547	district/faith-based	No	03/05/2017	1	0	0
2014306011	547	district/faith-based	No	03/05/2017	1	0	0
2014306009	547	district/faith-based	No	03/05/2017	1	0	0
2015382916	547	district/faith-based	No	03/05/2017	1	0	0
2015382915	547	district/faith-based	No	03/05/2017	1	0	0
2015382914	547	district/faith-based	No	03/05/2017	1	0	0
2015382913	547	district/faith-based	No	03/05/2017	1	0	0
2014327507	547	district/faith-based	No	03/05/2017	1	0	0
2014327508	547	district/faith-based	No	03/05/2017	1	0	0
2014357733	547	district/faith-based	No	03/05/2017	1	0	0
2014357734	547	district/faith-based	No	03/05/2017	1	0	0
2014357735	547	district/faith-based	No	03/05/2017	1	0	0
2014357736	547	district/faith-based	No	03/05/2017	1	0	0
2015320877	547	district/faith-based	No	03/05/2017	1	0	0
2014357729	547	district/faith-based	No	03/05/2017	1	0	0
2015397865	547	district/faith-based	No	03/05/2017	1	0	0
2015320875	547	district/faith-based	No	03/05/2017	1	0	0
2014357730	547	district/faith-based	No	03/05/2017	1	0	0
2014357731	547	district/faith-based	No	03/05/2017	1	0	0
2015320876	547	district/faith-based	No	03/05/2017	1	0	0
2014297967	547	district/faith-based	No	03/05/2017	1	0	0
2014375688	547	district/faith-based	No	03/05/2017	1	0	0
2012362560	547	district/faith-based	No	03/05/2017	1	0	0
2014375690	547	district/faith-based	No	03/05/2017	1	0	0
2017000029	547	district/faith-based	No	13/04/2017	1	0	0
2014375689	547	district/faith-based	No	03/05/2017	1	0	0
2014307525	547	district/faith-based	No	03/05/2017	1	0	0
2015339151	547	district/faith-based	No	03/05/2017	1	0	0
2014307527	547	district/faith-based	No	03/05/2017	1	0	0
2014375687	547	district/faith-based	No	03/05/2017	1	0	0

2014297968	547	district/faith-based	No	03/05/2017	1	0	0
2015349530	547	district/faith-based	No	19/04/2017	1	0	0
2012362554	547	district/faith-based	No	03/05/2017	1	0	0
2014297969	547	district/faith-based	No	03/05/2017	1	0	0
2014382916	547	district/faith-based	No	03/05/2017	1	0	0
2015377831	547	district/faith-based	No	03/05/2017	1	0	0
2012294060	547	district/faith-based	No	03/05/2017	1	0	0
2012362555	547	district/faith-based	No	03/05/2017	1	0	0
2014375691	547	district/faith-based	No	03/05/2017	1	0	0
2012362556	547	district/faith-based	No	03/05/2017	1	0	0
2015326751	547	district/faith-based	No	03/05/2017	1	0	0
2015289570	547	district/faith-based	No	03/05/2017	1	0	0
2012362558	547	district/faith-based	No	03/05/2017	1	0	0
2015326752	547	district/faith-based	No	03/05/2017	1	0	0
2015326753	547	district/faith-based	No	03/05/2017	1	0	0
2012362557	547	district/faith-based	No	03/05/2017	1	0	0
2015326754	547	district/faith-based	No	03/05/2017	1	0	0
2012362553	547	district/faith-based	No	03/05/2017	1	0	0
2015326755	547	district/faith-based	No	03/05/2017	1	0	0
2012362559	547	district/faith-based	No	03/05/2017	1	0	0
201085806	547	district/faith-based	No	03/05/2017	1	0	0
2015326756	547	district/faith-based	No	03/05/2017	1	0	0
2015376207	547	district/faith-based	No	03/05/2017	1	0	0
2015339550	547	district/faith-based	No	03/05/2017	1	0	0
2014363883	547	district/faith-based	No	03/05/2017	1	0	0
2014363881	547	district/faith-based	No	03/05/2017	1	0	0
2015376206	547	district/faith-based	No	03/05/2017	1	0	0
2014363882	547	district/faith-based	No	03/05/2017	1	0	0
2012276252	547	district/faith-based	No	03/05/2017	1	0	0
2014358700	547	district/faith-based	No	03/05/2017	1	0	0
2015344359	547	district/faith-based	No	03/05/2017	1	0	0
2012289685	547	district/faith-based	No	03/05/2017	1	0	0
2014307529	547	district/faith-based	No	03/05/2017	1	0	0
2014383701	547	district/faith-based	No	03/05/2017	1	0	0
2014307530	547	district/faith-based	No	03/05/2017	1	0	0
2012276150	547	district/faith-based	No	03/05/2017	1	0	0
2013266242	547	district/faith-based	No	03/05/2017	1	0	0
2014307537	547	district/faith-based	No	03/05/2017	1	0	0
2014363880	547	district/faith-based	No	03/05/2017	1	0	0
2014307532	547	district/faith-based	No	03/05/2017	1	0	0
2014363879	547	district/faith-based	No	03/05/2017	1	0	0
2014307538	547	district/faith-based	No	03/05/2017	1	0	0
2012276149	547	district/faith-based	No	03/05/2017	1	0	0
201053903	547	district/faith-based	No	13/04/2017	1	0	0
2013266244	547	district/faith-based	No	03/05/2017	1	0	0
2014371575	547	district/faith-based	No	03/05/2017	1	0	0
2015413905	547	district/faith-based	No	03/05/2017	1	0	0
2017000030	547	district/faith-based	No	03/05/2017	1	0	0
2015413902	547	district/faith-based	No	03/05/2017	1	0	0
2014371576	547	district/faith-based	No	03/05/2017	1	0	0
2015413906	547	district/faith-based	No	03/05/2017	1	0	0
2014327121	547	district/faith-based	No	03/05/2017	1	0	0
2015352704	547	district/faith-based	No	13/04/2017	1	0	0
2015413903	547	district/faith-based	No	03/05/2017	1	0	0
2012306141	547	district/faith-based	No	04/05/2017	1	0	0
2015413904	547	district/faith-based	No	03/05/2017	1	0	0
2012389493	547	district/faith-based	No	13/04/2017	1	0	0
2015357193	547	district/faith-based	No	04/05/2017	1	0	0
2015303626	547	district/faith-based	No	03/05/2017	1	0	0
2011203830	547	district/faith-based	No	13/04/2017	1	0	0
2015303627	547	district/faith-based	No	03/05/2017	1	0	0
2015357529	547	district/faith-based	No	04/05/2017	1	0	0
2015344360	547	district/faith-based	No	03/05/2017	1	0	0
2015302415	547	district/faith-based	No	04/05/2017	1	0	0
2017000031	547	district/faith-based	No	13/04/2017	1	0	0
2015344361	547	district/faith-based	No	03/05/2017	1	0	0
2015302416	547	district/faith-based	No	04/05/2017	1	0	0
2015342310	547	district/faith-based	No	03/05/2017	1	0	0
2015302417	547	district/faith-based	No	04/05/2017	1	0	0
2015342309	547	district/faith-based	No	03/05/2017	1	0	0
2012368542	547	district/faith-based	No	04/05/2017	1	0	0
2012359159	547	district/faith-based	No	03/05/2017	1	0	0
2017000032	547	district/faith-based	No	03/05/2017	1	0	0
2012368543	547	district/faith-based	No	04/05/2017	1	0	0
2012359160	547	district/faith-based	No	03/05/2017	1	0	0
2015368654	547	district/faith-based	No	04/05/2017	1	0	0
2014358211	547	district/faith-based	No	03/05/2017	1	0	0
2017000033	547	district/faith-based	No	03/05/2017	1	0	0
2014358212	547	district/faith-based	No	03/05/2017	1	0	0
2015368656	547	district/faith-based	No	04/05/2017	1	0	0
2015351681	547	district/faith-based	No	04/05/2017	1	0	0
2015418146	547	district/faith-based	No	03/05/2017	1	0	0

2013267892	547	district/faith-based	No	04/05/2017	1	0	0
2015351682	547	district/faith-based	No	04/05/2017	1	0	0
2015418150	547	district/faith-based	No	03/05/2017	1	0	0
2015418149	547	district/faith-based	No	03/05/2017	1	0	0
2015351683	547	district/faith-based	No	04/05/2017	1	0	0
2015331172	547	district/faith-based	No	04/05/2017	1	0	0
2015362476	547	district/faith-based	No	04/05/2017	1	0	0
2014334678	547	district/faith-based	No	04/05/2017	1	0	0
2015289328	547	district/faith-based	No	04/05/2017	1	0	0
2011212962	547	district/faith-based	No	04/05/2017	1	0	0
2014347737	547	district/faith-based	No	04/05/2017	1	0	0
201053904	547	district/faith-based	No	04/05/2017	1	0	0
2012253131	547	district/faith-based	No	04/05/2017	1	0	0
2014342522	547	district/faith-based	No	03/05/2017	1	0	0
2015375105	547	district/faith-based	No	04/05/2017	1	0	0
2015331774	547	district/faith-based	No	04/05/2017	1	0	0
2014342520	547	district/faith-based	No	03/05/2017	1	0	0
2014363591	547	district/faith-based	No	04/05/2017	1	0	0
2014358210	547	district/faith-based	No	03/05/2017	1	0	0
2014342521	547	district/faith-based	No	03/05/2017	1	0	0
2015351685	547	district/faith-based	No	04/05/2017	1	0	0
2015418145	547	district/faith-based	No	03/05/2017	1	0	0
2015360853	547	district/faith-based	No	04/05/2017	1	0	0
2015418148	547	district/faith-based	No	03/05/2017	1	0	0
2015418147	547	district/faith-based	No	03/05/2017	1	0	0
2015360855	547	district/faith-based	No	04/05/2017	1	0	0
2015335960	547	district/faith-based	No	04/05/2017	1	0	0
2015364393	547	district/faith-based	No	06/05/2017	1	0	0
2015361413	547	district/faith-based	No	04/05/2017	1	0	0
2015337971	547	district/faith-based	No	04/05/2017	1	0	0
2015364392	547	district/faith-based	No	03/05/2017	1	0	0
2015337970	547	district/faith-based	No	04/05/2017	1	0	0
2015364391	547	district/faith-based	No	03/05/2017	1	0	0
2015364390	547	district/faith-based	No	03/05/2017	1	0	0
2012316545	547	district/faith-based	No	04/05/2017	1	0	0
2015364389	547	district/faith-based	No	03/05/2017	1	0	0
2015364388	547	district/faith-based	No	03/05/2017	1	0	0
2012317316	547	district/faith-based	No	04/05/2017	1	0	0
2015364387	547	district/faith-based	No	03/05/2017	1	0	0
2012317315	547	district/faith-based	No	04/05/2017	1	0	0
2013266243	547	district/faith-based	No	03/05/2017	1	0	0
2015335588	547	district/faith-based	No	04/05/2017	1	0	0
2015335443	547	district/faith-based	No	04/05/2017	1	0	0
2012317281	547	district/faith-based	No	03/05/2017	1	0	0
2015413137	547	district/faith-based	No	04/05/2017	1	0	0
2015384809	547	district/faith-based	No	03/05/2017	1	0	0
2015289327	547	district/faith-based	No	03/05/2017	1	0	0
2015413138	547	district/faith-based	No	04/05/2017	1	0	0
2015289329	547	district/faith-based	No	03/05/2017	1	0	0
2015413139	547	district/faith-based	No	04/05/2017	1	0	0
2012295142	547	district/faith-based	No	03/05/2017	1	0	0
2015413140	547	district/faith-based	No	04/05/2017	1	0	0
2015413141	547	district/faith-based	No	04/05/2017	1	0	0
2015413412	547	district/faith-based	No	04/05/2017	1	0	0
2014360964	547	district/faith-based	No	03/05/2017	1	0	0
2014360963	547	district/faith-based	No	03/05/2017	1	0	0
2015413143	547	district/faith-based	No	04/05/2017	1	0	0
2014357315	547	district/faith-based	No	03/05/2017	1	0	0
2015413144	547	district/faith-based	No	04/05/2017	1	0	0
2014357316	547	district/faith-based	No	03/05/2017	1	0	0
2014357317	547	district/faith-based	No	03/05/2017	1	0	0
2014357318	547	district/faith-based	No	03/05/2017	1	0	0
2014357319	547	district/faith-based	No	03/05/2017	1	0	0
2014346283	547	district/faith-based	No	03/05/2017	1	0	0
2014358004	547	district/faith-based	No	03/05/2017	1	0	0
2014358005	547	district/faith-based	No	03/05/2017	1	0	0
2012272347	547	district/faith-based	No	03/05/2017	1	0	0
2015388292	330	rural/clinic	No	03/05/2017	0	0	0
2015413145	330	rural/clinic	No	04/05/2017	0	0	0
2015413146	330	rural/clinic	No	04/05/2017	0	0	0
2015413147	330	rural/clinic	No	04/05/2017	0	0	0
2015413148	330	rural/clinic	No	04/05/2017	0	0	0
2015413149	330	rural/clinic	No	04/05/2017	0	0	0
2012336622	330	rural/clinic	No	04/05/2017	0	0	0
2012336621	330	rural/clinic	No	04/05/2017	0	0	0
2012336619	330	rural/clinic	No	04/05/2017	0	0	0
2014302939	330	rural/clinic	No	04/05/2017	0	0	0
2014302940	330	rural/clinic	No	04/05/2017	0	0	0
2014302941	330	rural/clinic	No	04/05/2017	0	0	0
2014328436	330	rural/clinic	No	27/04/2017	0	0	0
2015383716	330	rural/clinic	No	27/04/2017	0	0	0
2015383716	330	rural/clinic	No	27/04/2017	0	0	0

2015369398	330	rural/clinic	No	27/04/2017	0	0	0
2015369398	330	rural/clinic	No	27/04/2017	0	0	0
2014373099	330	rural/clinic	No	27/04/2017	0	0	0
2014373099	330	rural/clinic	No	27/04/2017	0	0	0
2015369170	330	rural/clinic	No	27/04/2017	0	0	0
2015369170	330	rural/clinic	No	27/04/2017	0	0	0
2015301471	330	rural/clinic	No	04/05/2017	0	0	0
2015301472	330	rural/clinic	No	04/05/2017	0	0	0
2015349723	330	rural/clinic	No	04/05/2017	0	0	0
2015349724	330	rural/clinic	No	04/05/2017	0	0	0
2015349725	330	rural/clinic	No	04/05/2017	0	0	0
2015325999	330	rural/clinic	No	04/05/2017	0	0	0
2015326000	330	rural/clinic	No	04/05/2017	0	0	0
2014302848	330	rural/clinic	No	04/05/2017	0	0	0
2011199084	330	rural/clinic	No	04/05/2017	0	0	0
2011225648	330	rural/clinic	No	04/05/2017	0	0	0
2015326536	330	rural/clinic	No	25/04/2017	0	0	0
2015326536	330	rural/clinic	No	25/04/2017	0	0	0
2015349458	330	rural/clinic	No	04/05/2017	0	0	0
2014327037	330	rural/clinic	No	04/05/2017	0	0	0
2015301473	330	rural/clinic	No	04/05/2017	0	0	0
2015301503	330	rural/clinic	No	04/05/2017	0	0	0
2015301263	330	rural/clinic	No	04/05/2017	0	0	0
2015337972	330	rural/clinic	No	03/05/2017	0	0	0
2015301264	330	rural/clinic	No	04/05/2017	0	0	0
2015355739	330	rural/clinic	No	03/05/2017	0	0	0
2015301265	330	rural/clinic	No	04/05/2017	0	0	0
2015337969	330	rural/clinic	No	03/05/2017	0	0	0
2014335247	330	rural/clinic	No	04/05/2017	0	0	0
2012295141	330	rural/clinic	No	03/05/2017	0	0	0
2014335248	330	rural/clinic	No	04/05/2017	0	0	0
2015335958	330	rural/clinic	No	03/05/2017	0	0	0
2015360512	330	rural/clinic	No	04/05/2017	0	0	0
2015335959	330	rural/clinic	No	03/05/2017	0	0	0
2011221923	330	rural/clinic	No	03/05/2017	0	0	0
2014371573	330	rural/clinic	No	04/05/2017	0	0	0
2011221924	330	rural/clinic	No	03/05/2017	0	0	0
2015351684	330	rural/clinic	No	04/05/2017	0	0	0
2015355777	330	rural/clinic	No	03/05/2017	0	0	0
2015313344	330	rural/clinic	No	04/05/2017	0	0	0
2014359731	330	rural/clinic	No	03/05/2017	0	0	0
2015362477	330	rural/clinic	No	03/05/2017	0	0	0
2015335444	330	rural/clinic	No	03/05/2017	0	0	0
2015335445	330	rural/clinic	No	03/05/2017	0	0	0
2015294875	330	rural/clinic	No	03/05/2017	0	0	0
2015362482	400	rural/clinic	No	03/05/2017	0	0	0
2015287365	400	rural/clinic	No	03/05/2017	0	0	0
2015287363	400	rural/clinic	No	03/05/2017	0	0	0
2015287364	400	rural/clinic	No	03/05/2017	0	0	0
2015287362	400	rural/clinic	No	03/05/2017	0	0	0
2015313341	400	rural/clinic	No	04/05/2017	0	0	0
2015313340	400	rural/clinic	No	04/05/2017	0	0	0
2015287361	400	rural/clinic	No	03/05/2017	0	0	0
2015313336	400	rural/clinic	No	04/05/2017	0	0	0
2015287369	400	rural/clinic	No	03/05/2017	0	0	0
2015313335	400	rural/clinic	No	04/05/2017	0	0	0
2015313338	400	rural/clinic	No	04/05/2017	0	0	0
2015313339	400	rural/clinic	No	04/05/2017	0	0	0
2015287368	400	rural/clinic	No	03/05/2017	0	0	0
2015313337	400	rural/clinic	No	04/05/2017	0	0	0
2015287370	637	rural/clinic	No	03/05/2017	0	0	0
2012384793	637	rural/clinic	No	04/05/2017	0	0	0
2015287367	637	rural/clinic	No	03/05/2017	0	0	0
2012384794	637	rural/clinic	No	04/05/2017	0	0	0
2015359756	637	rural/clinic	No	03/05/2017	0	0	0
2015362508	637	rural/clinic	No	04/05/2017	0	0	0
2015362511	637	rural/clinic	No	04/05/2017	0	0	0
2015359759	637	rural/clinic	No	03/05/2017	0	0	0
2015362512	637	rural/clinic	No	04/05/2017	0	0	0
2015359757	637	rural/clinic	No	03/05/2017	0	0	0
2015359758	637	rural/clinic	No	03/05/2017	0	0	0
2015362513	637	rural/clinic	No	04/05/2017	0	0	0
2013249490	637	rural/clinic	No	03/05/2017	0	0	0
2015362514	156	rural/clinic	No	04/05/2017	0	0	0
2014347987	156	rural/clinic	No	04/05/2017	0	0	0
2015359848	156	rural/clinic	No	03/05/2017	0	0	0
2012341044	156	rural/clinic	No	04/05/2017	0	0	0
2012253130	156	rural/clinic	No	03/05/2017	0	0	0
2015331773	156	rural/clinic	No	03/05/2017	0	0	0
2015361603	156	rural/clinic	No	03/05/2017	0	0	0
2014301892	156	rural/clinic	No	04/05/2017	0	0	0
2015361604	156	rural/clinic	No	03/05/2017	0	0	0

2015407551	156	rural/clinic	No	04/05/2017	0	0	0
2015321804	156	rural/clinic	No	04/05/2017	0	0	0
2015321805	156	rural/clinic	No	04/05/2017	0	0	0
2015361602	156	rural/clinic	No	03/05/2017	0	0	0
2015321806	331	rural/clinic	No	04/05/2017	0	0	0
2015339754	331	rural/clinic	No	03/05/2017	0	0	0
2015405097	331	rural/clinic	No	04/05/2017	0	0	0
2015390305	331	rural/clinic	No	03/05/2017	0	0	0
2014298040	331	rural/clinic	No	04/05/2017	0	0	0
2012267000	331	rural/clinic	No	03/05/2017	0	0	0
2014298041	331	rural/clinic	No	04/05/2017	0	0	0
2013247717	331	rural/clinic	No	04/05/2017	0	0	0
2012266999	331	rural/clinic	No	03/05/2017	0	0	0
2013247718	331	rural/clinic	No	04/05/2017	0	0	0
2012266998	331	rural/clinic	No	03/05/2017	0	0	0
2015324257	331	rural/clinic	No	03/05/2017	0	0	0
2015384040	331	rural/clinic	No	04/05/2017	0	0	0
2015384041	331	rural/clinic	No	04/05/2017	0	0	0
2015332909	331	rural/clinic	No	03/05/2017	0	0	0
2015384042	331	rural/clinic	No	04/05/2017	0	0	0
2015377214	331	rural/clinic	No	04/05/2017	0	0	0
2015332017	48	rural/clinic	No	03/05/2017	0	0	0
2015377215	48	rural/clinic	No	04/05/2017	0	0	0
2015332018	48	rural/clinic	No	03/05/2017	0	0	0
2014346950	48	rural/clinic	No	04/05/2017	0	0	0
2015332019	48	rural/clinic	No	03/05/2017	0	0	0
2012317551	48	rural/clinic	No	04/05/2017	0	0	0
2015408172	48	rural/clinic	No	03/05/2017	0	0	0
2012317552	48	rural/clinic	No	04/05/2017	0	0	0
2015412090	48	rural/clinic	No	03/05/2017	0	0	0
2015331896	48	rural/clinic	No	03/05/2017	0	0	0
2015331897	48	rural/clinic	No	03/05/2017	0	0	0
2015415267	48	rural/clinic	No	04/05/2017	0	0	0
2015415268	48	rural/clinic	No	04/05/2017	0	0	0
2015375987	48	rural/clinic	No	03/05/2017	0	0	0
2014325201	48	rural/clinic	No	04/05/2017	0	0	0
2014288133	48	rural/clinic	No	03/05/2017	0	0	0
2014325202	48	rural/clinic	No	04/05/2017	0	0	0
2015375989	48	rural/clinic	No	03/05/2017	0	0	0
2014378480	48	rural/clinic	No	04/05/2017	0	0	0
2015375988	48	rural/clinic	No	03/05/2017	0	0	0
2014378483	48	rural/clinic	No	04/05/2017	0	0	0
2014288008	48	rural/clinic	No	03/05/2017	0	0	0
2014378484	48	rural/clinic	No	04/05/2017	0	0	0
2015344324	48	rural/clinic	No	03/05/2017	0	0	0
2014378485	48	rural/clinic	No	04/05/2017	0	0	0
2011212596	48	rural/clinic	No	03/05/2017	0	0	0
2011223615	48	rural/clinic	No	03/05/2017	0	0	0
2014371828	48	rural/clinic	No	03/05/2017	0	0	0
2012265281	48	rural/clinic	No	03/05/2017	0	0	0
2015303823	48	rural/clinic	No	03/05/2017	0	0	0
2011223612	48	rural/clinic	No	03/05/2017	0	0	0
2015334206	48	rural/clinic	No	03/05/2017	0	0	0
2015331650	48	rural/clinic	No	27/04/2017	0	0	0
2015331650	48	rural/clinic	No	27/04/2017	0	0	0
2015286707	48	rural/clinic	No	27/04/2017	0	0	0
2015332910	48	rural/clinic	No	27/04/2017	0	0	0
2014369844	48	rural/clinic	No	27/04/2017	0	0	0
2015297258	48	rural/clinic	No	27/04/2017	0	0	0
2015297258	48	rural/clinic	No	27/04/2017	0	0	0
2015344876	48	rural/clinic	No	27/04/2017	0	0	0
2015344876	48	rural/clinic	No	27/04/2017	0	0	0
2015352707	48	rural/clinic	No	27/04/2017	0	0	0
2015352707	48	rural/clinic	No	27/04/2017	0	0	0
2011191291	48	rural/clinic	No	26/04/2017	0	0	0
2014378556	48	rural/clinic	No	27/04/2017	0	0	0
2014378556	48	rural/clinic	No	27/04/2017	0	0	0
2014331410	48	rural/clinic	No	27/04/2017	0	0	0
2014331410	48	rural/clinic	No	27/04/2017	0	0	0
2015334731	48	rural/clinic	No	02/05/2017	0	0	0
2015334731	48	rural/clinic	No	02/05/2017	0	0	0
2015334204	48	rural/clinic	No	03/05/2017	0	0	0
2012261188	48	rural/clinic	No	03/05/2017	0	0	0
2011223614	48	rural/clinic	No	03/05/2017	0	0	0
2011141059	48	rural/clinic	No	03/05/2017	0	0	0
2011141060	48	rural/clinic	No	03/05/2017	0	0	0
2012265283	48	rural/clinic	No	03/05/2017	0	0	0
2012318936	48	rural/clinic	No	03/05/2017	0	0	0
2012265282	48	rural/clinic	No	03/05/2017	0	0	0
2015415263	48	rural/clinic	No	04/05/2017	0	0	0
2015415264	48	rural/clinic	No	04/05/2017	0	0	0
2014371829	48	rural/clinic	No	03/05/2017	0	0	0

2015415265	48	rural/clinic	No	04/05/2017	0	0	0
2014371831	48	rural/clinic	No	03/05/2017	0	0	0
2014371830	48	rural/clinic	No	03/05/2017	0	0	0
2014311368	48	rural/clinic	No	03/05/2017	0	0	0
2014311367	48	rural/clinic	No	03/05/2017	0	0	0
201085807	48	rural/clinic	No	03/05/2017	0	0	0
2015415266	48	rural/clinic	No	04/05/2017	0	0	0
201085805	48	rural/clinic	No	03/05/2017	0	0	0
2014378482	48	rural/clinic	No	04/05/2017	0	0	0
2015334529	48	rural/clinic	No	03/05/2017	0	0	0
2013267893	48	rural/clinic	No	04/05/2017	0	0	0
2015334530	48	rural/clinic	No	03/05/2017	0	0	0
2013267894	48	rural/clinic	No	04/05/2017	0	0	0
2015368582	48	rural/clinic	No	03/05/2017	0	0	0
2012389498	48	rural/clinic	No	04/05/2017	0	0	0
2015368581	48	rural/clinic	No	03/05/2017	0	0	0
2012389499	48	rural/clinic	No	04/05/2017	0	0	0
2015358188	48	rural/clinic	No	04/05/2017	0	0	0
2013255261	48	rural/clinic	No	03/05/2017	0	0	0
2015386400	48	rural/clinic	No	04/05/2017	0	0	0
2013255260	48	rural/clinic	No	03/05/2017	0	0	0
2015361408	48	rural/clinic	No	04/05/2017	0	0	0
2015361409	48	rural/clinic	No	04/05/2017	0	0	0
2015361410	48	rural/clinic	No	04/05/2017	0	0	0
2015361411	48	rural/clinic	No	04/05/2017	0	0	0
2015351434	48	rural/clinic	No	03/05/2017	0	0	0
2015361412	48	rural/clinic	No	04/05/2017	0	0	0
2015351435	48	rural/clinic	No	03/05/2017	0	0	0
2015351436	48	rural/clinic	No	03/05/2017	0	0	0
2015351437	48	rural/clinic	No	03/05/2017	0	0	0
2015351438	48	rural/clinic	No	03/05/2017	0	0	0
2014290239	48	rural/clinic	No	04/05/2017	0	0	0
20112000078	48	rural/clinic	No	04/05/2017	0	0	0
2015351439	48	rural/clinic	No	03/05/2017	0	0	0
2012252691	48	rural/clinic	No	04/05/2017	0	0	0
2015332967	48	rural/clinic	No	04/05/2017	0	0	0
2015357802	48	rural/clinic	No	04/05/2017	0	0	0
2015332965	48	rural/clinic	No	04/05/2017	0	0	0
2011200277	48	rural/clinic	No	04/05/2017	0	0	0
2014372443	48	rural/clinic	No	04/05/2017	0	0	0
2014372273	48	rural/clinic	No	04/05/2017	0	0	0
2015357648	48	rural/clinic	No	04/05/2017	0	0	0
2015351643	48	rural/clinic	No	04/05/2017	0	0	0
2015357649	48	rural/clinic	No	04/05/2017	0	0	0
2015351644	48	rural/clinic	No	04/05/2017	0	0	0
2015351645	48	rural/clinic	No	04/05/2017	0	0	0
2015351646	48	rural/clinic	No	04/05/2017	0	0	0
2015351647	48	rural/clinic	No	04/05/2017	0	0	0
2015331938	48	rural/clinic	No	04/05/2017	0	0	0
2015351648	48	rural/clinic	No	04/05/2017	0	0	0
2015331937	48	rural/clinic	No	04/05/2017	0	0	0
2015351649	48	rural/clinic	No	04/05/2017	0	0	0
2015340975	48	rural/clinic	No	04/05/2017	0	0	0
2015340974	48	rural/clinic	No	04/05/2017	0	0	0
2015328474	48	rural/clinic	No	04/05/2017	0	0	0
2015340973	48	rural/clinic	No	04/05/2017	0	0	0
2015328475	48	rural/clinic	No	04/05/2017	0	0	0
2015328476	48	rural/clinic	No	04/05/2017	0	0	0
2015340964	48	rural/clinic	No	04/05/2017	0	0	0
2015328479	48	rural/clinic	No	04/05/2017	0	0	0
2011133628	48	rural/clinic	No	04/05/2017	0	0	0
2011133631	48	rural/clinic	No	04/05/2017	0	0	0
2015328480	48	rural/clinic	No	04/05/2017	0	0	0
2011133630	48	rural/clinic	No	04/05/2017	0	0	0
2011133632	48	rural/clinic	No	04/05/2017	0	0	0
2015328491	48	rural/clinic	No	04/05/2017	0	0	0
2015328491	48	rural/clinic	No	04/05/2017	0	0	0
2011133633	48	rural/clinic	No	04/05/2017	0	0	0
2015328482	48	rural/clinic	No	04/05/2017	0	0	0
2011133634	48	rural/clinic	No	04/05/2017	0	0	0
2015328483	48	rural/clinic	No	04/05/2017	0	0	0
2011133635	48	rural/clinic	No	04/05/2017	0	0	0
2015328484	48	rural/clinic	No	04/05/2017	0	0	0
2015328485	48	rural/clinic	No	04/05/2017	0	0	0
2015361414	48	rural/clinic	No	04/05/2017	0	0	0
2012259879	48	rural/clinic	No	04/05/2017	0	0	0
2015361415	48	rural/clinic	No	04/05/2017	0	0	0
2015286539	48	rural/clinic	No	04/05/2017	0	0	0
2015286540	48	rural/clinic	No	04/05/2017	0	0	0
2015361416	48	rural/clinic	No	04/05/2017	0	0	0
2015361417	48	rural/clinic	No	04/05/2017	0	0	0
2015361418	48	rural/clinic	No	04/05/2017	0	0	0

2015361419	48	rural/clinic	No	04/05/2017	0	0	0
2015361420	48	rural/clinic	No	04/05/2017	0	0	0
2015361421	48	rural/clinic	No	04/05/2017	0	0	0
2015361422	48	rural/clinic	No	04/05/2017	0	0	0
2015361423	48	rural/clinic	No	04/05/2017	0	0	0
2015361424	48	rural/clinic	No	04/05/2017	0	0	0
2015413135	48	rural/clinic	No	04/05/2017	0	0	0
2015413136	48	rural/clinic	No	04/05/2017	0	0	0
2015335118	48	rural/clinic	No	11/05/2017	0	0	0
2015379315	48	rural/clinic	No	04/05/2017	0	0	0
2015379316	48	rural/clinic	No	04/05/2017	0	0	0
2015413150	48	rural/clinic	No	04/05/2017	0	0	0
2012295984	48	rural/clinic	No	04/05/2017	0	0	0
2015349005	48	rural/clinic	No	04/05/2017	0	0	0
2015331899	48	rural/clinic	No	04/05/2017	0	0	0
2015328057	48	rural/clinic	No	04/05/2017	0	0	0
2015328058	48	rural/clinic	No	04/05/2017	0	0	0
2015328059	48	rural/clinic	No	04/05/2017	0	0	0
2015331898	48	rural/clinic	No	04/05/2017	0	0	0
2015328060	48	rural/clinic	No	04/05/2017	0	0	0
2015289842	48	rural/clinic	No	04/05/2017	0	0	0
2015351636	48	rural/clinic	No	04/05/2017	0	0	0
2014356985	48	rural/clinic	No	04/05/2017	0	0	0
2015351637	48	rural/clinic	No	04/05/2017	0	0	0
2014356982	48	rural/clinic	No	04/05/2017	0	0	0
2014356983	48	rural/clinic	No	04/05/2017	0	0	0
2015351638	48	rural/clinic	No	04/05/2017	0	0	0
2015351639	48	rural/clinic	No	04/05/2017	0	0	0
2015351640	48	rural/clinic	No	04/05/2017	0	0	0
2015351641	48	rural/clinic	No	04/05/2017	0	0	0
2015351642	48	rural/clinic	No	04/05/2017	0	0	0
2014356984/D	48	rural/clinic	No	04/05/2017	0	0	0
2014372271	48	rural/clinic	No	04/05/2017	0	0	0
2015332963	48	rural/clinic	No	04/05/2017	0	0	0
2015418755	48	rural/clinic	No	04/05/2017	0	0	0
2012246283	48	rural/clinic	No	04/05/2017	0	0	0
2012246284	48	rural/clinic	No	04/05/2017	0	0	0
2015332964	48	rural/clinic	No	04/05/2017	0	0	0
2014306989	48	rural/clinic	No	04/05/2017	0	0	0
2014372442	48	rural/clinic	No	04/05/2017	0	0	0
2014362818	48	rural/clinic	No	04/05/2017	0	0	0
2014362819	48	rural/clinic	No	04/05/2017	0	0	0
2014362820	48	rural/clinic	No	04/05/2017	0	0	0
2014362821	503	rural/clinic	No	04/05/2017	0	0	0
2014338555	503	rural/clinic	No	04/05/2017	0	0	0
2015360854	503	rural/clinic	No	04/05/2017	0	0	0
2013273644	503	rural/clinic	No	04/05/2017	0	0	0
2015336704	503	rural/clinic	No	04/05/2017	0	0	0
2014338552	503	rural/clinic	No	04/05/2017	0	0	0
2015286298	157	rural/clinic	No	04/05/2017	0	0	0
2015286297	157	rural/clinic	No	04/05/2017	0	0	0
2015286299	157	rural/clinic	No	04/05/2017	0	0	0
2015310608	157	rural/clinic	No	04/05/2017	0	0	0
2014314468	157	rural/clinic	No	04/05/2017	0	0	0
2015310607	157	rural/clinic	No	04/05/2017	0	0	0
2015289846	157	rural/clinic	No	04/05/2017	0	0	0
2014314469	157	rural/clinic	No	04/05/2017	0	0	0
2015289841	157	rural/clinic	No	04/05/2017	0	0	0
2015362478	157	rural/clinic	No	04/05/2017	0	0	0
2014310207	157	rural/clinic	No	04/05/2017	0	0	0
2014288132	157	rural/clinic	No	04/05/2017	0	0	0
2015375412	157	rural/clinic	No	04/05/2017	0	0	0
2015362479	157	rural/clinic	No	04/05/2017	0	0	0
2014342789	157	rural/clinic	No	04/05/2017	0	0	0
2015362480	157	rural/clinic	No	05/05/2017	0	0	0
2015310611	157	rural/clinic	No	04/05/2017	0	0	0
2014342790	157	rural/clinic	No	04/05/2017	0	0	0
2015362481	157	rural/clinic	No	05/05/2017	0	0	0
2012253968	157	rural/clinic	No	04/05/2017	0	0	0
2015300696	157	rural/clinic	No	04/05/2017	0	0	0
2012350628	157	rural/clinic	No	05/05/2017	0	0	0
2014353638	157	rural/clinic	No	05/05/2017	0	0	0
2012350630	157	rural/clinic	No	04/05/2017	0	0	0
2012350631	157	rural/clinic	No	04/05/2017	0	0	0
2015359203	157	rural/clinic	No	04/05/2017	0	0	0
2015406491	157	rural/clinic	No	05/05/2017	0	0	0
2012284621	157	rural/clinic	No	04/05/2017	0	0	0
2015406492	157	rural/clinic	No	05/05/2017	0	0	0
2015300694	157	rural/clinic	No	04/05/2017	0	0	0
2015406493	662	district/faith-based	No	05/05/2017	1	0	0
2015300695	662	district/faith-based	No	04/05/2017	1	0	0
2015406494	662	district/faith-based	No	05/05/2017	1	0	0

2014291832	662	district/faith-based	No	04/05/2017	1	0	0
2015406496	662	district/faith-based	No	05/05/2017	1	0	0
2014291881	662	district/faith-based	No	04/05/2017	1	0	0
2015326542	662	district/faith-based	No	04/05/2017	1	0	0
2015406497	662	district/faith-based	No	05/05/2017	1	0	0
2015406498	662	district/faith-based	No	05/05/2017	1	0	0
2015326366	662	district/faith-based	No	05/05/2017	1	0	0
2015326367	662	district/faith-based	No	05/05/2017	1	0	0
2015326368	662	district/faith-based	No	05/05/2017	1	0	0
2015326369	662	district/faith-based	No	05/05/2017	1	0	0
2015326370	662	district/faith-based	No	05/05/2017	1	0	0
2014338554	662	district/faith-based	No	04/05/2017	1	0	0
2015286300	662	district/faith-based	No	04/05/2017	1	0	0
2011142218	662	district/faith-based	No	04/05/2017	1	0	0
2013273645	662	district/faith-based	No	04/05/2017	1	0	0
2015326576	662	district/faith-based	No	04/05/2017	1	0	0
2015326575	662	district/faith-based	No	04/05/2017	1	0	0
2015405410	662	district/faith-based	No	25/04/2017	1	0	0
2015293835	662	district/faith-based	No	02/05/2017	1	0	0
2015293835	662	district/faith-based	No	02/05/2017	1	0	0
2015334620	662	district/faith-based	No	02/05/2017	1	0	0
2015334620	662	district/faith-based	No	02/05/2017	1	0	0
2014289416	662	district/faith-based	No	02/05/2017	1	0	0
2014289416	662	district/faith-based	No	02/05/2017	1	0	0
2015417051	662	district/faith-based	No	02/05/2017	1	0	0
2015418856	662	district/faith-based	No	02/05/2017	1	0	0
2015379557	662	district/faith-based	No	02/05/2017	1	0	0
2015326751	662	district/faith-based	No	03/05/2017	1	0	0
2015326751	662	district/faith-based	No	03/05/2017	1	0	0
2015326753	662	district/faith-based	No	03/05/2017	1	0	0
2015326753	662	district/faith-based	No	03/05/2017	1	0	0
2015339550	662	district/faith-based	No	03/05/2017	1	0	0
2015339550	662	district/faith-based	No	03/05/2017	1	0	0
2014363882	662	district/faith-based	No	03/05/2017	1	0	0
2014374014	662	district/faith-based	No	03/05/2017	1	0	0
2014374014	662	district/faith-based	No	03/05/2017	1	0	0
2012295982	662	district/faith-based	No	03/05/2017	1	0	0
2012295982	662	district/faith-based	No	03/05/2017	1	0	0
2012295983	662	district/faith-based	No	03/05/2017	1	0	0
2012295983	662	district/faith-based	No	03/05/2017	1	0	0
2012253131	662	district/faith-based	No	04/05/2017	1	0	0
2012253131	662	district/faith-based	No	04/05/2017	1	0	0
2015287365	662	district/faith-based	No	03/05/2017	1	0	0
2015287365	662	district/faith-based	No	03/05/2017	1	0	0
2015287363	662	district/faith-based	No	03/05/2017	1	0	0
2015287363	662	district/faith-based	No	03/05/2017	1	0	0
2015314080	662	district/faith-based	No	03/05/2017	1	0	0
2015314080	662	district/faith-based	No	03/05/2017	1	0	0
2013266118	662	district/faith-based	No	04/05/2017	1	0	0
2012325691	662	district/faith-based	No	04/05/2017	1	0	0
2015358191	662	district/faith-based	No	04/05/2017	1	0	0
2015358190	662	district/faith-based	No	04/05/2017	1	0	0
2015358189	662	district/faith-based	No	04/05/2017	1	0	0
2015352712	662	district/faith-based	No	04/05/2017	1	0	0
2015352714	662	district/faith-based	No	04/05/2017	1	0	0
2015352715	662	district/faith-based	No	04/05/2017	1	0	0
2014383971	662	district/faith-based	No	04/05/2017	1	0	0
2015352710	662	district/faith-based	No	04/05/2017	1	0	0
2015352711	662	district/faith-based	No	04/05/2017	1	0	0
2015332021	662	district/faith-based	No	04/05/2017	1	0	0
2015332022	662	district/faith-based	No	04/05/2017	1	0	0
2015332023	662	district/faith-based	No	04/05/2017	1	0	0
2015324124	662	district/faith-based	No	04/05/2017	1	0	0
2015324125	662	district/faith-based	No	04/05/2017	1	0	0
2015324126	662	district/faith-based	No	04/05/2017	1	0	0
2014291942	662	district/faith-based	No	04/05/2017	1	0	0
2015362809	662	district/faith-based	No	04/05/2017	1	0	0
2015362810	925	rural/clinic	No	04/05/2017	0	0	0
2015362811	925	rural/clinic	No	04/05/2017	0	0	0
2015369174	925	rural/clinic	No	04/05/2017	0	0	0
2015369173	925	rural/clinic	No	04/05/2017	0	0	0
2015402612	925	rural/clinic	No	04/05/2017	0	0	0
2015402613	925	rural/clinic	No	04/05/2017	0	0	0
2015402614	925	rural/clinic	No	04/05/2017	0	0	0
2015402615	504	rural/clinic	No	04/05/2017	0	0	0
2015402616	504	rural/clinic	No	04/05/2017	0	0	0
2015402617	504	rural/clinic	No	04/05/2017	0	0	0
2015402618	504	rural/clinic	No	04/05/2017	0	0	0
2015402391	504	rural/clinic	No	04/05/2017	0	0	0
2015402390	504	rural/clinic	No	04/05/2017	0	0	0
2013255510	504	rural/clinic	No	04/05/2017	0	0	0
2014303083	504	rural/clinic	No	04/05/2017	0	0	0

2013255511	504	rural/clinic	No	04/05/2017	0	0	0
2012284744	504	rural/clinic	No	04/05/2017	0	0	0
2012305700	504	rural/clinic	No	04/05/2017	0	0	0
2012284227	504	rural/clinic	No	04/05/2017	0	0	0
2014334349	504	rural/clinic	No	04/05/2017	0	0	0
2014287978	504	rural/clinic	No	04/05/2017	0	0	0
2014347738	504	rural/clinic	No	04/05/2017	0	0	0
2014303500	504	rural/clinic	No	04/05/2017	0	0	0
2012253926	504	rural/clinic	No	04/05/2017	0	0	0
2012253927	504	rural/clinic	No	04/05/2017	0	0	0
2012251546	504	rural/clinic	No	11/05/2017	0	0	0
2015335119	504	rural/clinic	No	11/05/2017	0	0	0
2015314551	504	rural/clinic	No	09/05/2017	0	0	0
2015337504	504	rural/clinic	No	09/05/2017	0	0	0
2012374289	504	rural/clinic	No	09/05/2017	0	0	0
2012374291	504	rural/clinic	No	09/05/2017	0	0	0
2014311549	504	rural/clinic	No	09/05/2017	0	0	0
2014311550	504	rural/clinic	No	09/05/2017	0	0	0
2012374290	504	rural/clinic	No	09/05/2017	0	0	0
2014319430	504	rural/clinic	No	09/05/2017	0	0	0
2015287311	504	rural/clinic	No	09/05/2017	0	0	0
2015324439	504	rural/clinic	No	09/05/2017	0	0	0
2013264066	504	rural/clinic	No	09/05/2017	0	0	0
2015368703	504	rural/clinic	No	09/05/2017	0	0	0
2015368704	504	rural/clinic	No	09/05/2017	0	0	0
2015384484	504	rural/clinic	No	09/05/2017	0	0	0
2015368705	504	rural/clinic	No	09/05/2017	0	0	0
2015376654	504	rural/clinic	No	09/05/2017	0	0	0
2015413999	504	rural/clinic	No	09/05/2017	0	0	0
2014333798	504	rural/clinic	No	09/05/2017	0	0	0
2015414000	504	rural/clinic	No	09/05/2017	0	0	0
2014344365	504	rural/clinic	No	09/05/2017	0	0	0
2015368702	504	rural/clinic	No	09/05/2017	0	0	0
2012314019	504	rural/clinic	No	09/05/2017	0	0	0
2015326372	504	rural/clinic	No	09/05/2017	0	0	0
2012314020	504	rural/clinic	No	09/05/2017	0	0	0
2014319130	504	rural/clinic	No	09/05/2017	0	0	0
2015326373	504	rural/clinic	No	09/05/2017	0	0	0
2015372804	504	rural/clinic	No	09/05/2017	0	0	0
2013254144	504	rural/clinic	No	09/05/2017	0	0	0
2014334175	504	rural/clinic	No	09/05/2017	0	0	0
2015286639	504	rural/clinic	No	09/05/2017	0	0	0
2014291833	504	rural/clinic	No	09/05/2017	0	0	0
2014291884	504	rural/clinic	No	09/05/2017	0	0	0
2014352860	504	rural/clinic	No	09/05/2017	0	0	0
2015315905	504	rural/clinic	No	09/05/2017	0	0	0
2015324036	504	rural/clinic	No	09/05/2017	0	0	0
2014352861	504	rural/clinic	No	09/05/2017	0	0	0
2012374287	478	rural/clinic	No	09/05/2017	0	0	0
2014352862	478	rural/clinic	No	09/05/2017	0	0	0
2015355515	478	rural/clinic	No	09/05/2017	0	0	0
2015338464	478	rural/clinic	No	09/05/2017	0	0	0
2014324372	478	rural/clinic	No	09/05/2017	0	0	0
2015368661	478	rural/clinic	No	09/05/2017	0	0	0
2014342523	478	rural/clinic	No	09/05/2017	0	0	0
2014324373	478	rural/clinic	No	09/05/2017	0	0	0
2015368660	478	rural/clinic	No	09/05/2017	0	0	0
2014342524	478	rural/clinic	No	09/05/2017	0	0	0
2015368659	478	rural/clinic	No	09/05/2017	0	0	0
2014324374	478	rural/clinic	No	09/05/2017	0	0	0
2012291351	478	rural/clinic	No	09/05/2017	0	0	0
2012265922	478	rural/clinic	No	09/05/2017	0	0	0
2015356238	478	rural/clinic	No	09/05/2017	0	0	0
2015326757	478	rural/clinic	No	09/05/2017	0	0	0
2014346722	478	rural/clinic	No	09/05/2017	0	0	0
2015326758	478	rural/clinic	No	09/05/2017	0	0	0
2014346723	478	rural/clinic	No	09/05/2017	0	0	0
2015326759	478	rural/clinic	No	09/05/2017	0	0	0
2015356239	478	rural/clinic	No	09/05/2017	0	0	0
2015326760	478	rural/clinic	No	09/05/2017	0	0	0
2015356240	478	rural/clinic	No	09/05/2017	0	0	0
2015326761	478	rural/clinic	No	09/05/2017	0	0	0
2015326762	478	rural/clinic	No	09/05/2017	0	0	0
2015326371	478	rural/clinic	No	09/05/2017	0	0	0
2015326763	478	rural/clinic	No	09/05/2017	0	0	0
2015397611	478	rural/clinic	No	09/05/2017	0	0	0
2015326764	478	rural/clinic	No	09/05/2017	0	0	0
2015397612	478	rural/clinic	No	09/05/2017	0	0	0
2015326765	478	rural/clinic	No	09/05/2017	0	0	0
2015397613	478	rural/clinic	No	09/05/2017	0	0	0
2011135662	478	rural/clinic	No	09/05/2017	0	0	0
2011135661	478	rural/clinic	No	09/05/2017	0	0	0

2014327675	478	rural/clinic	No	09/05/2017	0	0	0
2014327676	456	rural/clinic	No	09/05/2017	0	0	0
2015397614	456	rural/clinic	No	09/05/2017	0	0	0
2014327677	456	rural/clinic	No	09/05/2017	0	0	0
2011196013	456	rural/clinic	No	09/05/2017	0	0	0
2014346724	456	rural/clinic	No	09/05/2017	0	0	0
2015344086	456	rural/clinic	No	09/05/2017	0	0	0
2015397615	456	rural/clinic	No	09/05/2017	0	0	0
2015344087	456	rural/clinic	No	09/05/2017	0	0	0
2014346725	456	rural/clinic	No	09/05/2017	0	0	0
2015397616	456	rural/clinic	No	09/05/2017	0	0	0
2014346726	456	rural/clinic	No	09/05/2017	0	0	0
2015344082	456	rural/clinic	No	09/05/2017	0	0	0
2015397617	456	rural/clinic	No	09/05/2017	0	0	0
2014346727	456	rural/clinic	No	09/05/2017	0	0	0
2015288747	456	rural/clinic	No	09/05/2017	0	0	0
2015344083	456	rural/clinic	No	09/05/2017	0	0	0
2015397922	456	rural/clinic	No	09/05/2017	0	0	0
2015344084	456	rural/clinic	No	09/05/2017	0	0	0
2015397923	456	rural/clinic	No	09/05/2017	0	0	0
2015344085	456	rural/clinic	No	09/05/2017	0	0	0
2015397924	456	rural/clinic	No	09/05/2017	0	0	0
2011196014	456	rural/clinic	No	09/05/2017	0	0	0
2015297468	456	rural/clinic	No	09/05/2017	0	0	0
2011135664	456	rural/clinic	No	09/05/2017	0	0	0
2015297469	456	rural/clinic	No	09/05/2017	0	0	0
2015334628	456	rural/clinic	No	09/05/2017	0	0	0
2015297471	456	rural/clinic	No	09/05/2017	0	0	0
2012384797	456	rural/clinic	No	09/05/2017	0	0	0
2015413907	456	rural/clinic	No	09/05/2017	0	0	0
2015297472	456	rural/clinic	No	09/05/2017	0	0	0
2012384796	456	rural/clinic	No	09/05/2017	0	0	0
2015324570	456	rural/clinic	No	09/05/2017	0	0	0
2014328439	456	rural/clinic	No	09/05/2017	0	0	0
2015413594	456	rural/clinic	No	09/05/2017	0	0	0
2012384795	456	rural/clinic	No	09/05/2017	0	0	0
2014382917	456	rural/clinic	No	09/05/2017	0	0	0
2011139401	456	rural/clinic	No	09/05/2017	0	0	0
2013264063	456	rural/clinic	No	09/05/2017	0	0	0
2015344880	456	rural/clinic	No	09/05/2017	0	0	0
2013264064	456	rural/clinic	No	09/05/2017	0	0	0
2011192839	456	rural/clinic	No	09/05/2017	0	0	0
2015344882	456	rural/clinic	No	09/05/2017	0	0	0
2011192840	456	rural/clinic	No	09/05/2017	0	0	0
2013264065	456	rural/clinic	No	09/05/2017	0	0	0
2015344883	456	rural/clinic	No	09/05/2017	0	0	0
2011192841	456	rural/clinic	No	09/05/2017	0	0	0
2012295143	456	rural/clinic	No	09/05/2017	0	0	0
2011192842	456	rural/clinic	No	09/05/2017	0	0	0
2015344885	456	rural/clinic	No	09/05/2017	0	0	0
2012316546	456	rural/clinic	No	09/05/2017	0	0	0
2011192843	456	rural/clinic	No	09/05/2017	0	0	0
2015344886	456	rural/clinic	No	09/05/2017	0	0	0
2012316547	456	rural/clinic	No	09/05/2017	0	0	0
2015412454	456	rural/clinic	No	09/05/2017	0	0	0
2015344888	456	rural/clinic	No	09/05/2017	0	0	0
2012316548	456	rural/clinic	No	09/05/2017	0	0	0
2014301675	456	rural/clinic	No	09/05/2017	0	0	0
2015344884	456	rural/clinic	No	09/05/2017	0	0	0
2012376593	356	rural/clinic	No	09/05/2017	0	0	0
2012316549	356	rural/clinic	No	09/05/2017	0	0	0
2015344881	356	rural/clinic	No	09/05/2017	0	0	0
2012376594	356	rural/clinic	No	09/05/2017	0	0	0
2015368658	356	rural/clinic	No	09/05/2017	0	0	0
2012317282	356	rural/clinic	No	09/05/2017	0	0	0
2014328437	356	rural/clinic	No	09/05/2017	0	0	0
2012295144	356	rural/clinic	No	09/05/2017	0	0	0
2015368655	356	rural/clinic	No	09/05/2017	0	0	0
2014328438	356	rural/clinic	No	09/05/2017	0	0	0
2015335961	356	rural/clinic	No	09/05/2017	0	0	0
2015368657	356	rural/clinic	No	09/05/2017	0	0	0
2014335996	356	rural/clinic	No	09/05/2017	0	0	0
2015413898	356	rural/clinic	No	09/05/2017	0	0	0
2015337974	356	rural/clinic	No	09/05/2017	0	0	0
2015383718	356	rural/clinic	No	09/05/2017	0	0	0
2015294621	356	rural/clinic	No	09/05/2017	0	0	0
2015337975	356	rural/clinic	No	09/05/2017	0	0	0
2015294622	356	rural/clinic	No	09/05/2017	0	0	0
2014314470	356	rural/clinic	No	09/05/2017	0	0	0
2011195782	356	rural/clinic	No	09/05/2017	0	0	0
2014314471	356	rural/clinic	No	09/05/2017	0	0	0
2015383719	356	rural/clinic	No	09/05/2017	0	0	0

2015344887	302	rural/clinic	No	09/05/2017	0	0	0
2015362484	302	rural/clinic	No	09/05/2017	0	0	0
2014382298	302	rural/clinic	No	09/05/2017	0	0	0
2015334925	302	rural/clinic	No	09/05/2017	0	0	0
2014382299	302	rural/clinic	No	09/05/2017	0	0	0
2015334926	302	rural/clinic	No	09/05/2017	0	0	0
2015355740	302	rural/clinic	No	09/05/2017	0	0	0
2015333101	302	rural/clinic	No	09/05/2017	0	0	0
2015334927	302	rural/clinic	No	09/05/2017	0	0	0
2014382300	302	rural/clinic	No	09/05/2017	0	0	0
2015334931	302	rural/clinic	No	09/05/2017	0	0	0
2015333102	302	rural/clinic	No	09/05/2017	0	0	0
2015334932	302	rural/clinic	No	09/05/2017	0	0	0
2015355741	302	rural/clinic	No	09/05/2017	0	0	0
2015297470	302	rural/clinic	No	09/05/2017	0	0	0
2015334929	302	rural/clinic	No	09/05/2017	0	0	0
2014335997	158	district/faith-based	No	09/05/2017	1	0	0
2015334930	158	district/faith-based	No	09/05/2017	1	0	0
2015335962	158	district/faith-based	No	09/05/2017	1	0	0
2014335998	158	district/faith-based	No	09/05/2017	1	0	0
2014312203	158	district/faith-based	No	09/05/2017	1	0	0
2015355778	158	district/faith-based	No	09/05/2017	1	0	0
2014335999	158	district/faith-based	No	09/05/2017	1	0	0
2015342311	158	district/faith-based	No	09/05/2017	1	0	0
2015355779	158	district/faith-based	No	09/05/2017	1	0	0
2012269015	158	district/faith-based	No	09/05/2017	1	0	0
2013255741	158	district/faith-based	No	09/05/2017	1	0	0
2015384810	158	district/faith-based	No	09/05/2017	1	0	0
2015377886	158	district/faith-based	No	09/05/2017	1	0	0
2015362483	158	district/faith-based	No	09/05/2017	1	0	0
2011157145	158	district/faith-based	No	09/05/2017	1	0	0
2011234896	158	district/faith-based	No	09/05/2017	1	0	0
2015337973	158	district/faith-based	No	09/05/2017	1	0	0
2015384358	158	district/faith-based	No	09/05/2017	1	0	0
2011234897	158	district/faith-based	No	09/05/2017	1	0	0
2014358189	158	district/faith-based	No	09/05/2017	1	0	0
2015384359	158	district/faith-based	No	09/05/2017	1	0	0
2014368847	158	district/faith-based	No	09/05/2017	1	0	0
2015362251	158	district/faith-based	No	09/05/2017	1	0	0
2014368848	158	district/faith-based	No	09/05/2017	1	0	0
2015413213	158	district/faith-based	No	09/05/2017	1	0	0
2015364394	158	district/faith-based	No	09/05/2017	1	0	0
2014349917	158	district/faith-based	No	09/05/2017	1	0	0
2015333851	158	district/faith-based	No	09/05/2017	1	0	0
2015364395	158	district/faith-based	No	09/05/2017	1	0	0
2012314571	158	district/faith-based	No	09/05/2017	1	0	0
2014327245	158	district/faith-based	No	09/05/2017	1	0	0
2015364396	158	district/faith-based	No	09/05/2017	1	0	0
2012314570	158	district/faith-based	No	09/05/2017	1	0	0
2013271843	158	district/faith-based	No	09/05/2017	1	0	0
2015364397	158	district/faith-based	No	09/05/2017	1	0	0
2015367207	158	district/faith-based	No	09/05/2017	1	0	0
2012368544	158	district/faith-based	No	09/05/2017	1	0	0
2015364398	158	district/faith-based	No	09/05/2017	1	0	0
2015367206	158	district/faith-based	No	09/05/2017	1	0	0
2014327125	158	district/faith-based	No	09/05/2017	1	0	0
2015364399	158	district/faith-based	No	09/05/2017	1	0	0
2015405669	158	district/faith-based	No	09/05/2017	1	0	0
2015302420	158	district/faith-based	No	09/05/2017	1	0	0
2015302419	158	district/faith-based	No	09/05/2017	1	0	0
2015405668	158	district/faith-based	No	09/05/2017	1	0	0
2015364400	158	district/faith-based	No	09/05/2017	1	0	0
2014327246	158	district/faith-based	No	09/05/2017	1	0	0
2015363710	158	district/faith-based	No	09/05/2017	1	0	0
2012306142	158	district/faith-based	No	09/05/2017	1	0	0
2011133910	158	district/faith-based	No	09/05/2017	1	0	0
2015303075	158	district/faith-based	No	09/05/2017	1	0	0
2015340088	158	district/faith-based	No	09/05/2017	1	0	0
2014306014	158	district/faith-based	No	09/05/2017	1	0	0
2015303074	158	district/faith-based	No	09/05/2017	1	0	0
2015340087	479	district/faith-based	No	09/05/2017	1	0	0
2015368962	479	district/faith-based	No	09/05/2017	1	0	0
2015302418	479	district/faith-based	No	09/05/2017	1	0	0
2012293700	479	district/faith-based	No	09/05/2017	1	0	0
2015340086	479	district/faith-based	No	09/05/2017	1	0	0
2015367205	479	district/faith-based	No	09/05/2017	1	0	0
2014350098	479	district/faith-based	No	09/05/2017	1	0	0
2015405620	479	district/faith-based	No	09/05/2017	1	0	0
2013271842	479	district/faith-based	No	09/05/2017	1	0	0
2015368963	479	district/faith-based	No	09/05/2017	1	0	0
2015405622	479	district/faith-based	No	09/05/2017	1	0	0
2015340085	479	district/faith-based	No	09/05/2017	1	0	0

2015340084	479	district/faith-based	No	09/05/2017	1	0	0
2014336826	479	district/faith-based	No	09/05/2017	1	0	0
2014303084	479	district/faith-based	No	09/05/2017	1	0	0
2014336825	479	district/faith-based	No	09/05/2017	1	0	0
2014303085	479	district/faith-based	No	09/05/2017	1	0	0
2015405619	479	district/faith-based	No	09/05/2017	1	0	0
2015405621	479	district/faith-based	No	09/05/2017	1	0	0
2012259882	479	district/faith-based	No	09/05/2017	1	0	0
2014303086	479	district/faith-based	No	09/05/2017	1	0	0
2014347817	479	district/faith-based	No	09/05/2017	1	0	0
2013255262	479	district/faith-based	No	09/05/2017	1	0	0
2014368682	479	district/faith-based	No	09/05/2017	1	0	0
2015360727	479	district/faith-based	No	09/05/2017	1	0	0
2014368679	479	district/faith-based	No	09/05/2017	1	0	0
2015360728	479	district/faith-based	No	09/05/2017	1	0	0
2014368680	479	district/faith-based	No	09/05/2017	1	0	0
2011143198	479	district/faith-based	No	09/05/2017	1	0	0
2012242583	479	district/faith-based	No	09/05/2017	1	0	0
2014368681	479	district/faith-based	No	09/05/2017	1	0	0
2014338312	479	district/faith-based	No	09/05/2017	1	0	0
2014357818	479	district/faith-based	No	09/05/2017	1	0	0
2014338313	479	district/faith-based	No	09/05/2017	1	0	0
2015368888	479	district/faith-based	No	09/05/2017	1	0	0
2014360965	479	district/faith-based	No	09/05/2017	1	0	0
2012369027	479	district/faith-based	No	09/05/2017	1	0	0
2014344364	479	district/faith-based	No	09/05/2017	1	0	0
2015368889	479	district/faith-based	No	09/05/2017	1	0	0
2014357819	479	district/faith-based	No	09/05/2017	1	0	0
2012242584	479	district/faith-based	No	09/05/2017	1	0	0
2014350367	479	district/faith-based	No	09/05/2017	1	0	0
2015368583	479	district/faith-based	No	09/05/2017	1	0	0
2014363678	479	district/faith-based	No	09/05/2017	1	0	0
2014350366	479	district/faith-based	No	09/05/2017	1	0	0
2014323739	479	district/faith-based	No	09/05/2017	1	0	0
2015412774	479	district/faith-based	No	09/05/2017	1	0	0
2014291885	479	district/faith-based	No	09/05/2017	1	0	0
2014369847	401	rural/clinic	No	09/05/2017	0	0	0
2015356660	401	rural/clinic	No	09/05/2017	0	0	0
2014291886	401	rural/clinic	No	10/05/2017	0	0	0
2014349190	401	rural/clinic	No	09/05/2017	0	0	0
2015376211	401	rural/clinic	No	09/05/2017	0	0	0
20171REJ	401	rural/clinic	No	27/04/2017	0	0	0
2015376209	401	rural/clinic	No	09/05/2017	0	0	0
2015376210	401	rural/clinic	No	09/05/2017	0	0	0
20172REJ	401	rural/clinic	No	25/04/2017	0	0	0
2012362561	401	rural/clinic	No	09/05/2017	0	0	0
2012362562	401	rural/clinic	No	09/05/2017	0	0	0
2015412776	401	rural/clinic	No	09/05/2017	0	0	0
2015333917	401	rural/clinic	No	25/04/2017	0	0	0
2015412775	401	rural/clinic	No	09/05/2017	0	0	0
2015382911	457	district/faith-based	No	09/05/2017	1	0	0
2014301465	457	district/faith-based	No	09/05/2017	1	0	0
2015333918	457	district/faith-based	No	25/04/2017	1	0	0
2012363962	457	district/faith-based	No	09/05/2017	1	0	0
2014369846	457	district/faith-based	No	09/05/2017	1	0	0
2014307542	457	district/faith-based	No	09/05/2017	1	0	0
2014369845	457	district/faith-based	No	09/05/2017	1	0	0
2015361605	457	district/faith-based	No	09/05/2017	1	0	0
2014307539	457	district/faith-based	No	09/05/2017	1	0	0
2015376652	457	district/faith-based	No	09/05/2017	1	0	0
2015359849	457	district/faith-based	No	09/05/2017	1	0	0
2015376653	457	district/faith-based	No	09/05/2017	1	0	0
2014369195	457	district/faith-based	No	09/05/2017	1	0	0
2015359850	457	district/faith-based	No	09/05/2017	1	0	0
2014369196	457	district/faith-based	No	09/05/2017	1	0	0
2012305801	457	district/faith-based	No	09/05/2017	1	0	0
2014307415	457	district/faith-based	No	09/05/2017	1	0	0
2013266119	457	district/faith-based	No	09/05/2017	1	0	0
2015418851	457	district/faith-based	No	09/05/2017	1	0	0
2015418852	457	district/faith-based	No	09/05/2017	1	0	0
2015320878	457	district/faith-based	No	09/05/2017	1	0	0
2015418857	457	district/faith-based	No	09/05/2017	1	0	0
2015320879	457	district/faith-based	No	09/05/2017	1	0	0
2014375694	457	district/faith-based	No	09/05/2017	1	0	0
2014363884	457	district/faith-based	No	09/05/2017	1	0	0
2015377832	457	district/faith-based	No	09/05/2017	1	0	0
2014375695	457	district/faith-based	No	09/05/2017	1	0	0
2015414731	457	district/faith-based	No	09/05/2017	1	0	0
2014363885	457	district/faith-based	No	09/05/2017	1	0	0
2015414729	457	district/faith-based	No	09/05/2017	1	0	0
2012289686	457	district/faith-based	No	09/05/2017	1	0	0
2015414732	457	district/faith-based	No	09/05/2017	1	0	0

2015338462	457	district/faith-based	No	09/05/2017	1	0	0
2015338463	457	district/faith-based	No	09/05/2017	1	0	0
2015414730	457	district/faith-based	No	09/05/2017	1	0	0
2014375692	457	district/faith-based	No	09/05/2017	1	0	0
2015368886	457	district/faith-based	No	09/05/2017	1	0	0
2015368887	457	district/faith-based	No	09/05/2017	1	0	0
2015368881	457	district/faith-based	No	09/05/2017	1	0	0
2015368880	457	district/faith-based	No	09/05/2017	1	0	0
2015368884	457	district/faith-based	No	09/05/2017	1	0	0
2015368885	457	district/faith-based	No	09/05/2017	1	0	0
2014375693	457	district/faith-based	No	09/05/2017	1	0	0
2014374016	457	district/faith-based	No	09/05/2017	1	0	0
2014374017	457	district/faith-based	No	09/05/2017	1	0	0
2015362812	457	district/faith-based	No	09/05/2017	1	0	0
2015355396	457	district/faith-based	No	09/05/2017	1	0	0
2012344497	457	district/faith-based	No	09/05/2017	1	0	0
2015355395	457	district/faith-based	No	09/05/2017	1	0	0
2015355001	457	district/faith-based	No	09/05/2017	1	0	0
2015355397	457	district/faith-based	No	09/05/2017	1	0	0
2015355400	457	district/faith-based	No	09/05/2017	1	0	0
2015355398	457	district/faith-based	No	09/05/2017	1	0	0
2015355399	457	district/faith-based	No	09/05/2017	1	0	0
2015384782	457	district/faith-based	No	09/05/2017	1	0	0
2015315903	457	district/faith-based	No	09/05/2017	1	0	0
2014291599	457	district/faith-based	No	09/05/2017	1	0	0
2012374271	457	district/faith-based	No	09/05/2017	1	0	0
2012344852	457	district/faith-based	No	09/05/2017	1	0	0
2015324438	457	district/faith-based	No	09/05/2017	1	0	0
2014291598	457	district/faith-based	No	09/05/2017	1	0	0
2012272347	457	district/faith-based	No	03/05/2017	1	0	0
2012272347	457	district/faith-based	No	03/05/2017	1	0	0
2015313336	457	district/faith-based	No	04/05/2017	1	0	0
2015313336	457	district/faith-based	No	04/05/2017	1	0	0
2015313338	457	district/faith-based	No	04/05/2017	1	0	0
2015313337	457	district/faith-based	No	04/05/2017	1	0	0
2015313337	457	district/faith-based	No	04/05/2017	1	0	0
2012384793	457	district/faith-based	No	04/05/2017	1	0	0
2012384793	457	district/faith-based	No	04/05/2017	1	0	0
2015287368	457	district/faith-based	No	03/05/2017	1	0	0
2015287368	457	district/faith-based	No	03/05/2017	1	0	0
2015332967	457	district/faith-based	No	04/05/2017	1	0	0
2015332967	457	district/faith-based	No	04/05/2017	1	0	0
2015351648	457	district/faith-based	No	04/05/2017	1	0	0
2015351648	457	district/faith-based	No	04/05/2017	1	0	0
2015328480	457	district/faith-based	No	04/05/2017	1	0	0
2015328480	457	district/faith-based	No	04/05/2017	1	0	0
2012344851	457	district/faith-based	No	09/05/2017	1	0	0
2011221923	457	district/faith-based	No	03/05/2017	1	0	0
2011221923	457	district/faith-based	No	03/05/2017	1	0	0
2015331897	457	district/faith-based	No	03/05/2017	1	0	0
2015331897	457	district/faith-based	No	03/05/2017	1	0	0
2015413902	457	district/faith-based	No	03/05/2017	1	0	0
2015413902	457	district/faith-based	No	03/05/2017	1	0	0
2015413906	457	district/faith-based	No	03/05/2017	1	0	0
2015413906	457	district/faith-based	No	03/05/2017	1	0	0
2015342310	457	district/faith-based	No	03/05/2017	1	0	0
2015342310	457	district/faith-based	No	03/05/2017	1	0	0
2014357316	457	district/faith-based	No	03/05/2017	1	0	0
2014357316	457	district/faith-based	No	03/05/2017	1	0	0
2014357318	457	district/faith-based	No	03/05/2017	1	0	0
2014357318	457	district/faith-based	No	03/05/2017	1	0	0
2014291597	457	district/faith-based	No	09/05/2017	1	0	0
2012388387	457	district/faith-based	No	09/05/2017	1	0	0
2015337505	457	district/faith-based	No	09/05/2017	1	0	0
2015337506	457	district/faith-based	No	09/05/2017	1	0	0
2014371162	457	district/faith-based	No	09/05/2017	1	0	0
2012374288	457	district/faith-based	No	09/05/2017	1	0	0
2015315904	457	district/faith-based	No	09/05/2017	1	0	0
2015333103	457	district/faith-based	No	09/05/2017	1	0	0
2014371163	457	district/faith-based	No	09/05/2017	1	0	0
2014291622	457	district/faith-based	No	09/05/2017	1	0	0
2014374435	457	district/faith-based	No	09/05/2017	1	0	0
2015338715	457	district/faith-based	No	09/05/2017	1	0	0
2015337619	457	district/faith-based	No	09/05/2017	1	0	0
2015409009	457	district/faith-based	No	11/05/2017	1	0	0
2015322386	457	district/faith-based	No	11/05/2017	1	0	0
1900407053	457	district/faith-based	No	11/05/2017	1	0	0
2015322390	459	rural/clinic	No	11/05/2017	0	0	0
2014348816	460	rural/clinic	No	11/05/2017	0	0	0
2014348815	460	rural/clinic	No	11/05/2017	0	0	0
2012367300	460	rural/clinic	No	11/05/2017	0	0	0
2015286548	460	rural/clinic	No	11/05/2017	0	0	0

2014294807	460	rural/clinic	No	11/05/2017	0	0	0
2015407054	460	rural/clinic	No	11/05/2017	0	0	0
2014294808	460	rural/clinic	No	11/05/2017	0	0	0
2015407052	460	rural/clinic	No	11/05/2017	0	0	0
2015407055	460	rural/clinic	No	11/05/2017	0	0	0
2015369180	460	rural/clinic	No	11/05/2017	0	0	0
2015286550	460	rural/clinic	No	11/05/2017	0	0	0
2015286645	460	rural/clinic	No	11/05/2017	0	0	0
2015369175	460	rural/clinic	No	11/05/2017	0	0	0
2015369179	460	rural/clinic	No	11/05/2017	0	0	0
2015316970	460	rural/clinic	No	11/05/2017	0	0	0
2015286646	460	rural/clinic	No	11/05/2017	0	0	0
2015286643	460	rural/clinic	No	11/05/2017	0	0	0
2015316968	460	rural/clinic	No	11/05/2017	0	0	0
2015299794	460	rural/clinic	No	11/05/2017	0	0	0
2015286642	460	rural/clinic	No	11/05/2017	0	0	0
2015299796	460	rural/clinic	No	11/05/2017	0	0	0
2015328493	460	rural/clinic	No	11/05/2017	0	0	0
2015299797	460	rural/clinic	No	11/05/2017	0	0	0
2015328478	460	rural/clinic	No	11/05/2017	0	0	0
2015299795	460	rural/clinic	No	11/05/2017	0	0	0
2015328477	460	rural/clinic	No	11/05/2017	0	0	0
2014371572	460	rural/clinic	No	11/05/2017	0	0	0
2012265150	460	rural/clinic	No	11/05/2017	0	0	0
2014371574	460	rural/clinic	No	11/05/2017	0	0	0
2015314601	460	rural/clinic	No	11/05/2017	0	0	0
2014358692	460	rural/clinic	No	11/05/2017	0	0	0
2015409010	460	rural/clinic	No	11/05/2017	0	0	0
2014301892	460	rural/clinic	No	04/05/2017	0	0	0
2012317552	460	rural/clinic	No	04/05/2017	0	0	0
2012317552	460	rural/clinic	No	04/05/2017	0	0	0
2015351439	460	rural/clinic	No	03/05/2017	0	0	0
2015351439	460	rural/clinic	No	03/05/2017	0	0	0
2014378484	461	rural/clinic	No	04/05/2017	0	0	0
2014378484	461	rural/clinic	No	04/05/2017	0	0	0
2012246284	461	rural/clinic	No	04/05/2017	0	0	0
2012246284	461	rural/clinic	No	04/05/2017	0	0	0
2014362821	461	rural/clinic	No	04/05/2017	0	0	0
2014362821	461	rural/clinic	No	04/05/2017	0	0	0
2013255510	461	rural/clinic	No	04/05/2017	0	0	0
2013255510	461	rural/clinic	No	04/05/2017	0	0	0
2015289842	461	rural/clinic	No	04/05/2017	0	0	0
2015289842	461	rural/clinic	No	04/05/2017	0	0	0
2015376206	461	rural/clinic	No	03/05/2017	0	0	0
2012388388	461	rural/clinic	No	11/05/2017	0	0	0
2015377172	461	rural/clinic	No	11/05/2017	0	0	0
2015335202	461	rural/clinic	No	11/05/2017	0	0	0
2011226481	461	rural/clinic	No	11/05/2017	0	0	0
2015335201	461	rural/clinic	No	11/05/2017	0	0	0
2011226483	461	rural/clinic	No	11/05/2017	0	0	0
2011226482	461	rural/clinic	No	11/05/2017	0	0	0
2015335114	461	rural/clinic	No	11/05/2017	0	0	0
2015335116	461	rural/clinic	No	11/05/2017	0	0	0
2015335115	461	rural/clinic	No	11/05/2017	0	0	0
2014339744	461	rural/clinic	No	11/05/2017	0	0	0
20150	461	rural/clinic	No	11/05/2017	0	0	0
2015369178	461	rural/clinic	No	11/05/2017	0	0	0
2015377264	461	rural/clinic	No	11/05/2017	0	0	0
2015377323	461	rural/clinic	No	11/05/2017	0	0	0
2015377262	461	rural/clinic	No	11/05/2017	0	0	0
2015377265	461	rural/clinic	No	11/05/2017	0	0	0
2015377263	461	rural/clinic	No	11/05/2017	0	0	0
2015286641	461	rural/clinic	No	11/05/2017	0	0	0
2015286647	461	rural/clinic	No	11/05/2017	0	0	0
2015407053	461	rural/clinic	No	11/05/2017	0	0	0
2015286648	461	rural/clinic	No	11/05/2017	0	0	0
2015397611	461	rural/clinic	No	09/05/2017	0	0	0
2015397611	461	rural/clinic	No	09/05/2017	0	0	0
2015326542	461	rural/clinic	No	04/05/2017	0	0	0
2015326542	461	rural/clinic	No	04/05/2017	0	0	0
2015406498	461	rural/clinic	No	05/05/2017	0	0	0
2015406498	461	rural/clinic	No	05/05/2017	0	0	0
2015361424	461	rural/clinic	No	04/05/2017	0	0	0
2015361424	461	rural/clinic	No	04/05/2017	0	0	0
2014291832	480	rural/clinic	No	04/05/2017	0	0	0
2014291832	480	rural/clinic	No	04/05/2017	0	0	0
2014291881	480	rural/clinic	No	04/05/2017	0	0	0
2014291881	480	rural/clinic	No	04/05/2017	0	0	0
2012368544	480	rural/clinic	No	09/05/2017	0	0	0
2012368544	480	rural/clinic	No	09/05/2017	0	0	0
2015340087	480	rural/clinic	No	09/05/2017	0	0	0
2014335997	480	rural/clinic	No	09/05/2017	0	0	0

2012269015	480	rural/clinic	No	09/05/2017	0	0	0
2012269015	480	rural/clinic	No	09/05/2017	0	0	0
2015418857	480	rural/clinic	No	09/05/2017	0	0	0
2015418857	480	rural/clinic	No	09/05/2017	0	0	0
2015368886	480	rural/clinic	No	09/05/2017	0	0	0
2015368881	480	rural/clinic	No	09/05/2017	0	0	0
2014342524	480	rural/clinic	No	09/05/2017	0	0	0
2014342524	480	rural/clinic	No	09/05/2017	0	0	0
2014327676	480	rural/clinic	No	09/05/2017	0	0	0
2015344087	480	rural/clinic	No	09/05/2017	0	0	0
2015355778	424	rural/clinic	No	09/05/2017	0	0	0
2015355778	424	rural/clinic	No	09/05/2017	0	0	0
2015355779	424	rural/clinic	No	09/05/2017	0	0	0
2015355779	424	rural/clinic	No	09/05/2017	0	0	0
2015384810	424	rural/clinic	No	09/05/2017	0	0	0
2015384810	424	rural/clinic	No	09/05/2017	0	0	0
2011179933	424	rural/clinic	No	11/05/2017	0	0	0
2012336624	424	rural/clinic	No	11/05/2017	0	0	0
2015335117	424	rural/clinic	No	11/05/2017	0	0	0
2015301475	424	rural/clinic	No	11/05/2017	0	0	0
2015369177	424	rural/clinic	No	11/05/2017	0	0	0
2015357803	424	rural/clinic	No	11/05/2017	0	0	0
2012252692	424	rural/clinic	No	11/05/2017	0	0	0
2015414381	424	rural/clinic	No	11/05/2017	0	0	0
2015340101	424	rural/clinic	No	11/05/2017	0	0	0
2015340102	424	rural/clinic	No	11/05/2017	0	0	0
2015408675	424	rural/clinic	No	11/05/2017	0	0	0
2015408676	424	rural/clinic	No	11/05/2017	0	0	0
2015339018	424	rural/clinic	No	11/05/2017	0	0	0
2015339020	424	rural/clinic	No	11/05/2017	0	0	0
2015339019	424	rural/clinic	No	11/05/2017	0	0	0
2015339021	424	rural/clinic	No	11/05/2017	0	0	0
2015339017	424	rural/clinic	No	11/05/2017	0	0	0
2014340273	424	rural/clinic	No	17/05/2017	0	0	0
2015331668	424	rural/clinic	No	11/05/2017	0	0	0
2015368583	424	rural/clinic	No	09/05/2017	0	0	0
2015368583	424	rural/clinic	No	09/05/2017	0	0	0
2014370433	424	rural/clinic	No	11/05/2017	0	0	0
2015351440	424	rural/clinic	No	11/05/2017	0	0	0
2015351441	424	rural/clinic	No	11/05/2017	0	0	0
2015351442	424	rural/clinic	No	11/05/2017	0	0	0
2015338756	424	rural/clinic	No	11/05/2017	0	0	0
2015373402	424	rural/clinic	No	11/05/2017	0	0	0
2015373403	424	rural/clinic	No	11/05/2017	0	0	0
2015379317	424	rural/clinic	No	11/05/2017	0	0	0
2013247831	424	rural/clinic	No	11/05/2017	0	0	0
2013247832	424	rural/clinic	No	11/05/2017	0	0	0
2012284485	424	rural/clinic	No	11/05/2017	0	0	0
2014370217	424	rural/clinic	No	11/05/2017	0	0	0
2015373404	424	rural/clinic	No	11/05/2017	0	0	0
2015340210	424	rural/clinic	No	11/05/2017	0	0	0
2015339302	424	rural/clinic	No	11/05/2017	0	0	0
2015373405	424	rural/clinic	No	11/05/2017	0	0	0
2015339303	424	rural/clinic	No	11/05/2017	0	0	0
2015373406	424	rural/clinic	No	11/05/2017	0	0	0
2012369969	424	rural/clinic	No	11/05/2017	0	0	0
2015373408	424	rural/clinic	No	11/05/2017	0	0	0
2014300429	424	rural/clinic	No	11/05/2017	0	0	0
2015373410	424	rural/clinic	No	11/05/2017	0	0	0
2014300428	424	rural/clinic	No	11/05/2017	0	0	0
2014296324	424	rural/clinic	No	11/05/2017	0	0	0
2014296325	424	rural/clinic	No	11/05/2017	0	0	0
2015373409	424	rural/clinic	No	11/05/2017	0	0	0
2014297787	424	rural/clinic	No	11/05/2017	0	0	0
2015373411	424	rural/clinic	No	11/05/2017	0	0	0
2015339463	424	rural/clinic	No	11/05/2017	0	0	0
2015297262	424	rural/clinic	No	11/05/2017	0	0	0
2015403277	424	rural/clinic	No	11/05/2017	0	0	0
2015297263	424	rural/clinic	No	11/05/2017	0	0	0
2015403274	424	rural/clinic	No	11/05/2017	0	0	0
2012376778	424	rural/clinic	No	11/05/2017	0	0	0
2015403278	424	rural/clinic	No	11/05/2017	0	0	0
2015331941	424	rural/clinic	No	11/05/2017	0	0	0
2015403276	424	rural/clinic	No	11/05/2017	0	0	0
2015331940	424	rural/clinic	No	11/05/2017	0	0	0
2015331939	424	rural/clinic	No	11/05/2017	0	0	0
2015403275	424	rural/clinic	No	11/05/2017	0	0	0
2015403260	424	rural/clinic	No	11/05/2017	0	0	0
2013255742	424	rural/clinic	No	11/05/2017	0	0	0
2011124087	424	rural/clinic	No	11/05/2017	0	0	0
2015339015	424	rural/clinic	No	11/05/2017	0	0	0
2015377433	424	rural/clinic	No	11/05/2017	0	0	0

2015339014	424	rural/clinic	No	11/05/2017	0	0	0
2015362053	424	rural/clinic	No	11/05/2017	0	0	0
2012369968	424	rural/clinic	No	11/05/2017	0	0	0
2015407552	424	rural/clinic	No	11/05/2017	0	0	0
2011157146	424	rural/clinic	No	11/05/2017	0	0	0
2015414383	424	rural/clinic	No	11/05/2017	0	0	0
2015363303	424	rural/clinic	No	11/05/2017	0	0	0
2015414382	424	rural/clinic	No	11/05/2017	0	0	0
2015407553	424	rural/clinic	No	11/05/2017	0	0	0
2012317553	424	rural/clinic	No	11/05/2017	0	0	0
2014347988	424	rural/clinic	No	11/05/2017	0	0	0
2015300698	424	rural/clinic	No	11/05/2017	0	0	0
2014301924	424	rural/clinic	No	11/05/2017	0	0	0
2015300697	424	rural/clinic	No	11/05/2017	0	0	0
2012341045	424	rural/clinic	No	11/05/2017	0	0	0
2012284574	424	rural/clinic	No	11/05/2017	0	0	0
201053905	424	rural/clinic	No	11/05/2017	0	0	0
201053906	424	rural/clinic	No	11/05/2017	0	0	0
2014317628	424	rural/clinic	No	11/05/2017	0	0	0
2014317626	424	rural/clinic	No	11/05/2017	0	0	0
2014317625	424	rural/clinic	No	11/05/2017	0	0	0
2014317627	424	rural/clinic	No	11/05/2017	0	0	0
2015400053	424	rural/clinic	No	11/05/2017	0	0	0
2012290994	424	rural/clinic	No	11/05/2017	0	0	0
2015400054	424	rural/clinic	No	11/05/2017	0	0	0
2015405004	424	rural/clinic	No	11/05/2017	0	0	0
2015358525	424	rural/clinic	No	11/05/2017	0	0	0
2014297346	424	rural/clinic	No	11/05/2017	0	0	0
2015358526	424	rural/clinic	No	11/05/2017	0	0	0
2015358527	424	rural/clinic	No	11/05/2017	0	0	0
2014301925	424	rural/clinic	No	11/05/2017	0	0	0
2014301923	424	rural/clinic	No	11/05/2017	0	0	0
2014385690	424	rural/clinic	No	11/05/2017	0	0	0
2015286301	424	rural/clinic	No	11/05/2017	0	0	0
2015363302	424	rural/clinic	No	11/05/2017	0	0	0
2015286303	424	rural/clinic	No	11/05/2017	0	0	0
2014350959	424	rural/clinic	No	11/05/2017	0	0	0
2015400055	424	rural/clinic	No	11/05/2017	0	0	0
2014350958	424	rural/clinic	No	11/05/2017	0	0	0
2014319724	424	rural/clinic	No	11/05/2017	0	0	0
2015400056	424	rural/clinic	No	11/05/2017	0	0	0
2015288581	424	rural/clinic	No	11/05/2017	0	0	0
2015286302	424	rural/clinic	No	11/05/2017	0	0	0
2015333256	424	rural/clinic	No	11/05/2017	0	0	0
2015400057	424	rural/clinic	No	11/05/2017	0	0	0
2012359161	424	rural/clinic	No	11/05/2017	0	0	0
2015400058	424	rural/clinic	No	11/05/2017	0	0	0
2012359162	424	rural/clinic	No	11/05/2017	0	0	0
2011193483	424	rural/clinic	No	11/05/2017	0	0	0
2015400059	424	rural/clinic	No	11/05/2017	0	0	0
2015400060	424	rural/clinic	No	11/05/2017	0	0	0
2015340976	424	rural/clinic	No	11/05/2017	0	0	0
2015400061	424	rural/clinic	No	11/05/2017	0	0	0
2015340977	424	rural/clinic	No	11/05/2017	0	0	0
2015400062	424	rural/clinic	No	11/05/2017	0	0	0
2015400063	424	rural/clinic	No	11/05/2017	0	0	0
2015340978	424	rural/clinic	No	11/05/2017	0	0	0
2015400064	424	rural/clinic	No	11/05/2017	0	0	0
2015340979	424	rural/clinic	No	11/05/2017	0	0	0
2015400065	424	rural/clinic	No	11/05/2017	0	0	0
2015340980	424	rural/clinic	No	11/05/2017	0	0	0
2015379318	424	rural/clinic	No	11/05/2017	0	0	0
2012268526	424	rural/clinic	No	11/05/2017	0	0	0
2012268529	424	rural/clinic	No	11/05/2017	0	0	0
2015331669	424	rural/clinic	No	11/05/2017	0	0	0
2014350454	424	rural/clinic	No	04/05/2017	0	0	0
2015333257	424	rural/clinic	No	11/05/2017	0	0	0
2015360514	424	rural/clinic	No	11/05/2017	0	0	0
2012300837	424	rural/clinic	No	11/05/2017	0	0	0
2014315702	424	rural/clinic	No	11/05/2017	0	0	0
2014315703	424	rural/clinic	No	11/05/2017	0	0	0
2011192936	424	rural/clinic	No	11/05/2017	0	0	0
2011193482	424	rural/clinic	No	11/05/2017	0	0	0
2011192935	424	rural/clinic	No	11/05/2017	0	0	0
2015397803	424	rural/clinic	No	11/05/2017	0	0	0
2014335250	424	rural/clinic	No	11/05/2017	0	0	0
2014335249	424	rural/clinic	No	11/05/2017	0	0	0
2011205722	424	rural/clinic	No	11/05/2017	0	0	0
2015333959	424	rural/clinic	No	11/05/2017	0	0	0
2015378731	424	rural/clinic	No	09/05/2017	0	0	0
2014378486	424	rural/clinic	No	11/05/2017	0	0	0
2015331670	424	rural/clinic	No	11/05/2017	0	0	0

2014304804	424	rural/clinic	No	11/05/2017	0	0	0
2015415912	424	rural/clinic	No	11/05/2017	0	0	0
2015408105	424	rural/clinic	No	11/05/2017	0	0	0
2015358536	424	rural/clinic	No	11/05/2017	0	0	0
2014304803	424	rural/clinic	No	11/05/2017	0	0	0
2011235379	424	rural/clinic	No	11/05/2017	0	0	0
2015358535	424	rural/clinic	No	11/05/2017	0	0	0
2015408104	424	rural/clinic	No	11/05/2017	0	0	0
2010099913	424	rural/clinic	No	11/05/2017	0	0	0
2010099914	424	rural/clinic	No	11/05/2017	0	0	0
2015408106	424	rural/clinic	No	11/05/2017	0	0	0
2015415913	424	rural/clinic	No	11/05/2017	0	0	0
2014334176	62	rural/clinic	No	11/05/2017	0	0	0
2014334177	62	rural/clinic	No	11/05/2017	0	0	0
2015301476	62	rural/clinic	No	11/05/2017	0	0	0
2015301474	62	rural/clinic	No	11/05/2017	0	0	0
2011223938	62	rural/clinic	No	11/05/2017	0	0	0
2014368022	62	rural/clinic	No	11/05/2017	0	0	0
2011179934	62	rural/clinic	No	11/05/2017	0	0	0
2012268528	62	rural/clinic	No	11/05/2017	0	0	0
2011179932	62	rural/clinic	No	11/05/2017	0	0	0
2015369181	62	rural/clinic	No	11/05/2017	0	0	0
2012336625	62	rural/clinic	No	11/05/2017	0	0	0
2011192489	62	rural/clinic	No	11/05/2017	0	0	0
2015369176	62	rural/clinic	No	11/05/2017	0	0	0
2015369182	62	rural/clinic	No	11/05/2017	0	0	0
1987000	62	rural/clinic	No	11/05/2017	0	0	0
2015406260	62	rural/clinic	No	11/05/2017	0	0	0
2015406257	62	rural/clinic	No	11/05/2017	0	0	0
2015406258	357	rural/clinic	No	11/05/2017	0	0	0
2011143620	357	rural/clinic	No	11/05/2017	0	0	0
2015406495	357	rural/clinic	No	11/05/2017	0	0	0
201053907	357	rural/clinic	No	11/05/2017	0	0	0
2015384542	357	rural/clinic	No	11/05/2017	0	0	0
2011205723	357	rural/clinic	No	11/05/2017	0	0	0
2015406499	357	rural/clinic	No	11/05/2017	0	0	0
2015384544	357	rural/clinic	No	11/05/2017	0	0	0
2014346900	357	rural/clinic	No	11/05/2017	0	0	0
2012265638	357	rural/clinic	No	11/05/2017	0	0	0
2015377434	357	rural/clinic	No	11/05/2017	0	0	0
2015379505	357	rural/clinic	No	11/05/2017	0	0	0
2015362052	357	rural/clinic	No	11/05/2017	0	0	0
2015406259	357	rural/clinic	No	11/05/2017	0	0	0
2015362051	357	rural/clinic	No	11/05/2017	0	0	0
2015352716	357	rural/clinic	No	11/05/2017	0	0	0
2011226864	357	rural/clinic	No	11/05/2017	0	0	0
2015332102	357	rural/clinic	No	11/05/2017	0	0	0
2015362813	357	rural/clinic	No	11/05/2017	0	0	0
2011179935	357	rural/clinic	No	11/05/2017	0	0	0
2014372335	357	rural/clinic	No	11/05/2017	0	0	0
2014314216	357	rural/clinic	No	11/05/2017	0	0	0
2015418709	357	rural/clinic	No	11/05/2017	0	0	0
2010099912	357	rural/clinic	No	11/05/2017	0	0	0
2015402619	357	rural/clinic	No	11/05/2017	0	0	0
2014368483	357	rural/clinic	No	11/05/2017	0	0	0
2014372274	357	rural/clinic	No	11/05/2017	0	0	0
2015402620	357	rural/clinic	No	11/05/2017	0	0	0
2013258453	357	rural/clinic	No	11/05/2017	0	0	0
2015385525	357	rural/clinic	No	11/05/2017	0	0	0
2015402621	357	rural/clinic	No	11/05/2017	0	0	0
2015402622	357	rural/clinic	No	11/05/2017	0	0	0
2015332101	357	rural/clinic	No	11/05/2017	0	0	0
2015385526	357	rural/clinic	No	11/05/2017	0	0	0
2015402623	357	rural/clinic	No	11/05/2017	0	0	0
2015349601	357	rural/clinic	No	11/05/2017	0	0	0
2014372726	358	rural/clinic	No	11/05/2017	0	0	0
2015372022	358	rural/clinic	No	11/05/2017	0	0	0
2015338291	358	rural/clinic	No	11/05/2017	0	0	0
2015358192	358	rural/clinic	No	11/05/2017	0	0	0
2014372727	358	rural/clinic	No	11/05/2017	0	0	0
2012359939	358	rural/clinic	No	11/05/2017	0	0	0
2015338293	358	rural/clinic	No	11/05/2017	0	0	0
2015338299	359	rural/clinic	No	11/05/2017	0	0	0
2012359940	359	rural/clinic	No	11/05/2017	0	0	0
2011226863	359	rural/clinic	No	11/05/2017	0	0	0
2015338292	359	rural/clinic	No	11/05/2017	0	0	0
2012359941	359	rural/clinic	No	11/05/2017	0	0	0
2015338297	359	rural/clinic	No	11/05/2017	0	0	0
2013258454	359	rural/clinic	No	11/05/2017	0	0	0
2015313350	359	rural/clinic	No	11/05/2017	0	0	0
2015338295	359	rural/clinic	No	11/05/2017	0	0	0
2014372334	359	rural/clinic	No	11/05/2017	0	0	0

2015338294	359	rural/clinic	No	11/05/2017	0	0	0
2015332104	359	rural/clinic	No	11/05/2017	0	0	0
2015332105	359	rural/clinic	No	11/05/2017	0	0	0
2015324241	359	rural/clinic	No	11/05/2017	0	0	0
2014346317	359	rural/clinic	No	11/05/2017	0	0	0
2014346318	359	rural/clinic	No	11/05/2017	0	0	0
2015332103	359	rural/clinic	No	11/05/2017	0	0	0
2014383972	359	rural/clinic	No	11/05/2017	0	0	0
2011188717	359	rural/clinic	No	11/05/2017	0	0	0
2011213934	359	rural/clinic	No	11/05/2017	0	0	0
2013276155	359	rural/clinic	No	11/05/2017	0	0	0
2011188715	359	rural/clinic	No	11/05/2017	0	0	0
2015301626	359	rural/clinic	No	11/05/2017	0	0	0
2011188716	359	rural/clinic	No	11/05/2017	0	0	0
2015330477	359	rural/clinic	No	11/05/2017	0	0	0
2014376895	462	rural/clinic	No	11/05/2017	0	0	0
2015299283	462	rural/clinic	No	11/05/2017	0	0	0
2015338296	462	rural/clinic	No	11/05/2017	0	0	0
2015318987	462	rural/clinic	No	11/05/2017	0	0	0
2015338298	462	rural/clinic	No	11/05/2017	0	0	0
2015318988	462	rural/clinic	No	11/05/2017	0	0	0
2012365987	462	rural/clinic	No	11/05/2017	0	0	0
2011188718	462	rural/clinic	No	11/05/2017	0	0	0
2012365985	462	rural/clinic	No	11/05/2017	0	0	0
2015360513	462	rural/clinic	No	11/05/2017	0	0	0
2011188703	462	rural/clinic	No	11/05/2017	0	0	0
2012365986	462	rural/clinic	No	11/05/2017	0	0	0
2015357656	462	rural/clinic	No	11/05/2017	0	0	0
2014337047	462	rural/clinic	No	11/05/2017	0	0	0
2015340211	462	rural/clinic	No	11/05/2017	0	0	0
2011188477	462	rural/clinic	No	11/05/2017	0	0	0
2014351228	462	rural/clinic	No	15/05/2017	0	0	0
2015299154	462	rural/clinic	No	11/05/2017	0	0	0
2015313345	462	rural/clinic	No	11/05/2017	0	0	0
2014351227	462	rural/clinic	No	11/05/2017	0	0	0
2015313348	462	rural/clinic	No	11/05/2017	0	0	0
2015397621	462	rural/clinic	No	15/05/2017	0	0	0
2013247834	462	rural/clinic	No	11/05/2017	0	0	0
2015313346	462	rural/clinic	No	11/05/2017	0	0	0
2015313347	462	rural/clinic	No	11/05/2017	0	0	0
2015397620	462	rural/clinic	No	15/05/2017	0	0	0
2015313349	462	rural/clinic	No	11/05/2017	0	0	0
2015397619	462	rural/clinic	No	15/05/2017	0	0	0
2014309384	462	rural/clinic	No	11/05/2017	0	0	0
2012259764	462	rural/clinic	No	11/05/2017	0	0	0
2014358334	462	rural/clinic	No	15/05/2017	0	0	0
2012259766	462	rural/clinic	No	11/05/2017	0	0	0
2014306100	462	rural/clinic	No	15/05/2017	0	0	0
2011155229	462	rural/clinic	No	11/05/2017	0	0	0
2015305076	462	rural/clinic	No	11/05/2017	0	0	0
2011133637	462	rural/clinic	No	11/05/2017	0	0	0
2012250092	462	rural/clinic	No	11/05/2017	0	0	0
2014321525	462	rural/clinic	No	15/05/2017	0	0	0
2015364607	462	rural/clinic	No	15/05/2017	0	0	0
2014307330	462	rural/clinic	No	11/05/2017	0	0	0
2014321526	462	rural/clinic	No	15/05/2017	0	0	0
2014306099	462	rural/clinic	No	11/05/2017	0	0	0
2014294153	462	rural/clinic	No	11/05/2017	0	0	0
2012352827	462	rural/clinic	No	15/05/2017	0	0	0
2014305944	462	rural/clinic	No	15/05/2017	0	0	0
2012352828	462	rural/clinic	No	15/05/2017	0	0	0
2011133638	462	rural/clinic	No	11/05/2017	0	0	0
2014321425	462	rural/clinic	No	15/05/2017	0	0	0
2011133639	462	rural/clinic	No	11/05/2017	0	0	0
2014351224	462	rural/clinic	No	15/05/2017	0	0	0
2011133640	462	rural/clinic	No	11/05/2017	0	0	0
2015407058	462	rural/clinic	No	11/05/2017	0	0	0
2015407059	462	rural/clinic	No	11/05/2017	0	0	0
2015407060	462	rural/clinic	No	11/05/2017	0	0	0
2015326577	462	rural/clinic	No	11/05/2017	0	0	0
2013273398	462	rural/clinic	No	11/05/2017	0	0	0
2015368207	462	rural/clinic	No	11/05/2017	0	0	0
2015326579	462	rural/clinic	No	11/05/2017	0	0	0
2013273399	462	rural/clinic	No	11/05/2017	0	0	0
2015326578	462	rural/clinic	No	11/05/2017	0	0	0
2014305939	462	rural/clinic	No	15/05/2017	0	0	0
2015351686	462	rural/clinic	No	11/05/2017	0	0	0
2014358333	462	rural/clinic	No	15/05/2017	0	0	0
2015351687	462	rural/clinic	No	11/05/2017	0	0	0
2015351688	462	rural/clinic	No	11/05/2017	0	0	0
2015351650	462	rural/clinic	No	11/05/2017	0	0	0
2014307328	462	rural/clinic	No	15/05/2017	0	0	0

2015373401	462	rural/clinic	No	11/05/2017	0	0	0
2014321524	462	rural/clinic	No	15/05/2017	0	0	0
2014321523	462	rural/clinic	No	15/05/2017	0	0	0
2014373097	462	rural/clinic	No	15/05/2017	0	0	0
2015333258	462	rural/clinic	No	11/05/2017	0	0	0
2015364605	462	rural/clinic	No	15/05/2017	0	0	0
2014291887	462	rural/clinic	No	16/05/2017	0	0	0
2014358335	462	rural/clinic	No	15/05/2017	0	0	0
2013272691	462	rural/clinic	No	15/05/2017	0	0	0
2014291888	462	rural/clinic	No	16/05/2017	0	0	0
2013272692	579	rural/clinic	No	15/05/2017	0	0	0
2014296651	579	rural/clinic	No	16/05/2017	0	0	0
2014328763	579	rural/clinic	No	15/05/2017	0	0	0
2015331727	579	rural/clinic	No	16/05/2017	0	0	0
2015364515	579	rural/clinic	No	15/05/2017	0	0	0
2015364514	579	rural/clinic	No	15/05/2017	0	0	0
2012335870	579	rural/clinic	No	16/05/2017	0	0	0
2015364513	579	rural/clinic	No	15/05/2017	0	0	0
2012335871	579	rural/clinic	No	16/05/2017	0	0	0
2015364512	579	rural/clinic	No	15/05/2017	0	0	0
2015364511	579	rural/clinic	No	15/05/2017	0	0	0
2012369810	579	rural/clinic	No	15/05/2017	0	0	0
2014351226	96	rural/clinic	No	15/05/2017	0	0	0
2015309002	96	rural/clinic	No	16/05/2017	0	0	0
2012259881	96	rural/clinic	No	16/05/2017	0	0	0
2012259884	96	rural/clinic	No	16/05/2017	0	0	0
2015377887	96	rural/clinic	No	16/05/2017	0	0	0
2015377888	96	rural/clinic	No	16/05/2017	0	0	0
2015297479	96	rural/clinic	No	16/05/2017	0	0	0
2015339755	96	rural/clinic	No	16/05/2017	0	0	0
2015297478	96	rural/clinic	No	16/05/2017	0	0	0
2015376403	96	rural/clinic	No	16/05/2017	0	0	0
2015297477	96	rural/clinic	No	16/05/2017	0	0	0
2012259886/d	96	rural/clinic	No	16/05/2017	0	0	0
2015297476	96	rural/clinic	No	16/05/2017	0	0	0
2014368234	96	rural/clinic	No	16/05/2017	0	0	0
2015297475	96	rural/clinic	No	16/05/2017	0	0	0
2014303090	96	rural/clinic	No	10/05/2017	0	0	0
2014368233	96	rural/clinic	No	16/05/2017	0	0	0
2015297474	96	rural/clinic	No	16/05/2017	0	0	0
2015376402	96	rural/clinic	No	16/05/2017	0	0	0
2014368236	96	rural/clinic	No	16/05/2017	0	0	0
2015297473	96	rural/clinic	No	16/05/2017	0	0	0
2015412302	96	rural/clinic	No	16/05/2017	0	0	0
2015350247	96	rural/clinic	No	16/05/2017	0	0	0
2014303091	505	rural/clinic	No	10/05/2017	0	0	0
2012253928	505	rural/clinic	No	16/05/2017	0	0	0
2015349008	505	rural/clinic	No	16/05/2017	0	0	0
2014303092	505	rural/clinic	No	10/05/2017	0	0	0
2015350246	505	rural/clinic	No	16/05/2017	0	0	0
2015376401	505	rural/clinic	No	16/05/2017	0	0	0
2015375059	505	rural/clinic	No	16/05/2017	0	0	0
2015375990	505	rural/clinic	No	16/05/2017	0	0	0
2015350248	505	rural/clinic	No	16/05/2017	0	0	0
2015375991	505	rural/clinic	No	16/05/2017	0	0	0
2015375992	505	rural/clinic	No	16/05/2017	0	0	0
2011193273	505	rural/clinic	No	16/05/2017	0	0	0
2015375211	505	rural/clinic	No	16/05/2017	0	0	0
2015412091	505	rural/clinic	No	16/05/2017	0	0	0
2015376404	505	rural/clinic	No	16/05/2017	0	0	0
2014324705	505	rural/clinic	No	16/05/2017	0	0	0
2014357322	505	rural/clinic	No	16/05/2017	0	0	0
2015349006	505	rural/clinic	No	16/05/2017	0	0	0
2014357321	505	rural/clinic	No	16/05/2017	0	0	0
2014357320	505	rural/clinic	No	16/05/2017	0	0	0
2014360967	505	rural/clinic	No	16/05/2017	0	0	0
2014303093	505	rural/clinic	No	16/05/2017	0	0	0
2014360966	505	rural/clinic	No	16/05/2017	0	0	0
2012346719	506	rural/clinic	No	16/05/2017	0	0	0
2015419001	506	rural/clinic	No	16/05/2017	0	0	0
2015359204	506	rural/clinic	No	16/05/2017	0	0	0
2015288506	506	rural/clinic	No	16/05/2017	0	0	0
2015349007	506	rural/clinic	No	16/05/2017	0	0	0
2012284745	506	rural/clinic	No	16/05/2017	0	0	0
2015375994	506	rural/clinic	No	16/05/2017	0	0	0
2011128283	506	rural/clinic	No	16/05/2017	0	0	0
2014310208	506	rural/clinic	No	16/05/2017	0	0	0
2015368788	506	rural/clinic	No	16/05/2017	0	0	0
2014382918	303	rural/clinic	No	10/05/2017	0	0	0
2015290027	303	rural/clinic	No	16/05/2017	0	0	0
2014367404	303	rural/clinic	No	16/05/2017	0	0	0
2014382920	303	rural/clinic	No	16/05/2017	0	0	0

2015375058	303	rural/clinic	No	16/05/2017	0	0	0
2014328440	303	rural/clinic	No	16/05/2017	0	0	0
2014367405	303	rural/clinic	No	16/05/2017	0	0	0
2015375993	303	rural/clinic	No	16/05/2017	0	0	0
2015297330	303	rural/clinic	No	16/05/2017	0	0	0
2014288134	765	rural/clinic	No	16/05/2017	0	0	0
2015297332	765	rural/clinic	No	16/05/2017	0	0	0
2014288135	765	rural/clinic	No	16/05/2017	0	0	0
2014342791	765	rural/clinic	No	16/05/2017	0	0	0
2015407052	765	rural/clinic	No	11/05/2017	0	0	0
2015407052	765	rural/clinic	No	11/05/2017	0	0	0
2015374201	765	rural/clinic	No	16/05/2017	0	0	0
2015412092	765	rural/clinic	No	16/05/2017	0	0	0
2015350249	765	rural/clinic	No	16/05/2017	0	0	0
2011193274	765	rural/clinic	No	16/05/2017	0	0	0
2011135665	765	rural/clinic	No	16/05/2017	0	0	0
2015412087	765	rural/clinic	No	16/05/2017	0	0	0
2012306143	765	rural/clinic	No	16/05/2017	0	0	0
2014307545	765	rural/clinic	No	16/05/2017	0	0	0
2012306144	765	rural/clinic	No	16/05/2017	0	0	0
2011135666	765	rural/clinic	No	16/05/2017	0	0	0
2015334933	765	rural/clinic	No	16/05/2017	0	0	0
2012363963	765	rural/clinic	No	16/05/2017	0	0	0
2015287315	765	rural/clinic	No	16/05/2017	0	0	0
2014291879	304	rural/clinic	No	27/04/2017	0	0	0
2014291879	304	rural/clinic	No	27/04/2017	0	0	0
2015305074	304	rural/clinic	No	27/04/2017	0	0	0
2015305074	304	rural/clinic	No	27/04/2017	0	0	0
2013266075	304	rural/clinic	No	16/05/2017	0	0	0
2015287316	304	rural/clinic	No	16/05/2017	0	0	0
2015357647	304	rural/clinic	No	27/04/2017	0	0	0
2015357647	304	rural/clinic	No	27/04/2017	0	0	0
2015340209	304	rural/clinic	No	27/04/2017	0	0	0
2015340209	304	rural/clinic	No	27/04/2017	0	0	0
20133255323	304	rural/clinic	No	27/04/2017	0	0	0
2014338312	304	rural/clinic	No	09/05/2017	0	0	0
2014338312	304	rural/clinic	No	09/05/2017	0	0	0
2014291886	304	rural/clinic	No	10/05/2017	0	0	0
2014291886	304	rural/clinic	No	10/05/2017	0	0	0
2015335115	304	rural/clinic	No	11/05/2017	0	0	0
2014327678	304	rural/clinic	No	10/05/2017	0	0	0
2014335558	304	rural/clinic	No	16/05/2017	0	0	0
2014368231	304	rural/clinic	No	16/05/2017	0	0	0
2012388387	304	rural/clinic	No	09/05/2017	0	0	0
2011136102	304	rural/clinic	No	16/05/2017	0	0	0
2014335559	304	rural/clinic	No	16/05/2017	0	0	0
2015413213	304	rural/clinic	No	09/05/2017	0	0	0
2015357530	304	rural/clinic	No	16/05/2017	0	0	0
2015303074	304	rural/clinic	No	09/05/2017	0	0	0
2015303074	304	rural/clinic	No	09/05/2017	0	0	0
2014368232	304	rural/clinic	No	16/05/2017	0	0	0
2014335560	304	rural/clinic	No	16/05/2017	0	0	0
2014319131	304	rural/clinic	No	16/05/2017	0	0	0
2014368235	304	rural/clinic	No	16/05/2017	0	0	0
2012306145	304	rural/clinic	No	16/05/2017	0	0	0
2015374202	304	rural/clinic	No	16/05/2017	0	0	0
2015368885	304	rural/clinic	No	09/05/2017	0	0	0
2015368885	304	rural/clinic	No	09/05/2017	0	0	0
2015324438	304	rural/clinic	No	09/05/2017	0	0	0
2015331776	304	rural/clinic	No	16/05/2017	0	0	0
2015296067	305	district/faith-based	No	16/05/2017	1	0	0
2015302422	305	district/faith-based	No	16/05/2017	1	0	0
2014335561	305	district/faith-based	No	16/05/2017	1	0	0
2015340090	305	district/faith-based	No	16/05/2017	1	0	0
2015296066	305	district/faith-based	No	16/05/2017	1	0	0
2015302421	305	district/faith-based	No	16/05/2017	1	0	0
2012305803	305	district/faith-based	No	16/05/2017	1	0	0
2013249282	305	district/faith-based	No	16/05/2017	1	0	0
2015344088	305	district/faith-based	No	16/05/2017	1	0	0
2014375696	305	district/faith-based	No	16/05/2017	1	0	0
2014297970	305	district/faith-based	No	10/05/2017	1	0	0
2014297971	305	district/faith-based	No	10/05/2017	1	0	0
2013249281	305	district/faith-based	No	16/05/2017	1	0	0
2015369926	305	district/faith-based	No	10/05/2017	1	0	0
2014327126	305	district/faith-based	No	16/05/2017	1	0	0
2015377833	305	district/faith-based	No	16/05/2017	1	0	0
2015359760	305	district/faith-based	No	16/05/2017	1	0	0
2015377836	305	district/faith-based	No	16/05/2017	1	0	0
2014375698	305	district/faith-based	No	16/05/2017	1	0	0
2015325933	305	district/faith-based	No	16/05/2017	1	0	0
2013254711	305	district/faith-based	No	16/05/2017	1	0	0
2014367623	305	district/faith-based	No	16/05/2017	1	0	0

2015331775	305	district/faith-based	No	16/05/2017	1	0	0
2015357194	305	district/faith-based	No	16/05/2017	1	0	0
2015377834	305	district/faith-based	No	16/05/2017	1	0	0
2013254714	305	district/faith-based	No	16/05/2017	1	0	0
2012284027	360	rural/clinic	No	16/05/2017	0	0	0
2015377835	360	rural/clinic	No	16/05/2017	0	0	0
2015340089	360	rural/clinic	No	16/05/2017	0	0	0
2015391521	360	rural/clinic	No	16/05/2017	0	0	0
2012284026	360	rural/clinic	No	16/05/2017	0	0	0
2015414733	360	rural/clinic	No	16/05/2017	0	0	0
2015357195	360	rural/clinic	No	16/05/2017	0	0	0
2015357531	360	rural/clinic	No	16/05/2017	0	0	0
2015414734	360	rural/clinic	No	16/05/2017	0	0	0
2015391522	360	rural/clinic	No	16/05/2017	0	0	0
2015368890	360	rural/clinic	No	16/05/2017	0	0	0
2015342051	360	rural/clinic	No	16/05/2017	0	0	0
2015297333	245	rural/clinic	No	16/05/2017	0	0	0
2015391900	245	rural/clinic	No	16/05/2017	0	0	0
2015383720	245	rural/clinic	No	16/05/2017	0	0	0
2015397126	245	rural/clinic	No	16/05/2017	0	0	0
2014319132	245	rural/clinic	No	16/05/2017	0	0	0
2013249417	245	rural/clinic	No	16/05/2017	0	0	0
2014358213	245	rural/clinic	No	16/05/2017	0	0	0
2015391878	245	rural/clinic	No	16/05/2017	0	0	0
2014342525	245	rural/clinic	No	16/05/2017	0	0	0
2015391877	245	rural/clinic	No	16/05/2017	0	0	0
2014327509	245	rural/clinic	No	16/05/2017	0	0	0
2012291352	245	rural/clinic	No	16/05/2017	0	0	0
2011134990	245	rural/clinic	No	16/05/2017	0	0	0
2011134992	245	rural/clinic	No	16/05/2017	0	0	0
2015296062	245	rural/clinic	No	16/05/2017	0	0	0
2012305802	245	rural/clinic	No	16/05/2017	0	0	0
2011134993	245	rural/clinic	No	16/05/2017	0	0	0
2011134994	245	rural/clinic	No	16/05/2017	0	0	0
2015296063	245	rural/clinic	No	16/05/2017	0	0	0
2011134995	245	rural/clinic	No	16/05/2017	0	0	0
2011134996	245	rural/clinic	No	16/05/2017	0	0	0
2015296064	245	rural/clinic	No	16/05/2017	0	0	0
2011134997	245	rural/clinic	No	16/05/2017	0	0	0
2011134998	245	rural/clinic	No	16/05/2017	0	0	0
2015296065	245	rural/clinic	No	16/05/2017	0	0	0
2011134999	245	rural/clinic	No	16/05/2017	0	0	0
2014358214	245	rural/clinic	No	16/05/2017	0	0	0
2015296068	245	rural/clinic	No	16/05/2017	0	0	0
2015334934	245	rural/clinic	No	16/05/2017	0	0	0
2011135000	245	rural/clinic	No	16/05/2017	0	0	0
2012291354	245	rural/clinic	No	16/05/2017	0	0	0
2015406267	245	rural/clinic	No	16/05/2017	0	0	0
2011140751	245	rural/clinic	No	16/05/2017	0	0	0
2014319632	245	rural/clinic	No	16/05/2017	0	0	0
2011140754	245	rural/clinic	No	16/05/2017	0	0	0
2015406266	245	rural/clinic	No	16/05/2017	0	0	0
2014319634	245	rural/clinic	No	16/05/2017	0	0	0
2011134987	245	rural/clinic	No	16/05/2017	0	0	0
2015406264	245	rural/clinic	No	16/05/2017	0	0	0
2013244116	245	rural/clinic	No	16/05/2017	0	0	0
2015405005	245	rural/clinic	No	16/05/2017	0	0	0
2015405006	245	rural/clinic	No	16/05/2017	0	0	0
2011134989	663	rural/clinic	No	16/05/2017	0	0	0
2015331726	663	rural/clinic	No	16/05/2017	0	0	0
2011134988	663	rural/clinic	No	16/05/2017	0	0	0
2011134967	663	rural/clinic	No	16/05/2017	0	0	0
2015331725	663	rural/clinic	No	16/05/2017	0	0	0
2015286543	663	rural/clinic	No	16/05/2017	0	0	0
2011140755	663	rural/clinic	No	16/05/2017	0	0	0
2015342312	663	rural/clinic	No	16/05/2017	0	0	0
2014357852	663	rural/clinic	No	16/05/2017	0	0	0
2015390306	663	rural/clinic	No	16/05/2017	0	0	0
2015286542	663	rural/clinic	No	16/05/2017	0	0	0
2015390307	663	rural/clinic	No	16/05/2017	0	0	0
2012244285	663	rural/clinic	No	16/05/2017	0	0	0
2015286545	663	rural/clinic	No	16/05/2017	0	0	0
2015390308	663	rural/clinic	No	16/05/2017	0	0	0
2014346284	663	rural/clinic	No	16/05/2017	0	0	0
2015286546	663	rural/clinic	No	16/05/2017	0	0	0
2014357820	663	rural/clinic	No	16/05/2017	0	0	0
2015390309	663	rural/clinic	No	16/05/2017	0	0	0
2015286544	663	rural/clinic	No	16/05/2017	0	0	0
2014357855	663	rural/clinic	No	16/05/2017	0	0	0
2015418373	663	rural/clinic	No	16/05/2017	0	0	0
2014348068	663	rural/clinic	No	16/05/2017	0	0	0
2014358006	663	rural/clinic	No	16/05/2017	0	0	0

2015286549	663	rural/clinic	No	16/05/2017	0	0	0
2014358008	663	rural/clinic	No	16/05/2017	0	0	0
2015286547	663	rural/clinic	No	16/05/2017	0	0	0
2014319633	663	rural/clinic	No	16/05/2017	0	0	0
2015407051	663	rural/clinic	No	16/05/2017	0	0	0
2015293836	663	rural/clinic	No	16/05/2017	0	0	0
2015352366	663	rural/clinic	No	16/05/2017	0	0	0
2013253948	663	rural/clinic	No	16/05/2017	0	0	0
2011211585	663	rural/clinic	No	16/05/2017	0	0	0
2015413910	663	rural/clinic	No	16/05/2017	0	0	0
2015368876	663	rural/clinic	No	16/05/2017	0	0	0
2014357854	663	rural/clinic	No	16/05/2017	0	0	0
2011211584	663	rural/clinic	No	16/05/2017	0	0	0
2015413908	663	rural/clinic	No	16/05/2017	0	0	0
2011195783	663	rural/clinic	No	16/05/2017	0	0	0
2012322039	663	rural/clinic	No	16/05/2017	0	0	0
2012322040	663	rural/clinic	No	10/05/2017	0	0	0
2015334629	663	rural/clinic	No	16/05/2017	0	0	0
2014372246	663	rural/clinic	No	16/05/2017	0	0	0
2014375697	663	rural/clinic	No	16/05/2017	0	0	0
2014357851	663	rural/clinic	No	16/05/2017	0	0	0
2014375699	246	rural/clinic	No	16/05/2017	0	0	0
2014375700	246	rural/clinic	No	16/05/2017	0	0	0
2013264067	246	rural/clinic	No	16/05/2017	0	0	0
2015413909	246	rural/clinic	No	16/05/2017	0	0	0
2014346729	246	rural/clinic	No	16/05/2017	0	0	0
2015326777	246	rural/clinic	No	16/05/2017	0	0	0
2013253950	246	rural/clinic	No	16/05/2017	0	0	0
2015326778	246	rural/clinic	No	16/05/2017	0	0	0
2013253949	246	rural/clinic	No	16/05/2017	0	0	0
2014383703	246	rural/clinic	No	16/05/2017	0	0	0
2015326779	246	rural/clinic	No	16/05/2017	0	0	0
2011136101	246	rural/clinic	No	16/05/2017	0	0	0
2015286870	246	rural/clinic	No	16/05/2017	0	0	0
2015326780	246	rural/clinic	No	16/05/2017	0	0	0
2012289687	246	rural/clinic	No	16/05/2017	0	0	0
2011136103	246	rural/clinic	No	16/05/2017	0	0	0
2015326781	246	rural/clinic	No	16/05/2017	0	0	0
2015286871	246	rural/clinic	No	16/05/2017	0	0	0
2011136104	246	rural/clinic	No	16/05/2017	0	0	0
2012276255	246	rural/clinic	No	16/05/2017	0	0	0
2011110727/d	246	rural/clinic	No	16/05/2017	0	0	0
2012276253	246	rural/clinic	No	10/05/2017	0	0	0
2011136105	246	rural/clinic	No	16/05/2017	0	0	0
2015363064	246	rural/clinic	No	16/05/2017	0	0	0
2012276254	246	rural/clinic	No	16/05/2017	0	0	0
2014289526	246	rural/clinic	No	16/05/2017	0	0	0
2014383702	246	rural/clinic	No	16/05/2017	0	0	0
2015293475	246	rural/clinic	No	16/05/2017	0	0	0
2015382923	246	rural/clinic	No	16/05/2017	0	0	0
2011144812	246	rural/clinic	No	16/05/2017	0	0	0
2011136106	246	rural/clinic	No	16/05/2017	0	0	0
2015382920	246	rural/clinic	No	10/05/2017	0	0	0
2015326782	246	rural/clinic	No	16/05/2017	0	0	0
2011144811	246	rural/clinic	No	16/05/2017	0	0	0
2015382921	246	rural/clinic	No	16/05/2017	0	0	0
2015382918	246	rural/clinic	No	10/05/2017	0	0	0
2015382919	246	rural/clinic	No	10/05/2017	0	0	0
2015382922	246	rural/clinic	No	16/05/2017	0	0	0
2014306016	246	rural/clinic	No	16/05/2017	0	0	0
2014306015	246	rural/clinic	No	16/05/2017	0	0	0
201066583	246	rural/clinic	No	16/05/2017	0	0	0
2015324474	246	rural/clinic	No	16/05/2017	0	0	0
201066584	246	rural/clinic	No	16/05/2017	0	0	0
2015324442	246	rural/clinic	No	16/05/2017	0	0	0
2015324440	246	rural/clinic	No	16/05/2017	0	0	0
2015324443	246	rural/clinic	No	16/05/2017	0	0	0
2014289527	246	rural/clinic	No	16/05/2017	0	0	0
2014389528	246	rural/clinic	No	16/05/2017	0	0	0
2015326783	384	rural/clinic	No	16/05/2017	0	0	0
2015326766	384	district/faith-based	No	16/05/2017	1	0	0
2014289529	384	district/faith-based	No	16/05/2017	1	0	0
2015372920	384	district/faith-based	No	16/05/2017	1	0	0
2015326767	384	district/faith-based	No	16/05/2017	1	0	0
2015372919	384	district/faith-based	No	16/05/2017	1	0	0
2015326768	384	district/faith-based	No	16/05/2017	1	0	0
2014307540	384	district/faith-based	No	16/05/2017	1	0	0
2015293473	384	district/faith-based	No	16/05/2017	1	0	0
2015326769	384	district/faith-based	No	16/05/2017	1	0	0
2015293474	384	district/faith-based	No	16/05/2017	1	0	0
2014307541	384	district/faith-based	No	16/05/2017	1	0	0
2015326770	384	district/faith-based	No	16/05/2017	1	0	0

2014289525	384	district/faith-based	No	16/05/2017	1	0	0
2015326771	384	district/faith-based	No	16/05/2017	1	0	0
2014307543	384	district/faith-based	No	16/05/2017	1	0	0
2015367209	384	district/faith-based	No	16/05/2017	1	0	0
2015326772	384	district/faith-based	No	16/05/2017	1	0	0
2014307547	384	district/faith-based	No	16/05/2017	1	0	0
2015303119	384	district/faith-based	No	16/05/2017	1	0	0
2015367208	384	district/faith-based	No	16/05/2017	1	0	0
2014307548	384	district/faith-based	No	16/05/2017	1	0	0
2015367210	384	district/faith-based	No	16/05/2017	1	0	0
2014306017	384	district/faith-based	No	16/05/2017	1	0	0
2015363059	384	district/faith-based	No	16/05/2017	1	0	0
2014306012	384	district/faith-based	No	16/05/2017	1	0	0
2014350430	384	district/faith-based	No	16/05/2017	1	0	0
2014350431	384	district/faith-based	No	16/05/2017	1	0	0
2015326773	384	district/faith-based	No	16/05/2017	1	0	0
2014306013	384	district/faith-based	No	16/05/2017	1	0	0
2015326774	384	district/faith-based	No	16/05/2017	1	0	0
2015363060	384	district/faith-based	No	16/05/2017	1	0	0
2015326775	384	district/faith-based	No	16/05/2017	1	0	0
2014371577	384	district/faith-based	No	16/05/2017	1	0	0
2014365383	384	district/faith-based	No	16/05/2017	1	0	0
2015326776	384	district/faith-based	No	16/05/2017	1	0	0
2015352368	384	district/faith-based	No	16/05/2017	1	0	0
2014295876	384	district/faith-based	No	16/05/2017	1	0	0
2012259WRONG	384	district/faith-based	No	16/05/2017	1	0	0
2015352367	384	district/faith-based	No	16/05/2017	1	0	0
2015405965	384	district/faith-based	No	16/05/2017	1	0	0
2012259885	384	district/faith-based	No	16/05/2017	1	0	0
2015352369	384	district/faith-based	No	16/05/2017	1	0	0
2013244115	384	district/faith-based	No	16/05/2017	1	0	0
2014303088	384	district/faith-based	No	16/05/2017	1	0	0
2013244114	384	district/faith-based	No	16/05/2017	1	0	0
2015352370	384	district/faith-based	No	16/05/2017	1	0	0
2015363063	384	district/faith-based	No	16/05/2017	1	0	0
2015334630	384	district/faith-based	No	16/05/2017	1	0	0
2012369496	384	district/faith-based	No	16/05/2017	1	0	0
2015334631	384	district/faith-based	No	16/05/2017	1	0	0
2012330882	384	district/faith-based	No	16/05/2017	1	0	0
2012259887	384	district/faith-based	No	16/05/2017	1	0	0
2014365384	384	district/faith-based	No	16/05/2017	1	0	0
2015362427	384	district/faith-based	No	16/05/2017	1	0	0
2014303089	384	district/faith-based	No	16/05/2017	1	0	0
2015363013	384	district/faith-based	No	16/05/2017	1	0	0
2015314085	384	district/faith-based	No	16/05/2017	1	0	0
2014365385	384	district/faith-based	No	16/05/2017	1	0	0
2015314086	384	district/faith-based	No	16/05/2017	1	0	0
2015368706	384	district/faith-based	No	16/05/2017	1	0	0
2015362426	384	district/faith-based	No	16/05/2017	1	0	0
2015354958	384	district/faith-based	No	16/05/2017	1	0	0
2014288009	384	district/faith-based	No	16/05/2017	1	0	0
2012323777	384	district/faith-based	No	10/05/2017	1	0	0
2015354957	384	district/faith-based	No	16/05/2017	1	0	0
2013278296	384	district/faith-based	No	16/05/2017	1	0	0
2014356987	384	district/faith-based	No	16/05/2017	1	0	0
2015354959	384	district/faith-based	No	16/05/2017	1	0	0
2015324473	384	district/faith-based	No	16/05/2017	1	0	0
2013278297	384	district/faith-based	No	16/05/2017	1	0	0
2015419002	384	district/faith-based	No	16/05/2017	1	0	0
2015354956	384	district/faith-based	No	16/05/2017	1	0	0
2015324441	384	district/faith-based	No	16/05/2017	1	0	0
2014288136	384	district/faith-based	No	16/05/2017	1	0	0
2015314087	384	district/faith-based	No	16/05/2017	1	0	0
2015327104	384	district/faith-based	No	16/05/2017	1	0	0
2015289847	384	district/faith-based	No	16/05/2017	1	0	0
2015315907	384	district/faith-based	No	16/05/2017	1	0	0
2015327106	384	district/faith-based	No	16/05/2017	1	0	0
2015314088	384	district/faith-based	No	16/05/2017	1	0	0
2015289849	384	district/faith-based	No	16/05/2017	1	0	0
2012388389	384	district/faith-based	No	16/05/2017	1	0	0
2015327105	384	district/faith-based	No	16/05/2017	1	0	0
2015354952	384	district/faith-based	No	16/05/2017	1	0	0
2015327107	384	district/faith-based	No	16/05/2017	1	0	0
2015314083	384	district/faith-based	No	16/05/2017	1	0	0
2015327109	384	district/faith-based	No	16/05/2017	1	0	0
2015289848	384	district/faith-based	No	16/05/2017	1	0	0
2015310609	384	district/faith-based	No	16/05/2017	1	0	0
2014287977	384	district/faith-based	No	16/05/2017	1	0	0
2015374449	384	district/faith-based	No	16/05/2017	1	0	0
2015310610	384	district/faith-based	No	16/05/2017	1	0	0
2015310612	384	district/faith-based	No	16/05/2017	1	0	0
2015310613	384	district/faith-based	No	16/05/2017	1	0	0

2015297261	384	district/faith-based	No	16/05/2017	1	0	0
2015297264	384	district/faith-based	No	16/05/2017	1	0	0
2012313809	384	district/faith-based	No	16/05/2017	1	0	0
2012313810	384	district/faith-based	No	16/05/2017	1	0	0
2014327122	384	district/faith-based	No	16/05/2017	1	0	0
2014327123	384	district/faith-based	No	16/05/2017	1	0	0
2014327124	384	district/faith-based	No	16/05/2017	1	0	0
2015301258	384	district/faith-based	No	16/05/2017	1	0	0
2015374442	384	district/faith-based	No	16/05/2017	1	0	0
2015374443	384	district/faith-based	No	16/05/2017	1	0	0
2015374444	384	district/faith-based	No	16/05/2017	1	0	0
2015374445	384	district/faith-based	No	16/05/2017	1	0	0
2015374446	384	district/faith-based	No	16/05/2017	1	0	0
2015374447	384	district/faith-based	No	16/05/2017	1	0	0
2015374448	384	district/faith-based	No	16/05/2017	1	0	0
2012298044	384	district/faith-based	No	16/05/2017	1	0	0
2012298047	384	district/faith-based	No	16/05/2017	1	0	0
2015317002	384	district/faith-based	No	16/05/2017	1	0	0
2015317006	384	district/faith-based	No	16/05/2017	1	0	0
2015317007	384	district/faith-based	No	16/05/2017	1	0	0
2015317008	384	district/faith-based	No	16/05/2017	1	0	0
2015317010	384	district/faith-based	No	16/05/2017	1	0	0
2015388514	384	district/faith-based	No	16/05/2017	1	0	0
2015309001	384	district/faith-based	No	16/05/2017	1	0	0
2015388513	384	district/faith-based	No	16/05/2017	1	0	0
2012370160	384	district/faith-based	No	11/05/2017	1	0	0
2015349602	384	district/faith-based	No	11/05/2017	1	0	0
2014290322	384	district/faith-based	No	16/05/2017	1	0	0
2014290323	384	district/faith-based	No	10/05/2017	1	0	0
2014318244	384	district/faith-based	No	17/05/2017	1	0	0
2013256203	384	district/faith-based	No	17/05/2017	1	0	0
2013256207	384	district/faith-based	No	17/05/2017	1	0	0
2014286903	384	district/faith-based	No	15/05/2017	1	0	0
201710	384	district/faith-based	No	15/05/2017	1	0	0
201611	384	district/faith-based	No	15/05/2017	1	0	0
2012253969	384	district/faith-based	No	16/05/2017	1	0	0
2014298588	384	district/faith-based	No	16/05/2017	1	0	0
2015352705	384	district/faith-based	No	03/05/2017	1	0	0
2015384485	384	district/faith-based	No	16/05/2017	1	0	0
2014317627	384	district/faith-based	No	11/05/2017	1	0	0
2014317627	384	district/faith-based	No	11/05/2017	1	0	0
2015400053	384	district/faith-based	No	11/05/2017	1	0	0
2015400053	384	district/faith-based	No	11/05/2017	1	0	0
2015403278	384	district/faith-based	No	11/05/2017	1	0	0
2015403278	384	district/faith-based	No	11/05/2017	1	0	0
2015407552	384	district/faith-based	No	11/05/2017	1	0	0
2015407552	384	district/faith-based	No	11/05/2017	1	0	0
2015363303	384	district/faith-based	No	11/05/2017	1	0	0
2012341045	384	district/faith-based	No	11/05/2017	1	0	0
2012341045	384	district/faith-based	No	11/05/2017	1	0	0
2015358525	384	district/faith-based	No	11/05/2017	1	0	0
2015358525	384	district/faith-based	No	11/05/2017	1	0	0
2015286302	384	district/faith-based	No	11/05/2017	1	0	0
2015286302	384	district/faith-based	No	11/05/2017	1	0	0
2015339021	384	district/faith-based	No	11/05/2017	1	0	0
2015384542	384	district/faith-based	No	11/05/2017	1	0	0
2012265638	384	district/faith-based	No	11/05/2017	1	0	0
2015369179	384	district/faith-based	No	11/05/2017	1	0	0
2015328477	384	district/faith-based	No	11/05/2017	1	0	0
2015328477	384	district/faith-based	No	11/05/2017	1	0	0
2015400065	384	district/faith-based	No	11/05/2017	1	0	0
2015400065	384	district/faith-based	No	11/05/2017	1	0	0
2015385525	384	district/faith-based	No	11/05/2017	1	0	0
2015385525	384	district/faith-based	No	11/05/2017	1	0	0
2015352704/d	384	district/faith-based	No	03/05/2017	1	0	0
2011140753	384	district/faith-based	No	16/05/2017	1	0	0
2011140752	384	district/faith-based	No	16/05/2017	1	0	0
2015305076	384	district/faith-based	No	11/05/2017	1	0	0
2015305076	384	district/faith-based	No	11/05/2017	1	0	0
2015407058	384	district/faith-based	No	11/05/2017	1	0	0
2012291353	280	rural/clinic	No	05/05/2017	0	0	0
2014291890	280	rural/clinic	No	17/05/2017	0	0	0
2015322044	280	rural/clinic	No	17/05/2017	0	0	0
2013256206	280	rural/clinic	No	17/05/2017	0	0	0
2014382919	280	rural/clinic	No	16/05/2017	0	0	0
2013271695	280	rural/clinic	No	16/05/2017	0	0	0
2012284746	280	rural/clinic	No	16/05/2017	0	0	0
2014295875	280	rural/clinic	No	16/05/2017	0	0	0
2015333852	280	rural/clinic	No	16/05/2017	0	0	0
2011144810	280	rural/clinic	No	16/05/2017	0	0	0
2011197677	280	rural/clinic	No	16/05/2017	0	0	0
2015413908	280	rural/clinic	No	16/05/2017	0	0	0

2015413908	280	rural/clinic	No	16/05/2017	0	0	0
2015326766	280	rural/clinic	No	16/05/2017	0	0	0
2015326766	280	rural/clinic	No	16/05/2017	0	0	0
2011135665	280	rural/clinic	No	16/05/2017	0	0	0
2011135665	280	rural/clinic	No	16/05/2017	0	0	0
2011135666	280	rural/clinic	No	16/05/2017	0	0	0
2011135666	280	rural/clinic	No	16/05/2017	0	0	0
2015344088	280	rural/clinic	No	16/05/2017	0	0	0
2015344088	280	rural/clinic	No	16/05/2017	0	0	0
2013249417	280	rural/clinic	No	16/05/2017	0	0	0
2013249417	280	rural/clinic	No	16/05/2017	0	0	0
2015377887	280	rural/clinic	No	16/05/2017	0	0	0
2015377887	280	rural/clinic	No	16/05/2017	0	0	0
2015377888	280	rural/clinic	No	16/05/2017	0	0	0
2015377888	280	rural/clinic	No	16/05/2017	0	0	0
2015297475	280	rural/clinic	No	16/05/2017	0	0	0
2015297475	280	rural/clinic	No	16/05/2017	0	0	0
2015297332	280	rural/clinic	No	16/05/2017	0	0	0
2015297332	280	rural/clinic	No	16/05/2017	0	0	0
2013247834	280	rural/clinic	No	11/05/2017	0	0	0
2013247834	280	rural/clinic	No	11/05/2017	0	0	0
2014307328	280	rural/clinic	No	15/05/2017	0	0	0
2014307328	280	rural/clinic	No	15/05/2017	0	0	0
2014300429	280	rural/clinic	No	11/05/2017	0	0	0
2011157146	280	rural/clinic	No	11/05/2017	0	0	0
2011157146	280	rural/clinic	No	11/05/2017	0	0	0
2015402623	280	rural/clinic	No	11/05/2017	0	0	0
2013276155	280	rural/clinic	No	11/05/2017	0	0	0
2013276155	280	rural/clinic	No	11/05/2017	0	0	0
2011134989	280	rural/clinic	No	16/05/2017	0	0	0
2015342312	280	rural/clinic	No	16/05/2017	0	0	0
2015342312	280	rural/clinic	No	16/05/2017	0	0	0
2014383050	280	rural/clinic	No	18/05/2017	0	0	0
2015317002	280	rural/clinic	No	16/05/2017	0	0	0
2014383048	280	rural/clinic	No	18/05/2017	0	0	0
2012330882	280	rural/clinic	No	16/05/2017	0	0	0
2012330882	280	rural/clinic	No	16/05/2017	0	0	0
2014356987	280	rural/clinic	No	16/05/2017	0	0	0
2014356987	280	rural/clinic	No	16/05/2017	0	0	0
2011218358	280	rural/clinic	No	19/05/2017	0	0	0
2014358213	280	rural/clinic	No	16/05/2017	0	0	0
2014358213	280	rural/clinic	No	16/05/2017	0	0	0
2014319633	280	rural/clinic	No	16/05/2017	0	0	0
2014319633	280	rural/clinic	No	16/05/2017	0	0	0
2015340981	280	rural/clinic	No	18/05/2017	0	0	0
2013261231	280	rural/clinic	No	19/05/2017	0	0	0
2013261232	280	rural/clinic	No	19/05/2017	0	0	0
2015340982	280	rural/clinic	No	18/05/2017	0	0	0
2013261233	280	rural/clinic	No	19/05/2017	0	0	0
2013261234	280	rural/clinic	No	19/05/2017	0	0	0
2014383527	280	rural/clinic	No	18/05/2017	0	0	0
2013261235	280	rural/clinic	No	19/05/2017	0	0	0
2015362487	280	rural/clinic	No	19/05/2017	0	0	0
2015332303	280	rural/clinic	No	18/05/2017	0	0	0
2015362488	280	rural/clinic	No	19/05/2017	0	0	0
2014306015	280	rural/clinic	No	16/05/2017	0	0	0
2014306015	280	rural/clinic	No	16/05/2017	0	0	0
2015324443	280	rural/clinic	No	16/05/2017	0	0	0
2015324443	280	rural/clinic	No	16/05/2017	0	0	0
2015362489	280	rural/clinic	No	19/05/2017	0	0	0
2012246285	280	rural/clinic	No	18/05/2017	0	0	0
2015362491	280	rural/clinic	No	19/05/2017	0	0	0
2015362492	280	rural/clinic	No	19/05/2017	0	0	0
2011218359	280	rural/clinic	No	19/05/2017	0	0	0
2013258729	280	rural/clinic	No	18/05/2017	0	0	0
2015362493	280	rural/clinic	No	19/05/2017	0	0	0
2014307506	280	rural/clinic	No	19/05/2017	0	0	0
2012361947	280	rural/clinic	No	19/05/2017	0	0	0
2014338556	280	rural/clinic	No	18/05/2017	0	0	0
2014307508	280	rural/clinic	No	19/05/2017	0	0	0
2014340325	280	rural/clinic	No	18/05/2017	0	0	0
2014348114	280	rural/clinic	No	18/05/2017	0	0	0
2014307509	280	rural/clinic	No	19/05/2017	0	0	0
2015412115	280	rural/clinic	No	18/05/2017	0	0	0
2014307510	280	rural/clinic	No	19/05/2017	0	0	0
2014307511	280	rural/clinic	No	19/05/2017	0	0	0
2015297265	280	rural/clinic	No	18/05/2017	0	0	0
2014296326	280	rural/clinic	No	18/05/2017	0	0	0
2014307512	280	rural/clinic	No	19/05/2017	0	0	0
2015286307	280	rural/clinic	No	18/05/2017	0	0	0
2014307513	280	rural/clinic	No	19/05/2017	0	0	0
2015297267	280	rural/clinic	No	18/05/2017	0	0	0

2015414559	280	rural/clinic	No	18/05/2017	0	0	0
2014307514	280	rural/clinic	No	19/05/2017	0	0	0
2011204795	280	rural/clinic	No	19/05/2017	0	0	0
2015286308	280	rural/clinic	No	18/05/2017	0	0	0
2011218357	280	rural/clinic	No	19/05/2017	0	0	0
2015286306	280	rural/clinic	No	18/05/2017	0	0	0
2012384798	280	rural/clinic	No	18/05/2017	0	0	0
2012384799	280	rural/clinic	No	18/05/2017	0	0	0
2012369970	280	rural/clinic	No	18/05/2017	0	0	0
2014319523	280	rural/clinic	No	18/05/2017	0	0	0
2015328062	280	rural/clinic	No	18/05/2017	0	0	0
2014326635	280	rural/clinic	No	18/05/2017	0	0	0
2015351976	280	rural/clinic	No	18/05/2017	0	0	0
2014326636	280	rural/clinic	No	18/05/2017	0	0	0
2011141104	280	rural/clinic	No	18/05/2017	0	0	0
2015351977	280	rural/clinic	No	18/05/2017	0	0	0
2011141105	280	rural/clinic	No	18/05/2017	0	0	0
2015328063	280	rural/clinic	No	18/05/2017	0	0	0
2015351978	280	rural/clinic	No	18/05/2017	0	0	0
2015328064	280	rural/clinic	No	18/05/2017	0	0	0
2014362826	280	rural/clinic	No	18/05/2017	0	0	0
2015351979	280	rural/clinic	No	18/05/2017	0	0	0
2015328061	280	rural/clinic	No	18/05/2017	0	0	0
2014362827	280	rural/clinic	No	18/05/2017	0	0	0
2015351980	280	rural/clinic	No	18/05/2017	0	0	0
2015408677	280	rural/clinic	No	18/05/2017	0	0	0
2014307501	280	rural/clinic	No	19/05/2017	0	0	0
2015408678	280	rural/clinic	No	18/05/2017	0	0	0
2015326580	280	rural/clinic	No	18/05/2017	0	0	0
2015351981	280	rural/clinic	No	18/05/2017	0	0	0
2015373412	280	rural/clinic	No	18/05/2017	0	0	0
2014307503	280	rural/clinic	No	19/05/2017	0	0	0
2012358288	280	rural/clinic	No	09/05/2017	0	0	0
2015351982	280	rural/clinic	No	18/05/2017	0	0	0
2014307504	280	rural/clinic	No	19/05/2017	0	0	0
2015373413	280	rural/clinic	No	18/05/2017	0	0	0
2015351983	280	rural/clinic	No	18/05/2017	0	0	0
2015373414	280	rural/clinic	No	18/05/2017	0	0	0
2014362816	280	rural/clinic	No	18/05/2017	0	0	0
2015373415	280	rural/clinic	No	18/05/2017	0	0	0
2015351975	280	rural/clinic	No	18/05/2017	0	0	0
2014362825	280	rural/clinic	No	18/05/2017	0	0	0
2015373416	280	rural/clinic	No	18/05/2017	0	0	0
2014307505	280	rural/clinic	No	19/05/2017	0	0	0
2011155230	280	rural/clinic	No	18/05/2017	0	0	0
2014362824	580	rural/clinic	No	18/05/2017	0	0	0
2015332352	580	rural/clinic	No	18/05/2017	0	0	0
2015334617	580	rural/clinic	No	19/05/2017	0	0	0
2015332351	580	rural/clinic	No	18/05/2017	0	0	0
2011155231	580	rural/clinic	No	18/05/2017	0	0	0
2011204474	580	rural/clinic	No	18/05/2017	0	0	0
2015334618	580	rural/clinic	No	19/05/2017	0	0	0
2015358193	580	rural/clinic	No	18/05/2017	0	0	0
2013258607	580	rural/clinic	No	18/05/2017	0	0	0
2015334619	580	rural/clinic	No	19/05/2017	0	0	0
2012365984	580	rural/clinic	No	18/05/2017	0	0	0
2015334622	580	rural/clinic	No	19/05/2017	0	0	0
2014362823	580	rural/clinic	No	18/05/2017	0	0	0
2012294473	580	rural/clinic	No	18/05/2017	0	0	0
2015334623	580	rural/clinic	No	19/05/2017	0	0	0
2014362822	580	rural/clinic	No	18/05/2017	0	0	0
2015358194	580	rural/clinic	No	18/05/2017	0	0	0
2014362817	580	rural/clinic	No	18/05/2017	0	0	0
2011141066	580	rural/clinic	No	19/05/2017	0	0	0
2015358196	580	rural/clinic	No	18/05/2017	0	0	0
2012359942	580	rural/clinic	No	18/05/2017	0	0	0
2011141067	580	rural/clinic	No	19/05/2017	0	0	0
2012359943	580	rural/clinic	No	18/05/2017	0	0	0
2015358197	580	rural/clinic	No	18/05/2017	0	0	0
2011141068	580	rural/clinic	No	19/05/2017	0	0	0
2012359944	580	rural/clinic	No	18/05/2017	0	0	0
2015332459	580	rural/clinic	No	18/05/2017	0	0	0
2015358198	580	rural/clinic	No	18/05/2017	0	0	0
2011141070	580	rural/clinic	No	19/05/2017	0	0	0
2015332462	580	rural/clinic	No	18/05/2017	0	0	0
2011141071	580	rural/clinic	No	19/05/2017	0	0	0
2012359945	580	rural/clinic	No	18/05/2017	0	0	0
2015358199	580	rural/clinic	No	18/05/2017	0	0	0
2011141072	580	rural/clinic	No	19/05/2017	0	0	0
2015332461	580	rural/clinic	No	18/05/2017	0	0	0
2012294472	580	rural/clinic	No	18/05/2017	0	0	0
2012359946	580	rural/clinic	No	18/05/2017	0	0	0

2015361432	580	rural/clinic	No	18/05/2017	0	0	0
2012359947	580	rural/clinic	No	18/05/2017	0	0	0
2012294471	580	rural/clinic	No	18/05/2017	0	0	0
2014371394	580	rural/clinic	No	18/05/2017	0	0	0
2015361433	580	rural/clinic	No	18/05/2017	0	0	0
2015355915	580	rural/clinic	No	18/05/2017	0	0	0
2014371396	580	rural/clinic	No	18/05/2017	0	0	0
2015361428	580	rural/clinic	No	18/05/2017	0	0	0
2015355916	580	rural/clinic	No	18/05/2017	0	0	0
2014371395	580	rural/clinic	No	11/05/2017	0	0	0
2015332460	580	rural/clinic	No	18/05/2017	0	0	0
2015362252	580	rural/clinic	No	18/05/2017	0	0	0
2015361425	580	rural/clinic	No	18/05/2017	0	0	0
2014371397	580	rural/clinic	No	18/05/2017	0	0	0
2015362253	580	rural/clinic	No	18/05/2017	0	0	0
2015361430	580	rural/clinic	No	18/05/2017	0	0	0
2015362254	580	rural/clinic	No	18/05/2017	0	0	0
2015332463	580	rural/clinic	No	18/05/2017	0	0	0
2015362255	580	rural/clinic	No	18/05/2017	0	0	0
2015315906	580	rural/clinic	No	09/05/2017	0	0	0
2015362256	580	rural/clinic	No	18/05/2017	0	0	0
2015361427	580	rural/clinic	No	18/05/2017	0	0	0
2011223206	580	rural/clinic	No	09/05/2017	0	0	0
2015362257	580	rural/clinic	No	18/05/2017	0	0	0
2015361436	580	rural/clinic	No	18/05/2017	0	0	0
2015362258	580	rural/clinic	No	18/05/2017	0	0	0
2015355516	580	rural/clinic	No	09/05/2017	0	0	0
2015362259	580	rural/clinic	No	18/05/2017	0	0	0
2011223207	580	rural/clinic	No	09/05/2017	0	0	0
2015361439	580	rural/clinic	No	18/05/2017	0	0	0
2013274248	580	rural/clinic	No	18/05/2017	0	0	0
2011133911	580	rural/clinic	No	18/05/2017	0	0	0
2015361437	580	rural/clinic	No	18/05/2017	0	0	0
2015355003	580	rural/clinic	No	18/05/2017	0	0	0
2013270348	580	rural/clinic	No	18/05/2017	0	0	0
2015361435	580	rural/clinic	No	18/05/2017	0	0	0
2015355002	580	rural/clinic	No	18/05/2017	0	0	0
2015326581	580	rural/clinic	No	18/05/2017	0	0	0
2015361438	580	rural/clinic	No	18/05/2017	0	0	0
2012344080	580	rural/clinic	No	18/05/2017	0	0	0
2015414659	580	rural/clinic	No	18/05/2017	0	0	0
2015361434	580	rural/clinic	No	18/05/2017	0	0	0
2015301479	580	rural/clinic	No	18/05/2017	0	0	0
2015368285	580	rural/clinic	No	18/05/2017	0	0	0
2015301267	580	rural/clinic	No	18/05/2017	0	0	0
2015361426	49	rural/clinic	No	18/05/2017	0	0	0
2015301478	49	rural/clinic	No	18/05/2017	0	0	0
2015368286	49	rural/clinic	No	18/05/2017	0	0	0
2015361429	49	rural/clinic	No	18/05/2017	0	0	0
2015301477	49	rural/clinic	No	18/05/2017	0	0	0
2015368287	49	rural/clinic	No	18/05/2017	0	0	0
2015302106	49	rural/clinic	No	18/05/2017	0	0	0
2015305077	49	rural/clinic	No	18/05/2017	0	0	0
2011225414	49	rural/clinic	No	18/05/2017	0	0	0
2011225413	49	rural/clinic	No	18/05/2017	0	0	0
2015361431	49	rural/clinic	No	18/05/2017	0	0	0
2015305078	49	rural/clinic	No	18/05/2017	0	0	0
2015326389	49	rural/clinic	No	18/05/2017	0	0	0
2015419003	49	rural/clinic	No	18/05/2017	0	0	0
2015305079	49	rural/clinic	No	18/05/2017	0	0	0
2015326543	49	rural/clinic	No	18/05/2017	0	0	0
2015305080	49	rural/clinic	No	18/05/2017	0	0	0
2015326544	49	rural/clinic	No	18/05/2017	0	0	0
2011133641	49	rural/clinic	No	18/05/2017	0	0	0
2015326545	49	rural/clinic	No	18/05/2017	0	0	0
2011133642	49	rural/clinic	No	18/05/2017	0	0	0
2015326546	49	rural/clinic	No	18/05/2017	0	0	0
2015419004	49	rural/clinic	No	18/05/2017	0	0	0
2011133643	49	rural/clinic	No	18/05/2017	0	0	0
2015326547	49	rural/clinic	No	18/05/2017	0	0	0
2015375997	49	rural/clinic	No	18/05/2017	0	0	0
2012323663	49	rural/clinic	No	18/05/2017	0	0	0
2015351689	49	rural/clinic	No	18/05/2017	0	0	0
2012323664	49	rural/clinic	No	18/05/2017	0	0	0
2015415269	49	rural/clinic	No	19/05/2017	0	0	0
2015351690	49	rural/clinic	No	18/05/2017	0	0	0
2015351691	49	rural/clinic	No	18/05/2017	0	0	0
2015415915	49	rural/clinic	No	19/05/2017	0	0	0
2015415914	49	rural/clinic	No	19/05/2017	0	0	0
2015375998	49	rural/clinic	No	18/05/2017	0	0	0
2015351692	49	rural/clinic	No	18/05/2017	0	0	0
2011203845	49	rural/clinic	No	18/05/2017	0	0	0

2010099915	49	rural/clinic	No	18/05/2017	0	0	0
2010099916	49	rural/clinic	No	18/05/2017	0	0	0
2010099917	49	rural/clinic	No	18/05/2017	0	0	0
2011200278	49	rural/clinic	No	18/05/2017	0	0	0
2012259883	49	rural/clinic	No	16/05/2017	0	0	0
2012253972	49	rural/clinic	No	18/05/2017	0	0	0
2015375996	49	rural/clinic	No	18/05/2017	0	0	0
2015375995	49	rural/clinic	No	18/05/2017	0	0	0
2015357650	49	rural/clinic	No	18/05/2017	0	0	0
2015412095	49	rural/clinic	No	18/05/2017	0	0	0
2015351693	49	rural/clinic	No	18/05/2017	0	0	0
2015340701	49	rural/clinic	No	18/05/2017	0	0	0
2015412093	49	rural/clinic	No	18/05/2017	0	0	0
2015351694	49	rural/clinic	No	18/05/2017	0	0	0
2014317629	49	rural/clinic	No	18/05/2017	0	0	0
2015325998	49	rural/clinic	No	18/05/2017	0	0	0
2015351695	49	rural/clinic	No	18/05/2017	0	0	0
2014332081	49	rural/clinic	No	16/05/2017	0	0	0
2014302942	49	rural/clinic	No	18/05/2017	0	0	0
2015351696	49	rural/clinic	No	18/05/2017	0	0	0
2014302943	49	rural/clinic	No	18/05/2017	0	0	0
2011199086	49	rural/clinic	No	18/05/2017	0	0	0
2015351697	49	rural/clinic	No	18/05/2017	0	0	0
2011199087	49	rural/clinic	No	18/05/2017	0	0	0
2011199085	49	rural/clinic	No	18/05/2017	0	0	0
2011212963	49	rural/clinic	No	18/05/2017	0	0	0
2015349459	49	rural/clinic	No	18/05/2017	0	0	0
2014357534	49	rural/clinic	No	18/05/2017	0	0	0
2011225372	49	rural/clinic	No	18/05/2017	0	0	0
2014357531	49	rural/clinic	No	18/05/2017	0	0	0
2014303299	49	rural/clinic	No	18/05/2017	0	0	0
2014350456	49	rural/clinic	No	18/05/2017	0	0	0
2014357533	49	rural/clinic	No	18/05/2017	0	0	0
2014350457	49	rural/clinic	No	18/05/2017	0	0	0
2014357532	49	rural/clinic	No	18/05/2017	0	0	0
2014350455	49	rural/clinic	No	18/05/2017	0	0	0
2014378268	49	rural/clinic	No	18/05/2017	0	0	0
2015385527	49	rural/clinic	No	18/05/2017	0	0	0
2015385530	49	rural/clinic	No	18/05/2017	0	0	0
2014357530	49	rural/clinic	No	18/05/2017	0	0	0
2014336000	49	rural/clinic	No	18/05/2017	0	0	0
2014357529	49	rural/clinic	No	18/05/2017	0	0	0
2015334616	49	rural/clinic	No	19/05/2017	0	0	0
2014315706	49	rural/clinic	No	18/05/2017	0	0	0
2015412094	49	rural/clinic	No	18/05/2017	0	0	0
2014315704	49	rural/clinic	No	18/05/2017	0	0	0
2014356988	49	rural/clinic	No	18/05/2017	0	0	0
2014315705	49	rural/clinic	No	18/05/2017	0	0	0
2015397804	49	rural/clinic	No	18/05/2017	0	0	0
2012253971	49	rural/clinic	No	18/05/2017	0	0	0
2015397805	49	rural/clinic	No	18/05/2017	0	0	0
2012313811	49	rural/clinic	No	18/05/2017	0	0	0
2015397806	49	rural/clinic	No	18/05/2017	0	0	0
2015397807	49	rural/clinic	No	18/05/2017	0	0	0
2014356986	49	rural/clinic	No	18/05/2017	0	0	0
2015397808	49	rural/clinic	No	18/05/2017	0	0	0
2015385529	49	rural/clinic	No	18/05/2017	0	0	0
2015397809	49	rural/clinic	No	18/05/2017	0	0	0
2015385528	49	rural/clinic	No	18/05/2017	0	0	0
2015328462	49	rural/clinic	No	18/05/2017	0	0	0
2014305222	49	rural/clinic	No	18/05/2017	0	0	0
2015328471	49	rural/clinic	No	18/05/2017	0	0	0
2012377878	49	rural/clinic	No	18/05/2017	0	0	0
2015328486	49	rural/clinic	No	18/05/2017	0	0	0
2015328487	49	rural/clinic	No	18/05/2017	0	0	0
2015328488	49	rural/clinic	No	18/05/2017	0	0	0
2015328489	49	rural/clinic	No	18/05/2017	0	0	0
2015328490	49	rural/clinic	No	18/05/2017	0	0	0
2015328491	49	rural/clinic	No	18/05/2017	0	0	0
2015328491	49	rural/clinic	No	18/05/2017	0	0	0
2015328492	49	rural/clinic	No	18/05/2017	0	0	0
2015328494	49	rural/clinic	No	18/05/2017	0	0	0
2015328495	49	rural/clinic	No	18/05/2017	0	0	0
2015358537	49	rural/clinic	No	18/05/2017	0	0	0
2015328496	49	rural/clinic	No	18/05/2017	0	0	0
2015358538	49	rural/clinic	No	18/05/2017	0	0	0
2015328497	49	rural/clinic	No	18/05/2017	0	0	0
2015358539	49	rural/clinic	No	18/05/2017	0	0	0
2015414384	49	rural/clinic	No	18/05/2017	0	0	0
2015414385	49	rural/clinic	No	18/05/2017	0	0	0
2015358528	49	rural/clinic	No	18/05/2017	0	0	0
2012265923	49	rural/clinic	No	18/05/2017	0	0	0

2015358529	49	rural/clinic	No	18/05/2017	0	0	0
2015358530	49	rural/clinic	No	18/05/2017	0	0	0
2015358531	49	rural/clinic	No	18/05/2017	0	0	0
2015358532	49	rural/clinic	No	18/05/2017	0	0	0
2015358534	49	rural/clinic	No	18/05/2017	0	0	0
2015379321	49	rural/clinic	No	18/05/2017	0	0	0
2015379319	49	rural/clinic	No	18/05/2017	0	0	0
2015362516	49	rural/clinic	No	18/05/2017	0	0	0
2015362515	49	rural/clinic	No	18/05/2017	0	0	0
2015324260	49	rural/clinic	No	18/05/2017	0	0	0
2015352717	49	rural/clinic	No	18/05/2017	0	0	0
2015332025	49	rural/clinic	No	18/05/2017	0	0	0
2015332024	49	rural/clinic	No	18/05/2017	0	0	0
2015324259	49	rural/clinic	No	18/05/2017	0	0	0
2014383047	49	rural/clinic	No	18/05/2017	0	0	0
2015332026	49	rural/clinic	No	18/05/2017	0	0	0
2012305587	49	rural/clinic	No	18/05/2017	0	0	0
2012305586	49	rural/clinic	No	18/05/2017	0	0	0
2013258455	49	rural/clinic	No	18/05/2017	0	0	0
2014372336	49	rural/clinic	No	18/05/2017	0	0	0
2015369183	49	rural/clinic	No	18/05/2017	0	0	0
2015369184	49	rural/clinic	No	18/05/2017	0	0	0
2015412116	49	rural/clinic	No	18/05/2017	0	0	0
2014326636	49	rural/clinic	No	18/05/2017	0	0	0
2014326636	49	rural/clinic	No	18/05/2017	0	0	0
2014362824	49	rural/clinic	No	18/05/2017	0	0	0
2014362824	49	rural/clinic	No	18/05/2017	0	0	0
2013261233	49	rural/clinic	No	19/05/2017	0	0	0
2013261233	49	rural/clinic	No	19/05/2017	0	0	0
2015361425	49	rural/clinic	No	18/05/2017	0	0	0
2015361425	49	rural/clinic	No	18/05/2017	0	0	0
2015361426	49	rural/clinic	No	18/05/2017	0	0	0
2015361426	49	rural/clinic	No	18/05/2017	0	0	0
2013256203	49	rural/clinic	No	17/05/2017	0	0	0
2013256203	49	rural/clinic	No	17/05/2017	0	0	0
2014291890	49	rural/clinic	No	17/05/2017	0	0	0
2014291890	49	rural/clinic	No	17/05/2017	0	0	0
2011223206	49	rural/clinic	No	09/05/2017	0	0	0
2011223206	49	rural/clinic	No	09/05/2017	0	0	0
2015412095	49	rural/clinic	No	18/05/2017	0	0	0
2015412095	49	rural/clinic	No	18/05/2017	0	0	0
2011141068	49	rural/clinic	No	19/05/2017	0	0	0
2015368287	49	rural/clinic	No	18/05/2017	0	0	0
2015368287	49	rural/clinic	No	18/05/2017	0	0	0
2015305077	49	rural/clinic	No	18/05/2017	0	0	0
2015305077	49	rural/clinic	No	18/05/2017	0	0	0
2015305080	49	rural/clinic	No	18/05/2017	0	0	0
2015305080	49	rural/clinic	No	18/05/2017	0	0	0
2011133643	49	rural/clinic	No	18/05/2017	0	0	0
2011133643	49	rural/clinic	No	18/05/2017	0	0	0
2011212963	49	rural/clinic	No	18/05/2017	0	0	0
2015334616	49	rural/clinic	No	19/05/2017	0	0	0
2015362515	49	rural/clinic	No	18/05/2017	0	0	0
2015362515	49	rural/clinic	No	18/05/2017	0	0	0
2015324260	49	rural/clinic	No	18/05/2017	0	0	0
2015324260	49	rural/clinic	No	18/05/2017	0	0	0
2015368968	49	rural/clinic	No	23/05/2017	0	0	0
2012344498	49	rural/clinic	No	23/05/2017	0	0	0
2015368969	49	rural/clinic	No	23/05/2017	0	0	0
2012344499	49	rural/clinic	No	23/05/2017	0	0	0
2015326548	49	rural/clinic	No	23/05/2017	0	0	0
2015368970	49	rural/clinic	No	23/05/2017	0	0	0
2015326549	49	rural/clinic	No	23/05/2017	0	0	0
2015368971	49	rural/clinic	No	23/05/2017	0	0	0
2015368972	49	rural/clinic	No	23/05/2017	0	0	0
2015326397	49	rural/clinic	No	23/05/2017	0	0	0
2015326398	49	rural/clinic	No	23/05/2017	0	0	0
2011221330	49	rural/clinic	No	23/05/2017	0	0	0
2014352406	49	rural/clinic	No	23/05/2017	0	0	0
2014352404	49	rural/clinic	No	23/05/2017	0	0	0
2014371164	49	rural/clinic	No	23/05/2017	0	0	0
2012295644	49	rural/clinic	No	23/05/2017	0	0	0
2012344495	49	rural/clinic	No	24/05/2017	0	0	0
2014328229	49	rural/clinic	No	24/05/2017	0	0	0
2014328230	49	rural/clinic	No	24/05/2017	0	0	0
2015335120	49	rural/clinic	No	24/05/2017	0	0	0
2015335121	49	rural/clinic	No	24/05/2017	0	0	0
2015335122	49	rural/clinic	No	24/05/2017	0	0	0
2015335123	49	rural/clinic	No	24/05/2017	0	0	0
2015335124	49	rural/clinic	No	24/05/2017	0	0	0
2015335381	49	rural/clinic	No	24/05/2017	0	0	0
2015335382	49	rural/clinic	No	24/05/2017	0	0	0

2015335383	49	rural/clinic	No	24/05/2017	0	0	0
2015335385	49	rural/clinic	No	24/05/2017	0	0	0
2015377324	49	rural/clinic	No	24/05/2017	0	0	0
2015377325	49	rural/clinic	No	24/05/2017	0	0	0
2014371579	49	rural/clinic	No	23/05/2017	0	0	0
2015331945	49	rural/clinic	No	24/05/2017	0	0	0
2014371578	49	rural/clinic	No	23/05/2017	0	0	0
2015331942	49	rural/clinic	No	24/05/2017	0	0	0
2015362260	49	rural/clinic	No	23/05/2017	0	0	0
2015331943	49	rural/clinic	No	24/05/2017	0	0	0
2015328498	49	rural/clinic	No	24/05/2017	0	0	0
2015362261	49	rural/clinic	No	23/05/2017	0	0	0
2015406272	49	rural/clinic	No	24/05/2017	0	0	0
2015362262	49	rural/clinic	No	23/05/2017	0	0	0
2014314217	49	rural/clinic	No	23/05/2017	0	0	0
2014314218	49	rural/clinic	No	23/05/2017	0	0	0
2011143085	49	rural/clinic	No	23/05/2017	0	0	0
2015360731	49	rural/clinic	No	23/05/2017	0	0	0
2015360730	49	rural/clinic	No	23/05/2017	0	0	0
2015360729	49	rural/clinic	No	23/05/2017	0	0	0
2015406271	49	rural/clinic	No	24/05/2017	0	0	0
2015327108	49	rural/clinic	No	24/05/2017	0	0	0
2011186260	49	rural/clinic	No	24/05/2017	0	0	0
2011186261	49	rural/clinic	No	24/05/2017	0	0	0
2015406275	49	rural/clinic	No	24/05/2017	0	0	0
2011226484	49	rural/clinic	No	24/05/2017	0	0	0
2015362054	49	rural/clinic	No	24/05/2017	0	0	0
2015362056	49	rural/clinic	No	24/05/2017	0	0	0
2013255263	49	rural/clinic	No	23/05/2017	0	0	0
2015362055	49	rural/clinic	No	24/05/2017	0	0	0
2015293477	49	rural/clinic	No	24/05/2017	0	0	0
2014291892	49	rural/clinic	No	23/05/2017	0	0	0
2015397622	49	rural/clinic	No	23/05/2017	0	0	0
2015397623	49	rural/clinic	No	23/05/2017	0	0	0
2015397624	49	rural/clinic	No	23/05/2017	0	0	0
2015397625	49	rural/clinic	No	23/05/2017	0	0	0
2015397626	49	rural/clinic	No	23/05/2017	0	0	0
2011123775	49	rural/clinic	No	23/05/2017	0	0	0
2011123780	49	rural/clinic	No	23/05/2017	0	0	0
2015326785	49	rural/clinic	No	23/05/2017	0	0	0
2015326786	49	rural/clinic	No	23/05/2017	0	0	0
2015326787	49	rural/clinic	No	23/05/2017	0	0	0
2015326788	49	rural/clinic	No	23/05/2017	0	0	0
2014347014	49	rural/clinic	No	24/05/2017	0	0	0
2015326789	49	rural/clinic	No	23/05/2017	0	0	0
2014306018	49	rural/clinic	No	23/05/2017	0	0	0
2014306019	49	rural/clinic	No	23/05/2017	0	0	0
2015368584	49	rural/clinic	No	23/05/2017	0	0	0
2015368585	49	rural/clinic	No	23/05/2017	0	0	0
2015368586	49	rural/clinic	No	23/05/2017	0	0	0
2013256269	49	rural/clinic	No	23/05/2017	0	0	0
2013256270	581	rural/clinic	No	23/05/2017	0	0	0
2014383704	581	rural/clinic	No	23/05/2017	0	0	0
2012275987	581	rural/clinic	No	23/05/2017	0	0	0
2012275986	581	rural/clinic	No	23/05/2017	0	0	0
2012275988	581	rural/clinic	No	23/05/2017	0	0	0
2015417058	581	rural/clinic	No	23/05/2017	0	0	0
2015417059	581	rural/clinic	No	23/05/2017	0	0	0
2015417060	581	rural/clinic	No	23/05/2017	0	0	0
2015289176	581	rural/clinic	No	23/05/2017	0	0	0
2015332401	581	rural/clinic	No	23/05/2017	0	0	0
2015418858	581	rural/clinic	No	23/05/2017	0	0	0
2012323778	581	rural/clinic	No	23/05/2017	0	0	0
2012388392	581	rural/clinic	No	23/05/2017	0	0	0
2014317629	581	rural/clinic	No	18/05/2017	0	0	0
2014317629	581	rural/clinic	No	18/05/2017	0	0	0
2015325998	581	rural/clinic	No	18/05/2017	0	0	0
2015325998	581	rural/clinic	No	18/05/2017	0	0	0
2014302942	581	rural/clinic	No	18/05/2017	0	0	0
2014302942	581	rural/clinic	No	18/05/2017	0	0	0
2011199087	581	rural/clinic	No	18/05/2017	0	0	0
2011199087	581	rural/clinic	No	18/05/2017	0	0	0
2012344853	581	rural/clinic	No	23/05/2017	0	0	0
2014350455	581	rural/clinic	No	18/05/2017	0	0	0
2014350455	581	rural/clinic	No	18/05/2017	0	0	0
2012388391	581	rural/clinic	No	23/05/2017	0	0	0
2015324444	581	rural/clinic	No	23/05/2017	0	0	0
2013254806	581	rural/clinic	No	23/05/2017	0	0	0
2015315908	581	rural/clinic	No	23/05/2017	0	0	0
2012345100	581	rural/clinic	No	23/05/2017	0	0	0
2015355517	581	rural/clinic	No	23/05/2017	0	0	0
2012344854	581	rural/clinic	No	23/05/2017	0	0	0

2015299095	581	rural/clinic	No	24/05/2017	0	0	0
2015324475	581	rural/clinic	No	23/05/2017	0	0	0
2015318732	581	rural/clinic	No	23/05/2017	0	0	0
2015355518	581	rural/clinic	No	23/05/2017	0	0	0
2015318733	581	rural/clinic	No	23/05/2017	0	0	0
2015315909	581	rural/clinic	No	23/05/2017	0	0	0
2015299098	581	rural/clinic	No	23/05/2017	0	0	0
2013254805	581	rural/clinic	No	23/05/2017	0	0	0
2015369770	581	rural/clinic	No	23/05/2017	0	0	0
2015337508	581	rural/clinic	No	23/05/2017	0	0	0
2015369772	581	rural/clinic	No	23/05/2017	0	0	0
2015337507	581	rural/clinic	No	23/05/2017	0	0	0
2015369771	581	rural/clinic	No	23/05/2017	0	0	0
2015337620	581	rural/clinic	No	23/05/2017	0	0	0
2014374020	581	rural/clinic	No	23/05/2017	0	0	0
2014374021	581	rural/clinic	No	23/05/2017	0	0	0
2014355850	581	rural/clinic	No	23/05/2017	0	0	0
2014374018	581	rural/clinic	No	23/05/2017	0	0	0
2015326784	581	rural/clinic	No	23/05/2017	0	0	0
2014298240	581	rural/clinic	No	23/05/2017	0	0	0
2015404954	581	rural/clinic	No	23/05/2017	0	0	0
2015404955	581	rural/clinic	No	23/05/2017	0	0	0
2015404957	581	rural/clinic	No	23/05/2017	0	0	0
2015404956	581	rural/clinic	No	23/05/2017	0	0	0
2015412303	581	rural/clinic	No	23/05/2017	0	0	0
2015412304	581	rural/clinic	No	23/05/2017	0	0	0
2015412305	581	rural/clinic	No	23/05/2017	0	0	0
2014368849	581	rural/clinic	No	23/05/2017	0	0	0
2015297480	581	rural/clinic	No	23/05/2017	0	0	0
2015297481	581	rural/clinic	No	23/05/2017	0	0	0
2015297482	581	rural/clinic	No	23/05/2017	0	0	0
2015397925	581	rural/clinic	No	23/05/2017	0	0	0
2015397926	581	rural/clinic	No	23/05/2017	0	0	0
2015397927	581	rural/clinic	No	23/05/2017	0	0	0
2015397928	581	rural/clinic	No	23/05/2017	0	0	0
2015397929	581	rural/clinic	No	23/05/2017	0	0	0
2015377889	581	rural/clinic	No	23/05/2017	0	0	0
2015377890	581	rural/clinic	No	23/05/2017	0	0	0
2012269016	581	rural/clinic	No	23/05/2017	0	0	0
2012269017	581	rural/clinic	No	23/05/2017	0	0	0
2015383721	581	rural/clinic	No	23/05/2017	0	0	0
2015383722	581	rural/clinic	No	23/05/2017	0	0	0
2015383723	581	rural/clinic	No	23/05/2017	0	0	0
2015383724	581	rural/clinic	No	23/05/2017	0	0	0
2015383725	581	rural/clinic	No	23/05/2017	0	0	0
2015333104	581	rural/clinic	No	23/05/2017	0	0	0
2015333105	581	rural/clinic	No	23/05/2017	0	0	0
2015333106	581	rural/clinic	No	23/05/2017	0	0	0
2014318608	581	rural/clinic	No	23/05/2017	0	0	0
2015297334	581	rural/clinic	No	23/05/2017	0	0	0
2014342527	581	rural/clinic	No	23/05/2017	0	0	0
2015299100	581	rural/clinic	No	23/05/2017	0	0	0
2014342528	581	rural/clinic	No	23/05/2017	0	0	0
2013248192	581	rural/clinic	No	23/05/2017	0	0	0
2014342529	581	rural/clinic	No	23/05/2017	0	0	0
2014342526	581	rural/clinic	No	23/05/2017	0	0	0
2011216096	581	rural/clinic	No	23/05/2017	0	0	0
2012291355	581	rural/clinic	No	23/05/2017	0	0	0
2014347015	581	rural/clinic	No	23/05/2017	0	0	0
2014358215	581	rural/clinic	No	23/05/2017	0	0	0
2015368966	581	rural/clinic	No	23/05/2017	0	0	0
2014347013	581	rural/clinic	No	23/05/2017	0	0	0
2012291356	581	rural/clinic	No	23/05/2017	0	0	0
2015368967	581	rural/clinic	No	23/05/2017	0	0	0
2011226485	581	rural/clinic	No	23/05/2017	0	0	0
2015377838	581	rural/clinic	No	23/05/2017	0	0	0
2015287373	581	rural/clinic	No	23/05/2017	0	0	0
2015377319	581	rural/clinic	No	23/05/2017	0	0	0
2015369733	581	rural/clinic	No	23/05/2017	0	0	0
2015377173	581	rural/clinic	No	23/05/2017	0	0	0
2015369738	581	rural/clinic	No	23/05/2017	0	0	0
2015377174	581	rural/clinic	No	23/05/2017	0	0	0
2015414342	581	rural/clinic	No	23/05/2017	0	0	0
2012259888	581	rural/clinic	No	23/05/2017	0	0	0
2015369734	581	rural/clinic	No	23/05/2017	0	0	0
2012259889	581	rural/clinic	No	23/05/2017	0	0	0
2015414346	581	rural/clinic	No	23/05/2017	0	0	0
2015369735	581	rural/clinic	No	23/05/2017	0	0	0
2015340093	581	rural/clinic	No	23/05/2017	0	0	0
2015287374	581	rural/clinic	No	23/05/2017	0	0	0
2015414345	581	rural/clinic	No	23/05/2017	0	0	0
2015287372	581	rural/clinic	No	23/05/2017	0	0	0

2015414344	581	rural/clinic	No	23/05/2017	0	0	0
2014370435	581	rural/clinic	No	23/05/2017	0	0	0
2015340092	581	rural/clinic	No	23/05/2017	0	0	0
2012250093	581	rural/clinic	No	23/05/2017	0	0	0
2015414340	581	rural/clinic	No	23/05/2017	0	0	0
2015340091	581	rural/clinic	No	23/05/2017	0	0	0
2015318751	581	rural/clinic	No	23/05/2017	0	0	0
2015414343	581	rural/clinic	No	23/05/2017	0	0	0
2012306146	581	rural/clinic	No	23/05/2017	0	0	0
2014303094	581	rural/clinic	No	23/05/2017	0	0	0
2015302423	581	rural/clinic	No	23/05/2017	0	0	0
2015377837	581	rural/clinic	No	23/05/2017	0	0	0
2015287371	581	rural/clinic	No	23/05/2017	0	0	0
2014350099	581	rural/clinic	No	23/05/2017	0	0	0
2015414336	581	rural/clinic	No	23/05/2017	0	0	0
2015318752	581	rural/clinic	No	23/05/2017	0	0	0
2013271844	581	rural/clinic	No	23/05/2017	0	0	0
2014375602	581	rural/clinic	No	23/05/2017	0	0	0
2015302426	581	rural/clinic	No	23/05/2017	0	0	0
2011215295	581	rural/clinic	No	23/05/2017	0	0	0
2015340094	581	rural/clinic	No	23/05/2017	0	0	0
2015301815	581	rural/clinic	No	23/05/2017	0	0	0
2012250095	581	rural/clinic	No	23/05/2017	0	0	0
2015414341	581	rural/clinic	No	23/05/2017	0	0	0
2012250094	581	rural/clinic	No	23/05/2017	0	0	0
2015302428/D	581	rural/clinic	No	23/05/2017	0	0	0
2015414347	581	rural/clinic	No	23/05/2017	0	0	0
2012336627	581	rural/clinic	No	23/05/2017	0	0	0
2012336626	581	rural/clinic	No	23/05/2017	0	0	0
2015302427	581	rural/clinic	No	23/05/2017	0	0	0
2015369736	581	rural/clinic	No	23/05/2017	0	0	0
2015418860	581	rural/clinic	No	23/05/2017	0	0	0
2015414339	581	rural/clinic	No	23/05/2017	0	0	0
2015414337	581	rural/clinic	No	23/05/2017	0	0	0
2015339152	581	rural/clinic	No	23/05/2017	0	0	0
2015368815	581	rural/clinic	No	23/05/2017	0	0	0
2015369737	581	rural/clinic	No	23/05/2017	0	0	0
2015368814	581	rural/clinic	No	23/05/2017	0	0	0
2012244287	581	rural/clinic	No	23/05/2017	0	0	0
2015368964	581	rural/clinic	No	23/05/2017	0	0	0
198536wrong	581	rural/clinic	No	23/05/2017	0	0	0
2015368965	581	rural/clinic	No	23/05/2017	0	0	0
2013263598	581	rural/clinic	No	24/05/2017	0	0	0
2013263599	581	rural/clinic	No	24/05/2017	0	0	0
2013263600	581	rural/clinic	No	24/05/2017	0	0	0
2015403164	581	rural/clinic	No	24/05/2017	0	0	0
2015403165	581	rural/clinic	No	24/05/2017	0	0	0
2015403166	581	rural/clinic	No	24/05/2017	0	0	0
2015403167	581	rural/clinic	No	24/05/2017	0	0	0
2015403168	581	rural/clinic	No	24/05/2017	0	0	0
2015403169	581	rural/clinic	No	24/05/2017	0	0	0
2015403170	581	rural/clinic	No	24/05/2017	0	0	0
2015403171	581	rural/clinic	No	24/05/2017	0	0	0
2015403172	581	rural/clinic	No	24/05/2017	0	0	0
2015403173	581	rural/clinic	No	24/05/2017	0	0	0
2015403174	581	rural/clinic	No	24/05/2017	0	0	0
2015302425	581	rural/clinic	No	23/05/2017	0	0	0
2015302424	581	rural/clinic	No	23/05/2017	0	0	0
2011144819	581	rural/clinic	No	23/05/2017	0	0	0
2011144817	581	rural/clinic	No	23/05/2017	0	0	0
2011197678	581	rural/clinic	No	23/05/2017	0	0	0
2014344533	581	rural/clinic	No	23/05/2017	0	0	0
2015417054	581	rural/clinic	No	24/05/2017	0	0	0
2015417055	581	rural/clinic	No	24/05/2017	0	0	0
2015417056	581	rural/clinic	No	24/05/2017	0	0	0
2015417057	581	rural/clinic	No	24/05/2017	0	0	0
2014323740	581	rural/clinic	No	24/05/2017	0	0	0
2011186265	581	rural/clinic	No	24/05/2017	0	0	0
2014352865	581	rural/clinic	No	24/05/2017	0	0	0
2013262841	581	rural/clinic	No	24/05/2017	0	0	0
2011186266	581	rural/clinic	No	24/05/2017	0	0	0
2014349103	581	rural/clinic	No	24/05/2017	0	0	0
2015306925	581	rural/clinic	No	24/05/2017	0	0	0
2014291894	581	rural/clinic	No	25/05/2017	0	0	0
2014291895	581	rural/clinic	No	24/05/2017	0	0	0
2014291893	581	rural/clinic	No	24/05/2017	0	0	0
2015289175	581	rural/clinic	No	24/05/2017	0	0	0
2015289174	581	rural/clinic	No	24/05/2017	0	0	0
2014316051	581	rural/clinic	No	24/05/2017	0	0	0
2014316053	581	rural/clinic	No	24/05/2017	0	0	0
2014316052	581	rural/clinic	No	24/05/2017	0	0	0
2015372922	581	rural/clinic	No	23/05/2017	0	0	0

2015372924	581	rural/clinic	No	23/05/2017	0	0	0
2015372925	581	rural/clinic	No	23/05/2017	0	0	0
2015368707	581	rural/clinic	No	23/05/2017	0	0	0
2015332401	50	rural/clinic	No	23/05/2017	0	0	0
2015332401	50	rural/clinic	No	23/05/2017	0	0	0
2015418858	50	rural/clinic	No	23/05/2017	0	0	0
2015418858	50	rural/clinic	No	23/05/2017	0	0	0
2012323778	50	rural/clinic	No	23/05/2017	0	0	0
2012323778	50	rural/clinic	No	23/05/2017	0	0	0
2012388392	50	rural/clinic	No	23/05/2017	0	0	0
2012388392	50	rural/clinic	No	23/05/2017	0	0	0
2012344853	50	rural/clinic	No	23/05/2017	0	0	0
2012344853	50	rural/clinic	No	23/05/2017	0	0	0
2012388391	50	rural/clinic	No	23/05/2017	0	0	0
2012388391	50	rural/clinic	No	23/05/2017	0	0	0
2015315908	50	rural/clinic	No	23/05/2017	0	0	0
2015315908	50	rural/clinic	No	23/05/2017	0	0	0
2012336627	50	rural/clinic	No	23/05/2017	0	0	0
2012336627	50	rural/clinic	No	23/05/2017	0	0	0
2015414341	50	rural/clinic	No	23/05/2017	0	0	0
2014374021	50	rural/clinic	No	23/05/2017	0	0	0
2014374021	50	rural/clinic	No	23/05/2017	0	0	0
2015383722	50	rural/clinic	No	23/05/2017	0	0	0
2015383722	50	rural/clinic	No	23/05/2017	0	0	0
2015383725	50	rural/clinic	No	23/05/2017	0	0	0
2015383725	50	rural/clinic	No	23/05/2017	0	0	0
2014358215	50	rural/clinic	No	23/05/2017	0	0	0
2014358215	50	rural/clinic	No	23/05/2017	0	0	0
2015377838	50	rural/clinic	No	23/05/2017	0	0	0
2015377838	50	rural/clinic	No	23/05/2017	0	0	0
2015369734	50	rural/clinic	No	23/05/2017	0	0	0
2015369734	50	rural/clinic	No	23/05/2017	0	0	0
2015368585	50	rural/clinic	No	23/05/2017	0	0	0
2015368585	50	rural/clinic	No	23/05/2017	0	0	0
2015368586	50	rural/clinic	No	23/05/2017	0	0	0
2015368586	50	rural/clinic	No	23/05/2017	0	0	0
2012275986	50	rural/clinic	No	23/05/2017	0	0	0
2012275986	50	rural/clinic	No	23/05/2017	0	0	0
2014344532	50	rural/clinic	No	23/05/2017	0	0	0
2011144814	50	rural/clinic	No	23/05/2017	0	0	0
2011144816	50	rural/clinic	No	23/05/2017	0	0	0
2011144813	50	rural/clinic	No	23/05/2017	0	0	0
2015293476	50	rural/clinic	No	23/05/2017	0	0	0
2011144818	50	rural/clinic	No	23/05/2017	0	0	0
2014346730	50	rural/clinic	No	23/05/2017	0	0	0
2015368662	50	rural/clinic	No	23/05/2017	0	0	0
2012330883	50	rural/clinic	No	23/05/2017	0	0	0
2014338314	50	rural/clinic	No	23/05/2017	0	0	0
2015405412	50	rural/clinic	No	23/05/2017	0	0	0
2015405411	50	rural/clinic	No	23/05/2017	0	0	0
2014363227	50	rural/clinic	No	23/05/2017	0	0	0
2015363065	50	rural/clinic	No	23/05/2017	0	0	0
2015372921	50	rural/clinic	No	23/05/2017	0	0	0
2015372923	50	rural/clinic	No	23/05/2017	0	0	0
2015368709	50	rural/clinic	No	23/05/2017	0	0	0
2012242585	50	rural/clinic	No	23/05/2017	0	0	0
2015300699	50	rural/clinic	No	23/05/2017	0	0	0
2012243652	50	rural/clinic	No	23/05/2017	0	0	0
2015359601	50	rural/clinic	No	23/05/2017	0	0	0
2015359602	50	rural/clinic	No	23/05/2017	0	0	0
2015359603	50	rural/clinic	No	23/05/2017	0	0	0
2015359604	50	rural/clinic	No	23/05/2017	0	0	0
2015359605	50	rural/clinic	No	23/05/2017	0	0	0
2012305804	50	rural/clinic	No	23/05/2017	0	0	0
2012305805	50	rural/clinic	No	23/05/2017	0	0	0
2015342283	50	rural/clinic	No	23/05/2017	0	0	0
2015342284	50	rural/clinic	No	23/05/2017	0	0	0
2015342285	50	rural/clinic	No	23/05/2017	0	0	0
2015342286	50	rural/clinic	No	23/05/2017	0	0	0
2015342287	50	rural/clinic	No	23/05/2017	0	0	0
2015342288	50	rural/clinic	No	23/05/2017	0	0	0
2015324301	50	rural/clinic	No	23/05/2017	0	0	0
2015293943	50	rural/clinic	No	23/05/2017	0	0	0
2015293946	50	rural/clinic	No	23/05/2017	0	0	0
2011212185	50	rural/clinic	No	23/05/2017	0	0	0
2015334533	50	rural/clinic	No	23/05/2017	0	0	0
2014344468	50	rural/clinic	No	23/05/2017	0	0	0
2015303479	50	rural/clinic	No	23/05/2017	0	0	0
2015303825	50	rural/clinic	No	23/05/2017	0	0	0
2011141069	50	rural/clinic	No	23/05/2017	0	0	0
2015334532	50	rural/clinic	No	23/05/2017	0	0	0
2015303824	50	rural/clinic	No	23/05/2017	0	0	0

2015334412	50	rural/clinic	No	23/05/2017	0	0	0
2015334207	50	rural/clinic	No	23/05/2017	0	0	0
2011157095	50	rural/clinic	No	23/05/2017	0	0	0
2015303826	50	rural/clinic	No	23/05/2017	0	0	0
2015334534	50	rural/clinic	No	23/05/2017	0	0	0
2015334413	50	rural/clinic	No	23/05/2017	0	0	0
2015293948	50	rural/clinic	No	23/05/2017	0	0	0
2012265285	50	rural/clinic	No	23/05/2017	0	0	0
2012261203	50	rural/clinic	No	23/05/2017	0	0	0
2015334208	50	rural/clinic	No	23/05/2017	0	0	0
2014311369	50	rural/clinic	No	23/05/2017	0	0	0
2015293949	50	rural/clinic	No	23/05/2017	0	0	0
2015293947	50	rural/clinic	No	23/05/2017	0	0	0
2015293944	50	rural/clinic	No	23/05/2017	0	0	0
2015344325	50	rural/clinic	No	23/05/2017	0	0	0
2011195903	50	rural/clinic	No	29/05/2017	0	0	0
2011212598	638	rural/clinic	No	23/05/2017	0	0	0
2015368708	638	rural/clinic	No	23/05/2017	0	0	0
2011144815	638	rural/clinic	No	23/05/2017	0	0	0
2012339734	638	rural/clinic	No	23/05/2017	0	0	0
2014367624	638	rural/clinic	No	23/05/2017	0	0	0
2015414338	638	rural/clinic	No	23/05/2017	0	0	0
2015377891	638	rural/clinic	No	23/05/2017	0	0	0
2012318890	638	rural/clinic	No	23/05/2017	0	0	0
2015378302	638	rural/clinic	No	23/05/2017	0	0	0
2015378301	638	rural/clinic	No	04/05/2017	0	0	0
2011212597	638	rural/clinic	No	23/05/2017	0	0	0
2015299099	638	rural/clinic	No	23/05/2017	0	0	0
2013256268	638	rural/clinic	No	23/05/2017	0	0	0
2012318891	638	rural/clinic	No	23/05/2017	0	0	0
2011135667	638	rural/clinic	No	29/05/2017	0	0	0
2015301814	638	rural/clinic	No	23/05/2017	0	0	0
2011142546	638	rural/clinic	No	19/04/2017	0	0	0
2015363015	638	rural/clinic	No	29/05/2017	0	0	0
2015286644	638	rural/clinic	No	29/05/2017	0	0	0
2015286640	638	rural/clinic	No	29/05/2017	0	0	0
2014334178	638	rural/clinic	No	29/05/2017	0	0	0
2014338504	638	rural/clinic	No	29/05/2017	0	0	0
2015378732	638	rural/clinic	No	29/05/2017	0	0	0
2014344534	638	rural/clinic	No	29/05/2017	0	0	0
2014338505	638	rural/clinic	No	29/05/2017	0	0	0
2014338507	721	district/faith-based	No	29/05/2017	1	0	0
2015372805	721	district/faith-based	No	29/05/2017	1	0	0
2015372806	721	district/faith-based	No	29/05/2017	1	0	0
2015372807	721	district/faith-based	No	29/05/2017	1	0	0
2014338508	721	district/faith-based	No	29/05/2017	1	0	0
2015403286	721	district/faith-based	No	29/05/2017	1	0	0
2014307544	721	district/faith-based	No	29/05/2017	1	0	0
2011136107	721	district/faith-based	No	29/05/2017	1	0	0
2015397627	721	district/faith-based	No	29/05/2017	1	0	0
2011136114	721	district/faith-based	No	29/05/2017	1	0	0
2011136115	721	district/faith-based	No	29/05/2017	1	0	0
2015306487	721	district/faith-based	No	24/05/2017	1	0	0
2015416522	721	district/faith-based	No	29/05/2017	1	0	0
2015328534	721	district/faith-based	No	29/05/2017	1	0	0
2015344891	721	district/faith-based	No	29/05/2017	1	0	0
2015397628	721	district/faith-based	No	28/05/2017	1	0	0
2015397629	721	district/faith-based	No	29/05/2017	1	0	0
2015344894	721	district/faith-based	No	29/05/2017	1	0	0
2015397630	721	district/faith-based	No	29/05/2017	1	0	0
2015397632	721	district/faith-based	No	29/05/2017	1	0	0
2014335567	721	district/faith-based	No	29/05/2017	1	0	0
2015397631	721	district/faith-based	No	29/05/2017	1	0	0
2014335570	721	district/faith-based	No	29/05/2017	1	0	0
2015397633	721	district/faith-based	No	29/05/2017	1	0	0
2015355742	721	district/faith-based	No	29/05/2017	1	0	0
2015397634	721	district/faith-based	No	29/05/2017	1	0	0
2015397635	721	district/faith-based	No	29/05/2017	1	0	0
2015326805	721	district/faith-based	No	29/05/2017	1	0	0
2015355743	721	district/faith-based	No	29/05/2017	1	0	0
2015355744	721	district/faith-based	No	29/05/2017	1	0	0
2015326806	721	district/faith-based	No	29/05/2017	1	0	0
2015334935	721	district/faith-based	No	29/05/2017	1	0	0
2015335964	721	district/faith-based	No	29/05/2017	1	0	0
2015334936	721	district/faith-based	No	29/05/2017	1	0	0
2015344089	721	district/faith-based	No	29/05/2017	1	0	0
2015335963	721	district/faith-based	No	25/05/2017	1	0	0
2015294877	721	district/faith-based	No	29/05/2017	1	0	0
2015344090	721	district/faith-based	No	29/05/2017	1	0	0
2015335446	721	district/faith-based	No	29/05/2017	1	0	0
2012317318	721	district/faith-based	No	29/05/2017	1	0	0
2015355781	721	district/faith-based	No	29/05/2017	1	0	0

2015362425	721	district/faith-based	No	29/05/2017	1	0	0
2015294876	721	district/faith-based	No	29/05/2017	1	0	0
2015362428	721	district/faith-based	No	29/05/2017	1	0	0
2012321335	721	district/faith-based	No	29/05/2017	1	0	0
2015294878	721	district/faith-based	No	29/05/2017	1	0	0
2012321336	721	district/faith-based	No	29/05/2017	1	0	0
2013265067	721	district/faith-based	No	29/05/2017	1	0	0
2012321337	721	district/faith-based	No	29/05/2017	1	0	0
2011136113	721	district/faith-based	No	29/05/2017	1	0	0
2014335565	721	district/faith-based	No	29/05/2017	1	0	0
2011148066	721	district/faith-based	No	29/05/2017	1	0	0
2015289330	721	district/faith-based	No	29/05/2017	1	0	0
2015355780	721	district/faith-based	No	29/05/2017	1	0	0
2015394645	721	district/faith-based	No	29/05/2017	1	0	0
2015394646	721	district/faith-based	No	29/05/2017	1	0	0
2015335589	721	district/faith-based	No	29/05/2017	1	0	0
2015394647	721	district/faith-based	No	29/05/2017	1	0	0
2015394648	721	district/faith-based	No	29/05/2017	1	0	0
2015362486	721	district/faith-based	No	29/05/2017	1	0	0
2015403283	721	district/faith-based	No	29/05/2017	1	0	0
2015362485	721	district/faith-based	No	29/05/2017	1	0	0
2015403284	721	district/faith-based	No	29/05/2017	1	0	0
2012261449	721	district/faith-based	No	29/05/2017	1	0	0
2015403285	721	district/faith-based	No	29/05/2017	1	0	0
2015337976	721	district/faith-based	No	29/05/2017	1	0	0
2015403287	721	district/faith-based	No	29/05/2017	1	0	0
2015337977	721	district/faith-based	No	29/05/2017	1	0	0
2015403289	721	district/faith-based	No	29/05/2017	1	0	0
2015337978	721	district/faith-based	No	29/05/2017	1	0	0
2011139402	721	district/faith-based	No	29/05/2017	1	0	0
2015344733	721	district/faith-based	No	29/05/2017	1	0	0
2015377325	721	district/faith-based	No	24/05/2017	1	0	0
2015377325	721	district/faith-based	No	24/05/2017	1	0	0
2015302428/D	721	district/faith-based	No	23/05/2017	1	0	0
2015302428/D	721	district/faith-based	No	23/05/2017	1	0	0
2015403171	721	district/faith-based	No	24/05/2017	1	0	0
2015403171	721	district/faith-based	No	24/05/2017	1	0	0
2014291894	721	district/faith-based	No	25/05/2017	1	0	0
2014291894	721	district/faith-based	No	25/05/2017	1	0	0
2014291893	721	district/faith-based	No	24/05/2017	1	0	0
2014291893	721	district/faith-based	No	24/05/2017	1	0	0
2011144816	721	district/faith-based	No	23/05/2017	1	0	0
2011144816	721	district/faith-based	No	23/05/2017	1	0	0
2015368971	721	district/faith-based	No	23/05/2017	1	0	0
2015289176	721	district/faith-based	No	23/05/2017	1	0	0
2015287374	721	district/faith-based	No	23/05/2017	1	0	0
2015287374	721	district/faith-based	No	23/05/2017	1	0	0
2015344735	721	district/faith-based	No	29/05/2017	1	0	0
2015344736	721	district/faith-based	No	29/05/2017	1	0	0
2015344737	721	district/faith-based	No	29/05/2017	1	0	0
2015344889	721	district/faith-based	No	29/05/2017	1	0	0
2015344890	721	district/faith-based	No	29/05/2017	1	0	0
2014335562	721	district/faith-based	No	29/05/2017	1	0	0
2014335563	721	district/faith-based	No	29/05/2017	1	0	0
2014327679	721	district/faith-based	No	29/05/2017	1	0	0
2014327680	721	district/faith-based	No	29/05/2017	1	0	0
2014327681	721	district/faith-based	No	29/05/2017	1	0	0
2014327510	721	district/faith-based	No	29/05/2017	1	0	0
2015413595	721	district/faith-based	No	29/05/2017	1	0	0
2015342285	721	district/faith-based	No	23/05/2017	1	0	0
2015342285	721	district/faith-based	No	23/05/2017	1	0	0
2015342288	721	district/faith-based	No	23/05/2017	1	0	0
2015342288	721	district/faith-based	No	23/05/2017	1	0	0
2015324301	721	district/faith-based	No	23/05/2017	1	0	0
2015293946	721	district/faith-based	No	23/05/2017	1	0	0
2015293946	721	district/faith-based	No	23/05/2017	1	0	0
2012259877	721	district/faith-based	No	04/05/2017	1	0	0
2011157147	721	district/faith-based	No	10/05/2017	1	0	0
2012370168	721	district/faith-based	No	17/03/2017	1	0	0
2014287177	721	district/faith-based	No	24/04/2017	1	0	0
2014287178	721	district/faith-based	No	30/05/2017	1	0	0
2015362520	721	district/faith-based	No	30/05/2017	1	0	0
2015362517	721	district/faith-based	No	30/05/2017	1	0	0
2015415270	721	district/faith-based	No	30/05/2017	1	0	0
2015384045	721	district/faith-based	No	30/05/2017	1	0	0
2015340702	721	district/faith-based	No	30/05/2017	1	0	0
2012317557	721	district/faith-based	No	30/05/2017	1	0	0
2015384043	721	district/faith-based	No	30/05/2017	1	0	0
2015384044	721	district/faith-based	No	30/05/2017	1	0	0
2012317556	721	district/faith-based	No	30/05/2017	1	0	0
2015406273	721	district/faith-based	No	30/05/2017	1	0	0
2015406274	721	district/faith-based	No	30/05/2017	1	0	0

2015406500	721	district/faith-based	No	30/05/2017	1	0	0
2015331947	721	district/faith-based	No	30/05/2017	1	0	0
2012305588	721	district/faith-based	No	30/05/2017	1	0	0
2012376595	721	district/faith-based	No	30/05/2017	1	0	0
2015297268	721	district/faith-based	No	30/05/2017	1	0	0
2015297269	721	district/faith-based	No	30/05/2017	1	0	0
2011229220	721	district/faith-based	No	30/05/2017	1	0	0
2014302850	721	district/faith-based	No	30/05/2017	1	0	0
2014302849	721	district/faith-based	No	30/05/2017	1	0	0
2015301268	721	district/faith-based	No	30/05/2017	1	0	0
2011229221	721	district/faith-based	No	30/05/2017	1	0	0
2014319090	721	district/faith-based	No	30/05/2017	1	0	0
2014319089	721	district/faith-based	No	30/05/2017	1	0	0
2011199089	721	district/faith-based	No	30/05/2017	1	0	0
2011199088	721	district/faith-based	No	30/05/2017	1	0	0
2015362519	721	district/faith-based	No	30/05/2017	1	0	0
2011225371	721	district/faith-based	No	30/05/2017	1	0	0
2014302944	721	district/faith-based	No	30/05/2017	1	0	0
2015362521	721	district/faith-based	No	30/05/2017	1	0	0
2015397633	721	district/faith-based	No	29/05/2017	1	0	0
2015397633	721	district/faith-based	No	29/05/2017	1	0	0
2015325044	721	district/faith-based	No	30/05/2017	1	0	0
2015368973	721	district/faith-based	No	30/05/2017	1	0	0
2014307544	721	district/faith-based	No	29/05/2017	1	0	0
2011136114	721	district/faith-based	No	29/05/2017	1	0	0
2015289330	721	district/faith-based	No	29/05/2017	1	0	0
2015289330	721	district/faith-based	No	29/05/2017	1	0	0
2015337977	721	district/faith-based	No	29/05/2017	1	0	0
2015344733	721	district/faith-based	No	29/05/2017	1	0	0
2015368974	721	district/faith-based	No	30/05/2017	1	0	0
2015325045	721	district/faith-based	No	30/05/2017	1	0	0
2015289176	721	district/faith-based	No	23/05/2017	1	0	0
2015340983	721	district/faith-based	No	30/05/2017	1	0	0
2015349536	721	district/faith-based	No	30/05/2017	1	0	0
2015340984	721	district/faith-based	No	30/05/2017	1	0	0
2015349535	721	district/faith-based	No	30/05/2017	1	0	0
2015340985	721	district/faith-based	No	30/05/2017	1	0	0
2015325043	721	district/faith-based	No	30/05/2017	1	0	0
2015358541	721	district/faith-based	No	30/05/2017	1	0	0
2015358540	721	district/faith-based	No	30/05/2017	1	0	0
2015358543	721	district/faith-based	No	30/05/2017	1	0	0
2011117495	721	district/faith-based	No	30/05/2017	1	0	0
2015358544	721	district/faith-based	No	30/05/2017	1	0	0
2015358542	721	district/faith-based	No	30/05/2017	1	0	0
2015319251	721	district/faith-based	No	30/05/2017	1	0	0
2012370982	721	district/faith-based	No	30/05/2017	1	0	0
2015351698	721	district/faith-based	No	30/05/2017	1	0	0
2012294063	721	district/faith-based	No	30/05/2017	1	0	0
2013285125	721	district/faith-based	No	30/05/2017	1	0	0
2015351699	721	district/faith-based	No	30/05/2017	1	0	0
2011136108	721	district/faith-based	No	30/05/2017	1	0	0
2014307549	721	district/faith-based	No	30/05/2017	1	0	0
2014307550	721	district/faith-based	No	30/05/2017	1	0	0
2011136110	721	district/faith-based	No	30/05/2017	1	0	0
2011136109	721	district/faith-based	No	30/05/2017	1	0	0
2012294062	721	district/faith-based	No	30/05/2017	1	0	0
2015351700	721	district/faith-based	No	30/05/2017	1	0	0
2012294061	721	district/faith-based	No	30/05/2017	1	0	0
2014350459	721	district/faith-based	No	30/05/2017	1	0	0
2015337621	721	district/faith-based	No	30/05/2017	1	0	0
2014307333	721	district/faith-based	No	30/05/2017	1	0	0
2014307332	721	district/faith-based	No	30/05/2017	1	0	0
2014307331	721	district/faith-based	No	30/05/2017	1	0	0
2014378733	721	district/faith-based	No	30/05/2017	1	0	0
2014317345	721	district/faith-based	No	30/05/2017	1	0	0
2015314602	721	district/faith-based	No	30/05/2017	1	0	0
2014351229	721	district/faith-based	No	30/05/2017	1	0	0
2012352829	721	district/faith-based	No	30/05/2017	1	0	0
2015337509	721	district/faith-based	No	30/05/2017	1	0	0
2015340212	721	district/faith-based	No	30/05/2017	1	0	0
2012388393	721	district/faith-based	No	30/05/2017	1	0	0
2014320528	721	district/faith-based	No	30/05/2017	1	0	0
2012252693	721	district/faith-based	No	30/05/2017	1	0	0
2010099918	721	district/faith-based	No	30/05/2017	1	0	0
2010099919	721	district/faith-based	No	30/05/2017	1	0	0
2014290729	721	district/faith-based	No	30/05/2017	1	0	0
2012374293	721	district/faith-based	No	30/05/2017	1	0	0
2012374295	721	district/faith-based	No	30/05/2017	1	0	0
2012374294	721	district/faith-based	No	30/05/2017	1	0	0
2015324445	721	district/faith-based	No	30/05/2017	1	0	0
2015324446	721	district/faith-based	No	30/05/2017	1	0	0
2015315910	721	district/faith-based	No	30/05/2017	1	0	0

2015315911	721	district/faith-based	No	30/05/2017	1	0	0
2015324037	721	district/faith-based	No	30/05/2017	1	0	0
2014385617	721	district/faith-based	No	30/05/2017	1	0	0
2012283597	721	district/faith-based	No	30/05/2017	1	0	0
2014350458	721	district/faith-based	No	30/05/2017	1	0	0
2014347989	721	district/faith-based	No	30/05/2017	1	0	0
2014350460	721	district/faith-based	No	30/05/2017	1	0	0
2012370167	721	district/faith-based	No	30/05/2017	1	0	0
2012268533	721	district/faith-based	No	30/05/2017	1	0	0
2014290727	721	district/faith-based	No	30/05/2017	1	0	0
2014357823	721	district/faith-based	No	30/05/2017	1	0	0
2014290730	721	district/faith-based	No	30/05/2017	1	0	0
2014312408	721	district/faith-based	No	30/05/2017	1	0	0
2014290728	721	district/faith-based	No	30/05/2017	1	0	0
2015409015	721	district/faith-based	No	30/05/2017	1	0	0
2015409012	721	district/faith-based	No	30/05/2017	1	0	0
2014290732	721	district/faith-based	No	30/05/2017	1	0	0
2014371398	721	district/faith-based	No	30/05/2017	1	0	0
2014290731	721	district/faith-based	No	30/05/2017	1	0	0
2014357822	721	district/faith-based	No	30/05/2017	1	0	0
2015332453	721	district/faith-based	No	30/05/2017	1	0	0
2015409013	721	district/faith-based	No	30/05/2017	1	0	0
2015342313	721	district/faith-based	No	30/05/2017	1	0	0
2015332464	721	district/faith-based	No	30/05/2017	1	0	0
2015339757	721	district/faith-based	No	30/05/2017	1	0	0
2014357821	721	district/faith-based	No	30/05/2017	1	0	0
2012336628	721	district/faith-based	No	30/05/2017	1	0	0
2015339756	721	district/faith-based	No	30/05/2017	1	0	0
2015409020	721	district/faith-based	No	30/05/2017	1	0	0
2012336629	721	district/faith-based	No	30/05/2017	1	0	0
2015339758	721	district/faith-based	No	30/05/2017	1	0	0
2015339759	721	district/faith-based	No	30/05/2017	1	0	0
2015409019	721	district/faith-based	No	30/05/2017	1	0	0
2014360968	721	district/faith-based	No	30/05/2017	1	0	0
2015385531	721	district/faith-based	No	30/05/2017	1	0	0
2015362518	721	district/faith-based	No	30/05/2017	1	0	0
2014360969	721	district/faith-based	No	30/05/2017	1	0	0
2015372023	721	district/faith-based	No	30/05/2017	1	0	0
2014357323	721	district/faith-based	No	30/05/2017	1	0	0
2015402627	721	district/faith-based	No	30/05/2017	1	0	0
2015409014	721	district/faith-based	No	30/05/2017	1	0	0
2015402628	721	district/faith-based	No	30/05/2017	1	0	0
2015409016	721	district/faith-based	No	30/05/2017	1	0	0
2015409017	721	district/faith-based	No	30/05/2017	1	0	0
2015402629	721	district/faith-based	No	30/05/2017	1	0	0
2015409018	721	district/faith-based	No	30/05/2017	1	0	0
2015335447	721	district/faith-based	No	30/05/2017	1	0	0
2015409011	721	district/faith-based	No	30/05/2017	1	0	0
2014358009	721	district/faith-based	No	30/05/2017	1	0	0
2014358010	721	district/faith-based	No	30/05/2017	1	0	0
2014358011	721	district/faith-based	No	30/05/2017	1	0	0
2014358012	721	district/faith-based	No	30/05/2017	1	0	0
2014358007	721	district/faith-based	No	30/05/2017	1	0	0
2014358013	721	district/faith-based	No	30/05/2017	1	0	0
2015362494	721	district/faith-based	No	30/05/2017	1	0	0
2014314654	721	district/faith-based	No	30/05/2017	1	0	0
2015294879	721	district/faith-based	No	30/05/2017	1	0	0
2015362497	721	district/faith-based	No	30/05/2017	1	0	0
2015368710	721	district/faith-based	No	30/05/2017	1	0	0
2015368713	721	district/faith-based	No	30/05/2017	1	0	0
2015289331	721	district/faith-based	No	30/05/2017	1	0	0
2015368712	721	district/faith-based	No	30/05/2017	1	0	0
2015368711	721	district/faith-based	No	30/05/2017	1	0	0
2015355745	721	district/faith-based	No	30/05/2017	1	0	0
2012314021	721	district/faith-based	No	30/05/2017	1	0	0
2015362496	721	district/faith-based	No	30/05/2017	1	0	0
2015355005	721	district/faith-based	No	30/05/2017	1	0	0
2015313203	721	district/faith-based	No	30/05/2017	1	0	0
2014302521	721	district/faith-based	No	31/05/2017	1	0	0
2015313202	721	district/faith-based	No	30/05/2017	1	0	0
2012344500	721	district/faith-based	No	31/05/2017	1	0	0
2013253028	721	district/faith-based	No	30/05/2017	1	0	0
2013256271	721	district/faith-based	No	30/05/2017	1	0	0
2014385691	721	district/faith-based	No	30/05/2017	1	0	0
2015301481	721	district/faith-based	No	31/05/2017	1	0	0
2015407554	721	district/faith-based	No	30/05/2017	1	0	0
2015326588	721	district/faith-based	No	30/05/2017	1	0	0
2015301482	721	district/faith-based	No	31/05/2017	1	0	0
2015326582	721	district/faith-based	No	30/05/2017	1	0	0
2014301893	721	district/faith-based	No	30/05/2017	1	0	0
2015331777	721	district/faith-based	No	31/05/2017	1	0	0
2015326583	721	district/faith-based	No	30/05/2017	1	0	0

2015386701	721	district/faith-based	No	30/05/2017	1	0	0
2012253133	721	district/faith-based	No	31/05/2017	1	0	0
2015326584	721	district/faith-based	No	30/05/2017	1	0	0
2015402630	721	district/faith-based	No	30/05/2017	1	0	0
2012253132	721	district/faith-based	No	31/05/2017	1	0	0
2015326585	721	district/faith-based	No	30/05/2017	1	0	0
2015359762	721	district/faith-based	No	31/05/2017	1	0	0
2015326586	721	district/faith-based	No	30/05/2017	1	0	0
2015402624	721	district/faith-based	No	30/05/2017	1	0	0
2015386702	721	district/faith-based	No	30/05/2017	1	0	0
2015326587	721	district/faith-based	No	30/05/2017	1	0	0
2012253445	721	district/faith-based	No	31/05/2017	1	0	0
2015402625	721	district/faith-based	No	30/05/2017	1	0	0
2014317346	721	district/faith-based	No	30/05/2017	1	0	0
2012253444	721	district/faith-based	No	31/05/2017	1	0	0
2015386703	721	district/faith-based	No	30/05/2017	1	0	0
2015402626	721	district/faith-based	No	30/05/2017	1	0	0
2014358338	721	district/faith-based	No	30/05/2017	1	0	0
2014358337	721	district/faith-based	No	30/05/2017	1	0	0
2015361606	721	district/faith-based	No	31/05/2017	1	0	0
2015415057	721	district/faith-based	No	30/05/2017	1	0	0
2015386704	721	district/faith-based	No	30/05/2017	1	0	0
2015359761	721	district/faith-based	No	31/05/2017	1	0	0
2015310014	721	district/faith-based	No	31/05/2017	1	0	0
2015386705	721	district/faith-based	No	30/05/2017	1	0	0
2015414459	721	district/faith-based	No	30/05/2017	1	0	0
2014348449	721	district/faith-based	No	31/05/2017	1	0	0
2012295985	721	district/faith-based	No	30/05/2017	1	0	0
2013262629	721	district/faith-based	No	31/05/2017	1	0	0
2012295986	721	district/faith-based	No	30/05/2017	1	0	0
2015369185	721	district/faith-based	No	30/05/2017	1	0	0
2013262628	721	district/faith-based	No	31/05/2017	1	0	0
2012295987	721	district/faith-based	No	30/05/2017	1	0	0
2015369186	721	district/faith-based	No	30/05/2017	1	0	0
2015335125	721	district/faith-based	No	31/05/2017	1	0	0
2015335126	721	district/faith-based	No	31/05/2017	1	0	0
2012316550	721	district/faith-based	No	31/05/2017	1	0	0
2015414736	721	district/faith-based	No	30/05/2017	1	0	0
2015335127	721	district/faith-based	No	31/05/2017	1	0	0
2015369187	721	district/faith-based	No	30/05/2017	1	0	0
2015335128	721	district/faith-based	No	31/05/2017	1	0	0
2014297972	721	district/faith-based	No	30/05/2017	1	0	0
2015384543	721	district/faith-based	No	31/05/2017	1	0	0
2015369188	721	district/faith-based	No	30/05/2017	1	0	0
2015384545	721	district/faith-based	No	31/05/2017	1	0	0
2014323741	617	rural/clinic	No	31/05/2017	0	0	0
2015369927	617	rural/clinic	No	30/05/2017	0	0	0
2015335590	617	rural/clinic	No	31/05/2017	0	0	0
2015362263	617	rural/clinic	No	30/05/2017	0	0	0
2014348889	617	rural/clinic	No	31/05/2017	0	0	0
2015362498	617	rural/clinic	No	31/05/2017	0	0	0
2015369741	617	rural/clinic	No	30/05/2017	0	0	0
2014349084	617	rural/clinic	No	31/05/2017	0	0	0
2015362264	617	rural/clinic	No	30/05/2017	0	0	0
2015369740	617	rural/clinic	No	30/05/2017	0	0	0
2014353069	617	rural/clinic	No	31/05/2017	0	0	0
2015356242	617	rural/clinic	No	31/05/2017	0	0	0
2012295145	617	rural/clinic	No	31/05/2017	0	0	0
2015362266	617	rural/clinic	No	30/05/2017	0	0	0
2015361450	617	rural/clinic	No	30/05/2017	0	0	0
2015356243	617	rural/clinic	No	31/05/2017	0	0	0
2015402392	617	rural/clinic	No	30/05/2017	0	0	0
2014314472	617	rural/clinic	No	31/05/2017	0	0	0
2015361444	617	rural/clinic	No	30/05/2017	0	0	0
2015402393	617	rural/clinic	No	30/05/2017	0	0	0
2014324375	617	rural/clinic	No	31/05/2017	0	0	0
2014359732	617	rural/clinic	No	31/05/2017	0	0	0
2015402394	206	rural/clinic	No	30/05/2017	0	0	0
2014324376	206	rural/clinic	No	31/05/2017	0	0	0
2015361440	206	rural/clinic	No	30/05/2017	0	0	0
2015402395	206	rural/clinic	No	30/05/2017	0	0	0
2015337979	463	rural/clinic	No	31/05/2017	0	0	0
2015361441	463	rural/clinic	No	30/05/2017	0	0	0
2015402396	463	rural/clinic	No	30/05/2017	0	0	0
2014324377	463	rural/clinic	No	31/05/2017	0	0	0
2015335965	463	rural/clinic	No	31/05/2017	0	0	0
2015402397	463	rural/clinic	No	30/05/2017	0	0	0
2015361445	463	rural/clinic	No	30/05/2017	0	0	0
2015301480	463	rural/clinic	No	31/05/2017	0	0	0
2014363592	463	rural/clinic	No	31/05/2017	0	0	0
2014378272	463	rural/clinic	No	30/05/2017	0	0	0
2015337981	463	rural/clinic	No	31/05/2017	0	0	0

2012323662	463	rural/clinic	No	30/05/2017	0	0	0
2015361442	463	rural/clinic	No	30/05/2017	0	0	0
2014313664	463	rural/clinic	No	31/05/2017	0	0	0
2011152132	463	rural/clinic	No	30/05/2017	0	0	0
2014365387	463	rural/clinic	No	30/05/2017	0	0	0
2015362495	463	rural/clinic	No	31/05/2017	0	0	0
2012305806	463	rural/clinic	No	31/05/2017	0	0	0
2014365386	463	rural/clinic	No	30/05/2017	0	0	0
2015337980	463	rural/clinic	No	31/05/2017	0	0	0
2014313666	463	rural/clinic	No	31/05/2017	0	0	0
2014365474	463	rural/clinic	No	30/05/2017	0	0	0
2014313662	463	rural/clinic	No	31/05/2017	0	0	0
2014374019	463	rural/clinic	No	30/05/2017	0	0	0
2015313205	463	rural/clinic	No	30/05/2017	0	0	0
2015369773	463	rural/clinic	No	30/05/2017	0	0	0
2015313204	463	rural/clinic	No	30/05/2017	0	0	0
2015369774	463	rural/clinic	No	30/05/2017	0	0	0
2014313660	463	rural/clinic	No	31/05/2017	0	0	0
2014358216	463	rural/clinic	No	30/05/2017	0	0	0
2015313211	463	rural/clinic	No	30/05/2017	0	0	0
2014313661	463	rural/clinic	No	31/05/2017	0	0	0
2015313201	463	rural/clinic	No	30/05/2017	0	0	0
2012295994	463	rural/clinic	No	31/05/2017	0	0	0
2015361446	463	rural/clinic	No	30/05/2017	0	0	0
2012291358	463	rural/clinic	No	30/05/2017	0	0	0
2015313343	463	rural/clinic	No	30/05/2017	0	0	0
2012295992	463	rural/clinic	No	31/05/2017	0	0	0
2015363305	463	rural/clinic	No	31/05/2017	0	0	0
2012291357	463	rural/clinic	No	30/05/2017	0	0	0
2015355006	463	rural/clinic	No	31/05/2017	0	0	0
2015361449	463	rural/clinic	No	30/05/2017	0	0	0
2012389493/d	463	rural/clinic	No	05/05/2017	0	0	0
2015361448	463	rural/clinic	No	30/05/2017	0	0	0
2015313206	463	rural/clinic	No	30/05/2017	0	0	0
201701	463	rural/clinic	No	31/05/2017	0	0	0
2015313208	463	rural/clinic	No	30/05/2017	0	0	0
2015361447	463	rural/clinic	No	30/05/2017	0	0	0
2015289850	463	rural/clinic	No	31/05/2017	0	0	0
2015313210	425	rural/clinic	No	30/05/2017	0	0	0
201702	425	rural/clinic	No	31/05/2017	0	0	0
2015361443	425	rural/clinic	No	30/05/2017	0	0	0
2015313209	425	rural/clinic	No	30/05/2017	0	0	0
2015354555	425	rural/clinic	No	31/05/2017	0	0	0
2015313213	425	rural/clinic	No	30/05/2017	0	0	0
2015354554	425	rural/clinic	No	31/05/2017	0	0	0
2012291359	425	rural/clinic	No	30/05/2017	0	0	0
2012388390	425	rural/clinic	No	31/05/2017	0	0	0
2012330885	425	rural/clinic	No	30/05/2017	0	0	0
2014342530	425	rural/clinic	No	30/05/2017	0	0	0
2015293364	425	rural/clinic	No	31/05/2017	0	0	0
2014342531	425	rural/clinic	No	30/05/2017	0	0	0
2015321401	425	rural/clinic	No	30/05/2017	0	0	0
2015313214	425	rural/clinic	No	30/05/2017	0	0	0
2012295988	425	rural/clinic	No	30/05/2017	0	0	0
2015313215	425	rural/clinic	No	30/05/2017	0	0	0
2012295989	425	rural/clinic	No	30/05/2017	0	0	0
2015313212	425	rural/clinic	No	30/05/2017	0	0	0
2015413359	425	rural/clinic	No	30/05/2017	0	0	0
2015338465	425	rural/clinic	No	30/05/2017	0	0	0
2015368790	425	rural/clinic	No	30/05/2017	0	0	0
2012295990	425	rural/clinic	No	30/05/2017	0	0	0
2015368789	425	rural/clinic	No	30/05/2017	0	0	0
2012268783	425	rural/clinic	No	30/05/2017	0	0	0
2015413361	926	rural/clinic	No	30/05/2017	0	0	0
2015413911	926	rural/clinic	No	30/05/2017	0	0	0
2015413914	926	rural/clinic	No	30/05/2017	0	0	0
2012295991	926	rural/clinic	No	30/05/2017	0	0	0
2015413913	926	rural/clinic	No	30/05/2017	0	0	0
2011193484	926	rural/clinic	No	30/05/2017	0	0	0
2011193487	926	rural/clinic	No	30/05/2017	0	0	0
2015378734	926	rural/clinic	No	30/05/2017	0	0	0
2011193486	926	rural/clinic	No	30/05/2017	0	0	0
2015378733	926	rural/clinic	No	30/05/2017	0	0	0
2011193485	926	rural/clinic	No	30/05/2017	0	0	0
2015288604	926	rural/clinic	No	30/05/2017	0	0	0
2015289177	926	rural/clinic	No	30/05/2017	0	0	0
2015333259	926	rural/clinic	No	30/05/2017	0	0	0
2013274249	926	rural/clinic	No	30/05/2017	0	0	0
2015307403	926	rural/clinic	No	30/05/2017	0	0	0
2015333958	926	rural/clinic	No	30/05/2017	0	0	0
2015368291	926	rural/clinic	No	30/05/2017	0	0	0
2015368288	926	rural/clinic	No	30/05/2017	0	0	0

2015368290	926	rural/clinic	No	30/05/2017	0	0	0
2015288582	926	rural/clinic	No	30/05/2017	0	0	0
2015368289	926	rural/clinic	No	30/05/2017	0	0	0
2015307404	926	rural/clinic	No	30/05/2017	0	0	0
2014305221	582	rural/clinic	No	30/05/2017	0	0	0
2012259765	582	rural/clinic	No	11/05/2017	0	0	0
2015333961	582	rural/clinic	No	30/05/2017	0	0	0
2015333960	582	rural/clinic	No	30/05/2017	0	0	0
2011143694	582	rural/clinic	No	30/05/2017	0	0	0
2015415910	582	rural/clinic	No	04/05/2017	0	0	0
2015286310	582	rural/clinic	No	30/05/2017	0	0	0
2011293844	582	rural/clinic	No	04/05/2017	0	0	0
2015541591	582	rural/clinic	No	04/05/2017	0	0	0
2015415262	582	rural/clinic	No	04/05/2017	0	0	0
2015286309	582	rural/clinic	No	30/05/2017	0	0	0
2015286708	695	rural/clinic	No	30/05/2017	0	0	0
2014319725	695	rural/clinic	No	30/05/2017	0	0	0
2013267895	695	rural/clinic	No	30/05/2017	0	0	0
2014378487	695	rural/clinic	No	30/05/2017	0	0	0
2015408113	695	rural/clinic	No	30/05/2017	0	0	0
201703	695	rural/clinic	No	11/05/2017	0	0	0
2015415271	695	rural/clinic	No	30/05/2017	0	0	0
201704	362	district/faith-based	No	11/05/2017	1	0	0
2014357740	362	district/faith-based	No	30/05/2017	1	0	0
2015378201	362	district/faith-based	No	30/05/2017	1	0	0
2011203830/D	362	district/faith-based	No	14/05/2017	1	0	0
2014357737	362	district/faith-based	No	30/05/2017	1	0	0
2014357739	362	district/faith-based	No	30/05/2017	1	0	0
2014330350	362	district/faith-based	No	30/05/2017	1	0	0
2015390310	362	district/faith-based	No	30/05/2017	1	0	0
2015390311	362	district/faith-based	No	30/05/2017	1	0	0
2014327513	362	district/faith-based	No	30/05/2017	1	0	0
2012369971	362	district/faith-based	No	30/05/2017	1	0	0
2011143947	362	district/faith-based	No	30/05/2017	1	0	0
2015294179	362	district/faith-based	No	30/05/2017	1	0	0
2014340274	362	district/faith-based	No	30/05/2017	1	0	0
2012348843	362	district/faith-based	No	31/05/2017	1	0	0
2015344734	362	district/faith-based	No	29/05/2017	1	0	0
2014349191	362	district/faith-based	No	31/05/2017	1	0	0
2014348448	362	district/faith-based	No	31/05/2017	1	0	0
201707	362	district/faith-based	No	09/05/2017	1	0	0
201708	362	district/faith-based	No	16/05/2017	1	0	0
201709	362	district/faith-based	No	16/05/2017	1	0	0
201711	362	district/faith-based	No	16/05/2017	1	0	0
201712	362	district/faith-based	No	16/05/2017	1	0	0
201713	362	district/faith-based	No	16/05/2017	1	0	0
2015340353	362	district/faith-based	No	31/05/2017	1	0	0
2015328500	362	district/faith-based	No	31/05/2017	1	0	0
2015331946	362	district/faith-based	No	31/05/2017	1	0	0
2015363304	362	district/faith-based	No	31/05/2017	1	0	0
2015405007	362	district/faith-based	No	31/05/2017	1	0	0
2013256214	362	district/faith-based	No	31/05/2017	1	0	0
2013256215	362	district/faith-based	No	31/05/2017	1	0	0
2013256216	362	district/faith-based	No	31/05/2017	1	0	0
2013256217	362	district/faith-based	No	31/05/2017	1	0	0
2013256218	362	district/faith-based	No	31/05/2017	1	0	0
2015322046	362	district/faith-based	No	31/05/2017	1	0	0
2015322398	362	district/faith-based	No	31/05/2017	1	0	0
2015314092	362	district/faith-based	No	31/05/2017	1	0	0
2015314093	362	district/faith-based	No	31/05/2017	1	0	0
2015314094	362	district/faith-based	No	31/05/2017	1	0	0
2015314095	362	district/faith-based	No	31/05/2017	1	0	0
2015314096	362	district/faith-based	No	31/05/2017	1	0	0
2015314097	362	district/faith-based	No	31/05/2017	1	0	0
2015314098	362	district/faith-based	No	31/05/2017	1	0	0
2015314099	362	district/faith-based	No	31/05/2017	1	0	0
2015314100	362	district/faith-based	No	31/05/2017	1	0	0
2015314001	362	district/faith-based	No	31/05/2017	1	0	0
2015314002	362	district/faith-based	No	31/05/2017	1	0	0
2015314003	362	district/faith-based	No	31/05/2017	1	0	0
2015314004	362	district/faith-based	No	31/05/2017	1	0	0
2015412307	362	district/faith-based	No	31/05/2017	1	0	0
2015412308	362	district/faith-based	No	31/05/2017	1	0	0
2015412306	362	district/faith-based	No	31/05/2017	1	0	0
2014356998	362	district/faith-based	No	01/06/2017	1	0	0
2011136108	362	district/faith-based	No	30/05/2017	1	0	0
2015293839	362	district/faith-based	No	01/06/2017	1	0	0
2014356996	362	district/faith-based	No	01/06/2017	1	0	0
2011125797	362	district/faith-based	No	01/06/2017	1	0	0
2012359949	362	district/faith-based	No	01/06/2017	1	0	0
2014335569	362	district/faith-based	No	01/06/2017	1	0	0
2014378733	362	district/faith-based	No	30/05/2017	1	0	0

2015344738	362	district/faith-based	No	01/06/2017	1	0	0
2012359950	362	district/faith-based	No	01/06/2017	1	0	0
2011125798	362	district/faith-based	No	01/06/2017	1	0	0
2015332466	362	district/faith-based	No	01/06/2017	1	0	0
2015413596	362	district/faith-based	No	01/06/2017	1	0	0
2015332465	362	district/faith-based	No	01/06/2017	1	0	0
2015413597	362	district/faith-based	No	01/06/2017	1	0	0
2014317630	362	district/faith-based	No	01/06/2017	1	0	0
2015324571	362	district/faith-based	No	01/06/2017	1	0	0
2015372023	362	district/faith-based	No	30/05/2017	1	0	0
2015372023	362	district/faith-based	No	30/05/2017	1	0	0
2015402629	362	district/faith-based	No	30/05/2017	1	0	0
2015402629	362	district/faith-based	No	30/05/2017	1	0	0
2014317631	362	district/faith-based	No	01/06/2017	1	0	0
2014335571	362	district/faith-based	No	01/06/2017	1	0	0
2014317632	362	district/faith-based	No	01/06/2017	1	0	0
2014321527	362	district/faith-based	No	01/06/2017	1	0	0
2015358544	362	district/faith-based	No	30/05/2017	1	0	0
2015358544	362	district/faith-based	No	30/05/2017	1	0	0
2015319251	362	district/faith-based	No	30/05/2017	1	0	0
2015351700	362	district/faith-based	No	30/05/2017	1	0	0
2015351447	362	district/faith-based	No	01/06/2017	1	0	0
2015364609	362	district/faith-based	No	01/06/2017	1	0	0
2014317633	362	district/faith-based	No	01/06/2017	1	0	0
2015364518	362	district/faith-based	No	01/06/2017	1	0	0
2014317634	362	district/faith-based	No	01/06/2017	1	0	0
2015351448	362	district/faith-based	No	01/06/2017	1	0	0
2014358339	362	district/faith-based	No	01/06/2017	1	0	0
2014317435	362	district/faith-based	No	01/06/2017	1	0	0
2015351449	362	district/faith-based	No	01/06/2017	1	0	0
2015364516	362	district/faith-based	No	01/06/2017	1	0	0
2015364517	362	district/faith-based	No	01/06/2017	1	0	0
2014314222	362	district/faith-based	No	01/06/2017	1	0	0
2014314221	362	district/faith-based	No	01/06/2017	1	0	0
2015351450	362	district/faith-based	No	01/06/2017	1	0	0
2014291896	362	district/faith-based	No	01/06/2017	1	0	0
2014314220	362	district/faith-based	No	01/06/2017	1	0	0
2014291897	362	district/faith-based	No	01/06/2017	1	0	0
2014314219	362	district/faith-based	No	01/06/2017	1	0	0
2014291898	362	district/faith-based	No	01/06/2017	1	0	0
2014356993	362	district/faith-based	No	01/06/2017	1	0	0
2015414386	362	district/faith-based	No	01/06/2017	1	0	0
2012376596	362	district/faith-based	No	01/06/2017	1	0	0
2015414387	362	district/faith-based	No	01/06/2017	1	0	0
2015362818	362	district/faith-based	No	01/06/2017	1	0	0
2014377408	362	district/faith-based	No	01/06/2017	1	0	0
2015338851	362	district/faith-based	No	01/06/2017	1	0	0
2014297347	362	district/faith-based	No	01/06/2017	1	0	0
2015302858	362	district/faith-based	No	01/06/2017	1	0	0
2014377409	362	district/faith-based	No	01/06/2017	1	0	0
2015302859	362	district/faith-based	No	01/06/2017	1	0	0
2015321751	362	district/faith-based	No	01/06/2017	1	0	0
2015338852	362	district/faith-based	No	01/06/2017	1	0	0
2014377410	362	district/faith-based	No	01/06/2017	1	0	0
2014350047	362	district/faith-based	No	01/06/2017	1	0	0
2013253029	362	district/faith-based	No	01/06/2017	1	0	0
2015418861	813	rural/clinic	No	01/06/2017	0	0	0
2015301629	813	rural/clinic	No	01/06/2017	0	0	0
2015377895	813	rural/clinic	No	01/06/2017	0	0	0
2014316056	813	rural/clinic	No	01/06/2017	0	0	0
2015318754	813	rural/clinic	No	01/06/2017	0	0	0
2015338853	813	rural/clinic	No	01/06/2017	0	0	0
2015397874	813	rural/clinic	No	01/06/2017	0	0	0
2015335129	813	rural/clinic	No	01/06/2017	0	0	0
2015397870	813	rural/clinic	No	01/06/2017	0	0	0
2015335130	813	rural/clinic	No	01/06/2017	0	0	0
2015301630	813	rural/clinic	No	01/06/2017	0	0	0
2015397875	813	rural/clinic	No	01/06/2017	0	0	0
2015335131	813	rural/clinic	No	01/06/2017	0	0	0
2011213937	813	rural/clinic	No	01/06/2017	0	0	0
2015397871	813	rural/clinic	No	01/06/2017	0	0	0
2015335132	813	rural/clinic	No	01/06/2017	0	0	0
2015397872	813	rural/clinic	No	01/06/2017	0	0	0
2013248193	813	rural/clinic	No	01/06/2017	0	0	0
2011168521	813	rural/clinic	No	01/06/2017	0	0	0
2015406276	813	rural/clinic	No	01/06/2017	0	0	0
2013248195	813	rural/clinic	No	01/06/2017	0	0	0
2015406277	813	rural/clinic	No	01/06/2017	0	0	0
2011168520	813	rural/clinic	No	01/06/2017	0	0	0
2015319252	813	rural/clinic	No	01/06/2017	0	0	0
2015406278	813	rural/clinic	No	01/06/2017	0	0	0
2015338854	813	rural/clinic	No	01/06/2017	0	0	0

2015377326	813	rural/clinic	No	01/06/2017	0	0	0
2015319253	813	rural/clinic	No	01/06/2017	0	0	0
2015406279	813	rural/clinic	No	01/06/2017	0	0	0
2015335203	813	rural/clinic	No	01/06/2017	0	0	0
2015406280	813	rural/clinic	No	01/06/2017	0	0	0
2015319254	813	rural/clinic	No	01/06/2017	0	0	0
2015338855	813	rural/clinic	No	01/06/2017	0	0	0
2015319255	813	rural/clinic	No	01/06/2017	0	0	0
2015373417	813	rural/clinic	No	01/06/2017	0	0	0
2015319256	813	rural/clinic	No	01/06/2017	0	0	0
2015338856	813	rural/clinic	No	01/06/2017	0	0	0
2015373418	813	rural/clinic	No	01/06/2017	0	0	0
2015319257	813	rural/clinic	No	01/06/2017	0	0	0
2011135668	281	rural/clinic	No	01/06/2017	0	0	0
2015373421	281	rural/clinic	No	01/06/2017	0	0	0
2015340114	281	rural/clinic	No	01/06/2017	0	0	0
2011141203	281	rural/clinic	No	01/06/2017	0	0	0
2011135669	281	rural/clinic	No	01/06/2017	0	0	0
2015340116	281	rural/clinic	No	01/06/2017	0	0	0
2011135671	281	rural/clinic	No	01/06/2017	0	0	0
2011141202	281	rural/clinic	No	01/06/2017	0	0	0
2015373422	281	rural/clinic	No	01/06/2017	0	0	0
2015301485	281	rural/clinic	No	01/06/2017	0	0	0
2015362057	281	rural/clinic	No	01/06/2017	0	0	0
2015340117	281	rural/clinic	No	01/06/2017	0	0	0
2015373423	281	rural/clinic	No	01/06/2017	0	0	0
2014347017	281	rural/clinic	No	01/06/2017	0	0	0
2014327511	281	rural/clinic	No	01/06/2017	0	0	0
2015373424	281	rural/clinic	No	01/06/2017	0	0	0
2014347018	281	rural/clinic	No	01/06/2017	0	0	0
2015340118	281	rural/clinic	No	01/06/2017	0	0	0
2014327512	281	rural/clinic	No	01/06/2017	0	0	0
2015373425	281	rural/clinic	No	01/06/2017	0	0	0
2014347016	281	rural/clinic	No	01/06/2017	0	0	0
2015368663	281	rural/clinic	No	01/06/2017	0	0	0
2015373426	281	rural/clinic	No	01/06/2017	0	0	0
2015334937	281	rural/clinic	No	01/06/2017	0	0	0
2012369811	281	rural/clinic	No	01/06/2017	0	0	0
2015288750	281	rural/clinic	No	01/06/2017	0	0	0
2015334938	281	rural/clinic	No	01/06/2017	0	0	0
2015397932	281	rural/clinic	No	01/06/2017	0	0	0
2015334939	281	rural/clinic	No	01/06/2017	0	0	0
2015355004	281	rural/clinic	No	01/06/2017	0	0	0
2015383726	281	rural/clinic	No	01/06/2017	0	0	0
2015334940	281	rural/clinic	No	01/06/2017	0	0	0
2014296328	281	rural/clinic	No	01/06/2017	0	0	0
2015383727	281	rural/clinic	No	01/06/2017	0	0	0
2014327682	281	rural/clinic	No	01/06/2017	0	0	0
2015403288	281	rural/clinic	No	01/06/2017	0	0	0
2015344091	281	rural/clinic	No	01/06/2017	0	0	0
2015340104	281	rural/clinic	No	01/06/2017	0	0	0
2015403282	281	rural/clinic	No	01/06/2017	0	0	0
2014327601	281	rural/clinic	No	01/06/2017	0	0	0
2015340108	281	rural/clinic	No	01/06/2017	0	0	0
2015332107	618	rural/clinic	No	01/06/2017	0	0	0
2015386706	618	rural/clinic	No	01/06/2017	0	0	0
2014383976	618	rural/clinic	No	01/06/2017	0	0	0
2015386707	618	rural/clinic	No	01/06/2017	0	0	0
2015379322	464	rural/clinic	No	01/06/2017	0	0	0
2015362523	464	rural/clinic	No	01/06/2017	0	0	0
2015289179	464	rural/clinic	No	01/06/2017	0	0	0
2012359948	464	rural/clinic	No	01/06/2017	0	0	0
2015385533	464	rural/clinic	No	01/06/2017	0	0	0
2015313216	464	rural/clinic	No	01/06/2017	0	0	0
2015313217	464	rural/clinic	No	01/06/2017	0	0	0
2015313218	464	rural/clinic	No	01/06/2017	0	0	0
2012359164	464	rural/clinic	No	01/06/2017	0	0	0
2015313219	464	rural/clinic	No	01/06/2017	0	0	0
2015313220	464	rural/clinic	No	01/06/2017	0	0	0
2015378859	464	rural/clinic	No	01/06/2017	0	0	0
2015313221	464	rural/clinic	No	01/06/2017	0	0	0
2015378857	464	rural/clinic	No	01/06/2017	0	0	0
2015378858	464	rural/clinic	No	01/06/2017	0	0	0
2015384046	814	rural/clinic	No	01/06/2017	0	0	0
2015286311	814	rural/clinic	No	01/06/2017	0	0	0
2012317558	814	rural/clinic	No	01/06/2017	0	0	0
2015344364	814	rural/clinic	No	01/06/2017	0	0	0
2015344363	814	rural/clinic	No	01/06/2017	0	0	0
2012317559	814	rural/clinic	No	01/06/2017	0	0	0
2015344362	814	rural/clinic	No	01/06/2017	0	0	0
2015334653	814	rural/clinic	No	01/06/2017	0	0	0
2015334654	814	rural/clinic	No	01/06/2017	0	0	0

2015351443	814	rural/clinic	No	01/06/2017	0	0	0
2015351444	814	rural/clinic	No	01/06/2017	0	0	0
2015334655	814	rural/clinic	No	01/06/2017	0	0	0
2015351445	814	rural/clinic	No	01/06/2017	0	0	0
2015303628	814	rural/clinic	No	01/06/2017	0	0	0
2015351446	814	rural/clinic	No	01/06/2017	0	0	0
2015303629	814	rural/clinic	No	01/06/2017	0	0	0
2015303630	814	rural/clinic	No	01/06/2017	0	0	0
2015316751	814	rural/clinic	No	02/06/2017	0	0	0
2014337046	814	rural/clinic	No	13/04/2017	0	0	0
2011193275	814	rural/clinic	No	01/06/2017	0	0	0
2015290028	814	rural/clinic	No	02/06/2017	0	0	0
2012253929	814	rural/clinic	No	02/06/2017	0	0	0
2012253930	814	rural/clinic	No	02/06/2017	0	0	0
2015397810	814	rural/clinic	No	02/06/2017	0	0	0
2015397811	814	rural/clinic	No	02/06/2017	0	0	0
2015397812	814	rural/clinic	No	02/06/2017	0	0	0
2015333260	814	rural/clinic	No	02/06/2017	0	0	0
2014305224	814	rural/clinic	No	02/06/2017	0	0	0
2014305225	814	rural/clinic	No	02/06/2017	0	0	0
2012377415	26	rural/clinic	No	02/06/2017	0	0	0
2012377416	26	rural/clinic	No	02/06/2017	0	0	0
2014382442	26	rural/clinic	No	02/06/2017	0	0	0
2014382443	26	rural/clinic	No	02/06/2017	0	0	0
2014305223	26	rural/clinic	No	02/06/2017	0	0	0
2014343025	26	rural/clinic	No	01/06/2017	0	0	0
2015346233	26	rural/clinic	No	02/06/2017	0	0	0
2012378366	26	rural/clinic	No	02/06/2017	0	0	0
2012384800	26	rural/clinic	No	02/06/2017	0	0	0
2011133644	26	rural/clinic	No	01/06/2017	0	0	0
2011133646	26	rural/clinic	No	01/06/2017	0	0	0
2011133647	26	rural/clinic	No	01/06/2017	0	0	0
2011133648	26	rural/clinic	No	01/06/2017	0	0	0
2011133649	26	rural/clinic	No	01/06/2017	0	0	0
2011133650	26	rural/clinic	No	01/06/2017	0	0	0
2015345051	26	rural/clinic	No	01/06/2017	0	0	0
2015345052	26	rural/clinic	No	01/06/2017	0	0	0
2015345053	26	rural/clinic	No	01/06/2017	0	0	0
2015345054	26	rural/clinic	No	01/06/2017	0	0	0
2015345055	26	rural/clinic	No	01/06/2017	0	0	0
2015305082	26	rural/clinic	No	01/06/2017	0	0	0
2015338757	26	rural/clinic	No	01/06/2017	0	0	0
2015297335	26	rural/clinic	No	01/06/2017	0	0	0
2015397873	26	rural/clinic	No	01/06/2017	0	0	0
2012284486	26	rural/clinic	No	01/06/2017	0	0	0
2014370218	26	rural/clinic	No	01/06/2017	0	0	0
2011133645	26	rural/clinic	No	01/06/2017	0	0	0
2015362817	26	rural/clinic	No	01/06/2017	0	0	0
2015340113	26	rural/clinic	No	01/06/2017	0	0	0
2015364519	26	rural/clinic	No	01/06/2017	0	0	0
2015303631	26	rural/clinic	No	01/06/2017	0	0	0
2014372248	26	rural/clinic	No	01/06/2017	0	0	0
2014372249	26	rural/clinic	No	01/06/2017	0	0	0
2015344652	26	rural/clinic	No	01/06/2017	0	0	0
2014372250	26	rural/clinic	No	01/06/2017	0	0	0
2015344654	26	rural/clinic	No	01/06/2017	0	0	0
2014372247	26	rural/clinic	No	01/06/2017	0	0	0
2015344651	26	rural/clinic	No	01/06/2017	0	0	0
2015326550	26	rural/clinic	No	01/06/2017	0	0	0
2015326951	26	rural/clinic	No	01/06/2017	0	0	0
2015326952	26	rural/clinic	No	01/06/2017	0	0	0
2015326953	26	rural/clinic	No	01/06/2017	0	0	0
2015403287	26	rural/clinic	No	29/05/2017	0	0	0
2015344092	26	rural/clinic	No	01/06/2017	0	0	0
2015324445	26	rural/clinic	No	30/05/2017	0	0	0
2015334632	26	rural/clinic	No	01/06/2017	0	0	0
2015293837	26	rural/clinic	No	01/06/2017	0	0	0
2015293838	26	rural/clinic	No	01/06/2017	0	0	0
2015305081	26	rural/clinic	No	01/06/2017	0	0	0
2015355008	26	rural/clinic	No	01/06/2017	0	0	0
2011133915	26	rural/clinic	No	01/06/2017	0	0	0
2015386704	26	rural/clinic	No	30/05/2017	0	0	0
2015386704	26	rural/clinic	No	30/05/2017	0	0	0
2014375601	26	rural/clinic	No	30/05/2017	0	0	0
2014375601	26	rural/clinic	No	30/05/2017	0	0	0
2015361450	26	rural/clinic	No	30/05/2017	0	0	0
2015361450	26	rural/clinic	No	30/05/2017	0	0	0
2011133912	26	rural/clinic	No	01/06/2017	0	0	0
2015361443	26	rural/clinic	No	30/05/2017	0	0	0
2015361443	26	rural/clinic	No	30/05/2017	0	0	0
2011133913	26	rural/clinic	No	01/06/2017	0	0	0
2011133914	26	rural/clinic	No	01/06/2017	0	0	0

2012295990	26	rural/clinic	No	30/05/2017	0	0	0
2012295990	26	rural/clinic	No	30/05/2017	0	0	0
2015349014	26	rural/clinic	No	01/06/2017	0	0	0
2015376000	26	rural/clinic	No	01/06/2017	0	0	0
2014288010	26	rural/clinic	No	01/06/2017	0	0	0
2015412096	26	rural/clinic	No	01/06/2017	0	0	0
2015349010	26	rural/clinic	No	01/06/2017	0	0	0
2014356991	26	rural/clinic	No	01/06/2017	0	0	0
2015349011	26	rural/clinic	No	01/06/2017	0	0	0
2014298590	26	rural/clinic	No	01/06/2017	0	0	0
2012284987	26	rural/clinic	No	01/06/2017	0	0	0
2015310659	26	rural/clinic	No	01/06/2017	0	0	0
2014298589	26	rural/clinic	No	01/06/2017	0	0	0
2014356994	26	rural/clinic	No	01/06/2017	0	0	0
2014288139	26	rural/clinic	No	01/06/2017	0	0	0
2014356989	26	rural/clinic	No	01/06/2017	0	0	0
2014288137	26	rural/clinic	No	01/06/2017	0	0	0
2014356997	26	rural/clinic	No	01/06/2017	0	0	0
2014288138	26	rural/clinic	No	01/06/2017	0	0	0
2015349009	26	rural/clinic	No	01/06/2017	0	0	0
2014356992	26	rural/clinic	No	01/06/2017	0	0	0
2014342792	26	rural/clinic	No	01/06/2017	0	0	0
2012284988	26	rural/clinic	No	01/06/2017	0	0	0
2015290201	26	rural/clinic	No	01/06/2017	0	0	0
2015290203	26	rural/clinic	No	01/06/2017	0	0	0
2015375999	26	rural/clinic	No	01/06/2017	0	0	0
2015349012	26	rural/clinic	No	01/06/2017	0	0	0
2014356995	26	rural/clinic	No	01/06/2017	0	0	0
2015349015	696	rural/clinic	No	01/06/2017	0	0	0
2012262491	696	rural/clinic	No	01/06/2017	0	0	0
2015290202	696	rural/clinic	No	01/06/2017	0	0	0
2015310614	696	rural/clinic	No	01/06/2017	0	0	0
2015349013	696	rural/clinic	No	01/06/2017	0	0	0
2014356990	696	rural/clinic	No	01/06/2017	0	0	0
2014290275	696	rural/clinic	No	01/06/2017	0	0	0
2014290324	696	rural/clinic	No	01/06/2017	0	0	0
2012275989	696	rural/clinic	No	01/06/2017	0	0	0
2015408113	696	rural/clinic	No	30/05/2017	0	0	0
2015408113	696	rural/clinic	No	30/05/2017	0	0	0
2014327513	696	rural/clinic	No	30/05/2017	0	0	0
2014327513	696	rural/clinic	No	30/05/2017	0	0	0
2011143947	696	rural/clinic	No	30/05/2017	0	0	0
2014363888	696	rural/clinic	No	02/06/2017	0	0	0
2015286872	696	rural/clinic	No	02/06/2017	0	0	0
2015313210	696	rural/clinic	No	30/05/2017	0	0	0
2012276256	696	rural/clinic	No	02/06/2017	0	0	0
2015301481	696	rural/clinic	No	31/05/2017	0	0	0
2015301481	696	rural/clinic	No	31/05/2017	0	0	0
2012289620	696	rural/clinic	No	02/06/2017	0	0	0
2014363886	696	rural/clinic	No	02/06/2017	0	0	0
2015334940	696	rural/clinic	No	01/06/2017	0	0	0
2015334940	696	rural/clinic	No	01/06/2017	0	0	0
2015413899	696	rural/clinic	No	01/06/2017	0	0	0
2015413899	696	rural/clinic	No	01/06/2017	0	0	0
2015413914	696	rural/clinic	No	30/05/2017	0	0	0
2015413914	696	rural/clinic	No	30/05/2017	0	0	0
2011125798	696	rural/clinic	No	01/06/2017	0	0	0
2011125798	13	rural/clinic	No	01/06/2017	0	0	0
2015334655	13	rural/clinic	No	01/06/2017	0	0	0
2015334655	13	rural/clinic	No	01/06/2017	0	0	0
2014335571	13	rural/clinic	No	01/06/2017	0	0	0
2014335571	13	rural/clinic	No	01/06/2017	0	0	0
2015364516	13	rural/clinic	No	01/06/2017	0	0	0
2015364516	13	rural/clinic	No	01/06/2017	0	0	0
2014291897	13	rural/clinic	No	01/06/2017	0	0	0
2014291897	13	rural/clinic	No	01/06/2017	0	0	0
2014377408	13	rural/clinic	No	01/06/2017	0	0	0
2014377409	13	rural/clinic	No	01/06/2017	0	0	0
2015406280	13	rural/clinic	No	01/06/2017	0	0	0
2015340109	13	rural/clinic	No	01/06/2017	0	0	0
2015340109	13	rural/clinic	No	01/06/2017	0	0	0
2015301484	13	rural/clinic	No	01/06/2017	0	0	0
2015301484	13	rural/clinic	No	01/06/2017	0	0	0
2010099625	13	rural/clinic	No	01/06/2017	0	0	0
2010099625	13	rural/clinic	No	01/06/2017	0	0	0
2012359948	13	rural/clinic	No	01/06/2017	0	0	0
2012359948	13	rural/clinic	No	01/06/2017	0	0	0
2013253029	13	rural/clinic	No	01/06/2017	0	0	0
2013253029	13	rural/clinic	No	01/06/2017	0	0	0
2012295992	13	rural/clinic	No	31/05/2017	0	0	0
2012295992	13	rural/clinic	No	31/05/2017	0	0	0
198537	13	rural/clinic	No	01/06/2017	0	0	0

2012335658	13	rural/clinic	No	28/03/2017	0	0	0
2012305808	13	rural/clinic	No	06/06/2017	0	0	0
2012305807	13	rural/clinic	No	06/06/2017	0	0	0
2012305809	13	rural/clinic	No	06/06/2017	0	0	0
2011136118	13	rural/clinic	No	06/06/2017	0	0	0
2015368587	13	rural/clinic	No	06/06/2017	0	0	0
2015320888	13	rural/clinic	No	06/06/2017	0	0	0
2013256272	13	rural/clinic	No	06/06/2017	0	0	0
2012293004	13	rural/clinic	No	06/06/2017	0	0	0
2011136112	13	rural/clinic	No	06/06/2017	0	0	0
2012361534	13	rural/clinic	No	06/06/2017	0	0	0
2012293006	13	rural/clinic	No	06/06/2017	0	0	0
2012293005	13	rural/clinic	No	06/06/2017	0	0	0
2015382930	13	rural/clinic	No	06/06/2017	0	0	0
2013261935	13	rural/clinic	No	06/06/2017	0	0	0
2015303083	13	rural/clinic	No	06/06/2017	0	0	0
2015303082	13	rural/clinic	No	06/06/2017	0	0	0
2014337221	13	rural/clinic	No	06/06/2017	0	0	0
2014337222	13	rural/clinic	No	06/06/2017	0	0	0
2015367213	13	rural/clinic	No	06/06/2017	0	0	0
2015367214	13	rural/clinic	No	06/06/2017	0	0	0
2013264390	13	rural/clinic	No	06/06/2017	0	0	0
2015376655	13	rural/clinic	No	06/06/2017	0	0	0
2015303085	13	rural/clinic	No	06/06/2017	0	0	0
2013255264	13	rural/clinic	No	06/06/2017	0	0	0
2015303084	13	rural/clinic	No	06/06/2017	0	0	0
2015303086	13	rural/clinic	No	06/06/2017	0	0	0
2012322688	13	rural/clinic	No	06/06/2017	0	0	0
2011114843	13	rural/clinic	No	06/06/2017	0	0	0
2015382926	13	rural/clinic	No	06/06/2017	0	0	0
2015355917	13	rural/clinic	No	06/06/2017	0	0	0
2015382927	13	rural/clinic	No	06/06/2017	0	0	0
2015382928	13	rural/clinic	No	06/06/2017	0	0	0
2015334539	13	rural/clinic	No	06/06/2017	0	0	0
2014371833	13	rural/clinic	No	06/06/2017	0	0	0
2015334209	13	rural/clinic	No	06/06/2017	0	0	0
2015344327	13	rural/clinic	No	06/06/2017	0	0	0
2015344326	13	rural/clinic	No	06/06/2017	0	0	0
2014371832	13	rural/clinic	No	06/06/2017	0	0	0
2014358190	13	rural/clinic	No	06/06/2017	0	0	0
2012365476	13	rural/clinic	No	06/06/2017	0	0	0
2012363967	13	rural/clinic	No	06/06/2017	0	0	0
2012365475	13	rural/clinic	No	06/06/2017	0	0	0
2012363965	13	rural/clinic	No	06/06/2017	0	0	0
2012365474	13	rural/clinic	No	06/06/2017	0	0	0
2012365473	13	rural/clinic	No	06/06/2017	0	0	0
2015368664	13	rural/clinic	No	06/06/2017	0	0	0
2015326790	13	rural/clinic	No	06/06/2017	0	0	0
2015326791	13	rural/clinic	No	06/06/2017	0	0	0
2015326792	14	rural/clinic	No	06/06/2017	0	0	0
2012363966	14	rural/clinic	No	06/06/2017	0	0	0
2015326793	14	rural/clinic	No	06/06/2017	0	0	0
2012363964	14	rural/clinic	No	06/06/2017	0	0	0
2015326794	14	rural/clinic	No	06/06/2017	0	0	0
2015326795	14	rural/clinic	No	06/06/2017	0	0	0
2015326796	14	rural/clinic	No	06/06/2017	0	0	0
2014366072	14	rural/clinic	No	06/06/2017	0	0	0
2015326797	14	rural/clinic	No	06/06/2017	0	0	0
2014366071	14	rural/clinic	No	06/06/2017	0	0	0
2015326798	14	rural/clinic	No	06/06/2017	0	0	0
2011136111	14	rural/clinic	No	06/06/2017	0	0	0
2015326799	14	rural/clinic	No	06/06/2017	0	0	0
2015326800	14	rural/clinic	No	06/06/2017	0	0	0
2014306027	14	rural/clinic	No	06/06/2017	0	0	0
2015326401	14	rural/clinic	No	06/06/2017	0	0	0
2015382929	14	rural/clinic	No	06/06/2017	0	0	0
2014306026	14	rural/clinic	No	06/06/2017	0	0	0
2015326402	14	rural/clinic	No	06/06/2017	0	0	0
2014306024	14	rural/clinic	No	06/06/2017	0	0	0
2015382925	14	rural/clinic	No	06/06/2017	0	0	0
2015326403	14	rural/clinic	No	06/06/2017	0	0	0
2014306023	14	rural/clinic	No	06/06/2017	0	0	0
2014306021	14	rural/clinic	No	06/06/2017	0	0	0
2015382924	14	rural/clinic	No	06/06/2017	0	0	0
2015326404	14	rural/clinic	No	06/06/2017	0	0	0
2014306020	14	rural/clinic	No	06/06/2017	0	0	0
2014327514	14	rural/clinic	No	06/06/2017	0	0	0
2013255265	14	rural/clinic	No	06/06/2017	0	0	0
2015326405	14	rural/clinic	No	06/06/2017	0	0	0
2015326406	14	rural/clinic	No	06/06/2017	0	0	0
2014291899	14	rural/clinic	No	05/06/2017	0	0	0
2015324448	14	rural/clinic	No	06/06/2017	0	0	0

2012388399	14	rural/clinic	No	07/06/2017	0	0	0
2017022	14	rural/clinic	No	23/05/2017	0	0	0
2015384785	14	rural/clinic	No	06/06/2017	0	0	0
201723	14	rural/clinic	No	22/05/2017	0	0	0
2014375501	14	rural/clinic	No	06/06/2017	0	0	0
2015414348	14	rural/clinic	No	06/06/2017	0	0	0
201724	14	rural/clinic	No	23/05/2017	0	0	0
2015414350	14	rural/clinic	No	06/06/2017	0	0	0
201725	14	rural/clinic	No	23/05/2017	0	0	0
2015414253	14	rural/clinic	No	06/06/2017	0	0	0
2015414254	14	rural/clinic	No	06/06/2017	0	0	0
2014349104	14	rural/clinic	No	24/05/2017	0	0	0
2014377624	14	rural/clinic	No	06/06/2017	0	0	0
2015414252	14	rural/clinic	No	06/06/2017	0	0	0
2015384487	14	rural/clinic	No	06/06/2017	0	0	0
2015414251	14	rural/clinic	No	06/06/2017	0	0	0
2015377839	14	rural/clinic	No	06/06/2017	0	0	0
2015377842	14	rural/clinic	No	06/06/2017	0	0	0
2015377841	14	rural/clinic	No	06/06/2017	0	0	0
2013264068	14	rural/clinic	No	06/06/2017	0	0	0
2015377830	14	rural/clinic	No	06/06/2017	0	0	0
2015377840	14	rural/clinic	No	06/06/2017	0	0	0
2015390262	14	rural/clinic	No	06/06/2017	0	0	0
2013264069	14	rural/clinic	No	06/06/2017	0	0	0
2015405625	14	rural/clinic	No	06/06/2017	0	0	0
2015390263	14	rural/clinic	No	06/06/2017	0	0	0
2015333853	14	rural/clinic	No	06/06/2017	0	0	0
2014358016	14	rural/clinic	No	06/06/2017	0	0	0
2014358017	14	rural/clinic	No	06/06/2017	0	0	0
2014358015	14	rural/clinic	No	06/06/2017	0	0	0
2014358014	14	rural/clinic	No	06/06/2017	0	0	0
2011140757	14	rural/clinic	No	06/06/2017	0	0	0
2011140758	14	rural/clinic	No	06/06/2017	0	0	0
2011140759	14	rural/clinic	No	06/06/2017	0	0	0
2011140760	14	rural/clinic	No	06/06/2017	0	0	0
2011140761	14	rural/clinic	No	06/06/2017	0	0	0
2011140762	14	rural/clinic	No	06/06/2017	0	0	0
2015326810	14	rural/clinic	No	05/06/2017	0	0	0
2015362273	14	rural/clinic	No	06/06/2017	0	0	0
2015326811	14	rural/clinic	No	05/06/2017	0	0	0
2015362272	14	rural/clinic	No	06/06/2017	0	0	0
2015362271	14	rural/clinic	No	06/06/2017	0	0	0
2011136117	14	rural/clinic	No	06/06/2017	0	0	0
2015326812	14	rural/clinic	No	05/06/2017	0	0	0
2015362270	14	rural/clinic	No	06/06/2017	0	0	0
2013266076	14	rural/clinic	No	06/06/2017	0	0	0
2015362269	14	rural/clinic	No	06/06/2017	0	0	0
2015362268	14	rural/clinic	No	06/06/2017	0	0	0
2012294064	14	rural/clinic	No	06/06/2017	0	0	0
2015362267	14	rural/clinic	No	06/06/2017	0	0	0
2015342010	14	rural/clinic	No	06/06/2017	0	0	0
2015320887	14	rural/clinic	No	06/06/2017	0	0	0
2012253242	14	rural/clinic	No	06/06/2017	0	0	0
2015326815	14	rural/clinic	No	05/06/2017	0	0	0
2013285126	14	rural/clinic	No	06/06/2017	0	0	0
2012253241	14	rural/clinic	No	06/06/2017	0	0	0
2015320886	14	rural/clinic	No	06/06/2017	0	0	0
2015326816	14	rural/clinic	No	05/06/2017	0	0	0
2015337369	14	rural/clinic	No	06/06/2017	0	0	0
2015326817	14	rural/clinic	No	05/06/2017	0	0	0
2015314552	14	rural/clinic	No	06/06/2017	0	0	0
2015326818	14	rural/clinic	No	05/06/2017	0	0	0
2015337368	14	rural/clinic	No	06/06/2017	0	0	0
2015384784	14	rural/clinic	No	06/06/2017	0	0	0
2015397636	14	rural/clinic	No	03/06/2017	0	0	0
2015324476	14	rural/clinic	No	06/06/2017	0	0	0
2015397637	14	rural/clinic	No	05/06/2017	0	0	0
2015324477	14	rural/clinic	No	06/06/2017	0	0	0
2015397638	14	rural/clinic	No	05/06/2017	0	0	0
201066585	14	rural/clinic	No	06/06/2017	0	0	0
2015397639	14	rural/clinic	No	05/06/2017	0	0	0
2012358292	14	rural/clinic	No	06/06/2017	0	0	0
2012358289	14	rural/clinic	No	06/06/2017	0	0	0
2012358291	14	rural/clinic	No	06/06/2017	0	0	0
2015397640	14	rural/clinic	No	05/06/2017	0	0	0
2012358290	14	rural/clinic	No	06/06/2017	0	0	0
2015397641	14	rural/clinic	No	05/06/2017	0	0	0
2012388395	14	rural/clinic	No	06/06/2017	0	0	0
2015397642	14	rural/clinic	No	05/06/2017	0	0	0
2012388396	14	rural/clinic	No	06/06/2017	0	0	0
2015328072	14	rural/clinic	No	06/06/2017	0	0	0
2012388397	14	rural/clinic	No	06/06/2017	0	0	0

2012388394	14	rural/clinic	No	06/06/2017	0	0	0
2015328071	14	rural/clinic	No	06/06/2017	0	0	0
2015328070	14	rural/clinic	No	06/06/2017	0	0	0
2015328069	14	rural/clinic	No	06/06/2017	0	0	0
2015328068	14	rural/clinic	No	06/06/2017	0	0	0
2015328067	14	rural/clinic	No	06/06/2017	0	0	0
2015328066	14	rural/clinic	No	06/06/2017	0	0	0
2015328065	14	rural/clinic	No	06/06/2017	0	0	0
2014316057	14	rural/clinic	No	06/06/2017	0	0	0
2015326954	14	rural/clinic	No	06/06/2017	0	0	0
2015326955	14	rural/clinic	No	06/06/2017	0	0	0
2015368975	14	rural/clinic	No	06/06/2017	0	0	0
2015368976	82	rural/clinic	No	06/06/2017	0	0	0
2015368977	82	rural/clinic	No	06/06/2017	0	0	0
2015368978	82	rural/clinic	No	06/06/2017	0	0	0
2015340103	82	rural/clinic	No	06/06/2017	0	0	0
2015340105	82	rural/clinic	No	06/06/2017	0	0	0
2015340106	82	rural/clinic	No	06/06/2017	0	0	0
2015340107	82	rural/clinic	No	06/06/2017	0	0	0
2015289180	82	rural/clinic	No	06/06/2017	0	0	0
2011235683	82	rural/clinic	No	06/06/2017	0	0	0
2015340753	82	rural/clinic	No	06/06/2017	0	0	0
2015340703	82	rural/clinic	No	06/06/2017	0	0	0
2011236081	82	rural/clinic	No	06/06/2017	0	0	0
2010099923	82	rural/clinic	No	06/06/2017	0	0	0
2010099921	82	rural/clinic	No	06/06/2017	0	0	0
2010099920	82	rural/clinic	No	06/06/2017	0	0	0
2015357805	332	rural/clinic	No	06/06/2017	0	0	0
2011236082	332	rural/clinic	No	06/06/2017	0	0	0
2015357804	332	rural/clinic	No	06/06/2017	0	0	0
2015357657	332	rural/clinic	No	06/06/2017	0	0	0
2014290240	332	rural/clinic	No	06/06/2017	0	0	0
2015340213	332	rural/clinic	No	06/06/2017	0	0	0
2015340214	332	rural/clinic	No	06/06/2017	0	0	0
2011235420	332	rural/clinic	No	06/06/2017	0	0	0
2015340704	332	rural/clinic	No	06/06/2017	0	0	0
2011235380	332	rural/clinic	No	06/06/2017	0	0	0
2015340705	332	rural/clinic	No	06/06/2017	0	0	0
2012291360	332	rural/clinic	No	06/06/2017	0	0	0
2012291361	332	rural/clinic	No	06/06/2017	0	0	0
2015363069	332	rural/clinic	No	06/06/2017	0	0	0
2012330886	332	rural/clinic	No	06/06/2017	0	0	0
2015363070	332	rural/clinic	No	06/06/2017	0	0	0
2015363066	332	rural/clinic	No	06/06/2017	0	0	0
2015363068	332	rural/clinic	No	06/06/2017	0	0	0
2015363067	332	rural/clinic	No	06/06/2017	0	0	0
2014308892	332	rural/clinic	No	06/06/2017	0	0	0
2014308893	332	rural/clinic	No	06/06/2017	0	0	0
2013261297	332	rural/clinic	No	06/06/2017	0	0	0
2014306028	332	rural/clinic	No	06/06/2017	0	0	0
2015293950	332	rural/clinic	No	06/06/2017	0	0	0
2015293710	332	rural/clinic	No	06/06/2017	0	0	0
2012261205	332	rural/clinic	No	06/06/2017	0	0	0
2012261204	332	rural/clinic	No	06/06/2017	0	0	0
2015345054	332	rural/clinic	No	01/06/2017	0	0	0
2015345054	332	rural/clinic	No	01/06/2017	0	0	0
2015344102	332	rural/clinic	No	06/06/2017	0	0	0
2015344103	332	rural/clinic	No	06/06/2017	0	0	0
2012284988	332	rural/clinic	No	01/06/2017	0	0	0
2012284988	332	rural/clinic	No	01/06/2017	0	0	0
2015344101	332	rural/clinic	No	06/06/2017	0	0	0
2014311370	332	rural/clinic	No	06/06/2017	0	0	0
2015397810	332	rural/clinic	No	02/06/2017	0	0	0
2015397810	332	rural/clinic	No	02/06/2017	0	0	0
2015338757	332	rural/clinic	No	01/06/2017	0	0	0
2015338757	332	rural/clinic	No	01/06/2017	0	0	0
2015338852	332	rural/clinic	No	01/06/2017	0	0	0
2015338852	332	rural/clinic	No	01/06/2017	0	0	0
2012261191	332	rural/clinic	No	06/06/2017	0	0	0
2012261192	332	rural/clinic	No	06/06/2017	0	0	0
2012261189	332	rural/clinic	No	06/06/2017	0	0	0
2015305082	332	rural/clinic	No	01/06/2017	0	0	0
2015305082	332	rural/clinic	No	01/06/2017	0	0	0
2014356991	332	rural/clinic	No	01/06/2017	0	0	0
2014356991	332	rural/clinic	No	01/06/2017	0	0	0
2015349011	332	rural/clinic	No	01/06/2017	0	0	0
2012261190	332	rural/clinic	No	06/06/2017	0	0	0
2015334535	332	rural/clinic	No	06/06/2017	0	0	0
2015334536	332	rural/clinic	No	06/06/2017	0	0	0
2015334538	332	rural/clinic	No	06/06/2017	0	0	0
2015318762	332	rural/clinic	No	07/06/2017	0	0	0
2011214932	332	rural/clinic	No	07/06/2017	0	0	0

2015318760	332	rural/clinic	No	07/06/2017	0	0	0
2011188479	332	rural/clinic	No	07/06/2017	0	0	0
2015318761	332	rural/clinic	No	07/06/2017	0	0	0
2011215296	332	rural/clinic	No	07/06/2017	0	0	0
2011215173	332	rural/clinic	No	07/06/2017	0	0	0
2015299481	332	rural/clinic	No	07/06/2017	0	0	0
2015299484	332	rural/clinic	No	07/06/2017	0	0	0
2013276156	332	rural/clinic	No	07/06/2017	0	0	0
2015318991	332	rural/clinic	No	07/06/2017	0	0	0
2015301818	332	rural/clinic	No	07/06/2017	0	0	0
2014376896	332	rural/clinic	No	07/06/2017	0	0	0
2013248197	332	rural/clinic	No	07/06/2017	0	0	0
2015301820	332	rural/clinic	No	07/06/2017	0	0	0
2015301819	332	rural/clinic	No	07/06/2017	0	0	0
2014328441	332	rural/clinic	No	07/06/2017	0	0	0
2014328444	332	rural/clinic	No	07/06/2017	0	0	0
2014328443	332	rural/clinic	No	07/06/2017	0	0	0
2015301817	332	rural/clinic	No	07/06/2017	0	0	0
2014334179	332	rural/clinic	No	07/06/2017	0	0	0
2015286649	332	rural/clinic	No	07/06/2017	0	0	0
2015289332	332	rural/clinic	No	07/06/2017	0	0	0
2014334180	332	rural/clinic	No	07/06/2017	0	0	0
2011169265	332	rural/clinic	No	07/06/2017	0	0	0
2015335594	332	rural/clinic	No	07/06/2017	0	0	0
2015293479	332	rural/clinic	No	07/06/2017	0	0	0
2015335591	332	rural/clinic	No	07/06/2017	0	0	0
2011213938	332	rural/clinic	No	07/06/2017	0	0	0
2015289285	332	rural/clinic	No	07/06/2017	0	0	0
2015334633	332	rural/clinic	No	07/06/2017	0	0	0
2015293840	332	rural/clinic	No	07/06/2017	0	0	0
2015293841	332	rural/clinic	No	07/06/2017	0	0	0
2015293842	332	rural/clinic	No	07/06/2017	0	0	0
2015334941	332	rural/clinic	No	07/06/2017	0	0	0
2015335592	332	rural/clinic	No	07/06/2017	0	0	0
2015334942	332	rural/clinic	No	07/06/2017	0	0	0
2015334943	332	rural/clinic	No	07/06/2017	0	0	0
2015413598	332	rural/clinic	No	07/06/2017	0	0	0
2011139403	332	rural/clinic	No	07/06/2017	0	0	0
2011139404	332	rural/clinic	No	07/06/2017	0	0	0
2014382701	332	rural/clinic	No	07/06/2017	0	0	0
2014382702	332	rural/clinic	No	07/06/2017	0	0	0
2014382703	332	rural/clinic	No	07/06/2017	0	0	0
2012260085	332	rural/clinic	No	07/06/2017	0	0	0
2012260086	332	rural/clinic	No	07/06/2017	0	0	0
2014327683	332	rural/clinic	No	07/06/2017	0	0	0
2014327684	332	rural/clinic	No	07/06/2017	0	0	0
2011196015	332	rural/clinic	No	07/06/2017	0	0	0
2013256272	332	rural/clinic	No	06/06/2017	0	0	0
2013256272	332	rural/clinic	No	06/06/2017	0	0	0
2011196016	332	rural/clinic	No	07/06/2017	0	0	0
2012363967	332	rural/clinic	No	06/06/2017	0	0	0
2012363967	332	rural/clinic	No	06/06/2017	0	0	0
2015326799	332	rural/clinic	No	06/06/2017	0	0	0
2015326799	332	rural/clinic	No	06/06/2017	0	0	0
2015326800	332	rural/clinic	No	06/06/2017	0	0	0
2015326800	332	rural/clinic	No	06/06/2017	0	0	0
2012363964	332	rural/clinic	No	06/06/2017	0	0	0
2012363964	332	rural/clinic	No	06/06/2017	0	0	0
2011135670	332	rural/clinic	No	07/06/2017	0	0	0
2014328442	332	rural/clinic	No	07/06/2017	0	0	0
2014291899	332	rural/clinic	No	05/06/2017	0	0	0
2014291899	332	rural/clinic	No	05/06/2017	0	0	0
2015328069	332	rural/clinic	No	06/06/2017	0	0	0
2015297483	332	rural/clinic	No	07/06/2017	0	0	0
2015340107	332	rural/clinic	No	06/06/2017	0	0	0
2015384811	332	rural/clinic	No	07/06/2017	0	0	0
2015335450	332	rural/clinic	No	07/06/2017	0	0	0
2011212964	332	rural/clinic	No	07/06/2017	0	0	0
2015297484	332	rural/clinic	No	07/06/2017	0	0	0
2015355782	332	rural/clinic	No	07/06/2017	0	0	0
2015297485	332	rural/clinic	No	07/06/2017	0	0	0
2015362499	332	rural/clinic	No	07/06/2017	0	0	0
2015297486	332	rural/clinic	No	07/06/2017	0	0	0
2012261383	332	rural/clinic	No	07/06/2017	0	0	0
2015335967	332	rural/clinic	No	07/06/2017	0	0	0
2015337984	332	rural/clinic	No	07/06/2017	0	0	0
2015337983	332	rural/clinic	No	07/06/2017	0	0	0
2015337986	332	rural/clinic	No	07/06/2017	0	0	0
2015337982	332	rural/clinic	No	07/06/2017	0	0	0
2011212961	332	rural/clinic	No	07/06/2017	0	0	0
2015289333	332	rural/clinic	No	07/06/2017	0	0	0
2015337988	332	rural/clinic	No	07/06/2017	0	0	0

2015337985	332	rural/clinic	No	07/06/2017	0	0	0
2012321338	332	rural/clinic	No	07/06/2017	0	0	0
2015335593	332	rural/clinic	No	07/06/2017	0	0	0
2015335449	332	rural/clinic	No	07/06/2017	0	0	0
2015289286	332	rural/clinic	No	07/06/2017	0	0	0
2015335448	332	rural/clinic	No	07/06/2017	0	0	0
2012317319	332	rural/clinic	No	07/06/2017	0	0	0
2014359733	332	rural/clinic	No	07/06/2017	0	0	0
2015297487	332	rural/clinic	No	07/06/2017	0	0	0
2015289334	332	rural/clinic	No	07/06/2017	0	0	0
2015297340	332	rural/clinic	No	07/06/2017	0	0	0
2015337987	332	rural/clinic	No	07/06/2017	0	0	0
2015297339	332	rural/clinic	No	07/06/2017	0	0	0
2015373851	332	rural/clinic	No	07/06/2017	0	0	0
2015373852	332	rural/clinic	No	07/06/2017	0	0	0
2014291900	332	rural/clinic	No	07/06/2017	0	0	0
2015377896	332	rural/clinic	No	07/06/2017	0	0	0
2015377897	332	rural/clinic	No	07/06/2017	0	0	0
2013248196	332	rural/clinic	No	07/06/2017	0	0	0
2015330480	332	rural/clinic	No	07/06/2017	0	0	0
2015330481	332	rural/clinic	No	07/06/2017	0	0	0
2015301816	332	rural/clinic	No	07/06/2017	0	0	0
2015299288	332	rural/clinic	No	07/06/2017	0	0	0
2011213939	332	rural/clinic	No	07/06/2017	0	0	0
2015318734	332	rural/clinic	No	07/06/2017	0	0	0
2013248199	332	rural/clinic	No	07/06/2017	0	0	0
2015318759	332	rural/clinic	No	07/06/2017	0	0	0
2015318756	332	rural/clinic	No	07/06/2017	0	0	0
2015318758	332	rural/clinic	No	07/06/2017	0	0	0
2015318757	332	rural/clinic	No	07/06/2017	0	0	0
2015415056	332	rural/clinic	No	30/05/2017	0	0	0
2014301468	332	rural/clinic	No	08/06/2017	0	0	0
2015297271	332	rural/clinic	No	08/06/2017	0	0	0
2012305591	332	rural/clinic	No	08/06/2017	0	0	0
2012305592	332	rural/clinic	No	08/06/2017	0	0	0
2015297270	332	rural/clinic	No	08/06/2017	0	0	0
2015297272	332	rural/clinic	No	08/06/2017	0	0	0
2015352729	332	rural/clinic	No	08/06/2017	0	0	0
2015352722	332	rural/clinic	No	08/06/2017	0	0	0
2015352720	332	rural/clinic	No	08/06/2017	0	0	0
2015332028	332	rural/clinic	No	08/06/2017	0	0	0
2015352725	332	rural/clinic	No	08/06/2017	0	0	0
2015324242	332	rural/clinic	No	08/06/2017	0	0	0
2015324244	332	rural/clinic	No	08/06/2017	0	0	0
2015324243	332	rural/clinic	No	08/06/2017	0	0	0
2015352719	332	rural/clinic	No	08/06/2017	0	0	0
2015352723	332	rural/clinic	No	08/06/2017	0	0	0
2015332027	332	rural/clinic	No	08/06/2017	0	0	0
2015352724	332	rural/clinic	No	08/06/2017	0	0	0
2015352721	332	rural/clinic	No	08/06/2017	0	0	0
2015352728	332	rural/clinic	No	08/06/2017	0	0	0
2014378557	207	rural/clinic	No	08/06/2017	0	0	0
2015324245	207	rural/clinic	No	08/06/2017	0	0	0
2013258456	207	rural/clinic	No	08/06/2017	0	0	0
2015324246	207	rural/clinic	No	08/06/2017	0	0	0
2014357538	207	rural/clinic	No	08/06/2017	0	0	0
2014357539	207	rural/clinic	No	08/06/2017	0	0	0
2014357537	207	rural/clinic	No	08/06/2017	0	0	0
2014357536	207	rural/clinic	No	08/06/2017	0	0	0
2014357535	207	rural/clinic	No	08/06/2017	0	0	0
2014370302	207	rural/clinic	No	08/06/2017	0	0	0
2015332968	207	rural/clinic	No	08/06/2017	0	0	0
2015332969	207	rural/clinic	No	08/06/2017	0	0	0
2012344502	207	rural/clinic	No	08/06/2017	0	0	0
2012246287	333	rural/clinic	No	08/06/2017	0	0	0
2014385024	333	rural/clinic	No	08/06/2017	0	0	0
2012246286	333	rural/clinic	No	08/06/2017	0	0	0
2012348842	333	rural/clinic	No	08/06/2017	0	0	0
2012331325	333	rural/clinic	No	08/06/2017	0	0	0
2015306488	333	rural/clinic	No	08/06/2017	0	0	0
2015332970	333	rural/clinic	No	08/06/2017	0	0	0
2015374450	333	rural/clinic	No	08/06/2017	0	0	0
2015356455	333	rural/clinic	No	08/06/2017	0	0	0
2014323742	333	rural/clinic	No	08/06/2017	0	0	0
2015374451	333	rural/clinic	No	08/06/2017	0	0	0
2012259892	333	rural/clinic	No	08/06/2017	0	0	0
2014349105	333	rural/clinic	No	08/06/2017	0	0	0
2014383530	333	rural/clinic	No	08/06/2017	0	0	0
2012249937	333	rural/clinic	No	08/06/2017	0	0	0
2014383529	333	rural/clinic	No	08/06/2017	0	0	0
2012331324	333	rural/clinic	No	08/06/2017	0	0	0
2012259891	333	rural/clinic	No	08/06/2017	0	0	0

2015406282	333	rural/clinic	No	08/06/2017	0	0	0
2012336632	333	rural/clinic	No	08/06/2017	0	0	0
2015406268	333	rural/clinic	No	08/06/2017	0	0	0
2014370436	333	rural/clinic	No	08/06/2017	0	0	0
2012259894	333	rural/clinic	No	08/06/2017	0	0	0
2015406265	333	rural/clinic	No	08/06/2017	0	0	0
201053908	333	rural/clinic	No	08/06/2017	0	0	0
2015406269	333	rural/clinic	No	08/06/2017	0	0	0
2015406261	697	rural/clinic	No	08/06/2017	0	0	0
2014317635	697	rural/clinic	No	08/06/2017	0	0	0
2012259895	697	rural/clinic	No	08/06/2017	0	0	0
2015319258	697	rural/clinic	No	08/06/2017	0	0	0
2015374452	697	rural/clinic	No	08/06/2017	0	0	0
2015406263	697	rural/clinic	No	08/06/2017	0	0	0
2015340120	697	rural/clinic	No	08/06/2017	0	0	0
2015319259	697	rural/clinic	No	08/06/2017	0	0	0
2015406270	697	rural/clinic	No	08/06/2017	0	0	0
2015319260	697	rural/clinic	No	08/06/2017	0	0	0
2015374453	697	rural/clinic	No	08/06/2017	0	0	0
2015406286	697	rural/clinic	No	08/06/2017	0	0	0
2015319261	697	rural/clinic	No	08/06/2017	0	0	0
2015406285	697	rural/clinic	No	08/06/2017	0	0	0
2015340121	697	rural/clinic	No	08/06/2017	0	0	0
2015319262	697	rural/clinic	No	08/06/2017	0	0	0
2015406287	697	rural/clinic	No	08/06/2017	0	0	0
2015406289	697	rural/clinic	No	08/06/2017	0	0	0
2015319263	697	rural/clinic	No	08/06/2017	0	0	0
2015406288	697	rural/clinic	No	08/06/2017	0	0	0
2015319264	697	rural/clinic	No	08/06/2017	0	0	0
2015406283	697	rural/clinic	No	08/06/2017	0	0	0
2015340122	697	rural/clinic	No	08/06/2017	0	0	0
2015319265	697	rural/clinic	No	08/06/2017	0	0	0
2015319266	697	rural/clinic	No	08/06/2017	0	0	0
2015340986	697	rural/clinic	No	08/06/2017	0	0	0
2015340988	363	rural/clinic	No	08/06/2017	0	0	0
2015340123	363	rural/clinic	No	08/06/2017	0	0	0
2015369051	363	rural/clinic	No	08/06/2017	0	0	0
2015374454	363	rural/clinic	No	08/06/2017	0	0	0
2015340987	363	rural/clinic	No	08/06/2017	0	0	0
2015340989	363	rural/clinic	No	08/06/2017	0	0	0
2015374455	363	rural/clinic	No	08/06/2017	0	0	0
2015369052	363	rural/clinic	No	08/06/2017	0	0	0
2015340990	363	rural/clinic	No	08/06/2017	0	0	0
2015374458	894	rural/clinic	No	08/06/2017	0	0	0
2015415275	894	rural/clinic	No	08/06/2017	0	0	0
2015301270	894	rural/clinic	No	08/06/2017	0	0	0
2015415276	894	rural/clinic	No	08/06/2017	0	0	0
2015340013	894	rural/clinic	No	08/06/2017	0	0	0
2014325203	894	rural/clinic	No	08/06/2017	0	0	0
2015340012	894	rural/clinic	No	08/06/2017	0	0	0
2015415278	894	rural/clinic	No	08/06/2017	0	0	0
2015391530	894	rural/clinic	No	08/06/2017	0	0	0
2015415916	894	rural/clinic	No	08/06/2017	0	0	0
2015301487	894	rural/clinic	No	08/06/2017	0	0	0
2015391528	894	rural/clinic	No	08/06/2017	0	0	0
2015415277	894	rural/clinic	No	08/06/2017	0	0	0
2015301486	894	rural/clinic	No	08/06/2017	0	0	0
2011203849	894	rural/clinic	No	08/06/2017	0	0	0
2015340099	639	rural/clinic	No	08/06/2017	0	0	0
2011203848	639	rural/clinic	No	08/06/2017	0	0	0
2015325251	639	rural/clinic	No	08/06/2017	0	0	0
2014358278	639	rural/clinic	No	08/06/2017	0	0	0
2015325201	639	rural/clinic	No	08/06/2017	0	0	0
2014327127	639	rural/clinic	No	08/06/2017	0	0	0
2015415280	639	rural/clinic	No	08/06/2017	0	0	0
2015302434	639	rural/clinic	No	08/06/2017	0	0	0
2015415279	639	rural/clinic	No	08/06/2017	0	0	0
2012312202	639	rural/clinic	No	08/06/2017	0	0	0
2015391882	639	rural/clinic	No	08/06/2017	0	0	0
2014358276	639	rural/clinic	No	08/06/2017	0	0	0
2015357532	639	rural/clinic	No	08/06/2017	0	0	0
2012284228	639	rural/clinic	No	08/06/2017	0	0	0
2014348818	639	rural/clinic	No	08/06/2017	0	0	0
2012306149	639	rural/clinic	No	08/06/2017	0	0	0
2015331180	639	rural/clinic	No	08/06/2017	0	0	0
2015302429	639	rural/clinic	No	08/06/2017	0	0	0
2015340124	639	rural/clinic	No	08/06/2017	0	0	0
2014349919	639	rural/clinic	No	08/06/2017	0	0	0
2012284905	639	rural/clinic	No	08/06/2017	0	0	0
2012306148	639	rural/clinic	No	08/06/2017	0	0	0
2015340125	639	rural/clinic	No	08/06/2017	0	0	0
2012263203	639	rural/clinic	No	08/06/2017	0	0	0

2014334679	639	rural/clinic	No	08/06/2017	0	0	0
2015340126	187	rural/clinic	No	08/06/2017	0	0	0
2014334367	187	rural/clinic	No	08/06/2017	0	0	0
2015340127	187	rural/clinic	No	08/06/2017	0	0	0
2014334369	187	rural/clinic	No	08/06/2017	0	0	0
2015345056	187	rural/clinic	No	08/06/2017	0	0	0
2015340128	187	rural/clinic	No	08/06/2017	0	0	0
2015345057	187	rural/clinic	No	08/06/2017	0	0	0
2015359607	187	rural/clinic	No	08/06/2017	0	0	0
2015345058	187	rural/clinic	No	08/06/2017	0	0	0
2014347020	187	rural/clinic	No	08/06/2017	0	0	0
2012306147	187	rural/clinic	No	08/06/2017	0	0	0
2015302430	187	rural/clinic	No	08/06/2017	0	0	0
2015384546	187	rural/clinic	No	08/06/2017	0	0	0
2015400066	187	rural/clinic	No	08/06/2017	0	0	0
2015302431	187	rural/clinic	No	08/06/2017	0	0	0
2015400067	187	rural/clinic	No	08/06/2017	0	0	0
2015377176	187	rural/clinic	No	08/06/2017	0	0	0
2015340098	187	rural/clinic	No	08/06/2017	0	0	0
2015400068	187	rural/clinic	No	08/06/2017	0	0	0
2015302433	187	rural/clinic	No	08/06/2017	0	0	0
2015400069	187	rural/clinic	No	08/06/2017	0	0	0
2015340097	187	rural/clinic	No	08/06/2017	0	0	0
2015377327	187	rural/clinic	No	08/06/2017	0	0	0
2015400070	187	rural/clinic	No	08/06/2017	0	0	0
2015340096	187	rural/clinic	No	08/06/2017	0	0	0
2015400071	187	rural/clinic	No	08/06/2017	0	0	0
2015302432	187	rural/clinic	No	08/06/2017	0	0	0
2015359608	187	rural/clinic	No	08/06/2017	0	0	0
2014350100	187	rural/clinic	No	08/06/2017	0	0	0
2012312201	187	rural/clinic	No	08/06/2017	0	0	0
2012284902	187	rural/clinic	No	08/06/2017	0	0	0
2015369191	187	rural/clinic	No	08/06/2017	0	0	0
2015400072	187	rural/clinic	No	08/06/2017	0	0	0
2015335133	187	rural/clinic	No	08/06/2017	0	0	0
2014328233	187	rural/clinic	No	08/06/2017	0	0	0
2015400073	187	rural/clinic	No	08/06/2017	0	0	0
2012243651	187	rural/clinic	No	08/06/2017	0	0	0
2011123781	187	rural/clinic	No	08/06/2017	0	0	0
2015384047	187	rural/clinic	No	08/06/2017	0	0	0
2015400074	187	rural/clinic	No	08/06/2017	0	0	0
2012344081	187	rural/clinic	No	08/06/2017	0	0	0
2015359609	187	rural/clinic	No	08/06/2017	0	0	0
2014328234	187	rural/clinic	No	08/06/2017	0	0	0
2015335135	187	rural/clinic	No	08/06/2017	0	0	0
2014328232	187	rural/clinic	No	08/06/2017	0	0	0
2015400075	187	rural/clinic	No	08/06/2017	0	0	0
2015335136	187	rural/clinic	No	08/06/2017	0	0	0
2012344503	187	rural/clinic	No	08/06/2017	0	0	0
2015400076	187	rural/clinic	No	08/06/2017	0	0	0
2015400077	187	rural/clinic	No	08/06/2017	0	0	0
2012243653	187	rural/clinic	No	08/06/2017	0	0	0
2014328231	187	rural/clinic	No	08/06/2017	0	0	0
2015335137	187	rural/clinic	No	08/06/2017	0	0	0
2014302524	187	rural/clinic	No	08/06/2017	0	0	0
2015363101	187	rural/clinic	No	08/06/2017	0	0	0
2015386708	187	rural/clinic	No	08/06/2017	0	0	0
2015400078	187	rural/clinic	No	08/06/2017	0	0	0
2015386709	187	rural/clinic	No	08/06/2017	0	0	0
2014314473	187	rural/clinic	No	08/06/2017	0	0	0
2015386710	187	rural/clinic	No	08/06/2017	0	0	0
2015400079	187	rural/clinic	No	08/06/2017	0	0	0
2015400080	187	rural/clinic	No	08/06/2017	0	0	0
2015314607	187	rural/clinic	No	08/06/2017	0	0	0
2015400081	187	rural/clinic	No	08/06/2017	0	0	0
2015400082	187	rural/clinic	No	08/06/2017	0	0	0
2015400083	187	rural/clinic	No	08/06/2017	0	0	0
2015400084	187	rural/clinic	No	08/06/2017	0	0	0
2015400085	187	rural/clinic	No	08/06/2017	0	0	0
2015400086	187	rural/clinic	No	08/06/2017	0	0	0
2015400087	187	rural/clinic	No	08/06/2017	0	0	0
2015400089	187	rural/clinic	No	08/06/2017	0	0	0
2015400090	187	rural/clinic	No	08/06/2017	0	0	0
2015400091	187	rural/clinic	No	08/06/2017	0	0	0
2015400092	187	rural/clinic	No	08/06/2017	0	0	0
2015400093	187	rural/clinic	No	08/06/2017	0	0	0
2014346731	187	rural/clinic	No	08/06/2017	0	0	0
2014346732	187	rural/clinic	No	08/06/2017	0	0	0
2015360515	187	rural/clinic	No	08/06/2017	0	0	0
2015360516	187	rural/clinic	No	08/06/2017	0	0	0
2015360517	583	rural/clinic	No	08/06/2017	0	0	0
2011204478	583	rural/clinic	No	08/06/2017	0	0	0

2011204479	583	rural/clinic	No	08/06/2017	0	0	0
2013258608	583	rural/clinic	No	08/06/2017	0	0	0
2015332357	583	rural/clinic	No	08/06/2017	0	0	0
2015332356	583	rural/clinic	No	08/06/2017	0	0	0
2015332358	583	rural/clinic	No	08/06/2017	0	0	0
2015332355	583	rural/clinic	No	08/06/2017	0	0	0
2015332354	583	rural/clinic	No	08/06/2017	0	0	0
2015332353	583	rural/clinic	No	08/06/2017	0	0	0
2015332359	583	rural/clinic	No	08/06/2017	0	0	0
2015332360	583	rural/clinic	No	08/06/2017	0	0	0
2014377625	583	rural/clinic	No	08/06/2017	0	0	0
2015340215	583	rural/clinic	No	08/06/2017	0	0	0
2014302525	583	rural/clinic	No	08/06/2017	0	0	0
2014358017	583	rural/clinic	No	06/06/2017	0	0	0
2014358017	583	rural/clinic	No	06/06/2017	0	0	0
2014358015	583	rural/clinic	No	06/06/2017	0	0	0
2014358015	583	rural/clinic	No	06/06/2017	0	0	0
2011140761	583	rural/clinic	No	06/06/2017	0	0	0
2015314606	583	rural/clinic	No	08/06/2017	0	0	0
2015404251	583	rural/clinic	No	08/06/2017	0	0	0
2014290324	583	rural/clinic	No	01/06/2017	0	0	0
2014290324	583	rural/clinic	No	01/06/2017	0	0	0
2012275989	583	rural/clinic	No	01/06/2017	0	0	0
2012275989	583	rural/clinic	No	01/06/2017	0	0	0
2014363888	583	rural/clinic	No	02/06/2017	0	0	0
2014363888	583	rural/clinic	No	02/06/2017	0	0	0
2015286872	583	rural/clinic	No	02/06/2017	0	0	0
2015286872	583	rural/clinic	No	02/06/2017	0	0	0
2012276256	583	rural/clinic	No	02/06/2017	0	0	0
2012276256	583	rural/clinic	No	02/06/2017	0	0	0
2012289620	583	rural/clinic	No	02/06/2017	0	0	0
2012289620	583	rural/clinic	No	02/06/2017	0	0	0
2014363886	583	rural/clinic	No	02/06/2017	0	0	0
2014363886	583	rural/clinic	No	02/06/2017	0	0	0
2015334539	583	rural/clinic	No	06/06/2017	0	0	0
2015334539	583	rural/clinic	No	06/06/2017	0	0	0
2014371833	583	rural/clinic	No	06/06/2017	0	0	0
2014371833	583	rural/clinic	No	06/06/2017	0	0	0
2015363102	583	rural/clinic	No	08/06/2017	0	0	0
2015334209	583	rural/clinic	No	06/06/2017	0	0	0
2015334209	583	rural/clinic	No	06/06/2017	0	0	0
2015344327	583	rural/clinic	No	06/06/2017	0	0	0
2015344327	583	rural/clinic	No	06/06/2017	0	0	0
2015344326	583	rural/clinic	No	06/06/2017	0	0	0
2015344326	583	rural/clinic	No	06/06/2017	0	0	0
2014314474	583	rural/clinic	No	08/06/2017	0	0	0
2015386711	583	rural/clinic	No	08/06/2017	0	0	0
2015386712	583	rural/clinic	No	08/06/2017	0	0	0
2011141073	583	rural/clinic	No	08/06/2017	0	0	0
2015386713	583	rural/clinic	No	08/06/2017	0	0	0
2015303480	583	rural/clinic	No	08/06/2017	0	0	0
2015402398	583	rural/clinic	No	08/06/2017	0	0	0
2015402399	583	rural/clinic	No	08/06/2017	0	0	0
2015414348	583	rural/clinic	No	06/06/2017	0	0	0
2015414252	583	rural/clinic	No	06/06/2017	0	0	0
201066585	583	rural/clinic	No	06/06/2017	0	0	0
201066585	583	rural/clinic	No	06/06/2017	0	0	0
2015340216	583	rural/clinic	No	08/06/2017	0	0	0
2015402400	583	rural/clinic	No	08/06/2017	0	0	0
201053909	583	rural/clinic	No	08/06/2017	0	0	0
2014317348	583	rural/clinic	No	08/06/2017	0	0	0
2014305945	583	rural/clinic	No	29/05/2017	0	0	0
2015339464	583	rural/clinic	No	08/06/2017	0	0	0
2014301467	583	rural/clinic	No	08/06/2017	0	0	0
2012284904	583	rural/clinic	No	08/06/2017	0	0	0
2012284903	529	rural/clinic	No	08/06/2017	0	0	0
2015406284	529	rural/clinic	No	08/06/2017	0	0	0
2012348841	529	rural/clinic	No	08/06/2017	0	0	0
2015356580	529	rural/clinic	No	12/06/2017	0	0	0
2015290206	529	rural/clinic	No	09/06/2017	0	0	0
2012348840	529	rural/clinic	No	19/04/2017	0	0	0
2012348844	529	rural/clinic	No	08/06/2017	0	0	0
2014349187	426	rural/clinic	No	08/06/2017	0	0	0
2015412097	426	rural/clinic	No	09/06/2017	0	0	0
2014324378	426	rural/clinic	No	08/06/2017	0	0	0
2014298591	426	rural/clinic	No	09/06/2017	0	0	0
2011187520	426	rural/clinic	No	08/06/2017	0	0	0
2015335134	426	rural/clinic	No	08/06/2017	0	0	0
2012253931	426	rural/clinic	No	09/06/2017	0	0	0
2011226866	426	rural/clinic	No	08/06/2017	0	0	0
2014356999	426	rural/clinic	No	09/06/2017	0	0	0
2015377175	426	rural/clinic	No	08/06/2017	0	0	0

2014309385	426	rural/clinic	No	18/05/2017	0	0	0
2015375414	426	rural/clinic	No	09/06/2017	0	0	0
2015375417	426	rural/clinic	No	09/06/2017	0	0	0
2014364210	426	rural/clinic	No	08/06/2017	0	0	0
2014288140	426	rural/clinic	No	09/06/2017	0	0	0
2011141082	426	rural/clinic	No	08/06/2017	0	0	0
2015349017	426	rural/clinic	No	09/06/2017	0	0	0
2015303498	426	rural/clinic	No	08/06/2017	0	0	0
2014310209	426	rural/clinic	No	09/06/2017	0	0	0
2015375415	426	rural/clinic	No	09/06/2017	0	0	0
2015375416	426	rural/clinic	No	09/06/2017	0	0	0
2011141081	426	rural/clinic	No	08/06/2017	0	0	0
2015415651	426	rural/clinic	No	08/06/2017	0	0	0
2011141076	426	rural/clinic	No	08/06/2017	0	0	0
2015415652	426	rural/clinic	No	08/06/2017	0	0	0
2015415653	426	rural/clinic	No	09/06/2017	0	0	0
2011141075	426	rural/clinic	No	08/06/2017	0	0	0
2015415654	426	rural/clinic	No	08/06/2017	0	0	0
2011141079	426	rural/clinic	No	08/06/2017	0	0	0
2015415655	426	rural/clinic	No	08/06/2017	0	0	0
2011141080	426	rural/clinic	No	08/06/2017	0	0	0
2011155233	426	rural/clinic	No	08/06/2017	0	0	0
2011141074	426	rural/clinic	No	08/06/2017	0	0	0
2015372024	426	rural/clinic	No	08/06/2017	0	0	0
2015372025	426	rural/clinic	No	08/06/2017	0	0	0
2011141078	426	rural/clinic	No	08/06/2017	0	0	0
2011141077	481	rural/clinic	No	08/06/2017	0	0	0
2015373430	481	rural/clinic	No	08/06/2017	0	0	0
2015373431	481	rural/clinic	No	08/06/2017	0	0	0
2015373432	481	rural/clinic	No	08/06/2017	0	0	0
2015373433	481	rural/clinic	No	08/06/2017	0	0	0
2015373434	481	rural/clinic	No	08/06/2017	0	0	0
2015373435	481	rural/clinic	No	08/06/2017	0	0	0
2011141084	481	rural/clinic	No	08/06/2017	0	0	0
2015373436	481	rural/clinic	No	08/06/2017	0	0	0
2014288976	481	rural/clinic	No	08/06/2017	0	0	0
2015373437	481	rural/clinic	No	08/06/2017	0	0	0
2011110729/D	481	rural/clinic	No	08/06/2017	0	0	0
2015366429	481	rural/clinic	No	08/06/2017	0	0	0
2015373438	481	rural/clinic	No	08/06/2017	0	0	0
2014364209	481	rural/clinic	No	08/06/2017	0	0	0
2015373439	481	rural/clinic	No	08/06/2017	0	0	0
2014364202	481	rural/clinic	No	08/06/2017	0	0	0
2014364203	481	rural/clinic	No	08/06/2017	0	0	0
2011226865	481	rural/clinic	No	08/06/2017	0	0	0
2014364204	481	rural/clinic	No	08/06/2017	0	0	0
2014347021	481	rural/clinic	No	08/06/2017	0	0	0
2015335384	481	rural/clinic	No	08/06/2017	0	0	0
2014347023	481	rural/clinic	No	08/06/2017	0	0	0
2014364205	481	rural/clinic	No	08/06/2017	0	0	0
2014347024	481	rural/clinic	No	08/06/2017	0	0	0
2014364206	481	rural/clinic	No	08/06/2017	0	0	0
2014364207	282	district/faith-based	No	08/06/2017	1	0	0
2015384547	282	district/faith-based	No	08/06/2017	1	0	0
2014364208	282	district/faith-based	No	08/06/2017	1	0	0
2011226867	282	district/faith-based	No	08/06/2017	1	0	0
2015405551	282	district/faith-based	No	08/06/2017	1	0	0
2014347019	282	district/faith-based	No	08/06/2017	1	0	0
2015405552	282	district/faith-based	No	08/06/2017	1	0	0
2015321807	282	district/faith-based	No	08/06/2017	1	0	0
2011144111	282	district/faith-based	No	08/06/2017	1	0	0
2015344895	282	district/faith-based	No	08/06/2017	1	0	0
2014297348	282	district/faith-based	No	08/06/2017	1	0	0
2015335204	282	district/faith-based	No	08/06/2017	1	0	0
2015344896	282	district/faith-based	No	08/06/2017	1	0	0
2015377216	282	district/faith-based	No	08/06/2017	1	0	0
2015413915	282	district/faith-based	No	08/06/2017	1	0	0
2015377217	282	district/faith-based	No	08/06/2017	1	0	0
2015413916	282	district/faith-based	No	08/06/2017	1	0	0
2015352753	282	district/faith-based	No	08/06/2017	1	0	0
2015352754	282	district/faith-based	No	08/06/2017	1	0	0
2015413917	282	district/faith-based	No	08/06/2017	1	0	0
2015352755	282	district/faith-based	No	08/06/2017	1	0	0
2015413918	282	district/faith-based	No	08/06/2017	1	0	0
2015413919	282	district/faith-based	No	08/06/2017	1	0	0
2015352756	282	district/faith-based	No	08/06/2017	1	0	0
2015413920	282	district/faith-based	No	08/06/2017	1	0	0
2015352757	282	district/faith-based	No	08/06/2017	1	0	0
2015290204	282	district/faith-based	No	09/06/2017	1	0	0
2015352758	282	district/faith-based	No	08/06/2017	1	0	0
2015290205	282	district/faith-based	No	09/06/2017	1	0	0
2015362528	282	district/faith-based	No	08/06/2017	1	0	0

2014288011	282	district/faith-based	No	09/06/2017	1	0	0
2015362526	282	district/faith-based	No	08/06/2017	1	0	0
2011235684	282	district/faith-based	No	08/06/2017	1	0	0
2011192490	282	district/faith-based	No	08/06/2017	1	0	0
2015362524	282	district/faith-based	No	08/06/2017	1	0	0
2015339465	282	district/faith-based	No	08/06/2017	1	0	0
2015362527	282	district/faith-based	No	08/06/2017	1	0	0
2014296327	282	district/faith-based	No	08/06/2017	1	0	0
2015339468	282	district/faith-based	No	08/06/2017	1	0	0
2015339466	282	district/faith-based	No	08/06/2017	1	0	0
2011157148	282	district/faith-based	No	08/06/2017	1	0	0
2015339023	282	district/faith-based	No	08/06/2017	1	0	0
2013264070	282	district/faith-based	No	08/06/2017	1	0	0
2015414560	282	district/faith-based	No	08/06/2017	1	0	0
2015339467	282	district/faith-based	No	08/06/2017	1	0	0
2012344501	282	district/faith-based	No	08/06/2017	1	0	0
2014338557	282	district/faith-based	No	08/06/2017	1	0	0
2015326592	282	district/faith-based	No	08/06/2017	1	0	0
2015326593	282	district/faith-based	No	08/06/2017	1	0	0
2015286709	282	district/faith-based	No	08/06/2017	1	0	0
2015286710	282	district/faith-based	No	08/06/2017	1	0	0
2013273397	282	district/faith-based	No	08/06/2017	1	0	0
2014289658	282	district/faith-based	No	08/06/2017	1	0	0
2014338558	282	district/faith-based	No	08/06/2017	1	0	0
2011198695	282	district/faith-based	No	08/06/2017	1	0	0
2014358340	282	district/faith-based	No	08/06/2017	1	0	0
2011198693	282	district/faith-based	No	08/06/2017	1	0	0
2015364521	282	district/faith-based	No	08/06/2017	1	0	0
2013270349	282	district/faith-based	No	08/06/2017	1	0	0
2012369812	282	district/faith-based	No	08/06/2017	1	0	0
2014378734	282	district/faith-based	No	08/06/2017	1	0	0
2011198694	282	district/faith-based	No	08/06/2017	1	0	0
2014378732	282	district/faith-based	No	08/06/2017	1	0	0
2015417064	282	district/faith-based	No	08/06/2017	1	0	0
2014287179	282	district/faith-based	No	08/06/2017	1	0	0
2014317347	282	district/faith-based	No	08/06/2017	1	0	0
2015417063	282	district/faith-based	No	08/06/2017	1	0	0
2015415058	282	district/faith-based	No	08/06/2017	1	0	0
2015415061	282	district/faith-based	No	08/06/2017	1	0	0
2015417067	282	district/faith-based	No	08/06/2017	1	0	0
2015415060	282	district/faith-based	No	08/06/2017	1	0	0
2015417066	282	district/faith-based	No	08/06/2017	1	0	0
2015415059	282	district/faith-based	No	08/06/2017	1	0	0
2015417065	282	district/faith-based	No	08/06/2017	1	0	0
2014303097	282	district/faith-based	No	08/06/2017	1	0	0
2014303098	282	district/faith-based	No	08/06/2017	1	0	0
2015417062	282	district/faith-based	No	08/06/2017	1	0	0
2014303099	282	district/faith-based	No	08/06/2017	1	0	0
2014303095	282	district/faith-based	No	08/06/2017	1	0	0
2015417061	282	district/faith-based	No	08/06/2017	1	0	0
2014303096	282	district/faith-based	No	08/06/2017	1	0	0
2014301469	282	district/faith-based	No	08/06/2017	1	0	0
2015290029	282	district/faith-based	No	09/06/2017	1	0	0
2012253973	282	district/faith-based	No	09/06/2017	1	0	0
2013258730	282	district/faith-based	No	09/06/2017	1	0	0
2015332971	282	district/faith-based	No	09/06/2017	1	0	0
2015316008	282	district/faith-based	No	08/06/2017	1	0	0
2015316011	282	district/faith-based	No	09/06/2017	1	0	0
2015316012	282	district/faith-based	No	08/06/2017	1	0	0
2015316013	282	district/faith-based	No	09/06/2017	1	0	0
2010098885	282	district/faith-based	No	09/06/2017	1	0	0
2015363068	143	rural/clinic	No	06/06/2017	0	0	0
2015363068	143	rural/clinic	No	06/06/2017	0	0	0
2015363067	143	rural/clinic	No	06/06/2017	0	0	0
2015363067	143	rural/clinic	No	06/06/2017	0	0	0
2014306028	143	rural/clinic	No	06/06/2017	0	0	0
2014306028	143	rural/clinic	No	06/06/2017	0	0	0
2015293950	143	rural/clinic	No	06/06/2017	0	0	0
2015293950	143	rural/clinic	No	06/06/2017	0	0	0
2015330481	143	rural/clinic	No	07/06/2017	0	0	0
2015330481	143	rural/clinic	No	07/06/2017	0	0	0
2013276156	143	rural/clinic	No	07/06/2017	0	0	0
2013276156	143	rural/clinic	No	07/06/2017	0	0	0
2015301818	143	rural/clinic	No	07/06/2017	0	0	0
2015301818	143	rural/clinic	No	07/06/2017	0	0	0
2014376896	143	rural/clinic	No	07/06/2017	0	0	0
2014376896	143	rural/clinic	No	07/06/2017	0	0	0
2011135670	143	rural/clinic	No	07/06/2017	0	0	0
2011135670	143	rural/clinic	No	07/06/2017	0	0	0
2015293842	143	rural/clinic	No	07/06/2017	0	0	0
2015293842	143	rural/clinic	No	07/06/2017	0	0	0
2015400073	143	rural/clinic	No	08/06/2017	0	0	0

2015400073	143	rural/clinic	No	08/06/2017	0	0	0
2015400081	143	rural/clinic	No	08/06/2017	0	0	0
2015400082	143	rural/clinic	No	08/06/2017	0	0	0
2015400082	143	rural/clinic	No	08/06/2017	0	0	0
2015415272	143	rural/clinic	No	08/06/2017	0	0	0
2015415272	143	rural/clinic	No	08/06/2017	0	0	0
2015415277	143	rural/clinic	No	08/06/2017	0	0	0
2012259891	306	rural/clinic	No	08/06/2017	0	0	0
2012259891	306	rural/clinic	No	08/06/2017	0	0	0
2015337985	306	rural/clinic	No	07/06/2017	0	0	0
2015337985	306	rural/clinic	No	07/06/2017	0	0	0
2015320888	306	rural/clinic	No	06/06/2017	0	0	0
2015320888	306	rural/clinic	No	06/06/2017	0	0	0
2012293006	306	rural/clinic	No	06/06/2017	0	0	0
2012293006	306	rural/clinic	No	06/06/2017	0	0	0
2015320887	306	rural/clinic	No	06/06/2017	0	0	0
2015320887	306	rural/clinic	No	06/06/2017	0	0	0
2015352371	306	rural/clinic	No	13/06/2017	0	0	0
2015357533	306	rural/clinic	No	13/06/2017	0	0	0
2015352372	306	rural/clinic	No	13/06/2017	0	0	0
2014320297	306	rural/clinic	No	13/06/2017	0	0	0
2015325934	306	rural/clinic	No	13/06/2017	0	0	0
2014349920	306	rural/clinic	No	13/06/2017	0	0	0
2012306150	306	rural/clinic	No	13/06/2017	0	0	0
2015332420	306	rural/clinic	No	13/06/2017	0	0	0
2015368589	306	rural/clinic	No	13/06/2017	0	0	0
2015367216	306	rural/clinic	No	13/06/2017	0	0	0
2015368590	306	rural/clinic	No	13/06/2017	0	0	0
2015367217	306	rural/clinic	No	13/06/2017	0	0	0
2015368591	306	rural/clinic	No	13/06/2017	0	0	0
2015303089	306	rural/clinic	No	13/06/2017	0	0	0
2015287378	306	rural/clinic	No	13/06/2017	0	0	0
2011142495	306	rural/clinic	No	13/06/2017	0	0	0
2015287379	306	rural/clinic	No	13/06/2017	0	0	0
2015360732	306	rural/clinic	No	13/06/2017	0	0	0
2015287376	306	rural/clinic	No	13/06/2017	0	0	0
2014347818	306	rural/clinic	No	13/06/2017	0	0	0
2015287375	306	rural/clinic	No	13/06/2017	0	0	0
2015287377	306	rural/clinic	No	13/06/2017	0	0	0
2014347819	306	rural/clinic	No	14/06/2017	0	0	0
2014336900	306	rural/clinic	No	13/06/2017	0	0	0
2015401851	306	rural/clinic	No	13/06/2017	0	0	0
2015293365	306	rural/clinic	No	13/06/2017	0	0	0
2015321601	306	rural/clinic	No	13/06/2017	0	0	0
2015401852	306	rural/clinic	No	13/06/2017	0	0	0
2015360734	306	rural/clinic	No	13/06/2017	0	0	0
2015401853	306	rural/clinic	No	13/06/2017	0	0	0
2014365159	306	rural/clinic	No	13/06/2017	0	0	0
2015360733	306	rural/clinic	No	13/06/2017	0	0	0
2015401854	306	rural/clinic	No	13/06/2017	0	0	0
2014365162	306	rural/clinic	No	13/06/2017	0	0	0
2012261384	306	rural/clinic	No	13/06/2017	0	0	0
2014303100	306	rural/clinic	No	13/06/2017	0	0	0
2014365161	306	rural/clinic	No	13/06/2017	0	0	0
2014374024	306	rural/clinic	No	13/06/2017	0	0	0
2015360601	306	rural/clinic	No	13/06/2017	0	0	0
2014374023	306	rural/clinic	No	13/06/2017	0	0	0
2014374022	306	rural/clinic	No	13/06/2017	0	0	0
2015331778	306	rural/clinic	No	13/06/2017	0	0	0
2013249283	306	rural/clinic	No	13/06/2017	0	0	0
2015342291	306	rural/clinic	No	13/06/2017	0	0	0
2012276257	386	rural/clinic	No	13/06/2017	0	0	0
2014289658	386	rural/clinic	No	08/06/2017	0	0	0
2014289658	386	rural/clinic	No	08/06/2017	0	0	0
2015367215	386	rural/clinic	No	13/06/2017	0	0	0
2014338451	386	rural/clinic	No	13/06/2017	0	0	0
2012321339	386	rural/clinic	No	13/06/2017	0	0	0
2014337223	386	rural/clinic	No	13/06/2017	0	0	0
2014338452	386	rural/clinic	No	13/06/2017	0	0	0
2014314475	386	rural/clinic	No	13/06/2017	0	0	0
2014363889	386	rural/clinic	No	13/06/2017	0	0	0
2014314476	386	rural/clinic	No	13/06/2017	0	0	0
2015368714	386	rural/clinic	No	13/06/2017	0	0	0
2011212965	386	rural/clinic	No	13/06/2017	0	0	0
2015303088	386	rural/clinic	No	13/06/2017	0	0	0
2015368717	266	rural/clinic	No	13/06/2017	0	0	0
2015368715	266	rural/clinic	No	13/06/2017	0	0	0
2015384812	266	rural/clinic	No	13/06/2017	0	0	0
2014288577	266	rural/clinic	No	13/06/2017	0	0	0
2015362500	266	rural/clinic	No	13/06/2017	0	0	0
2015384813	266	rural/clinic	No	13/06/2017	0	0	0
2015344332	266	rural/clinic	No	13/06/2017	0	0	0

2015335597	266	rural/clinic	No	13/06/2017	0	0	0
2014311371	266	rural/clinic	No	13/06/2017	0	0	0
2012259148	266	rural/clinic	No	13/06/2017	0	0	0
2014363651	266	rural/clinic	No	13/06/2017	0	0	0
2015377898	266	rural/clinic	No	13/06/2017	0	0	0
2015383728	266	rural/clinic	No	13/06/2017	0	0	0
2013264391	266	rural/clinic	No	13/06/2017	0	0	0
2015412455	266	rural/clinic	No	13/06/2017	0	0	0
2014382921	266	rural/clinic	No	13/06/2017	0	0	0
2014382922	266	rural/clinic	No	13/06/2017	0	0	0
2014335901	698	rural/clinic	No	13/06/2017	0	0	0
2011192844	698	rural/clinic	No	13/06/2017	0	0	0
2011192845	698	rural/clinic	No	13/06/2017	0	0	0
2015342314	698	rural/clinic	No	13/06/2017	0	0	0
2015342289	698	rural/clinic	No	13/06/2017	0	0	0
2015342290	698	rural/clinic	No	13/06/2017	0	0	0
2014319635	815	rural/clinic	No	13/06/2017	0	0	0
2013261933	815	rural/clinic	No	13/06/2017	0	0	0
2015303079	815	rural/clinic	No	13/06/2017	0	0	0
2015413215	815	rural/clinic	No	13/06/2017	0	0	0
2015367211	815	rural/clinic	No	13/06/2017	0	0	0
2015413214	815	rural/clinic	No	13/06/2017	0	0	0
2015303076	815	rural/clinic	No	13/06/2017	0	0	0
2013261936	815	rural/clinic	No	13/06/2017	0	0	0
2015303087	815	rural/clinic	No	13/06/2017	0	0	0
2015303078	815	rural/clinic	No	13/06/2017	0	0	0
2015405623	815	rural/clinic	No	13/06/2017	0	0	0
2015405624	815	rural/clinic	No	13/06/2017	0	0	0
2015367212	815	rural/clinic	No	13/06/2017	0	0	0
2015303081	815	rural/clinic	No	13/06/2017	0	0	0
2015326407	815	rural/clinic	No	13/06/2017	0	0	0
2012290399	815	rural/clinic	No	13/06/2017	0	0	0
2015326408	815	rural/clinic	No	13/06/2017	0	0	0
2017027	815	rural/clinic	No	18/05/2017	0	0	0
2015326409	815	rural/clinic	No	13/06/2017	0	0	0
2015326410	815	rural/clinic	No	13/06/2017	0	0	0
2015326411	815	rural/clinic	No	13/06/2017	0	0	0
2011144820	815	rural/clinic	No	13/06/2017	0	0	0
2015403176	815	rural/clinic	No	13/06/2017	0	0	0
2015403175	815	rural/clinic	No	13/06/2017	0	0	0
2015405726	815	rural/clinic	No	13/06/2017	0	0	0
2015413212	815	rural/clinic	No	13/06/2017	0	0	0
2011144821	815	rural/clinic	No	13/06/2017	0	0	0
2015303632	815	rural/clinic	No	13/06/2017	0	0	0
2015403178	815	rural/clinic	No	13/06/2017	0	0	0
2015303633	815	rural/clinic	No	13/06/2017	0	0	0
2015403177	815	rural/clinic	No	13/06/2017	0	0	0
2014289532	815	rural/clinic	No	13/06/2017	0	0	0
2015303634	815	rural/clinic	No	13/06/2017	0	0	0
2015303636	815	rural/clinic	No	13/06/2017	0	0	0
2015303637	815	rural/clinic	No	13/06/2017	0	0	0
2015303638	815	rural/clinic	No	13/06/2017	0	0	0
2015303639	815	rural/clinic	No	13/06/2017	0	0	0
2011141083	815	rural/clinic	No	13/06/2017	0	0	0
2015344331	815	rural/clinic	No	13/06/2017	0	0	0
2014290726	815	rural/clinic	No	13/06/2017	0	0	0
2015303640	507	district/faith-based	No	13/06/2017	1	0	0
2015344328	507	district/faith-based	No	13/06/2017	1	0	0
2014344537	507	district/faith-based	No	13/06/2017	1	0	0
2014338509	507	district/faith-based	No	13/06/2017	1	0	0
2015385851	507	district/faith-based	No	13/06/2017	1	0	0
2011223208	507	district/faith-based	No	13/06/2017	1	0	0
2014344535	507	district/faith-based	No	13/06/2017	1	0	0
2015344329	507	district/faith-based	No	13/06/2017	1	0	0
2014316058	507	district/faith-based	No	13/06/2017	1	0	0
2015324478	507	district/faith-based	No	13/06/2017	1	0	0
2014316059	507	district/faith-based	No	13/06/2017	1	0	0
2012291362	507	district/faith-based	No	13/06/2017	1	0	0
2013254809	507	district/faith-based	No	13/06/2017	1	0	0
2014338510	507	district/faith-based	No	13/06/2017	1	0	0
2012291363	507	district/faith-based	No	13/06/2017	1	0	0
2014289536	507	district/faith-based	No	13/06/2017	1	0	0
2014358217	507	district/faith-based	No	13/06/2017	1	0	0
2011144822	507	district/faith-based	No	13/06/2017	1	0	0
2015372928	507	district/faith-based	No	13/06/2017	1	0	0
2013254808	507	district/faith-based	No	13/06/2017	1	0	0
2015372929	507	district/faith-based	No	13/06/2017	1	0	0
2015372930	507	district/faith-based	No	13/06/2017	1	0	0
2011223210	507	district/faith-based	No	13/06/2017	1	0	0
2015339760	507	district/faith-based	No	13/06/2017	1	0	0
2015339761	507	district/faith-based	No	13/06/2017	1	0	0
2015339762	507	district/faith-based	No	13/06/2017	1	0	0

2015339763	507	district/faith-based	No	13/06/2017	1	0	0
2012363968	507	district/faith-based	No	13/06/2017	1	0	0
2012363969	507	district/faith-based	No	13/06/2017	1	0	0
2012362563	507	district/faith-based	No	13/06/2017	1	0	0
2014366073	507	district/faith-based	No	13/06/2017	1	0	0
2015372926	507	district/faith-based	No	13/06/2017	1	0	0
2015372927	507	district/faith-based	No	13/06/2017	1	0	0
2015314604	507	district/faith-based	No	13/06/2017	1	0	0
2014289535	507	district/faith-based	No	13/06/2017	1	0	0
2014290734	507	district/faith-based	No	13/06/2017	1	0	0
2011133151	507	district/faith-based	No	13/06/2017	1	0	0
2014290735	507	district/faith-based	No	13/06/2017	1	0	0
2014289530	507	district/faith-based	No	13/06/2017	1	0	0
2014289538	507	district/faith-based	No	13/06/2017	1	0	0
2012344856	507	district/faith-based	No	13/06/2017	1	0	0
2014289537	507	district/faith-based	No	13/06/2017	1	0	0
2015337370	507	district/faith-based	No	13/06/2017	1	0	0
2014289539	507	district/faith-based	No	13/06/2017	1	0	0
2011223211	507	district/faith-based	No	13/06/2017	1	0	0
2014290733	507	district/faith-based	No	13/06/2017	1	0	0
2015347701	507	district/faith-based	No	13/06/2017	1	0	0
2014344536	507	district/faith-based	No	13/06/2017	1	0	0
2014289534	507	district/faith-based	No	13/06/2017	1	0	0
2015314605	507	district/faith-based	No	13/06/2017	1	0	0
2014289533	507	district/faith-based	No	13/06/2017	1	0	0
2014289531	507	district/faith-based	No	13/06/2017	1	0	0
2015335595	507	district/faith-based	No	13/06/2017	1	0	0
2012361535	507	district/faith-based	No	13/06/2017	1	0	0
2015324038	507	district/faith-based	No	13/06/2017	1	0	0
2015335596	507	district/faith-based	No	13/06/2017	1	0	0
2012344857	507	district/faith-based	No	13/06/2017	1	0	0
2014307416	507	district/faith-based	No	13/06/2017	1	0	0
2011136126	507	district/faith-based	No	13/06/2017	1	0	0
2011136125	507	district/faith-based	No	13/06/2017	1	0	0
2011136124	507	district/faith-based	No	13/06/2017	1	0	0
2013266077	507	district/faith-based	No	13/06/2017	1	0	0
2011136119	507	district/faith-based	No	13/06/2017	1	0	0
2011136120	507	district/faith-based	No	13/06/2017	1	0	0
2015355519	507	district/faith-based	No	13/06/2017	1	0	0
2011136122	507	district/faith-based	No	13/06/2017	1	0	0
2012344855	507	district/faith-based	No	13/06/2017	1	0	0
2011223209	507	district/faith-based	No	13/06/2017	1	0	0
2011136121	507	district/faith-based	No	13/06/2017	1	0	0
2015314608	507	district/faith-based	No	13/06/2017	1	0	0
2015376661	507	district/faith-based	No	13/06/2017	1	0	0
2012388400	507	district/faith-based	No	13/06/2017	1	0	0
2015376660	507	district/faith-based	No	13/06/2017	1	0	0
2013254807	507	district/faith-based	No	13/06/2017	1	0	0
2015376659	507	district/faith-based	No	13/06/2017	1	0	0
2015324449	507	district/faith-based	No	13/06/2017	1	0	0
2015376657	507	district/faith-based	No	13/06/2017	1	0	0
2015338656	507	district/faith-based	No	13/06/2017	1	0	0
2014360970	507	district/faith-based	No	13/06/2017	1	0	0
2015324480	507	district/faith-based	No	13/06/2017	1	0	0
2011192846	507	district/faith-based	No	13/06/2017	1	0	0
2015337510	507	district/faith-based	No	13/06/2017	1	0	0
2012346720	507	district/faith-based	No	13/06/2017	1	0	0
2013254804	507	district/faith-based	No	13/06/2017	1	0	0
2015383729	507	district/faith-based	No	13/06/2017	1	0	0
2015314603	507	district/faith-based	No	13/06/2017	1	0	0
2013271696	507	district/faith-based	No	13/06/2017	1	0	0
2015324479	507	district/faith-based	No	13/06/2017	1	0	0
2014357324	507	district/faith-based	No	13/06/2017	1	0	0
2014368683	507	district/faith-based	No	13/06/2017	1	0	0
2015338657	507	district/faith-based	No	13/06/2017	1	0	0
2014368684	507	district/faith-based	No	13/06/2017	1	0	0
2014347739	507	district/faith-based	No	13/06/2017	1	0	0
2015334801	507	district/faith-based	No	13/06/2017	1	0	0
2015385852	507	district/faith-based	No	13/06/2017	1	0	0
2015405966	507	district/faith-based	No	13/06/2017	1	0	0
2012304334	507	district/faith-based	No	13/06/2017	1	0	0
2012330887	507	district/faith-based	No	13/06/2017	1	0	0
2012304333	507	district/faith-based	No	13/06/2017	1	0	0
2015363072	507	district/faith-based	No	13/06/2017	1	0	0
2011152133	507	district/faith-based	No	13/06/2017	1	0	0
2012304335	507	district/faith-based	No	13/06/2017	1	0	0
2011152134	507	district/faith-based	No	13/06/2017	1	0	0
2014297974	507	district/faith-based	No	13/06/2017	1	0	0
2015363071	507	district/faith-based	No	13/06/2017	1	0	0
2014297975	507	district/faith-based	No	13/06/2017	1	0	0
2015405413	507	district/faith-based	No	13/06/2017	1	0	0
2014365388	507	district/faith-based	No	13/06/2017	1	0	0

2014291817	507	district/faith-based	No	13/06/2017	1	0	0
2015414258	507	district/faith-based	No	13/06/2017	1	0	0
2015413362	507	district/faith-based	No	13/06/2017	1	0	0
2014365475	507	district/faith-based	No	13/06/2017	1	0	0
2014367625	507	district/faith-based	No	13/06/2017	1	0	0
2015405916	507	district/faith-based	No	13/06/2017	1	0	0
2015344655	507	district/faith-based	No	13/06/2017	1	0	0
2014375503	507	district/faith-based	No	13/06/2017	1	0	0
2015344656	507	district/faith-based	No	13/06/2017	1	0	0
2015369929	507	district/faith-based	No	13/06/2017	1	0	0
2014335572	507	district/faith-based	No	13/06/2017	1	0	0
2015368818	507	district/faith-based	No	13/06/2017	1	0	0
2014335573	507	district/faith-based	No	13/06/2017	1	0	0
2015368817	507	district/faith-based	No	13/06/2017	1	0	0
2014335574	507	district/faith-based	No	13/06/2017	1	0	0
2014335576	507	district/faith-based	No	13/06/2017	1	0	0
2014335577	507	district/faith-based	No	13/06/2017	1	0	0
2015302435	507	district/faith-based	No	13/06/2017	1	0	0
2010099627	507	district/faith-based	No	13/06/2017	1	0	0
2015325203	507	district/faith-based	No	13/06/2017	1	0	0
2015368816	507	district/faith-based	No	13/06/2017	1	0	0
2014309976	507	district/faith-based	No	13/06/2017	1	0	0
2015368892	507	district/faith-based	No	13/06/2017	1	0	0
2015414257	507	district/faith-based	No	13/06/2017	1	0	0
2015412309	507	district/faith-based	No	13/06/2017	1	0	0
2014375502	507	district/faith-based	No	13/06/2017	1	0	0
2015412311	507	district/faith-based	No	13/06/2017	1	0	0
2015339828	507	district/faith-based	No	13/06/2017	1	0	0
2015414256	507	district/faith-based	No	13/06/2017	1	0	0
2015412312	507	district/faith-based	No	13/06/2017	1	0	0
2015368891	507	district/faith-based	No	13/06/2017	1	0	0
2015412313	507	district/faith-based	No	13/06/2017	1	0	0
2015414259	507	district/faith-based	No	13/06/2017	1	0	0
2011137305	507	district/faith-based	No	13/06/2017	1	0	0
2011137306	507	district/faith-based	No	13/06/2017	1	0	0
2014371580	119	rural/clinic	No	13/06/2017	0	0	0
2011137310	119	rural/clinic	No	13/06/2017	0	0	0
2014371581	119	rural/clinic	No	13/06/2017	0	0	0
2014358191	119	rural/clinic	No	13/06/2017	0	0	0
2011137312	119	rural/clinic	No	13/06/2017	0	0	0
2014371582	119	rural/clinic	No	13/06/2017	0	0	0
2014358192	119	rural/clinic	No	13/06/2017	0	0	0
2011137309	119	rural/clinic	No	13/06/2017	0	0	0
2014371583	119	rural/clinic	No	13/06/2017	0	0	0
2014363887	119	rural/clinic	No	13/06/2017	0	0	0
2012288056	119	rural/clinic	No	13/06/2017	0	0	0
2014371584	119	rural/clinic	No	13/06/2017	0	0	0
2015289181	119	rural/clinic	No	13/06/2017	0	0	0
2012285887	119	rural/clinic	No	13/06/2017	0	0	0
2015289182	119	rural/clinic	No	13/06/2017	0	0	0
2014362830	119	rural/clinic	No	13/06/2017	0	0	0
2015289183	119	rural/clinic	No	13/06/2017	0	0	0
2015368979	119	rural/clinic	No	13/06/2017	0	0	0
2014362829	119	rural/clinic	No	13/06/2017	0	0	0
2015351987	119	rural/clinic	No	13/06/2017	0	0	0
2015362274	119	rural/clinic	No	13/06/2017	0	0	0
2015351986	119	rural/clinic	No	13/06/2017	0	0	0
2015351985	119	rural/clinic	No	13/06/2017	0	0	0
2015362275	119	rural/clinic	No	13/06/2017	0	0	0
2015351984	119	rural/clinic	No	13/06/2017	0	0	0
2015326957	119	rural/clinic	No	13/06/2017	0	0	0
2015351993	119	rural/clinic	No	13/06/2017	0	0	0
2012320390	119	rural/clinic	No	13/06/2017	0	0	0
2015351992	119	rural/clinic	No	13/06/2017	0	0	0
2012320391	119	rural/clinic	No	13/06/2017	0	0	0
2015351991	119	rural/clinic	No	13/06/2017	0	0	0
2015334946	119	rural/clinic	No	13/06/2017	0	0	0
2015334947	364	rural/clinic	No	13/06/2017	0	0	0
2015334945	364	rural/clinic	No	13/06/2017	0	0	0
2015334944	364	rural/clinic	No	13/06/2017	0	0	0
2015351988	364	rural/clinic	No	13/06/2017	0	0	0
2015338716	364	rural/clinic	No	13/06/2017	0	0	0
2015351990	364	rural/clinic	No	13/06/2017	0	0	0
2015351989	364	rural/clinic	No	13/06/2017	0	0	0
2015397648	364	rural/clinic	No	13/06/2017	0	0	0
2015397647	364	rural/clinic	No	13/06/2017	0	0	0
2015397646	364	rural/clinic	No	13/06/2017	0	0	0
2015397645	364	rural/clinic	No	13/06/2017	0	0	0
2015397644	364	rural/clinic	No	13/06/2017	0	0	0
2015397643	364	rural/clinic	No	13/06/2017	0	0	0
2014334181	364	rural/clinic	No	13/06/2017	0	0	0
2015372808	364	rural/clinic	No	13/06/2017	0	0	0

2015372809	364	rural/clinic	No	13/06/2017	0	0	0
2012320392	364	rural/clinic	No	13/06/2017	0	0	0
2015340100	364	rural/clinic	No	08/06/2017	0	0	0
2015378303	364	rural/clinic	No	13/06/2017	0	0	0
2015303635	364	rural/clinic	No	13/06/2017	0	0	0
2012321340	364	rural/clinic	No	13/06/2017	0	0	0
2014319431	364	rural/clinic	No	13/06/2017	0	0	0
2015303120	364	rural/clinic	No	13/06/2017	0	0	0
2015303080	364	rural/clinic	No	13/06/2017	0	0	0
2015303121	364	rural/clinic	No	13/06/2017	0	0	0
2014365160	364	rural/clinic	No	13/06/2017	0	0	0
2015377178	364	rural/clinic	No	16/06/2017	0	0	0
2012314022	364	rural/clinic	No	15/06/2017	0	0	0
2011179937	364	rural/clinic	No	14/06/2017	0	0	0
2014350049	364	rural/clinic	No	15/06/2017	0	0	0
2015340994	364	rural/clinic	No	15/06/2017	0	0	0
2015340995	364	rural/clinic	No	15/06/2017	0	0	0
2013272694	364	rural/clinic	No	15/06/2017	0	0	0
2014307334	364	rural/clinic	No	15/06/2017	0	0	0
2015336324	364	rural/clinic	No	15/06/2017	0	0	0
2015336323	364	rural/clinic	No	15/06/2017	0	0	0
2013272693	364	rural/clinic	No	15/06/2017	0	0	0
2012268532	364	rural/clinic	No	15/06/2017	0	0	0
2015303077	364	rural/clinic	No	13/06/2017	0	0	0
2014291818	364	rural/clinic	No	13/06/2017	0	0	0
2014346321	364	rural/clinic	No	15/06/2017	0	0	0
2012242586	364	rural/clinic	No	13/06/2017	0	0	0
2014346323		district/faith-based	No	15/06/2017	1	0	0
2015368716		district/faith-based	No	13/06/2017	1	0	0
2015352733		district/faith-based	No	15/06/2017	1	0	0
2015378205		district/faith-based	No	15/06/2017	1	0	0
2015331674		district/faith-based	No	15/06/2017	1	0	0
2015378203		district/faith-based	No	15/06/2017	1	0	0
2012331326		district/faith-based	No	15/06/2017	1	0	0
2015352734		district/faith-based	No	15/06/2017	1	0	0
2015378204		district/faith-based	No	15/06/2017	1	0	0
2015349461		district/faith-based	No	15/06/2017	1	0	0
2013284288		district/faith-based	No	29/05/2017	1	0	0
2015378206		district/faith-based	No	15/06/2017	1	0	0
2015408175		district/faith-based	No	15/06/2017	1	0	0
2015369053		district/faith-based	No	15/06/2017	1	0	0
2015325048		district/faith-based	No	15/06/2017	1	0	0
2014000571		district/faith-based	No	13/06/2017	1	0	0
2015324262		district/faith-based	No	15/06/2017	1	0	0
2013258457		district/faith-based	No	15/06/2017	1	0	0
2015369054		district/faith-based	No	15/06/2017	1	0	0
2015374273		district/faith-based	No	15/06/2017	1	0	0
2015369055		district/faith-based	No	15/06/2017	1	0	0
2015408177		district/faith-based	No	15/06/2017	1	0	0
2015374203		district/faith-based	No	15/06/2017	1	0	0
2015338857		district/faith-based	No	15/06/2017	1	0	0
2015369056		district/faith-based	No	15/06/2017	1	0	0
2015374204	247	district/faith-based	No	15/06/2017	1	0	0
2015338858	247	district/faith-based	No	15/06/2017	1	0	0
2015385534	247	district/faith-based	No	15/06/2017	1	0	0
2015374271	247	district/faith-based	No	15/06/2017	1	0	0
2015338859	247	district/faith-based	No	15/06/2017	1	0	0
2015385532	247	district/faith-based	No	13/06/2017	1	0	0
2015385535	247	district/faith-based	No	15/06/2017	1	0	0
2015338860	247	district/faith-based	No	15/06/2017	1	0	0
2015372422	247	district/faith-based	No	15/06/2017	1	0	0
2015385536	247	district/faith-based	No	15/06/2017	1	0	0
2015372416	247	district/faith-based	No	15/06/2017	1	0	0
2015338861	247	district/faith-based	No	15/06/2017	1	0	0
2015372425	247	district/faith-based	No	15/06/2017	1	0	0
2011117497	247	district/faith-based	No	15/06/2017	1	0	0
2015372408	247	district/faith-based	No	15/06/2017	1	0	0
2015372417	247	district/faith-based	No	15/06/2017	1	0	0
2012268534	247	district/faith-based	No	15/06/2017	1	0	0
2015372413	247	district/faith-based	No	15/06/2017	1	0	0
2015408679	247	district/faith-based	No	15/06/2017	1	0	0
2014354760	247	district/faith-based	No	15/06/2017	1	0	0
2012268784	247	district/faith-based	No	15/06/2017	1	0	0
2015352759	247	district/faith-based	No	15/06/2017	1	0	0
2015372415	247	district/faith-based	No	15/06/2017	1	0	0
2014347990	247	district/faith-based	No	15/06/2017	1	0	0
2011117499	247	district/faith-based	No	15/06/2017	1	0	0
2015352760	247	district/faith-based	No	15/06/2017	1	0	0
2015372414	247	district/faith-based	No	15/06/2017	1	0	0
2015363306	247	district/faith-based	No	15/06/2017	1	0	0
2011117498	247	district/faith-based	No	15/06/2017	1	0	0
2015415281	247	district/faith-based	No	15/06/2017	1	0	0

2015372421	247	district/faith-based	No	15/06/2017	1	0	0
2015401153	247	district/faith-based	No	15/06/2017	1	0	0
2015372424	247	district/faith-based	No	15/06/2017	1	0	0
2014378273	247	district/faith-based	No	15/06/2017	1	0	0
2015401152	247	district/faith-based	No	15/06/2017	1	0	0
2015372423	247	district/faith-based	No	15/06/2017	1	0	0
2015401151	247	district/faith-based	No	15/06/2017	1	0	0
2015352761	247	district/faith-based	No	15/06/2017	1	0	0
2015418862	247	district/faith-based	No	15/06/2017	1	0	0
2015340706	247	district/faith-based	No	15/06/2017	1	0	0
2014303701	247	district/faith-based	No	15/06/2017	1	0	0
2015418863	247	district/faith-based	No	15/06/2017	1	0	0
2015340707	247	district/faith-based	No	15/06/2017	1	0	0
2015418864	247	district/faith-based	No	15/06/2017	1	0	0
2015331900	247	district/faith-based	No	15/06/2017	1	0	0
2015340708	247	district/faith-based	No	15/06/2017	1	0	0
2015310660	247	district/faith-based	No	15/06/2017	1	0	0
2012370903	247	district/faith-based	No	15/06/2017	1	0	0
2011136116	247	district/faith-based	No	15/06/2017	1	0	0
2015340217	247	district/faith-based	No	15/06/2017	1	0	0
2015310615	247	district/faith-based	No	15/06/2017	1	0	0
2012370902	247	district/faith-based	No	15/06/2017	1	0	0
2011136123	247	district/faith-based	No	15/06/2017	1	0	0
2015340218	247	district/faith-based	No	15/06/2017	1	0	0
2012370901	247	district/faith-based	No	15/06/2017	1	0	0
2015290207	247	district/faith-based	No	15/06/2017	1	0	0
2015340219	247	district/faith-based	No	15/06/2017	1	0	0
2015369192	247	district/faith-based	No	15/06/2017	1	0	0
2015290208	247	district/faith-based	No	15/06/2017	1	0	0
2015392892	247	district/faith-based	No	15/06/2017	1	0	0
2015369193	247	district/faith-based	No	15/06/2017	1	0	0
2015340220	247	district/faith-based	No	15/06/2017	1	0	0
2015392893	247	district/faith-based	No	15/06/2017	1	0	0
2014288141	247	district/faith-based	No	15/06/2017	1	0	0
2011144028	247	district/faith-based	No	15/06/2017	1	0	0
2010099925	247	district/faith-based	No	15/06/2017	1	0	0
2014357000	247	district/faith-based	No	15/06/2017	1	0	0
2015392894	247	district/faith-based	No	15/06/2017	1	0	0
2014288012	247	district/faith-based	No	15/06/2017	1	0	0
2015392895	247	district/faith-based	No	15/06/2017	1	0	0
2014300538	247	district/faith-based	No	15/06/2017	1	0	0
2014303703	247	district/faith-based	No	15/06/2017	1	0	0
2010099924	247	district/faith-based	No	15/06/2017	1	0	0
2014340276	247	district/faith-based	No	15/06/2017	1	0	0
2015392898	247	district/faith-based	No	15/06/2017	1	0	0
2015290030	247	district/faith-based	No	15/06/2017	1	0	0
2014334182	247	district/faith-based	No	15/06/2017	1	0	0
2015314005	247	district/faith-based	No	15/06/2017	1	0	0
2015373440	247	district/faith-based	No	15/06/2017	1	0	0
2014340275	247	district/faith-based	No	15/06/2017	1	0	0
2015373441	247	district/faith-based	No	15/06/2017	1	0	0
2015314006	247	district/faith-based	No	15/06/2017	1	0	0
2014287979	247	district/faith-based	No	15/06/2017	1	0	0
2015331671	247	district/faith-based	No	15/06/2017	1	0	0
2015287375	247	district/faith-based	No	13/06/2017	1	0	0
2015287375	247	district/faith-based	No	13/06/2017	1	0	0
2015314007	247	district/faith-based	No	15/06/2017	1	0	0
2015373442	247	district/faith-based	No	15/06/2017	1	0	0
2015331673	247	district/faith-based	No	15/06/2017	1	0	0
2012284749	247	district/faith-based	No	15/06/2017	1	0	0
2015314008	247	district/faith-based	No	15/06/2017	1	0	0
2015373443	247	district/faith-based	No	15/06/2017	1	0	0
2012369972	247	district/faith-based	No	15/06/2017	1	0	0
2015373444	247	district/faith-based	No	15/06/2017	1	0	0
2015314009	247	district/faith-based	No	15/06/2017	1	0	0
2011145396	247	district/faith-based	No	15/06/2017	1	0	0
2014287980	247	district/faith-based	No	15/06/2017	1	0	0
2015378202	247	district/faith-based	No	15/06/2017	1	0	0
2015314010	247	district/faith-based	No	15/06/2017	1	0	0
2015331728	247	district/faith-based	No	15/06/2017	1	0	0
2015314011	247	district/faith-based	No	15/06/2017	1	0	0
2015362058	247	district/faith-based	No	15/06/2017	1	0	0
2015331730	247	district/faith-based	No	15/06/2017	1	0	0
2015331401	247	district/faith-based	No	15/06/2017	1	0	0
2015362059	247	district/faith-based	No	15/06/2017	1	0	0
2015314012	247	district/faith-based	No	15/06/2017	1	0	0
2015362060	247	district/faith-based	No	15/06/2017	1	0	0
2015331729	247	district/faith-based	No	15/06/2017	1	0	0
2014309977	247	district/faith-based	No	15/06/2017	1	0	0
2015314013	247	district/faith-based	No	15/06/2017	1	0	0
2015331733	247	district/faith-based	No	15/06/2017	1	0	0
2015319267	247	district/faith-based	No	15/06/2017	1	0	0

2015354964	247	district/faith-based	No	15/06/2017	1	0	0
2015335138	247	district/faith-based	No	15/06/2017	1	0	0
2015319268	247	district/faith-based	No	15/06/2017	1	0	0
2015331731	247	district/faith-based	No	15/06/2017	1	0	0
2015354966	247	district/faith-based	No	15/06/2017	1	0	0
2015319269	247	district/faith-based	No	15/06/2017	1	0	0
2015331732	247	district/faith-based	No	15/06/2017	1	0	0
2015354967	247	district/faith-based	No	15/06/2017	1	0	0
2015335139	247	district/faith-based	No	15/06/2017	1	0	0
2011144029	247	district/faith-based	No	15/06/2017	1	0	0
2015354968	247	district/faith-based	No	15/06/2017	1	0	0
2015319270	247	district/faith-based	No	15/06/2017	1	0	0
2015360734	247	district/faith-based	No	13/06/2017	1	0	0
2015335140	247	district/faith-based	No	15/06/2017	1	0	0
2014296330	247	district/faith-based	No	15/06/2017	1	0	0
2015319271	247	district/faith-based	No	15/06/2017	1	0	0
2015319272	247	district/faith-based	No	15/06/2017	1	0	0
2014296329	247	district/faith-based	No	15/06/2017	1	0	0
2015335141	247	district/faith-based	No	15/06/2017	1	0	0
2015303079	247	district/faith-based	No	13/06/2017	1	0	0
2015303079	247	district/faith-based	No	13/06/2017	1	0	0
2015403290	247	district/faith-based	No	15/06/2017	1	0	0
2015340129	247	district/faith-based	No	15/06/2017	1	0	0
2015335142	247	district/faith-based	No	15/06/2017	1	0	0
2015340130	247	district/faith-based	No	15/06/2017	1	0	0
2015403291	247	district/faith-based	No	15/06/2017	1	0	0
2015340131	247	district/faith-based	No	15/06/2017	1	0	0
2012321339	247	district/faith-based	No	13/06/2017	1	0	0
2012321339	247	district/faith-based	No	13/06/2017	1	0	0
2015403292	247	district/faith-based	No	15/06/2017	1	0	0
2015335386	247	district/faith-based	No	15/06/2017	1	0	0
2015340132	247	district/faith-based	No	15/06/2017	1	0	0
2015362529	247	district/faith-based	No	15/06/2017	1	0	0
2015340133	247	district/faith-based	No	15/06/2017	1	0	0
2015367216	247	district/faith-based	No	13/06/2017	1	0	0
2015367216	247	district/faith-based	No	13/06/2017	1	0	0
2015340134	247	district/faith-based	No	15/06/2017	1	0	0
2015335387	247	district/faith-based	No	15/06/2017	1	0	0
2014377412	247	district/faith-based	No	15/06/2017	1	0	0
2015352732	247	district/faith-based	No	15/06/2017	1	0	0
2014377413	247	district/faith-based	No	13/06/2017	1	0	0
2015352731	247	district/faith-based	No	15/06/2017	1	0	0
2015377266	247	district/faith-based	No	15/06/2017	1	0	0
2015352730	247	district/faith-based	No	15/06/2017	1	0	0
2015379323	247	district/faith-based	No	15/06/2017	1	0	0
2014346322	247	district/faith-based	No	15/06/2017	1	0	0
2015379324	247	district/faith-based	No	15/06/2017	1	0	0
2015377177	247	district/faith-based	No	15/06/2017	1	0	0
201053910	247	district/faith-based	No	15/06/2017	1	0	0
2015355918	247	district/faith-based	No	15/06/2017	1	0	0
2013255446	247	district/faith-based	No	15/06/2017	1	0	0
2014347025	247	district/faith-based	No	15/06/2017	1	0	0
2014338559	247	district/faith-based	No	15/06/2017	1	0	0
2014347026	247	district/faith-based	No	15/06/2017	1	0	0
2015286315	247	district/faith-based	No	15/06/2017	1	0	0
2012265639	247	district/faith-based	No	15/06/2017	1	0	0
2015286316	247	district/faith-based	No	15/06/2017	1	0	0
2013247023	247	district/faith-based	No	15/06/2017	1	0	0
2014339746	247	district/faith-based	No	15/06/2017	1	0	0
2015286314	247	district/faith-based	No	15/06/2017	1	0	0
2015384548	247	district/faith-based	No	15/06/2017	1	0	0
2015340993	247	district/faith-based	No	15/06/2017	1	0	0
2015373853	247	district/faith-based	No	15/06/2017	1	0	0
2015373854	796	rural/clinic	No	15/06/2017	0	0	0
2012322689	796	rural/clinic	No	13/06/2017	0	0	0
2015406281	796	rural/clinic	No	16/06/2017	0	0	0
2015406292	796	rural/clinic	No	16/06/2017	0	0	0
2015406290	796	rural/clinic	No	16/06/2017	0	0	0
2015406291	796	rural/clinic	No	16/06/2017	0	0	0
2015404961	796	rural/clinic	No	16/06/2017	0	0	0
2015404962	796	rural/clinic	No	16/06/2017	0	0	0
2015404965	796	rural/clinic	No	16/06/2017	0	0	0
2012259890	796	rural/clinic	No	16/06/2017	0	0	0
2012259893	796	rural/clinic	No	16/06/2017	0	0	0
2012298048	796	rural/clinic	No	16/06/2017	0	0	0
2015317011	796	rural/clinic	No	16/06/2017	0	0	0
2012298043	796	rural/clinic	No	16/06/2017	0	0	0
2015317014	796	rural/clinic	No	16/06/2017	0	0	0
2014349231	796	rural/clinic	No	16/06/2017	0	0	0
2014349235	796	rural/clinic	No	16/06/2017	0	0	0
2014349232	796	rural/clinic	No	16/06/2017	0	0	0
2012298049	796	rural/clinic	No	16/06/2017	0	0	0

2015356581	796	rural/clinic	No	16/06/2017	0	0	0
2014369047	796	rural/clinic	No	16/06/2017	0	0	0
2015320880	796	rural/clinic	No	16/06/2017	0	0	0
2015320881	796	rural/clinic	No	16/06/2017	0	0	0
2015320882	796	rural/clinic	No	16/06/2017	0	0	0
2015320883	796	rural/clinic	No	16/06/2017	0	0	0
2015320884	796	rural/clinic	No	16/06/2017	0	0	0
2015320885	796	rural/clinic	No	16/06/2017	0	0	0
2015320889	796	rural/clinic	No	16/06/2017	0	0	0
2015320890	796	rural/clinic	No	16/06/2017	0	0	0
2015320891	796	rural/clinic	No	16/06/2017	0	0	0
2015320892	796	rural/clinic	No	16/06/2017	0	0	0
2013266123	796	rural/clinic	No	16/06/2017	0	0	0
2012325694	796	rural/clinic	No	16/06/2017	0	0	0
2015334946	796	rural/clinic	No	13/06/2017	0	0	0
2015334946	796	rural/clinic	No	13/06/2017	0	0	0
2015376660	796	rural/clinic	No	13/06/2017	0	0	0
2015376660	796	rural/clinic	No	13/06/2017	0	0	0
2015376659	796	rural/clinic	No	13/06/2017	0	0	0
2015376659	796	rural/clinic	No	13/06/2017	0	0	0
2015376657	796	rural/clinic	No	13/06/2017	0	0	0
2015376657	796	rural/clinic	No	13/06/2017	0	0	0
2014360970	796	rural/clinic	No	13/06/2017	0	0	0
2014360970	796	rural/clinic	No	13/06/2017	0	0	0
2015334801	796	rural/clinic	No	13/06/2017	0	0	0
2015405966	796	rural/clinic	No	13/06/2017	0	0	0
2015405966	796	rural/clinic	No	13/06/2017	0	0	0
2014367625	796	rural/clinic	No	13/06/2017	0	0	0
2014367625	796	rural/clinic	No	13/06/2017	0	0	0
2015390312	796	rural/clinic	No	13/06/2017	0	0	0
2011192846	796	rural/clinic	No	13/06/2017	0	0	0
2011192846	796	rural/clinic	No	13/06/2017	0	0	0
2014363887	796	rural/clinic	No	13/06/2017	0	0	0
2014363887	796	rural/clinic	No	13/06/2017	0	0	0
2015397650	796	rural/clinic	No	19/06/2017	0	0	0
2015397306	796	rural/clinic	No	19/06/2017	0	0	0
2015397305	796	rural/clinic	No	19/06/2017	0	0	0
2015397304	796	rural/clinic	No	19/06/2017	0	0	0
2015397303	796	rural/clinic	No	19/06/2017	0	0	0
2015397302	796	rural/clinic	No	19/06/2017	0	0	0
2015397649	796	rural/clinic	No	19/06/2017	0	0	0
2015397301	796	rural/clinic	No	19/06/2017	0	0	0
2015286650	796	rural/clinic	No	19/06/2017	0	0	0
2015373855	796	rural/clinic	No	20/06/2017	0	0	0
2017WRONG	796	rural/clinic	No	20/06/2017	0	0	0
2011137310	796	rural/clinic	No	13/06/2017	0	0	0
2011137310	796	rural/clinic	No	13/06/2017	0	0	0
2011137309	796	rural/clinic	No	13/06/2017	0	0	0
2015340100	796	rural/clinic	No	08/06/2017	0	0	0
2015340100	796	rural/clinic	No	08/06/2017	0	0	0
2015352734	796	rural/clinic	No	15/06/2017	0	0	0
2015352734	796	rural/clinic	No	15/06/2017	0	0	0
2014357000	796	rural/clinic	No	15/06/2017	0	0	0
2014303703	796	rural/clinic	No	15/06/2017	0	0	0
2014303703	796	rural/clinic	No	15/06/2017	0	0	0
2015373440	796	rural/clinic	No	15/06/2017	0	0	0
2015373440	796	rural/clinic	No	15/06/2017	0	0	0
2014340276	796	rural/clinic	No	15/06/2017	0	0	0
2014340276	796	rural/clinic	No	15/06/2017	0	0	0
2015379323	796	rural/clinic	No	15/06/2017	0	0	0
2015379323	796	rural/clinic	No	15/06/2017	0	0	0
2014350049	796	rural/clinic	No	15/06/2017	0	0	0
2015377177	796	rural/clinic	No	15/06/2017	0	0	0
2015402632	796	rural/clinic	No	20/06/2017	0	0	0
2015386262	796	rural/clinic	No	20/06/2017	0	0	0
2015362276	796	rural/clinic	No	20/06/2017	0	0	0
2015313226	796	rural/clinic	No	20/06/2017	0	0	0
2014306032	796	rural/clinic	No	20/06/2017	0	0	0
2015415657	465	rural/clinic	No	20/06/2017	0	0	0
2012291492	465	rural/clinic	No	20/06/2017	0	0	0
2015313225	465	rural/clinic	No	20/06/2017	0	0	0
2015402631	465	rural/clinic	No	20/06/2017	0	0	0
2012244288	465	rural/clinic	No	20/06/2017	0	0	0
2015386715	465	rural/clinic	No	20/06/2017	0	0	0
2015313224	465	rural/clinic	No	20/06/2017	0	0	0
2014361225	465	rural/clinic	No	20/06/2017	0	0	0
2014361224	465	rural/clinic	No	20/06/2017	0	0	0
2015313223	465	rural/clinic	No	20/06/2017	0	0	0
2015386714	465	rural/clinic	No	20/06/2017	0	0	0
2015376405	465	rural/clinic	No	20/06/2017	0	0	0
2015386716	465	rural/clinic	No	20/06/2017	0	0	0
2012291491	465	rural/clinic	No	20/06/2017	0	0	0

2015313231	465	rural/clinic	No	20/06/2017	0	0	0
2015386717	465	rural/clinic	No	20/06/2017	0	0	0
2015402639	465	rural/clinic	No	20/06/2017	0	0	0
2015402640	465	rural/clinic	No	20/06/2017	0	0	0
2012259896	465	rural/clinic	No	20/06/2017	0	0	0
2015402641	465	rural/clinic	No	20/06/2017	0	0	0
2014327517	465	rural/clinic	No	20/06/2017	0	0	0
2015402642	465	rural/clinic	No	20/06/2017	0	0	0
2014327515	465	rural/clinic	No	20/06/2017	0	0	0
2015402636	465	rural/clinic	No	20/06/2017	0	0	0
2014327516	465	rural/clinic	No	20/06/2017	0	0	0
2015402637	465	rural/clinic	No	20/06/2017	0	0	0
2015339766	465	rural/clinic	No	20/06/2017	0	0	0
2014358193	465	rural/clinic	No	20/06/2017	0	0	0
2015339765	465	rural/clinic	No	20/06/2017	0	0	0
2015382931	465	rural/clinic	No	20/06/2017	0	0	0
2014326639	465	rural/clinic	No	20/06/2017	0	0	0
2015339764	465	rural/clinic	No	20/06/2017	0	0	0
2015382935	465	rural/clinic	No	20/06/2017	0	0	0
2014383706	465	rural/clinic	No	20/06/2017	0	0	0
2014371585	465	rural/clinic	No	20/06/2017	0	0	0
2012289786	465	rural/clinic	No	20/06/2017	0	0	0
2015382933	465	rural/clinic	No	20/06/2017	0	0	0
2014326638	465	rural/clinic	No	20/06/2017	0	0	0
2015382932	465	rural/clinic	No	20/06/2017	0	0	0
2014348243	766	rural/clinic	No	20/06/2017	0	0	0
2014326637	766	rural/clinic	No	20/06/2017	0	0	0
2015382934	766	rural/clinic	No	20/06/2017	0	0	0
2012289787	766	rural/clinic	No	20/06/2017	0	0	0
2015382938	766	rural/clinic	No	20/06/2017	0	0	0
2015386267	766	rural/clinic	No	20/06/2017	0	0	0
2014348236	766	rural/clinic	No	20/06/2017	0	0	0
2015382936	766	rural/clinic	No	20/06/2017	0	0	0
2015386266	766	rural/clinic	No	20/06/2017	0	0	0
2015382940	766	rural/clinic	No	20/06/2017	0	0	0
2014348237	766	rural/clinic	No	20/06/2017	0	0	0
2015386265	766	rural/clinic	No	20/06/2017	0	0	0
2015368791	766	rural/clinic	No	20/06/2017	0	0	0
2014348238	766	rural/clinic	No	20/06/2017	0	0	0
2015386264	766	rural/clinic	No	20/06/2017	0	0	0
2014348239	766	rural/clinic	No	20/06/2017	0	0	0
2015368722	766	rural/clinic	No	20/06/2017	0	0	0
2014348240	766	rural/clinic	No	20/06/2017	0	0	0
2015386263	766	rural/clinic	No	20/06/2017	0	0	0
2014348241	724	rural/clinic	No	20/06/2017	0	0	0
2014290736	724	rural/clinic	No	20/06/2017	0	0	0
2014289540	724	rural/clinic	No	20/06/2017	0	0	0
2015368719	724	rural/clinic	No	20/06/2017	0	0	0
2015368720	724	rural/clinic	No	20/06/2017	0	0	0
2011144823	724	rural/clinic	No	20/06/2017	0	0	0
2015412320	724	rural/clinic	No	20/06/2017	0	0	0
2015297488	724	rural/clinic	No	20/06/2017	0	0	0
2015368721	724	rural/clinic	No	20/06/2017	0	0	0
2015297489	724	rural/clinic	No	20/06/2017	0	0	0
2015368718	724	rural/clinic	No	20/06/2017	0	0	0
2015297490	724	rural/clinic	No	20/06/2017	0	0	0
2015400094	724	rural/clinic	No	20/06/2017	0	0	0
2015297491	724	rural/clinic	No	20/06/2017	0	0	0
2015386251	724	rural/clinic	No	20/06/2017	0	0	0
2015386252	724	rural/clinic	No	20/06/2017	0	0	0
2015386253	724	rural/clinic	No	20/06/2017	0	0	0
2015386254	724	rural/clinic	No	20/06/2017	0	0	0
2015386255	724	rural/clinic	No	20/06/2017	0	0	0
2015386256	724	rural/clinic	No	31/05/2017	0	0	0
2015400095	724	rural/clinic	No	20/06/2017	0	0	0
2015400096	724	rural/clinic	No	20/06/2017	0	0	0
2015386257	724	rural/clinic	No	20/06/2017	0	0	0
2015386258	724	rural/clinic	No	20/06/2017	0	0	0
2015386259	724	rural/clinic	No	20/06/2017	0	0	0
2015386260	724	rural/clinic	No	20/06/2017	0	0	0
2015400097	724	rural/clinic	No	20/06/2017	0	0	0
2015400098	724	rural/clinic	No	20/06/2017	0	0	0
2015386261	724	rural/clinic	No	20/06/2017	0	0	0
2015400099	724	rural/clinic	No	20/06/2017	0	0	0
2015400100	725	rural/clinic	No	20/06/2017	0	0	0
2015373701	725	rural/clinic	No	20/06/2017	0	0	0
2015373702	725	rural/clinic	No	20/06/2017	0	0	0
2015373703	725	rural/clinic	No	20/06/2017	0	0	0
2015373704	725	rural/clinic	No	20/06/2017	0	0	0
2015373705	725	rural/clinic	No	20/06/2017	0	0	0
2015373706	725	rural/clinic	No	20/06/2017	0	0	0
2015404964	725	rural/clinic	No	20/06/2017	0	0	0

2015404963	725	rural/clinic	No	20/06/2017	0	0	0
2015404958	725	rural/clinic	No	20/06/2017	0	0	0
2015404960	725	rural/clinic	No	20/06/2017	0	0	0
2015405626	725	rural/clinic	No	20/06/2017	0	0	0
2015367218	725	rural/clinic	No	20/06/2017	0	0	0
2015363759	725	rural/clinic	No	20/06/2017	0	0	0
2013264393	725	rural/clinic	No	20/06/2017	0	0	0
2013264392	725	rural/clinic	No	20/06/2017	0	0	0
2015362822	725	rural/clinic	No	20/06/2017	0	0	0
2015362821	725	rural/clinic	No	20/06/2017	0	0	0
2015313229	725	rural/clinic	No	20/06/2017	0	0	0
2015313228	725	rural/clinic	No	20/06/2017	0	0	0
2015313227	725	rural/clinic	No	20/06/2017	0	0	0
2015289184	725	rural/clinic	No	20/06/2017	0	0	0
2012359165	725	rural/clinic	No	20/06/2017	0	0	0
2012268536	725	rural/clinic	No	20/06/2017	0	0	0
2012268535	725	rural/clinic	No	20/06/2017	0	0	0
2015337989	725	rural/clinic	No	20/06/2017	0	0	0
2015360201	725	rural/clinic	No	20/06/2017	0	0	0
2015363454	725	rural/clinic	No	20/06/2017	0	0	0
2015384817	725	rural/clinic	No	20/06/2017	0	0	0
2011221925	725	rural/clinic	No	20/06/2017	0	0	0
2015384816	725	rural/clinic	No	20/06/2017	0	0	0
2015289571	725	rural/clinic	No	20/06/2017	0	0	0
2014314477	725	rural/clinic	No	20/06/2017	0	0	0
2015363451	725	rural/clinic	No	20/06/2017	0	0	0
2015363453	725	rural/clinic	No	20/06/2017	0	0	0
2015363452	725	rural/clinic	No	20/06/2017	0	0	0
2012317321	725	rural/clinic	No	20/06/2017	0	0	0
2012317322	725	rural/clinic	No	20/06/2017	0	0	0
2012261385	725	rural/clinic	No	20/06/2017	0	0	0
2012295146	725	rural/clinic	No	20/06/2017	0	0	0
2014309386	725	rural/clinic	No	20/06/2017	0	0	0
2015384815	816	rural/clinic	No	20/06/2017	0	0	0
2012268537	816	rural/clinic	No	20/06/2017	0	0	0
2014370306	816	rural/clinic	No	20/06/2017	0	0	0
2014370305	816	rural/clinic	No	20/06/2017	0	0	0
2015368980	816	rural/clinic	No	20/06/2017	0	0	0
2015368981	816	rural/clinic	No	20/06/2017	0	0	0
2015368982	816	rural/clinic	No	20/06/2017	0	0	0
2015368983	816	rural/clinic	No	20/06/2017	0	0	0
2015368984	816	rural/clinic	No	20/06/2017	0	0	0
2015368985	816	rural/clinic	No	20/06/2017	0	0	0
2015297341	816	rural/clinic	No	20/06/2017	0	0	0
2015368986	816	rural/clinic	No	20/06/2017	0	0	0
2015297342	816	rural/clinic	No	20/06/2017	0	0	0
2015362280	816	rural/clinic	No	20/06/2017	0	0	0
2015297343	816	rural/clinic	No	20/06/2017	0	0	0
2015362279	816	rural/clinic	No	20/06/2017	0	0	0
2015377899	816	rural/clinic	No	20/06/2017	0	0	0
2015362278	816	rural/clinic	No	20/06/2017	0	0	0
2015362277	816	rural/clinic	No	20/06/2017	0	0	0
2015377900	816	rural/clinic	No	20/06/2017	0	0	0
2015383733	816	rural/clinic	No	20/06/2017	0	0	0
2015402633	816	rural/clinic	No	20/06/2017	0	0	0
201024359	816	rural/clinic	No	20/06/2017	0	0	0
201024360	816	rural/clinic	No	20/06/2017	0	0	0
2015383734	816	rural/clinic	No	20/06/2017	0	0	0
2015383730	816	rural/clinic	No	20/06/2017	0	0	0
2015383731	816	rural/clinic	No	20/06/2017	0	0	0
2015383732	816	rural/clinic	No	20/06/2017	0	0	0
2014316060	816	rural/clinic	No	20/06/2017	0	0	0
2011139352	816	rural/clinic	No	20/06/2017	0	0	0
2011139351	816	rural/clinic	No	20/06/2017	0	0	0
2015344451	816	rural/clinic	No	20/06/2017	0	0	0
2011137307	816	rural/clinic	No	20/06/2017	0	0	0
2015404959	816	rural/clinic	No	20/06/2017	0	0	0
2015402634	816	rural/clinic	No	20/06/2017	0	0	0
2014348242	816	rural/clinic	No	20/06/2017	0	0	0
2015402638	816	rural/clinic	No	20/06/2017	0	0	0
2011137308	816	rural/clinic	No	20/06/2017	0	0	0
2015344657	816	rural/clinic	No	20/06/2017	0	0	0
2015344658	816	rural/clinic	No	20/06/2017	0	0	0
2015413599	816	rural/clinic	No	20/06/2017	0	0	0
2015402635	816	rural/clinic	No	20/06/2017	0	0	0
2015382937	816	rural/clinic	No	20/06/2017	0	0	0
2014370219	816	rural/clinic	No	20/06/2017	0	0	0
2014367626	816	rural/clinic	No	20/06/2017	0	0	0
2015369744	816	rural/clinic	No	20/06/2017	0	0	0
2015369743	816	rural/clinic	No	20/06/2017	0	0	0
2015326956	816	rural/clinic	No	06/06/2017	0	0	0
2015415274	816	rural/clinic	No	08/06/2017	0	0	0

2015368668	816	rural/clinic	No	20/06/2017	0	0	0
2014358277	816	rural/clinic	No	08/06/2017	0	0	0
2014358280	816	rural/clinic	No	08/06/2017	0	0	0
2014358279	816	rural/clinic	No	08/06/2017	0	0	0
2015368665	816	rural/clinic	No	20/06/2017	0	0	0
2015368666	816	rural/clinic	No	20/06/2017	0	0	0
2015368667	816	rural/clinic	No	20/06/2017	0	0	0
2015326961	816	rural/clinic	No	20/06/2017	0	0	0
2015326964	816	rural/clinic	No	20/06/2017	0	0	0
2015326963	816	rural/clinic	No	20/06/2017	0	0	0
2015326962	816	rural/clinic	No	20/06/2017	0	0	0
2014371399	816	rural/clinic	No	20/06/2017	0	0	0
2014371400	816	rural/clinic	No	20/06/2017	0	0	0
2015413922	816	rural/clinic	No	20/06/2017	0	0	0
2015368592	816	rural/clinic	No	20/06/2017	0	0	0
2015368593	816	rural/clinic	No	20/06/2017	0	0	0
2011143199	816	rural/clinic	No	20/06/2017	0	0	0
2011143200	816	rural/clinic	No	20/06/2017	0	0	0
2012291364	816	rural/clinic	No	20/06/2017	0	0	0
2014358218	816	rural/clinic	No	20/06/2017	0	0	0
2014375509	816	rural/clinic	No	20/06/2017	0	0	0
2014375504	816	rural/clinic	No	20/06/2017	0	0	0
2015369742	816	rural/clinic	No	20/06/2017	0	0	0
2015369933	816	rural/clinic	No	20/06/2017	0	0	0
2015368819	816	rural/clinic	No	20/06/2017	0	0	0
2015368820	816	rural/clinic	No	20/06/2017	0	0	0
2015390264	816	rural/clinic	No	20/06/2017	0	0	0
2014375510	816	rural/clinic	No	20/06/2017	0	0	0
2015326594	816	rural/clinic	No	20/06/2017	0	0	0
2015414461	816	rural/clinic	No	20/06/2017	0	0	0
2015368301	816	rural/clinic	No	20/06/2017	0	0	0
2015368293	816	rural/clinic	No	20/06/2017	0	0	0
2015368295	816	rural/clinic	No	20/06/2017	0	0	0
2015368294	816	rural/clinic	No	20/06/2017	0	0	0
2015368292	816	rural/clinic	No	20/06/2017	0	0	0
2011136127	816	rural/clinic	No	20/06/2017	0	0	0
2014306029	816	rural/clinic	No	20/06/2017	0	0	0
2014306030	816	rural/clinic	No	20/06/2017	0	0	0
2014306031	816	rural/clinic	No	20/06/2017	0	0	0
2015320893	816	rural/clinic	No	20/06/2017	0	0	0
2015320894	816	rural/clinic	No	20/06/2017	0	0	0
2014290737	816	rural/clinic	No	20/06/2017	0	0	0
2011136128	816	rural/clinic	No	20/06/2017	0	0	0
2011144824	816	rural/clinic	No	20/06/2017	0	0	0
2012362564	816	rural/clinic	No	20/06/2017	0	0	0
2014375505	816	rural/clinic	No	20/06/2017	0	0	0
2012362565	816	rural/clinic	No	20/06/2017	0	0	0
2012244288	816	rural/clinic	No	20/06/2017	0	0	0
2012244288	816	rural/clinic	No	20/06/2017	0	0	0
2015368721	816	rural/clinic	No	20/06/2017	0	0	0
2015368721	816	rural/clinic	No	20/06/2017	0	0	0
2014298241	816	rural/clinic	No	20/06/2017	0	0	0
2015369931	816	rural/clinic	No	20/06/2017	0	0	0
2015386264	816	rural/clinic	No	20/06/2017	0	0	0
2015386251	816	rural/clinic	No	20/06/2017	0	0	0
2015369775	816	rural/clinic	No	20/06/2017	0	0	0
2015402631	816	rural/clinic	No	20/06/2017	0	0	0
2015402631	816	rural/clinic	No	20/06/2017	0	0	0
2015369934	816	rural/clinic	No	20/06/2017	0	0	0
2014374026	816	rural/clinic	No	20/06/2017	0	0	0
2014375507	816	rural/clinic	No	20/06/2017	0	0	0
2012365477	816	rural/clinic	No	20/06/2017	0	0	0
2014374027	816	rural/clinic	No	20/06/2017	0	0	0
2014374025	816	rural/clinic	No	20/06/2017	0	0	0
2014297976	816	rural/clinic	No	20/06/2017	0	0	0
2015369930	816	rural/clinic	No	20/06/2017	0	0	0
2015358549	816	rural/clinic	No	20/06/2017	0	0	0
2012388251	816	rural/clinic	No	20/06/2017	0	0	0
2015414737	816	rural/clinic	No	20/06/2017	0	0	0
2015369932	816	rural/clinic	No	20/06/2017	0	0	0
2012388252	816	rural/clinic	No	20/06/2017	0	0	0
2014375506	816	rural/clinic	No	20/06/2017	0	0	0
2014375508	816	rural/clinic	No	20/06/2017	0	0	0
2012388253	816	rural/clinic	No	20/06/2017	0	0	0
2015326420	816	rural/clinic	No	20/06/2017	0	0	0
2012388254	816	rural/clinic	No	20/06/2017	0	0	0
2015326421	816	rural/clinic	No	20/06/2017	0	0	0
2011193488	816	rural/clinic	No	20/06/2017	0	0	0
2011193489	816	rural/clinic	No	20/06/2017	0	0	0
2011193490	816	rural/clinic	No	20/06/2017	0	0	0
2015333962	816	rural/clinic	No	20/06/2017	0	0	0
2014315707	816	rural/clinic	No	20/06/2017	0	0	0

2015311592	816	rural/clinic	No	20/06/2017	0	0	0
2014315709	816	rural/clinic	No	20/06/2017	0	0	0
2015397813	816	rural/clinic	No	20/06/2017	0	0	0
2015333921	816	rural/clinic	No	20/06/2017	0	0	0
2015333261	816	rural/clinic	No	20/06/2017	0	0	0
2015346092	816	rural/clinic	No	20/06/2017	0	0	0
2014370190	816	rural/clinic	No	20/06/2017	0	0	0
2014370191	816	rural/clinic	No	20/06/2017	0	0	0
2014315710	816	rural/clinic	No	20/06/2017	0	0	0
2014315708	816	rural/clinic	No	20/06/2017	0	0	0
2015346234	816	rural/clinic	No	20/06/2017	0	0	0
2014382444	816	rural/clinic	No	20/06/2017	0	0	0
2014382445	816	rural/clinic	No	20/06/2017	0	0	0
201024352	816	rural/clinic	No	20/06/2017	0	0	0
2015311223	816	rural/clinic	No	20/06/2017	0	0	0
2015311221	816	rural/clinic	No	20/06/2017	0	0	0
201021863	816	rural/clinic	No	20/06/2017	0	0	0
2015311220	816	rural/clinic	No	20/06/2017	0	0	0
2015311222	816	rural/clinic	No	20/06/2017	0	0	0
201021862	816	rural/clinic	No	20/06/2017	0	0	0
2015311595	816	rural/clinic	No	20/06/2017	0	0	0
201021860	816	rural/clinic	No	20/06/2017	0	0	0
201021854	816	rural/clinic	No	20/06/2017	0	0	0
201021855	816	rural/clinic	No	20/06/2017	0	0	0
201021861	816	rural/clinic	No	20/06/2017	0	0	0
2015327224	816	rural/clinic	No	20/06/2017	0	0	0
201021858	816	rural/clinic	No	20/06/2017	0	0	0
2015327225	816	rural/clinic	No	20/06/2017	0	0	0
2015311593	816	rural/clinic	No	20/06/2017	0	0	0
2015327228	816	rural/clinic	No	20/06/2017	0	0	0
2015327229	816	rural/clinic	No	20/06/2017	0	0	0
201021857	816	rural/clinic	No	20/06/2017	0	0	0
2015311920	816	rural/clinic	No	20/06/2017	0	0	0
2015311921	816	rural/clinic	No	20/06/2017	0	0	0
2015311591	816	rural/clinic	No	20/06/2017	0	0	0
2015327352	816	rural/clinic	No	20/06/2017	0	0	0
201024357	816	rural/clinic	No	20/06/2017	0	0	0
2015327354	816	rural/clinic	No	20/06/2017	0	0	0
201021864	816	rural/clinic	No	20/06/2017	0	0	0
201021856	816	rural/clinic	No	20/06/2017	0	0	0
2015311589	816	rural/clinic	No	20/06/2017	0	0	0
2015311606	816	rural/clinic	No	20/06/2017	0	0	0
2015327355	816	rural/clinic	No	20/06/2017	0	0	0
2015311607	816	rural/clinic	No	20/06/2017	0	0	0
2015327226	816	rural/clinic	No	20/06/2017	0	0	0
201024358	816	rural/clinic	No	20/06/2017	0	0	0
2015327230	816	rural/clinic	No	20/06/2017	0	0	0
2012350340	816	rural/clinic	No	20/06/2017	0	0	0
2014310547	816	rural/clinic	No	20/06/2017	0	0	0
2012350341	816	rural/clinic	No	20/06/2017	0	0	0
2012350344	816	rural/clinic	No	20/06/2017	0	0	0
2014344800	816	rural/clinic	No	20/06/2017	0	0	0
201024361	816	rural/clinic	No	20/06/2017	0	0	0
2015357196	816	rural/clinic	No	20/06/2017	0	0	0
2013271845	816	rural/clinic	No	20/06/2017	0	0	0
2015325204	816	rural/clinic	No	20/06/2017	0	0	0
2015357534	816	rural/clinic	No	20/06/2017	0	0	0
2015302436	816	rural/clinic	No	20/06/2017	0	0	0
2015325936	816	rural/clinic	No	20/06/2017	0	0	0
2015325205	816	rural/clinic	No	20/06/2017	0	0	0
2013271846	816	rural/clinic	No	20/06/2017	0	0	0
2015357199	816	rural/clinic	No	20/06/2017	0	0	0
2015357198	816	rural/clinic	No	20/06/2017	0	0	0
2015302438	816	rural/clinic	No	20/06/2017	0	0	0
2015302437	63	rural/clinic	No	20/06/2017	0	0	0
2015325935	63	rural/clinic	No	20/06/2017	0	0	0
2015313230	63	rural/clinic	No	20/06/2017	0	0	0
2015313232	63	rural/clinic	No	20/06/2017	0	0	0
2015313233	63	rural/clinic	No	20/06/2017	0	0	0
2015313234	63	rural/clinic	No	20/06/2017	0	0	0
2015313235	63	rural/clinic	No	20/06/2017	0	0	0
2015313236	63	rural/clinic	No	20/06/2017	0	0	0
2015313237	63	rural/clinic	No	20/06/2017	0	0	0
2015313238	63	rural/clinic	No	20/06/2017	0	0	0
2015313239	63	rural/clinic	No	20/06/2017	0	0	0
2015313240	63	rural/clinic	No	20/06/2017	0	0	0
2015326412	63	rural/clinic	No	20/06/2017	0	0	0
2015326413	664	rural/clinic	No	20/06/2017	0	0	0
2015326414	664	rural/clinic	No	20/06/2017	0	0	0
2015326415	664	rural/clinic	No	20/06/2017	0	0	0
2015326416	664	rural/clinic	No	20/06/2017	0	0	0
2015326417	664	rural/clinic	No	20/06/2017	0	0	0

2015326418	664	rural/clinic	No	20/06/2017	0	0	0
2015326419	664	rural/clinic	No	20/06/2017	0	0	0
2015327343	664	rural/clinic	No	20/06/2017	0	0	0
2015327344	699	rural/clinic	No	20/06/2017	0	0	0
2015311133	699	rural/clinic	No	20/06/2017	0	0	0
2015311131	699	rural/clinic	No	20/06/2017	0	0	0
2015311132	699	rural/clinic	No	20/06/2017	0	0	0
2015311134	699	rural/clinic	No	20/06/2017	0	0	0
2015311605	699	rural/clinic	No	20/06/2017	0	0	0
2014345241	699	rural/clinic	No	20/06/2017	0	0	0
2014345242	64	rural/clinic	No	20/06/2017	0	0	0
2015311351	64	rural/clinic	No	20/06/2017	0	0	0
2015337512	64	rural/clinic	No	20/06/2017	0	0	0
2015337623	64	rural/clinic	No	20/06/2017	0	0	0
2015337511	64	rural/clinic	No	20/06/2017	0	0	0
2015355520	64	rural/clinic	No	20/06/2017	0	0	0
2012358293	64	rural/clinic	No	20/06/2017	0	0	0
2015324481	64	rural/clinic	No	20/06/2017	0	0	0
2015315912	64	rural/clinic	No	20/06/2017	0	0	0
2015337624	64	rural/clinic	No	20/06/2017	0	0	0
2015315913	64	rural/clinic	No	20/06/2017	0	0	0
2015314610	64	rural/clinic	No	20/06/2017	0	0	0
2015355521	64	rural/clinic	No	20/06/2017	0	0	0
2015314609	64	rural/clinic	No	20/06/2017	0	0	0
2015324039	64	rural/clinic	No	20/06/2017	0	0	0
2012305810	64	rural/clinic	No	20/06/2017	0	0	0
2014303653	64	rural/clinic	No	20/06/2017	0	0	0
2013249418	64	rural/clinic	No	20/06/2017	0	0	0
2012305811	64	rural/clinic	No	20/06/2017	0	0	0
2012305812	64	rural/clinic	No	20/06/2017	0	0	0
2015342011	64	rural/clinic	No	20/06/2017	0	0	0
2015385853	64	rural/clinic	No	20/06/2017	0	0	0
2012305814	64	rural/clinic	No	20/06/2017	0	0	0
2015331779	64	rural/clinic	No	20/06/2017	0	0	0
2012305813	64	rural/clinic	No	20/06/2017	0	0	0
2015406293	64	rural/clinic	No	22/06/2017	0	0	0
2011199093	64	rural/clinic	No	22/06/2017	0	0	0
2011199092	64	rural/clinic	No	22/06/2017	0	0	0
2011191292	64	rural/clinic	No	22/06/2017	0	0	0
2015345059	64	rural/clinic	No	20/06/2017	0	0	0
2015369195	64	rural/clinic	No	22/06/2017	0	0	0
2015399256	64	rural/clinic	No	22/06/2017	0	0	0
2015345060	64	rural/clinic	No	20/06/2017	0	0	0
2015345061	64	rural/clinic	No	20/06/2017	0	0	0
2015405801	64	rural/clinic	No	22/06/2017	0	0	0
2015305083	64	rural/clinic	No	20/06/2017	0	0	0
2015301490	64	rural/clinic	No	22/06/2017	0	0	0
2015305084	64	rural/clinic	No	20/06/2017	0	0	0
2014378271	64	rural/clinic	No	22/06/2017	0	0	0
2012251548	64	rural/clinic	No	22/06/2017	0	0	0
2015305085	64	rural/clinic	No	20/06/2017	0	0	0
2014350461	64	rural/clinic	No	22/06/2017	0	0	0
2012251549	64	rural/clinic	No	22/06/2017	0	0	0
2015373856	64	rural/clinic	No	21/06/2017	0	0	0
2014378270	64	rural/clinic	No	22/06/2017	0	0	0
2012251547	64	rural/clinic	No	22/06/2017	0	0	0
2015373857	64	rural/clinic	No	21/06/2017	0	0	0
2014326091	64	rural/clinic	No	22/06/2017	0	0	0
2015326958	64	rural/clinic	No	21/06/2017	0	0	0
2015352736	64	rural/clinic	No	22/06/2017	0	0	0
2014326089	64	rural/clinic	No	22/06/2017	0	0	0
2015326959	64	rural/clinic	No	21/06/2017	0	0	0
2015301492	64	rural/clinic	No	22/06/2017	0	0	0
2014326090	64	rural/clinic	No	22/06/2017	0	0	0
2015326819	64	rural/clinic	No	21/06/2017	0	0	0
2015297202	64	rural/clinic	No	22/06/2017	0	0	0
2015301491	64	rural/clinic	No	22/06/2017	0	0	0
2015326820	64	rural/clinic	No	21/06/2017	0	0	0
2014369848	64	rural/clinic	No	22/06/2017	0	0	0
2015301493	64	rural/clinic	No	22/06/2017	0	0	0
2015326821	64	rural/clinic	No	21/06/2017	0	0	0
2011199245	64	rural/clinic	No	22/06/2017	0	0	0
2015326822	64	rural/clinic	No	21/06/2017	0	0	0
2012317561	64	rural/clinic	No	22/06/2017	0	0	0
2015326823	64	rural/clinic	No	21/06/2017	0	0	0
2012350632	64	rural/clinic	No	22/06/2017	0	0	0
2014369849	64	rural/clinic	No	22/06/2017	0	0	0
2015326824	64	rural/clinic	No	21/06/2017	0	0	0
2012350633	64	rural/clinic	No	22/06/2017	0	0	0
2014369850	64	rural/clinic	No	22/06/2017	0	0	0
2014334680	64	rural/clinic	No	22/06/2017	0	0	0
2011128285	64	rural/clinic	No	22/06/2017	0	0	0

2012284906	64	rural/clinic	No	22/06/2017	0	0	0
2014287982	64	rural/clinic	No	22/06/2017	0	0	0
2015310616	64	rural/clinic	No	22/06/2017	0	0	0
2014287981	64	rural/clinic	No	22/06/2017	0	0	0
2015326825	64	rural/clinic	No	21/06/2017	0	0	0
2012284229	64	rural/clinic	No	22/06/2017	0	0	0
2012243654	64	rural/clinic	No	22/06/2017	0	0	0
2012284750	665	rural/clinic	No	22/06/2017	0	0	0
2015380806	665	rural/clinic	No	22/06/2017	0	0	0
2015338862	665	rural/clinic	No	22/06/2017	0	0	0
2015380807	665	rural/clinic	No	22/06/2017	0	0	0
2015338863	665	rural/clinic	No	22/06/2017	0	0	0
2015380808	665	rural/clinic	No	22/06/2017	0	0	0
2012344504	665	rural/clinic	No	22/06/2017	0	0	0
2011141085	665	rural/clinic	No	22/06/2017	0	0	0
2015326826	665	rural/clinic	No	21/06/2017	0	0	0
2017123782	665	rural/clinic	No	22/06/2017	0	0	0
2011141086	665	rural/clinic	No	22/06/2017	0	0	0
2015326827	665	rural/clinic	No	21/06/2017	0	0	0
2015404730	665	rural/clinic	No	22/06/2017	0	0	0
2015404732	665	rural/clinic	No	22/06/2017	0	0	0
2015326828	665	rural/clinic	No	21/06/2017	0	0	0
2015404731	665	rural/clinic	No	22/06/2017	0	0	0
2015326829	665	rural/clinic	No	21/06/2017	0	0	0
2015417068	665	rural/clinic	No	22/06/2017	0	0	0
2015326830	665	rural/clinic	No	21/06/2017	0	0	0
2011141088	665	rural/clinic	No	22/06/2017	0	0	0
2015326831	665	rural/clinic	No	21/06/2017	0	0	0
2015417070	665	rural/clinic	No	22/06/2017	0	0	0
2011141090	665	rural/clinic	No	22/06/2017	0	0	0
2015417069	665	rural/clinic	No	22/06/2017	0	0	0
2015392899	665	rural/clinic	No	22/06/2017	0	0	0
2015417071	665	rural/clinic	No	22/06/2017	0	0	0
2015296070	665	rural/clinic	No	22/06/2017	0	0	0
2015340996	665	rural/clinic	No	22/06/2017	0	0	0
2015340997	665	rural/clinic	No	22/06/2017	0	0	0
2015296069	665	rural/clinic	No	22/06/2017	0	0	0
2015326832	665	rural/clinic	No	21/06/2017	0	0	0
2015340998	665	rural/clinic	No	22/06/2017	0	0	0
2012272777	665	rural/clinic	No	22/06/2017	0	0	0
2015324247	665	rural/clinic	No	22/06/2017	0	0	0
2015326833	665	rural/clinic	No	21/06/2017	0	0	0
2012272778	665	rural/clinic	No	22/06/2017	0	0	0
2015352737	508	rural/clinic	No	22/06/2017	0	0	0
2014346324	508	rural/clinic	No	22/06/2017	0	0	0
2014346320	508	rural/clinic	No	22/06/2017	0	0	0
2015399255	508	rural/clinic	No	22/06/2017	0	0	0
2015399253	508	rural/clinic	No	22/06/2017	0	0	0
2012272779	508	rural/clinic	No	22/06/2017	0	0	0
2014346327	508	rural/clinic	No	22/06/2017	0	0	0
2015399254	508	rural/clinic	No	22/06/2017	0	0	0
2014346325	508	rural/clinic	No	22/06/2017	0	0	0
2015399252	508	rural/clinic	No	22/06/2017	0	0	0
2015326834	508	rural/clinic	No	21/06/2017	0	0	0
2012350343	508	rural/clinic	No	22/06/2017	0	0	0
2015399251	508	rural/clinic	No	22/06/2017	0	0	0
2014346326	508	rural/clinic	No	22/06/2017	0	0	0
2015326835	508	rural/clinic	No	21/06/2017	0	0	0
2014346319	508	rural/clinic	No	22/06/2017	0	0	0
2012350346	508	rural/clinic	No	22/06/2017	0	0	0
2015337622	508	rural/clinic	No	20/06/2017	0	0	0
2015399258	508	rural/clinic	No	22/06/2017	0	0	0
2015324248	508	rural/clinic	No	22/06/2017	0	0	0
2015326836	508	rural/clinic	No	21/06/2017	0	0	0
2015372800	508	rural/clinic	No	22/06/2017	0	0	0
2015332029	508	rural/clinic	No	22/06/2017	0	0	0
2015399257	508	rural/clinic	No	22/06/2017	0	0	0
2015326837	508	rural/clinic	No	21/06/2017	0	0	0
2015352738	508	rural/clinic	No	22/06/2017	0	0	0
2015372427	508	rural/clinic	No	22/06/2017	0	0	0
2015399259	508	rural/clinic	No	22/06/2017	0	0	0
2015326838	508	rural/clinic	No	21/06/2017	0	0	0
2014383981	508	rural/clinic	No	22/06/2017	0	0	0
2015326839	508	rural/clinic	No	21/06/2017	0	0	0
2015324240	1	rural/clinic	No	22/06/2017	0	0	0
2015326840	1	rural/clinic	No	21/06/2017	0	0	0
2012317560	1	rural/clinic	No	22/06/2017	0	0	0
2015372428	1	rural/clinic	No	22/06/2017	0	0	0
2014368485	1	rural/clinic	No	22/06/2017	0	0	0
2015359763	1	rural/clinic	No	21/06/2017	0	0	0
2012317555	1	rural/clinic	No	22/06/2017	0	0	0
2015340011	1	rural/clinic	No	22/06/2017	0	0	0

2015342292	1	rural/clinic	No	20/06/2017	0	0	0
2015372429	1	rural/clinic	No	22/06/2017	0	0	0
2014383980	208	rural/clinic	No	22/06/2017	0	0	0
2015286317	208	rural/clinic	No	22/06/2017	0	0	0
2015342293	208	rural/clinic	No	20/06/2017	0	0	0
2015286318	208	rural/clinic	No	22/06/2017	0	0	0
2014368024	208	rural/clinic	No	22/06/2017	0	0	0
2015340992	530	rural/clinic	No	20/06/2017	0	0	0
2014368023	530	rural/clinic	No	22/06/2017	0	0	0
2015362819	530	rural/clinic	No	20/06/2017	0	0	0
2014334370	530	rural/clinic	No	22/06/2017	0	0	0
2015372430	530	rural/clinic	No	22/06/2017	0	0	0
2015286319	530	rural/clinic	No	22/06/2017	0	0	0
2015362820	402	rural/clinic	No	20/06/2017	0	0	0
2015332468	402	rural/clinic	No	22/06/2017	0	0	0
2015369194	402	rural/clinic	No	22/06/2017	0	0	0
2015372431	402	rural/clinic	No	22/06/2017	0	0	0
2015332470	402	rural/clinic	No	22/06/2017	0	0	0
2015332469	402	rural/clinic	No	22/06/2017	0	0	0
2015379507	402	rural/clinic	No	22/06/2017	0	0	0
2015360522	402	rural/clinic	No	22/06/2017	0	0	0
2014347029	402	rural/clinic	No	22/06/2017	0	0	0
2015360521	402	rural/clinic	No	22/06/2017	0	0	0
2014347028	402	rural/clinic	No	22/06/2017	0	0	0
2015360518	402	rural/clinic	No	22/06/2017	0	0	0
2014371586	402	rural/clinic	No	22/06/2017	0	0	0
2015362061	402	rural/clinic	No	22/06/2017	0	0	0
2015414462	402	rural/clinic	No	22/06/2017	0	0	0
2015352373	402	rural/clinic	No	22/06/2017	0	0	0
2011226486	402	rural/clinic	No	22/06/2017	0	0	0
2015376656	402	rural/clinic	No	22/06/2017	0	0	0
2014370437	402	rural/clinic	No	22/06/2017	0	0	0
2015360519	402	rural/clinic	No	22/06/2017	0	0	0
2015340138	402	rural/clinic	No	22/06/2017	0	0	0
2015376658	402	rural/clinic	No	22/06/2017	0	0	0
2011204480	402	rural/clinic	No	22/06/2017	0	0	0
2014347027	402	rural/clinic	No	22/06/2017	0	0	0
2015340137	666	rural/clinic	No	22/06/2017	0	0	0
2015415283	666	rural/clinic	No	22/06/2017	0	0	0
2015335390	666	rural/clinic	No	22/06/2017	0	0	0
2013260325	666	rural/clinic	No	22/06/2017	0	0	0
2013267896	666	rural/clinic	No	22/06/2017	0	0	0
2015340136	666	rural/clinic	No	22/06/2017	0	0	0
2015335391	666	rural/clinic	No	22/06/2017	0	0	0
2015340135	666	rural/clinic	No	22/06/2017	0	0	0
2015415282	666	rural/clinic	No	22/06/2017	0	0	0
2014372275	666	rural/clinic	No	22/06/2017	0	0	0
2015340139	666	rural/clinic	No	22/06/2017	0	0	0
2015355009	666	rural/clinic	No	22/06/2017	0	0	0
2015414394	666	rural/clinic	No	22/06/2017	0	0	0
2015340140	666	rural/clinic	No	22/06/2017	0	0	0
2011221336	666	rural/clinic	No	22/06/2017	0	0	0
2015414393	666	rural/clinic	No	22/06/2017	0	0	0
2012344082	666	rural/clinic	No	22/06/2017	0	0	0
2015414391	666	rural/clinic	No	22/06/2017	0	0	0
2014309590	666	rural/clinic	No	22/06/2017	0	0	0
2015355010	666	rural/clinic	No	22/06/2017	0	0	0
2015414389	666	rural/clinic	No	22/06/2017	0	0	0
2014317636	666	rural/clinic	No	22/06/2017	0	0	0
2015414388	666	rural/clinic	No	22/06/2017	0	0	0
2015414390	666	rural/clinic	No	22/06/2017	0	0	0
2015352767	666	rural/clinic	No	22/06/2017	0	0	0
2015352766	666	rural/clinic	No	22/06/2017	0	0	0
2015352765	531	rural/clinic	No	22/06/2017	0	0	0
2015404970	531	rural/clinic	No	22/06/2017	0	0	0
2015352764	531	rural/clinic	No	22/06/2017	0	0	0
2014317637	531	rural/clinic	No	22/06/2017	0	0	0
2015414392	531	rural/clinic	No	22/06/2017	0	0	0
2015336154	531	rural/clinic	No	22/06/2017	0	0	0
2015352763	531	rural/clinic	No	22/06/2017	0	0	0
2015415063	531	rural/clinic	No	22/06/2017	0	0	0
2015352762	531	rural/clinic	No	22/06/2017	0	0	0
2015336153	531	rural/clinic	No	22/06/2017	0	0	0
2014317638	531	rural/clinic	No	22/06/2017	0	0	0
2015326966	531	rural/clinic	No	22/06/2017	0	0	0
2015336152	531	rural/clinic	No	22/06/2017	0	0	0
2014317437	531	rural/clinic	No	22/06/2017	0	0	0
2015326965	531	rural/clinic	No	22/06/2017	0	0	0
2014321844	531	rural/clinic	No	22/06/2017	0	0	0
2014349233	531	rural/clinic	No	22/06/2017	0	0	0
2012365478	531	rural/clinic	No	22/06/2017	0	0	0
2014349234	531	rural/clinic	No	22/06/2017	0	0	0

2015406302	531	rural/clinic	No	22/06/2017	0	0	0
2014349236	531	rural/clinic	No	22/06/2017	0	0	0
2015369062	531	rural/clinic	No	22/06/2017	0	0	0
2015406311	531	rural/clinic	No	22/06/2017	0	0	0
2015373449	531	rural/clinic	No	22/06/2017	0	0	0
2015386270	531	rural/clinic	No	22/06/2017	0	0	0
2015404969	531	rural/clinic	No	22/06/2017	0	0	0
2015373448	531	rural/clinic	No	22/06/2017	0	0	0
2015386268	531	rural/clinic	No	22/06/2017	0	0	0
2011196018	531	rural/clinic	No	22/06/2017	0	0	0
2015386271	531	rural/clinic	No	22/06/2017	0	0	0
2015373447	531	rural/clinic	No	22/06/2017	0	0	0
2011196017	531	rural/clinic	No	22/06/2017	0	0	0
2015386269	531	rural/clinic	No	22/06/2017	0	0	0
2015373446	531	rural/clinic	No	22/06/2017	0	0	0
2015386272	817	rural/clinic	No	22/06/2017	0	0	0
2015373445	817	rural/clinic	No	22/06/2017	0	0	0
2015386273	817	rural/clinic	No	22/06/2017	0	0	0
2015303515	817	rural/clinic	No	22/06/2017	0	0	0
2015406002	817	rural/clinic	No	22/06/2017	0	0	0
2015406001	817	rural/clinic	No	22/06/2017	0	0	0
2014312272	817	rural/clinic	No	22/06/2017	0	0	0
2013262232	817	rural/clinic	No	22/06/2017	0	0	0
2015386721	817	rural/clinic	No	22/06/2017	0	0	0
2014347991	817	rural/clinic	No	22/06/2017	0	0	0
2012283598	817	rural/clinic	No	22/06/2017	0	0	0
2015406295	817	rural/clinic	No	22/06/2017	0	0	0
2015406296	817	rural/clinic	No	22/06/2017	0	0	0
2015406298	817	rural/clinic	No	22/06/2017	0	0	0
2015406299	817	rural/clinic	No	22/06/2017	0	0	0
2015406300	817	rural/clinic	No	22/06/2017	0	0	0
2015297273	817	rural/clinic	No	22/06/2017	0	0	0
2012376779	817	rural/clinic	No	22/06/2017	0	0	0
2015412777	817	rural/clinic	No	22/06/2017	0	0	0
2015412778	817	rural/clinic	No	22/06/2017	0	0	0
2014371587	817	rural/clinic	No	22/06/2017	0	0	0
2013255448	817	rural/clinic	No	22/06/2017	0	0	0
2014287168	817	rural/clinic	No	22/06/2017	0	0	0
2014378710	817	rural/clinic	No	22/06/2017	0	0	0
2014358341	817	rural/clinic	No	22/06/2017	0	0	0
2013272695	817	rural/clinic	No	22/06/2017	0	0	0
2014378709	817	rural/clinic	No	22/06/2017	0	0	0
2014287167	817	rural/clinic	No	22/06/2017	0	0	0
2014307336	817	rural/clinic	No	22/06/2017	0	0	0
2015340141	817	rural/clinic	No	22/06/2017	0	0	0
2015364522	817	rural/clinic	No	22/06/2017	0	0	0
2015364523	817	rural/clinic	No	22/06/2017	0	0	0
2015364505	817	rural/clinic	No	22/06/2017	0	0	0
2015386720	817	rural/clinic	No	22/06/2017	0	0	0
2014307335	817	rural/clinic	No	22/06/2017	0	0	0
2015386719	817	rural/clinic	No	22/06/2017	0	0	0
2015335148	817	rural/clinic	No	22/06/2017	0	0	0
2015355919	817	rural/clinic	No	22/06/2017	0	0	0
2015335147	817	rural/clinic	No	22/06/2017	0	0	0
2015386718	817	rural/clinic	No	22/06/2017	0	0	0
2015369058	817	rural/clinic	No	22/06/2017	0	0	0
2015335146	817	rural/clinic	No	22/06/2017	0	0	0
2015369057	817	rural/clinic	No	22/06/2017	0	0	0
2015339024	817	rural/clinic	No	22/06/2017	0	0	0
2015369059	817	rural/clinic	No	22/06/2017	0	0	0
2015335145	817	rural/clinic	No	22/06/2017	0	0	0
2015339025	817	rural/clinic	No	22/06/2017	0	0	0
2015369060	817	rural/clinic	No	22/06/2017	0	0	0
2015335144	817	rural/clinic	No	22/06/2017	0	0	0
2015369061	817	rural/clinic	No	22/06/2017	0	0	0
2015326062	584	rural/clinic	No	22/06/2017	0	0	0
2015335143	584	rural/clinic	No	22/06/2017	0	0	0
2015313244	584	rural/clinic	No	22/06/2017	0	0	0
2015313246	584	rural/clinic	No	22/06/2017	0	0	0
2014321843	584	rural/clinic	No	22/06/2017	0	0	0
2015313242	584	rural/clinic	No	22/06/2017	0	0	0
2015335109	584	rural/clinic	No	22/06/2017	0	0	0
2015313243	584	rural/clinic	No	22/06/2017	0	0	0
2014383531	584	rural/clinic	No	22/06/2017	0	0	0
2015335388	584	rural/clinic	No	22/06/2017	0	0	0
2015345065	584	rural/clinic	No	22/06/2017	0	0	0
2015332972	584	rural/clinic	No	22/06/2017	0	0	0
2015335389	584	rural/clinic	No	22/06/2017	0	0	0
2015345062	584	rural/clinic	No	22/06/2017	0	0	0
2015379506	584	rural/clinic	No	22/06/2017	0	0	0
2015345063	584	rural/clinic	No	22/06/2017	0	0	0
2013255447	584	rural/clinic	No	22/06/2017	0	0	0

2015345064	584	rural/clinic	No	22/06/2017	0	0	0
2015406305	584	rural/clinic	No	22/06/2017	0	0	0
2015406309	584	rural/clinic	No	22/06/2017	0	0	0
2015406310	584	rural/clinic	No	22/06/2017	0	0	0
2015406314	584	rural/clinic	No	22/06/2017	0	0	0
2015410451	584	rural/clinic	No	22/06/2017	0	0	0
2015406313	584	rural/clinic	No	22/06/2017	0	0	0
2011187029	584	rural/clinic	No	22/06/2017	0	0	0
2015406312	584	rural/clinic	No	22/06/2017	0	0	0
2015406306	584	rural/clinic	No	22/06/2017	0	0	0
2015406308	584	rural/clinic	No	22/06/2017	0	0	0
2015406303	584	rural/clinic	No	22/06/2017	0	0	0
2015406307	584	rural/clinic	No	22/06/2017	0	0	0
2015406304	584	rural/clinic	No	22/06/2017	0	0	0
2015406294	584	rural/clinic	No	22/06/2017	0	0	0
2015331735	584	rural/clinic	No	22/06/2017	0	0	0
2015373860	584	rural/clinic	No	22/06/2017	0	0	0
2015373859	584	rural/clinic	No	22/06/2017	0	0	0
2015373861	619	rural/clinic	No	22/06/2017	0	0	0
2015373858	619	rural/clinic	No	22/06/2017	0	0	0
2014357546	619	rural/clinic	No	22/06/2017	0	0	0
2014357542	619	rural/clinic	No	22/06/2017	0	0	0
2014357545	619	rural/clinic	No	22/06/2017	0	0	0
2014357540	619	rural/clinic	No	22/06/2017	0	0	0
2014357541	619	rural/clinic	No	22/06/2017	0	0	0
2014357543	619	rural/clinic	No	22/06/2017	0	0	0
2015360520	619	rural/clinic	No	22/06/2017	0	0	0
2015319273	619	rural/clinic	No	22/06/2017	0	0	0
2015319274	619	rural/clinic	No	22/06/2017	0	0	0
2015319275	619	rural/clinic	No	22/06/2017	0	0	0
2015319276	619	rural/clinic	No	22/06/2017	0	0	0
2015319277	619	rural/clinic	No	22/06/2017	0	0	0
200012Wrong	619	rural/clinic	No	26/06/2017	0	0	0
200012Wrong	619	rural/clinic	No	26/06/2017	0	0	0
2015344658	619	rural/clinic	No	20/06/2017	0	0	0
2015344658	619	rural/clinic	No	20/06/2017	0	0	0
2015368665	619	rural/clinic	No	20/06/2017	0	0	0
2015368665	619	rural/clinic	No	20/06/2017	0	0	0
2015368983	619	rural/clinic	No	20/06/2017	0	0	0
2015368983	619	rural/clinic	No	20/06/2017	0	0	0
2015362277	619	rural/clinic	No	20/06/2017	0	0	0
2015362276	619	rural/clinic	No	20/06/2017	0	0	0
2015406297	619	rural/clinic	No	09/06/2017	0	0	0
2015404729	403	rural/clinic	No	22/06/2017	0	0	0
2015289186	403	rural/clinic	No	22/06/2017	0	0	0
2014348237	403	rural/clinic	No	20/06/2017	0	0	0
2014348237	403	rural/clinic	No	20/06/2017	0	0	0
2015305083	403	rural/clinic	No	20/06/2017	0	0	0
2015305083	403	rural/clinic	No	20/06/2017	0	0	0
2015305084	403	rural/clinic	No	20/06/2017	0	0	0
2015305084	403	rural/clinic	No	20/06/2017	0	0	0
2015373856	403	rural/clinic	No	21/06/2017	0	0	0
2015373856	403	rural/clinic	No	21/06/2017	0	0	0
2015373857	403	rural/clinic	No	21/06/2017	0	0	0
2015373857	403	rural/clinic	No	21/06/2017	0	0	0
2014382444	403	rural/clinic	No	20/06/2017	0	0	0
2014382444	403	rural/clinic	No	20/06/2017	0	0	0
2015319278	403	rural/clinic	No	22/06/2017	0	0	0
2015358550	403	rural/clinic	No	22/06/2017	0	0	0
2012388255	403	rural/clinic	No	22/06/2017	0	0	0
2012388256	404	rural/clinic	No	22/06/2017	0	0	0
2012388257	404	rural/clinic	No	22/06/2017	0	0	0
2014362833	404	rural/clinic	No	22/06/2017	0	0	0
2014362832	404	rural/clinic	No	22/06/2017	0	0	0
2014362834	404	rural/clinic	No	22/06/2017	0	0	0
2014362831	404	rural/clinic	No	22/06/2017	0	0	0
2015357806	404	rural/clinic	No	22/06/2017	0	0	0
2015340710	404	rural/clinic	No	22/06/2017	0	0	0
2015340709	404	rural/clinic	No	22/06/2017	0	0	0
2010099926	404	rural/clinic	No	22/06/2017	0	0	0
2010099927	404	rural/clinic	No	22/06/2017	0	0	0
2015301363	404	rural/clinic	No	22/06/2017	0	0	0
2011179939	404	rural/clinic	No	22/06/2017	0	0	0
2014319091	404	rural/clinic	No	22/06/2017	0	0	0
2015326829	927	rural/clinic	No	21/06/2017	0	0	0
2015326829	387	rural/clinic	No	21/06/2017	0	0	0
2015313240	387	rural/clinic	No	20/06/2017	0	0	0
2015313240	387	rural/clinic	No	20/06/2017	0	0	0
2015326416	387	rural/clinic	No	20/06/2017	0	0	0
2015326416	387	rural/clinic	No	20/06/2017	0	0	0
2011136137	387	rural/clinic	No	26/06/2017	0	0	0
2011136133	387	rural/clinic	No	26/06/2017	0	0	0

2011136132	387	rural/clinic	No	26/06/2017	0	0	0
2011136129	387	rural/clinic	No	26/06/2017	0	0	0
2015339831	387	rural/clinic	No	26/06/2017	0	0	0
2015339154	387	rural/clinic	No	26/06/2017	0	0	0
2015339829	387	rural/clinic	No	26/06/2017	0	0	0
2015368894	387	rural/clinic	No	26/06/2017	0	0	0
2015339832	387	rural/clinic	No	26/06/2017	0	0	0
2015403296	387	rural/clinic	No	26/06/2017	0	0	0
2015403298	387	rural/clinic	No	26/06/2017	0	0	0
2012243578	387	rural/clinic	No	26/06/2017	0	0	0
2015388520	387	rural/clinic	No	26/06/2017	0	0	0
2014331614	387	rural/clinic	No	26/06/2017	0	0	0
2015397311	387	rural/clinic	No	26/06/2017	0	0	0
2015397309	387	rural/clinic	No	26/06/2017	0	0	0
2015397308	387	rural/clinic	No	26/06/2017	0	0	0
2015397307	387	rural/clinic	No	26/06/2017	0	0	0
2014309389	387	rural/clinic	No	27/06/2017	0	0	0
2015303514	387	rural/clinic	No	27/06/2017	0	0	0
2015334948	387	rural/clinic	No	27/06/2017	0	0	0
2011195784	387	rural/clinic	No	27/06/2017	0	0	0
2011139405	387	rural/clinic	No	27/06/2017	0	0	0
2011139406	387	rural/clinic	No	27/06/2017	0	0	0
2014335580	387	rural/clinic	No	27/06/2017	0	0	0
2015344094	387	rural/clinic	No	27/06/2017	0	0	0
2015344739	387	rural/clinic	No	27/06/2017	0	0	0
2015344095	387	rural/clinic	No	27/06/2017	0	0	0
2015344096	387	rural/clinic	No	27/06/2017	0	0	0
2015344097	387	rural/clinic	No	27/06/2017	0	0	0
2011137314	387	rural/clinic	No	27/06/2017	0	0	0
2015293843	387	rural/clinic	No	27/06/2017	0	0	0
2015334949	387	rural/clinic	No	27/06/2017	0	0	0
2015344452	387	rural/clinic	No	27/06/2017	0	0	0
2015344660	387	rural/clinic	No	27/06/2017	0	0	0
2014313667	387	rural/clinic	No	27/06/2017	0	0	0
2014313663	387	rural/clinic	No	27/06/2017	0	0	0
2014301354	387	rural/clinic	No	27/06/2017	0	0	0
2014301353	387	rural/clinic	No	27/06/2017	0	0	0
2014326464	387	rural/clinic	No	27/06/2017	0	0	0
2015342052	387	rural/clinic	No	27/06/2017	0	0	0
2015333854	387	rural/clinic	No	27/06/2017	0	0	0
2013261937	387	rural/clinic	No	27/06/2017	0	0	0
2011114845	387	rural/clinic	No	27/06/2017	0	0	0
2015349065	387	rural/clinic	No	27/06/2017	0	0	0
2011114844	387	rural/clinic	No	27/06/2017	0	0	0
2015349064	387	rural/clinic	No	27/06/2017	0	0	0
2012284575	387	rural/clinic	No	27/06/2017	0	0	0
2015363712	387	rural/clinic	No	27/06/2017	0	0	0
2015303122	387	rural/clinic	No	27/06/2017	0	0	0
2014313668	387	rural/clinic	No	12/06/2017	0	0	0
2015321602	387	rural/clinic	No	27/06/2017	0	0	0
2015293366	387	rural/clinic	No	27/06/2017	0	0	0
2015367220	387	rural/clinic	No	27/06/2017	0	0	0
2014358219	387	rural/clinic	No	27/06/2017	0	0	0
2012320389	387	rural/clinic	No	13/06/2017	0	0	0
2015367219	387	rural/clinic	No	27/06/2017	0	0	0
2012388390/D	387	rural/clinic	No	13/06/2017	0	0	0
2015367221	387	rural/clinic	No	27/06/2017	0	0	0
2015355746	387	rural/clinic	No	27/06/2017	0	0	0
2015332422	797	rural/clinic	No	27/06/2017	0	0	0
2012365479	797	rural/clinic	No	27/06/2017	0	0	0
2015332423	797	rural/clinic	No	27/06/2017	0	0	0
2014342533	797	rural/clinic	No	27/06/2017	0	0	0
2015332424	797	rural/clinic	No	27/06/2017	0	0	0
2012291365	797	rural/clinic	No	27/06/2017	0	0	0
2015332426	797	rural/clinic	No	27/06/2017	0	0	0
2012291367	797	rural/clinic	No	27/06/2017	0	0	0
2012291366	27	rural/clinic	No	27/06/2017	0	0	0
2015368671	27	rural/clinic	No	27/06/2017	0	0	0
2014342534	27	rural/clinic	No	27/06/2017	0	0	0
2015368672	27	rural/clinic	No	27/06/2017	0	0	0
2012259897	27	rural/clinic	No	27/06/2017	0	0	0
2014342535	27	rural/clinic	No	27/06/2017	0	0	0
2013244117	27	rural/clinic	No	27/06/2017	0	0	0
2014342532	27	rural/clinic	No	27/06/2017	0	0	0
2015407066	27	rural/clinic	No	27/06/2017	0	0	0
2015326967	27	rural/clinic	No	27/06/2017	0	0	0
2012296000	27	rural/clinic	No	27/06/2017	0	0	0
2015407065	27	rural/clinic	No	27/06/2017	0	0	0
2012295801	27	rural/clinic	No	27/06/2017	0	0	0
2015407064	27	rural/clinic	No	27/06/2017	0	0	0
2012295803	27	rural/clinic	No	27/06/2017	0	0	0
2015407063	27	rural/clinic	No	27/06/2017	0	0	0

2012295804	27	rural/clinic	No	27/06/2017	0	0	0
2015407062	27	rural/clinic	No	27/06/2017	0	0	0
2012295805	27	rural/clinic	No	27/06/2017	0	0	0
2015407061	27	rural/clinic	No	27/06/2017	0	0	0
2012295806	27	rural/clinic	No	27/06/2017	0	0	0
2011140763	27	rural/clinic	No	27/06/2017	0	0	0
2012295807	27	rural/clinic	No	27/06/2017	0	0	0
2011140764	27	rural/clinic	No	27/06/2017	0	0	0
2014358019	27	rural/clinic	No	27/06/2017	0	0	0
2014358020	27	rural/clinic	No	27/06/2017	0	0	0
2011140765	27	rural/clinic	No	27/06/2017	0	0	0
2014358021	27	rural/clinic	No	27/06/2017	0	0	0
2011140766	27	rural/clinic	No	27/06/2017	0	0	0
2014361226	27	rural/clinic	No	27/06/2017	0	0	0
2011140767	27	rural/clinic	No	27/06/2017	0	0	0
2011140768	27	rural/clinic	No	27/06/2017	0	0	0
2014357327	27	rural/clinic	No	27/06/2017	0	0	0
2011140769	27	rural/clinic	No	27/06/2017	0	0	0
2014357325	27	rural/clinic	No	27/06/2017	0	0	0
2011140770	27	rural/clinic	No	27/06/2017	0	0	0
2014357326	27	rural/clinic	No	27/06/2017	0	0	0
2014346286	27	rural/clinic	No	27/06/2017	0	0	0
2011140771/D	27	rural/clinic	No	27/06/2017	0	0	0
2014346285	27	rural/clinic	No	27/06/2017	0	0	0
2011140772	27	rural/clinic	No	27/06/2017	0	0	0
2014368685	27	rural/clinic	No	27/06/2017	0	0	0
2011140773	27	rural/clinic	No	27/06/2017	0	0	0
2015369935	27	rural/clinic	No	27/06/2017	0	0	0
2011140774	27	rural/clinic	No	27/06/2017	0	0	0
2015339153	27	rural/clinic	No	27/06/2017	0	0	0
2014332701	27	rural/clinic	No	27/06/2017	0	0	0
2015377848	27	rural/clinic	No	27/06/2017	0	0	0
2015377846	27	rural/clinic	No	27/06/2017	0	0	0
2014332703	27	rural/clinic	No	27/06/2017	0	0	0
2015377845	27	rural/clinic	No	27/06/2017	0	0	0
2014332704	27	rural/clinic	No	27/06/2017	0	0	0
2015368821	27	rural/clinic	No	27/06/2017	0	0	0
2014332705	27	rural/clinic	No	27/06/2017	0	0	0
2014375511	27	rural/clinic	No	27/06/2017	0	0	0
2014375512	27	rural/clinic	No	27/06/2017	0	0	0
2014358194	27	rural/clinic	No	27/06/2017	0	0	0
2013255266	27	rural/clinic	No	27/06/2017	0	0	0
2014363890	27	rural/clinic	No	27/06/2017	0	0	0
2013255267	27	rural/clinic	No	27/06/2017	0	0	0
2014363891	27	rural/clinic	No	27/06/2017	0	0	0
2014363892	27	rural/clinic	No	27/06/2017	0	0	0
2014363893	283	provincial	No	27/06/2017	0	1	0
2014290738	283	provincial	No	27/06/2017	0	1	0
2014290739	283	provincial	No	27/06/2017	0	1	0
2013256273	283	provincial	No	27/06/2017	0	1	0
2015368594	283	provincial	No	27/06/2017	0	1	0
2015368596	283	provincial	No	27/06/2017	0	1	0
2015368597	283	provincial	No	27/06/2017	0	1	0
2015368598	283	provincial	No	27/06/2017	0	1	0
2015368599	283	provincial	No	27/06/2017	0	1	0
2015368595	283	provincial	No	27/06/2017	0	1	0
2013264071	283	provincial	No	27/06/2017	0	1	0
2015359613	283	provincial	No	27/06/2017	0	1	0
2015363017	283	provincial	No	27/06/2017	0	1	0
2012350635	283	provincial	No	27/06/2017	0	1	0
2014350434	283	provincial	No	27/06/2017	0	1	0
2010099628	283	provincial	No	27/06/2017	0	1	0
2015359614	283	provincial	No	27/06/2017	0	1	0
2014350432	283	provincial	No	27/06/2017	0	1	0
2015331101	283	provincial	No	27/06/2017	0	1	0
2014350433	283	provincial	No	27/06/2017	0	1	0
2015363016	283	provincial	No	27/06/2017	0	1	0
2015359612	283	provincial	No	27/06/2017	0	1	0
2014308895	283	provincial	No	27/06/2017	0	1	0
2015359611	283	provincial	No	27/06/2017	0	1	0
2015405968	283	provincial	No	27/06/2017	0	1	0
2015405967	283	provincial	No	27/06/2017	0	1	0
2015331182	283	provincial	No	27/06/2017	0	1	0
2014308896	283	provincial	No	27/06/2017	0	1	0
2014287984	283	provincial	No	27/06/2017	0	1	0
2014308897	283	provincial	No	27/06/2017	0	1	0
2014350682	283	provincial	No	27/06/2017	0	1	0
2015363455	283	provincial	No	28/06/2017	0	1	0
2015382939	283	provincial	No	27/06/2017	0	1	0
2014314479	283	provincial	No	28/06/2017	0	1	0
2015382941	283	provincial	No	27/06/2017	0	1	0
2014314481	283	provincial	No	27/06/2017	0	1	0

2014291623	283	provincial	No	27/06/2017	0	1	0
2014314478	283	provincial	No	28/06/2017	0	1	0
2014288425	283	provincial	No	27/06/2017	0	1	0
2015355522	283	provincial	No	27/06/2017	0	1	0
2012374298	283	provincial	No	27/06/2017	0	1	0
2012374297	283	provincial	No	27/06/2017	0	1	0
2012374296	283	provincial	No	27/06/2017	0	1	0
2011133152	283	provincial	No	27/06/2017	0	1	0
2015385854	283	provincial	No	27/06/2017	0	1	0
2015338717	283	provincial	No	27/06/2017	0	1	0
2015324482	283	provincial	No	27/06/2017	0	1	0
2015384786	283	provincial	No	27/06/2017	0	1	0
2015337371	283	provincial	No	27/06/2017	0	1	0
2015337513	283	provincial	No	27/06/2017	0	1	0
2015337514	283	provincial	No	27/06/2017	0	1	0
2011136136	283	provincial	No	27/06/2017	0	1	0
2012363972	283	provincial	No	27/06/2017	0	1	0
2015376664	283	provincial	No	27/06/2017	0	1	0
2015376663	283	provincial	No	27/06/2017	0	1	0
2012363973	283	provincial	No	27/06/2017	0	1	0
2011136135	283	provincial	No	27/06/2017	0	1	0
2012363974	283	provincial	No	27/06/2017	0	1	0
2012363971	283	provincial	No	27/06/2017	0	1	0
2012294065	283	provincial	No	27/06/2017	0	1	0
2015376662	283	provincial	No	27/06/2017	0	1	0
2013285127	283	provincial	No	27/06/2017	0	1	0
2014314480	283	provincial	No	27/06/2017	0	1	0
2014366070	283	provincial	No	27/06/2017	0	1	0
2011136134	283	provincial	No	27/06/2017	0	1	0
2011136130	283	provincial	No	27/06/2017	0	1	0
2014287436	283	provincial	No	28/06/2017	0	1	0
2011136131	283	provincial	No	27/06/2017	0	1	0
2015328541	283	provincial	No	28/06/2017	0	1	0
2014316063	283	provincial	No	27/06/2017	0	1	0
2014316064	283	provincial	No	27/06/2017	0	1	0
2015391531	283	provincial	No	28/06/2017	0	1	0
2014316061	283	provincial	No	27/06/2017	0	1	0
2015328542	283	provincial	No	28/06/2017	0	1	0
2014316062	283	provincial	No	27/06/2017	0	1	0
2015368881	283	provincial	No	09/05/2017	0	1	0
2015368881	283	provincial	No	09/05/2017	0	1	0
2015362281	283	provincial	No	27/06/2017	0	1	0
2015390315	283	provincial	No	27/06/2017	0	1	0
2012322041	283	provincial	No	27/06/2017	0	1	0
2014335581	283	provincial	No	27/06/2017	0	1	0
2014327685	283	provincial	No	27/06/2017	0	1	0
2014382704	283	provincial	No	27/06/2017	0	1	0
2015368669	283	provincial	No	27/06/2017	0	1	0
2015368670	283	provincial	No	27/06/2017	0	1	0
2015344093	283	provincial	No	27/06/2017	0	1	0
2015384488	283	provincial	No	27/06/2017	0	1	0
2014290325	283	provincial	No	27/06/2017	0	1	0
2015340011	283	provincial	No	22/06/2017	0	1	0
2015340011	283	provincial	No	22/06/2017	0	1	0
2015415282	283	provincial	No	22/06/2017	0	1	0
2015415282	283	provincial	No	22/06/2017	0	1	0
2015386273	283	provincial	No	22/06/2017	0	1	0
2015386273	283	provincial	No	22/06/2017	0	1	0
2015386721	284	rural/clinic	No	22/06/2017	0	0	0
2015386721	284	rural/clinic	No	22/06/2017	0	0	0
2015286318	284	rural/clinic	No	22/06/2017	0	0	0
2015286318	284	rural/clinic	No	22/06/2017	0	0	0
2015414462	284	rural/clinic	No	22/06/2017	0	0	0
2015414462	284	rural/clinic	No	22/06/2017	0	0	0
2013262232	284	rural/clinic	No	22/06/2017	0	0	0
2013262232	173	district/faith-based	No	22/06/2017	1	0	0
2015402645	173	district/faith-based	No	08/06/2017	1	0	0
2015402646	173	district/faith-based	No	08/06/2017	1	0	0
2015402647	173	district/faith-based	No	08/06/2017	1	0	0
2015402648	173	district/faith-based	No	08/06/2017	1	0	0
2014325207	173	district/faith-based	No	29/06/2017	1	0	0
2015326841	173	district/faith-based	No	29/06/2017	1	0	0
2014325208	173	district/faith-based	No	29/06/2017	1	0	0
2014378490	173	district/faith-based	No	29/06/2017	1	0	0
2015286323	173	district/faith-based	No	29/06/2017	1	0	0
2015286325	173	district/faith-based	No	29/06/2017	1	0	0
2015286324	173	district/faith-based	No	29/06/2017	1	0	0
2013255450	173	district/faith-based	No	29/06/2017	1	0	0
2013255449	173	district/faith-based	No	29/06/2017	1	0	0
2015338864	173	district/faith-based	No	29/06/2017	1	0	0
2015338865	173	district/faith-based	No	29/06/2017	1	0	0
2015338866	173	district/faith-based	No	29/06/2017	1	0	0

2015338867	173	district/faith-based	No	29/06/2017	1	0	0
2014357544	173	district/faith-based	No	29/06/2017	1	0	0
2014357547	173	district/faith-based	No	29/06/2017	1	0	0
2015289187	173	district/faith-based	No	29/06/2017	1	0	0
2014370307	173	district/faith-based	No	29/06/2017	1	0	0
2014378491	173	district/faith-based	No	29/06/2017	1	0	0
2015326597	173	district/faith-based	No	29/06/2017	1	0	0
2015369064	173	district/faith-based	No	29/06/2017	1	0	0
2015326599	173	district/faith-based	No	29/06/2017	1	0	0
2015402649	466	rural/clinic	No	08/06/2017	0	0	0
2015402643	466	rural/clinic	No	29/06/2017	0	0	0
2015369065	466	rural/clinic	No	29/06/2017	0	0	0
2015369066	466	rural/clinic	No	29/06/2017	0	0	0
2015326595	466	rural/clinic	No	29/06/2017	0	0	0
2015326598	466	rural/clinic	No	29/06/2017	0	0	0
2015338466	466	rural/clinic	No	29/06/2017	0	0	0
2015369067	466	rural/clinic	No	29/06/2017	0	0	0
2015297276	466	rural/clinic	No	29/06/2017	0	0	0
2015369068	466	rural/clinic	No	29/06/2017	0	0	0
2011234898	466	rural/clinic	No	29/06/2017	0	0	0
2015297274	466	rural/clinic	No	29/06/2017	0	0	0
2015297275	466	rural/clinic	No	29/06/2017	0	0	0
2015369069	466	rural/clinic	No	29/06/2017	0	0	0
2015412779	466	rural/clinic	No	29/06/2017	0	0	0
2015369063	466	rural/clinic	No	27/06/2017	0	0	0
2015326842	466	rural/clinic	No	29/06/2017	0	0	0
2012388258	466	rural/clinic	No	29/06/2017	0	0	0
2015404733	466	rural/clinic	No	29/06/2017	0	0	0
2015326843	466	rural/clinic	No	29/06/2017	0	0	0
2012388259	466	rural/clinic	No	29/06/2017	0	0	0
2015404735	466	rural/clinic	No	29/06/2017	0	0	0
2015326844	466	rural/clinic	No	29/06/2017	0	0	0
2012388260	466	rural/clinic	No	29/06/2017	0	0	0
2015326845	466	rural/clinic	No	29/06/2017	0	0	0
2015326846	466	rural/clinic	No	29/06/2017	0	0	0
2012388261	466	rural/clinic	No	29/06/2017	0	0	0
2015326847	466	rural/clinic	No	29/06/2017	0	0	0
2015404736	466	rural/clinic	No	29/06/2017	0	0	0
2015326848	466	rural/clinic	No	29/06/2017	0	0	0
2012388262	466	rural/clinic	No	29/06/2017	0	0	0
2015326849	466	rural/clinic	No	29/06/2017	0	0	0
2015404737	466	rural/clinic	No	29/06/2017	0	0	0
2015326850	466	rural/clinic	No	29/06/2017	0	0	0
2015326851	466	rural/clinic	No	29/06/2017	0	0	0
2012388263/D	466	rural/clinic	No	29/06/2017	0	0	0
2015404738	466	rural/clinic	No	29/06/2017	0	0	0
2015326852	466	rural/clinic	No	29/06/2017	0	0	0
2012388264	466	rural/clinic	No	29/06/2017	0	0	0
2015404739	466	rural/clinic	No	29/06/2017	0	0	0
2015326853	466	rural/clinic	No	29/06/2017	0	0	0
2012388265	466	rural/clinic	No	29/06/2017	0	0	0
2015404740	466	rural/clinic	No	29/06/2017	0	0	0
2015326960	466	rural/clinic	No	29/06/2017	0	0	0
2015404741	466	rural/clinic	No	29/06/2017	0	0	0
2012359166	466	rural/clinic	No	29/06/2017	0	0	0
2015404742	466	rural/clinic	No	29/06/2017	0	0	0
2015362757	466	rural/clinic	No	29/06/2017	0	0	0
2015404743	466	rural/clinic	No	29/06/2017	0	0	0
2014351230	466	rural/clinic	No	29/06/2017	0	0	0
2015408680	466	rural/clinic	No	29/06/2017	0	0	0
2014305947	466	rural/clinic	No	29/06/2017	0	0	0
2015408681	466	rural/clinic	No	29/06/2017	0	0	0
2015328073	466	rural/clinic	No	29/06/2017	0	0	0
2015364610	466	rural/clinic	No	29/06/2017	0	0	0
2015328074	466	rural/clinic	No	29/06/2017	0	0	0
2015364520	466	rural/clinic	No	29/06/2017	0	0	0
2015328075	466	rural/clinic	No	29/06/2017	0	0	0
2015332913	466	rural/clinic	No	29/06/2017	0	0	0
2015328076	466	rural/clinic	No	29/06/2017	0	0	0
2015352741	466	rural/clinic	No	29/06/2017	0	0	0
2015406315	466	rural/clinic	No	29/06/2017	0	0	0
2015289573	466	rural/clinic	No	27/06/2017	0	0	0
2014365951	307	rural/clinic	No	29/06/2017	0	0	0
2015406316	307	rural/clinic	No	29/06/2017	0	0	0
2014346328	307	rural/clinic	No	29/06/2017	0	0	0
2014335578	307	rural/clinic	No	27/06/2017	0	0	0
2015352739	307	rural/clinic	No	29/06/2017	0	0	0
2014335579	307	rural/clinic	No	27/06/2017	0	0	0
2015406317	307	rural/clinic	No	29/06/2017	0	0	0
2015373718	307	rural/clinic	No	29/06/2017	0	0	0
2014314482	307	rural/clinic	No	27/06/2017	0	0	0
2015352740	307	rural/clinic	No	29/06/2017	0	0	0

2015373719	307	rural/clinic	No	29/06/2017	0	0	0
2014372337	307	rural/clinic	No	29/06/2017	0	0	0
2014309388	307	rural/clinic	No	27/06/2017	0	0	0
2015373720	307	rural/clinic	No	29/06/2017	0	0	0
2015328499	307	rural/clinic	No	29/05/2017	0	0	0
2012260087	307	rural/clinic	No	27/06/2017	0	0	0
2015328473	307	rural/clinic	No	29/06/2017	0	0	0
2015344659	307	rural/clinic	No	27/06/2017	0	0	0
2015340351	307	rural/clinic	No	29/06/2017	0	0	0
2015373721	307	rural/clinic	No	29/06/2017	0	0	0
2015355747	307	rural/clinic	No	27/06/2017	0	0	0
2015340352	307	rural/clinic	No	29/06/2017	0	0	0
2015373722	307	rural/clinic	No	29/06/2017	0	0	0
2015340354	307	rural/clinic	No	29/06/2017	0	0	0
2015340355	307	rural/clinic	No	29/06/2017	0	0	0
2014309385/D	307	rural/clinic	No	27/06/2017	0	0	0
2015340356	307	rural/clinic	No	29/06/2017	0	0	0
2015289572	307	rural/clinic	No	27/06/2017	0	0	0
2015340357	307	rural/clinic	No	29/06/2017	0	0	0
2015334634	307	rural/clinic	No	27/06/2017	0	0	0
2015373723	307	rural/clinic	No	29/06/2017	0	0	0
2015373724	209	rural/clinic	No	29/06/2017	0	0	0
2015334635	209	rural/clinic	No	27/06/2017	0	0	0
2015340358	209	rural/clinic	No	29/06/2017	0	0	0
2015373725	209	rural/clinic	No	29/06/2017	0	0	0
2015344898	209	rural/clinic	No	27/06/2017	0	0	0
2015340359	209	rural/clinic	No	29/06/2017	0	0	0
2015344899	209	rural/clinic	No	27/06/2017	0	0	0
2015340360	209	rural/clinic	No	29/06/2017	0	0	0
2015373726	209	rural/clinic	No	29/06/2017	0	0	0
2015344900	209	rural/clinic	No	27/06/2017	0	0	0
2015369196	209	rural/clinic	No	29/06/2017	0	0	0
2015369197	209	rural/clinic	No	29/06/2017	0	0	0
2015344892	388	rural/clinic	No	27/06/2017	0	0	0
2011137302	388	rural/clinic	No	27/06/2017	0	0	0
2014316065	388	rural/clinic	No	29/06/2017	0	0	0
2015344897	388	rural/clinic	No	27/06/2017	0	0	0
2014316066	388	rural/clinic	No	29/06/2017	0	0	0
2015344893	388	rural/clinic	No	27/06/2017	0	0	0
2014316067	767	district/faith-based	No	29/06/2017	1	0	0
2011137301	767	district/faith-based	No	27/06/2017	1	0	0
2015373727	767	district/faith-based	No	29/06/2017	1	0	0
2014316068	767	district/faith-based	No	29/06/2017	1	0	0
2015373728	767	district/faith-based	No	29/06/2017	1	0	0
2014316069	767	district/faith-based	No	29/06/2017	1	0	0
2011141106	767	district/faith-based	No	29/06/2017	1	0	0
2011141107	767	district/faith-based	No	29/06/2017	1	0	0
2015414395	767	district/faith-based	No	29/06/2017	1	0	0
2015335392	767	district/faith-based	No	29/06/2017	1	0	0
2015373729	767	district/faith-based	No	29/06/2017	1	0	0
2014347030	767	district/faith-based	No	29/06/2017	1	0	0
2014347031	767	district/faith-based	No	29/06/2017	1	0	0
2015373730	767	district/faith-based	No	29/06/2017	1	0	0
2014347032	767	district/faith-based	No	29/06/2017	1	0	0
2014347033	767	district/faith-based	No	29/06/2017	1	0	0
2015373731	767	district/faith-based	No	29/06/2017	1	0	0
2015335149	767	district/faith-based	No	29/06/2017	1	0	0
2015373732	767	district/faith-based	No	29/06/2017	1	0	0
2015373733	767	district/faith-based	No	29/06/2017	1	0	0
2015373734	767	district/faith-based	No	29/06/2017	1	0	0
2015373735	767	district/faith-based	No	29/06/2017	1	0	0
2015373736	767	district/faith-based	No	29/06/2017	1	0	0
2015373737	767	district/faith-based	No	29/06/2017	1	0	0
2015373738	767	district/faith-based	No	29/06/2017	1	0	0
2015373739	767	district/faith-based	No	29/06/2017	1	0	0
2015386734	767	district/faith-based	No	29/06/2017	1	0	0
2015386733	767	district/faith-based	No	29/06/2017	1	0	0
2015386724	767	district/faith-based	No	29/06/2017	1	0	0
2015386725	767	district/faith-based	No	29/06/2017	1	0	0
2015386726	767	district/faith-based	No	29/06/2017	1	0	0
2015386727	767	district/faith-based	No	29/06/2017	1	0	0
2015386728	767	district/faith-based	No	29/06/2017	1	0	0
2015386729	767	district/faith-based	No	29/06/2017	1	0	0
2015386730	767	district/faith-based	No	29/06/2017	1	0	0
2015386731	767	district/faith-based	No	29/06/2017	1	0	0
2015340014	767	district/faith-based	No	22/06/2017	1	0	0
2015373710	767	district/faith-based	No	22/06/2017	1	0	0
2015373710	767	district/faith-based	No	22/06/2017	1	0	0
2015373708	767	district/faith-based	No	22/06/2017	1	0	0
2015373708	767	district/faith-based	No	22/06/2017	1	0	0
2015386732	767	district/faith-based	No	29/06/2017	1	0	0
2017123782	308	rural/clinic	No	22/06/2017	0	0	0

2017123782	308	rural/clinic	No	22/06/2017	0	0	0
2015361158	308	rural/clinic	No	29/06/2017	0	0	0
2012272779	308	rural/clinic	No	22/06/2017	0	0	0
2012272779	308	rural/clinic	No	22/06/2017	0	0	0
2015361159	308	rural/clinic	No	29/06/2017	0	0	0
2015373861	308	rural/clinic	No	22/06/2017	0	0	0
2015373861	308	rural/clinic	No	22/06/2017	0	0	0
2015361160	308	rural/clinic	No	29/06/2017	0	0	0
2013255447	405	rural/clinic	No	22/06/2017	0	0	0
2013255447	405	rural/clinic	No	22/06/2017	0	0	0
2015386723	405	rural/clinic	No	29/06/2017	0	0	0
2014312273	405	rural/clinic	No	29/06/2017	0	0	0
2015361151	405	rural/clinic	No	29/06/2017	0	0	0
2015361152	405	rural/clinic	No	29/06/2017	0	0	0
2015361153	405	rural/clinic	No	29/06/2017	0	0	0
2015361154	405	rural/clinic	No	29/06/2017	0	0	0
2015361155	405	rural/clinic	No	29/06/2017	0	0	0
2015361156	405	rural/clinic	No	29/06/2017	0	0	0
2015361157	405	rural/clinic	No	29/06/2017	0	0	0
2015415658	405	rural/clinic	No	29/06/2017	0	0	0
2015415659	405	rural/clinic	No	29/06/2017	0	0	0
2015313247	405	rural/clinic	No	29/06/2017	0	0	0
2015313248	405	rural/clinic	No	29/06/2017	0	0	0
2015335150	405	rural/clinic	No	29/06/2017	0	0	0
2015313249	405	rural/clinic	No	29/06/2017	0	0	0
2015364301	405	rural/clinic	No	29/06/2017	0	0	0
2015364302	405	rural/clinic	No	29/06/2017	0	0	0
2015364303	405	rural/clinic	No	29/06/2017	0	0	0
2015364304	405	rural/clinic	No	29/06/2017	0	0	0
2015377179	405	rural/clinic	No	29/06/2017	0	0	0
2014314225	405	rural/clinic	No	29/06/2017	0	0	0
2011137303	405	rural/clinic	No	27/06/2017	0	0	0
2015313250	405	rural/clinic	No	29/06/2017	0	0	0
2011137304	405	rural/clinic	No	27/06/2017	0	0	0
2015382551	405	rural/clinic	No	29/06/2017	0	0	0
2015382552	405	rural/clinic	No	29/06/2017	0	0	0
2015368993	405	rural/clinic	No	27/06/2017	0	0	0
2015319279	640	rural/clinic	No	29/06/2017	0	0	0
2015368992	640	rural/clinic	No	27/06/2017	0	0	0
2015319280	640	rural/clinic	No	29/06/2017	0	0	0
2015319281	640	rural/clinic	No	29/06/2017	0	0	0
2015368991	640	rural/clinic	No	27/06/2017	0	0	0
2015319282	640	rural/clinic	No	29/06/2017	0	0	0
2015368990	640	rural/clinic	No	27/06/2017	0	0	0
2015319283	640	rural/clinic	No	29/06/2017	0	0	0
2015319284	640	rural/clinic	No	29/06/2017	0	0	0
2015368989	640	rural/clinic	No	27/06/2017	0	0	0
2015406003	640	rural/clinic	No	29/06/2017	0	0	0
2015368987	640	rural/clinic	No	27/06/2017	0	0	0
2015406004	640	rural/clinic	No	29/06/2017	0	0	0
2013252935	640	rural/clinic	No	29/06/2017	0	0	0
2014306037	640	rural/clinic	No	27/06/2017	0	0	0
2014306035	640	rural/clinic	No	27/06/2017	0	0	0
2014306033	640	rural/clinic	No	27/06/2017	0	0	0
2014314224	640	rural/clinic	No	29/06/2017	0	0	0
2014306034	640	rural/clinic	No	27/06/2017	0	0	0
2014306036	467	rural/clinic	No	27/06/2017	0	0	0
2014372276	467	rural/clinic	No	29/06/2017	0	0	0
2015302439	467	rural/clinic	No	27/06/2017	0	0	0
2014372445	467	rural/clinic	No	29/06/2017	0	0	0
2015357200	467	rural/clinic	No	27/06/2017	0	0	0
2015332974	467	rural/clinic	No	29/06/2017	0	0	0
2015357535	467	rural/clinic	No	27/06/2017	0	0	0
2014371711	467	rural/clinic	No	29/06/2017	0	0	0
2015325206	467	rural/clinic	No	27/06/2017	0	0	0
2015325937	467	rural/clinic	No	27/06/2017	0	0	0
2015332973	467	rural/clinic	No	29/06/2017	0	0	0
2015357301	467	rural/clinic	No	27/06/2017	0	0	0
2015340999	467	rural/clinic	No	29/06/2017	0	0	0
2012368545	467	rural/clinic	No	27/06/2017	0	0	0
2015341000	467	rural/clinic	No	29/06/2017	0	0	0
2015325207	467	rural/clinic	No	27/06/2017	0	0	0
2015369001	467	rural/clinic	No	29/06/2017	0	0	0
2015326422	467	rural/clinic	No	27/06/2017	0	0	0
2015369002	286	rural/clinic	No	29/06/2017	0	0	0
2015360523	286	rural/clinic	No	29/06/2017	0	0	0
2015326423	286	rural/clinic	No	27/06/2017	0	0	0
2015326424	286	rural/clinic	No	27/06/2017	0	0	0
2015360524	286	rural/clinic	No	29/06/2017	0	0	0
2015326425	286	rural/clinic	No	27/06/2017	0	0	0
2015360525	286	rural/clinic	No	29/06/2017	0	0	0
2015326426	286	rural/clinic	No	27/06/2017	0	0	0

2015360526	286	rural/clinic	No	29/06/2017	0	0	0
2015418866	286	rural/clinic	No	29/06/2017	0	0	0
2015326427	286	rural/clinic	No	27/06/2017	0	0	0
2015332362	286	rural/clinic	No	29/06/2017	0	0	0
2015326428	286	rural/clinic	No	27/06/2017	0	0	0
2015332363	286	rural/clinic	No	29/06/2017	0	0	0
2015332364	286	rural/clinic	No	29/06/2017	0	0	0
2015326429	286	rural/clinic	No	27/06/2017	0	0	0
2014377415	286	rural/clinic	No	27/06/2017	0	0	0
2015297344	286	rural/clinic	No	27/06/2017	0	0	0
2015379325	286	rural/clinic	No	29/06/2017	0	0	0
2015379326	286	rural/clinic	No	29/06/2017	0	0	0
2015297345	286	rural/clinic	No	27/06/2017	0	0	0
2015379327	286	rural/clinic	No	29/06/2017	0	0	0
2014377414	286	rural/clinic	No	27/06/2017	0	0	0
2014319524	286	rural/clinic	No	29/06/2017	0	0	0
2014328445	286	rural/clinic	No	27/06/2017	0	0	0
2015297493	286	rural/clinic	No	27/06/2017	0	0	0
2015286322	286	rural/clinic	No	29/06/2017	0	0	0
2015297494	248	rural/clinic	No	27/06/2017	0	0	0
2014350960	248	rural/clinic	No	29/06/2017	0	0	0
2015297492	248	rural/clinic	No	27/06/2017	0	0	0
2014301351	248	rural/clinic	No	27/06/2017	0	0	0
2014301352	248	rural/clinic	No	27/06/2017	0	0	0
2014326148	248	rural/clinic	No	27/06/2017	0	0	0
2015383851	248	rural/clinic	No	27/06/2017	0	0	0
2015383852	248	rural/clinic	No	27/06/2017	0	0	0
2014335848	248	rural/clinic	No	27/06/2017	0	0	0
2014335847	248	rural/clinic	No	27/06/2017	0	0	0
2014335846	248	rural/clinic	No	27/06/2017	0	0	0
2015333108	248	rural/clinic	No	27/06/2017	0	0	0
2014335844	248	rural/clinic	No	27/06/2017	0	0	0
2014350961	248	rural/clinic	No	29/06/2017	0	0	0
2014385692	248	rural/clinic	No	29/06/2017	0	0	0
2014385693	248	rural/clinic	No	29/06/2017	0	0	0
2014385694	248	rural/clinic	No	29/06/2017	0	0	0
2015373862	248	rural/clinic	No	29/06/2017	0	0	0
2014382301	248	rural/clinic	No	27/06/2017	0	0	0
2015383736	248	rural/clinic	No	27/06/2017	0	0	0
2013267897	248	rural/clinic	No	29/06/2017	0	0	0
2014325206	248	rural/clinic	No	29/06/2017	0	0	0
2015383735	248	rural/clinic	No	27/06/2017	0	0	0
2015297151	248	rural/clinic	No	27/06/2017	0	0	0
2015297152	248	rural/clinic	No	27/06/2017	0	0	0
2012349289	585	rural/clinic	No	30/06/2017	0	0	0
2012305815	585	rural/clinic	No	27/06/2017	0	0	0
2012305816	585	rural/clinic	No	27/06/2017	0	0	0
2015403302	585	rural/clinic	No	30/06/2017	0	0	0
2012253243	585	rural/clinic	No	27/06/2017	0	0	0
2015359610	585	rural/clinic	No	27/06/2017	0	0	0
2012349288	585	rural/clinic	No	30/06/2017	0	0	0
2013271697	585	rural/clinic	No	27/06/2017	0	0	0
2015355013	585	rural/clinic	No	30/06/2017	0	0	0
2015349066	585	rural/clinic	No	27/06/2017	0	0	0
2015337991	585	rural/clinic	No	27/06/2017	0	0	0
2012358217	585	rural/clinic	No	30/06/2017	0	0	0
2015363457	585	rural/clinic	No	27/06/2017	0	0	0
2014343877	585	rural/clinic	No	27/06/2017	0	0	0
2015294880	585	rural/clinic	No	27/06/2017	0	0	0
2015294881	585	rural/clinic	No	27/06/2017	0	0	0
2015355011	144	rural/clinic	No	30/06/2017	0	0	0
2015289335	145	rural/clinic	No	27/06/2017	0	0	0
2015355012	145	rural/clinic	No	30/06/2017	0	0	0
2014369098	145	rural/clinic	No	30/06/2017	0	0	0
2014363653	145	rural/clinic	No	27/06/2017	0	0	0
2015287803	145	rural/clinic	No	30/06/2017	0	0	0
2014363657	145	rural/clinic	No	27/06/2017	0	0	0
2015287802	145	rural/clinic	No	30/06/2017	0	0	0
2014363656	145	rural/clinic	No	27/06/2017	0	0	0
2015320896	145	rural/clinic	No	30/06/2017	0	0	0
2014363655	145	rural/clinic	No	27/06/2017	0	0	0
2014363654	145	rural/clinic	No	27/06/2017	0	0	0
2015320895	145	rural/clinic	No	30/06/2017	0	0	0
2014363652	145	rural/clinic	No	27/06/2017	0	0	0
2012325698	145	rural/clinic	No	30/06/2017	0	0	0
2015337993	145	rural/clinic	No	27/06/2017	0	0	0
2012325695	667	rural/clinic	No	30/06/2017	0	0	0
2015363456	667	rural/clinic	No	27/06/2017	0	0	0
2012325696	667	rural/clinic	No	30/06/2017	0	0	0
2015337990	667	rural/clinic	No	28/06/2017	0	0	0
2012325697	667	rural/clinic	No	30/06/2017	0	0	0
2015355784	667	rural/clinic	No	27/06/2017	0	0	0

2015337992	667	rural/clinic	No	27/06/2017	0	0	0
2014302526	667	rural/clinic	No	30/06/2017	0	0	0
2015360603	667	rural/clinic	No	27/06/2017	0	0	0
2012261450	667	rural/clinic	No	27/06/2017	0	0	0
2015335969	667	rural/clinic	No	27/06/2017	0	0	0
2015335968	667	rural/clinic	No	27/06/2017	0	0	0
2015360604	700	rural/clinic	No	27/06/2017	0	0	0
2015355783	700	rural/clinic	No	27/06/2017	0	0	0
2014301355	768	rural/clinic	No	27/06/2017	0	0	0
2014350432	768	rural/clinic	No	27/06/2017	0	0	0
2015376662	768	rural/clinic	No	27/06/2017	0	0	0
2015376662	768	rural/clinic	No	27/06/2017	0	0	0
2014366070	768	rural/clinic	No	27/06/2017	0	0	0
2014366070	768	rural/clinic	No	27/06/2017	0	0	0
2015324482	768	rural/clinic	No	27/06/2017	0	0	0
2015324482	768	rural/clinic	No	27/06/2017	0	0	0
2015376664	768	rural/clinic	No	27/06/2017	0	0	0
2015376664	768	rural/clinic	No	27/06/2017	0	0	0
2015335391	768	rural/clinic	No	22/06/2017	0	0	0
2015335391	768	rural/clinic	No	22/06/2017	0	0	0
2011137314	768	rural/clinic	No	27/06/2017	0	0	0
2011137314	768	rural/clinic	No	27/06/2017	0	0	0
2015335147	768	rural/clinic	No	22/06/2017	0	0	0
2015335147	768	rural/clinic	No	22/06/2017	0	0	0
2015397321	768	rural/clinic	No	03/07/2017	0	0	0
2014365390	768	rural/clinic	No	03/07/2017	0	0	0
2011144827	768	rural/clinic	No	03/07/2017	0	0	0
2014365389	768	rural/clinic	No	03/07/2017	0	0	0
2014365392	768	rural/clinic	No	03/07/2017	0	0	0
2014365391	768	rural/clinic	No	03/07/2017	0	0	0
2014289541	768	rural/clinic	No	03/07/2017	0	0	0
2012339735	768	rural/clinic	No	03/07/2017	0	0	0
2014289543	768	rural/clinic	No	03/07/2017	0	0	0
2014289542	768	rural/clinic	No	03/07/2017	0	0	0
2011144825	768	rural/clinic	No	03/07/2017	0	0	0
2014289544	768	rural/clinic	No	03/07/2017	0	0	0
2015397312	768	rural/clinic	No	03/07/2017	0	0	0
2015397313	768	rural/clinic	No	03/07/2017	0	0	0
2015397314	768	rural/clinic	No	03/07/2017	0	0	0
2015397315	768	rural/clinic	No	03/07/2017	0	0	0
2015397316	768	rural/clinic	No	03/07/2017	0	0	0
2015397317	768	rural/clinic	No	03/07/2017	0	0	0
2015397318	768	rural/clinic	No	03/07/2017	0	0	0
2015397319	768	rural/clinic	No	03/07/2017	0	0	0
2015397320	768	rural/clinic	No	03/07/2017	0	0	0
2014325207	768	rural/clinic	No	29/06/2017	0	0	0
2014325207	768	rural/clinic	No	29/06/2017	0	0	0
2014335578	768	rural/clinic	No	27/06/2017	0	0	0
2014335578	768	rural/clinic	No	27/06/2017	0	0	0
2015326844	768	rural/clinic	No	29/06/2017	0	0	0
2015326844	768	rural/clinic	No	29/06/2017	0	0	0
2012291365	768	rural/clinic	No	27/06/2017	0	0	0
2012291365	768	rural/clinic	No	27/06/2017	0	0	0
2014342534	768	rural/clinic	No	27/06/2017	0	0	0
2012295801	768	rural/clinic	No	27/06/2017	0	0	0
2015352741	768	rural/clinic	No	29/06/2017	0	0	0
2015352741	768	rural/clinic	No	29/06/2017	0	0	0
2015352739	768	rural/clinic	No	29/06/2017	0	0	0
2015352739	768	rural/clinic	No	29/06/2017	0	0	0
2015340354	768	rural/clinic	No	29/06/2017	0	0	0
2015338865	768	rural/clinic	No	29/06/2017	0	0	0
2015294880	365	rural/clinic	No	27/06/2017	0	0	0
2015289335	365	rural/clinic	No	27/06/2017	0	0	0
2014363656	365	rural/clinic	No	27/06/2017	0	0	0
2015355784	365	rural/clinic	No	27/06/2017	0	0	0
2015355784	769	rural/clinic	No	27/06/2017	0	0	0
2015368989	769	rural/clinic	No	27/06/2017	0	0	0
2015368989	769	rural/clinic	No	27/06/2017	0	0	0
2015357535	769	rural/clinic	No	27/06/2017	0	0	0
2015357535	769	rural/clinic	No	27/06/2017	0	0	0
2015297151	769	rural/clinic	No	27/06/2017	0	0	0
2015297151	769	rural/clinic	No	27/06/2017	0	0	0
2015319280	769	rural/clinic	No	29/06/2017	0	0	0
2014385694	769	rural/clinic	No	29/06/2017	0	0	0
2014385694	769	rural/clinic	No	29/06/2017	0	0	0
2015373862	769	rural/clinic	No	29/06/2017	0	0	0
2015373862	769	rural/clinic	No	29/06/2017	0	0	0
2015355748	769	rural/clinic	No	04/07/2017	0	0	0
2015355749	769	rural/clinic	No	04/07/2017	0	0	0
2015363459	769	rural/clinic	No	04/07/2017	0	0	0
2015363462	769	rural/clinic	No	04/07/2017	0	0	0
2015363460	769	rural/clinic	No	04/07/2017	0	0	0

2014327686	669	rural/clinic	No	04/07/2017	0	0	0
2014327687	669	rural/clinic	No	04/07/2017	0	0	0
2015334802	669	rural/clinic	No	04/07/2017	0	0	0
2011135672	669	rural/clinic	No	04/07/2017	0	0	0
2011135674	669	rural/clinic	No	04/07/2017	0	0	0
2015294623	669	rural/clinic	No	04/07/2017	0	0	0
2014382705	669	rural/clinic	No	04/07/2017	0	0	0
2015344098	669	rural/clinic	No	04/07/2017	0	0	0
2015344100	669	rural/clinic	No	04/07/2017	0	0	0
2015344661	669	rural/clinic	No	04/07/2017	0	0	0
2015289188	669	rural/clinic	No	04/07/2017	0	0	0
2015344662	669	rural/clinic	No	04/07/2017	0	0	0
2015368895	669	rural/clinic	No	04/07/2017	0	0	0
2015344740	669	rural/clinic	No	04/07/2017	0	0	0
2014375513	669	rural/clinic	No	04/07/2017	0	0	0
2015414260	669	rural/clinic	No	04/07/2017	0	0	0
2015287317	669	rural/clinic	No	04/07/2017	0	0	0
2015414263	669	rural/clinic	No	04/07/2017	0	0	0
2015287318	669	rural/clinic	No	04/07/2017	0	0	0
2015414261	669	rural/clinic	No	04/07/2017	0	0	0
2015324572	669	rural/clinic	No	04/07/2017	0	0	0
2015377843	669	rural/clinic	No	04/07/2017	0	0	0
2014365391	669	rural/clinic	No	03/07/2017	0	0	0
2014365391	669	rural/clinic	No	03/07/2017	0	0	0
2014289544	818	rural/clinic	No	03/07/2017	0	0	0
2015377844	818	rural/clinic	No	04/07/2017	0	0	0
2012291369	818	rural/clinic	No	04/07/2017	0	0	0
2015344453	818	rural/clinic	No	04/07/2017	0	0	0
2012291368	818	rural/clinic	No	04/07/2017	0	0	0
2015344454	818	rural/clinic	No	04/07/2017	0	0	0
2015287380	818	rural/clinic	No	04/07/2017	0	0	0
2015344455	818	rural/clinic	No	04/07/2017	0	0	0
2015287381	818	rural/clinic	No	04/07/2017	0	0	0
2015287382	818	rural/clinic	No	04/07/2017	0	0	0
2012295808/D	818	rural/clinic	No	04/07/2017	0	0	0
2015287383	818	rural/clinic	No	04/07/2017	0	0	0
2015413924	818	rural/clinic	No	04/07/2017	0	0	0
2014327519	818	rural/clinic	No	04/07/2017	0	0	0
2014327520	818	rural/clinic	No	04/07/2017	0	0	0
2015413925	818	rural/clinic	No	04/07/2017	0	0	0
2015339769	818	rural/clinic	No	04/07/2017	0	0	0
2015413217	818	rural/clinic	No	04/07/2017	0	0	0
2015339768	818	rural/clinic	No	04/07/2017	0	0	0
2015405627	818	rural/clinic	No	04/07/2017	0	0	0
2015339767	818	rural/clinic	No	04/07/2017	0	0	0
2015367252	818	rural/clinic	No	04/07/2017	0	0	0
2014302946	818	rural/clinic	No	04/07/2017	0	0	0
2015367251	818	rural/clinic	No	04/07/2017	0	0	0
2015363760	818	rural/clinic	No	04/07/2017	0	0	0
2015303093	818	rural/clinic	No	04/07/2017	0	0	0
2015333855	818	rural/clinic	No	04/07/2017	0	0	0
2013264394	818	rural/clinic	No	04/07/2017	0	0	0
2015352768	818	rural/clinic	No	04/07/2017	0	0	0
2015413216	818	rural/clinic	No	04/07/2017	0	0	0
2015352769	818	rural/clinic	No	04/07/2017	0	0	0
2013256274	818	rural/clinic	No	04/07/2017	0	0	0
2015352770	670	rural/clinic	No	04/07/2017	0	0	0
2013256275	670	rural/clinic	No	04/07/2017	0	0	0
2015352771	670	rural/clinic	No	04/07/2017	0	0	0
2015368600	670	rural/clinic	No	04/07/2017	0	0	0
2014306038	670	rural/clinic	No	04/07/2017	0	0	0
2015368601	670	rural/clinic	No	04/07/2017	0	0	0
2014306039	670	rural/clinic	No	04/07/2017	0	0	0
2015340221	670	rural/clinic	No	04/07/2017	0	0	0
2014306040	670	rural/clinic	No	04/07/2017	0	0	0
2014306041	670	rural/clinic	No	04/07/2017	0	0	0
2010099928	670	rural/clinic	No	04/07/2017	0	0	0
2015362532	670	rural/clinic	No	04/07/2017	0	0	0
2011200279	670	rural/clinic	No	04/07/2017	0	0	0
201066587	670	rural/clinic	No	04/07/2017	0	0	0
2015337517	670	rural/clinic	No	04/07/2017	0	0	0
2015337518	670	rural/clinic	No	04/07/2017	0	0	0
2015362533	670	rural/clinic	No	04/07/2017	0	0	0
2015384787	670	rural/clinic	No	04/07/2017	0	0	0
2012358294	670	rural/clinic	No	04/07/2017	0	0	0
2015368994	670	rural/clinic	No	04/07/2017	0	0	0
2015324042	670	rural/clinic	No	04/07/2017	0	0	0
2015368995	670	rural/clinic	No	04/07/2017	0	0	0
2015324043	670	rural/clinic	No	04/07/2017	0	0	0
2012358295	670	rural/clinic	No	04/07/2017	0	0	0
2015368996	670	rural/clinic	No	04/07/2017	0	0	0
2015368997	670	rural/clinic	No	04/07/2017	0	0	0

2015337515	670	rural/clinic	No	04/07/2017	0	0	0
2015368988	670	rural/clinic	No	04/07/2017	0	0	0
2015297347	670	rural/clinic	No	04/07/2017	0	0	0
2014289545	670	rural/clinic	No	04/07/2017	0	0	0
2014289546	670	rural/clinic	No	04/07/2017	0	0	0
2015342315	670	rural/clinic	No	04/07/2017	0	0	0
2015297348	670	rural/clinic	No	04/07/2017	0	0	0
2015390316	670	rural/clinic	No	04/07/2017	0	0	0
2015409023	670	rural/clinic	No	04/07/2017	0	0	0
2015415284	670	rural/clinic	No	04/07/2017	0	0	0
2014318609	670	rural/clinic	No	04/07/2017	0	0	0
2015409024	670	rural/clinic	No	04/07/2017	0	0	0
2015415285	670	rural/clinic	No	04/07/2017	0	0	0
2015325209	928	rural/clinic	No	04/07/2017	0	0	0
2015325938	928	rural/clinic	No	04/07/2017	0	0	0
2015362430	928	rural/clinic	No	04/07/2017	0	0	0
2013271847	928	rural/clinic	No	04/07/2017	0	0	0
2015337994	928	rural/clinic	No	04/07/2017	0	0	0
2015362429	928	rural/clinic	No	04/07/2017	0	0	0
2010099929	928	rural/clinic	No	04/07/2017	0	0	0
2014370439	928	rural/clinic	No	04/07/2017	0	0	0
2015301495	928	rural/clinic	No	04/07/2017	0	0	0
2015363458	928	rural/clinic	No	04/07/2017	0	0	0
2015360735	928	rural/clinic	No	04/07/2017	0	0	0
2015301494	928	rural/clinic	No	04/07/2017	0	0	0
2014309390	928	rural/clinic	No	04/07/2017	0	0	0
2012306344	928	rural/clinic	No	04/07/2017	0	0	0
2015301496	928	rural/clinic	No	04/07/2017	0	0	0
2015368724	928	rural/clinic	No	04/07/2017	0	0	0
2015301497	928	rural/clinic	No	04/07/2017	0	0	0
2011142497	928	rural/clinic	No	04/07/2017	0	0	0
2015301489	928	rural/clinic	No	04/07/2017	0	0	0
2015368723	928	rural/clinic	No	04/07/2017	0	0	0
2015301498	928	rural/clinic	No	04/07/2017	0	0	0
2015289190	928	rural/clinic	No	04/07/2017	0	0	0
2015301500	509	rural/clinic	No	04/07/2017	0	0	0
2011136141	509	rural/clinic	No	04/07/2017	0	0	0
2015373863	509	rural/clinic	No	04/07/2017	0	0	0
2015301499	509	rural/clinic	No	04/07/2017	0	0	0
2011136140	509	rural/clinic	No	04/07/2017	0	0	0
2015419051	509	rural/clinic	No	04/07/2017	0	0	0
2014369197	509	rural/clinic	No	04/07/2017	0	0	0
2015373864	509	rural/clinic	No	04/07/2017	0	0	0
2011136138	509	rural/clinic	No	04/07/2017	0	0	0
2012314025	509	rural/clinic	No	04/07/2017	0	0	0
2015326430	509	rural/clinic	No	04/07/2017	0	0	0
2011136139	509	rural/clinic	No	04/07/2017	0	0	0
2015372812	509	rural/clinic	No	04/07/2017	0	0	0
2015326431	509	rural/clinic	No	04/07/2017	0	0	0
2014317438	929	rural/clinic	No	04/07/2017	0	0	0
2011136142	929	rural/clinic	No	04/07/2017	0	0	0
2014317639	929	rural/clinic	No	04/07/2017	0	0	0
2014317640	929	rural/clinic	No	04/07/2017	0	0	0
201053912	97	rural/clinic	No	04/07/2017	0	0	0
2015417074	97	rural/clinic	No	04/07/2017	0	0	0
201053911	97	rural/clinic	No	04/07/2017	0	0	0
2015417073	97	rural/clinic	No	04/07/2017	0	0	0
2015417072	97	rural/clinic	No	04/07/2017	0	0	0
201053913	97	rural/clinic	No	04/07/2017	0	0	0
2014317436	97	rural/clinic	No	04/07/2017	0	0	0
2012289895	97	rural/clinic	No	04/07/2017	0	0	0
2012314024	97	rural/clinic	No	04/07/2017	0	0	0
2015417075	97	rural/clinic	No	04/07/2017	0	0	0
2014290326	97	rural/clinic	No	04/07/2017	0	0	0
2014348244	97	rural/clinic	No	04/07/2017	0	0	0
2014383707	97	rural/clinic	No	04/07/2017	0	0	0
2012305817	97	rural/clinic	No	04/07/2017	0	0	0
2012305818	97	rural/clinic	No	04/07/2017	0	0	0
2012305819	97	rural/clinic	No	04/07/2017	0	0	0
2012263921	97	rural/clinic	No	04/07/2017	0	0	0
2015342294	97	rural/clinic	No	04/07/2017	0	0	0
2013266248	97	rural/clinic	No	04/07/2017	0	0	0
2015326432	97	rural/clinic	No	04/07/2017	0	0	0
2015326433	334	rural/clinic	No	04/07/2017	0	0	0
2015326434	334	rural/clinic	No	04/07/2017	0	0	0
2015372811	334	rural/clinic	No	04/07/2017	0	0	0
2013266246	334	rural/clinic	No	04/07/2017	0	0	0
2015372810	334	rural/clinic	No	04/07/2017	0	0	0
2013266245	334	rural/clinic	No	04/07/2017	0	0	0
2013266247	334	rural/clinic	No	04/07/2017	0	0	0
2015315914	334	rural/clinic	No	04/07/2017	0	0	0
2012261386	334	rural/clinic	No	04/07/2017	0	0	0

2015417076	334	rural/clinic	No	04/07/2017	0	0	0
2015417077	334	rural/clinic	No	04/07/2017	0	0	0
2014291819	334	rural/clinic	No	26/06/2017	0	0	0
2011235685	334	rural/clinic	No	04/07/2017	0	0	0
2015382942	334	rural/clinic	No	04/07/2017	0	0	0
2015384814	334	rural/clinic	No	22/06/2017	0	0	0
2015382943	334	rural/clinic	No	04/07/2017	0	0	0
2015414262	334	rural/clinic	No	04/07/2017	0	0	0
2014369099	334	rural/clinic	No	04/07/2017	0	0	0
2015382944	334	rural/clinic	No	04/07/2017	0	0	0
2014374033	334	rural/clinic	No	04/07/2017	0	0	0
2015325182	309	rural/clinic	No	04/07/2017	0	0	0
2014374030	309	rural/clinic	No	04/07/2017	0	0	0
2015382945	309	rural/clinic	No	04/07/2017	0	0	0
2014383705	309	rural/clinic	No	20/06/2017	0	0	0
2014327518	309	rural/clinic	No	20/06/2017	0	0	0
2014374031	309	rural/clinic	No	04/07/2017	0	0	0
2015325202	309	rural/clinic	No	01/06/2017	0	0	0
2015382946	309	rural/clinic	No	04/07/2017	0	0	0
2014382924	309	rural/clinic	No	04/07/2017	0	0	0
2014374029	309	rural/clinic	No	04/07/2017	0	0	0
2014374032	309	rural/clinic	No	04/07/2017	0	0	0
2012259898	309	rural/clinic	No	04/07/2017	0	0	0
2015418374	309	rural/clinic	No	04/07/2017	0	0	0
2014374028	309	rural/clinic	No	04/07/2017	0	0	0
2014371590	309	rural/clinic	No	04/07/2017	0	0	0
2015355523	770	rural/clinic	No	04/07/2017	0	0	0
2014291624	770	rural/clinic	No	04/07/2017	0	0	0
2015324450	770	rural/clinic	No	04/07/2017	0	0	0
2015385856	770	rural/clinic	No	04/07/2017	0	0	0
2015324041	770	rural/clinic	No	04/07/2017	0	0	0
2015324040	770	rural/clinic	No	04/07/2017	0	0	0
2012344858	770	rural/clinic	No	04/07/2017	0	0	0
2015337372	770	rural/clinic	No	04/07/2017	0	0	0
2015385855	770	rural/clinic	No	04/07/2017	0	0	0
2015337516	770	rural/clinic	No	04/07/2017	0	0	0
2015337308	770	rural/clinic	No	04/07/2017	0	0	0
2014371589	770	rural/clinic	No	04/07/2017	0	0	0
2014371588	770	rural/clinic	No	04/07/2017	0	0	0
2015372931	770	rural/clinic	No	04/07/2017	0	0	0
2015372932	770	rural/clinic	No	04/07/2017	0	0	0
2015372933	770	rural/clinic	No	04/07/2017	0	0	0
2015372934	770	rural/clinic	No	04/07/2017	0	0	0
2014328450	770	rural/clinic	No	04/07/2017	0	0	0
2014382302	770	rural/clinic	No	04/07/2017	0	0	0
2015412325	770	rural/clinic	No	04/07/2017	0	0	0
2012269021	770	rural/clinic	No	04/07/2017	0	0	0
2012269019	770	rural/clinic	No	04/07/2017	0	0	0
2014382923	770	rural/clinic	No	04/07/2017	0	0	0
2015412324	770	rural/clinic	No	04/07/2017	0	0	0
2015412323	770	rural/clinic	No	04/07/2017	0	0	0
2015412322	770	rural/clinic	No	04/07/2017	0	0	0
2015412321	770	rural/clinic	No	04/07/2017	0	0	0
2014328449	770	rural/clinic	No	04/07/2017	0	0	0
2012269018	770	rural/clinic	No	04/07/2017	0	0	0
2012269022	770	rural/clinic	No	04/07/2017	0	0	0
2015297346	770	rural/clinic	No	04/07/2017	0	0	0
2015294623	770	rural/clinic	No	04/07/2017	0	0	0
2015294623	770	rural/clinic	No	04/07/2017	0	0	0
2015344100	770	rural/clinic	No	04/07/2017	0	0	0
2015344100	770	rural/clinic	No	04/07/2017	0	0	0
2015344662	770	rural/clinic	No	04/07/2017	0	0	0
2015324572	770	rural/clinic	No	04/07/2017	0	0	0
2015324572	770	rural/clinic	No	04/07/2017	0	0	0
2015373864	770	rural/clinic	No	04/07/2017	0	0	0
2015373864	770	rural/clinic	No	04/07/2017	0	0	0
2015337516	770	rural/clinic	No	04/07/2017	0	0	0
2014357824	770	rural/clinic	No	04/07/2017	0	0	0
2014357824	770	rural/clinic	No	04/07/2017	0	0	0
2014358023	770	rural/clinic	No	04/07/2017	0	0	0
2014358023	770	rural/clinic	No	04/07/2017	0	0	0
2014318609	770	rural/clinic	No	04/07/2017	0	0	0
2014318609	770	rural/clinic	No	04/07/2017	0	0	0
2015414260	770	rural/clinic	No	04/07/2017	0	0	0
2015414260	770	rural/clinic	No	04/07/2017	0	0	0
2015414263	770	rural/clinic	No	04/07/2017	0	0	0
2015414263	770	rural/clinic	No	04/07/2017	0	0	0
2015303093	770	rural/clinic	No	04/07/2017	0	0	0
2015303093	770	rural/clinic	No	04/07/2017	0	0	0
2012294475	726	rural/clinic	No	06/07/2017	0	0	0
2012294477	726	rural/clinic	No	06/07/2017	0	0	0
2015368299	726	rural/clinic	No	06/07/2017	0	0	0

2015332475	726	rural/clinic	No	06/07/2017	0	0	0
2015332474	726	rural/clinic	No	06/07/2017	0	0	0
2015368298	726	rural/clinic	No	06/07/2017	0	0	0
2015368297	726	rural/clinic	No	06/07/2017	0	0	0
2014312410	726	rural/clinic	No	06/07/2017	0	0	0
2010099932	482	rural/clinic	No	06/07/2017	0	0	0
2015368296	482	rural/clinic	No	06/07/2017	0	0	0
2015368300	482	rural/clinic	No	06/07/2017	0	0	0
2015382556	482	rural/clinic	No	06/07/2017	0	0	0
2015382555	482	rural/clinic	No	06/07/2017	0	0	0
2013258610	482	rural/clinic	No	06/07/2017	0	0	0
2015332471	482	rural/clinic	No	06/07/2017	0	0	0
2015332472	482	rural/clinic	No	06/07/2017	0	0	0
2015382558	482	rural/clinic	No	06/07/2017	0	0	0
2012275201	482	rural/clinic	No	06/07/2017	0	0	0
2015382559	482	rural/clinic	No	06/07/2017	0	0	0
2015332473	482	rural/clinic	No	06/07/2017	0	0	0
2015382560	727	rural/clinic	No	06/07/2017	0	0	0
2011205724	727	rural/clinic	No	06/07/2017	0	0	0
2011205725	727	rural/clinic	No	06/07/2017	0	0	0
2015384170	727	rural/clinic	No	06/07/2017	0	0	0
2015303370	727	rural/clinic	No	06/07/2017	0	0	0
2012275203	727	rural/clinic	No	06/07/2017	0	0	0
2015335205	727	rural/clinic	No	06/07/2017	0	0	0
2012275202	727	rural/clinic	No	06/07/2017	0	0	0
2012317563	727	rural/clinic	No	06/07/2017	0	0	0
2014297349	727	rural/clinic	No	06/07/2017	0	0	0
2012317562	727	rural/clinic	No	06/07/2017	0	0	0
2015303371	727	rural/clinic	No	06/07/2017	0	0	0
2015373749	727	rural/clinic	No	06/07/2017	0	0	0
2014301896	727	rural/clinic	No	06/07/2017	0	0	0
2015373747	727	rural/clinic	No	06/07/2017	0	0	0
2015369003	727	rural/clinic	No	06/07/2017	0	0	0
2015369071	727	rural/clinic	No	06/07/2017	0	0	0
2015373745	727	rural/clinic	No	06/07/2017	0	0	0
2015369004	727	rural/clinic	No	06/07/2017	0	0	0
2015369005	727	rural/clinic	No	06/07/2017	0	0	0
2015373746	727	rural/clinic	No	06/07/2017	0	0	0
2015373744	727	rural/clinic	No	06/07/2017	0	0	0
2015369073	727	rural/clinic	No	06/07/2017	0	0	0
2015373743	727	rural/clinic	No	06/07/2017	0	0	0
2015369075	727	rural/clinic	No	06/07/2017	0	0	0
2015373742	727	rural/clinic	No	06/07/2017	0	0	0
2015369006	727	rural/clinic	No	06/07/2017	0	0	0
2015373741	727	rural/clinic	No	06/07/2017	0	0	0
2015373740	727	rural/clinic	No	06/07/2017	0	0	0
2015338758	727	rural/clinic	No	06/07/2017	0	0	0
2015369070	727	rural/clinic	No	06/07/2017	0	0	0
2015373865	727	rural/clinic	No	06/07/2017	0	0	0
2015369072	727	rural/clinic	No	06/07/2017	0	0	0
2015359616	727	rural/clinic	No	06/07/2017	0	0	0
2015340362	727	rural/clinic	No	06/07/2017	0	0	0
2012284487	798	rural/clinic	No	06/07/2017	0	0	0
2015340361	798	rural/clinic	No	06/07/2017	0	0	0
2015382553	798	rural/clinic	No	06/07/2017	0	0	0
2015359615	798	rural/clinic	No	06/07/2017	0	0	0
2015297278	798	rural/clinic	No	06/07/2017	0	0	0
2015384360	798	rural/clinic	No	06/07/2017	0	0	0
2015382554	798	rural/clinic	No	06/07/2017	0	0	0
2015297277	798	rural/clinic	No	06/07/2017	0	0	0
2015377361	798	rural/clinic	No	06/07/2017	0	0	0
2011234899	798	rural/clinic	No	06/07/2017	0	0	0
2011143949	798	rural/clinic	No	06/07/2017	0	0	0
2015418710	771	rural/clinic	No	06/07/2017	0	0	0
2011143948	771	rural/clinic	No	06/07/2017	0	0	0
2014362835	771	rural/clinic	No	06/07/2017	0	0	0
2015408178	771	rural/clinic	No	06/07/2017	0	0	0
2014319094	771	rural/clinic	No	06/07/2017	0	0	0
2014325209	771	rural/clinic	No	06/07/2017	0	0	0
2014383983	771	rural/clinic	No	06/07/2017	0	0	0
2011199246	771	rural/clinic	No	06/07/2017	0	0	0
2014378494	771	rural/clinic	No	06/07/2017	0	0	0
2014378493	771	rural/clinic	No	06/07/2017	0	0	0
2015332914	771	rural/clinic	No	06/07/2017	0	0	0
2014378492	771	rural/clinic	No	06/07/2017	0	0	0
2015361167	771	rural/clinic	No	06/07/2017	0	0	0
2015361166	771	rural/clinic	No	06/07/2017	0	0	0
2014378558	771	rural/clinic	No	06/07/2017	0	0	0
2015301505	771	rural/clinic	No	06/07/2017	0	0	0
2015361164	174	rural/clinic	No	06/07/2017	0	0	0
2014372339	174	rural/clinic	No	06/07/2017	0	0	0
2015361163	174	rural/clinic	No	06/07/2017	0	0	0

2011229222	174	rural/clinic	No	06/07/2017	0	0	0
2015361165	174	rural/clinic	No	06/07/2017	0	0	0
2015361162	174	rural/clinic	No	06/07/2017	0	0	0
2015349538	174	rural/clinic	No	06/07/2017	0	0	0
2015319288	174	rural/clinic	No	06/07/2017	0	0	0
2015361161	174	rural/clinic	No	06/07/2017	0	0	0
2011216825	174	rural/clinic	No	06/07/2017	0	0	0
2015319289	120	rural/clinic	No	06/07/2017	0	0	0
2015415660	120	rural/clinic	No	06/07/2017	0	0	0
2015319290	120	rural/clinic	No	06/07/2017	0	0	0
2015415662	120	rural/clinic	No	06/07/2017	0	0	0
2015364307	120	rural/clinic	No	06/07/2017	0	0	0
2011199094	120	rural/clinic	No	06/07/2017	0	0	0
2015415663	120	rural/clinic	No	06/07/2017	0	0	0
2015364308	120	rural/clinic	No	06/07/2017	0	0	0
2014327089	120	rural/clinic	No	06/07/2017	0	0	0
2015415664	120	rural/clinic	No	06/07/2017	0	0	0
2015385538	120	rural/clinic	No	06/07/2017	0	0	0
2015352742	120	rural/clinic	No	06/07/2017	0	0	0
2015385539	120	rural/clinic	No	06/07/2017	0	0	0
2011199095	120	rural/clinic	No	06/07/2017	0	0	0
2014383982	120	rural/clinic	No	06/07/2017	0	0	0
2015385537	120	rural/clinic	No	06/07/2017	0	0	0
2015301272	120	rural/clinic	No	06/07/2017	0	0	0
2015364305	120	rural/clinic	No	06/07/2017	0	0	0
2014319092	120	rural/clinic	No	06/07/2017	0	0	0
2015364306	120	rural/clinic	No	06/07/2017	0	0	0
2014319093	120	rural/clinic	No	06/07/2017	0	0	0
2015340222	120	rural/clinic	No	06/07/2017	0	0	0
2012306432	120	rural/clinic	No	06/07/2017	0	0	0
2015386737	120	rural/clinic	No	06/07/2017	0	0	0
2010099931	120	rural/clinic	No	06/07/2017	0	0	0
2015372027	120	rural/clinic	No	06/07/2017	0	0	0
2010099930	121	rural/clinic	No	06/07/2017	0	0	0
2015384302	121	rural/clinic	No	06/07/2017	0	0	0
2015386739	121	rural/clinic	No	06/07/2017	0	0	0
2011155235	121	rural/clinic	No	06/07/2017	0	0	0
2015386735	121	rural/clinic	No	06/07/2017	0	0	0
2015384050	121	rural/clinic	No	06/07/2017	0	0	0
2015401154	121	rural/clinic	No	06/07/2017	0	0	0
2015386738	121	rural/clinic	No	06/07/2017	0	0	0
2015384048	121	rural/clinic	No	06/07/2017	0	0	0
2015386736	121	rural/clinic	No	06/07/2017	0	0	0
2015401155	121	rural/clinic	No	06/07/2017	0	0	0
2015384049	121	rural/clinic	No	06/07/2017	0	0	0
2015401156	121	rural/clinic	No	06/07/2017	0	0	0
2015369199	121	rural/clinic	No	06/07/2017	0	0	0
2015364309	121	rural/clinic	No	06/07/2017	0	0	0
2015362282	121	rural/clinic	No	06/07/2017	0	0	0
2015364310	121	rural/clinic	No	06/07/2017	0	0	0
2015369198	121	rural/clinic	No	06/07/2017	0	0	0
2015369200	121	rural/clinic	No	06/07/2017	0	0	0
2015364311	121	rural/clinic	No	06/07/2017	0	0	0
2011198648	121	rural/clinic	No	06/07/2017	0	0	0
2014382447	121	rural/clinic	No	06/07/2017	0	0	0
2014314227	121	rural/clinic	No	06/07/2017	0	0	0
2014382448	121	rural/clinic	No	06/07/2017	0	0	0
2014314226	122	rural/clinic	No	06/07/2017	0	0	0
2014320529	41	rural/clinic	No	06/07/2017	0	0	0
2015412506	41	rural/clinic	No	06/07/2017	0	0	0
2012265640	41	rural/clinic	No	06/07/2017	0	0	0
2012377419	41	rural/clinic	No	06/07/2017	0	0	0
2011200079	41	rural/clinic	No	06/07/2017	0	0	0
2012377418	41	rural/clinic	No	06/07/2017	0	0	0
2011192937	41	rural/clinic	No	06/07/2017	0	0	0
2015340225	41	rural/clinic	No	06/07/2017	0	0	0
2015340144	41	rural/clinic	No	06/07/2017	0	0	0
2015384169	41	rural/clinic	No	06/07/2017	0	0	0
2011193492	41	rural/clinic	No	06/07/2017	0	0	0
2015340223	41	rural/clinic	No	06/07/2017	0	0	0
2015340145	41	rural/clinic	No	06/07/2017	0	0	0
2015397814	41	rural/clinic	No	06/07/2017	0	0	0
2015340146	41	rural/clinic	No	06/07/2017	0	0	0
2011193493	41	rural/clinic	No	06/07/2017	0	0	0
2015340224	41	rural/clinic	No	06/07/2017	0	0	0
2015340147	41	rural/clinic	No	06/07/2017	0	0	0
2014315711	41	rural/clinic	No	06/07/2017	0	0	0
2015326600	41	rural/clinic	No	06/07/2017	0	0	0
2015345072	41	rural/clinic	No	06/07/2017	0	0	0
2015288583	41	rural/clinic	No	06/07/2017	0	0	0
2015307406	41	rural/clinic	No	06/07/2017	0	0	0
2015288607	41	rural/clinic	No	06/07/2017	0	0	0

2015319285	41	rural/clinic	No	06/07/2017	0	0	0
2015319286	41	rural/clinic	No	06/07/2017	0	0	0
2015319287	41	rural/clinic	No	06/07/2017	0	0	0
2011168522	41	rural/clinic	No	06/07/2017	0	0	0
2015328083	41	rural/clinic	No	06/07/2017	0	0	0
2015328080	41	rural/clinic	No	06/07/2017	0	0	0
2014339747	41	rural/clinic	No	06/07/2017	0	0	0
2015340148	41	rural/clinic	No	06/07/2017	0	0	0
2015362062	41	rural/clinic	No	06/07/2017	0	0	0
2011143621	41	rural/clinic	No	06/07/2017	0	0	0
2015328079	41	rural/clinic	No	06/07/2017	0	0	0
2014319726	41	rural/clinic	No	06/07/2017	0	0	0
2015286313	41	rural/clinic	No	06/07/2017	0	0	0
2014338561	41	rural/clinic	No	06/07/2017	0	0	0
2015328081	41	rural/clinic	No	06/07/2017	0	0	0
2015328078	41	rural/clinic	No	06/07/2017	0	0	0
2015378860	41	rural/clinic	No	06/07/2017	0	0	0
2015328077	41	rural/clinic	No	06/07/2017	0	0	0
2015286329	41	rural/clinic	No	06/07/2017	0	0	0
2011190504	41	rural/clinic	No	06/07/2017	0	0	0
2015286328	41	rural/clinic	No	06/07/2017	0	0	0
2015328082	41	rural/clinic	No	06/07/2017	0	0	0
2015286327	41	rural/clinic	No	06/07/2017	0	0	0
2015408682	41	rural/clinic	No	06/07/2017	0	0	0
2014332706	41	rural/clinic	No	06/07/2017	0	0	0
2011190503	41	rural/clinic	No	06/07/2017	0	0	0
2014332707	41	rural/clinic	No	06/07/2017	0	0	0
2014332708	41	rural/clinic	No	06/07/2017	0	0	0
2011213940	41	rural/clinic	No	06/07/2017	0	0	0
2014332709	41	rural/clinic	No	06/07/2017	0	0	0
2014332710	41	rural/clinic	No	06/07/2017	0	0	0
2014332711	41	rural/clinic	No	06/07/2017	0	0	0
2012377420	41	rural/clinic	No	06/07/2017	0	0	0
2015288605	41	rural/clinic	No	06/07/2017	0	0	0
2015288606	41	rural/clinic	No	06/07/2017	0	0	0
2012377417	41	rural/clinic	No	06/07/2017	0	0	0
2014305226	41	rural/clinic	No	06/07/2017	0	0	0
2014382446	41	rural/clinic	No	06/07/2017	0	0	0
2015339026	41	rural/clinic	No	06/07/2017	0	0	0
2015355015	41	rural/clinic	No	06/07/2017	0	0	0
2014302527	41	rural/clinic	No	06/07/2017	0	0	0
2015355014	41	rural/clinic	No	06/07/2017	0	0	0
2015318773	41	rural/clinic	No	06/07/2017	0	0	0
2012344505	41	rural/clinic	No	06/07/2017	0	0	0
2012301000	41	rural/clinic	No	06/07/2017	0	0	0
2011214896	41	rural/clinic	No	06/07/2017	0	0	0
2011188727	41	rural/clinic	No	06/07/2017	0	0	0
2011126923	41	rural/clinic	No	06/07/2017	0	0	0
2011126925	41	rural/clinic	No	06/07/2017	0	0	0
2015318772	41	rural/clinic	No	06/07/2017	0	0	0
2012300999	41	rural/clinic	No	06/07/2017	0	0	0
2015355921	41	rural/clinic	No	06/07/2017	0	0	0
2011126924	41	rural/clinic	No	06/07/2017	0	0	0
2015355920	41	rural/clinic	No	06/07/2017	0	0	0
2011188480	41	rural/clinic	No	06/07/2017	0	0	0
2015406007	41	rural/clinic	No	06/07/2017	0	0	0
2015330506	41	rural/clinic	No	06/07/2017	0	0	0
2015406006	41	rural/clinic	No	06/07/2017	0	0	0
2015406005	41	rural/clinic	No	06/07/2017	0	0	0
2015330505	41	rural/clinic	No	06/07/2017	0	0	0
2015386284	41	rural/clinic	No	06/07/2017	0	0	0
2015330504	41	rural/clinic	No	06/07/2017	0	0	0
2011188726	41	rural/clinic	No	06/07/2017	0	0	0
2015299485	41	rural/clinic	No	06/07/2017	0	0	0
2015299290	41	rural/clinic	No	06/07/2017	0	0	0
2011214937	41	rural/clinic	No	06/07/2017	0	0	0
2011215769	41	rural/clinic	No	06/07/2017	0	0	0
2015386283	41	rural/clinic	No	06/07/2017	0	0	0
2015403201	41	rural/clinic	No	06/07/2017	0	0	0
2015372813	41	rural/clinic	No	06/07/2017	0	0	0
2015386285	41	rural/clinic	No	06/07/2017	0	0	0
2015372814	41	rural/clinic	No	06/07/2017	0	0	0
2015386282	41	rural/clinic	No	06/07/2017	0	0	0
2015303642	41	rural/clinic	No	06/07/2017	0	0	0
2015303643	41	rural/clinic	No	06/07/2017	0	0	0
2015386288	41	rural/clinic	No	06/07/2017	0	0	0
2015303646	41	rural/clinic	No	06/07/2017	0	0	0
2015303645	41	rural/clinic	No	06/07/2017	0	0	0
2015386289	41	rural/clinic	No	06/07/2017	0	0	0
2014344471	41	rural/clinic	No	06/07/2017	0	0	0
2015386286	41	rural/clinic	No	06/07/2017	0	0	0
2014344470	41	rural/clinic	No	06/07/2017	0	0	0

2014344469	41	rural/clinic	No	06/07/2017	0	0	0
2015386287	41	rural/clinic	No	06/07/2017	0	0	0
2015334658	41	rural/clinic	No	06/07/2017	0	0	0
2014368026	41	rural/clinic	No	06/07/2017	0	0	0
2015386279	41	rural/clinic	No	06/07/2017	0	0	0
2015386280	41	rural/clinic	No	06/07/2017	0	0	0
2014327090	41	rural/clinic	No	06/07/2017	0	0	0
2014368025	41	rural/clinic	No	06/07/2017	0	0	0
2015386281	41	rural/clinic	No	06/07/2017	0	0	0
2015325049	41	rural/clinic	No	06/07/2017	0	0	0
2015419053	41	rural/clinic	No	06/07/2017	0	0	0
2015386274	41	rural/clinic	No	06/07/2017	0	0	0
2011225415	41	rural/clinic	No	06/07/2017	0	0	0
2015386278	41	rural/clinic	No	06/07/2017	0	0	0
2015325050	41	rural/clinic	No	06/07/2017	0	0	0
2015386277	41	rural/clinic	No	06/07/2017	0	0	0
2015338869	41	rural/clinic	No	06/07/2017	0	0	0
2011192938	41	rural/clinic	No	06/07/2017	0	0	0
2015386276	41	rural/clinic	No	06/07/2017	0	0	0
2011223940	41	rural/clinic	No	06/07/2017	0	0	0
2015303644	41	rural/clinic	No	06/07/2017	0	0	0
2015386275	41	rural/clinic	No	06/07/2017	0	0	0
2015377329	41	rural/clinic	No	06/07/2017	0	0	0
2015384362	41	rural/clinic	No	06/07/2017	0	0	0
2015338868	41	rural/clinic	No	06/07/2017	0	0	0
2015369779	41	rural/clinic	No	06/07/2017	0	0	0
2015369778	41	rural/clinic	No	06/07/2017	0	0	0
2015369780	41	rural/clinic	No	06/07/2017	0	0	0
2015369777	41	rural/clinic	No	06/07/2017	0	0	0
2015369776	41	rural/clinic	No	06/07/2017	0	0	0
2015345071	41	rural/clinic	No	06/07/2017	0	0	0
2015345070	41	rural/clinic	No	06/07/2017	0	0	0
2015340143	41	rural/clinic	No	06/07/2017	0	0	0
2015345074	41	rural/clinic	No	06/07/2017	0	0	0
2015345073	41	rural/clinic	No	06/07/2017	0	0	0
2014305227	41	rural/clinic	No	06/07/2017	0	0	0
2014371712	41	rural/clinic	No	06/07/2017	0	0	0
2015369746	41	rural/clinic	No	12/06/2017	0	0	0
2011157149	41	rural/clinic	No	06/06/2017	0	0	0
2015352772	41	rural/clinic	No	06/07/2017	0	0	0
2015352774	41	rural/clinic	No	06/07/2017	0	0	0
2015352773	41	rural/clinic	No	06/07/2017	0	0	0
2015357807	41	rural/clinic	No	06/07/2017	0	0	0
2015357808	41	rural/clinic	No	06/07/2017	0	0	0
2011191293	41	rural/clinic	No	06/07/2017	0	0	0
2011191294	41	rural/clinic	No	06/07/2017	0	0	0
2014320530	41	rural/clinic	No	06/07/2017	0	0	0
2014374034	41	rural/clinic	No	06/07/2017	0	0	0
2014338560	41	rural/clinic	No	06/07/2017	0	0	0
2014306022	41	rural/clinic	No	06/06/2017	0	0	0
2015287383	41	rural/clinic	No	04/07/2017	0	0	0
2015287383	41	rural/clinic	No	04/07/2017	0	0	0
2015352769	41	rural/clinic	No	04/07/2017	0	0	0
2015352769	41	rural/clinic	No	04/07/2017	0	0	0
2015417075	41	rural/clinic	No	04/07/2017	0	0	0
2015417075	41	rural/clinic	No	04/07/2017	0	0	0
2012305819	41	rural/clinic	No	04/07/2017	0	0	0
2012305819	41	rural/clinic	No	04/07/2017	0	0	0
2014371589	41	rural/clinic	No	04/07/2017	0	0	0
2012269021	41	rural/clinic	No	04/07/2017	0	0	0
2015412323	41	rural/clinic	No	04/07/2017	0	0	0
2015332914	41	rural/clinic	No	06/07/2017	0	0	0
2014372339	41	rural/clinic	No	06/07/2017	0	0	0
2014372339	41	rural/clinic	No	06/07/2017	0	0	0
2015340146	41	rural/clinic	No	06/07/2017	0	0	0
2015415664	41	rural/clinic	No	06/07/2017	0	0	0
2015415664	41	rural/clinic	No	06/07/2017	0	0	0
2015385539	41	rural/clinic	No	06/07/2017	0	0	0
2015385539	41	rural/clinic	No	06/07/2017	0	0	0
2015364311	41	rural/clinic	No	06/07/2017	0	0	0
2015364311	41	rural/clinic	No	06/07/2017	0	0	0
2015344099	41	rural/clinic	No	04/07/2017	0	0	0
2014303705	41	rural/clinic	No	11/07/2017	0	0	0
2015363075	41	rural/clinic	No	11/07/2017	0	0	0
2015363074	41	rural/clinic	No	11/07/2017	0	0	0
2012259899	41	rural/clinic	No	11/07/2017	0	0	0
2012369031	41	rural/clinic	No	11/07/2017	0	0	0
2012259900	41	rural/clinic	No	11/07/2017	0	0	0
2015362284	41	rural/clinic	No	11/07/2017	0	0	0
2015409025	41	rural/clinic	No	11/07/2017	0	0	0
2014365393	41	rural/clinic	No	11/07/2017	0	0	0
2015362285	41	rural/clinic	No	11/07/2017	0	0	0

2015362286	41	rural/clinic	No	11/07/2017	0	0	0
2014365394	41	rural/clinic	No	11/07/2017	0	0	0
2015362287	41	rural/clinic	No	11/07/2017	0	0	0
2012369028	41	rural/clinic	No	11/07/2017	0	0	0
2012369030	41	rural/clinic	No	11/07/2017	0	0	0
2015362288	41	rural/clinic	No	11/07/2017	0	0	0
2015362289	41	rural/clinic	No	11/07/2017	0	0	0
2014338315	41	rural/clinic	No	11/07/2017	0	0	0
2012369029	41	rural/clinic	No	11/07/2017	0	0	0
2015405415	41	rural/clinic	No	11/07/2017	0	0	0
2015362290	41	rural/clinic	No	11/07/2017	0	0	0
2015405414	41	rural/clinic	No	11/07/2017	0	0	0
2014350685	41	rural/clinic	No	11/07/2017	0	0	0
2015362291	41	rural/clinic	No	11/07/2017	0	0	0
2014350683	41	rural/clinic	No	11/07/2017	0	0	0
2013264072	41	rural/clinic	No	11/07/2017	0	0	0
2014350684	41	rural/clinic	No	11/07/2017	0	0	0
2014383532	41	rural/clinic	No	11/07/2017	0	0	0
2012293007	41	rural/clinic	No	27/06/2017	0	0	0
2014358018	41	rural/clinic	No	27/06/2017	0	0	0
2014363194	41	rural/clinic	No	11/07/2017	0	0	0
2015418758	41	rural/clinic	No	11/07/2017	0	0	0
2012363970	41	rural/clinic	No	27/06/2017	0	0	0
2014350632	41	rural/clinic	No	11/07/2017	0	0	0
2015375212	41	rural/clinic	No	11/07/2017	0	0	0
2014358220	41	rural/clinic	No	11/07/2017	0	0	0
2014350636	41	rural/clinic	No	11/07/2017	0	0	0
2014344366	41	rural/clinic	No	11/07/2017	0	0	0
2014350635	41	rural/clinic	No	11/07/2017	0	0	0
2012388398	41	rural/clinic	No	11/07/2017	0	0	0
2012244289	41	rural/clinic	No	11/07/2017	0	0	0
2015324045	41	rural/clinic	No	11/07/2017	0	0	0
2012271401	41	rural/clinic	No	11/07/2017	0	0	0
2012253975	41	rural/clinic	No	11/07/2017	0	0	0
2011135951	41	rural/clinic	No	11/07/2017	0	0	0
2014350634/D	41	rural/clinic	No	11/07/2017	0	0	0
2015375413	41	rural/clinic	No	11/07/2017	0	0	0
2011135952	41	rural/clinic	No	11/07/2017	0	0	0
2015313245	41	rural/clinic	No	22/06/2017	0	0	0
2015337374	41	rural/clinic	No	11/07/2017	0	0	0
2014350631	41	rural/clinic	No	11/07/2017	0	0	0
2014368484	41	rural/clinic	No	22/06/2017	0	0	0
2015363079	41	rural/clinic	No	11/07/2017	0	0	0
2015337373	41	rural/clinic	No	11/07/2017	0	0	0
2015363080	41	rural/clinic	No	11/07/2017	0	0	0
2014363229	41	rural/clinic	No	11/07/2017	0	0	0
2015324483	41	rural/clinic	No	11/07/2017	0	0	0
2012344860	41	rural/clinic	No	11/07/2017	0	0	0
2012344861	41	rural/clinic	No	11/07/2017	0	0	0
2014346734	41	rural/clinic	No	11/07/2017	0	0	0
2014346735	41	rural/clinic	No	11/07/2017	0	0	0
2014346736	41	rural/clinic	No	11/07/2017	0	0	0
2015368998	41	rural/clinic	No	11/07/2017	0	0	0
2015368999	41	rural/clinic	No	11/07/2017	0	0	0
2011152226	41	rural/clinic	No	11/07/2017	0	0	0
2015342316	41	rural/clinic	No	11/07/2017	0	0	0
2012369497	41	rural/clinic	No	11/07/2017	0	0	0
2015303094	41	rural/clinic	No	11/07/2017	0	0	0
2012344859	41	rural/clinic	No	11/07/2017	0	0	0
2015303095	41	rural/clinic	No	11/07/2017	0	0	0
2015363076	41	rural/clinic	No	11/07/2017	0	0	0
2012244290	41	rural/clinic	No	11/07/2017	0	0	0
2015303096	41	rural/clinic	No	11/07/2017	0	0	0
2014368688	41	rural/clinic	No	11/07/2017	0	0	0
2015303097	41	rural/clinic	No	11/07/2017	0	0	0
2014360971	41	rural/clinic	No	11/07/2017	0	0	0
2015303098	41	rural/clinic	No	11/07/2017	0	0	0
2014360972	41	rural/clinic	No	11/07/2017	0	0	0
2015338658	41	rural/clinic	No	11/07/2017	0	0	0
2015367255	41	rural/clinic	No	11/07/2017	0	0	0
2015385858	41	rural/clinic	No	11/07/2017	0	0	0
2014368686	41	rural/clinic	No	11/07/2017	0	0	0
2012290400	41	rural/clinic	No	11/07/2017	0	0	0
2015385857	41	rural/clinic	No	11/07/2017	0	0	0
2014368687	41	rural/clinic	No	11/07/2017	0	0	0
2015367253	41	rural/clinic	No	11/07/2017	0	0	0
2011223212	41	rural/clinic	No	11/07/2017	0	0	0
2014358024	41	rural/clinic	No	11/07/2017	0	0	0
2015367254	41	rural/clinic	No	11/07/2017	0	0	0
2015337625	41	rural/clinic	No	11/07/2017	0	0	0
2015337626	41	rural/clinic	No	11/07/2017	0	0	0
2015303123	41	rural/clinic	No	11/07/2017	0	0	0

2015375901	41	rural/clinic	No	11/07/2017	0	0	0
2012374299	41	rural/clinic	No	11/07/2017	0	0	0
2015290209	41	rural/clinic	No	11/07/2017	0	0	0
2014337224	41	rural/clinic	No	11/07/2017	0	0	0
2012374300	41	rural/clinic	No	11/07/2017	0	0	0
2015290210	41	rural/clinic	No	11/07/2017	0	0	0
2015405628	41	rural/clinic	No	11/07/2017	0	0	0
2015355524	41	rural/clinic	No	11/07/2017	0	0	0
2015290211	41	rural/clinic	No	11/07/2017	0	0	0
2015397310	41	rural/clinic	No	11/07/2017	0	0	0
2015324044	41	rural/clinic	No	11/07/2017	0	0	0
2014303706	41	rural/clinic	No	11/07/2017	0	0	0
2015397322	41	rural/clinic	No	11/07/2017	0	0	0
2014303707	41	rural/clinic	No	11/07/2017	0	0	0
2015314611	41	rural/clinic	No	11/07/2017	0	0	0
2015397323	41	rural/clinic	No	11/07/2017	0	0	0
2015397324	41	rural/clinic	No	11/07/2017	0	0	0
2015314613	41	rural/clinic	No	11/07/2017	0	0	0
2012253932	41	rural/clinic	No	11/07/2017	0	0	0
2015375906	41	rural/clinic	No	11/07/2017	0	0	0
2015314612	41	rural/clinic	No	11/07/2017	0	0	0
2015397325	41	rural/clinic	No	11/07/2017	0	0	0
2015290212	41	rural/clinic	No	11/07/2017	0	0	0
2012363403	41	rural/clinic	No	11/07/2017	0	0	0
2011135953	41	rural/clinic	No	11/07/2017	0	0	0
2015290213	41	rural/clinic	No	11/07/2017	0	0	0
2015351994	41	rural/clinic	No	11/07/2017	0	0	0
2014297978	41	rural/clinic	No	11/07/2017	0	0	0
2015412100	41	rural/clinic	No	11/07/2017	0	0	0
2014297977	41	rural/clinic	No	11/07/2017	0	0	0
2015351995	41	rural/clinic	No	11/07/2017	0	0	0
2015290032	41	rural/clinic	No	11/07/2017	0	0	0
2014375514	41	rural/clinic	No	11/07/2017	0	0	0
2015351996	41	rural/clinic	No	11/07/2017	0	0	0
2015369937	41	rural/clinic	No	11/07/2017	0	0	0
2014303708	41	rural/clinic	No	11/07/2017	0	0	0
2015375213	41	rural/clinic	No	11/07/2017	0	0	0
2015351997	41	rural/clinic	No	11/07/2017	0	0	0
2015369747	41	rural/clinic	No	11/07/2017	0	0	0
2015375420	41	rural/clinic	No	11/07/2017	0	0	0
2015369936	41	rural/clinic	No	11/07/2017	0	0	0
2015351998	41	rural/clinic	No	11/07/2017	0	0	0
2015368822	41	rural/clinic	No	11/07/2017	0	0	0
2014288013	41	rural/clinic	No	11/07/2017	0	0	0
2015351999	41	rural/clinic	No	11/07/2017	0	0	0
2015377850	41	rural/clinic	No	11/07/2017	0	0	0
2015290031	41	rural/clinic	No	11/07/2017	0	0	0
2015352000	41	rural/clinic	No	11/07/2017	0	0	0
2015377849	41	rural/clinic	No	11/07/2017	0	0	0
2015339603	41	rural/clinic	No	11/07/2017	0	0	0
2014383708	41	rural/clinic	No	11/07/2017	0	0	0
2015339601	41	rural/clinic	No	11/07/2017	0	0	0
2012276261	41	rural/clinic	No	11/07/2017	0	0	0
2015367702	41	rural/clinic	No	11/07/2017	0	0	0
2011144828	41	rural/clinic	No	11/07/2017	0	0	0
2015297500	41	rural/clinic	No	11/07/2017	0	0	0
2014290741	41	rural/clinic	No	11/07/2017	0	0	0
2015367701	41	rural/clinic	No	11/07/2017	0	0	0
2014290740	41	rural/clinic	No	11/07/2017	0	0	0
2012276262	41	rural/clinic	No	11/07/2017	0	0	0
2015297498	41	rural/clinic	No	11/07/2017	0	0	0
2014316071	41	rural/clinic	No	11/07/2017	0	0	0
2014332712	41	rural/clinic	No	11/07/2017	0	0	0
2014363894	41	rural/clinic	No	11/07/2017	0	0	0
2015360736	41	rural/clinic	No	11/07/2017	0	0	0
2014316070	41	rural/clinic	No	11/07/2017	0	0	0
2014363895	41	rural/clinic	No	11/07/2017	0	0	0
2015297349	41	rural/clinic	No	11/07/2017	0	0	0
2015384489	41	rural/clinic	No	11/07/2017	0	0	0
2014363896	41	rural/clinic	No	11/07/2017	0	0	0
2015333107	41	rural/clinic	No	11/07/2017	0	0	0
2012289688	41	rural/clinic	No	11/07/2017	0	0	0
2015333109	41	rural/clinic	No	11/07/2017	0	0	0
2011136144	41	rural/clinic	No	11/07/2017	0	0	0
2012289788	41	rural/clinic	No	11/07/2017	0	0	0
2015333110	41	rural/clinic	No	11/07/2017	0	0	0
2011136145	41	rural/clinic	No	11/07/2017	0	0	0
2015333111	41	rural/clinic	No	11/07/2017	0	0	0
2011136146	41	rural/clinic	No	11/07/2017	0	0	0
2015373751	41	rural/clinic	No	11/07/2017	0	0	0
2015383739	41	rural/clinic	No	11/07/2017	0	0	0
2013266078	41	rural/clinic	No	11/07/2017	0	0	0

2015373752	41	rural/clinic	No	11/07/2017	0	0	0
2015383737	41	rural/clinic	No	11/07/2017	0	0	0
2012325700	41	rural/clinic	No	11/07/2017	0	0	0
2014346737	41	rural/clinic	No	11/07/2017	0	0	0
2015320897	41	rural/clinic	No	11/07/2017	0	0	0
2012336633	41	rural/clinic	No	11/07/2017	0	0	0
2015320898	41	rural/clinic	No	11/07/2017	0	0	0
2015390317	41	rural/clinic	No	11/07/2017	0	0	0
2014366074	41	rural/clinic	No	11/07/2017	0	0	0
2012294066	41	rural/clinic	No	11/07/2017	0	0	0
2015407068	41	rural/clinic	No	11/07/2017	0	0	0
2012291370	41	rural/clinic	No	11/07/2017	0	0	0
2015407070	41	rural/clinic	No	11/07/2017	0	0	0
2015407069	41	rural/clinic	No	11/07/2017	0	0	0
2014342537	41	rural/clinic	No	11/07/2017	0	0	0
2014358223	41	rural/clinic	No	11/07/2017	0	0	0
2015360737	41	rural/clinic	No	11/07/2017	0	0	0
2014342536	41	rural/clinic	No	11/07/2017	0	0	0
2014347821	41	rural/clinic	No	11/07/2017	0	0	0
2014358221	41	rural/clinic	No	11/07/2017	0	0	0
2014347820	41	rural/clinic	No	11/07/2017	0	0	0
2014358222	41	rural/clinic	No	11/07/2017	0	0	0
2015413926	41	rural/clinic	No	11/07/2017	0	0	0
2013261938	41	rural/clinic	No	11/07/2017	0	0	0
2014327521	41	rural/clinic	No	11/07/2017	0	0	0
2015405670	41	rural/clinic	No	11/07/2017	0	0	0
2014327522	41	rural/clinic	No	11/07/2017	0	0	0
2015412329	41	rural/clinic	No	11/07/2017	0	0	0
2012314572	41	rural/clinic	No	11/07/2017	0	0	0
2015412328	41	rural/clinic	No	11/07/2017	0	0	0
2015335970	41	rural/clinic	No	11/07/2017	0	0	0
2015412327	41	rural/clinic	No	11/07/2017	0	0	0
2013271848	41	rural/clinic	No	11/07/2017	0	0	0
2015412326	41	rural/clinic	No	11/07/2017	0	0	0
2015335971	41	rural/clinic	No	11/07/2017	0	0	0
2015302445	41	rural/clinic	No	11/07/2017	0	0	0
2014382634	41	rural/clinic	No	11/07/2017	0	0	0
2015289337	41	rural/clinic	No	11/07/2017	0	0	0
2015325942	41	rural/clinic	No	11/07/2017	0	0	0
2015297497	41	rural/clinic	No	11/07/2017	0	0	0
2015289287	41	rural/clinic	No	11/07/2017	0	0	0
2015297495	41	rural/clinic	No	11/07/2017	0	0	0
2015297350	41	rural/clinic	No	11/07/2017	0	0	0
2015289289	41	rural/clinic	No	11/07/2017	0	0	0
2015412257	41	rural/clinic	No	11/07/2017	0	0	0
2015337995	41	rural/clinic	No	11/07/2017	0	0	0
2014382925/D	41	rural/clinic	No	11/07/2017	0	0	0
2015412902	41	rural/clinic	No	11/07/2017	0	0	0
2015412456	41	rural/clinic	No	11/07/2017	0	0	0
2015297158	41	rural/clinic	No	11/07/2017	0	0	0
2015297157	41	rural/clinic	No	11/07/2017	0	0	0
2015297156	41	rural/clinic	No	11/07/2017	0	0	0
2012269020	41	rural/clinic	No	11/07/2017	0	0	0
2014301328	41	rural/clinic	No	11/07/2017	0	0	0
2015383738	41	rural/clinic	No	11/07/2017	0	0	0
2013264073	41	rural/clinic	No	11/07/2017	0	0	0
2014327089	41	rural/clinic	No	06/07/2017	0	0	0
2014327089	41	rural/clinic	No	06/07/2017	0	0	0
2015301272	41	rural/clinic	No	06/07/2017	0	0	0
2015301272	41	rural/clinic	No	06/07/2017	0	0	0
2015369199	41	rural/clinic	No	06/07/2017	0	0	0
2015369199	41	rural/clinic	No	06/07/2017	0	0	0
2014314227	41	rural/clinic	No	06/07/2017	0	0	0
2014314227	41	rural/clinic	No	06/07/2017	0	0	0
2013264074	41	rural/clinic	No	11/07/2017	0	0	0
2015334658	41	rural/clinic	No	06/07/2017	0	0	0
2015334658	41	rural/clinic	No	06/07/2017	0	0	0
2015384048	41	rural/clinic	No	06/07/2017	0	0	0
2015384048	41	rural/clinic	No	06/07/2017	0	0	0
2015340148	41	rural/clinic	No	06/07/2017	0	0	0
2015340148	41	rural/clinic	No	06/07/2017	0	0	0
2013264075	41	rural/clinic	No	11/07/2017	0	0	0
2014334184	41	rural/clinic	No	11/07/2017	0	0	0
2015326435	41	rural/clinic	No	11/07/2017	0	0	0
2015326436	41	rural/clinic	No	11/07/2017	0	0	0
2015326437	41	rural/clinic	No	11/07/2017	0	0	0
2015326438	41	rural/clinic	No	11/07/2017	0	0	0
2015326439	41	rural/clinic	No	11/07/2017	0	0	0
2015326440	41	rural/clinic	No	11/07/2017	0	0	0
2015326441	41	rural/clinic	No	11/07/2017	0	0	0
2015372301	41	rural/clinic	No	11/07/2017	0	0	0
2015337627	41	rural/clinic	No	11/07/2017	0	0	0

2015409361	772	district/faith-based	No	11/07/2017	1	0	0
2015362432	772	district/faith-based	No	11/07/2017	1	0	0
2015362431	772	district/faith-based	No	11/07/2017	1	0	0
2015310661	772	district/faith-based	No	11/07/2017	1	0	0
2012253977	772	district/faith-based	No	11/07/2017	1	0	0
2014287900	772	district/faith-based	No	11/07/2017	1	0	0
2015325253	772	district/faith-based	No	11/07/2017	1	0	0
2015375905	772	district/faith-based	No	11/07/2017	1	0	0
2015325252	772	district/faith-based	No	11/07/2017	1	0	0
2017324706	772	district/faith-based	No	11/07/2017	1	0	0
2014303704	772	district/faith-based	No	11/07/2017	1	0	0
2014349923	772	district/faith-based	No	11/07/2017	1	0	0
2014349922	772	district/faith-based	No	11/07/2017	1	0	0
2014303702	772	district/faith-based	No	11/07/2017	1	0	0
2012271402	772	district/faith-based	No	11/07/2017	1	0	0
2015325254	772	district/faith-based	No	11/07/2017	1	0	0
2015302446	772	district/faith-based	No	11/07/2017	1	0	0
2014288142	772	district/faith-based	No	11/07/2017	1	0	0
2015375903	772	district/faith-based	No	11/07/2017	1	0	0
2015325183	772	district/faith-based	No	11/07/2017	1	0	0
2015375904	772	district/faith-based	No	11/07/2017	1	0	0
2014342794	772	district/faith-based	No	11/07/2017	1	0	0
2014342793	772	district/faith-based	No	11/07/2017	1	0	0
2015412098	772	district/faith-based	No	11/07/2017	1	0	0
2014298592	772	district/faith-based	No	11/07/2017	1	0	0
2012253976	772	district/faith-based	No	11/07/2017	1	0	0
2012253974	772	district/faith-based	No	11/07/2017	1	0	0
2015412099	772	district/faith-based	No	11/07/2017	1	0	0
2015375418	772	district/faith-based	No	11/07/2017	1	0	0
2015375419	772	district/faith-based	No	11/07/2017	1	0	0
2015375902	772	district/faith-based	No	11/07/2017	1	0	0
2013283505	772	district/faith-based	No	11/07/2017	1	0	0
2015357302	772	district/faith-based	No	11/07/2017	1	0	0
2014320301	772	district/faith-based	No	11/07/2017	1	0	0
2012330888	772	district/faith-based	No	11/07/2017	1	0	0
2014320303	772	district/faith-based	No	11/07/2017	1	0	0
2015363018	772	district/faith-based	No	11/07/2017	1	0	0
2012312203	772	district/faith-based	No	11/07/2017	1	0	0
2013256276	772	district/faith-based	No	11/07/2017	1	0	0
2015368602	772	district/faith-based	No	11/07/2017	1	0	0
2015360738	772	district/faith-based	No	11/07/2017	1	0	0
2015404744	772	district/faith-based	No	11/07/2017	1	0	0
2015404745	772	district/faith-based	No	11/07/2017	1	0	0
2015404746	772	district/faith-based	No	11/07/2017	1	0	0
2014290327	772	district/faith-based	No	11/07/2017	1	0	0
2014290328	772	district/faith-based	No	11/07/2017	1	0	0
1	772	district/faith-based	No	27/06/2017	1	0	0
2015373866	772	district/faith-based	No	13/07/2017	1	0	0
2015373867	772	district/faith-based	No	13/07/2017	1	0	0
2015326601	772	district/faith-based	No	13/07/2017	1	0	0
2014314228	772	district/faith-based	No	13/07/2017	1	0	0
2011198696	772	district/faith-based	No	13/07/2017	1	0	0
2015305808	772	district/faith-based	No	11/07/2017	1	0	0
2013274250	772	district/faith-based	No	13/07/2017	1	0	0
2015369074	772	district/faith-based	No	11/07/2017	1	0	0
2015369076	772	district/faith-based	No	11/07/2017	1	0	0
2012275207/D	772	district/faith-based	No	13/07/2017	1	0	0
2014287437	772	district/faith-based	No	11/07/2017	1	0	0
2015408683	772	district/faith-based	No	13/07/2017	1	0	0
2012275206	772	district/faith-based	No	13/07/2017	1	0	0
2015394650	772	district/faith-based	No	11/07/2017	1	0	0
2012275205	772	district/faith-based	No	13/07/2017	1	0	0
2012275204	772	district/faith-based	No	13/07/2017	1	0	0
2015394649	772	district/faith-based	No	11/07/2017	1	0	0
2012275208	772	district/faith-based	No	13/07/2017	1	0	0
2014382434	772	district/faith-based	No	11/07/2017	1	0	0
2012275209	772	district/faith-based	No	13/07/2017	1	0	0
2015412310	772	district/faith-based	No	13/07/2017	1	0	0
2015404974	772	district/faith-based	No	13/07/2017	1	0	0
2015404966	772	district/faith-based	No	13/07/2017	1	0	0
2015406325	772	district/faith-based	No	13/07/2017	1	0	0
2015404973	772	district/faith-based	No	13/07/2017	1	0	0
2015404972	772	district/faith-based	No	13/07/2017	1	0	0
2015404971	772	district/faith-based	No	13/07/2017	1	0	0
2014296331	772	district/faith-based	No	13/07/2017	1	0	0
2015339027	772	district/faith-based	No	13/07/2017	1	0	0
2015414561	772	district/faith-based	No	13/07/2017	1	0	0
2013252788	772	district/faith-based	No	13/07/2017	1	0	0
2014296332	772	district/faith-based	No	13/07/2017	1	0	0
2015408684	772	district/faith-based	No	13/07/2017	1	0	0
2011143950	772	district/faith-based	No	13/07/2017	1	0	0
2015384550	772	district/faith-based	No	13/07/2017	1	0	0

2015331551	772	district/faith-based	No	13/07/2017	1	0	0
2015384549	772	district/faith-based	No	13/07/2017	1	0	0
2015331552	772	district/faith-based	No	13/07/2017	1	0	0
2015340365	772	district/faith-based	No	13/07/2017	1	0	0
2014296333	772	district/faith-based	No	13/07/2017	1	0	0
2015379508	772	district/faith-based	No	13/07/2017	1	0	0
2015377183	772	district/faith-based	No	13/07/2017	1	0	0
2012341046	772	district/faith-based	No	13/07/2017	1	0	0
2015402572	772	district/faith-based	No	13/07/2017	1	0	0
2015377181	772	district/faith-based	No	13/07/2017	1	0	0
2014301897	772	district/faith-based	No	13/07/2017	1	0	0
2015402574	772	district/faith-based	No	13/07/2017	1	0	0
2015335396	772	district/faith-based	No	13/07/2017	1	0	0
2014301898	772	district/faith-based	No	13/07/2017	1	0	0
2015402575	772	district/faith-based	No	13/07/2017	1	0	0
2015335395	772	district/faith-based	No	13/07/2017	1	0	0
2014297350	772	district/faith-based	No	13/07/2017	1	0	0
2015402576	772	district/faith-based	No	13/07/2017	1	0	0
2015335394	772	district/faith-based	No	13/07/2017	1	0	0
2015402577	772	district/faith-based	No	13/07/2017	1	0	0
2015335393	772	district/faith-based	No	13/07/2017	1	0	0
2015293481	772	district/faith-based	No	13/07/2017	1	0	0
2011155236	772	district/faith-based	No	13/07/2017	1	0	0
2015364314	772	district/faith-based	No	13/07/2017	1	0	0
2011144829	772	district/faith-based	No	13/07/2017	1	0	0
2015372028	772	district/faith-based	No	13/07/2017	1	0	0
2015364313	772	district/faith-based	No	13/07/2017	1	0	0
2012336635	772	district/faith-based	No	13/07/2017	1	0	0
2015386294	772	district/faith-based	No	13/07/2017	1	0	0
2011140301	772	district/faith-based	No	13/07/2017	1	0	0
2012336634	772	district/faith-based	No	13/07/2017	1	0	0
2015386292	772	district/faith-based	No	13/07/2017	1	0	0
2015385540	772	district/faith-based	No	13/07/2017	1	0	0
2015386290	772	district/faith-based	No	13/07/2017	1	0	0
2011123920	772	district/faith-based	No	13/07/2017	1	0	0
2015386293	772	district/faith-based	No	13/07/2017	1	0	0
2015385542	772	district/faith-based	No	13/07/2017	1	0	0
2011226488	772	district/faith-based	No	13/07/2017	1	0	0
2015385541	772	district/faith-based	No	13/07/2017	1	0	0
2015386291	772	district/faith-based	No	13/07/2017	1	0	0
2011226487	772	district/faith-based	No	13/07/2017	1	0	0
2015386295	772	district/faith-based	No	13/07/2017	1	0	0
2015369010	772	district/faith-based	No	13/07/2017	1	0	0
2014371592	772	district/faith-based	No	13/07/2017	1	0	0
2014287987	772	district/faith-based	No	13/07/2017	1	0	0
2015386743	772	district/faith-based	No	13/07/2017	1	0	0
2014287986	772	district/faith-based	No	13/07/2017	1	0	0
2014371591	772	district/faith-based	No	13/07/2017	1	0	0
2015386740	772	district/faith-based	No	13/07/2017	1	0	0
2015332436	772	district/faith-based	No	13/07/2017	1	0	0
2014287985	772	district/faith-based	No	13/07/2017	1	0	0
2014358195	772	district/faith-based	No	13/07/2017	1	0	0
2015332435	772	district/faith-based	No	13/07/2017	1	0	0
2013272761	772	district/faith-based	No	13/07/2017	1	0	0
2017359205	772	district/faith-based	No	13/07/2017	1	0	0
2015332434	772	district/faith-based	No	13/07/2017	1	0	0
2011204483	772	district/faith-based	No	13/07/2017	1	0	0
2015286331	772	district/faith-based	No	13/07/2017	1	0	0
2011204481	772	district/faith-based	No	13/07/2017	1	0	0
2011204482	772	district/faith-based	No	13/07/2017	1	0	0
2013272762	772	district/faith-based	No	13/07/2017	1	0	0
2015418375	772	district/faith-based	No	13/07/2017	1	0	0
2015286330	772	district/faith-based	No	13/07/2017	1	0	0
2015418376	772	district/faith-based	No	13/07/2017	1	0	0
2015382563	772	district/faith-based	No	13/07/2017	1	0	0
2015340017	772	district/faith-based	No	13/07/2017	1	0	0
2014350462	772	district/faith-based	No	13/07/2017	1	0	0
2015340018	772	district/faith-based	No	13/07/2017	1	0	0
2015382565	772	district/faith-based	No	13/07/2017	1	0	0
2014370308	772	district/faith-based	No	13/07/2017	1	0	0
2015382562	772	district/faith-based	No	13/07/2017	1	0	0
2015382561	772	district/faith-based	No	13/07/2017	1	0	0
2015362824	772	district/faith-based	No	13/07/2017	1	0	0
2015382564	772	district/faith-based	No	13/07/2017	1	0	0
2015362823	772	district/faith-based	No	13/07/2017	1	0	0
2015382567	772	district/faith-based	No	13/07/2017	1	0	0
2015360202	772	district/faith-based	No	13/07/2017	1	0	0
2015362825	772	district/faith-based	No	13/07/2017	1	0	0
2015382566	772	district/faith-based	No	13/07/2017	1	0	0
2013247135	772	district/faith-based	No	13/07/2017	1	0	0
2015362826	772	district/faith-based	No	13/07/2017	1	0	0
2015401160	772	district/faith-based	No	13/07/2017	1	0	0

2015415065	772	district/faith-based	No	13/07/2017	1	0	0
2014358342	772	district/faith-based	No	13/07/2017	1	0	0
2015293480	772	district/faith-based	No	13/07/2017	1	0	0
2015401159	772	district/faith-based	No	13/07/2017	1	0	0
2015415062	772	district/faith-based	No	13/07/2017	1	0	0
2014338512	772	district/faith-based	No	13/07/2017	1	0	0
2015401157	772	district/faith-based	No	13/07/2017	1	0	0
2014358343	772	district/faith-based	No	13/07/2017	1	0	0
2015293483	772	district/faith-based	No	13/07/2017	1	0	0
2015352778	772	district/faith-based	No	13/07/2017	1	0	0
2012369814	772	district/faith-based	No	13/07/2017	1	0	0
2015293484	772	district/faith-based	No	13/07/2017	1	0	0
2012359167	772	district/faith-based	No	13/07/2017	1	0	0
2015336101	772	district/faith-based	No	13/07/2017	1	0	0
2013247134	772	district/faith-based	No	13/07/2017	1	0	0
2011199097	772	district/faith-based	No	13/07/2017	1	0	0
2013272760	772	district/faith-based	No	13/07/2017	1	0	0
2011199098	772	district/faith-based	No	13/07/2017	1	0	0
2015360203	772	district/faith-based	No	13/07/2017	1	0	0
2011225374	772	district/faith-based	No	13/07/2017	1	0	0
2012268540	772	district/faith-based	No	13/07/2017	1	0	0
2015419056	772	district/faith-based	No	13/07/2017	1	0	0
2015384301	772	district/faith-based	No	13/07/2017	1	0	0
2011225375	772	district/faith-based	No	13/07/2017	1	0	0
2012317565	772	district/faith-based	No	13/07/2017	1	0	0
2015419052	772	district/faith-based	No	13/07/2017	1	0	0
2012317564	772	district/faith-based	No	13/07/2017	1	0	0
2015419054	772	district/faith-based	No	13/07/2017	1	0	0
2015352775	772	district/faith-based	No	13/07/2017	1	0	0
2015352776	210	rural/clinic	No	13/07/2017	0	0	0
2011199096/D	210	rural/clinic	No	13/07/2017	0	0	0
2015419055	210	rural/clinic	No	13/07/2017	0	0	0
2015386747	210	rural/clinic	No	13/07/2017	0	0	0
2015386746	210	rural/clinic	No	13/07/2017	0	0	0
2015386745	210	rural/clinic	No	13/07/2017	0	0	0
2015386744	210	rural/clinic	No	13/07/2017	0	0	0
2015386741	210	rural/clinic	No	13/07/2017	0	0	0
2015386742	210	rural/clinic	No	13/07/2017	0	0	0
2015352777	210	rural/clinic	No	13/07/2017	0	0	0
2015402566	210	rural/clinic	No	13/07/2017	0	0	0
2015402567	210	rural/clinic	No	13/07/2017	0	0	0
2014378274	210	rural/clinic	No	13/07/2017	0	0	0
2015402568	210	rural/clinic	No	13/07/2017	0	0	0
2015289201	210	rural/clinic	No	13/07/2017	0	0	0
2015402569	210	rural/clinic	No	13/07/2017	0	0	0
2014323098	728	rural/clinic	No	13/07/2017	0	0	0
2014323099	728	rural/clinic	No	13/07/2017	0	0	0
2014323100	728	rural/clinic	No	13/07/2017	0	0	0
2014323100	728	rural/clinic	No	13/07/2017	0	0	0
2014323100	728	rural/clinic	No	13/07/2017	0	0	0
2015377851	728	rural/clinic	No	13/07/2017	0	0	0
2015377852	728	rural/clinic	No	13/07/2017	0	0	0
2015377853	728	rural/clinic	No	13/07/2017	0	0	0
2015377854	728	rural/clinic	No	13/07/2017	0	0	0
2015377855	728	rural/clinic	No	04/07/2017	0	0	0
2015377856	728	rural/clinic	No	13/07/2017	0	0	0
2015377857	728	rural/clinic	No	13/07/2017	0	0	0
2015377858	728	rural/clinic	No	13/07/2017	0	0	0
2015377859	728	rural/clinic	No	13/07/2017	0	0	0
2015377860	728	rural/clinic	No	13/07/2017	0	0	0
2015377861	728	rural/clinic	No	13/07/2017	0	0	0
2015377864	728	rural/clinic	No	13/07/2017	0	0	0
2015377863	728	rural/clinic	No	13/07/2017	0	0	0
2015377862	728	rural/clinic	No	13/07/2017	0	0	0
2015377865	728	rural/clinic	No	13/07/2017	0	0	0
2015377871	728	rural/clinic	No	13/07/2017	0	0	0
2015377870	728	rural/clinic	No	13/07/2017	0	0	0
2015377866	728	rural/clinic	No	13/07/2017	0	0	0
2015377868	728	rural/clinic	No	13/07/2017	0	0	0
2015377869	728	rural/clinic	No	13/07/2017	0	0	0
2015377870	728	rural/clinic	No	13/07/2017	0	0	0
2015377870	728	rural/clinic	No	13/07/2017	0	0	0
2015377874	728	rural/clinic	No	13/07/2017	0	0	0
2015377873	728	rural/clinic	No	13/07/2017	0	0	0
2015377872	728	rural/clinic	No	13/07/2017	0	0	0
2015377880	728	rural/clinic	No	13/07/2017	0	0	0
2015377878	728	rural/clinic	No	13/07/2017	0	0	0
2015377879	728	rural/clinic	No	13/07/2017	0	0	0
2015377877	728	rural/clinic	No	06/07/2017	0	0	0
2015377885	728	rural/clinic	No	06/07/2017	0	0	0
2015377884	728	rural/clinic	No	13/07/2017	0	0	0
2015377883	728	rural/clinic	No	11/07/2017	0	0	0

2015377882	728	rural/clinic	No	11/07/2017	0	0	0
2015377876	728	rural/clinic	No	11/07/2017	0	0	0
2015377875	728	rural/clinic	No	11/07/2017	0	0	0
2015377886	728	rural/clinic	No	11/07/2017	0	0	0
2015377887	728	rural/clinic	No	11/07/2017	0	0	0
2015377888	728	rural/clinic	No	06/07/2017	0	0	0
2015377887	728	rural/clinic	No	06/07/2017	0	0	0
2015377887	728	rural/clinic	No	13/07/2017	0	0	0
2015377888	728	rural/clinic	No	06/07/2017	0	0	0
2015377888	728	rural/clinic	No	06/07/2017	0	0	0
2015377889	728	rural/clinic	No	06/07/2017	0	0	0
2015377890	728	rural/clinic	No	06/07/2017	0	0	0
2015377891	728	rural/clinic	No	06/07/2017	0	0	0
2015377893	728	rural/clinic	No	06/07/2017	0	0	0
2015377892	728	rural/clinic	No	06/07/2017	0	0	0
2015377894	728	rural/clinic	No	06/07/2017	0	0	0
2015377895	728	rural/clinic	No	13/07/2017	0	0	0
2015377896	728	rural/clinic	No	13/07/2017	0	0	0
2015377897	728	rural/clinic	No	13/07/2017	0	0	0
2015377899	728	rural/clinic	No	13/07/2017	0	0	0
2015377900	728	rural/clinic	No	13/07/2017	0	0	0
2015297151	728	rural/clinic	No	13/07/2017	0	0	0
2015297152	728	rural/clinic	No	13/07/2017	0	0	0
2015297151	728	rural/clinic	No	13/07/2017	0	0	0
2015297151	728	rural/clinic	No	13/07/2017	0	0	0
2015297154	728	rural/clinic	No	13/07/2017	0	0	0
2015297153	728	rural/clinic	No	13/07/2017	0	0	0
2015297155	728	rural/clinic	No	13/07/2017	0	0	0
2015297158	728	rural/clinic	No	13/07/2017	0	0	0
2015297157	728	rural/clinic	No	13/07/2017	0	0	0
2015297156	728	rural/clinic	No	13/07/2017	0	0	0
2015297158	728	rural/clinic	No	13/07/2017	0	0	0
2015297160	728	rural/clinic	No	13/07/2017	0	0	0
2015297159	728	rural/clinic	No	13/07/2017	0	0	0
2015297161	728	rural/clinic	No	13/07/2017	0	0	0
2015297162	728	rural/clinic	No	13/07/2017	0	0	0
2015297163	728	rural/clinic	No	13/07/2017	0	0	0
2015297162	728	rural/clinic	No	13/07/2017	0	0	0
2015297162	728	rural/clinic	No	13/07/2017	0	0	0
2015297167	728	rural/clinic	No	13/07/2017	0	0	0
2015297164	728	rural/clinic	No	13/07/2017	0	0	0
2015297165	728	rural/clinic	No	13/07/2017	0	0	0
2015297166	728	rural/clinic	No	13/07/2017	0	0	0
2015297165	728	rural/clinic	No	13/07/2017	0	0	0
2015297169	728	rural/clinic	No	13/07/2017	0	0	0
2015297168	728	rural/clinic	No	13/07/2017	0	0	0
2015297170	728	rural/clinic	No	13/07/2017	0	0	0
2015297171	728	rural/clinic	No	13/07/2017	0	0	0
2015297175	728	rural/clinic	No	13/07/2017	0	0	0
2015297172	728	rural/clinic	No	13/07/2017	0	0	0
2015297173	728	rural/clinic	No	13/07/2017	0	0	0
2015297174	728	rural/clinic	No	13/07/2017	0	0	0
2015297173	728	rural/clinic	No	13/07/2017	0	0	0
2015297173	728	rural/clinic	No	13/07/2017	0	0	0
2015297176	728	rural/clinic	No	13/07/2017	0	0	0
2015297178	728	rural/clinic	No	13/07/2017	0	0	0
2015297177	728	rural/clinic	No	13/07/2017	0	0	0
2015297179	728	rural/clinic	No	13/07/2017	0	0	0
2015297180	728	rural/clinic	No	13/07/2017	0	0	0
2015297281	729	rural/clinic	No	13/07/2017	0	0	0
2015297282	729	rural/clinic	No	13/07/2017	0	0	0
2015297280	729	rural/clinic	No	13/07/2017	0	0	0
2015297283	729	rural/clinic	No	13/07/2017	0	0	0
2015297284	729	rural/clinic	No	13/07/2017	0	0	0
2015297286	729	rural/clinic	No	13/07/2017	0	0	0
2015369009	729	rural/clinic	No	13/07/2017	0	0	0
2015369011	729	rural/clinic	No	13/07/2017	0	0	0
2015369008	729	rural/clinic	No	13/07/2017	0	0	0
2015369007	729	rural/clinic	No	13/07/2017	0	0	0
2015319293	729	rural/clinic	No	13/07/2017	0	0	0
2015319292	729	rural/clinic	No	13/07/2017	0	0	0
2015319291	729	rural/clinic	No	13/07/2017	0	0	0
2011227276	729	rural/clinic	No	13/07/2017	0	0	0
2015377437	729	rural/clinic	No	13/07/2017	0	0	0
2015377436	729	rural/clinic	No	13/07/2017	0	0	0
2015377435	729	rural/clinic	No	13/07/2017	0	0	0
2013255743	729	rural/clinic	No	13/07/2017	0	0	0
2015384361	729	rural/clinic	No	13/07/2017	0	0	0
2014314655	729	rural/clinic	No	13/07/2017	0	0	0
2013255744	729	rural/clinic	No	13/07/2017	0	0	0
2015414463	729	rural/clinic	No	13/07/2017	0	0	0
2015368302	729	rural/clinic	No	13/07/2017	0	0	0

2014349240	729	rural/clinic	No	13/07/2017	0	0	0
2014290741	729	rural/clinic	No	11/07/2017	0	0	0
2014290741	427	rural/clinic	No	11/07/2017	0	0	0
2011136145	427	rural/clinic	No	11/07/2017	0	0	0
2011136145	427	rural/clinic	No	11/07/2017	0	0	0
2014363896	427	rural/clinic	No	11/07/2017	0	0	0
2014363896	427	rural/clinic	No	11/07/2017	0	0	0
2015375903	427	rural/clinic	No	11/07/2017	0	0	0
2015375903	427	rural/clinic	No	11/07/2017	0	0	0
2015362432	427	rural/clinic	No	11/07/2017	0	0	0
2015362432	427	rural/clinic	No	11/07/2017	0	0	0
2015397325	427	rural/clinic	No	11/07/2017	0	0	0
2014327521	427	rural/clinic	No	11/07/2017	0	0	0
2014327521	427	rural/clinic	No	11/07/2017	0	0	0
2013271848	427	rural/clinic	No	11/07/2017	0	0	0
2013271848	427	rural/clinic	No	11/07/2017	0	0	0
2013256276	427	rural/clinic	No	11/07/2017	0	0	0
2013256276	427	rural/clinic	No	11/07/2017	0	0	0
2014296332	427	rural/clinic	No	13/07/2017	0	0	0
2015369763	249	rural/clinic	No	13/07/2017	0	0	0
2015369761	249	rural/clinic	No	13/07/2017	0	0	0
2015369762	249	rural/clinic	No	13/07/2017	0	0	0
2015369767	249	rural/clinic	No	13/07/2017	0	0	0
2015369765	249	rural/clinic	No	13/07/2017	0	0	0
2015369766	249	rural/clinic	No	13/07/2017	0	0	0
2015369768	249	rural/clinic	No	13/07/2017	0	0	0
2015369769	249	rural/clinic	No	13/07/2017	0	0	0
2015369770	249	rural/clinic	No	13/07/2017	0	0	0
2015369772	249	rural/clinic	No	13/07/2017	0	0	0
2015369771	249	rural/clinic	No	13/07/2017	0	0	0
2015369773	249	rural/clinic	No	13/07/2017	0	0	0
2015369774	249	rural/clinic	No	13/07/2017	0	0	0
2015369775	249	rural/clinic	No	13/07/2017	0	0	0
2015369779	249	rural/clinic	No	13/07/2017	0	0	0
2015369778	249	rural/clinic	No	13/07/2017	0	0	0
2015369780	249	rural/clinic	No	13/07/2017	0	0	0
2015369777	249	rural/clinic	No	13/07/2017	0	0	0
2015369776	249	rural/clinic	No	13/07/2017	0	0	0
2015369785	249	rural/clinic	No	13/07/2017	0	0	0
2015369786	249	rural/clinic	No	13/07/2017	0	0	0
2015369784	249	rural/clinic	No	13/07/2017	0	0	0
2015369788	249	rural/clinic	No	13/07/2017	0	0	0
2015369787	249	rural/clinic	No	13/07/2017	0	0	0
2015369787	249	rural/clinic	No	13/07/2017	0	0	0
2015369787	249	rural/clinic	No	13/07/2017	0	0	0
2015369789	249	rural/clinic	No	13/07/2017	0	0	0
2013273901	249	rural/clinic	No	13/07/2017	0	0	0
2015369789	249	rural/clinic	No	13/07/2017	0	0	0
2015369789	249	rural/clinic	No	13/07/2017	0	0	0
2015369791	249	rural/clinic	No	13/07/2017	0	0	0
2015369792	249	rural/clinic	No	13/07/2017	0	0	0
2015369790	249	rural/clinic	No	13/07/2017	0	0	0
2015369793	249	rural/clinic	No	13/07/2017	0	0	0
2015369790	249	rural/clinic	No	13/07/2017	0	0	0
2015369790	249	rural/clinic	No	13/07/2017	0	0	0
2015369795	249	rural/clinic	No	13/07/2017	0	0	0
2015369794	249	rural/clinic	No	13/07/2017	0	0	0
2015369796	249	rural/clinic	No	13/07/2017	0	0	0
2015357519	819	rural/clinic	No	13/07/2017	0	0	0
2015357521	819	rural/clinic	No	13/07/2017	0	0	0
2015357522	819	rural/clinic	No	13/07/2017	0	0	0
2015357526	819	rural/clinic	No	13/07/2017	0	0	0
2015357524	819	rural/clinic	No	13/07/2017	0	0	0
2015357523	819	rural/clinic	No	13/07/2017	0	0	0
2015357525	819	rural/clinic	No	13/07/2017	0	0	0
2015357527	819	rural/clinic	No	13/07/2017	0	0	0
2015357528	819	rural/clinic	No	13/07/2017	0	0	0
2015357529	819	rural/clinic	No	13/07/2017	0	0	0
2015357530	819	rural/clinic	No	13/07/2017	0	0	0
2015357531	819	rural/clinic	No	13/07/2017	0	0	0
2015357532	819	rural/clinic	No	13/07/2017	0	0	0
2015357532	819	rural/clinic	No	13/07/2017	0	0	0
2015357532	819	rural/clinic	No	13/07/2017	0	0	0
2015357533	819	rural/clinic	No	13/07/2017	0	0	0
2015357534	819	rural/clinic	No	13/07/2017	0	0	0
2015357535	819	rural/clinic	No	13/07/2017	0	0	0
2015357535	819	rural/clinic	No	13/07/2017	0	0	0
2015357535	819	rural/clinic	No	13/07/2017	0	0	0
2015357536	819	rural/clinic	No	13/07/2017	0	0	0
2015357537	819	rural/clinic	No	13/07/2017	0	0	0
2015357537	819	rural/clinic	No	13/07/2017	0	0	0
2015357537	819	rural/clinic	No	13/07/2017	0	0	0

2015357538	819	rural/clinic	No	13/07/2017	0	0	0
2015357540	819	rural/clinic	No	13/07/2017	0	0	0
2015357539	819	rural/clinic	No	13/07/2017	0	0	0
2015357541	819	rural/clinic	No	13/07/2017	0	0	0
2015357542	819	rural/clinic	No	13/07/2017	0	0	0
2015357543	819	rural/clinic	No	13/07/2017	0	0	0
2014309376	428	rural/clinic	No	13/07/2017	0	0	0
2014309376	428	rural/clinic	No	13/07/2017	0	0	0
2014309376	428	rural/clinic	No	13/07/2017	0	0	0
2014309378	428	rural/clinic	No	13/07/2017	0	0	0
2014309377	428	rural/clinic	No	13/07/2017	0	0	0
2014309379	428	rural/clinic	No	13/07/2017	0	0	0
2014309379	428	rural/clinic	No	13/07/2017	0	0	0
2014309379	428	rural/clinic	No	13/07/2017	0	0	0
2014309380	428	rural/clinic	No	13/07/2017	0	0	0
2014309381	428	rural/clinic	No	13/07/2017	0	0	0
2011000001	428	rural/clinic	No	13/07/2017	0	0	0
2014309382	428	rural/clinic	No	13/07/2017	0	0	0
2014309383	428	rural/clinic	No	13/07/2017	0	0	0
2014309384	428	rural/clinic	No	13/07/2017	0	0	0
2014309386	428	rural/clinic	No	13/07/2017	0	0	0
2014309389	428	rural/clinic	No	13/07/2017	0	0	0
2014309388	428	rural/clinic	No	13/07/2017	0	0	0
2014309385/D	428	rural/clinic	No	13/07/2017	0	0	0
2014309391	428	rural/clinic	No	13/07/2017	0	0	0
2014309390	428	rural/clinic	No	13/07/2017	0	0	0
2014309392	428	rural/clinic	No	13/07/2017	0	0	0
2014309394	428	rural/clinic	No	13/07/2017	0	0	0
2014309393	428	rural/clinic	No	13/07/2017	0	0	0
2014309395	428	rural/clinic	No	13/07/2017	0	0	0
2014309397	428	rural/clinic	No	13/07/2017	0	0	0
2014309396	428	rural/clinic	No	13/07/2017	0	0	0
2014309400	428	rural/clinic	No	13/07/2017	0	0	0
2014309399	428	rural/clinic	No	13/07/2017	0	0	0
2015385505	429	rural/clinic	No	13/07/2017	0	0	0
2015385506	429	rural/clinic	No	13/07/2017	0	0	0
2015385507	429	rural/clinic	No	13/07/2017	0	0	0
2015385510	429	rural/clinic	No	13/07/2017	0	0	0
2015385509	429	rural/clinic	No	13/07/2017	0	0	0
2015385508	429	rural/clinic	No	13/07/2017	0	0	0
2015385512	429	rural/clinic	No	13/07/2017	0	0	0
2015385511	429	rural/clinic	No	13/07/2017	0	0	0
2015385514	429	rural/clinic	No	13/07/2017	0	0	0
2015385513	429	rural/clinic	No	13/07/2017	0	0	0
2015385515	429	rural/clinic	No	13/07/2017	0	0	0
2015385517	429	rural/clinic	No	13/07/2017	0	0	0
2015385516	429	rural/clinic	No	13/07/2017	0	0	0
2015385518	429	rural/clinic	No	13/07/2017	0	0	0
2015385519	429	rural/clinic	No	11/07/2017	0	0	0
2015385521	429	rural/clinic	No	11/07/2017	0	0	0
2015385520	429	rural/clinic	No	11/07/2017	0	0	0
2015385521	429	rural/clinic	No	13/07/2017	0	0	0
2015385521	429	rural/clinic	No	18/07/2017	0	0	0
2015385522	429	rural/clinic	No	18/07/2017	0	0	0
2015385524	429	rural/clinic	No	18/07/2017	0	0	0
2015385523	429	rural/clinic	No	17/07/2017	0	0	0
2015385524	429	rural/clinic	No	17/07/2017	0	0	0
2015385524	429	rural/clinic	No	18/07/2017	0	0	0
2015385525	429	rural/clinic	No	17/07/2017	0	0	0
2015385526	429	rural/clinic	No	18/07/2017	0	0	0
2015385525	429	rural/clinic	No	17/07/2017	0	0	0
2015385525	429	rural/clinic	No	18/07/2017	0	0	0
2015385527	429	rural/clinic	No	18/07/2017	0	0	0
2015385530	429	rural/clinic	No	17/07/2017	0	0	0
2015385529	429	rural/clinic	No	17/07/2017	0	0	0
2015385528	429	rural/clinic	No	17/07/2017	0	0	0
2015385531	429	rural/clinic	No	18/07/2017	0	0	0
2015385533	429	rural/clinic	No	18/07/2017	0	0	0
2015385534	429	rural/clinic	No	18/07/2017	0	0	0
2015385532	429	rural/clinic	No	18/07/2017	0	0	0
2015385535	429	rural/clinic	No	18/07/2017	0	0	0
2015385536	429	rural/clinic	No	18/07/2017	0	0	0
2015385539	429	rural/clinic	No	18/07/2017	0	0	0
2015385537	429	rural/clinic	No	17/07/2017	0	0	0
2015385539	429	rural/clinic	No	18/07/2017	0	0	0
2015385539	429	rural/clinic	No	18/07/2017	0	0	0
2015385540	429	rural/clinic	No	18/07/2017	0	0	0
2015385542	429	rural/clinic	No	18/07/2017	0	0	0
2015385541	429	rural/clinic	No	18/07/2017	0	0	0
2015385543	429	rural/clinic	No	18/07/2017	0	0	0
2015385544	429	rural/clinic	No	17/07/2017	0	0	0
2015385545	429	rural/clinic	No	18/07/2017	0	0	0

2015385546	429	rural/clinic	No	18/07/2017	0	0	0
2015385548	429	rural/clinic	No	17/07/2017	0	0	0
2015385547	429	rural/clinic	No	18/07/2017	0	0	0
2015385549	429	rural/clinic	No	18/07/2017	0	0	0
2015385550	429	rural/clinic	No	17/07/2017	0	0	0
2013256701	429	rural/clinic	No	18/07/2017	0	0	0
2013256702	429	rural/clinic	No	18/07/2017	0	0	0
2013256704	429	rural/clinic	No	18/07/2017	0	0	0
2013256705	429	rural/clinic	No	18/07/2017	0	0	0
2013256703	429	rural/clinic	No	18/07/2017	0	0	0
2013256706	429	rural/clinic	No	18/07/2017	0	0	0
2013256708	429	rural/clinic	No	18/07/2017	0	0	0
2013256709	429	rural/clinic	No	18/07/2017	0	0	0
2013256710	429	rural/clinic	No	18/07/2017	0	0	0
2013256711	429	rural/clinic	No	18/07/2017	0	0	0
2013256713	429	rural/clinic	No	17/07/2017	0	0	0
2013256712	429	rural/clinic	No	18/07/2017	0	0	0
2013256715	429	rural/clinic	No	18/07/2017	0	0	0
2013256716	429	rural/clinic	No	18/07/2017	0	0	0
2013256717	429	rural/clinic	No	18/07/2017	0	0	0
2013256718	429	rural/clinic	No	18/07/2017	0	0	0
2013256720	429	rural/clinic	No	18/07/2017	0	0	0
2013256719	429	rural/clinic	No	18/07/2017	0	0	0
2013256721	429	rural/clinic	No	18/07/2017	0	0	0
2013256724	429	rural/clinic	No	18/07/2017	0	0	0
2013256723	429	rural/clinic	No	18/07/2017	0	0	0
2013256722	429	rural/clinic	No	18/07/2017	0	0	0
2013256726	429	rural/clinic	No	18/07/2017	0	0	0
2013256725	429	rural/clinic	No	17/07/2017	0	0	0
2013256727	429	rural/clinic	No	18/07/2017	0	0	0
2013256728	429	rural/clinic	No	18/07/2017	0	0	0
2013256729	429	rural/clinic	No	18/07/2017	0	0	0
2013249420	930	rural/clinic	No	18/07/2017	0	0	0
2012271103	930	rural/clinic	No	18/07/2017	0	0	0
2013281901	930	rural/clinic	No	18/07/2017	0	0	0
2013249421	930	rural/clinic	No	18/07/2017	0	0	0
2011212599	930	rural/clinic	No	18/07/2017	0	0	0
2015342053	930	rural/clinic	No	18/07/2017	0	0	0
2015334656	930	rural/clinic	No	18/07/2017	0	0	0
2015342056	930	rural/clinic	No	18/07/2017	0	0	0
2015334657	930	rural/clinic	No	18/07/2017	0	0	0
2014288015	930	rural/clinic	No	18/07/2017	0	0	0
2015334210	930	rural/clinic	No	18/07/2017	0	0	0
2015342057	930	rural/clinic	No	18/07/2017	0	0	0
2012271101	930	rural/clinic	No	18/07/2017	0	0	0
2014297602	930	rural/clinic	No	18/07/2017	0	0	0
2015384490	930	rural/clinic	No	18/07/2017	0	0	0
2014297601	930	rural/clinic	No	18/07/2017	0	0	0
2014327523	930	rural/clinic	No	18/07/2017	0	0	0
2012271104	930	rural/clinic	No	18/07/2017	0	0	0
2012265286	930	rural/clinic	No	18/07/2017	0	0	0
2014327524	930	rural/clinic	No	18/07/2017	0	0	0
2015310664	774	rural/clinic	No	18/07/2017	0	0	0
2015310663	774	rural/clinic	No	18/07/2017	0	0	0
2015377751	774	rural/clinic	No	18/07/2017	0	0	0
2015375907	774	rural/clinic	No	18/07/2017	0	0	0
2015377752	774	rural/clinic	No	18/07/2017	0	0	0
2014303716	774	rural/clinic	No	18/07/2017	0	0	0
2015369000	774	rural/clinic	No	18/07/2017	0	0	0
2015390318	774	rural/clinic	No	18/07/2017	0	0	0
2011157097	774	rural/clinic	No	18/07/2017	0	0	0
2015390320	774	rural/clinic	No	18/07/2017	0	0	0
2015340226	774	rural/clinic	No	18/07/2017	0	0	0
2015390319	774	rural/clinic	No	18/07/2017	0	0	0
2014297605	774	rural/clinic	No	18/07/2017	0	0	0
2015305810	774	rural/clinic	No	18/07/2017	0	0	0
2015340227	774	rural/clinic	No	18/07/2017	0	0	0
2015340228	774	rural/clinic	No	18/07/2017	0	0	0
2015305811	774	rural/clinic	No	18/07/2017	0	0	0
2015340229	774	rural/clinic	No	18/07/2017	0	0	0
2015363081	774	rural/clinic	No	18/07/2017	0	0	0
2014332713	774	rural/clinic	No	18/07/2017	0	0	0
2015405918	774	rural/clinic	No	18/07/2017	0	0	0
2012330890	603	rural/clinic	No	18/07/2017	0	0	0
2014332714	603	rural/clinic	No	18/07/2017	0	0	0
2014350637	603	rural/clinic	No	18/07/2017	0	0	0
2015339770	603	rural/clinic	No	18/07/2017	0	0	0
2014346287	603	rural/clinic	No	18/07/2017	0	0	0
2015344341	603	rural/clinic	No	18/07/2017	0	0	0
2014346288	603	rural/clinic	No	18/07/2017	0	0	0
2015287386/D	603	rural/clinic	No	18/07/2017	0	0	0
2014357328	603	rural/clinic	No	18/07/2017	0	0	0

2011139408	603	rural/clinic	No	18/07/2017	0	0	0
2014357856	603	rural/clinic	No	18/07/2017	0	0	0
2011139407	603	rural/clinic	No	18/07/2017	0	0	0
2014357853	604	rural/clinic	No	18/07/2017	0	0	0
2011195785	604	rural/clinic	No	18/07/2017	0	0	0
2015344340	604	rural/clinic	No	18/07/2017	0	0	0
2015287319	604	rural/clinic	No	18/07/2017	0	0	0
2015344342	604	rural/clinic	No	18/07/2017	0	0	0
2015344339	604	rural/clinic	No	18/07/2017	0	0	0
2012322691	604	rural/clinic	No	18/07/2017	0	0	0
2014311373	604	rural/clinic	No	18/07/2017	0	0	0
2015287385	604	rural/clinic	No	18/07/2017	0	0	0
2014357857	604	rural/clinic	No	18/07/2017	0	0	0
2015287384	604	rural/clinic	No	18/07/2017	0	0	0
2014311374	604	rural/clinic	No	18/07/2017	0	0	0
2015409026	604	rural/clinic	No	18/07/2017	0	0	0
2012322690	604	rural/clinic	No	18/07/2017	0	0	0
2011195904	604	rural/clinic	No	18/07/2017	0	0	0
2014368689	604	rural/clinic	No	18/07/2017	0	0	0
2015368725	604	rural/clinic	No	18/07/2017	0	0	0
2014368690	604	rural/clinic	No	18/07/2017	0	0	0
2015368728	604	rural/clinic	No	18/07/2017	0	0	0
2015413927	604	rural/clinic	No	18/07/2017	0	0	0
2015368727	604	rural/clinic	No	18/07/2017	0	0	0
2015293844	604	rural/clinic	No	18/07/2017	0	0	0
2015342317	604	rural/clinic	No	18/07/2017	0	0	0
2014327688	672	rural/clinic	No	18/07/2017	0	0	0
2015342318	672	rural/clinic	No	18/07/2017	0	0	0
2015303483	672	rural/clinic	No	18/07/2017	0	0	0
2015342320	672	rural/clinic	No	18/07/2017	0	0	0
2015303482	672	rural/clinic	No	18/07/2017	0	0	0
2012362566	672	rural/clinic	No	18/07/2017	0	0	0
2015344663	672	rural/clinic	No	18/07/2017	0	0	0
2015303485	672	rural/clinic	No	18/07/2017	0	0	0
2012362567	672	rural/clinic	No	18/07/2017	0	0	0
2012362568	672	rural/clinic	No	18/07/2017	0	0	0
2015344664	672	rural/clinic	No	18/07/2017	0	0	0
2012362569	672	rural/clinic	No	18/07/2017	0	0	0
2015324573	672	rural/clinic	No	18/07/2017	0	0	0
2012362570	672	rural/clinic	No	18/07/2017	0	0	0
2014335582	672	rural/clinic	No	18/07/2017	0	0	0
2014335583	672	rural/clinic	No	18/07/2017	0	0	0
2012362571	672	rural/clinic	No	18/07/2017	0	0	0
2012318892	672	rural/clinic	No	18/07/2017	0	0	0
2014307417	672	rural/clinic	No	18/07/2017	0	0	0
2014335587	672	rural/clinic	No	18/07/2017	0	0	0
2011136147	730	rural/clinic	No	18/07/2017	0	0	0
2014288579	730	rural/clinic	No	18/07/2017	0	0	0
2011136148	730	rural/clinic	No	18/07/2017	0	0	0
2014288580	730	rural/clinic	No	18/07/2017	0	0	0
2011136149	730	rural/clinic	No	18/07/2017	0	0	0
2014288581	730	rural/clinic	No	18/07/2017	0	0	0
2011136150	730	rural/clinic	No	18/07/2017	0	0	0
2015344741	730	rural/clinic	No	18/07/2017	0	0	0
2014350556	730	rural/clinic	No	18/07/2017	0	0	0
2012267320	730	rural/clinic	No	18/07/2017	0	0	0
2012267319	730	rural/clinic	No	18/07/2017	0	0	0
2015344742	730	rural/clinic	No	18/07/2017	0	0	0
2014306043	730	rural/clinic	No	18/07/2017	0	0	0
2015344743	730	rural/clinic	No	18/07/2017	0	0	0
2014306044	730	rural/clinic	No	18/07/2017	0	0	0
2014288582	730	rural/clinic	No	18/07/2017	0	0	0
2011167841	730	rural/clinic	No	18/07/2017	0	0	0
2015344336	730	rural/clinic	No	18/07/2017	0	0	0
2015344338	730	rural/clinic	No	18/07/2017	0	0	0
2015344337	730	rural/clinic	No	18/07/2017	0	0	0
2015344335	730	rural/clinic	No	18/07/2017	0	0	0
2015344334	730	rural/clinic	No	18/07/2017	0	0	0
2012265287	730	rural/clinic	No	18/07/2017	0	0	0
2014311372	730	rural/clinic	No	18/07/2017	0	0	0
2014297603	730	rural/clinic	No	18/07/2017	0	0	0
2014297604	189	rural/clinic	No	18/07/2017	0	0	0
2015378305	189	rural/clinic	No	18/07/2017	0	0	0
2011140775	189	rural/clinic	No	18/07/2017	0	0	0
2011140776	189	rural/clinic	No	18/07/2017	0	0	0
2011140777	189	rural/clinic	No	18/07/2017	0	0	0
2011140778	189	rural/clinic	No	18/07/2017	0	0	0
2011140779	189	rural/clinic	No	18/07/2017	0	0	0
2011140780	189	rural/clinic	No	18/07/2017	0	0	0
2011140781	189	rural/clinic	No	18/07/2017	0	0	0
2015344456	189	rural/clinic	No	18/07/2017	0	0	0
2015344457	189	rural/clinic	No	18/07/2017	0	0	0

2015344458	189	rural/clinic	No	18/07/2017	0	0	0
2015344459	189	rural/clinic	No	18/07/2017	0	0	0
2015344460	189	rural/clinic	No	18/07/2017	0	0	0
2014335585	189	rural/clinic	No	18/07/2017	0	0	0
2011140782	189	rural/clinic	No	18/07/2017	0	0	0
2014306042	189	rural/clinic	No	18/07/2017	0	0	0
2014290276	189	rural/clinic	No	18/07/2017	0	0	0
2012289789	189	rural/clinic	No	18/07/2017	0	0	0
2014306045	189	rural/clinic	No	18/07/2017	0	0	0
2014306046	189	rural/clinic	No	18/07/2017	0	0	0
2014383709	189	rural/clinic	No	18/07/2017	0	0	0
2014306047	483	rural/clinic	No	18/07/2017	0	0	0
2014290274	483	rural/clinic	No	18/07/2017	0	0	0
2014306049	483	rural/clinic	No	18/07/2017	0	0	0
2014338453	483	rural/clinic	No	18/07/2017	0	0	0
2014363897	483	rural/clinic	No	18/07/2017	0	0	0
2012275990	483	rural/clinic	No	18/07/2017	0	0	0
2015368674	483	rural/clinic	No	18/07/2017	0	0	0
2014375515	483	rural/clinic	No	18/07/2017	0	0	0
2015368673	483	rural/clinic	No	18/07/2017	0	0	0
2015414264	483	rural/clinic	No	18/07/2017	0	0	0
2015414265	483	rural/clinic	No	18/07/2017	0	0	0
2014306050	483	rural/clinic	No	18/07/2017	0	0	0
2015414266	483	rural/clinic	No	18/07/2017	0	0	0
2014306048	483	rural/clinic	No	18/07/2017	0	0	0
2014375516	483	rural/clinic	No	18/07/2017	0	0	0
2011117851	483	rural/clinic	No	18/07/2017	0	0	0
2015339602	483	rural/clinic	No	18/07/2017	0	0	0
2014303711	483	rural/clinic	No	18/07/2017	0	0	0
2014367627	483	rural/clinic	No	18/07/2017	0	0	0
2015339157	430	rural/clinic	No	18/07/2017	0	0	0
2015290033	430	rural/clinic	No	18/07/2017	0	0	0
2014303714	430	rural/clinic	No	18/07/2017	0	0	0
2015419006	430	rural/clinic	No	18/07/2017	0	0	0
2015419005	430	rural/clinic	No	18/07/2017	0	0	0
2014303710	430	rural/clinic	No	18/07/2017	0	0	0
2014303713	430	rural/clinic	No	18/07/2017	0	0	0
2014303717	430	rural/clinic	No	18/07/2017	0	0	0
2015310618	430	rural/clinic	No	18/07/2017	0	0	0
2012379902	430	rural/clinic	No	18/07/2017	0	0	0
2015294624	430	rural/clinic	No	18/07/2017	0	0	0
2011135673	430	rural/clinic	No	18/07/2017	0	0	0
2011135675	430	rural/clinic	No	18/07/2017	0	0	0
2015413600	430	rural/clinic	No	18/07/2017	0	0	0
2011140551	430	rural/clinic	No	18/07/2017	0	0	0
2011136252	430	rural/clinic	No	18/07/2017	0	0	0
2011136253	430	rural/clinic	No	18/07/2017	0	0	0
2014327602	430	rural/clinic	No	18/07/2017	0	0	0
2014327603	430	rural/clinic	No	18/07/2017	0	0	0
2015362292	430	rural/clinic	No	18/07/2017	0	0	0
2014327604	430	rural/clinic	No	18/07/2017	0	0	0
2015362293	430	rural/clinic	No	18/07/2017	0	0	0
2012291371	430	rural/clinic	No	18/07/2017	0	0	0
2011135676	430	rural/clinic	No	18/07/2017	0	0	0
2011135677	430	rural/clinic	No	18/07/2017	0	0	0
2014335588	430	rural/clinic	No	18/07/2017	0	0	0
2014335589	430	rural/clinic	No	18/07/2017	0	0	0
2011140552	430	rural/clinic	No	18/07/2017	0	0	0
2014312204	430	rural/clinic	No	18/07/2017	0	0	0
2014382706	430	rural/clinic	No	18/07/2017	0	0	0
2014342538	430	rural/clinic	No	18/07/2017	0	0	0
2011137315	430	rural/clinic	No	18/07/2017	0	0	0
2014342539	430	rural/clinic	No	18/07/2017	0	0	0
2014342541	430	rural/clinic	No	18/07/2017	0	0	0
2011137316	430	rural/clinic	No	13/07/2017	0	0	0
2014342540	430	rural/clinic	No	18/07/2017	0	0	0
2011137317	430	rural/clinic	No	18/07/2017	0	0	0
2014358224	430	rural/clinic	No	18/07/2017	0	0	0
2014358225	430	rural/clinic	No	18/07/2017	0	0	0
2015326971	430	rural/clinic	No	18/07/2017	0	0	0
2015397935	430	rural/clinic	No	18/07/2017	0	0	0
2015326969	430	rural/clinic	No	18/07/2017	0	0	0
2015383742	430	rural/clinic	No	18/07/2017	0	0	0
2015297160	430	rural/clinic	No	18/07/2017	0	0	0
2015397933	430	rural/clinic	No	18/07/2017	0	0	0
2015326970	430	rural/clinic	No	18/07/2017	0	0	0
2014327689	430	rural/clinic	No	18/07/2017	0	0	0
2014365072	366	rural/clinic	No	20/07/2017	0	0	0
2014365074	366	rural/clinic	No	20/07/2017	0	0	0
2014365075	366	rural/clinic	No	20/07/2017	0	0	0
2014365076	366	rural/clinic	No	20/07/2017	0	0	0
2014365077	366	rural/clinic	No	20/07/2017	0	0	0

2014365078	366	rural/clinic	No	20/07/2017	0	0	0
2015337605	931	rural/clinic	No	20/07/2017	0	0	0
2015337607	931	rural/clinic	No	20/07/2017	0	0	0
2015337606	931	rural/clinic	No	20/07/2017	0	0	0
2015337608	931	rural/clinic	No	20/07/2017	0	0	0
2015337609	931	rural/clinic	No	20/07/2017	0	0	0
2015337608	931	rural/clinic	No	20/07/2017	0	0	0
2015337608	931	rural/clinic	No	20/07/2017	0	0	0
2015337611	931	rural/clinic	No	20/07/2017	0	0	0
2015337610	931	rural/clinic	No	20/07/2017	0	0	0
2015337612	931	rural/clinic	No	20/07/2017	0	0	0
2015337614	931	rural/clinic	No	20/07/2017	0	0	0
2015337616	931	rural/clinic	No	20/07/2017	0	0	0
2015337613	931	rural/clinic	No	20/07/2017	0	0	0
2015337615	931	rural/clinic	No	20/07/2017	0	0	0
2015337617	931	rural/clinic	No	20/07/2017	0	0	0
2015337618	931	rural/clinic	No	20/07/2017	0	0	0
2015337619	931	rural/clinic	No	20/07/2017	0	0	0
2015337620	931	rural/clinic	No	20/07/2017	0	0	0
2015337621	931	rural/clinic	No	20/07/2017	0	0	0
2015337623	931	rural/clinic	No	20/07/2017	0	0	0
2015337624	931	rural/clinic	No	20/07/2017	0	0	0
2015337622	931	rural/clinic	No	20/07/2017	0	0	0
2015337625	931	rural/clinic	No	20/07/2017	0	0	0
2015337626	931	rural/clinic	No	20/07/2017	0	0	0
2015337627	931	rural/clinic	No	20/07/2017	0	0	0
2015337628	931	rural/clinic	No	20/07/2017	0	0	0
2015337630	931	rural/clinic	No	20/07/2017	0	0	0
2015337629	931	rural/clinic	No	20/07/2017	0	0	0
2015337632	931	rural/clinic	No	20/07/2017	0	0	0
2015337631	931	rural/clinic	No	20/07/2017	0	0	0
2015337635	931	rural/clinic	No	20/07/2017	0	0	0
2015337634	931	rural/clinic	No	20/07/2017	0	0	0
2015337633	931	rural/clinic	No	20/07/2017	0	0	0
2015337636	931	rural/clinic	No	20/07/2017	0	0	0
2015337637	931	rural/clinic	No	20/07/2017	0	0	0
2015337638	931	rural/clinic	No	20/07/2017	0	0	0
2015337640	931	rural/clinic	No	20/07/2017	0	0	0
2015337641	931	rural/clinic	No	20/07/2017	0	0	0
2015337639	931	rural/clinic	No	20/07/2017	0	0	0
2015337640	931	rural/clinic	No	20/07/2017	0	0	0
2015337639	931	rural/clinic	No	20/07/2017	0	0	0
2015337639	931	rural/clinic	No	20/07/2017	0	0	0
2015337642	931	rural/clinic	No	20/07/2017	0	0	0
2015337645	931	rural/clinic	No	20/07/2017	0	0	0
2015337643	931	rural/clinic	No	20/07/2017	0	0	0
2015337645	931	rural/clinic	No	20/07/2017	0	0	0
2015337645	931	rural/clinic	No	20/07/2017	0	0	0
2015337647	931	rural/clinic	No	20/07/2017	0	0	0
2015337646	931	rural/clinic	No	20/07/2017	0	0	0
2015337648	931	rural/clinic	No	20/07/2017	0	0	0
2015337644	931	rural/clinic	No	20/07/2017	0	0	0
2015337646	931	rural/clinic	No	20/07/2017	0	0	0
2015337646	931	rural/clinic	No	20/07/2017	0	0	0
2015337649	931	rural/clinic	No	20/07/2017	0	0	0
2015289561	932	rural/clinic	No	20/07/2017	0	0	0
2015289562	324	rural/clinic	No	20/07/2017	0	0	0
2015289563	324	rural/clinic	No	20/07/2017	0	0	0
2015289564	324	rural/clinic	No	20/07/2017	0	0	0
2015289565	324	rural/clinic	No	20/07/2017	0	0	0
2015289566	324	rural/clinic	No	20/07/2017	0	0	0
2015289567	324	rural/clinic	No	20/07/2017	0	0	0
2015289568	324	rural/clinic	No	20/07/2017	0	0	0
2015289569	324	rural/clinic	No	20/07/2017	0	0	0
2015289570	324	rural/clinic	No	20/07/2017	0	0	0
2015289571	324	rural/clinic	No	20/07/2017	0	0	0
2015289573	324	rural/clinic	No	20/07/2017	0	0	0
2015289572	324	rural/clinic	No	20/07/2017	0	0	0
2015289574	324	rural/clinic	No	20/07/2017	0	0	0
2015289577	324	rural/clinic	No	20/07/2017	0	0	0
2015289578	324	rural/clinic	No	20/07/2017	0	0	0
2015289579	324	rural/clinic	No	20/07/2017	0	0	0
2015289580	324	rural/clinic	No	20/07/2017	0	0	0
2015289580	324	rural/clinic	No	20/07/2017	0	0	0
2015289580	324	rural/clinic	No	20/07/2017	0	0	0
2015289581	324	rural/clinic	No	20/07/2017	0	0	0
2011199238	775	rural/clinic	No	20/07/2017	0	0	0
2011199239	775	rural/clinic	No	20/07/2017	0	0	0
2011199240	775	rural/clinic	No	20/07/2017	0	0	0
2011199240	775	rural/clinic	No	20/07/2017	0	0	0
2011199240	775	rural/clinic	No	20/07/2017	0	0	0
2011199243	775	rural/clinic	No	20/07/2017	0	0	0

2011199244	775	rural/clinic	No	20/07/2017	0	0	0
2011199242	775	rural/clinic	No	20/07/2017	0	0	0
2011199245	775	rural/clinic	No	20/07/2017	0	0	0
2011199246	775	rural/clinic	No	20/07/2017	0	0	0
2011199247	775	rural/clinic	No	20/07/2017	0	0	0
2011199248	775	rural/clinic	No	20/07/2017	0	0	0
2011199250	775	rural/clinic	No	20/07/2017	0	0	0
2011199249	775	rural/clinic	No	20/07/2017	0	0	0
2015410151	775	rural/clinic	No	20/07/2017	0	0	0
2015410154	775	rural/clinic	No	20/07/2017	0	0	0
2015410152	775	rural/clinic	No	20/07/2017	0	0	0
2015410156	775	rural/clinic	No	20/07/2017	0	0	0
2014368013	776	rural/clinic	No	20/07/2017	0	0	0
2014368015	776	rural/clinic	No	20/07/2017	0	0	0
2014368014	776	rural/clinic	No	20/07/2017	0	0	0
2014368016	776	rural/clinic	No	20/07/2017	0	0	0
2014368018	776	rural/clinic	No	20/07/2017	0	0	0
2014368017	776	rural/clinic	No	20/07/2017	0	0	0
2014368019	776	rural/clinic	No	20/07/2017	0	0	0
2014368020	776	rural/clinic	No	20/07/2017	0	0	0
2014368021	776	rural/clinic	No	20/07/2017	0	0	0
2014368022	776	rural/clinic	No	20/07/2017	0	0	0
2014368024	776	rural/clinic	No	20/07/2017	0	0	0
2014368023	776	rural/clinic	No	20/07/2017	0	0	0
2014368026	776	rural/clinic	No	20/07/2017	0	0	0
2014368025	776	rural/clinic	No	20/07/2017	0	0	0
2014368028	776	rural/clinic	No	20/07/2017	0	0	0
2014368029	776	rural/clinic	No	20/07/2017	0	0	0
2014368027	776	rural/clinic	No	20/07/2017	0	0	0
2014368029	776	rural/clinic	No	20/07/2017	0	0	0
2015402565	776	rural/clinic	No	20/07/2017	0	0	0
2015402552	776	rural/clinic	No	20/07/2017	0	0	0
2015404652	776	rural/clinic	No	26/07/2017	0	0	0
2015402553	776	rural/clinic	No	20/07/2017	0	0	0
2015402554	776	rural/clinic	No	20/07/2017	0	0	0
2015349539	776	rural/clinic	No	20/07/2017	0	0	0
2015402555	776	rural/clinic	No	20/07/2017	0	0	0
2015402556	776	rural/clinic	No	20/07/2017	0	0	0
2014302947	776	rural/clinic	No	20/07/2017	0	0	0
2015402557	776	rural/clinic	No	20/07/2017	0	0	0
2014302948	776	rural/clinic	No	20/07/2017	0	0	0
2015349730	776	rural/clinic	No	20/07/2017	0	0	0
2015338467	776	rural/clinic	No	20/07/2017	0	0	0
2014338562	776	rural/clinic	No	20/07/2017	0	0	0
2015349729	587	rural/clinic	No	20/07/2017	0	0	0
2015349727	587	rural/clinic	No	20/07/2017	0	0	0
2015418867	587	rural/clinic	No	20/07/2017	0	0	0
2015338468	587	rural/clinic	No	20/07/2017	0	0	0
2012331327	587	rural/clinic	No	20/07/2017	0	0	0
2015402650	587	rural/clinic	No	20/07/2017	0	0	0
2015379329	932	rural/clinic	No	20/07/2017	0	0	0
2015326974	932	rural/clinic	No	20/07/2017	0	0	0
2015326975	932	rural/clinic	No	20/07/2017	0	0	0
2012388274	932	rural/clinic	No	20/07/2017	0	0	0
2014301471	932	rural/clinic	No	20/07/2017	0	0	0
2015339028	932	rural/clinic	No	20/07/2017	0	0	0
2015339304	932	rural/clinic	No	20/07/2017	0	0	0
2014301472	932	rural/clinic	No	20/07/2017	0	0	0
2015339305	932	rural/clinic	No	20/07/2017	0	0	0
2015412781	932	rural/clinic	No	20/07/2017	0	0	0
2015294182	932	rural/clinic	No	20/07/2017	0	0	0
2015412780	821	rural/clinic	No	20/07/2017	0	0	0
2012369973	821	rural/clinic	No	20/07/2017	0	0	0
2015294181	821	rural/clinic	No	20/07/2017	0	0	0
2015297290	821	rural/clinic	No	20/07/2017	0	0	0
2015294183	821	rural/clinic	No	20/07/2017	0	0	0
2015297289	821	rural/clinic	No	20/07/2017	0	0	0
2014340279	821	rural/clinic	No	20/07/2017	0	0	0
2015297288	821	rural/clinic	No	20/07/2017	0	0	0
2015339029	821	rural/clinic	No	20/07/2017	0	0	0
2012369976	821	rural/clinic	No	20/07/2017	0	0	0
2015297287	821	rural/clinic	No	20/07/2017	0	0	0
2015349732	821	rural/clinic	No	20/07/2017	0	0	0
2015349731	821	rural/clinic	No	20/07/2017	0	0	0
2015349726	821	rural/clinic	No	20/07/2017	0	0	0
2015419059	821	rural/clinic	No	20/07/2017	0	0	0
2012251550	821	rural/clinic	No	20/07/2017	0	0	0
2012388273	821	rural/clinic	No	20/07/2017	0	0	0
2014317641	673	rural/clinic	No	20/07/2017	0	0	0
2012369974	673	rural/clinic	No	20/07/2017	0	0	0
2015302151	673	rural/clinic	No	20/07/2017	0	0	0
2014317642	673	rural/clinic	No	20/07/2017	0	0	0

2014317643	673	rural/clinic	No	20/07/2017	0	0	0
2015414562	673	rural/clinic	No	20/07/2017	0	0	0
2015419057	673	rural/clinic	No	20/07/2017	0	0	0
2014317644	673	rural/clinic	No	20/07/2017	0	0	0
2015294180	673	rural/clinic	No	20/07/2017	0	0	0
2014317645	673	rural/clinic	No	20/07/2017	0	0	0
2012294623	673	rural/clinic	No	20/07/2017	0	0	0
2014317646	673	rural/clinic	No	20/07/2017	0	0	0
2012275211	673	rural/clinic	No	20/07/2017	0	0	0
2014369048	673	rural/clinic	No	20/07/2017	0	0	0
2014317647	673	rural/clinic	No	20/07/2017	0	0	0
2012275210	673	rural/clinic	No	20/07/2017	0	0	0
2013266124	673	rural/clinic	No	20/07/2017	0	0	0
2015289191	673	rural/clinic	No	20/07/2017	0	0	0
2012275212	673	rural/clinic	No	20/07/2017	0	0	0
2010053915	673	rural/clinic	No	20/07/2017	0	0	0
2015289192	673	rural/clinic	No	20/07/2017	0	0	0
2015339551	673	rural/clinic	No	20/07/2017	0	0	0
2010053916	673	rural/clinic	No	20/07/2017	0	0	0
2015289193	484	rural/clinic	No	20/07/2017	0	0	0
2014357548	484	rural/clinic	No	20/07/2017	0	0	0
2010053914	484	rural/clinic	No	20/07/2017	0	0	0
2015355922	484	rural/clinic	No	20/07/2017	0	0	0
2014357550	484	rural/clinic	No	20/07/2017	0	0	0
2011221338	484	rural/clinic	No	20/07/2017	0	0	0
2015406011	484	rural/clinic	No	20/07/2017	0	0	0
2015386751	484	rural/clinic	No	20/07/2017	0	0	0
2011221337	484	rural/clinic	No	20/07/2017	0	0	0
2015406009	484	rural/clinic	No	20/07/2017	0	0	0
2015386753	484	rural/clinic	No	20/07/2017	0	0	0
2015406010	484	rural/clinic	No	20/07/2017	0	0	0
2011221339	431	rural/clinic	No	20/07/2017	0	0	0
2015386752	431	rural/clinic	No	20/07/2017	0	0	0
2015406008	431	rural/clinic	No	20/07/2017	0	0	0
2011133918	431	rural/clinic	No	20/07/2017	0	0	0
2011155237	431	rural/clinic	No	20/07/2017	0	0	0
2015406013	431	rural/clinic	No	20/07/2017	0	0	0
2011133917	431	rural/clinic	No	20/07/2017	0	0	0
2015361168	431	rural/clinic	No	20/07/2017	0	0	0
2015340149	431	rural/clinic	No	20/07/2017	0	0	0
2015361169	431	rural/clinic	No	20/07/2017	0	0	0
2014302522	431	rural/clinic	No	20/07/2017	0	0	0
2015289205	431	rural/clinic	No	20/07/2017	0	0	0
2015361170	431	rural/clinic	No	20/07/2017	0	0	0
2015289204	431	rural/clinic	No	20/07/2017	0	0	0
2015385543	431	rural/clinic	No	20/07/2017	0	0	0
2015289203	431	rural/clinic	No	20/07/2017	0	0	0
2015404747	431	rural/clinic	No	20/07/2017	0	0	0
2013272763	431	rural/clinic	No	20/07/2017	0	0	0
2015361171	431	rural/clinic	No	20/07/2017	0	0	0
2015289202	431	rural/clinic	No	20/07/2017	0	0	0
2015360205	431	rural/clinic	No	20/07/2017	0	0	0
2015361172	431	rural/clinic	No	20/07/2017	0	0	0
2015361173	431	rural/clinic	No	20/07/2017	0	0	0
2015347401	431	rural/clinic	No	20/07/2017	0	0	0
2015361174	431	rural/clinic	No	20/07/2017	0	0	0
2011143695	431	rural/clinic	No	20/07/2017	0	0	0
2015286333	732	rural/clinic	No	20/07/2017	0	0	0
2015360204	732	rural/clinic	No	20/07/2017	0	0	0
2015361175	732	rural/clinic	No	20/07/2017	0	0	0
2015286334	732	rural/clinic	No	20/07/2017	0	0	0
2013247025	732	rural/clinic	No	20/07/2017	0	0	0
2013255745	732	rural/clinic	No	20/07/2017	0	0	0
2011141108	732	rural/clinic	No	20/07/2017	0	0	0
2014362836	732	rural/clinic	No	20/07/2017	0	0	0
2015338873	732	rural/clinic	No	20/07/2017	0	0	0
2015404748	732	rural/clinic	No	20/07/2017	0	0	0
2015364528	732	rural/clinic	No	20/07/2017	0	0	0
2015402578	883	district/faith-based	No	20/07/2017	1	0	0
2015404749	883	district/faith-based	No	20/07/2017	1	0	0
2015364527	883	district/faith-based	No	20/07/2017	1	0	0
2015404750	883	district/faith-based	No	20/07/2017	1	0	0
2015404651	883	district/faith-based	No	20/07/2017	1	0	0
2014321426	883	district/faith-based	No	20/07/2017	1	0	0
2015340712	883	district/faith-based	No	20/07/2017	1	0	0
2015402579	883	district/faith-based	No	20/07/2017	1	0	0
2014328764	883	district/faith-based	No	20/07/2017	1	0	0
2015340231	883	district/faith-based	No	20/07/2017	1	0	0
2015373753	883	district/faith-based	No	20/07/2017	1	0	0
2014321427	883	district/faith-based	No	20/07/2017	1	0	0
2015402580	883	district/faith-based	No	20/07/2017	1	0	0
2015373754	883	district/faith-based	No	20/07/2017	1	0	0

2015373755	883	district/faith-based No	20/07/2017	1	0	0
2015320899	883	district/faith-based No	20/07/2017	1	0	0
2015402581	883	district/faith-based No	20/07/2017	1	0	0
2015373756	883	district/faith-based No	20/07/2017	1	0	0
2015345078	883	district/faith-based No	20/07/2017	1	0	0
2015373757	883	district/faith-based No	20/07/2017	1	0	0
2015373758	883	district/faith-based No	20/07/2017	1	0	0
2014378559	883	district/faith-based No	19/07/2017	1	0	0
2015373759	883	district/faith-based No	20/07/2017	1	0	0
2014335529	883	district/faith-based No	19/07/2017	1	0	0
2015382569	883	district/faith-based No	20/07/2017	1	0	0
2015345075	883	district/faith-based No	20/07/2017	1	0	0
2015332108	883	district/faith-based No	19/07/2017	1	0	0
2015382571	883	district/faith-based No	20/07/2017	1	0	0
2014378560	883	district/faith-based No	19/07/2017	1	0	0
2015345076	883	district/faith-based No	20/07/2017	1	0	0
2015382570	883	district/faith-based No	20/07/2017	1	0	0
2014378561	883	district/faith-based No	19/07/2017	1	0	0
2015382568	883	district/faith-based No	20/07/2017	1	0	0
2015345079	883	district/faith-based No	20/07/2017	1	0	0
2014378562	883	district/faith-based No	19/07/2017	1	0	0
2015382574	883	district/faith-based No	20/07/2017	1	0	0
2015352651	883	district/faith-based No	19/07/2017	1	0	0
2015332030	883	district/faith-based No	19/07/2017	1	0	0
2015382573	883	district/faith-based No	20/07/2017	1	0	0
2015352745	883	district/faith-based No	19/07/2017	1	0	0
2015305090	883	district/faith-based No	20/07/2017	1	0	0
2015382572	883	district/faith-based No	20/07/2017	1	0	0
2014334187	883	district/faith-based No	20/07/2017	1	0	0
2015414396	883	district/faith-based No	20/07/2017	1	0	0
2014377416	883	district/faith-based No	20/07/2017	1	0	0
2012388280	883	district/faith-based No	20/07/2017	1	0	0
2012388279	883	district/faith-based No	20/07/2017	1	0	0
2015382576	883	district/faith-based No	20/07/2017	1	0	0
2015382575	883	district/faith-based No	20/07/2017	1	0	0
2012388278	883	district/faith-based No	20/07/2017	1	0	0
2015369081	883	district/faith-based No	20/07/2017	1	0	0
2012388277	883	district/faith-based No	20/07/2017	1	0	0
2015369080	883	district/faith-based No	20/07/2017	1	0	0
2012388276	883	district/faith-based No	20/07/2017	1	0	0
2015369079	883	district/faith-based No	20/07/2017	1	0	0
2012388275	883	district/faith-based No	20/07/2017	1	0	0
2015369077	883	district/faith-based No	20/07/2017	1	0	0
2015369078	883	district/faith-based No	20/07/2017	1	0	0
2015404151	883	district/faith-based No	20/07/2017	1	0	0
2015319300	883	district/faith-based No	20/07/2017	1	0	0
2015352750	883	district/faith-based No	20/07/2017	1	0	0
2015352749	883	district/faith-based No	20/07/2017	1	0	0
2015319298	883	district/faith-based No	20/07/2017	1	0	0
2015319299	883	district/faith-based No	20/07/2017	1	0	0
2015352748	883	district/faith-based No	20/07/2017	1	0	0
2015319295	883	district/faith-based No	20/07/2017	1	0	0
2015352747	883	district/faith-based No	20/07/2017	1	0	0
2015319294	883	district/faith-based No	20/07/2017	1	0	0
2014372728	883	district/faith-based No	20/07/2017	1	0	0
2015340364	883	district/faith-based No	20/07/2017	1	0	0
2015328085	883	district/faith-based No	20/07/2017	1	0	0
2015352743	883	district/faith-based No	20/07/2017	1	0	0
2015328093	883	district/faith-based No	20/07/2017	1	0	0
2015328090	883	district/faith-based No	20/07/2017	1	0	0
2015328089	883	district/faith-based No	20/07/2017	1	0	0
2015328086	883	district/faith-based No	20/07/2017	1	0	0
2015328084	883	district/faith-based No	20/07/2017	1	0	0
2015328091	883	district/faith-based No	20/07/2017	1	0	0
2015352744	883	district/faith-based No	20/07/2017	1	0	0
2015328087	883	district/faith-based No	20/07/2017	1	0	0
2015328088	883	district/faith-based No	20/07/2017	1	0	0
2015328092	883	district/faith-based No	20/07/2017	1	0	0
2015324250	883	district/faith-based No	19/07/2017	1	0	0
2015324264	883	district/faith-based No	20/07/2017	1	0	0
2015404252	883	district/faith-based No	20/07/2017	1	0	0
2015332915	883	district/faith-based No	20/07/2017	1	0	0
2015406326	883	district/faith-based No	20/07/2017	1	0	0
2015406322	883	district/faith-based No	20/07/2017	1	0	0
2015406324	883	district/faith-based No	20/07/2017	1	0	0
2015406328	883	district/faith-based No	20/07/2017	1	0	0
2015352652	883	district/faith-based No	20/07/2017	1	0	0
2015406321	883	district/faith-based No	20/07/2017	1	0	0
2015406327	883	district/faith-based No	20/07/2017	1	0	0
2015324249	883	district/faith-based No	20/07/2017	1	0	0
2015406323	883	district/faith-based No	20/07/2017	1	0	0
2015406329	883	district/faith-based No	20/07/2017	1	0	0

2014335530	883	district/faith-based	No	20/07/2017	1	0	0
2015347402	883	district/faith-based	No	20/07/2017	1	0	0
2015347403	883	district/faith-based	No	20/07/2017	1	0	0
2015402582	883	district/faith-based	No	20/07/2017	1	0	0
2015372815	883	district/faith-based	No	20/07/2017	1	0	0
2014313669	883	district/faith-based	No	20/07/2017	1	0	0
2015402583	883	district/faith-based	No	20/07/2017	1	0	0
2015378735	883	district/faith-based	No	20/07/2017	1	0	0
2012365648	883	district/faith-based	No	20/07/2017	1	0	0
2015402584	883	district/faith-based	No	20/07/2017	1	0	0
2015372817	883	district/faith-based	No	20/07/2017	1	0	0
2015402585	883	district/faith-based	No	20/07/2017	1	0	0
2015372816	883	district/faith-based	No	20/07/2017	1	0	0
2015402586	883	district/faith-based	No	20/07/2017	1	0	0
2015402587	883	district/faith-based	No	20/07/2017	1	0	0
2015402588	883	district/faith-based	No	20/07/2017	1	0	0
2015402589	883	district/faith-based	No	20/07/2017	1	0	0
2015402590	883	district/faith-based	No	20/07/2017	1	0	0
2015402591	883	district/faith-based	No	20/07/2017	1	0	0
2015402593	883	district/faith-based	No	20/07/2017	1	0	0
2015386748	883	district/faith-based	No	20/07/2017	1	0	0
2014334186	883	district/faith-based	No	20/07/2017	1	0	0
2015358302	883	district/faith-based	No	20/07/2017	1	0	0
2015358301	883	district/faith-based	No	20/07/2017	1	0	0
2014334185	883	district/faith-based	No	20/07/2017	1	0	0
2015386299	883	district/faith-based	No	20/07/2017	1	0	0
2015403203	883	district/faith-based	No	20/07/2017	1	0	0
2015386300	883	district/faith-based	No	20/07/2017	1	0	0
2015403202	883	district/faith-based	No	20/07/2017	1	0	0
2015349733	883	district/faith-based	No	20/07/2017	1	0	0
2015386296	883	district/faith-based	No	20/07/2017	1	0	0
2015386298	883	district/faith-based	No	20/07/2017	1	0	0
2015319296	883	district/faith-based	No	20/07/2017	1	0	0
2015319297	883	district/faith-based	No	20/07/2017	1	0	0
2015386297	883	district/faith-based	No	20/07/2017	1	0	0
2013253030	883	district/faith-based	No	20/07/2017	1	0	0
2014350962	883	district/faith-based	No	20/07/2017	1	0	0
2014347993	883	district/faith-based	No	20/07/2017	1	0	0
2014347992	883	district/faith-based	No	20/07/2017	1	0	0
2014363101	883	district/faith-based	No	20/07/2017	1	0	0
2015405803	883	district/faith-based	No	20/07/2017	1	0	0
2014385695	883	district/faith-based	No	20/07/2017	1	0	0
2015305813	883	district/faith-based	No	20/07/2017	1	0	0
2015305812	883	district/faith-based	No	20/07/2017	1	0	0
2015332478	883	district/faith-based	No	20/07/2017	1	0	0
2015360528	883	district/faith-based	No	20/07/2017	1	0	0
2015360529	883	district/faith-based	No	20/07/2017	1	0	0
2012365989	883	district/faith-based	No	20/07/2017	1	0	0
2014312411	883	district/faith-based	No	20/07/2017	1	0	0
2012365988	883	district/faith-based	No	20/07/2017	1	0	0
2015332476	883	district/faith-based	No	20/07/2017	1	0	0
2015360527	883	district/faith-based	No	20/07/2017	1	0	0
2012294478	883	district/faith-based	No	20/07/2017	1	0	0
2011204485	883	district/faith-based	No	20/07/2017	1	0	0
2011204484	883	district/faith-based	No	20/07/2017	1	0	0
2015332477	883	district/faith-based	No	20/07/2017	1	0	0
2015418868	883	district/faith-based	No	20/07/2017	1	0	0
2015418869	883	district/faith-based	No	20/07/2017	1	0	0
2014372277	883	district/faith-based	No	20/07/2017	1	0	0
2014372446	883	district/faith-based	No	20/07/2017	1	0	0
2014372447	883	district/faith-based	No	20/07/2017	1	0	0
2014357853	883	district/faith-based	No	18/07/2017	1	0	0
2014357853	883	district/faith-based	No	18/07/2017	1	0	0
2014327688	883	district/faith-based	No	18/07/2017	1	0	0
2014327688	883	district/faith-based	No	18/07/2017	1	0	0
2011216414	883	district/faith-based	No	18/07/2017	1	0	0
2011216414	883	district/faith-based	No	18/07/2017	1	0	0
2015405918	883	district/faith-based	No	18/07/2017	1	0	0
2012271401/D	883	district/faith-based	No	18/07/2017	1	0	0
2012271401/D	883	district/faith-based	No	18/07/2017	1	0	0
2014306044	883	district/faith-based	No	18/07/2017	1	0	0
2014306044	883	district/faith-based	No	18/07/2017	1	0	0
2015342320	883	district/faith-based	No	18/07/2017	1	0	0
2015342320	883	district/faith-based	No	18/07/2017	1	0	0
2015369000	883	district/faith-based	No	18/07/2017	1	0	0
2015369000	883	district/faith-based	No	18/07/2017	1	0	0
2015340226	883	district/faith-based	No	18/07/2017	1	0	0
2015340226	883	district/faith-based	No	18/07/2017	1	0	0
2015337996	883	district/faith-based	No	18/07/2017	1	0	0
2015337996	883	district/faith-based	No	18/07/2017	1	0	0
2015326971	883	district/faith-based	No	18/07/2017	1	0	0
2015326971	883	district/faith-based	No	18/07/2017	1	0	0

2015303483	883	district/faith-based	No	18/07/2017	1	0	0
2015303483	883	district/faith-based	No	18/07/2017	1	0	0
2013247137	883	district/faith-based	No	20/07/2017	1	0	0
2014342538	883	district/faith-based	No	18/07/2017	1	0	0
2014342538	883	district/faith-based	No	18/07/2017	1	0	0
2015297290	883	district/faith-based	No	20/07/2017	1	0	0
2015297290	883	district/faith-based	No	20/07/2017	1	0	0
2015297288	883	district/faith-based	No	20/07/2017	1	0	0
2015297288	883	district/faith-based	No	20/07/2017	1	0	0
2015332030	883	district/faith-based	No	19/07/2017	1	0	0
2015332030	883	district/faith-based	No	19/07/2017	1	0	0
2015352745	883	district/faith-based	No	19/07/2017	1	0	0
2015352745	883	district/faith-based	No	19/07/2017	1	0	0
2014377416	883	district/faith-based	No	20/07/2017	1	0	0
2014377416	883	district/faith-based	No	20/07/2017	1	0	0
2014378560	898	rural/clinic	No	19/07/2017	0	0	0
2014378560	898	rural/clinic	No	19/07/2017	0	0	0
2010099933	898	rural/clinic	No	20/07/2017	0	0	0
2011191295	898	rural/clinic	No	20/07/2017	0	0	0
2011200280	898	rural/clinic	No	20/07/2017	0	0	0
2014290241	898	rural/clinic	No	20/07/2017	0	0	0
2015357809	898	rural/clinic	No	20/07/2017	0	0	0
2015357810	898	rural/clinic	No	20/07/2017	0	0	0
2012313139	898	rural/clinic	No	20/07/2017	0	0	0
2010099934	898	rural/clinic	No	20/07/2017	0	0	0
2015340230	898	rural/clinic	No	20/07/2017	0	0	0
2015373869	898	rural/clinic	No	20/07/2017	0	0	0
2015373870	898	rural/clinic	No	20/07/2017	0	0	0
2015373871	898	rural/clinic	No	21/07/2017	0	0	0
2015373872	898	rural/clinic	No	21/07/2017	0	0	0
2014319525	898	rural/clinic	No	21/07/2017	0	0	0
2014319526	898	rural/clinic	No	21/07/2017	0	0	0
2015286711	898	rural/clinic	No	21/07/2017	0	0	0
2015286712	898	rural/clinic	No	20/07/2017	0	0	0
2015286321	898	rural/clinic	No	21/07/2017	0	0	0
2015334803	898	rural/clinic	No	18/07/2017	0	0	0
2010050499	898	rural/clinic	No	24/07/2017	0	0	0
2015397336	898	rural/clinic	No	24/07/2017	0	0	0
2015397337	898	rural/clinic	No	24/07/2017	0	0	0
2015397338	898	rural/clinic	No	24/07/2017	0	0	0
2015397339	898	rural/clinic	No	24/07/2017	0	0	0
2015397340	898	rural/clinic	No	24/07/2017	0	0	0
2012247412	898	rural/clinic	No	24/07/2017	0	0	0
2012247348	898	rural/clinic	No	24/07/2017	0	0	0
2013251318	898	rural/clinic	No	24/07/2017	0	0	0
2015391917	898	rural/clinic	No	24/07/2017	0	0	0
2015328092	898	rural/clinic	No	20/07/2017	0	0	0
2015328092	898	rural/clinic	No	20/07/2017	0	0	0
2014317643	898	rural/clinic	No	20/07/2017	0	0	0
2014317643	898	rural/clinic	No	20/07/2017	0	0	0
2010053916	898	rural/clinic	No	20/07/2017	0	0	0
2010053916	898	rural/clinic	No	20/07/2017	0	0	0
2011133918	898	rural/clinic	No	20/07/2017	0	0	0
2011133918	898	rural/clinic	No	20/07/2017	0	0	0
2015347401	898	rural/clinic	No	20/07/2017	0	0	0
2015347401	898	rural/clinic	No	20/07/2017	0	0	0
2014328764	898	rural/clinic	No	20/07/2017	0	0	0
2014328764	898	rural/clinic	No	20/07/2017	0	0	0
2015379329	898	rural/clinic	No	20/07/2017	0	0	0
2015379329	898	rural/clinic	No	20/07/2017	0	0	0
2012369976	898	rural/clinic	No	20/07/2017	0	0	0
2015382572	898	rural/clinic	No	20/07/2017	0	0	0
2015382572	898	rural/clinic	No	20/07/2017	0	0	0
2012388267	898	rural/clinic	No	13/07/2017	0	0	0
2015297162	898	rural/clinic	No	18/07/2017	0	0	0
2015297162	898	rural/clinic	No	18/07/2017	0	0	0
2015326445	898	rural/clinic	No	18/07/2017	0	0	0
2015326445	898	rural/clinic	No	18/07/2017	0	0	0
2015326447	898	rural/clinic	No	18/07/2017	0	0	0
2015403180	898	rural/clinic	No	25/07/2017	0	0	0
2014322243	898	rural/clinic	No	25/07/2017	0	0	0
2014322244	898	rural/clinic	No	25/07/2017	0	0	0
2014358226	898	rural/clinic	No	25/07/2017	0	0	0
2013254145	898	rural/clinic	No	25/07/2017	0	0	0
2014322248	898	rural/clinic	No	25/07/2017	0	0	0
2014322239	898	rural/clinic	No	25/07/2017	0	0	0
2014322240	898	rural/clinic	No	25/07/2017	0	0	0
2014322241	898	rural/clinic	No	25/07/2017	0	0	0
2014322242	898	rural/clinic	No	25/07/2017	0	0	0
2015393005	898	rural/clinic	No	25/07/2017	0	0	0
2011143087	898	rural/clinic	No	25/07/2017	0	0	0
2015374460	898	rural/clinic	No	25/07/2017	0	0	0

2015363082	898	rural/clinic	No	25/07/2017	0	0	0
2014365398	898	rural/clinic	No	25/07/2017	0	0	0
2015374459	898	rural/clinic	No	25/07/2017	0	0	0
2014365397	898	rural/clinic	No	25/07/2017	0	0	0
2014346738	898	rural/clinic	No	25/07/2017	0	0	0
2015403309	898	rural/clinic	No	25/07/2017	0	0	0
2014327526	898	rural/clinic	No	25/07/2017	0	0	0
2011135051	898	rural/clinic	No	25/07/2017	0	0	0
2014327525	898	rural/clinic	No	25/07/2017	0	0	0
2011136143	898	rural/clinic	No	25/07/2017	0	0	0
2015360741	898	rural/clinic	No	25/07/2017	0	0	0
2011135052	898	rural/clinic	No	25/07/2017	0	0	0
2015360740	898	rural/clinic	No	25/07/2017	0	0	0
2015360739	898	rural/clinic	No	25/07/2017	0	0	0
2011135054	898	rural/clinic	No	25/07/2017	0	0	0
2011142499	898	rural/clinic	No	25/07/2017	0	0	0
2014319432	898	rural/clinic	No	25/07/2017	0	0	0
2013256281	898	rural/clinic	No	25/07/2017	0	0	0
2011142498	898	rural/clinic	No	25/07/2017	0	0	0
2014338513	898	rural/clinic	No	25/07/2017	0	0	0
2015367226	898	rural/clinic	No	25/07/2017	0	0	0
2011142500	898	rural/clinic	No	25/07/2017	0	0	0
2014365641	898	rural/clinic	No	25/07/2017	0	0	0
2011144834	898	rural/clinic	No	25/07/2017	0	0	0
2015362299	898	rural/clinic	No	25/07/2017	0	0	0
2013264395	898	rural/clinic	No	25/07/2017	0	0	0
2015359619	898	rural/clinic	No	25/07/2017	0	0	0
2015331183	898	rural/clinic	No	25/07/2017	0	0	0
2014287988	898	rural/clinic	No	25/07/2017	0	0	0
2015363761	898	rural/clinic	No	25/07/2017	0	0	0
2015331103	898	rural/clinic	No	25/07/2017	0	0	0
2015349067	898	rural/clinic	No	25/07/2017	0	0	0
2015333857	898	rural/clinic	No	25/07/2017	0	0	0
2015359620	898	rural/clinic	No	25/07/2017	0	0	0
2014358025	898	rural/clinic	No	25/07/2017	0	0	0
2017314573	898	rural/clinic	No	25/07/2017	0	0	0
2014358026	898	rural/clinic	No	25/07/2017	0	0	0
2015367224	898	rural/clinic	No	25/07/2017	0	0	0
2012291493	898	rural/clinic	No	25/07/2017	0	0	0
2015409027	898	rural/clinic	No	25/07/2017	0	0	0
2017405629	898	rural/clinic	No	25/07/2017	0	0	0
2014358027	898	rural/clinic	No	25/07/2017	0	0	0
2011114847	898	rural/clinic	No	25/07/2017	0	0	0
2012369032	898	rural/clinic	No	25/07/2017	0	0	0
2014357329	898	rural/clinic	No	25/07/2017	0	0	0
2012369033	898	rural/clinic	No	25/07/2017	0	0	0
2011114846	898	rural/clinic	No	25/07/2017	0	0	0
2015287387	898	rural/clinic	No	25/07/2017	0	0	0
2015337526	898	rural/clinic	No	25/07/2017	0	0	0
2014336829	898	rural/clinic	No	25/07/2017	0	0	0
2014368691	898	rural/clinic	No	25/07/2017	0	0	0
2015338718	898	rural/clinic	No	25/07/2017	0	0	0
2015363762	898	rural/clinic	No	25/07/2017	0	0	0
2012358299	898	rural/clinic	No	25/07/2017	0	0	0
2015332439	898	rural/clinic	No	25/07/2017	0	0	0
2015363763	898	rural/clinic	No	25/07/2017	0	0	0
2015347204	898	rural/clinic	No	25/07/2017	0	0	0
2015347205	898	rural/clinic	No	25/07/2017	0	0	0
2015367256	898	rural/clinic	No	25/07/2017	0	0	0
2015315001	898	rural/clinic	No	25/07/2017	0	0	0
2015332438	898	rural/clinic	No	25/07/2017	0	0	0
2015367223	898	rural/clinic	No	25/07/2017	0	0	0
2012358297	898	rural/clinic	No	25/07/2017	0	0	0
2015332437	898	rural/clinic	No	25/07/2017	0	0	0
2015303124	898	rural/clinic	No	25/07/2017	0	0	0
2015352374	898	rural/clinic	No	25/07/2017	0	0	0
2015352375	898	rural/clinic	No	25/07/2017	0	0	0
2015367225	898	rural/clinic	No	25/07/2017	0	0	0
2012295997	898	rural/clinic	No	25/07/2017	0	0	0
2015385955	898	rural/clinic	No	25/07/2017	0	0	0
2012304338	898	rural/clinic	No	25/07/2017	0	0	0
2012295809	898	rural/clinic	No	25/07/2017	0	0	0
2013254811	898	rural/clinic	No	25/07/2017	0	0	0
2012295810	898	rural/clinic	No	25/07/2017	0	0	0
2012304336	898	rural/clinic	No	25/07/2017	0	0	0
2012295811	898	rural/clinic	No	25/07/2017	0	0	0
2012304337	898	rural/clinic	No	25/07/2017	0	0	0
2015355527	898	rural/clinic	No	25/07/2017	0	0	0
2014326640	898	rural/clinic	No	25/07/2017	0	0	0
2014288426	898	rural/clinic	No	25/07/2017	0	0	0
2012359168	898	rural/clinic	No	25/07/2017	0	0	0
2014326641	898	rural/clinic	No	25/07/2017	0	0	0

2015337525	898	rural/clinic	No	25/07/2017	0	0	0
2014326642	898	rural/clinic	No	25/07/2017	0	0	0
2015345080	898	rural/clinic	No	25/07/2017	0	0	0
2015403182	898	rural/clinic	No	25/07/2017	0	0	0
2015305091	898	rural/clinic	No	25/07/2017	0	0	0
2012358296	898	rural/clinic	No	25/07/2017	0	0	0
2015405728	898	rural/clinic	No	25/07/2017	0	0	0
2015305092	898	rural/clinic	No	25/07/2017	0	0	0
2015405729	898	rural/clinic	No	25/07/2017	0	0	0
2015405727	898	rural/clinic	No	25/07/2017	0	0	0
2015355526	898	rural/clinic	No	25/07/2017	0	0	0
2015377753	898	rural/clinic	No	25/07/2017	0	0	0
2015362298	898	rural/clinic	No	25/07/2017	0	0	0
2012323779	898	rural/clinic	No	25/07/2017	0	0	0
2015377754	898	rural/clinic	No	25/07/2017	0	0	0
2011135954	898	rural/clinic	No	25/07/2017	0	0	0
2015413928	898	rural/clinic	No	25/07/2017	0	0	0
2011135956	898	rural/clinic	No	25/07/2017	0	0	0
2015362297	898	rural/clinic	No	25/07/2017	0	0	0
2015413929	898	rural/clinic	No	25/07/2017	0	0	0
2011135955	898	rural/clinic	No	25/07/2017	0	0	0
2015362296	898	rural/clinic	No	25/07/2017	0	0	0
2015326977	898	rural/clinic	No	25/07/2017	0	0	0
2015362295	898	rural/clinic	No	25/07/2017	0	0	0
2014299919	898	rural/clinic	No	25/07/2017	0	0	0
2012305825	898	rural/clinic	No	25/07/2017	0	0	0
2015362294	898	rural/clinic	No	25/07/2017	0	0	0
2014299920	898	rural/clinic	No	25/07/2017	0	0	0
2015362300	898	rural/clinic	No	25/07/2017	0	0	0
2012305824	898	rural/clinic	No	25/07/2017	0	0	0
2011135957	898	rural/clinic	No	25/07/2017	0	0	0
2015372939	898	rural/clinic	No	25/07/2017	0	0	0
2012253446	898	rural/clinic	No	25/07/2017	0	0	0
2015337628	898	rural/clinic	No	25/07/2017	0	0	0
2015372940	898	rural/clinic	No	25/07/2017	0	0	0
2011144832	898	rural/clinic	No	25/07/2017	0	0	0
2015337630	898	rural/clinic	No	25/07/2017	0	0	0
2012284028	898	rural/clinic	No	25/07/2017	0	0	0
2015337629	898	rural/clinic	No	25/07/2017	0	0	0
2015336651	898	rural/clinic	No	25/07/2017	0	0	0
2011144831	898	rural/clinic	No	25/07/2017	0	0	0
2015385860	898	rural/clinic	No	25/07/2017	0	0	0
2015320900	898	rural/clinic	No	25/07/2017	0	0	0
2014289547	898	rural/clinic	No	25/07/2017	0	0	0
2015337524	898	rural/clinic	No	25/07/2017	0	0	0
2014289548	898	rural/clinic	No	25/07/2017	0	0	0
2011135053	898	rural/clinic	No	25/07/2017	0	0	0
2011144826	898	rural/clinic	No	25/07/2017	0	0	0
2015337375	898	rural/clinic	No	25/07/2017	0	0	0
2014289549	898	rural/clinic	No	25/07/2017	0	0	0
2011135059	898	rural/clinic	No	25/07/2017	0	0	0
2015372851	898	rural/clinic	No	25/07/2017	0	0	0
2015337521	898	rural/clinic	No	25/07/2017	0	0	0
2014289550	898	rural/clinic	No	25/07/2017	0	0	0
2011135055	898	rural/clinic	No	25/07/2017	0	0	0
2015324484	898	rural/clinic	No	25/07/2017	0	0	0
2011144830	898	rural/clinic	No	25/07/2017	0	0	0
2011135058	898	rural/clinic	No	25/07/2017	0	0	0
2015385859	898	rural/clinic	No	25/07/2017	0	0	0
2015372936	898	rural/clinic	No	25/07/2017	0	0	0
2015372938	898	rural/clinic	No	25/07/2017	0	0	0
2011135060	898	rural/clinic	No	25/07/2017	0	0	0
2015372937	898	rural/clinic	No	25/07/2017	0	0	0
2015369939	32	rural/clinic	No	25/07/2017	0	0	0
2012344863	32	rural/clinic	No	25/07/2017	0	0	0
2015376213	32	rural/clinic	No	25/07/2017	0	0	0
2014367628	32	rural/clinic	No	25/07/2017	0	0	0
2014375517	32	rural/clinic	No	25/07/2017	0	0	0
2015337309	32	rural/clinic	No	25/07/2017	0	0	0
2015369938	32	rural/clinic	No	25/07/2017	0	0	0
2015355525	32	rural/clinic	No	25/07/2017	0	0	0
2015368896	32	rural/clinic	No	25/07/2017	0	0	0
2012361949	32	rural/clinic	No	25/07/2017	0	0	0
2014291625	32	rural/clinic	No	25/07/2017	0	0	0
2011135057	32	rural/clinic	No	25/07/2017	0	0	0
2014298242	32	rural/clinic	No	25/07/2017	0	0	0
2015376212	32	rural/clinic	No	25/07/2017	0	0	0
2014374038	32	rural/clinic	No	25/07/2017	0	0	0
2015372302	32	rural/clinic	No	25/07/2017	0	0	0
2012335667	32	rural/clinic	No	25/07/2017	0	0	0
2014374037	32	rural/clinic	No	25/07/2017	0	0	0
2015409021	32	rural/clinic	No	25/07/2017	0	0	0

2014374039	32	rural/clinic	No	25/07/2017	0	0	0
2015407071	32	rural/clinic	No	25/07/2017	0	0	0
2012379903	32	rural/clinic	No	25/07/2017	0	0	0
2012291372	32	rural/clinic	No	25/07/2017	0	0	0
2015347201	32	rural/clinic	No	25/07/2017	0	0	0
2015407072	32	rural/clinic	No	25/07/2017	0	0	0
2015347202	32	rural/clinic	No	25/07/2017	0	0	0
2012291373	32	rural/clinic	No	25/07/2017	0	0	0
2015289338	32	rural/clinic	No	25/07/2017	0	0	0
2015363467	32	rural/clinic	No	25/07/2017	0	0	0
2014342544	32	rural/clinic	No	25/07/2017	0	0	0
2014342543	32	rural/clinic	No	25/07/2017	0	0	0
2014342545	32	rural/clinic	No	25/07/2017	0	0	0
2015347203	32	rural/clinic	No	25/07/2017	0	0	0
2014342542	32	rural/clinic	No	25/07/2017	0	0	0
2015407073	32	rural/clinic	No	25/07/2017	0	0	0
2011144835	32	rural/clinic	No	25/07/2017	0	0	0
2014371596	32	rural/clinic	No	25/07/2017	0	0	0
2014371595	32	rural/clinic	No	25/07/2017	0	0	0
2014344539	32	rural/clinic	No	25/07/2017	0	0	0
2014371594	32	rural/clinic	No	25/07/2017	0	0	0
2014371593	32	rural/clinic	No	25/07/2017	0	0	0
2015372852	32	rural/clinic	No	25/07/2017	0	0	0
2014332715	32	rural/clinic	No	25/07/2017	0	0	0
2011112304	32	rural/clinic	No	25/07/2017	0	0	0
2011112305	32	rural/clinic	No	25/07/2017	0	0	0
2010099151	32	rural/clinic	No	25/07/2017	0	0	0
2015313711	32	rural/clinic	No	25/07/2017	0	0	0
2012299684	32	rural/clinic	No	25/07/2017	0	0	0
2012299683	32	rural/clinic	No	25/07/2017	0	0	0
2012299682	32	rural/clinic	No	25/07/2017	0	0	0
2014322238	32	rural/clinic	No	25/07/2017	0	0	0
2014322245	32	rural/clinic	No	25/07/2017	0	0	0
2014322247	32	rural/clinic	No	25/07/2017	0	0	0
2015419058	32	rural/clinic	No	25/07/2017	0	0	0
2011144833	32	rural/clinic	No	25/07/2017	0	0	0
2015359622	32	rural/clinic	No	25/07/2017	0	0	0
2010099933	32	rural/clinic	No	20/07/2017	0	0	0
2015360605	32	rural/clinic	No	25/07/2017	0	0	0
2015360606	267	district/faith-based	No	25/07/2017	1	0	0
2015305813	267	district/faith-based	No	20/07/2017	1	0	0
2014312411	267	district/faith-based	No	20/07/2017	1	0	0
2014312411	267	district/faith-based	No	20/07/2017	1	0	0
2011204484	267	district/faith-based	No	20/07/2017	1	0	0
2011204484	267	district/faith-based	No	20/07/2017	1	0	0
2014314485	267	district/faith-based	No	25/07/2017	1	0	0
2015363463	267	district/faith-based	No	25/07/2017	1	0	0
2015386748	267	district/faith-based	No	20/07/2017	1	0	0
2015386748	267	district/faith-based	No	20/07/2017	1	0	0
2015294888	267	district/faith-based	No	25/07/2017	1	0	0
2015337998	267	district/faith-based	No	25/07/2017	1	0	0
2015363464	267	district/faith-based	No	25/07/2017	1	0	0
2015294887	267	district/faith-based	No	25/07/2017	1	0	0
2015363466	267	district/faith-based	No	25/07/2017	1	0	0
2014343879	267	district/faith-based	No	25/07/2017	1	0	0
2011212966	267	district/faith-based	No	25/07/2017	1	0	0
2015355786	267	district/faith-based	No	25/07/2017	1	0	0
2012312204	267	district/faith-based	No	25/07/2017	1	0	0
2015325943	267	district/faith-based	No	25/07/2017	1	0	0
2015325944	267	district/faith-based	No	25/07/2017	1	0	0
2015302448	267	district/faith-based	No	25/07/2017	1	0	0
2015325211	267	district/faith-based	No	25/07/2017	1	0	0
2015325212	267	district/faith-based	No	25/07/2017	1	0	0
2015325213	267	district/faith-based	No	25/07/2017	1	0	0
2013256280	267	district/faith-based	No	25/07/2017	1	0	0
2015302447	267	district/faith-based	No	25/07/2017	1	0	0
2014327128	267	district/faith-based	No	25/07/2017	1	0	0
2014349924	267	district/faith-based	No	25/07/2017	1	0	0
2014319133	267	district/faith-based	No	25/07/2017	1	0	0
2012243655	267	district/faith-based	No	25/07/2017	1	0	0
2015316032	267	district/faith-based	No	25/07/2017	1	0	0
2015326897	267	district/faith-based	No	25/07/2017	1	0	0
2015326898	267	district/faith-based	No	25/07/2017	1	0	0
2015326899	267	district/faith-based	No	25/07/2017	1	0	0
2013254810	267	district/faith-based	No	25/07/2017	1	0	0
2015326900	267	district/faith-based	No	25/07/2017	1	0	0
2015337519	267	district/faith-based	No	25/07/2017	1	0	0
2015337523	267	district/faith-based	No	25/07/2017	1	0	0
2015403701	267	district/faith-based	No	25/07/2017	1	0	0
2015337522	267	district/faith-based	No	25/07/2017	1	0	0
2015337520	267	district/faith-based	No	25/07/2017	1	0	0
2011133153	267	district/faith-based	No	25/07/2017	1	0	0

2015403702	822	rural/clinic	No	25/07/2017	0	0	0
2015303185	822	rural/clinic	No	25/07/2017	0	0	0
2015303186	822	rural/clinic	No	25/07/2017	0	0	0
2015303187	822	rural/clinic	No	25/07/2017	0	0	0
2015397341	822	rural/clinic	No	25/07/2017	0	0	0
2015412333	822	rural/clinic	No	25/07/2017	0	0	0
2015297167	822	rural/clinic	No	25/07/2017	0	0	0
2015383743	822	rural/clinic	No	25/07/2017	0	0	0
2015412330	822	rural/clinic	No	25/07/2017	0	0	0
2015412331	822	rural/clinic	No	25/07/2017	0	0	0
2015412332	822	rural/clinic	No	25/07/2017	0	0	0
2011192847	822	rural/clinic	No	25/07/2017	0	0	0
2014314739	822	rural/clinic	No	25/07/2017	0	0	0
2011192849	822	rural/clinic	No	25/07/2017	0	0	0
2011133154	822	rural/clinic	No	25/07/2017	0	0	0
2015337376	822	rural/clinic	No	25/07/2017	0	0	0
2011192848	822	rural/clinic	No	25/07/2017	0	0	0
2015326976	822	rural/clinic	No	25/07/2017	0	0	0
2014291820	822	rural/clinic	No	25/07/2017	0	0	0
2011187034	822	rural/clinic	No	25/07/2017	0	0	0
2011192850	822	rural/clinic	No	25/07/2017	0	0	0
2014353183	822	rural/clinic	No	25/07/2017	0	0	0
2014322246	822	rural/clinic	No	22/06/2017	0	0	0
2013261244	822	rural/clinic	No	25/07/2017	0	0	0
2015287388	822	rural/clinic	No	25/07/2017	0	0	0
2013261245	822	rural/clinic	No	25/07/2017	0	0	0
2015333856	822	rural/clinic	No	03/07/2017	0	0	0
2014353184	822	rural/clinic	No	25/07/2017	0	0	0
2014348381	822	rural/clinic	No	25/07/2017	0	0	0
2014321845	674	rural/clinic	No	25/07/2017	0	0	0
2014321846	674	rural/clinic	No	25/07/2017	0	0	0
2015326067	674	rural/clinic	No	25/07/2017	0	0	0
2015326069	674	rural/clinic	No	25/07/2017	0	0	0
2015326068	674	rural/clinic	No	25/07/2017	0	0	0
2015297164	674	rural/clinic	No	25/07/2017	0	0	0
2015297165	674	rural/clinic	No	25/07/2017	0	0	0
2015297166	674	rural/clinic	No	25/07/2017	0	0	0
2015359621	674	rural/clinic	No	25/07/2017	0	0	0
2015340901	674	rural/clinic	No	25/07/2017	0	0	0
2014319213	674	rural/clinic	No	25/07/2017	0	0	0
2015325214	674	rural/clinic	No	25/07/2017	0	0	0
2015357303	674	rural/clinic	No	25/07/2017	0	0	0
2014320304	674	rural/clinic	No	25/07/2017	0	0	0
2014349926	674	rural/clinic	No	25/07/2017	0	0	0
2015325184	674	rural/clinic	No	25/07/2017	0	0	0
2015302450	674	rural/clinic	No	25/07/2017	0	0	0
2015302449	674	rural/clinic	No	25/07/2017	0	0	0
2014349925	674	rural/clinic	No	25/07/2017	0	0	0
2012312205	674	rural/clinic	No	25/07/2017	0	0	0
2015357304	860	rural/clinic	No	25/07/2017	0	0	0
2015357305	860	rural/clinic	No	25/07/2017	0	0	0
2015326902	860	rural/clinic	No	25/07/2017	0	0	0
2015326450	860	rural/clinic	No	25/07/2017	0	0	0
2015326901	860	rural/clinic	No	25/07/2017	0	0	0
2013285128	860	rural/clinic	No	25/07/2017	0	0	0
2011117852	860	rural/clinic	No	25/07/2017	0	0	0
2011117853	860	rural/clinic	No	25/07/2017	0	0	0
2013266249	860	rural/clinic	No	25/07/2017	0	0	0
2013266250	860	rural/clinic	No	25/07/2017	0	0	0
2015368726	860	rural/clinic	No	25/07/2017	0	0	0
2015368729	841	district/faith-based	No	25/07/2017	1	0	0
2015331402	841	district/faith-based	No	25/07/2017	1	0	0
2015331102	841	district/faith-based	No	25/07/2017	1	0	0
2015359618	841	district/faith-based	No	25/07/2017	1	0	0
2015349011	841	district/faith-based	No	01/06/2017	1	0	0
2011203850	841	district/faith-based	No	27/07/2017	1	0	0
2014333601	841	district/faith-based	No	27/07/2017	1	0	0
2015415067	841	district/faith-based	No	27/07/2017	1	0	0
2015340234	841	district/faith-based	No	27/07/2017	1	0	0
2014368028	841	district/faith-based	No	27/07/2017	1	0	0
2015301506	841	district/faith-based	No	27/07/2017	1	0	0
2015364611	841	district/faith-based	No	27/07/2017	1	0	0
2014378708	841	district/faith-based	No	27/07/2017	1	0	0
2015314034	841	district/faith-based	No	27/07/2017	1	0	0
2015314033	841	district/faith-based	No	27/07/2017	1	0	0
2015314032	841	district/faith-based	No	11/07/2017	1	0	0
2015378210	841	district/faith-based	No	17/07/2017	1	0	0
2015404657	841	district/faith-based	No	27/07/2017	1	0	0
2015404659	841	district/faith-based	No	27/07/2017	1	0	0
2014297842	841	district/faith-based	No	13/07/2017	1	0	0
2012317567	841	district/faith-based	No	27/07/2017	1	0	0
2015358200	841	district/faith-based	No	27/07/2017	1	0	0

2011225373	841	district/faith-based	No	13/07/2017	1	0	0
2014350963	841	district/faith-based	No	27/07/2017	1	0	0
2014347994	841	district/faith-based	No	27/07/2017	1	0	0
2015339605	841	district/faith-based	No	27/07/2017	1	0	0
2015339606	841	district/faith-based	No	27/07/2017	1	0	0
2015402001	841	district/faith-based	No	27/07/2017	1	0	0
2014375518	841	district/faith-based	No	27/07/2017	1	0	0
2015402002	841	district/faith-based	No	27/07/2017	1	0	0
2014375519	841	district/faith-based	No	27/07/2017	1	0	0
2015402003	841	district/faith-based	No	27/07/2017	1	0	0
2012291377	841	district/faith-based	No	27/07/2017	1	0	0
2015402004	841	district/faith-based	No	27/07/2017	1	0	0
2012291376	841	district/faith-based	No	27/07/2017	1	0	0
2012291374	841	district/faith-based	No	27/07/2017	1	0	0
2015402005	841	district/faith-based	No	27/07/2017	1	0	0
2014358227	841	district/faith-based	No	27/07/2017	1	0	0
2015326981	841	district/faith-based	No	27/07/2017	1	0	0
2014375520	841	district/faith-based	No	27/07/2017	1	0	0
2012291375	335	rural/clinic	No	27/07/2017	0	0	0
2014332082	335	rural/clinic	No	27/07/2017	0	0	0
2014368029	335	rural/clinic	No	27/07/2017	0	0	0
2014368027	335	rural/clinic	No	27/07/2017	0	0	0
2015332109	335	rural/clinic	No	27/07/2017	0	0	0
2015352653	335	rural/clinic	No	27/07/2017	0	0	0
2015352656	335	rural/clinic	No	27/07/2017	0	0	0
2015352654	335	rural/clinic	No	27/07/2017	0	0	0
2015352655	335	rural/clinic	No	27/07/2017	0	0	0
2014346329	335	rural/clinic	No	27/07/2017	0	0	0
2014346330	335	rural/clinic	No	27/07/2017	0	0	0
2014335532	335	rural/clinic	No	27/07/2017	0	0	0
2014329074	335	rural/clinic	No	27/07/2017	0	0	0
2015404152	335	rural/clinic	No	27/07/2017	0	0	0
2014367406	335	rural/clinic	No	27/07/2017	0	0	0
2014367407	335	rural/clinic	No	27/07/2017	0	0	0
2014367408	335	rural/clinic	No	27/07/2017	0	0	0
2015384305	335	rural/clinic	No	27/07/2017	0	0	0
2012317566	335	rural/clinic	No	27/07/2017	0	0	0
2012317568	389	rural/clinic	No	27/07/2017	0	0	0
2015384303	389	rural/clinic	No	27/07/2017	0	0	0
2015384304	389	rural/clinic	No	27/07/2017	0	0	0
2015355923	389	rural/clinic	No	27/07/2017	0	0	0
2015355924	389	rural/clinic	No	27/07/2017	0	0	0
2012388283	389	rural/clinic	No	27/07/2017	0	0	0
2012388284	389	rural/clinic	No	27/07/2017	0	0	0
2012388285	389	rural/clinic	No	27/07/2017	0	0	0
2012388281	389	rural/clinic	No	27/07/2017	0	0	0
2012388282	389	rural/clinic	No	27/07/2017	0	0	0
2015369748	389	rural/clinic	No	27/07/2017	0	0	0
2015319297	389	rural/clinic	No	20/07/2017	0	0	0
2015319297	389	rural/clinic	No	20/07/2017	0	0	0
2015373869	389	rural/clinic	No	20/07/2017	0	0	0
2015373869	389	rural/clinic	No	20/07/2017	0	0	0
2015373870	389	rural/clinic	No	20/07/2017	0	0	0
2015373870	389	rural/clinic	No	20/07/2017	0	0	0
2015397338	389	rural/clinic	No	24/07/2017	0	0	0
2015397338	389	rural/clinic	No	24/07/2017	0	0	0
2015369749	389	rural/clinic	No	27/07/2017	0	0	0
2013251318	389	rural/clinic	No	24/07/2017	0	0	0
2013251318	389	rural/clinic	No	24/07/2017	0	0	0
2015358304	389	rural/clinic	No	27/07/2017	0	0	0
2011155239	389	rural/clinic	No	27/07/2017	0	0	0
2011155238	389	rural/clinic	No	27/07/2017	0	0	0
2014312274	389	rural/clinic	No	27/07/2017	0	0	0
2015358305	389	rural/clinic	No	27/07/2017	0	0	0
2015358303	389	rural/clinic	No	27/07/2017	0	0	0
2015349540	389	rural/clinic	No	27/07/2017	0	0	0
2015419060	389	rural/clinic	No	27/07/2017	0	0	0
2015349462	933	rural/clinic	No	27/07/2017	0	0	0
2015419061	933	rural/clinic	No	27/07/2017	0	0	0
2011229223	933	rural/clinic	No	27/07/2017	0	0	0
2012291378	933	rural/clinic	No	27/07/2017	0	0	0
2015297291	933	rural/clinic	No	27/07/2017	0	0	0
2015349541	933	rural/clinic	No	27/07/2017	0	0	0
2011225376	933	rural/clinic	No	27/07/2017	0	0	0
2015369012	933	rural/clinic	No	27/07/2017	0	0	0
2015369013	933	rural/clinic	No	27/07/2017	0	0	0
2015369014	933	rural/clinic	No	27/07/2017	0	0	0
2015369017	933	rural/clinic	No	27/07/2017	0	0	0
2015369016	933	rural/clinic	No	27/07/2017	0	0	0
2015305093	933	rural/clinic	No	27/07/2017	0	0	0
2015345084	933	rural/clinic	No	27/07/2017	0	0	0
2015345083	933	rural/clinic	No	27/07/2017	0	0	0

2015302152	933	rural/clinic	No	27/07/2017	0	0	0
2015345081	933	rural/clinic	No	27/07/2017	0	0	0
2015345082	933	rural/clinic	No	27/07/2017	0	0	0
2015340019	287	rural/clinic	No	27/07/2017	0	0	0
2015404660	287	rural/clinic	No	27/07/2017	0	0	0
2015404658	287	rural/clinic	No	27/07/2017	0	0	0
2015349463	287	rural/clinic	No	27/07/2017	0	0	0
2015404656	287	rural/clinic	No	27/07/2017	0	0	0
2015404655	287	rural/clinic	No	27/07/2017	0	0	0
2015404654	287	rural/clinic	No	27/07/2017	0	0	0
2014316073	287	rural/clinic	No	27/07/2017	0	0	0
2015313001	287	rural/clinic	No	27/07/2017	0	0	0
2015286335	287	rural/clinic	No	27/07/2017	0	0	0
2015326978	287	rural/clinic	No	27/07/2017	0	0	0
2015286336	287	rural/clinic	No	27/07/2017	0	0	0
2015286337	287	rural/clinic	No	27/07/2017	0	0	0
2015326980	287	rural/clinic	No	27/07/2017	0	0	0
2015378211	287	rural/clinic	No	27/07/2017	0	0	0
2015326982	287	rural/clinic	No	27/07/2017	0	0	0
2015378212	287	rural/clinic	No	27/07/2017	0	0	0
2015369082	287	rural/clinic	No	27/07/2017	0	0	0
2015326979	287	rural/clinic	No	27/07/2017	0	0	0
2015369085	287	rural/clinic	No	27/07/2017	0	0	0
2015369084	287	rural/clinic	No	27/07/2017	0	0	0
2012275213	287	rural/clinic	No	27/07/2017	0	0	0
2015369083	287	rural/clinic	No	27/07/2017	0	0	0
2012275215	287	rural/clinic	No	27/07/2017	0	0	0
2015369086	287	rural/clinic	No	27/07/2017	0	0	0
2015369087	287	rural/clinic	No	27/07/2017	0	0	0
2012275214	47	rural/clinic	No	27/07/2017	0	0	0
2014317648	47	rural/clinic	No	27/07/2017	0	0	0
2015415290	47	rural/clinic	No	27/07/2017	0	0	0
2015326602	47	rural/clinic	No	27/07/2017	0	0	0
2015415291	47	rural/clinic	No	27/07/2017	0	0	0
2015415289	47	rural/clinic	No	27/07/2017	0	0	0
2014333605	47	rural/clinic	No	27/07/2017	0	0	0
2015415917	47	rural/clinic	No	27/07/2017	0	0	0
2015415918	47	rural/clinic	No	27/07/2017	0	0	0
2015415288	47	rural/clinic	No	27/07/2017	0	0	0
2015415201	47	rural/clinic	No	27/07/2017	0	0	0
2015373760	47	rural/clinic	No	27/07/2017	0	0	0
2015373761	47	rural/clinic	No	27/07/2017	0	0	0
2015373762	47	rural/clinic	No	27/07/2017	0	0	0
2015373763	47	rural/clinic	No	27/07/2017	0	0	0
2015373764	47	rural/clinic	No	27/07/2017	0	0	0
2015373765	47	rural/clinic	No	27/07/2017	0	0	0
2015373766	47	rural/clinic	No	27/07/2017	0	0	0
2014333602	47	rural/clinic	No	27/07/2017	0	0	0
2015373767	47	rural/clinic	No	27/07/2017	0	0	0
2015373768	47	rural/clinic	No	27/07/2017	0	0	0
2015373769	47	rural/clinic	No	27/07/2017	0	0	0
2014333603	47	rural/clinic	No	27/07/2017	0	0	0
2014358345	47	rural/clinic	No	27/07/2017	0	0	0
2014358344	47	rural/clinic	No	27/07/2017	0	0	0
2014333604	47	rural/clinic	No	27/07/2017	0	0	0
2014358346	47	rural/clinic	No	27/07/2017	0	0	0
2014358347	47	rural/clinic	No	27/07/2017	0	0	0
2015376407	47	rural/clinic	No	27/07/2017	0	0	0
2014314531	47	rural/clinic	No	27/07/2017	0	0	0
2014368692	47	rural/clinic	No	27/07/2017	0	0	0
2014373151	47	rural/clinic	No	27/07/2017	0	0	0
2012369815	47	rural/clinic	No	27/07/2017	0	0	0
2014357332	47	rural/clinic	No	27/07/2017	0	0	0
2014314526	47	rural/clinic	No	27/07/2017	0	0	0
2014357331	47	rural/clinic	No	27/07/2017	0	0	0
2015364613	47	rural/clinic	No	27/07/2017	0	0	0
2014357330	47	rural/clinic	No	27/07/2017	0	0	0
2014307339	47	rural/clinic	No	27/07/2017	0	0	0
2014307340	47	rural/clinic	No	27/07/2017	0	0	0
2015364612	47	rural/clinic	No	27/07/2017	0	0	0
2015376409	47	rural/clinic	No	27/07/2017	0	0	0
2014287166	47	rural/clinic	No	27/07/2017	0	0	0
2014314529	47	rural/clinic	No	27/07/2017	0	0	0
2015376406	47	rural/clinic	No	27/07/2017	0	0	0
2014358348	47	rural/clinic	No	27/07/2017	0	0	0
2014361227	47	rural/clinic	No	27/07/2017	0	0	0
2015364530	47	rural/clinic	No	27/07/2017	0	0	0
2014357858	47	rural/clinic	No	27/07/2017	0	0	0
2015364526	47	rural/clinic	No	27/07/2017	0	0	0
2014357859	47	rural/clinic	No	27/07/2017	0	0	0
2015364525	47	rural/clinic	No	27/07/2017	0	0	0
2015364529	47	rural/clinic	No	27/07/2017	0	0	0

2011135063	47	rural/clinic	No	27/07/2017	0	0	0
2015360206	47	rural/clinic	No	27/07/2017	0	0	0
2014314230	47	rural/clinic	No	27/07/2017	0	0	0
2015336652	47	rural/clinic	No	27/07/2017	0	0	0
2013264076	47	rural/clinic	No	27/07/2017	0	0	0
2015376665	47	rural/clinic	No	27/07/2017	0	0	0
2015376666	47	rural/clinic	No	27/07/2017	0	0	0
2015385544	47	rural/clinic	No	27/07/2017	0	0	0
2012335668	47	rural/clinic	No	27/07/2017	0	0	0
2015385545	47	rural/clinic	No	27/07/2017	0	0	0
2015401158	47	rural/clinic	No	27/07/2017	0	0	0
2012335669	47	rural/clinic	No	27/07/2017	0	0	0
2015401161	47	rural/clinic	No	27/07/2017	0	0	0
2012335670	47	rural/clinic	No	27/07/2017	0	0	0
2014338454	47	rural/clinic	No	27/07/2017	0	0	0
2012362572	47	rural/clinic	No	27/07/2017	0	0	0
2012275991	47	rural/clinic	No	27/07/2017	0	0	0
2012362573	47	rural/clinic	No	27/07/2017	0	0	0
2012275992	47	rural/clinic	No	27/07/2017	0	0	0
2012289790	47	rural/clinic	No	27/07/2017	0	0	0
2012363976	47	rural/clinic	No	27/07/2017	0	0	0
2012363977	47	rural/clinic	No	27/07/2017	0	0	0
2012363978	47	rural/clinic	No	27/07/2017	0	0	0
2015340713	47	rural/clinic	No	27/07/2017	0	0	0
2010099935	47	rural/clinic	No	27/07/2017	0	0	0
2012363979	47	rural/clinic	No	27/07/2017	0	0	0
2015340714	47	rural/clinic	No	27/07/2017	0	0	0
2014290242	47	rural/clinic	No	27/07/2017	0	0	0
2012363980	47	rural/clinic	No	27/07/2017	0	0	0
2015340232	47	rural/clinic	No	27/07/2017	0	0	0
2012306345	47	rural/clinic	No	27/07/2017	0	0	0
2015340366	47	rural/clinic	No	27/07/2017	0	0	0
2015340716	47	rural/clinic	No	27/07/2017	0	0	0
2015340367	47	rural/clinic	No	27/07/2017	0	0	0
2015340715	47	rural/clinic	No	27/07/2017	0	0	0
2011200080	47	rural/clinic	No	27/07/2017	0	0	0
2015340368	47	rural/clinic	No	27/07/2017	0	0	0
2015340233	47	rural/clinic	No	27/07/2017	0	0	0
2015340369	47	rural/clinic	No	27/07/2017	0	0	0
2015340370	47	rural/clinic	No	27/07/2017	0	0	0
2015340371	47	rural/clinic	No	27/07/2017	0	0	0
2015340372	47	rural/clinic	No	27/07/2017	0	0	0
2015340373	47	rural/clinic	No	27/07/2017	0	0	0
2015340374	47	rural/clinic	No	27/07/2017	0	0	0
2015305815	47	rural/clinic	No	27/07/2017	0	0	0
2015305816	47	rural/clinic	No	27/07/2017	0	0	0
2015305817	47	rural/clinic	No	27/07/2017	0	0	0
2015305814	47	rural/clinic	No	27/07/2017	0	0	0
2015321808	47	rural/clinic	No	27/07/2017	0	0	0
2015363308	47	rural/clinic	No	27/07/2017	0	0	0
2012368546	47	rural/clinic	No	25/07/2017	0	0	0
2012368546	47	rural/clinic	No	25/07/2017	0	0	0
2012312204	47	rural/clinic	No	25/07/2017	0	0	0
2014332715	47	rural/clinic	No	25/07/2017	0	0	0
2014332715	47	rural/clinic	No	25/07/2017	0	0	0
2012299682	47	rural/clinic	No	25/07/2017	0	0	0
2012299682	47	rural/clinic	No	25/07/2017	0	0	0
2014367628	47	rural/clinic	No	25/07/2017	0	0	0
2014367628	47	rural/clinic	No	25/07/2017	0	0	0
2014374037	47	rural/clinic	No	25/07/2017	0	0	0
2014374037	47	rural/clinic	No	25/07/2017	0	0	0
2014326640	47	rural/clinic	No	25/07/2017	0	0	0
2015405727	47	rural/clinic	No	25/07/2017	0	0	0
2015362298	47	rural/clinic	No	25/07/2017	0	0	0
2015357304	47	rural/clinic	No	25/07/2017	0	0	0
2015357304	47	rural/clinic	No	25/07/2017	0	0	0
2015338718	47	rural/clinic	No	25/07/2017	0	0	0
2015338718	47	rural/clinic	No	25/07/2017	0	0	0
2015368609	47	rural/clinic	No	25/07/2017	0	0	0
2015368609	47	rural/clinic	No	25/07/2017	0	0	0
2015377753	47	rural/clinic	No	25/07/2017	0	0	0
2015377753	47	rural/clinic	No	25/07/2017	0	0	0
2011144833	47	rural/clinic	No	25/07/2017	0	0	0
2011144833	47	rural/clinic	No	25/07/2017	0	0	0
2015363763	47	rural/clinic	No	25/07/2017	0	0	0
2015363763	47	rural/clinic	No	25/07/2017	0	0	0
2015349011	47	rural/clinic	No	01/06/2017	0	0	0
2015397341	47	rural/clinic	No	25/07/2017	0	0	0
2015397341	47	rural/clinic	No	25/07/2017	0	0	0
2015347203	47	rural/clinic	No	25/07/2017	0	0	0
2015347203	47	rural/clinic	No	25/07/2017	0	0	0
2015402001	47	rural/clinic	No	27/07/2017	0	0	0

2015402001	47	rural/clinic	No	27/07/2017	0	0	0
2014368029	47	rural/clinic	No	27/07/2017	0	0	0
2014368029	47	rural/clinic	No	27/07/2017	0	0	0
2015297165	47	rural/clinic	No	25/07/2017	0	0	0
2015340107	47	rural/clinic	No	06/06/2017	0	0	0
2015340107	47	rural/clinic	No	06/06/2017	0	0	0
2013256279	47	rural/clinic	No	25/07/2017	0	0	0
2015372803	47	rural/clinic	No	25/04/2017	0	0	0
2015340146	47	rural/clinic	No	06/07/2017	0	0	0
2015340146	47	rural/clinic	No	06/07/2017	0	0	0
2014371589	47	rural/clinic	No	04/07/2017	0	0	0
2014371589	47	rural/clinic	No	04/07/2017	0	0	0
2015332914	47	rural/clinic	No	06/07/2017	0	0	0
2015332914	47	rural/clinic	No	06/07/2017	0	0	0
2015344333	47	rural/clinic	No	18/07/2017	0	0	0
2015368606	47	rural/clinic	No	18/07/2017	0	0	0
2015397936	47	rural/clinic	No	18/07/2017	0	0	0
2015337999	47	rural/clinic	No	01/08/2017	0	0	0
2014343876	47	rural/clinic	No	18/07/2017	0	0	0
2015372819	47	rural/clinic	No	01/08/2017	0	0	0
2015397931	47	rural/clinic	No	18/07/2017	0	0	0
2014338515	47	rural/clinic	No	01/08/2017	0	0	0
2014301356	47	rural/clinic	No	01/08/2017	0	0	0
2015372818	47	rural/clinic	No	01/08/2017	0	0	0
2015372820	47	rural/clinic	No	01/08/2017	0	0	0
2014338514	47	rural/clinic	No	01/08/2017	0	0	0
2014314486	47	rural/clinic	No	01/08/2017	0	0	0
2014340326	47	rural/clinic	No	01/08/2017	0	0	0
2011212967	47	rural/clinic	No	01/08/2017	0	0	0
2011197679	47	rural/clinic	No	01/08/2017	0	0	0
2015335976	47	rural/clinic	No	01/08/2017	0	0	0
2015382955	47	rural/clinic	No	01/08/2017	0	0	0
2015382956	47	rural/clinic	No	01/08/2017	0	0	0
2015335975	47	rural/clinic	No	01/08/2017	0	0	0
2015382953	47	rural/clinic	No	01/08/2017	0	0	0
2015360610	47	rural/clinic	No	01/08/2017	0	0	0
2015382954	47	rural/clinic	No	01/08/2017	0	0	0
2015360608	47	rural/clinic	No	01/08/2017	0	0	0
2015382952	47	rural/clinic	No	01/08/2017	0	0	0
2012379905	47	rural/clinic	No	01/08/2017	0	0	0
2015326903	47	rural/clinic	No	01/08/2017	0	0	0
2012379906	47	rural/clinic	No	01/08/2017	0	0	0
2015326904	47	rural/clinic	No	01/08/2017	0	0	0
2012379904	47	rural/clinic	No	01/08/2017	0	0	0
2011117854	47	rural/clinic	No	01/08/2017	0	0	0
2011117855	47	rural/clinic	No	01/08/2017	0	0	0
2015361609	47	rural/clinic	No	01/08/2017	0	0	0
2011117856	47	rural/clinic	No	01/08/2017	0	0	0
2012305827	47	rural/clinic	No	01/08/2017	0	0	0
2014346739	47	rural/clinic	No	01/08/2017	0	0	0
2015342012	47	rural/clinic	No	01/08/2017	0	0	0
2014332716	47	rural/clinic	No	01/08/2017	0	0	0
2015342013	47	rural/clinic	No	01/08/2017	0	0	0
2015342297	47	rural/clinic	No	01/08/2017	0	0	0
2014377417	47	rural/clinic	No	01/08/2017	0	0	0
2015372943	47	rural/clinic	No	01/08/2017	0	0	0
2015342295	47	rural/clinic	No	01/08/2017	0	0	0
2014358196	47	rural/clinic	No	01/08/2017	0	0	0
2015342296	47	rural/clinic	No	01/08/2017	0	0	0
2015367706	47	rural/clinic	No	01/08/2017	0	0	0
2015359764	47	rural/clinic	No	01/08/2017	0	0	0
2015367705	47	rural/clinic	No	01/08/2017	0	0	0
2015361610	47	rural/clinic	No	01/08/2017	0	0	0
2014382304	47	rural/clinic	No	01/08/2017	0	0	0
2012305826	47	rural/clinic	No	01/08/2017	0	0	0
2014382303	47	rural/clinic	No	01/08/2017	0	0	0
2015378736	47	rural/clinic	No	01/08/2017	0	0	0
2014301361	47	rural/clinic	No	01/08/2017	0	0	0
2015360858	47	rural/clinic	No	01/08/2017	0	0	0
2015368730	47	rural/clinic	No	01/08/2017	0	0	0
2014301358	47	rural/clinic	No	01/08/2017	0	0	0
2015368732	47	rural/clinic	No	01/08/2017	0	0	0
2014301357	47	rural/clinic	No	01/08/2017	0	0	0
2015368733	47	rural/clinic	No	01/08/2017	0	0	0
2015321106	47	rural/clinic	No	01/08/2017	0	0	0
2015321105	47	rural/clinic	No	01/08/2017	0	0	0
2015321104	47	rural/clinic	No	01/08/2017	0	0	0
2015368731	47	rural/clinic	No	01/08/2017	0	0	0
2015305089	47	rural/clinic	No	01/08/2017	0	0	0
2015305086	47	rural/clinic	No	01/08/2017	0	0	0
2015321103	47	rural/clinic	No	01/08/2017	0	0	0
2015305087	47	rural/clinic	No	01/08/2017	0	0	0

2015321101	47	rural/clinic	No	01/08/2017	0	0	0
2015305088	47	rural/clinic	No	01/08/2017	0	0	0
2015321102	47	rural/clinic	No	01/08/2017	0	0	0
2015345066	47	rural/clinic	No	01/08/2017	0	0	0
2015367707	47	rural/clinic	No	01/08/2017	0	0	0
2015345067	47	rural/clinic	No	01/08/2017	0	0	0
2015367703	47	rural/clinic	No	01/08/2017	0	0	0
2015345068	47	rural/clinic	No	01/08/2017	0	0	0
2014377418	47	rural/clinic	No	01/08/2017	0	0	0
2015345069	47	rural/clinic	No	01/08/2017	0	0	0
2015367704	47	rural/clinic	No	01/08/2017	0	0	0
2015383749	47	rural/clinic	No	01/08/2017	0	0	0
2015383744	211	rural/clinic	No	01/08/2017	0	0	0
2015383745	211	rural/clinic	No	01/08/2017	0	0	0
2015383746	211	rural/clinic	No	01/08/2017	0	0	0
2011144836	211	rural/clinic	No	01/08/2017	0	0	0
2015364530	98	rural/clinic	No	27/07/2017	0	0	0
2015364530	98	rural/clinic	No	27/07/2017	0	0	0
2015325220	98	rural/clinic	No	01/08/2017	0	0	0
2015332441	98	rural/clinic	No	01/08/2017	0	0	0
2015364951	98	rural/clinic	No	01/08/2017	0	0	0
2015305816	98	rural/clinic	No	27/07/2017	0	0	0
2015364952	98	rural/clinic	No	01/08/2017	0	0	0
2015419061	98	rural/clinic	No	27/07/2017	0	0	0
2015335810	98	rural/clinic	No	01/08/2017	0	0	0
2015417083	98	rural/clinic	No	01/08/2017	0	0	0
2015326983	98	rural/clinic	No	01/08/2017	0	0	0
2015417084	98	rural/clinic	No	01/08/2017	0	0	0
2015417085	98	rural/clinic	No	01/08/2017	0	0	0
2015326984	98	rural/clinic	No	01/08/2017	0	0	0
2015414397	98	rural/clinic	No	01/08/2017	0	0	0
2015373750	98	rural/clinic	No	01/08/2017	0	0	0
2015326985	98	rural/clinic	No	01/08/2017	0	0	0
2015373770	98	rural/clinic	No	01/08/2017	0	0	0
2015373771	98	rural/clinic	No	01/08/2017	0	0	0
2015373772	98	rural/clinic	No	01/08/2017	0	0	0
2015373774	98	rural/clinic	No	01/08/2017	0	0	0
2012304339	98	rural/clinic	No	01/08/2017	0	0	0
2015373773	98	rural/clinic	No	01/08/2017	0	0	0
2015368610	98	rural/clinic	No	01/08/2017	0	0	0
2015373775	98	rural/clinic	No	01/08/2017	0	0	0
2015373776	98	rural/clinic	No	01/08/2017	0	0	0
2015373777	98	rural/clinic	No	01/08/2017	0	0	0
2015373778	98	rural/clinic	No	01/08/2017	0	0	0
2015373779	98	rural/clinic	No	01/08/2017	0	0	0
2015373780	98	rural/clinic	No	01/08/2017	0	0	0
2015360856	98	rural/clinic	No	01/08/2017	0	0	0
2015373781	98	rural/clinic	No	01/08/2017	0	0	0
2015360857	98	rural/clinic	No	01/08/2017	0	0	0
2015373782	98	rural/clinic	No	01/08/2017	0	0	0
2015373873	268	rural/clinic	No	01/08/2017	0	0	0
2015373783	268	rural/clinic	No	01/08/2017	0	0	0
2015373875	268	rural/clinic	No	01/08/2017	0	0	0
2015373784	268	rural/clinic	No	01/08/2017	0	0	0
2015373874	268	rural/clinic	No	01/08/2017	0	0	0
2015397342	268	rural/clinic	No	01/08/2017	0	0	0
2015384048	268	rural/clinic	No	06/07/2017	0	0	0
2015397343	268	rural/clinic	No	01/08/2017	0	0	0
2015397344	268	rural/clinic	No	01/08/2017	0	0	0
2015397345	268	rural/clinic	No	01/08/2017	0	0	0
2015373785	268	rural/clinic	No	01/08/2017	0	0	0
2015397346	268	rural/clinic	No	01/08/2017	0	0	0
2015293489	268	rural/clinic	No	01/08/2017	0	0	0
2015293494	268	rural/clinic	No	01/08/2017	0	0	0
2015373786	268	rural/clinic	No	01/08/2017	0	0	0
2015293493	268	rural/clinic	No	01/08/2017	0	0	0
2015373787	268	rural/clinic	No	01/08/2017	0	0	0
2015293491	268	rural/clinic	No	01/08/2017	0	0	0
2015340902	268	rural/clinic	No	01/08/2017	0	0	0
2015293488	268	rural/clinic	No	01/08/2017	0	0	0
2014320299	268	rural/clinic	No	01/08/2017	0	0	0
2015293492	268	rural/clinic	No	01/08/2017	0	0	0
2015403204	268	rural/clinic	No	01/08/2017	0	0	0
2015293490	268	rural/clinic	No	01/08/2017	0	0	0
2015372856	268	rural/clinic	No	01/08/2017	0	0	0
2015372854	268	rural/clinic	No	01/08/2017	0	0	0
2015372942	268	rural/clinic	No	01/08/2017	0	0	0
2015372941	532	rural/clinic	No	01/08/2017	0	0	0
2015372855	532	rural/clinic	No	01/08/2017	0	0	0
2011144837	532	rural/clinic	No	01/08/2017	0	0	0
2015372853	532	rural/clinic	No	01/08/2017	0	0	0
2015373876	532	rural/clinic	No	01/08/2017	0	0	0

2015373877	532	rural/clinic	No	01/08/2017	0	0	0
2015373878	532	rural/clinic	No	01/08/2017	0	0	0
2015373879	532	rural/clinic	No	01/08/2017	0	0	0
2015373880	532	rural/clinic	No	01/08/2017	0	0	0
2015373881	532	rural/clinic	No	01/08/2017	0	0	0
2014319134	532	rural/clinic	No	01/08/2017	0	0	0
2015325216	532	rural/clinic	No	01/08/2017	0	0	0
2015325217	532	rural/clinic	No	01/08/2017	0	0	0
2012260088	510	rural/clinic	No	01/08/2017	0	0	0
2015287392	510	rural/clinic	No	01/08/2017	0	0	0
2015287391	510	rural/clinic	No	01/08/2017	0	0	0
2015287390	510	rural/clinic	No	01/08/2017	0	0	0
2011136254	510	rural/clinic	No	01/08/2017	0	0	0
2011136255	510	rural/clinic	No	01/08/2017	0	0	0
2011136256	175	rural/clinic	No	01/08/2017	0	0	0
2014363595	175	rural/clinic	No	01/08/2017	0	0	0
2015338000	175	rural/clinic	No	01/08/2017	0	0	0
2014363594	175	rural/clinic	No	01/08/2017	0	0	0
2014363593	175	rural/clinic	No	01/08/2017	0	0	0
2011212968	176	rural/clinic	No	01/08/2017	0	0	0
2015335974	176	rural/clinic	No	01/08/2017	0	0	0
2014363660	176	rural/clinic	No	01/08/2017	0	0	0
2015335973	176	rural/clinic	No	01/08/2017	0	0	0
2015363469	176	rural/clinic	No	01/08/2017	0	0	0
2012321343	176	rural/clinic	No	01/08/2017	0	0	0
2014363659	176	rural/clinic	No	01/08/2017	0	0	0
2015289339	176	rural/clinic	No	01/08/2017	0	0	0
2015363151	176	rural/clinic	No	01/08/2017	0	0	0
2015382955	162	rural/clinic	No	01/08/2017	0	0	0
2015342295	162	rural/clinic	No	01/08/2017	0	0	0
2015342295	162	rural/clinic	No	01/08/2017	0	0	0
2015378208	162	rural/clinic	No	03/08/2017	0	0	0
2017099	162	rural/clinic	No	20/07/2017	0	0	0
2015378209	162	rural/clinic	No	03/08/2017	0	0	0
2015415068	162	rural/clinic	No	03/08/2017	0	0	0
2012331329	162	rural/clinic	No	03/08/2017	0	0	0
2015334805	162	rural/clinic	No	03/08/2017	0	0	0
2011226490	162	rural/clinic	No	03/08/2017	0	0	0
2014309592	162	rural/clinic	No	03/08/2017	0	0	0
2015373777	162	rural/clinic	No	01/08/2017	0	0	0
2015373777	162	rural/clinic	No	01/08/2017	0	0	0
2014319134	162	rural/clinic	No	01/08/2017	0	0	0
2014319134	162	rural/clinic	No	01/08/2017	0	0	0
2015373874	162	rural/clinic	No	01/08/2017	0	0	0
2015373874	162	rural/clinic	No	01/08/2017	0	0	0
2015360611	162	rural/clinic	No	01/08/2017	0	0	0
2015360611	162	rural/clinic	No	01/08/2017	0	0	0
2011139409	162	rural/clinic	No	01/08/2017	0	0	0
2011139409	162	rural/clinic	No	01/08/2017	0	0	0
2015305088	162	rural/clinic	No	01/08/2017	0	0	0
2015305088	162	rural/clinic	No	01/08/2017	0	0	0
2011117854	162	rural/clinic	No	01/08/2017	0	0	0
2011117854	162	rural/clinic	No	01/08/2017	0	0	0
2015349735	162	rural/clinic	No	03/08/2017	0	0	0
2015412782	162	rural/clinic	No	03/08/2017	0	0	0
2015355017	162	rural/clinic	No	03/08/2017	0	0	0
2014309591	162	rural/clinic	No	03/08/2017	0	0	0
2014309593	162	rural/clinic	No	03/08/2017	0	0	0
2015289213	162	rural/clinic	No	03/08/2017	0	0	0
2011225650	162	rural/clinic	No	03/08/2017	0	0	0
2015412783	162	rural/clinic	No	03/08/2017	0	0	0
2015289211	162	rural/clinic	No	03/08/2017	0	0	0
2015297297	162	rural/clinic	No	03/08/2017	0	0	0
2015289214	162	rural/clinic	No	03/08/2017	0	0	0
2014357051	162	rural/clinic	No	03/08/2017	0	0	0
2015338880	162	rural/clinic	No	03/08/2017	0	0	0
2015289212	162	rural/clinic	No	03/08/2017	0	0	0
2015349734	162	rural/clinic	No	03/08/2017	0	0	0
2015338878	162	rural/clinic	No	03/08/2017	0	0	0
2015412652	162	rural/clinic	No	03/08/2017	0	0	0
2014358282	162	rural/clinic	No	03/08/2017	0	0	0
2012363407	336	rural/clinic	No	03/08/2017	0	0	0
2014358281	336	rural/clinic	No	03/08/2017	0	0	0
2015414398	336	rural/clinic	No	03/08/2017	0	0	0
2015412651	336	rural/clinic	No	03/08/2017	0	0	0
2012363408	336	rural/clinic	No	04/08/2017	0	0	0
2015414399	336	rural/clinic	No	03/08/2017	0	0	0
2015415069	336	rural/clinic	No	03/08/2017	0	0	0
2012363409	336	rural/clinic	No	04/08/2017	0	0	0
2013272696	336	rural/clinic	No	03/08/2017	0	0	0
2012363410	336	rural/clinic	No	04/08/2017	0	0	0
2012388292	336	rural/clinic	No	03/08/2017	0	0	0

2012363411	336	rural/clinic	No	04/08/2017	0	0	0
2014358350	336	rural/clinic	No	03/08/2017	0	0	0
2015297295	336	rural/clinic	No	03/08/2017	0	0	0
2012388291	336	rural/clinic	No	03/08/2017	0	0	0
2012363412	336	rural/clinic	No	04/08/2017	0	0	0
2014305948	336	rural/clinic	No	03/08/2017	0	0	0
2015352658	336	rural/clinic	No	04/08/2017	0	0	0
2015352002	336	rural/clinic	No	03/08/2017	0	0	0
2015324265	336	rural/clinic	No	04/08/2017	0	0	0
2015297294	336	rural/clinic	No	03/08/2017	0	0	0
2014321428	336	rural/clinic	No	03/08/2017	0	0	0
2014346333	336	rural/clinic	No	04/08/2017	0	0	0
2015352001	336	rural/clinic	No	03/08/2017	0	0	0
2012388290	336	rural/clinic	No	03/08/2017	0	0	0
2015404153	336	rural/clinic	No	04/08/2017	0	0	0
2015352003	336	rural/clinic	No	03/08/2017	0	0	0
2014372338	336	rural/clinic	No	04/08/2017	0	0	0
2012388289	336	rural/clinic	No	03/08/2017	0	0	0
2014358349	336	rural/clinic	No	03/08/2017	0	0	0
2014326093	336	rural/clinic	No	03/08/2017	0	0	0
2012388288	336	rural/clinic	No	03/08/2017	0	0	0
2014317350	336	rural/clinic	No	03/08/2017	0	0	0
2012388287	336	rural/clinic	No	03/08/2017	0	0	0
2014287180	777	district/faith-based	No	03/08/2017	1	0	0
2014326092	777	district/faith-based	No	03/08/2017	1	0	0
2014287182	777	district/faith-based	No	03/08/2017	1	0	0
2012388286	777	district/faith-based	No	03/08/2017	1	0	0
2014287181	777	district/faith-based	No	03/08/2017	1	0	0
2015297293	777	district/faith-based	No	03/08/2017	1	0	0
2015419063	777	district/faith-based	No	03/08/2017	1	0	0
2014294155	777	district/faith-based	No	03/08/2017	1	0	0
2014368489	777	district/faith-based	No	04/08/2017	1	0	0
2011225377	777	district/faith-based	No	03/08/2017	1	0	0
2015336102	777	district/faith-based	No	03/08/2017	1	0	0
2015297292	777	district/faith-based	No	03/08/2017	1	0	0
2011225378	777	district/faith-based	No	03/08/2017	1	0	0
2014373152	777	district/faith-based	No	03/08/2017	1	0	0
2015419062	777	district/faith-based	No	03/08/2017	1	0	0
2015352657	777	district/faith-based	No	04/08/2017	1	0	0
2014307341	777	district/faith-based	No	03/08/2017	1	0	0
2015364531	777	district/faith-based	No	03/08/2017	1	0	0
2015332031	777	district/faith-based	No	04/08/2017	1	0	0
2015419065	777	district/faith-based	No	03/08/2017	1	0	0
2014365953	777	district/faith-based	No	04/08/2017	1	0	0
2015364533	777	district/faith-based	No	03/08/2017	1	0	0
2015404158	777	district/faith-based	No	04/08/2017	1	0	0
2015364532	777	district/faith-based	No	03/08/2017	1	0	0
2015305094	777	district/faith-based	No	03/08/2017	1	0	0
2011200184	733	rural/clinic	No	03/08/2017	0	0	0
2015305095	733	rural/clinic	No	03/08/2017	0	0	0
2014368488	733	rural/clinic	No	04/08/2017	0	0	0
2015357658	733	rural/clinic	No	03/08/2017	0	0	0
2015305096	66	rural/clinic	No	03/08/2017	0	0	0
2015340717	66	rural/clinic	No	03/08/2017	0	0	0
2012312151	66	rural/clinic	No	03/08/2017	0	0	0
2013264673	66	rural/clinic	No	03/08/2017	0	0	0
2014372449	66	rural/clinic	No	03/08/2017	0	0	0
2015345086	66	rural/clinic	No	03/08/2017	0	0	0
2014372278	66	rural/clinic	No	03/08/2017	0	0	0
2014372448	66	rural/clinic	No	03/08/2017	0	0	0
2015332975	66	rural/clinic	No	03/08/2017	0	0	0
2015345085	66	rural/clinic	No	03/08/2017	0	0	0
2015345087	66	rural/clinic	No	03/08/2017	0	0	0
2012246288	66	rural/clinic	No	03/08/2017	0	0	0
2012294625	66	rural/clinic	No	03/08/2017	0	0	0
2015332976	66	rural/clinic	No	03/08/2017	0	0	0
2015345088	66	rural/clinic	No	03/08/2017	0	0	0
2012361537	66	rural/clinic	No	03/08/2017	0	0	0
2014371713	66	rural/clinic	No	03/08/2017	0	0	0
2014335533	66	rural/clinic	No	04/08/2017	0	0	0
2015355018	66	rural/clinic	No	03/08/2017	0	0	0
2015324132	66	rural/clinic	No	04/08/2017	0	0	0
2015414464	66	rural/clinic	No	03/08/2017	0	0	0
2013266126	66	rural/clinic	No	04/08/2017	0	0	0
2015336655	67	rural/clinic	No	03/08/2017	0	0	0
2015305819	67	rural/clinic	No	03/08/2017	0	0	0
2012361536	67	rural/clinic	No	04/08/2017	0	0	0
2015336653	67	rural/clinic	No	03/08/2017	0	0	0
2015305818	67	rural/clinic	No	03/08/2017	0	0	0
2012294624	67	rural/clinic	No	03/08/2017	0	0	0
2013266125	67	rural/clinic	No	04/08/2017	0	0	0
2011155240	67	rural/clinic	No	03/08/2017	0	0	0

2015326208	67	rural/clinic	No	03/08/2017	0	0	0
2015401702	67	rural/clinic	No	04/08/2017	0	0	0
2015386801	67	rural/clinic	No	03/08/2017	0	0	0
2015368679	67	rural/clinic	No	03/08/2017	0	0	0
2015401701	67	rural/clinic	No	04/08/2017	0	0	0
2015386750	67	rural/clinic	No	03/08/2017	0	0	0
2015368678	67	rural/clinic	No	03/08/2017	0	0	0
2015386802	67	rural/clinic	No	03/08/2017	0	0	0
2015326242	67	rural/clinic	No	04/08/2017	0	0	0
2015326219	67	rural/clinic	No	03/08/2017	0	0	0
2015386749	67	rural/clinic	No	03/08/2017	0	0	0
2015336657	67	rural/clinic	No	04/08/2017	0	0	0
2015326218	67	rural/clinic	No	03/08/2017	0	0	0
2015336656	67	rural/clinic	No	04/08/2017	0	0	0
2015386803	67	rural/clinic	No	03/08/2017	0	0	0
2011133919	67	rural/clinic	No	03/08/2017	0	0	0
2015368675	67	rural/clinic	No	03/08/2017	0	0	0
2015386805	67	rural/clinic	No	03/08/2017	0	0	0
2014346331	67	rural/clinic	No	04/08/2017	0	0	0
2015386806	67	rural/clinic	No	03/08/2017	0	0	0
2015355019	67	rural/clinic	No	03/08/2017	0	0	0
2014372342	67	rural/clinic	No	04/08/2017	0	0	0
2015368676	67	rural/clinic	No	03/08/2017	0	0	0
2014382709	67	rural/clinic	No	03/08/2017	0	0	0
2014378563	67	rural/clinic	No	04/08/2017	0	0	0
2015368677	934	rural/clinic	No	03/08/2017	0	0	0
2014370310	934	rural/clinic	No	03/08/2017	0	0	0
2014372341	934	rural/clinic	No	04/08/2017	0	0	0
2015404156	934	rural/clinic	No	04/08/2017	0	0	0
2015289210	934	rural/clinic	No	03/08/2017	0	0	0
2015344744	934	rural/clinic	No	03/08/2017	0	0	0
2015404157	934	rural/clinic	No	04/08/2017	0	0	0
2015289209	934	rural/clinic	No	03/08/2017	0	0	0
2015402007	934	rural/clinic	No	03/08/2017	0	0	0
2015404155	934	rural/clinic	No	04/08/2017	0	0	0
2015404253	934	rural/clinic	No	04/08/2017	0	0	0
2015344668	934	rural/clinic	No	03/08/2017	0	0	0
2014365952	934	rural/clinic	No	04/08/2017	0	0	0
2015289208	934	rural/clinic	No	03/08/2017	0	0	0
2015404202	934	rural/clinic	No	04/08/2017	0	0	0
2015289207	934	rural/clinic	No	03/08/2017	0	0	0
2015402008	934	rural/clinic	No	03/08/2017	0	0	0
2011140553	934	rural/clinic	No	03/08/2017	0	0	0
2014383985	934	rural/clinic	No	04/08/2017	0	0	0
2015402006	934	rural/clinic	No	03/08/2017	0	0	0
2014375525	934	rural/clinic	No	04/08/2017	0	0	0
2015402009	934	rural/clinic	No	03/08/2017	0	0	0
2015402010	934	rural/clinic	No	03/08/2017	0	0	0
2015289206	934	rural/clinic	No	03/08/2017	0	0	0
2015402011	934	rural/clinic	No	03/08/2017	0	0	0
2015344464	934	rural/clinic	No	03/08/2017	0	0	0
2015369090	934	rural/clinic	No	03/08/2017	0	0	0
2015402012	934	rural/clinic	No	03/08/2017	0	0	0
2015344465	934	rural/clinic	No	03/08/2017	0	0	0
2015369088	934	rural/clinic	No	03/08/2017	0	0	0
2015294627	934	rural/clinic	No	03/08/2017	0	0	0
2015364325	934	rural/clinic	No	03/08/2017	0	0	0
2015369089	934	rural/clinic	No	03/08/2017	0	0	0
2015364326	190	rural/clinic	No	03/08/2017	0	0	0
2015369091	190	rural/clinic	No	03/08/2017	0	0	0
2012381654	190	rural/clinic	No	03/08/2017	0	0	0
2011135678	190	rural/clinic	No	03/08/2017	0	0	0
2015364327	190	rural/clinic	No	03/08/2017	0	0	0
2015294625	190	rural/clinic	No	03/08/2017	0	0	0
2015364328	190	rural/clinic	No	03/08/2017	0	0	0
2015344463	190	rural/clinic	No	03/08/2017	0	0	0
2015364329	190	rural/clinic	No	03/08/2017	0	0	0
2015414271	190	rural/clinic	No	04/08/2017	0	0	0
2011140302	190	rural/clinic	No	03/08/2017	0	0	0
2015321605	190	rural/clinic	No	03/08/2017	0	0	0
2012381653	190	rural/clinic	No	03/08/2017	0	0	0
2014375527	190	rural/clinic	No	04/08/2017	0	0	0
2015321604	190	rural/clinic	No	03/08/2017	0	0	0
2015355152	734	rural/clinic	No	03/08/2017	0	0	0
2012381651	734	rural/clinic	No	03/08/2017	0	0	0
2015414272	734	rural/clinic	No	04/08/2017	0	0	0
2014362843	734	rural/clinic	No	03/08/2017	0	0	0
2012381652	734	rural/clinic	No	03/08/2017	0	0	0
2015377330	734	rural/clinic	No	03/08/2017	0	0	0
2015355926	734	rural/clinic	No	03/08/2017	0	0	0
2014362840	734	rural/clinic	No	03/08/2017	0	0	0
2015355925	734	rural/clinic	No	03/08/2017	0	0	0

2015377187	734	rural/clinic	No	03/08/2017	0	0	0
2014362842	734	rural/clinic	No	03/08/2017	0	0	0
2015377184	734	rural/clinic	No	03/08/2017	0	0	0
2014362841	468	rural/clinic	No	03/08/2017	0	0	0
2015414273	468	rural/clinic	No	04/08/2017	0	0	0
2014375526	468	rural/clinic	No	04/08/2017	0	0	0
2015377185	468	rural/clinic	No	03/08/2017	0	0	0
2015369940	468	rural/clinic	No	04/08/2017	0	0	0
2015340377	468	rural/clinic	No	03/08/2017	0	0	0
2014347034	468	rural/clinic	No	03/08/2017	0	0	0
2014375529	468	rural/clinic	No	04/08/2017	0	0	0
2015340376	468	rural/clinic	No	03/08/2017	0	0	0
2015414274	468	rural/clinic	No	04/08/2017	0	0	0
2012265641	468	rural/clinic	No	03/08/2017	0	0	0
2015340375	468	rural/clinic	No	03/08/2017	0	0	0
2015364319	468	rural/clinic	No	03/08/2017	0	0	0
2015360533	468	rural/clinic	No	04/08/2017	0	0	0
2015406018	468	rural/clinic	No	03/08/2017	0	0	0
2015364315	468	rural/clinic	No	03/08/2017	0	0	0
2015406017	468	rural/clinic	No	03/08/2017	0	0	0
2015406016	468	rural/clinic	No	03/08/2017	0	0	0
2015364317	468	rural/clinic	No	03/08/2017	0	0	0
2015364316	468	rural/clinic	No	03/08/2017	0	0	0
2015364318	468	rural/clinic	No	03/08/2017	0	0	0
2015406015	468	rural/clinic	No	03/08/2017	0	0	0
2014347035	468	rural/clinic	No	03/08/2017	0	0	0
2015406014	468	rural/clinic	No	03/08/2017	0	0	0
2011227013	468	rural/clinic	No	03/08/2017	0	0	0
2015362065	468	rural/clinic	No	03/08/2017	0	0	0
2015335397	468	rural/clinic	No	03/08/2017	0	0	0
2015335398	468	rural/clinic	No	03/08/2017	0	0	0
2015335399	468	rural/clinic	No	03/08/2017	0	0	0
2015335206	533	rural/clinic	No	03/08/2017	0	0	0
2015362064	533	rural/clinic	No	03/08/2017	0	0	0
2011227277	533	rural/clinic	No	03/08/2017	0	0	0
2015362063	533	rural/clinic	No	03/08/2017	0	0	0
2011226489	533	rural/clinic	No	03/08/2017	0	0	0
2015377186	533	rural/clinic	No	03/08/2017	0	0	0
2015326217	533	rural/clinic	No	03/08/2017	0	0	0
2015326210	533	rural/clinic	No	03/08/2017	0	0	0
2015362535	533	rural/clinic	No	03/08/2017	0	0	0
2015360534	533	rural/clinic	No	04/08/2017	0	0	0
2015360532	533	rural/clinic	No	03/08/2017	0	0	0
2012284622	533	rural/clinic	No	03/08/2017	0	0	0
2015349068	533	rural/clinic	No	03/08/2017	0	0	0
2014287990	533	rural/clinic	No	03/08/2017	0	0	0
2014287989	533	rural/clinic	No	03/08/2017	0	0	0
2015349069	533	rural/clinic	No	03/08/2017	0	0	0
2013247138	533	rural/clinic	No	03/08/2017	0	0	0
2015347404	533	rural/clinic	No	03/08/2017	0	0	0
2015360207	533	rural/clinic	No	03/08/2017	0	0	0
2015360208	533	rural/clinic	No	03/08/2017	0	0	0
2014362844	533	rural/clinic	No	03/08/2017	0	0	0
2015338879	533	rural/clinic	No	03/08/2017	0	0	0
2015355016	533	rural/clinic	No	03/08/2017	0	0	0
2014362838	533	rural/clinic	No	03/08/2017	0	0	0
2015326603	533	rural/clinic	No	03/08/2017	0	0	0
2011133916	533	rural/clinic	No	03/08/2017	0	0	0
2014362845	533	rural/clinic	No	03/08/2017	0	0	0
2014362839	533	rural/clinic	No	03/08/2017	0	0	0
2015352786	533	rural/clinic	No	03/08/2017	0	0	0
2015352785	533	rural/clinic	No	03/08/2017	0	0	0
2014333606	533	rural/clinic	No	03/08/2017	0	0	0
2012275219	533	rural/clinic	No	03/08/2017	0	0	0
2015352784	533	rural/clinic	No	03/08/2017	0	0	0
2015352783	533	rural/clinic	No	03/08/2017	0	0	0
2014362837	533	rural/clinic	No	03/08/2017	0	0	0
2012275220	533	rural/clinic	No	03/08/2017	0	0	0
2015352782	533	rural/clinic	No	03/08/2017	0	0	0
2014382708	533	rural/clinic	No	03/08/2017	0	0	0
2015352781	533	rural/clinic	No	03/08/2017	0	0	0
2014382707	533	rural/clinic	No	03/08/2017	0	0	0
2015362536	533	rural/clinic	No	03/08/2017	0	0	0
2015352779	533	rural/clinic	No	03/08/2017	0	0	0
2015352780	533	rural/clinic	No	03/08/2017	0	0	0
2012369819	533	rural/clinic	No	03/08/2017	0	0	0
2015362537	533	rural/clinic	No	03/08/2017	0	0	0
2015364320	533	rural/clinic	No	03/08/2017	0	0	0
2012369816	533	rural/clinic	No	03/08/2017	0	0	0
2015364321	533	rural/clinic	No	03/08/2017	0	0	0
2014370311	533	rural/clinic	No	03/08/2017	0	0	0
2014327692	533	rural/clinic	No	03/08/2017	0	0	0

2014314232	533	rural/clinic	No	03/08/2017	0	0	0
2011135679	533	rural/clinic	No	03/08/2017	0	0	0
2015344665	533	rural/clinic	No	03/08/2017	0	0	0
2015364322	533	rural/clinic	No	03/08/2017	0	0	0
2015364323	533	rural/clinic	No	03/08/2017	0	0	0
2015364324	511	rural/clinic	No	03/08/2017	0	0	0
2015372821	511	rural/clinic	No	03/08/2017	0	0	0
2015286713	511	rural/clinic	No	03/08/2017	0	0	0
2014358228	511	rural/clinic	No	03/08/2017	0	0	0
2014314231	511	rural/clinic	No	03/08/2017	0	0	0
2015404975	511	rural/clinic	No	03/08/2017	0	0	0
2015344666	511	rural/clinic	No	03/08/2017	0	0	0
2015404976	511	rural/clinic	No	03/08/2017	0	0	0
2015344667	511	rural/clinic	No	03/08/2017	0	0	0
2012265926	511	rural/clinic	No	03/08/2017	0	0	0
2015334804	511	rural/clinic	No	03/08/2017	0	0	0
2012291379	511	rural/clinic	No	03/08/2017	0	0	0
2015334806	511	rural/clinic	No	03/08/2017	0	0	0
2011124088	511	rural/clinic	No	03/08/2017	0	0	0
2014360973	511	rural/clinic	No	03/08/2017	0	0	0
2015334807	511	rural/clinic	No	03/08/2017	0	0	0
2014357826	511	rural/clinic	No	03/08/2017	0	0	0
2015344461	511	rural/clinic	No	03/08/2017	0	0	0
2014357827	511	rural/clinic	No	03/08/2017	0	0	0
2012265925	511	rural/clinic	No	03/08/2017	0	0	0
2015344462	511	rural/clinic	No	03/08/2017	0	0	0
2011141109	511	rural/clinic	No	06/08/2017	0	0	0
2014357830	511	rural/clinic	No	03/08/2017	0	0	0
2011141110	511	rural/clinic	No	03/08/2017	0	0	0
2014357829	511	rural/clinic	No	03/08/2017	0	0	0
201053918	511	rural/clinic	No	03/08/2017	0	0	0
201053917	511	rural/clinic	No	03/08/2017	0	0	0
2015377438	511	rural/clinic	No	03/08/2017	0	0	0
2015361176	511	rural/clinic	No	03/08/2017	0	0	0
2014357828	511	rural/clinic	No	03/08/2017	0	0	0
2015384363	511	rural/clinic	No	03/08/2017	0	0	0
2015361177	511	rural/clinic	No	03/08/2017	0	0	0
2014368693	511	rural/clinic	No	03/08/2017	0	0	0
2012275216	511	rural/clinic	No	03/08/2017	0	0	0
2012275217	511	rural/clinic	No	03/08/2017	0	0	0
2015332368	511	rural/clinic	No	03/08/2017	0	0	0
2012275218	511	rural/clinic	No	03/08/2017	0	0	0
2014368694	511	rural/clinic	No	03/08/2017	0	0	0
2014307419	511	rural/clinic	No	03/08/2017	0	0	0
2015332366	511	rural/clinic	No	03/08/2017	0	0	0
2014357831	511	rural/clinic	No	03/08/2017	0	0	0
2015332367	511	rural/clinic	No	03/08/2017	0	0	0
2013285131	511	rural/clinic	No	03/08/2017	0	0	0
2015332365	511	rural/clinic	No	03/08/2017	0	0	0
2013285129	511	rural/clinic	No	03/08/2017	0	0	0
2015361178	511	rural/clinic	No	03/08/2017	0	0	0
2014357335	511	rural/clinic	No	03/08/2017	0	0	0
2011135065	511	rural/clinic	No	03/08/2017	0	0	0
2012346721	511	rural/clinic	No	03/08/2017	0	0	0
2011205728	511	rural/clinic	No	03/08/2017	0	0	0
2015361179	511	rural/clinic	No	03/08/2017	0	0	0
2011135066	337	district/faith-based	No	03/08/2017	1	0	0
2012346722	337	district/faith-based	No	03/08/2017	1	0	0
2015361180	337	district/faith-based	No	03/08/2017	1	0	0
2012346652	337	district/faith-based	No	03/08/2017	1	0	0
2014357334	337	district/faith-based	No	03/08/2017	1	0	0
2011135067	337	district/faith-based	No	03/08/2017	1	0	0
2015362539	337	district/faith-based	No	03/08/2017	1	0	0
2015362538	337	district/faith-based	No	03/08/2017	1	0	0
2011135068	337	district/faith-based	No	03/08/2017	1	0	0
2015378207	337	district/faith-based	No	03/08/2017	1	0	0
2015414270	337	district/faith-based	No	04/08/2017	1	0	0
2015297296	337	district/faith-based	No	03/08/2017	1	0	0
2015378213	337	district/faith-based	No	03/08/2017	1	0	0
2012363981	337	district/faith-based	No	03/08/2017	1	0	0
2012363982	337	district/faith-based	No	03/08/2017	1	0	0
2015338874	337	district/faith-based	No	03/08/2017	1	0	0
2014375528	337	district/faith-based	No	04/08/2017	1	0	0
2015338875	337	district/faith-based	No	03/08/2017	1	0	0
2015362827	337	district/faith-based	No	03/08/2017	1	0	0
2013285130	337	district/faith-based	No	03/08/2017	1	0	0
2015362828	337	district/faith-based	No	03/08/2017	1	0	0
2014370440	337	district/faith-based	No	03/08/2017	1	0	0
2015332704	337	district/faith-based	No	03/08/2017	1	0	0
2015402653	337	district/faith-based	No	03/08/2017	1	0	0
2015338876	337	district/faith-based	No	03/08/2017	1	0	0
2015402594	337	district/faith-based	No	03/08/2017	1	0	0

2015402595	337	district/faith-based	No	03/08/2017	1	0	0
2015338877	337	district/faith-based	No	03/08/2017	1	0	0
2015402596	337	district/faith-based	No	03/08/2017	1	0	0
2015373882	337	district/faith-based	No	04/08/2017	1	0	0
2015402597	337	district/faith-based	No	03/08/2017	1	0	0
2012363404	337	district/faith-based	No	04/08/2017	1	0	0
2015402598	337	district/faith-based	No	03/08/2017	1	0	0
2015402599	337	district/faith-based	No	03/08/2017	1	0	0
2012363405	337	district/faith-based	No	04/08/2017	1	0	0
2015402600	337	district/faith-based	No	03/08/2017	1	0	0
2012363406	337	district/faith-based	No	04/08/2017	1	0	0
2015402651	337	district/faith-based	No	03/08/2017	1	0	0
2015402652	337	district/faith-based	No	03/08/2017	1	0	0
2015402654	337	district/faith-based	No	03/08/2017	1	0	0
2012344083	337	district/faith-based	No	03/08/2017	1	0	0
2014368488	337	district/faith-based	No	04/08/2017	1	0	0
2014368488	337	district/faith-based	No	04/08/2017	1	0	0
2012361536	337	district/faith-based	No	04/08/2017	1	0	0
2012361536	337	district/faith-based	No	04/08/2017	1	0	0
2012388286	337	district/faith-based	No	03/08/2017	1	0	0
2012388286	337	district/faith-based	No	03/08/2017	1	0	0
2015419063	337	district/faith-based	No	03/08/2017	1	0	0
2015419063	337	district/faith-based	No	03/08/2017	1	0	0
2015338880	338	rural/clinic	No	03/08/2017	0	0	0
2015338880	338	rural/clinic	No	03/08/2017	0	0	0
2015373881	338	rural/clinic	No	01/08/2017	0	0	0
2015373881	338	rural/clinic	No	01/08/2017	0	0	0
2015373878	338	rural/clinic	No	01/08/2017	0	0	0
2015373878	338	rural/clinic	No	01/08/2017	0	0	0
2015336102	338	rural/clinic	No	03/08/2017	0	0	0
2015336102	338	rural/clinic	No	03/08/2017	0	0	0
2014372278	338	rural/clinic	No	03/08/2017	0	0	0
2014372278	338	rural/clinic	No	03/08/2017	0	0	0
2015414464	338	rural/clinic	No	03/08/2017	0	0	0
2015414464	338	rural/clinic	No	03/08/2017	0	0	0
2015386749	338	rural/clinic	No	03/08/2017	0	0	0
2015386749	338	rural/clinic	No	03/08/2017	0	0	0
2012363409	338	rural/clinic	No	04/08/2017	0	0	0
2012363409	338	rural/clinic	No	04/08/2017	0	0	0
2015297292	338	rural/clinic	No	03/08/2017	0	0	0
2015297292	338	rural/clinic	No	03/08/2017	0	0	0
2015305094	338	rural/clinic	No	03/08/2017	0	0	0
2015305094	338	rural/clinic	No	03/08/2017	0	0	0
2015305096	338	rural/clinic	No	03/08/2017	0	0	0
2015305096	338	rural/clinic	No	03/08/2017	0	0	0
2015402009	338	rural/clinic	No	03/08/2017	0	0	0
2015377330	338	rural/clinic	No	03/08/2017	0	0	0
2015334804	338	rural/clinic	No	03/08/2017	0	0	0
2015336656	338	rural/clinic	No	04/08/2017	0	0	0
2015336656	338	rural/clinic	No	04/08/2017	0	0	0
2015364323	338	rural/clinic	No	03/08/2017	0	0	0
2015364323	338	rural/clinic	No	03/08/2017	0	0	0
2015402652	338	rural/clinic	No	03/08/2017	0	0	0
2015402652	338	rural/clinic	No	03/08/2017	0	0	0
2012265925	338	rural/clinic	No	03/08/2017	0	0	0
2012265925	338	rural/clinic	No	03/08/2017	0	0	0
2015397347	338	rural/clinic	No	08/08/2017	0	0	0
2015397348	338	rural/clinic	No	08/08/2017	0	0	0
2015397349	338	rural/clinic	No	08/08/2017	0	0	0
2015397651	338	rural/clinic	No	08/08/2017	0	0	0
2015397652	338	rural/clinic	No	08/08/2017	0	0	0
2015397653	338	rural/clinic	No	08/08/2017	0	0	0
2015397654	338	rural/clinic	No	08/08/2017	0	0	0
2015373788	338	rural/clinic	No	08/08/2017	0	0	0
2015373790	338	rural/clinic	No	08/08/2017	0	0	0
2015373791	338	rural/clinic	No	08/08/2017	0	0	0
2015373793	338	rural/clinic	No	08/08/2017	0	0	0
2015403710	338	rural/clinic	No	08/08/2017	0	0	0
2014377419	338	rural/clinic	No	08/08/2017	0	0	0
2015373792	212	rural/clinic	No	08/08/2017	0	0	0
2015373794	212	rural/clinic	No	08/08/2017	0	0	0
2015373795	212	rural/clinic	No	08/08/2017	0	0	0
2015373796	212	rural/clinic	No	08/08/2017	0	0	0
2015373797	588	rural/clinic	No	08/08/2017	0	0	0
2015373798	588	rural/clinic	No	08/08/2017	0	0	0
2015373799	588	rural/clinic	No	08/08/2017	0	0	0
2015373800	588	rural/clinic	No	08/08/2017	0	0	0
2015378151	588	rural/clinic	No	08/08/2017	0	0	0
2015378153	588	rural/clinic	No	08/08/2017	0	0	0
2015378154	588	rural/clinic	No	08/08/2017	0	0	0
2015378155	588	rural/clinic	No	08/08/2017	0	0	0
2015378737	588	rural/clinic	No	08/08/2017	0	0	0

2011195786	588	rural/clinic	No	08/08/2017	0	0	0
2015287393	588	rural/clinic	No	08/08/2017	0	0	0
2015342321	588	rural/clinic	No	08/08/2017	0	0	0
2015406338	588	rural/clinic	No	08/08/2017	0	0	0
2015412336	588	rural/clinic	No	08/08/2017	0	0	0
2015406337	588	rural/clinic	No	08/08/2017	0	0	0
2015412334	588	rural/clinic	No	08/08/2017	0	0	0
2015406331	588	rural/clinic	No	08/08/2017	0	0	0
2015406332	588	rural/clinic	No	08/08/2017	0	0	0
2015406333	588	rural/clinic	No	08/08/2017	0	0	0
2015406334	588	rural/clinic	No	08/08/2017	0	0	0
2015406335	588	rural/clinic	No	08/08/2017	0	0	0
2015412335	588	rural/clinic	No	08/08/2017	0	0	0
2015383750	588	rural/clinic	No	08/08/2017	0	0	0
2015333451	588	rural/clinic	No	08/08/2017	0	0	0
2012305594	588	rural/clinic	No	08/08/2017	0	0	0
2015367710	588	rural/clinic	No	08/08/2017	0	0	0
2015367709	588	rural/clinic	No	08/08/2017	0	0	0
2015412457	588	rural/clinic	No	08/08/2017	0	0	0
2015367708	588	rural/clinic	No	08/08/2017	0	0	0
2015301770	588	rural/clinic	No	08/08/2017	0	0	0
2015367711	588	rural/clinic	No	08/08/2017	0	0	0
2012305595	588	rural/clinic	No	08/08/2017	0	0	0
2015301771	588	rural/clinic	No	08/08/2017	0	0	0
2015301767	588	rural/clinic	No	08/08/2017	0	0	0
2015297170	588	rural/clinic	No	08/08/2017	0	0	0
2015297171	588	rural/clinic	No	08/08/2017	0	0	0
2015301769	588	rural/clinic	No	08/08/2017	0	0	0
2015301768	588	rural/clinic	No	08/08/2017	0	0	0
2014374042	588	rural/clinic	No	08/08/2017	0	0	0
2014374044	588	rural/clinic	No	08/08/2017	0	0	0
2014374041	588	rural/clinic	No	08/08/2017	0	0	0
2014374040	588	rural/clinic	No	08/08/2017	0	0	0
2014374043	588	rural/clinic	No	08/08/2017	0	0	0
2014298243	588	rural/clinic	No	08/08/2017	0	0	0
2013256301	588	rural/clinic	No	08/08/2017	0	0	0
2015368611	588	rural/clinic	No	08/08/2017	0	0	0
2015368612	588	rural/clinic	No	08/08/2017	0	0	0
2015368613	588	rural/clinic	No	08/08/2017	0	0	0
2015364954	588	rural/clinic	No	08/08/2017	0	0	0
2012289896	588	rural/clinic	No	08/08/2017	0	0	0
2015364955	588	rural/clinic	No	08/08/2017	0	0	0
2014338457	588	rural/clinic	No	08/08/2017	0	0	0
2015364953	588	rural/clinic	No	08/08/2017	0	0	0
2015286751	588	rural/clinic	No	08/08/2017	0	0	0
2015355532	588	rural/clinic	No	08/08/2017	0	0	0
2013254812	588	rural/clinic	No	08/08/2017	0	0	0
2012276260	588	rural/clinic	No	08/08/2017	0	0	0
2012276263	588	rural/clinic	No	08/08/2017	0	0	0
2012276259	588	rural/clinic	No	08/08/2017	0	0	0
2014363899	588	rural/clinic	No	08/08/2017	0	0	0
2014338456	588	rural/clinic	No	08/08/2017	0	0	0
2014338455	588	rural/clinic	No	08/08/2017	0	0	0
2014350370	588	rural/clinic	No	08/08/2017	0	0	0
2015337532	588	rural/clinic	No	08/08/2017	0	0	0
2015413364	588	rural/clinic	No	08/08/2017	0	0	0
2014363195	778	rural/clinic	No	08/08/2017	0	0	0
2015303091	778	rural/clinic	No	08/08/2017	0	0	0
2015414269	778	rural/clinic	No	25/07/2017	0	0	0
2015363084	778	rural/clinic	No	08/08/2017	0	0	0
2015337632	778	rural/clinic	No	08/08/2017	0	0	0
2015414268	778	rural/clinic	No	25/07/2017	0	0	0
2015363083	778	rural/clinic	No	08/08/2017	0	0	0
201066588	778	rural/clinic	No	08/08/2017	0	0	0
2011152230	778	rural/clinic	No	08/08/2017	0	0	0
2011152229	778	rural/clinic	No	08/08/2017	0	0	0
2015403181	778	rural/clinic	No	25/07/2017	0	0	0
2012330891	778	rural/clinic	No	08/08/2017	0	0	0
2015324046	778	rural/clinic	No	08/08/2017	0	0	0
2015355528	778	rural/clinic	No	19/07/2017	0	0	0
2015363019	778	rural/clinic	No	08/08/2017	0	0	0
2014365076	778	rural/clinic	No	25/07/2017	0	0	0
2015337379	778	rural/clinic	No	08/08/2017	0	0	0
2013271849	778	rural/clinic	No	25/07/2017	0	0	0
2015413363	778	rural/clinic	No	08/08/2017	0	0	0
2015384789	778	rural/clinic	No	08/08/2017	0	0	0
2015406336	778	rural/clinic	No	08/08/2017	0	0	0
2011133155	778	rural/clinic	No	08/08/2017	0	0	0
2011152231	641	rural/clinic	No	08/08/2017	0	0	0
2015405917	641	rural/clinic	No	08/08/2017	0	0	0
2014350681	641	rural/clinic	No	08/08/2017	0	0	0
2012330889	641	rural/clinic	No	08/08/2017	0	0	0

2015384492	641	rural/clinic	No	08/08/2017	0	0	0
2014365396	641	rural/clinic	No	08/08/2017	0	0	0
2014365395	641	rural/clinic	No	08/08/2017	0	0	0
2013264079	641	rural/clinic	No	08/08/2017	0	0	0
2013264078	641	rural/clinic	No	08/08/2017	0	0	0
2015337705	346	district/faith-based	No	08/08/2017	1	0	0
2015337703	346	district/faith-based	No	08/08/2017	1	0	0
2013264077	346	district/faith-based	No	08/08/2017	1	0	0
2015337704	346	district/faith-based	No	08/08/2017	1	0	0
2012323780	346	district/faith-based	No	08/08/2017	1	0	0
2015355533	346	district/faith-based	No	08/08/2017	1	0	0
2014299922	346	district/faith-based	No	08/08/2017	1	0	0
2014299926	346	district/faith-based	No	08/08/2017	1	0	0
2014299925	346	district/faith-based	No	08/08/2017	1	0	0
2014299923	620	rural/clinic	No	08/08/2017	0	0	0
2014299921	620	rural/clinic	No	08/08/2017	0	0	0
2015385861	620	rural/clinic	No	08/08/2017	0	0	0
2015355531	620	rural/clinic	No	08/08/2017	0	0	0
2015337531	620	rural/clinic	No	08/08/2017	0	0	0
2014299924	620	rural/clinic	No	08/08/2017	0	0	0
2015315915	620	rural/clinic	No	08/08/2017	0	0	0
2015337377	620	rural/clinic	No	08/08/2017	0	0	0
2013244118	620	rural/clinic	No	08/08/2017	0	0	0
2015314614	620	rural/clinic	No	08/08/2017	0	0	0
2014338316	620	rural/clinic	No	08/08/2017	0	0	0
2015324485	146	rural/clinic	No	08/08/2017	0	0	0
2014350369	146	rural/clinic	No	08/08/2017	0	0	0
2014367630	146	rural/clinic	No	08/08/2017	0	0	0
2015339607	367	rural/clinic	No	08/08/2017	0	0	0
2015294891	367	rural/clinic	No	08/08/2017	0	0	0
2015337534	367	rural/clinic	No	08/08/2017	0	0	0
2015294890	367	rural/clinic	No	08/08/2017	0	0	0
2015339608	367	rural/clinic	No	08/08/2017	0	0	0
2015384788	367	rural/clinic	No	08/08/2017	0	0	0
2014343880	367	rural/clinic	No	08/08/2017	0	0	0
2015337533	367	rural/clinic	No	08/08/2017	0	0	0
2014367629	367	rural/clinic	No	08/08/2017	0	0	0
2015294889	367	rural/clinic	No	08/08/2017	0	0	0
2015315003	367	rural/clinic	No	08/08/2017	0	0	0
2015294892	367	rural/clinic	No	08/08/2017	0	0	0
2014375523	367	rural/clinic	No	08/08/2017	0	0	0
2015289340	367	rural/clinic	No	08/08/2017	0	0	0
2015315005	367	rural/clinic	No	08/08/2017	0	0	0
2014340201	367	rural/clinic	No	08/08/2017	0	0	0
2014372770	367	rural/clinic	No	08/08/2017	0	0	0
2015315004	367	rural/clinic	No	08/08/2017	0	0	0
2015295198	367	rural/clinic	No	08/08/2017	0	0	0
2014371166	367	rural/clinic	No	08/08/2017	0	0	0
2015337631	367	rural/clinic	No	08/08/2017	0	0	0
2015314615	213	rural/clinic	No	08/08/2017	0	0	0
2015314616	213	rural/clinic	No	08/08/2017	0	0	0
2012344865	213	rural/clinic	No	08/08/2017	0	0	0
2012344866	213	rural/clinic	No	08/08/2017	0	0	0
2015355534	213	rural/clinic	No	08/08/2017	0	0	0
2015324486	147	rural/clinic	No	08/08/2017	0	0	0
2014340204	147	rural/clinic	No	08/08/2017	0	0	0
2015326910	147	rural/clinic	No	08/08/2017	0	0	0
2015326911	147	rural/clinic	No	08/08/2017	0	0	0
2015326905	147	rural/clinic	No	08/08/2017	0	0	0
2015326906	147	rural/clinic	No	08/08/2017	0	0	0
2015326907	147	rural/clinic	No	08/08/2017	0	0	0
2015326908	147	rural/clinic	No	08/08/2017	0	0	0
2015335979	147	rural/clinic	No	08/08/2017	0	0	0
2015335978	147	rural/clinic	No	08/08/2017	0	0	0
2015326909	147	rural/clinic	No	08/08/2017	0	0	0
2011222231	147	rural/clinic	No	08/08/2017	0	0	0
2015335977	147	rural/clinic	No	08/08/2017	0	0	0
2015325187	147	rural/clinic	No	08/08/2017	0	0	0
2012312206	147	rural/clinic	No	08/08/2017	0	0	0
2015363156	147	rural/clinic	No	08/08/2017	0	0	0
2015325186	147	rural/clinic	No	08/08/2017	0	0	0
2015363157	147	rural/clinic	No	08/08/2017	0	0	0
2015325221	147	rural/clinic	No	08/08/2017	0	0	0
2015340903	147	rural/clinic	No	08/08/2017	0	0	0
2014383710	147	rural/clinic	No	08/08/2017	0	0	0
2014363900	147	rural/clinic	No	08/08/2017	0	0	0
2014320300	147	rural/clinic	No	08/08/2017	0	0	0
2012312207	147	rural/clinic	No	08/08/2017	0	0	0
201066589	147	rural/clinic	No	08/08/2017	0	0	0
2014327530	147	rural/clinic	No	08/08/2017	0	0	0
2015385862	147	rural/clinic	No	08/08/2017	0	0	0
2014327529	147	rural/clinic	No	08/08/2017	0	0	0

2015385863	147	rural/clinic	No	08/08/2017	0	0	0
2015403313	147	rural/clinic	No	07/08/2017	0	0	0
2015355530	147	rural/clinic	No	08/08/2017	0	0	0
2014327130	147	rural/clinic	No	07/08/2017	0	0	0
2014327528	147	rural/clinic	No	08/08/2017	0	0	0
2011144840	147	rural/clinic	No	08/08/2017	0	0	0
2015372858	147	rural/clinic	No	08/08/2017	0	0	0
2014327527	147	rural/clinic	No	08/08/2017	0	0	0
2015372859	406	rural/clinic	No	08/08/2017	0	0	0
2015368735	406	rural/clinic	No	08/08/2017	0	0	0
2011144841	406	rural/clinic	No	08/08/2017	0	0	0
2015368734	406	rural/clinic	No	08/08/2017	0	0	0
2015368737	406	rural/clinic	No	08/08/2017	0	0	0
2015373885	406	rural/clinic	No	08/08/2017	0	0	0
2015403703	406	rural/clinic	No	08/08/2017	0	0	0
2015405630	406	rural/clinic	No	08/08/2017	0	0	0
2014336942	406	rural/clinic	No	08/08/2017	0	0	0
2015403704	406	rural/clinic	No	08/08/2017	0	0	0
2015321603	406	rural/clinic	No	08/08/2017	0	0	0
2015403705	406	rural/clinic	No	08/08/2017	0	0	0
2015403706	406	rural/clinic	No	08/08/2017	0	0	0
2015403707	406	rural/clinic	No	08/08/2017	0	0	0
2015403708	406	rural/clinic	No	08/08/2017	0	0	0
2014403709	406	rural/clinic	No	08/08/2017	0	0	0
2015355529	406	rural/clinic	No	08/08/2017	0	0	0
2015347206	406	rural/clinic	No	08/08/2017	0	0	0
2014291548	406	rural/clinic	No	08/08/2017	0	0	0
2014291549	406	rural/clinic	No	08/08/2017	0	0	0
2015367230	406	rural/clinic	No	08/08/2017	0	0	0
2014291550	406	rural/clinic	No	08/08/2017	0	0	0
2015337530	406	rural/clinic	No	08/08/2017	0	0	0
2015337529	406	rural/clinic	No	08/08/2017	0	0	0
2015321451	406	rural/clinic	No	08/08/2017	0	0	0
2015337528	406	rural/clinic	No	08/08/2017	0	0	0
2015367227	406	rural/clinic	No	08/08/2017	0	0	0
2015368736	406	rural/clinic	No	08/08/2017	0	0	0
2011144842	406	rural/clinic	No	08/08/2017	0	0	0
2015367229	406	rural/clinic	No	08/08/2017	0	0	0
2015303099	406	rural/clinic	No	08/08/2017	0	0	0
2015407101	406	rural/clinic	No	08/08/2017	0	0	0
2015303100	406	rural/clinic	No	08/08/2017	0	0	0
2015407102	406	rural/clinic	No	08/08/2017	0	0	0
2015314035	406	rural/clinic	No	08/08/2017	0	0	0
2015382959	406	rural/clinic	No	08/08/2017	0	0	0
2015382958	406	rural/clinic	No	08/08/2017	0	0	0
2012365649	406	rural/clinic	No	08/08/2017	0	0	0
2015360742	406	rural/clinic	No	08/08/2017	0	0	0
2013249284	406	rural/clinic	No	08/08/2017	0	0	0
2012365650	406	rural/clinic	No	08/08/2017	0	0	0
2014319636	406	rural/clinic	No	08/08/2017	0	0	0
2011144839	406	rural/clinic	No	08/08/2017	0	0	0
2012305829	406	rural/clinic	No	08/08/2017	0	0	0
2011143088	406	rural/clinic	No	08/08/2017	0	0	0
2015405632	406	rural/clinic	No	08/08/2017	0	0	0
2012305828	406	rural/clinic	No	08/08/2017	0	0	0
2015342059	406	rural/clinic	No	08/08/2017	0	0	0
2012253244	406	rural/clinic	No	08/08/2017	0	0	0
2015342058	406	rural/clinic	No	08/08/2017	0	0	0
2015342060	406	rural/clinic	No	08/08/2017	0	0	0
2015390321	406	rural/clinic	No	08/08/2017	0	0	0
2015337378	406	rural/clinic	No	01/08/2017	0	0	0
2015390322	406	rural/clinic	No	08/08/2017	0	0	0
2015303090	406	rural/clinic	No	21/06/2017	0	0	0
2015326986	406	rural/clinic	No	08/08/2017	0	0	0
2015373789	433	rural/clinic	No	08/08/2017	0	0	0
2015326987	433	rural/clinic	No	08/08/2017	0	0	0
2015367228	433	rural/clinic	No	08/08/2017	0	0	0
2015377756	433	rural/clinic	No	08/08/2017	0	0	0
2015390265	433	rural/clinic	No	31/07/2017	0	0	0
2015377757	433	rural/clinic	No	08/08/2017	0	0	0
2015363153	433	rural/clinic	No	08/08/2017	0	0	0
2015363155	433	rural/clinic	No	08/08/2017	0	0	0
2015363154	433	rural/clinic	No	08/08/2017	0	0	0
2015319302	433	rural/clinic	No	08/08/2017	0	0	0
2013244118	433	rural/clinic	No	08/08/2017	0	0	0
2013244118	433	rural/clinic	No	08/08/2017	0	0	0
2014375524	433	rural/clinic	No	08/08/2017	0	0	0
2014375524	433	rural/clinic	No	08/08/2017	0	0	0
2014375521	433	rural/clinic	No	08/08/2017	0	0	0
2014375521	433	rural/clinic	No	08/08/2017	0	0	0
2011222231	433	rural/clinic	No	08/08/2017	0	0	0
2011222231	433	rural/clinic	No	08/08/2017	0	0	0

2015326908	433	rural/clinic	No	08/08/2017	0	0	0
2015326908	433	rural/clinic	No	08/08/2017	0	0	0
2012289899	433	rural/clinic	No	08/08/2017	0	0	0
2012289899	433	rural/clinic	No	08/08/2017	0	0	0
2012330889	433	rural/clinic	No	08/08/2017	0	0	0
2012330889	433	rural/clinic	No	08/08/2017	0	0	0
2014365395	433	rural/clinic	No	08/08/2017	0	0	0
2014365395	433	rural/clinic	No	08/08/2017	0	0	0
2015417087	433	rural/clinic	No	10/08/2017	0	0	0
2015344368	433	rural/clinic	No	10/08/2017	0	0	0
2011226492	433	rural/clinic	No	10/08/2017	0	0	0
2015364333	433	rural/clinic	No	10/08/2017	0	0	0
2014325210	433	rural/clinic	No	10/08/2017	0	0	0
2013267898	433	rural/clinic	No	10/08/2017	0	0	0
2015418760	433	rural/clinic	No	10/08/2017	0	0	0
2015418759	433	rural/clinic	No	10/08/2017	0	0	0
2015418761	433	rural/clinic	No	10/08/2017	0	0	0
2014317520	433	rural/clinic	No	10/08/2017	0	0	0
2015331676	433	rural/clinic	No	10/08/2017	0	0	0
2015331677	433	rural/clinic	No	10/08/2017	0	0	0
2015360860	433	rural/clinic	No	10/08/2017	0	0	0
2015331675	433	rural/clinic	No	28/06/2017	0	0	0
2012379347	433	rural/clinic	No	10/08/2017	0	0	0
2014297844	433	rural/clinic	No	10/08/2017	0	0	0
2014297845	433	rural/clinic	No	10/08/2017	0	0	0
2013283507	433	rural/clinic	No	10/08/2017	0	0	0
2012379349	433	rural/clinic	No	10/08/2017	0	0	0
2012244292	433	rural/clinic	No	10/08/2017	0	0	0
2015303650	433	rural/clinic	No	10/08/2017	0	0	0
2015303651	433	rural/clinic	No	10/08/2017	0	0	0
2015369095	433	rural/clinic	No	10/08/2017	0	0	0
2015369096	433	rural/clinic	No	10/08/2017	0	0	0
2015369094	433	rural/clinic	No	10/08/2017	0	0	0
2015369093	433	rural/clinic	No	10/08/2017	0	0	0
2015402013	433	rural/clinic	No	10/08/2017	0	0	0
2015402016	433	rural/clinic	No	10/08/2017	0	0	0
2015402015	433	rural/clinic	No	10/08/2017	0	0	0
2015402014	433	rural/clinic	No	10/08/2017	0	0	0
2015305820	433	rural/clinic	No	10/08/2017	0	0	0
2011223942	675	rural/clinic	No	10/08/2017	0	0	0
2011223941	675	rural/clinic	No	10/08/2017	0	0	0
2015349604	675	rural/clinic	No	10/08/2017	0	0	0
2015349603	675	rural/clinic	No	10/08/2017	0	0	0
2015349608	675	rural/clinic	No	10/08/2017	0	0	0
2015349605	675	rural/clinic	No	10/08/2017	0	0	0
2015349607	675	rural/clinic	No	10/08/2017	0	0	0
2015302153	675	rural/clinic	No	10/08/2017	0	0	0
2015349464	675	rural/clinic	No	10/08/2017	0	0	0
2013264223	675	rural/clinic	No	10/08/2017	0	0	0
2015384365	675	rural/clinic	No	10/08/2017	0	0	0
2015384364	675	rural/clinic	No	10/08/2017	0	0	0
2013264222	675	rural/clinic	No	10/08/2017	0	0	0
2011124089	675	rural/clinic	No	10/08/2017	0	0	0
2014340206	675	rural/clinic	No	10/08/2017	0	0	0
2012344506	675	rural/clinic	No	10/08/2017	0	0	0
2012344084	675	rural/clinic	No	10/08/2017	0	0	0
2014340205	675	rural/clinic	No	10/08/2017	0	0	0
2015382578	675	rural/clinic	No	10/08/2017	0	0	0
2015289216	675	rural/clinic	No	10/08/2017	0	0	0
2015382579	675	rural/clinic	No	10/08/2017	0	0	0
2014340207	675	rural/clinic	No	10/08/2017	0	0	0
2015382580	675	rural/clinic	No	10/08/2017	0	0	0
2015369750	675	rural/clinic	No	10/08/2017	0	0	0
2015328095	675	rural/clinic	No	10/08/2017	0	0	0
2015382581	675	rural/clinic	No	10/08/2017	0	0	0
2011140785	675	rural/clinic	No	10/08/2017	0	0	0
2015328097	675	rural/clinic	No	10/08/2017	0	0	0
2015382582	675	rural/clinic	No	10/08/2017	0	0	0
2015328099	675	rural/clinic	No	10/08/2017	0	0	0
2011140793	675	rural/clinic	No	10/08/2017	0	0	0
2015286340	675	rural/clinic	No	10/08/2017	0	0	0
2015328098	675	rural/clinic	No	10/08/2017	0	0	0
2014327695	675	rural/clinic	No	10/08/2017	0	0	0
2015305097	675	rural/clinic	No	10/08/2017	0	0	0
2011140794	675	rural/clinic	No	10/08/2017	0	0	0
2014327696	675	rural/clinic	No	10/08/2017	0	0	0
2015345089	675	rural/clinic	No	10/08/2017	0	0	0
2011140791	675	rural/clinic	No	10/08/2017	0	0	0
2012275221	675	rural/clinic	No	10/08/2017	0	0	0
2015345090	779	district/faith-based	No	10/08/2017	1	0	0
2014370312	779	district/faith-based	No	10/08/2017	1	0	0
2011140783	779	district/faith-based	No	10/08/2017	1	0	0

2011117859	779	district/faith-based	No	10/08/2017	1	0	0
2015361181	779	district/faith-based	No	10/08/2017	1	0	0
2011135070	779	district/faith-based	No	10/08/2017	1	0	0
2011117860	779	district/faith-based	No	10/08/2017	1	0	0
2011135069	779	district/faith-based	No	10/08/2017	1	0	0
2011117858	779	district/faith-based	No	10/08/2017	1	0	0
2015352789	779	district/faith-based	No	10/08/2017	1	0	0
2011140784	779	district/faith-based	No	10/08/2017	1	0	0
2015352788	779	district/faith-based	No	10/08/2017	1	0	0
2011117857	779	district/faith-based	No	10/08/2017	1	0	0
2011140789	779	district/faith-based	No	10/08/2017	1	0	0
2015352787	779	district/faith-based	No	10/08/2017	1	0	0
2015303655	779	district/faith-based	No	10/08/2017	1	0	0
2015402655	779	district/faith-based	No	10/08/2017	1	0	0
2015303654	779	district/faith-based	No	10/08/2017	1	0	0
2015402657	779	district/faith-based	No	10/08/2017	1	0	0
2015303653	779	district/faith-based	No	10/08/2017	1	0	0
2015402658	779	district/faith-based	No	10/08/2017	1	0	0
2015303652	779	district/faith-based	No	10/08/2017	1	0	0
2015402659	779	district/faith-based	No	10/08/2017	1	0	0
2013252884	779	district/faith-based	No	10/08/2017	1	0	0
2015303649	779	district/faith-based	No	10/08/2017	1	0	0
2011140790	779	district/faith-based	No	10/08/2017	1	0	0
2015402660	779	district/faith-based	No	10/08/2017	1	0	0
2015303372	779	district/faith-based	No	10/08/2017	1	0	0
2015303648	779	district/faith-based	No	10/08/2017	1	0	0
2015402661	214	rural/clinic	No	10/08/2017	0	0	0
2015303373	214	rural/clinic	No	10/08/2017	0	0	0
2011140786	214	rural/clinic	No	10/08/2017	0	0	0
2015402662	214	rural/clinic	No	10/08/2017	0	0	0
2014385696	214	rural/clinic	No	10/08/2017	0	0	0
2014358032	846	district/faith-based	No	10/08/2017	1	0	0
2015407001	846	district/faith-based	No	10/08/2017	1	0	0
2012324251	846	district/faith-based	No	10/08/2017	1	0	0
2015286338	846	district/faith-based	No	10/08/2017	1	0	0
2014358031	846	district/faith-based	No	10/08/2017	1	0	0
2015286714	846	district/faith-based	No	10/08/2017	1	0	0
2011198697	846	district/faith-based	No	10/08/2017	1	0	0
2015286339	846	district/faith-based	No	10/08/2017	1	0	0
2014358030	846	district/faith-based	No	10/08/2017	1	0	0
2015339552	846	district/faith-based	No	10/08/2017	1	0	0
2015338469	163	rural/clinic	No	10/08/2017	0	0	0
2013250825	163	rural/clinic	No	10/08/2017	0	0	0
2011137324	163	rural/clinic	No	10/08/2017	0	0	0
2015409034	163	rural/clinic	No	10/08/2017	0	0	0
2014375535	163	rural/clinic	No	10/08/2017	0	0	0
2015368680	163	rural/clinic	No	10/08/2017	0	0	0
2011140554	163	rural/clinic	No	10/08/2017	0	0	0
2015409031	163	rural/clinic	No	10/08/2017	0	0	0
2014375537	407	district/faith-based	No	10/08/2017	1	0	0
2015368681	407	district/faith-based	No	10/08/2017	1	0	0
2014327606	407	district/faith-based	No	10/08/2017	1	0	0
2014375534	407	district/faith-based	No	10/08/2017	1	0	0
2014368695	407	district/faith-based	No	10/08/2017	1	0	0
2014385618	407	district/faith-based	No	10/08/2017	1	0	0
2014375536	407	district/faith-based	No	10/08/2017	1	0	0
2011196020	407	district/faith-based	No	10/08/2017	1	0	0
2014385619	407	district/faith-based	No	10/08/2017	1	0	0
2014375533	407	district/faith-based	No	10/08/2017	1	0	0
2014368696	407	district/faith-based	No	10/08/2017	1	0	0
2011135683	407	district/faith-based	No	10/08/2017	1	0	0
2014347997	407	district/faith-based	No	10/08/2017	1	0	0
2014375530	407	district/faith-based	No	10/08/2017	1	0	0
2015293845	407	district/faith-based	No	10/08/2017	1	0	0
2014375531	407	district/faith-based	No	10/08/2017	1	0	0
2014347995	407	district/faith-based	No	10/08/2017	1	0	0
2014357832	407	district/faith-based	No	10/08/2017	1	0	0
2015293846	407	district/faith-based	No	10/08/2017	1	0	0
2014375532	407	district/faith-based	No	10/08/2017	1	0	0
2014347996	407	district/faith-based	No	10/08/2017	1	0	0
2015349609	407	district/faith-based	No	10/08/2017	1	0	0
2015368897	407	district/faith-based	No	10/08/2017	1	0	0
2015344746	407	district/faith-based	No	10/08/2017	1	0	0
2015321810	407	district/faith-based	No	10/08/2017	1	0	0
2014297846	407	district/faith-based	No	10/08/2017	1	0	0
2014327694	407	district/faith-based	No	10/08/2017	1	0	0
2014291822	407	district/faith-based	No	10/08/2017	1	0	0
2015321762	407	district/faith-based	No	10/08/2017	1	0	0
2013247719	407	district/faith-based	No	10/08/2017	1	0	0
2014291821	407	district/faith-based	No	10/08/2017	1	0	0
2014327693	407	district/faith-based	No	10/08/2017	1	0	0
2015404662	407	district/faith-based	No	10/08/2017	1	0	0

2014297979	407	district/faith-based	No	10/08/2017	1	0	0
2011144033	407	district/faith-based	No	10/08/2017	1	0	0
2011136259	407	district/faith-based	No	10/08/2017	1	0	0
2014340281	370	district/faith-based	No	10/08/2017	1	0	0
2014335599	370	district/faith-based	No	10/08/2017	1	0	0
2012314026	370	district/faith-based	No	10/08/2017	1	0	0
2015331737	370	district/faith-based	No	10/08/2017	1	0	0
2015344472	370	district/faith-based	No	10/08/2017	1	0	0
2015361182	370	district/faith-based	No	10/08/2017	1	0	0
2015344473	370	district/faith-based	No	10/08/2017	1	0	0
2015361183	370	district/faith-based	No	10/08/2017	1	0	0
2015331739	370	district/faith-based	No	10/08/2017	1	0	0
2014374046	370	district/faith-based	No	10/08/2017	1	0	0
2011135680	370	district/faith-based	No	10/08/2017	1	0	0
2015331741	370	district/faith-based	No	10/08/2017	1	0	0
2014374047	370	district/faith-based	No	10/08/2017	1	0	0
2011135681	370	district/faith-based	No	10/08/2017	1	0	0
2015331553	370	district/faith-based	No	10/08/2017	1	0	0
2014374048	370	district/faith-based	No	10/08/2017	1	0	0
2011135682	370	district/faith-based	No	10/08/2017	1	0	0
2015368824	370	district/faith-based	No	10/08/2017	1	0	0
2015331555	370	district/faith-based	No	10/08/2017	1	0	0
2014374045	370	district/faith-based	No	10/08/2017	1	0	0
2011136257	370	district/faith-based	No	10/08/2017	1	0	0
2015401162	370	district/faith-based	No	10/08/2017	1	0	0
2013247720	370	district/faith-based	No	10/08/2017	1	0	0
2015334810	370	district/faith-based	No	10/08/2017	1	0	0
2015401163	370	district/faith-based	No	10/08/2017	1	0	0
2012369977	370	district/faith-based	No	10/08/2017	1	0	0
2015372029	370	district/faith-based	No	10/08/2017	1	0	0
2015358311	370	district/faith-based	No	10/08/2017	1	0	0
2015334809	370	district/faith-based	No	10/08/2017	1	0	0
2014300540	370	district/faith-based	No	10/08/2017	1	0	0
2015334808	370	district/faith-based	No	10/08/2017	1	0	0
2012379348	370	district/faith-based	No	10/08/2017	1	0	0
2011221340	370	district/faith-based	No	10/08/2017	1	0	0
2012379346	370	district/faith-based	No	10/08/2017	1	0	0
2012369975	370	district/faith-based	No	10/08/2017	1	0	0
2015344466	370	district/faith-based	No	10/08/2017	1	0	0
2011144032	370	district/faith-based	No	10/08/2017	1	0	0
2015373886	370	district/faith-based	No	10/08/2017	1	0	0
2015344467	370	district/faith-based	No	10/08/2017	1	0	0
2015344468	370	district/faith-based	No	10/08/2017	1	0	0
2012253981	370	district/faith-based	No	10/08/2017	1	0	0
2015358315	370	district/faith-based	No	10/08/2017	1	0	0
2015344469	370	district/faith-based	No	10/08/2017	1	0	0
2015310620	370	district/faith-based	No	10/08/2017	1	0	0
2015344470	370	district/faith-based	No	10/08/2017	1	0	0
2011216416	370	district/faith-based	No	10/08/2017	1	0	0
2015368823	370	district/faith-based	No	10/08/2017	1	0	0
2015290226	370	district/faith-based	No	10/08/2017	1	0	0
2015344471	370	district/faith-based	No	10/08/2017	1	0	0
2015368827	370	district/faith-based	No	10/08/2017	1	0	0
2015368828	370	district/faith-based	No	10/08/2017	1	0	0
2011193282	370	district/faith-based	No	10/08/2017	1	0	0
2015344745	370	district/faith-based	No	10/08/2017	1	0	0
2015339610	370	district/faith-based	No	10/08/2017	1	0	0
2011193281	370	district/faith-based	No	10/08/2017	1	0	0
2012253980	370	district/faith-based	No	10/08/2017	1	0	0
2015324574	370	district/faith-based	No	10/08/2017	1	0	0
2012253982	370	district/faith-based	No	10/08/2017	1	0	0
2014335590	370	district/faith-based	No	10/08/2017	1	0	0
2014303722	370	district/faith-based	No	10/08/2017	1	0	0
2011216415	370	district/faith-based	No	10/08/2017	1	0	0
2015358313	370	district/faith-based	No	10/08/2017	1	0	0
2014303724	370	district/faith-based	No	10/08/2017	1	0	0
2011193280	370	district/faith-based	No	10/08/2017	1	0	0
2014303723	370	district/faith-based	No	10/08/2017	1	0	0
2015358312	370	district/faith-based	No	10/08/2017	1	0	0
2014335591	370	district/faith-based	No	10/08/2017	1	0	0
2015290034	370	district/faith-based	No	10/08/2017	1	0	0
2011216417	370	district/faith-based	No	10/08/2017	1	0	0
2015358314	370	district/faith-based	No	10/08/2017	1	0	0
2014303726	370	district/faith-based	No	10/08/2017	1	0	0
2014303721	370	district/faith-based	No	10/08/2017	1	0	0
2015358306	370	district/faith-based	No	10/08/2017	1	0	0
2014335592	370	district/faith-based	No	10/08/2017	1	0	0
2015290225	370	district/faith-based	No	10/08/2017	1	0	0
2015358308	370	district/faith-based	No	10/08/2017	1	0	0
2014340277	370	district/faith-based	No	10/08/2017	1	0	0
2012313814	370	district/faith-based	No	10/08/2017	1	0	0
2015358307	370	district/faith-based	No	10/08/2017	1	0	0

2014324712	370	district/faith-based	No	10/08/2017	1	0	0
2014340278	370	district/faith-based	No	10/08/2017	1	0	0
2015358310	370	district/faith-based	No	10/08/2017	1	0	0
2014335593	370	district/faith-based	No	10/08/2017	1	0	0
2014324710	370	district/faith-based	No	10/08/2017	1	0	0
2015358309	370	district/faith-based	No	10/08/2017	1	0	0
2014297789	370	district/faith-based	No	10/08/2017	1	0	0
2015418762	370	district/faith-based	No	10/08/2017	1	0	0
2011155241	370	district/faith-based	No	10/08/2017	1	0	0
2014314235	370	district/faith-based	No	10/08/2017	1	0	0
2011144030	370	district/faith-based	No	10/08/2017	1	0	0
2015373884	370	district/faith-based	No	10/08/2017	1	0	0
2014335594	370	district/faith-based	No	10/08/2017	1	0	0
2014314234	370	district/faith-based	No	10/08/2017	1	0	0
2015373883	370	district/faith-based	No	10/08/2017	1	0	0
2015290221	370	district/faith-based	No	10/08/2017	1	0	0
2014314233	370	district/faith-based	No	10/08/2017	1	0	0
2012388293	370	district/faith-based	No	10/08/2017	1	0	0
2014335595	370	district/faith-based	No	10/08/2017	1	0	0
2012388294	370	district/faith-based	No	10/08/2017	1	0	0
2014335596	370	district/faith-based	No	10/08/2017	1	0	0
2012271403	370	district/faith-based	No	10/08/2017	1	0	0
2012388295	370	district/faith-based	No	10/08/2017	1	0	0
2012388296	370	district/faith-based	No	10/08/2017	1	0	0
2014335597	370	district/faith-based	No	10/08/2017	1	0	0
2012388297	370	district/faith-based	No	10/08/2017	1	0	0
2011137319	370	district/faith-based	No	10/08/2017	1	0	0
2012388298	370	district/faith-based	No	10/08/2017	1	0	0
2011137320	370	district/faith-based	No	10/08/2017	1	0	0
2015349024	370	district/faith-based	No	10/08/2017	1	0	0
2014377626	370	district/faith-based	No	10/08/2017	1	0	0
2011137321	370	district/faith-based	No	10/08/2017	1	0	0
2011200082	370	district/faith-based	No	10/08/2017	1	0	0
2015375423	370	district/faith-based	No	10/08/2017	1	0	0
2011200081	370	district/faith-based	No	10/08/2017	1	0	0
2015375422	370	district/faith-based	No	10/08/2017	1	0	0
2015289220	370	district/faith-based	No	10/08/2017	1	0	0
2015375421	370	district/faith-based	No	10/08/2017	1	0	0
2015289219	370	district/faith-based	No	10/08/2017	1	0	0
2015289218	370	district/faith-based	No	10/08/2017	1	0	0
2015289217	370	district/faith-based	No	10/08/2017	1	0	0
2015289215	370	district/faith-based	No	10/08/2017	1	0	0
2014288144	370	district/faith-based	No	10/08/2017	1	0	0
2015349016	370	district/faith-based	No	10/08/2017	1	0	0
2011191297	370	district/faith-based	No	10/08/2017	1	0	0
2015340718	370	district/faith-based	No	10/08/2017	1	0	0
2017324709	589	rural/clinic	No	10/08/2017	0	0	0
2011191296	589	rural/clinic	No	10/08/2017	0	0	0
2015310619	589	rural/clinic	No	10/08/2017	0	0	0
2011137322	589	rural/clinic	No	10/08/2017	0	0	0
2015340719	589	rural/clinic	No	10/08/2017	0	0	0
2015403304	589	rural/clinic	No	10/08/2017	0	0	0
2015290222	589	rural/clinic	No	10/08/2017	0	0	0
2015294184	589	rural/clinic	No	10/08/2017	0	0	0
2015331734	589	rural/clinic	No	10/08/2017	0	0	0
2015375910	589	rural/clinic	No	10/08/2017	0	0	0
2015403299	589	rural/clinic	No	10/08/2017	0	0	0
2015414563	589	rural/clinic	No	10/08/2017	0	0	0
2015290224	589	rural/clinic	No	10/08/2017	0	0	0
2015331736	589	rural/clinic	No	10/08/2017	0	0	0
2015403295	589	rural/clinic	No	10/08/2017	0	0	0
2015403303	589	rural/clinic	No	10/08/2017	0	0	0
2015403305	589	rural/clinic	No	10/08/2017	0	0	0
2015403301	589	rural/clinic	No	10/08/2017	0	0	0
2015403293	589	rural/clinic	No	10/08/2017	0	0	0
2015364330	589	rural/clinic	No	10/08/2017	0	0	0
2015403294	589	rural/clinic	No	10/08/2017	0	0	0
2015364331	589	rural/clinic	No	10/08/2017	0	0	0
2015364332	589	rural/clinic	No	10/08/2017	0	0	0
2014316076	589	rural/clinic	No	10/08/2017	0	0	0
2015364334	589	rural/clinic	No	10/08/2017	0	0	0
2014316079	589	rural/clinic	No	10/08/2017	0	0	0
2015377188	589	rural/clinic	No	10/08/2017	0	0	0
2014316078	589	rural/clinic	No	10/08/2017	0	0	0
2015382585	589	rural/clinic	No	10/08/2017	0	0	0
2015382584	589	rural/clinic	No	10/08/2017	0	0	0
2015382591	589	rural/clinic	No	10/08/2017	0	0	0
2015382583	589	rural/clinic	No	10/08/2017	0	0	0
2015382592	589	rural/clinic	No	10/08/2017	0	0	0
2014317649	589	rural/clinic	No	10/08/2017	0	0	0
2014317650	589	rural/clinic	No	10/08/2017	0	0	0
201053920	589	rural/clinic	No	10/08/2017	0	0	0

201053919	589	rural/clinic	No	10/08/2017	0	0	0
2015338883	589	rural/clinic	No	10/08/2017	0	0	0
2015338882	589	rural/clinic	No	10/08/2017	0	0	0
2015338881	589	rural/clinic	No	10/08/2017	0	0	0
2015415292	589	rural/clinic	No	10/08/2017	0	0	0
2014317519	589	rural/clinic	No	10/08/2017	0	0	0
2015415203	589	rural/clinic	No	10/08/2017	0	0	0
2015415202	589	rural/clinic	No	10/08/2017	0	0	0
2015377189	589	rural/clinic	No	10/08/2017	0	0	0
2011140304	589	rural/clinic	No	10/08/2017	0	0	0
2015362066	589	rural/clinic	No	10/08/2017	0	0	0
2011123921	589	rural/clinic	No	10/08/2017	0	0	0
2014347036	589	rural/clinic	No	10/08/2017	0	0	0
2014347037	589	rural/clinic	No	10/08/2017	0	0	0
2014347039	589	rural/clinic	No	10/08/2017	0	0	0
2015340903	589	rural/clinic	No	08/08/2017	0	0	0
2014383710	589	rural/clinic	No	08/08/2017	0	0	0
2014383710	589	rural/clinic	No	08/08/2017	0	0	0
2015385863	589	rural/clinic	No	08/08/2017	0	0	0
2015385863	589	rural/clinic	No	08/08/2017	0	0	0
2013249284	589	rural/clinic	No	08/08/2017	0	0	0
2013249284	589	rural/clinic	No	08/08/2017	0	0	0
2011144841	589	rural/clinic	No	08/08/2017	0	0	0
2011144841	589	rural/clinic	No	08/08/2017	0	0	0
2015326986	589	rural/clinic	No	08/08/2017	0	0	0
2015326986	589	rural/clinic	No	08/08/2017	0	0	0
2015335400	589	rural/clinic	No	10/08/2017	0	0	0
2015362002	589	rural/clinic	No	10/08/2017	0	0	0
2011226491	589	rural/clinic	No	10/08/2017	0	0	0
2015417086	589	rural/clinic	No	10/08/2017	0	0	0
2015417089	589	rural/clinic	No	10/08/2017	0	0	0
2015417088	589	rural/clinic	No	10/08/2017	0	0	0
2014298042	589	rural/clinic	No	10/08/2017	0	0	0
2011145398	589	rural/clinic	No	10/08/2017	0	0	0
2011145397	589	rural/clinic	No	10/08/2017	0	0	0
2011145399	589	rural/clinic	No	10/08/2017	0	0	0
2015294185	589	rural/clinic	No	10/08/2017	0	0	0
2015410351	589	rural/clinic	No	10/08/2017	0	0	0
2011140794	589	rural/clinic	No	10/08/2017	0	0	0
2011140794	589	rural/clinic	No	10/08/2017	0	0	0
2015352787	589	rural/clinic	No	10/08/2017	0	0	0
2015352787	589	rural/clinic	No	10/08/2017	0	0	0
2015328097	589	rural/clinic	No	10/08/2017	0	0	0
2015328097	589	rural/clinic	No	10/08/2017	0	0	0
2015328099	589	rural/clinic	No	10/08/2017	0	0	0
2015328099	589	rural/clinic	No	10/08/2017	0	0	0
2015305097	589	rural/clinic	No	10/08/2017	0	0	0
2015305097	589	rural/clinic	No	10/08/2017	0	0	0
2011140554	589	rural/clinic	No	10/08/2017	0	0	0
2015336880	589	rural/clinic	No	16/08/2017	0	0	0
2012294628	589	rural/clinic	No	16/08/2017	0	0	0
2012294626	589	rural/clinic	No	16/08/2017	0	0	0
2014329031	589	rural/clinic	No	16/08/2017	0	0	0
2014329032	589	rural/clinic	No	16/08/2017	0	0	0
2015336884	589	rural/clinic	No	16/08/2017	0	0	0
2015373887	589	rural/clinic	No	17/08/2017	0	0	0
2015373888	215	rural/clinic	No	17/08/2017	0	0	0
2012305831	215	rural/clinic	No	16/08/2017	0	0	0
2015342298	164	rural/clinic	No	16/08/2017	0	0	0
2013249285	164	rural/clinic	No	16/08/2017	0	0	0
2015336882	164	rural/clinic	No	16/08/2017	0	0	0
2015336883	164	rural/clinic	No	16/08/2017	0	0	0
2015336881	164	rural/clinic	No	16/08/2017	0	0	0
2015373889	164	rural/clinic	No	17/08/2017	0	0	0
2015397658	164	rural/clinic	No	16/08/2017	0	0	0
2015397659	164	rural/clinic	No	16/08/2017	0	0	0
2015342061	164	rural/clinic	No	16/08/2017	0	0	0
2015331780	164	rural/clinic	No	16/08/2017	0	0	0
2013249491	164	rural/clinic	No	16/08/2017	0	0	0
2012305830	164	rural/clinic	No	16/08/2017	0	0	0
2015409029	164	rural/clinic	No	10/08/2017	0	0	0
2014297790	164	rural/clinic	No	10/08/2017	0	0	0
2014297790	164	rural/clinic	No	10/08/2017	0	0	0
2015294059	164	rural/clinic	No	10/08/2017	0	0	0
2015294059	164	rural/clinic	No	10/08/2017	0	0	0
2015373886	164	rural/clinic	No	10/08/2017	0	0	0
2015373886	164	rural/clinic	No	10/08/2017	0	0	0
2011193282	164	rural/clinic	No	10/08/2017	0	0	0
2011193282	164	rural/clinic	No	10/08/2017	0	0	0
2015386754	164	rural/clinic	No	10/08/2017	0	0	0
2015386754	164	rural/clinic	No	10/08/2017	0	0	0
2015286338	164	rural/clinic	No	10/08/2017	0	0	0

2015286338	164	rural/clinic	No	10/08/2017	0	0	0
2015375908	164	rural/clinic	No	10/08/2017	0	0	0
2014375530	164	rural/clinic	No	10/08/2017	0	0	0
2014375530	164	rural/clinic	No	10/08/2017	0	0	0
2014375531	164	rural/clinic	No	10/08/2017	0	0	0
2014375531	164	rural/clinic	No	10/08/2017	0	0	0
2014335595	164	rural/clinic	No	10/08/2017	0	0	0
2014335595	164	rural/clinic	No	10/08/2017	0	0	0
2015294184	164	rural/clinic	No	10/08/2017	0	0	0
2015294184	164	rural/clinic	No	10/08/2017	0	0	0
201053920	164	rural/clinic	No	10/08/2017	0	0	0
201053920	164	rural/clinic	No	10/08/2017	0	0	0
2014314235	164	rural/clinic	No	10/08/2017	0	0	0
2014314235	164	rural/clinic	No	10/08/2017	0	0	0
2015290225	164	rural/clinic	No	10/08/2017	0	0	0
2015290225	550	rural/clinic	No	10/08/2017	0	0	0
2012388298	550	rural/clinic	No	10/08/2017	0	0	0
2012388298	550	rural/clinic	No	10/08/2017	0	0	0
2015403303	550	rural/clinic	No	10/08/2017	0	0	0
2015403303	550	rural/clinic	No	10/08/2017	0	0	0
2014332720	621	district/faith-based	No	17/08/2017	1	0	0
2014332718	621	district/faith-based	No	17/08/2017	1	0	0
2014332717	621	district/faith-based	No	17/08/2017	1	0	0
2014332721	621	district/faith-based	No	17/08/2017	1	0	0
2012242588	621	district/faith-based	No	17/08/2017	1	0	0
2012242590	621	district/faith-based	No	17/08/2017	1	0	0
2012242591	621	district/faith-based	No	17/08/2017	1	0	0
2015349851	621	district/faith-based	No	17/08/2017	1	0	0
2015373890	621	district/faith-based	No	17/08/2017	1	0	0
2015302303	621	district/faith-based	No	17/08/2017	1	0	0
2015302301	621	district/faith-based	No	17/08/2017	1	0	0
2014383986	621	district/faith-based	No	16/08/2017	1	0	0
2015301273	621	district/faith-based	No	17/08/2017	1	0	0
2015332110	621	district/faith-based	No	16/08/2017	1	0	0
2011199099	621	district/faith-based	No	17/08/2017	1	0	0
2011199100	621	district/faith-based	No	17/08/2017	1	0	0
2015301274	621	district/faith-based	No	17/08/2017	1	0	0
2015340020	621	district/faith-based	No	17/08/2017	1	0	0
2011225418	621	district/faith-based	No	17/08/2017	1	0	0
2011225417	621	district/faith-based	No	17/08/2017	1	0	0
2011225416	621	district/faith-based	No	17/08/2017	1	0	0
2011225419	621	district/faith-based	No	17/08/2017	1	0	0
2014383987	621	district/faith-based	No	16/08/2017	1	0	0
2015397656	621	district/faith-based	No	16/08/2017	1	0	0
2011225420	621	district/faith-based	No	17/08/2017	1	0	0
2015397655	621	district/faith-based	No	16/08/2017	1	0	0
2015397657	621	district/faith-based	No	16/08/2017	1	0	0
2014319095	621	district/faith-based	No	17/08/2017	1	0	0
2015403297	621	district/faith-based	No	17/08/2017	1	0	0
2015326988	621	district/faith-based	No	17/08/2017	1	0	0
2015326989	621	district/faith-based	No	17/08/2017	1	0	0
2015326990	621	district/faith-based	No	17/08/2017	1	0	0
2015331742	621	district/faith-based	No	17/08/2017	1	0	0
2015364335	83	rural/clinic	No	17/08/2017	0	0	0
2015364336	83	rural/clinic	No	17/08/2017	0	0	0
2015364337	83	rural/clinic	No	17/08/2017	0	0	0
2012242589	83	rural/clinic	No	17/08/2017	0	0	0
2015364339	83	rural/clinic	No	17/08/2017	0	0	0
2015364340	83	rural/clinic	No	17/08/2017	0	0	0
2011168524	83	rural/clinic	No	17/08/2017	0	0	0
2011168523	83	rural/clinic	No	17/08/2017	0	0	0
2015362003	83	rural/clinic	No	17/08/2017	0	0	0
2014347038	83	rural/clinic	No	17/08/2017	0	0	0
2011140305	83	rural/clinic	No	17/08/2017	0	0	0
2012312156	83	rural/clinic	No	17/08/2017	0	0	0
2015297300	83	rural/clinic	No	17/08/2017	0	0	0
2015297299	83	rural/clinic	No	17/08/2017	0	0	0
2012312154	83	rural/clinic	No	17/08/2017	0	0	0
2012312155	83	rural/clinic	No	17/08/2017	0	0	0
2015297298	83	rural/clinic	No	17/08/2017	0	0	0
2015412784	83	rural/clinic	No	17/08/2017	0	0	0
2012312153	83	rural/clinic	No	17/08/2017	0	0	0
2012312152	83	rural/clinic	No	17/08/2017	0	0	0
2015362541	83	rural/clinic	No	17/08/2017	0	0	0
2015362540	83	rural/clinic	No	17/08/2017	0	0	0
2015294893	83	rural/clinic	No	17/08/2017	0	0	0
2015294885	83	rural/clinic	No	17/08/2017	0	0	0
2015363472	83	rural/clinic	No	17/08/2017	0	0	0
2015360612	83	rural/clinic	No	17/08/2017	0	0	0
2015363471	83	rural/clinic	No	17/08/2017	0	0	0
2015363158	83	rural/clinic	No	17/08/2017	0	0	0
2015363470	83	rural/clinic	No	17/08/2017	0	0	0

2015335980	83	rural/clinic	No	17/08/2017	0	0	0
2015414738	83	rural/clinic	No	17/08/2017	0	0	0
2015414740	83	rural/clinic	No	17/08/2017	0	0	0
2014367409	83	rural/clinic	No	17/08/2017	0	0	0
2012365480	83	rural/clinic	No	17/08/2017	0	0	0
2015414277	534	rural/clinic	No	17/08/2017	0	0	0
2015414275	534	rural/clinic	No	17/08/2017	0	0	0
2015414276	534	rural/clinic	No	17/08/2017	0	0	0
2015315916	534	rural/clinic	No	17/08/2017	0	0	0
2012365481	534	rural/clinic	No	17/08/2017	0	0	0
2011133158	534	rural/clinic	No	17/08/2017	0	0	0
2015368792	534	rural/clinic	No	17/08/2017	0	0	0
2015364959	534	rural/clinic	No	17/08/2017	0	0	0
2015337535	534	rural/clinic	No	17/08/2017	0	0	0
2015364958	534	rural/clinic	No	17/08/2017	0	0	0
2015315917	534	rural/clinic	No	17/08/2017	0	0	0
2015364957	534	rural/clinic	No	17/08/2017	0	0	0
2012323781	534	rural/clinic	No	17/08/2017	0	0	0
2015364956	622	district/faith-based	No	17/08/2017	1	0	0
2015368740	622	district/faith-based	No	17/08/2017	1	0	0
2014346740	622	district/faith-based	No	17/08/2017	1	0	0
2015377761	622	district/faith-based	No	17/08/2017	1	0	0
2014346741	622	district/faith-based	No	17/08/2017	1	0	0
2015377760	622	district/faith-based	No	17/08/2017	1	0	0
2014346742	622	district/faith-based	No	17/08/2017	1	0	0
2015377759	622	district/faith-based	No	17/08/2017	1	0	0
2015377758	622	district/faith-based	No	17/08/2017	1	0	0
2015385546	622	district/faith-based	No	17/08/2017	1	0	0
2015385548	622	district/faith-based	No	17/08/2017	1	0	0
2015385547	622	district/faith-based	No	17/08/2017	1	0	0
2014332722	622	district/faith-based	No	17/08/2017	1	0	0
2014332723	622	district/faith-based	No	17/08/2017	1	0	0
2015372945	622	district/faith-based	No	17/08/2017	1	0	0
2014371599	622	district/faith-based	No	17/08/2017	1	0	0
2014371598	622	district/faith-based	No	17/08/2017	1	0	0
2012379907	622	district/faith-based	No	17/08/2017	1	0	0
2015419066	622	district/faith-based	No	17/08/2017	1	0	0
2015301366	622	district/faith-based	No	17/08/2017	1	0	0
2015419071	622	district/faith-based	No	17/08/2017	1	0	0
2015373891	622	district/faith-based	No	17/08/2017	1	0	0
2015301365	622	district/faith-based	No	17/08/2017	1	0	0
2013254146	622	district/faith-based	No	17/08/2017	1	0	0
2015301364	622	district/faith-based	No	17/08/2017	1	0	0
2015419073	622	district/faith-based	No	17/08/2017	1	0	0
2015382951	622	district/faith-based	No	17/08/2017	1	0	0
2015419072	622	district/faith-based	No	17/08/2017	1	0	0
2015382947	622	district/faith-based	No	17/08/2017	1	0	0
2015419067	622	district/faith-based	No	17/08/2017	1	0	0
2012295822	622	district/faith-based	No	17/08/2017	1	0	0
2012295821	622	district/faith-based	No	17/08/2017	1	0	0
2012295820	622	district/faith-based	No	17/08/2017	1	0	0
2015382948	622	district/faith-based	No	17/08/2017	1	0	0
2012295815	622	district/faith-based	No	17/08/2017	1	0	0
2015382949	622	district/faith-based	No	17/08/2017	1	0	0
2012295816	622	district/faith-based	No	17/08/2017	1	0	0
2012295817	622	district/faith-based	No	17/08/2017	1	0	0
2015382950	622	district/faith-based	No	17/08/2017	1	0	0
2012295818	622	district/faith-based	No	17/08/2017	1	0	0
2012295819	622	district/faith-based	No	17/08/2017	1	0	0
2012295814	622	district/faith-based	No	17/08/2017	1	0	0
2012295813	622	district/faith-based	No	17/08/2017	1	0	0
2012295812	622	district/faith-based	No	17/08/2017	1	0	0
2015305098	622	district/faith-based	No	17/08/2017	1	0	0
2011135959	622	district/faith-based	No	17/08/2017	1	0	0
2014362846	622	district/faith-based	No	17/08/2017	1	0	0
2014362847	622	district/faith-based	No	17/08/2017	1	0	0
2014358200	622	district/faith-based	No	17/08/2017	1	0	0
2014358199	622	district/faith-based	No	17/08/2017	1	0	0
2014358197	622	district/faith-based	No	17/08/2017	1	0	0
2014358198	622	district/faith-based	No	17/08/2017	1	0	0
2011136260	622	district/faith-based	No	17/08/2017	1	0	0
2015368738	622	district/faith-based	No	17/08/2017	1	0	0
2011135960	622	district/faith-based	No	17/08/2017	1	0	0
2015368741	622	district/faith-based	No	17/08/2017	1	0	0
2013256303	622	district/faith-based	No	17/08/2017	1	0	0
2011135958	622	district/faith-based	No	17/08/2017	1	0	0
2015368614	622	district/faith-based	No	17/08/2017	1	0	0
2015368615	622	district/faith-based	No	17/08/2017	1	0	0
2015385865	622	district/faith-based	No	17/08/2017	1	0	0
2015362543	622	district/faith-based	No	17/08/2017	1	0	0
2015347207	622	district/faith-based	No	17/08/2017	1	0	0
2015362542	622	district/faith-based	No	17/08/2017	1	0	0

2015385866	622	district/faith-based	No	17/08/2017	1	0	0
2015372944	622	district/faith-based	No	17/08/2017	1	0	0
2015314617	622	district/faith-based	No	17/08/2017	1	0	0
2015326912	622	district/faith-based	No	17/08/2017	1	0	0
2012358300	622	district/faith-based	No	17/08/2017	1	0	0
2015326913	622	district/faith-based	No	17/08/2017	1	0	0
2011133157	622	district/faith-based	No	17/08/2017	1	0	0
2015315006	622	district/faith-based	No	17/08/2017	1	0	0
2015326914	622	district/faith-based	No	17/08/2017	1	0	0
2015338720	622	district/faith-based	No	17/08/2017	1	0	0
2015326915	622	district/faith-based	No	17/08/2017	1	0	0
2015326916	622	district/faith-based	No	17/08/2017	1	0	0
2015326917	622	district/faith-based	No	17/08/2017	1	0	0
2015382960	622	district/faith-based	No	17/08/2017	1	0	0
2015417089	622	district/faith-based	No	10/08/2017	1	0	0
2015373887	622	district/faith-based	No	17/08/2017	1	0	0
2015373887	622	district/faith-based	No	17/08/2017	1	0	0
2015373889	622	district/faith-based	No	17/08/2017	1	0	0
2015373889	622	district/faith-based	No	17/08/2017	1	0	0
2015373890	622	district/faith-based	No	17/08/2017	1	0	0
2015373890	622	district/faith-based	No	17/08/2017	1	0	0
2012242588	622	district/faith-based	No	17/08/2017	1	0	0
2015315916	622	district/faith-based	No	17/08/2017	1	0	0
2015315916	622	district/faith-based	No	17/08/2017	1	0	0
2011133158	622	district/faith-based	No	17/08/2017	1	0	0
2011133158	622	district/faith-based	No	17/08/2017	1	0	0
2015338720	622	district/faith-based	No	17/08/2017	1	0	0
2015338720	622	district/faith-based	No	17/08/2017	1	0	0
2015347207	622	district/faith-based	No	17/08/2017	1	0	0
2015347207	622	district/faith-based	No	17/08/2017	1	0	0
2015364963	622	district/faith-based	No	22/08/2017	1	0	0
2015342322	622	district/faith-based	No	22/08/2017	1	0	0
2012284576	622	district/faith-based	No	22/07/2017	1	0	0
2015379331	622	district/faith-based	No	22/08/2017	1	0	0
2015372862	622	district/faith-based	No	22/08/2017	1	0	0
2011235746	622	district/faith-based	No	22/08/2017	1	0	0
2012379908	622	district/faith-based	No	22/08/2017	1	0	0
2012330892	622	district/faith-based	No	22/08/2017	1	0	0
2015363086	622	district/faith-based	No	22/08/2017	1	0	0
2014346743	622	district/faith-based	No	22/08/2017	1	0	0
2015363088	622	district/faith-based	No	22/08/2017	1	0	0
2014346744	622	district/faith-based	No	22/08/2017	1	0	0
2014350372	622	district/faith-based	No	22/08/2017	1	0	0
2015368742	622	district/faith-based	No	22/08/2017	1	0	0
2015363087	622	district/faith-based	No	22/08/2017	1	0	0
2015368743	622	district/faith-based	No	22/08/2017	1	0	0
2012371451	622	district/faith-based	No	22/08/2017	1	0	0
2015368744	126	district/faith-based	No	22/08/2017	1	0	0
2014348245	126	district/faith-based	No	22/08/2017	1	0	0
2014287992	126	district/faith-based	No	22/08/2017	1	0	0
2014348246	126	district/faith-based	No	22/08/2017	1	0	0
2015331104	126	district/faith-based	No	22/08/2017	1	0	0
2012284625	126	district/faith-based	No	22/08/2017	1	0	0
2012371453	126	district/faith-based	No	22/08/2017	1	0	0
2012371452	126	district/faith-based	No	22/08/2017	1	0	0
2015368616	126	district/faith-based	No	22/08/2017	1	0	0
2012275993	126	district/faith-based	No	22/08/2017	1	0	0
2015368617	126	district/faith-based	No	22/08/2017	1	0	0
2012371455	126	district/faith-based	No	22/08/2017	1	0	0
2015368618	126	district/faith-based	No	22/08/2017	1	0	0
2015367712	126	district/faith-based	No	22/08/2017	1	0	0
2015325224	126	district/faith-based	No	22/08/2017	1	0	0
2015301154	126	district/faith-based	No	22/08/2017	1	0	0
2015325225	867	rural/clinic	No	22/08/2017	0	0	0
2014320305	867	rural/clinic	No	22/08/2017	0	0	0
2015397937	867	rural/clinic	No	22/08/2017	0	0	0
2014301678	867	rural/clinic	No	22/08/2017	0	0	0
2015340906	867	rural/clinic	No	22/08/2017	0	0	0
2015297203	867	rural/clinic	No	22/08/2017	0	0	0
2015325222	867	rural/clinic	No	22/08/2017	0	0	0
2015297175	867	rural/clinic	No	22/08/2017	0	0	0
2015297172	867	rural/clinic	No	22/08/2017	0	0	0
2015325223	867	rural/clinic	No	22/08/2017	0	0	0
2015301153	867	rural/clinic	No	22/08/2017	0	0	0
2015340908	867	rural/clinic	No	22/08/2017	0	0	0
2015301151	867	rural/clinic	No	22/08/2017	0	0	0
2015340909	867	rural/clinic	No	22/08/2017	0	0	0
2014327131	867	rural/clinic	No	22/08/2017	0	0	0
2015301152	867	rural/clinic	No	22/08/2017	0	0	0
2015340904	867	rural/clinic	No	22/08/2017	0	0	0
2015340947	867	rural/clinic	No	22/08/2017	0	0	0
2015325255	867	rural/clinic	No	22/08/2017	0	0	0

2015297173	867	rural/clinic	No	22/08/2017	0	0	0
2015325226	867	rural/clinic	No	22/08/2017	0	0	0
2015297174	867	rural/clinic	No	22/08/2017	0	0	0
2015357307	867	rural/clinic	No	22/08/2017	0	0	0
2014332724	867	rural/clinic	No	22/08/2017	0	0	0
2012368547	867	rural/clinic	No	22/08/2017	0	0	0
2014332725	867	rural/clinic	No	22/08/2017	0	0	0
2015340907	867	rural/clinic	No	22/08/2017	0	0	0
2015360613	867	rural/clinic	No	22/08/2017	0	0	0
2015325945	867	rural/clinic	No	22/08/2017	0	0	0
2015325946	867	rural/clinic	No	22/08/2017	0	0	0
2011222232	867	rural/clinic	No	22/08/2017	0	0	0
2015384494	867	rural/clinic	No	22/08/2017	0	0	0
2015363475	867	rural/clinic	No	22/08/2017	0	0	0
2015363476	867	rural/clinic	No	22/08/2017	0	0	0
2015384495	867	rural/clinic	No	22/08/2017	0	0	0
2015363474	867	rural/clinic	No	22/08/2017	0	0	0
2015384497	867	rural/clinic	No	22/08/2017	0	0	0
2015384496	867	rural/clinic	No	22/08/2017	0	0	0
2015363473	867	rural/clinic	No	22/08/2017	0	0	0
2013264080	867	rural/clinic	No	22/08/2017	0	0	0
2015326918	867	rural/clinic	No	22/08/2017	0	0	0
2015326919	867	rural/clinic	No	22/08/2017	0	0	0
2015404977	867	rural/clinic	No	22/08/2017	0	0	0
2015404978	867	rural/clinic	No	22/08/2017	0	0	0
2013264081	867	rural/clinic	No	22/08/2017	0	0	0
2013264082	867	rural/clinic	No	22/08/2017	0	0	0
2015404979	867	rural/clinic	No	22/08/2017	0	0	0
2015352800	867	rural/clinic	No	22/08/2017	0	0	0
2015404980	867	rural/clinic	No	22/08/2017	0	0	0
2015404981	867	rural/clinic	No	22/08/2017	0	0	0
2015352799	867	rural/clinic	No	22/08/2017	0	0	0
2015372822	867	rural/clinic	No	22/08/2017	0	0	0
2014329029	867	rural/clinic	No	04/08/2017	0	0	0
2014334188	867	rural/clinic	No	22/08/2017	0	0	0
2015352798	867	rural/clinic	No	22/08/2017	0	0	0
2014329030	867	rural/clinic	No	22/08/2017	0	0	0
2015372823	867	rural/clinic	No	22/08/2017	0	0	0
2015352797	867	rural/clinic	No	22/08/2017	0	0	0
2015372825	867	rural/clinic	No	22/08/2017	0	0	0
2015352796	867	rural/clinic	No	22/08/2017	0	0	0
2015372824	867	rural/clinic	No	22/08/2017	0	0	0
2015352795	867	rural/clinic	No	22/08/2017	0	0	0
2015301157	867	rural/clinic	No	22/08/2017	0	0	0
2015352794	867	rural/clinic	No	22/08/2017	0	0	0
2015352793	867	rural/clinic	No	22/08/2017	0	0	0
2015301156	867	rural/clinic	No	22/08/2017	0	0	0
2015352792	867	rural/clinic	No	22/08/2017	0	0	0
2014382305	867	rural/clinic	No	22/08/2017	0	0	0
2015352791	867	rural/clinic	No	22/08/2017	0	0	0
2015301774	867	rural/clinic	No	22/08/2017	0	0	0
2015301773	867	rural/clinic	No	22/08/2017	0	0	0
2015301772	867	rural/clinic	No	22/08/2017	0	0	0
2014335904	867	rural/clinic	No	22/08/2017	0	0	0
2015382957	867	rural/clinic	No	08/08/2017	0	0	0
2014335903	867	rural/clinic	No	22/08/2017	0	0	0
2015315970	867	rural/clinic	No	22/08/2017	0	0	0
2015315971	867	rural/clinic	No	22/08/2017	0	0	0
2015315972	867	rural/clinic	No	22/08/2017	0	0	0
2015315974	867	rural/clinic	No	22/08/2017	0	0	0
2015315973	867	rural/clinic	No	22/08/2017	0	0	0
2015315976	867	rural/clinic	No	22/08/2017	0	0	0
2015315978	867	rural/clinic	No	22/08/2017	0	0	0
2015315977	867	rural/clinic	No	22/08/2017	0	0	0
2015315975	867	rural/clinic	No	22/08/2017	0	0	0
2015315977	867	rural/clinic	No	22/08/2017	0	0	0
2015315977	867	rural/clinic	No	22/08/2017	0	0	0
2015315979	867	rural/clinic	No	22/08/2017	0	0	0
2015315981	867	rural/clinic	No	22/08/2017	0	0	0
2015315980	867	rural/clinic	No	22/08/2017	0	0	0
2015315982	867	rural/clinic	No	22/08/2017	0	0	0
2015315984	867	rural/clinic	No	22/08/2017	0	0	0
2015315987	867	rural/clinic	No	22/08/2017	0	0	0
2015315986	867	rural/clinic	No	22/08/2017	0	0	0
2015315985	867	rural/clinic	No	22/08/2017	0	0	0
2015315988	867	rural/clinic	No	22/08/2017	0	0	0
2015315989	867	rural/clinic	No	22/08/2017	0	0	0
2015315983	867	rural/clinic	No	22/08/2017	0	0	0
2015315994	867	rural/clinic	No	22/08/2017	0	0	0
2015315993	867	rural/clinic	No	22/08/2017	0	0	0
2015315992	867	rural/clinic	No	22/08/2017	0	0	0
2015315991	867	rural/clinic	No	22/08/2017	0	0	0

2015315990	867	rural/clinic	No	22/08/2017	0	0	0
2015315995	867	rural/clinic	No	22/08/2017	0	0	0
2015315996	867	rural/clinic	No	22/08/2017	0	0	0
2015315997	867	rural/clinic	No	22/08/2017	0	0	0
2015315998	867	rural/clinic	No	22/08/2017	0	0	0
2015316000	867	rural/clinic	No	22/08/2017	0	0	0
2015315999	867	rural/clinic	No	22/08/2017	0	0	0
2015337502	867	rural/clinic	No	22/08/2017	0	0	0
2015337503	867	rural/clinic	No	22/08/2017	0	0	0
2015337504	867	rural/clinic	No	22/08/2017	0	0	0
2015337505	867	rural/clinic	No	22/08/2017	0	0	0
2015337506	867	rural/clinic	No	22/08/2017	0	0	0
2015337508	867	rural/clinic	No	22/08/2017	0	0	0
2015337507	867	rural/clinic	No	22/08/2017	0	0	0
2015337509	867	rural/clinic	No	22/08/2017	0	0	0
2015347701	867	rural/clinic	No	22/08/2017	0	0	0
2015337510	867	rural/clinic	No	22/08/2017	0	0	0
2015337512	867	rural/clinic	No	22/08/2017	0	0	0
2015337511	867	rural/clinic	No	22/08/2017	0	0	0
2015337513	867	rural/clinic	No	22/08/2017	0	0	0
2015337514	867	rural/clinic	No	22/08/2017	0	0	0
2015337517	867	rural/clinic	No	22/08/2017	0	0	0
2015337518	867	rural/clinic	No	22/08/2017	0	0	0
2015337515	867	rural/clinic	No	22/08/2017	0	0	0
2015337516	867	rural/clinic	No	22/08/2017	0	0	0
2015337516	867	rural/clinic	No	22/08/2017	0	0	0
2015337526	867	rural/clinic	No	22/08/2017	0	0	0
2015337525	867	rural/clinic	No	22/08/2017	0	0	0
2015337524	867	rural/clinic	No	22/08/2017	0	0	0
2015337521	867	rural/clinic	No	22/08/2017	0	0	0
2015337519	867	rural/clinic	No	22/08/2017	0	0	0
2015337523	780	rural/clinic	No	22/08/2017	0	0	0
2015337522	780	rural/clinic	No	22/08/2017	0	0	0
2015337520	780	rural/clinic	No	22/08/2017	0	0	0
2015337532	780	rural/clinic	No	22/08/2017	0	0	0
2015337531	780	rural/clinic	No	22/08/2017	0	0	0
2015337527	780	rural/clinic	No	22/08/2017	0	0	0
2015337534	780	rural/clinic	No	22/08/2017	0	0	0
2015337533	780	rural/clinic	No	22/08/2017	0	0	0
2015337530	780	rural/clinic	No	22/08/2017	0	0	0
2015337529	780	rural/clinic	No	22/08/2017	0	0	0
2015337528	780	rural/clinic	No	22/08/2017	0	0	0
2015337535	780	rural/clinic	No	22/08/2017	0	0	0
2015337536	780	rural/clinic	No	22/08/2017	0	0	0
2015337537	780	rural/clinic	No	22/08/2017	0	0	0
2015337546	780	rural/clinic	No	22/08/2017	0	0	0
2015337548	780	rural/clinic	No	22/08/2017	0	0	0
2015337547	780	rural/clinic	No	22/08/2017	0	0	0
2015337540	250	rural/clinic	No	22/08/2017	0	0	0
2015337545	250	rural/clinic	No	22/08/2017	0	0	0
2015337541	250	rural/clinic	No	22/08/2017	0	0	0
2015337542	250	rural/clinic	No	22/08/2017	0	0	0
2015337543	250	rural/clinic	No	22/08/2017	0	0	0
2015337544	250	rural/clinic	No	22/08/2017	0	0	0
2015347702	250	rural/clinic	No	22/08/2017	0	0	0
2015337549	250	rural/clinic	No	22/08/2017	0	0	0
2015347704	250	rural/clinic	No	22/08/2017	0	0	0
2015347705	250	rural/clinic	No	22/08/2017	0	0	0
2015347703	250	rural/clinic	No	22/08/2017	0	0	0
2015347706	250	rural/clinic	No	22/08/2017	0	0	0
2015347707	250	rural/clinic	No	22/08/2017	0	0	0
2015347712	250	rural/clinic	No	22/08/2017	0	0	0
2015347711	250	rural/clinic	No	22/08/2017	0	0	0
2015347710	250	rural/clinic	No	22/08/2017	0	0	0
2015347709	250	rural/clinic	No	22/08/2017	0	0	0
2015347715	250	rural/clinic	No	22/08/2017	0	0	0
2015347708	250	rural/clinic	No	22/08/2017	0	0	0
2015347713	250	rural/clinic	No	22/08/2017	0	0	0
2015347714	250	rural/clinic	No	22/08/2017	0	0	0
2015347716	250	rural/clinic	No	22/08/2017	0	0	0
2015347717	250	rural/clinic	No	22/08/2017	0	0	0
2015347718	250	rural/clinic	No	22/08/2017	0	0	0
2015347719	250	rural/clinic	No	22/08/2017	0	0	0
2015347720	250	rural/clinic	No	22/08/2017	0	0	0
2015347718	250	rural/clinic	No	22/08/2017	0	0	0
2015347724	250	rural/clinic	No	22/08/2017	0	0	0
2015347722	250	rural/clinic	No	22/08/2017	0	0	0
2015347725	250	rural/clinic	No	22/08/2017	0	0	0
2015347723	250	rural/clinic	No	22/08/2017	0	0	0
2015347727	250	rural/clinic	No	22/08/2017	0	0	0
2015347728	250	rural/clinic	No	22/08/2017	0	0	0
2015347729	250	rural/clinic	No	22/08/2017	0	0	0

2015347730	250	rural/clinic	No	22/08/2017	0	0	0
2015347731	250	rural/clinic	No	22/08/2017	0	0	0
2015347732	250	rural/clinic	No	22/08/2017	0	0	0
2015347726	250	rural/clinic	No	22/08/2017	0	0	0
2015347733	250	rural/clinic	No	22/08/2017	0	0	0
2015347733	250	rural/clinic	No	22/08/2017	0	0	0
2015347733	250	rural/clinic	No	22/08/2017	0	0	0
2015347734	250	rural/clinic	No	22/08/2017	0	0	0
2015347739	250	rural/clinic	No	22/08/2017	0	0	0
2015347740	250	rural/clinic	No	22/08/2017	0	0	0
2015347736	250	rural/clinic	No	22/08/2017	0	0	0
2015347735	250	rural/clinic	No	22/08/2017	0	0	0
2015347737	250	rural/clinic	No	22/08/2017	0	0	0
2015347741	250	rural/clinic	No	22/08/2017	0	0	0
2015347738	250	rural/clinic	No	22/08/2017	0	0	0
2015347742	250	rural/clinic	No	22/08/2017	0	0	0
2015347747	250	rural/clinic	No	22/08/2017	0	0	0
2015347745	250	rural/clinic	No	22/08/2017	0	0	0
2015347743	250	rural/clinic	No	22/08/2017	0	0	0
2015347749	250	rural/clinic	No	22/08/2017	0	0	0
2015347744	250	rural/clinic	No	22/08/2017	0	0	0
2015347746	250	rural/clinic	No	22/08/2017	0	0	0
2015347743	250	rural/clinic	No	22/08/2017	0	0	0
2015347743	250	rural/clinic	No	22/08/2017	0	0	0
2011224027	250	rural/clinic	No	22/08/2017	0	0	0
2011224028	250	rural/clinic	No	22/08/2017	0	0	0
2014303289	250	rural/clinic	No	22/08/2017	0	0	0
2014303286	250	rural/clinic	No	22/08/2017	0	0	0
2011224031	250	rural/clinic	No	22/08/2017	0	0	0
2014303292	250	rural/clinic	No	22/08/2017	0	0	0
2014303290	250	rural/clinic	No	22/08/2017	0	0	0
2014303293	250	rural/clinic	No	22/08/2017	0	0	0
2014303295	250	rural/clinic	No	22/08/2017	0	0	0
2014303296	250	rural/clinic	No	22/08/2017	0	0	0
2014303299	250	rural/clinic	No	22/08/2017	0	0	0
2015410451	250	rural/clinic	No	22/08/2017	0	0	0
2015410456	250	rural/clinic	No	22/08/2017	0	0	0
2015410457	250	rural/clinic	No	22/08/2017	0	0	0
2015410459	250	rural/clinic	No	22/08/2017	0	0	0
2015410458	250	rural/clinic	No	22/08/2017	0	0	0
2015410461	250	rural/clinic	No	22/08/2017	0	0	0
2015368859	250	rural/clinic	No	22/08/2017	0	0	0
2015368861	250	rural/clinic	No	22/08/2017	0	0	0
2015368862	250	rural/clinic	No	22/08/2017	0	0	0
2015368859	250	rural/clinic	No	22/08/2017	0	0	0
2015368859	935	rural/clinic	No	22/08/2017	0	0	0
2015339806	216	rural/clinic	No	22/08/2017	0	0	0
2015368864	216	rural/clinic	No	22/08/2017	0	0	0
2015368903	216	rural/clinic	No	22/08/2017	0	0	0
2015368868	216	rural/clinic	No	22/08/2017	0	0	0
2013253936	216	rural/clinic	No	22/08/2017	0	0	0
2015368867	216	rural/clinic	No	22/08/2017	0	0	0
2015368865	216	rural/clinic	No	22/08/2017	0	0	0
2015368866	216	rural/clinic	No	22/08/2017	0	0	0
2013253938	216	rural/clinic	No	22/08/2017	0	0	0
2015368869	216	rural/clinic	No	22/08/2017	0	0	0
2015368871	216	rural/clinic	No	22/08/2017	0	0	0
2015339816	216	rural/clinic	No	22/08/2017	0	0	0
2015368872	216	rural/clinic	No	22/08/2017	0	0	0
2015368874	216	rural/clinic	No	22/08/2017	0	0	0
2015368870	216	rural/clinic	No	22/08/2017	0	0	0
2015368873	216	rural/clinic	No	22/08/2017	0	0	0
2015368875	216	rural/clinic	No	22/08/2017	0	0	0
2015339816	216	rural/clinic	No	22/08/2017	0	0	0
2015339816	216	rural/clinic	No	22/08/2017	0	0	0
2015368878	216	rural/clinic	No	22/08/2017	0	0	0
2015368877	216	rural/clinic	No	22/08/2017	0	0	0
2015368879	216	rural/clinic	No	22/08/2017	0	0	0
2015339821	216	rural/clinic	No	22/08/2017	0	0	0
2015368882	216	rural/clinic	No	22/08/2017	0	0	0
2015368883	216	rural/clinic	No	22/08/2017	0	0	0
2015368888	216	rural/clinic	No	22/08/2017	0	0	0
2015368889	216	rural/clinic	No	22/08/2017	0	0	0
2015368886	216	rural/clinic	No	22/08/2017	0	0	0
2015368887	216	rural/clinic	No	22/08/2017	0	0	0
2015368881	216	rural/clinic	No	22/08/2017	0	0	0
2015368880	216	rural/clinic	No	22/08/2017	0	0	0
2015368884	216	rural/clinic	No	22/08/2017	0	0	0
2015368885	216	rural/clinic	No	22/08/2017	0	0	0
2015368886	216	rural/clinic	No	22/08/2017	0	0	0
2015368881	216	rural/clinic	No	22/08/2017	0	0	0
2015368885	676	rural/clinic	No	22/08/2017	0	0	0

2015368885	676	rural/clinic	No	21/08/2017	0	0	0
2015368890	676	rural/clinic	No	21/08/2017	0	0	0
2013253948	676	rural/clinic	No	21/08/2017	0	0	0
2015368876	676	rural/clinic	No	21/08/2017	0	0	0
2015368892	676	rural/clinic	No	21/08/2017	0	0	0
2015368891	676	rural/clinic	No	21/08/2017	0	0	0
2015368893	676	rural/clinic	No	21/08/2017	0	0	0
2015368894	676	rural/clinic	No	21/08/2017	0	0	0
2015339832	676	rural/clinic	No	21/08/2017	0	0	0
2015368881	676	rural/clinic	No	21/08/2017	0	0	0
2015368881	485	rural/clinic	No	22/08/2017	0	0	0
2015368895	485	rural/clinic	No	22/08/2017	0	0	0
2015368896	99	rural/clinic	No	22/08/2017	0	0	0
2015368897	99	rural/clinic	No	22/08/2017	0	0	0
2015368898	99	rural/clinic	No	22/08/2017	0	0	0
2015368899	99	rural/clinic	No	22/08/2017	0	0	0
2015368899	99	rural/clinic	No	22/08/2017	0	0	0
2015368899	99	rural/clinic	No	22/08/2017	0	0	0
2015368900	99	rural/clinic	No	22/08/2017	0	0	0
2015368908	99	rural/clinic	No	22/08/2017	0	0	0
2015368906	99	rural/clinic	No	22/08/2017	0	0	0
2015368907	99	rural/clinic	No	17/08/2017	0	0	0
2015368909	99	rural/clinic	No	17/08/2017	0	0	0
2015368910	99	rural/clinic	No	22/08/2017	0	0	0
2015368911	99	rural/clinic	No	22/08/2017	0	0	0
2015368912	99	rural/clinic	No	22/08/2017	0	0	0
2015368913	99	rural/clinic	No	22/08/2017	0	0	0
2015368914	99	rural/clinic	No	22/08/2017	0	0	0
2015368917	99	rural/clinic	No	22/08/2017	0	0	0
2015368918	99	rural/clinic	No	22/08/2017	0	0	0
2015368915	99	rural/clinic	No	22/08/2017	0	0	0
2015368919	99	rural/clinic	No	22/08/2017	0	0	0
2015368914	781	rural/clinic	No	22/08/2017	0	0	0
2015368920	781	rural/clinic	No	22/08/2017	0	0	0
2015368921	781	rural/clinic	No	22/08/2017	0	0	0
2015368922	781	rural/clinic	No	22/08/2017	0	0	0
2015368923	781	rural/clinic	No	22/08/2017	0	0	0
2015368924	781	rural/clinic	No	22/08/2017	0	0	0
2014303287	781	rural/clinic	No	22/08/2017	0	0	0
2014353159	781	rural/clinic	No	22/08/2017	0	0	0
2013261230	781	rural/clinic	No	22/08/2017	0	0	0
2014348449	781	rural/clinic	No	22/08/2017	0	0	0
2014348448	781	rural/clinic	No	22/08/2017	0	0	0
2011223621	781	rural/clinic	No	22/08/2017	0	0	0
2011223618	781	rural/clinic	No	22/08/2017	0	0	0
2012259150	781	rural/clinic	No	22/08/2017	0	0	0
2012259149	781	rural/clinic	No	22/08/2017	0	0	0
2014344475	781	rural/clinic	No	22/08/2017	0	0	0
2014288588	781	rural/clinic	No	22/08/2017	0	0	0
2014288586	781	rural/clinic	No	22/08/2017	0	0	0
2014288585	781	rural/clinic	No	22/08/2017	0	0	0
2014288584	781	rural/clinic	No	22/08/2017	0	0	0
2014288583	781	rural/clinic	No	22/08/2017	0	0	0
2015344347	781	rural/clinic	No	22/08/2017	0	0	0
2015334662	781	rural/clinic	No	22/08/2017	0	0	0
2015303487	781	rural/clinic	No	22/08/2017	0	0	0
2011128286	781	rural/clinic	No	22/08/2017	0	0	0
2014334681	781	rural/clinic	No	22/08/2017	0	0	0
2014334371	781	rural/clinic	No	22/08/2017	0	0	0
2015349070	781	rural/clinic	No	22/08/2017	0	0	0
2015349071	781	rural/clinic	No	22/08/2017	0	0	0
2014347740	781	rural/clinic	No	22/08/2017	0	0	0
2015359630	781	rural/clinic	No	22/08/2017	0	0	0
2015359629	781	rural/clinic	No	22/08/2017	0	0	0
2015359628	781	rural/clinic	No	22/08/2017	0	0	0
2011128287	781	rural/clinic	No	22/08/2017	0	0	0
2015359627	781	rural/clinic	No	22/08/2017	0	0	0
2015359623	781	rural/clinic	No	22/08/2017	0	0	0
2014347741	781	rural/clinic	No	22/08/2017	0	0	0
2012284623	781	rural/clinic	No	22/08/2017	0	0	0
2014287991	781	rural/clinic	No	22/08/2017	0	0	0
2015359626	781	rural/clinic	No	22/08/2017	0	0	0
2015359625	781	rural/clinic	No	22/08/2017	0	0	0
2015359624	781	rural/clinic	No	22/08/2017	0	0	0
2013254147	781	rural/clinic	No	22/08/2017	0	0	0
2013254148	781	rural/clinic	No	22/08/2017	0	0	0
2013256302	781	rural/clinic	No	22/08/2017	0	0	0
2015417092	781	rural/clinic	No	22/08/2017	0	0	0
2015417091	781	rural/clinic	No	22/08/2017	0	0	0
2015379332	781	rural/clinic	No	22/08/2017	0	0	0
2015379330	781	rural/clinic	No	22/08/2017	0	0	0
2015404670	781	rural/clinic	No	22/08/2017	0	0	0

2015404668	781	rural/clinic	No	22/08/2017	0	0	0
2015404667	781	rural/clinic	No	22/08/2017	0	0	0
2015340381	781	rural/clinic	No	22/08/2017	0	0	0
2015405805	165	rural/clinic	No	24/08/2017	0	0	0
2011144113	165	rural/clinic	No	24/08/2017	0	0	0
2015362829	165	rural/clinic	No	24/08/2017	0	0	0
2011144114	165	rural/clinic	No	24/08/2017	0	0	0
2015362830	165	rural/clinic	No	24/08/2017	0	0	0
2013252885	165	rural/clinic	No	24/08/2017	0	0	0
2014385697	165	rural/clinic	No	24/08/2017	0	0	0
2014385698	165	rural/clinic	No	24/08/2017	0	0	0
2015332852	165	rural/clinic	No	24/08/2017	0	0	0
2015404672	165	rural/clinic	No	24/08/2017	0	0	0
2015404671	165	rural/clinic	No	24/08/2017	0	0	0
2015404673	165	rural/clinic	No	24/08/2017	0	0	0
2015369097	165	rural/clinic	No	24/08/2017	0	0	0
2015369098	165	rural/clinic	No	24/08/2017	0	0	0
2015404674	165	rural/clinic	No	24/08/2017	0	0	0
2015369099	165	rural/clinic	No	24/08/2017	0	0	0
2015336706	165	rural/clinic	No	24/08/2017	0	0	0
2015360865	165	rural/clinic	No	24/08/2017	0	0	0
2015364341	165	rural/clinic	No	24/08/2017	0	0	0
2015360864	165	rural/clinic	No	24/08/2017	0	0	0
2015364342	165	rural/clinic	No	24/08/2017	0	0	0
2015286348	165	rural/clinic	No	24/08/2017	0	0	0
2015286349	165	rural/clinic	No	24/08/2017	0	0	0
2015286341	165	rural/clinic	No	24/08/2017	0	0	0
2011142272	165	rural/clinic	No	24/08/2017	0	0	0
2015286342	165	rural/clinic	No	24/08/2017	0	0	0
2015360862	165	rural/clinic	No	24/08/2017	0	0	0
2015360861	642	rural/clinic	No	24/08/2017	0	0	0
2015289230	642	rural/clinic	No	24/08/2017	0	0	0
2015286344	642	rural/clinic	No	24/08/2017	0	0	0
2015289231	642	rural/clinic	No	24/08/2017	0	0	0
2015289232	642	rural/clinic	No	24/08/2017	0	0	0
2015289233	642	rural/clinic	No	24/08/2017	0	0	0
2015289234	642	rural/clinic	No	24/08/2017	0	0	0
2012381662	642	rural/clinic	No	24/08/2017	0	0	0
2012381660	642	rural/clinic	No	24/08/2017	0	0	0
2012381658	642	rural/clinic	No	24/08/2017	0	0	0
2012381661	642	rural/clinic	No	24/08/2017	0	0	0
2012381655	642	rural/clinic	No	24/08/2017	0	0	0
2012291386	551	rural/clinic	No	24/08/2017	0	0	0
2012291387	551	rural/clinic	No	24/08/2017	0	0	0
2015360863	551	rural/clinic	No	24/08/2017	0	0	0
2015305824	551	rural/clinic	No	24/08/2017	0	0	0
2015305825	551	rural/clinic	No	24/08/2017	0	0	0
2015305826	551	rural/clinic	No	24/08/2017	0	0	0
2014342548	551	rural/clinic	No	24/08/2017	0	0	0
2014342546	936	rural/clinic	No	24/08/2017	0	0	0
2015305827	936	rural/clinic	No	24/08/2017	0	0	0
2015305828	936	rural/clinic	No	24/08/2017	0	0	0
2012291389	936	rural/clinic	No	24/08/2017	0	0	0
2015305829	936	rural/clinic	No	24/08/2017	0	0	0
2012379909	936	rural/clinic	No	24/08/2017	0	0	0
2015402670	936	rural/clinic	No	24/08/2017	0	0	0
2015402671	936	rural/clinic	No	24/08/2017	0	0	0
2015402672	936	rural/clinic	No	24/08/2017	0	0	0
2014297848	936	rural/clinic	No	24/08/2017	0	0	0
2015402673	936	rural/clinic	No	24/08/2017	0	0	0
2015402674	936	rural/clinic	No	24/08/2017	0	0	0
2015403307	936	rural/clinic	No	24/08/2017	0	0	0
2015402675	936	rural/clinic	No	24/08/2017	0	0	0
2012291388	936	rural/clinic	No	24/08/2017	0	0	0
2011135075	936	rural/clinic	No	24/08/2017	0	0	0
2014297791	936	rural/clinic	No	24/08/2017	0	0	0
2014307344	936	rural/clinic	No	24/08/2017	0	0	0
2015368899	936	rural/clinic	No	24/08/2017	0	0	0
2015403318	936	rural/clinic	No	24/08/2017	0	0	0
2015403306	936	rural/clinic	No	24/08/2017	0	0	0
2015369943	936	rural/clinic	No	24/08/2017	0	0	0
2015402676	936	rural/clinic	No	24/08/2017	0	0	0
2015402665	936	rural/clinic	No	24/08/2017	0	0	0
2015339158	936	rural/clinic	No	24/08/2017	0	0	0
2015402666	936	rural/clinic	No	24/08/2017	0	0	0
2015402677	936	rural/clinic	No	24/08/2017	0	0	0
2015339611	936	rural/clinic	No	24/08/2017	0	0	0
2015339030	936	rural/clinic	No	24/08/2017	0	0	0
2015402667	936	rural/clinic	No	24/08/2017	0	0	0
2015361190	936	rural/clinic	No	24/08/2017	0	0	0
2015402668	936	rural/clinic	No	24/08/2017	0	0	0
2015361191	936	rural/clinic	No	24/08/2017	0	0	0

2015361192	936	rural/clinic	No	24/08/2017	0	0	0
2015361194	936	rural/clinic	No	24/08/2017	0	0	0
2015402669	936	rural/clinic	No	24/08/2017	0	0	0
2011135074	936	rural/clinic	No	24/08/2017	0	0	0
2015361184	936	rural/clinic	No	24/08/2017	0	0	0
2015376667	936	rural/clinic	No	24/08/2017	0	0	0
2015361185	936	rural/clinic	No	24/08/2017	0	0	0
2015336663	936	rural/clinic	No	24/08/2017	0	0	0
2015361186	936	rural/clinic	No	24/08/2017	0	0	0
2012363987	936	rural/clinic	No	24/08/2017	0	0	0
2015361187	936	rural/clinic	No	24/08/2017	0	0	0
2015352806	936	rural/clinic	No	24/08/2017	0	0	0
2015336660	936	rural/clinic	No	24/08/2017	0	0	0
2015361188	936	rural/clinic	No	24/08/2017	0	0	0
2015352807	936	rural/clinic	No	24/08/2017	0	0	0
2015361189	936	rural/clinic	No	24/08/2017	0	0	0
2014307420	936	rural/clinic	No	24/08/2017	0	0	0
2014333610	936	rural/clinic	No	24/08/2017	0	0	0
2015376668	936	rural/clinic	No	24/08/2017	0	0	0
2015373895	936	rural/clinic	No	26/08/2017	0	0	0
2014333611	936	rural/clinic	No	23/08/2017	0	0	0
2012363984	936	rural/clinic	No	24/08/2017	0	0	0
2015373896	936	rural/clinic	No	25/08/2017	0	0	0
2012363986	936	rural/clinic	No	24/08/2017	0	0	0
2012363985	936	rural/clinic	No	24/08/2017	0	0	0
2014333608	936	rural/clinic	No	24/08/2017	0	0	0
2015373893	936	rural/clinic	No	25/08/2017	0	0	0
2011142910	936	rural/clinic	No	24/08/2017	0	0	0
2015289194	936	rural/clinic	No	24/08/2017	0	0	0
2014333607	936	rural/clinic	No	24/08/2017	0	0	0
2015339159	936	rural/clinic	No	24/08/2017	0	0	0
2015286347	936	rural/clinic	No	24/08/2017	0	0	0
2015352805	936	rural/clinic	No	24/08/2017	0	0	0
2015286345	936	rural/clinic	No	24/08/2017	0	0	0
2014333609	936	rural/clinic	No	24/08/2017	0	0	0
2015286343	936	rural/clinic	No	24/08/2017	0	0	0
2015326604	936	rural/clinic	No	24/08/2017	0	0	0
2015336707	936	rural/clinic	No	24/08/2017	0	0	0
2015326605	936	rural/clinic	No	24/08/2017	0	0	0
2013272764	936	rural/clinic	No	24/08/2017	0	0	0
2015326607	936	rural/clinic	No	24/08/2017	0	0	0
2015326606	936	rural/clinic	No	24/08/2017	0	0	0
2012275229	936	rural/clinic	No	24/08/2017	0	0	0
2013247032	936	rural/clinic	No	24/08/2017	0	0	0
2012275227	936	rural/clinic	No	24/08/2017	0	0	0
2014350463	936	rural/clinic	No	24/08/2017	0	0	0
2012275226	936	rural/clinic	No	24/08/2017	0	0	0
2014317439	936	rural/clinic	No	24/08/2017	0	0	0
2012275225	936	rural/clinic	No	24/08/2017	0	0	0
2014317651	936	rural/clinic	No	24/08/2017	0	0	0
2012275222	936	rural/clinic	No	24/08/2017	0	0	0
2014317652	936	rural/clinic	No	24/08/2017	0	0	0
2012275223	936	rural/clinic	No	24/08/2017	0	0	0
2014317654	936	rural/clinic	No	24/08/2017	0	0	0
2015326915	936	rural/clinic	No	17/08/2017	0	0	0
2015360209	936	rural/clinic	No	24/08/2017	0	0	0
2015372824	936	rural/clinic	No	22/08/2017	0	0	0
2015372824	936	rural/clinic	No	22/08/2017	0	0	0
2014378275	936	rural/clinic	No	24/08/2017	0	0	0
2015297173	936	rural/clinic	No	22/08/2017	0	0	0
2015297173	936	rural/clinic	No	22/08/2017	0	0	0
2015404979	936	rural/clinic	No	22/08/2017	0	0	0
2015404979	936	rural/clinic	No	22/08/2017	0	0	0
2013264224	936	rural/clinic	No	24/08/2017	0	0	0
2015368618	936	rural/clinic	No	22/08/2017	0	0	0
2015340908	936	rural/clinic	No	22/08/2017	0	0	0
2015340908	936	rural/clinic	No	22/08/2017	0	0	0
2015325945	936	rural/clinic	No	22/08/2017	0	0	0
2015325945	936	rural/clinic	No	22/08/2017	0	0	0
2014329030	936	rural/clinic	No	22/08/2017	0	0	0
2014329030	936	rural/clinic	No	22/08/2017	0	0	0
2013255746	936	rural/clinic	No	24/08/2017	0	0	0
2013264181	936	rural/clinic	No	24/08/2017	0	0	0
2013264182	936	rural/clinic	No	24/08/2017	0	0	0
2011157150	936	rural/clinic	No	24/08/2017	0	0	0
2015377441	936	rural/clinic	No	24/08/2017	0	0	0
2015377439	936	rural/clinic	No	24/08/2017	0	0	0
2015377440	936	rural/clinic	No	24/08/2017	0	0	0
2014317653	936	rural/clinic	No	24/08/2017	0	0	0
2012370169	936	rural/clinic	No	24/08/2017	0	0	0
2012275224	936	rural/clinic	No	24/08/2017	0	0	0
2014300351	936	rural/clinic	No	24/08/2017	0	0	0

2015414400	936	rural/clinic	No	24/08/2017	0	0	0
2014300352	936	rural/clinic	No	24/08/2017	0	0	0
2014300353	936	rural/clinic	No	24/08/2017	0	0	0
2015372030	936	rural/clinic	No	24/08/2017	0	0	0
2015372031	936	rural/clinic	No	24/08/2017	0	0	0
2011155242	936	rural/clinic	No	24/08/2017	0	0	0
2015409037	936	rural/clinic	No	24/08/2017	0	0	0
2015409036	936	rural/clinic	No	24/08/2017	0	0	0
2015409038	936	rural/clinic	No	24/08/2017	0	0	0
2014358033	936	rural/clinic	No	24/08/2017	0	0	0
2015409035	936	rural/clinic	No	24/08/2017	0	0	0
2014357337	936	rural/clinic	No	24/08/2017	0	0	0
2014357336	936	rural/clinic	No	24/08/2017	0	0	0
2014361228	936	rural/clinic	No	24/08/2017	0	0	0
2015355928	936	rural/clinic	No	24/08/2017	0	0	0
2015355927	936	rural/clinic	No	24/08/2017	0	0	0
2015385549	936	rural/clinic	No	24/08/2017	0	0	0
2015289229	936	rural/clinic	No	24/08/2017	0	0	0
2015345092	936	rural/clinic	No	24/08/2017	0	0	0
2015289228	936	rural/clinic	No	24/08/2017	0	0	0
2015289227	936	rural/clinic	No	24/08/2017	0	0	0
2015345093	936	rural/clinic	No	24/08/2017	0	0	0
2015289226	936	rural/clinic	No	24/08/2017	0	0	0
2015305099	936	rural/clinic	No	24/08/2017	0	0	0
2015289225	936	rural/clinic	No	24/08/2017	0	0	0
2015289224	936	rural/clinic	No	24/08/2017	0	0	0
2015360538	936	rural/clinic	No	24/08/2017	0	0	0
2015289223	936	rural/clinic	No	24/08/2017	0	0	0
2015289222	936	rural/clinic	No	24/08/2017	0	0	0
2012359170	936	rural/clinic	No	24/08/2017	0	0	0
2014314236	936	rural/clinic	No	24/08/2017	0	0	0
2015402025	936	rural/clinic	No	24/08/2017	0	0	0
2015402024	552	rural/clinic	No	24/08/2017	0	0	0
2015402023	552	rural/clinic	No	24/08/2017	0	0	0
2015402022	552	rural/clinic	No	24/08/2017	0	0	0
2015402021	552	rural/clinic	No	24/08/2017	0	0	0
2015360539	552	rural/clinic	No	24/08/2017	0	0	0
2015402020	552	rural/clinic	No	24/08/2017	0	0	0
2015360535	552	rural/clinic	No	24/08/2017	0	0	0
2015360537	552	rural/clinic	No	24/08/2017	0	0	0
2015332374	552	rural/clinic	No	24/08/2017	0	0	0
2015332375	552	rural/clinic	No	24/08/2017	0	0	0
2015360540	552	rural/clinic	No	24/08/2017	0	0	0
2015360536	552	rural/clinic	No	24/08/2017	0	0	0
2015332376	552	rural/clinic	No	24/08/2017	0	0	0
2015332377	552	rural/clinic	No	24/08/2017	0	0	0
2011205726	552	rural/clinic	No	24/08/2017	0	0	0
2012294479	552	rural/clinic	No	24/08/2017	0	0	0
2015332479	552	rural/clinic	No	24/08/2017	0	0	0
2015332372	552	rural/clinic	No	24/08/2017	0	0	0
2015360531	552	rural/clinic	No	24/08/2017	0	0	0
2015332482	552	rural/clinic	No	24/08/2017	0	0	0
2015402019	552	rural/clinic	No	24/08/2017	0	0	0
2015332481	552	rural/clinic	No	24/08/2017	0	0	0
2015332484	552	rural/clinic	No	24/08/2017	0	0	0
2015402018	552	rural/clinic	No	24/08/2017	0	0	0
2015332373	552	rural/clinic	No	24/08/2017	0	0	0
2015402017	552	rural/clinic	No	24/08/2017	0	0	0
2015332480	552	rural/clinic	No	24/08/2017	0	0	0
2015408686	552	rural/clinic	No	24/08/2017	0	0	0
2014312413	552	rural/clinic	No	24/08/2017	0	0	0
2015408687	552	rural/clinic	No	24/08/2017	0	0	0
2012268998	552	rural/clinic	No	24/08/2017	0	0	0
2015408672	552	rural/clinic	No	24/08/2017	0	0	0
2015358322	552	rural/clinic	No	24/08/2017	0	0	0
2015373892	552	rural/clinic	No	24/08/2017	0	0	0
2015358320	552	rural/clinic	No	24/08/2017	0	0	0
2015408688	552	rural/clinic	No	24/08/2017	0	0	0
2015358323	552	rural/clinic	No	24/08/2017	0	0	0
2015358321	552	rural/clinic	No	24/08/2017	0	0	0
2015358324	552	rural/clinic	No	24/08/2017	0	0	0
2015358316	552	rural/clinic	No	24/08/2017	0	0	0
2015358317	552	rural/clinic	No	24/08/2017	0	0	0
2015358318	552	rural/clinic	No	24/08/2017	0	0	0
2015358319	552	rural/clinic	No	24/08/2017	0	0	0
2015401169	535	rural/clinic	No	24/08/2017	0	0	0
2015408685	535	rural/clinic	No	24/08/2017	0	0	0
2015401168	535	rural/clinic	No	24/08/2017	0	0	0
2015408689	535	rural/clinic	No	24/08/2017	0	0	0
2015401167	535	rural/clinic	No	24/08/2017	0	0	0
2012336636	535	rural/clinic	No	24/08/2017	0	0	0
2015338884	535	rural/clinic	No	24/08/2017	0	0	0

2015401164	535	rural/clinic	No	24/08/2017	0	0	0
2015403314	535	rural/clinic	No	24/08/2017	0	0	0
2015401166	535	rural/clinic	No	24/08/2017	0	0	0
2015401165	535	rural/clinic	No	24/08/2017	0	0	0
2015362901	535	rural/clinic	No	24/08/2017	0	0	0
2015362902	535	rural/clinic	No	24/08/2017	0	0	0
2015362903	535	rural/clinic	No	24/08/2017	0	0	0
2015403310	535	rural/clinic	No	24/08/2017	0	0	0
2011135073	535	rural/clinic	No	24/08/2017	0	0	0
2015362904	535	rural/clinic	No	24/08/2017	0	0	0
2011135072	535	rural/clinic	No	24/08/2017	0	0	0
2011135071	535	rural/clinic	No	24/08/2017	0	0	0
2015336659	535	rural/clinic	No	24/08/2017	0	0	0
2015362905	535	rural/clinic	No	24/08/2017	0	0	0
2015336661	535	rural/clinic	No	24/08/2017	0	0	0
2015362907	535	rural/clinic	No	24/08/2017	0	0	0
2015362908	535	rural/clinic	No	24/08/2017	0	0	0
2015336658	535	rural/clinic	No	24/08/2017	0	0	0
2015362909	535	rural/clinic	No	24/08/2017	0	0	0
2012362576	535	rural/clinic	No	24/08/2017	0	0	0
2015362912	535	rural/clinic	No	24/08/2017	0	0	0
2012362574	535	rural/clinic	No	24/08/2017	0	0	0
2015362911	535	rural/clinic	No	24/08/2017	0	0	0
2015362910	535	rural/clinic	No	24/08/2017	0	0	0
2012362575	535	rural/clinic	No	24/08/2017	0	0	0
2015382752	535	rural/clinic	No	24/08/2017	0	0	0
2015397668	782	rural/clinic	No	28/08/2017	0	0	0
2015397669	782	rural/clinic	No	28/08/2017	0	0	0
2015326995	782	rural/clinic	No	24/08/2017	0	0	0
2015326994	782	rural/clinic	No	24/08/2017	0	0	0
2015397670	782	rural/clinic	No	28/08/2017	0	0	0
2015397671	782	rural/clinic	No	28/08/2017	0	0	0
2015397672	782	rural/clinic	No	28/08/2017	0	0	0
2015403315	782	rural/clinic	No	24/08/2017	0	0	0
2015397673	782	rural/clinic	No	28/08/2017	0	0	0
2014297847	782	rural/clinic	No	24/08/2017	0	0	0
2015338886	782	rural/clinic	No	24/08/2017	0	0	0
2015338887	782	rural/clinic	No	24/08/2017	0	0	0
2015403311	782	rural/clinic	No	24/08/2017	0	0	0
2015338888	782	rural/clinic	No	24/08/2017	0	0	0
2015338889	782	rural/clinic	No	24/08/2017	0	0	0
2015386808	782	rural/clinic	No	24/08/2017	0	0	0
2015403317	782	rural/clinic	No	24/08/2017	0	0	0
2015338890	782	rural/clinic	No	24/08/2017	0	0	0
2015386809	782	rural/clinic	No	24/08/2017	0	0	0
2015403312	782	rural/clinic	No	24/08/2017	0	0	0
2015386807	342	rural/clinic	No	24/08/2017	0	0	0
2015338891	342	rural/clinic	No	24/08/2017	0	0	0
2015403308	342	rural/clinic	No	24/08/2017	0	0	0
2015386804	342	rural/clinic	No	24/08/2017	0	0	0
2015362832	342	rural/clinic	No	24/08/2017	0	0	0
2014316080	342	rural/clinic	No	24/08/2017	0	0	0
2015386820	342	rural/clinic	No	24/08/2017	0	0	0
2014316077	342	rural/clinic	No	24/08/2017	0	0	0
2015386818	342	rural/clinic	No	24/08/2017	0	0	0
2014374049	342	rural/clinic	No	24/08/2017	0	0	0
2015386815	342	rural/clinic	No	24/08/2017	0	0	0
2015386814	342	rural/clinic	No	24/08/2017	0	0	0
2015390451	342	rural/clinic	No	24/08/2017	0	0	0
2015386813	342	rural/clinic	No	24/08/2017	0	0	0
2014374050	342	rural/clinic	No	24/08/2017	0	0	0
2015386812	342	rural/clinic	No	24/08/2017	0	0	0
2015386811	342	rural/clinic	No	24/08/2017	0	0	0
2014298245	342	rural/clinic	No	24/08/2017	0	0	0
2015386810	342	rural/clinic	No	24/08/2017	0	0	0
2015390452	342	rural/clinic	No	24/08/2017	0	0	0
2015386816	342	rural/clinic	No	24/08/2017	0	0	0
2015369784	342	rural/clinic	No	24/08/2017	0	0	0
2015332851	342	rural/clinic	No	24/08/2017	0	0	0
2012390655	408	rural/clinic	No	24/08/2017	0	0	0
2012390654	408	rural/clinic	No	24/08/2017	0	0	0
2012390653	408	rural/clinic	No	24/08/2017	0	0	0
2012390652	408	rural/clinic	No	24/08/2017	0	0	0
2012388300	408	rural/clinic	No	24/08/2017	0	0	0
2012390656	408	rural/clinic	No	24/08/2017	0	0	0
2012390651	408	rural/clinic	No	24/08/2017	0	0	0
2012388299	408	rural/clinic	No	24/08/2017	0	0	0
2015372868	408	rural/clinic	No	22/08/2017	0	0	0
2015372868	408	rural/clinic	No	22/08/2017	0	0	0
2015368738	408	rural/clinic	No	17/08/2017	0	0	0
2015368738	408	rural/clinic	No	17/08/2017	0	0	0
2015359625	408	rural/clinic	No	22/08/2017	0	0	0

2015359625	408	rural/clinic	No	22/08/2017	0	0	0
2011135077	408	rural/clinic	No	24/08/2017	0	0	0
2014342547	408	rural/clinic	No	24/08/2017	0	0	0
2014298244	408	rural/clinic	No	24/08/2017	0	0	0
2012275228	408	rural/clinic	No	24/08/2017	0	0	0
2015362906	408	rural/clinic	No	24/08/2017	0	0	0
2012244293	408	rural/clinic	No	24/08/2017	0	0	0
2015384366	408	rural/clinic	No	24/08/2017	0	0	0
2015362546	408	rural/clinic	No	29/08/2017	0	0	0
2015337380	408	rural/clinic	No	29/08/2017	0	0	0
2015364968	408	rural/clinic	No	29/08/2017	0	0	0
2015287396	408	rural/clinic	No	29/08/2017	0	0	0
2015287395	408	rural/clinic	No	29/08/2017	0	0	0
2014327605	408	rural/clinic	No	10/08/2017	0	0	0
2012368549	408	rural/clinic	No	29/08/2017	0	0	0
2013283508	408	rural/clinic	No	29/08/2017	0	0	0
2012253978	408	rural/clinic	No	29/08/2017	0	0	0
2015349026	217	rural/clinic	No	29/08/2017	0	0	0
2014298595	217	rural/clinic	No	29/08/2017	0	0	0
2015417093	217	rural/clinic	No	29/08/2017	0	0	0
2015417094	217	rural/clinic	No	29/08/2017	0	0	0
2015406349	217	rural/clinic	No	29/08/2017	0	0	0
2015406401	217	rural/clinic	No	29/08/2017	0	0	0
2015313002	217	rural/clinic	No	29/08/2017	0	0	0
2014321529	217	rural/clinic	No	29/08/2017	0	0	0
2015415070	217	rural/clinic	No	29/08/2017	0	0	0
2015413365	217	rural/clinic	No	29/08/2017	0	0	0
2015382962	217	rural/clinic	No	29/08/2017	0	0	0
2015382603	218	rural/clinic	No	29/08/2017	0	0	0
2015382606	218	rural/clinic	No	29/08/2017	0	0	0
2015382963	218	rural/clinic	No	29/08/2017	0	0	0
2015382964	218	rural/clinic	No	29/08/2017	0	0	0
2015382961	218	rural/clinic	No	29/08/2017	0	0	0
2015382965	218	rural/clinic	No	29/08/2017	0	0	0
2015382966	218	rural/clinic	No	29/08/2017	0	0	0
2015368619	218	rural/clinic	No	29/08/2017	0	0	0
2015368620	218	rural/clinic	No	29/08/2017	0	0	0
2015382607	218	rural/clinic	No	29/08/2017	0	0	0
2015382602	218	rural/clinic	No	29/08/2017	0	0	0
2015368621	218	rural/clinic	No	29/08/2017	0	0	0
2012295823	218	rural/clinic	No	29/08/2017	0	0	0
2015368622	218	rural/clinic	No	29/08/2017	0	0	0
2012295824	218	rural/clinic	No	29/08/2017	0	0	0
2015368623	218	rural/clinic	No	29/08/2017	0	0	0
2012295825	218	rural/clinic	No	29/08/2017	0	0	0
2014350638	218	rural/clinic	No	29/08/2017	0	0	0
2012295826	218	rural/clinic	No	29/08/2017	0	0	0
2012295827	218	rural/clinic	No	29/08/2017	0	0	0
2012295828	218	rural/clinic	No	29/08/2017	0	0	0
2014363196	218	rural/clinic	No	29/08/2017	0	0	0
2014350368	218	rural/clinic	No	29/08/2017	0	0	0
2015364964	218	rural/clinic	No	29/08/2017	0	0	0
2015363078	218	rural/clinic	No	29/08/2017	0	0	0
2015364965	218	rural/clinic	No	29/08/2017	0	0	0
2015415299	218	rural/clinic	No	29/08/2017	0	0	0
2015364966	218	rural/clinic	No	29/08/2017	0	0	0
2015364967	218	rural/clinic	No	29/08/2017	0	0	0
2015364969	218	rural/clinic	No	29/08/2017	0	0	0
2015364970	218	rural/clinic	No	29/08/2017	0	0	0
2012322043	218	rural/clinic	No	29/08/2017	0	0	0
2015362547	218	rural/clinic	No	29/08/2017	0	0	0
2012322044	218	rural/clinic	No	29/08/2017	0	0	0
2015364971	218	rural/clinic	No	29/08/2017	0	0	0
2015362545	218	rural/clinic	No	29/08/2017	0	0	0
2012322045	218	rural/clinic	No	29/08/2017	0	0	0
2011142800	218	rural/clinic	No	29/08/2017	0	0	0
2015362544	177	rural/clinic	No	29/08/2017	0	0	0
2014335600	177	rural/clinic	No	29/08/2017	0	0	0
2015382593	177	rural/clinic	No	29/08/2017	0	0	0
2015293847	177	rural/clinic	No	29/08/2017	0	0	0
2015377766	177	rural/clinic	No	29/08/2017	0	0	0
2015382594	177	rural/clinic	No	29/08/2017	0	0	0
2015293848	177	rural/clinic	No	29/08/2017	0	0	0
2015377767	177	rural/clinic	No	29/08/2017	0	0	0
2015382595	177	rural/clinic	No	29/08/2017	0	0	0
2015377768	177	rural/clinic	No	29/08/2017	0	0	0
2015293849	177	rural/clinic	No	29/08/2017	0	0	0
2015382596	177	rural/clinic	No	29/08/2017	0	0	0
2015382599	177	rural/clinic	No	29/08/2017	0	0	0
2014375541	177	rural/clinic	No	29/08/2017	0	0	0
2015382600	177	rural/clinic	No	29/08/2017	0	0	0
2014375540	177	rural/clinic	No	29/08/2017	0	0	0

2015382601	177	rural/clinic	No	29/08/2017	0	0	0
2015382605	177	rural/clinic	No	29/08/2017	0	0	0
2014375539	177	rural/clinic	No	29/08/2017	0	0	0
2015368830	177	rural/clinic	No	29/08/2017	0	0	0
2015382608	177	rural/clinic	No	29/08/2017	0	0	0
2015368829	177	rural/clinic	No	29/08/2017	0	0	0
2015382604	177	rural/clinic	No	29/08/2017	0	0	0
2014375538	177	rural/clinic	No	29/08/2017	0	0	0
2015349028	177	rural/clinic	No	29/08/2017	0	0	0
2015414281	177	rural/clinic	No	29/08/2017	0	0	0
2015375426	177	rural/clinic	No	29/08/2017	0	0	0
2015359054	177	rural/clinic	No	29/08/2017	0	0	0
2015364540	177	rural/clinic	No	29/08/2017	0	0	0
2015359053	177	rural/clinic	No	29/08/2017	0	0	0
2015364541	177	rural/clinic	No	29/08/2017	0	0	0
2015349025	177	rural/clinic	No	29/08/2017	0	0	0
2015364539	177	rural/clinic	No	29/08/2017	0	0	0
2014303725	177	rural/clinic	No	29/08/2017	0	0	0
2015415064	177	rural/clinic	No	29/08/2017	0	0	0
2015264702	177	rural/clinic	No	29/08/2017	0	0	0
2015375062	177	rural/clinic	No	29/08/2017	0	0	0
2014298599	177	district/faith-based	No	29/08/2017	1	0	0
2014321528	177	district/faith-based	No	29/08/2017	1	0	0
2014288145	177	district/faith-based	No	29/08/2017	1	0	0
2015364535	177	district/faith-based	No	29/08/2017	1	0	0
2015364534	177	district/faith-based	No	29/08/2017	1	0	0
2014307748	177	district/faith-based	No	29/08/2017	1	0	0
2014307747	177	district/faith-based	No	29/08/2017	1	0	0
2015415071	177	district/faith-based	No	29/08/2017	1	0	0
2015382597	177	district/faith-based	No	29/08/2017	1	0	0
2015382598	177	district/faith-based	No	29/08/2017	1	0	0
2014321429	177	district/faith-based	No	29/08/2017	1	0	0
2011216418	177	district/faith-based	No	29/08/2017	1	0	0
2015419077	177	district/faith-based	No	29/08/2017	1	0	0
2015364537	177	district/faith-based	No	29/08/2017	1	0	0
2014298598	177	district/faith-based	No	29/08/2017	1	0	0
2013264085	677	rural/clinic	No	29/08/2017	0	0	0
2015364536	677	rural/clinic	No	29/08/2017	0	0	0
2013264084	677	rural/clinic	No	29/08/2017	0	0	0
2014298596	677	rural/clinic	No	29/08/2017	0	0	0
2015338892	677	rural/clinic	No	29/08/2017	0	0	0
2014351231	677	rural/clinic	No	29/08/2017	0	0	0
2015349027	677	rural/clinic	No	29/08/2017	0	0	0
2015338893	677	rural/clinic	No	29/08/2017	0	0	0
2015338894	677	rural/clinic	No	29/08/2017	0	0	0
2015338895	677	rural/clinic	No	29/08/2017	0	0	0
2015290228	677	rural/clinic	No	29/08/2017	0	0	0
2015290227	677	rural/clinic	No	29/08/2017	0	0	0
2010099938	677	rural/clinic	No	29/08/2017	0	0	0
2014303727	677	rural/clinic	No	29/08/2017	0	0	0
2015340754	677	rural/clinic	No	29/08/2017	0	0	0
2015290229	677	rural/clinic	No	29/08/2017	0	0	0
2010099939	677	rural/clinic	No	29/08/2017	0	0	0
2015290220	677	rural/clinic	No	29/08/2017	0	0	0
2011191299	677	rural/clinic	No	29/08/2017	0	0	0
2014324708	677	rural/clinic	No	29/08/2017	0	0	0
2015375909	677	rural/clinic	No	29/08/2017	0	0	0
2011191300	677	rural/clinic	No	29/08/2017	0	0	0
2011193278	677	rural/clinic	No	29/08/2017	0	0	0
2012289791	677	rural/clinic	No	29/08/2017	0	0	0
2012289621	677	rural/clinic	No	29/08/2017	0	0	0
2012275994	677	rural/clinic	No	29/08/2017	0	0	0
2012253979	677	rural/clinic	No	29/08/2017	0	0	0
2012371454	677	rural/clinic	No	29/08/2017	0	0	0
2015375107	677	rural/clinic	No	29/08/2017	0	0	0
2015332853	677	rural/clinic	No	29/08/2017	0	0	0
2014370441	677	rural/clinic	No	29/08/2017	0	0	0
2014288146	677	rural/clinic	No	29/08/2017	0	0	0
2015385869	677	rural/clinic	No	29/08/2017	0	0	0
2012253934	677	rural/clinic	No	29/08/2017	0	0	0
2015324487	677	rural/clinic	No	29/08/2017	0	0	0
2015290036	677	rural/clinic	No	29/08/2017	0	0	0
201066590	677	rural/clinic	No	29/08/2017	0	0	0
2015290214	677	rural/clinic	No	29/08/2017	0	0	0
2015355536	677	rural/clinic	No	29/08/2017	0	0	0
2015290219	677	rural/clinic	No	29/08/2017	0	0	0
2015324488	677	rural/clinic	No	29/08/2017	0	0	0
2015290217	677	rural/clinic	No	29/08/2017	0	0	0
2014303720	677	rural/clinic	No	29/08/2017	0	0	0
2014299929	677	rural/clinic	No	29/08/2017	0	0	0
2014299928	677	rural/clinic	No	29/08/2017	0	0	0
2014314743	677	rural/clinic	No	29/08/2017	0	0	0

2014314740	677	rural/clinic	No	29/08/2017	0	0	0
2014299927	677	rural/clinic	No	29/08/2017	0	0	0
2011133160	677	rural/clinic	No	29/08/2017	0	0	0
2014314742	677	rural/clinic	No	29/08/2017	0	0	0
2014299930	677	rural/clinic	No	29/08/2017	0	0	0
2014314741	677	rural/clinic	No	29/08/2017	0	0	0
2014314745	677	rural/clinic	No	29/08/2017	0	0	0
2011133163	677	rural/clinic	No	29/08/2017	0	0	0
2011123783	677	rural/clinic	No	29/08/2017	0	0	0
2015324049	677	rural/clinic	No	29/08/2017	0	0	0
2015324489	677	rural/clinic	No	29/08/2017	0	0	0
2015337381	677	rural/clinic	No	29/08/2017	0	0	0
2015315009	677	rural/clinic	No	29/08/2017	0	0	0
2015315008	677	rural/clinic	No	29/08/2017	0	0	0
2015385870	677	rural/clinic	No	29/08/2017	0	0	0
2014314744	677	rural/clinic	No	29/08/2017	0	0	0
2015337536	677	rural/clinic	No	29/08/2017	0	0	0
2015368745	677	rural/clinic	No	29/08/2017	0	0	0
2015324048	677	rural/clinic	No	29/08/2017	0	0	0
2012368548	677	rural/clinic	No	29/08/2017	0	0	0
2015314618	677	rural/clinic	No	29/08/2017	0	0	0
2015340916	677	rural/clinic	No	29/08/2017	0	0	0
2014319214	677	rural/clinic	No	29/08/2017	0	0	0
2015290216	677	rural/clinic	No	29/08/2017	0	0	0
2014349927	677	rural/clinic	No	29/08/2017	0	0	0
2015290215	677	rural/clinic	No	29/08/2017	0	0	0
2015340905	677	rural/clinic	No	29/08/2017	0	0	0
2015349021	677	rural/clinic	No	29/08/2017	0	0	0
2015325233	677	rural/clinic	No	29/08/2017	0	0	0
2015325234	677	rural/clinic	No	29/08/2017	0	0	0
2015337537	677	rural/clinic	No	30/08/2017	0	0	0
2011135688	643	rural/clinic	No	29/08/2017	0	0	0
2011133162	643	rural/clinic	No	29/08/2017	0	0	0
2011135689	643	rural/clinic	No	29/08/2017	0	0	0
2015344669	643	rural/clinic	No	29/08/2017	0	0	0
2015344670	643	rural/clinic	No	29/08/2017	0	0	0
2015368682	643	rural/clinic	No	29/08/2017	0	0	0
2015368683	643	rural/clinic	No	29/08/2017	0	0	0
2015368684	643	rural/clinic	No	29/08/2017	0	0	0
2015344671	643	rural/clinic	No	29/08/2017	0	0	0
2015344480	643	rural/clinic	No	29/08/2017	0	0	0
2015344481	643	rural/clinic	No	29/08/2017	0	0	0
2011135690	643	rural/clinic	No	29/08/2017	0	0	0
2014382711	643	rural/clinic	No	29/08/2017	0	0	0
2014382712	643	rural/clinic	No	29/08/2017	0	0	0
2015334811	643	rural/clinic	No	29/08/2017	0	0	0
2015334812	643	rural/clinic	No	29/08/2017	0	0	0
2015334813	643	rural/clinic	No	29/08/2017	0	0	0
2015289233	643	rural/clinic	No	24/08/2017	0	0	0
2015289233	643	rural/clinic	No	24/08/2017	0	0	0
2015403318	643	rural/clinic	No	24/08/2017	0	0	0
2015403318	643	rural/clinic	No	24/08/2017	0	0	0
2015402665	643	rural/clinic	No	24/08/2017	0	0	0
2015402665	643	rural/clinic	No	24/08/2017	0	0	0
2015336661	643	rural/clinic	No	24/08/2017	0	0	0
2015336661	643	rural/clinic	No	24/08/2017	0	0	0
2012291388	643	rural/clinic	No	24/08/2017	0	0	0
2012291388	643	rural/clinic	No	24/08/2017	0	0	0
2011135075	643	rural/clinic	No	24/08/2017	0	0	0
2011135075	643	rural/clinic	No	24/08/2017	0	0	0
2015368899	643	rural/clinic	No	24/08/2017	0	0	0
2015368899	643	rural/clinic	No	24/08/2017	0	0	0
2012315334	435	district/faith-based	No	29/08/2017	1	0	0
2014332731	435	district/faith-based	No	29/08/2017	1	0	0
2012330893	435	district/faith-based	No	29/08/2017	1	0	0
2015313003	435	district/faith-based	No	29/08/2017	1	0	0
2015313004	435	district/faith-based	No	29/08/2017	1	0	0
2014332726	435	district/faith-based	No	29/08/2017	1	0	0
2015289195	435	district/faith-based	No	29/08/2017	1	0	0
2014332730	435	district/faith-based	No	29/08/2017	1	0	0
2015355929	435	district/faith-based	No	29/08/2017	1	0	0
2014332728	435	district/faith-based	No	29/08/2017	1	0	0
2015406347	435	district/faith-based	No	29/08/2017	1	0	0
2015406346	435	district/faith-based	No	29/08/2017	1	0	0
2014332729	435	district/faith-based	No	29/08/2017	1	0	0
2015406350	435	district/faith-based	No	29/08/2017	1	0	0
2012322042	435	district/faith-based	No	29/08/2017	1	0	0
2015406348	435	district/faith-based	No	29/08/2017	1	0	0
2012379910	435	district/faith-based	No	29/08/2017	1	0	0
2012359172	435	district/faith-based	No	29/08/2017	1	0	0
2015352801	435	district/faith-based	No	29/08/2017	1	0	0
2015352802	435	district/faith-based	No	29/08/2017	1	0	0

2015352803	435	district/faith-based	No	29/08/2017	1	0	0
2015352804	435	district/faith-based	No	29/08/2017	1	0	0
2015407074	435	district/faith-based	No	29/08/2017	1	0	0
2015407075	435	district/faith-based	No	29/08/2017	1	0	0
2015373893	435	district/faith-based	No	25/08/2017	1	0	0
2015373893	435	district/faith-based	No	25/08/2017	1	0	0
2012390652	435	district/faith-based	No	24/08/2017	1	0	0
2012390652	435	district/faith-based	No	24/08/2017	1	0	0
2015368622	435	district/faith-based	No	29/08/2017	1	0	0
2015368622	435	district/faith-based	No	29/08/2017	1	0	0
2015358319	435	district/faith-based	No	24/08/2017	1	0	0
2015358319	435	district/faith-based	No	24/08/2017	1	0	0
2015401166	435	district/faith-based	No	24/08/2017	1	0	0
2015401166	435	district/faith-based	No	24/08/2017	1	0	0
2015373892	435	district/faith-based	No	24/08/2017	1	0	0
2015373892	435	district/faith-based	No	24/08/2017	1	0	0
2011135073	435	district/faith-based	No	24/08/2017	1	0	0
2011135073	435	district/faith-based	No	24/08/2017	1	0	0
2014375540	435	district/faith-based	No	29/08/2017	1	0	0
2015368829	435	district/faith-based	No	29/08/2017	1	0	0
2015368829	435	district/faith-based	No	29/08/2017	1	0	0
2015414281	435	district/faith-based	No	29/08/2017	1	0	0
2015414281	435	district/faith-based	No	29/08/2017	1	0	0
2013267251	435	district/faith-based	No	29/08/2017	1	0	0
2013267251	435	district/faith-based	No	29/08/2017	1	0	0
2015305830	435	district/faith-based	No	31/08/2017	1	0	0
2015305831	435	district/faith-based	No	31/08/2017	1	0	0
2015305832	435	district/faith-based	No	31/08/2017	1	0	0
2015302154	435	district/faith-based	No	31/08/2017	1	0	0
2014327045	435	district/faith-based	No	31/08/2017	1	0	0
2015349543	435	district/faith-based	No	31/08/2017	1	0	0
2015349545	435	district/faith-based	No	31/08/2017	1	0	0
2015401172	435	district/faith-based	No	31/08/2017	1	0	0
2014327046	435	district/faith-based	No	31/08/2017	1	0	0
2015403711	435	district/faith-based	No	31/08/2017	1	0	0
2014345751	435	district/faith-based	No	31/08/2017	1	0	0
2015403712	435	district/faith-based	No	31/08/2017	1	0	0
2015369100	435	district/faith-based	No	31/08/2017	1	0	0
2015403713	435	district/faith-based	No	31/08/2017	1	0	0
2012381663	435	district/faith-based	No	31/08/2017	1	0	0
2015403714	435	district/faith-based	No	31/08/2017	1	0	0
2015403715	435	district/faith-based	No	31/08/2017	1	0	0
2015355153	435	district/faith-based	No	31/08/2017	1	0	0
2015403716	435	district/faith-based	No	31/08/2017	1	0	0
2015403717	435	district/faith-based	No	31/08/2017	1	0	0
2015355154	435	district/faith-based	No	31/08/2017	1	0	0
2015403718	435	district/faith-based	No	31/08/2017	1	0	0
2015355155	435	district/faith-based	No	31/08/2017	1	0	0
2015403719	435	district/faith-based	No	31/08/2017	1	0	0
2015403720	435	district/faith-based	No	31/08/2017	1	0	0
2015403721	435	district/faith-based	No	31/08/2017	1	0	0
2015403723	435	district/faith-based	No	31/08/2017	1	0	0
2015403724	435	district/faith-based	No	31/08/2017	1	0	0
2015403725	435	district/faith-based	No	31/08/2017	1	0	0
2015364343	435	district/faith-based	No	31/08/2017	1	0	0
2015403726	435	district/faith-based	No	31/08/2017	1	0	0
2015403727	435	district/faith-based	No	31/08/2017	1	0	0
2015364344	435	district/faith-based	No	31/08/2017	1	0	0
2015403728	435	district/faith-based	No	31/08/2017	1	0	0
2015364345	435	district/faith-based	No	31/08/2017	1	0	0
2015403729	435	district/faith-based	No	31/08/2017	1	0	0
2015364346	435	district/faith-based	No	31/08/2017	1	0	0
2015403730	435	district/faith-based	No	31/08/2017	1	0	0
2015364347	435	district/faith-based	No	31/08/2017	1	0	0
2015403731	435	district/faith-based	No	31/08/2017	1	0	0
2011226493	435	district/faith-based	No	31/08/2017	1	0	0
2015377333	435	district/faith-based	No	31/08/2017	1	0	0
2015403732	435	district/faith-based	No	31/08/2017	1	0	0
2015362067	435	district/faith-based	No	31/08/2017	1	0	0
2015373897	435	district/faith-based	No	31/08/2017	1	0	0
2015362005	435	district/faith-based	No	31/08/2017	1	0	0
2015362004	435	district/faith-based	No	31/08/2017	1	0	0
2015362007	435	district/faith-based	No	31/08/2017	1	0	0
2015373898	435	district/faith-based	No	31/08/2017	1	0	0
2012390657	435	district/faith-based	No	31/08/2017	1	0	0
2015385550	435	district/faith-based	No	31/08/2017	1	0	0
2015362006	435	district/faith-based	No	31/08/2017	1	0	0
2012390658	435	district/faith-based	No	31/08/2017	1	0	0
2014300542	435	district/faith-based	No	31/08/2017	1	0	0
2015377331	435	district/faith-based	No	31/08/2017	1	0	0
2012390659	435	district/faith-based	No	31/08/2017	1	0	0
2015364338	435	district/faith-based	No	31/08/2017	1	0	0

2012369979	435	district/faith-based	No	31/08/2017	1	0	0
2015376669	435	district/faith-based	No	31/08/2017	1	0	0
2015408690	435	district/faith-based	No	31/08/2017	1	0	0
2012369980	435	district/faith-based	No	31/08/2017	1	0	0
2015376670	435	district/faith-based	No	31/08/2017	1	0	0
2012324252	435	district/faith-based	No	31/08/2017	1	0	0
2012324253	435	district/faith-based	No	31/08/2017	1	0	0
2012324254	435	district/faith-based	No	31/08/2017	1	0	0
2015376671	435	district/faith-based	No	31/08/2017	1	0	0
2012363989	435	district/faith-based	No	31/08/2017	1	0	0
2012369978	435	district/faith-based	No	31/08/2017	1	0	0
2012275230	435	district/faith-based	No	31/08/2017	1	0	0
2015390323	435	district/faith-based	No	31/08/2017	1	0	0
2012275231	435	district/faith-based	No	31/08/2017	1	0	0
2015390324	435	district/faith-based	No	31/08/2017	1	0	0
2015363309	435	district/faith-based	No	31/08/2017	1	0	0
2012346601	435	district/faith-based	No	31/08/2017	1	0	0
2014363051	435	district/faith-based	No	31/08/2017	1	0	0
2015362833	435	district/faith-based	No	31/08/2017	1	0	0
2014363053	435	district/faith-based	No	31/08/2017	1	0	0
2015362834	435	district/faith-based	No	31/08/2017	1	0	0
2014306853	435	district/faith-based	No	31/08/2017	1	0	0
2014363052	435	district/faith-based	No	31/08/2017	1	0	0
2015415919	435	district/faith-based	No	31/08/2017	1	0	0
2015321765	435	district/faith-based	No	31/08/2017	1	0	0
2015415296	701	rural/clinic	No	31/08/2017	0	0	0
2015332980	701	rural/clinic	No	31/08/2017	0	0	0
2014317524	701	rural/clinic	No	31/08/2017	0	0	0
2013258731	701	rural/clinic	No	31/08/2017	0	0	0
2014317522	701	rural/clinic	No	31/08/2017	0	0	0
2014372279	701	rural/clinic	No	31/08/2017	0	0	0
2014306854	701	rural/clinic	No	31/08/2017	0	0	0
2014317518	701	rural/clinic	No	31/08/2017	0	0	0
2015332977	701	rural/clinic	No	31/08/2017	0	0	0
2014348000	701	rural/clinic	No	31/08/2017	0	0	0
2014317521	701	rural/clinic	No	31/08/2017	0	0	0
2015332978	701	rural/clinic	No	31/08/2017	0	0	0
2015362913	701	rural/clinic	No	31/08/2017	0	0	0
2015332979	701	rural/clinic	No	31/08/2017	0	0	0
2015382613	735	rural/clinic	No	31/08/2017	0	0	0
2015382614	735	rural/clinic	No	31/08/2017	0	0	0
2015333457	735	rural/clinic	No	31/08/2017	0	0	0
2014301362	735	rural/clinic	No	31/08/2017	0	0	0
2015382615	735	rural/clinic	No	31/08/2017	0	0	0
2015333456	735	rural/clinic	No	31/08/2017	0	0	0
2012336638	735	rural/clinic	No	31/08/2017	0	0	0
2015367715	735	rural/clinic	No	31/08/2017	0	0	0
2012336637	735	rural/clinic	No	31/08/2017	0	0	0
2011117863	735	rural/clinic	No	31/08/2017	0	0	0
2011117861	735	rural/clinic	No	31/08/2017	0	0	0
2015297176	735	rural/clinic	No	31/08/2017	0	0	0
2011117862	735	rural/clinic	No	31/08/2017	0	0	0
2015297178	735	rural/clinic	No	31/08/2017	0	0	0
2011117866	735	rural/clinic	No	31/08/2017	0	0	0
2015362916	735	rural/clinic	No	31/08/2017	0	0	0
2015297177	735	rural/clinic	No	31/08/2017	0	0	0
2015362917	735	rural/clinic	No	31/08/2017	0	0	0
2011117867	735	rural/clinic	No	31/08/2017	0	0	0
2015362921	735	rural/clinic	No	31/08/2017	0	0	0
2015362922	735	rural/clinic	No	31/08/2017	0	0	0
2011117864	735	rural/clinic	No	31/08/2017	0	0	0
2015369788	735	rural/clinic	No	31/08/2017	0	0	0
2011117865	735	rural/clinic	No	31/08/2017	0	0	0
2015386822	735	rural/clinic	No	31/08/2017	0	0	0
2015386821	735	rural/clinic	No	31/08/2017	0	0	0
2011117868	735	rural/clinic	No	31/08/2017	0	0	0
2015386819	735	rural/clinic	No	31/08/2017	0	0	0
2011117869	735	rural/clinic	No	31/08/2017	0	0	0
2014314237	735	rural/clinic	No	31/08/2017	0	0	0
2014314238	735	rural/clinic	No	31/08/2017	0	0	0
2015390454	735	rural/clinic	No	31/08/2017	0	0	0
2015406402	735	rural/clinic	No	31/08/2017	0	0	0
2012317572	735	rural/clinic	No	31/08/2017	0	0	0
2015390453	735	rural/clinic	No	31/08/2017	0	0	0
2014300354	735	rural/clinic	No	31/08/2017	0	0	0
2012379913	735	rural/clinic	No	31/08/2017	0	0	0
2014300355	735	rural/clinic	No	31/08/2017	0	0	0
2015369030	553	rural/clinic	No	31/08/2017	0	0	0
2014370314	553	rural/clinic	No	05/09/2017	0	0	0
2015406047	553	rural/clinic	No	05/09/2017	0	0	0
2015406047	553	rural/clinic	No	05/09/2017	0	0	0
2015372946	553	rural/clinic	No	05/09/2017	0	0	0

2015372946	590	rural/clinic	No	05/09/2017	0	0	0
2012263923	590	rural/clinic	No	05/09/2017	0	0	0
2012263923	590	rural/clinic	No	05/09/2017	0	0	0
2015362436	590	rural/clinic	No	05/09/2017	0	0	0
2015362436	590	rural/clinic	No	05/09/2017	0	0	0
2011128290	590	rural/clinic	No	05/09/2017	0	0	0
2011128290	590	rural/clinic	No	05/09/2017	0	0	0
2011199247	590	rural/clinic	No	07/09/2017	0	0	0
2014345752	590	rural/clinic	No	07/09/2017	0	0	0
2012269024	590	rural/clinic	No	07/09/2017	0	0	0
2012260091	590	rural/clinic	No	07/09/2017	0	0	0
2015403321	590	rural/clinic	No	07/09/2017	0	0	0
2012269023	590	rural/clinic	No	07/09/2017	0	0	0
2015331745	590	rural/clinic	No	07/09/2017	0	0	0
2015287401	590	rural/clinic	No	07/09/2017	0	0	0
2015331744	590	rural/clinic	No	07/09/2017	0	0	0
2015331747	590	rural/clinic	No	07/09/2017	0	0	0
2012379916	590	rural/clinic	No	07/09/2017	0	0	0
2015331746	590	rural/clinic	No	07/09/2017	0	0	0
201075904	590	rural/clinic	No	07/09/2017	0	0	0
2015403320	590	rural/clinic	No	07/09/2017	0	0	0
2012379350	590	rural/clinic	No	07/09/2017	0	0	0
201075905	590	rural/clinic	No	07/09/2017	0	0	0
2014296602	590	rural/clinic	No	07/09/2017	0	0	0
201075906	590	rural/clinic	No	07/09/2017	0	0	0
201075907	590	rural/clinic	No	07/09/2017	0	0	0
2011144034	590	rural/clinic	No	07/09/2017	0	0	0
201075908	590	rural/clinic	No	07/09/2017	0	0	0
2015339031	590	rural/clinic	No	07/09/2017	0	0	0
2015287405	590	rural/clinic	No	07/09/2017	0	0	0
2014298046	590	rural/clinic	No	07/09/2017	0	0	0
2014298045	590	rural/clinic	No	07/09/2017	0	0	0
2015287404	590	rural/clinic	No	07/09/2017	0	0	0
2015403324	590	rural/clinic	No	07/09/2017	0	0	0
2015289221	590	rural/clinic	No	24/08/2017	0	0	0
2015287403	590	rural/clinic	No	07/09/2017	0	0	0
2015378186	590	rural/clinic	No	07/09/2017	0	0	0
2014301894	590	rural/clinic	No	07/09/2017	0	0	0
2015378184	590	rural/clinic	No	07/09/2017	0	0	0
2015287402	590	rural/clinic	No	07/09/2017	0	0	0
2014301895	590	rural/clinic	No	07/09/2017	0	0	0
2013255747	590	rural/clinic	No	07/09/2017	0	0	0
2015384368	590	rural/clinic	No	07/09/2017	0	0	0
2011144115	590	rural/clinic	No	07/09/2017	0	0	0
2015378187	590	rural/clinic	No	07/09/2017	0	0	0
2011144116	590	rural/clinic	No	07/09/2017	0	0	0
2011137329	590	rural/clinic	No	07/09/2017	0	0	0
2015378188	590	rural/clinic	No	07/09/2017	0	0	0
2014363103	590	rural/clinic	No	07/09/2017	0	0	0
2015378189	590	rural/clinic	No	07/09/2017	0	0	0
2014354764	590	rural/clinic	No	07/09/2017	0	0	0
2015405806	590	rural/clinic	No	07/09/2017	0	0	0
2014385621	590	rural/clinic	No	07/09/2017	0	0	0
2015378190	590	rural/clinic	No	07/09/2017	0	0	0
2015377442	590	rural/clinic	No	07/09/2017	0	0	0
2015377443	590	rural/clinic	No	07/09/2017	0	0	0
2015378191	590	rural/clinic	No	07/09/2017	0	0	0
2011115101	590	rural/clinic	No	07/09/2017	0	0	0
2015377444	590	rural/clinic	No	07/09/2017	0	0	0
2015378193	590	rural/clinic	No	07/09/2017	0	0	0
2015406405	590	rural/clinic	No	07/09/2017	0	0	0
2012265927	590	rural/clinic	No	07/09/2017	0	0	0
2015406408	590	rural/clinic	No	07/09/2017	0	0	0
2015378194	590	rural/clinic	No	07/09/2017	0	0	0
2015321459	590	rural/clinic	No	07/09/2017	0	0	0
2015406406	590	rural/clinic	No	07/09/2017	0	0	0
2015362758	590	rural/clinic	No	07/09/2017	0	0	0
2015363765	590	rural/clinic	No	07/09/2017	0	0	0
2015406339	590	rural/clinic	No	10/08/2017	0	0	0
2015362760	590	rural/clinic	No	07/09/2017	0	0	0
2015321458	590	rural/clinic	No	07/09/2017	0	0	0
2015406341	590	rural/clinic	No	07/09/2017	0	0	0
2015367236	590	rural/clinic	No	07/09/2017	0	0	0
2015406344	590	rural/clinic	No	07/09/2017	0	0	0
2014365644	590	rural/clinic	No	07/09/2017	0	0	0
2015406345	590	rural/clinic	No	07/09/2017	0	0	0
2014337227	590	rural/clinic	No	07/09/2017	0	0	0
2011224439	590	rural/clinic	No	07/09/2017	0	0	0
2014337225	590	rural/clinic	No	07/09/2017	0	0	0
2015302109	590	rural/clinic	No	07/09/2017	0	0	0
2014337226	590	rural/clinic	No	07/09/2017	0	0	0
2015302107	590	rural/clinic	No	07/09/2017	0	0	0

2011229224	372	district/faith-based	No	07/09/2017	1	0	0
2014365077	372	district/faith-based	No	07/09/2017	1	0	0
2015410456	372	district/faith-based	No	07/09/2017	1	0	0
2014336828	372	district/faith-based	No	07/09/2017	1	0	0
2015410457	372	district/faith-based	No	07/09/2017	1	0	0
2015405631	372	district/faith-based	No	07/09/2017	1	0	0
2015419080	372	district/faith-based	No	07/09/2017	1	0	0
2015362759	372	district/faith-based	No	07/09/2017	1	0	0
2015419078	372	district/faith-based	No	07/09/2017	1	0	0
2015367237	372	district/faith-based	No	07/09/2017	1	0	0
2014287186	372	district/faith-based	No	07/09/2017	1	0	0
2015419079	372	district/faith-based	No	07/09/2017	1	0	0
2015405636	372	district/faith-based	No	07/09/2017	1	0	0
2015382760	372	district/faith-based	No	07/09/2017	1	0	0
2015352004	372	district/faith-based	No	07/09/2017	1	0	0
2015382761	372	district/faith-based	No	07/09/2017	1	0	0
2015405635	372	district/faith-based	No	07/09/2017	1	0	0
2015382762	372	district/faith-based	No	07/09/2017	1	0	0
2015364543	372	district/faith-based	No	07/09/2017	1	0	0
2015405673	372	district/faith-based	No	07/09/2017	1	0	0
2015338896	372	district/faith-based	No	07/09/2017	1	0	0
2014321432	372	district/faith-based	No	07/09/2017	1	0	0
2013261942	372	district/faith-based	No	07/09/2017	1	0	0
2015338897	372	district/faith-based	No	07/09/2017	1	0	0
2015405672	372	district/faith-based	No	07/09/2017	1	0	0
2015338898	372	district/faith-based	No	07/09/2017	1	0	0
2014336827	372	district/faith-based	No	07/09/2017	1	0	0
2015338899	372	district/faith-based	No	07/09/2017	1	0	0
2014340202	372	district/faith-based	No	07/09/2017	1	0	0
2015405671	372	district/faith-based	No	07/09/2017	1	0	0
2014340210	372	district/faith-based	No	07/09/2017	1	0	0
2015363764	372	district/faith-based	No	07/09/2017	1	0	0
2014340211	372	district/faith-based	No	07/09/2017	1	0	0
2011114848	372	district/faith-based	No	07/09/2017	1	0	0
2014298246	372	district/faith-based	No	07/09/2017	1	0	0
2014336943	372	district/faith-based	No	07/09/2017	1	0	0
2014340209	372	district/faith-based	No	07/09/2017	1	0	0
2015321452	372	district/faith-based	No	07/09/2017	1	0	0
2014340208	372	district/faith-based	No	07/09/2017	1	0	0
2015405633	372	district/faith-based	No	07/09/2017	1	0	0
2015390455	372	district/faith-based	No	07/09/2017	1	0	0
2013261939	372	district/faith-based	No	07/09/2017	1	0	0
2015390456	372	district/faith-based	No	07/09/2017	1	0	0
2015402686	372	district/faith-based	No	07/09/2017	1	0	0
2014365642	372	district/faith-based	No	07/09/2017	1	0	0
2014321431	372	district/faith-based	No	07/09/2017	1	0	0
2014321430	372	district/faith-based	No	07/09/2017	1	0	0
2015402687	372	district/faith-based	No	07/09/2017	1	0	0
2015367231	372	district/faith-based	No	07/09/2017	1	0	0
2014321433	372	district/faith-based	No	07/09/2017	1	0	0
2015405634	372	district/faith-based	No	07/09/2017	1	0	0
2015402688	372	district/faith-based	No	07/09/2017	1	0	0
2014321434	372	district/faith-based	No	07/09/2017	1	0	0
2015402689	372	district/faith-based	No	07/09/2017	1	0	0
2015321453	372	district/faith-based	No	07/09/2017	1	0	0
2015364544	372	district/faith-based	No	07/09/2017	1	0	0
2015321454	372	district/faith-based	No	07/09/2017	1	0	0
2015402690	372	district/faith-based	No	07/09/2017	1	0	0
2015364542	372	district/faith-based	No	07/09/2017	1	0	0
2015367233	373	rural/clinic	No	07/09/2017	0	0	0
2014287184	373	rural/clinic	No	07/09/2017	0	0	0
2015358351	373	rural/clinic	No	07/09/2017	0	0	0
2014287185	373	rural/clinic	No	07/09/2017	0	0	0
2015367235	373	rural/clinic	No	07/09/2017	0	0	0
2015358327	373	rural/clinic	No	07/09/2017	0	0	0
2015352005	373	rural/clinic	No	07/09/2017	0	0	0
2015358325	373	rural/clinic	No	07/09/2017	0	0	0
2015334815	373	rural/clinic	No	07/09/2017	0	0	0
2015367234	373	rural/clinic	No	07/09/2017	0	0	0
2015334816	373	rural/clinic	No	07/09/2017	0	0	0
2015358326	373	rural/clinic	No	07/09/2017	0	0	0
2015321456	373	rural/clinic	No	07/09/2017	0	0	0
2015406050	373	rural/clinic	No	07/09/2017	0	0	0
2015406049	373	rural/clinic	No	07/09/2017	0	0	0
2015358329	373	rural/clinic	No	07/09/2017	0	0	0
2015321455	373	rural/clinic	No	07/09/2017	0	0	0
2015406048	373	rural/clinic	No	07/09/2017	0	0	0
2015358328	373	rural/clinic	No	07/09/2017	0	0	0
2013261940	373	rural/clinic	No	07/09/2017	0	0	0
2015406056	373	rural/clinic	No	07/09/2017	0	0	0
201075901	311	rural/clinic	No	07/09/2017	0	0	0
2011155243	311	rural/clinic	No	07/09/2017	0	0	0

2015361193	311	rural/clinic	No	07/09/2017	0	0	0
201075902	311	rural/clinic	No	07/09/2017	0	0	0
2015361195	311	rural/clinic	No	07/09/2017	0	0	0
2015361197	311	rural/clinic	No	07/09/2017	0	0	0
2015334814	311	rural/clinic	No	07/09/2017	0	0	0
2015361196	311	rural/clinic	No	07/09/2017	0	0	0
2014312205	311	rural/clinic	No	07/09/2017	0	0	0
2015294628	311	rural/clinic	No	07/09/2017	0	0	0
2014382713	678	rural/clinic	No	07/09/2017	0	0	0
2012390667	678	rural/clinic	No	07/09/2017	0	0	0
2012390666	678	rural/clinic	No	07/09/2017	0	0	0
2015344482	678	rural/clinic	No	07/09/2017	0	0	0
2012390665	678	rural/clinic	No	07/09/2017	0	0	0
2015344483	678	rural/clinic	No	07/09/2017	0	0	0
2012390664	678	rural/clinic	No	07/09/2017	0	0	0
2015344747	678	rural/clinic	No	07/09/2017	0	0	0
2012390662	678	rural/clinic	No	07/09/2017	0	0	0
2011136261	678	rural/clinic	No	07/09/2017	0	0	0
2012390663	678	rural/clinic	No	07/09/2017	0	0	0
2012390660	678	rural/clinic	No	07/09/2017	0	0	0
2015344672	678	rural/clinic	No	07/09/2017	0	0	0
2012390661	678	rural/clinic	No	07/09/2017	0	0	0
2015289238	678	rural/clinic	No	07/09/2017	0	0	0
2011137325	678	rural/clinic	No	07/09/2017	0	0	0
2015289239	678	rural/clinic	No	07/09/2017	0	0	0
2015289240	678	rural/clinic	No	07/09/2017	0	0	0
2015289241	678	rural/clinic	No	07/09/2017	0	0	0
2015289242	678	rural/clinic	No	07/09/2017	0	0	0
2015289243	678	rural/clinic	No	07/09/2017	0	0	0
2015289244	678	rural/clinic	No	07/09/2017	0	0	0
2015289237	678	rural/clinic	No	07/09/2017	0	0	0
2014365374	678	rural/clinic	No	07/09/2017	0	0	0
2014350639	678	rural/clinic	No	07/09/2017	0	0	0
2011152232	678	rural/clinic	No	07/09/2017	0	0	0
2014308898	678	rural/clinic	No	07/09/2017	0	0	0
2011137326	678	rural/clinic	No	07/09/2017	0	0	0
2011137327	678	rural/clinic	No	07/09/2017	0	0	0
2014365478	678	rural/clinic	No	07/09/2017	0	0	0
2015378185	678	rural/clinic	No	07/09/2017	0	0	0
2011137328	678	rural/clinic	No	07/09/2017	0	0	0
2015336155	678	rural/clinic	No	07/09/2017	0	0	0
2015404683	678	rural/clinic	No	07/09/2017	0	0	0
2015404675	678	rural/clinic	No	07/09/2017	0	0	0
2015402033	678	rural/clinic	No	07/09/2017	0	0	0
2015360866	678	rural/clinic	No	07/09/2017	0	0	0
2015406052	678	rural/clinic	No	07/09/2017	0	0	0
2015406051	678	rural/clinic	No	07/09/2017	0	0	0
2015412653	678	rural/clinic	No	07/09/2017	0	0	0
2015367719	678	rural/clinic	No	07/09/2017	0	0	0
2015367717	678	rural/clinic	No	07/09/2017	0	0	0
2015367718	678	rural/clinic	No	07/09/2017	0	0	0
2014365643	678	rural/clinic	No	07/09/2017	0	0	0
2015321460	678	rural/clinic	No	07/09/2017	0	0	0
2014301253	678	rural/clinic	No	07/09/2017	0	0	0
2012312168	100	rural/clinic	No	07/09/2017	0	0	0
2015340755	100	rural/clinic	No	07/09/2017	0	0	0
2014288017	100	rural/clinic	No	07/09/2017	0	0	0
2011199250	100	rural/clinic	No	07/09/2017	0	0	0
2015321457	100	rural/clinic	No	07/09/2017	0	0	0
2011168526	100	rural/clinic	No	07/09/2017	0	0	0
2012312163	100	rural/clinic	No	07/09/2017	0	0	0
2014338564	100	rural/clinic	No	07/09/2017	0	0	0
2015378192	100	rural/clinic	No	07/09/2017	0	0	0
2012284577	100	rural/clinic	No	07/09/2017	0	0	0
2011133922	100	rural/clinic	No	07/09/2017	0	0	0
2015373899	100	rural/clinic	No	05/09/2017	0	0	0
2015373899	100	rural/clinic	No	05/09/2017	0	0	0
2015287401	100	rural/clinic	No	07/09/2017	0	0	0
2015287401	100	rural/clinic	No	07/09/2017	0	0	0
2015331745	409	rural/clinic	No	07/09/2017	0	0	0
2015287405	409	rural/clinic	No	07/09/2017	0	0	0
2015287405	409	rural/clinic	No	07/09/2017	0	0	0
2015287402	409	rural/clinic	No	07/09/2017	0	0	0
2015287402	409	rural/clinic	No	07/09/2017	0	0	0
2015377442	409	rural/clinic	No	07/09/2017	0	0	0
2015377442	409	rural/clinic	No	07/09/2017	0	0	0
2015419079	409	rural/clinic	No	07/09/2017	0	0	0
2015419079	409	rural/clinic	No	07/09/2017	0	0	0
2015382760	409	rural/clinic	No	07/09/2017	0	0	0
2015382760	409	rural/clinic	No	07/09/2017	0	0	0
2015382761	128	rural/clinic	No	07/09/2017	0	0	0
2015382761	128	rural/clinic	No	07/09/2017	0	0	0

2015382762	128	rural/clinic	No	07/09/2017	0	0	0
2015382762	128	rural/clinic	No	07/09/2017	0	0	0
2014298246	128	rural/clinic	No	07/09/2017	0	0	0
2014298246	128	rural/clinic	No	07/09/2017	0	0	0
2014321430	128	rural/clinic	No	07/09/2017	0	0	0
2014321430	128	rural/clinic	No	07/09/2017	0	0	0
2014321433	128	rural/clinic	No	07/09/2017	0	0	0
2014321433	128	rural/clinic	No	07/09/2017	0	0	0
2015378195	128	rural/clinic	No	07/09/2017	0	0	0
2015405672	128	rural/clinic	No	07/09/2017	0	0	0
2015405672	128	rural/clinic	No	07/09/2017	0	0	0
2015363764	128	rural/clinic	No	07/09/2017	0	0	0
2015363764	128	rural/clinic	No	07/09/2017	0	0	0
2015405633	702	rural/clinic	No	07/09/2017	0	0	0
2015367235	702	rural/clinic	No	07/09/2017	0	0	0
2015367235	702	rural/clinic	No	07/09/2017	0	0	0
2014300432	702	rural/clinic	No	07/09/2017	0	0	0
2015305835	702	rural/clinic	No	07/09/2017	0	0	0
2015305836	702	rural/clinic	No	07/09/2017	0	0	0
2015386823	702	rural/clinic	No	07/09/2017	0	0	0
2015305837	702	rural/clinic	No	07/09/2017	0	0	0
2015332370	702	rural/clinic	No	07/09/2017	0	0	0
2015360545	702	rural/clinic	No	07/09/2017	0	0	0
2013258611	702	rural/clinic	No	07/09/2017	0	0	0
2015332756	702	rural/clinic	No	07/09/2017	0	0	0
2015332755	702	rural/clinic	No	07/09/2017	0	0	0
2015360542	702	rural/clinic	No	07/09/2017	0	0	0
2015332753	702	rural/clinic	No	07/09/2017	0	0	0
2015418378	702	rural/clinic	No	07/09/2017	0	0	0
2015332378	702	rural/clinic	No	07/09/2017	0	0	0
2015332369	702	rural/clinic	No	07/09/2017	0	0	0
2015418379	702	rural/clinic	No	07/09/2017	0	0	0
2011204486	702	rural/clinic	No	07/09/2017	0	0	0
2015360541	702	rural/clinic	No	07/09/2017	0	0	0
2015332485	702	rural/clinic	No	07/09/2017	0	0	0
2015332486	702	rural/clinic	No	07/09/2017	0	0	0
2015360544	702	rural/clinic	No	07/09/2017	0	0	0
2015286403	702	rural/clinic	No	07/09/2017	0	0	0
2015286401	702	rural/clinic	No	07/09/2017	0	0	0
2014338563	702	rural/clinic	No	07/09/2017	0	0	0
2015286406	702	rural/clinic	No	07/09/2017	0	0	0
2015382620	702	rural/clinic	No	07/09/2017	0	0	0
2015382619	702	rural/clinic	No	02/09/2017	0	0	0
2015382618	702	rural/clinic	No	07/09/2017	0	0	0
2015382617	702	rural/clinic	No	07/09/2017	0	0	0
2010099943	702	rural/clinic	No	07/09/2017	0	0	0
2015340726	702	rural/clinic	No	07/09/2017	0	0	0
2010099942	702	rural/clinic	No	07/09/2017	0	0	0
2015382624	702	rural/clinic	No	07/09/2017	0	0	0
2015340240	702	rural/clinic	No	07/09/2017	0	0	0
2015382616	702	rural/clinic	No	07/09/2017	0	0	0
2015340239	702	rural/clinic	No	07/09/2017	0	0	0
2015382622	702	rural/clinic	No	07/09/2017	0	0	0
2010099941	702	rural/clinic	No	07/09/2017	0	0	0
2015382623	702	rural/clinic	No	07/09/2017	0	0	0
2014320532	166	rural/clinic	No	07/09/2017	0	0	0
2015382621	166	rural/clinic	No	07/09/2017	0	0	0
2015404677	166	rural/clinic	No	07/09/2017	0	0	0
2015404678	166	rural/clinic	No	07/09/2017	0	0	0
2015404679	166	rural/clinic	No	07/09/2017	0	0	0
2015404680	166	rural/clinic	No	07/09/2017	0	0	0
2015404676	166	rural/clinic	No	07/09/2017	0	0	0
2014320531	166	rural/clinic	No	07/09/2017	0	0	0
2015404682	166	rural/clinic	No	07/09/2017	0	0	0
2015302304	166	rural/clinic	No	07/09/2017	0	0	0
2015340727	166	rural/clinic	No	07/09/2017	0	0	0
2015302305	166	rural/clinic	No	07/09/2017	0	0	0
2015402032	166	rural/clinic	No	07/09/2017	0	0	0
2014327091	166	rural/clinic	No	07/09/2017	0	0	0
2015402031	166	rural/clinic	No	07/09/2017	0	0	0
2011199249	166	rural/clinic	No	07/09/2017	0	0	0
2015404681	470	rural/clinic	No	07/09/2017	0	0	0
2015402030	470	rural/clinic	No	07/09/2017	0	0	0
2015402029	470	rural/clinic	No	07/09/2017	0	0	0
2014301252	470	rural/clinic	No	07/09/2017	0	0	0
2015362550	470	rural/clinic	No	07/09/2017	0	0	0
2014301251	470	rural/clinic	No	07/09/2017	0	0	0
2014301473	470	rural/clinic	No	07/09/2017	0	0	0
2015333459	470	rural/clinic	No	07/09/2017	0	0	0
2015333118	470	rural/clinic	No	07/09/2017	0	0	0
2015333119	470	rural/clinic	No	07/09/2017	0	0	0
2015333120	470	rural/clinic	No	07/09/2017	0	0	0

2015333121	470	rural/clinic	No	07/09/2017	0	0	0
2015362548	470	rural/clinic	No	07/09/2017	0	0	0
2015412258	470	rural/clinic	No	07/09/2017	0	0	0
2015412122	470	rural/clinic	No	07/09/2017	0	0	0
2015402678	470	rural/clinic	No	07/09/2017	0	0	0
2015402680	470	rural/clinic	No	07/09/2017	0	0	0
2015402679	470	rural/clinic	No	07/09/2017	0	0	0
2015402681	470	rural/clinic	No	07/09/2017	0	0	0
2015402682	470	rural/clinic	No	07/09/2017	0	0	0
2015402683	470	rural/clinic	No	07/09/2017	0	0	0
2015402684	470	rural/clinic	No	07/09/2017	0	0	0
2015402685	470	rural/clinic	No	07/09/2017	0	0	0
2015397689	470	rural/clinic	No	11/09/2017	0	0	0
2015397688	470	rural/clinic	No	11/09/2017	0	0	0
2015397687	470	rural/clinic	No	11/09/2017	0	0	0
2015397686	470	rural/clinic	No	11/09/2017	0	0	0
2015397684	470	rural/clinic	No	11/09/2017	0	0	0
2015397683	470	rural/clinic	No	11/09/2017	0	0	0
2015362549	470	rural/clinic	No	07/09/2017	0	0	0
2015397682	470	rural/clinic	No	11/09/2017	0	0	0
2012317573	470	rural/clinic	No	07/09/2017	0	0	0
2015397681	470	rural/clinic	No	11/09/2017	0	0	0
2012312167	470	rural/clinic	No	07/09/2017	0	0	0
2012312164	470	rural/clinic	No	07/09/2017	0	0	0
2015397685	470	rural/clinic	No	11/09/2017	0	0	0
2012312165	470	rural/clinic	No	07/09/2017	0	0	0
2012312166	470	rural/clinic	No	07/09/2017	0	0	0
2015412785	470	rural/clinic	No	07/09/2017	0	0	0
2012312161	470	rural/clinic	No	07/09/2017	0	0	0
2012312157	470	rural/clinic	No	07/09/2017	0	0	0
2012312159	470	rural/clinic	No	07/09/2017	0	0	0
2012312160	470	rural/clinic	No	07/09/2017	0	0	0
2014345756	470	rural/clinic	No	07/09/2017	0	0	0
2014345755	470	rural/clinic	No	07/09/2017	0	0	0
2014345754	470	rural/clinic	No	07/09/2017	0	0	0
2014345753	470	rural/clinic	No	07/09/2017	0	0	0
2015413936	470	rural/clinic	No	07/09/2017	0	0	0
2015413935	470	rural/clinic	No	07/09/2017	0	0	0
2015340389	470	rural/clinic	No	07/09/2017	0	0	0
2015340388	470	rural/clinic	No	07/09/2017	0	0	0
2014346745	470	rural/clinic	No	07/09/2017	0	0	0
2014346746	470	rural/clinic	No	07/09/2017	0	0	0
2014346747	470	rural/clinic	No	07/09/2017	0	0	0
2014346749	470	rural/clinic	No	07/09/2017	0	0	0
2012344507	470	rural/clinic	No	07/09/2017	0	0	0
2011133923	470	rural/clinic	No	07/09/2017	0	0	0
2011133925	470	rural/clinic	No	07/09/2017	0	0	0
2012295645	470	rural/clinic	No	07/09/2017	0	0	0
2011123784	470	rural/clinic	No	07/09/2017	0	0	0
2012344510	470	rural/clinic	No	07/09/2017	0	0	0
2012344508	470	rural/clinic	No	07/09/2017	0	0	0
2014371169	470	rural/clinic	No	07/09/2017	0	0	0
2014300430	470	rural/clinic	No	07/09/2017	0	0	0
2015403323	470	rural/clinic	No	07/09/2017	0	0	0
2015403322	470	rural/clinic	No	07/09/2017	0	0	0
2015403319	470	rural/clinic	No	07/09/2017	0	0	0
2011137330	470	rural/clinic	No	07/09/2017	0	0	0
2015290235	470	rural/clinic	No	07/09/2017	0	0	0
2015310671	470	rural/clinic	No	07/09/2017	0	0	0
2015310670	470	rural/clinic	No	07/09/2017	0	0	0
2015290234	470	rural/clinic	No	07/09/2017	0	0	0
2014298600	470	rural/clinic	No	07/09/2017	0	0	0
2015290037	470	rural/clinic	No	07/09/2017	0	0	0
2014303732	470	rural/clinic	No	07/09/2017	0	0	0
2014303735	470	rural/clinic	No	07/09/2017	0	0	0
2014303729	470	rural/clinic	No	07/09/2017	0	0	0
2014303733	470	rural/clinic	No	07/09/2017	0	0	0
2014303730	470	rural/clinic	No	07/09/2017	0	0	0
2015403205	470	rural/clinic	No	07/09/2017	0	0	0
2015403208	470	rural/clinic	No	07/09/2017	0	0	0
2015403207	470	rural/clinic	No	07/09/2017	0	0	0
2015293498	470	rural/clinic	No	07/09/2017	0	0	0
2015293496	470	rural/clinic	No	07/09/2017	0	0	0
2015372831	470	rural/clinic	No	07/09/2017	0	0	0
2015386825	470	rural/clinic	No	07/09/2017	0	0	0
2015386828	312	rural/clinic	No	07/09/2017	0	0	0
2015386827	312	rural/clinic	No	07/09/2017	0	0	0
2015386826	312	rural/clinic	No	07/09/2017	0	0	0
2015386824	312	rural/clinic	No	07/09/2017	0	0	0
2014303731	312	rural/clinic	No	07/09/2017	0	0	0
2014303728	312	rural/clinic	No	07/09/2017	0	0	0
2014303734	312	rural/clinic	No	07/09/2017	0	0	0

2015310621	312	rural/clinic	No	07/09/2017	0	0	0
2014288172	312	rural/clinic	No	07/09/2017	0	0	0
2014327533	312	rural/clinic	No	07/09/2017	0	0	0
2015364348	312	rural/clinic	No	07/09/2017	0	0	0
2015364349	312	rural/clinic	No	07/09/2017	0	0	0
2015364350	312	rural/clinic	No	07/09/2017	0	0	0
2015382620	313	rural/clinic	No	07/09/2017	0	0	0
2010099941	313	rural/clinic	No	07/09/2017	0	0	0
2011133923	313	rural/clinic	No	07/09/2017	0	0	0
2011133923	313	rural/clinic	No	07/09/2017	0	0	0
201075903	313	rural/clinic	No	07/09/2017	0	0	0
201075903	313	rural/clinic	No	07/09/2017	0	0	0
2012390661	313	rural/clinic	No	07/09/2017	0	0	0
2012390661	313	rural/clinic	No	07/09/2017	0	0	0
2011137325	313	rural/clinic	No	07/09/2017	0	0	0
2011137325	313	rural/clinic	No	07/09/2017	0	0	0
2014350639	314	rural/clinic	No	07/09/2017	0	0	0
2014350639	314	rural/clinic	No	07/09/2017	0	0	0
2015367718	314	rural/clinic	No	07/09/2017	0	0	0
2015367718	314	rural/clinic	No	07/09/2017	0	0	0
2015287394	314	rural/clinic	No	17/08/2017	0	0	0
2015324047	314	rural/clinic	No	07/08/2017	0	0	0
2015402684	314	rural/clinic	No	07/09/2017	0	0	0
2015402684	314	rural/clinic	No	07/09/2017	0	0	0
2014303655	314	rural/clinic	No	12/09/2017	0	0	0
2012304342	314	rural/clinic	No	12/09/2017	0	0	0
2014303657	314	rural/clinic	No	12/09/2017	0	0	0
2015326999	314	rural/clinic	No	12/09/2017	0	0	0
2015289574	314	rural/clinic	No	12/09/2017	0	0	0
2014367410	314	rural/clinic	No	12/09/2017	0	0	0
2015337562	314	rural/clinic	No	12/09/2017	0	0	0
2014326644	314	rural/clinic	No	12/09/2017	0	0	0
2015368793	314	rural/clinic	No	12/09/2017	0	0	0
2015337560	314	rural/clinic	No	12/09/2017	0	0	0
2014326645	314	rural/clinic	No	12/09/2017	0	0	0
2014367411	314	rural/clinic	No	12/09/2017	0	0	0
2012291494	314	rural/clinic	No	12/09/2017	0	0	0
2014326646	314	rural/clinic	No	12/09/2017	0	0	0
2014367412	314	rural/clinic	No	12/09/2017	0	0	0
2015352380	314	rural/clinic	No	12/09/2017	0	0	0
2014326647	314	rural/clinic	No	12/09/2017	0	0	0
2015335812	314	rural/clinic	No	12/09/2017	0	0	0
2015345096	314	rural/clinic	No	12/09/2017	0	0	0
2015315010	314	rural/clinic	No	12/09/2017	0	0	0
2012371459	314	rural/clinic	No	12/09/2017	0	0	0
2015368739	314	rural/clinic	No	12/09/2017	0	0	0
2012369851	314	rural/clinic	No	12/09/2017	0	0	0
2014326648	314	rural/clinic	No	12/09/2017	0	0	0
2015368750	314	rural/clinic	No	12/09/2017	0	0	0
2014326649	314	rural/clinic	No	12/09/2017	0	0	0
2014367413	314	rural/clinic	No	12/09/2017	0	0	0
2015368749	314	rural/clinic	No	12/09/2017	0	0	0
2015332449	314	rural/clinic	No	12/09/2017	0	0	0
2015384499	314	rural/clinic	No	12/09/2017	0	0	0
2014327534	314	rural/clinic	No	12/09/2017	0	0	0
2015327000	314	rural/clinic	No	12/09/2017	0	0	0
2015326998	314	rural/clinic	No	12/09/2017	0	0	0
2015368794	314	rural/clinic	No	12/09/2017	0	0	0
2015342062	314	rural/clinic	No	12/09/2017	0	0	0
2015331782	314	rural/clinic	No	12/09/2017	0	0	0
2014327535	314	rural/clinic	No	12/09/2017	0	0	0
2015342300	314	rural/clinic	No	12/09/2017	0	0	0
2014291823	314	rural/clinic	No	12/09/2017	0	0	0
2015342299	314	rural/clinic	No	12/09/2017	0	0	0
2014291824	314	rural/clinic	No	12/09/2017	0	0	0
2015331781	314	rural/clinic	No	12/09/2017	0	0	0
2014303656	314	rural/clinic	No	12/09/2017	0	0	0
2015414282	314	rural/clinic	No	12/09/2017	0	0	0
2015414283	314	rural/clinic	No	12/09/2017	0	0	0
2015403739	314	rural/clinic	No	12/09/2017	0	0	0
2015414284	314	rural/clinic	No	12/09/2017	0	0	0
2015414285	314	rural/clinic	No	12/09/2017	0	0	0
2014375545	314	rural/clinic	No	12/09/2017	0	0	0
2015352379	314	rural/clinic	No	12/09/2017	0	0	0
2014375546	314	rural/clinic	No	12/09/2017	0	0	0
2015352378	314	rural/clinic	No	12/09/2017	0	0	0
2015368900	314	rural/clinic	No	12/09/2017	0	0	0
2012294357	314	rural/clinic	No	12/09/2017	0	0	0
2015369944	314	rural/clinic	No	12/09/2017	0	0	0
2015403738	314	rural/clinic	No	12/09/2017	0	0	0
2015332450	314	rural/clinic	No	12/09/2017	0	0	0
2015403737	314	rural/clinic	No	12/09/2017	0	0	0

2013256304	314	rural/clinic	No	12/09/2017	0	0	0
2012291391	314	rural/clinic	No	12/09/2017	0	0	0
2015403736	314	rural/clinic	No	12/09/2017	0	0	0
2015289196	314	rural/clinic	No	12/09/2017	0	0	0
2012359173	314	rural/clinic	No	12/09/2017	0	0	0
2015403735	314	rural/clinic	No	12/09/2017	0	0	0
2015368630	314	rural/clinic	No	12/09/2017	0	0	0
2015377771	314	rural/clinic	No	12/09/2017	0	0	0
2015377772	314	rural/clinic	No	12/09/2017	0	0	0
2015368629	314	rural/clinic	No	12/09/2017	0	0	0
2015403734	314	rural/clinic	No	12/09/2017	0	0	0
2015377773	314	rural/clinic	No	12/09/2017	0	0	0
2015403733	314	rural/clinic	No	12/09/2017	0	0	0
2013264088	314	rural/clinic	No	12/09/2017	0	0	0
2015377774	314	rural/clinic	No	12/09/2017	0	0	0
2013264086	314	rural/clinic	No	12/09/2017	0	0	0
2012294067	314	rural/clinic	No	12/09/2017	0	0	0
2013264087	314	rural/clinic	No	12/09/2017	0	0	0
2012294068	314	rural/clinic	No	12/09/2017	0	0	0
2013264089	314	rural/clinic	No	12/09/2017	0	0	0
2011135081	314	rural/clinic	No	12/09/2017	0	0	0
2015325948	191	rural/clinic	No	12/09/2017	0	0	0
2013285133	191	rural/clinic	No	12/09/2017	0	0	0
2012363992	191	rural/clinic	No	12/09/2017	0	0	0
2015340921	191	rural/clinic	No	12/09/2017	0	0	0
2012336639	191	rural/clinic	No	12/09/2017	0	0	0
2015325947	191	rural/clinic	No	12/09/2017	0	0	0
2015401705	191	rural/clinic	No	12/09/2017	0	0	0
2014349928	191	rural/clinic	No	12/09/2017	0	0	0
2012362102	191	rural/clinic	No	12/09/2017	0	0	0
2014370318	191	rural/clinic	No	12/09/2017	0	0	0
2014349929	191	rural/clinic	No	12/09/2017	0	0	0
2015293497	191	rural/clinic	No	12/09/2017	0	0	0
2012379915	191	rural/clinic	No	12/09/2017	0	0	0
2015401703	191	rural/clinic	No	12/09/2017	0	0	0
2015401176	192	rural/clinic	No	12/09/2017	0	0	0
2015401177	192	rural/clinic	No	12/09/2017	0	0	0
2015401175	192	rural/clinic	No	12/09/2017	0	0	0
2015401174	192	rural/clinic	No	12/09/2017	0	0	0
2015340940	192	rural/clinic	No	12/09/2017	0	0	0
2015401173	192	rural/clinic	No	12/09/2017	0	0	0
2014357338	192	rural/clinic	No	12/09/2017	0	0	0
2015325189	192	rural/clinic	No	12/09/2017	0	0	0
2014360974	192	rural/clinic	No	12/09/2017	0	0	0
2015357536	192	rural/clinic	No	12/09/2017	0	0	0
2014358034	192	rural/clinic	No	12/09/2017	0	0	0
2015357537	192	rural/clinic	No	12/09/2017	0	0	0
2014358035	192	rural/clinic	No	12/09/2017	0	0	0
2015325190	192	rural/clinic	No	12/09/2017	0	0	0
2015340939	192	rural/clinic	No	12/09/2017	0	0	0
2015325238	192	rural/clinic	No	12/09/2017	0	0	0
2015409039	192	rural/clinic	No	12/09/2017	0	0	0
2014357833	192	rural/clinic	No	12/09/2017	0	0	0
2012252101	192	rural/clinic	No	12/09/2017	0	0	0
2014320307	192	rural/clinic	No	12/09/2017	0	0	0
2014320306	192	rural/clinic	No	12/09/2017	0	0	0
2014357834	192	rural/clinic	No	12/09/2017	0	0	0
2015355931	192	rural/clinic	No	12/09/2017	0	0	0
2014361229	192	rural/clinic	No	12/09/2017	0	0	0
2015355930	192	rural/clinic	No	12/09/2017	0	0	0
2015409040	192	rural/clinic	No	12/09/2017	0	0	0
2015390328	192	rural/clinic	No	12/09/2017	0	0	0
2014332732	192	rural/clinic	No	12/09/2017	0	0	0
2014368699	192	rural/clinic	No	12/09/2017	0	0	0
2014358233	192	rural/clinic	No	12/09/2017	0	0	0
2014368698	192	rural/clinic	No	12/09/2017	0	0	0
2014368697	192	rural/clinic	No	12/09/2017	0	0	0
2012291390	192	rural/clinic	No	12/09/2017	0	0	0
2014360975/D	192	rural/clinic	No	12/09/2017	0	0	0
2012244294	192	rural/clinic	No	12/09/2017	0	0	0
2015326243	192	rural/clinic	No	12/09/2017	0	0	0
2015401704	192	rural/clinic	No	12/09/2017	0	0	0
2012362101	192	rural/clinic	No	12/09/2017	0	0	0
2015326244	192	rural/clinic	No	12/09/2017	0	0	0
2014363597	192	rural/clinic	No	12/09/2017	0	0	0
2015335987	192	rural/clinic	No	12/09/2017	0	0	0
2015335988	192	rural/clinic	No	12/09/2017	0	0	0
2015335600	192	rural/clinic	No	12/09/2017	0	0	0
2015368628	192	rural/clinic	No	12/09/2017	0	0	0
2015368627	192	rural/clinic	No	12/09/2017	0	0	0
2015335599	554	rural/clinic	No	12/09/2017	0	0	0
2015294895	554	rural/clinic	No	12/09/2017	0	0	0

2015303190	554	rural/clinic	No	12/09/2017	0	0	0
2015303191	554	rural/clinic	No	12/09/2017	0	0	0
2014365476	554	rural/clinic	No	12/09/2017	0	0	0
2014365477	554	rural/clinic	No	12/09/2017	0	0	0
2015405416	554	rural/clinic	No	12/09/2017	0	0	0
2015362434	554	rural/clinic	No	12/09/2017	0	0	0
2015363478	554	rural/clinic	No	12/09/2017	0	0	0
2015363161	554	rural/clinic	No	12/09/2017	0	0	0
2015319306	554	rural/clinic	No	12/09/2017	0	0	0
2013265068	554	rural/clinic	No	12/09/2017	0	0	0
2015355787	554	rural/clinic	No	12/09/2017	0	0	0
2015294896	554	rural/clinic	No	12/09/2017	0	0	0
2015294897	554	rural/clinic	No	12/09/2017	0	0	0
2014372771	554	rural/clinic	No	12/09/2017	0	0	0
2015363162	554	rural/clinic	No	12/09/2017	0	0	0
2015363163	554	rural/clinic	No	12/09/2017	0	0	0
2014372772	554	rural/clinic	No	12/09/2017	0	0	0
2014309392	554	rural/clinic	No	12/09/2017	0	0	0
2015363159	554	rural/clinic	No	12/09/2017	0	0	0
2014372773	315	rural/clinic	No	12/09/2017	0	0	0
2015404178	315	rural/clinic	No	12/09/2017	0	0	0
2015363166	315	rural/clinic	No	12/09/2017	0	0	0
2015404177	315	rural/clinic	No	12/09/2017	0	0	0
2011222233	315	rural/clinic	No	12/09/2017	0	0	0
2014342549	315	rural/clinic	No	12/09/2017	0	0	0
2014358232	315	rural/clinic	No	12/09/2017	0	0	0
2015363479	315	rural/clinic	No	12/09/2017	0	0	0
2012344872	315	rural/clinic	No	12/09/2017	0	0	0
2013254813	315	rural/clinic	No	12/09/2017	0	0	0
2015315919	315	rural/clinic	No	12/09/2017	0	0	0
2014309394	315	rural/clinic	No	12/09/2017	0	0	0
2015337546	315	rural/clinic	No	12/09/2017	0	0	0
2012344867	315	rural/clinic	No	12/09/2017	0	0	0
2015363160	315	rural/clinic	No	12/09/2017	0	0	0
2012344874	315	rural/clinic	No	12/09/2017	0	0	0
2012317284	315	rural/clinic	No	12/09/2017	0	0	0
2015335982	315	rural/clinic	No	12/09/2017	0	0	0
2015337548	315	rural/clinic	No	12/09/2017	0	0	0
2012321344	315	rural/clinic	No	12/09/2017	0	0	0
2015315921	315	rural/clinic	No	12/09/2017	0	0	0
2015385872	315	rural/clinic	No	12/09/2017	0	0	0
2015289341	315	rural/clinic	No	12/09/2017	0	0	0
2015363477	315	rural/clinic	No	12/09/2017	0	0	0
2015385958	178	rural/clinic	No	12/09/2017	0	0	0
2014309393	178	rural/clinic	No	12/09/2017	0	0	0
2015385960	178	rural/clinic	No	12/09/2017	0	0	0
2012344869	178	rural/clinic	No	12/09/2017	0	0	0
2015335981	178	rural/clinic	No	12/09/2017	0	0	0
2015385875	178	rural/clinic	No	12/09/2017	0	0	0
2015314622	178	rural/clinic	No	12/09/2017	0	0	0
2015294894	178	rural/clinic	No	12/09/2017	0	0	0
2015338721	178	rural/clinic	No	12/09/2017	0	0	0
2011135963	178	rural/clinic	No	12/09/2017	0	0	0
2014343882	178	rural/clinic	No	12/09/2017	0	0	0
2015337547	178	rural/clinic	No	12/09/2017	0	0	0
2015314624	178	rural/clinic	No	12/09/2017	0	0	0
2015364981	178	rural/clinic	No	12/09/2017	0	0	0
2015385880	178	rural/clinic	No	12/09/2017	0	0	0
2015362435	148	rural/clinic	No	12/09/2017	0	0	0
2015364980	148	rural/clinic	No	12/09/2017	0	0	0
2012344871	148	rural/clinic	No	12/09/2017	0	0	0
2015404176	148	rural/clinic	No	12/09/2017	0	0	0
2015364879	288	rural/clinic	No	12/09/2017	0	0	0
2015347208	288	rural/clinic	No	12/09/2017	0	0	0
2015364978	288	rural/clinic	No	12/09/2017	0	0	0
2015315918	288	rural/clinic	No	12/09/2017	0	0	0
2012371460	288	rural/clinic	No	12/09/2017	0	0	0
2013254814	288	rural/clinic	No	12/09/2017	0	0	0
2015364977	288	rural/clinic	No	12/09/2017	0	0	0
2012371461	288	rural/clinic	No	12/09/2017	0	0	0
2015385877	288	rural/clinic	No	12/09/2017	0	0	0
2015364976	288	rural/clinic	No	12/09/2017	0	0	0
2011223213	288	rural/clinic	No	17/09/2017	0	0	0
2015364975	288	rural/clinic	No	12/09/2017	0	0	0
2012371462	288	rural/clinic	No	12/09/2017	0	0	0
2015364974	288	rural/clinic	No	12/09/2017	0	0	0
2015314623	288	rural/clinic	No	12/09/2017	0	0	0
2015364973	288	rural/clinic	No	12/09/2017	0	0	0
2014348250	288	rural/clinic	No	12/09/2017	0	0	0
2012323782	288	rural/clinic	No	12/09/2017	0	0	0
2015364972	288	rural/clinic	No	12/09/2017	0	0	0
2012371457	288	rural/clinic	No	12/09/2017	0	0	0

2015337556	288	rural/clinic	No	12/09/2017	0	0	0
2011140795	288	rural/clinic	No	12/09/2017	0	0	0
2014338458	288	rural/clinic	No	12/09/2017	0	0	0
2015337557	288	rural/clinic	No	12/09/2017	0	0	0
2011140796	288	rural/clinic	No	12/09/2017	0	0	0
2014348249	288	rural/clinic	No	12/09/2017	0	0	0
2015337565	288	rural/clinic	No	12/09/2017	0	0	0
2011140797	288	rural/clinic	No	12/09/2017	0	0	0
2015385881	288	rural/clinic	No	12/09/2017	0	0	0
2014348247	288	rural/clinic	No	12/09/2017	0	0	0
2011140798	288	rural/clinic	No	12/09/2017	0	0	0
2015385957	288	rural/clinic	No	12/09/2017	0	0	0
2014348248	288	rural/clinic	No	12/09/2017	0	0	0
2011140799	288	rural/clinic	No	12/09/2017	0	0	0
2012371456	288	rural/clinic	No	12/09/2017	0	0	0
2014372730	288	rural/clinic	No	12/09/2017	0	0	0
2011140800	288	rural/clinic	No	12/09/2017	0	0	0
2012371458	288	rural/clinic	No	12/09/2017	0	0	0
2015324135	288	rural/clinic	No	12/09/2017	0	0	0
2011138601	288	rural/clinic	No	12/09/2017	0	0	0
2011138602	288	rural/clinic	No	12/09/2017	0	0	0
2015413937	288	rural/clinic	No	12/09/2017	0	0	0
2011138603	288	rural/clinic	No	12/09/2017	0	0	0
2015345100	288	rural/clinic	No	12/09/2017	0	0	0
2011138604	288	rural/clinic	No	12/09/2017	0	0	0
2011138605	288	rural/clinic	No	12/09/2017	0	0	0
2015345099	288	rural/clinic	No	12/09/2017	0	0	0
2011138606	288	rural/clinic	No	12/09/2017	0	0	0
2011138607	288	rural/clinic	No	12/09/2017	0	0	0
2011138608	288	rural/clinic	No	12/09/2017	0	0	0
2011138609	288	rural/clinic	No	12/09/2017	0	0	0
2015345098	288	rural/clinic	No	12/09/2017	0	0	0
2015345097	288	rural/clinic	No	12/09/2017	0	0	0
2015372949	288	rural/clinic	No	12/09/2017	0	0	0
2015372877	288	rural/clinic	No	12/09/2017	0	0	0
2014338517	288	rural/clinic	No	12/09/2017	0	0	0
2011144845	288	rural/clinic	No	12/09/2017	0	0	0
2014383711	288	rural/clinic	No	12/09/2017	0	0	0
2011144844	288	rural/clinic	No	12/09/2017	0	0	0
2012276264	288	rural/clinic	No	12/09/2017	0	0	0
2015324490	288	rural/clinic	No	12/09/2017	0	0	0
2012276266	288	rural/clinic	No	12/09/2017	0	0	0
2012276265	288	rural/clinic	No	12/09/2017	0	0	0
2015324491	288	rural/clinic	No	12/09/2017	0	0	0
2014363752	288	rural/clinic	No	12/09/2017	0	0	0
2011135964	288	rural/clinic	No	12/09/2017	0	0	0
2015324492	288	rural/clinic	No	12/09/2017	0	0	0
2015385879	288	rural/clinic	No	12/09/2017	0	0	0
2015324493	288	rural/clinic	No	12/09/2017	0	0	0
2015385874	288	rural/clinic	No	12/09/2017	0	0	0
2015385878	288	rural/clinic	No	12/09/2017	0	0	0
2015337540	288	rural/clinic	No	12/09/2017	0	0	0
2015324494	288	rural/clinic	No	12/09/2017	0	0	0
2013254815	288	rural/clinic	No	12/09/2017	0	0	0
2015314621	288	rural/clinic	No	12/09/2017	0	0	0
2015338722	288	rural/clinic	No	12/09/2017	0	0	0
2015385876	288	rural/clinic	No	12/09/2017	0	0	0
2015337545	288	rural/clinic	No	12/09/2017	0	0	0
2012344875	288	rural/clinic	No	12/09/2017	0	0	0
2012344873	288	rural/clinic	No	12/09/2017	0	0	0
2015385873	288	rural/clinic	No	12/09/2017	0	0	0
2015337559	288	rural/clinic	No	12/09/2017	0	0	0
2015337558	288	rural/clinic	No	12/09/2017	0	0	0
2015337541	288	rural/clinic	No	12/09/2017	0	0	0
2011135965	288	rural/clinic	No	12/09/2017	0	0	0
2015338723	288	rural/clinic	No	12/09/2017	0	0	0
2015385959	288	rural/clinic	No	12/09/2017	0	0	0
2011135962	288	rural/clinic	No	12/09/2017	0	0	0
2011135961	288	rural/clinic	No	12/09/2017	0	0	0
2015337542	288	rural/clinic	No	12/09/2017	0	0	0
2015337543	288	rural/clinic	No	12/09/2017	0	0	0
2015337564	288	rural/clinic	No	12/09/2017	0	0	0
2015385956	288	rural/clinic	No	12/09/2017	0	0	0
2015347001	288	rural/clinic	No	12/09/2017	0	0	0
2015385962	288	rural/clinic	No	12/09/2017	0	0	0
2015337635	288	rural/clinic	No	12/09/2017	0	0	0
2015337634	288	rural/clinic	No	12/09/2017	0	0	0
2015337633	288	rural/clinic	No	12/09/2017	0	0	0
2015337636	219	rural/clinic	No	12/09/2017	0	0	0
2012344870	219	rural/clinic	No	12/09/2017	0	0	0
2015315920	219	rural/clinic	No	12/09/2017	0	0	0
2015337544	219	rural/clinic	No	12/09/2017	0	0	0

2015385954	219	rural/clinic	No	12/09/2017	0	0	0
2015345154	219	rural/clinic	No	14/09/2017	0	0	0
2015345155	219	rural/clinic	No	14/09/2017	0	0	0
2012390668	101	rural/clinic	No	14/09/2017	0	0	0
2012390669	101	rural/clinic	No	14/09/2017	0	0	0
2012390670	101	rural/clinic	No	14/09/2017	0	0	0
2012390671	101	rural/clinic	No	14/09/2017	0	0	0
2012275232	101	rural/clinic	No	29/08/2017	0	0	0
2012275233	101	rural/clinic	No	14/09/2017	0	0	0
2012275235	101	rural/clinic	No	14/09/2017	0	0	0
2012275238	101	rural/clinic	No	14/09/2017	0	0	0
2012275239	101	rural/clinic	No	14/09/2017	0	0	0
2014333612	101	rural/clinic	No	14/09/2017	0	0	0
2015369209	101	rural/clinic	No	14/09/2017	0	0	0
2015339553	101	rural/clinic	No	14/09/2017	0	0	0
2015326608	101	rural/clinic	No	14/09/2017	0	0	0
2015368303	101	rural/clinic	No	14/09/2017	0	0	0
2015362926	101	rural/clinic	No	14/09/2017	0	0	0
2015332379	101	rural/clinic	No	14/09/2017	0	0	0
2015418873	101	rural/clinic	No	14/09/2017	0	0	0
2011198698	101	rural/clinic	No	14/09/2017	0	0	0
2015414282	101	rural/clinic	No	12/09/2017	0	0	0
2015414282	101	rural/clinic	No	12/09/2017	0	0	0
2011135078	101	rural/clinic	No	12/09/2017	0	0	0
2011135078	101	rural/clinic	No	12/09/2017	0	0	0
2012275241	101	rural/clinic	No	14/09/2017	0	0	0
2012275240	101	rural/clinic	No	14/09/2017	0	0	0
2012275237	101	rural/clinic	No	14/09/2017	0	0	0
2012275234	101	rural/clinic	No	14/09/2017	0	0	0
2015357537	486	rural/clinic	No	12/09/2017	0	0	0
2015357537	486	rural/clinic	No	12/09/2017	0	0	0
2011198699	486	rural/clinic	No	14/09/2017	0	0	0
2015352379	486	rural/clinic	No	12/09/2017	0	0	0
2015352379	486	rural/clinic	No	12/09/2017	0	0	0
2014326649	486	rural/clinic	No	12/09/2017	0	0	0
2014326649	486	rural/clinic	No	12/09/2017	0	0	0
2011198700	486	rural/clinic	No	14/09/2017	0	0	0
2015406414	486	rural/clinic	No	14/09/2017	0	0	0
2015406412	486	rural/clinic	No	14/09/2017	0	0	0
2015386829	486	rural/clinic	No	14/09/2017	0	0	0
2011155245	486	rural/clinic	No	14/09/2017	0	0	0
2015406411	486	rural/clinic	No	14/09/2017	0	0	0
2015406410	486	rural/clinic	No	14/09/2017	0	0	0
2015406415	486	rural/clinic	No	14/09/2017	0	0	0
2015368208	486	rural/clinic	No	14/09/2017	0	0	0
2015289249	486	rural/clinic	No	14/09/2017	0	0	0
2015414660	486	rural/clinic	No	14/09/2017	0	0	0
2015382625	486	rural/clinic	No	14/09/2017	0	0	0
2015289250	486	rural/clinic	No	14/09/2017	0	0	0
2015382626	486	rural/clinic	No	14/09/2017	0	0	0
2015382627	486	rural/clinic	No	14/09/2017	0	0	0
2015382628	486	rural/clinic	No	14/09/2017	0	0	0
2015382629	486	rural/clinic	No	14/09/2017	0	0	0
2015382630	486	rural/clinic	No	14/09/2017	0	0	0
2015382631	486	rural/clinic	No	14/09/2017	0	0	0
2015382632	486	rural/clinic	No	14/09/2017	0	0	0
2015382633	486	rural/clinic	No	14/09/2017	0	0	0
2011100951	486	rural/clinic	No	14/09/2017	0	0	0
2015382763	486	rural/clinic	No	14/09/2017	0	0	0
2015382764	486	rural/clinic	No	14/09/2017	0	0	0
2011100952	486	rural/clinic	No	14/09/2017	0	0	0
2015382765	486	rural/clinic	No	14/09/2017	0	0	0
2011100953	486	rural/clinic	No	14/09/2017	0	0	0
2015382766	486	rural/clinic	No	14/09/2017	0	0	0
2014354765	486	rural/clinic	No	14/09/2017	0	0	0
2015321811	486	rural/clinic	No	14/09/2017	0	0	0
2011100954	486	rural/clinic	No	14/09/2017	0	0	0
2015321812	486	rural/clinic	No	14/09/2017	0	0	0
2011100955	486	rural/clinic	No	14/09/2017	0	0	0
2014363104	486	rural/clinic	No	14/09/2017	0	0	0
2015345153	486	rural/clinic	No	14/09/2017	0	0	0
2015405807	486	rural/clinic	No	14/09/2017	0	0	0
2015361198	486	rural/clinic	No	14/09/2017	0	0	0
2015361199	486	rural/clinic	No	14/09/2017	0	0	0
2012336640	486	rural/clinic	No	14/09/2017	0	0	0
2015361200	486	rural/clinic	No	14/09/2017	0	0	0
2015326932	486	rural/clinic	No	14/09/2017	0	0	0
2015415665	486	rural/clinic	No	14/09/2017	0	0	0
2015326931	486	rural/clinic	No	14/09/2017	0	0	0
2015415666	486	rural/clinic	No	14/09/2017	0	0	0
2015326933	486	rural/clinic	No	14/09/2017	0	0	0
2015415667	486	rural/clinic	No	14/09/2017	0	0	0

2015326934	486	rural/clinic	No	14/09/2017	0	0	0
2015415668	486	rural/clinic	No	14/09/2017	0	0	0
2015287406	486	rural/clinic	No	14/09/2017	0	0	0
2013256701	486	rural/clinic	No	14/09/2017	0	0	0
2015332982	486	rural/clinic	No	14/09/2017	0	0	0
2013256702	486	rural/clinic	No	14/09/2017	0	0	0
2014306855	486	rural/clinic	No	14/09/2017	0	0	0
2014306856	486	rural/clinic	No	14/09/2017	0	0	0
2011144847	486	rural/clinic	No	14/09/2017	0	0	0
2015372950	486	rural/clinic	No	14/09/2017	0	0	0
2015332981	486	rural/clinic	No	14/09/2017	0	0	0
2015403551	486	rural/clinic	No	14/09/2017	0	0	0
2012242592	486	rural/clinic	No	14/09/2017	0	0	0
2015403552	486	rural/clinic	No	14/09/2017	0	0	0
2014383989	486	rural/clinic	No	14/09/2017	0	0	0
2015406409	486	rural/clinic	No	14/09/2017	0	0	0
2015406413	486	rural/clinic	No	14/09/2017	0	0	0
2015404175	486	rural/clinic	No	14/09/2017	0	0	0
2015352660	486	rural/clinic	No	14/09/2017	0	0	0
2015324138	486	rural/clinic	No	14/09/2017	0	0	0
2015403207	486	rural/clinic	No	07/09/2017	0	0	0
2015403207	486	rural/clinic	No	07/09/2017	0	0	0
2012265644	486	rural/clinic	No	07/09/2017	0	0	0
2012265644	486	rural/clinic	No	07/09/2017	0	0	0
2014313670	486	rural/clinic	No	07/09/2017	0	0	0
2014313670	486	rural/clinic	No	07/09/2017	0	0	0
2014346337	486	rural/clinic	No	14/09/2017	0	0	0
2015404173	486	rural/clinic	No	14/09/2017	0	0	0
2014335535	486	rural/clinic	No	14/09/2017	0	0	0
2015332035	486	rural/clinic	No	14/09/2017	0	0	0
2010099944	486	rural/clinic	No	14/09/2017	0	0	0
2010099945	486	rural/clinic	No	14/09/2017	0	0	0
2010099946	486	rural/clinic	No	14/09/2017	0	0	0
2010099947	486	rural/clinic	No	14/09/2017	0	0	0
2015412786	486	rural/clinic	No	14/09/2017	0	0	0
2015340241	486	rural/clinic	No	14/09/2017	0	0	0
2015340242	486	rural/clinic	No	14/09/2017	0	0	0
2012312169	486	rural/clinic	No	14/09/2017	0	0	0
2014314657	486	rural/clinic	No	14/09/2017	0	0	0
2015340243	486	rural/clinic	No	14/09/2017	0	0	0
2013264225	486	rural/clinic	No	14/09/2017	0	0	0
2015340244	486	rural/clinic	No	14/09/2017	0	0	0
2014314656	486	rural/clinic	No	14/09/2017	0	0	0
2012381664	486	rural/clinic	No	14/09/2017	0	0	0
2015340245	486	rural/clinic	No	14/09/2017	0	0	0
2012381666	486	rural/clinic	No	14/09/2017	0	0	0
2015340246	486	rural/clinic	No	14/09/2017	0	0	0
2012381665	486	rural/clinic	No	14/09/2017	0	0	0
2014290243	486	rural/clinic	No	14/09/2017	0	0	0
2014345757	486	rural/clinic	No	14/09/2017	0	0	0
2012379917	486	rural/clinic	No	14/09/2017	0	0	0
2015305838	486	rural/clinic	No	14/09/2017	0	0	0
2015289245	486	rural/clinic	No	14/09/2017	0	0	0
2015305839	486	rural/clinic	No	14/09/2017	0	0	0
2015289246	486	rural/clinic	No	14/09/2017	0	0	0
2015305840	486	rural/clinic	No	14/09/2017	0	0	0
2015289247	486	rural/clinic	No	14/09/2017	0	0	0
2015305841	486	rural/clinic	No	14/09/2017	0	0	0
2015305842	486	rural/clinic	No	14/09/2017	0	0	0
2014298907	486	rural/clinic	No	14/09/2017	0	0	0
2014298908	486	rural/clinic	No	14/09/2017	0	0	0
2014317657	486	rural/clinic	No	14/09/2017	0	0	0
2014317658	486	rural/clinic	No	14/09/2017	0	0	0
2015369015	486	rural/clinic	No	14/09/2017	0	0	0
2015369037	486	rural/clinic	No	14/09/2017	0	0	0
2015369038	471	rural/clinic	No	14/09/2017	0	0	0
2015369039	471	rural/clinic	No	14/09/2017	0	0	0
2012365992	471	rural/clinic	No	14/09/2017	0	0	0
2012365993	471	rural/clinic	No	14/09/2017	0	0	0
2014360886	471	rural/clinic	No	14/09/2017	0	0	0
2015402034	471	rural/clinic	No	14/09/2017	0	0	0
2015402035	471	rural/clinic	No	14/09/2017	0	0	0
2015402036	471	rural/clinic	No	14/09/2017	0	0	0
2015402037	471	rural/clinic	No	14/09/2017	0	0	0
2015402038	471	rural/clinic	No	14/09/2017	0	0	0
2015402039	471	rural/clinic	No	14/09/2017	0	0	0
2015402040	471	rural/clinic	No	14/09/2017	0	0	0
2015402041	471	rural/clinic	No	14/09/2017	0	0	0
2015402042	471	rural/clinic	No	14/09/2017	0	0	0
2015402692	471	rural/clinic	No	14/09/2017	0	0	0
2015402693	471	rural/clinic	No	14/09/2017	0	0	0
2015402694	471	rural/clinic	No	14/09/2017	0	0	0

2015402695	471	rural/clinic	No	14/09/2017	0	0	0
2015289248	471	rural/clinic	No	14/09/2017	0	0	0
2011225381	471	rural/clinic	No	14/09/2017	0	0	0
2015349546	471	rural/clinic	No	14/09/2017	0	0	0
2014319098	471	rural/clinic	No	14/09/2017	0	0	0
2011224440	471	rural/clinic	No	14/09/2017	0	0	0
2015302155	471	rural/clinic	No	14/09/2017	0	0	0
2015302156	471	rural/clinic	No	14/09/2017	0	0	0
2015349852	471	rural/clinic	No	14/09/2017	0	0	0
2011179940	471	rural/clinic	No	14/09/2017	0	0	0
2014319099	471	rural/clinic	No	14/09/2017	0	0	0
2014350050	471	rural/clinic	No	14/09/2017	0	0	0
2015301508	471	rural/clinic	No	14/09/2017	0	0	0
2015419081	512	rural/clinic	No	14/09/2017	0	0	0
2015419082	512	rural/clinic	No	14/09/2017	0	0	0
2015419083	512	rural/clinic	No	14/09/2017	0	0	0
2015419084	512	rural/clinic	No	14/09/2017	0	0	0
2015419085	512	rural/clinic	No	14/09/2017	0	0	0
2015402696	512	rural/clinic	No	14/09/2017	0	0	0
2015340391	512	rural/clinic	No	14/09/2017	0	0	0
2014319527	512	rural/clinic	No	14/09/2017	0	0	0
2015286718	512	rural/clinic	No	14/09/2017	0	0	0
2015286407	512	rural/clinic	No	14/09/2017	0	0	0
2015286720	512	rural/clinic	No	14/09/2017	0	0	0
2015286408	512	rural/clinic	No	14/09/2017	0	0	0
2015286404	512	rural/clinic	No	14/09/2017	0	0	0
2014319528	512	rural/clinic	No	14/09/2017	0	0	0
2012370984	512	rural/clinic	No	14/09/2017	0	0	0
2015360215	512	rural/clinic	No	14/09/2017	0	0	0
2014350464	512	rural/clinic	No	14/09/2017	0	0	0
2015360213	512	rural/clinic	No	14/09/2017	0	0	0
2015360211	512	rural/clinic	No	14/09/2017	0	0	0
2012268530	512	rural/clinic	No	14/09/2017	0	0	0
2015360214	512	rural/clinic	No	14/09/2017	0	0	0
2015386830	512	rural/clinic	No	14/09/2017	0	0	0
2015386831	512	rural/clinic	No	14/09/2017	0	0	0
2015386832	512	rural/clinic	No	14/09/2017	0	0	0
2015386833	512	rural/clinic	No	14/09/2017	0	0	0
2015386834	512	rural/clinic	No	14/09/2017	0	0	0
2015386835	512	rural/clinic	No	14/09/2017	0	0	0
2015345154	512	rural/clinic	No	14/09/2017	0	0	0
2015345154	512	rural/clinic	No	14/09/2017	0	0	0
2015386836	316	rural/clinic	No	14/09/2017	0	0	0
2015362925	316	rural/clinic	No	14/09/2017	0	0	0
2012276264	316	rural/clinic	No	12/09/2017	0	0	0
2012276264	316	rural/clinic	No	12/09/2017	0	0	0
2015362927	316	rural/clinic	No	14/09/2017	0	0	0
2015337559	316	rural/clinic	No	12/09/2017	0	0	0
2011140799	316	rural/clinic	No	12/09/2017	0	0	0
2011140799	316	rural/clinic	No	12/09/2017	0	0	0
2011138605	316	rural/clinic	No	12/09/2017	0	0	0
2011138605	316	rural/clinic	No	12/09/2017	0	0	0
2015362928	316	rural/clinic	No	14/09/2017	0	0	0
2015362929	316	rural/clinic	No	14/09/2017	0	0	0
2015338721	316	rural/clinic	No	12/09/2017	0	0	0
2015338721	316	rural/clinic	No	12/09/2017	0	0	0
2011223213	316	rural/clinic	No	17/09/2017	0	0	0
2011223213	344	rural/clinic	No	17/09/2017	0	0	0
2015362930	344	rural/clinic	No	14/09/2017	0	0	0
2015362931	344	rural/clinic	No	14/09/2017	0	0	0
2012371461	344	rural/clinic	No	12/09/2017	0	0	0
2012371461	344	rural/clinic	No	12/09/2017	0	0	0
2015372878	344	rural/clinic	No	14/09/2017	0	0	0
2015372879	344	rural/clinic	No	14/09/2017	0	0	0
2014327092	344	rural/clinic	No	14/09/2017	0	0	0
2015302860	344	rural/clinic	No	14/09/2017	0	0	0
2011225380	344	rural/clinic	No	14/09/2017	0	0	0
2011225379	344	rural/clinic	No	14/09/2017	0	0	0
2014368030	344	rural/clinic	No	14/09/2017	0	0	0
2014368033	344	rural/clinic	No	14/09/2017	0	0	0
2012268786	344	rural/clinic	No	14/09/2017	0	0	0
2012268785	344	rural/clinic	No	14/09/2017	0	0	0
2015400301	344	rural/clinic	No	14/09/2017	0	0	0
2015400302	344	rural/clinic	No	14/09/2017	0	0	0
2015410501	344	rural/clinic	No	14/09/2017	0	0	0
2015360213	344	rural/clinic	No	14/09/2017	0	0	0
2015360213	344	rural/clinic	No	14/09/2017	0	0	0
2015360211	344	rural/clinic	No	14/09/2017	0	0	0
2015360211	344	rural/clinic	No	14/09/2017	0	0	0
2014317658	344	rural/clinic	No	14/09/2017	0	0	0
2014317658	344	rural/clinic	No	14/09/2017	0	0	0
2014346337	344	rural/clinic	No	14/09/2017	0	0	0

2014346337	344	rural/clinic	No	14/09/2017	0	0	0
2015404173	344	rural/clinic	No	14/09/2017	0	0	0
2015404173	344	rural/clinic	No	14/09/2017	0	0	0
2012275233	344	rural/clinic	No	14/09/2017	0	0	0
2012275233	344	rural/clinic	No	14/09/2017	0	0	0
2015382764	344	rural/clinic	No	14/09/2017	0	0	0
2015382764	344	rural/clinic	No	14/09/2017	0	0	0
2014329145	344	rural/clinic	No	18/09/2017	0	0	0
2015336890	344	rural/clinic	No	18/09/2017	0	0	0
2015336891	344	rural/clinic	No	18/09/2017	0	0	0
2015286717	344	rural/clinic	No	18/09/2017	0	0	0
2011142911	344	rural/clinic	No	18/09/2017	0	0	0
2015336708	344	rural/clinic	No	18/09/2017	0	0	0
2014329146	344	rural/clinic	No	18/09/2017	0	0	0
2015340390	344	rural/clinic	No	18/09/2017	0	0	0
2015400302	344	rural/clinic	No	14/09/2017	0	0	0
2015400302	344	rural/clinic	No	14/09/2017	0	0	0
2015289245	344	rural/clinic	No	14/09/2017	0	0	0
2015289245	344	rural/clinic	No	14/09/2017	0	0	0
2015334215	344	rural/clinic	No	19/09/2017	0	0	0
2014297608	344	rural/clinic	No	19/09/2017	0	0	0
2011223622	344	rural/clinic	No	19/09/2017	0	0	0
2011139413	344	rural/clinic	No	19/09/2017	0	0	0
2014311378	344	rural/clinic	No	19/09/2017	0	0	0
201085817	344	rural/clinic	No	19/09/2017	0	0	0
2011223623	344	rural/clinic	No	19/09/2017	0	0	0
2014297609	344	rural/clinic	No	19/09/2017	0	0	0
2015403740	344	rural/clinic	No	19/09/2017	0	0	0
2015403741	344	rural/clinic	No	19/09/2017	0	0	0
2015403742	344	rural/clinic	No	19/09/2017	0	0	0
2015403743	344	rural/clinic	No	19/09/2017	0	0	0
2015390459	344	rural/clinic	No	19/09/2017	0	0	0
2015403744	344	rural/clinic	No	19/09/2017	0	0	0
2015403745	344	rural/clinic	No	19/09/2017	0	0	0
2015390460	344	rural/clinic	No	19/09/2017	0	0	0
2015403746	344	rural/clinic	No	19/09/2017	0	0	0
2015390457	344	rural/clinic	No	19/09/2017	0	0	0
2015407601	344	rural/clinic	No	19/09/2017	0	0	0
2015390461	344	rural/clinic	No	13/09/2017	0	0	0
2015407602	344	rural/clinic	No	19/09/2017	0	0	0
2015390458	344	rural/clinic	No	19/09/2017	0	0	0
2014365400	344	rural/clinic	No	19/09/2017	0	0	0
2011152393	374	rural/clinic	No	19/09/2017	0	0	0
2015338900	374	rural/clinic	No	07/09/2017	0	0	0
2015405417	374	rural/clinic	No	19/09/2017	0	0	0
2013244119	374	rural/clinic	No	19/09/2017	0	0	0
2015355025	374	rural/clinic	No	19/09/2017	0	0	0
2015325243	374	rural/clinic	No	19/09/2017	0	0	0
2015355027	374	rural/clinic	No	19/09/2017	0	0	0
2015355028	374	rural/clinic	No	19/09/2017	0	0	0
2015325949	374	rural/clinic	No	19/09/2017	0	0	0
2015355029	374	rural/clinic	No	19/09/2017	0	0	0
2015340924	374	rural/clinic	No	19/09/2017	0	0	0
2011133926	374	rural/clinic	No	19/09/2017	0	0	0
2012312211	374	rural/clinic	No	19/09/2017	0	0	0
2015313005	374	rural/clinic	No	19/09/2017	0	0	0
2015417098	374	rural/clinic	No	19/09/2017	0	0	0
2012312210	374	rural/clinic	No	19/09/2017	0	0	0
2015313006	374	rural/clinic	No	19/09/2017	0	0	0
2014385699	375	rural/clinic	No	29/08/2017	0	0	0
2014291826	375	rural/clinic	No	19/09/2017	0	0	0
2014297610	375	rural/clinic	No	19/09/2017	0	0	0
2012305838	375	rural/clinic	No	19/09/2017	0	0	0
2015397881	375	rural/clinic	No	19/09/2017	0	0	0
2015325241	375	rural/clinic	No	19/09/2017	0	0	0
2015344674	375	rural/clinic	No	19/09/2017	0	0	0
2015334218	375	rural/clinic	No	19/09/2017	0	0	0
2015334415	375	rural/clinic	No	19/07/2017	0	0	0
2015325950	375	rural/clinic	No	19/09/2017	0	0	0
2015334416	375	rural/clinic	No	19/09/2017	0	0	0
2015344675	375	rural/clinic	No	19/09/2017	0	0	0
201085816	375	rural/clinic	No	19/09/2017	0	0	0
201085815	375	rural/clinic	No	19/09/2017	0	0	0
2015325245	375	rural/clinic	No	19/09/2017	0	0	0
2015344676	375	rural/clinic	No	19/09/2017	0	0	0
2015355026	375	rural/clinic	No	19/09/2017	0	0	0
2015355030	375	rural/clinic	No	19/09/2017	0	0	0
2015344677	375	rural/clinic	No	19/09/2017	0	0	0
2015325244	375	rural/clinic	No	19/09/2017	0	0	0
2015334823	375	rural/clinic	No	19/09/2017	0	0	0
2015344678	375	rural/clinic	No	19/09/2017	0	0	0
2014343883	375	rural/clinic	No	17/07/2017	0	0	0

2015325242	375	rural/clinic	No	19/09/2017	0	0	0
2015337388	375	rural/clinic	No	19/09/2017	0	0	0
2015337392	375	rural/clinic	No	19/09/2017	0	0	0
2015337391	472	rural/clinic	No	19/09/2017	0	0	0
2015324050	472	rural/clinic	No	19/09/2017	0	0	0
2015344680	472	rural/clinic	No	19/09/2017	0	0	0
2014320308	472	rural/clinic	No	19/09/2017	0	0	0
2012261387	472	rural/clinic	No	19/09/2017	0	0	0
2014320309	472	rural/clinic	No	19/09/2017	0	0	0
2015335989	472	rural/clinic	No	19/09/2017	0	0	0
2015344681	472	rural/clinic	No	19/09/2017	0	0	0
2014320310	472	rural/clinic	No	19/09/2017	0	0	0
2014309395	472	rural/clinic	No	19/09/2017	0	0	0
2011135692	472	rural/clinic	No	19/09/2017	0	0	0
2014314487	472	rural/clinic	No	19/09/2017	0	0	0
2014320312	472	rural/clinic	No	19/09/2017	0	0	0
2012317285	472	rural/clinic	No	19/09/2017	0	0	0
2015360953	472	rural/clinic	No	19/09/2017	0	0	0
2015344748	472	rural/clinic	No	19/09/2017	0	0	0
2014320311	472	rural/clinic	No	19/09/2017	0	0	0
2015344749	410	rural/clinic	No	19/09/2017	0	0	0
2015397882	410	rural/clinic	No	19/09/2017	0	0	0
2015372832	410	rural/clinic	No	19/09/2017	0	0	0
2015397877	410	rural/clinic	No	19/09/2017	0	0	0
2015294630	410	rural/clinic	No	19/09/2017	0	0	0
2015397879	410	rural/clinic	No	19/09/2017	0	0	0
2015368631	410	rural/clinic	No	19/09/2017	0	0	0
2015397880	555	rural/clinic	No	19/09/2017	0	0	0
2015344488	555	rural/clinic	No	19/09/2017	0	0	0
2015412654	555	rural/clinic	No	19/09/2017	0	0	0
2013256283	555	rural/clinic	No	19/09/2017	0	0	0
2014382716	555	rural/clinic	No	19/09/2017	0	0	0
2014301474	555	rural/clinic	No	19/09/2017	0	0	0
2014377421	555	rural/clinic	No	19/09/2017	0	0	0
2014382714	555	rural/clinic	No	19/09/2017	0	0	0
2013256282	555	rural/clinic	No	19/09/2017	0	0	0
2015412459	555	rural/clinic	No	19/09/2017	0	0	0
2014382715	555	rural/clinic	No	19/09/2017	0	0	0
2011141096	555	rural/clinic	No	19/09/2017	0	0	0
2015397878	555	rural/clinic	No	19/09/2017	0	0	0
2015412458	317	rural/clinic	No	19/09/2017	0	0	0
2011141097	317	rural/clinic	No	19/09/2017	0	0	0
2011125799	317	rural/clinic	No	19/09/2017	0	0	0
2014338459	317	rural/clinic	No	19/09/2017	0	0	0
2012276268	937	rural/clinic	No	19/09/2017	0	0	0
2011140555	938	rural/clinic	No	19/09/2017	0	0	0
2011141095	938	rural/clinic	No	19/09/2017	0	0	0
2014290277	938	rural/clinic	No	19/09/2017	0	0	0
2014327698	938	rural/clinic	No	19/09/2017	0	0	0
2015287407	938	rural/clinic	No	19/09/2017	0	0	0
2012371463	938	rural/clinic	No	19/09/2017	0	0	0
201075909	938	rural/clinic	No	19/09/2017	0	0	0
2012276267	938	rural/clinic	No	19/09/2017	0	0	0
2015397690	623	rural/clinic	No	19/09/2017	0	0	0
2015336665	623	rural/clinic	No	19/09/2017	0	0	0
2015397691	623	rural/clinic	No	19/09/2017	0	0	0
201075910	623	rural/clinic	No	19/09/2017	0	0	0
2015336666	623	rural/clinic	No	19/09/2017	0	0	0
2015336664	623	rural/clinic	No	19/09/2017	0	0	0
2015336668	623	rural/clinic	No	19/09/2017	0	0	0
2015336667	623	rural/clinic	No	19/09/2017	0	0	0
2011135084	623	rural/clinic	No	19/09/2017	0	0	0
2011135083	623	rural/clinic	No	19/09/2017	0	0	0
2011135085	623	rural/clinic	No	19/09/2017	0	0	0
2011100956	623	rural/clinic	No	19/09/2017	0	0	0
2015345156	623	rural/clinic	No	19/09/2017	0	0	0
201075911	623	rural/clinic	No	19/09/2017	0	0	0
2012291392	623	rural/clinic	No	19/09/2017	0	0	0
2015397692	623	rural/clinic	No	19/09/2017	0	0	0
201075912	623	rural/clinic	No	19/09/2017	0	0	0
2013264090	623	rural/clinic	No	19/09/2017	0	0	0
201075913	68	rural/clinic	No	19/09/2017	0	0	0
2013264092	68	rural/clinic	No	19/09/2017	0	0	0
2015397693	68	rural/clinic	No	19/09/2017	0	0	0
2011196024	68	rural/clinic	No	19/09/2017	0	0	0
201075914	68	rural/clinic	No	19/09/2017	0	0	0
2015397694	68	rural/clinic	No	19/09/2017	0	0	0
2015287399	68	rural/clinic	No	19/09/2017	0	0	0
201075915	68	rural/clinic	No	19/09/2017	0	0	0
2015397695	68	rural/clinic	No	19/09/2017	0	0	0
2015303518	68	rural/clinic	No	19/09/2017	0	0	0
2011137335	68	rural/clinic	No	19/09/2017	0	0	0

2011196025	68	rural/clinic	No	19/09/2017	0	0	0
2015397696	68	rural/clinic	No	19/09/2017	0	0	0
2011137334	68	rural/clinic	No	19/09/2017	0	0	0
2015397697	68	rural/clinic	No	19/09/2017	0	0	0
2015334216	68	rural/clinic	No	19/09/2017	0	0	0
2011137333	68	rural/clinic	No	19/09/2017	0	0	0
2015287398	68	rural/clinic	No	19/09/2017	0	0	0
2015303519	68	rural/clinic	No	19/09/2017	0	0	0
2015303256	68	rural/clinic	No	19/09/2017	0	0	0
2015334821	68	rural/clinic	No	19/09/2017	0	0	0
2015303255	68	rural/clinic	No	19/09/2017	0	0	0
2015303253	68	rural/clinic	No	19/09/2017	0	0	0
2014288589	68	rural/clinic	No	19/09/2017	0	0	0
2015334822	68	rural/clinic	No	19/09/2017	0	0	0
2015303254	68	rural/clinic	No	19/09/2017	0	0	0
2015303257	68	rural/clinic	No	19/09/2017	0	0	0
2015334219	68	rural/clinic	No	18/09/2017	0	0	0
2015334818	68	rural/clinic	No	19/09/2017	0	0	0
2015368686	68	rural/clinic	No	19/09/2017	0	0	0
2015368685	68	rural/clinic	No	19/09/2017	0	0	0
2015334817	68	rural/clinic	No	19/09/2017	0	0	0
2012295832	68	rural/clinic	No	19/09/2017	0	0	0
2014288587/D	68	rural/clinic	No	19/09/2017	0	0	0
2012295829	68	rural/clinic	No	19/09/2017	0	0	0
2014288679	68	rural/clinic	No	19/09/2017	0	0	0
2015334217	68	rural/clinic	No	18/09/2017	0	0	0
2015334819	68	rural/clinic	No	19/09/2017	0	0	0
2015334820	251	rural/clinic	No	19/09/2017	0	0	0
2011135691	251	rural/clinic	No	19/09/2017	0	0	0
2015334540	251	rural/clinic	No	19/09/2017	0	0	0
2015344486	251	rural/clinic	No	19/09/2017	0	0	0
2015303827	251	rural/clinic	No	19/09/2017	0	0	0
2015344487	251	rural/clinic	No	19/09/2017	0	0	0
2015344348	251	rural/clinic	No	19/09/2017	0	0	0
2015303829	251	rural/clinic	No	19/09/2017	0	0	0
2013281903	251	rural/clinic	No	19/09/2017	0	0	0
2012291393	251	rural/clinic	No	19/09/2017	0	0	0
2015342323	251	rural/clinic	No	19/09/2017	0	0	0
2015401178	251	rural/clinic	No	19/09/2017	0	0	0
2011196023	251	rural/clinic	No	19/09/2017	0	0	0
2015401179	251	rural/clinic	No	19/09/2017	0	0	0
2015401180	251	rural/clinic	No	19/09/2017	0	0	0
2014327697	251	rural/clinic	No	19/09/2017	0	0	0
2014327610	251	rural/clinic	No	19/09/2017	0	0	0
2015417100	251	rural/clinic	No	19/09/2017	0	0	0
2015417003	251	rural/clinic	No	19/09/2017	0	0	0
2011137332	251	rural/clinic	No	19/09/2017	0	0	0
2012295831	251	rural/clinic	No	19/09/2017	0	0	0
2012295830	251	rural/clinic	No	19/09/2017	0	0	0
2015347002	251	rural/clinic	No	19/09/2017	0	0	0
2015417002	251	rural/clinic	No	19/09/2017	0	0	0
2015337393	251	rural/clinic	No	19/09/2017	0	0	0
2015337549	289	rural/clinic	No	19/09/2017	0	0	0
2015385963	289	rural/clinic	No	19/09/2017	0	0	0
2015377775	289	rural/clinic	No	19/09/2017	0	0	0
2015324495	289	rural/clinic	No	19/09/2017	0	0	0
2015355537	289	rural/clinic	No	19/09/2017	0	0	0
2015377776	289	rural/clinic	No	19/09/2017	0	0	0
2012369852	289	rural/clinic	No	19/09/2017	0	0	0
201066593	289	rural/clinic	No	19/09/2017	0	0	0
2011195905	289	rural/clinic	No	19/09/2017	0	0	0
2015347004	289	rural/clinic	No	19/09/2017	0	0	0
2015339779	289	rural/clinic	No	19/09/2017	0	0	0
2012324255	289	rural/clinic	No	19/09/2017	0	0	0
2015385964	289	rural/clinic	No	19/09/2017	0	0	0
2014327536	289	rural/clinic	No	19/09/2017	0	0	0
2015347003	289	rural/clinic	No	19/09/2017	0	0	0
2015347005	289	rural/clinic	No	19/09/2017	0	0	0
2012284029	289	rural/clinic	No	19/09/2017	0	0	0
2015364982	289	rural/clinic	No	19/09/2017	0	0	0
2012305835	289	rural/clinic	No	19/09/2017	0	0	0
2015364983	289	rural/clinic	No	19/09/2017	0	0	0
2012284030	289	rural/clinic	No	19/09/2017	0	0	0
2015364984	289	rural/clinic	No	19/09/2017	0	0	0
2015342063	289	rural/clinic	No	19/09/2017	0	0	0
2015384500	289	rural/clinic	No	19/09/2017	0	0	0
2012253134	289	rural/clinic	No	19/09/2017	0	0	0
2012305836	289	rural/clinic	No	19/09/2017	0	0	0
2013256704	289	rural/clinic	No	19/09/2017	0	0	0
2013256705	289	rural/clinic	No	19/09/2017	0	0	0
2013256703	289	rural/clinic	No	19/09/2017	0	0	0
2015400303	289	rural/clinic	No	19/09/2017	0	0	0

2015413938	289	rural/clinic	No	19/09/2017	0	0	0
2015413939	289	rural/clinic	No	19/09/2017	0	0	0
2015352820	289	rural/clinic	No	19/09/2017	0	0	0
2015352819	289	rural/clinic	No	19/09/2017	0	0	0
2015352818	289	rural/clinic	No	19/09/2017	0	0	0
2015352817	289	rural/clinic	No	19/09/2017	0	0	0
2015352816	289	rural/clinic	No	19/09/2017	0	0	0
2015352815	289	rural/clinic	No	19/09/2017	0	0	0
2011117870	289	rural/clinic	No	19/09/2017	0	0	0
2011117871	289	rural/clinic	No	19/09/2017	0	0	0
2011117872	289	rural/clinic	No	19/09/2017	0	0	0
2014340212	289	rural/clinic	No	19/09/2017	0	0	0
2014291825	289	rural/clinic	No	19/09/2017	0	0	0
2015368908	289	rural/clinic	No	19/09/2017	0	0	0
2015339613	289	rural/clinic	No	19/09/2017	0	0	0
2015339614	289	rural/clinic	No	19/09/2017	0	0	0
2015369945	289	rural/clinic	No	19/09/2017	0	0	0
2015368906	289	rural/clinic	No	19/09/2017	0	0	0
2012253984	624	rural/clinic	No	21/09/2017	0	0	0
2012253983	624	rural/clinic	No	21/09/2017	0	0	0
2015303253	624	rural/clinic	No	19/09/2017	0	0	0
2015303253	624	rural/clinic	No	19/09/2017	0	0	0
2015294631	624	rural/clinic	No	19/09/2017	0	0	0
2015294631	624	rural/clinic	No	19/09/2017	0	0	0
2015349031	624	rural/clinic	No	21/09/2017	0	0	0
2014288170	624	rural/clinic	No	21/09/2017	0	0	0
2015415702	624	rural/clinic	No	21/09/2017	0	0	0
2015402700	624	rural/clinic	No	21/09/2017	0	0	0
2015402697	624	rural/clinic	No	21/09/2017	0	0	0
2015415703	624	rural/clinic	No	21/09/2017	0	0	0
2014303739	624	rural/clinic	No	21/09/2017	0	0	0
2011216420	624	rural/clinic	No	21/09/2017	0	0	0
2015375916	624	rural/clinic	No	21/09/2017	0	0	0
2015415704	624	rural/clinic	No	21/09/2017	0	0	0
2015415705	624	rural/clinic	No	21/09/2017	0	0	0
2011216419	624	rural/clinic	No	21/09/2017	0	0	0
2014303736	624	rural/clinic	No	21/09/2017	0	0	0
2015324136	624	rural/clinic	No	21/09/2017	0	0	0
2015415706	624	rural/clinic	No	21/09/2017	0	0	0
2014373210	624	rural/clinic	No	21/09/2017	0	0	0
2015415707	624	rural/clinic	No	21/09/2017	0	0	0
2014346334	624	rural/clinic	No	21/09/2017	0	0	0
2015415708	624	rural/clinic	No	21/09/2017	0	0	0
2015332032	624	rural/clinic	No	21/09/2017	0	0	0
2015331554	624	rural/clinic	No	21/09/2017	0	0	0
2015331556	624	rural/clinic	No	21/09/2017	0	0	0
2015286409	624	rural/clinic	No	21/09/2017	0	0	0
2015352822	736	rural/clinic	No	21/09/2017	0	0	0
2015404169	736	rural/clinic	No	21/09/2017	0	0	0
2015360212	736	rural/clinic	No	14/09/2017	0	0	0
2015404172	736	rural/clinic	No	21/09/2017	0	0	0
2015352821	736	rural/clinic	No	21/09/2017	0	0	0
2015404171	736	rural/clinic	No	21/09/2017	0	0	0
2014296338	736	rural/clinic	No	21/09/2017	0	0	0
2015404170	736	rural/clinic	No	21/09/2017	0	0	0
2014296336	736	rural/clinic	No	21/09/2017	0	0	0
2015403329	736	rural/clinic	No	21/09/2017	0	0	0
2015332111	736	rural/clinic	No	21/09/2017	0	0	0
2015403328	736	rural/clinic	No	21/09/2017	0	0	0
2015324137	736	rural/clinic	No	21/09/2017	0	0	0
2015404254	736	rural/clinic	No	21/09/2017	0	0	0
2015375220	736	rural/clinic	No	21/09/2017	0	0	0
2015403327	736	rural/clinic	No	21/09/2017	0	0	0
2015375216	736	rural/clinic	No	21/09/2017	0	0	0
2015404180	736	rural/clinic	No	21/09/2017	0	0	0
2015290236	736	rural/clinic	No	21/09/2017	0	0	0
2015403326	736	rural/clinic	No	21/09/2017	0	0	0
2012245053	736	rural/clinic	No	21/09/2017	0	0	0
2012271405	736	rural/clinic	No	21/09/2017	0	0	0
2015403325	736	rural/clinic	No	21/09/2017	0	0	0
2014303740	736	rural/clinic	No	21/09/2017	0	0	0
2011179941	736	rural/clinic	No	21/09/2017	0	0	0
2011134803	736	rural/clinic	No	21/09/2017	0	0	0
2012271106	736	rural/clinic	No	21/09/2017	0	0	0
2011226499	736	rural/clinic	No	21/09/2017	0	0	0
2014296451	736	rural/clinic	No	21/09/2017	0	0	0
2015384171	736	rural/clinic	No	21/09/2017	0	0	0
2015375913	376	rural/clinic	No	21/09/2017	0	0	0
2015324131	376	rural/clinic	No	21/09/2017	0	0	0
2015310672	376	rural/clinic	No	21/09/2017	0	0	0
2015339306	376	rural/clinic	No	21/09/2017	0	0	0
2015375914	376	rural/clinic	No	21/09/2017	0	0	0

2014296335	376	rural/clinic	No	21/09/2017	0	0	0
2015324133	376	rural/clinic	No	21/09/2017	0	0	0
2012265928	376	rural/clinic	No	21/09/2017	0	0	0
2014312414	376	rural/clinic	No	21/09/2017	0	0	0
2014296337	376	rural/clinic	No	21/09/2017	0	0	0
2015402047	376	rural/clinic	No	21/09/2017	0	0	0
2012271406	376	rural/clinic	No	21/09/2017	0	0	0
2014296334	376	rural/clinic	No	21/09/2017	0	0	0
2015338470	376	rural/clinic	No	21/09/2017	0	0	0
2012275242	376	rural/clinic	No	21/09/2017	0	0	0
2012313816	376	rural/clinic	No	21/09/2017	0	0	0
2015403331	129	rural/clinic	No	21/09/2017	0	0	0
2015347352	129	rural/clinic	No	21/09/2017	0	0	0
2011140307	129	rural/clinic	No	21/09/2017	0	0	0
2015347354	129	rural/clinic	No	21/09/2017	0	0	0
2015384311	129	rural/clinic	No	21/09/2017	0	0	0
2015347355	129	rural/clinic	No	21/09/2017	0	0	0
2015384312	129	rural/clinic	No	21/09/2017	0	0	0
2014347041	129	rural/clinic	No	21/09/2017	0	0	0
2012317576	129	rural/clinic	No	21/09/2017	0	0	0
2012317575	129	rural/clinic	No	21/09/2017	0	0	0
2014347047	129	rural/clinic	No	21/09/2017	0	0	0
2015362068	129	rural/clinic	No	21/09/2017	0	0	0
2014347049	129	rural/clinic	No	21/09/2017	0	0	0
2012317574	129	rural/clinic	No	21/09/2017	0	0	0
2015403330	129	rural/clinic	No	21/09/2017	0	0	0
2014347048	129	rural/clinic	No	21/09/2017	0	0	0
2011141112	129	rural/clinic	No	21/09/2017	0	0	0
2014347044	129	rural/clinic	No	21/09/2017	0	0	0
2014296652	129	rural/clinic	No	21/09/2017	0	0	0
2015331748	129	rural/clinic	No	21/09/2017	0	0	0
2015384173	129	rural/clinic	No	21/09/2017	0	0	0
2013255748	129	rural/clinic	No	21/09/2017	0	0	0
2011141111	129	rural/clinic	No	21/09/2017	0	0	0
2014347045	129	rural/clinic	No	21/09/2017	0	0	0
2011216421	129	rural/clinic	No	21/09/2017	0	0	0
2015331743	129	rural/clinic	No	21/09/2017	0	0	0
2015384172	129	rural/clinic	No	21/09/2017	0	0	0
2015331749	129	rural/clinic	No	21/09/2017	0	0	0
2015377335	129	rural/clinic	No	21/09/2017	0	0	0
2015290039	129	rural/clinic	No	21/09/2017	0	0	0
2014347040	129	rural/clinic	No	21/09/2017	0	0	0
2014357052	129	rural/clinic	No	21/09/2017	0	0	0
2015377336	129	rural/clinic	No	21/09/2017	0	0	0
2014303742	129	rural/clinic	No	21/09/2017	0	0	0
2015377334	129	rural/clinic	No	21/09/2017	0	0	0
2015419087	129	rural/clinic	No	21/09/2017	0	0	0
2012313815	129	rural/clinic	No	21/09/2017	0	0	0
2014347046	129	rural/clinic	No	21/09/2017	0	0	0
2014303741	129	rural/clinic	No	21/09/2017	0	0	0
2014347042	129	rural/clinic	No	21/09/2017	0	0	0
2011224441	129	rural/clinic	No	21/09/2017	0	0	0
2014303743	129	rural/clinic	No	21/09/2017	0	0	0
2011140309	129	rural/clinic	No	21/09/2017	0	0	0
2015410353	129	rural/clinic	No	21/09/2017	0	0	0
2014314240	129	rural/clinic	No	21/09/2017	0	0	0
2015326610	129	rural/clinic	No	21/09/2017	0	0	0
2014314241	129	rural/clinic	No	21/09/2017	0	0	0
2015302861	129	rural/clinic	No	21/09/2017	0	0	0
2015414465	129	rural/clinic	No	21/09/2017	0	0	0
2015326609	129	rural/clinic	No	21/09/2017	0	0	0
2015302862	129	rural/clinic	No	21/09/2017	0	0	0
2015402698	129	rural/clinic	No	21/09/2017	0	0	0
2012275243	129	rural/clinic	No	21/09/2017	0	0	0
2015419086	129	rural/clinic	No	21/09/2017	0	0	0
2015402699	129	rural/clinic	No	21/09/2017	0	0	0
2012275244	129	rural/clinic	No	21/09/2017	0	0	0
2015290233	129	rural/clinic	No	21/09/2017	0	0	0
2015349853	129	rural/clinic	No	21/09/2017	0	0	0
2012275245	129	rural/clinic	No	21/09/2017	0	0	0
2012275246	129	rural/clinic	No	21/09/2017	0	0	0
2015358341	129	rural/clinic	No	21/09/2017	0	0	0
2012275247	129	rural/clinic	No	21/09/2017	0	0	0
2015349854	513	rural/clinic	No	21/09/2017	0	0	0
2012275248	513	rural/clinic	No	21/09/2017	0	0	0
2015358342	513	rural/clinic	No	21/09/2017	0	0	0
2015362835	513	rural/clinic	No	21/09/2017	0	0	0
2015301278	513	rural/clinic	No	21/09/2017	0	0	0
2015362836	513	rural/clinic	No	21/09/2017	0	0	0
2015358343	513	rural/clinic	No	21/09/2017	0	0	0
2015302110	513	rural/clinic	No	21/09/2017	0	0	0
2014304701	556	rural/clinic	No	21/09/2017	0	0	0

2014304702	556	rural/clinic	No	21/09/2017	0	0	0
2015301371	556	rural/clinic	No	21/09/2017	0	0	0
2014304703	556	rural/clinic	No	21/09/2017	0	0	0
2015358355	556	rural/clinic	No	21/09/2017	0	0	0
2015358353	556	rural/clinic	No	21/09/2017	0	0	0
2015301370	556	rural/clinic	No	21/09/2017	0	0	0
2014304704	556	rural/clinic	No	21/09/2017	0	0	0
2015360105	556	rural/clinic	No	21/09/2017	0	0	0
2015301369	556	rural/clinic	No	21/09/2017	0	0	0
2015408116	556	rural/clinic	No	21/09/2017	0	0	0
2015352659	556	rural/clinic	No	21/09/2017	0	0	0
2015404160	556	rural/clinic	No	21/09/2017	0	0	0
2015404161	556	rural/clinic	No	21/09/2017	0	0	0
2015358354	556	rural/clinic	No	21/09/2017	0	0	0
2015404162	556	rural/clinic	No	21/09/2017	0	0	0
2015404174	556	rural/clinic	No	21/09/2017	0	0	0
2015418711	556	rural/clinic	No	21/09/2017	0	0	0
2015358352	390	rural/clinic	No	21/09/2017	0	0	0
2014346335	390	rural/clinic	No	21/09/2017	0	0	0
2015332034	390	rural/clinic	No	21/09/2017	0	0	0
2015358350	390	rural/clinic	No	21/09/2017	0	0	0
2015404179	390	rural/clinic	No	21/09/2017	0	0	0
2014372731	390	rural/clinic	No	21/09/2017	0	0	0
2015358349	390	rural/clinic	No	21/09/2017	0	0	0
2014335534	390	rural/clinic	No	21/09/2017	0	0	0
2012381667	390	rural/clinic	No	21/09/2017	0	0	0
2015358347	390	rural/clinic	No	21/09/2017	0	0	0
2014383984	390	rural/clinic	No	21/09/2017	0	0	0
2012390672	390	rural/clinic	No	21/09/2017	0	0	0
2015358346	390	rural/clinic	No	21/09/2017	0	0	0
2015386837	390	rural/clinic	No	21/09/2017	0	0	0
2015358344	390	rural/clinic	No	21/09/2017	0	0	0
2015400304	390	rural/clinic	No	21/09/2017	0	0	0
2015358345	390	rural/clinic	No	21/09/2017	0	0	0
2015386838	390	rural/clinic	No	21/09/2017	0	0	0
2015386839	390	rural/clinic	No	21/09/2017	0	0	0
2015386760	390	rural/clinic	No	21/09/2017	0	0	0
2015386761	390	rural/clinic	No	21/09/2017	0	0	0
2015386762	390	rural/clinic	No	21/09/2017	0	0	0
2015386763	390	rural/clinic	No	21/09/2017	0	0	0
2015386764	390	rural/clinic	No	21/09/2017	0	0	0
2015386765	390	rural/clinic	No	21/09/2017	0	0	0
2015415673	390	rural/clinic	No	21/09/2017	0	0	0
2015415672	390	rural/clinic	No	21/09/2017	0	0	0
2015415671	390	rural/clinic	No	21/09/2017	0	0	0
2015415670	390	rural/clinic	No	21/09/2017	0	0	0
2015415669	390	rural/clinic	No	21/09/2017	0	0	0
2012346654	390	rural/clinic	No	21/09/2017	0	0	0
2015360547	390	rural/clinic	No	21/09/2017	0	0	0
2015332371	390	rural/clinic	No	21/09/2017	0	0	0
2012365990	390	rural/clinic	No	21/09/2017	0	0	0
2011204487	390	rural/clinic	No	21/09/2017	0	0	0
2012294480	390	rural/clinic	No	21/09/2017	0	0	0
2015332487	390	rural/clinic	No	21/09/2017	0	0	0
2012365991	390	rural/clinic	No	21/09/2017	0	0	0
2014360887	390	rural/clinic	No	21/09/2017	0	0	0
2015360546	390	rural/clinic	No	21/09/2017	0	0	0
2011134801	390	rural/clinic	No	21/09/2017	0	0	0
2015406059	390	rural/clinic	No	21/09/2017	0	0	0
2011134802	390	rural/clinic	No	21/09/2017	0	0	0
2011134806	390	rural/clinic	No	21/09/2017	0	0	0
2015406062	390	rural/clinic	No	21/09/2017	0	0	0
2011134804	390	rural/clinic	No	21/09/2017	0	0	0
2011134805	390	rural/clinic	No	21/09/2017	0	0	0
2015358356	390	rural/clinic	No	21/09/2017	0	0	0
2015406063	390	rural/clinic	No	21/09/2017	0	0	0
2015378223	390	rural/clinic	No	21/09/2017	0	0	0
2015382641	390	rural/clinic	No	21/09/2017	0	0	0
2015406064	390	rural/clinic	No	21/09/2017	0	0	0
2011140308	390	rural/clinic	No	21/09/2017	0	0	0
2014347050	390	rural/clinic	No	21/09/2017	0	0	0
2015406060	390	rural/clinic	No	21/09/2017	0	0	0
2015406057	390	rural/clinic	No	21/09/2017	0	0	0
2015347353	390	rural/clinic	No	21/09/2017	0	0	0
2015406058	390	rural/clinic	No	21/09/2017	0	0	0
2015378220	390	rural/clinic	No	21/09/2017	0	0	0
2015378221	390	rural/clinic	No	21/09/2017	0	0	0
2015406061	390	rural/clinic	No	21/09/2017	0	0	0
2015378222	390	rural/clinic	No	21/09/2017	0	0	0
2015382639	390	rural/clinic	No	21/09/2017	0	0	0
2015321813	390	rural/clinic	No	21/09/2017	0	0	0
2015382634	390	rural/clinic	No	21/09/2017	0	0	0

2015340394	390	rural/clinic	No	21/09/2017	0	0	0
2015382635	390	rural/clinic	No	21/09/2017	0	0	0
2015340395	390	rural/clinic	No	21/09/2017	0	0	0
2015382636	390	rural/clinic	No	21/09/2017	0	0	0
2015340393	390	rural/clinic	No	21/09/2017	0	0	0
2015340392	390	rural/clinic	No	21/09/2017	0	0	0
2015382637	390	rural/clinic	No	21/09/2017	0	0	0
2015349029	390	rural/clinic	No	21/09/2017	0	0	0
2015382638	390	rural/clinic	No	20/09/2017	0	0	0
2014342797	390	rural/clinic	No	21/09/2017	0	0	0
2015290242	390	rural/clinic	No	21/09/2017	0	0	0
2010099949	390	rural/clinic	No	21/09/2017	0	0	0
2015290241	390	rural/clinic	No	21/09/2017	0	0	0
2010099948	390	rural/clinic	No	21/09/2017	0	0	0
2015290239	390	rural/clinic	No	21/09/2017	0	0	0
2015290240	390	rural/clinic	No	21/09/2017	0	0	0
2015357814	390	rural/clinic	No	21/09/2017	0	0	0
2015349030	390	rural/clinic	No	21/09/2017	0	0	0
2015340728	390	rural/clinic	No	21/09/2017	0	0	0
2014303737	390	rural/clinic	No	21/09/2017	0	0	0
2015340729	390	rural/clinic	No	21/09/2017	0	0	0
2015340248	390	rural/clinic	No	21/09/2017	0	0	0
2015340247	390	rural/clinic	No	21/09/2017	0	0	0
2015402050	390	rural/clinic	No	21/09/2017	0	0	0
2015402049	390	rural/clinic	No	21/09/2017	0	0	0
2015402048	390	rural/clinic	No	21/09/2017	0	0	0
2015402046	390	rural/clinic	No	21/09/2017	0	0	0
2015402045	390	rural/clinic	No	21/09/2017	0	0	0
2015402044	390	rural/clinic	No	21/09/2017	0	0	0
2015402043	390	rural/clinic	No	21/09/2017	0	0	0
2015406416	390	rural/clinic	No	21/09/2017	0	0	0
2015406417	390	rural/clinic	No	21/09/2017	0	0	0
2015406418	390	rural/clinic	No	21/09/2017	0	0	0
2015406419	390	rural/clinic	No	21/09/2017	0	0	0
2015406421	390	rural/clinic	No	21/09/2017	0	0	0
2015406420	390	rural/clinic	No	21/09/2017	0	0	0
2015406422	390	rural/clinic	No	21/09/2017	0	0	0
2015352008	390	rural/clinic	No	21/09/2017	0	0	0
2015352009	390	rural/clinic	No	21/09/2017	0	0	0
2015336103	390	rural/clinic	No	21/09/2017	0	0	0
2012352831	390	rural/clinic	No	21/09/2017	0	0	0
2012352830	390	rural/clinic	No	21/09/2017	0	0	0
2015324134	390	rural/clinic	No	21/09/2017	0	0	0
2012352832	390	rural/clinic	No	21/09/2017	0	0	0
2015287408	390	rural/clinic	No	21/09/2017	0	0	0
2015339033	390	rural/clinic	No	21/09/2017	0	0	0
2012390673	390	rural/clinic	No	21/09/2017	0	0	0
2015382640	390	rural/clinic	No	21/09/2017	0	0	0
2015339032	390	rural/clinic	No	21/09/2017	0	0	0
2015362011	390	rural/clinic	No	21/09/2017	0	0	0
2011226498	390	rural/clinic	No	21/09/2017	0	0	0
2011226497	390	rural/clinic	No	21/09/2017	0	0	0
2014347047	390	rural/clinic	No	21/09/2017	0	0	0
2014347047	390	rural/clinic	No	21/09/2017	0	0	0
2011134802	390	rural/clinic	No	21/09/2017	0	0	0
2011134802	390	rural/clinic	No	21/09/2017	0	0	0
2014304701	390	rural/clinic	No	21/09/2017	0	0	0
2014304701	390	rural/clinic	No	21/09/2017	0	0	0
2015324137	390	rural/clinic	No	21/09/2017	0	0	0
2015324137	390	rural/clinic	No	21/09/2017	0	0	0
2015349031	390	rural/clinic	No	21/09/2017	0	0	0
2015349031	390	rural/clinic	No	21/09/2017	0	0	0
2015358341	390	rural/clinic	No	21/09/2017	0	0	0
2015358341	390	rural/clinic	No	21/09/2017	0	0	0
2015358349	390	rural/clinic	No	21/09/2017	0	0	0
2015358349	390	rural/clinic	No	21/09/2017	0	0	0
2015358356	390	rural/clinic	No	21/09/2017	0	0	0
2015358356	390	rural/clinic	No	21/09/2017	0	0	0
2015402697	390	rural/clinic	No	21/09/2017	0	0	0
2015402697	390	rural/clinic	No	21/09/2017	0	0	0
2014303738	390	rural/clinic	No	21/09/2017	0	0	0
2015375219	390	rural/clinic	No	21/09/2017	0	0	0
2015375214	390	rural/clinic	No	21/09/2017	0	0	0
2015375215	390	rural/clinic	No	21/09/2017	0	0	0
2015375217	390	rural/clinic	No	21/09/2017	0	0	0
2015375218	390	rural/clinic	No	21/09/2017	0	0	0
2015290038	390	rural/clinic	No	21/09/2017	0	0	0
2015290237	390	rural/clinic	No	21/09/2017	0	0	0
2015290238	390	rural/clinic	No	21/09/2017	0	0	0
2015404166	390	rural/clinic	No	21/09/2017	0	0	0
2015404168	390	rural/clinic	No	21/09/2017	0	0	0
2015404164	390	rural/clinic	No	21/09/2017	0	0	0

2015404163	390	rural/clinic	No	21/09/2017	0	0	0
2012265929	390	rural/clinic	No	21/09/2017	0	0	0
2011144117	390	rural/clinic	No	21/09/2017	0	0	0
2015417003	390	rural/clinic	No	19/09/2017	0	0	0
2015402700	390	rural/clinic	No	21/09/2017	0	0	0
2015301509	390	rural/clinic	No	21/09/2017	0	0	0
2015301509	390	rural/clinic	No	21/09/2017	0	0	0
2014303742	390	rural/clinic	No	21/09/2017	0	0	0
2014303742	390	rural/clinic	No	21/09/2017	0	0	0
2015403331	390	rural/clinic	No	21/09/2017	0	0	0
2015403331	390	rural/clinic	No	21/09/2017	0	0	0
2015302110	390	rural/clinic	No	21/09/2017	0	0	0
2015302110	390	rural/clinic	No	21/09/2017	0	0	0
2012390672	390	rural/clinic	No	21/09/2017	0	0	0
2012390672	390	rural/clinic	No	21/09/2017	0	0	0
2015400304	390	rural/clinic	No	21/09/2017	0	0	0
2015400304	390	rural/clinic	No	21/09/2017	0	0	0
2015326937	390	rural/clinic	No	26/09/2017	0	0	0
2015390469	390	rural/clinic	No	26/09/2017	0	0	0
2015390462	390	rural/clinic	No	26/09/2017	0	0	0
2014332733	390	rural/clinic	No	26/09/2017	0	0	0
2014332734	390	rural/clinic	No	26/09/2017	0	0	0
2015326939	390	rural/clinic	No	28/09/2017	0	0	0
2015326936	390	rural/clinic	No	26/09/2017	0	0	0
2015405638	390	rural/clinic	No	26/09/2017	0	0	0
2015303126	390	rural/clinic	No	26/09/2017	0	0	0
2015405637	390	rural/clinic	No	26/09/2017	0	0	0
2015385884	390	rural/clinic	No	21/09/2017	0	0	0
2015363713	390	rural/clinic	No	26/09/2017	0	0	0
2015385885	390	rural/clinic	No	29/09/2017	0	0	0
2013264397	390	rural/clinic	No	26/09/2017	0	0	0
2015385883	390	rural/clinic	No	29/09/2017	0	0	0
2015397555	390	rural/clinic	No	26/09/2017	0	0	0
2015397556	390	rural/clinic	No	26/09/2017	0	0	0
2015367240	390	rural/clinic	No	26/09/2017	0	0	0
2015367242	390	rural/clinic	No	26/09/2017	0	0	0
2015315551	390	rural/clinic	No	26/09/2017	0	0	0
2015367243	390	rural/clinic	No	26/09/2017	0	0	0
2014365645	390	rural/clinic	No	26/09/2017	0	0	0
2015315012	390	rural/clinic	No	29/09/2017	0	0	0
2015347007	390	rural/clinic	No	29/09/2017	0	0	0
2015314625	390	rural/clinic	No	26/09/2017	0	0	0
2011135970	390	rural/clinic	No	29/09/2017	0	0	0
2011135971	390	rural/clinic	No	29/09/2017	0	0	0
2015385882	390	rural/clinic	No	29/09/2017	0	0	0
2014291626	390	rural/clinic	No	29/09/2017	0	0	0
2014365646	390	rural/clinic	No	26/09/2017	0	0	0
2015367238	390	rural/clinic	No	26/09/2017	0	0	0
2015364904	390	rural/clinic	No	26/09/2017	0	0	0
2015363766	390	rural/clinic	No	26/09/2017	0	0	0
2015364902	390	rural/clinic	No	26/09/2017	0	0	0
2015367241	390	rural/clinic	No	26/09/2017	0	0	0
2015364903	390	rural/clinic	No	26/09/2017	0	0	0
2015413218	390	rural/clinic	No	26/09/2017	0	0	0
2014336830	390	rural/clinic	No	26/09/2017	0	0	0
2015347704	390	rural/clinic	No	26/09/2017	0	0	0
2014337228	390	rural/clinic	No	26/09/2017	0	0	0
2015347705	390	rural/clinic	No	26/09/2017	0	0	0
2015363767	390	rural/clinic	No	26/09/2017	0	0	0
2015347703	390	rural/clinic	No	26/09/2017	0	0	0
2013264093	390	rural/clinic	No	26/09/2017	0	0	0
2013264091	390	rural/clinic	No	26/09/2017	0	0	0
2015315011	390	rural/clinic	No	26/09/2017	0	0	0
2015364901	390	rural/clinic	No	26/09/2017	0	0	0
2014350687	390	rural/clinic	No	26/09/2017	0	0	0
2014350686	390	rural/clinic	No	26/09/2017	0	0	0
2012324257	390	rural/clinic	No	26/09/2017	0	0	0
2013261299	390	rural/clinic	No	26/09/2017	0	0	0
2012324258	390	rural/clinic	No	26/09/2017	0	0	0
2015363020	390	rural/clinic	No	26/09/2017	0	0	0
2015408692	390	rural/clinic	No	26/09/2017	0	0	0
2015408691	390	rural/clinic	No	26/09/2017	0	0	0
2014332451	390	rural/clinic	No	26/09/2017	0	0	0
2014346750	390	rural/clinic	No	26/09/2017	0	0	0
2014375549	390	rural/clinic	No	26/09/2017	0	0	0
2014295881	390	rural/clinic	No	26/09/2017	0	0	0
2014375548	390	rural/clinic	No	26/09/2017	0	0	0
2014295879	390	rural/clinic	No	26/09/2017	0	0	0
2014367631	390	rural/clinic	No	26/09/2017	0	0	0
2014295880	390	rural/clinic	No	26/09/2017	0	0	0
2015414289	390	rural/clinic	No	26/09/2017	0	0	0
2015352823	390	rural/clinic	No	26/09/2017	0	0	0

2015414288	390	rural/clinic	No	26/09/2017	0	0	0
2014375550	390	rural/clinic	No	26/09/2017	0	0	0
2015293369	390	rural/clinic	No	26/09/2017	0	0	0
2015293368	390	rural/clinic	No	26/09/2017	0	0	0
2014375604	390	rural/clinic	No	26/09/2017	0	0	0
2015360746	390	rural/clinic	No	26/09/2017	0	0	0
2015369946	390	rural/clinic	No	26/09/2017	0	0	0
2015360747	390	rural/clinic	No	26/09/2017	0	0	0
2014375607	390	rural/clinic	No	26/09/2017	0	0	0
2014375605	390	rural/clinic	No	26/09/2017	0	0	0
2014332735	390	rural/clinic	No	26/09/2017	0	0	0
2015360952	390	rural/clinic	No	26/09/2017	0	0	0
2014375547	390	rural/clinic	No	26/09/2017	0	0	0
2014347822	390	rural/clinic	No	26/09/2017	0	0	0
2014332736	390	rural/clinic	No	26/09/2017	0	0	0
2015414286	390	rural/clinic	No	26/09/2017	0	0	0
2014347823	390	rural/clinic	No	26/09/2017	0	0	0
2014332737	390	rural/clinic	No	26/09/2017	0	0	0
2015360951	390	rural/clinic	No	26/09/2017	0	0	0
2015414287	390	rural/clinic	No	26/09/2017	0	0	0
2014332738	390	rural/clinic	No	26/09/2017	0	0	0
2014319434	390	rural/clinic	No	26/09/2017	0	0	0
2014332739	390	rural/clinic	No	26/09/2017	0	0	0
2014375606	390	rural/clinic	No	26/09/2017	0	0	0
2015287409	390	rural/clinic	No	26/09/2017	0	0	0
2014332740	390	rural/clinic	No	26/09/2017	0	0	0
2014375608	390	rural/clinic	No	26/09/2017	0	0	0
2015287410	390	rural/clinic	No	26/09/2017	0	0	0
2014375611	390	rural/clinic	No	26/09/2017	0	0	0
2015390332	390	rural/clinic	No	26/09/2017	0	0	0
2014375609	390	rural/clinic	No	26/09/2017	0	0	0
2015390331	390	rural/clinic	No	26/09/2017	0	0	0
2015360745	390	rural/clinic	No	26/09/2017	0	0	0
2014375610	390	rural/clinic	No	26/09/2017	0	0	0
2015390330	390	rural/clinic	No	26/09/2017	0	0	0
2014375612	390	rural/clinic	No	26/09/2017	0	0	0
2015360744	390	rural/clinic	No	26/09/2017	0	0	0
2015418380	390	rural/clinic	No	26/09/2017	0	0	0
2015390465	390	rural/clinic	No	26/09/2017	0	0	0
2015390470	390	rural/clinic	No	26/09/2017	0	0	0
2012294358	390	rural/clinic	No	26/09/2017	0	0	0
2015390467	390	rural/clinic	No	26/09/2017	0	0	0
2015417004	390	rural/clinic	No	26/09/2017	0	0	0
2015339783	390	rural/clinic	No	26/09/2017	0	0	0
2015390464	390	rural/clinic	No	26/09/2017	0	0	0
2015417005	390	rural/clinic	No	26/09/2017	0	0	0
2015339782	390	rural/clinic	No	26/09/2017	0	0	0
2015390466	390	rural/clinic	No	26/09/2017	0	0	0
2015382767	390	rural/clinic	No	26/09/2017	0	0	0
2015339780	390	rural/clinic	No	26/09/2017	0	0	0
2015390471	390	rural/clinic	No	26/09/2017	0	0	0
2015390463	390	rural/clinic	No	26/09/2017	0	0	0
2015339781	390	rural/clinic	No	26/09/2017	0	0	0
2015382772	390	rural/clinic	No	26/09/2017	0	0	0
2015337394	390	rural/clinic	No	26/09/2017	0	0	0
2015305845	390	rural/clinic	No	26/09/2017	0	0	0
2015337386	390	rural/clinic	No	26/09/2017	0	0	0
2015382771	390	rural/clinic	No	26/09/2017	0	0	0
2015305843	390	rural/clinic	No	26/09/2017	0	0	0
2014309979	390	rural/clinic	No	26/09/2017	0	0	0
2013271698	390	rural/clinic	No	26/09/2017	0	0	0
2011144352	390	rural/clinic	No	26/09/2017	0	0	0
2015382770	390	rural/clinic	No	26/09/2017	0	0	0
2012284580	390	rural/clinic	No	26/09/2017	0	0	0
2015414661	390	rural/clinic	No	26/09/2017	0	0	0
2012324256	390	rural/clinic	No	26/09/2017	0	0	0
2015382769	390	rural/clinic	No	26/09/2017	0	0	0
2011144351	390	rural/clinic	No	26/09/2017	0	0	0
2012370171	390	rural/clinic	No	26/09/2017	0	0	0
2012370170	390	rural/clinic	No	26/09/2017	0	0	0
2015332855	390	rural/clinic	No	26/09/2017	0	0	0
2015382768	390	rural/clinic	No	26/09/2017	0	0	0
2014319436	390	rural/clinic	No	26/09/2017	0	0	0
2015332854	390	rural/clinic	No	26/09/2017	0	0	0
2014319437	390	rural/clinic	No	26/09/2017	0	0	0
2014332741	390	rural/clinic	No	26/09/2017	0	0	0
2014367632	390	rural/clinic	No	26/09/2017	0	0	0
2014368700	390	rural/clinic	No	26/09/2017	0	0	0
2015321462	390	rural/clinic	No	26/09/2017	0	0	0
2011100959	390	rural/clinic	No	26/09/2017	0	0	0
2014332742	390	rural/clinic	No	26/09/2017	0	0	0
2011100960	390	rural/clinic	No	26/09/2017	0	0	0

2014358037	390	rural/clinic	No	26/09/2017	0	0	0
2014358038	390	rural/clinic	No	26/09/2017	0	0	0
2014358036	390	rural/clinic	No	26/09/2017	0	0	0
2015409043	390	rural/clinic	No	26/09/2017	0	0	0
2015409044	390	rural/clinic	No	26/09/2017	0	0	0
2015409041	390	rural/clinic	No	26/09/2017	0	0	0
2015409042	390	rural/clinic	No	26/09/2017	0	0	0
2015313501	390	rural/clinic	No	26/09/2017	0	0	0
2011100957	390	rural/clinic	No	26/09/2017	0	0	0
2012291399	390	rural/clinic	No	26/09/2017	0	0	0
2011100958	390	rural/clinic	No	26/09/2017	0	0	0
2012291394	390	rural/clinic	No	26/09/2017	0	0	0
2013256284	390	rural/clinic	No	26/09/2017	0	0	0
2014358236	390	rural/clinic	No	26/09/2017	0	0	0
2015368632	390	rural/clinic	No	26/09/2017	0	0	0
2014358235	390	rural/clinic	No	26/09/2017	0	0	0
2015368633	390	rural/clinic	No	26/09/2017	0	0	0
2012291398	390	rural/clinic	No	26/09/2017	0	0	0
2015382968	390	rural/clinic	No	26/09/2017	0	0	0
2015382974	390	rural/clinic	No	26/09/2017	0	0	0
2015382973	390	rural/clinic	No	26/09/2017	0	0	0
2012291400	390	rural/clinic	No	26/09/2017	0	0	0
2015382971	390	rural/clinic	No	26/09/2017	0	0	0
2012291396	390	rural/clinic	No	26/09/2017	0	0	0
2015382969	390	rural/clinic	No	26/09/2017	0	0	0
2015382972	390	rural/clinic	No	26/09/2017	0	0	0
2015382970	390	rural/clinic	No	26/09/2017	0	0	0
2015376852	390	rural/clinic	No	26/09/2017	0	0	0
2015376853	390	rural/clinic	No	26/09/2017	0	0	0
2015376854	390	rural/clinic	No	26/09/2017	0	0	0
2012291397	390	rural/clinic	No	26/09/2017	0	0	0
2014314239	390	rural/clinic	No	26/09/2017	0	0	0
2011117873	390	rural/clinic	No	26/09/2017	0	0	0
2015326938	390	rural/clinic	No	26/09/2017	0	0	0
2012379920	390	rural/clinic	No	26/09/2017	0	0	0
2015397703	390	rural/clinic	No	26/09/2017	0	0	0
2012284578	390	rural/clinic	No	26/09/2017	0	0	0
2015397698	390	rural/clinic	No	26/09/2017	0	0	0
2015397699	390	rural/clinic	No	26/09/2017	0	0	0
2015364985	390	rural/clinic	No	26/09/2017	0	0	0
2012243656	390	rural/clinic	No	28/09/2017	0	0	0
2014300356	390	rural/clinic	No	26/09/2017	0	0	0
2015397701	390	rural/clinic	No	26/09/2017	0	0	0
2012284911	390	rural/clinic	No	26/09/2017	0	0	0
2012370172	390	rural/clinic	No	26/09/2017	0	0	0
2015331186	390	rural/clinic	No	26/09/2017	0	0	0
2015360216	390	rural/clinic	No	26/09/2017	0	0	0
2015331187	390	rural/clinic	No	26/09/2017	0	0	0
2013272765	390	rural/clinic	No	26/09/2017	0	0	0
2015347405	390	rural/clinic	No	26/09/2017	0	0	0
2012350637	390	rural/clinic	No	26/09/2017	0	0	0
2014309978	390	rural/clinic	No	26/09/2017	0	0	0
2011117877	390	rural/clinic	No	26/09/2017	0	0	0
2015359633	390	rural/clinic	No	29/09/2017	0	0	0
2011117880	390	rural/clinic	No	26/09/2017	0	0	0
2012284579	390	rural/clinic	No	26/09/2017	0	0	0
2011117878	390	rural/clinic	No	26/09/2017	0	0	0
2017128291	390	rural/clinic	No	26/09/2017	0	0	0
2015364988	390	rural/clinic	No	26/09/2017	0	0	0
2015364987	390	rural/clinic	No	26/09/2017	0	0	0
2015349075	390	rural/clinic	No	26/09/2017	0	0	0
2015349076	390	rural/clinic	No	26/09/2017	0	0	0
2015364986	390	rural/clinic	No	26/09/2017	0	0	0
2015336669	390	rural/clinic	No	26/09/2017	0	0	0
2014334683	390	rural/clinic	No	26/09/2017	0	0	0
2014366077	390	rural/clinic	No	26/09/2017	0	0	0
2015349077	390	rural/clinic	No	26/09/2017	0	0	0
2012362578	390	rural/clinic	No	26/09/2017	0	0	0
2015331108	167	rural/clinic	No	26/09/2017	0	0	0
2014307422	167	rural/clinic	No	26/09/2017	0	0	0
2015331107	167	rural/clinic	No	26/09/2017	0	0	0
2015349074	167	rural/clinic	No	29/09/2017	0	0	0
2017359207	167	rural/clinic	No	26/09/2017	0	0	0
2014366076	167	rural/clinic	No	26/09/2017	0	0	0
2015331106	167	rural/clinic	No	26/09/2017	0	0	0
2015376216	167	rural/clinic	No	26/09/2017	0	0	0
2015410001	167	rural/clinic	No	26/09/2017	0	0	0
2012363994	167	rural/clinic	No	26/09/2017	0	0	0
2015349073	167	rural/clinic	No	26/09/2017	0	0	0
2012363996	167	rural/clinic	No	26/09/2017	0	0	0
2012363995	167	rural/clinic	No	26/09/2017	0	0	0
2012363993	167	rural/clinic	No	26/09/2017	0	0	0

2011135086	167	rural/clinic	No	26/09/2017	0	0	0
2011135087	167	rural/clinic	No	26/09/2017	0	0	0
2011135089	167	rural/clinic	No	26/09/2017	0	0	0
2011135088	167	rural/clinic	No	26/09/2017	0	0	0
2015349072	167	rural/clinic	No	26/09/2017	0	0	0
2011135090	240	rural/clinic	No	26/09/2017	0	0	0
2015340929	240	rural/clinic	No	26/09/2017	0	0	0
2014319136	240	rural/clinic	No	26/09/2017	0	0	0
2014335849	240	rural/clinic	No	26/09/2017	0	0	0
2015340930	240	rural/clinic	No	26/09/2017	0	0	0
2014377255	240	rural/clinic	No	26/09/2017	0	0	0
2014320313	240	rural/clinic	No	26/09/2017	0	0	0
2015340925	240	rural/clinic	No	26/09/2017	0	0	0
2014377253	240	rural/clinic	No	26/09/2017	0	0	0
2015397702	240	rural/clinic	No	26/09/2017	0	0	0
2015340926	240	rural/clinic	No	26/09/2017	0	0	0
2014377254	240	rural/clinic	No	26/09/2017	0	0	0
2015340948	240	rural/clinic	No	26/09/2017	0	0	0
2012304345	240	rural/clinic	No	26/09/2017	0	0	0
2015400307	240	rural/clinic	No	26/09/2017	0	0	0
2015340931	240	rural/clinic	No	26/09/2017	0	0	0
2012304344	240	rural/clinic	No	26/09/2017	0	0	0
2015325246	240	rural/clinic	No	26/09/2017	0	0	0
2015342324	240	rural/clinic	No	26/09/2017	0	0	0
2015340927	240	rural/clinic	No	26/09/2017	0	0	0
2015400306	240	rural/clinic	No	26/09/2017	0	0	0
2015325257	240	rural/clinic	No	26/09/2017	0	0	0
2015340928	240	rural/clinic	No	26/09/2017	0	0	0
2015400305	240	rural/clinic	No	26/09/2017	0	0	0
2015369040	240	rural/clinic	No	26/09/2017	0	0	0
2015401901	240	rural/clinic	No	26/09/2017	0	0	0
2015369041	240	rural/clinic	No	26/09/2017	0	0	0
2015287804	240	rural/clinic	No	26/09/2017	0	0	0
2014378740	240	rural/clinic	No	26/09/2017	0	0	0
2015369042	240	rural/clinic	No	26/09/2017	0	0	0
2014378741	240	rural/clinic	No	26/09/2017	0	0	0
2015369043	240	rural/clinic	No	26/09/2017	0	0	0
2015352824	240	rural/clinic	No	26/09/2017	0	0	0
2015402051	240	rural/clinic	No	26/09/2017	0	0	0
2015352825	240	rural/clinic	No	26/09/2017	0	0	0
2015402052	240	rural/clinic	No	26/09/2017	0	0	0
2015352827	240	rural/clinic	No	26/09/2017	0	0	0
2015402054	799	rural/clinic	No	26/09/2017	0	0	0
2015352828	799	rural/clinic	No	26/09/2017	0	0	0
2015402055	799	rural/clinic	No	26/09/2017	0	0	0
2015361612	799	rural/clinic	No	26/09/2017	0	0	0
2015367244	799	rural/clinic	No	26/09/2017	0	0	0
2015359767	799	rural/clinic	No	26/09/2017	0	0	0
2015321463	799	rural/clinic	No	26/09/2017	0	0	0
2015289197	799	rural/clinic	No	26/09/2017	0	0	0
2015359768	939	rural/clinic	No	26/09/2017	0	0	0
2015321461	939	rural/clinic	No	26/09/2017	0	0	0
2015359152	939	rural/clinic	No	26/09/2017	0	0	0
2015326941	939	rural/clinic	No	26/09/2017	0	0	0
2015359153	865	referral	No	26/09/2017	0	0	1
2015405674	865	referral	No	26/09/2017	0	0	1
2015359151	865	referral	No	26/09/2017	0	0	1
2015405675	865	referral	No	26/09/2017	0	0	1
2015342064	865	referral	No	26/09/2017	0	0	1
2015321606	865	referral	No	26/09/2017	0	0	1
2012305839	865	referral	No	26/09/2017	0	0	1
2015303125	865	referral	No	26/09/2017	0	0	1
2015326940	865	referral	No	26/09/2017	0	0	1
2015359766	865	referral	No	26/09/2017	0	0	1
2014319637	865	referral	No	26/09/2017	0	0	1
2014319638	865	referral	No	26/09/2017	0	0	1
2012275995	865	referral	No	26/09/2017	0	0	1
2015286874	865	referral	No	26/09/2017	0	0	1
2015347211	865	referral	No	26/09/2017	0	0	1
2015347209	865	referral	No	26/09/2017	0	0	1
2015347210	865	referral	No	26/09/2017	0	0	1
2015347006	865	referral	No	26/09/2017	0	0	1
2015337385	865	referral	No	26/09/2017	0	0	1
2015347212	865	referral	No	26/09/2017	0	0	1
2012305840	865	referral	No	26/09/2017	0	0	1
2015397698	865	referral	No	26/09/2017	0	0	1
2015397698	865	referral	No	26/09/2017	0	0	1
2015400305	865	referral	No	26/09/2017	0	0	1
2015400305	865	referral	No	26/09/2017	0	0	1
2014378741	865	referral	No	26/09/2017	0	0	1
2014378741	865	referral	No	26/09/2017	0	0	1
2015417003	865	referral	No	19/09/2017	0	0	1

2015417003	865	referral	No	19/09/2017	0	0	1
2015390464	865	referral	No	26/09/2017	0	0	1
2015390464	865	referral	No	26/09/2017	0	0	1
2015339782	865	referral	No	26/09/2017	0	0	1
2015339782	865	referral	No	26/09/2017	0	0	1
2015405637	865	referral	No	26/09/2017	0	0	1
2015405637	865	referral	No	26/09/2017	0	0	1
2014332736	865	referral	No	26/09/2017	0	0	1
2014332736	865	referral	No	26/09/2017	0	0	1
2012324257	865	referral	No	26/09/2017	0	0	1
2015369050	865	referral	No	28/09/2017	0	0	1
2015369049	865	referral	No	28/09/2017	0	0	1
2015369048	865	referral	No	28/09/2017	0	0	1
2015369044	865	referral	No	28/09/2017	0	0	1
2015369045	865	referral	No	28/09/2017	0	0	1
2015415710	865	referral	No	28/09/2017	0	0	1
2015369046	865	referral	No	28/09/2017	0	0	1
2015369047	865	referral	No	28/09/2017	0	0	1
2015415711	865	referral	No	28/09/2017	0	0	1
2014345901	865	referral	No	28/09/2017	0	0	1
2014345902	865	referral	No	28/09/2017	0	0	1
2015378224	865	referral	No	28/09/2017	0	0	1
2012363435	865	referral	No	28/09/2017	0	0	1
2015403336	865	referral	No	28/09/2017	0	0	1
2014304705	865	referral	No	28/09/2017	0	0	1
2014304706	865	referral	No	28/09/2017	0	0	1
2015403332	865	referral	No	28/09/2017	0	0	1
2015404181	865	referral	No	28/09/2017	0	0	1
2015415712	865	referral	No	28/09/2017	0	0	1
2015404182	865	referral	No	28/09/2017	0	0	1
2015403334	865	referral	No	28/09/2017	0	0	1
2015352661	865	referral	No	28/09/2017	0	0	1
2015415713	865	referral	No	28/09/2017	0	0	1
2014297849	865	referral	No	28/09/2017	0	0	1
2015339469	865	referral	No	28/09/2017	0	0	1
2015415714	865	referral	No	28/09/2017	0	0	1
2015324139	865	referral	No	28/09/2017	0	0	1
2014300434	865	referral	No	28/09/2017	0	0	1
2015415715	865	referral	No	28/09/2017	0	0	1
2014346338	865	referral	No	28/09/2017	0	0	1
2015415716	865	referral	No	28/09/2017	0	0	1
2015404183	865	referral	No	28/09/2017	0	0	1
2011155345	865	referral	No	28/09/2017	0	0	1
2014346339	865	referral	No	28/09/2017	0	0	1
2015403335	865	referral	No	28/09/2017	0	0	1
2012269000	865	referral	No	28/09/2017	0	0	1
2014346336	865	referral	No	28/09/2017	0	0	1
2015403333	865	referral	No	28/09/2017	0	0	1
2014372729	865	referral	No	28/09/2017	0	0	1
2012268999	865	referral	No	28/09/2017	0	0	1
2015404165	865	referral	No	28/09/2017	0	0	1
2015294186	865	referral	No	28/09/2017	0	0	1
2015386768	865	referral	No	28/09/2017	0	0	1
2014298341	865	referral	No	28/09/2017	0	0	1
2015339034	865	referral	No	28/09/2017	0	0	1
2014373211	865	referral	No	28/09/2017	0	0	1
2015332916	865	referral	No	28/09/2017	0	0	1
2014300543	865	referral	No	28/09/2017	0	0	1
2015386767	865	referral	No	28/09/2017	0	0	1
2015331680	865	referral	No	28/09/2017	0	0	1
2015386840	865	referral	No	28/09/2017	0	0	1
2015294187	865	referral	No	28/09/2017	0	0	1
2014296339	865	referral	No	28/09/2017	0	0	1
2015386766	865	referral	No	28/09/2017	0	0	1
2014296340	865	referral	No	28/09/2017	0	0	1
2015331557	865	referral	No	28/09/2017	0	0	1
2015376218	865	referral	No	28/09/2017	0	0	1
2015376217	865	referral	No	28/09/2017	0	0	1
2012317577/D	865	referral	No	28/09/2017	0	0	1
2015384314	865	referral	No	28/09/2017	0	0	1
2014314243	865	referral	No	28/09/2017	0	0	1
2014314242	865	referral	No	28/09/2017	0	0	1
2015364403	865	referral	No	28/09/2017	0	0	1
2015364402	865	referral	No	28/09/2017	0	0	1
2015364550	865	referral	No	28/09/2017	0	0	1
2015352012	865	referral	No	28/09/2017	0	0	1
2014294159	865	referral	No	28/09/2017	0	0	1
2014294162	865	referral	No	28/09/2017	0	0	1
2014294160	865	referral	No	28/09/2017	0	0	1
2015415072	865	referral	No	28/09/2017	0	0	1
2015364548	865	referral	No	28/09/2017	0	0	1
2014372346	865	referral	No	28/09/2017	0	0	1

2015364549	865	referral	No	28/09/2017	0	0	1
2015332036	865	referral	No	28/09/2017	0	0	1
2014372343	865	referral	No	28/09/2017	0	0	1
2015364401	865	referral	No	28/09/2017	0	0	1
2014372344	865	referral	No	28/09/2017	0	0	1
2014347754	865	referral	No	28/09/2017	0	0	1
2014321435	865	referral	No	28/09/2017	0	0	1
2014347751	865	referral	No	28/09/2017	0	0	1
2014321436	865	referral	No	28/09/2017	0	0	1
2015352013	865	referral	No	28/09/2017	0	0	1
2014346340	865	referral	No	28/09/2017	0	0	1
2014372345	865	referral	No	28/09/2017	0	0	1
2015352010	865	referral	No	28/09/2017	0	0	1
2014307749	865	referral	No	28/09/2017	0	0	1
2014347752	865	referral	No	28/09/2017	0	0	1
2015358360	865	referral	No	28/09/2017	0	0	1
2014347753	865	referral	No	28/09/2017	0	0	1
2015358357	865	referral	No	28/09/2017	0	0	1
2015364404	865	referral	No	28/09/2017	0	0	1
2015358359	865	referral	No	28/09/2017	0	0	1
2015336104	865	referral	No	28/09/2017	0	0	1
2015358358	865	referral	No	28/09/2017	0	0	1
2015349613	865	referral	No	28/09/2017	0	0	1
2015302306	865	referral	No	28/09/2017	0	0	1
2015358363	865	referral	No	28/09/2017	0	0	1
2015358364	865	referral	No	28/09/2017	0	0	1
2015302159	865	referral	No	28/09/2017	0	0	1
2015358368	865	referral	No	28/09/2017	0	0	1
2015302158	865	referral	No	28/09/2017	0	0	1
2015358365	865	referral	No	28/09/2017	0	0	1
2015358366	865	referral	No	28/09/2017	0	0	1
2015358367	865	referral	No	28/09/2017	0	0	1
2015347356	865	referral	No	28/09/2017	0	0	1
2015347357	865	referral	No	28/09/2017	0	0	1
2015347359	865	referral	No	28/09/2017	0	0	1
2015347358	865	referral	No	28/09/2017	0	0	1
2015347360	865	referral	No	28/09/2017	0	0	1
2015362014	865	referral	No	28/09/2017	0	0	1
2013272765	865	referral	No	26/09/2017	0	0	1
2015347405	865	referral	No	26/09/2017	0	0	1
2015336669	865	referral	No	26/09/2017	0	0	1
2014366077	865	referral	No	26/09/2017	0	0	1
2012362578	865	referral	No	26/09/2017	0	0	1
2014307422	865	referral	No	26/09/2017	0	0	1
2014366076	865	referral	No	26/09/2017	0	0	1
2015376216	865	referral	No	26/09/2017	0	0	1
2012363994	865	referral	No	26/09/2017	0	0	1
2012363996	865	referral	No	26/09/2017	0	0	1
2012363995	865	referral	No	26/09/2017	0	0	1
2015362012	865	referral	No	28/09/2017	0	0	1
2015355156	865	referral	No	28/09/2017	0	0	1
2011168527	865	referral	No	28/09/2017	0	0	1
2015379510	865	referral	No	28/09/2017	0	0	1
2015377192	865	referral	No	28/09/2017	0	0	1
2015415674	865	referral	No	28/09/2017	0	0	1
2015355934	865	referral	No	28/09/2017	0	0	1
2015415675	865	referral	No	28/09/2017	0	0	1
2015355933	865	referral	No	28/09/2017	0	0	1
2015415676	865	referral	No	28/09/2017	0	0	1
2015355932	865	referral	No	28/09/2017	0	0	1
2015415677	865	referral	No	28/09/2017	0	0	1
2015360106	865	referral	No	28/09/2017	0	0	1
2015305846	865	referral	No	28/09/2017	0	0	1
2015360107	865	referral	No	28/09/2017	0	0	1
2015360108	865	referral	No	28/09/2017	0	0	1
2015305847	865	referral	No	28/09/2017	0	0	1
2015305849	865	referral	No	28/09/2017	0	0	1
2015305848	865	referral	No	28/09/2017	0	0	1
2015360110	865	referral	No	28/09/2017	0	0	1
2015368210	865	referral	No	28/09/2017	0	0	1
2015368211	865	referral	No	28/09/2017	0	0	1
2015368212	865	referral	No	28/09/2017	0	0	1
2014375608	865	referral	No	26/09/2017	0	0	1
2014375608	865	referral	No	26/09/2017	0	0	1
2012291398	865	referral	No	26/09/2017	0	0	1
2012291398	865	referral	No	26/09/2017	0	0	1
2015331186	865	referral	No	26/09/2017	0	0	1
2015331186	865	referral	No	26/09/2017	0	0	1
2015331187	865	referral	No	26/09/2017	0	0	1
2015331187	865	referral	No	26/09/2017	0	0	1
2015376854	865	referral	No	26/09/2017	0	0	1
2015376854	865	referral	No	26/09/2017	0	0	1

2015347209	865	referral	No	26/09/2017	0	0	1
2015347209	865	referral	No	26/09/2017	0	0	1
2015360101	865	referral	No	28/09/2017	0	0	1
2014298911	865	referral	No	28/09/2017	0	0	1
2012312176	865	referral	No	28/09/2017	0	0	1
2012312177	865	referral	No	28/09/2017	0	0	1
2012312178	865	referral	No	28/09/2017	0	0	1
2011155246/D	865	referral	No	28/09/2017	0	0	1
2014326094	865	referral	No	28/09/2017	0	0	1
2014326098	865	referral	No	28/09/2017	0	0	1
2012363423	865	referral	No	28/09/2017	0	0	1
2015412787	865	referral	No	28/09/2017	0	0	1
2012363424	865	referral	No	29/09/2017	0	0	1
2012363425	865	referral	No	28/09/2017	0	0	1
2012363427	865	referral	No	28/09/2017	0	0	1
2012363428	865	referral	No	28/09/2017	0	0	1
2012363429	865	referral	No	28/09/2017	0	0	1
2012312173	865	referral	No	28/09/2017	0	0	1
2012312172	865	referral	No	28/09/2017	0	0	1
2012312170	865	referral	No	28/09/2017	0	0	1
2012312174	865	referral	No	28/09/2017	0	0	1
2014326097	865	referral	No	28/09/2017	0	0	1
2012363430	865	referral	No	29/09/2017	0	0	1
2012363431	865	referral	No	28/09/2017	0	0	1
2012376648	865	referral	No	28/09/2017	0	0	1
2012363432	865	referral	No	28/09/2017	0	0	1
2012312171	865	referral	No	28/09/2017	0	0	1
2012363433	865	referral	No	28/09/2017	0	0	1
2011134807	865	referral	No	28/09/2017	0	0	1
2011134808	865	referral	No	28/09/2017	0	0	1
2014350964	865	referral	No	28/09/2017	0	0	1
2014350965	865	referral	No	28/09/2017	0	0	1
2012363434	865	referral	No	28/09/2017	0	0	1
2014363105	865	referral	No	28/09/2017	0	0	1
2015321814	865	referral	No	28/09/2017	0	0	1
2014363106	865	referral	No	28/09/2017	0	0	1
2014317659	865	referral	No	28/09/2017	0	0	1
2015382647	865	referral	No	28/09/2017	0	0	1
2015382646	865	referral	No	28/09/2017	0	0	1
2015382645	865	referral	No	28/09/2017	0	0	1
2015382648	865	referral	No	28/09/2017	0	0	1
2015301510	865	referral	No	28/09/2017	0	0	1
2015345158	865	referral	No	28/09/2017	0	0	1
2015345157	865	referral	No	28/09/2017	0	0	1
2011100961	865	referral	No	28/09/2017	0	0	1
2015402056	865	referral	No	28/09/2017	0	0	1
2015402057	865	referral	No	28/09/2017	0	0	1
2015382642	865	referral	No	28/09/2017	0	0	1
2015382643	865	referral	No	28/09/2017	0	0	1
2015382644	865	referral	No	28/09/2017	0	0	1
2015406068	865	referral	No	28/09/2017	0	0	1
2015406067	865	referral	No	28/09/2017	0	0	1
2015406065	865	referral	No	28/09/2017	0	0	1
2015406066	865	referral	No	28/09/2017	0	0	1
2011141113	865	referral	No	28/09/2017	0	0	1
2011142219	865	referral	No	28/09/2017	0	0	1
2015286410	865	referral	No	28/09/2017	0	0	1
2015340730	865	referral	No	28/09/2017	0	0	1
2015340249	865	referral	No	28/09/2017	0	0	1
2011235748	865	referral	No	28/09/2017	0	0	1
2015340250	865	referral	No	28/09/2017	0	0	1
2011200281	865	referral	No	28/09/2017	0	0	1
2010099950	865	referral	No	28/09/2017	0	0	1
2012314029	865	referral	No	28/09/2017	0	0	1
2015362933	865	referral	No	28/09/2017	0	0	1
2015362932	865	referral	No	28/09/2017	0	0	1
2015415709	865	referral	No	28/09/2017	0	0	1
2015331681	865	referral	No	21/09/2017	0	0	1
2014321532	865	referral	No	28/09/2017	0	0	1
2014321531	865	referral	No	14/09/2017	0	0	1
2015302157	865	referral	No	13/09/2017	0	0	1
2011235749	865	referral	No	28/09/2017	0	0	1
2012283599	865	referral	No	28/09/2017	0	0	1
2014326096	865	referral	No	07/09/2017	0	0	1
2012312175	865	referral	No	15/09/2017	0	0	1
2015412788	865	referral	No	18/09/2017	0	0	1
2014298909	865	referral	No	13/09/2017	0	0	1
2014298912	865	referral	No	19/09/2017	0	0	1
2014298910	865	referral	No	13/09/2017	0	0	1
2014298913	865	referral	No	19/09/2017	0	0	1
2015377191	865	referral	No	19/09/2017	0	0	1
2015377190	865	referral	No	14/09/2017	0	0	1

2015362013	865 referral	No	28/09/2017	0	0	1
2015358361	865 referral	No	28/09/2017	0	0	1
2015358362	865 referral	No	28/09/2017	0	0	1
2015369946/D	865 referral	No	28/09/2017	0	0	1
2015369947	865 referral	No	28/09/2017	0	0	1
2015362015	865 referral	No	28/09/2017	0	0	1
2015377780	865 referral	No	03/10/2017	0	0	1
2015377781	865 referral	No	03/10/2017	0	0	1
2015405418	865 referral	No	03/10/2017	0	0	1
2015363021	865 referral	No	03/10/2017	0	0	1
2012369034	865 referral	No	03/10/2017	0	0	1
2015377782	865 referral	No	03/10/2017	0	0	1
2015413942	865 referral	No	03/10/2017	0	0	1
2014346336	865 referral	No	28/09/2017	0	0	1
2014346336	865 referral	No	28/09/2017	0	0	1
2015332916	865 referral	No	28/09/2017	0	0	1
2015332916	865 referral	No	28/09/2017	0	0	1
2015413941	865 referral	No	03/10/2017	0	0	1
2015382647	865 referral	No	28/09/2017	0	0	1
2015382647	865 referral	No	28/09/2017	0	0	1
2015289199	865 referral	No	03/10/2017	0	0	1
2012369036	865 referral	No	03/10/2017	0	0	1
2015415674	865 referral	No	28/09/2017	0	0	1
2015415674	865 referral	No	28/09/2017	0	0	1
2015403333	865 referral	No	28/09/2017	0	0	1
2015403333	865 referral	No	28/09/2017	0	0	1
2012369037	865 referral	No	03/10/2017	0	0	1
2015326968	865 referral	No	03/10/2017	0	0	1
2015376672	865 referral	No	03/10/2017	0	0	1
2015355031	865 referral	No	03/10/2017	0	0	1
2011135091	865 referral	No	03/10/2017	0	0	1
2015401707	865 referral	No	03/10/2017	0	0	1
2015303192	865 referral	No	03/10/2017	0	0	1
2012335671	865 referral	No	03/10/2017	0	0	1
2012335672	865 referral	No	03/10/2017	0	0	1
2014363233	865 referral	No	03/10/2017	0	0	1
2015344493	865 referral	No	03/10/2017	0	0	1
2015344494	865 referral	No	03/10/2017	0	0	1
2011100962	865 referral	No	03/10/2017	0	0	1
2015345159	865 referral	No	03/10/2017	0	0	1
2015336671	865 referral	No	03/10/2017	0	0	1
2015336670	865 referral	No	03/10/2017	0	0	1
2015345160	865 referral	No	03/10/2017	0	0	1
2015336672	865 referral	No	03/10/2017	0	0	1
2015345161	865 referral	No	03/10/2017	0	0	1
2015336673	865 referral	No	03/10/2017	0	0	1
2015345162	865 referral	No	03/10/2017	0	0	1
2012363997	865 referral	No	03/10/2017	0	0	1
2015377777	865 referral	No	03/10/2017	0	0	1
2015401706	865 referral	No	03/10/2017	0	0	1
2015377778	865 referral	No	03/10/2017	0	0	1
2012362581	865 referral	No	03/10/2017	0	0	1
2015377779	865 referral	No	03/10/2017	0	0	1
2011117882	865 referral	No	03/10/2017	0	0	1
2011117883	865 referral	No	03/10/2017	0	0	1
2017pari747223	865 referral	No	03/10/2017	0	0	1
2012362580	865 referral	No	03/10/2017	0	0	1
2011117884	865 referral	No	03/10/2017	0	0	1
2011117885	865 referral	No	03/10/2017	0	0	1
2012362579	865 referral	No	03/10/2017	0	0	1
2015364989	865 referral	No	03/10/2017	0	0	1
2014378742	865 referral	No	02/10/2017	0	0	1
2014307421	865 referral	No	03/10/2017	0	0	1
2015364990	865 referral	No	03/10/2017	0	0	1
2014375615	865 referral	No	03/10/2017	0	0	1
2015364991	865 referral	No	03/10/2017	0	0	1
2015368305	865 referral	No	02/10/2017	0	0	1
2015369948	865 referral	No	03/10/2017	0	0	1
2015344495	865 referral	No	03/10/2017	0	0	1
2013254149	865 referral	No	02/10/2017	0	0	1
2015344489	865 referral	No	03/10/2017	0	0	1
20170218951	865 referral	No	03/10/2017	0	0	1
2015287805	865 referral	No	02/10/2017	0	0	1
2015344490	865 referral	No	03/10/2017	0	0	1
2012371464	865 referral	No	03/10/2017	0	0	1
2011137336	865 referral	No	03/10/2017	0	0	1
2015397704	865 referral	No	02/10/2017	0	0	1
2012289792	865 referral	No	03/10/2017	0	0	1
2011137337	865 referral	No	03/10/2017	0	0	1
2015397705	865 referral	No	02/10/2017	0	0	1
2012289794	865 referral	No	03/10/2017	0	0	1
2011137338	865 referral	No	03/10/2017	0	0	1

2015397706	865	referral	No	02/10/2017	0	0	1
2015286873	865	referral	No	03/10/2017	0	0	1
2011137339	865	referral	No	03/10/2017	0	0	1
2015397707	865	referral	No	02/10/2017	0	0	1
2012289793	865	referral	No	03/10/2017	0	0	1
2011137340	865	referral	No	03/10/2017	0	0	1
2015397708	865	referral	No	02/10/2017	0	0	1
2012275996	865	referral	No	03/10/2017	0	0	1
2011137341	865	referral	No	03/10/2017	0	0	1
2015397709	865	referral	No	02/10/2017	0	0	1
2015397710	865	referral	No	02/10/2017	0	0	1
2011135695	865	referral	No	03/10/2017	0	0	1
2012294637	865	referral	No	03/10/2017	0	0	1
2011135696	865	referral	No	03/10/2017	0	0	1
2011135092	865	referral	No	03/10/2017	0	0	1
2015397711	865	referral	No	02/10/2017	0	0	1
2011135693	865	referral	No	03/10/2017	0	0	1
2015336675	865	referral	No	03/10/2017	0	0	1
2015397712	865	referral	No	02/10/2017	0	0	1
2011135694	865	referral	No	03/10/2017	0	0	1
2015336674	865	referral	No	03/10/2017	0	0	1
2015360103	865	referral	No	02/10/2017	0	0	1
2015294632	865	referral	No	03/10/2017	0	0	1
2015336676	865	referral	No	03/10/2017	0	0	1
2014358283	865	referral	No	02/10/2017	0	0	1
2011135093	865	referral	No	03/10/2017	0	0	1
2015294634	865	referral	No	03/10/2017	0	0	1
2015360102	865	referral	No	02/10/2017	0	0	1
2012294632	865	referral	No	03/10/2017	0	0	1
2012351702	865	referral	No	02/10/2017	0	0	1
2015294635	865	referral	No	03/10/2017	0	0	1
2013267900	865	referral	No	02/10/2017	0	0	1
201075916	865	referral	No	03/10/2017	0	0	1
2015415204	865	referral	No	02/10/2017	0	0	1
2015334824	865	referral	No	03/10/2017	0	0	1
2015415920	865	referral	No	02/10/2017	0	0	1
2015334825	865	referral	No	03/10/2017	0	0	1
2015408115	865	referral	No	02/10/2017	0	0	1
2011136264	865	referral	No	03/10/2017	0	0	1
2015360104	865	referral	No	02/10/2017	0	0	1
2012294631	865	referral	No	03/10/2017	0	0	1
2012294629	865	referral	No	03/10/2017	0	0	1
2011140556	865	referral	No	03/10/2017	0	0	1
2012294636	865	referral	No	03/10/2017	0	0	1
2015368304	865	referral	No	02/10/2017	0	0	1
2015344491	865	referral	No	03/10/2017	0	0	1
2013266079	865	referral	No	03/10/2017	0	0	1
2014319639	865	referral	No	03/10/2017	0	0	1
2015344492	865	referral	No	03/10/2017	0	0	1
2015362924	865	referral	No	25/08/2017	0	0	1
2015360748	865	referral	No	03/10/2017	0	0	1
2015369092	865	referral	No	31/08/2017	0	0	1
2015360955	865	referral	No	03/10/2017	0	0	1
2014312415	865	referral	No	19/07/2017	0	0	1
2012359174	865	referral	No	03/10/2017	0	0	1
2012341048	865	referral	No	15/09/2017	0	0	1
2012291395	865	referral	No	03/10/2017	0	0	1
2015337390	21	rural/clinic	No	19/09/2017	0	0	0
2015313502	21	rural/clinic	No	03/10/2017	0	0	0
2014327537	21	rural/clinic	No	03/10/2017	0	0	0
2015400310	21	rural/clinic	No	03/10/2017	0	0	0
2014327538	21	rural/clinic	No	03/10/2017	0	0	0
2015400309	21	rural/clinic	No	03/10/2017	0	0	0
2015400308	21	rural/clinic	No	03/10/2017	0	0	0
2014327539	21	rural/clinic	No	03/10/2017	0	0	0
2015347406	21	rural/clinic	No	03/10/2017	0	0	0
2014327540	21	rural/clinic	No	03/10/2017	0	0	0
2012379924	21	rural/clinic	No	03/10/2017	0	0	0
2015419088	21	rural/clinic	No	18/09/2017	0	0	0
2012304346	21	rural/clinic	No	03/10/2017	0	0	0
2012379926	21	rural/clinic	No	03/10/2017	0	0	0
2014326095	21	rural/clinic	No	19/09/2017	0	0	0
2012317326	21	rural/clinic	No	03/10/2017	0	0	0
2015335983	21	rural/clinic	No	09/09/2017	0	0	0
2012379923	21	rural/clinic	No	03/10/2017	0	0	0
2012379921	21	rural/clinic	No	03/10/2017	0	0	0
2012379922	21	rural/clinic	No	03/10/2017	0	0	0
2017father ohea	21	rural/clinic	No	17/09/2017	0	0	0
2015332856	21	rural/clinic	No	03/10/2017	0	0	0
2013256707	21	rural/clinic	No	03/10/2017	0	0	0
2017mberengwa2	21	rural/clinic	No	07/09/2017	0	0	0
2013256706	21	rural/clinic	No	03/10/2017	0	0	0

2012294635	21	rural/clinic	No	03/10/2017	0	0	0
2013252936	21	rural/clinic	No	19/09/2017	0	0	0
2015355034	21	rural/clinic	No	03/10/2017	0	0	0
2012242593	21	rural/clinic	No	03/10/2017	0	0	0
2015355033	21	rural/clinic	No	03/10/2017	0	0	0
2012242594	21	rural/clinic	No	03/10/2017	0	0	0
2015376215	21	rural/clinic	No	03/10/2017	0	0	0
2015338471	21	rural/clinic	No	03/10/2017	0	0	0
2015338472	21	rural/clinic	No	21/09/2017	0	0	0
2014361230	21	rural/clinic	No	03/10/2017	0	0	0
2014371170	21	rural/clinic	No	20/09/2017	0	0	0
2014358234	21	rural/clinic	No	03/10/2017	0	0	0
2011133928	21	rural/clinic	No	03/10/2017	0	0	0
2012344512	21	rural/clinic	No	03/10/2017	0	0	0
2011133927	21	rural/clinic	No	03/10/2017	0	0	0
2011221341	21	rural/clinic	No	03/10/2017	0	0	0
2015418763	21	rural/clinic	No	03/10/2017	0	0	0
2014360976	21	rural/clinic	No	03/10/2017	0	0	0
2012244295	21	rural/clinic	No	03/10/2017	0	0	0
2015355032	21	rural/clinic	No	03/10/2017	0	0	0
2014361231	21	rural/clinic	No	03/10/2017	0	0	0
2014361232	21	rural/clinic	No	03/10/2017	0	0	0
2014358040	21	rural/clinic	No	03/10/2017	0	0	0
2014358039	21	rural/clinic	No	03/10/2017	0	0	0
2014326643	21	rural/clinic	No	03/10/2017	0	0	0
2012293901	21	rural/clinic	No	03/10/2017	0	0	0
2014326650	21	rural/clinic	No	03/10/2017	0	0	0
2012252103	21	rural/clinic	No	03/10/2017	0	0	0
2015325258	21	rural/clinic	No	03/10/2017	0	0	0
2014319215	21	rural/clinic	No	03/10/2017	0	0	0
2015325250	21	rural/clinic	No	03/10/2017	0	0	0
2014319216	21	rural/clinic	No	03/10/2017	0	0	0
2012252104	21	rural/clinic	No	03/10/2017	0	0	0
2015325249	21	rural/clinic	No	03/10/2017	0	0	0
2015357309	21	rural/clinic	No	03/10/2017	0	0	0
2015325192	21	rural/clinic	No	03/10/2017	0	0	0
2015325191	21	rural/clinic	No	03/10/2017	0	0	0
2015325248	21	rural/clinic	No	03/10/2017	0	0	0
2015325247	21	rural/clinic	No	03/10/2017	0	0	0
2015340941	21	rural/clinic	No	03/10/2017	0	0	0
2014319144	21	rural/clinic	No	03/10/2017	0	0	0
2012312212	21	rural/clinic	No	03/10/2017	0	0	0
2015325259	21	rural/clinic	No	03/10/2017	0	0	0
2014309594	557	rural/clinic	No	03/10/2017	0	0	0
2015335173	557	rural/clinic	No	03/10/2017	0	0	0
2015335174	557	rural/clinic	No	03/10/2017	0	0	0
2015335175	557	rural/clinic	No	03/10/2017	0	0	0
2015335176	557	rural/clinic	No	03/10/2017	0	0	0
2015335177	557	rural/clinic	No	03/10/2017	0	0	0
2015335178	557	rural/clinic	No	03/10/2017	0	0	0
2015335179	557	rural/clinic	No	03/10/2017	0	0	0
2015335180	557	rural/clinic	No	03/10/2017	0	0	0
2015335181	557	rural/clinic	No	03/10/2017	0	0	0
2015335182	557	rural/clinic	No	03/10/2017	0	0	0
2015335183	557	rural/clinic	No	03/10/2017	0	0	0
2015335184	557	rural/clinic	No	03/10/2017	0	0	0
2015335185	557	rural/clinic	No	03/10/2017	0	0	0
2015335186	557	rural/clinic	No	03/10/2017	0	0	0
2015335187	557	rural/clinic	No	03/10/2017	0	0	0
2015335188	557	rural/clinic	No	03/10/2017	0	0	0
2015335189	557	rural/clinic	No	03/10/2017	0	0	0
2015335190	557	rural/clinic	No	03/10/2017	0	0	0
2015335191	557	rural/clinic	No	03/10/2017	0	0	0
2015335192	557	rural/clinic	No	03/10/2017	0	0	0
2015335193	557	rural/clinic	No	03/10/2017	0	0	0
2015335194	557	rural/clinic	No	03/10/2017	0	0	0
2015335195	557	rural/clinic	No	03/10/2017	0	0	0
2015335196	557	rural/clinic	No	03/10/2017	0	0	0
2015335197	557	rural/clinic	No	03/10/2017	0	0	0
2015335198	557	rural/clinic	No	03/10/2017	0	0	0
2015335199	557	rural/clinic	No	03/10/2017	0	0	0
2015335200	557	rural/clinic	No	03/10/2017	0	0	0
2015335201	557	rural/clinic	No	03/10/2017	0	0	0
2015335202	557	rural/clinic	No	03/10/2017	0	0	0
2015335203	557	rural/clinic	No	03/10/2017	0	0	0
2015335204	557	rural/clinic	No	03/10/2017	0	0	0
2015335205	557	rural/clinic	No	03/10/2017	0	0	0
2015335206	557	rural/clinic	No	03/10/2017	0	0	0
2015335207	557	rural/clinic	No	03/10/2017	0	0	0
2015335208	557	rural/clinic	No	03/10/2017	0	0	0
2015335209	557	rural/clinic	No	03/10/2017	0	0	0
2015335210	557	rural/clinic	No	03/10/2017	0	0	0

2015335211	557	rural/clinic	No	03/10/2017	0	0	0
2015335212	557	rural/clinic	No	03/10/2017	0	0	0
2015335213	557	rural/clinic	No	03/10/2017	0	0	0
2015335214	557	rural/clinic	No	03/10/2017	0	0	0
2015335215	557	rural/clinic	No	03/10/2017	0	0	0
2015335216	557	rural/clinic	No	03/10/2017	0	0	0
2015335217	557	rural/clinic	No	03/10/2017	0	0	0
2015335218	557	rural/clinic	No	03/10/2017	0	0	0
2015335219	557	rural/clinic	No	03/10/2017	0	0	0
2015335220	557	rural/clinic	No	03/10/2017	0	0	0
2015335221	557	rural/clinic	No	03/10/2017	0	0	0
2015335222	557	rural/clinic	No	03/10/2017	0	0	0
2015335223	557	rural/clinic	No	03/10/2017	0	0	0
2015335224	557	rural/clinic	No	03/10/2017	0	0	0
2015335225	557	rural/clinic	No	03/10/2017	0	0	0
2015335226	557	rural/clinic	No	03/10/2017	0	0	0
2015335227	557	rural/clinic	No	03/10/2017	0	0	0
2015335228	557	rural/clinic	No	03/10/2017	0	0	0
2015335229	557	rural/clinic	No	03/10/2017	0	0	0
2015335230	557	rural/clinic	No	03/10/2017	0	0	0
2015335231	557	rural/clinic	No	03/10/2017	0	0	0
2015335232	557	rural/clinic	No	03/10/2017	0	0	0
2015335233	557	rural/clinic	No	03/10/2017	0	0	0
2015335234	557	rural/clinic	No	03/10/2017	0	0	0
2015335235	557	rural/clinic	No	03/10/2017	0	0	0
2015335236	557	rural/clinic	No	03/10/2017	0	0	0
2015335237	557	rural/clinic	No	03/10/2017	0	0	0
2015335238	557	rural/clinic	No	03/10/2017	0	0	0
2015335239	557	rural/clinic	No	03/10/2017	0	0	0
2015335240	557	rural/clinic	No	03/10/2017	0	0	0
2015335241	557	rural/clinic	No	03/10/2017	0	0	0
2015335242	557	rural/clinic	No	03/10/2017	0	0	0
2015335243	557	rural/clinic	No	03/10/2017	0	0	0
2015335244	557	rural/clinic	No	03/10/2017	0	0	0
2015335245	557	rural/clinic	No	03/10/2017	0	0	0
2015335246	557	rural/clinic	No	03/10/2017	0	0	0
2015335247	557	rural/clinic	No	03/10/2017	0	0	0
2015335248	557	rural/clinic	No	03/10/2017	0	0	0
2015335249	557	rural/clinic	No	03/10/2017	0	0	0
2015335250	557	rural/clinic	No	03/10/2017	0	0	0
2015335251	557	rural/clinic	No	03/10/2017	0	0	0
2015335252	557	rural/clinic	No	03/10/2017	0	0	0
2015335253	557	rural/clinic	No	03/10/2017	0	0	0
2015335254	557	rural/clinic	No	03/10/2017	0	0	0
2015335255	557	rural/clinic	No	03/10/2017	0	0	0
2015335256	557	rural/clinic	No	03/10/2017	0	0	0
2015335257	557	rural/clinic	No	03/10/2017	0	0	0
2015335258	557	rural/clinic	No	03/10/2017	0	0	0
2015335259	557	rural/clinic	No	03/10/2017	0	0	0
2015335260	557	rural/clinic	No	03/10/2017	0	0	0
2015335261	557	rural/clinic	No	03/10/2017	0	0	0
2015335262	557	rural/clinic	No	03/10/2017	0	0	0
2015335263	557	rural/clinic	No	03/10/2017	0	0	0
2015335264	557	rural/clinic	No	03/10/2017	0	0	0
2015335265	557	rural/clinic	No	03/10/2017	0	0	0
2015335266	557	rural/clinic	No	03/10/2017	0	0	0
2015335267	557	rural/clinic	No	03/10/2017	0	0	0
2015335268	557	rural/clinic	No	03/10/2017	0	0	0
2015335269	557	rural/clinic	No	03/10/2017	0	0	0
2015335270	557	rural/clinic	No	03/10/2017	0	0	0
2015335271	557	rural/clinic	No	03/10/2017	0	0	0
2015335272	557	rural/clinic	No	03/10/2017	0	0	0
2015335273	557	rural/clinic	No	03/10/2017	0	0	0
2015335274	557	rural/clinic	No	03/10/2017	0	0	0
2015335275	557	rural/clinic	No	03/10/2017	0	0	0
2015335276	557	rural/clinic	No	03/10/2017	0	0	0
2015335277	557	rural/clinic	No	03/10/2017	0	0	0
2015335278	558	rural/clinic	No	03/10/2017	0	0	0
2015335279	558	rural/clinic	No	03/10/2017	0	0	0
2015335280	558	rural/clinic	No	03/10/2017	0	0	0
2015335281	558	rural/clinic	No	03/10/2017	0	0	0
2015335282	168	rural/clinic	No	03/10/2017	0	0	0
2015335283	168	rural/clinic	No	03/10/2017	0	0	0
2015335284	168	rural/clinic	No	03/10/2017	0	0	0
2015335285	168	rural/clinic	No	03/10/2017	0	0	0
2015335286	168	rural/clinic	No	03/10/2017	0	0	0
2015335287	168	rural/clinic	No	03/10/2017	0	0	0
2015335288	168	rural/clinic	No	03/10/2017	0	0	0
2015335289	168	rural/clinic	No	03/10/2017	0	0	0
2015335290	168	rural/clinic	No	03/10/2017	0	0	0
2015335291	168	rural/clinic	No	03/10/2017	0	0	0
2015335292	168	rural/clinic	No	03/10/2017	0	0	0

2015335293	168	rural/clinic	No	03/10/2017	0	0	0
2015335294	168	rural/clinic	No	03/10/2017	0	0	0
2015335295	168	rural/clinic	No	03/10/2017	0	0	0
2015335296	168	rural/clinic	No	03/10/2017	0	0	0
2015335297	168	rural/clinic	No	03/10/2017	0	0	0
2015335298	168	rural/clinic	No	03/10/2017	0	0	0
2015335299	168	rural/clinic	No	03/10/2017	0	0	0
2015335300	168	rural/clinic	No	03/10/2017	0	0	0
2015335301	168	rural/clinic	No	03/10/2017	0	0	0
2015335302	168	rural/clinic	No	03/10/2017	0	0	0
2015335303	168	rural/clinic	No	03/10/2017	0	0	0
2015335304	168	rural/clinic	No	03/10/2017	0	0	0
2015335305	168	rural/clinic	No	03/10/2017	0	0	0
2015335306	168	rural/clinic	No	03/10/2017	0	0	0
2015335307	168	rural/clinic	No	28/09/2017	0	0	0
2015335308	168	rural/clinic	No	28/09/2017	0	0	0
2015335309	436	rural/clinic	No	03/10/2017	0	0	0
2015335310	436	rural/clinic	No	03/10/2017	0	0	0
2015335311	436	rural/clinic	No	03/10/2017	0	0	0
2015335312	436	rural/clinic	No	03/10/2017	0	0	0
2015335313	436	rural/clinic	No	03/10/2017	0	0	0
2015335314	436	rural/clinic	No	03/10/2017	0	0	0
2015335315	436	rural/clinic	No	03/10/2017	0	0	0
2015335316	436	rural/clinic	No	03/10/2017	0	0	0
2015335317	436	rural/clinic	No	03/10/2017	0	0	0
2015335318	436	rural/clinic	No	03/10/2017	0	0	0
2015335319	436	rural/clinic	No	03/10/2017	0	0	0
2015335320	436	rural/clinic	No	03/10/2017	0	0	0
2015335321	436	rural/clinic	No	03/10/2017	0	0	0
2015335322	436	rural/clinic	No	26/09/2017	0	0	0
2015335323	436	rural/clinic	No	29/09/2017	0	0	0
2015335324	436	rural/clinic	No	26/09/2017	0	0	0
2015335325	436	rural/clinic	No	26/09/2017	0	0	0
2015335326	436	rural/clinic	No	26/09/2017	0	0	0
2015335327	436	rural/clinic	No	28/09/2017	0	0	0
2015335328	436	rural/clinic	No	28/09/2017	0	0	0
2015335329	436	rural/clinic	No	05/10/2017	0	0	0
2015335330	436	rural/clinic	No	05/10/2017	0	0	0
2015335331	436	rural/clinic	No	03/10/2017	0	0	0
2015335332	436	rural/clinic	No	03/10/2017	0	0	0
2015335333	940	rural/clinic	No	28/09/2017	0	0	0
2015335334	940	rural/clinic	No	05/10/2017	0	0	0
2015335335	940	rural/clinic	No	05/10/2017	0	0	0
2015335336	940	rural/clinic	No	05/10/2017	0	0	0
2015335337	940	rural/clinic	No	05/10/2017	0	0	0
2015335338	940	rural/clinic	No	05/10/2017	0	0	0
2015335339	940	rural/clinic	No	05/10/2017	0	0	0
2015335340	940	rural/clinic	No	05/10/2017	0	0	0
2015335341	940	rural/clinic	No	05/10/2017	0	0	0
2015335342	940	rural/clinic	No	05/10/2017	0	0	0
2015335343	940	rural/clinic	No	05/10/2017	0	0	0
2015335344	940	rural/clinic	No	05/10/2017	0	0	0
2015335345	940	rural/clinic	No	05/10/2017	0	0	0
2015335346	940	rural/clinic	No	05/10/2017	0	0	0
2015335347	940	rural/clinic	No	05/10/2017	0	0	0
2015335348	940	rural/clinic	No	05/10/2017	0	0	0
2015335349	940	rural/clinic	No	05/10/2017	0	0	0
2015335350	940	rural/clinic	No	05/10/2017	0	0	0
2015335351	940	rural/clinic	No	05/10/2017	0	0	0
2015335352	940	rural/clinic	No	05/10/2017	0	0	0
2015335353	940	rural/clinic	No	05/10/2017	0	0	0
2015335354	940	rural/clinic	No	05/10/2017	0	0	0
2015335355	940	rural/clinic	No	05/10/2017	0	0	0
2015335356	940	rural/clinic	No	05/10/2017	0	0	0
2015335357	940	rural/clinic	No	05/10/2017	0	0	0
2015335358	940	rural/clinic	No	05/10/2017	0	0	0
2015335359	940	rural/clinic	No	05/10/2017	0	0	0
2015335360	940	rural/clinic	No	05/10/2017	0	0	0
2015335361	940	rural/clinic	No	05/10/2017	0	0	0
2015335362	940	rural/clinic	No	06/10/2017	0	0	0
2015335363	940	rural/clinic	No	05/10/2017	0	0	0
2015335364	940	rural/clinic	No	05/10/2017	0	0	0
2015335365	940	rural/clinic	No	05/10/2017	0	0	0
2015335366	940	rural/clinic	No	05/10/2017	0	0	0
2015335367	940	rural/clinic	No	05/10/2017	0	0	0
2015335368	940	rural/clinic	No	05/10/2017	0	0	0
2015335369	940	rural/clinic	No	05/10/2017	0	0	0
2015335370	940	rural/clinic	No	05/10/2017	0	0	0
2015335371	940	rural/clinic	No	05/10/2017	0	0	0
2015335372	940	rural/clinic	No	05/10/2017	0	0	0
2015335373	940	rural/clinic	No	05/10/2017	0	0	0
2015335374	940	rural/clinic	No	05/10/2017	0	0	0

2015335375	940	rural/clinic	No	05/10/2017	0	0	0
2015335376	940	rural/clinic	No	05/10/2017	0	0	0
2015335377	330	rural/clinic	No	05/10/2017	0	0	0
2015335378	330	rural/clinic	No	05/10/2017	0	0	0
2015335379	330	rural/clinic	No	05/10/2017	0	0	0
2015335380	330	rural/clinic	No	05/10/2017	0	0	0
2015335381	330	rural/clinic	No	05/10/2017	0	0	0
2015335382	330	rural/clinic	No	05/10/2017	0	0	0
2015335383	330	rural/clinic	No	05/10/2017	0	0	0
2015335384	330	rural/clinic	No	05/10/2017	0	0	0
2015335385	330	rural/clinic	No	05/10/2017	0	0	0
2015335386	330	rural/clinic	No	05/10/2017	0	0	0
2015335387	330	rural/clinic	No	05/10/2017	0	0	0
2015335388	330	rural/clinic	No	05/10/2017	0	0	0
2015335389	220	rural/clinic	No	05/10/2017	0	0	0
2015335390	220	rural/clinic	No	05/10/2017	0	0	0
2015335391	220	rural/clinic	No	05/10/2017	0	0	0
2015335392	220	rural/clinic	No	05/10/2017	0	0	0
2015335393	220	rural/clinic	No	05/10/2017	0	0	0
2015335394	220	rural/clinic	No	05/10/2017	0	0	0
2015335395	220	rural/clinic	No	05/10/2017	0	0	0
2015335396	220	rural/clinic	No	05/10/2017	0	0	0
2015335397	220	rural/clinic	No	05/10/2017	0	0	0
2015335398	220	rural/clinic	No	05/10/2017	0	0	0
2015335399	220	rural/clinic	No	05/10/2017	0	0	0
2015335400	220	rural/clinic	No	05/10/2017	0	0	0
2015335401	220	rural/clinic	No	05/10/2017	0	0	0
2015335402	220	rural/clinic	No	05/10/2017	0	0	0
2015335403	220	rural/clinic	No	05/10/2017	0	0	0
2015335404	220	rural/clinic	No	05/10/2017	0	0	0
2015335405	220	rural/clinic	No	05/10/2017	0	0	0
2015335406	220	rural/clinic	No	05/10/2017	0	0	0
2015335407	220	rural/clinic	No	05/10/2017	0	0	0
2015335408	220	rural/clinic	No	05/10/2017	0	0	0
2015335409	220	rural/clinic	No	05/10/2017	0	0	0
2015335410	514	rural/clinic	No	05/10/2017	0	0	0
2015378225	514	rural/clinic	No	05/10/2017	0	0	0
2015294898	514	rural/clinic	No	05/10/2017	0	0	0
2015382777	514	rural/clinic	No	05/10/2017	0	0	0
2015382776	514	rural/clinic	No	05/10/2017	0	0	0
2014363661	514	rural/clinic	No	05/10/2017	0	0	0
2015382775	514	rural/clinic	No	05/10/2017	0	0	0
2015337851	514	rural/clinic	No	05/10/2017	0	0	0
2015382774	514	rural/clinic	No	05/10/2017	0	0	0
2015362940	514	rural/clinic	No	05/10/2017	0	0	0
2015355750	514	rural/clinic	No	05/10/2017	0	0	0
2015382773	514	rural/clinic	No	05/10/2017	0	0	0
2015362939	514	rural/clinic	No	05/10/2017	0	0	0
2015377445	514	rural/clinic	No	05/10/2017	0	0	0
2015377446	514	rural/clinic	No	05/10/2017	0	0	0
2015355788	514	rural/clinic	No	05/10/2017	0	0	0
2015362937	514	rural/clinic	No	05/10/2017	0	0	0
2015377447	514	rural/clinic	No	05/10/2017	0	0	0
2015362936	514	rural/clinic	No	05/10/2017	0	0	0
2011100963	514	rural/clinic	No	05/10/2017	0	0	0
2015377448	514	rural/clinic	No	05/10/2017	0	0	0
2015384370	514	rural/clinic	No	05/10/2017	0	0	0
2015362935	514	rural/clinic	No	05/10/2017	0	0	0
2011100964	514	rural/clinic	No	05/10/2017	0	0	0
2015303374	514	rural/clinic	No	05/10/2017	0	0	0
2015362934	514	rural/clinic	No	05/10/2017	0	0	0
2011100965	514	rural/clinic	No	05/10/2017	0	0	0
2015403751	514	rural/clinic	No	05/10/2017	0	0	0
2015362938	514	rural/clinic	No	05/10/2017	0	0	0
2015403752	514	rural/clinic	No	05/10/2017	0	0	0
2015362837	514	rural/clinic	No	05/10/2017	0	0	0
2015403753	252	rural/clinic	No	05/10/2017	0	0	0
2015347361	252	rural/clinic	No	05/10/2017	0	0	0
2015286415	252	rural/clinic	No	05/10/2017	0	0	0
2015403754	252	rural/clinic	No	05/10/2017	0	0	0
2015286416	252	rural/clinic	No	05/10/2017	0	0	0
2015347362	252	rural/clinic	No	05/10/2017	0	0	0
2015403755	252	rural/clinic	No	05/10/2017	0	0	0
2015347363	252	rural/clinic	No	05/10/2017	0	0	0
2015286411	252	rural/clinic	No	05/10/2017	0	0	0
2015347364	252	rural/clinic	No	05/10/2017	0	0	0
2015286413	252	rural/clinic	No	05/10/2017	0	0	0
2015347365	252	rural/clinic	No	05/10/2017	0	0	0
2011115102	252	rural/clinic	No	05/10/2017	0	0	0
2014304707	252	rural/clinic	No	05/10/2017	0	0	0
2015355157	252	rural/clinic	No	05/10/2017	0	0	0
2015337801	252	rural/clinic	No	31/07/2017	0	0	0

2014304708	252	rural/clinic	No	05/10/2017	0	0	0
2014304709	252	rural/clinic	No	05/10/2017	0	0	0
2014309396	252	rural/clinic	No	05/10/2017	0	0	0
2014300305	252	rural/clinic	No	05/10/2017	0	0	0
2011168528	252	rural/clinic	No	05/10/2017	0	0	0
2014304710	252	rural/clinic	No	05/10/2017	0	0	0
2015406423	252	rural/clinic	No	05/10/2017	0	0	0
2015377337	252	rural/clinic	No	05/10/2017	0	0	0
2015406424	252	rural/clinic	No	05/10/2017	0	0	0
2014304711	252	rural/clinic	No	05/10/2017	0	0	0
2015406425	252	rural/clinic	No	05/10/2017	0	0	0
2015406069	252	rural/clinic	No	05/10/2017	0	0	0
2012265645	252	rural/clinic	No	05/10/2017	0	0	0
2015406426	252	rural/clinic	No	05/10/2017	0	0	0
2014301926	252	rural/clinic	No	05/10/2017	0	0	0
2015377338	252	rural/clinic	No	05/10/2017	0	0	0
2014334192	252	rural/clinic	No	05/10/2017	0	0	0
2015372833	252	rural/clinic	No	05/10/2017	0	0	0
2014300301	252	rural/clinic	No	05/10/2017	0	0	0
2015372834	252	rural/clinic	No	05/10/2017	0	0	0
2015305850	252	rural/clinic	No	05/10/2017	0	0	0
2015401185	252	rural/clinic	No	05/10/2017	0	0	0
2014300307	252	rural/clinic	No	05/10/2017	0	0	0
2015401186	252	rural/clinic	No	05/10/2017	0	0	0
2014300302	252	rural/clinic	No	05/10/2017	0	0	0
2014345758	252	rural/clinic	No	05/10/2017	0	0	0
2014300303	252	rural/clinic	No	05/10/2017	0	0	0
2014345759	252	rural/clinic	No	05/10/2017	0	0	0
2014345760	252	rural/clinic	No	05/10/2017	0	0	0
2014300304	252	rural/clinic	No	05/10/2017	0	0	0
2014345761	252	rural/clinic	No	05/10/2017	0	0	0
2014345903	252	rural/clinic	No	05/10/2017	0	0	0
2014300306	252	rural/clinic	No	05/10/2017	0	0	0
2015404184	252	rural/clinic	No	05/10/2017	0	0	0
2015324140	252	rural/clinic	No	05/10/2017	0	0	0
2014345904	252	rural/clinic	No	05/10/2017	0	0	0
2014345905	252	rural/clinic	No	05/10/2017	0	0	0
2014368492	252	rural/clinic	No	05/10/2017	0	0	0
2014345906	252	rural/clinic	No	05/10/2017	0	0	0
2014345907	252	rural/clinic	No	05/10/2017	0	0	0
2015406427	252	rural/clinic	No	05/10/2017	0	0	0
2015287413	252	rural/clinic	No	05/10/2017	0	0	0
2015287413	252	rural/clinic	No	05/10/2017	0	0	0
2015369044	252	rural/clinic	No	28/09/2017	0	0	0
2015369044	252	rural/clinic	No	28/09/2017	0	0	0
2015417003	252	rural/clinic	No	19/09/2017	0	0	0
2015417003	252	rural/clinic	No	19/09/2017	0	0	0
2015403559	252	rural/clinic	No	05/10/2017	0	0	0
2015403559	252	rural/clinic	No	05/10/2017	0	0	0
2015355791	252	rural/clinic	No	05/10/2017	0	0	0
2015355791	252	rural/clinic	No	05/10/2017	0	0	0
2015355789	252	rural/clinic	No	05/10/2017	0	0	0
2015355789	252	rural/clinic	No	05/10/2017	0	0	0
2015352829	252	rural/clinic	No	05/10/2017	0	0	0
2015352829	252	rural/clinic	No	05/10/2017	0	0	0
2015402058	252	rural/clinic	No	05/10/2017	0	0	0
2015402058	252	rural/clinic	No	05/10/2017	0	0	0
2015377445	252	rural/clinic	No	05/10/2017	0	0	0
2015377445	252	rural/clinic	No	05/10/2017	0	0	0
2015362939	252	rural/clinic	No	05/10/2017	0	0	0
2015362939	252	rural/clinic	No	05/10/2017	0	0	0
2012284624	252	rural/clinic	No	06/10/2017	0	0	0
2012284624	252	rural/clinic	No	06/10/2017	0	0	0
2015417003	252	rural/clinic	No	19/09/2017	0	0	0
2015417003	252	rural/clinic	No	19/09/2017	0	0	0
2014304707	252	rural/clinic	No	05/10/2017	0	0	0
2014304707	252	rural/clinic	No	05/10/2017	0	0	0
2014300307	252	rural/clinic	No	05/10/2017	0	0	0
2014300307	252	rural/clinic	No	05/10/2017	0	0	0
2015402158	252	rural/clinic	No	05/10/2017	0	0	0
2015402158	252	rural/clinic	No	05/10/2017	0	0	0
2012265645	252	rural/clinic	No	05/10/2017	0	0	0
2012265645	252	rural/clinic	No	05/10/2017	0	0	0
2015362932	252	rural/clinic	No	28/09/2017	0	0	0
2015362932	252	rural/clinic	No	28/09/2017	0	0	0
2012362582	252	rural/clinic	No	10/10/2017	0	0	0
2015287414	252	rural/clinic	No	10/10/2017	0	0	0
2011135094	252	rural/clinic	No	10/10/2017	0	0	0
2011135095	252	rural/clinic	No	10/10/2017	0	0	0
2015287415	252	rural/clinic	No	10/10/2017	0	0	0
2012294633	252	rural/clinic	No	10/10/2017	0	0	0
2015397713	252	rural/clinic	No	10/10/2017	0	0	0

2012294640	252	rural/clinic	No	10/10/2017	0	0	0
2015397714	252	rural/clinic	No	10/10/2017	0	0	0
2015397715	252	rural/clinic	No	10/10/2017	0	0	0
2015397716	252	rural/clinic	No	10/10/2017	0	0	0
2012359175	252	rural/clinic	No	10/10/2017	0	0	0
2012359176	252	rural/clinic	No	10/10/2017	0	0	0
2012359177	252	rural/clinic	No	10/10/2017	0	0	0
2015326611	252	rural/clinic	No	10/10/2017	0	0	0
2015326612	252	rural/clinic	No	10/10/2017	0	0	0
2014333613	252	rural/clinic	No	10/10/2017	0	0	0
2014333614	252	rural/clinic	No	10/10/2017	0	0	0
2014333615	252	rural/clinic	No	10/10/2017	0	0	0
2014333616	252	rural/clinic	No	10/10/2017	0	0	0
2012275249	252	rural/clinic	No	10/10/2017	0	0	0
2012275250	252	rural/clinic	No	10/10/2017	0	0	0
2015336677	252	rural/clinic	No	10/10/2017	0	0	0
2014358237	252	rural/clinic	No	10/10/2017	0	0	0
2015336678	252	rural/clinic	No	10/10/2017	0	0	0
2014358238	252	rural/clinic	No	10/10/2017	0	0	0
2015313503	252	rural/clinic	No	10/10/2017	0	0	0
2015336679	252	rural/clinic	No	10/10/2017	0	0	0
2015336680	252	rural/clinic	No	10/10/2017	0	0	0
2015313504	252	rural/clinic	No	10/10/2017	0	0	0
2012294359	252	rural/clinic	No	10/10/2017	0	0	0
2015313505	252	rural/clinic	No	10/10/2017	0	0	0
2015352381	252	rural/clinic	No	10/10/2017	0	0	0
2015352383	252	rural/clinic	No	10/10/2017	0	0	0
2015313506	252	rural/clinic	No	10/10/2017	0	0	0
2015352384	252	rural/clinic	No	10/10/2017	0	0	0
2015313507	252	rural/clinic	No	10/10/2017	0	0	0
2015303258	252	rural/clinic	No	10/10/2017	0	0	0
2015303259	252	rural/clinic	No	10/10/2017	0	0	0
2015313508	252	rural/clinic	No	10/10/2017	0	0	0
2015303260	252	rural/clinic	No	10/10/2017	0	0	0
2015313509	252	rural/clinic	No	10/10/2017	0	0	0
2015303261	252	rural/clinic	No	10/10/2017	0	0	0
2015303264	252	rural/clinic	No	10/10/2017	0	0	0
2011136265	252	rural/clinic	No	10/10/2017	0	0	0
2015289342	252	rural/clinic	No	10/10/2017	0	0	0
2012322692	252	rural/clinic	No	10/10/2017	0	0	0
2014340328	252	rural/clinic	No	10/10/2017	0	0	0
2015319446	252	rural/clinic	No	10/10/2017	0	0	0
2014343884	252	rural/clinic	No	10/10/2017	0	0	0
2015368636	252	rural/clinic	No	10/10/2017	0	0	0
2015415678	252	rural/clinic	No	10/10/2017	0	0	0
2014367414	252	rural/clinic	No	10/10/2017	0	0	0
2015415679	252	rural/clinic	No	10/10/2017	0	0	0
2015415680	252	rural/clinic	No	10/10/2017	0	0	0
2015415681	252	rural/clinic	No	10/10/2017	0	0	0
2015332380	252	rural/clinic	No	10/10/2017	0	0	0
2015415682	252	rural/clinic	No	10/10/2017	0	0	0
2015415683	252	rural/clinic	No	10/10/2017	0	0	0
2015332381	252	rural/clinic	No	10/10/2017	0	0	0
2015415684	252	rural/clinic	No	10/10/2017	0	0	0
2012253135	252	rural/clinic	No	10/10/2017	0	0	0
2014303659	252	rural/clinic	No	10/10/2017	0	0	0
2015332382	252	rural/clinic	No	10/10/2017	0	0	0
2015360548	252	rural/clinic	No	10/10/2017	0	0	0
2014303660	252	rural/clinic	No	10/10/2017	0	0	0
2015360549	252	rural/clinic	No	10/10/2017	0	0	0
2012346655	252	rural/clinic	No	10/10/2017	0	0	0
2015419089	252	rural/clinic	No	10/10/2017	0	0	0
2015419090	252	rural/clinic	No	10/10/2017	0	0	0
2015419091	252	rural/clinic	No	10/10/2017	0	0	0
2015419092	252	rural/clinic	No	10/10/2017	0	0	0
2015349736	252	rural/clinic	No	10/10/2017	0	0	0
2015332488	252	rural/clinic	No	10/10/2017	0	0	0
2015349738	252	rural/clinic	No	10/10/2017	0	0	0
2015349739	252	rural/clinic	No	10/10/2017	0	0	0
2015415717	252	rural/clinic	No	10/10/2017	0	0	0
2015415718	252	rural/clinic	No	10/10/2017	0	0	0
2012295833	252	rural/clinic	No	10/10/2017	0	0	0
2015415719	252	rural/clinic	No	10/10/2017	0	0	0
2012295834	252	rural/clinic	No	10/10/2017	0	0	0
2012295835	252	rural/clinic	No	10/10/2017	0	0	0
2015415720	252	rural/clinic	No	10/10/2017	0	0	0
2015338473	252	rural/clinic	No	10/10/2017	0	0	0
2015326944	252	rural/clinic	No	10/10/2017	0	0	0
2012295836	252	rural/clinic	No	10/10/2017	0	0	0
2012295837	252	rural/clinic	No	10/10/2017	0	0	0
2015326945	252	rural/clinic	No	10/10/2017	0	0	0
2015326947	252	rural/clinic	No	10/10/2017	0	0	0

2012295838	252	rural/clinic	No	10/10/2017	0	0	0
2015326946	252	rural/clinic	No	10/10/2017	0	0	0
2012295839	252	rural/clinic	No	10/10/2017	0	0	0
2015362761	252	rural/clinic	No	10/10/2017	0	0	0
2015364992	252	rural/clinic	No	10/10/2017	0	0	0
2015376673	252	rural/clinic	No	10/10/2017	0	0	0
2015364993	252	rural/clinic	No	10/10/2017	0	0	0
2015364994	252	rural/clinic	No	10/10/2017	0	0	0
2015401708	252	rural/clinic	No	10/10/2017	0	0	0
2011193701	252	rural/clinic	No	10/10/2017	0	0	0
2012335674	252	rural/clinic	No	10/10/2017	0	0	0
2014307423	252	rural/clinic	No	10/10/2017	0	0	0
2015347010	252	rural/clinic	No	10/10/2017	0	0	0
2015347706	252	rural/clinic	No	10/10/2017	0	0	0
2011117889	252	rural/clinic	No	10/10/2017	0	0	0
2011117887	252	rural/clinic	No	10/10/2017	0	0	0
2012390677	252	rural/clinic	No	10/10/2017	0	0	0
2012390678	252	rural/clinic	No	10/10/2017	0	0	0
2015340756	252	rural/clinic	No	10/10/2017	0	0	0
2015325402	252	rural/clinic	No	10/10/2017	0	0	0
2012390679	252	rural/clinic	No	10/10/2017	0	0	0
2012390680	252	rural/clinic	No	10/10/2017	0	0	0
2015325403	252	rural/clinic	No	10/10/2017	0	0	0
2015325404	252	rural/clinic	No	10/10/2017	0	0	0
2015325405	252	rural/clinic	No	10/10/2017	0	0	0
2015386769	252	rural/clinic	No	10/10/2017	0	0	0
2015386771	252	rural/clinic	No	10/10/2017	0	0	0
2015386772	252	rural/clinic	No	10/10/2017	0	0	0
2015340731	252	rural/clinic	No	10/10/2017	0	0	0
2015386773	252	rural/clinic	No	16/10/2017	0	0	0
2011236181	252	rural/clinic	No	10/10/2017	0	0	0
2015386774	252	rural/clinic	No	10/10/2017	0	0	0
2011236182	252	rural/clinic	No	10/10/2017	0	0	0
2015339784	252	rural/clinic	No	10/10/2017	0	0	0
2015376855	252	rural/clinic	No	10/10/2017	0	0	0
2012390151	252	rural/clinic	No	10/10/2017	0	0	0
2015376856	252	rural/clinic	No	10/10/2017	0	0	0
2011117886	252	rural/clinic	No	10/10/2017	0	0	0
2014290330	252	rural/clinic	No	10/10/2017	0	0	0
2014300357	252	rural/clinic	No	10/10/2017	0	0	0
2015337708	252	rural/clinic	No	10/10/2017	0	0	0
2014300358	252	rural/clinic	No	10/10/2017	0	0	0
2015337709	252	rural/clinic	No	10/10/2017	0	0	0
2015337710	252	rural/clinic	No	10/10/2017	0	0	0
2015355935	252	rural/clinic	No	10/10/2017	0	0	0
2015337638	252	rural/clinic	No	10/10/2017	0	0	0
2015355936	252	rural/clinic	No	10/10/2017	0	0	0
2015303702	252	rural/clinic	No	10/10/2017	0	0	0
2015303703	252	rural/clinic	No	10/10/2017	0	0	0
2015303704	252	rural/clinic	No	10/10/2017	0	0	0
2014299932	252	rural/clinic	No	10/10/2017	0	0	0
2015303705	252	rural/clinic	No	10/10/2017	0	0	0
201066594	252	rural/clinic	No	10/10/2017	0	0	0
2015337707	252	rural/clinic	No	10/10/2017	0	0	0
2015303657	252	rural/clinic	No	10/10/2017	0	0	0
2011223214	252	rural/clinic	No	10/10/2017	0	0	0
2015303658	252	rural/clinic	No	10/10/2017	0	0	0
2011223215	252	rural/clinic	No	10/10/2017	0	0	0
2015303659	252	rural/clinic	No	10/10/2017	0	0	0
2015303660	252	rural/clinic	No	10/10/2017	0	0	0
2014297611	252	rural/clinic	No	10/10/2017	0	0	0
2014297612	252	rural/clinic	No	10/10/2017	0	0	0
2015334663	252	rural/clinic	No	10/10/2017	0	0	0
2015334664	252	rural/clinic	No	10/10/2017	0	0	0
2014311379	252	rural/clinic	No	10/10/2017	0	0	0
2011157099	252	rural/clinic	No	10/10/2017	0	0	0
2015344369	252	rural/clinic	No	10/10/2017	0	0	0
2015337395	252	rural/clinic	No	10/10/2017	0	0	0
2015400311	252	rural/clinic	No	10/10/2017	0	0	0
2015337397	252	rural/clinic	No	10/10/2017	0	0	0
2015337568	252	rural/clinic	No	10/10/2017	0	0	0
2012289622	252	rural/clinic	No	10/10/2017	0	0	0
2013254816	252	rural/clinic	No	10/10/2017	0	0	0
2015286875	252	rural/clinic	No	10/10/2017	0	0	0
2014383712	252	rural/clinic	No	10/10/2017	0	0	0
201066595	252	rural/clinic	No	10/10/2017	0	0	0
2015377783	252	rural/clinic	No	10/10/2017	0	0	0
2015377784	252	rural/clinic	No	10/10/2017	0	0	0
2011133167	252	rural/clinic	No	10/10/2017	0	0	0
2015377785	252	rural/clinic	No	10/10/2017	0	0	0
2011135972	252	rural/clinic	No	10/10/2017	0	0	0
2011135973	252	rural/clinic	No	10/10/2017	0	0	0

2015377786	252	rural/clinic	No	10/10/2017	0	0	0
2011135974	252	rural/clinic	No	10/10/2017	0	0	0
2015377787	252	rural/clinic	No	10/10/2017	0	0	0
2015355538	252	rural/clinic	No	10/10/2017	0	0	0
2015385974	252	rural/clinic	No	10/10/2017	0	0	0
2015315552	252	rural/clinic	No	10/10/2017	0	0	0
2015377788	252	rural/clinic	No	10/10/2017	0	0	0
2015315553	252	rural/clinic	No	10/10/2017	0	0	0
2015377789	252	rural/clinic	No	10/10/2017	0	0	0
2015315554	252	rural/clinic	No	10/10/2017	0	0	0
2015377790	252	rural/clinic	No	10/10/2017	0	0	0
2015377791	252	rural/clinic	No	10/10/2017	0	0	0
2015382975	252	rural/clinic	No	10/10/2017	0	0	0
2015347008	252	rural/clinic	No	10/10/2017	0	0	0
2015382976	252	rural/clinic	No	10/10/2017	0	0	0
2015347009	252	rural/clinic	No	10/10/2017	0	0	0
2015382977	252	rural/clinic	No	10/10/2017	0	0	0
2015347707	252	rural/clinic	No	10/10/2017	0	0	0
2013264094	252	rural/clinic	No	10/10/2017	0	0	0
2015355539	252	rural/clinic	No	10/10/2017	0	0	0
2015355664	252	rural/clinic	No	10/10/2017	0	0	0
2015338659	252	rural/clinic	No	10/10/2017	0	0	0
2015355662	252	rural/clinic	No	10/10/2017	0	0	0
2014357340	252	rural/clinic	No	10/10/2017	0	0	0
2014346291	252	rural/clinic	No	10/10/2017	0	0	0
2015409045	252	rural/clinic	No	10/10/2017	0	0	0
2015409049	252	rural/clinic	No	10/10/2017	0	0	0
2012244296	252	rural/clinic	No	10/10/2017	0	0	0
2014357835	252	rural/clinic	No	10/10/2017	0	0	0
2014357836	252	rural/clinic	No	10/10/2017	0	0	0
2014357837	252	rural/clinic	No	10/10/2017	0	0	0
2014357838	252	rural/clinic	No	10/10/2017	0	0	0
2014357339	252	rural/clinic	No	10/10/2017	0	0	0
2015385965	252	rural/clinic	No	10/10/2017	0	0	0
2015385966	252	rural/clinic	No	10/10/2017	0	0	0
2015385967	252	rural/clinic	No	10/10/2017	0	0	0
2015409046	252	rural/clinic	No	10/10/2017	0	0	0
2015385968	252	rural/clinic	No	10/10/2017	0	0	0
2015409047	252	rural/clinic	No	10/10/2017	0	0	0
2015385969	252	rural/clinic	No	10/10/2017	0	0	0
2015409048	252	rural/clinic	No	10/10/2017	0	0	0
2015385970	252	rural/clinic	No	10/10/2017	0	0	0
2014360977	252	rural/clinic	No	10/10/2017	0	0	0
2014360978	252	rural/clinic	No	10/10/2017	0	0	0
2015385971	252	rural/clinic	No	10/10/2017	0	0	0
2015376408	252	rural/clinic	No	10/10/2017	0	0	0
2015385972	252	rural/clinic	No	10/10/2017	0	0	0
2015385976	252	rural/clinic	No	10/10/2017	0	0	0
2012291495	252	rural/clinic	No	10/10/2017	0	0	0
2015385977	252	rural/clinic	No	10/10/2017	0	0	0
2015390476	252	rural/clinic	No	10/10/2017	0	0	0
2014298247	252	rural/clinic	No	10/10/2017	0	0	0
2014332452	252	rural/clinic	No	10/10/2017	0	0	0
2015385978	252	rural/clinic	No	10/10/2017	0	0	0
2015385979	252	rural/clinic	No	10/10/2017	0	0	0
2014332453	252	rural/clinic	No	10/10/2017	0	0	0
2015385980	252	rural/clinic	No	10/10/2017	0	0	0
2011155247	252	rural/clinic	No	10/10/2017	0	0	0
2015355659	252	rural/clinic	No	10/10/2017	0	0	0
2015301372	252	rural/clinic	No	10/10/2017	0	0	0
2015355661	252	rural/clinic	No	10/10/2017	0	0	0
2015355660	252	rural/clinic	No	10/10/2017	0	0	0
2015302863	252	rural/clinic	No	10/10/2017	0	0	0
2015385981	252	rural/clinic	No	10/10/2017	0	0	0
2015302864	252	rural/clinic	No	10/10/2017	0	0	0
2015355658	252	rural/clinic	No	10/10/2017	0	0	0
2011134809	252	rural/clinic	No	10/10/2017	0	0	0
2015390334	252	rural/clinic	No	10/10/2017	0	0	0
2015315555	252	rural/clinic	No	10/10/2017	0	0	0
2015390335	252	rural/clinic	No	10/10/2017	0	0	0
2014288255	252	rural/clinic	No	10/10/2017	0	0	0
2012312180	252	rural/clinic	No	10/10/2017	0	0	0
2014288251	252	rural/clinic	No	10/10/2017	0	0	0
2012312181	252	rural/clinic	No	10/10/2017	0	0	0
2012312182	252	rural/clinic	No	10/10/2017	0	0	0
2012312185	252	rural/clinic	No	10/10/2017	0	0	0
2014288252	252	rural/clinic	No	10/10/2017	0	0	0
2015363090	252	rural/clinic	No	10/10/2017	0	0	0
2015363091	252	rural/clinic	No	10/10/2017	0	0	0
2015363092	252	rural/clinic	No	10/10/2017	0	0	0
2015363093	252	rural/clinic	No	10/10/2017	0	0	0
2014288253	252	rural/clinic	No	10/10/2017	0	0	0

2014288254	252	rural/clinic	No	10/10/2017	0	0	0
2015355663	252	rural/clinic	No	10/10/2017	0	0	0
2015363094	252	rural/clinic	No	10/10/2017	0	0	0
2015355657	252	rural/clinic	No	10/10/2017	0	0	0
2015363095	252	rural/clinic	No	10/10/2017	0	0	0
2015337570	252	rural/clinic	No	10/10/2017	0	0	0
2015385975	252	rural/clinic	No	25/09/2017	0	0	0
2012330896	252	rural/clinic	No	10/10/2017	0	0	0
2015337569	252	rural/clinic	No	10/10/2017	0	0	0
2014363197	252	rural/clinic	No	10/10/2017	0	0	0
2015337398	252	rural/clinic	No	10/10/2017	0	0	0
2012330895	252	rural/clinic	No	10/10/2017	0	0	0
2015337396	252	rural/clinic	No	10/10/2017	0	0	0
2015413367	252	rural/clinic	No	10/10/2017	0	0	0
2015344370	252	rural/clinic	No	10/10/2017	0	0	0
2015413368	252	rural/clinic	No	10/10/2017	0	0	0
2015385973	252	rural/clinic	No	19/09/2017	0	0	0
2012379925	252	rural/clinic	No	10/10/2017	0	0	0
2014338460	252	rural/clinic	No	02/10/2017	0	0	0
2015303263	252	rural/clinic	No	10/10/2017	0	0	0
2012390681	252	rural/clinic	No	10/10/2017	0	0	0
2012390683	252	rural/clinic	No	10/10/2017	0	0	0
2012390682	252	rural/clinic	No	10/10/2017	0	0	0
2012390674	252	rural/clinic	No	10/10/2017	0	0	0
2014358239	252	rural/clinic	No	10/10/2017	0	0	0
2012390676	252	rural/clinic	No	10/10/2017	0	0	0
2012294638	252	rural/clinic	No	10/10/2017	0	0	0
2012390675	252	rural/clinic	No	10/10/2017	0	0	0
2012294639	252	rural/clinic	No	10/10/2017	0	0	0
2012312183	252	rural/clinic	No	10/10/2017	0	0	0
2012312184	252	rural/clinic	No	10/10/2017	0	0	0
2014303658	252	rural/clinic	No	10/10/2017	0	0	0
2015340732	252	rural/clinic	No	10/10/2017	0	0	0
2015303701	252	rural/clinic	No	10/10/2017	0	0	0
2015334665	252	rural/clinic	No	10/10/2017	0	0	0
2013267751	252	rural/clinic	No	10/10/2017	0	0	0
2014348069	252	rural/clinic	No	26/09/2017	0	0	0
2015326935	252	rural/clinic	No	06/09/2017	0	0	0
2014335850	252	rural/clinic	No	26/09/2017	0	0	0
0	252	rural/clinic	No	21/09/2017	0	0	0
0	252	rural/clinic	No	21/09/2017	0	0	0
2014300433	252	rural/clinic	No	27/09/2017	0	0	0
2015384369	252	rural/clinic	No	21/09/2017	0	0	0
2015382976	252	rural/clinic	No	10/10/2017	0	0	0
2015382976	252	rural/clinic	No	10/10/2017	0	0	0
2015355936	252	rural/clinic	No	10/10/2017	0	0	0
2015355936	252	rural/clinic	No	10/10/2017	0	0	0
2014297612	252	rural/clinic	No	10/10/2017	0	0	0
2014297612	252	rural/clinic	No	10/10/2017	0	0	0
2014311379	252	rural/clinic	No	10/10/2017	0	0	0
2014311379	252	rural/clinic	No	10/10/2017	0	0	0
2011236182	252	rural/clinic	No	10/10/2017	0	0	0
2011236182	252	rural/clinic	No	10/10/2017	0	0	0
2015340756	252	rural/clinic	No	10/10/2017	0	0	0
2015340756	252	rural/clinic	No	10/10/2017	0	0	0
2015397716	252	rural/clinic	No	10/10/2017	0	0	0
2015397716	252	rural/clinic	No	10/10/2017	0	0	0
2015326611	252	rural/clinic	No	10/10/2017	0	0	0
2015326611	252	rural/clinic	No	10/10/2017	0	0	0
2014333614	252	rural/clinic	No	10/10/2017	0	0	0
2014333614	252	rural/clinic	No	10/10/2017	0	0	0
2015362761	252	rural/clinic	No	10/10/2017	0	0	0
2015362761	252	rural/clinic	No	10/10/2017	0	0	0
2015313509	252	rural/clinic	No	10/10/2017	0	0	0
2015313509	252	rural/clinic	No	10/10/2017	0	0	0
2015332488	252	rural/clinic	No	10/10/2017	0	0	0
2015332488	252	rural/clinic	No	10/10/2017	0	0	0
2015397700	252	rural/clinic	No	26/09/2017	0	0	0
2015347367	252	rural/clinic	No	12/10/2017	0	0	0
2012304347	252	rural/clinic	No	12/10/2017	0	0	0
2015352015	252	rural/clinic	No	12/10/2017	0	0	0
2015347366	252	rural/clinic	No	12/10/2017	0	0	0
2015347369	252	rural/clinic	No	12/10/2017	0	0	0
2015347370	252	rural/clinic	No	12/10/2017	0	0	0
2015347371	252	rural/clinic	No	12/10/2017	0	0	0
2015347372	252	rural/clinic	No	12/10/2017	0	0	0
2015347373	252	rural/clinic	No	12/10/2017	0	0	0
2015347374	252	rural/clinic	No	12/10/2017	0	0	0
2015347375	252	rural/clinic	No	12/10/2017	0	0	0
2015347376	252	rural/clinic	No	12/10/2017	0	0	0
2015362017	252	rural/clinic	No	12/10/2017	0	0	0
2015362018	252	rural/clinic	No	12/10/2017	0	0	0

2015362019	252	rural/clinic	No	12/10/2017	0	0	0
2015377193	252	rural/clinic	No	12/10/2017	0	0	0
2015335254	252	rural/clinic	No	12/10/2017	0	0	0
2015335251	252	rural/clinic	No	12/10/2017	0	0	0
2015401557	252	rural/clinic	No	12/10/2017	0	0	0
2015401556	252	rural/clinic	No	12/10/2017	0	0	0
2015401555	252	rural/clinic	No	12/10/2017	0	0	0
2015401554	252	rural/clinic	No	12/10/2017	0	0	0
2015401553	252	rural/clinic	No	12/10/2017	0	0	0
2015401551	252	rural/clinic	No	12/10/2017	0	0	0
2015402061	252	rural/clinic	No	12/10/2017	0	0	0
2015402062	252	rural/clinic	No	12/10/2017	0	0	0
2015402063	252	rural/clinic	No	12/10/2017	0	0	0
2015402064	252	rural/clinic	No	12/10/2017	0	0	0
2014304713	252	rural/clinic	No	12/10/2017	0	0	0
2014304712	252	rural/clinic	No	12/10/2017	0	0	0
2015403210	252	rural/clinic	No	12/10/2017	0	0	0
2014296654	252	rural/clinic	No	12/10/2017	0	0	0
2014296342	252	rural/clinic	No	12/10/2017	0	0	0
2013252790	252	rural/clinic	No	12/10/2017	0	0	0
2014298343	252	rural/clinic	No	12/10/2017	0	0	0
2015339036	252	rural/clinic	No	12/10/2017	0	0	0
2011144036	252	rural/clinic	No	12/10/2017	0	0	0
2014298047	252	rural/clinic	No	12/10/2017	0	0	0
2015419502	252	rural/clinic	No	12/10/2017	0	0	0
2015302307	252	rural/clinic	No	12/10/2017	0	0	0
2015302161	252	rural/clinic	No	12/10/2017	0	0	0
2015302160	252	rural/clinic	No	12/10/2017	0	0	0
2015301281	252	rural/clinic	No	12/10/2017	0	0	0
2015301280	252	rural/clinic	No	12/10/2017	0	0	0
2011229226	252	rural/clinic	No	12/10/2017	0	0	0
2015301279	252	rural/clinic	No	12/10/2017	0	0	0
2011229225	252	rural/clinic	No	12/10/2017	0	0	0
2015419094	252	rural/clinic	No	12/10/2017	0	0	0
2015349614	252	rural/clinic	No	12/10/2017	0	0	0
2015302308	252	rural/clinic	No	12/10/2017	0	0	0
2015339035	252	rural/clinic	No	12/10/2017	0	0	0
2014344542	252	rural/clinic	No	12/10/2017	0	0	0
2014344541	252	rural/clinic	No	12/10/2017	0	0	0
2014344543	252	rural/clinic	No	12/10/2017	0	0	0
2012265930	252	rural/clinic	No	12/10/2017	0	0	0
2015372885	252	rural/clinic	No	12/10/2017	0	0	0
2015372884	515	rural/clinic	No	12/10/2017	0	0	0
2015372886	515	rural/clinic	No	12/10/2017	0	0	0
2015372883	515	rural/clinic	No	12/10/2017	0	0	0
2015382778	515	rural/clinic	No	12/10/2017	0	0	0
2015382779	515	rural/clinic	No	12/10/2017	0	0	0
2015382780	515	rural/clinic	No	12/10/2017	0	0	0
2015382781	515	rural/clinic	No	12/10/2017	0	0	0
2015382784	515	rural/clinic	No	12/10/2017	0	0	0
2015375065	515	rural/clinic	No	12/10/2017	0	0	0
2015415075	515	rural/clinic	No	12/10/2017	0	0	0
2015382782	515	rural/clinic	No	12/10/2017	0	0	0
2015415073	515	rural/clinic	No	12/10/2017	0	0	0
2015415076	515	rural/clinic	No	12/10/2017	0	0	0
2015334544	515	rural/clinic	No	12/10/2017	0	0	0
2015352382	591	rural/clinic	No	10/10/2017	0	0	0
2015302863	591	rural/clinic	No	10/10/2017	0	0	0
2015302863	591	rural/clinic	No	10/10/2017	0	0	0
2015385977	591	rural/clinic	No	10/10/2017	0	0	0
2012390682	591	rural/clinic	No	10/10/2017	0	0	0
2012390682	591	rural/clinic	No	10/10/2017	0	0	0
2012390674	591	rural/clinic	No	10/10/2017	0	0	0
2012390674	591	rural/clinic	No	10/10/2017	0	0	0
2015331107	591	rural/clinic	No	26/09/2017	0	0	0
2015349074	591	rural/clinic	No	29/09/2017	0	0	0
2014377253	591	rural/clinic	No	26/09/2017	0	0	0
2014377253	591	rural/clinic	No	26/09/2017	0	0	0
2015326940	591	rural/clinic	No	26/09/2017	0	0	0
2011144120	591	rural/clinic	No	12/10/2017	0	0	0
2011144118	591	rural/clinic	No	12/10/2017	0	0	0
2011144119	591	rural/clinic	No	12/10/2017	0	0	0
2014385622	591	rural/clinic	No	12/10/2017	0	0	0
2015403101	591	rural/clinic	No	12/10/2017	0	0	0
2015355663	591	rural/clinic	No	10/10/2017	0	0	0
2015355663	591	rural/clinic	No	10/10/2017	0	0	0
2015347366	591	rural/clinic	No	12/10/2017	0	0	0
2012265930	591	rural/clinic	No	12/10/2017	0	0	0
2012265930	591	rural/clinic	No	12/10/2017	0	0	0
2011193703	591	rural/clinic	No	12/10/2017	0	0	0
2011193703	591	rural/clinic	No	12/10/2017	0	0	0
2015401561	591	rural/clinic	No	12/10/2017	0	0	0

2015401561	591	rural/clinic	No	12/10/2017	0	0	0
2015321766	591	rural/clinic	No	12/10/2017	0	0	0
2015321766	591	rural/clinic	No	12/10/2017	0	0	0
2015397717	591	rural/clinic	No	16/10/2017	0	0	0
2015397718	591	rural/clinic	No	16/10/2017	0	0	0
2015397719	591	rural/clinic	No	16/10/2017	0	0	0
2015397720	591	rural/clinic	No	16/10/2017	0	0	0
2014345913	591	rural/clinic	No	16/10/2017	0	0	0
2014345914	591	rural/clinic	No	16/10/2017	0	0	0
2012369035	591	rural/clinic	No	08/10/2017	0	0	0
2014358240	591	rural/clinic	No	17/10/2017	0	0	0
2015390336	591	rural/clinic	No	17/10/2017	0	0	0
2015390339	591	rural/clinic	No	17/10/2017	0	0	0
2015390337	591	rural/clinic	No	17/10/2017	0	0	0
2015390338	591	rural/clinic	No	17/10/2017	0	0	0
2015377792	591	rural/clinic	No	17/10/2017	0	0	0
2012369862	591	rural/clinic	No	17/10/2017	0	0	0
2015313512	591	rural/clinic	No	17/10/2017	0	0	0
2015313511	591	rural/clinic	No	17/10/2017	0	0	0
2015313513	591	rural/clinic	No	17/10/2017	0	0	0
2015313515	591	rural/clinic	No	17/10/2017	0	0	0
2015417006	591	rural/clinic	No	17/10/2017	0	0	0
2015417007	591	rural/clinic	No	17/10/2017	0	0	0
2015417008	591	rural/clinic	No	17/10/2017	0	0	0
2015417009	591	rural/clinic	No	17/10/2017	0	0	0
2015403192	591	rural/clinic	No	17/10/2017	0	0	0
2015400313	591	rural/clinic	No	17/10/2017	0	0	0
2015403191	591	rural/clinic	No	17/10/2017	0	0	0
2015400312	591	rural/clinic	No	17/10/2017	0	0	0
2014290278	591	rural/clinic	No	17/10/2017	0	0	0
2012252105	591	rural/clinic	No	17/10/2017	0	0	0
2014319145	591	rural/clinic	No	17/10/2017	0	0	0
2014290279	591	rural/clinic	No	17/10/2017	0	0	0
2012252102	591	rural/clinic	No	17/10/2017	0	0	0
2012371466	591	rural/clinic	No	17/10/2017	0	0	0
2015340943	591	rural/clinic	No	17/10/2017	0	0	0
2014383713	591	rural/clinic	No	17/10/2017	0	0	0
2015340942	591	rural/clinic	No	17/10/2017	0	0	0
2012371467	591	rural/clinic	No	17/10/2017	0	0	0
2012275997	591	rural/clinic	No	17/10/2017	0	0	0
2015340944	591	rural/clinic	No	17/10/2017	0	0	0
2015352830	591	rural/clinic	No	17/10/2017	0	0	0
2014319224	591	rural/clinic	No	17/10/2017	0	0	0
2015352831	591	rural/clinic	No	17/10/2017	0	0	0
2015357538	591	rural/clinic	No	17/10/2017	0	0	0
2015340932	591	rural/clinic	No	17/10/2017	0	0	0
2015352833	591	rural/clinic	No	17/10/2017	0	0	0
2015340934	591	rural/clinic	No	17/10/2017	0	0	0
2015386776	591	rural/clinic	No	17/10/2017	0	0	0
2015386777	591	rural/clinic	No	17/10/2017	0	0	0
2015326948	591	rural/clinic	No	17/10/2017	0	0	0
2015386778	591	rural/clinic	No	17/10/2017	0	0	0
2015386775	591	rural/clinic	No	17/10/2017	0	0	0
2012369861	591	rural/clinic	No	17/10/2017	0	0	0
2015326949	591	rural/clinic	No	17/10/2017	0	0	0
2014346292	591	rural/clinic	No	17/10/2017	0	0	0
2012369860	591	rural/clinic	No	17/10/2017	0	0	0
2015362839	591	rural/clinic	No	17/10/2017	0	0	0
2015409050	591	rural/clinic	No	17/10/2017	0	0	0
2015332383	591	rural/clinic	No	17/10/2017	0	0	0
2012369859	591	rural/clinic	No	17/10/2017	0	0	0
2015332384	591	rural/clinic	No	17/10/2017	0	0	0
2015332489	591	rural/clinic	No	17/10/2017	0	0	0
2012294069	591	rural/clinic	No	17/10/2017	0	0	0
2015376411	591	rural/clinic	No	17/10/2017	0	0	0
2011135096	591	rural/clinic	No	17/10/2017	0	0	0
2015364995	591	rural/clinic	No	17/10/2017	0	0	0
2014358041	591	rural/clinic	No	17/10/2017	0	0	0
2015364996	591	rural/clinic	No	17/10/2017	0	0	0
2015415721	591	rural/clinic	No	17/10/2017	0	0	0
2014358042	591	rural/clinic	No	17/10/2017	0	0	0
2015415722	591	rural/clinic	No	17/10/2017	0	0	0
2015364997	591	rural/clinic	No	17/10/2017	0	0	0
2014358044	591	rural/clinic	No	17/10/2017	0	0	0
2015352385	591	rural/clinic	No	17/10/2017	0	0	0
2015364998	591	rural/clinic	No	17/10/2017	0	0	0
2015365000	591	rural/clinic	No	17/10/2017	0	0	0
2012294361	591	rural/clinic	No	17/10/2017	0	0	0
2015358369	591	rural/clinic	No	17/10/2017	0	0	0
2012294360	591	rural/clinic	No	17/10/2017	0	0	0
2015358370	591	rural/clinic	No	17/10/2017	0	0	0
2013267753	591	rural/clinic	No	17/10/2017	0	0	0

2015358371	591	rural/clinic	No	17/10/2017	0	0	0
2015358372	591	rural/clinic	No	17/10/2017	0	0	0
2015358373	591	rural/clinic	No	17/10/2017	0	0	0
2015368306	591	rural/clinic	No	17/10/2017	0	0	0
2015358374	591	rural/clinic	No	17/10/2017	0	0	0
2015337571	591	rural/clinic	No	17/10/2017	0	0	0
2015337572	591	rural/clinic	No	17/10/2017	0	0	0
2015400315	591	rural/clinic	No	17/10/2017	0	0	0
2015337573	591	rural/clinic	No	17/10/2017	0	0	0
2012294070	591	rural/clinic	No	17/10/2017	0	0	0
2015337574	591	rural/clinic	No	17/10/2017	0	0	0
2015337575	591	rural/clinic	No	17/10/2017	0	0	0
2012314030	591	rural/clinic	No	17/10/2017	0	0	0
2015415723	591	rural/clinic	No	17/10/2017	0	0	0
2015415724	591	rural/clinic	No	17/10/2017	0	0	0
2015415725	591	rural/clinic	No	17/10/2017	0	0	0
2015345164	591	rural/clinic	No	17/10/2017	0	0	0
2014327133	591	rural/clinic	No	17/10/2017	0	0	0
2015340935	591	rural/clinic	No	17/10/2017	0	0	0
2015325260	591	rural/clinic	No	17/10/2017	0	0	0
2014319147	591	rural/clinic	No	17/10/2017	0	0	0
2014327132	221	rural/clinic	No	17/10/2017	0	0	0
2011144846	737	rural/clinic	No	17/10/2017	0	0	0
2014319220	737	rural/clinic	No	17/10/2017	0	0	0
2011144848	737	rural/clinic	No	17/10/2017	0	0	0
2015340938	737	rural/clinic	No	17/10/2017	0	0	0
2015340936	737	rural/clinic	No	17/10/2017	0	0	0
2015340937	737	rural/clinic	No	17/10/2017	0	0	0
2014290744	737	rural/clinic	No	17/10/2017	0	0	0
2013248361	737	rural/clinic	No	17/10/2017	0	0	0
2014319223	737	rural/clinic	No	17/10/2017	0	0	0
2014290743	737	rural/clinic	No	17/10/2017	0	0	0
2015340933	737	rural/clinic	No	17/10/2017	0	0	0
2015403188	737	rural/clinic	No	17/10/2017	0	0	0
2015337576	737	rural/clinic	No	17/10/2017	0	0	0
2015337578	737	rural/clinic	No	17/10/2017	0	0	0
2014319221	737	rural/clinic	No	17/10/2017	0	0	0
2015337579	737	rural/clinic	No	17/10/2017	0	0	0
2015405734	737	rural/clinic	No	17/10/2017	0	0	0
2014319218	737	rural/clinic	No	17/10/2017	0	0	0
2014319219	737	rural/clinic	No	17/10/2017	0	0	0
2012390687	559	rural/clinic	No	17/10/2017	0	0	0
2012390690	559	rural/clinic	No	17/10/2017	0	0	0
2014319217	559	rural/clinic	No	17/10/2017	0	0	0
2013256708	559	rural/clinic	No	17/10/2017	0	0	0
2014319222	559	rural/clinic	No	17/10/2017	0	0	0
2015357310	559	rural/clinic	No	17/10/2017	0	0	0
2015403190	559	rural/clinic	No	17/10/2017	0	0	0
2013256709	559	rural/clinic	No	17/10/2017	0	0	0
2015390480	559	rural/clinic	No	17/10/2017	0	0	0
2015390479	559	rural/clinic	No	17/10/2017	0	0	0
2015303265	559	rural/clinic	No	17/10/2017	0	0	0
2015390477	559	rural/clinic	No	17/10/2017	0	0	0
2015390478	559	rural/clinic	No	17/10/2017	0	0	0
2012295840	559	rural/clinic	No	17/10/2017	0	0	0
2012295841	559	rural/clinic	No	17/10/2017	0	0	0
2012269025	559	rural/clinic	No	17/10/2017	0	0	0
2015333461	559	rural/clinic	No	17/10/2017	0	0	0
2015412460	559	rural/clinic	No	17/10/2017	0	0	0
2014377422	559	rural/clinic	No	17/10/2017	0	0	0
2014326100	559	rural/clinic	No	17/10/2017	0	0	0
2012376780	559	rural/clinic	No	17/10/2017	0	0	0
2012336643	559	rural/clinic	No	17/10/2017	0	0	0
2014301403	559	rural/clinic	No	17/10/2017	0	0	0
2014301402	559	rural/clinic	No	17/10/2017	0	0	0
2014334194	559	rural/clinic	No	17/10/2017	0	0	0
2015405731	559	rural/clinic	No	17/10/2017	0	0	0
2015372837	559	rural/clinic	No	17/10/2017	0	0	0
2014334193	559	rural/clinic	No	17/10/2017	0	0	0
2015403189	559	rural/clinic	No	17/10/2017	0	0	0
2014334195	559	rural/clinic	No	17/10/2017	0	0	0
2015403183	559	rural/clinic	No	17/10/2017	0	0	0
2015372838	559	rural/clinic	No	17/10/2017	0	0	0
2015403186	559	rural/clinic	No	17/10/2017	0	0	0
2011143089	559	rural/clinic	No	17/10/2017	0	0	0
2015289296	559	rural/clinic	No	17/10/2017	0	0	0
2015405730	559	rural/clinic	No	17/10/2017	0	0	0
2015335992	559	rural/clinic	No	17/10/2017	0	0	0
2015403184	559	rural/clinic	No	17/10/2017	0	0	0
2015319308	559	rural/clinic	No	17/10/2017	0	0	0
2015403187	559	rural/clinic	No	17/10/2017	0	0	0
2015403185	559	rural/clinic	No	17/10/2017	0	0	0

2014363598	559	rural/clinic	No	17/10/2017	0	0	0
2015403200	559	rural/clinic	No	17/10/2017	0	0	0
2015335991	559	rural/clinic	No	17/10/2017	0	0	0
2015405733	559	rural/clinic	No	17/10/2017	0	0	0
2015294900	559	rural/clinic	No	17/10/2017	0	0	0
2014327542	559	rural/clinic	No	17/10/2017	0	0	0
2015385983	559	rural/clinic	No	17/10/2017	0	0	0
2015385984	559	rural/clinic	No	17/10/2017	0	0	0
2015294899	559	rural/clinic	No	17/10/2017	0	0	0
2015337640	559	rural/clinic	No	17/10/2017	0	0	0
2015337641	559	rural/clinic	No	17/10/2017	0	0	0
2015363103	559	rural/clinic	No	17/10/2017	0	0	0
2015315556	559	rural/clinic	No	17/10/2017	0	0	0
2015347213	559	rural/clinic	No	17/10/2017	0	0	0
2012323784	559	rural/clinic	No	17/10/2017	0	0	0
201066596	559	rural/clinic	No	17/10/2017	0	0	0
2012344513	559	rural/clinic	No	17/10/2017	0	0	0
2015337639	559	rural/clinic	No	17/10/2017	0	0	0
2015347217	559	rural/clinic	No	17/10/2017	0	0	0
2015347712	559	rural/clinic	No	17/10/2017	0	0	0
2015347711	559	rural/clinic	No	17/10/2017	0	0	0
2015315014	559	rural/clinic	No	17/10/2017	0	0	0
2015347011	559	rural/clinic	No	17/10/2017	0	0	0
2015385890	559	rural/clinic	No	17/10/2017	0	0	0
2012323785	559	rural/clinic	No	17/10/2017	0	0	0
2015385889	559	rural/clinic	No	17/10/2017	0	0	0
2015385886	559	rural/clinic	No	17/10/2017	0	0	0
2015385887	559	rural/clinic	No	17/10/2017	0	0	0
2014291627	559	rural/clinic	No	17/10/2017	0	0	0
2014288428	559	rural/clinic	No	17/10/2017	0	0	0
2015385888	559	rural/clinic	No	17/10/2017	0	0	0
2015324496	559	rural/clinic	No	17/10/2017	0	0	0
2015355540	559	rural/clinic	No	17/10/2017	0	0	0
2015347710	559	rural/clinic	No	17/10/2017	0	0	0
2011223216	559	rural/clinic	No	17/10/2017	0	0	0
2014301404	559	rural/clinic	No	17/10/2017	0	0	0
2014301681	559	rural/clinic	No	17/10/2017	0	0	0
2015412123	559	rural/clinic	No	17/10/2017	0	0	0
2014301679	559	rural/clinic	No	17/10/2017	0	0	0
2014301680	559	rural/clinic	No	17/10/2017	0	0	0
2012317287	559	rural/clinic	No	17/10/2017	0	0	0
2014343885	559	rural/clinic	No	17/10/2017	0	0	0
2012321345	559	rural/clinic	No	17/10/2017	0	0	0
2015289295	559	rural/clinic	No	17/10/2017	0	0	0
2015289297	559	rural/clinic	No	17/10/2017	0	0	0
20170153252	559	rural/clinic	No	17/10/2017	0	0	0
2015335993	559	rural/clinic	No	17/10/2017	0	0	0
2012317286	559	rural/clinic	No	17/10/2017	0	0	0
2012317288	559	rural/clinic	No	17/10/2017	0	0	0
2015384819	559	rural/clinic	No	17/10/2017	0	0	0
2015289343	559	rural/clinic	No	17/10/2017	0	0	0
2015368638	559	rural/clinic	No	17/10/2017	0	0	0
2015407077	559	rural/clinic	No	17/10/2017	0	0	0
2014303661	559	rural/clinic	No	17/10/2017	0	0	0
2012305842	559	rural/clinic	No	17/10/2017	0	0	0
2012305843	559	rural/clinic	No	17/10/2017	0	0	0
2012305844	559	rural/clinic	No	17/10/2017	0	0	0
2015359769	559	rural/clinic	No	17/10/2017	0	0	0
2015415685	559	rural/clinic	No	17/10/2017	0	0	0
2012379927	559	rural/clinic	No	17/10/2017	0	0	0
2012379928	559	rural/clinic	No	17/10/2017	0	0	0
2015303266	559	rural/clinic	No	17/10/2017	0	0	0
2015313510	559	rural/clinic	No	17/10/2017	0	0	0
2015347368	559	rural/clinic	No	17/10/2017	0	0	0
2015340944	559	rural/clinic	No	17/10/2017	0	0	0
2015386778	559	rural/clinic	No	17/10/2017	0	0	0
2015352385	559	rural/clinic	No	17/10/2017	0	0	0
2015352385	559	rural/clinic	No	17/10/2017	0	0	0
2012369860	559	rural/clinic	No	17/10/2017	0	0	0
2012369860	559	rural/clinic	No	17/10/2017	0	0	0
2015345164	559	rural/clinic	No	17/10/2017	0	0	0
2015345164	559	rural/clinic	No	17/10/2017	0	0	0
2015352831	559	rural/clinic	No	17/10/2017	0	0	0
2015352831	559	rural/clinic	No	17/10/2017	0	0	0
2015326949	559	rural/clinic	No	17/10/2017	0	0	0
2015326949	559	rural/clinic	No	17/10/2017	0	0	0
2015332489	559	rural/clinic	No	17/10/2017	0	0	0
2015332489	559	rural/clinic	No	17/10/2017	0	0	0
2015390337	559	rural/clinic	No	17/10/2017	0	0	0
2015390337	559	rural/clinic	No	17/10/2017	0	0	0
2015397718	559	rural/clinic	No	16/10/2017	0	0	0
2014290278	559	rural/clinic	No	17/10/2017	0	0	0

2014290278	559	rural/clinic	No	17/10/2017	0	0	0
2015403184	559	rural/clinic	No	17/10/2017	0	0	0
2015403184	559	rural/clinic	No	17/10/2017	0	0	0
2015337640	559	rural/clinic	No	17/10/2017	0	0	0
2014319219	559	rural/clinic	No	17/10/2017	0	0	0
2014319219	559	rural/clinic	No	17/10/2017	0	0	0
2012390688	559	rural/clinic	No	17/10/2017	0	0	0
2012390688	559	rural/clinic	No	17/10/2017	0	0	0
2012390685	559	rural/clinic	No	17/10/2017	0	0	0
2012390685	559	rural/clinic	No	17/10/2017	0	0	0
2012390690	559	rural/clinic	No	17/10/2017	0	0	0
2012390690	559	rural/clinic	No	17/10/2017	0	0	0
2014319222	559	rural/clinic	No	17/10/2017	0	0	0
1900WRONG	559	rural/clinic	No	17/10/2017	0	0	0
1900WRONG	559	rural/clinic	No	17/10/2017	0	0	0
2014326099	559	rural/clinic	No	17/10/2017	0	0	0
2015384820	559	rural/clinic	No	17/10/2017	0	0	0
2015347214	559	rural/clinic	No	17/10/2017	0	0	0
2015347709	559	rural/clinic	No	06/10/2017	0	0	0
2015337400	559	rural/clinic	No	17/10/2017	0	0	0
2015315013	559	rural/clinic	No	17/10/2017	0	0	0
2015347216	559	rural/clinic	No	17/10/2017	0	0	0
[truncated: 654,283 more chars]
